# A significant and persistent rise in the global burden of adolescent NAFLD and NASH estimated by BMI

**DOI:** 10.3389/fpubh.2024.1437432

**Published:** 2024-10-25

**Authors:** Xiao-Yun Lin, Jing-Mao Li, Ling-Yi Huang, Li-Yan Lin, Mei-Zhu Hong, Shangeng Weng, Jin-Shui Pan

**Affiliations:** ^1^Department of Hepatology, the First Affiliated Hospital of Fujian Medical University, Fuzhou, Fujian, China; ^2^Hepatology Research Institute, Fujian Medical University, Fuzhou, Fujian, China; ^3^Department of Hepatology, National Regional Medical Center, Binhai Campus of the First Affiliated Hospital, Fujian Medical University, Fuzhou, Fujian, China; ^4^Clinical Research Center for Liver and Intestinal Diseases of Fujian Province, the First Affiliated Hospital, Fujian Medical University, Fuzhou, Fujian, China; ^5^Department of Statistics and Data Science, School of Economics, Xiamen University, Xiamen, Fujian, China; ^6^Department of Neonatology, the First Affiliated Hospital of Fujian Medical University, Fuzhou, Fujian, China; ^7^Department of Traditional Chinese Medicine, Mengchao Hepatobiliary Hospital of Fujian Medical University, Fuzhou, Fujian, China; ^8^Hepatopancreatobiliary Surgery Department, the First Affiliated Hospital of Fujian Medical University, Fuzhou, Fujian, China

**Keywords:** epidemiology, nonalcoholic fatty liver disease, non-alcoholic steatohepatitis, body mass index, model simulation

## Abstract

**Background:**

Currently, there is a lack of global or even country/regional level data on adolescent non-alcoholic fatty liver disease (NAFLD) prevalence. However, an evidenced dose-dependent relationship exists between body mass index (BMI) and the risk of NAFLD. We aim to estimate the global and regional prevalence of adolescent NAFLD and related non-alcoholic steatohepatitis (NASH) based on BMI.

**Methods:**

Sigmoidal fitting curves were generated between BMI and the risk of NAFLD/NASH using the data extracted from the NHANES database. With global and regional BMI data from the NCD-RisC database, adolescent NAFLD/NASH prevalence was estimated at the international, regional, and country levels from 1975 to 2016. The prevalence of adolescent NAFLD/NASH from 2017 to 2030 was also forecasted.

**Results:**

The mean NAFLD prevalence was 15.31, and 12.68%, while the mean NASH prevalence was 2.50, and 2.47%, in boys, and girls aged 12–18, respectively. For both boys and girls, NAFLD/NASH prevalence increased with increasing BMI, and age. The global prevalence of adolescent NAFLD/NASH has gradually increased in the period from 1975 to 2016 and will maintain a similar trend between 2017 and 2030. High-income Western Countries had higher adolescent NAFLD/NASH whereas South Asia and Sub-Saharan Africa exhibited relatively lower adolescent NAFLD/NASH prevalence. The estimated annual percentage change (EAPC) of NAFLD prevalence in boys ranged from 0.72% (age 18) to 1.16% (age 12) while that in girls ranged from 0.69% (age 18) to 0.92% (age 12). EAPC of NASH prevalence in boys ranged from 1.65% (age 18) to 1.77% (age 12), and in girls from 1.48% (age 18) to 1.68% (age 12).

**Conclusion:**

The adolescent NAFLD/NASH prevalence increases year by year, and its burden varies significantly among different countries and regions. BMI is a precise predictor of NAFLD/NASH prevalence.

## Introduction

The prevalence of nonalcoholic fatty liver disease (NAFLD) is consistently rising, which is a recognized problem for global public health (1, 2). The annual increasing epidemic of NAFLD among children is even more substantial in recent years (3–5). NAFLD is characterized by excess liver fat without other etiologies such as infection, autoimmune conditions, hepatotoxic medications, and storage disorders (6). With the development of the economy, NAFLD has gradually become the leading cause of chronic liver disease in children and adolescents (7). NAFLD not only causes serious and progressive liver damage, but also parallels high rates of insulin resistance, metabolic syndrome, type 2 diabetes mellitus (T2DM), and cardiovascular diseases, which seriously lower the life quality and bring a huge burden to the social economy (8, 9). Our study has also shown that NAFLD, usually accompanied by metabolic syndrome, has a significant correlation with intrahepatic and extrahepatic cancers (10).

A systematic review of over 100,000 individuals found that NAFLD prevalence was up to 69.99% and NASH prevalence was 33.50% among the overweight population (11). Similarly, Li et al. (12) reported a NAFLD prevalence of 52.49% among obese participants and 39.17% among overweight/obese participants in children and adolescents, respectively, in their meta-analysis. Body mass index (BMI) is a well-known factor for NAFLD and is considered the most reliable predictor in both sexes (8, 13). Multiple studies have shown a dose-dependent link between BMI and NAFLD risk (2, 13–15), suggesting that NAFLD prevalence can be estimated from BMI rates.

Childhood overweight or obesity often raises the risk of adolescent NAFLD (14, 16, 17), which can lead to early death from liver-related complications. While meta-analyses have examined NAFLD in young people (12, 18), there is a shortage of large-scale epidemiological studies. Moreover, most research has not considered how BMI changes or impacts different life stages of NAFLD. There is a lack of longitudinal data on how BMI changes over time affect NAFLD/NASH prevalence in adolescents. In this study, we modeled and predicted NAFLD/NASH prevalence based on BMI to better understand the current and future burden in adolescents, enabling detailed comparisons.

## Materials and methods

### Definition of NAFLD/NASH

Based on the literature (19, 20), we employed the following criteria to determine whether an adolescent had NAFLD or NASH:

NAFLD, controlled attenuation parameter (CAP, an index reflecting the degree of fatty liver) ≥ 238 dB/m;

NASH, CAP≥238 dB/m with alanine aminotransferase (ALT, which indicates definite liver cell injury due to many causes) ≥ 29 IU/L (boys) or ALT≥19 IU/L (girls); or CAP≥238 dB/m accompanied by liver stiffness (E) ≥ 7.3 kilopascal (kpa).

### NHANES database

We download data from the NHANES database, which includes demographic data, questionnaire data, dietary data, examination data, laboratory data, and limited access data. The detailed NHANES study design and data are publicly available.^1^ From NHANES 2017-March 2020 Pre-Pandemic Examination Data, we obtained the data for body measures, laboratory assays, and liver Transient Elastography. The adolescents aged 12–18 with completed information on sex, age, BMI, ALT, aspartate transaminase (AST, an enzyme released when the liver or muscles are damaged), CAP, and liver stiffness (E), were enrolled in this study.

### Curve-fitting the correlation between BMI and risk of NAFLD/NASH

Four different models were developed to characterize the correlation between BMI and NAFLD/NASH prevalence for boys and girls. Curve-fitting method was employed to build the model, which was generated process as follows:

1. According to quantiles of BMI, we equally divided the enrolled adolescents into 8 groups, and then calculated the prevalence of NAFLD/NASH within each group;
2. We denoted $x_{i}$(i=1,…,12) as the median BMI within each group, and $y_{i}$ (i=1,…,8) as the corresponding disease prevalence. Based on these points ${\left\{\left(x_{i},y_{i}\right)\right\}}_{i=1}^{8}$, we used the curve-fitting method to find the best function, which can be used to estimate NAFLD/NASH prevalence for the population with a specific BMI value.

The estimated functions were as follows:

$$y=f\left(x\right)=\frac{a}{1+\exp\left\{\left(b-x\right)/c\right\}}$$

### Modeling NAFLD/NASH prevalence based on BMI

The prevalence of NAFLD/NASH was modeled based on BMI using the estimated functions, which were shown as follows:

1. NCD-RisC provided the BMI data^2^ for boys or girls of different ages at global, regional, or country levels. We obtained the prevalence of the adolescents with different BMIs, which were divided into five levels according to the degree of obesity, i.e., obesity, overweight, normal, underweight, and severely underweight. We denoted $l_{k}$ (k=1,…,5) and $r_{k}$ as the left and right boundary, respectively, for the BMI interval of the kth level of obesity. We additionally calculated the median BMI value $z_{k}=\left(l_{k}+r_{k}\right)/2$ for the latter analysis.
2. From the BMI data provided by NCD-RisC, we obtained the prevalence of five obesity levels in adolescents of different ages, genders, and from different countries or regions in the years 1975–2016. The $q_{k}$ (k=1,…,5) was denoted as the corresponding prevalence of NAFLD/NASH for adolescents from the kth level of obesity. The cumulative prevalence of NAFLD/NASH for the adolescents with five obesity levels at a specific age, sex, year, and region, was calculated using the following formula: $p=\sum_{k=1}^{5}q_{k}f\left(z_{k}\right)$. In this formula, $f\left(z_{k}\right)$ estimated the disease prevalence for the children from kth obesity level. By the weighted summation, the value p can serve as the estimated cumulative prevalence of NAFLD/NASH.

### Projecting NAFLD/NASH prevalence from 2017 to 2030 based on BMI

Based on the prevalence of NAFLD/NASH between 1975 and 2016, we used autoregressive integrated moving average (ARIMA) to project the prevalence for the latter years (21). ARIMA model is a widely used time-series forecasting method. It can help comprehend the data to forecast upcoming series points.

### Generation of estimated annual percentage change

The estimated annual percentage change (EAPC) was generated by the method described by Hankey *et al* (22). In brief, first, we assumed that the natural logarithm of NAFLD/NASH prevalence was submitted to linear distribution over time; second, Y = *α* + *β*X + *ε*, where Y = ln (prevalence), X = calendar year, and ε = the error term; and third, EAPC of prevalence was calculated by the formula 100× (exp[β]-1), while 95% CI was obtained from the linear regression model (22).

## Results

### Study population

**Figure 1:**
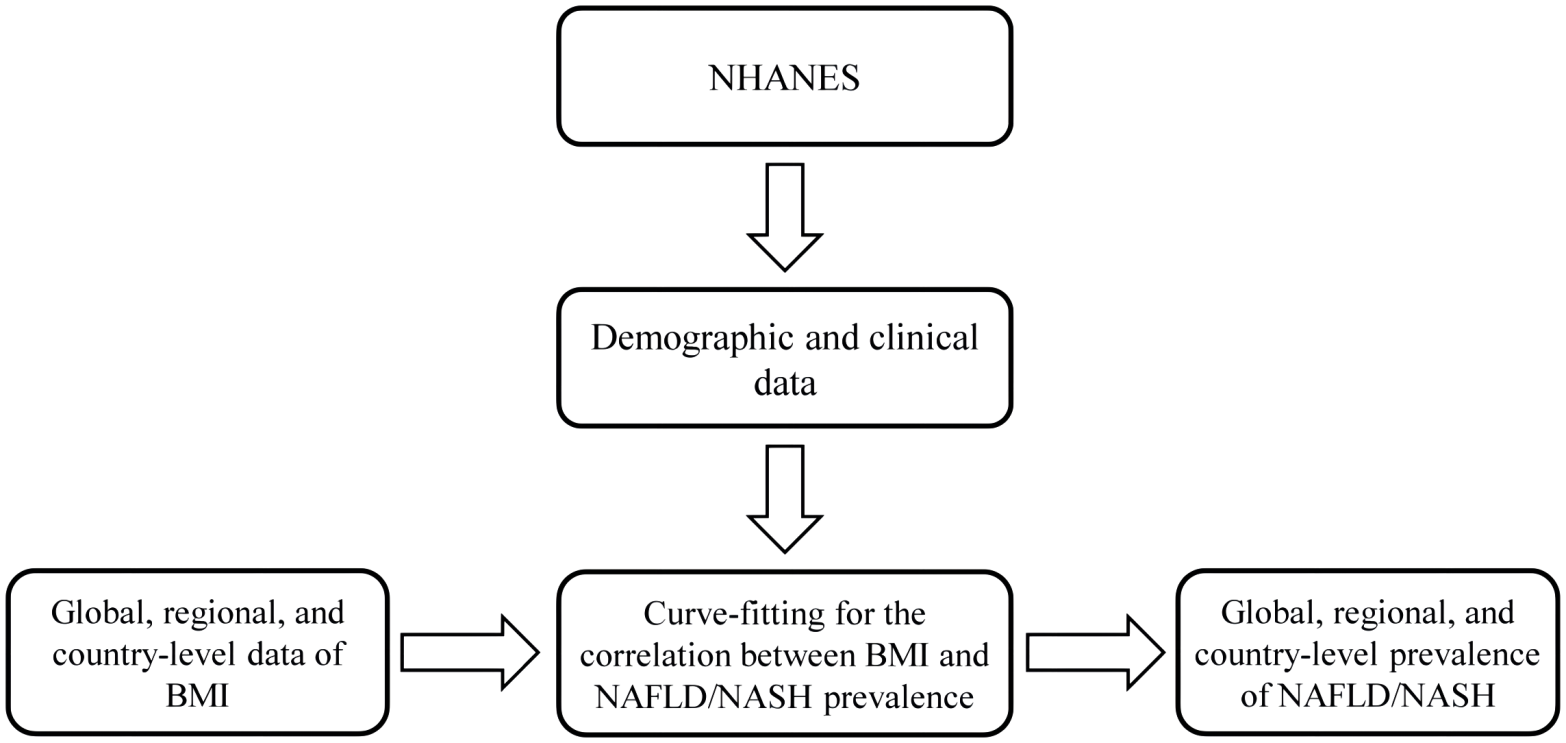
Algorithm of the study.

The algorithm of this study was shown in Figure 1. From NHANES 2017–March 2020 Pre-Pandemic Examination Data, a total of 1,348 individuals (717 boys, and 631 girls) aged 12–18 were enrolled in this study, who had completed information on sex, age, BMI, ALT, AST, CAP, and liver stiffness (E), see also Appendix pp. 1–28.

### Curve-fitting the correlation between BMI and risk of NAFLD/NASH

**Figure 2:**
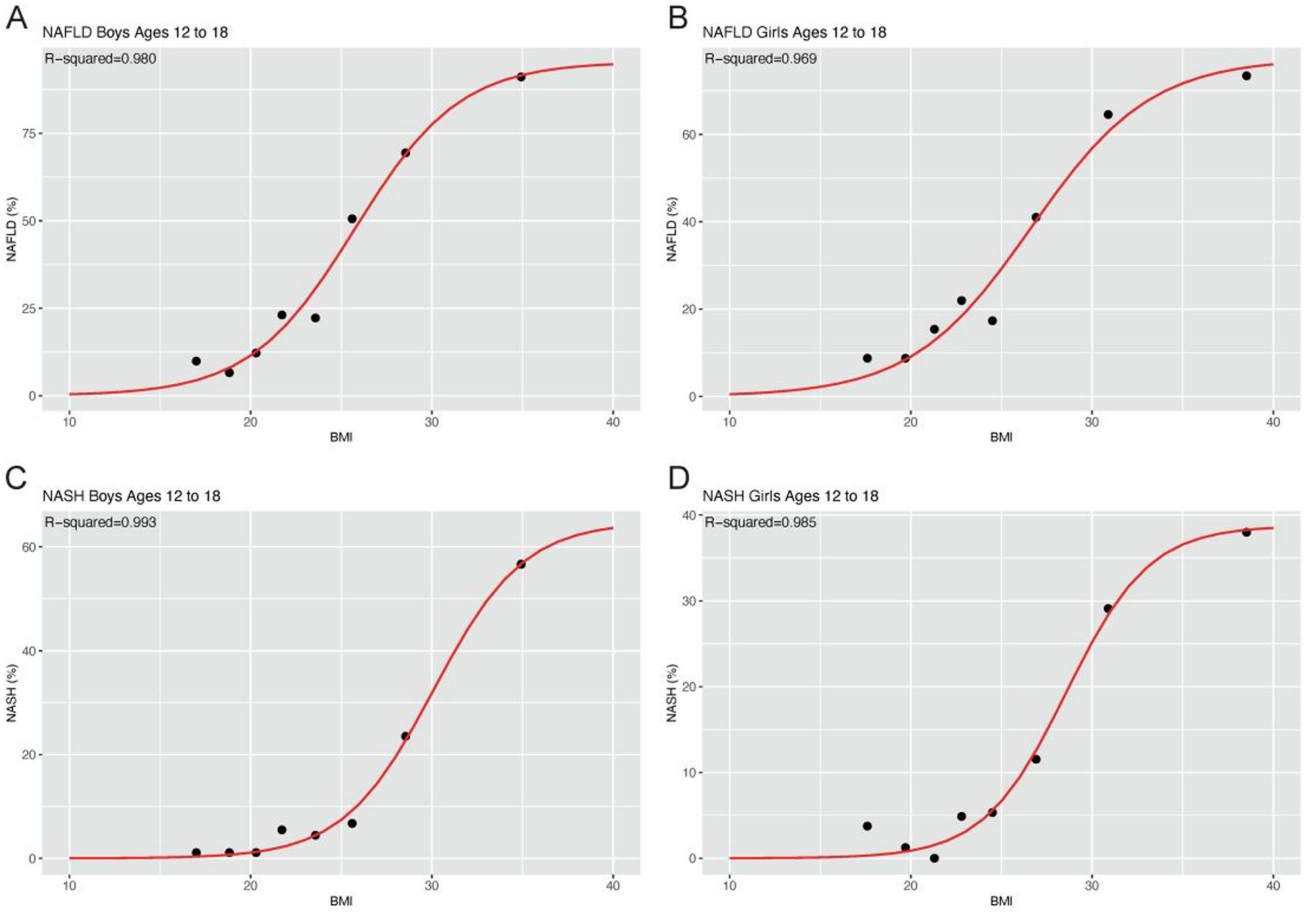
Fitting curves model the correlation between BMI and risk of NAFLD/NASH BMI and risk of NAFLD in boys **(A)** and girls **(B)**; BMI and risk of NASH in boys **(C)** and girls **(D)**.

The correlation between BMI and the risk of NAFLD/NASH was modeled by the curve-fitting method. The related parameters of the generated models were shown in Table 1 while the fitting curves were shown in Figure 2. The curves indicated that the risk of NAFLD/NASH increased with increasing BMI, although it was not a linear relationship. There was a high degree of consistency between the predicted and the actual risk of NAFLD/NASH. The lowest R^2^ was seen in the NAFLD model of girls, which was still as high as 0.969. The highest R^2^ was observed in the NASH model of boys, which was as high as 0.993.

**Table 1:** Parameters of the estimation models.

Model	a	b	c	R^2^
NAFLD, Boys	0.9542	25.7531	2.8929	0.980
NAFLD, Girls	0.7739	26.6447	3.2978	0.969
NASH, Boys	0.6169	29.7734	2.3269	0.993
NASH, Girls	0.3869	28.7782	2.3384	0.985

### Estimated global NAFLD/NASH prevalence

**Figure 3:**
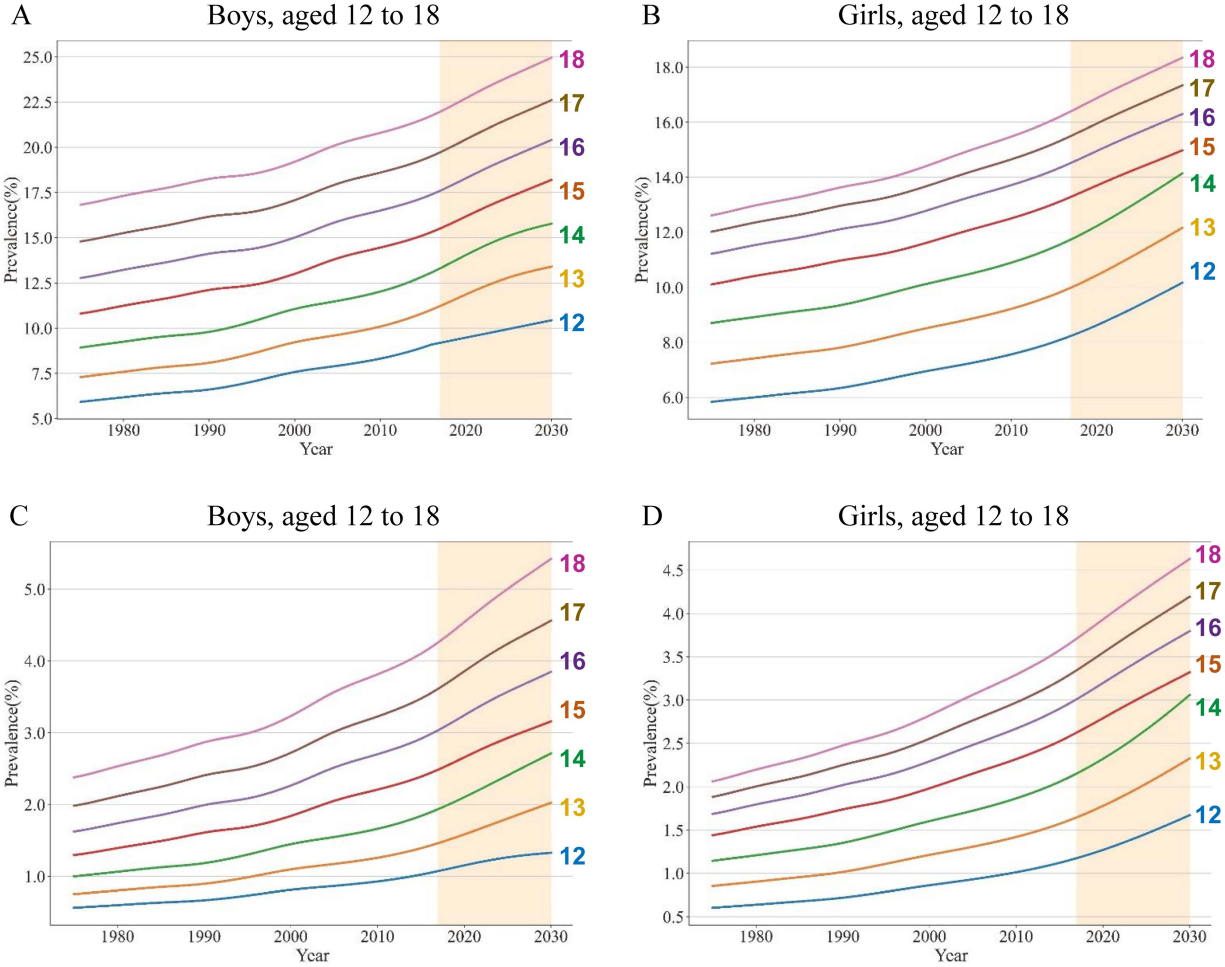
Estimated global prevalence of NAFLD/NASH in adolescents from 1975 to 2030, stratified by age Estimated global prevalence of NAFLD in boys **(A)** and girls **(B)**; Estimated global prevalence of NASH in boys **(C)** and girls **(D)**.

Overall, from 1975 to 2016, the global NAFLD/NASH prevalence increased year by year, and also increased by increasing age. The global NAFLD/NASH prevalence was higher in boys than that in girls. The estimated NAFLD prevalence for boys ranged from 9.10% (age 12) to 21.75% (age 18) in 2016, while that ranged from 8.13% (age 12) to 16.25% (age 18) for girls in 2016. The mean NAFLD prevalence was 15.31, and 12.68%, in boys, and girls aged 12–18, respectively (Figures 3A,B; Appendix pp. 29–40). For NASH, the estimated prevalence for boys ranged from 1.05% (age 12) to 4.18% (age 18) in 2016, while it ranged from 1.15% (age 12) to 3.64% (age 18) for girls in 2016. The mean NASH prevalence was 2.50, and 2.47%, in boys, and girls aged 12–18, respectively (Figures 3C,D; Appendix pp. 41–52). From 1975 to 2016, of interest, EAPC of NAFLD and NASH prevalence decreased with increasing age in both boys and girls. EAPC of NAFLD prevalence in boys ranged from 0.72% (age 18) to 1.16% (age 12) while that in girls ranged from 0.69% (age 18) to 0.92% (age 12; Appendix p 53). EAPC of NASH prevalence in boys ranged from 1.65% (age 18) to 1.77% (age 12), and in girls from 1.48% (age 18) to 1.68% (age 12; Appendix p 54). Notably, the EAPC of NASH was significantly higher than that of NAFLD. By 2030, global NAFLD prevalence is projected to rise to 24.96% in 18-year-old boys and 18.35% in 18-year-old girls (Appendix pp. 55–58). The global NASH prevalence was projected to increase to 5.42% in 18-year-old boys and 4.63% in 18-year-old girls by 2030 (Appendix pp. 59–62).

### Estimated regional NAFLD/NASH prevalence

**Figure 4:**
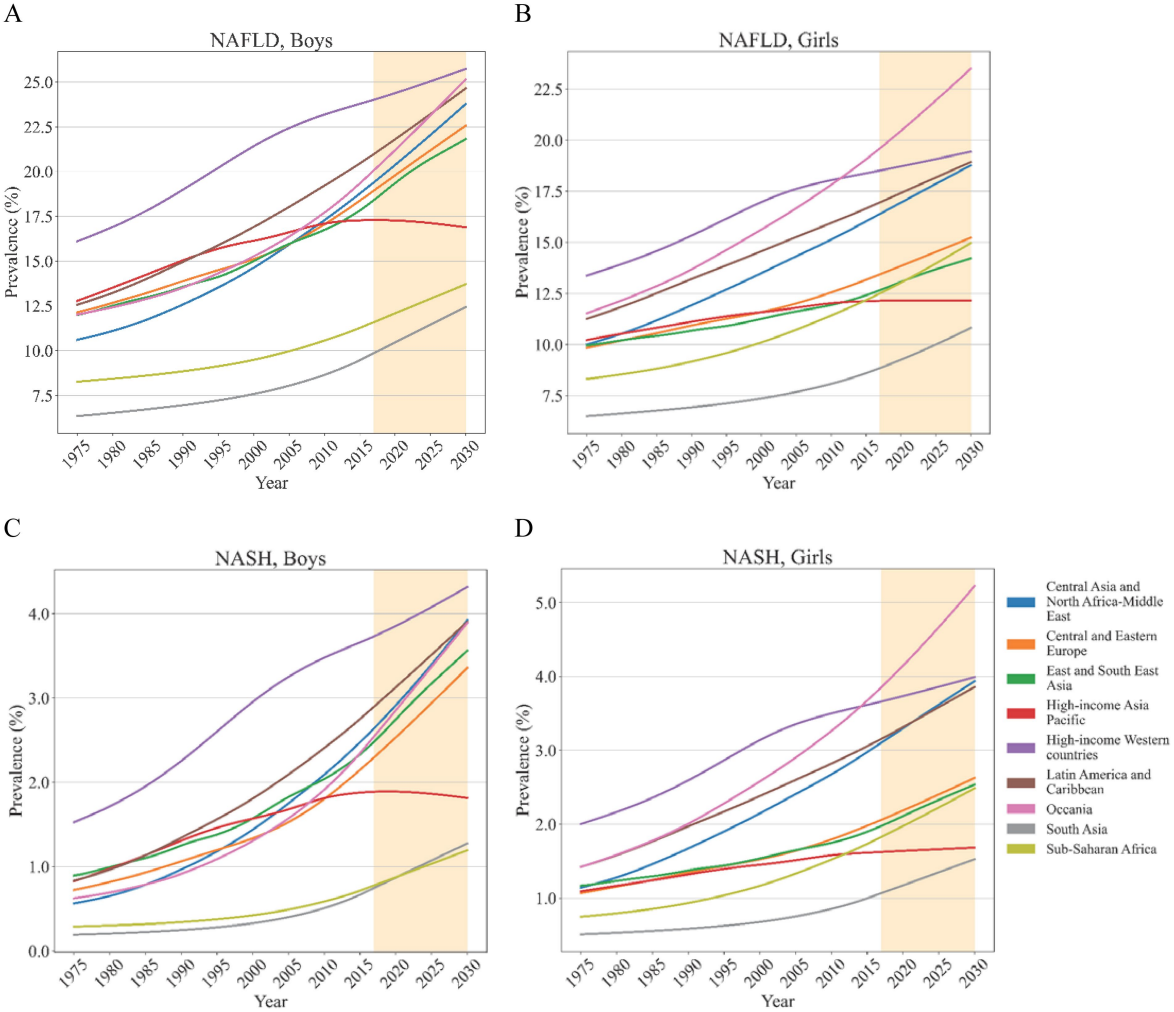
Estimated mean of regional NAFLD/NASH prevalence in adolescents from 1975 to 2030 Estimated mean of regional NAFLD prevalence in boys **(A)** and girls **(B)**; Estimated mean of regional NASH prevalence in boys **(C)** and girls **(D)**.

From 1975 to 2016, the prevalence of adolescents with NAFLD/NASH increased in most regions (Figure 4). In the past several decades, South Asia had the lowest prevalence of NAFLD and NASH in both boys and girls. High-income Western countries had the highest prevalence of NAFLD and NASH in both boys and girls. However, for girls, the prevalence of NAFLD in Oceania surpassed that in High-income Western countries after 2012. In girls, the prevalence of NASH in Oceania surpassed that in High-income Western countries after 2015. From then on until 2030, the girls in Oceania had the highest NAFLD/NASH prevalence (Figure 4). Fortunately, after reaching its peak in 2016, the NAFLD/NASH prevalence in High-income Asia Pacific gradually decreased until 2030 (Figure 4; Appendix pp. 63–300). Apart from Sub-Saharan Africa, which had a higher EAPC of NAFLD prevalence in girls than in boys, most regions had higher EAPC of NAFLD prevalence in boys than in girls. More than that, EAPC of NAFLD prevalence decreased with increasing ages in both boys and girls. Although boys consistently had higher EAPC of NASH prevalence than girls except for Sub-Saharan Africa, there was no apparent age-related bias in EAPC of NASH prevalence. Regional annual change of NAFLD prevalence in girls ranged from 0.31% (95% CI 0.30–0.32) in girls aged 17 from High-income Asia Pacific to 1.51% (1.50–1.52) in girls aged 12 from Oceania. The range for boys was from 0.44% (0.42–0.46) in the boys aged 18 from High-income Asia Pacific to 2.05% (2.04–2.07) in the boys aged 12 from Central Asia and North Africa-Middle East (Appendix pp. 301–302). For the EAPC of NASH prevalence in boys, it ranged from 1.00% (0.91–1.09) in boys aged 12 from High-income Asia Pacific to 3.16% (3.13–3.19) in boys aged 14 from Central Asia and North Africa-Middle East. While for the EAPC of NASH prevalence in girls, it ranged from 0.76% (0.74–0.78) in girls aged 17 from High-income Asia Pacific to 2.77% (2.73–2.81) in girls aged 12 from Sub-Saharan Africa (Appendix pp. 303–304).

In boys, the highest NAFLD/NASH prevalence in 2016 was estimated for the High-income Western countries whereas South Asia and Sub-Saharan Africa had the lowest NAFLD/NASH prevalence. This trend remains unchanged until 2030. For girls, High-income Western countries also had the highest NAFLD prevalence while that was exceeded by Oceania in 2012 (Appendix pp. 305–338). The changing trend of NASH prevalence was similar to that. Girls from South Asia consistently had the lowest NAFLD/NASH prevalence from 2016 to 2030. In the same period, NAFLD/NASH prevalence was estimated to experience a sustained growth for both boys and girls in most regions apart from High-income Asia Pacific, which ranged from virtually no change to a slight decline (Appendix pp. 339–371). Regional NAFLD prevalence, stratified by age, was supplied in Supplementary Figure 1. Regional NASH prevalence, stratified by age, was supplied in Supplementary Figure 2.

### Estimated NAFLD/NASH prevalence at country/region level

**Figure 5:**
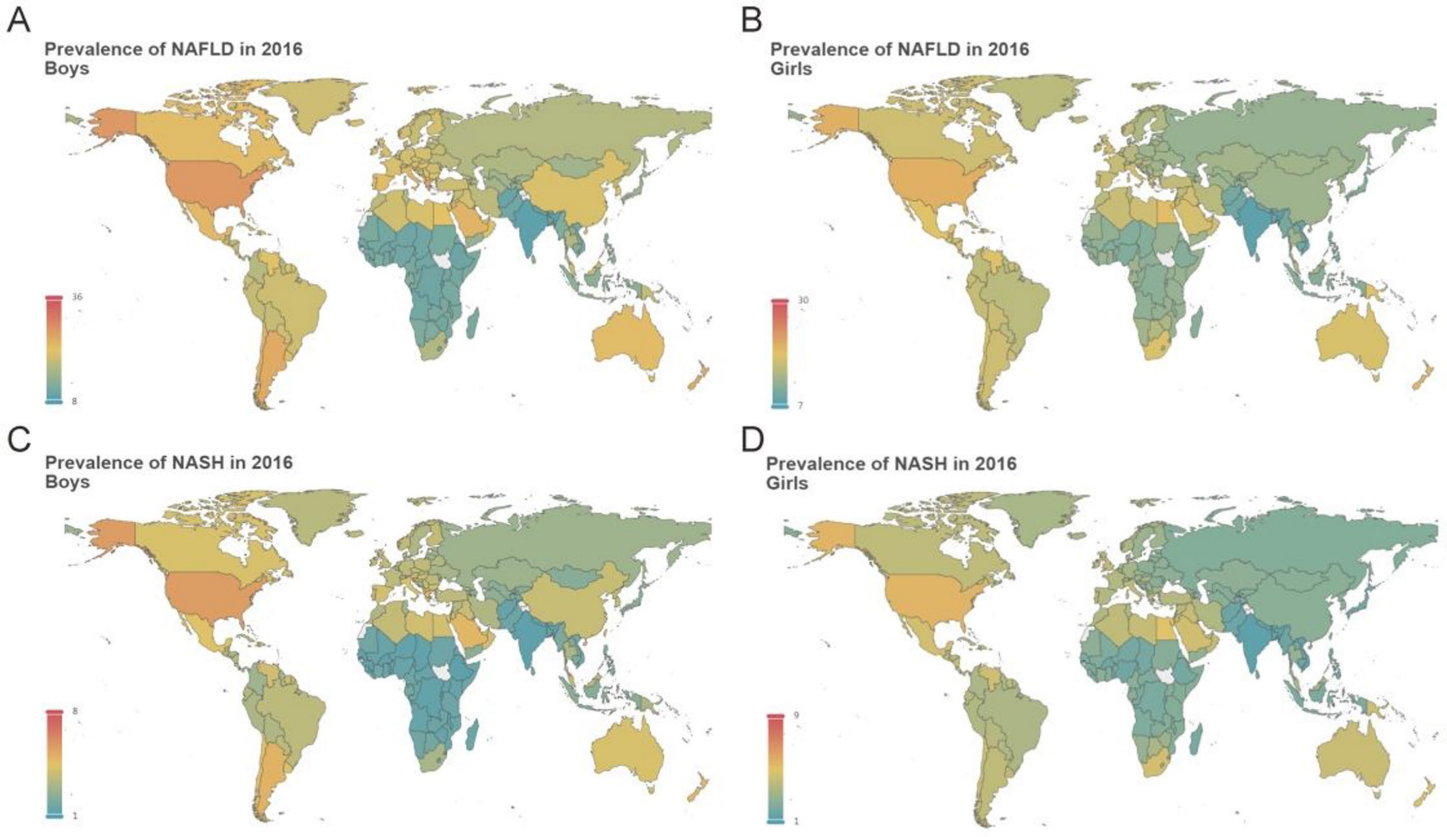
Estimated mean prevalence of adolescent NAFLD/NASH at country or region level in 2016 Estimated mean prevalence of adolescent NAFLD at country or region level in boys **(A)** and girls **(B)**; Estimated mean prevalence of adolescent NASH at country or region level in boys **(C)** and girls **(D)**.

In 2016, the NAFLD prevalence differed dramatically between countries or regions. For boys, Nauru, Cook Islands, and Palau, located in the Pacific, showed the highest estimated burden of NAFLD. In boys, the highest NAFLD prevalence was estimated for Nauru at 34.45% whereas the lowest NAFLD prevalence was estimated for India (9.34%), followed by Nepal, and Afghanistan (Figure 5A). Similar to that, girls from Nauru (29.34%), Palau, and American Samoa, had the highest NAFLD prevalence while India (8.35%), Viet Nam, and Nepal, had the lowest NAFLD prevalence (Figure 5B; Appendix pp. 372–2956). Not surprisingly, country or region-specific burden of NASH prevalence was roughly similar to NAFLD prevalence (Figures 5C,D; Appendix pp. 2957–5541). From 1975 to 2016, most countries or regions apart from Belgium experienced significantly increasing EAPC of NAFLD/NASH prevalence. In boys, the highest increasing EAPC of NAFLD prevalence was estimated for South Africa (3.64%, aged 12), and China (3.55%, aged 12), whereas the lowest increasing EAPC of NAFLD prevalence was estimated for Belgium, which ranged from virtually no change to slightly decline (Appendix 5,542–5,589). For boys, the highest increasing EAPC of NASH prevalence was estimated for South Africa (5.74%, aged 12), and China (5.38%, aged 12), whereas the lowest increasing EAPC of NASH prevalence was estimated for Belgium (0.11%, aged 12), and Andorra (0.32%, aged 12; Appendix 5,590–5,637). In girls, the highest increasing EAPC of NAFLD prevalence was recorded in South Africa (3.30%, aged 12), followed by Lesotho (2.70%, aged 12), whereas the lowest increasing EAPC of NAFLD prevalence was estimated for Belgium, which showed slightly declined (Appendix 5,542–5,589). For girls, the highest increasing EAPC of NASH prevalence was estimated for South Africa (5.67%, aged 12), and Lesotho (4.91%, aged 12), whereas the lowest increasing EAPC of NASH prevalence was estimated for Belgium, which remained virtually no change (Appendix 5,590–5,637).

Nationally, NAFLD prevalence was high throughout Pacific Island Countries, reaching an amazing 53.04% (Niue, aged 18) among boys and 40.06% (Tonga, aged 18) among girls in 2030. The highest NASH prevalence was estimated for Cook Islands (16.69%, aged 18), Niue (16.65%, aged 18), and Palau (16.04%, aged 18) among boys while the highest NASH prevalence was estimated for Tuvalu (14.62%, aged 18), Cook Islands (14.57%, aged 18), and Tonga (14.33%, aged 18) among girls. In 2030, the lowest NAFLD prevalence was estimated for Ethiopia (6.59%, aged 12), Guinea (7.06%, aged 12), and Chad (7.10%, aged 12) among boys while the lowest NAFLD prevalence was estimated for India (6.23%, aged 12), Viet Nam (7.13%, aged 12), and Japan (7.26%, aged 18) among girls (Appendix 5,638–6,391). In 2030, Ethiopia (0.64%, aged 12), Rwanda (0.67%, aged 12), and Niger (0.68%, aged 12), were estimated to have the lowest NASH prevalence among boys while India (0.81%, aged 12), Japan (0.83%, aged 12), and Cambodia (0.95%, aged 12), was estimated to have the lowest NASH prevalence among girls (Appendix 6,392–7,145). Interactive web pages for mean NAFLD/NASH prevalence, age-stratified NAFLD prevalence, and age-stratified NASH prevalence at the country/region level were supplied in Supplementary Files 1–3, respectively.

## Discussion

With the aid of NHANES 2017-March 2020 and the NCD-RisC dataset, we have described a panoramic view of the NAFLD/NASH prevalence among global adolescents, which allowed for multiple comparisons across sexes, ages, regions, or countries, and timelines. Our main findings include the following:

(i) Sigmoidal fitting models precisely described the correlation between the BMI and adolescent NAFLD/NASH prevalence. (ii) The NAFLD/NASH prevalence increased with increasing BMI between the ages of 12 and 18 in both boys and girls. (iii) The global adolescent NAFLD/NASH prevalence has increased from 1975 to 2016 and will maintain the increasing trend between 2017 and 2030. (iv) The NAFLD/NASH prevalence gradually rose with increasing age. (v) Boys tended to have higher NAFLD/NASH prevalence than that in girls. (vi) For boys, High-income Western Countries had the highest NAFLD/NASH prevalence, which was also true for girls before 2012. However, after 2012, the NAFLD/NASH prevalence for girls in Oceania surpassed that of High-income Western Countries. By contrast, both South Asia and Sub-Saharan Africa exhibited relatively low adolescent NAFLD/NASH prevalence. Despite having an increased burden of metabolic risk factors, African Americans tend to have a lower risk of NAFLD compared with Hispanic and White Americans. Underlying influencing factors such as genetic factors and gut microbiota associated with NAFLD in sub-Saharan Africa may contribute to this apparent paradox (23).

Several studies have revealed a dose-dependent relationship between BMI and the risk of NAFLD (13, 15). The burden of NAFLD increases proportionally with increasing BMI in most populations (1). However, the dose-dependent relationship between BMI and NAFLD burden was S-shaped, as shown in our estimation models, rather than a linear pattern. The S-shaped correlation between BMI and NAFLD burden exactly serves as the basis of building our estimation model for NAFLD/NASH prevalence. It should be pointed out that lean NAFLD is not necessarily ruled out in the case of employing our estimation model. The likelihood of developing NAFLD/NASH is lower in the case of low BMI, which is consistent with clinical practice.

This study employed a sigmoidal fitting curve to describe the relationship between BMI and adolescent NAFLD/NASH prevalence. The sigmoidal fitting curve was proposed by Venegas et al. (24). The curve consists of relative parameters to shape the lower bound of the dependent variable (i.e., NAFLD/NASH prevalence), the distance between the lower and the upper bound of the dependent variable (from 0 to 100% in this case), the inflection point of the curve, and the dose-effect relationship between the independent variable and dependent variable. In this study, the sigmoidal fitting curve perfectly simulated the relationship between BMI and NAFLD/NASH prevalence, which was proven by the near-perfect R^2^. Our prediction model possessed impressive accuracy. For example, the United States was estimated to have an adolescent NAFLD prevalence of 23.90% based on our prediction model, which was very close to the reported adolescent NAFLD prevalence of 24.16% by Ciardullo et al. (25). The estimated NAFLD prevalence in the United Kingdom was 19.27%, which was also highly consistent with the reported adolescent NAFLD prevalence of 20.7% by Abeysekera et al. (2). A case–control study indicated that 15.77% of Chinese children aged 7–18 years were diagnosed to have NAFLD (26), which was similar to the NAFLD prevalence of 16.96% estimated by our prediction model.

As shown in Figure 3, the burden of NAFLD/NASH became heavier with increasing age. Although several meta-analyses have been implemented to evaluate the prevalence of adolescent NAFLD (12, 27), age-related differences in the NAFLD/NASH prevalence have not been fully discussed. However, several population-based studies have demonstrated that increased age was associated with a significantly increased risk of NAFLD (3, 28, 29). Similar age-related difference in the NAFLD/NASH prevalence was estimated according to our sigmoidal fitting models. In addition, a higher prevalence of NAFLD/NASH was estimated by our fitting curves in boys than that in girls. In line with this sexual dimorphism, the male-predominant trend has been observed in cumulating pediatric NAFLD studies (2, 25, 27–29).

According to our estimation model, the global burden of NAFLD/NASH in terms of disease prevalence underwent a continuous and marked increase between 1975 and 2016. Additionally, this upward trend will continue until 2030. Multiple studies have also indicated that the burden of NAFLD originated from adults and adolescents (30–32), and is continuously increasing globally. Undoubtedly, the NAFLD/NASH prevalence is likely to increase concomitantly with the growing BMI level. Besides growing BMI, increasing prevalence of obesity and T2DM, and aging populations, may also act as important drivers for increasing global NAFLD burden (33). Over the past three decades, the pediatric NAFLD burden has increased with an estimated annual change of 1.35%, driven in part by rising obesity levels (34). In addition, dysfunctional glucose and lipid metabolic pathways, propelled by the growing global prevalence of obesity and T2DM, are most likely behind the expanding population of NAFLD (35).

With persistent efforts to prevent and treat hepatitis B and C, NAFLD has become the leading cause of chronic liver disease while NASH is expected to be the leading cause of cirrhosis. Weight gain in childhood or late adolescence usually leads to a greater risk of NAFLD than weight gain in late adulthood (36). Increment BMI in adolescence would increase the risk of cirrhosis and liver cancer in adulthood (17, 37). Another study from Sweden also indicates that pediatric and young adult patients with NAFLD have substantially higher rates of causes, including cancer, liver, and cardiometabolic-specific mortality (38). Given that adolescent NAFLD/NASH may lead to serious consequences, prevalence modeling can inform the public and professionals about the coming disease burden associated with NAFLD and NASH, and help to take targeted preventive measurements. As indicated by our estimation models, excess BMI is a critical while reversible risk factor for NAFLD. It highlights the possibility of reducing excess BMI by taking effective strategies, including dietary patterns and exercise programs, to prevent and treat NAFLD/NASH. Given the projected increase in adolescent NAFLD/NASH prevalence, specific public health strategies or interventions to address this growing issue would have significant practical value. Lifestyle interventions, including diet (i.e., Mediterranean diet) and physical activity, are the main public health strategies for losing weight (39).

Our estimation model’s limitation is the assumption that BMI consistently correlates with NAFLD/NASH prevalence, regardless of special physical features like abdominal obesity, region, or ethnicity. However, Asians with BMIs under 25 kg/m^2^ may develop “lean” or “non-obese” NAFLD (40). Studies indicate that waist-to-height ratio might predict NAFLD as effectively, or better, than BMI, especially in adolescents (41, 42). Further research is required to confirm and expand these findings. Our correlation model between BMI and NAFLD/NASH, based on the predominantly Caucasian NHANES 2017-March 2020 data, likely has greater accuracy for Caucasian populations. Another limitation is our use of liver Transient Elastography instead of the more common ultrasound for detecting NAFLD/NASH in population studies. However, liver Transient Elastography offers more objective measurements with less inter-observer bias.

## Data Availability

The original contributions presented in the study are included in the article/Supplementary material, further inquiries can be directed to the corresponding authors.

## Footnotes

^1^ https://www.cdc.gov/nchs/nhanes/

^2^ https://ncdrisc.org/data-downloads-adiposity-ado.html

## Supplementary material

Supplementary Presentation 1 Appendix: Corresponding data of Figure 2, Figure 3, Figure 4, and Figure 5.

Supplementary Figure 1 Regional NAFLD prevalence, stratified by age.

Supplementary Figure 2 Regional NASH prevalence, stratified by age.

Supplementary File 1 Interactive web pages for average NAFLD/NASH prevalence at the country/region level.

Supplementary File 2 Interactive web pages for NAFLD prevalence at the country/region level, stratified by age, and sex.

Supplementary File 3 Interactive web pages for NASH prevalence at country/region level, stratified by age, and sex.
