# Supplementary figures and images for: A significant and persistent rise in the global burden of adolescent NAFLD and NASH estimated by BMI
Source: Front Public Health. 2024 Oct 25;12:1437432. doi: 10.3389/fpubh.2024.1437432 (PMC11544631; doi:10.3389/fpubh.2024.1437432)

# Boys, 12

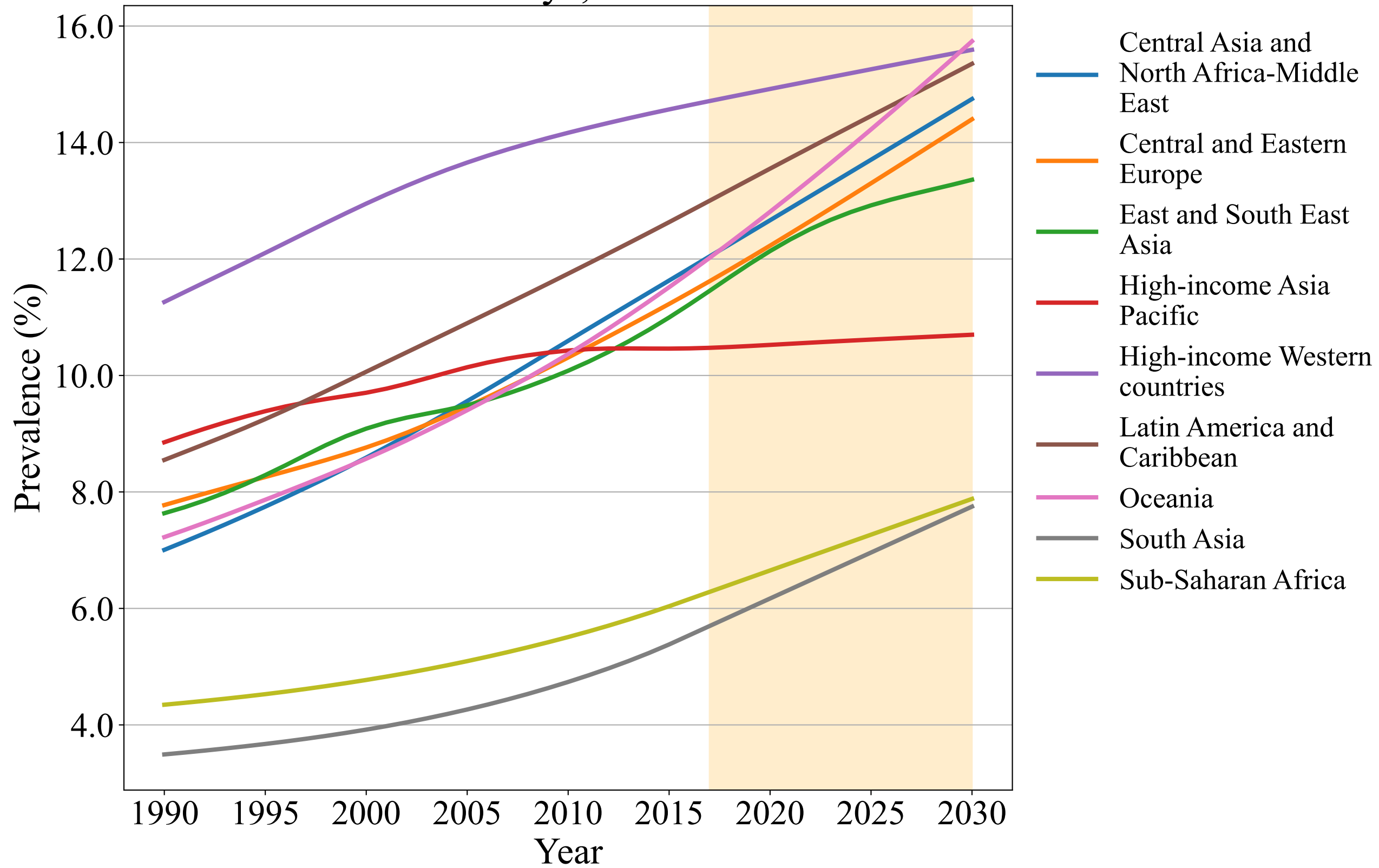

# Boys, 13

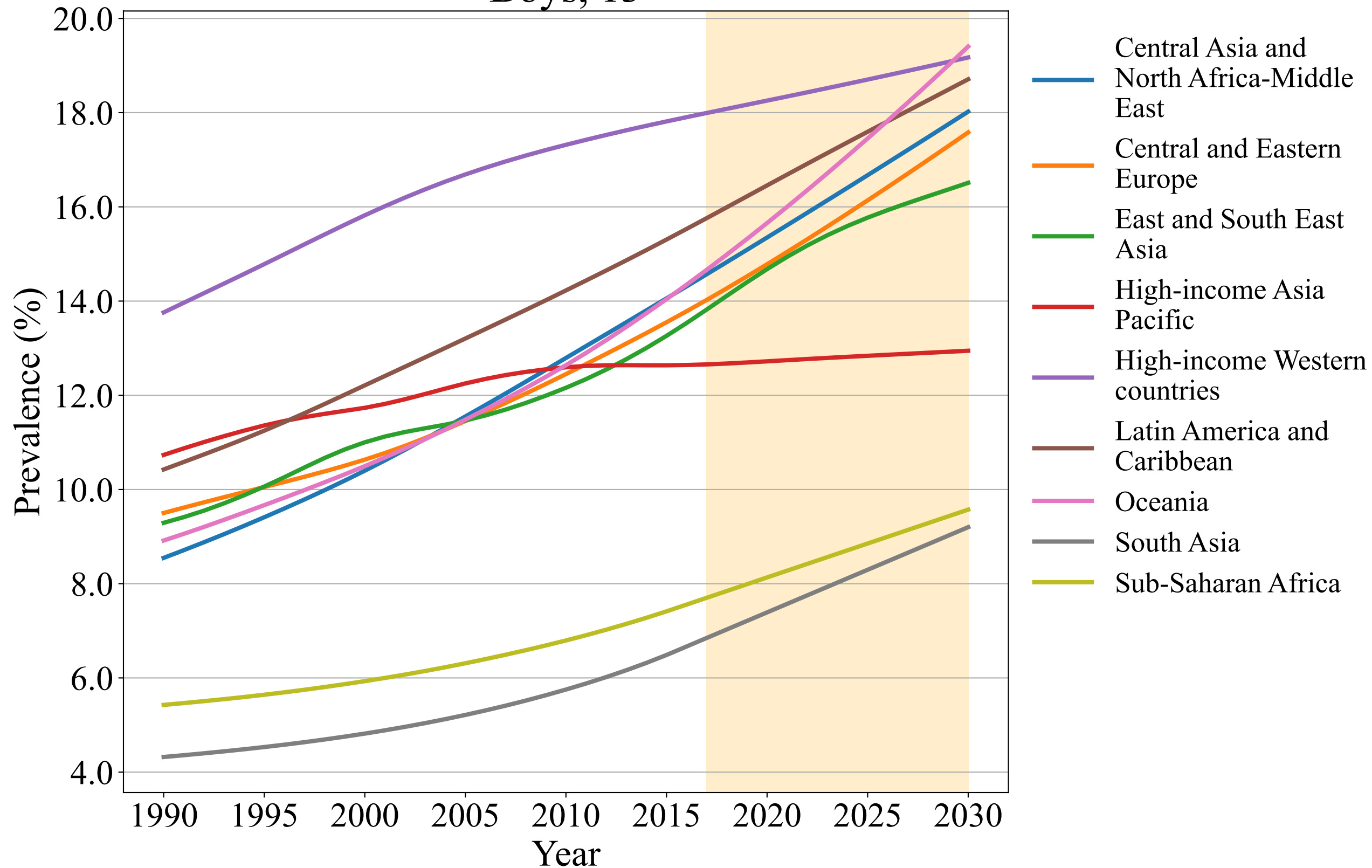

# Boys, 14

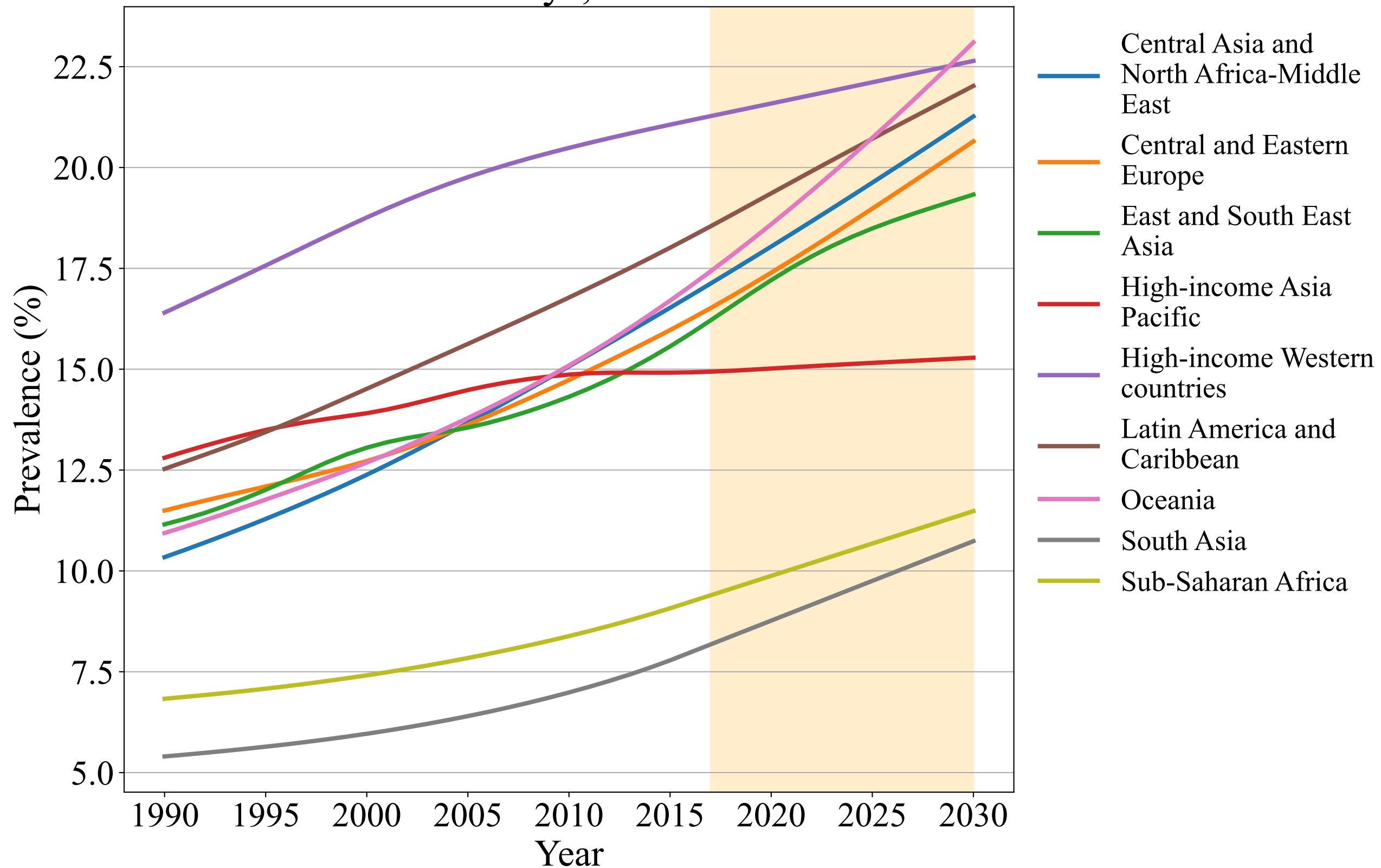

# Boys, 15

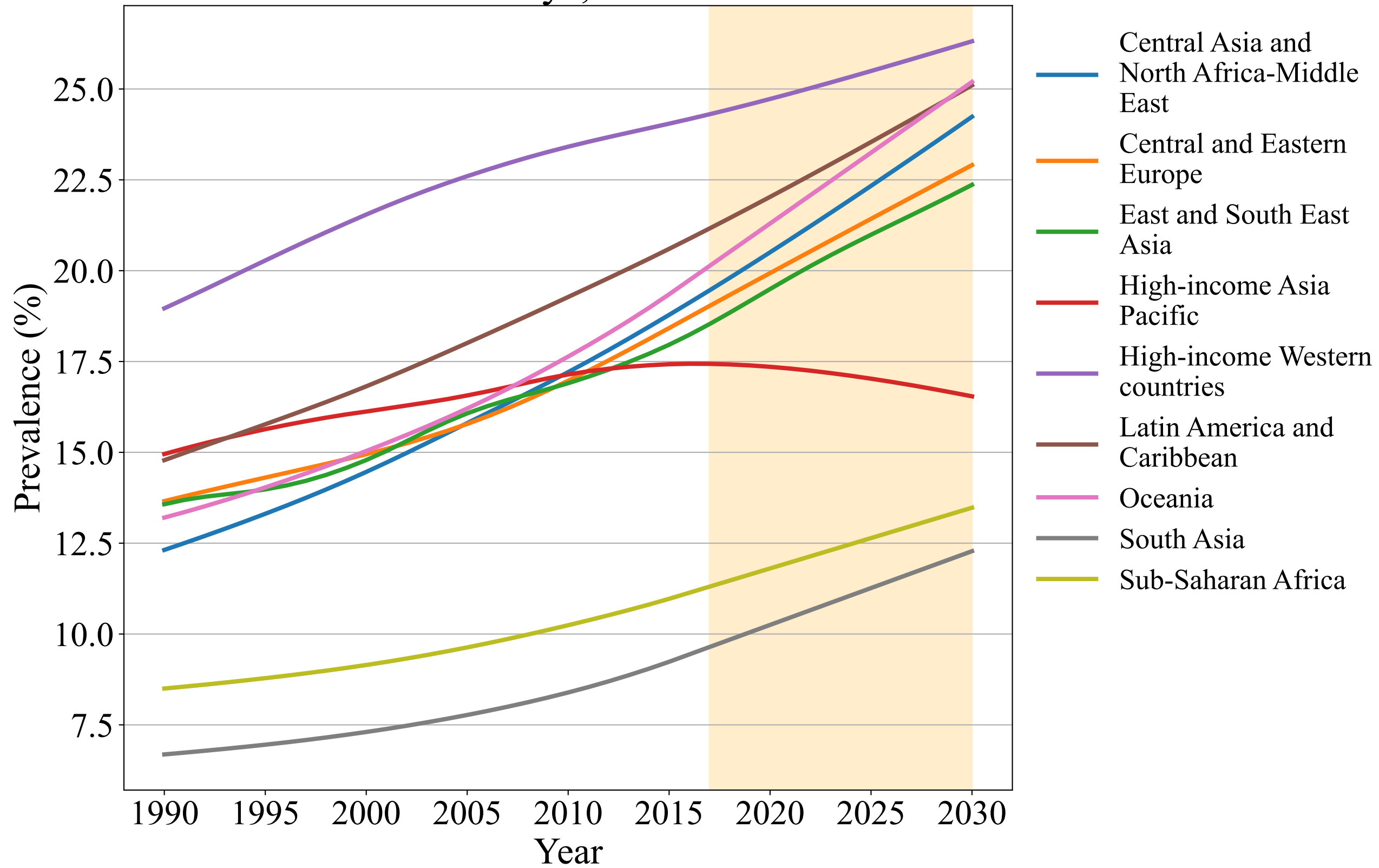

# Boys, 16

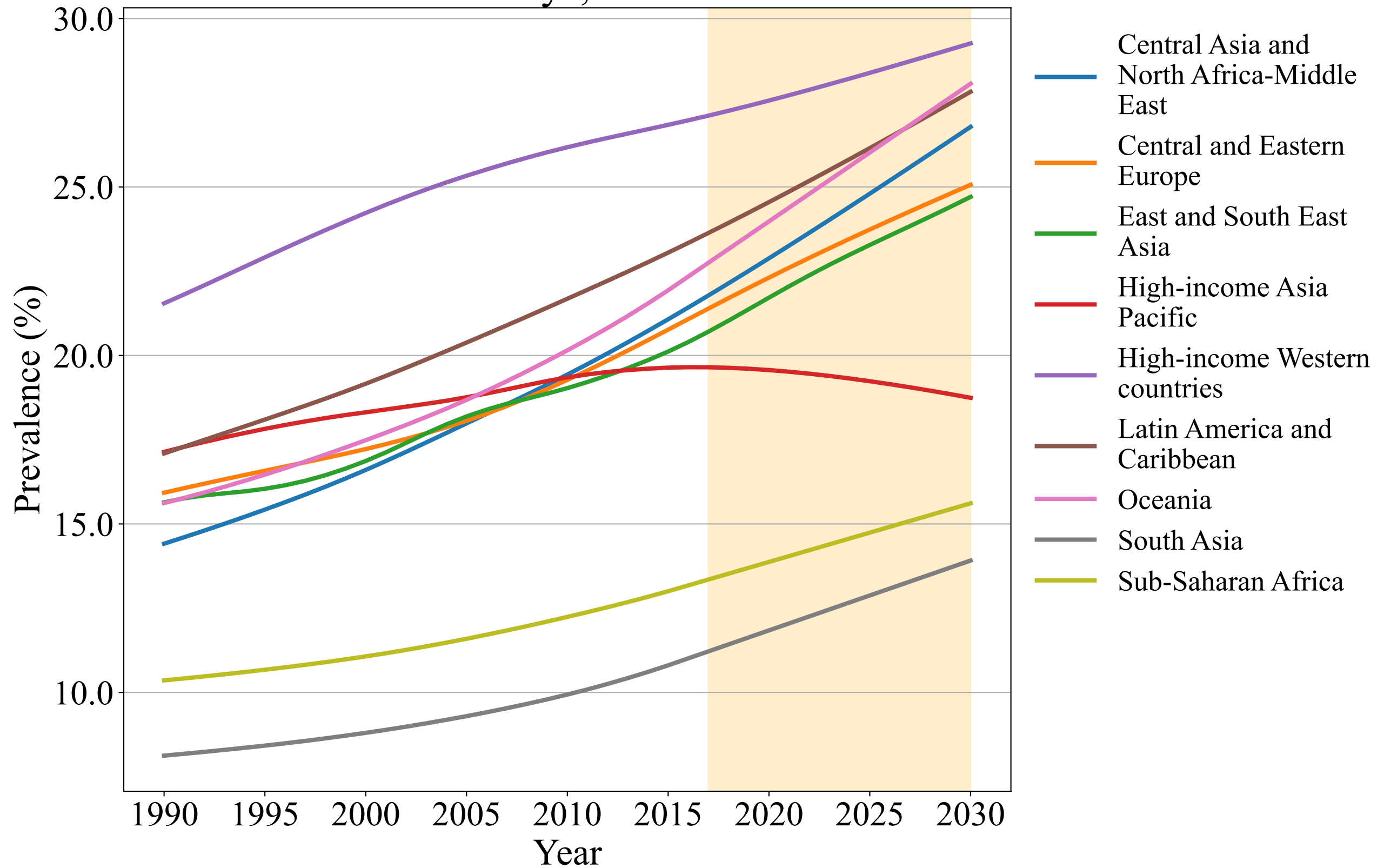

# Boys, 17

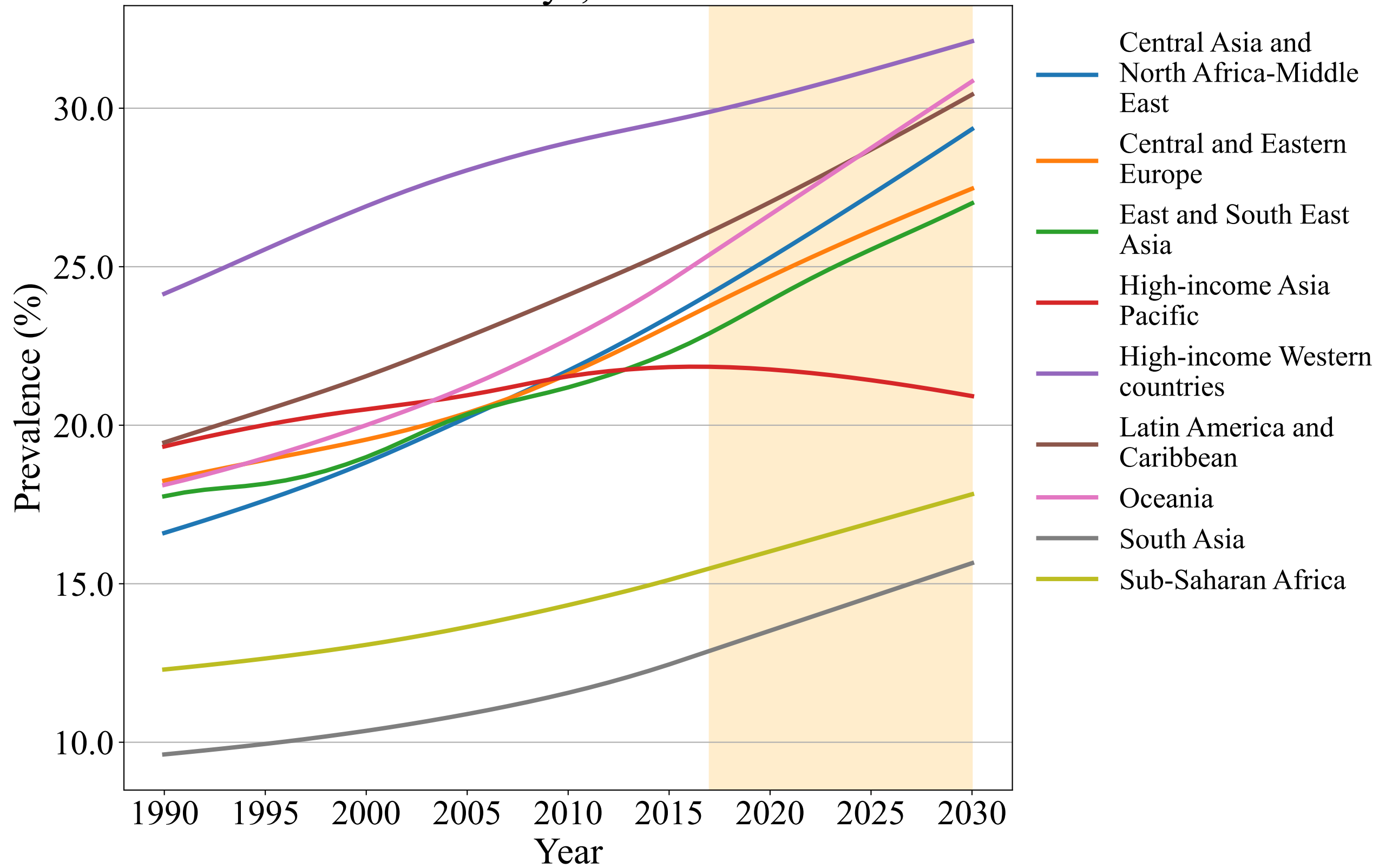

# Boys, 18

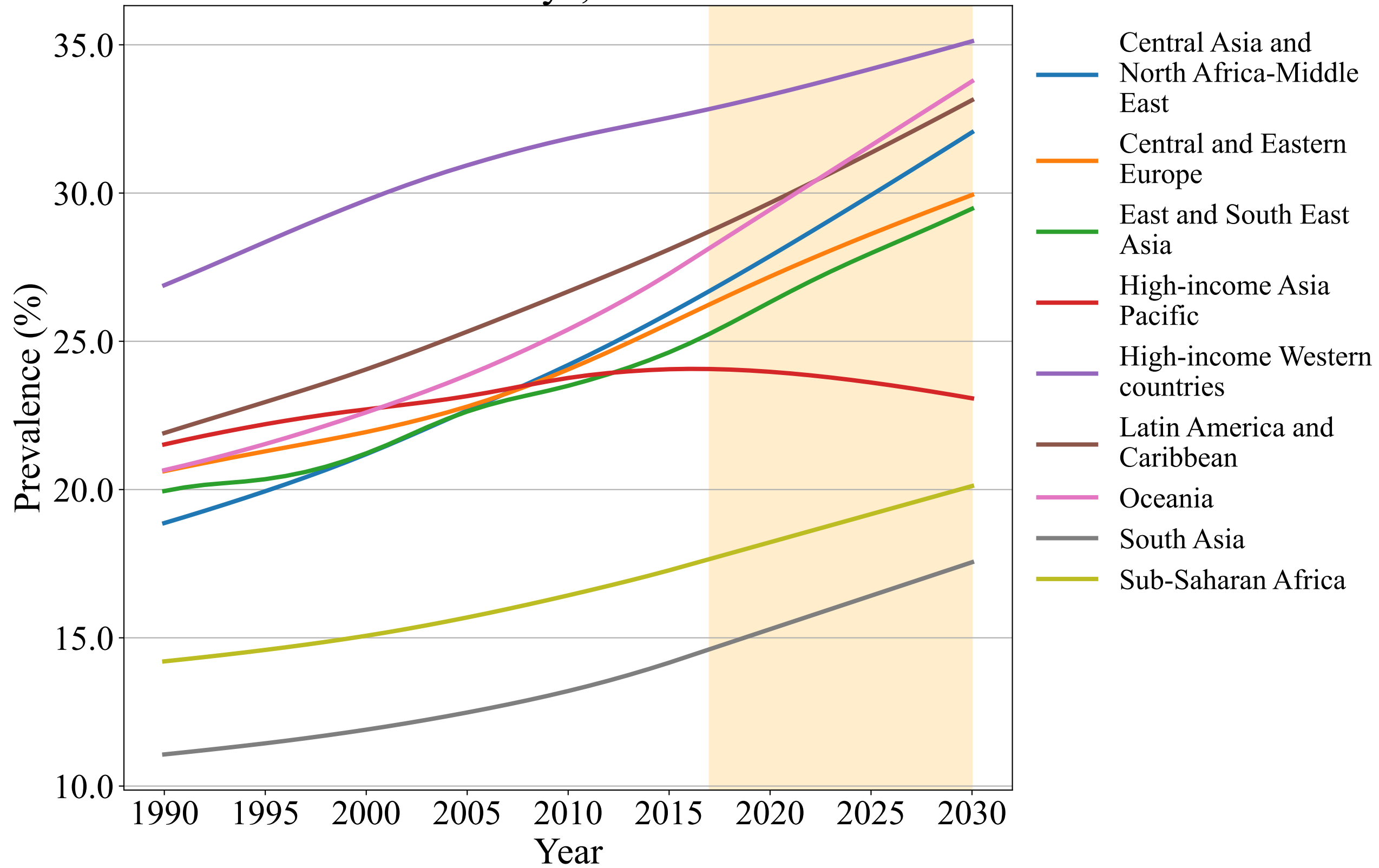

## Girls, 12

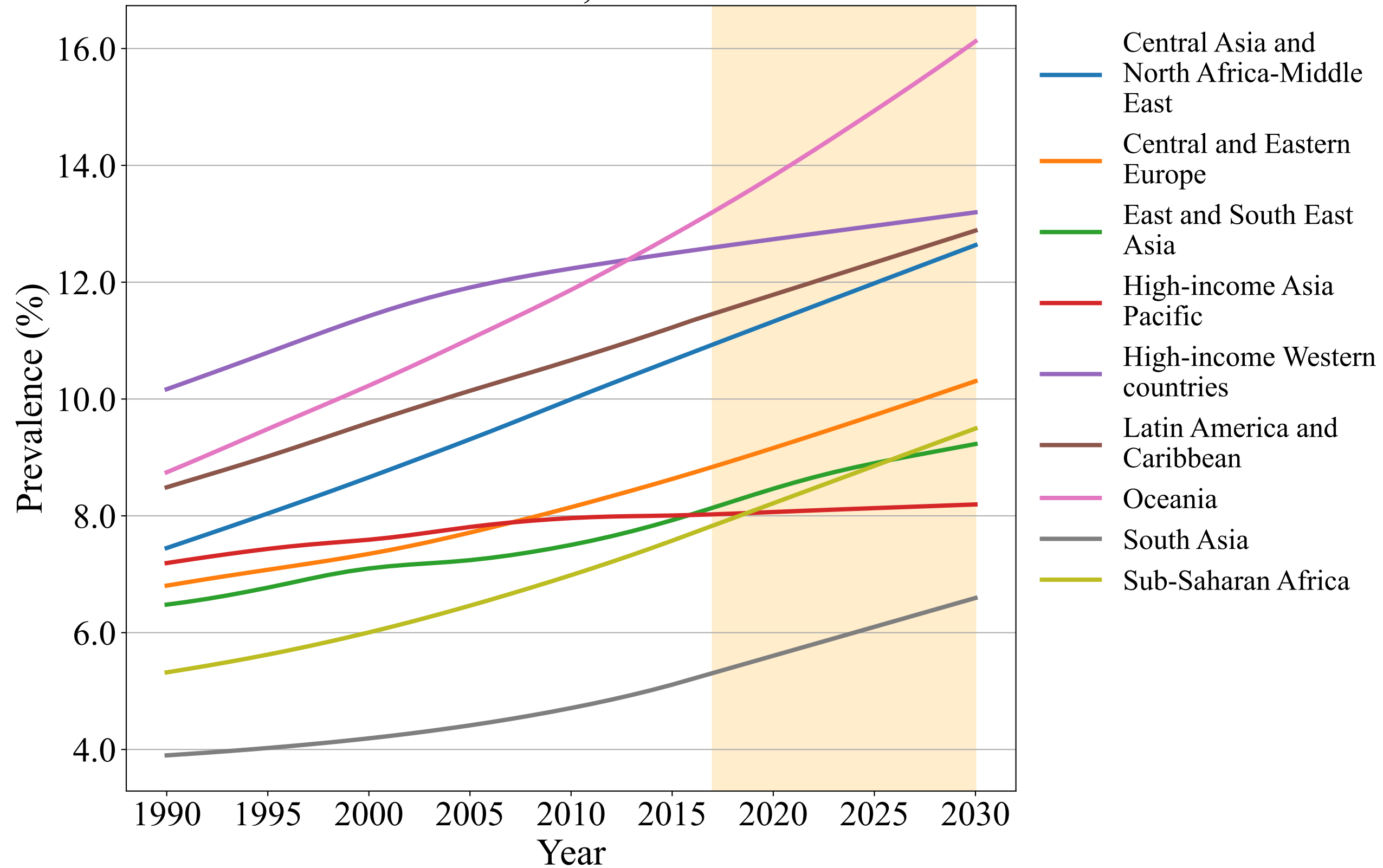

# Girls, 13

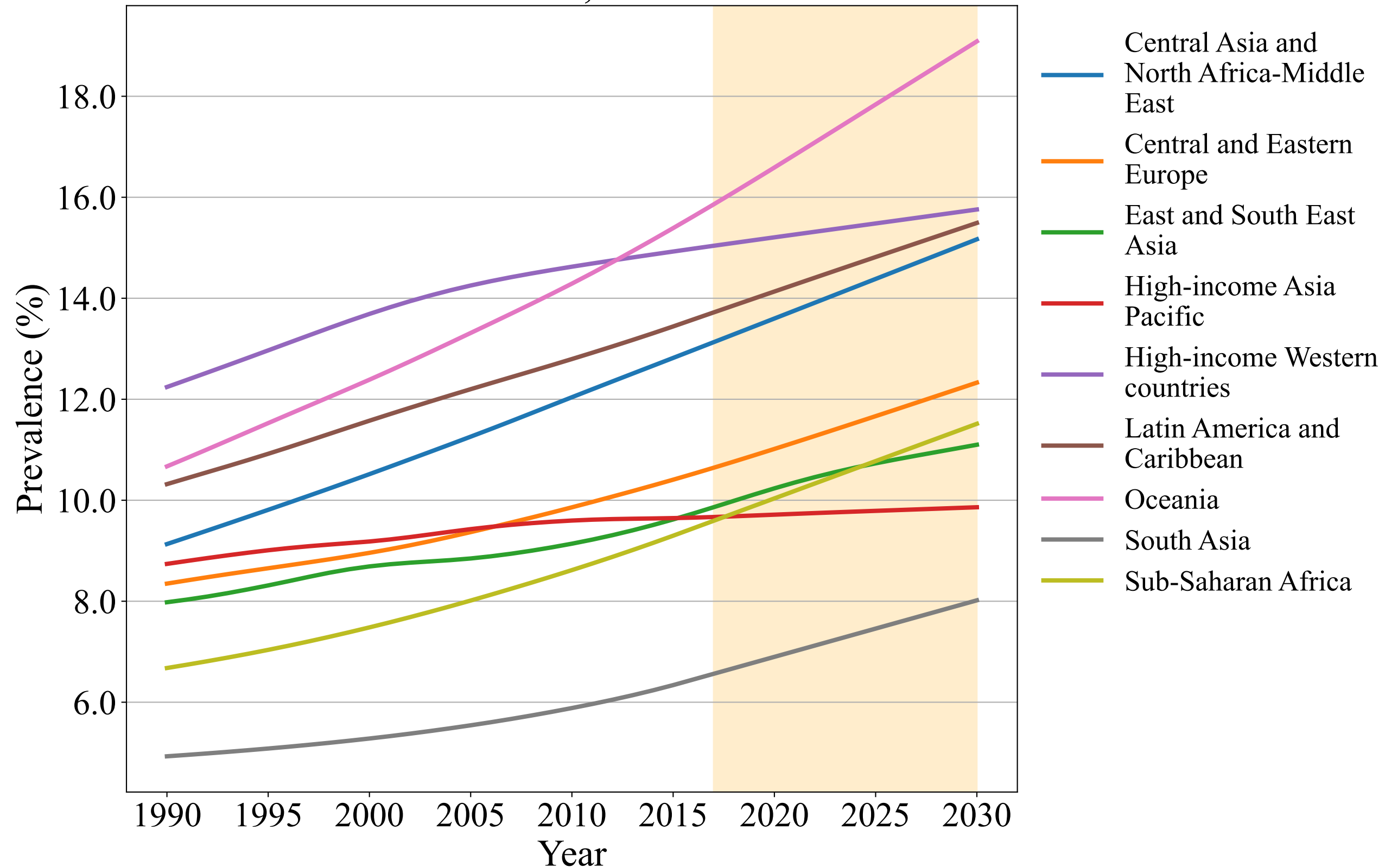

# Girls, 14

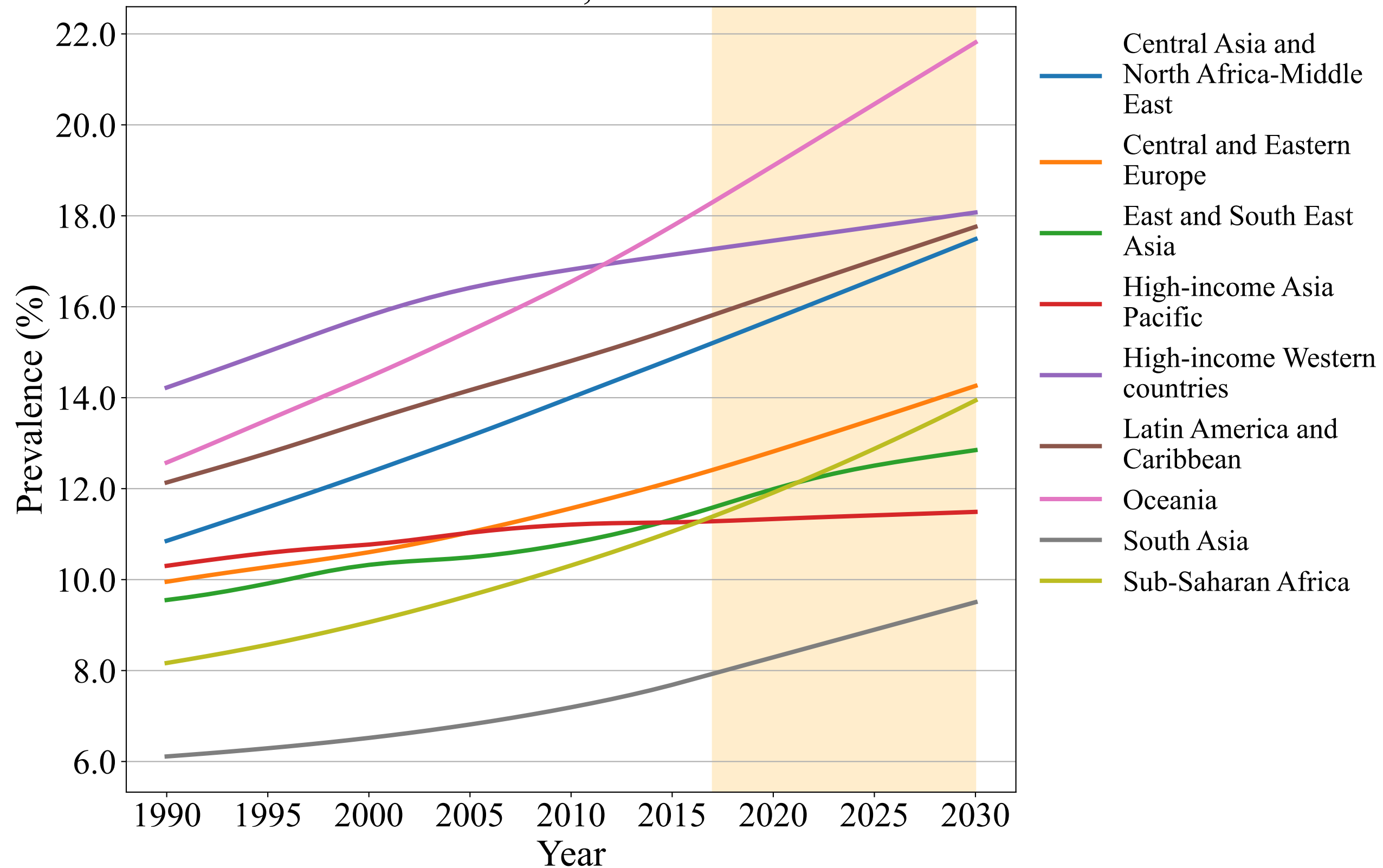

# Girls, 15

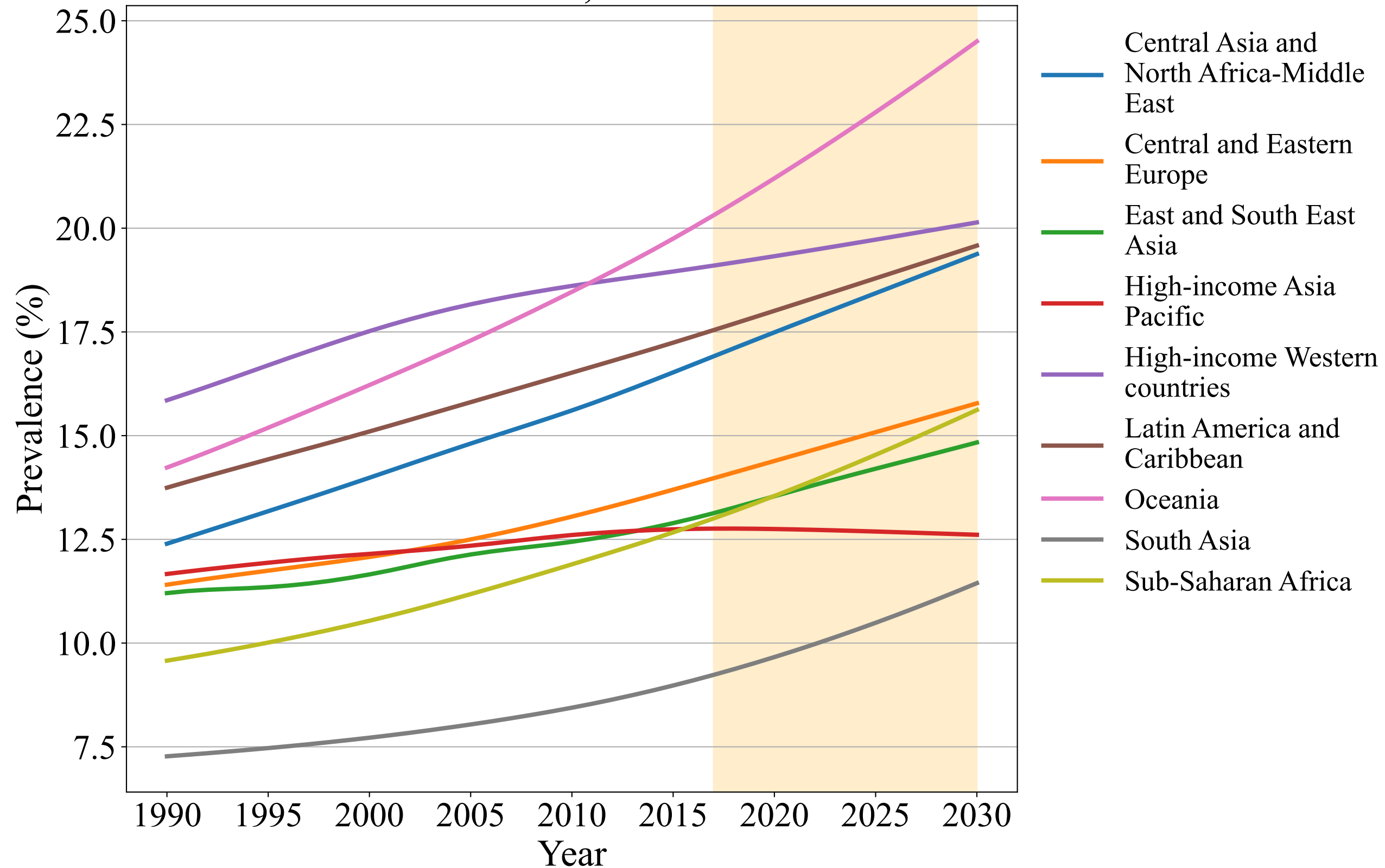

# Girls, 16

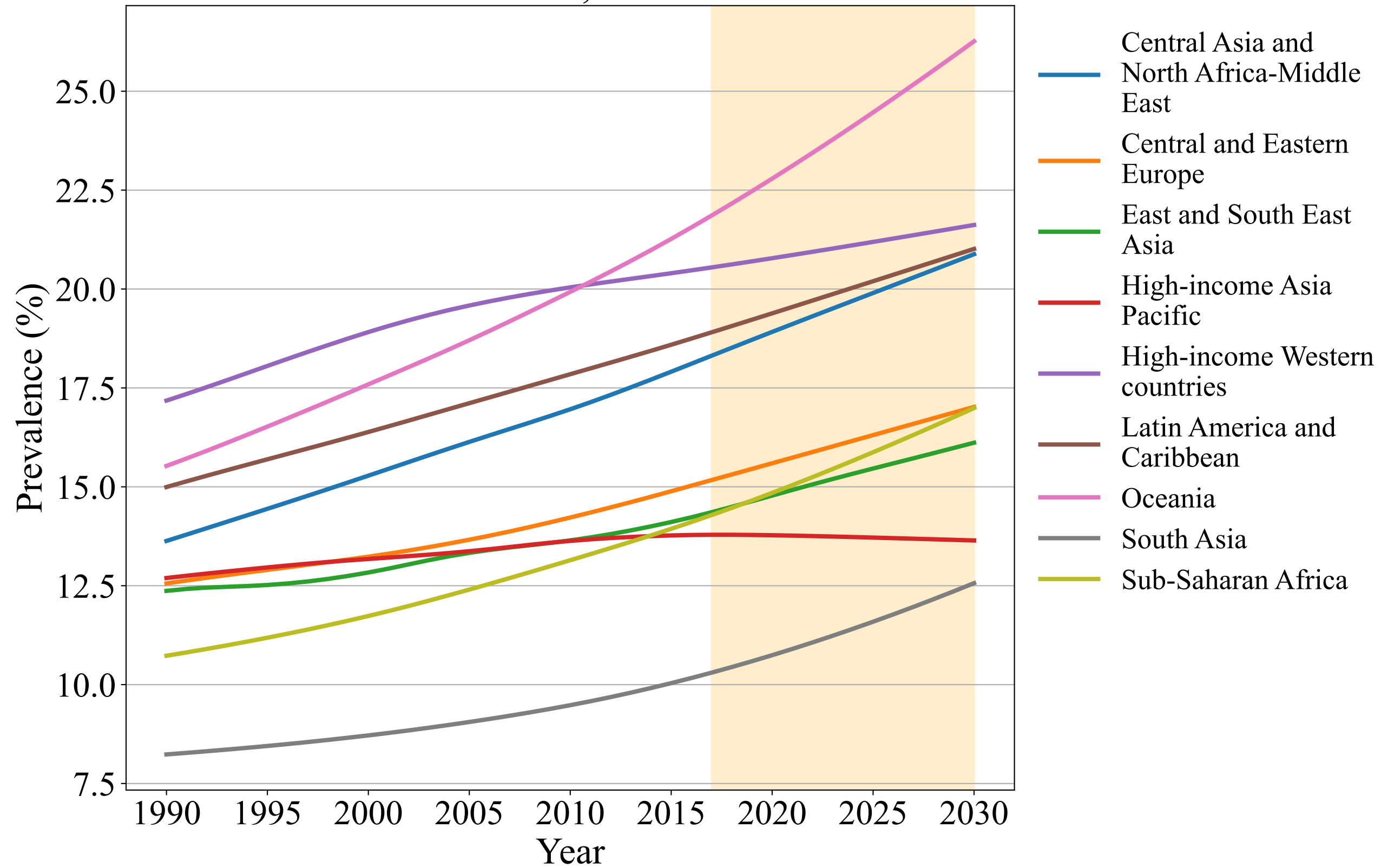

# Girls, 17

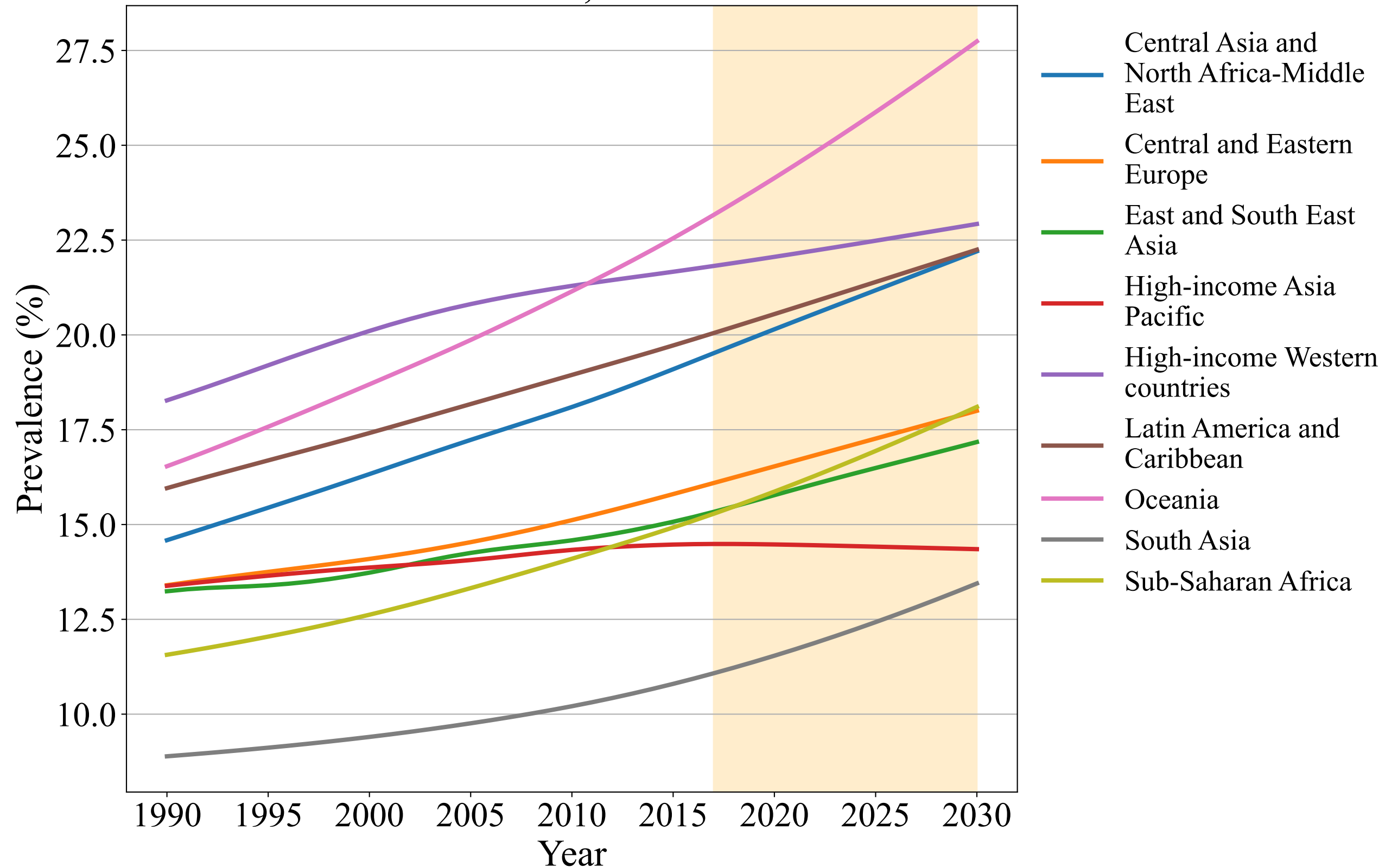

# Girls, 18

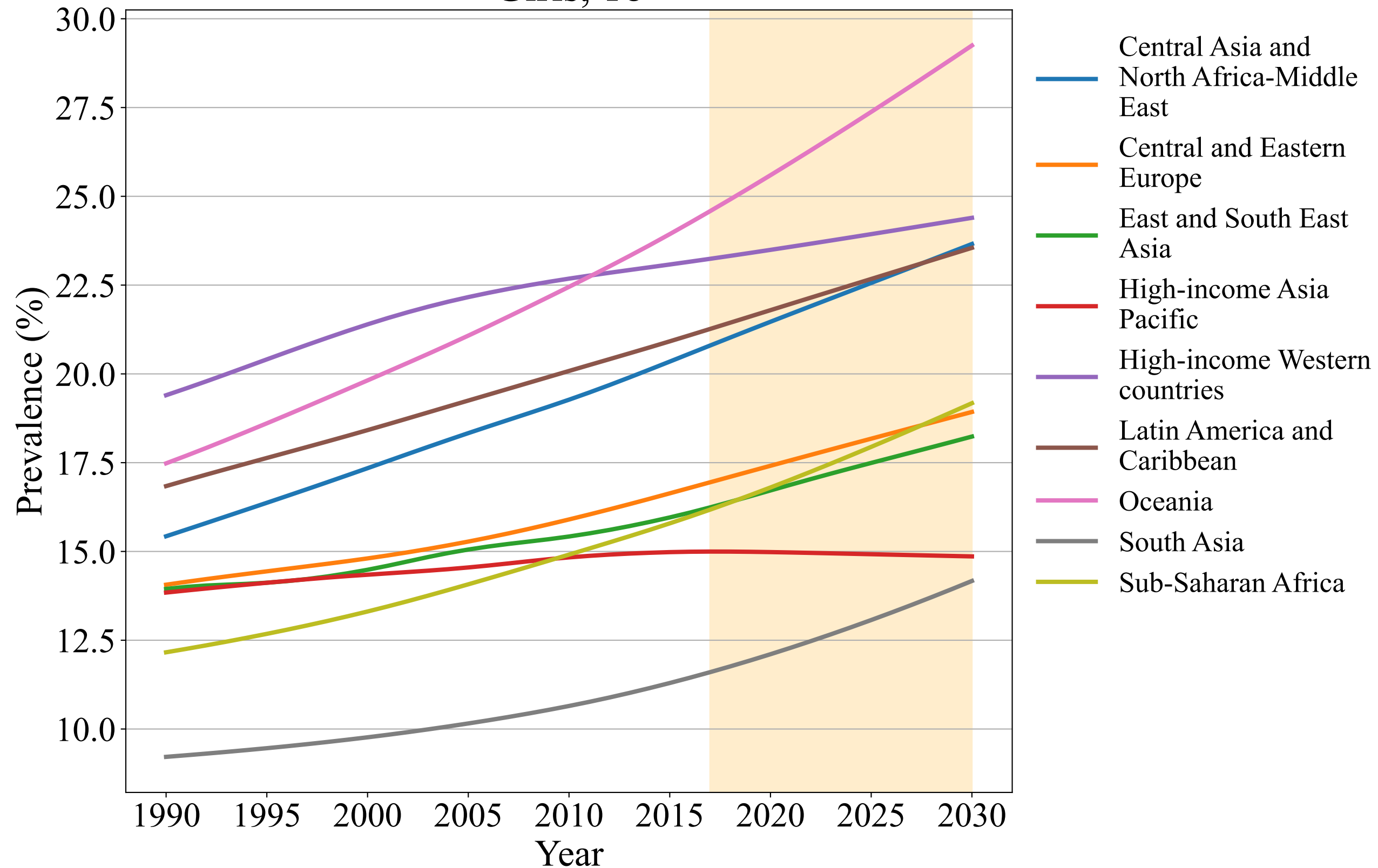

Supplement: Supplementary Figure 1 — Regional NAFLD prevalence, stratified by age. [file Image_1.pdf]

## Boys, 12

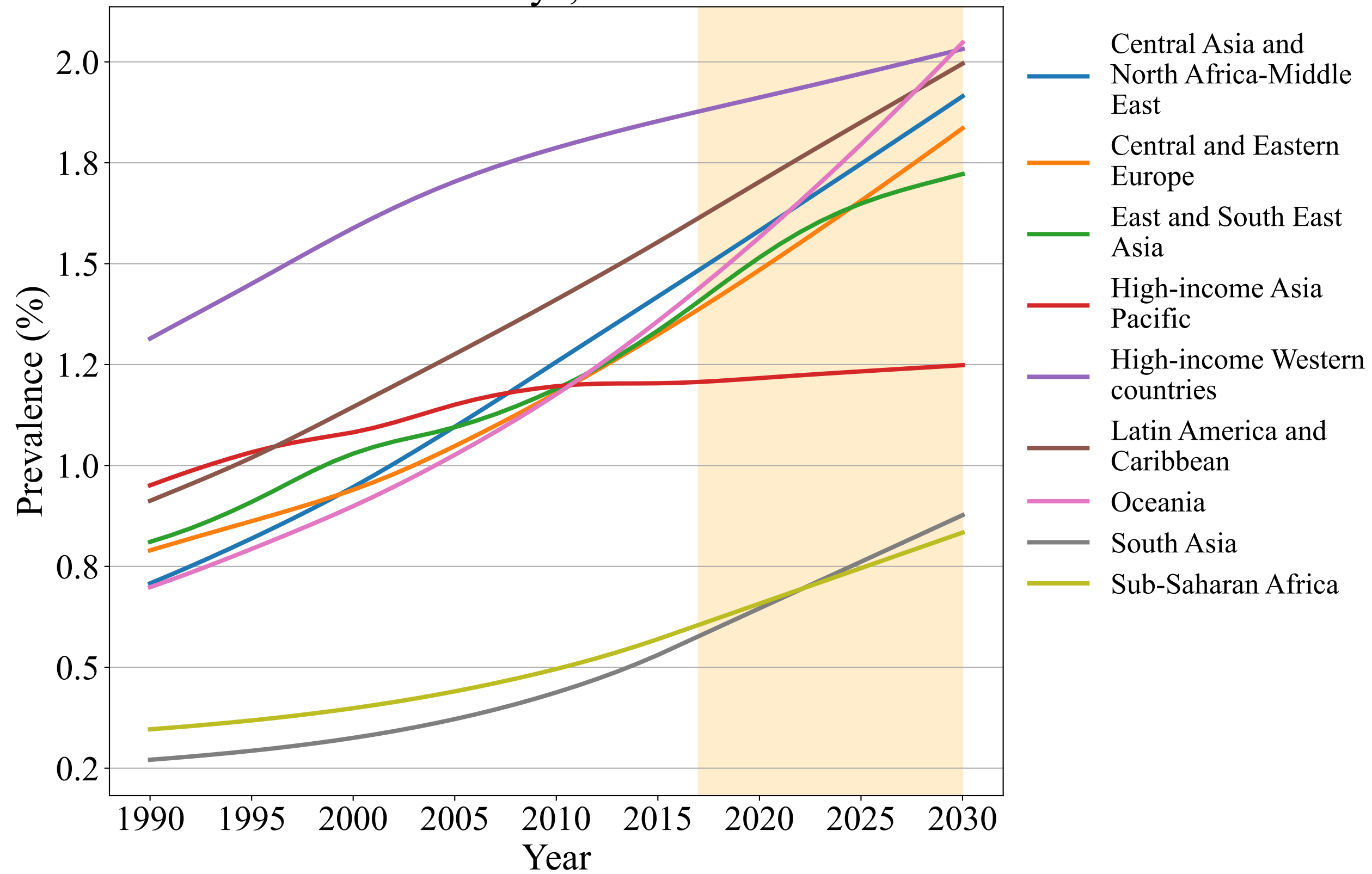

## Boys, 13

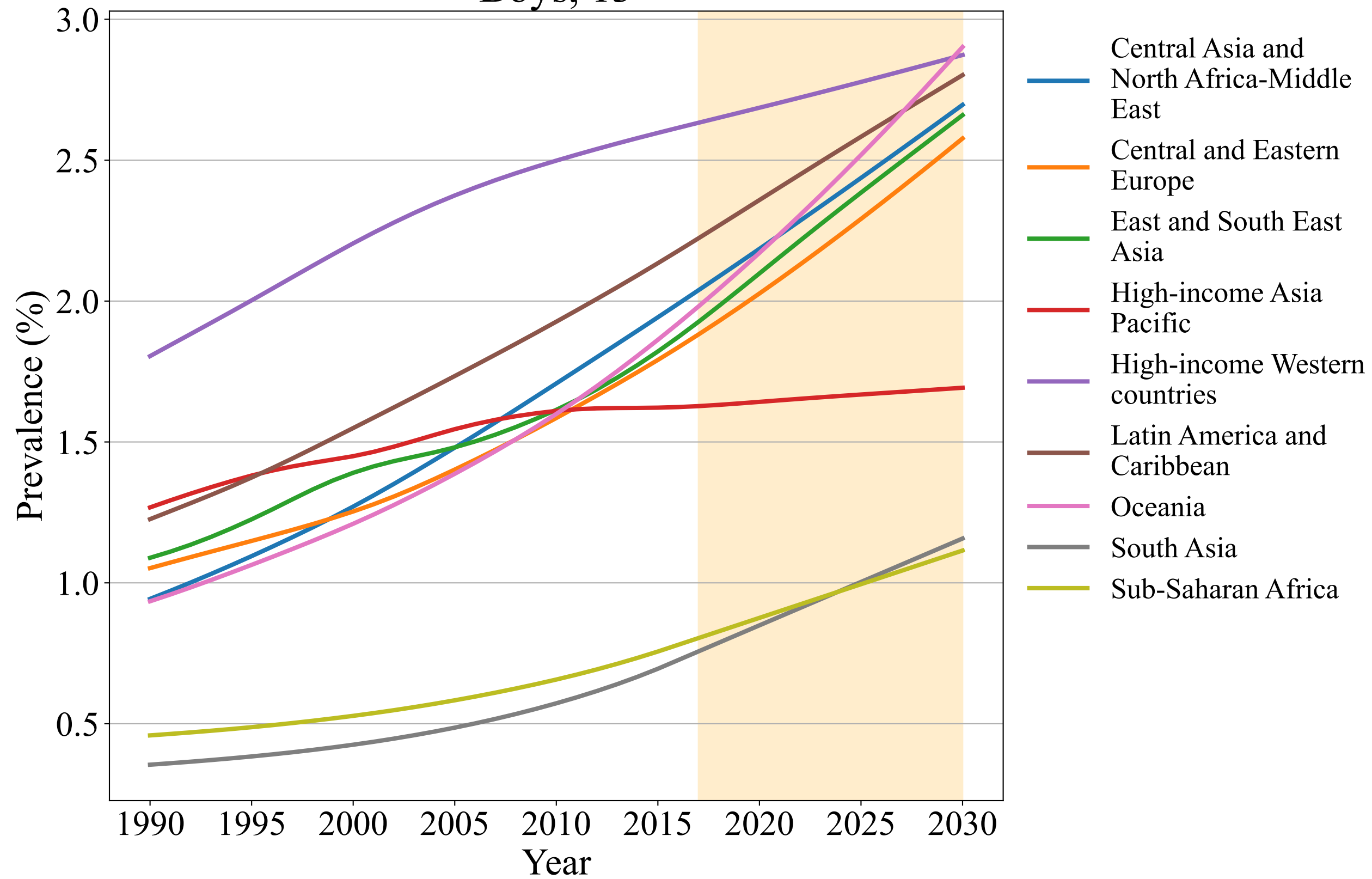

## Boys, 14

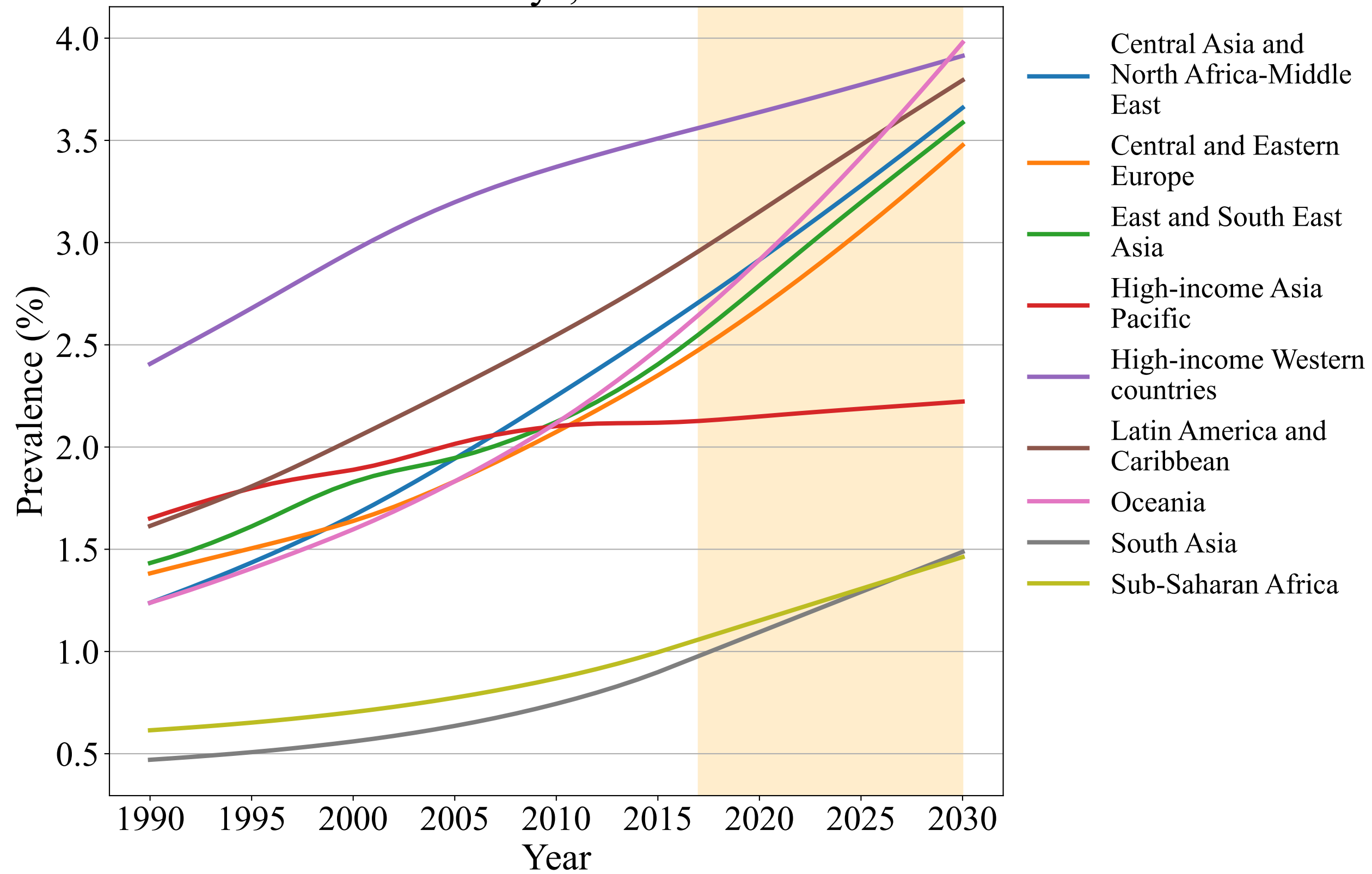

## Boys, 15

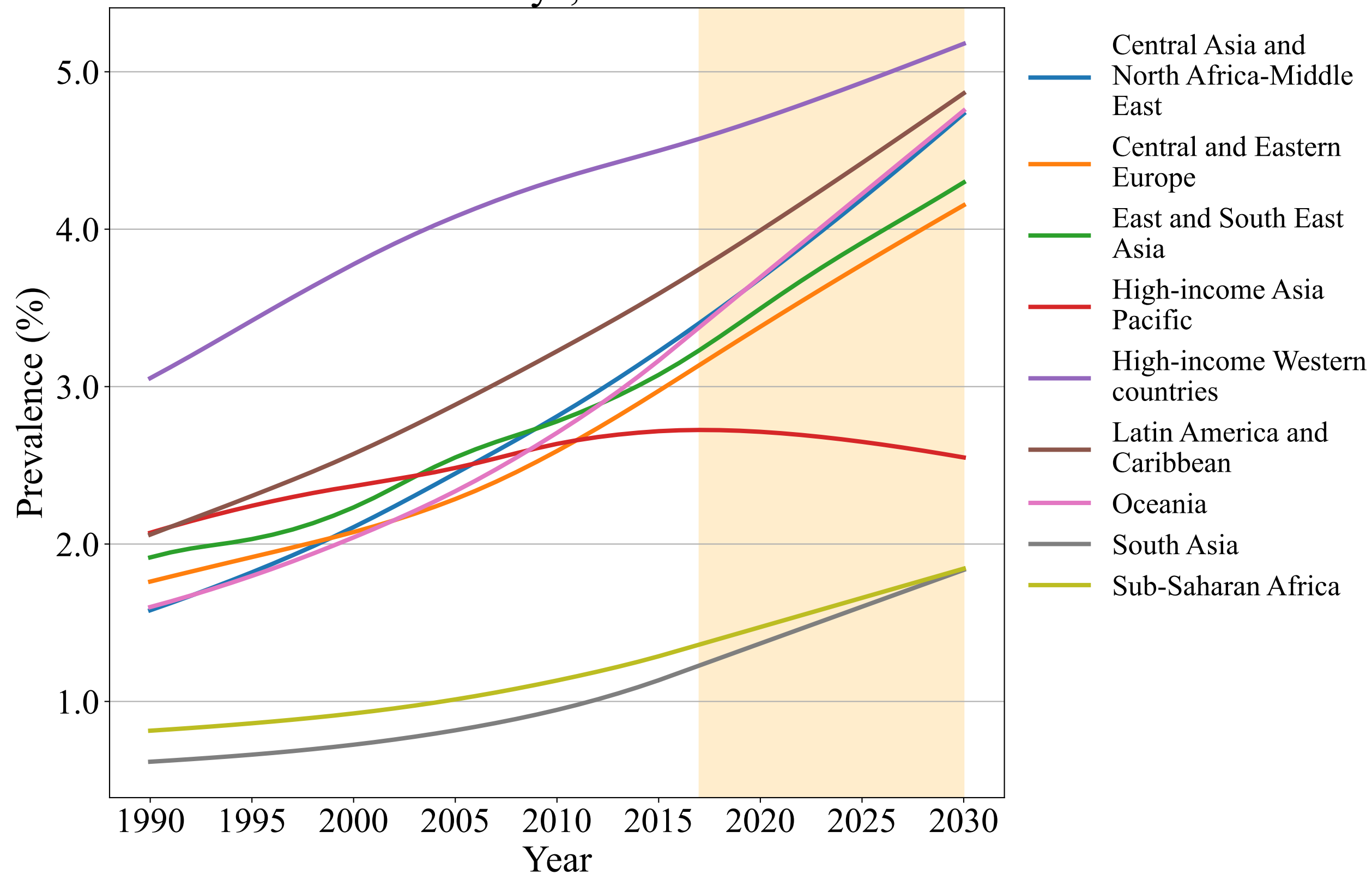

## Boys, 16

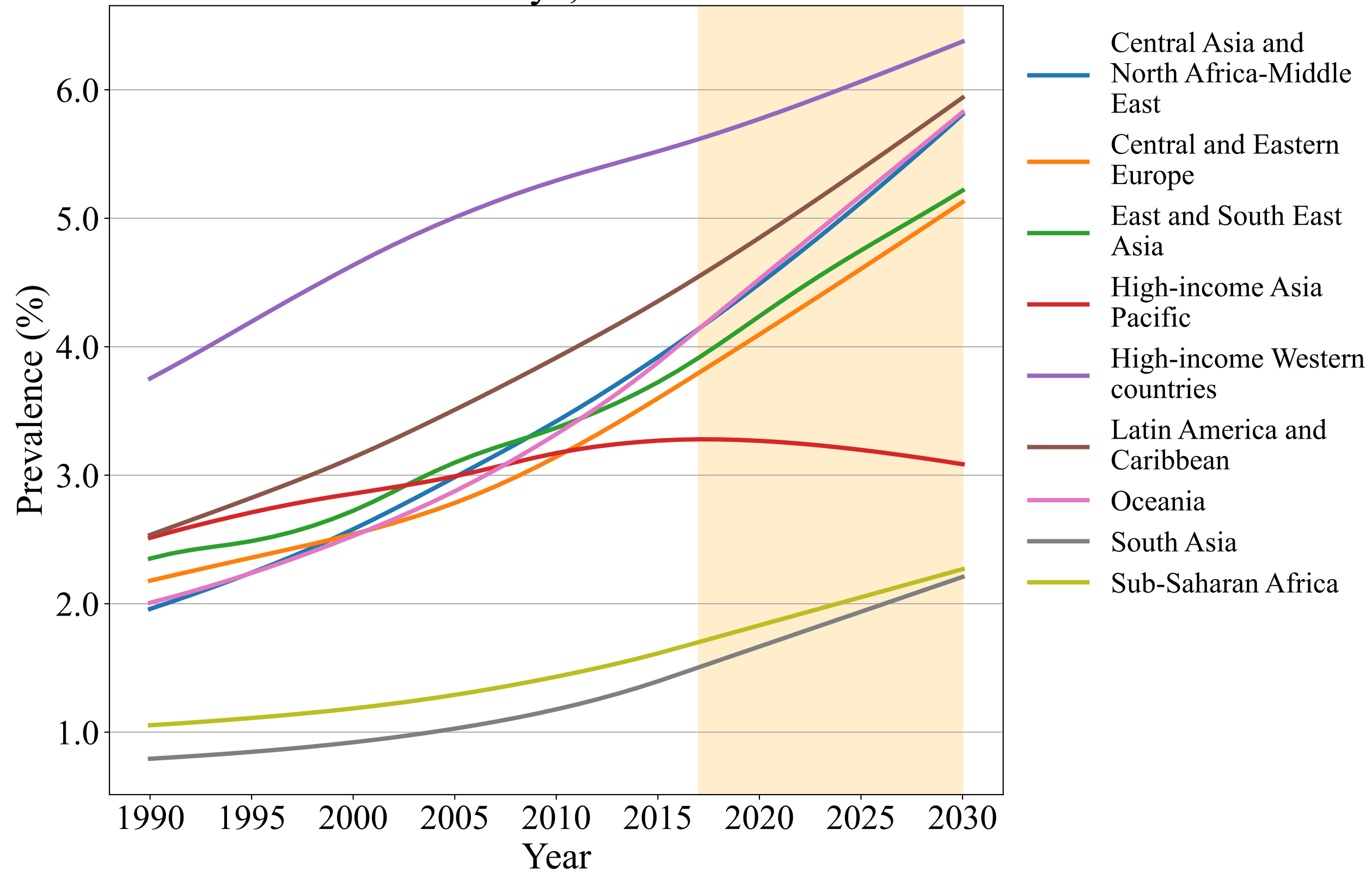

## Boys, 17

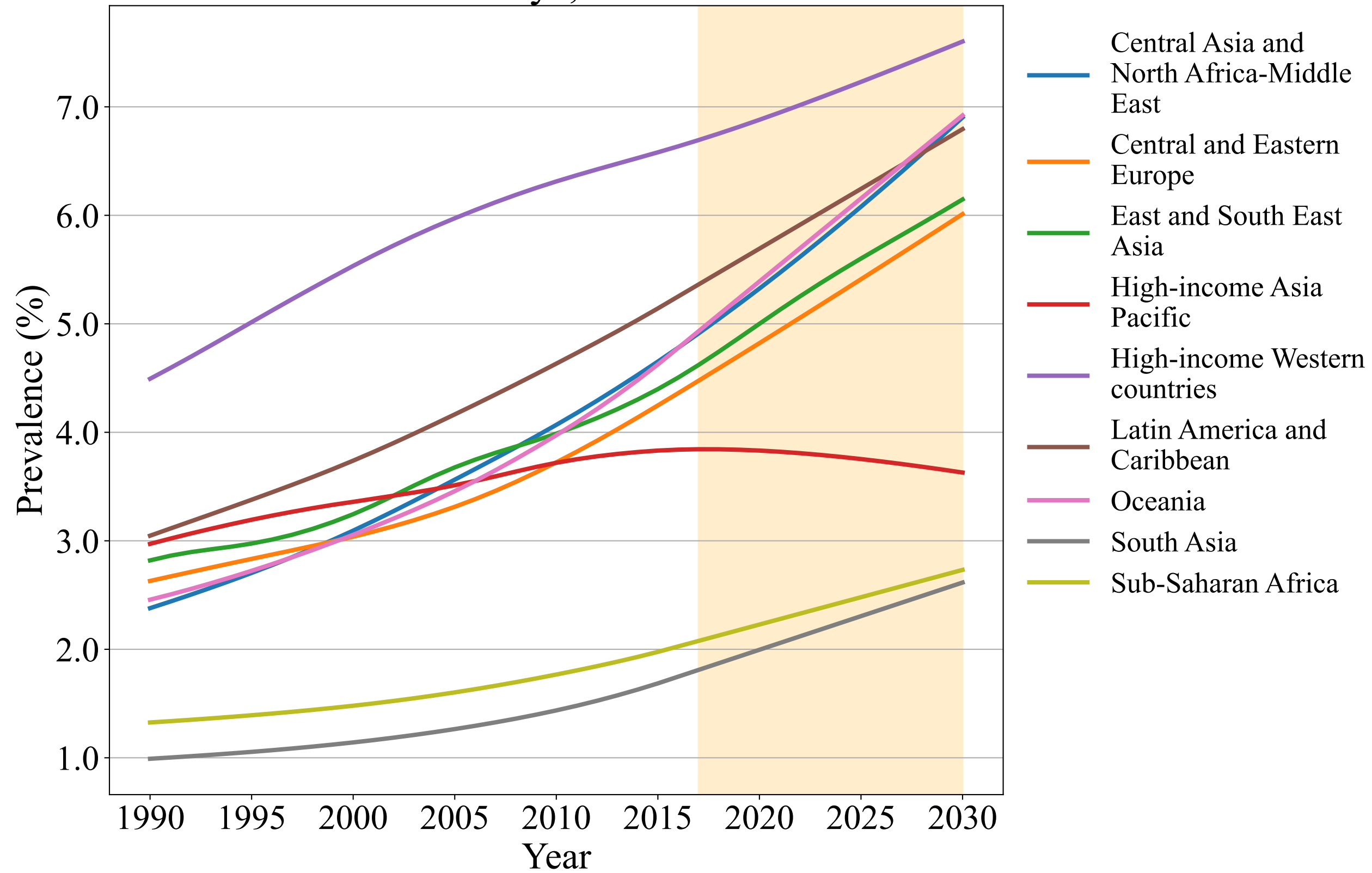

## Boys, 18

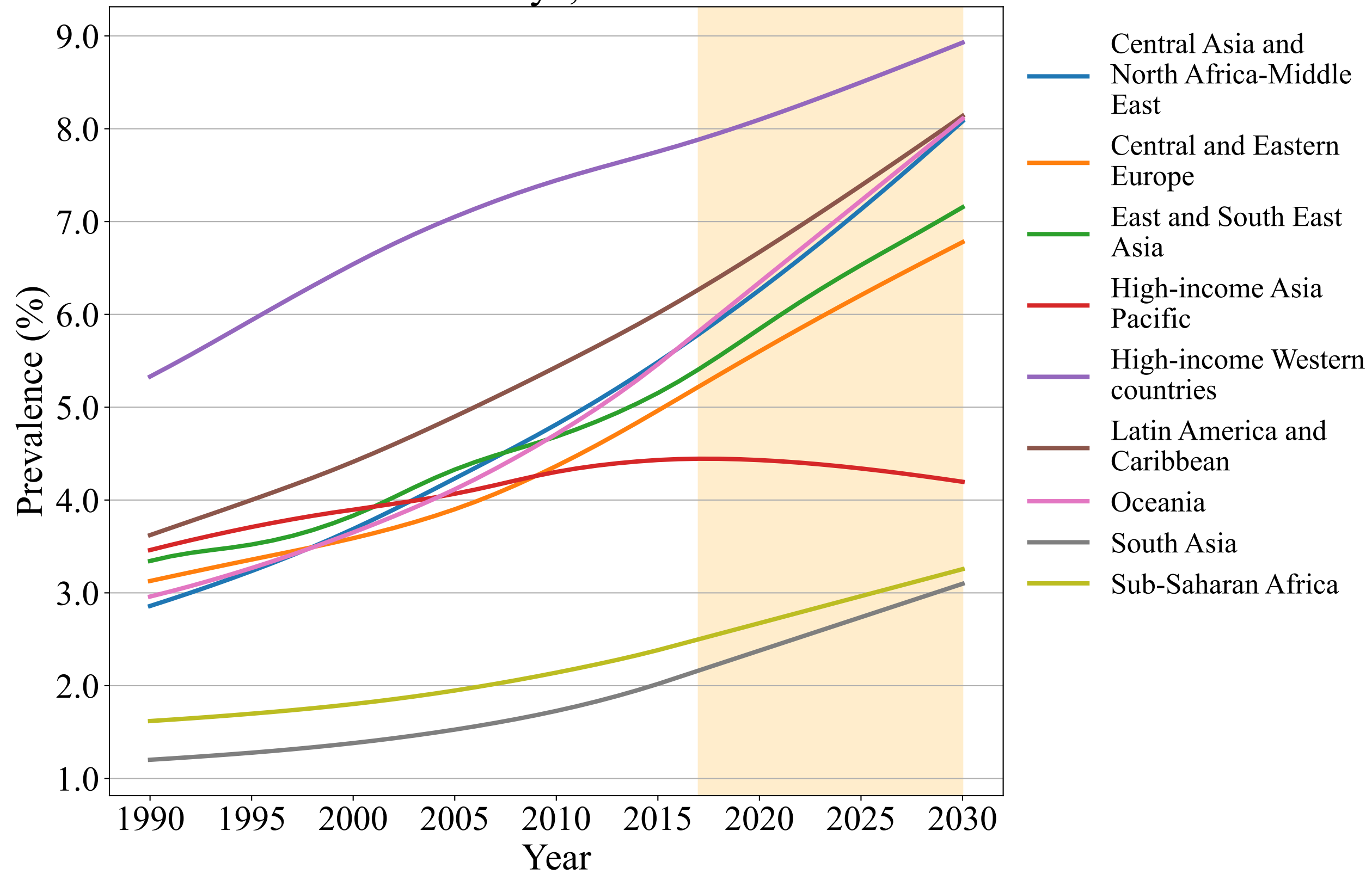

# Girls, 12

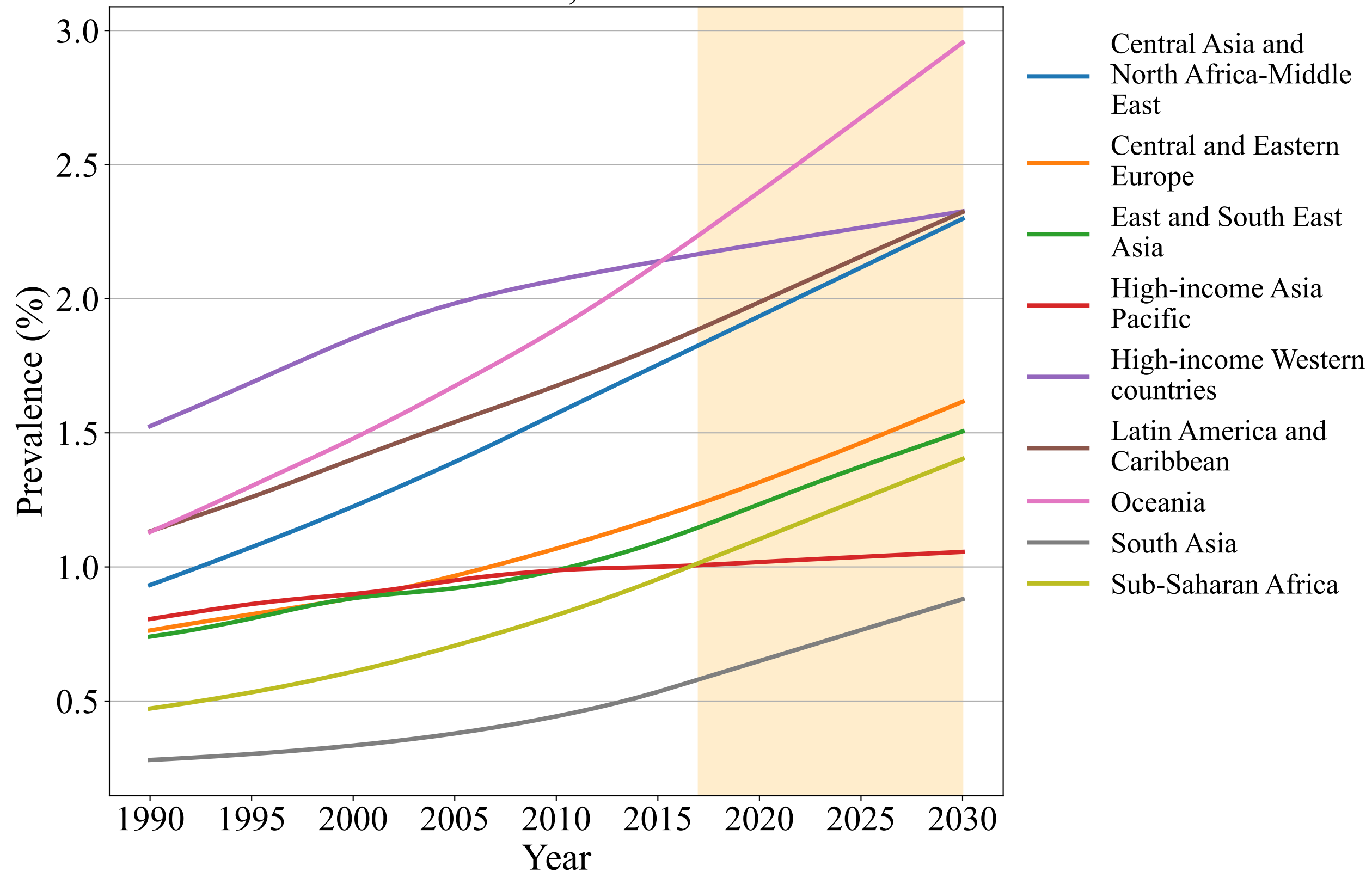

# Girls, 13

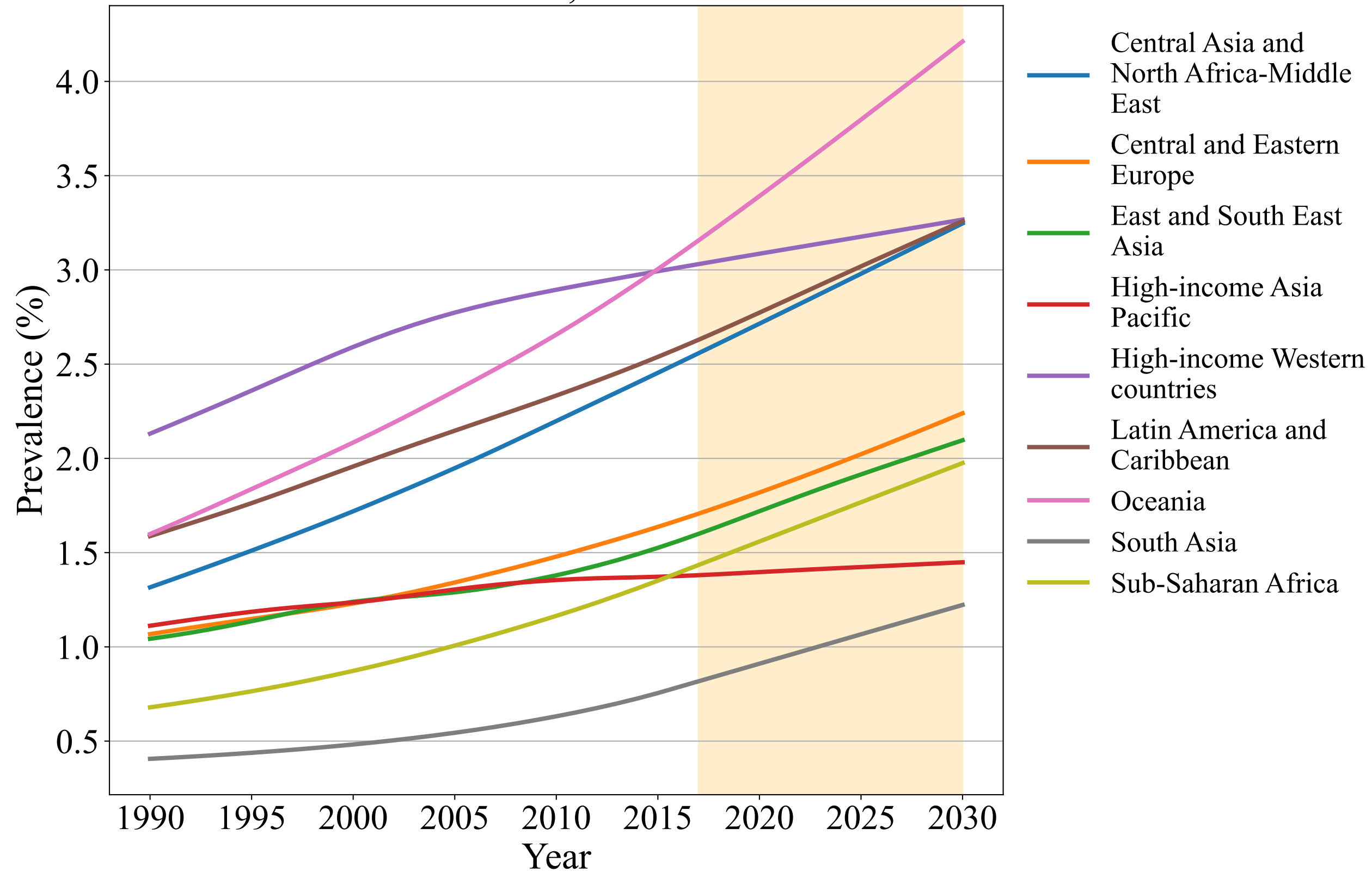

# Girls, 14

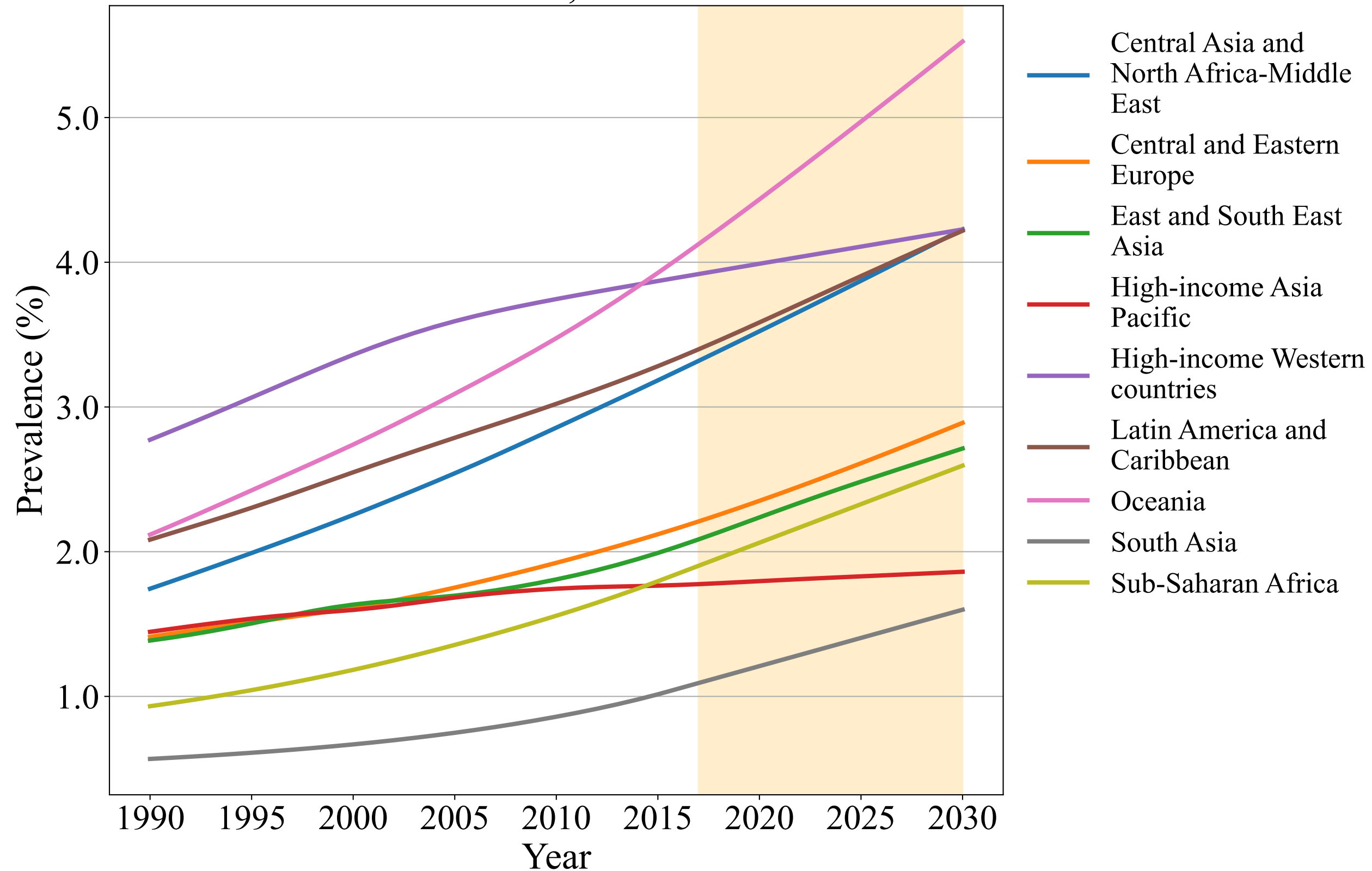

# Girls, 15

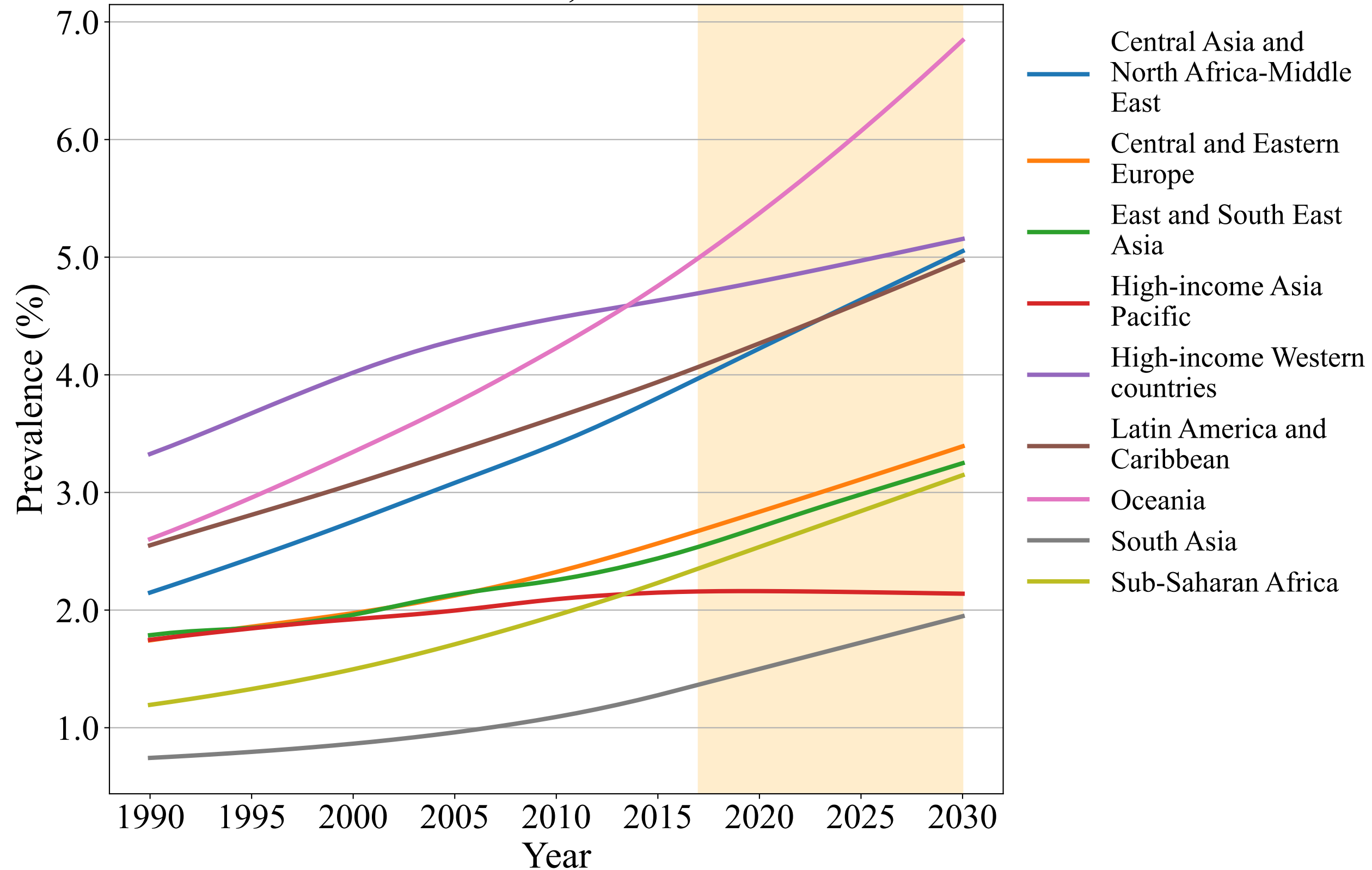

# Girls, 16

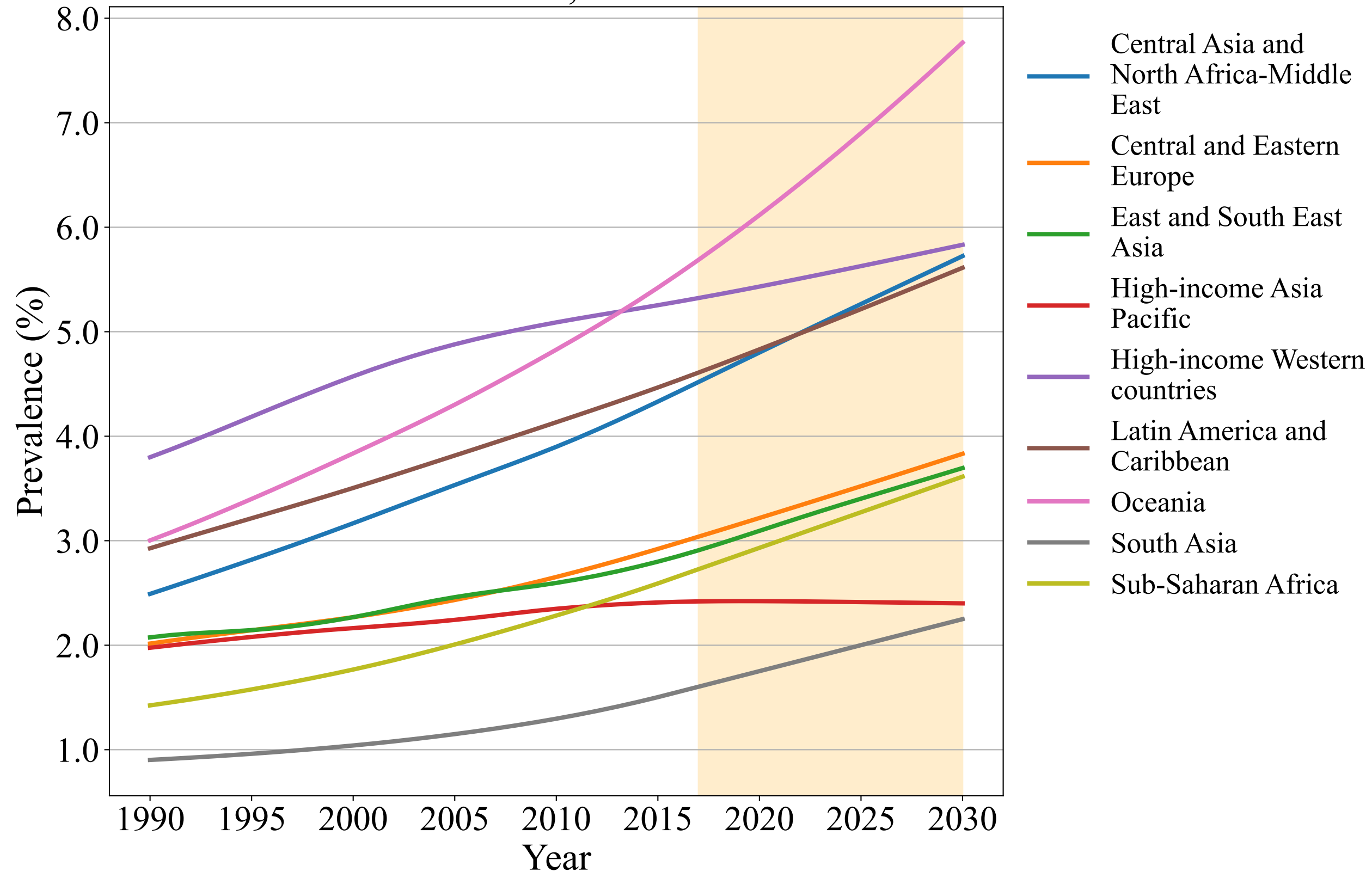

# Girls, 17

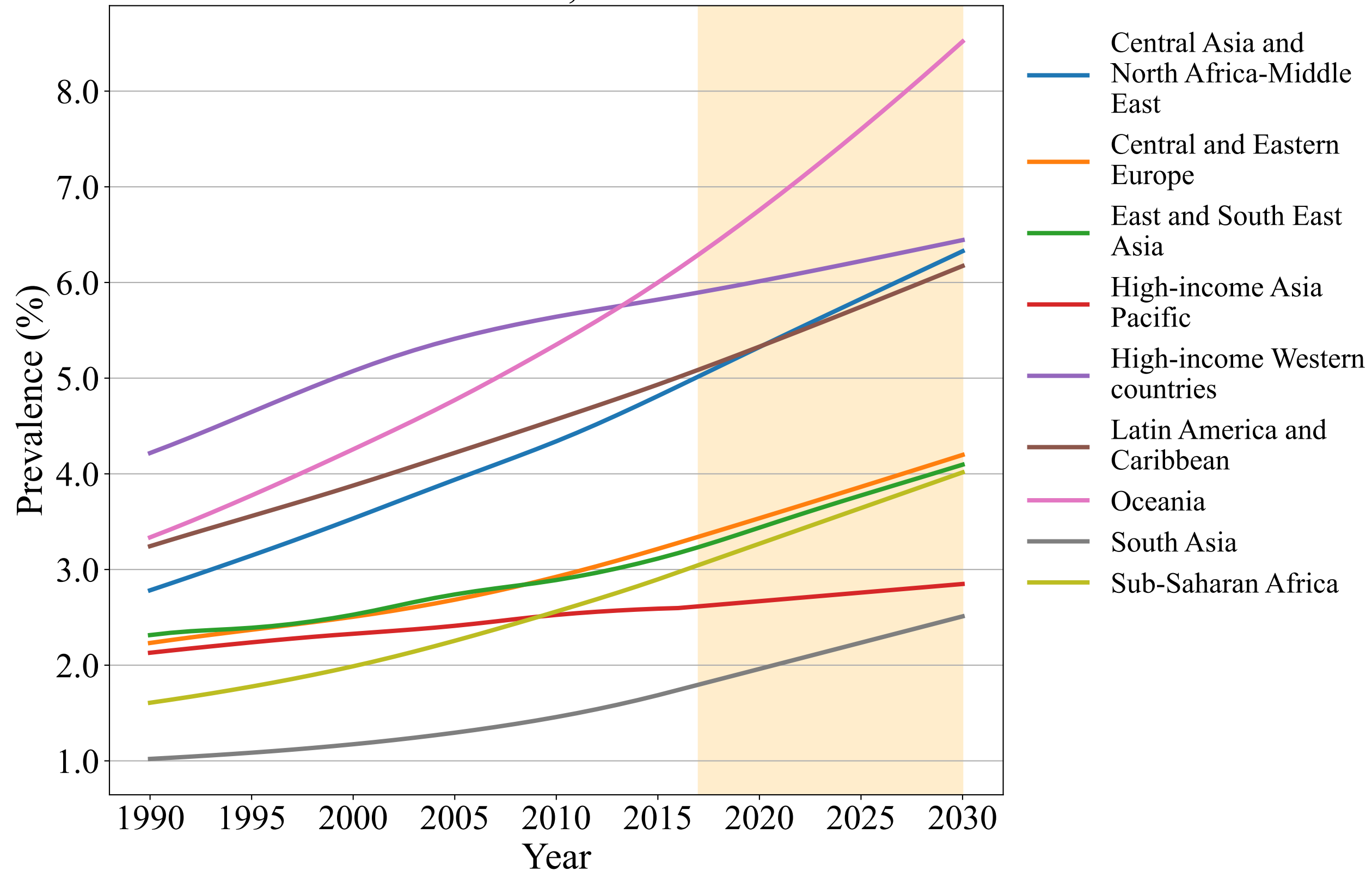

# Girls, 18

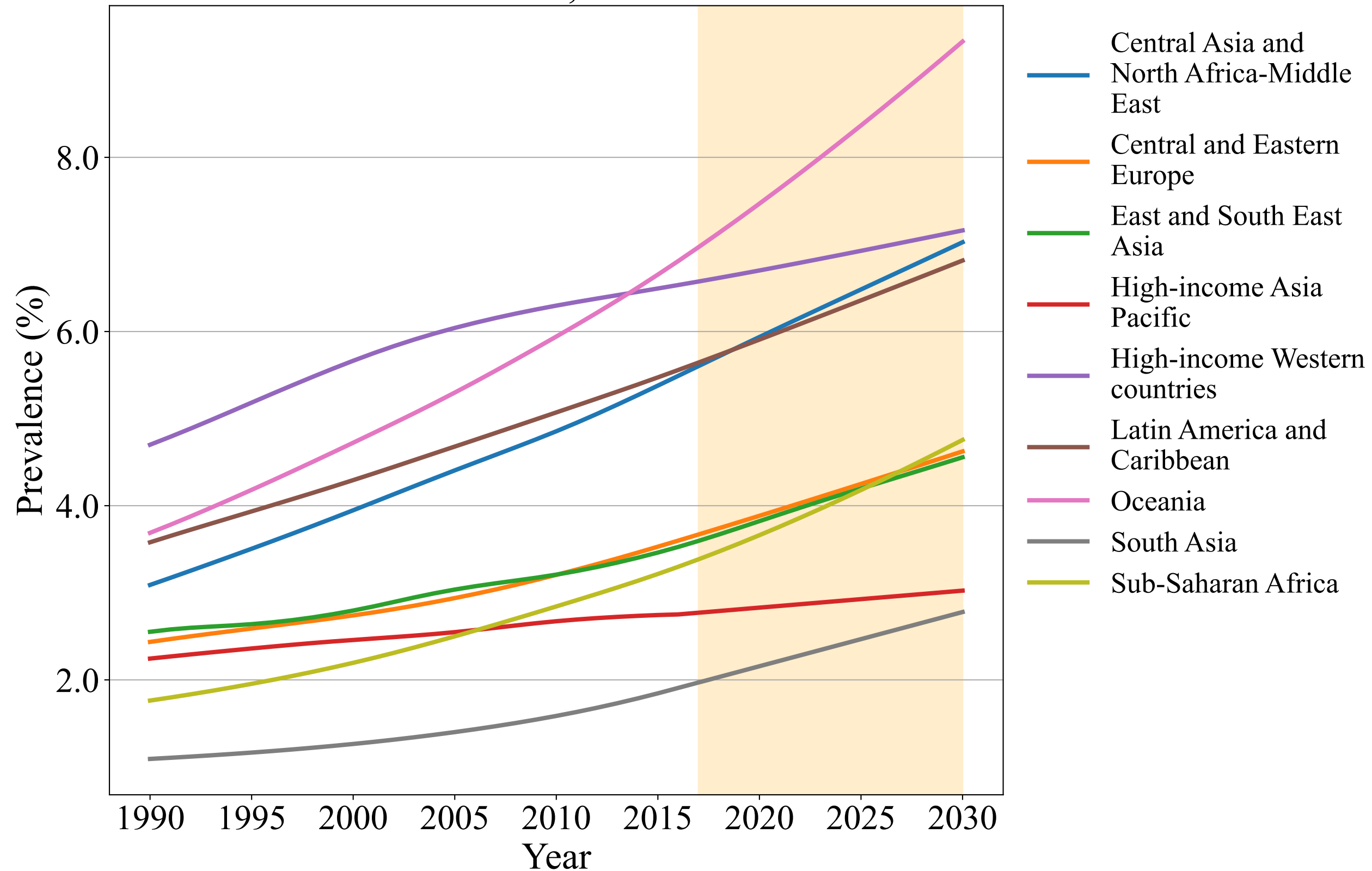

Supplement: Supplementary Figure 2 — Regional NASH prevalence, stratified by age. [file Image_2.pdf]
