# Supplementary material for: A significant and persistent rise in the global burden of adolescent NAFLD and NASH estimated by BMI
Source: Front Public Health. 2024 Oct 25;12:1437432. doi: 10.3389/fpubh.2024.1437432 (PMC11544631; doi:10.3389/fpubh.2024.1437432)
Supplement: Supplementary File 1 — Interactive web pages for average NAFLD/NASH prevalence at the country/region level. [file Data_Sheet_1.zip › Prevalence NASH country_Girls (all ages average).html]

Awesome-pyecharts
