# Supplementary material for: A significant and persistent rise in the global burden of adolescent NAFLD and NASH estimated by BMI
Source: Front Public Health. 2024 Oct 25;12:1437432. doi: 10.3389/fpubh.2024.1437432 (PMC11544631; doi:10.3389/fpubh.2024.1437432)
Supplement: Supplementary Presentation 1 — Appendix: Corresponding data of Figure 2, Figure 3, Figure 4, and Figure 5. [file Presentation_1.pdf]

Contents of appendix

Contents	Pages
Figure 2 Extracted data from NHANES 2017-March 2020 Pre-Pandemic Examination Data	1–28
Figure 3A, 3B Global prevalence of NAFLD in the boys and girls aged 12-18, 1975 to 2016	29–40
Figure 3C, 3D Global prevalence of NASH in the boys and girls aged 12-18, 1975 to 2016	41–52
Figure 3A, 3B Annual % change of global prevalence of NAFLD in the boys and girls aged 12-18, 1975 to 2016	53
Figure 3C, 3D Annual % change of global prevalence of NASH in the boys and girls aged 12-18, 1975 to 2016	54
Figure 3A, 3B Projected global prevalence of NAFLD in the boys and girls aged 12-18, 2017 to 2030	55–58
Figure 3C, 3D Projected global prevalence of NASH in the boys and girls aged 12-18, 2017 to 2030	59–62
Figure 4A, 4B Regional prevalence of NAFLD in the boys and girls aged 12-18, 1975 to 2016	41–52
Figure 4A, 4B Regional prevalence of NAFLD in the boys and girls aged 12-18, 1975 to 2016	63–181
Figure 4C, 4D Regional prevalence of NASH in the boys and girls aged 12-18, 1975 to 2016	182–300
Figure 4A, 4B Annual % change of regional prevalence of NAFLD in the boys and girls aged 12-18, 1975 to 2016	301–302
Figure 4C, 4D Annual % change of regional prevalence of NASH in the boys and girls aged 12-18, 1975 to 2016	303–304
Figure 4A, 4B Projected regional prevalence of NAFLD in the boys and	305–338

girls aged 12-18, 2017 to 2030	
Figure 4C, 4D Projected regional prevalence of NASH in the boys and girls aged 12-18, 2017 to 2030	339–371
Figure 5A, 5B Country/region-level prevalence of NAFLD in the boys and girls aged 12-18, 1975 to 2016	372–2956
Figure 5C, 5D Country/region-level prevalence of NASH in the boys and girls aged 12-18, 1975 to 2016	2957–5541
Figure 5A, 5B Annual % change of country/region-level prevalence of NAFLD in the boys and girls aged 12-18, 1975 to 2016	5542–5589
Figure 5C, 5D Annual % change of country/region-level prevalence of NASH in the boys and girls aged 12-18, 1975 to 2016	5590–5637
Figure 5A, 5B Projected country/region-level prevalence of NAFLD in the boys and girls aged 12-18, 2017 to 2030	5638–6391
Figure 5C, 5D Projected country/region-level prevalence of NASH in the boys and girls aged 12-18, 2017 to 2030	6392–7145

Serial Number	Sex	Age	BMI	E (kPa)	CAP (dB/m)	ALT (IU/L)	AST (IU/L)
110740	Boy	12	13.80	4.4	162	17	25
122332	Boy	12	15.00	4.2	201	12	20
122561	Boy	12	15.10	3.8	157	8	17
124447	Boy	12	15.90	6.1	174	9	22
119290	Boy	12	16.10	2.5	155	10	15
114881	Boy	12	16.10	5.1	215	12	27
123715	Boy	12	16.10	4.2	202	14	23
112417	Boy	12	16.10	4.4	137	22	42
120872	Boy	12	16.20	4.0	298	9	16
120134	Boy	12	16.30	3.5	170	11	21
116224	Boy	12	16.50	6.0	235	9	21
120439	Boy	12	16.50	4.6	205	10	19
113836	Boy	12	16.70	6.1	196	17	24
121161	Boy	12	16.80	3.2	205	10	24
115618	Boy	12	16.80	4.3	217	10	17
119154	Boy	12	16.80	4.9	213	11	28
122866	Boy	12	16.80	3.9	177	12	24
112651	Boy	12	16.80	3.8	115	15	22
111592	Boy	12	16.90	3.7	152	11	24
114164	Boy	12	17.00	7.5	196	11	21
112216	Boy	12	17.00	4.1	140	12	22
123550	Boy	12	17.00	8.6	225	15	20
117317	Boy	12	17.10	3.7	259	9	20
112175	Boy	12	17.10	3.6	225	20	25
121939	Boy	12	17.30	3.3	237	18	22
116681	Boy	12	17.30	5.2	191	24	28
111915	Boy	12	17.50	5.2	211	13	19
113160	Boy	12	17.50	3.7	175	19	25
110856	Boy	12	17.80	3.8	178	14	23
115199	Boy	12	17.90	5.2	221	18	28
113805	Boy	12	18.00	4.6	146	13	20
118613	Boy	12	18.00	4.0	245	14	18
113186	Boy	12	18.00	5.7	159	23	29
112735	Boy	12	18.10	5.0	167	11	24
122162	Boy	12	18.10	4.4	214	17	27
109706	Boy	12	18.20	3.1	100	11	21
110275	Boy	12	18.20	5.0	194	20	28
111529	Boy	12	18.30	3.7	189	14	NA
123887	Boy	12	18.50	4.2	133	4	17
122183	Boy	12	18.70	4.5	278	11	27
123308	Boy	12	18.80	4.8	196	11	19
121412	Boy	12	18.80	4.7	213	16	25
122961	Boy	12	18.80	3.5	128	22	27
114061	Boy	12	18.90	8.1	205	12	21
123158	Boy	12	19.40	3.2	227	13	18
115264	Boy	12	19.40	4.1	229	16	24
112656	Boy	12	19.70	4.0	256	16	26

112374	Boy	12	19.80	5.1	197	13	20
121886	Boy	12	19.80	6.9	188	40	33
120657	Boy	12	19.90	5.5	195	8	15
112197	Boy	12	19.90	6.0	128	11	20
117070	Boy	12	19.90	3.8	188	18	22
124084	Boy	12	20.00	4.3	256	26	30
110143	Boy	12	20.30	5.2	197	9	18
112026	Boy	12	20.30	4.2	248	11	21
117259	Boy	12	20.30	4.7	249	19	21
112144	Boy	12	20.40	4.0	176	13	22
115775	Boy	12	20.40	4.1	220	15	19
119793	Boy	12	21.10	4.8	177	11	22
123500	Boy	12	21.20	4.0	244	10	16
117791	Boy	12	21.20	4.9	229	15	23
112794	Boy	12	21.20	2.7	162	18	27
111636	Boy	12	21.30	4.0	245	17	27
121024	Boy	12	21.30	4.0	235	22	23
115556	Boy	12	21.40	4.6	226	18	21
112867	Boy	12	21.40	6.0	245	20	22
111241	Boy	12	22.10	6.8	173	11	20
109710	Boy	12	22.60	5.6	170	12	21
121574	Boy	12	23.00	5.2	114	13	19
118626	Boy	12	23.00	3.3	225	13	25
111346	Boy	12	23.20	5.1	227	21	28
117548	Boy	12	23.60	3.7	188	13	19
118059	Boy	12	23.80	5.5	223	10	15
115473	Boy	12	23.80	4.4	209	14	19
109446	Boy	12	24.50	3.4	205	19	18
123383	Boy	12	24.60	5.1	213	16	20
111836	Boy	12	24.90	4.6	294	49	34
124214	Boy	12	25.00	3.9	261	15	23
115502	Boy	12	25.40	4.9	197	15	19
112064	Boy	12	25.50	3.7	252	31	30
123618	Boy	12	25.60	5.1	209	35	31
122065	Boy	12	25.60	3.8	279	56	34
114832	Boy	12	26.00	4.9	227	9	15
110203	Boy	12	26.10	5.7	251	12	19
118502	Boy	12	26.40	4.9	242	11	18
117247	Boy	12	26.70	4.7	199	11	21
117844	Boy	12	26.70	3.1	214	24	25
121555	Boy	12	26.70	4.2	248	34	24
119851	Boy	12	27.70	5.3	279	40	32
120514	Boy	12	28.60	4.1	183	17	23
115118	Boy	12	28.70	6.4	262	12	20
123397	Boy	12	29.40	5.4	269	15	19
121437	Boy	12	29.90	8.5	260	16	30
123147	Boy	12	30.30	6.8	228	16	17
111057	Boy	12	30.60	4.6	263	19	20
120547	Boy	12	30.90	4.5	252	17	23

120758	Boy	12	31.20	7.0	351	45	33
122773	Boy	12	31.80	4.8	299	56	41
122403	Boy	12	32.30	5.1	268	14	21
114839	Boy	12	33.10	9.3	309	18	17
115812	Boy	12	34.90	4.8	272	14	16
122316	Boy	12	39.50	4.9	279	28	25
123081	Boy	12	40.10	4.1	270	24	22
117859	Boy	13	15.10	5.2	221	12	19
116246	Boy	13	15.60	5.9	125	12	18
111087	Boy	13	15.70	4.6	196	12	20
119006	Boy	13	15.70	4.0	248	12	20
124511	Boy	13	16.10	4.6	197	8	17
115061	Boy	13	16.20	4.1	169	9	19
109911	Boy	13	16.40	4.2	221	7	17
123250	Boy	13	16.40	4.2	157	10	20
116245	Boy	13	16.50	4.1	211	8	19
122710	Boy	13	16.90	3.8	180	10	19
117042	Boy	13	17.30	6.1	226	11	17
117284	Boy	13	17.40	4.6	260	9	18
120155	Boy	13	17.40	4.5	190	12	22
119205	Boy	13	17.40	5.1	213	15	27
112261	Boy	13	17.50	10.3	175	9	16
120853	Boy	13	17.50	4.2	184	12	22
123659	Boy	13	17.70	3.7	169	9	18
122308	Boy	13	17.90	3.9	269	9	16
114581	Boy	13	18.10	3.8	180	12	23
110696	Boy	13	18.10	4.9	209	13	24
119214	Boy	13	18.20	3.7	222	10	16
110743	Boy	13	18.40	5.9	158	11	22
122194	Boy	13	18.50	7.0	190	9	14
116498	Boy	13	18.50	5.1	194	14	17
122398	Boy	13	18.60	5.0	236	10	19
110160	Boy	13	18.60	3.7	188	21	19
115462	Boy	13	18.70	6.3	228	17	27
121935	Boy	13	18.90	4.8	191	11	18
116838	Boy	13	18.90	3.4	204	11	17
120014	Boy	13	18.90	3.7	247	11	19
115343	Boy	13	19.00	4.5	182	10	22
122554	Boy	13	19.10	5.6	205	13	21
118011	Boy	13	19.10	6.6	199	14	20
112204	Boy	13	19.20	4.5	190	8	18
109381	Boy	13	19.20	4.9	201	11	20
110205	Boy	13	19.30	2.5	338	11	24
111048	Boy	13	19.40	2.2	171	14	20
112036	Boy	13	19.60	5.9	196	19	27
122277	Boy	13	19.70	5.6	225	20	30
112051	Boy	13	20.00	4.8	239	10	16
118998	Boy	13	20.10	4.1	188	15	25
109929	Boy	13	20.30	3.6	250	14	21

112423	Boy	13	20.60	5.8	189	13	23
109795	Boy	13	20.70	5.8	157	21	21
120595	Boy	13	20.90	4.6	182	12	17
120347	Boy	13	21.00	4.7	224	11	16
119278	Boy	13	21.00	5.6	168	16	25
110133	Boy	13	21.10	4.2	163	41	40
122242	Boy	13	21.20	3.7	192	13	13
109449	Boy	13	21.30	6.0	185	10	20
112290	Boy	13	21.50	3.6	181	13	24
110864	Boy	13	21.50	2.9	148	37	33
109880	Boy	13	21.60	4.9	172	11	18
119921	Boy	13	21.60	7.2	211	15	28
115121	Boy	13	21.70	4.5	281	11	20
124321	Boy	13	21.70	3.9	220	12	17
114295	Boy	13	21.80	5.9	196	17	16
113698	Boy	13	21.90	5.8	192	17	19
113489	Boy	13	22.10	4.6	176	11	22
120786	Boy	13	22.10	5.9	239	15	21
112638	Boy	13	22.50	3.3	209	17	20
118329	Boy	13	22.70	3.5	280	12	15
109456	Boy	13	22.70	4.5	244	17	30
109511	Boy	13	23.00	4.0	225	14	21
110863	Boy	13	23.40	2.7	237	14	23
115024	Boy	13	23.50	3.6	202	11	16
119534	Boy	13	23.50	5.3	183	15	20
121947	Boy	13	23.60	5.3	222	14	18
112932	Boy	13	23.80	3.8	253	25	29
112914	Boy	13	23.90	4.7	241	11	19
124725	Boy	13	24.30	4.9	220	14	18
110790	Boy	13	25.20	3.5	194	19	18
117876	Boy	13	26.10	4.9	266	13	20
123987	Boy	13	26.20	5.9	245	14	26
117757	Boy	13	26.30	4.9	246	14	17
113544	Boy	13	26.50	5.4	247	26	29
122739	Boy	13	26.60	5.8	290	18	20
123589	Boy	13	27.60	3.8	207	18	26
117456	Boy	13	27.80	5.1	306	20	20
113051	Boy	13	27.90	3.5	263	18	22
123131	Boy	13	28.10	3.5	207	28	23
119033	Boy	13	28.40	5.1	255	16	22
111709	Boy	13	29.00	6.3	233	32	31
122174	Boy	13	30.70	5.1	257	58	59
118837	Boy	13	31.20	6.1	284	30	22
117085	Boy	13	31.20	5.1	272	31	25
119379	Boy	13	31.60	4.2	277	19	20
118355	Boy	13	32.10	6.2	313	12	31
114137	Boy	13	32.20	7.6	291	18	16
118021	Boy	13	33.10	4.9	327	46	24
120955	Boy	13	33.30	8.8	242	15	25

116577	Boy	13	33.30	4.1	211	16	15
120740	Boy	13	34.00	4.4	314	22	20
110677	Boy	13	48.20	9.3	400	18	17
113359	Boy	14	15.20	5.0	130	13	16
118968	Boy	14	15.40	4.1	238	14	19
124291	Boy	14	16.20	6.1	143	14	18
118379	Boy	14	16.30	5.6	157	11	19
111880	Boy	14	16.60	5.4	191	9	16
118511	Boy	14	16.70	24.0	278	10	20
123585	Boy	14	16.90	4.5	174	12	16
118112	Boy	14	17.10	5.4	186	27	24
113634	Boy	14	17.30	5.3	184	8	NA
117501	Boy	14	17.30	5.1	229	13	18
124077	Boy	14	17.30	5.5	215	21	27
116214	Boy	14	17.50	3.8	180	16	25
116285	Boy	14	17.60	4.9	124	8	18
111172	Boy	14	17.60	4.6	157	12	17
112447	Boy	14	17.60	3.4	127	20	20
111855	Boy	14	17.70	5.1	162	15	21
111151	Boy	14	17.80	6.6	166	11	24
120871	Boy	14	17.80	3.9	144	14	24
117245	Boy	14	17.80	4.1	163	16	17
113710	Boy	14	18.00	4.4	140	16	21
110095	Boy	14	18.10	4.6	219	11	25
112809	Boy	14	18.40	4.5	271	13	18
119281	Boy	14	18.60	5.5	190	12	20
119714	Boy	14	18.60	5.8	214	17	17
124220	Boy	14	18.60	5.5	189	61	162
117956	Boy	14	18.70	4.0	192	11	18
112376	Boy	14	19.00	7.3	238	11	21
123520	Boy	14	19.10	5.1	187	10	15
113952	Boy	14	19.10	5.2	183	16	21
114574	Boy	14	19.30	3.9	168	11	14
110646	Boy	14	19.40	4.1	195	12	15
121035	Boy	14	19.40	4.6	230	12	22
120649	Boy	14	19.50	5.3	166	9	13
109848	Boy	14	19.50	7.5	210	21	25
118558	Boy	14	19.90	6.1	165	10	21
109582	Boy	14	19.90	4.2	215	14	26
115826	Boy	14	20.20	4.1	224	9	12
112129	Boy	14	20.20	4.7	179	10	20
114710	Boy	14	20.70	5.0	177	13	25
123661	Boy	14	20.80	5.2	195	8	17
112035	Boy	14	20.90	6.7	191	24	25
117673	Boy	14	21.00	4.1	232	13	26
116982	Boy	14	21.10	6.3	232	13	17
110148	Boy	14	21.50	5.2	206	15	20
116237	Boy	14	21.50	4.8	208	20	24
113269	Boy	14	21.60	4.8	202	9	18

121449	Boy	14	21.60	6.3	166	14	22
110404	Boy	14	21.70	6.7	245	16	22
114140	Boy	14	21.80	5.1	207	9	16
110298	Boy	14	21.80	7.2	176	17	23
124427	Boy	14	22.00	4.1	243	63	68
109471	Boy	14	22.10	5.1	217	18	22
117396	Boy	14	22.30	5.6	192	9	15
113335	Boy	14	22.60	4.2	174	18	28
122273	Boy	14	22.70	4.1	182	15	19
111844	Boy	14	22.70	4.6	254	25	25
124480	Boy	14	22.90	4.7	172	11	13
110930	Boy	14	23.00	4.0	229	17	16
123839	Boy	14	23.10	7.3	174	14	19
111347	Boy	14	23.20	4.8	281	13	20
114766	Boy	14	23.20	4.7	234	17	22
122413	Boy	14	23.30	5.6	178	15	15
113700	Boy	14	23.40	4.4	207	15	23
114155	Boy	14	23.60	4.8	197	18	27
124493	Boy	14	23.80	7.5	209	15	30
114302	Boy	14	23.90	5.1	221	10	18
122733	Boy	14	24.00	4.8	257	8	10
119850	Boy	14	24.00	5.0	212	10	13
120305	Boy	14	24.20	4.4	238	14	20
122437	Boy	14	24.20	5.9	265	24	23
110066	Boy	14	24.30	4.5	259	14	16
124768	Boy	14	24.40	7.4	258	17	19
124763	Boy	14	24.40	5.7	187	19	31
115540	Boy	14	24.90	3.8	222	26	20
114468	Boy	14	25.10	5.4	304	22	23
121742	Boy	14	25.20	6.3	240	15	19
124233	Boy	14	25.20	3.2	243	20	20
110284	Boy	14	25.30	6.3	317	152	86
117572	Boy	14	25.50	6.5	227	12	17
110195	Boy	14	25.50	6.7	319	23	20
116663	Boy	14	25.70	4.5	308	14	18
121790	Boy	14	25.70	4.5	231	29	26
116606	Boy	14	25.90	2.8	302	13	20
112641	Boy	14	25.90	4.4	318	15	20
116593	Boy	14	26.00	4.5	270	15	17
115832	Boy	14	26.00	3.5	177	22	24
120114	Boy	14	26.10	4.4	270	14	18
118891	Boy	14	26.30	4.4	220	18	18
123710	Boy	14	26.30	3.5	325	18	19
118754	Boy	14	26.30	6.8	261	22	19
115700	Boy	14	26.50	4.6	230	11	16
110632	Boy	14	26.80	6.2	316	10	17
109641	Boy	14	27.10	4.2	259	9	16
119952	Boy	14	27.50	6.6	343	59	31
112728	Boy	14	28.00	5.3	302	22	29

122313	Boy	14	28.10	4.6	320	79	47
122382	Boy	14	28.20	5.1	203	11	14
123555	Boy	14	28.20	6.6	283	24	22
114282	Boy	14	28.30	3.9	209	10	15
117489	Boy	14	28.80	5.3	263	18	23
118043	Boy	14	29.00	4.8	320	22	18
110943	Boy	14	29.10	4.9	280	27	25
114717	Boy	14	29.10	5.4	278	49	33
117385	Boy	14	29.50	3.4	228	27	21
118744	Boy	14	30.00	4.6	205	17	20
121760	Boy	14	30.80	4.3	208	30	26
117955	Boy	14	30.90	5.0	295	16	17
117874	Boy	14	31.50	8.7	294	28	26
118208	Boy	14	32.60	6.6	263	34	18
119089	Boy	14	33.10	4.6	288	16	28
110281	Boy	14	33.10	8.4	320	111	55
123910	Boy	14	33.20	4.7	345	30	24
122464	Boy	14	33.30	7.7	330	14	14
119120	Boy	14	34.50	5.0	279	33	24
121196	Boy	14	35.00	4.3	273	19	24
114378	Boy	14	37.00	4.7	260	19	19
121127	Boy	14	38.60	4.4	286	19	22
110623	Boy	14	39.30	6.1	236	18	24
120935	Boy	14	39.70	7.8	278	20	21
118383	Boy	14	43.40	4.2	301	23	24
122114	Boy	15	16.00	4.1	127	14	24
110480	Boy	15	16.10	4.0	196	11	17
114875	Boy	15	17.40	3.8	218	10	23
112762	Boy	15	17.40	4.8	192	11	16
122799	Boy	15	17.70	6.5	229	8	14
114561	Boy	15	18.00	11.9	167	20	31
123917	Boy	15	18.10	4.6	194	12	17
114704	Boy	15	18.30	5.7	220	8	15
111581	Boy	15	18.40	5.1	155	9	14
120996	Boy	15	18.50	4.0	165	12	17
113571	Boy	15	18.50	6.3	179	12	16
115158	Boy	15	18.70	3.7	266	12	14
110481	Boy	15	18.80	4.6	185	10	14
116603	Boy	15	18.90	3.8	205	12	20
112655	Boy	15	18.90	4.0	210	13	30
121307	Boy	15	19.00	3.3	237	11	15
113238	Boy	15	19.00	14.6	219	14	22
118053	Boy	15	19.20	3.8	233	7	19
122980	Boy	15	19.20	5.6	201	11	19
122981	Boy	15	19.20	4.5	161	13	22
123616	Boy	15	19.20	8.9	237	15	21
123633	Boy	15	19.30	4.7	188	11	17
119818	Boy	15	19.50	3.2	196	14	20
121014	Boy	15	19.60	4.5	189	9	18

123725	Boy	15	19.60	16.2	170	11	18
113291	Boy	15	19.80	7.0	155	9	16
111861	Boy	15	19.80	6.0	198	12	18
115384	Boy	15	19.90	5.7	233	26	26
115477	Boy	15	20.10	4.5	190	14	19
123110	Boy	15	20.20	5.4	241	11	17
119757	Boy	15	20.30	5.4	182	8	16
118892	Boy	15	20.30	5.3	142	13	20
117597	Boy	15	20.30	5.2	205	13	19
115896	Boy	15	20.40	6.3	223	13	17
119008	Boy	15	20.40	5.3	198	14	20
123027	Boy	15	20.50	6.5	207	11	17
118102	Boy	15	20.80	4.6	205	10	20
112082	Boy	15	20.80	4.3	217	11	19
116419	Boy	15	20.80	4.5	200	15	13
109434	Boy	15	20.90	8.9	241	10	17
118243	Boy	15	20.90	11.9	141	15	22
119691	Boy	15	21.00	11.1	191	16	29
124692	Boy	15	21.10	5.2	237	11	39
116261	Boy	15	21.30	5.9	163	12	17
117614	Boy	15	21.40	4.5	214	10	17
118934	Boy	15	21.40	4.1	230	12	22
118497	Boy	15	21.40	7.6	268	21	30
110162	Boy	15	21.60	4.1	175	34	26
115486	Boy	15	21.90	5.0	171	17	17
116803	Boy	15	22.00	6.2	204	9	16
111607	Boy	15	22.00	4.0	240	11	16
121054	Boy	15	22.10	6.0	225	11	24
118982	Boy	15	22.10	4.5	232	12	21
110667	Boy	15	22.20	5.2	177	9	26
119409	Boy	15	22.30	5.4	188	19	27
120605	Boy	15	22.50	6.5	155	11	16
115163	Boy	15	22.80	5.9	229	13	25
120085	Boy	15	23.00	6.3	195	16	21
122190	Boy	15	23.70	5.5	192	13	19
114830	Boy	15	23.80	3.8	241	20	16
114471	Boy	15	24.30	4.5	216	11	17
124674	Boy	15	24.30	4.3	201	26	73
110685	Boy	15	24.60	3.9	275	10	16
116258	Boy	15	24.70	3.6	236	8	14
121959	Boy	15	24.80	3.9	190	13	17
113950	Boy	15	25.30	4.6	186	10	14
120695	Boy	15	25.60	4.0	273	9	13
110671	Boy	15	25.70	3.9	327	27	18
111103	Boy	15	25.80	6.6	339	27	25
116876	Boy	15	26.10	4.5	229	15	16
116212	Boy	15	26.10	5.4	248	23	23
118005	Boy	15	26.60	4.7	245	37	33
122907	Boy	15	26.80	5.6	258	20	21

119138	Boy	15	26.90	4.0	199	11	18
117100	Boy	15	27.20	6.4	256	14	16
117231	Boy	15	27.30	3.8	242	19	21
118507	Boy	15	27.70	4.9	235	23	39
120660	Boy	15	27.80	4.7	313	7	16
119436	Boy	15	27.80	4.9	290	30	28
117344	Boy	15	28.20	4.8	251	28	24
112245	Boy	15	28.60	4.4	222	12	16
122860	Boy	15	28.80	11.4	283	15	14
109380	Boy	15	29.10	6.3	253	16	18
117694	Boy	15	29.80	5.2	295	12	19
118694	Boy	15	30.10	3.1	272	15	17
117104	Boy	15	30.40	6.8	345	39	28
110598	Boy	15	30.70	4.8	333	54	41
122648	Boy	15	30.90	3.7	279	12	16
123906	Boy	15	31.30	6.0	213	21	19
114629	Boy	15	31.80	3.7	309	22	22
112763	Boy	15	32.40	6.4	342	39	27
110267	Boy	15	32.50	5.9	244	49	28
114270	Boy	15	33.00	5.0	284	20	18
123716	Boy	15	35.10	8.8	311	20	20
112873	Boy	15	36.10	6.0	259	46	26
111547	Boy	15	36.60	6.1	285	16	15
119717	Boy	15	36.80	4.9	358	93	52
114675	Boy	15	38.00	6.3	227	13	22
109847	Boy	15	43.10	4.8	322	28	22
120210	Boy	15	43.20	6.0	400	71	51
113782	Boy	16	13.20	5.4	100	30	29
123532	Boy	16	16.70	4.1	245	10	15
113961	Boy	16	17.00	5.8	115	14	23
120958	Boy	16	17.20	3.5	188	10	16
122311	Boy	16	18.30	4.8	147	10	16
110002	Boy	16	18.30	5.1	191	12	15
120035	Boy	16	18.50	5.7	217	11	13
123101	Boy	16	18.80	5.2	136	13	21
119946	Boy	16	19.00	6.2	237	14	23
111530	Boy	16	19.10	5.2	191	11	21
121273	Boy	16	19.20	6.1	130	19	21
120534	Boy	16	19.30	5.0	131	10	18
114630	Boy	16	19.60	3.6	192	10	14
114210	Boy	16	19.60	4.6	169	12	14
120007	Boy	16	19.70	2.7	237	28	19
121611	Boy	16	19.80	6.5	183	16	14
117515	Boy	16	19.90	5.5	185	11	20
119903	Boy	16	19.90	5.7	138	12	19
111838	Boy	16	20.10	6.8	194	12	22
118472	Boy	16	20.10	3.3	275	14	18
122775	Boy	16	20.20	4.6	107	9	18
116521	Boy	16	20.20	4.1	232	18	23

117196	Boy	16	20.40	4.8	226	9	17
119963	Boy	16	20.40	4.0	230	13	18
119647	Boy	16	20.50	6.4	162	11	27
122080	Boy	16	20.50	4.3	222	14	13
110542	Boy	16	20.50	7.0	166	19	30
124154	Boy	16	20.60	4.5	148	20	27
121524	Boy	16	20.70	5.6	177	20	23
112223	Boy	16	21.00	5.5	120	8	19
110009	Boy	16	21.00	6.1	173	8	13
124337	Boy	16	21.10	4.0	234	14	20
115275	Boy	16	21.30	11.5	225	16	22
117563	Boy	16	21.50	5.8	219	11	17
111167	Boy	16	21.50	7.2	164	19	34
124774	Boy	16	21.60	4.3	194	29	24
122884	Boy	16	21.60	37.6	246	47	60
113948	Boy	16	21.80	5.4	130	13	22
115165	Boy	16	22.00	4.7	182	13	20
120736	Boy	16	22.20	6.7	259	8	13
113070	Boy	16	22.30	5.9	196	14	16
116525	Boy	16	22.30	6.1	215	14	15
124003	Boy	16	22.30	5.5	244	18	34
112649	Boy	16	22.50	3.7	177	15	21
115001	Boy	16	22.50	6.4	206	16	18
110330	Boy	16	22.70	3.4	255	12	16
122049	Boy	16	22.90	4.1	171	14	13
120255	Boy	16	23.00	6.0	249	17	17
109750	Boy	16	23.10	6.0	206	11	18
113996	Boy	16	23.20	2.9	137	15	15
114609	Boy	16	23.30	4.6	234	12	18
121491	Boy	16	23.40	6.3	171	12	17
116927	Boy	16	23.50	4.8	178	18	19
117005	Boy	16	23.60	3.5	182	21	25
113080	Boy	16	23.90	5.0	196	8	13
111184	Boy	16	24.00	5.8	225	11	13
114485	Boy	16	24.40	4.6	185	13	17
120976	Boy	16	24.50	4.0	286	15	17
122804	Boy	16	24.80	5.6	204	12	18
121433	Boy	16	24.80	5.3	214	14	24
121777	Boy	16	24.90	4.6	249	10	13
110297	Boy	16	25.10	5.6	224	11	18
120216	Boy	16	25.10	4.7	183	12	17
113185	Boy	16	25.10	5.5	253	25	24
113679	Boy	16	25.60	5.3	189	29	29
111912	Boy	16	25.90	4.7	241	22	24
110432	Boy	16	26.00	6.7	175	9	17
120399	Boy	16	26.00	6.0	188	22	28
119786	Boy	16	26.00	6.1	188	22	21
121474	Boy	16	26.10	4.4	273	23	18
117248	Boy	16	26.40	4.4	228	13	22

122825	Boy	16	26.90	4.5	224	19	27
116539	Boy	16	27.50	4.6	260	13	18
111654	Boy	16	27.70	4.4	193	15	16
110420	Boy	16	28.70	4.4	295	51	33
118645	Boy	16	28.90	5.0	322	12	18
111020	Boy	16	29.20	7.0	194	14	17
120031	Boy	16	29.40	6.5	261	32	27
122558	Boy	16	29.50	6.4	280	18	23
115543	Boy	16	29.60	4.6	182	18	21
110720	Boy	16	30.80	6.1	272	15	16
124438	Boy	16	30.90	7.3	248	8	11
115267	Boy	16	31.20	3.8	295	16	21
111665	Boy	16	31.40	3.8	325	40	28
117473	Boy	16	31.50	4.8	244	49	50
122226	Boy	16	32.10	6.9	324	26	24
118644	Boy	16	32.10	4.5	395	56	26
111141	Boy	16	34.30	5.3	199	24	21
121380	Boy	16	34.50	6.4	400	60	36
109910	Boy	16	35.10	6.0	298	6	18
115194	Boy	16	36.00	5.6	283	13	20
112625	Boy	16	39.50	6.0	263	21	18
121598	Boy	16	43.00	6.2	400	54	26
122257	Boy	17	16.90	8.5	224	9	15
114223	Boy	17	16.90	5.6	183	12	13
113939	Boy	17	17.00	4.4	196	12	15
117898	Boy	17	17.00	6.1	228	19	29
120503	Boy	17	17.50	6.1	164	8	15
115572	Boy	17	17.50	3.6	230	13	23
119411	Boy	17	17.90	4.1	197	11	20
114909	Boy	17	18.00	3.5	181	9	18
123653	Boy	17	18.30	4.7	167	10	19
121701	Boy	17	18.60	5.2	226	14	28
113226	Boy	17	18.70	11.8	151	21	30
120195	Boy	17	19.00	5.6	222	15	23
123481	Boy	17	19.20	5.0	162	13	19
115169	Boy	17	19.30	4.3	165	20	15
110756	Boy	17	19.30	5.5	223	41	32
114016	Boy	17	19.50	4.9	189	11	16
124141	Boy	17	19.90	5.3	169	7	23
109682	Boy	17	20.20	4.3	171	10	19
124105	Boy	17	20.30	3.1	194	9	17
122704	Boy	17	20.30	5.3	206	9	15
113411	Boy	17	20.40	5.1	210	19	18
119319	Boy	17	20.70	3.5	211	11	18
111627	Boy	17	20.70	3.8	247	11	14
113999	Boy	17	20.70	4.2	174	13	16
117378	Boy	17	20.70	4.4	155	15	16
109417	Boy	17	21.00	6.2	224	14	14
122772	Boy	17	21.10	6.3	192	12	17

113221	Boy	17	21.30	4.9	145	12	13
109632	Boy	17	21.30	6.8	182	26	24
109557	Boy	17	21.50	3.9	206	12	17
120940	Boy	17	21.50	7.2	205	16	22
116779	Boy	17	21.50	4.1	230	19	20
121807	Boy	17	21.50	6.8	194	20	20
117126	Boy	17	21.60	4.7	235	19	19
123093	Boy	17	21.70	5.4	234	14	18
111056	Boy	17	21.80	2.4	237	13	18
116887	Boy	17	22.00	4.6	207	38	26
124586	Boy	17	22.20	7.8	214	6	11
123008	Boy	17	22.30	4.3	158	9	16
110534	Boy	17	22.30	10.5	190	25	21
118526	Boy	17	22.40	4.7	212	38	19
119165	Boy	17	22.80	5.0	173	12	20
114856	Boy	17	22.90	4.0	184	8	16
117927	Boy	17	22.90	7.1	191	12	16
120066	Boy	17	23.00	7.5	217	24	28
113656	Boy	17	23.10	5.8	213	21	20
117721	Boy	17	23.20	5.8	161	15	24
113321	Boy	17	23.50	5.2	243	15	18
111314	Boy	17	23.70	2.9	111	19	31
116682	Boy	17	23.80	5.9	166	16	17
114373	Boy	17	23.80	4.5	305	30	19
120504	Boy	17	23.80	5.3	240	60	23
119392	Boy	17	23.90	8.4	199	5	10
116790	Boy	17	23.90	4.5	230	13	18
123700	Boy	17	24.00	4.6	190	8	17
118064	Boy	17	24.00	4.8	206	9	19
109528	Boy	17	24.00	4.3	161	12	11
118976	Boy	17	24.20	5.8	245	19	16
119844	Boy	17	24.20	5.1	148	53	24
109465	Boy	17	24.30	4.0	237	9	13
122479	Boy	17	24.40	5.7	172	17	24
117337	Boy	17	24.50	10.2	220	9	21
112498	Boy	17	24.50	5.2	163	11	16
118516	Boy	17	24.50	6.1	178	16	14
123132	Boy	17	24.60	5.5	224	14	16
114732	Boy	17	24.60	5.8	261	19	23
119546	Boy	17	24.60	4.0	169	21	30
120074	Boy	17	24.70	8.9	188	9	13
122910	Boy	17	24.90	3.1	194	15	14
111041	Boy	17	25.40	6.9	185	10	17
122484	Boy	17	25.40	7.5	219	23	25
112526	Boy	17	25.80	6.1	215	20	44
110693	Boy	17	26.00	6.0	239	18	19
123888	Boy	17	26.00	4.6	131	55	47
112916	Boy	17	26.20	5.0	273	8	12
122748	Boy	17	26.40	4.3	228	9	15

117765	Boy	17	26.60	5.7	199	10	15
113408	Boy	17	26.60	4.0	283	37	20
118067	Boy	17	27.30	8.6	275	34	18
114974	Boy	17	27.40	6.3	201	8	18
123599	Boy	17	27.40	6.7	242	14	17
122447	Boy	17	27.50	5.6	250	26	26
109479	Boy	17	28.70	6.3	320	50	29
110199	Boy	17	29.00	5.1	313	21	17
122626	Boy	17	29.30	8.5	300	29	25
119074	Boy	17	29.40	3.8	325	15	17
115124	Boy	17	29.60	4.1	216	47	19
117413	Boy	17	29.70	5.7	267	45	71
112058	Boy	17	29.70	5.3	330	70	23
121594	Boy	17	30.00	5.6	292	10	12
119176	Boy	17	31.20	5.2	360	18	21
117356	Boy	17	32.50	6.9	291	15	22
122613	Boy	17	32.70	3.4	330	19	26
116054	Boy	17	33.40	5.0	279	19	21
119203	Boy	17	34.90	6.5	254	37	27
115706	Boy	17	35.20	6.4	357	34	17
115200	Boy	17	35.40	13.0	336	39	45
121875	Boy	17	37.40	21.4	249	15	16
113006	Boy	17	40.20	17.1	400	66	33
118720	Boy	17	40.40	3.4	267	25	24
111269	Boy	17	41.30	5.4	100	25	22
115562	Boy	17	43.10	4.7	336	20	30
121832	Boy	17	44.80	7.4	303	23	19
110642	Boy	17	47.30	7.9	389	56	36
110225	Boy	17	47.90	6.8	280	36	21
109361	Boy	17	49.60	4.1	339	18	14
116935	Boy	18	17.70	3.6	154	16	20
109303	Boy	18	17.90	4.8	182	13	17
110367	Boy	18	18.20	4.0	169	52	192
115756	Boy	18	18.30	4.4	205	15	18
117347	Boy	18	18.60	5.4	200	9	14
122179	Boy	18	18.60	2.6	198	17	15
124463	Boy	18	18.80	4.0	215	15	23
116372	Boy	18	19.50	8.9	151	8	22
111302	Boy	18	19.50	3.4	214	10	12
113272	Boy	18	19.70	4.6	222	15	21
112631	Boy	18	19.70	3.7	218	46	35
112468	Boy	18	19.90	5.9	159	10	20
115731	Boy	18	19.90	4.9	170	10	14
124513	Boy	18	20.10	4.0	220	19	20
118865	Boy	18	20.20	5.2	166	13	16
122007	Boy	18	20.40	6.3	240	6	28
113058	Boy	18	20.60	5.6	151	12	17
124210	Boy	18	20.60	4.8	175	22	36
111249	Boy	18	21.00	8.0	165	9	14

109458	Boy	18	21.00	4.3	194	26	18
116026	Boy	18	21.40	7.8	284	10	20
110871	Boy	18	21.50	5.5	207	10	14
122916	Boy	18	21.60	4.2	260	21	20
123182	Boy	18	21.70	3.9	209	23	17
114074	Boy	18	21.90	5.6	197	11	23
122149	Boy	18	21.90	4.3	261	44	22
114657	Boy	18	22.00	7.0	192	20	20
113432	Boy	18	22.00	5.6	230	22	21
119308	Boy	18	22.10	3.8	323	27	51
112991	Boy	18	22.20	3.5	210	16	13
122378	Boy	18	22.60	4.1	237	13	18
115512	Boy	18	22.60	3.8	239	22	20
114718	Boy	18	22.70	6.1	206	22	32
119397	Boy	18	23.00	4.5	200	12	14
111403	Boy	18	23.10	4.7	160	11	18
122135	Boy	18	23.10	8.1	203	12	18
124313	Boy	18	23.20	3.6	219	8	16
114343	Boy	18	23.20	4.4	139	11	14
118465	Boy	18	23.20	3.2	242	11	12
119702	Boy	18	23.20	4.4	198	14	19
115776	Boy	18	23.30	5.7	252	19	18
114755	Boy	18	23.50	3.4	176	13	18
124606	Boy	18	23.50	3.6	241	14	14
121558	Boy	18	23.80	4.1	241	10	16
124020	Boy	18	23.80	4.7	172	64	52
121897	Boy	18	24.10	4.4	274	31	25
124654	Boy	18	24.20	3.6	163	14	15
120498	Boy	18	24.20	3.2	162	17	15
116971	Boy	18	24.30	4.4	155	18	23
111181	Boy	18	24.50	6.8	205	13	27
124314	Boy	18	24.60	4.1	233	11	16
122360	Boy	18	24.70	6.1	256	11	20
109980	Boy	18	24.70	2.5	256	25	19
111372	Boy	18	24.70	4.6	236	30	31
122481	Boy	18	25.10	3.2	180	22	34
113204	Boy	18	25.20	4.3	188	19	21
118797	Boy	18	25.30	8.9	169	9	13
117227	Boy	18	25.30	5.0	182	11	17
116534	Boy	18	25.30	4.8	250	19	19
121342	Boy	18	25.60	4.1	215	10	13
123174	Boy	18	25.60	4.6	272	19	19
120461	Boy	18	25.70	4.1	194	15	14
116201	Boy	18	26.10	4.2	222	9	18
120389	Boy	18	26.20	5.9	245	10	18
121235	Boy	18	26.30	3.1	238	11	13
115447	Boy	18	26.30	6.4	252	11	17
118605	Boy	18	26.50	5.6	195	12	15
109654	Boy	18	26.60	3.9	247	21	17

120250	Boy	18	26.90	4.3	255	14	16
124059	Boy	18	27.20	5.8	267	19	16
112031	Boy	18	27.50	3.3	227	20	31
124636	Boy	18	27.80	6.7	214	13	18
110478	Boy	18	27.80	6.0	215	18	19
123675	Boy	18	28.10	4.9	240	14	19
117868	Boy	18	28.30	4.8	275	25	17
114396	Boy	18	28.40	4.9	282	53	24
124081	Boy	18	30.00	4.8	100	23	21
124740	Boy	18	30.30	5.3	310	27	20
124136	Boy	18	30.30	4.7	147	36	22
110988	Boy	18	30.60	3.6	268	15	17
113052	Boy	18	31.30	6.5	252	26	31
121481	Boy	18	31.70	3.8	254	15	16
113894	Boy	18	31.90	2.6	111	24	20
113270	Boy	18	32.20	7.5	321	23	22
123964	Boy	18	32.40	4.6	272	27	18
119316	Boy	18	33.70	5.6	396	42	28
119598	Boy	18	34.30	6.9	334	33	20
111055	Boy	18	35.00	6.3	227	17	17
110719	Boy	18	35.60	5.1	281	36	31
115539	Boy	18	35.70	4.6	268	39	22
122818	Boy	18	36.20	7.9	363	52	27
124706	Boy	18	36.80	5.9	259	29	21
113216	Boy	18	39.00	7.7	357	47	20
114152	Boy	18	39.20	7.5	298	37	21
115926	Boy	18	40.10	4.8	307	33	24
110541	Boy	18	40.70	9.7	371	21	16
119997	Boy	18	44.30	4.9	396	41	24
117586	Boy	18	46.70	8.1	286	29	22
110352	Boy	18	48.70	44.2	399	19	11
119454	Boy	18	51.70	6.6	322	43	24
121139	Boy	18	59.40	14.8	266	24	16
118715	Girl	12	13.80	3.0	174	9	19
118133	Girl	12	14.80	3.7	183	16	24
110245	Girl	12	16.00	4.2	115	10	15
117688	Girl	12	16.00	4.8	145	15	20
123486	Girl	12	16.10	3.5	206	8	16
121560	Girl	12	16.10	4.2	188	10	19
112588	Girl	12	16.20	3.7	158	9	14
119490	Girl	12	16.20	3.1	175	13	21
118032	Girl	12	16.70	4.6	139	14	31
121628	Girl	12	17.00	3.6	205	11	22
122593	Girl	12	17.00	5.2	109	14	22
113618	Girl	12	17.10	4.1	236	13	24
112688	Girl	12	17.30	3.4	173	24	26
109778	Girl	12	17.80	4.2	177	11	18
124137	Girl	12	18.30	3.9	219	12	23
119414	Girl	12	18.30	3.7	100	13	19

114408	Girl	12	18.40	6.5	192	9	15
122410	Girl	12	18.40	3.6	189	12	16
112443	Girl	12	18.50	5.9	255	21	21
109277	Girl	12	18.60	4.0	132	11	20
120755	Girl	12	18.70	3.9	211	13	19
124537	Girl	12	19.00	4.0	244	10	15
121617	Girl	12	19.30	3.9	194	10	16
109944	Girl	12	19.30	4.0	215	19	23
114776	Girl	12	19.50	6.3	168	11	19
117909	Girl	12	19.80	6.4	210	31	26
121704	Girl	12	20.20	4.1	164	10	17
116879	Girl	12	20.30	4.1	187	8	14
117452	Girl	12	20.30	4.4	181	10	17
117801	Girl	12	20.50	7.1	194	13	16
115903	Girl	12	20.80	5.7	224	13	18
119956	Girl	12	21.30	4.3	176	10	15
112662	Girl	12	21.50	5.9	261	10	15
120294	Girl	12	21.70	5.0	158	8	14
112854	Girl	12	21.70	4.5	195	8	15
119862	Girl	12	21.70	5.0	288	9	15
120877	Girl	12	21.80	3.6	187	14	18
122087	Girl	12	21.80	2.4	103	20	20
119018	Girl	12	21.90	3.3	100	10	22
122591	Girl	12	21.90	5.8	223	17	21
120184	Girl	12	22.00	5.1	202	8	17
114714	Girl	12	22.10	3.2	167	18	21
119336	Girl	12	22.30	4.6	194	9	19
123559	Girl	12	22.60	8.0	242	7	19
123676	Girl	12	22.80	5.3	220	11	16
110880	Girl	12	23.10	5.8	261	17	20
118928	Girl	12	23.60	3.7	232	10	18
119046	Girl	12	24.00	27.6	400	8	14
118260	Girl	12	24.00	4.3	210	10	15
120705	Girl	12	24.00	3.7	213	14	19
113783	Girl	12	24.10	5.8	237	23	20
118625	Girl	12	24.30	2.8	236	8	15
121189	Girl	12	24.40	3.7	198	9	21
115496	Girl	12	24.40	4.2	183	26	22
112698	Girl	12	24.50	4.9	186	9	13
119093	Girl	12	24.60	4.9	100	17	19
122994	Girl	12	24.70	3.5	188	15	22
118798	Girl	12	24.80	4.9	187	11	17
112730	Girl	12	24.80	5.6	218	22	24
120337	Girl	12	25.60	3.6	213	9	14
123561	Girl	12	25.80	4.4	243	16	18
112872	Girl	12	26.00	3.3	228	9	17
119268	Girl	12	26.10	4.0	264	15	17
119388	Girl	12	26.20	6.4	272	13	17
117479	Girl	12	26.40	4.6	259	12	16

116818	Girl	12	27.10	4.7	226	15	20
119153	Girl	12	28.70	8.1	226	8	13
115033	Girl	12	28.70	3.5	197	9	15
124027	Girl	12	29.50	3.8	298	14	16
109572	Girl	12	29.60	4.0	228	20	19
112573	Girl	12	30.30	5.1	223	41	31
110585	Girl	12	30.40	5.7	232	11	15
112196	Girl	12	30.50	6.6	220	12	18
121879	Girl	12	31.50	7.3	325	25	17
111521	Girl	12	32.00	5.0	271	9	12
120766	Girl	12	32.90	4.1	166	12	11
117770	Girl	12	34.50	4.6	295	13	20
112302	Girl	12	37.20	6.1	230	21	22
114678	Girl	12	42.60	5.2	319	7	14
117550	Girl	12	45.70	4.8	311	57	43
110746	Girl	13	15.10	5.6	145	9	18
112166	Girl	13	16.10	7.7	191	11	14
116774	Girl	13	16.30	3.9	174	11	21
120505	Girl	13	16.50	3.4	139	15	20
118366	Girl	13	16.70	4.7	227	16	20
111622	Girl	13	16.80	4.6	273	11	19
114033	Girl	13	17.20	4.4	161	10	22
117296	Girl	13	17.20	6.8	194	14	15
122693	Girl	13	17.30	4.0	133	9	21
109384	Girl	13	17.30	3.5	227	11	16
122706	Girl	13	17.40	3.4	240	11	19
123430	Girl	13	17.40	5.1	177	12	19
122326	Girl	13	17.50	4.2	153	9	13
112267	Girl	13	17.50	4.1	227	10	17
112776	Girl	13	17.60	5.0	208	9	18
116795	Girl	13	17.60	3.1	231	15	18
115054	Girl	13	17.80	4.2	100	7	13
118360	Girl	13	18.00	4.8	193	6	23
120175	Girl	13	18.10	3.5	159	9	17
115949	Girl	13	18.20	4.1	202	14	22
117443	Girl	13	18.40	3.8	160	7	17
112996	Girl	13	18.40	3.9	261	11	21
113043	Girl	13	18.60	5.1	158	6	15
115549	Girl	13	18.60	5.2	197	10	15
109777	Girl	13	18.90	3.9	149	9	22
111648	Girl	13	18.90	3.6	188	14	20
110923	Girl	13	19.00	9.0	261	11	14
109372	Girl	13	19.10	3.4	150	18	21
122157	Girl	13	19.20	6.6	184	8	18
111349	Girl	13	19.70	4.9	215	18	20
120852	Girl	13	19.80	5.3	169	11	15
111926	Girl	13	19.80	4.1	100	12	18
124005	Girl	13	20.20	3.0	216	9	15
113099	Girl	13	20.20	4.5	223	12	17

110601	Girl	13	20.40	5.9	166	13	14
123213	Girl	13	20.60	5.0	234	9	18
115002	Girl	13	20.70	4.5	190	15	21
112905	Girl	13	20.80	4.8	203	10	14
113721	Girl	13	21.10	5.9	191	11	15
119137	Girl	13	21.40	6.7	201	13	24
121434	Girl	13	21.70	4.1	123	8	15
121557	Girl	13	21.90	4.2	247	9	15
114479	Girl	13	22.10	3.2	162	12	19
124098	Girl	13	22.10	4.8	194	12	20
122075	Girl	13	22.30	4.2	185	11	18
110577	Girl	13	22.30	5.5	273	13	16
118281	Girl	13	22.40	6.4	218	9	25
113449	Girl	13	22.40	6.6	263	18	15
119284	Girl	13	22.80	5.8	201	15	18
121684	Girl	13	23.10	5.4	119	8	15
112061	Girl	13	23.20	6.1	246	5	18
116735	Girl	13	23.20	3.5	235	13	18
113557	Girl	13	23.80	4.9	213	9	14
118392	Girl	13	24.10	5.9	249	11	16
112896	Girl	13	24.20	6.9	155	16	27
113497	Girl	13	24.30	4.7	202	11	12
118200	Girl	13	24.30	4.0	183	15	17
113174	Girl	13	24.40	5.2	148	8	18
121529	Girl	13	24.70	5.3	232	8	14
116318	Girl	13	24.70	4.5	173	11	17
111127	Girl	13	25.00	3.6	199	9	15
117584	Girl	13	25.10	3.2	193	8	16
110249	Girl	13	25.10	4.8	174	14	25
124494	Girl	13	25.40	4.2	211	10	14
115404	Girl	13	26.00	3.2	169	17	14
110163	Girl	13	26.10	2.7	211	13	23
110486	Girl	13	26.50	4.1	349	15	22
120638	Girl	13	26.80	4.2	209	4	13
119468	Girl	13	26.90	5.2	175	6	14
121862	Girl	13	27.10	3.8	265	8	19
120318	Girl	13	27.10	4.3	262	11	11
115847	Girl	13	27.70	3.6	267	16	14
110360	Girl	13	28.00	4.8	231	9	15
121478	Girl	13	28.20	5.5	234	16	22
118757	Girl	13	28.30	5.5	263	9	18
114768	Girl	13	28.50	5.5	328	14	16
117741	Girl	13	29.00	5.3	315	18	19
121225	Girl	13	29.50	5.1	224	21	15
109829	Girl	13	30.20	9.3	280	12	13
123449	Girl	13	30.30	5.2	224	13	27
120805	Girl	13	30.80	3.0	356	16	18
114750	Girl	13	30.90	6.2	227	6	12
124343	Girl	13	30.90	3.6	230	10	16

109589	Girl	13	31.20	4.7	189	9	11
124727	Girl	13	31.40	4.6	320	16	18
113752	Girl	13	33.90	3.3	214	11	12
116361	Girl	13	34.80	6.7	274	14	13
114869	Girl	13	37.80	3.8	295	8	10
117366	Girl	13	39.40	4.0	284	29	37
110815	Girl	13	40.80	12.0	334	26	23
113495	Girl	13	41.60	5.0	215	7	17
110237	Girl	14	14.60	4.6	160	9	19
120811	Girl	14	16.20	4.9	129	12	18
122877	Girl	14	17.10	4.3	180	13	18
124307	Girl	14	17.80	10.5	275	7	17
109360	Girl	14	18.30	4.2	234	10	18
110154	Girl	14	18.40	9.7	262	11	15
115227	Girl	14	19.00	4.8	217	9	16
119690	Girl	14	19.40	3.9	208	8	18
119771	Girl	14	19.40	4.8	221	12	18
120009	Girl	14	19.40	4.3	195	14	19
115623	Girl	14	19.70	6.7	196	10	17
115553	Girl	14	19.90	5.9	220	10	14
112750	Girl	14	20.00	5.3	269	10	17
118717	Girl	14	20.10	3.6	210	11	15
113564	Girl	14	20.60	5.2	205	9	13
115114	Girl	14	20.60	4.1	248	10	19
116478	Girl	14	20.70	7.8	138	8	13
119129	Girl	14	20.80	5.3	203	11	23
117105	Girl	14	20.80	4.1	124	18	26
118346	Girl	14	20.90	3.0	209	10	17
112478	Girl	14	20.90	5.1	212	12	18
114233	Girl	14	21.00	4.4	259	13	14
111689	Girl	14	21.10	4.1	148	8	15
119362	Girl	14	21.10	4.9	172	10	16
117003	Girl	14	21.10	4.1	204	12	17
115981	Girl	14	21.20	6.3	185	8	14
109689	Girl	14	21.20	4.8	202	8	15
112668	Girl	14	21.20	4.9	157	10	17
114022	Girl	14	21.30	3.8	166	8	19
117272	Girl	14	21.50	6.6	167	15	13
109882	Girl	14	21.60	4.7	151	11	15
115839	Girl	14	21.70	3.9	229	10	12
117921	Girl	14	21.70	3.8	247	13	15
110345	Girl	14	21.70	5.7	157	16	19
119763	Girl	14	22.10	3.1	194	11	18
119539	Girl	14	22.20	4.0	189	13	22
115571	Girl	14	22.30	4.5	236	16	16
117892	Girl	14	22.30	3.3	210	17	22
120200	Girl	14	22.40	6.5	203	8	15
111694	Girl	14	22.60	21.0	320	36	28
121288	Girl	14	22.80	5.0	197	13	15

115296	Girl	14	22.80	4.8	145	17	23
110922	Girl	14	23.10	3.2	227	15	19
116255	Girl	14	23.60	3.6	201	15	21
118002	Girl	14	23.70	3.6	216	9	17
111686	Girl	14	23.80	3.6	145	23	22
121666	Girl	14	23.90	3.2	207	12	13
116108	Girl	14	24.20	4.6	202	7	14
112825	Girl	14	24.40	4.2	257	13	16
114964	Girl	14	24.50	5.2	174	11	10
110516	Girl	14	24.60	3.0	244	13	18
124639	Girl	14	24.70	6.6	166	10	14
120895	Girl	14	24.80	4.6	208	9	16
117689	Girl	14	24.90	4.3	169	16	24
113081	Girl	14	25.00	3.7	198	11	19
112017	Girl	14	25.10	11.6	282	9	17
113441	Girl	14	25.40	4.5	136	8	12
112255	Girl	14	25.60	5.4	216	12	20
117764	Girl	14	26.10	3.7	230	5	18
116491	Girl	14	26.10	5.0	234	12	20
114000	Girl	14	26.20	4.8	211	8	13
119439	Girl	14	26.40	3.2	206	10	18
115115	Girl	14	26.50	5.1	270	15	22
113579	Girl	14	26.80	5.0	261	12	16
124748	Girl	14	26.80	6.0	213	15	NA
110008	Girl	14	27.20	4.6	250	11	13
121004	Girl	14	27.50	6.5	272	19	17
117404	Girl	14	27.60	6.3	261	23	32
111822	Girl	14	27.80	5.3	234	8	12
113297	Girl	14	28.10	5.1	252	16	15
119110	Girl	14	28.20	6.0	170	12	19
123855	Girl	14	28.50	5.5	185	11	16
113711	Girl	14	28.60	6.4	288	10	18
122695	Girl	14	29.30	6.6	230	8	18
122967	Girl	14	29.80	4.2	300	15	19
116080	Girl	14	29.90	5.2	353	16	15
114749	Girl	14	30.10	3.6	221	13	15
121267	Girl	14	30.10	5.7	335	20	21
114662	Girl	14	30.60	4.8	100	11	18
121300	Girl	14	30.80	4.7	240	9	15
120980	Girl	14	30.90	4.4	221	25	23
113626	Girl	14	31.10	4.2	205	14	19
118902	Girl	14	31.30	3.6	253	9	16
112013	Girl	14	31.30	6.6	254	14	16
116597	Girl	14	33.80	5.0	255	6	10
121575	Girl	14	34.00	5.9	274	15	16
123014	Girl	14	35.10	3.5	198	8	17
118534	Girl	14	36.00	4.1	235	12	15
110049	Girl	14	37.30	3.4	226	8	11
116192	Girl	14	38.00	6.5	228	14	18

113611	Girl	14	39.90	4.1	297	13	15
112496	Girl	14	40.10	5.5	279	23	16
122869	Girl	14	41.80	7.8	355	25	22
122580	Girl	14	41.90	3.7	354	21	19
112795	Girl	14	45.60	5.2	278	11	16
110590	Girl	15	17.10	3.7	141	9	15
113759	Girl	15	17.20	4.6	166	9	14
109556	Girl	15	17.60	4.0	135	11	16
111931	Girl	15	17.90	5.9	180	12	19
120365	Girl	15	18.50	3.9	189	8	15
119431	Girl	15	18.50	4.2	157	12	18
113806	Girl	15	18.60	2.9	115	9	16
109399	Girl	15	19.00	4.0	204	13	21
117935	Girl	15	19.10	4.3	215	16	23
118667	Girl	15	19.20	5.0	232	14	20
124694	Girl	15	19.50	4.6	168	15	23
123207	Girl	15	19.60	4.1	100	8	15
119125	Girl	15	19.60	5.2	231	10	18
124682	Girl	15	20.00	3.4	188	10	19
116055	Girl	15	20.10	3.6	176	7	15
111989	Girl	15	20.20	3.8	190	8	12
112533	Girl	15	20.50	4.4	165	7	17
111090	Girl	15	20.50	3.1	205	10	14
123443	Girl	15	20.60	2.7	195	9	14
111964	Girl	15	20.70	4.7	210	9	17
119865	Girl	15	21.40	4.0	242	12	19
110044	Girl	15	21.60	5.2	218	15	17
118830	Girl	15	21.70	4.4	255	9	14
124301	Girl	15	21.90	3.7	178	11	20
112232	Girl	15	21.90	5.1	165	12	19
115429	Girl	15	22.20	2.6	202	3	12
110433	Girl	15	22.20	5.8	192	8	14
123629	Girl	15	22.30	3.8	208	10	15
118325	Girl	15	22.40	3.6	237	6	15
116797	Girl	15	22.50	4.3	310	12	18
114362	Girl	15	22.60	3.4	255	12	26
118070	Girl	15	22.60	3.7	164	15	21
117658	Girl	15	22.90	4.6	200	15	16
111684	Girl	15	23.20	4.1	228	11	15
115282	Girl	15	23.40	4.9	172	37	33
112311	Girl	15	23.60	5.5	201	7	14
123480	Girl	15	23.80	7.9	254	13	17
109702	Girl	15	23.90	2.8	158	8	13
121409	Girl	15	24.20	3.3	255	8	16
116427	Girl	15	24.30	4.6	204	12	17
113829	Girl	15	24.70	3.5	196	13	19
113208	Girl	15	24.70	4.9	221	14	18
109707	Girl	15	24.80	5.1	221	11	17
124754	Girl	15	25.20	5.5	265	9	12

111251	Girl	15	25.80	7.4	266	11	11
120138	Girl	15	25.90	2.8	171	8	14
124134	Girl	15	26.10	4.4	275	14	15
120306	Girl	15	26.10	4.8	229	15	18
117228	Girl	15	26.20	3.6	251	10	15
113190	Girl	15	26.40	4.1	301	28	21
118255	Girl	15	27.40	4.4	169	9	13
113645	Girl	15	27.50	4.4	231	9	19
112119	Girl	15	27.90	4.6	224	9	17
122402	Girl	15	27.90	4.8	251	24	16
111485	Girl	15	28.40	5.2	301	22	17
123051	Girl	15	28.90	4.8	217	13	16
124767	Girl	15	29.90	5.0	252	12	16
116497	Girl	15	29.90	3.3	238	19	17
110963	Girl	15	30.10	4.3	291	18	16
117459	Girl	15	30.90	5.1	189	11	13
111295	Girl	15	31.40	6.7	313	66	32
122958	Girl	15	32.20	4.7	302	25	31
113243	Girl	15	33.20	6.9	289	22	15
117896	Girl	15	33.20	4.9	302	33	24
119657	Girl	15	33.60	3.7	305	23	19
112229	Girl	15	34.00	6.3	232	10	16
123012	Girl	15	34.40	6.0	274	11	14
114277	Girl	15	34.40	5.3	232	22	25
120716	Girl	15	34.60	4.3	279	22	17
116659	Girl	15	34.90	3.7	215	12	18
110072	Girl	15	36.50	6.1	354	17	38
117096	Girl	15	37.80	6.4	243	12	11
121859	Girl	15	39.40	4.4	361	12	13
109729	Girl	15	40.00	5.0	326	19	14
116751	Girl	15	41.20	4.7	303	47	22
118326	Girl	15	41.80	4.2	221	9	14
111681	Girl	15	43.90	4.0	248	11	15
121541	Girl	15	44.80	5.6	274	12	17
115106	Girl	15	45.60	10.3	336	13	16
122422	Girl	15	45.60	6.0	359	31	20
111091	Girl	15	45.90	5.8	288	12	10
115152	Girl	16	15.70	5.1	166	8	14
109692	Girl	16	16.30	4.7	193	27	26
119174	Girl	16	17.70	6.0	203	11	15
109698	Girl	16	17.90	5.8	168	11	14
110003	Girl	16	18.20	4.2	170	11	17
117449	Girl	16	18.30	3.7	161	8	17
111748	Girl	16	18.30	4.3	150	16	17
121119	Girl	16	18.40	2.5	193	8	13
110517	Girl	16	18.70	6.3	198	7	13
119682	Girl	16	19.20	6.8	195	9	17
114952	Girl	16	19.20	5.0	212	9	17
117743	Girl	16	19.30	6.0	179	4	15

119052	Girl	16	19.40	4.8	212	7	13
119204	Girl	16	19.40	6.3	278	18	26
116401	Girl	16	19.50	3.9	194	8	13
116466	Girl	16	19.90	10.1	232	10	14
122969	Girl	16	19.90	4.5	244	10	18
113592	Girl	16	20.00	3.9	173	11	17
120086	Girl	16	20.10	7.6	100	19	25
118843	Girl	16	20.20	6.0	225	8	14
124681	Girl	16	20.30	3.5	204	10	15
122198	Girl	16	20.40	3.5	178	9	21
110022	Girl	16	20.40	5.4	175	10	15
111015	Girl	16	20.40	2.9	202	20	17
117122	Girl	16	20.50	4.0	193	8	16
117457	Girl	16	20.50	4.8	198	9	15
117684	Girl	16	20.60	3.9	215	9	15
123573	Girl	16	20.80	3.2	169	8	16
117805	Girl	16	21.00	6.2	204	8	13
118828	Girl	16	21.10	4.5	210	9	17
116069	Girl	16	21.10	4.6	159	13	16
122205	Girl	16	21.20	3.5	184	16	15
122224	Girl	16	21.30	5.1	251	7	13
124481	Girl	16	21.30	3.9	153	8	15
117381	Girl	16	21.40	5.2	130	9	14
112633	Girl	16	21.50	4.5	217	11	16
113260	Girl	16	21.60	4.0	213	11	17
115020	Girl	16	21.70	3.5	196	12	16
115892	Girl	16	21.70	3.1	134	17	27
113084	Girl	16	21.80	3.9	279	9	14
119601	Girl	16	22.00	3.8	201	16	17
122801	Girl	16	22.00	3.8	186	24	12
118570	Girl	16	22.40	6.4	383	11	19
122270	Girl	16	22.60	3.0	208	11	12
117001	Girl	16	22.80	4.5	206	11	14
111066	Girl	16	22.90	3.1	218	9	15
111509	Girl	16	22.90	5.3	221	15	16
122124	Girl	16	23.00	3.5	216	11	17
124733	Girl	16	23.10	4.3	238	10	20
118909	Girl	16	23.50	5.5	237	16	23
109781	Girl	16	23.80	4.3	216	10	18
109398	Girl	16	23.80	3.5	173	14	16
113422	Girl	16	23.80	3.2	300	14	15
116514	Girl	16	23.90	5.9	213	14	22
109804	Girl	16	24.00	3.1	225	27	20
115811	Girl	16	24.10	5.7	175	8	19
115593	Girl	16	24.10	4.0	233	9	14
110166	Girl	16	24.20	4.9	115	6	11
124318	Girl	16	24.40	6.1	152	22	21
120012	Girl	16	24.50	7.0	224	9	16
110020	Girl	16	24.50	3.4	216	11	17

110847	Girl	16	24.60	4.6	173	4	13
121908	Girl	16	24.60	3.6	223	10	18
116811	Girl	16	24.90	2.1	283	14	15
122899	Girl	16	25.00	3.5	268	14	22
119957	Girl	16	25.20	5.4	224	15	15
113670	Girl	16	25.30	4.0	200	9	16
109822	Girl	16	25.40	6.0	151	10	17
117503	Girl	16	25.60	5.1	292	19	19
114255	Girl	16	26.10	5.5	229	9	13
114496	Girl	16	26.10	3.7	200	10	21
118517	Girl	16	26.20	6.0	225	8	12
121164	Girl	16	26.30	5.7	165	20	16
124305	Girl	16	26.60	4.6	258	10	14
123145	Girl	16	26.80	4.7	201	15	14
110972	Girl	16	27.10	5.8	151	14	18
114684	Girl	16	27.10	4.8	292	36	25
112784	Girl	16	27.60	5.1	219	16	21
114004	Girl	16	27.90	3.5	218	15	17
111285	Girl	16	28.60	6.1	228	11	17
116230	Girl	16	29.20	4.5	203	9	15
111477	Girl	16	29.80	3.3	200	15	16
118763	Girl	16	30.40	5.1	275	19	21
111478	Girl	16	30.70	6.2	243	14	16
121843	Girl	16	30.90	5.5	273	17	15
117367	Girl	16	30.90	7.5	291	19	19
117345	Girl	16	31.10	10.6	310	14	14
114411	Girl	16	31.50	5.3	324	14	25
124139	Girl	16	31.60	5.3	269	15	16
109810	Girl	16	33.00	3.5	266	17	31
110727	Girl	16	33.00	4.6	251	19	20
116477	Girl	16	33.40	3.7	377	31	30
119303	Girl	16	34.20	3.0	236	19	17
124131	Girl	16	34.90	11.0	277	9	12
118130	Girl	16	35.30	5.9	282	16	16
122667	Girl	16	35.90	20.1	297	9	12
111756	Girl	16	36.40	5.3	206	13	15
112558	Girl	16	36.40	3.4	232	18	19
117246	Girl	16	38.10	5.1	264	19	18
122971	Girl	16	41.10	5.0	294	13	17
115533	Girl	16	41.40	4.4	246	11	14
115930	Girl	16	43.10	3.6	257	23	21
110694	Girl	16	44.80	5.3	269	10	13
121702	Girl	16	45.70	5.2	337	14	16
114599	Girl	16	46.60	3.4	339	14	14
122064	Girl	16	54.40	8.3	285	18	16
116827	Girl	17	17.70	5.1	242	6	14
114748	Girl	17	17.90	4.5	194	5	12
124140	Girl	17	18.20	4.4	222	8	13
122690	Girl	17	18.30	4.0	167	18	19

111438	Girl	17	18.40	3.6	182	2	12
117034	Girl	17	18.40	4.1	200	10	16
124395	Girl	17	18.70	3.8	166	21	21
122292	Girl	17	19.40	4.6	220	11	20
122299	Girl	17	19.60	4.6	179	19	19
120975	Girl	17	19.70	3.0	216	9	15
122538	Girl	17	20.00	6.1	146	14	32
110470	Girl	17	20.20	3.0	193	9	20
110444	Girl	17	20.20	4.3	158	11	14
121825	Girl	17	20.20	4.8	226	13	12
113686	Girl	17	20.30	3.7	246	13	16
124599	Girl	17	20.70	3.6	202	10	19
120357	Girl	17	20.70	3.2	156	12	20
109279	Girl	17	21.00	4.6	235	9	15
114192	Girl	17	21.20	4.2	206	11	22
112172	Girl	17	21.30	5.3	198	7	11
117361	Girl	17	21.30	3.1	205	9	12
116729	Girl	17	21.50	3.5	150	8	11
113329	Girl	17	21.60	3.8	174	9	18
115208	Girl	17	21.60	3.8	153	11	19
111955	Girl	17	21.80	3.7	197	11	17
124773	Girl	17	22.00	4.3	219	9	12
112892	Girl	17	22.10	7.2	225	8	15
110649	Girl	17	22.10	3.5	197	11	22
123422	Girl	17	22.20	5.1	144	19	23
123880	Girl	17	22.30	3.2	231	6	11
122533	Girl	17	22.30	3.9	208	12	16
114598	Girl	17	22.40	5.4	173	7	15
114689	Girl	17	22.40	4.9	239	9	12
115009	Girl	17	22.50	5.2	175	12	19
112203	Girl	17	22.60	3.7	253	15	16
122433	Girl	17	22.60	3.6	269	17	15
122267	Girl	17	22.70	3.3	112	8	15
110109	Girl	17	22.90	3.4	249	14	18
113845	Girl	17	22.90	4.5	246	62	29
117899	Girl	17	23.00	4.1	183	7	14
116155	Girl	17	23.10	3.3	214	20	19
116759	Girl	17	23.20	4.8	211	11	15
118848	Girl	17	23.40	5.2	162	11	16
117756	Girl	17	23.40	5.5	223	11	16
109387	Girl	17	23.40	5.2	230	12	15
115403	Girl	17	23.60	4.6	177	11	12
116134	Girl	17	23.90	4.0	155	14	16
123667	Girl	17	23.90	3.7	199	16	19
119661	Girl	17	24.10	6.0	217	9	15
111082	Girl	17	24.30	3.5	217	14	20
118212	Girl	17	24.30	5.1	362	14	20
119824	Girl	17	24.50	2.7	191	13	23
111369	Girl	17	24.50	4.8	257	17	14

110312	Girl	17	26.10	3.2	238	7	17
121902	Girl	17	26.10	3.8	183	9	18
117661	Girl	17	26.10	4.5	237	10	14
115690	Girl	17	26.20	5.7	186	15	14
122926	Girl	17	26.30	4.6	269	10	18
119566	Girl	17	26.30	4.3	209	12	14
115525	Girl	17	26.50	5.1	210	13	24
120256	Girl	17	26.60	5.3	214	13	20
120385	Girl	17	27.00	4.8	194	10	18
112033	Girl	17	27.00	4.1	230	15	14
117268	Girl	17	27.00	4.3	241	19	19
115933	Girl	17	27.20	4.3	252	13	14
109499	Girl	17	27.30	3.3	318	15	16
119477	Girl	17	28.00	5.7	217	7	11
123189	Girl	17	28.10	4.4	232	12	20
124299	Girl	17	28.20	4.4	268	10	16
112429	Girl	17	29.20	5.5	266	15	25
118410	Girl	17	29.40	6.1	251	10	15
112529	Girl	17	29.50	4.8	155	15	13
122279	Girl	17	29.50	4.7	213	26	24
120264	Girl	17	29.90	4.5	245	22	16
119198	Girl	17	30.40	3.7	214	11	12
118181	Girl	17	30.70	5.6	271	16	16
113205	Girl	17	31.00	5.8	314	26	21
110616	Girl	17	31.20	5.4	251	12	15
113772	Girl	17	31.30	5.5	313	30	23
119051	Girl	17	31.70	7.6	238	8	12
110745	Girl	17	32.40	5.8	205	20	26
115391	Girl	17	32.80	5.4	199	11	14
121399	Girl	17	33.10	4.1	177	13	17
113079	Girl	17	33.50	5.5	275	26	18
113748	Girl	17	34.00	6.5	263	19	22
111630	Girl	17	34.10	3.8	265	15	20
118273	Girl	17	34.10	3.9	277	25	22
115948	Girl	17	34.60	5.8	342	22	18
116126	Girl	17	35.50	4.2	222	11	12
121090	Girl	17	37.80	15.8	302	15	19
120866	Girl	17	38.40	6.5	333	45	28
117141	Girl	17	38.60	3.4	286	11	14
119012	Girl	17	38.60	5.1	243	23	37
119895	Girl	17	38.70	4.8	296	27	18
116690	Girl	17	39.40	4.9	258	10	14
113512	Girl	17	41.90	3.7	282	25	22
111131	Girl	17	43.00	5.5	189	19	13
112745	Girl	17	48.80	6.9	357	33	23
120302	Girl	17	50.50	10.8	319	9	11
120135	Girl	17	50.60	8.0	236	11	11
120951	Girl	17	51.60	10.7	337	24	19
113914	Girl	18	16.30	5.7	168	10	13

115545	Girl	18	16.40	4.2	168	11	13
124074	Girl	18	16.70	3.8	214	11	15
123681	Girl	18	17.60	5.0	198	13	17
114437	Girl	18	17.70	5.2	170	10	13
112388	Girl	18	17.80	3.4	102	7	15
113302	Girl	18	18.20	4.4	230	26	23
121919	Girl	18	18.30	4.9	221	9	19
116182	Girl	18	18.50	4.8	154	9	17
119167	Girl	18	18.90	3.6	209	14	20
115053	Girl	18	19.40	5.0	162	6	13
111773	Girl	18	19.50	4.8	146	7	16
116446	Girl	18	19.50	4.1	196	10	14
110895	Girl	18	19.50	4.0	176	13	16
113849	Girl	18	19.50	5.8	181	13	18
113513	Girl	18	19.60	3.7	217	17	18
117474	Girl	18	19.90	5.5	294	11	15
120990	Girl	18	20.00	3.5	165	7	17
110773	Girl	18	20.00	3.6	184	11	16
112479	Girl	18	20.30	6.1	150	11	18
111265	Girl	18	20.50	3.7	167	7	15
124786	Girl	18	20.50	6.2	180	12	14
119930	Girl	18	20.90	2.6	240	9	14
121091	Girl	18	21.40	4.5	256	8	15
121177	Girl	18	21.60	5.0	231	31	25
124594	Girl	18	21.80	3.4	218	14	15
123217	Girl	18	21.90	3.8	131	7	14
124626	Girl	18	22.00	4.5	225	7	13
115088	Girl	18	22.10	2.2	174	9	16
116664	Girl	18	22.30	4.6	208	10	16
123465	Girl	18	23.10	6.1	203	7	16
112599	Girl	18	23.20	4.5	249	12	19
116356	Girl	18	23.30	4.1	190	8	14
121483	Girl	18	23.30	5.0	196	13	17
116436	Girl	18	23.50	3.7	188	20	26
109873	Girl	18	23.50	3.9	236	21	26
118182	Girl	18	23.70	4.4	219	11	17
116046	Girl	18	23.70	7.3	201	18	24
115435	Girl	18	24.10	4.0	195	12	18
118981	Girl	18	24.10	5.1	197	15	15
122035	Girl	18	24.20	3.4	213	15	19
110769	Girl	18	24.40	4.4	165	11	15
116575	Girl	18	25.10	5.3	216	13	16
119569	Girl	18	25.20	3.5	230	15	21
112899	Girl	18	25.40	4.5	234	12	16
119929	Girl	18	25.50	3.4	224	10	11
117719	Girl	18	25.50	5.8	273	23	18
120020	Girl	18	26.10	5.8	169	9	12
121828	Girl	18	26.70	5.6	243	35	30
110063	Girl	18	27.10	3.0	193	7	12

124376	Girl	18	27.20	3.7	164	10	17
122278	Girl	18	27.30	4.4	220	11	17
120880	Girl	18	27.40	4.4	229	12	20
122143	Girl	18	27.80	5.4	223	14	21
121192	Girl	18	28.20	3.2	235	10	13
115806	Girl	18	28.30	4.9	335	13	14
119927	Girl	18	28.80	3.6	270	15	13
113593	Girl	18	29.70	5.6	266	16	18
119128	Girl	18	30.00	6.1	243	15	19
123286	Girl	18	30.90	3.5	280	12	15
111830	Girl	18	30.90	4.9	241	15	17
113743	Girl	18	31.10	6.6	321	24	20
121076	Girl	18	31.40	5.0	283	15	18
121258	Girl	18	32.40	8.7	266	150	69
113673	Girl	18	33.00	7.5	284	7	15
120458	Girl	18	33.50	5.7	317	41	31
119443	Girl	18	33.90	5.8	312	19	19
123054	Girl	18	34.60	5.9	259	18	17
122127	Girl	18	35.70	4.0	204	8	10
118176	Girl	18	36.30	5.5	225	12	13
122586	Girl	18	36.50	8.8	215	12	17
113328	Girl	18	36.50	4.4	218	12	17
116338	Girl	18	38.20	2.7	275	7	12
112420	Girl	18	38.70	5.3	332	29	28
109542	Girl	18	38.90	3.6	337	14	18
120099	Girl	18	45.90	6.4	245	13	14
124276	Girl	18	72.60	71.3	396	53	31

sex	year	age	Prevalence (%)
Boys	1975	12	5.92
Boys	1976	12	5.98
Boys	1977	12	6.03
Boys	1978	12	6.08
Boys	1979	12	6.13
Boys	1980	12	6.18
Boys	1981	12	6.23
Boys	1982	12	6.28
Boys	1983	12	6.33
Boys	1984	12	6.38
Boys	1985	12	6.41
Boys	1986	12	6.45
Boys	1987	12	6.48
Boys	1988	12	6.51
Boys	1989	12	6.55
Boys	1990	12	6.61
Boys	1991	12	6.67
Boys	1992	12	6.75
Boys	1993	12	6.84
Boys	1994	12	6.94
Boys	1995	12	7.04
Boys	1996	12	7.15
Boys	1997	12	7.26
Boys	1998	12	7.37
Boys	1999	12	7.48
Boys	2000	12	7.58
Boys	2001	12	7.66
Boys	2002	12	7.73
Boys	2003	12	7.79
Boys	2004	12	7.85
Boys	2005	12	7.92
Boys	2006	12	7.99
Boys	2007	12	8.06
Boys	2008	12	8.14
Boys	2009	12	8.22
Boys	2010	12	8.32
Boys	2011	12	8.42
Boys	2012	12	8.53
Boys	2013	12	8.66
Boys	2014	12	8.79
Boys	2015	12	8.94
Boys	2016	12	9.10
Boys	1975	13	7.29
Boys	1976	13	7.35
Boys	1977	13	7.41
Boys	1978	13	7.47
Boys	1979	13	7.53
Boys	1980	13	7.59
Boys	1981	13	7.65
Boys	1982	13	7.71

Boys	1983	13	7.77
Boys	1984	13	7.82
Boys	1985	13	7.86
Boys	1986	13	7.90
Boys	1987	13	7.94
Boys	1988	13	7.98
Boys	1989	13	8.02
Boys	1990	13	8.09
Boys	1991	13	8.16
Boys	1992	13	8.26
Boys	1993	13	8.36
Boys	1994	13	8.47
Boys	1995	13	8.59
Boys	1996	13	8.72
Boys	1997	13	8.85
Boys	1998	13	8.99
Boys	1999	13	9.11
Boys	2000	13	9.22
Boys	2001	13	9.32
Boys	2002	13	9.41
Boys	2003	13	9.48
Boys	2004	13	9.55
Boys	2005	13	9.63
Boys	2006	13	9.71
Boys	2007	13	9.79
Boys	2008	13	9.89
Boys	2009	13	9.99
Boys	2010	13	10.09
Boys	2011	13	10.22
Boys	2012	13	10.35
Boys	2013	13	10.50
Boys	2014	13	10.66
Boys	2015	13	10.84
Boys	2016	13	11.03
Boys	1975	14	8.93
Boys	1976	14	9.00
Boys	1977	14	9.07
Boys	1978	14	9.13
Boys	1979	14	9.19
Boys	1980	14	9.26
Boys	1981	14	9.32
Boys	1982	14	9.39
Boys	1983	14	9.45
Boys	1984	14	9.51
Boys	1985	14	9.56
Boys	1986	14	9.60
Boys	1987	14	9.64
Boys	1988	14	9.68
Boys	1989	14	9.73
Boys	1990	14	9.80
Boys	1991	14	9.88

Boys	1992	14	9.99
Boys	1993	14	10.10
Boys	1994	14	10.23
Boys	1995	14	10.36
Boys	1996	14	10.50
Boys	1997	14	10.65
Boys	1998	14	10.80
Boys	1999	14	10.94
Boys	2000	14	11.07
Boys	2001	14	11.17
Boys	2002	14	11.27
Boys	2003	14	11.35
Boys	2004	14	11.43
Boys	2005	14	11.51
Boys	2006	14	11.60
Boys	2007	14	11.69
Boys	2008	14	11.80
Boys	2009	14	11.91
Boys	2010	14	12.03
Boys	2011	14	12.17
Boys	2012	14	12.32
Boys	2013	14	12.48
Boys	2014	14	12.67
Boys	2015	14	12.86
Boys	2016	14	13.08
Boys	1975	15	10.81
Boys	1976	15	10.88
Boys	1977	15	10.97
Boys	1978	15	11.06
Boys	1979	15	11.16
Boys	1980	15	11.25
Boys	1981	15	11.33
Boys	1982	15	11.41
Boys	1983	15	11.49
Boys	1984	15	11.57
Boys	1985	15	11.65
Boys	1986	15	11.74
Boys	1987	15	11.84
Boys	1988	15	11.94
Boys	1989	15	12.03
Boys	1990	15	12.12
Boys	1991	15	12.19
Boys	1992	15	12.24
Boys	1993	15	12.28
Boys	1994	15	12.32
Boys	1995	15	12.39
Boys	1996	15	12.47
Boys	1997	15	12.58
Boys	1998	15	12.71
Boys	1999	15	12.85
Boys	2000	15	13.00

Boys	2001	15	13.17
Boys	2002	15	13.35
Boys	2003	15	13.53
Boys	2004	15	13.70
Boys	2005	15	13.86
Boys	2006	15	14.00
Boys	2007	15	14.12
Boys	2008	15	14.23
Boys	2009	15	14.34
Boys	2010	15	14.45
Boys	2011	15	14.57
Boys	2012	15	14.70
Boys	2013	15	14.83
Boys	2014	15	14.97
Boys	2015	15	15.14
Boys	2016	15	15.31
Boys	1975	16	12.78
Boys	1976	16	12.85
Boys	1977	16	12.94
Boys	1978	16	13.04
Boys	1979	16	13.13
Boys	1980	16	13.23
Boys	1981	16	13.31
Boys	1982	16	13.40
Boys	1983	16	13.48
Boys	1984	16	13.56
Boys	1985	16	13.64
Boys	1986	16	13.73
Boys	1987	16	13.83
Boys	1988	16	13.93
Boys	1989	16	14.03
Boys	1990	16	14.12
Boys	1991	16	14.18
Boys	1992	16	14.23
Boys	1993	16	14.27
Boys	1994	16	14.31
Boys	1995	16	14.38
Boys	1996	16	14.47
Boys	1997	16	14.58
Boys	1998	16	14.71
Boys	1999	16	14.85
Boys	2000	16	15.01
Boys	2001	16	15.19
Boys	2002	16	15.37
Boys	2003	16	15.56
Boys	2004	16	15.74
Boys	2005	16	15.90
Boys	2006	16	16.04
Boys	2007	16	16.17
Boys	2008	16	16.28
Boys	2009	16	16.39

Boys	2010	16	16.51
Boys	2011	16	16.63
Boys	2012	16	16.75
Boys	2013	16	16.89
Boys	2014	16	17.04
Boys	2015	16	17.21
Boys	2016	16	17.39
Boys	1975	17	14.80
Boys	1976	17	14.87
Boys	1977	17	14.97
Boys	1978	17	15.07
Boys	1979	17	15.17
Boys	1980	17	15.26
Boys	1981	17	15.35
Boys	1982	17	15.44
Boys	1983	17	15.52
Boys	1984	17	15.60
Boys	1985	17	15.68
Boys	1986	17	15.77
Boys	1987	17	15.88
Boys	1988	17	15.98
Boys	1989	17	16.08
Boys	1990	17	16.17
Boys	1991	17	16.23
Boys	1992	17	16.28
Boys	1993	17	16.32
Boys	1994	17	16.36
Boys	1995	17	16.42
Boys	1996	17	16.51
Boys	1997	17	16.63
Boys	1998	17	16.76
Boys	1999	17	16.91
Boys	2000	17	17.08
Boys	2001	17	17.25
Boys	2002	17	17.44
Boys	2003	17	17.64
Boys	2004	17	17.82
Boys	2005	17	17.99
Boys	2006	17	18.13
Boys	2007	17	18.26
Boys	2008	17	18.38
Boys	2009	17	18.49
Boys	2010	17	18.61
Boys	2011	17	18.73
Boys	2012	17	18.86
Boys	2013	17	19.00
Boys	2014	17	19.15
Boys	2015	17	19.32
Boys	2016	17	19.52
Boys	1975	18	16.83
Boys	1976	18	16.91

Boys	1977	18	17.01
Boys	1978	18	17.11
Boys	1979	18	17.22
Boys	1980	18	17.32
Boys	1981	18	17.41
Boys	1982	18	17.50
Boys	1983	18	17.59
Boys	1984	18	17.67
Boys	1985	18	17.76
Boys	1986	18	17.85
Boys	1987	18	17.96
Boys	1988	18	18.07
Boys	1989	18	18.17
Boys	1990	18	18.26
Boys	1991	18	18.33
Boys	1992	18	18.37
Boys	1993	18	18.41
Boys	1994	18	18.46
Boys	1995	18	18.52
Boys	1996	18	18.61
Boys	1997	18	18.73
Boys	1998	18	18.87
Boys	1999	18	19.03
Boys	2000	18	19.20
Boys	2001	18	19.39
Boys	2002	18	19.59
Boys	2003	18	19.79
Boys	2004	18	19.98
Boys	2005	18	20.16
Boys	2006	18	20.31
Boys	2007	18	20.45
Boys	2008	18	20.57
Boys	2009	18	20.68
Boys	2010	18	20.80
Boys	2011	18	20.93
Boys	2012	18	21.07
Boys	2013	18	21.21
Boys	2014	18	21.37
Boys	2015	18	21.55
Boys	2016	18	21.75
Girls	1975	12	5.84
Girls	1976	12	5.87
Girls	1977	12	5.91
Girls	1978	12	5.94
Girls	1979	12	5.97
Girls	1980	12	6.00
Girls	1981	12	6.04
Girls	1982	12	6.07
Girls	1983	12	6.11
Girls	1984	12	6.14
Girls	1985	12	6.17

Girls	1986	12	6.20
Girls	1987	12	6.23
Girls	1988	12	6.26
Girls	1989	12	6.30
Girls	1990	12	6.34
Girls	1991	12	6.39
Girls	1992	12	6.45
Girls	1993	12	6.51
Girls	1994	12	6.57
Girls	1995	12	6.63
Girls	1996	12	6.70
Girls	1997	12	6.76
Girls	1998	12	6.83
Girls	1999	12	6.89
Girls	2000	12	6.95
Girls	2001	12	7.01
Girls	2002	12	7.06
Girls	2003	12	7.12
Girls	2004	12	7.17
Girls	2005	12	7.23
Girls	2006	12	7.29
Girls	2007	12	7.35
Girls	2008	12	7.42
Girls	2009	12	7.49
Girls	2010	12	7.57
Girls	2011	12	7.64
Girls	2012	12	7.73
Girls	2013	12	7.82
Girls	2014	12	7.91
Girls	2015	12	8.02
Girls	2016	12	8.13
Girls	1975	13	7.23
Girls	1976	13	7.27
Girls	1977	13	7.31
Girls	1978	13	7.34
Girls	1979	13	7.38
Girls	1980	13	7.42
Girls	1981	13	7.46
Girls	1982	13	7.50
Girls	1983	13	7.54
Girls	1984	13	7.58
Girls	1985	13	7.61
Girls	1986	13	7.65
Girls	1987	13	7.68
Girls	1988	13	7.72
Girls	1989	13	7.76
Girls	1990	13	7.81
Girls	1991	13	7.87
Girls	1992	13	7.93
Girls	1993	13	8.00
Girls	1994	13	8.07

Girls	1995	13	8.15
Girls	1996	13	8.22
Girls	1997	13	8.30
Girls	1998	13	8.37
Girls	1999	13	8.44
Girls	2000	13	8.51
Girls	2001	13	8.58
Girls	2002	13	8.64
Girls	2003	13	8.71
Girls	2004	13	8.77
Girls	2005	13	8.84
Girls	2006	13	8.91
Girls	2007	13	8.98
Girls	2008	13	9.05
Girls	2009	13	9.14
Girls	2010	13	9.22
Girls	2011	13	9.31
Girls	2012	13	9.41
Girls	2013	13	9.51
Girls	2014	13	9.62
Girls	2015	13	9.74
Girls	2016	13	9.86
Girls	1975	14	8.71
Girls	1976	14	8.75
Girls	1977	14	8.80
Girls	1978	14	8.84
Girls	1979	14	8.88
Girls	1980	14	8.92
Girls	1981	14	8.96
Girls	1982	14	9.01
Girls	1983	14	9.05
Girls	1984	14	9.09
Girls	1985	14	9.13
Girls	1986	14	9.17
Girls	1987	14	9.21
Girls	1988	14	9.25
Girls	1989	14	9.30
Girls	1990	14	9.35
Girls	1991	14	9.42
Girls	1992	14	9.49
Girls	1993	14	9.56
Girls	1994	14	9.64
Girls	1995	14	9.72
Girls	1996	14	9.80
Girls	1997	14	9.89
Girls	1998	14	9.97
Girls	1999	14	10.05
Girls	2000	14	10.12
Girls	2001	14	10.19
Girls	2002	14	10.26
Girls	2003	14	10.33

Girls	2004	14	10.40
Girls	2005	14	10.48
Girls	2006	14	10.55
Girls	2007	14	10.63
Girls	2008	14	10.71
Girls	2009	14	10.80
Girls	2010	14	10.90
Girls	2011	14	10.99
Girls	2012	14	11.10
Girls	2013	14	11.21
Girls	2014	14	11.33
Girls	2015	14	11.46
Girls	2016	14	11.60
Girls	1975	15	10.11
Girls	1976	15	10.17
Girls	1977	15	10.23
Girls	1978	15	10.29
Girls	1979	15	10.35
Girls	1980	15	10.41
Girls	1981	15	10.47
Girls	1982	15	10.52
Girls	1983	15	10.57
Girls	1984	15	10.61
Girls	1985	15	10.66
Girls	1986	15	10.72
Girls	1987	15	10.78
Girls	1988	15	10.85
Girls	1989	15	10.91
Girls	1990	15	10.97
Girls	1991	15	11.02
Girls	1992	15	11.07
Girls	1993	15	11.11
Girls	1994	15	11.16
Girls	1995	15	11.22
Girls	1996	15	11.28
Girls	1997	15	11.35
Girls	1998	15	11.43
Girls	1999	15	11.52
Girls	2000	15	11.61
Girls	2001	15	11.70
Girls	2002	15	11.80
Girls	2003	15	11.89
Girls	2004	15	11.98
Girls	2005	15	12.07
Girls	2006	15	12.16
Girls	2007	15	12.25
Girls	2008	15	12.33
Girls	2009	15	12.42
Girls	2010	15	12.51
Girls	2011	15	12.60
Girls	2012	15	12.70

Girls	2013	15	12.81
Girls	2014	15	12.92
Girls	2015	15	13.04
Girls	2016	15	13.16
Girls	1975	16	11.22
Girls	1976	16	11.28
Girls	1977	16	11.34
Girls	1978	16	11.41
Girls	1979	16	11.47
Girls	1980	16	11.53
Girls	1981	16	11.59
Girls	1982	16	11.64
Girls	1983	16	11.69
Girls	1984	16	11.74
Girls	1985	16	11.80
Girls	1986	16	11.85
Girls	1987	16	11.92
Girls	1988	16	11.98
Girls	1989	16	12.05
Girls	1990	16	12.11
Girls	1991	16	12.17
Girls	1992	16	12.22
Girls	1993	16	12.26
Girls	1994	16	12.31
Girls	1995	16	12.37
Girls	1996	16	12.44
Girls	1997	16	12.51
Girls	1998	16	12.60
Girls	1999	16	12.69
Girls	2000	16	12.78
Girls	2001	16	12.88
Girls	2002	16	12.98
Girls	2003	16	13.07
Girls	2004	16	13.17
Girls	2005	16	13.26
Girls	2006	16	13.35
Girls	2007	16	13.44
Girls	2008	16	13.53
Girls	2009	16	13.62
Girls	2010	16	13.72
Girls	2011	16	13.81
Girls	2012	16	13.92
Girls	2013	16	14.03
Girls	2014	16	14.14
Girls	2015	16	14.26
Girls	2016	16	14.39
Girls	1975	17	12.03
Girls	1976	17	12.09
Girls	1977	17	12.15
Girls	1978	17	12.22
Girls	1979	17	12.29

Girls	1980	17	12.35
Girls	1981	17	12.41
Girls	1982	17	12.47
Girls	1983	17	12.52
Girls	1984	17	12.58
Girls	1985	17	12.63
Girls	1986	17	12.69
Girls	1987	17	12.76
Girls	1988	17	12.83
Girls	1989	17	12.90
Girls	1990	17	12.96
Girls	1991	17	13.02
Girls	1992	17	13.07
Girls	1993	17	13.12
Girls	1994	17	13.17
Girls	1995	17	13.23
Girls	1996	17	13.31
Girls	1997	17	13.39
Girls	1998	17	13.48
Girls	1999	17	13.57
Girls	2000	17	13.67
Girls	2001	17	13.77
Girls	2002	17	13.87
Girls	2003	17	13.98
Girls	2004	17	14.08
Girls	2005	17	14.18
Girls	2006	17	14.27
Girls	2007	17	14.37
Girls	2008	17	14.46
Girls	2009	17	14.56
Girls	2010	17	14.66
Girls	2011	17	14.76
Girls	2012	17	14.87
Girls	2013	17	14.98
Girls	2014	17	15.10
Girls	2015	17	15.23
Girls	2016	17	15.37
Girls	1975	18	12.62
Girls	1976	18	12.68
Girls	1977	18	12.75
Girls	1978	18	12.83
Girls	1979	18	12.90
Girls	1980	18	12.97
Girls	1981	18	13.04
Girls	1982	18	13.10
Girls	1983	18	13.15
Girls	1984	18	13.21
Girls	1985	18	13.27
Girls	1986	18	13.34
Girls	1987	18	13.41
Girls	1988	18	13.48

Girls	1989	18	13.56
Girls	1990	18	13.63
Girls	1991	18	13.70
Girls	1992	18	13.75
Girls	1993	18	13.80
Girls	1994	18	13.86
Girls	1995	18	13.93
Girls	1996	18	14.01
Girls	1997	18	14.10
Girls	1998	18	14.19
Girls	1999	18	14.30
Girls	2000	18	14.41
Girls	2001	18	14.52
Girls	2002	18	14.63
Girls	2003	18	14.74
Girls	2004	18	14.85
Girls	2005	18	14.96
Girls	2006	18	15.07
Girls	2007	18	15.17
Girls	2008	18	15.27
Girls	2009	18	15.37
Girls	2010	18	15.48
Girls	2011	18	15.59
Girls	2012	18	15.71
Girls	2013	18	15.83
Girls	2014	18	15.96
Girls	2015	18	16.10
Girls	2016	18	16.25

sex	year	age	Prevalence (%)
Boys	1975	12	0.56
Boys	1976	12	0.57
Boys	1977	12	0.58
Boys	1978	12	0.59
Boys	1979	12	0.59
Boys	1980	12	0.60
Boys	1981	12	0.61
Boys	1982	12	0.62
Boys	1983	12	0.62
Boys	1984	12	0.63
Boys	1985	12	0.64
Boys	1986	12	0.64
Boys	1987	12	0.65
Boys	1988	12	0.65
Boys	1989	12	0.66
Boys	1990	12	0.67
Boys	1991	12	0.68
Boys	1992	12	0.69
Boys	1993	12	0.70
Boys	1994	12	0.72
Boys	1995	12	0.73
Boys	1996	12	0.75
Boys	1997	12	0.77
Boys	1998	12	0.78
Boys	1999	12	0.80
Boys	2000	12	0.81
Boys	2001	12	0.83
Boys	2002	12	0.84
Boys	2003	12	0.85
Boys	2004	12	0.86
Boys	2005	12	0.87
Boys	2006	12	0.88
Boys	2007	12	0.89
Boys	2008	12	0.90
Boys	2009	12	0.92
Boys	2010	12	0.93
Boys	2011	12	0.95
Boys	2012	12	0.96
Boys	2013	12	0.98
Boys	2014	12	1.00
Boys	2015	12	1.03
Boys	2016	12	1.05
Boys	1975	13	0.76
Boys	1976	13	0.77
Boys	1977	13	0.78
Boys	1978	13	0.79
Boys	1979	13	0.80
Boys	1980	13	0.81
Boys	1981	13	0.82
Boys	1982	13	0.83

Boys	1983	13	0.84
Boys	1984	13	0.85
Boys	1985	13	0.85
Boys	1986	13	0.86
Boys	1987	13	0.87
Boys	1988	13	0.88
Boys	1989	13	0.89
Boys	1990	13	0.90
Boys	1991	13	0.91
Boys	1992	13	0.93
Boys	1993	13	0.95
Boys	1994	13	0.97
Boys	1995	13	0.99
Boys	1996	13	1.01
Boys	1997	13	1.03
Boys	1998	13	1.06
Boys	1999	13	1.08
Boys	2000	13	1.10
Boys	2001	13	1.12
Boys	2002	13	1.13
Boys	2003	13	1.15
Boys	2004	13	1.16
Boys	2005	13	1.17
Boys	2006	13	1.19
Boys	2007	13	1.21
Boys	2008	13	1.22
Boys	2009	13	1.24
Boys	2010	13	1.26
Boys	2011	13	1.28
Boys	2012	13	1.31
Boys	2013	13	1.33
Boys	2014	13	1.36
Boys	2015	13	1.39
Boys	2016	13	1.43
Boys	1975	14	1.00
Boys	1976	14	1.01
Boys	1977	14	1.03
Boys	1978	14	1.04
Boys	1979	14	1.05
Boys	1980	14	1.07
Boys	1981	14	1.08
Boys	1982	14	1.09
Boys	1983	14	1.10
Boys	1984	14	1.12
Boys	1985	14	1.13
Boys	1986	14	1.14
Boys	1987	14	1.15
Boys	1988	14	1.16
Boys	1989	14	1.17
Boys	1990	14	1.19
Boys	1991	14	1.20

Boys	1992	14	1.23
Boys	1993	14	1.25
Boys	1994	14	1.28
Boys	1995	14	1.30
Boys	1996	14	1.33
Boys	1997	14	1.36
Boys	1998	14	1.39
Boys	1999	14	1.42
Boys	2000	14	1.45
Boys	2001	14	1.47
Boys	2002	14	1.49
Boys	2003	14	1.51
Boys	2004	14	1.53
Boys	2005	14	1.55
Boys	2006	14	1.57
Boys	2007	14	1.59
Boys	2008	14	1.61
Boys	2009	14	1.64
Boys	2010	14	1.66
Boys	2011	14	1.69
Boys	2012	14	1.73
Boys	2013	14	1.76
Boys	2014	14	1.80
Boys	2015	14	1.84
Boys	2016	14	1.89
Boys	1975	15	1.30
Boys	1976	15	1.31
Boys	1977	15	1.33
Boys	1978	15	1.35
Boys	1979	15	1.37
Boys	1980	15	1.40
Boys	1981	15	1.42
Boys	1982	15	1.44
Boys	1983	15	1.45
Boys	1984	15	1.47
Boys	1985	15	1.49
Boys	1986	15	1.52
Boys	1987	15	1.54
Boys	1988	15	1.56
Boys	1989	15	1.59
Boys	1990	15	1.61
Boys	1991	15	1.63
Boys	1992	15	1.65
Boys	1993	15	1.66
Boys	1994	15	1.67
Boys	1995	15	1.69
Boys	1996	15	1.71
Boys	1997	15	1.74
Boys	1998	15	1.77
Boys	1999	15	1.81
Boys	2000	15	1.84

Boys	2001	15	1.88
Boys	2002	15	1.93
Boys	2003	15	1.97
Boys	2004	15	2.01
Boys	2005	15	2.05
Boys	2006	15	2.09
Boys	2007	15	2.12
Boys	2008	15	2.15
Boys	2009	15	2.18
Boys	2010	15	2.21
Boys	2011	15	2.24
Boys	2012	15	2.27
Boys	2013	15	2.31
Boys	2014	15	2.35
Boys	2015	15	2.39
Boys	2016	15	2.44
Boys	1975	16	1.63
Boys	1976	16	1.64
Boys	1977	16	1.67
Boys	1978	16	1.69
Boys	1979	16	1.72
Boys	1980	16	1.74
Boys	1981	16	1.76
Boys	1982	16	1.79
Boys	1983	16	1.81
Boys	1984	16	1.83
Boys	1985	16	1.86
Boys	1986	16	1.88
Boys	1987	16	1.91
Boys	1988	16	1.94
Boys	1989	16	1.97
Boys	1990	16	1.99
Boys	1991	16	2.02
Boys	1992	16	2.03
Boys	1993	16	2.05
Boys	1994	16	2.07
Boys	1995	16	2.09
Boys	1996	16	2.11
Boys	1997	16	2.14
Boys	1998	16	2.18
Boys	1999	16	2.22
Boys	2000	16	2.27
Boys	2001	16	2.32
Boys	2002	16	2.37
Boys	2003	16	2.42
Boys	2004	16	2.47
Boys	2005	16	2.52
Boys	2006	16	2.56
Boys	2007	16	2.60
Boys	2008	16	2.63
Boys	2009	16	2.67

Boys	2010	16	2.70
Boys	2011	16	2.74
Boys	2012	16	2.78
Boys	2013	16	2.82
Boys	2014	16	2.87
Boys	2015	16	2.92
Boys	2016	16	2.97
Boys	1975	17	1.99
Boys	1976	17	2.01
Boys	1977	17	2.03
Boys	1978	17	2.06
Boys	1979	17	2.09
Boys	1980	17	2.12
Boys	1981	17	2.14
Boys	1982	17	2.17
Boys	1983	17	2.20
Boys	1984	17	2.22
Boys	1985	17	2.25
Boys	1986	17	2.28
Boys	1987	17	2.31
Boys	1988	17	2.34
Boys	1989	17	2.38
Boys	1990	17	2.41
Boys	1991	17	2.43
Boys	1992	17	2.45
Boys	1993	17	2.47
Boys	1994	17	2.49
Boys	1995	17	2.51
Boys	1996	17	2.54
Boys	1997	17	2.58
Boys	1998	17	2.62
Boys	1999	17	2.67
Boys	2000	17	2.72
Boys	2001	17	2.78
Boys	2002	17	2.84
Boys	2003	17	2.90
Boys	2004	17	2.96
Boys	2005	17	3.01
Boys	2006	17	3.06
Boys	2007	17	3.11
Boys	2008	17	3.15
Boys	2009	17	3.19
Boys	2010	17	3.23
Boys	2011	17	3.27
Boys	2012	17	3.32
Boys	2013	17	3.36
Boys	2014	17	3.42
Boys	2015	17	3.48
Boys	2016	17	3.54
Boys	1975	18	2.38
Boys	1976	18	2.41

Boys	1977	18	2.44
Boys	1978	18	2.47
Boys	1979	18	2.50
Boys	1980	18	2.53
Boys	1981	18	2.57
Boys	1982	18	2.60
Boys	1983	18	2.63
Boys	1984	18	2.66
Boys	1985	18	2.69
Boys	1986	18	2.72
Boys	1987	18	2.76
Boys	1988	18	2.80
Boys	1989	18	2.83
Boys	1990	18	2.87
Boys	1991	18	2.90
Boys	1992	18	2.92
Boys	1993	18	2.94
Boys	1994	18	2.96
Boys	1995	18	2.99
Boys	1996	18	3.03
Boys	1997	18	3.07
Boys	1998	18	3.12
Boys	1999	18	3.17
Boys	2000	18	3.23
Boys	2001	18	3.30
Boys	2002	18	3.37
Boys	2003	18	3.44
Boys	2004	18	3.50
Boys	2005	18	3.57
Boys	2006	18	3.62
Boys	2007	18	3.67
Boys	2008	18	3.72
Boys	2009	18	3.77
Boys	2010	18	3.81
Boys	2011	18	3.86
Boys	2012	18	3.92
Boys	2013	18	3.97
Boys	2014	18	4.03
Boys	2015	18	4.10
Boys	2016	18	4.18
Girls	1975	12	0.60
Girls	1976	12	0.61
Girls	1977	12	0.62
Girls	1978	12	0.63
Girls	1979	12	0.63
Girls	1980	12	0.64
Girls	1981	12	0.65
Girls	1982	12	0.65
Girls	1983	12	0.66
Girls	1984	12	0.67
Girls	1985	12	0.68

Girls	1986	12	0.68
Girls	1987	12	0.69
Girls	1988	12	0.70
Girls	1989	12	0.71
Girls	1990	12	0.72
Girls	1991	12	0.73
Girls	1992	12	0.75
Girls	1993	12	0.76
Girls	1994	12	0.77
Girls	1995	12	0.79
Girls	1996	12	0.80
Girls	1997	12	0.82
Girls	1998	12	0.83
Girls	1999	12	0.85
Girls	2000	12	0.86
Girls	2001	12	0.88
Girls	2002	12	0.89
Girls	2003	12	0.90
Girls	2004	12	0.92
Girls	2005	12	0.93
Girls	2006	12	0.95
Girls	2007	12	0.96
Girls	2008	12	0.98
Girls	2009	12	0.99
Girls	2010	12	1.01
Girls	2011	12	1.03
Girls	2012	12	1.05
Girls	2013	12	1.07
Girls	2014	12	1.10
Girls	2015	12	1.12
Girls	2016	12	1.15
Girls	1975	13	0.85
Girls	1976	13	0.86
Girls	1977	13	0.87
Girls	1978	13	0.88
Girls	1979	13	0.89
Girls	1980	13	0.90
Girls	1981	13	0.91
Girls	1982	13	0.93
Girls	1983	13	0.94
Girls	1984	13	0.95
Girls	1985	13	0.96
Girls	1986	13	0.97
Girls	1987	13	0.98
Girls	1988	13	0.99
Girls	1989	13	1.00
Girls	1990	13	1.02
Girls	1991	13	1.03
Girls	1992	13	1.05
Girls	1993	13	1.07
Girls	1994	13	1.09

Girls	1995	13	1.11
Girls	1996	13	1.13
Girls	1997	13	1.15
Girls	1998	13	1.17
Girls	1999	13	1.19
Girls	2000	13	1.21
Girls	2001	13	1.23
Girls	2002	13	1.25
Girls	2003	13	1.27
Girls	2004	13	1.29
Girls	2005	13	1.31
Girls	2006	13	1.33
Girls	2007	13	1.35
Girls	2008	13	1.37
Girls	2009	13	1.40
Girls	2010	13	1.42
Girls	2011	13	1.45
Girls	2012	13	1.48
Girls	2013	13	1.51
Girls	2014	13	1.54
Girls	2015	13	1.57
Girls	2016	13	1.61
Girls	1975	14	1.14
Girls	1976	14	1.16
Girls	1977	14	1.17
Girls	1978	14	1.18
Girls	1979	14	1.20
Girls	1980	14	1.21
Girls	1981	14	1.22
Girls	1982	14	1.24
Girls	1983	14	1.25
Girls	1984	14	1.26
Girls	1985	14	1.28
Girls	1986	14	1.29
Girls	1987	14	1.30
Girls	1988	14	1.32
Girls	1989	14	1.33
Girls	1990	14	1.35
Girls	1991	14	1.37
Girls	1992	14	1.40
Girls	1993	14	1.42
Girls	1994	14	1.45
Girls	1995	14	1.47
Girls	1996	14	1.50
Girls	1997	14	1.53
Girls	1998	14	1.55
Girls	1999	14	1.58
Girls	2000	14	1.60
Girls	2001	14	1.63
Girls	2002	14	1.65
Girls	2003	14	1.67

Girls	2004	14	1.70
Girls	2005	14	1.72
Girls	2006	14	1.75
Girls	2007	14	1.78
Girls	2008	14	1.81
Girls	2009	14	1.83
Girls	2010	14	1.87
Girls	2011	14	1.90
Girls	2012	14	1.94
Girls	2013	14	1.97
Girls	2014	14	2.01
Girls	2015	14	2.06
Girls	2016	14	2.10
Girls	1975	15	1.44
Girls	1976	15	1.46
Girls	1977	15	1.48
Girls	1978	15	1.50
Girls	1979	15	1.52
Girls	1980	15	1.54
Girls	1981	15	1.56
Girls	1982	15	1.57
Girls	1983	15	1.59
Girls	1984	15	1.61
Girls	1985	15	1.63
Girls	1986	15	1.65
Girls	1987	15	1.67
Girls	1988	15	1.69
Girls	1989	15	1.72
Girls	1990	15	1.74
Girls	1991	15	1.76
Girls	1992	15	1.78
Girls	1993	15	1.80
Girls	1994	15	1.82
Girls	1995	15	1.84
Girls	1996	15	1.86
Girls	1997	15	1.89
Girls	1998	15	1.92
Girls	1999	15	1.95
Girls	2000	15	1.98
Girls	2001	15	2.02
Girls	2002	15	2.05
Girls	2003	15	2.08
Girls	2004	15	2.12
Girls	2005	15	2.15
Girls	2006	15	2.19
Girls	2007	15	2.22
Girls	2008	15	2.25
Girls	2009	15	2.28
Girls	2010	15	2.32
Girls	2011	15	2.36
Girls	2012	15	2.40

Girls	2013	15	2.44
Girls	2014	15	2.48
Girls	2015	15	2.53
Girls	2016	15	2.57
Girls	1975	16	1.69
Girls	1976	16	1.71
Girls	1977	16	1.73
Girls	1978	16	1.75
Girls	1979	16	1.77
Girls	1980	16	1.80
Girls	1981	16	1.82
Girls	1982	16	1.84
Girls	1983	16	1.86
Girls	1984	16	1.88
Girls	1985	16	1.90
Girls	1986	16	1.92
Girls	1987	16	1.94
Girls	1988	16	1.97
Girls	1989	16	1.99
Girls	1990	16	2.02
Girls	1991	16	2.04
Girls	1992	16	2.06
Girls	1993	16	2.09
Girls	1994	16	2.11
Girls	1995	16	2.13
Girls	1996	16	2.16
Girls	1997	16	2.19
Girls	1998	16	2.22
Girls	1999	16	2.26
Girls	2000	16	2.29
Girls	2001	16	2.33
Girls	2002	16	2.37
Girls	2003	16	2.41
Girls	2004	16	2.45
Girls	2005	16	2.48
Girls	2006	16	2.52
Girls	2007	16	2.56
Girls	2008	16	2.59
Girls	2009	16	2.63
Girls	2010	16	2.67
Girls	2011	16	2.71
Girls	2012	16	2.76
Girls	2013	16	2.80
Girls	2014	16	2.85
Girls	2015	16	2.90
Girls	2016	16	2.95
Girls	1975	17	1.88
Girls	1976	17	1.91
Girls	1977	17	1.93
Girls	1978	17	1.95
Girls	1979	17	1.98

Girls	1980	17	2.00
Girls	1981	17	2.03
Girls	1982	17	2.05
Girls	1983	17	2.07
Girls	1984	17	2.09
Girls	1985	17	2.11
Girls	1986	17	2.14
Girls	1987	17	2.17
Girls	1988	17	2.19
Girls	1989	17	2.22
Girls	1990	17	2.25
Girls	1991	17	2.28
Girls	1992	17	2.30
Girls	1993	17	2.32
Girls	1994	17	2.35
Girls	1995	17	2.37
Girls	1996	17	2.41
Girls	1997	17	2.44
Girls	1998	17	2.48
Girls	1999	17	2.52
Girls	2000	17	2.56
Girls	2001	17	2.60
Girls	2002	17	2.64
Girls	2003	17	2.68
Girls	2004	17	2.72
Girls	2005	17	2.77
Girls	2006	17	2.81
Girls	2007	17	2.85
Girls	2008	17	2.89
Girls	2009	17	2.93
Girls	2010	17	2.97
Girls	2011	17	3.02
Girls	2012	17	3.06
Girls	2013	17	3.11
Girls	2014	17	3.17
Girls	2015	17	3.22
Girls	2016	17	3.28
Girls	1975	18	2.06
Girls	1976	18	2.09
Girls	1977	18	2.11
Girls	1978	18	2.14
Girls	1979	18	2.17
Girls	1980	18	2.20
Girls	1981	18	2.22
Girls	1982	18	2.25
Girls	1983	18	2.27
Girls	1984	18	2.30
Girls	1985	18	2.32
Girls	1986	18	2.35
Girls	1987	18	2.38
Girls	1988	18	2.41

Girls	1989	18	2.45
Girls	1990	18	2.48
Girls	1991	18	2.51
Girls	1992	18	2.53
Girls	1993	18	2.56
Girls	1994	18	2.59
Girls	1995	18	2.62
Girls	1996	18	2.65
Girls	1997	18	2.69
Girls	1998	18	2.73
Girls	1999	18	2.78
Girls	2000	18	2.82
Girls	2001	18	2.87
Girls	2002	18	2.92
Girls	2003	18	2.97
Girls	2004	18	3.01
Girls	2005	18	3.06
Girls	2006	18	3.11
Girls	2007	18	3.15
Girls	2008	18	3.20
Girls	2009	18	3.25
Girls	2010	18	3.29
Girls	2011	18	3.34
Girls	2012	18	3.40
Girls	2013	18	3.45
Girls	2014	18	3.51
Girls	2015	18	3.58
Girls	2016	18	3.64

Figure 3A, 3B Annual % change of global prevalence of NAFLD in the boys and girls aged 12-18, 1975 to 2016

region	sex	age	EPAC (%)	lower 95% CI (%)	upper 95% CI (%)
global	Boys	12	1.16	1.12	1.20
global	Boys	13	1.13	1.09	1.16
global	Boys	14	1.05	1.01	1.08
global	Boys	15	0.96	0.92	1.00
global	Boys	16	0.86	0.82	0.89
global	Boys	17	0.78	0.74	0.81
global	Boys	18	0.72	0.69	0.75
global	Girls	12	0.92	0.90	0.94
global	Girls	13	0.86	0.84	0.88
global	Girls	14	0.79	0.78	0.81
global	Girls	15	0.71	0.69	0.73
global	Girls	16	0.68	0.66	0.70
global	Girls	17	0.67	0.65	0.69
global	Girls	18	0.69	0.67	0.71

Figure 3C, 3D Annual % change of global prevalence of NASH
in the boys and girls aged 12-18, 1975 to 2016

region	sex	age	EPAC (%)	lower 95% CI (%)	upper 95% CI (%)
global	Boys	12	1.77	1.71	1.83
global	Boys	13	1.82	1.76	1.88
global	Boys	14	1.83	1.77	1.89
global	Boys	15	1.82	1.76	1.88
global	Boys	16	1.76	1.70	1.82
global	Boys	17	1.69	1.64	1.75
global	Boys	18	1.65	1.59	1.70
global	Girls	12	1.68	1.66	1.71
global	Girls	13	1.66	1.63	1.69
global	Girls	14	1.60	1.57	1.63
global	Girls	15	1.50	1.47	1.53
global	Girls	16	1.45	1.42	1.48
global	Girls	17	1.45	1.42	1.47
global	Girls	18	1.48	1.45	1.51

Figure 3A, 3B Projected global prevalence of NAFLD in the boys and girls aged 12-18, 2017 to 2030

sex	year	age	Predicted prevalence (%)
Boys	2017	12	9.19
Boys	2018	12	9.29
Boys	2019	12	9.39
Boys	2020	12	9.48
Boys	2021	12	9.58
Boys	2022	12	9.67
Boys	2023	12	9.77
Boys	2024	12	9.86
Boys	2025	12	9.96
Boys	2026	12	10.06
Boys	2027	12	10.15
Boys	2028	12	10.25
Boys	2029	12	10.34
Boys	2030	12	10.44
Boys	2017	13	11.23
Boys	2018	13	11.44
Boys	2019	13	11.65
Boys	2020	13	11.87
Boys	2021	13	12.08
Boys	2022	13	12.28
Boys	2023	13	12.47
Boys	2024	13	12.64
Boys	2025	13	12.80
Boys	2026	13	12.94
Boys	2027	13	13.07
Boys	2028	13	13.19
Boys	2029	13	13.30
Boys	2030	13	13.40
Boys	2017	14	13.31
Boys	2018	14	13.55
Boys	2019	14	13.80
Boys	2020	14	14.04
Boys	2021	14	14.28
Boys	2022	14	14.51
Boys	2023	14	14.72
Boys	2024	14	14.92
Boys	2025	14	15.10
Boys	2026	14	15.27
Boys	2027	14	15.41
Boys	2028	14	15.55
Boys	2029	14	15.67
Boys	2030	14	15.78
Boys	2017	15	15.51
Boys	2018	15	15.73
Boys	2019	15	15.95
Boys	2020	15	16.18
Boys	2021	15	16.40
Boys	2022	15	16.62
Boys	2023	15	16.83
Boys	2024	15	17.04
Boys	2025	15	17.24

Boys	2026	15	17.43
Boys	2027	15	17.62
Boys	2028	15	17.81
Boys	2029	15	18.01
Boys	2030	15	18.20
Boys	2017	16	17.60
Boys	2018	16	17.82
Boys	2019	16	18.06
Boys	2020	16	18.29
Boys	2021	16	18.53
Boys	2022	16	18.76
Boys	2023	16	18.98
Boys	2024	16	19.19
Boys	2025	16	19.40
Boys	2026	16	19.60
Boys	2027	16	19.80
Boys	2028	16	20.00
Boys	2029	16	20.21
Boys	2030	16	20.41
Boys	2017	17	19.73
Boys	2018	17	19.96
Boys	2019	17	20.20
Boys	2020	17	20.44
Boys	2021	17	20.68
Boys	2022	17	20.92
Boys	2023	17	21.15
Boys	2024	17	21.37
Boys	2025	17	21.58
Boys	2026	17	21.78
Boys	2027	17	21.99
Boys	2028	17	22.19
Boys	2029	17	22.40
Boys	2030	17	22.61
Boys	2017	18	21.97
Boys	2018	18	22.21
Boys	2019	18	22.46
Boys	2020	18	22.71
Boys	2021	18	22.96
Boys	2022	18	23.21
Boys	2023	18	23.44
Boys	2024	18	23.67
Boys	2025	18	23.89
Boys	2026	18	24.10
Boys	2027	18	24.32
Boys	2028	18	24.53
Boys	2029	18	24.74
Boys	2030	18	24.96
Girls	2017	12	8.24
Girls	2018	12	8.36
Girls	2019	12	8.49
Girls	2020	12	8.63
Girls	2021	12	8.77

Girls	2022	12	8.91
Girls	2023	12	9.06
Girls	2024	12	9.21
Girls	2025	12	9.37
Girls	2026	12	9.52
Girls	2027	12	9.68
Girls	2028	12	9.85
Girls	2029	12	10.01
Girls	2030	12	10.17
Girls	2017	13	10.00
Girls	2018	13	10.14
Girls	2019	13	10.28
Girls	2020	13	10.44
Girls	2021	13	10.60
Girls	2022	13	10.76
Girls	2023	13	10.93
Girls	2024	13	11.10
Girls	2025	13	11.27
Girls	2026	13	11.45
Girls	2027	13	11.63
Girls	2028	13	11.80
Girls	2029	13	11.98
Girls	2030	13	12.16
Girls	2017	14	11.74
Girls	2018	14	11.90
Girls	2019	14	12.06
Girls	2020	14	12.23
Girls	2021	14	12.40
Girls	2022	14	12.58
Girls	2023	14	12.77
Girls	2024	14	12.96
Girls	2025	14	13.15
Girls	2026	14	13.35
Girls	2027	14	13.54
Girls	2028	14	13.74
Girls	2029	14	13.94
Girls	2030	14	14.15
Girls	2017	15	13.29
Girls	2018	15	13.43
Girls	2019	15	13.57
Girls	2020	15	13.70
Girls	2021	15	13.84
Girls	2022	15	13.97
Girls	2023	15	14.10
Girls	2024	15	14.23
Girls	2025	15	14.36
Girls	2026	15	14.48
Girls	2027	15	14.60
Girls	2028	15	14.73
Girls	2029	15	14.86
Girls	2030	15	14.98
Girls	2017	16	14.53

Girls	2018	16	14.67
Girls	2019	16	14.81
Girls	2020	16	14.96
Girls	2021	16	15.10
Girls	2022	16	15.24
Girls	2023	16	15.38
Girls	2024	16	15.51
Girls	2025	16	15.65
Girls	2026	16	15.78
Girls	2027	16	15.91
Girls	2028	16	16.04
Girls	2029	16	16.17
Girls	2030	16	16.30
Girls	2017	17	15.51
Girls	2018	17	15.66
Girls	2019	17	15.80
Girls	2020	17	15.95
Girls	2021	17	16.10
Girls	2022	17	16.25
Girls	2023	17	16.39
Girls	2024	17	16.52
Girls	2025	17	16.66
Girls	2026	17	16.80
Girls	2027	17	16.93
Girls	2028	17	17.07
Girls	2029	17	17.20
Girls	2030	17	17.34
Girls	2017	18	16.40
Girls	2018	18	16.56
Girls	2019	18	16.72
Girls	2020	18	16.88
Girls	2021	18	17.03
Girls	2022	18	17.19
Girls	2023	18	17.34
Girls	2024	18	17.48
Girls	2025	18	17.63
Girls	2026	18	17.77
Girls	2027	18	17.91
Girls	2028	18	18.06
Girls	2029	18	18.20
Girls	2030	18	18.35

Figure 3C, 3D Projected global prevalence of NASH in the boys and girls aged 12-18, 2017 to 2030

sex	year	age	Predicted prevalence (%)
Boys	2017	12	1.07
Boys	2018	12	1.10
Boys	2019	12	1.13
Boys	2020	12	1.15
Boys	2021	12	1.18
Boys	2022	12	1.20
Boys	2023	12	1.23
Boys	2024	12	1.25
Boys	2025	12	1.27
Boys	2026	12	1.28
Boys	2027	12	1.30
Boys	2028	12	1.31
Boys	2029	12	1.32
Boys	2030	12	1.33
Boys	2017	13	1.46
Boys	2018	13	1.50
Boys	2019	13	1.54
Boys	2020	13	1.59
Boys	2021	13	1.63
Boys	2022	13	1.67
Boys	2023	13	1.72
Boys	2024	13	1.76
Boys	2025	13	1.81
Boys	2026	13	1.85
Boys	2027	13	1.90
Boys	2028	13	1.94
Boys	2029	13	1.98
Boys	2030	13	2.03
Boys	2017	14	1.94
Boys	2018	14	1.99
Boys	2019	14	2.05
Boys	2020	14	2.10
Boys	2021	14	2.16
Boys	2022	14	2.22
Boys	2023	14	2.28
Boys	2024	14	2.35
Boys	2025	14	2.41
Boys	2026	14	2.47
Boys	2027	14	2.53
Boys	2028	14	2.59
Boys	2029	14	2.65
Boys	2030	14	2.71
Boys	2017	15	2.49
Boys	2018	15	2.54
Boys	2019	15	2.60
Boys	2020	15	2.66
Boys	2021	15	2.72
Boys	2022	15	2.77
Boys	2023	15	2.83
Boys	2024	15	2.88
Boys	2025	15	2.93

Boys	2026	15	2.98
Boys	2027	15	3.02
Boys	2028	15	3.07
Boys	2029	15	3.11
Boys	2030	15	3.16
Boys	2017	16	3.04
Boys	2018	16	3.10
Boys	2019	16	3.17
Boys	2020	16	3.24
Boys	2021	16	3.31
Boys	2022	16	3.38
Boys	2023	16	3.45
Boys	2024	16	3.51
Boys	2025	16	3.57
Boys	2026	16	3.63
Boys	2027	16	3.68
Boys	2028	16	3.74
Boys	2029	16	3.79
Boys	2030	16	3.85
Boys	2017	17	3.61
Boys	2018	17	3.69
Boys	2019	17	3.77
Boys	2020	17	3.86
Boys	2021	17	3.94
Boys	2022	17	4.02
Boys	2023	17	4.10
Boys	2024	17	4.17
Boys	2025	17	4.24
Boys	2026	17	4.30
Boys	2027	17	4.37
Boys	2028	17	4.43
Boys	2029	17	4.50
Boys	2030	17	4.56
Boys	2017	18	4.26
Boys	2018	18	4.35
Boys	2019	18	4.44
Boys	2020	18	4.54
Boys	2021	18	4.64
Boys	2022	18	4.73
Boys	2023	18	4.83
Boys	2024	18	4.92
Boys	2025	18	5.00
Boys	2026	18	5.09
Boys	2027	18	5.17
Boys	2028	18	5.25
Boys	2029	18	5.34
Boys	2030	18	5.42
Girls	2017	12	1.18
Girls	2018	12	1.21
Girls	2019	12	1.24
Girls	2020	12	1.27
Girls	2021	12	1.31

Girls	2022	12	1.34
Girls	2023	12	1.38
Girls	2024	12	1.42
Girls	2025	12	1.46
Girls	2026	12	1.50
Girls	2027	12	1.54
Girls	2028	12	1.58
Girls	2029	12	1.63
Girls	2030	12	1.67
Girls	2017	13	1.65
Girls	2018	13	1.69
Girls	2019	13	1.73
Girls	2020	13	1.78
Girls	2021	13	1.83
Girls	2022	13	1.88
Girls	2023	13	1.93
Girls	2024	13	1.98
Girls	2025	13	2.04
Girls	2026	13	2.09
Girls	2027	13	2.15
Girls	2028	13	2.21
Girls	2029	13	2.27
Girls	2030	13	2.33
Girls	2017	14	2.15
Girls	2018	14	2.21
Girls	2019	14	2.26
Girls	2020	14	2.32
Girls	2021	14	2.38
Girls	2022	14	2.45
Girls	2023	14	2.52
Girls	2024	14	2.59
Girls	2025	14	2.66
Girls	2026	14	2.73
Girls	2027	14	2.81
Girls	2028	14	2.89
Girls	2029	14	2.97
Girls	2030	14	3.06
Girls	2017	15	2.63
Girls	2018	15	2.68
Girls	2019	15	2.73
Girls	2020	15	2.79
Girls	2021	15	2.85
Girls	2022	15	2.90
Girls	2023	15	2.96
Girls	2024	15	3.01
Girls	2025	15	3.06
Girls	2026	15	3.12
Girls	2027	15	3.17
Girls	2028	15	3.22
Girls	2029	15	3.27
Girls	2030	15	3.32
Girls	2017	16	3.01

Girls	2018	16	3.07
Girls	2019	16	3.13
Girls	2020	16	3.20
Girls	2021	16	3.26
Girls	2022	16	3.32
Girls	2023	16	3.38
Girls	2024	16	3.45
Girls	2025	16	3.51
Girls	2026	16	3.57
Girls	2027	16	3.62
Girls	2028	16	3.68
Girls	2029	16	3.74
Girls	2030	16	3.80
Girls	2017	17	3.35
Girls	2018	17	3.41
Girls	2019	17	3.48
Girls	2020	17	3.55
Girls	2021	17	3.61
Girls	2022	17	3.68
Girls	2023	17	3.75
Girls	2024	17	3.81
Girls	2025	17	3.88
Girls	2026	17	3.94
Girls	2027	17	4.01
Girls	2028	17	4.07
Girls	2029	17	4.13
Girls	2030	17	4.19
Girls	2017	18	3.71
Girls	2018	18	3.78
Girls	2019	18	3.86
Girls	2020	18	3.93
Girls	2021	18	4.00
Girls	2022	18	4.08
Girls	2023	18	4.15
Girls	2024	18	4.22
Girls	2025	18	4.29
Girls	2026	18	4.36
Girls	2027	18	4.43
Girls	2028	18	4.49
Girls	2029	18	4.56
Girls	2030	18	4.63

region	sex	year	age	Prevalence (%)
Central and Eastern Europe	Boys	1975	12	6.41
Central and Eastern Europe	Boys	1976	12	6.48
Central and Eastern Europe	Boys	1977	12	6.56
Central and Eastern Europe	Boys	1978	12	6.63
Central and Eastern Europe	Boys	1979	12	6.71
Central and Eastern Europe	Boys	1980	12	6.79
Central and Eastern Europe	Boys	1981	12	6.88
Central and Eastern Europe	Boys	1982	12	6.98
Central and Eastern Europe	Boys	1983	12	7.08
Central and Eastern Europe	Boys	1984	12	7.18
Central and Eastern Europe	Boys	1985	12	7.28
Central and Eastern Europe	Boys	1986	12	7.38
Central and Eastern Europe	Boys	1987	12	7.48
Central and Eastern Europe	Boys	1988	12	7.57
Central and Eastern Europe	Boys	1989	12	7.67
Central and Eastern Europe	Boys	1990	12	7.77
Central and Eastern Europe	Boys	1991	12	7.87
Central and Eastern Europe	Boys	1992	12	7.97
Central and Eastern Europe	Boys	1993	12	8.07
Central and Eastern Europe	Boys	1994	12	8.16
Central and Eastern Europe	Boys	1995	12	8.26
Central and Eastern Europe	Boys	1996	12	8.35
Central and Eastern Europe	Boys	1997	12	8.45
Central and Eastern Europe	Boys	1998	12	8.55
Central and Eastern Europe	Boys	1999	12	8.65
Central and Eastern Europe	Boys	2000	12	8.76
Central and Eastern Europe	Boys	2001	12	8.89
Central and Eastern Europe	Boys	2002	12	9.02
Central and Eastern Europe	Boys	2003	12	9.16
Central and Eastern Europe	Boys	2004	12	9.31
Central and Eastern Europe	Boys	2005	12	9.47
Central and Eastern Europe	Boys	2006	12	9.63
Central and Eastern Europe	Boys	2007	12	9.79
Central and Eastern Europe	Boys	2008	12	9.96
Central and Eastern Europe	Boys	2009	12	10.13
Central and Eastern Europe	Boys	2010	12	10.31
Central and Eastern Europe	Boys	2011	12	10.49
Central and Eastern Europe	Boys	2012	12	10.67
Central and Eastern Europe	Boys	2013	12	10.85
Central and Eastern Europe	Boys	2014	12	11.04
Central and Eastern Europe	Boys	2015	12	11.23
Central and Eastern Europe	Boys	2016	12	11.42
Central and Eastern Europe	Boys	1975	13	7.92
Central and Eastern Europe	Boys	1976	13	8.01
Central and Eastern Europe	Boys	1977	13	8.09
Central and Eastern Europe	Boys	1978	13	8.17
Central and Eastern Europe	Boys	1979	13	8.26
Central and Eastern Europe	Boys	1980	13	8.36
Central and Eastern Europe	Boys	1981	13	8.47
Central and Eastern Europe	Boys	1982	13	8.58
Central and Eastern Europe	Boys	1983	13	8.70

Central and Eastern Europe	Boys	1984	13	8.81
Central and Eastern Europe	Boys	1985	13	8.93
Central and Eastern Europe	Boys	1986	13	9.04
Central and Eastern Europe	Boys	1987	13	9.16
Central and Eastern Europe	Boys	1988	13	9.27
Central and Eastern Europe	Boys	1989	13	9.38
Central and Eastern Europe	Boys	1990	13	9.50
Central and Eastern Europe	Boys	1991	13	9.62
Central and Eastern Europe	Boys	1992	13	9.73
Central and Eastern Europe	Boys	1993	13	9.84
Central and Eastern Europe	Boys	1994	13	9.94
Central and Eastern Europe	Boys	1995	13	10.05
Central and Eastern Europe	Boys	1996	13	10.16
Central and Eastern Europe	Boys	1997	13	10.27
Central and Eastern Europe	Boys	1998	13	10.38
Central and Eastern Europe	Boys	1999	13	10.50
Central and Eastern Europe	Boys	2000	13	10.63
Central and Eastern Europe	Boys	2001	13	10.77
Central and Eastern Europe	Boys	2002	13	10.93
Central and Eastern Europe	Boys	2003	13	11.09
Central and Eastern Europe	Boys	2004	13	11.27
Central and Eastern Europe	Boys	2005	13	11.45
Central and Eastern Europe	Boys	2006	13	11.64
Central and Eastern Europe	Boys	2007	13	11.84
Central and Eastern Europe	Boys	2008	13	12.04
Central and Eastern Europe	Boys	2009	13	12.24
Central and Eastern Europe	Boys	2010	13	12.45
Central and Eastern Europe	Boys	2011	13	12.66
Central and Eastern Europe	Boys	2012	13	12.88
Central and Eastern Europe	Boys	2013	13	13.10
Central and Eastern Europe	Boys	2014	13	13.32
Central and Eastern Europe	Boys	2015	13	13.55
Central and Eastern Europe	Boys	2016	13	13.78
Central and Eastern Europe	Boys	1975	14	9.76
Central and Eastern Europe	Boys	1976	14	9.85
Central and Eastern Europe	Boys	1977	14	9.94
Central and Eastern Europe	Boys	1978	14	10.04
Central and Eastern Europe	Boys	1979	14	10.14
Central and Eastern Europe	Boys	1980	14	10.25
Central and Eastern Europe	Boys	1981	14	10.37
Central and Eastern Europe	Boys	1982	14	10.49
Central and Eastern Europe	Boys	1983	14	10.62
Central and Eastern Europe	Boys	1984	14	10.74
Central and Eastern Europe	Boys	1985	14	10.87
Central and Eastern Europe	Boys	1986	14	11.00
Central and Eastern Europe	Boys	1987	14	11.12
Central and Eastern Europe	Boys	1988	14	11.24
Central and Eastern Europe	Boys	1989	14	11.37
Central and Eastern Europe	Boys	1990	14	11.50
Central and Eastern Europe	Boys	1991	14	11.62
Central and Eastern Europe	Boys	1992	14	11.74
Central and Eastern Europe	Boys	1993	14	11.86

Central and Eastern Europe	Boys	1994	14	11.98
Central and Eastern Europe	Boys	1995	14	12.09
Central and Eastern Europe	Boys	1996	14	12.21
Central and Eastern Europe	Boys	1997	14	12.33
Central and Eastern Europe	Boys	1998	14	12.45
Central and Eastern Europe	Boys	1999	14	12.58
Central and Eastern Europe	Boys	2000	14	12.72
Central and Eastern Europe	Boys	2001	14	12.88
Central and Eastern Europe	Boys	2002	14	13.04
Central and Eastern Europe	Boys	2003	14	13.23
Central and Eastern Europe	Boys	2004	14	13.42
Central and Eastern Europe	Boys	2005	14	13.63
Central and Eastern Europe	Boys	2006	14	13.84
Central and Eastern Europe	Boys	2007	14	14.05
Central and Eastern Europe	Boys	2008	14	14.27
Central and Eastern Europe	Boys	2009	14	14.50
Central and Eastern Europe	Boys	2010	14	14.73
Central and Eastern Europe	Boys	2011	14	14.97
Central and Eastern Europe	Boys	2012	14	15.21
Central and Eastern Europe	Boys	2013	14	15.46
Central and Eastern Europe	Boys	2014	14	15.71
Central and Eastern Europe	Boys	2015	14	15.97
Central and Eastern Europe	Boys	2016	14	16.24
Central and Eastern Europe	Boys	1975	15	11.82
Central and Eastern Europe	Boys	1976	15	11.93
Central and Eastern Europe	Boys	1977	15	12.04
Central and Eastern Europe	Boys	1978	15	12.16
Central and Eastern Europe	Boys	1979	15	12.28
Central and Eastern Europe	Boys	1980	15	12.40
Central and Eastern Europe	Boys	1981	15	12.51
Central and Eastern Europe	Boys	1982	15	12.62
Central and Eastern Europe	Boys	1983	15	12.73
Central and Eastern Europe	Boys	1984	15	12.85
Central and Eastern Europe	Boys	1985	15	12.97
Central and Eastern Europe	Boys	1986	15	13.10
Central and Eastern Europe	Boys	1987	15	13.24
Central and Eastern Europe	Boys	1988	15	13.38
Central and Eastern Europe	Boys	1989	15	13.52
Central and Eastern Europe	Boys	1990	15	13.66
Central and Eastern Europe	Boys	1991	15	13.79
Central and Eastern Europe	Boys	1992	15	13.92
Central and Eastern Europe	Boys	1993	15	14.05
Central and Eastern Europe	Boys	1994	15	14.18
Central and Eastern Europe	Boys	1995	15	14.31
Central and Eastern Europe	Boys	1996	15	14.43
Central and Eastern Europe	Boys	1997	15	14.56
Central and Eastern Europe	Boys	1998	15	14.68
Central and Eastern Europe	Boys	1999	15	14.81
Central and Eastern Europe	Boys	2000	15	14.95
Central and Eastern Europe	Boys	2001	15	15.09
Central and Eastern Europe	Boys	2002	15	15.25
Central and Eastern Europe	Boys	2003	15	15.41

Central and Eastern Europe	Boys	2004	15	15.59
Central and Eastern Europe	Boys	2005	15	15.78
Central and Eastern Europe	Boys	2006	15	15.99
Central and Eastern Europe	Boys	2007	15	16.21
Central and Eastern Europe	Boys	2008	15	16.45
Central and Eastern Europe	Boys	2009	15	16.71
Central and Eastern Europe	Boys	2010	15	16.97
Central and Eastern Europe	Boys	2011	15	17.25
Central and Eastern Europe	Boys	2012	15	17.53
Central and Eastern Europe	Boys	2013	15	17.82
Central and Eastern Europe	Boys	2014	15	18.12
Central and Eastern Europe	Boys	2015	15	18.42
Central and Eastern Europe	Boys	2016	15	18.72
Central and Eastern Europe	Boys	1975	16	14.05
Central and Eastern Europe	Boys	1976	16	14.16
Central and Eastern Europe	Boys	1977	16	14.27
Central and Eastern Europe	Boys	1978	16	14.40
Central and Eastern Europe	Boys	1979	16	14.52
Central and Eastern Europe	Boys	1980	16	14.64
Central and Eastern Europe	Boys	1981	16	14.76
Central and Eastern Europe	Boys	1982	16	14.87
Central and Eastern Europe	Boys	1983	16	14.99
Central and Eastern Europe	Boys	1984	16	15.11
Central and Eastern Europe	Boys	1985	16	15.23
Central and Eastern Europe	Boys	1986	16	15.36
Central and Eastern Europe	Boys	1987	16	15.50
Central and Eastern Europe	Boys	1988	16	15.64
Central and Eastern Europe	Boys	1989	16	15.79
Central and Eastern Europe	Boys	1990	16	15.93
Central and Eastern Europe	Boys	1991	16	16.06
Central and Eastern Europe	Boys	1992	16	16.20
Central and Eastern Europe	Boys	1993	16	16.33
Central and Eastern Europe	Boys	1994	16	16.45
Central and Eastern Europe	Boys	1995	16	16.58
Central and Eastern Europe	Boys	1996	16	16.70
Central and Eastern Europe	Boys	1997	16	16.83
Central and Eastern Europe	Boys	1998	16	16.96
Central and Eastern Europe	Boys	1999	16	17.09
Central and Eastern Europe	Boys	2000	16	17.22
Central and Eastern Europe	Boys	2001	16	17.37
Central and Eastern Europe	Boys	2002	16	17.52
Central and Eastern Europe	Boys	2003	16	17.69
Central and Eastern Europe	Boys	2004	16	17.87
Central and Eastern Europe	Boys	2005	16	18.06
Central and Eastern Europe	Boys	2006	16	18.27
Central and Eastern Europe	Boys	2007	16	18.50
Central and Eastern Europe	Boys	2008	16	18.74
Central and Eastern Europe	Boys	2009	16	19.00
Central and Eastern Europe	Boys	2010	16	19.27
Central and Eastern Europe	Boys	2011	16	19.56
Central and Eastern Europe	Boys	2012	16	19.85
Central and Eastern Europe	Boys	2013	16	20.15

Central and Eastern Europe	Boys	2014	16	20.45
Central and Eastern Europe	Boys	2015	16	20.77
Central and Eastern Europe	Boys	2016	16	21.08
Central and Eastern Europe	Boys	1975	17	16.34
Central and Eastern Europe	Boys	1976	17	16.46
Central and Eastern Europe	Boys	1977	17	16.58
Central and Eastern Europe	Boys	1978	17	16.70
Central and Eastern Europe	Boys	1979	17	16.83
Central and Eastern Europe	Boys	1980	17	16.95
Central and Eastern Europe	Boys	1981	17	17.07
Central and Eastern Europe	Boys	1982	17	17.19
Central and Eastern Europe	Boys	1983	17	17.30
Central and Eastern Europe	Boys	1984	17	17.42
Central and Eastern Europe	Boys	1985	17	17.55
Central and Eastern Europe	Boys	1986	17	17.69
Central and Eastern Europe	Boys	1987	17	17.83
Central and Eastern Europe	Boys	1988	17	17.97
Central and Eastern Europe	Boys	1989	17	18.11
Central and Eastern Europe	Boys	1990	17	18.25
Central and Eastern Europe	Boys	1991	17	18.39
Central and Eastern Europe	Boys	1992	17	18.52
Central and Eastern Europe	Boys	1993	17	18.65
Central and Eastern Europe	Boys	1994	17	18.78
Central and Eastern Europe	Boys	1995	17	18.91
Central and Eastern Europe	Boys	1996	17	19.03
Central and Eastern Europe	Boys	1997	17	19.16
Central and Eastern Europe	Boys	1998	17	19.28
Central and Eastern Europe	Boys	1999	17	19.41
Central and Eastern Europe	Boys	2000	17	19.55
Central and Eastern Europe	Boys	2001	17	19.69
Central and Eastern Europe	Boys	2002	17	19.85
Central and Eastern Europe	Boys	2003	17	20.01
Central and Eastern Europe	Boys	2004	17	20.19
Central and Eastern Europe	Boys	2005	17	20.39
Central and Eastern Europe	Boys	2006	17	20.60
Central and Eastern Europe	Boys	2007	17	20.83
Central and Eastern Europe	Boys	2008	17	21.08
Central and Eastern Europe	Boys	2009	17	21.34
Central and Eastern Europe	Boys	2010	17	21.61
Central and Eastern Europe	Boys	2011	17	21.90
Central and Eastern Europe	Boys	2012	17	22.19
Central and Eastern Europe	Boys	2013	17	22.50
Central and Eastern Europe	Boys	2014	17	22.81
Central and Eastern Europe	Boys	2015	17	23.13
Central and Eastern Europe	Boys	2016	17	23.44
Central and Eastern Europe	Boys	1975	18	18.65
Central and Eastern Europe	Boys	1976	18	18.77
Central and Eastern Europe	Boys	1977	18	18.89
Central and Eastern Europe	Boys	1978	18	19.02
Central and Eastern Europe	Boys	1979	18	19.15
Central and Eastern Europe	Boys	1980	18	19.28
Central and Eastern Europe	Boys	1981	18	19.41

Central and Eastern Europe	Boys	1982	18	19.53
Central and Eastern Europe	Boys	1983	18	19.65
Central and Eastern Europe	Boys	1984	18	19.77
Central and Eastern Europe	Boys	1985	18	19.90
Central and Eastern Europe	Boys	1986	18	20.04
Central and Eastern Europe	Boys	1987	18	20.18
Central and Eastern Europe	Boys	1988	18	20.33
Central and Eastern Europe	Boys	1989	18	20.48
Central and Eastern Europe	Boys	1990	18	20.62
Central and Eastern Europe	Boys	1991	18	20.76
Central and Eastern Europe	Boys	1992	18	20.90
Central and Eastern Europe	Boys	1993	18	21.03
Central and Eastern Europe	Boys	1994	18	21.16
Central and Eastern Europe	Boys	1995	18	21.29
Central and Eastern Europe	Boys	1996	18	21.42
Central and Eastern Europe	Boys	1997	18	21.54
Central and Eastern Europe	Boys	1998	18	21.67
Central and Eastern Europe	Boys	1999	18	21.80
Central and Eastern Europe	Boys	2000	18	21.94
Central and Eastern Europe	Boys	2001	18	22.09
Central and Eastern Europe	Boys	2002	18	22.25
Central and Eastern Europe	Boys	2003	18	22.42
Central and Eastern Europe	Boys	2004	18	22.60
Central and Eastern Europe	Boys	2005	18	22.80
Central and Eastern Europe	Boys	2006	18	23.01
Central and Eastern Europe	Boys	2007	18	23.25
Central and Eastern Europe	Boys	2008	18	23.50
Central and Eastern Europe	Boys	2009	18	23.77
Central and Eastern Europe	Boys	2010	18	24.05
Central and Eastern Europe	Boys	2011	18	24.34
Central and Eastern Europe	Boys	2012	18	24.64
Central and Eastern Europe	Boys	2013	18	24.95
Central and Eastern Europe	Boys	2014	18	25.27
Central and Eastern Europe	Boys	2015	18	25.60
Central and Eastern Europe	Boys	2016	18	25.92
Central and Eastern Europe	Boys	1975	Average	12.14
Central and Eastern Europe	Boys	1976	Average	12.24
Central and Eastern Europe	Boys	1977	Average	12.34
Central and Eastern Europe	Boys	1978	Average	12.44
Central and Eastern Europe	Boys	1979	Average	12.55
Central and Eastern Europe	Boys	1980	Average	12.67
Central and Eastern Europe	Boys	1981	Average	12.78
Central and Eastern Europe	Boys	1982	Average	12.89
Central and Eastern Europe	Boys	1983	Average	13.01
Central and Eastern Europe	Boys	1984	Average	13.13
Central and Eastern Europe	Boys	1985	Average	13.25
Central and Eastern Europe	Boys	1986	Average	13.37
Central and Eastern Europe	Boys	1987	Average	13.50
Central and Eastern Europe	Boys	1988	Average	13.63
Central and Eastern Europe	Boys	1989	Average	13.76
Central and Eastern Europe	Boys	1990	Average	13.89
Central and Eastern Europe	Boys	1991	Average	14.02

Central and Eastern Europe	Boys	1992	Average	14.14
Central and Eastern Europe	Boys	1993	Average	14.26
Central and Eastern Europe	Boys	1994	Average	14.38
Central and Eastern Europe	Boys	1995	Average	14.50
Central and Eastern Europe	Boys	1996	Average	14.61
Central and Eastern Europe	Boys	1997	Average	14.73
Central and Eastern Europe	Boys	1998	Average	14.85
Central and Eastern Europe	Boys	1999	Average	14.98
Central and Eastern Europe	Boys	2000	Average	15.11
Central and Eastern Europe	Boys	2001	Average	15.25
Central and Eastern Europe	Boys	2002	Average	15.41
Central and Eastern Europe	Boys	2003	Average	15.57
Central and Eastern Europe	Boys	2004	Average	15.75
Central and Eastern Europe	Boys	2005	Average	15.94
Central and Eastern Europe	Boys	2006	Average	16.14
Central and Eastern Europe	Boys	2007	Average	16.35
Central and Eastern Europe	Boys	2008	Average	16.58
Central and Eastern Europe	Boys	2009	Average	16.81
Central and Eastern Europe	Boys	2010	Average	17.06
Central and Eastern Europe	Boys	2011	Average	17.31
Central and Eastern Europe	Boys	2012	Average	17.57
Central and Eastern Europe	Boys	2013	Average	17.83
Central and Eastern Europe	Boys	2014	Average	18.10
Central and Eastern Europe	Boys	2015	Average	18.38
Central and Eastern Europe	Boys	2016	Average	18.66
Central and Eastern Europe	Boys	2017	Average	18.94
Central and Eastern Europe	Boys	2018	Average	19.22
Central and Eastern Europe	Boys	2019	Average	19.51
Central and Eastern Europe	Boys	2020	Average	19.79
Central and Eastern Europe	Boys	2021	Average	20.07
Central and Eastern Europe	Boys	2022	Average	20.35
Central and Eastern Europe	Boys	2023	Average	20.63
Central and Eastern Europe	Boys	2024	Average	20.91
Central and Eastern Europe	Boys	2025	Average	21.19
Central and Eastern Europe	Boys	2026	Average	21.47
Central and Eastern Europe	Boys	2027	Average	21.74
Central and Eastern Europe	Boys	2028	Average	22.02
Central and Eastern Europe	Boys	2029	Average	22.30
Central and Eastern Europe	Boys	2030	Average	22.57
Central and Eastern Europe	Girls	1975	12	5.94
Central and Eastern Europe	Girls	1976	12	5.99
Central and Eastern Europe	Girls	1977	12	6.04
Central and Eastern Europe	Girls	1978	12	6.09
Central and Eastern Europe	Girls	1979	12	6.14
Central and Eastern Europe	Girls	1980	12	6.20
Central and Eastern Europe	Girls	1981	12	6.26
Central and Eastern Europe	Girls	1982	12	6.32
Central and Eastern Europe	Girls	1983	12	6.39
Central and Eastern Europe	Girls	1984	12	6.45
Central and Eastern Europe	Girls	1985	12	6.51
Central and Eastern Europe	Girls	1986	12	6.57
Central and Eastern Europe	Girls	1987	12	6.63

Central and Eastern Europe	Girls	1988	12	6.69
Central and Eastern Europe	Girls	1989	12	6.75
Central and Eastern Europe	Girls	1990	12	6.81
Central and Eastern Europe	Girls	1991	12	6.86
Central and Eastern Europe	Girls	1992	12	6.92
Central and Eastern Europe	Girls	1993	12	6.97
Central and Eastern Europe	Girls	1994	12	7.03
Central and Eastern Europe	Girls	1995	12	7.08
Central and Eastern Europe	Girls	1996	12	7.13
Central and Eastern Europe	Girls	1997	12	7.18
Central and Eastern Europe	Girls	1998	12	7.23
Central and Eastern Europe	Girls	1999	12	7.29
Central and Eastern Europe	Girls	2000	12	7.35
Central and Eastern Europe	Girls	2001	12	7.41
Central and Eastern Europe	Girls	2002	12	7.48
Central and Eastern Europe	Girls	2003	12	7.55
Central and Eastern Europe	Girls	2004	12	7.63
Central and Eastern Europe	Girls	2005	12	7.71
Central and Eastern Europe	Girls	2006	12	7.80
Central and Eastern Europe	Girls	2007	12	7.88
Central and Eastern Europe	Girls	2008	12	7.97
Central and Eastern Europe	Girls	2009	12	8.06
Central and Eastern Europe	Girls	2010	12	8.15
Central and Eastern Europe	Girls	2011	12	8.24
Central and Eastern Europe	Girls	2012	12	8.34
Central and Eastern Europe	Girls	2013	12	8.43
Central and Eastern Europe	Girls	2014	12	8.53
Central and Eastern Europe	Girls	2015	12	8.63
Central and Eastern Europe	Girls	2016	12	8.73
Central and Eastern Europe	Girls	1975	13	7.36
Central and Eastern Europe	Girls	1976	13	7.42
Central and Eastern Europe	Girls	1977	13	7.48
Central and Eastern Europe	Girls	1978	13	7.53
Central and Eastern Europe	Girls	1979	13	7.59
Central and Eastern Europe	Girls	1980	13	7.66
Central and Eastern Europe	Girls	1981	13	7.73
Central and Eastern Europe	Girls	1982	13	7.80
Central and Eastern Europe	Girls	1983	13	7.87
Central and Eastern Europe	Girls	1984	13	7.95
Central and Eastern Europe	Girls	1985	13	8.02
Central and Eastern Europe	Girls	1986	13	8.09
Central and Eastern Europe	Girls	1987	13	8.15
Central and Eastern Europe	Girls	1988	13	8.22
Central and Eastern Europe	Girls	1989	13	8.28
Central and Eastern Europe	Girls	1990	13	8.35
Central and Eastern Europe	Girls	1991	13	8.41
Central and Eastern Europe	Girls	1992	13	8.48
Central and Eastern Europe	Girls	1993	13	8.54
Central and Eastern Europe	Girls	1994	13	8.60
Central and Eastern Europe	Girls	1995	13	8.65
Central and Eastern Europe	Girls	1996	13	8.71
Central and Eastern Europe	Girls	1997	13	8.77

Central and Eastern Europe	Girls	1998	13	8.83
Central and Eastern Europe	Girls	1999	13	8.89
Central and Eastern Europe	Girls	2000	13	8.96
Central and Eastern Europe	Girls	2001	13	9.03
Central and Eastern Europe	Girls	2002	13	9.11
Central and Eastern Europe	Girls	2003	13	9.19
Central and Eastern Europe	Girls	2004	13	9.28
Central and Eastern Europe	Girls	2005	13	9.37
Central and Eastern Europe	Girls	2006	13	9.46
Central and Eastern Europe	Girls	2007	13	9.56
Central and Eastern Europe	Girls	2008	13	9.66
Central and Eastern Europe	Girls	2009	13	9.76
Central and Eastern Europe	Girls	2010	13	9.86
Central and Eastern Europe	Girls	2011	13	9.97
Central and Eastern Europe	Girls	2012	13	10.07
Central and Eastern Europe	Girls	2013	13	10.18
Central and Eastern Europe	Girls	2014	13	10.29
Central and Eastern Europe	Girls	2015	13	10.41
Central and Eastern Europe	Girls	2016	13	10.52
Central and Eastern Europe	Girls	1975	14	8.88
Central and Eastern Europe	Girls	1976	14	8.95
Central and Eastern Europe	Girls	1977	14	9.01
Central and Eastern Europe	Girls	1978	14	9.07
Central and Eastern Europe	Girls	1979	14	9.14
Central and Eastern Europe	Girls	1980	14	9.21
Central and Eastern Europe	Girls	1981	14	9.28
Central and Eastern Europe	Girls	1982	14	9.36
Central and Eastern Europe	Girls	1983	14	9.44
Central and Eastern Europe	Girls	1984	14	9.52
Central and Eastern Europe	Girls	1985	14	9.60
Central and Eastern Europe	Girls	1986	14	9.67
Central and Eastern Europe	Girls	1987	14	9.74
Central and Eastern Europe	Girls	1988	14	9.81
Central and Eastern Europe	Girls	1989	14	9.88
Central and Eastern Europe	Girls	1990	14	9.95
Central and Eastern Europe	Girls	1991	14	10.02
Central and Eastern Europe	Girls	1992	14	10.09
Central and Eastern Europe	Girls	1993	14	10.15
Central and Eastern Europe	Girls	1994	14	10.22
Central and Eastern Europe	Girls	1995	14	10.28
Central and Eastern Europe	Girls	1996	14	10.34
Central and Eastern Europe	Girls	1997	14	10.40
Central and Eastern Europe	Girls	1998	14	10.46
Central and Eastern Europe	Girls	1999	14	10.53
Central and Eastern Europe	Girls	2000	14	10.60
Central and Eastern Europe	Girls	2001	14	10.68
Central and Eastern Europe	Girls	2002	14	10.76
Central and Eastern Europe	Girls	2003	14	10.85
Central and Eastern Europe	Girls	2004	14	10.94
Central and Eastern Europe	Girls	2005	14	11.04
Central and Eastern Europe	Girls	2006	14	11.14
Central and Eastern Europe	Girls	2007	14	11.24

Central and Eastern Europe	Girls	2008	14	11.35
Central and Eastern Europe	Girls	2009	14	11.46
Central and Eastern Europe	Girls	2010	14	11.57
Central and Eastern Europe	Girls	2011	14	11.68
Central and Eastern Europe	Girls	2012	14	11.80
Central and Eastern Europe	Girls	2013	14	11.91
Central and Eastern Europe	Girls	2014	14	12.03
Central and Eastern Europe	Girls	2015	14	12.16
Central and Eastern Europe	Girls	2016	14	12.28
Central and Eastern Europe	Girls	1975	15	10.28
Central and Eastern Europe	Girls	1976	15	10.36
Central and Eastern Europe	Girls	1977	15	10.44
Central and Eastern Europe	Girls	1978	15	10.52
Central and Eastern Europe	Girls	1979	15	10.59
Central and Eastern Europe	Girls	1980	15	10.67
Central and Eastern Europe	Girls	1981	15	10.74
Central and Eastern Europe	Girls	1982	15	10.81
Central and Eastern Europe	Girls	1983	15	10.88
Central and Eastern Europe	Girls	1984	15	10.95
Central and Eastern Europe	Girls	1985	15	11.02
Central and Eastern Europe	Girls	1986	15	11.09
Central and Eastern Europe	Girls	1987	15	11.17
Central and Eastern Europe	Girls	1988	15	11.25
Central and Eastern Europe	Girls	1989	15	11.33
Central and Eastern Europe	Girls	1990	15	11.41
Central and Eastern Europe	Girls	1991	15	11.48
Central and Eastern Europe	Girls	1992	15	11.55
Central and Eastern Europe	Girls	1993	15	11.62
Central and Eastern Europe	Girls	1994	15	11.68
Central and Eastern Europe	Girls	1995	15	11.75
Central and Eastern Europe	Girls	1996	15	11.81
Central and Eastern Europe	Girls	1997	15	11.87
Central and Eastern Europe	Girls	1998	15	11.94
Central and Eastern Europe	Girls	1999	15	12.01
Central and Eastern Europe	Girls	2000	15	12.08
Central and Eastern Europe	Girls	2001	15	12.15
Central and Eastern Europe	Girls	2002	15	12.23
Central and Eastern Europe	Girls	2003	15	12.31
Central and Eastern Europe	Girls	2004	15	12.40
Central and Eastern Europe	Girls	2005	15	12.50
Central and Eastern Europe	Girls	2006	15	12.60
Central and Eastern Europe	Girls	2007	15	12.70
Central and Eastern Europe	Girls	2008	15	12.81
Central and Eastern Europe	Girls	2009	15	12.93
Central and Eastern Europe	Girls	2010	15	13.05
Central and Eastern Europe	Girls	2011	15	13.17
Central and Eastern Europe	Girls	2012	15	13.30
Central and Eastern Europe	Girls	2013	15	13.43
Central and Eastern Europe	Girls	2014	15	13.56
Central and Eastern Europe	Girls	2015	15	13.70
Central and Eastern Europe	Girls	2016	15	13.84
Central and Eastern Europe	Girls	1975	16	11.41

Central and Eastern Europe	Girls	1976	16	11.49
Central and Eastern Europe	Girls	1977	16	11.57
Central and Eastern Europe	Girls	1978	16	11.65
Central and Eastern Europe	Girls	1979	16	11.73
Central and Eastern Europe	Girls	1980	16	11.81
Central and Eastern Europe	Girls	1981	16	11.88
Central and Eastern Europe	Girls	1982	16	11.95
Central and Eastern Europe	Girls	1983	16	12.02
Central and Eastern Europe	Girls	1984	16	12.09
Central and Eastern Europe	Girls	1985	16	12.16
Central and Eastern Europe	Girls	1986	16	12.24
Central and Eastern Europe	Girls	1987	16	12.32
Central and Eastern Europe	Girls	1988	16	12.40
Central and Eastern Europe	Girls	1989	16	12.48
Central and Eastern Europe	Girls	1990	16	12.56
Central and Eastern Europe	Girls	1991	16	12.63
Central and Eastern Europe	Girls	1992	16	12.70
Central and Eastern Europe	Girls	1993	16	12.77
Central and Eastern Europe	Girls	1994	16	12.84
Central and Eastern Europe	Girls	1995	16	12.90
Central and Eastern Europe	Girls	1996	16	12.97
Central and Eastern Europe	Girls	1997	16	13.03
Central and Eastern Europe	Girls	1998	16	13.10
Central and Eastern Europe	Girls	1999	16	13.16
Central and Eastern Europe	Girls	2000	16	13.23
Central and Eastern Europe	Girls	2001	16	13.31
Central and Eastern Europe	Girls	2002	16	13.39
Central and Eastern Europe	Girls	2003	16	13.48
Central and Eastern Europe	Girls	2004	16	13.57
Central and Eastern Europe	Girls	2005	16	13.66
Central and Eastern Europe	Girls	2006	16	13.76
Central and Eastern Europe	Girls	2007	16	13.87
Central and Eastern Europe	Girls	2008	16	13.98
Central and Eastern Europe	Girls	2009	16	14.10
Central and Eastern Europe	Girls	2010	16	14.22
Central and Eastern Europe	Girls	2011	16	14.35
Central and Eastern Europe	Girls	2012	16	14.48
Central and Eastern Europe	Girls	2013	16	14.61
Central and Eastern Europe	Girls	2014	16	14.75
Central and Eastern Europe	Girls	2015	16	14.89
Central and Eastern Europe	Girls	2016	16	15.03
Central and Eastern Europe	Girls	1975	17	12.22
Central and Eastern Europe	Girls	1976	17	12.30
Central and Eastern Europe	Girls	1977	17	12.38
Central and Eastern Europe	Girls	1978	17	12.46
Central and Eastern Europe	Girls	1979	17	12.55
Central and Eastern Europe	Girls	1980	17	12.63
Central and Eastern Europe	Girls	1981	17	12.70
Central and Eastern Europe	Girls	1982	17	12.78
Central and Eastern Europe	Girls	1983	17	12.85
Central and Eastern Europe	Girls	1984	17	12.92
Central and Eastern Europe	Girls	1985	17	12.99

Central and Eastern Europe	Girls	1986	17	13.07
Central and Eastern Europe	Girls	1987	17	13.15
Central and Eastern Europe	Girls	1988	17	13.24
Central and Eastern Europe	Girls	1989	17	13.32
Central and Eastern Europe	Girls	1990	17	13.40
Central and Eastern Europe	Girls	1991	17	13.48
Central and Eastern Europe	Girls	1992	17	13.55
Central and Eastern Europe	Girls	1993	17	13.62
Central and Eastern Europe	Girls	1994	17	13.69
Central and Eastern Europe	Girls	1995	17	13.75
Central and Eastern Europe	Girls	1996	17	13.82
Central and Eastern Europe	Girls	1997	17	13.88
Central and Eastern Europe	Girls	1998	17	13.95
Central and Eastern Europe	Girls	1999	17	14.02
Central and Eastern Europe	Girls	2000	17	14.09
Central and Eastern Europe	Girls	2001	17	14.17
Central and Eastern Europe	Girls	2002	17	14.25
Central and Eastern Europe	Girls	2003	17	14.34
Central and Eastern Europe	Girls	2004	17	14.44
Central and Eastern Europe	Girls	2005	17	14.54
Central and Eastern Europe	Girls	2006	17	14.64
Central and Eastern Europe	Girls	2007	17	14.75
Central and Eastern Europe	Girls	2008	17	14.87
Central and Eastern Europe	Girls	2009	17	14.99
Central and Eastern Europe	Girls	2010	17	15.12
Central and Eastern Europe	Girls	2011	17	15.25
Central and Eastern Europe	Girls	2012	17	15.38
Central and Eastern Europe	Girls	2013	17	15.52
Central and Eastern Europe	Girls	2014	17	15.66
Central and Eastern Europe	Girls	2015	17	15.80
Central and Eastern Europe	Girls	2016	17	15.95
Central and Eastern Europe	Girls	1975	18	12.80
Central and Eastern Europe	Girls	1976	18	12.88
Central and Eastern Europe	Girls	1977	18	12.97
Central and Eastern Europe	Girls	1978	18	13.06
Central and Eastern Europe	Girls	1979	18	13.15
Central and Eastern Europe	Girls	1980	18	13.24
Central and Eastern Europe	Girls	1981	18	13.32
Central and Eastern Europe	Girls	1982	18	13.40
Central and Eastern Europe	Girls	1983	18	13.47
Central and Eastern Europe	Girls	1984	18	13.55
Central and Eastern Europe	Girls	1985	18	13.63
Central and Eastern Europe	Girls	1986	18	13.71
Central and Eastern Europe	Girls	1987	18	13.80
Central and Eastern Europe	Girls	1988	18	13.89
Central and Eastern Europe	Girls	1989	18	13.98
Central and Eastern Europe	Girls	1990	18	14.06
Central and Eastern Europe	Girls	1991	18	14.14
Central and Eastern Europe	Girls	1992	18	14.22
Central and Eastern Europe	Girls	1993	18	14.30
Central and Eastern Europe	Girls	1994	18	14.37
Central and Eastern Europe	Girls	1995	18	14.44

Central and Eastern Europe	Girls	1996	18	14.51
Central and Eastern Europe	Girls	1997	18	14.58
Central and Eastern Europe	Girls	1998	18	14.65
Central and Eastern Europe	Girls	1999	18	14.73
Central and Eastern Europe	Girls	2000	18	14.80
Central and Eastern Europe	Girls	2001	18	14.89
Central and Eastern Europe	Girls	2002	18	14.98
Central and Eastern Europe	Girls	2003	18	15.07
Central and Eastern Europe	Girls	2004	18	15.17
Central and Eastern Europe	Girls	2005	18	15.28
Central and Eastern Europe	Girls	2006	18	15.39
Central and Eastern Europe	Girls	2007	18	15.51
Central and Eastern Europe	Girls	2008	18	15.63
Central and Eastern Europe	Girls	2009	18	15.77
Central and Eastern Europe	Girls	2010	18	15.90
Central and Eastern Europe	Girls	2011	18	16.04
Central and Eastern Europe	Girls	2012	18	16.18
Central and Eastern Europe	Girls	2013	18	16.33
Central and Eastern Europe	Girls	2014	18	16.48
Central and Eastern Europe	Girls	2015	18	16.64
Central and Eastern Europe	Girls	2016	18	16.79
Central and Eastern Europe	Girls	1975	Average	9.84
Central and Eastern Europe	Girls	1976	Average	9.91
Central and Eastern Europe	Girls	1977	Average	9.98
Central and Eastern Europe	Girls	1978	Average	10.05
Central and Eastern Europe	Girls	1979	Average	10.13
Central and Eastern Europe	Girls	1980	Average	10.20
Central and Eastern Europe	Girls	1981	Average	10.27
Central and Eastern Europe	Girls	1982	Average	10.35
Central and Eastern Europe	Girls	1983	Average	10.42
Central and Eastern Europe	Girls	1984	Average	10.49
Central and Eastern Europe	Girls	1985	Average	10.56
Central and Eastern Europe	Girls	1986	Average	10.63
Central and Eastern Europe	Girls	1987	Average	10.71
Central and Eastern Europe	Girls	1988	Average	10.79
Central and Eastern Europe	Girls	1989	Average	10.86
Central and Eastern Europe	Girls	1990	Average	10.93
Central and Eastern Europe	Girls	1991	Average	11.00
Central and Eastern Europe	Girls	1992	Average	11.07
Central and Eastern Europe	Girls	1993	Average	11.14
Central and Eastern Europe	Girls	1994	Average	11.20
Central and Eastern Europe	Girls	1995	Average	11.26
Central and Eastern Europe	Girls	1996	Average	11.33
Central and Eastern Europe	Girls	1997	Average	11.39
Central and Eastern Europe	Girls	1998	Average	11.45
Central and Eastern Europe	Girls	1999	Average	11.52
Central and Eastern Europe	Girls	2000	Average	11.59
Central and Eastern Europe	Girls	2001	Average	11.66
Central and Eastern Europe	Girls	2002	Average	11.74
Central and Eastern Europe	Girls	2003	Average	11.83
Central and Eastern Europe	Girls	2004	Average	11.92
Central and Eastern Europe	Girls	2005	Average	12.01

Central and Eastern Europe	Girls	2006	Average	12.11
Central and Eastern Europe	Girls	2007	Average	12.22
Central and Eastern Europe	Girls	2008	Average	12.32
Central and Eastern Europe	Girls	2009	Average	12.44
Central and Eastern Europe	Girls	2010	Average	12.55
Central and Eastern Europe	Girls	2011	Average	12.67
Central and Eastern Europe	Girls	2012	Average	12.79
Central and Eastern Europe	Girls	2013	Average	12.92
Central and Eastern Europe	Girls	2014	Average	13.04
Central and Eastern Europe	Girls	2015	Average	13.17
Central and Eastern Europe	Girls	2016	Average	13.31
Central and Eastern Europe	Girls	2017	Average	13.44
Central and Eastern Europe	Girls	2018	Average	13.57
Central and Eastern Europe	Girls	2019	Average	13.71
Central and Eastern Europe	Girls	2020	Average	13.85
Central and Eastern Europe	Girls	2021	Average	13.98
Central and Eastern Europe	Girls	2022	Average	14.12
Central and Eastern Europe	Girls	2023	Average	14.26
Central and Eastern Europe	Girls	2024	Average	14.40
Central and Eastern Europe	Girls	2025	Average	14.54
Central and Eastern Europe	Girls	2026	Average	14.67
Central and Eastern Europe	Girls	2027	Average	14.81
Central and Eastern Europe	Girls	2028	Average	14.95
Central and Eastern Europe	Girls	2029	Average	15.09
Central and Eastern Europe	Girls	2030	Average	15.23
Central Asia and North Africa-Middle East	Boys	1975	12	5.47
Central Asia and North Africa-Middle East	Boys	1976	12	5.54
Central Asia and North Africa-Middle East	Boys	1977	12	5.61
Central Asia and North Africa-Middle East	Boys	1978	12	5.68
Central Asia and North Africa-Middle East	Boys	1979	12	5.76
Central Asia and North Africa-Middle East	Boys	1980	12	5.85
Central Asia and North Africa-Middle East	Boys	1981	12	5.94
Central Asia and North Africa-Middle East	Boys	1982	12	6.03
Central Asia and North Africa-Middle East	Boys	1983	12	6.13
Central Asia and North Africa-Middle East	Boys	1984	12	6.24
Central Asia and North Africa-Middle East	Boys	1985	12	6.36
Central Asia and North Africa-Middle East	Boys	1986	12	6.48
Central Asia and North Africa-Middle East	Boys	1987	12	6.60
Central Asia and North Africa-Middle East	Boys	1988	12	6.73
Central Asia and North Africa-Middle East	Boys	1989	12	6.87
Central Asia and North Africa-Middle East	Boys	1990	12	7.01
Central Asia and North Africa-Middle East	Boys	1991	12	7.15
Central Asia and North Africa-Middle East	Boys	1992	12	7.29
Central Asia and North Africa-Middle East	Boys	1993	12	7.44
Central Asia and North Africa-Middle East	Boys	1994	12	7.59
Central Asia and North Africa-Middle East	Boys	1995	12	7.75
Central Asia and North Africa-Middle East	Boys	1996	12	7.91
Central Asia and North Africa-Middle East	Boys	1997	12	8.07
Central Asia and North Africa-Middle East	Boys	1998	12	8.24
Central Asia and North Africa-Middle East	Boys	1999	12	8.41
Central Asia and North Africa-Middle East	Boys	2000	12	8.59
Central Asia and North Africa-Middle East	Boys	2001	12	8.77

Central Asia and North Africa-Middle East	Boys	2002	12	8.97
Central Asia and North Africa-Middle East	Boys	2003	12	9.16
Central Asia and North Africa-Middle East	Boys	2004	12	9.36
Central Asia and North Africa-Middle East	Boys	2005	12	9.56
Central Asia and North Africa-Middle East	Boys	2006	12	9.76
Central Asia and North Africa-Middle East	Boys	2007	12	9.97
Central Asia and North Africa-Middle East	Boys	2008	12	10.18
Central Asia and North Africa-Middle East	Boys	2009	12	10.39
Central Asia and North Africa-Middle East	Boys	2010	12	10.60
Central Asia and North Africa-Middle East	Boys	2011	12	10.80
Central Asia and North Africa-Middle East	Boys	2012	12	11.01
Central Asia and North Africa-Middle East	Boys	2013	12	11.22
Central Asia and North Africa-Middle East	Boys	2014	12	11.42
Central Asia and North Africa-Middle East	Boys	2015	12	11.63
Central Asia and North Africa-Middle East	Boys	2016	12	11.84
Central Asia and North Africa-Middle East	Boys	1975	13	6.77
Central Asia and North Africa-Middle East	Boys	1976	13	6.85
Central Asia and North Africa-Middle East	Boys	1977	13	6.93
Central Asia and North Africa-Middle East	Boys	1978	13	7.02
Central Asia and North Africa-Middle East	Boys	1979	13	7.11
Central Asia and North Africa-Middle East	Boys	1980	13	7.21
Central Asia and North Africa-Middle East	Boys	1981	13	7.31
Central Asia and North Africa-Middle East	Boys	1982	13	7.42
Central Asia and North Africa-Middle East	Boys	1983	13	7.54
Central Asia and North Africa-Middle East	Boys	1984	13	7.67
Central Asia and North Africa-Middle East	Boys	1985	13	7.80
Central Asia and North Africa-Middle East	Boys	1986	13	7.94
Central Asia and North Africa-Middle East	Boys	1987	13	8.08
Central Asia and North Africa-Middle East	Boys	1988	13	8.23
Central Asia and North Africa-Middle East	Boys	1989	13	8.39
Central Asia and North Africa-Middle East	Boys	1990	13	8.55
Central Asia and North Africa-Middle East	Boys	1991	13	8.71
Central Asia and North Africa-Middle East	Boys	1992	13	8.88
Central Asia and North Africa-Middle East	Boys	1993	13	9.05
Central Asia and North Africa-Middle East	Boys	1994	13	9.23
Central Asia and North Africa-Middle East	Boys	1995	13	9.41
Central Asia and North Africa-Middle East	Boys	1996	13	9.60
Central Asia and North Africa-Middle East	Boys	1997	13	9.79
Central Asia and North Africa-Middle East	Boys	1998	13	9.99
Central Asia and North Africa-Middle East	Boys	1999	13	10.19
Central Asia and North Africa-Middle East	Boys	2000	13	10.40
Central Asia and North Africa-Middle East	Boys	2001	13	10.62
Central Asia and North Africa-Middle East	Boys	2002	13	10.84
Central Asia and North Africa-Middle East	Boys	2003	13	11.07
Central Asia and North Africa-Middle East	Boys	2004	13	11.31
Central Asia and North Africa-Middle East	Boys	2005	13	11.55
Central Asia and North Africa-Middle East	Boys	2006	13	11.79
Central Asia and North Africa-Middle East	Boys	2007	13	12.04
Central Asia and North Africa-Middle East	Boys	2008	13	12.29
Central Asia and North Africa-Middle East	Boys	2009	13	12.54
Central Asia and North Africa-Middle East	Boys	2010	13	12.79
Central Asia and North Africa-Middle East	Boys	2011	13	13.04

Central Asia and North Africa-Middle East	Boys	2012	13	13.30
Central Asia and North Africa-Middle East	Boys	2013	13	13.55
Central Asia and North Africa-Middle East	Boys	2014	13	13.80
Central Asia and North Africa-Middle East	Boys	2015	13	14.06
Central Asia and North Africa-Middle East	Boys	2016	13	14.31
Central Asia and North Africa-Middle East	Boys	1975	14	8.39
Central Asia and North Africa-Middle East	Boys	1976	14	8.48
Central Asia and North Africa-Middle East	Boys	1977	14	8.57
Central Asia and North Africa-Middle East	Boys	1978	14	8.66
Central Asia and North Africa-Middle East	Boys	1979	14	8.76
Central Asia and North Africa-Middle East	Boys	1980	14	8.87
Central Asia and North Africa-Middle East	Boys	1981	14	8.98
Central Asia and North Africa-Middle East	Boys	1982	14	9.11
Central Asia and North Africa-Middle East	Boys	1983	14	9.24
Central Asia and North Africa-Middle East	Boys	1984	14	9.37
Central Asia and North Africa-Middle East	Boys	1985	14	9.52
Central Asia and North Africa-Middle East	Boys	1986	14	9.67
Central Asia and North Africa-Middle East	Boys	1987	14	9.83
Central Asia and North Africa-Middle East	Boys	1988	14	10.00
Central Asia and North Africa-Middle East	Boys	1989	14	10.17
Central Asia and North Africa-Middle East	Boys	1990	14	10.34
Central Asia and North Africa-Middle East	Boys	1991	14	10.52
Central Asia and North Africa-Middle East	Boys	1992	14	10.70
Central Asia and North Africa-Middle East	Boys	1993	14	10.89
Central Asia and North Africa-Middle East	Boys	1994	14	11.09
Central Asia and North Africa-Middle East	Boys	1995	14	11.29
Central Asia and North Africa-Middle East	Boys	1996	14	11.49
Central Asia and North Africa-Middle East	Boys	1997	14	11.71
Central Asia and North Africa-Middle East	Boys	1998	14	11.92
Central Asia and North Africa-Middle East	Boys	1999	14	12.15
Central Asia and North Africa-Middle East	Boys	2000	14	12.38
Central Asia and North Africa-Middle East	Boys	2001	14	12.63
Central Asia and North Africa-Middle East	Boys	2002	14	12.88
Central Asia and North Africa-Middle East	Boys	2003	14	13.14
Central Asia and North Africa-Middle East	Boys	2004	14	13.40
Central Asia and North Africa-Middle East	Boys	2005	14	13.67
Central Asia and North Africa-Middle East	Boys	2006	14	13.94
Central Asia and North Africa-Middle East	Boys	2007	14	14.22
Central Asia and North Africa-Middle East	Boys	2008	14	14.50
Central Asia and North Africa-Middle East	Boys	2009	14	14.78
Central Asia and North Africa-Middle East	Boys	2010	14	15.07
Central Asia and North Africa-Middle East	Boys	2011	14	15.36
Central Asia and North Africa-Middle East	Boys	2012	14	15.65
Central Asia and North Africa-Middle East	Boys	2013	14	15.94
Central Asia and North Africa-Middle East	Boys	2014	14	16.23
Central Asia and North Africa-Middle East	Boys	2015	14	16.52
Central Asia and North Africa-Middle East	Boys	2016	14	16.82
Central Asia and North Africa-Middle East	Boys	1975	15	10.26
Central Asia and North Africa-Middle East	Boys	1976	15	10.35
Central Asia and North Africa-Middle East	Boys	1977	15	10.45
Central Asia and North Africa-Middle East	Boys	1978	15	10.55
Central Asia and North Africa-Middle East	Boys	1979	15	10.66

Central Asia and North Africa-Middle East	Boys	1980	15	10.77
Central Asia and North Africa-Middle East	Boys	1981	15	10.89
Central Asia and North Africa-Middle East	Boys	1982	15	11.01
Central Asia and North Africa-Middle East	Boys	1983	15	11.15
Central Asia and North Africa-Middle East	Boys	1984	15	11.29
Central Asia and North Africa-Middle East	Boys	1985	15	11.45
Central Asia and North Africa-Middle East	Boys	1986	15	11.61
Central Asia and North Africa-Middle East	Boys	1987	15	11.78
Central Asia and North Africa-Middle East	Boys	1988	15	11.95
Central Asia and North Africa-Middle East	Boys	1989	15	12.13
Central Asia and North Africa-Middle East	Boys	1990	15	12.31
Central Asia and North Africa-Middle East	Boys	1991	15	12.50
Central Asia and North Africa-Middle East	Boys	1992	15	12.70
Central Asia and North Africa-Middle East	Boys	1993	15	12.90
Central Asia and North Africa-Middle East	Boys	1994	15	13.10
Central Asia and North Africa-Middle East	Boys	1995	15	13.31
Central Asia and North Africa-Middle East	Boys	1996	15	13.52
Central Asia and North Africa-Middle East	Boys	1997	15	13.75
Central Asia and North Africa-Middle East	Boys	1998	15	13.97
Central Asia and North Africa-Middle East	Boys	1999	15	14.21
Central Asia and North Africa-Middle East	Boys	2000	15	14.46
Central Asia and North Africa-Middle East	Boys	2001	15	14.72
Central Asia and North Africa-Middle East	Boys	2002	15	14.98
Central Asia and North Africa-Middle East	Boys	2003	15	15.25
Central Asia and North Africa-Middle East	Boys	2004	15	15.53
Central Asia and North Africa-Middle East	Boys	2005	15	15.80
Central Asia and North Africa-Middle East	Boys	2006	15	16.08
Central Asia and North Africa-Middle East	Boys	2007	15	16.36
Central Asia and North Africa-Middle East	Boys	2008	15	16.63
Central Asia and North Africa-Middle East	Boys	2009	15	16.92
Central Asia and North Africa-Middle East	Boys	2010	15	17.21
Central Asia and North Africa-Middle East	Boys	2011	15	17.51
Central Asia and North Africa-Middle East	Boys	2012	15	17.82
Central Asia and North Africa-Middle East	Boys	2013	15	18.13
Central Asia and North Africa-Middle East	Boys	2014	15	18.46
Central Asia and North Africa-Middle East	Boys	2015	15	18.78
Central Asia and North Africa-Middle East	Boys	2016	15	19.12
Central Asia and North Africa-Middle East	Boys	1975	16	12.30
Central Asia and North Africa-Middle East	Boys	1976	16	12.40
Central Asia and North Africa-Middle East	Boys	1977	16	12.51
Central Asia and North Africa-Middle East	Boys	1978	16	12.61
Central Asia and North Africa-Middle East	Boys	1979	16	12.72
Central Asia and North Africa-Middle East	Boys	1980	16	12.84
Central Asia and North Africa-Middle East	Boys	1981	16	12.96
Central Asia and North Africa-Middle East	Boys	1982	16	13.09
Central Asia and North Africa-Middle East	Boys	1983	16	13.23
Central Asia and North Africa-Middle East	Boys	1984	16	13.37
Central Asia and North Africa-Middle East	Boys	1985	16	13.53
Central Asia and North Africa-Middle East	Boys	1986	16	13.70
Central Asia and North Africa-Middle East	Boys	1987	16	13.87
Central Asia and North Africa-Middle East	Boys	1988	16	14.04
Central Asia and North Africa-Middle East	Boys	1989	16	14.22

Central Asia and North Africa-Middle East	Boys	1990	16	14.41
Central Asia and North Africa-Middle East	Boys	1991	16	14.61
Central Asia and North Africa-Middle East	Boys	1992	16	14.80
Central Asia and North Africa-Middle East	Boys	1993	16	15.01
Central Asia and North Africa-Middle East	Boys	1994	16	15.21
Central Asia and North Africa-Middle East	Boys	1995	16	15.43
Central Asia and North Africa-Middle East	Boys	1996	16	15.64
Central Asia and North Africa-Middle East	Boys	1997	16	15.87
Central Asia and North Africa-Middle East	Boys	1998	16	16.10
Central Asia and North Africa-Middle East	Boys	1999	16	16.35
Central Asia and North Africa-Middle East	Boys	2000	16	16.60
Central Asia and North Africa-Middle East	Boys	2001	16	16.87
Central Asia and North Africa-Middle East	Boys	2002	16	17.14
Central Asia and North Africa-Middle East	Boys	2003	16	17.42
Central Asia and North Africa-Middle East	Boys	2004	16	17.70
Central Asia and North Africa-Middle East	Boys	2005	16	17.98
Central Asia and North Africa-Middle East	Boys	2006	16	18.27
Central Asia and North Africa-Middle East	Boys	2007	16	18.55
Central Asia and North Africa-Middle East	Boys	2008	16	18.84
Central Asia and North Africa-Middle East	Boys	2009	16	19.13
Central Asia and North Africa-Middle East	Boys	2010	16	19.43
Central Asia and North Africa-Middle East	Boys	2011	16	19.74
Central Asia and North Africa-Middle East	Boys	2012	16	20.06
Central Asia and North Africa-Middle East	Boys	2013	16	20.39
Central Asia and North Africa-Middle East	Boys	2014	16	20.73
Central Asia and North Africa-Middle East	Boys	2015	16	21.07
Central Asia and North Africa-Middle East	Boys	2016	16	21.42
Central Asia and North Africa-Middle East	Boys	1975	17	14.44
Central Asia and North Africa-Middle East	Boys	1976	17	14.54
Central Asia and North Africa-Middle East	Boys	1977	17	14.65
Central Asia and North Africa-Middle East	Boys	1978	17	14.76
Central Asia and North Africa-Middle East	Boys	1979	17	14.87
Central Asia and North Africa-Middle East	Boys	1980	17	14.99
Central Asia and North Africa-Middle East	Boys	1981	17	15.12
Central Asia and North Africa-Middle East	Boys	1982	17	15.25
Central Asia and North Africa-Middle East	Boys	1983	17	15.39
Central Asia and North Africa-Middle East	Boys	1984	17	15.54
Central Asia and North Africa-Middle East	Boys	1985	17	15.70
Central Asia and North Africa-Middle East	Boys	1986	17	15.87
Central Asia and North Africa-Middle East	Boys	1987	17	16.04
Central Asia and North Africa-Middle East	Boys	1988	17	16.22
Central Asia and North Africa-Middle East	Boys	1989	17	16.41
Central Asia and North Africa-Middle East	Boys	1990	17	16.60
Central Asia and North Africa-Middle East	Boys	1991	17	16.80
Central Asia and North Africa-Middle East	Boys	1992	17	17.00
Central Asia and North Africa-Middle East	Boys	1993	17	17.20
Central Asia and North Africa-Middle East	Boys	1994	17	17.41
Central Asia and North Africa-Middle East	Boys	1995	17	17.63
Central Asia and North Africa-Middle East	Boys	1996	17	17.85
Central Asia and North Africa-Middle East	Boys	1997	17	18.08
Central Asia and North Africa-Middle East	Boys	1998	17	18.32
Central Asia and North Africa-Middle East	Boys	1999	17	18.57

Central Asia and North Africa-Middle East	Boys	2000	17	18.83
Central Asia and North Africa-Middle East	Boys	2001	17	19.10
Central Asia and North Africa-Middle East	Boys	2002	17	19.38
Central Asia and North Africa-Middle East	Boys	2003	17	19.66
Central Asia and North Africa-Middle East	Boys	2004	17	19.95
Central Asia and North Africa-Middle East	Boys	2005	17	20.24
Central Asia and North Africa-Middle East	Boys	2006	17	20.53
Central Asia and North Africa-Middle East	Boys	2007	17	20.82
Central Asia and North Africa-Middle East	Boys	2008	17	21.12
Central Asia and North Africa-Middle East	Boys	2009	17	21.42
Central Asia and North Africa-Middle East	Boys	2010	17	21.72
Central Asia and North Africa-Middle East	Boys	2011	17	22.04
Central Asia and North Africa-Middle East	Boys	2012	17	22.37
Central Asia and North Africa-Middle East	Boys	2013	17	22.71
Central Asia and North Africa-Middle East	Boys	2014	17	23.05
Central Asia and North Africa-Middle East	Boys	2015	17	23.41
Central Asia and North Africa-Middle East	Boys	2016	17	23.77
Central Asia and North Africa-Middle East	Boys	1975	18	16.58
Central Asia and North Africa-Middle East	Boys	1976	18	16.70
Central Asia and North Africa-Middle East	Boys	1977	18	16.81
Central Asia and North Africa-Middle East	Boys	1978	18	16.93
Central Asia and North Africa-Middle East	Boys	1979	18	17.05
Central Asia and North Africa-Middle East	Boys	1980	18	17.18
Central Asia and North Africa-Middle East	Boys	1981	18	17.31
Central Asia and North Africa-Middle East	Boys	1982	18	17.45
Central Asia and North Africa-Middle East	Boys	1983	18	17.60
Central Asia and North Africa-Middle East	Boys	1984	18	17.76
Central Asia and North Africa-Middle East	Boys	1985	18	17.93
Central Asia and North Africa-Middle East	Boys	1986	18	18.10
Central Asia and North Africa-Middle East	Boys	1987	18	18.29
Central Asia and North Africa-Middle East	Boys	1988	18	18.47
Central Asia and North Africa-Middle East	Boys	1989	18	18.67
Central Asia and North Africa-Middle East	Boys	1990	18	18.87
Central Asia and North Africa-Middle East	Boys	1991	18	19.07
Central Asia and North Africa-Middle East	Boys	1992	18	19.28
Central Asia and North Africa-Middle East	Boys	1993	18	19.50
Central Asia and North Africa-Middle East	Boys	1994	18	19.72
Central Asia and North Africa-Middle East	Boys	1995	18	19.94
Central Asia and North Africa-Middle East	Boys	1996	18	20.18
Central Asia and North Africa-Middle East	Boys	1997	18	20.42
Central Asia and North Africa-Middle East	Boys	1998	18	20.66
Central Asia and North Africa-Middle East	Boys	1999	18	20.92
Central Asia and North Africa-Middle East	Boys	2000	18	21.19
Central Asia and North Africa-Middle East	Boys	2001	18	21.47
Central Asia and North Africa-Middle East	Boys	2002	18	21.76
Central Asia and North Africa-Middle East	Boys	2003	18	22.06
Central Asia and North Africa-Middle East	Boys	2004	18	22.36
Central Asia and North Africa-Middle East	Boys	2005	18	22.67
Central Asia and North Africa-Middle East	Boys	2006	18	22.97
Central Asia and North Africa-Middle East	Boys	2007	18	23.27
Central Asia and North Africa-Middle East	Boys	2008	18	23.57
Central Asia and North Africa-Middle East	Boys	2009	18	23.88

Central Asia and North Africa-Middle East	Boys	2010	18	24.20
Central Asia and North Africa-Middle East	Boys	2011	18	24.53
Central Asia and North Africa-Middle East	Boys	2012	18	24.87
Central Asia and North Africa-Middle East	Boys	2013	18	25.22
Central Asia and North Africa-Middle East	Boys	2014	18	25.58
Central Asia and North Africa-Middle East	Boys	2015	18	25.94
Central Asia and North Africa-Middle East	Boys	2016	18	26.32
Central Asia and North Africa-Middle East	Boys	1975	Average	10.60
Central Asia and North Africa-Middle East	Boys	1976	Average	10.69
Central Asia and North Africa-Middle East	Boys	1977	Average	10.79
Central Asia and North Africa-Middle East	Boys	1978	Average	10.89
Central Asia and North Africa-Middle East	Boys	1979	Average	10.99
Central Asia and North Africa-Middle East	Boys	1980	Average	11.10
Central Asia and North Africa-Middle East	Boys	1981	Average	11.21
Central Asia and North Africa-Middle East	Boys	1982	Average	11.34
Central Asia and North Africa-Middle East	Boys	1983	Average	11.47
Central Asia and North Africa-Middle East	Boys	1984	Average	11.61
Central Asia and North Africa-Middle East	Boys	1985	Average	11.76
Central Asia and North Africa-Middle East	Boys	1986	Average	11.91
Central Asia and North Africa-Middle East	Boys	1987	Average	12.07
Central Asia and North Africa-Middle East	Boys	1988	Average	12.24
Central Asia and North Africa-Middle East	Boys	1989	Average	12.41
Central Asia and North Africa-Middle East	Boys	1990	Average	12.58
Central Asia and North Africa-Middle East	Boys	1991	Average	12.77
Central Asia and North Africa-Middle East	Boys	1992	Average	12.95
Central Asia and North Africa-Middle East	Boys	1993	Average	13.14
Central Asia and North Africa-Middle East	Boys	1994	Average	13.34
Central Asia and North Africa-Middle East	Boys	1995	Average	13.54
Central Asia and North Africa-Middle East	Boys	1996	Average	13.74
Central Asia and North Africa-Middle East	Boys	1997	Average	13.95
Central Asia and North Africa-Middle East	Boys	1998	Average	14.17
Central Asia and North Africa-Middle East	Boys	1999	Average	14.40
Central Asia and North Africa-Middle East	Boys	2000	Average	14.64
Central Asia and North Africa-Middle East	Boys	2001	Average	14.88
Central Asia and North Africa-Middle East	Boys	2002	Average	15.14
Central Asia and North Africa-Middle East	Boys	2003	Average	15.40
Central Asia and North Africa-Middle East	Boys	2004	Average	15.66
Central Asia and North Africa-Middle East	Boys	2005	Average	15.93
Central Asia and North Africa-Middle East	Boys	2006	Average	16.19
Central Asia and North Africa-Middle East	Boys	2007	Average	16.46
Central Asia and North Africa-Middle East	Boys	2008	Average	16.73
Central Asia and North Africa-Middle East	Boys	2009	Average	17.01
Central Asia and North Africa-Middle East	Boys	2010	Average	17.29
Central Asia and North Africa-Middle East	Boys	2011	Average	17.58
Central Asia and North Africa-Middle East	Boys	2012	Average	17.87
Central Asia and North Africa-Middle East	Boys	2013	Average	18.16
Central Asia and North Africa-Middle East	Boys	2014	Average	18.47
Central Asia and North Africa-Middle East	Boys	2015	Average	18.77
Central Asia and North Africa-Middle East	Boys	2016	Average	19.09
Central Asia and North Africa-Middle East	Boys	2017	Average	19.40
Central Asia and North Africa-Middle East	Boys	2018	Average	19.72
Central Asia and North Africa-Middle East	Boys	2019	Average	20.04

Central Asia and North Africa-Middle East	Boys	2020	Average	20.37
Central Asia and North Africa-Middle East	Boys	2021	Average	20.70
Central Asia and North Africa-Middle East	Boys	2022	Average	21.03
Central Asia and North Africa-Middle East	Boys	2023	Average	21.37
Central Asia and North Africa-Middle East	Boys	2024	Average	21.71
Central Asia and North Africa-Middle East	Boys	2025	Average	22.05
Central Asia and North Africa-Middle East	Boys	2026	Average	22.39
Central Asia and North Africa-Middle East	Boys	2027	Average	22.74
Central Asia and North Africa-Middle East	Boys	2028	Average	23.08
Central Asia and North Africa-Middle East	Boys	2029	Average	23.43
Central Asia and North Africa-Middle East	Boys	2030	Average	23.78
Central Asia and North Africa-Middle East	Girls	1975	12	5.99
Central Asia and North Africa-Middle East	Girls	1976	12	6.07
Central Asia and North Africa-Middle East	Girls	1977	12	6.15
Central Asia and North Africa-Middle East	Girls	1978	12	6.23
Central Asia and North Africa-Middle East	Girls	1979	12	6.31
Central Asia and North Africa-Middle East	Girls	1980	12	6.40
Central Asia and North Africa-Middle East	Girls	1981	12	6.49
Central Asia and North Africa-Middle East	Girls	1982	12	6.58
Central Asia and North Africa-Middle East	Girls	1983	12	6.68
Central Asia and North Africa-Middle East	Girls	1984	12	6.78
Central Asia and North Africa-Middle East	Girls	1985	12	6.89
Central Asia and North Africa-Middle East	Girls	1986	12	6.99
Central Asia and North Africa-Middle East	Girls	1987	12	7.10
Central Asia and North Africa-Middle East	Girls	1988	12	7.22
Central Asia and North Africa-Middle East	Girls	1989	12	7.33
Central Asia and North Africa-Middle East	Girls	1990	12	7.45
Central Asia and North Africa-Middle East	Girls	1991	12	7.57
Central Asia and North Africa-Middle East	Girls	1992	12	7.68
Central Asia and North Africa-Middle East	Girls	1993	12	7.80
Central Asia and North Africa-Middle East	Girls	1994	12	7.92
Central Asia and North Africa-Middle East	Girls	1995	12	8.04
Central Asia and North Africa-Middle East	Girls	1996	12	8.16
Central Asia and North Africa-Middle East	Girls	1997	12	8.28
Central Asia and North Africa-Middle East	Girls	1998	12	8.41
Central Asia and North Africa-Middle East	Girls	1999	12	8.53
Central Asia and North Africa-Middle East	Girls	2000	12	8.66
Central Asia and North Africa-Middle East	Girls	2001	12	8.79
Central Asia and North Africa-Middle East	Girls	2002	12	8.92
Central Asia and North Africa-Middle East	Girls	2003	12	9.05
Central Asia and North Africa-Middle East	Girls	2004	12	9.18
Central Asia and North Africa-Middle East	Girls	2005	12	9.31
Central Asia and North Africa-Middle East	Girls	2006	12	9.44
Central Asia and North Africa-Middle East	Girls	2007	12	9.58
Central Asia and North Africa-Middle East	Girls	2008	12	9.72
Central Asia and North Africa-Middle East	Girls	2009	12	9.86
Central Asia and North Africa-Middle East	Girls	2010	12	9.99
Central Asia and North Africa-Middle East	Girls	2011	12	10.13
Central Asia and North Africa-Middle East	Girls	2012	12	10.27
Central Asia and North Africa-Middle East	Girls	2013	12	10.40
Central Asia and North Africa-Middle East	Girls	2014	12	10.53
Central Asia and North Africa-Middle East	Girls	2015	12	10.67

Central Asia and North Africa-Middle East	Girls	2016	12	10.80
Central Asia and North Africa-Middle East	Girls	1975	13	7.45
Central Asia and North Africa-Middle East	Girls	1976	13	7.54
Central Asia and North Africa-Middle East	Girls	1977	13	7.63
Central Asia and North Africa-Middle East	Girls	1978	13	7.72
Central Asia and North Africa-Middle East	Girls	1979	13	7.82
Central Asia and North Africa-Middle East	Girls	1980	13	7.92
Central Asia and North Africa-Middle East	Girls	1981	13	8.02
Central Asia and North Africa-Middle East	Girls	1982	13	8.13
Central Asia and North Africa-Middle East	Girls	1983	13	8.24
Central Asia and North Africa-Middle East	Girls	1984	13	8.36
Central Asia and North Africa-Middle East	Girls	1985	13	8.48
Central Asia and North Africa-Middle East	Girls	1986	13	8.61
Central Asia and North Africa-Middle East	Girls	1987	13	8.74
Central Asia and North Africa-Middle East	Girls	1988	13	8.87
Central Asia and North Africa-Middle East	Girls	1989	13	9.00
Central Asia and North Africa-Middle East	Girls	1990	13	9.13
Central Asia and North Africa-Middle East	Girls	1991	13	9.27
Central Asia and North Africa-Middle East	Girls	1992	13	9.40
Central Asia and North Africa-Middle East	Girls	1993	13	9.54
Central Asia and North Africa-Middle East	Girls	1994	13	9.67
Central Asia and North Africa-Middle East	Girls	1995	13	9.81
Central Asia and North Africa-Middle East	Girls	1996	13	9.95
Central Asia and North Africa-Middle East	Girls	1997	13	10.09
Central Asia and North Africa-Middle East	Girls	1998	13	10.23
Central Asia and North Africa-Middle East	Girls	1999	13	10.37
Central Asia and North Africa-Middle East	Girls	2000	13	10.52
Central Asia and North Africa-Middle East	Girls	2001	13	10.66
Central Asia and North Africa-Middle East	Girls	2002	13	10.81
Central Asia and North Africa-Middle East	Girls	2003	13	10.96
Central Asia and North Africa-Middle East	Girls	2004	13	11.11
Central Asia and North Africa-Middle East	Girls	2005	13	11.26
Central Asia and North Africa-Middle East	Girls	2006	13	11.41
Central Asia and North Africa-Middle East	Girls	2007	13	11.57
Central Asia and North Africa-Middle East	Girls	2008	13	11.72
Central Asia and North Africa-Middle East	Girls	2009	13	11.88
Central Asia and North Africa-Middle East	Girls	2010	13	12.04
Central Asia and North Africa-Middle East	Girls	2011	13	12.20
Central Asia and North Africa-Middle East	Girls	2012	13	12.35
Central Asia and North Africa-Middle East	Girls	2013	13	12.51
Central Asia and North Africa-Middle East	Girls	2014	13	12.66
Central Asia and North Africa-Middle East	Girls	2015	13	12.82
Central Asia and North Africa-Middle East	Girls	2016	13	12.97
Central Asia and North Africa-Middle East	Girls	1975	14	9.00
Central Asia and North Africa-Middle East	Girls	1976	14	9.10
Central Asia and North Africa-Middle East	Girls	1977	14	9.20
Central Asia and North Africa-Middle East	Girls	1978	14	9.30
Central Asia and North Africa-Middle East	Girls	1979	14	9.41
Central Asia and North Africa-Middle East	Girls	1980	14	9.52
Central Asia and North Africa-Middle East	Girls	1981	14	9.64
Central Asia and North Africa-Middle East	Girls	1982	14	9.76
Central Asia and North Africa-Middle East	Girls	1983	14	9.88

Central Asia and North Africa-Middle East	Girls	1984	14	10.01
Central Asia and North Africa-Middle East	Girls	1985	14	10.14
Central Asia and North Africa-Middle East	Girls	1986	14	10.28
Central Asia and North Africa-Middle East	Girls	1987	14	10.42
Central Asia and North Africa-Middle East	Girls	1988	14	10.56
Central Asia and North Africa-Middle East	Girls	1989	14	10.71
Central Asia and North Africa-Middle East	Girls	1990	14	10.85
Central Asia and North Africa-Middle East	Girls	1991	14	11.00
Central Asia and North Africa-Middle East	Girls	1992	14	11.14
Central Asia and North Africa-Middle East	Girls	1993	14	11.29
Central Asia and North Africa-Middle East	Girls	1994	14	11.44
Central Asia and North Africa-Middle East	Girls	1995	14	11.59
Central Asia and North Africa-Middle East	Girls	1996	14	11.74
Central Asia and North Africa-Middle East	Girls	1997	14	11.89
Central Asia and North Africa-Middle East	Girls	1998	14	12.05
Central Asia and North Africa-Middle East	Girls	1999	14	12.20
Central Asia and North Africa-Middle East	Girls	2000	14	12.36
Central Asia and North Africa-Middle East	Girls	2001	14	12.51
Central Asia and North Africa-Middle East	Girls	2002	14	12.67
Central Asia and North Africa-Middle East	Girls	2003	14	12.83
Central Asia and North Africa-Middle East	Girls	2004	14	13.00
Central Asia and North Africa-Middle East	Girls	2005	14	13.16
Central Asia and North Africa-Middle East	Girls	2006	14	13.32
Central Asia and North Africa-Middle East	Girls	2007	14	13.49
Central Asia and North Africa-Middle East	Girls	2008	14	13.66
Central Asia and North Africa-Middle East	Girls	2009	14	13.83
Central Asia and North Africa-Middle East	Girls	2010	14	14.00
Central Asia and North Africa-Middle East	Girls	2011	14	14.17
Central Asia and North Africa-Middle East	Girls	2012	14	14.34
Central Asia and North Africa-Middle East	Girls	2013	14	14.52
Central Asia and North Africa-Middle East	Girls	2014	14	14.69
Central Asia and North Africa-Middle East	Girls	2015	14	14.86
Central Asia and North Africa-Middle East	Girls	2016	14	15.03
Central Asia and North Africa-Middle East	Girls	1975	15	10.44
Central Asia and North Africa-Middle East	Girls	1976	15	10.54
Central Asia and North Africa-Middle East	Girls	1977	15	10.65
Central Asia and North Africa-Middle East	Girls	1978	15	10.75
Central Asia and North Africa-Middle East	Girls	1979	15	10.87
Central Asia and North Africa-Middle East	Girls	1980	15	10.98
Central Asia and North Africa-Middle East	Girls	1981	15	11.10
Central Asia and North Africa-Middle East	Girls	1982	15	11.23
Central Asia and North Africa-Middle East	Girls	1983	15	11.36
Central Asia and North Africa-Middle East	Girls	1984	15	11.50
Central Asia and North Africa-Middle East	Girls	1985	15	11.64
Central Asia and North Africa-Middle East	Girls	1986	15	11.79
Central Asia and North Africa-Middle East	Girls	1987	15	11.94
Central Asia and North Africa-Middle East	Girls	1988	15	12.09
Central Asia and North Africa-Middle East	Girls	1989	15	12.24
Central Asia and North Africa-Middle East	Girls	1990	15	12.40
Central Asia and North Africa-Middle East	Girls	1991	15	12.55
Central Asia and North Africa-Middle East	Girls	1992	15	12.71
Central Asia and North Africa-Middle East	Girls	1993	15	12.86

Central Asia and North Africa-Middle East	Girls	1994	15	13.02
Central Asia and North Africa-Middle East	Girls	1995	15	13.18
Central Asia and North Africa-Middle East	Girls	1996	15	13.34
Central Asia and North Africa-Middle East	Girls	1997	15	13.50
Central Asia and North Africa-Middle East	Girls	1998	15	13.66
Central Asia and North Africa-Middle East	Girls	1999	15	13.82
Central Asia and North Africa-Middle East	Girls	2000	15	13.99
Central Asia and North Africa-Middle East	Girls	2001	15	14.15
Central Asia and North Africa-Middle East	Girls	2002	15	14.32
Central Asia and North Africa-Middle East	Girls	2003	15	14.48
Central Asia and North Africa-Middle East	Girls	2004	15	14.65
Central Asia and North Africa-Middle East	Girls	2005	15	14.81
Central Asia and North Africa-Middle East	Girls	2006	15	14.97
Central Asia and North Africa-Middle East	Girls	2007	15	15.13
Central Asia and North Africa-Middle East	Girls	2008	15	15.28
Central Asia and North Africa-Middle East	Girls	2009	15	15.44
Central Asia and North Africa-Middle East	Girls	2010	15	15.61
Central Asia and North Africa-Middle East	Girls	2011	15	15.78
Central Asia and North Africa-Middle East	Girls	2012	15	15.96
Central Asia and North Africa-Middle East	Girls	2013	15	16.14
Central Asia and North Africa-Middle East	Girls	2014	15	16.33
Central Asia and North Africa-Middle East	Girls	2015	15	16.52
Central Asia and North Africa-Middle East	Girls	2016	15	16.72
Central Asia and North Africa-Middle East	Girls	1975	16	11.60
Central Asia and North Africa-Middle East	Girls	1976	16	11.71
Central Asia and North Africa-Middle East	Girls	1977	16	11.82
Central Asia and North Africa-Middle East	Girls	1978	16	11.93
Central Asia and North Africa-Middle East	Girls	1979	16	12.04
Central Asia and North Africa-Middle East	Girls	1980	16	12.16
Central Asia and North Africa-Middle East	Girls	1981	16	12.29
Central Asia and North Africa-Middle East	Girls	1982	16	12.42
Central Asia and North Africa-Middle East	Girls	1983	16	12.56
Central Asia and North Africa-Middle East	Girls	1984	16	12.70
Central Asia and North Africa-Middle East	Girls	1985	16	12.85
Central Asia and North Africa-Middle East	Girls	1986	16	13.00
Central Asia and North Africa-Middle East	Girls	1987	16	13.16
Central Asia and North Africa-Middle East	Girls	1988	16	13.31
Central Asia and North Africa-Middle East	Girls	1989	16	13.47
Central Asia and North Africa-Middle East	Girls	1990	16	13.63
Central Asia and North Africa-Middle East	Girls	1991	16	13.79
Central Asia and North Africa-Middle East	Girls	1992	16	13.95
Central Asia and North Africa-Middle East	Girls	1993	16	14.12
Central Asia and North Africa-Middle East	Girls	1994	16	14.28
Central Asia and North Africa-Middle East	Girls	1995	16	14.44
Central Asia and North Africa-Middle East	Girls	1996	16	14.61
Central Asia and North Africa-Middle East	Girls	1997	16	14.77
Central Asia and North Africa-Middle East	Girls	1998	16	14.94
Central Asia and North Africa-Middle East	Girls	1999	16	15.11
Central Asia and North Africa-Middle East	Girls	2000	16	15.28
Central Asia and North Africa-Middle East	Girls	2001	16	15.45
Central Asia and North Africa-Middle East	Girls	2002	16	15.63
Central Asia and North Africa-Middle East	Girls	2003	16	15.80

Central Asia and North Africa-Middle East	Girls	2004	16	15.97
Central Asia and North Africa-Middle East	Girls	2005	16	16.14
Central Asia and North Africa-Middle East	Girls	2006	16	16.30
Central Asia and North Africa-Middle East	Girls	2007	16	16.46
Central Asia and North Africa-Middle East	Girls	2008	16	16.63
Central Asia and North Africa-Middle East	Girls	2009	16	16.79
Central Asia and North Africa-Middle East	Girls	2010	16	16.96
Central Asia and North Africa-Middle East	Girls	2011	16	17.14
Central Asia and North Africa-Middle East	Girls	2012	16	17.32
Central Asia and North Africa-Middle East	Girls	2013	16	17.51
Central Asia and North Africa-Middle East	Girls	2014	16	17.71
Central Asia and North Africa-Middle East	Girls	2015	16	17.91
Central Asia and North Africa-Middle East	Girls	2016	16	18.11
Central Asia and North Africa-Middle East	Girls	1975	17	12.45
Central Asia and North Africa-Middle East	Girls	1976	17	12.56
Central Asia and North Africa-Middle East	Girls	1977	17	12.67
Central Asia and North Africa-Middle East	Girls	1978	17	12.79
Central Asia and North Africa-Middle East	Girls	1979	17	12.91
Central Asia and North Africa-Middle East	Girls	1980	17	13.04
Central Asia and North Africa-Middle East	Girls	1981	17	13.17
Central Asia and North Africa-Middle East	Girls	1982	17	13.31
Central Asia and North Africa-Middle East	Girls	1983	17	13.46
Central Asia and North Africa-Middle East	Girls	1984	17	13.61
Central Asia and North Africa-Middle East	Girls	1985	17	13.76
Central Asia and North Africa-Middle East	Girls	1986	17	13.92
Central Asia and North Africa-Middle East	Girls	1987	17	14.09
Central Asia and North Africa-Middle East	Girls	1988	17	14.25
Central Asia and North Africa-Middle East	Girls	1989	17	14.42
Central Asia and North Africa-Middle East	Girls	1990	17	14.59
Central Asia and North Africa-Middle East	Girls	1991	17	14.76
Central Asia and North Africa-Middle East	Girls	1992	17	14.93
Central Asia and North Africa-Middle East	Girls	1993	17	15.10
Central Asia and North Africa-Middle East	Girls	1994	17	15.27
Central Asia and North Africa-Middle East	Girls	1995	17	15.44
Central Asia and North Africa-Middle East	Girls	1996	17	15.62
Central Asia and North Africa-Middle East	Girls	1997	17	15.79
Central Asia and North Africa-Middle East	Girls	1998	17	15.97
Central Asia and North Africa-Middle East	Girls	1999	17	16.15
Central Asia and North Africa-Middle East	Girls	2000	17	16.33
Central Asia and North Africa-Middle East	Girls	2001	17	16.51
Central Asia and North Africa-Middle East	Girls	2002	17	16.69
Central Asia and North Africa-Middle East	Girls	2003	17	16.88
Central Asia and North Africa-Middle East	Girls	2004	17	17.05
Central Asia and North Africa-Middle East	Girls	2005	17	17.23
Central Asia and North Africa-Middle East	Girls	2006	17	17.40
Central Asia and North Africa-Middle East	Girls	2007	17	17.58
Central Asia and North Africa-Middle East	Girls	2008	17	17.75
Central Asia and North Africa-Middle East	Girls	2009	17	17.92
Central Asia and North Africa-Middle East	Girls	2010	17	18.10
Central Asia and North Africa-Middle East	Girls	2011	17	18.29
Central Asia and North Africa-Middle East	Girls	2012	17	18.48
Central Asia and North Africa-Middle East	Girls	2013	17	18.68

Central Asia and North Africa-Middle East	Girls	2014	17	18.89
Central Asia and North Africa-Middle East	Girls	2015	17	19.10
Central Asia and North Africa-Middle East	Girls	2016	17	19.31
Central Asia and North Africa-Middle East	Girls	1975	18	13.08
Central Asia and North Africa-Middle East	Girls	1976	18	13.21
Central Asia and North Africa-Middle East	Girls	1977	18	13.33
Central Asia and North Africa-Middle East	Girls	1978	18	13.46
Central Asia and North Africa-Middle East	Girls	1979	18	13.59
Central Asia and North Africa-Middle East	Girls	1980	18	13.73
Central Asia and North Africa-Middle East	Girls	1981	18	13.88
Central Asia and North Africa-Middle East	Girls	1982	18	14.03
Central Asia and North Africa-Middle East	Girls	1983	18	14.19
Central Asia and North Africa-Middle East	Girls	1984	18	14.35
Central Asia and North Africa-Middle East	Girls	1985	18	14.53
Central Asia and North Africa-Middle East	Girls	1986	18	14.70
Central Asia and North Africa-Middle East	Girls	1987	18	14.88
Central Asia and North Africa-Middle East	Girls	1988	18	15.06
Central Asia and North Africa-Middle East	Girls	1989	18	15.24
Central Asia and North Africa-Middle East	Girls	1990	18	15.43
Central Asia and North Africa-Middle East	Girls	1991	18	15.61
Central Asia and North Africa-Middle East	Girls	1992	18	15.80
Central Asia and North Africa-Middle East	Girls	1993	18	15.99
Central Asia and North Africa-Middle East	Girls	1994	18	16.18
Central Asia and North Africa-Middle East	Girls	1995	18	16.37
Central Asia and North Africa-Middle East	Girls	1996	18	16.56
Central Asia and North Africa-Middle East	Girls	1997	18	16.75
Central Asia and North Africa-Middle East	Girls	1998	18	16.95
Central Asia and North Africa-Middle East	Girls	1999	18	17.15
Central Asia and North Africa-Middle East	Girls	2000	18	17.34
Central Asia and North Africa-Middle East	Girls	2001	18	17.54
Central Asia and North Africa-Middle East	Girls	2002	18	17.74
Central Asia and North Africa-Middle East	Girls	2003	18	17.94
Central Asia and North Africa-Middle East	Girls	2004	18	18.14
Central Asia and North Africa-Middle East	Girls	2005	18	18.33
Central Asia and North Africa-Middle East	Girls	2006	18	18.52
Central Asia and North Africa-Middle East	Girls	2007	18	18.71
Central Asia and North Africa-Middle East	Girls	2008	18	18.89
Central Asia and North Africa-Middle East	Girls	2009	18	19.08
Central Asia and North Africa-Middle East	Girls	2010	18	19.27
Central Asia and North Africa-Middle East	Girls	2011	18	19.47
Central Asia and North Africa-Middle East	Girls	2012	18	19.68
Central Asia and North Africa-Middle East	Girls	2013	18	19.90
Central Asia and North Africa-Middle East	Girls	2014	18	20.12
Central Asia and North Africa-Middle East	Girls	2015	18	20.35
Central Asia and North Africa-Middle East	Girls	2016	18	20.57
Central Asia and North Africa-Middle East	Girls	1975	Average	10.00
Central Asia and North Africa-Middle East	Girls	1976	Average	10.10
Central Asia and North Africa-Middle East	Girls	1977	Average	10.21
Central Asia and North Africa-Middle East	Girls	1978	Average	10.31
Central Asia and North Africa-Middle East	Girls	1979	Average	10.42
Central Asia and North Africa-Middle East	Girls	1980	Average	10.54
Central Asia and North Africa-Middle East	Girls	1981	Average	10.65

Central Asia and North Africa-Middle East	Girls	1982	Average	10.78
Central Asia and North Africa-Middle East	Girls	1983	Average	10.91
Central Asia and North Africa-Middle East	Girls	1984	Average	11.04
Central Asia and North Africa-Middle East	Girls	1985	Average	11.19
Central Asia and North Africa-Middle East	Girls	1986	Average	11.33
Central Asia and North Africa-Middle East	Girls	1987	Average	11.47
Central Asia and North Africa-Middle East	Girls	1988	Average	11.62
Central Asia and North Africa-Middle East	Girls	1989	Average	11.77
Central Asia and North Africa-Middle East	Girls	1990	Average	11.92
Central Asia and North Africa-Middle East	Girls	1991	Average	12.08
Central Asia and North Africa-Middle East	Girls	1992	Average	12.23
Central Asia and North Africa-Middle East	Girls	1993	Average	12.39
Central Asia and North Africa-Middle East	Girls	1994	Average	12.54
Central Asia and North Africa-Middle East	Girls	1995	Average	12.70
Central Asia and North Africa-Middle East	Girls	1996	Average	12.85
Central Asia and North Africa-Middle East	Girls	1997	Average	13.01
Central Asia and North Africa-Middle East	Girls	1998	Average	13.17
Central Asia and North Africa-Middle East	Girls	1999	Average	13.33
Central Asia and North Africa-Middle East	Girls	2000	Average	13.50
Central Asia and North Africa-Middle East	Girls	2001	Average	13.66
Central Asia and North Africa-Middle East	Girls	2002	Average	13.83
Central Asia and North Africa-Middle East	Girls	2003	Average	13.99
Central Asia and North Africa-Middle East	Girls	2004	Average	14.16
Central Asia and North Africa-Middle East	Girls	2005	Average	14.32
Central Asia and North Africa-Middle East	Girls	2006	Average	14.48
Central Asia and North Africa-Middle East	Girls	2007	Average	14.64
Central Asia and North Africa-Middle East	Girls	2008	Average	14.81
Central Asia and North Africa-Middle East	Girls	2009	Average	14.97
Central Asia and North Africa-Middle East	Girls	2010	Average	15.14
Central Asia and North Africa-Middle East	Girls	2011	Average	15.31
Central Asia and North Africa-Middle East	Girls	2012	Average	15.49
Central Asia and North Africa-Middle East	Girls	2013	Average	15.67
Central Asia and North Africa-Middle East	Girls	2014	Average	15.85
Central Asia and North Africa-Middle East	Girls	2015	Average	16.03
Central Asia and North Africa-Middle East	Girls	2016	Average	16.22
Central Asia and North Africa-Middle East	Girls	2017	Average	16.40
Central Asia and North Africa-Middle East	Girls	2018	Average	16.59
Central Asia and North Africa-Middle East	Girls	2019	Average	16.77
Central Asia and North Africa-Middle East	Girls	2020	Average	16.95
Central Asia and North Africa-Middle East	Girls	2021	Average	17.14
Central Asia and North Africa-Middle East	Girls	2022	Average	17.32
Central Asia and North Africa-Middle East	Girls	2023	Average	17.50
Central Asia and North Africa-Middle East	Girls	2024	Average	17.68
Central Asia and North Africa-Middle East	Girls	2025	Average	17.86
Central Asia and North Africa-Middle East	Girls	2026	Average	18.04
Central Asia and North Africa-Middle East	Girls	2027	Average	18.23
Central Asia and North Africa-Middle East	Girls	2028	Average	18.41
Central Asia and North Africa-Middle East	Girls	2029	Average	18.59
Central Asia and North Africa-Middle East	Girls	2030	Average	18.77
East and South East Asia	Boys	1975	12	6.67
East and South East Asia	Boys	1976	12	6.74
East and South East Asia	Boys	1977	12	6.81

East and South East Asia	Boys	1978	12	6.88
East and South East Asia	Boys	1979	12	6.95
East and South East Asia	Boys	1980	12	7.03
East and South East Asia	Boys	1981	12	7.09
East and South East Asia	Boys	1982	12	7.16
East and South East Asia	Boys	1983	12	7.22
East and South East Asia	Boys	1984	12	7.28
East and South East Asia	Boys	1985	12	7.33
East and South East Asia	Boys	1986	12	7.38
East and South East Asia	Boys	1987	12	7.43
East and South East Asia	Boys	1988	12	7.48
East and South East Asia	Boys	1989	12	7.55
East and South East Asia	Boys	1990	12	7.63
East and South East Asia	Boys	1991	12	7.74
East and South East Asia	Boys	1992	12	7.85
East and South East Asia	Boys	1993	12	7.99
East and South East Asia	Boys	1994	12	8.13
East and South East Asia	Boys	1995	12	8.29
East and South East Asia	Boys	1996	12	8.46
East and South East Asia	Boys	1997	12	8.63
East and South East Asia	Boys	1998	12	8.80
East and South East Asia	Boys	1999	12	8.96
East and South East Asia	Boys	2000	12	9.09
East and South East Asia	Boys	2001	12	9.19
East and South East Asia	Boys	2002	12	9.28
East and South East Asia	Boys	2003	12	9.34
East and South East Asia	Boys	2004	12	9.41
East and South East Asia	Boys	2005	12	9.49
East and South East Asia	Boys	2006	12	9.58
East and South East Asia	Boys	2007	12	9.69
East and South East Asia	Boys	2008	12	9.81
East and South East Asia	Boys	2009	12	9.94
East and South East Asia	Boys	2010	12	10.08
East and South East Asia	Boys	2011	12	10.24
East and South East Asia	Boys	2012	12	10.40
East and South East Asia	Boys	2013	12	10.59
East and South East Asia	Boys	2014	12	10.78
East and South East Asia	Boys	2015	12	11.00
East and South East Asia	Boys	2016	12	11.22
East and South East Asia	Boys	1975	13	8.17
East and South East Asia	Boys	1976	13	8.25
East and South East Asia	Boys	1977	13	8.34
East and South East Asia	Boys	1978	13	8.42
East and South East Asia	Boys	1979	13	8.50
East and South East Asia	Boys	1980	13	8.58
East and South East Asia	Boys	1981	13	8.66
East and South East Asia	Boys	1982	13	8.74
East and South East Asia	Boys	1983	13	8.81
East and South East Asia	Boys	1984	13	8.88
East and South East Asia	Boys	1985	13	8.93
East and South East Asia	Boys	1986	13	8.99
East and South East Asia	Boys	1987	13	9.05

East and South East Asia	Boys	1988	13	9.11
East and South East Asia	Boys	1989	13	9.19
East and South East Asia	Boys	1990	13	9.29
East and South East Asia	Boys	1991	13	9.41
East and South East Asia	Boys	1992	13	9.55
East and South East Asia	Boys	1993	13	9.71
East and South East Asia	Boys	1994	13	9.88
East and South East Asia	Boys	1995	13	10.06
East and South East Asia	Boys	1996	13	10.26
East and South East Asia	Boys	1997	13	10.47
East and South East Asia	Boys	1998	13	10.67
East and South East Asia	Boys	1999	13	10.85
East and South East Asia	Boys	2000	13	11.00
East and South East Asia	Boys	2001	13	11.12
East and South East Asia	Boys	2002	13	11.22
East and South East Asia	Boys	2003	13	11.30
East and South East Asia	Boys	2004	13	11.38
East and South East Asia	Boys	2005	13	11.46
East and South East Asia	Boys	2006	13	11.57
East and South East Asia	Boys	2007	13	11.70
East and South East Asia	Boys	2008	13	11.84
East and South East Asia	Boys	2009	13	11.99
East and South East Asia	Boys	2010	13	12.16
East and South East Asia	Boys	2011	13	12.34
East and South East Asia	Boys	2012	13	12.55
East and South East Asia	Boys	2013	13	12.77
East and South East Asia	Boys	2014	13	13.00
East and South East Asia	Boys	2015	13	13.26
East and South East Asia	Boys	2016	13	13.53
East and South East Asia	Boys	1975	14	9.92
East and South East Asia	Boys	1976	14	10.01
East and South East Asia	Boys	1977	14	10.11
East and South East Asia	Boys	1978	14	10.20
East and South East Asia	Boys	1979	14	10.29
East and South East Asia	Boys	1980	14	10.38
East and South East Asia	Boys	1981	14	10.47
East and South East Asia	Boys	1982	14	10.55
East and South East Asia	Boys	1983	14	10.63
East and South East Asia	Boys	1984	14	10.70
East and South East Asia	Boys	1985	14	10.76
East and South East Asia	Boys	1986	14	10.83
East and South East Asia	Boys	1987	14	10.89
East and South East Asia	Boys	1988	14	10.96
East and South East Asia	Boys	1989	14	11.04
East and South East Asia	Boys	1990	14	11.15
East and South East Asia	Boys	1991	14	11.29
East and South East Asia	Boys	1992	14	11.44
East and South East Asia	Boys	1993	14	11.61
East and South East Asia	Boys	1994	14	11.80
East and South East Asia	Boys	1995	14	12.01
East and South East Asia	Boys	1996	14	12.23
East and South East Asia	Boys	1997	14	12.46

East and South East Asia	Boys	1998	14	12.69
East and South East Asia	Boys	1999	14	12.89
East and South East Asia	Boys	2000	14	13.05
East and South East Asia	Boys	2001	14	13.19
East and South East Asia	Boys	2002	14	13.29
East and South East Asia	Boys	2003	14	13.38
East and South East Asia	Boys	2004	14	13.46
East and South East Asia	Boys	2005	14	13.55
East and South East Asia	Boys	2006	14	13.67
East and South East Asia	Boys	2007	14	13.81
East and South East Asia	Boys	2008	14	13.96
East and South East Asia	Boys	2009	14	14.13
East and South East Asia	Boys	2010	14	14.32
East and South East Asia	Boys	2011	14	14.52
East and South East Asia	Boys	2012	14	14.75
East and South East Asia	Boys	2013	14	15.00
East and South East Asia	Boys	2014	14	15.27
East and South East Asia	Boys	2015	14	15.57
East and South East Asia	Boys	2016	14	15.88
East and South East Asia	Boys	1975	15	11.72
East and South East Asia	Boys	1976	15	11.80
East and South East Asia	Boys	1977	15	11.90
East and South East Asia	Boys	1978	15	12.02
East and South East Asia	Boys	1979	15	12.15
East and South East Asia	Boys	1980	15	12.28
East and South East Asia	Boys	1981	15	12.40
East and South East Asia	Boys	1982	15	12.51
East and South East Asia	Boys	1983	15	12.63
East and South East Asia	Boys	1984	15	12.74
East and South East Asia	Boys	1985	15	12.85
East and South East Asia	Boys	1986	15	12.98
East and South East Asia	Boys	1987	15	13.12
East and South East Asia	Boys	1988	15	13.27
East and South East Asia	Boys	1989	15	13.42
East and South East Asia	Boys	1990	15	13.57
East and South East Asia	Boys	1991	15	13.69
East and South East Asia	Boys	1992	15	13.78
East and South East Asia	Boys	1993	15	13.84
East and South East Asia	Boys	1994	15	13.90
East and South East Asia	Boys	1995	15	13.98
East and South East Asia	Boys	1996	15	14.08
East and South East Asia	Boys	1997	15	14.21
East and South East Asia	Boys	1998	15	14.38
East and South East Asia	Boys	1999	15	14.57
East and South East Asia	Boys	2000	15	14.79
East and South East Asia	Boys	2001	15	15.04
East and South East Asia	Boys	2002	15	15.31
East and South East Asia	Boys	2003	15	15.58
East and South East Asia	Boys	2004	15	15.84
East and South East Asia	Boys	2005	15	16.07
East and South East Asia	Boys	2006	15	16.27
East and South East Asia	Boys	2007	15	16.44

East and South East Asia	Boys	2008	15	16.59
East and South East Asia	Boys	2009	15	16.74
East and South East Asia	Boys	2010	15	16.90
East and South East Asia	Boys	2011	15	17.08
East and South East Asia	Boys	2012	15	17.28
East and South East Asia	Boys	2013	15	17.49
East and South East Asia	Boys	2014	15	17.71
East and South East Asia	Boys	2015	15	17.96
East and South East Asia	Boys	2016	15	18.23
East and South East Asia	Boys	1975	16	13.74
East and South East Asia	Boys	1976	16	13.82
East and South East Asia	Boys	1977	16	13.92
East and South East Asia	Boys	1978	16	14.05
East and South East Asia	Boys	1979	16	14.18
East and South East Asia	Boys	1980	16	14.31
East and South East Asia	Boys	1981	16	14.44
East and South East Asia	Boys	1982	16	14.56
East and South East Asia	Boys	1983	16	14.68
East and South East Asia	Boys	1984	16	14.79
East and South East Asia	Boys	1985	16	14.90
East and South East Asia	Boys	1986	16	15.03
East and South East Asia	Boys	1987	16	15.18
East and South East Asia	Boys	1988	16	15.33
East and South East Asia	Boys	1989	16	15.49
East and South East Asia	Boys	1990	16	15.64
East and South East Asia	Boys	1991	16	15.76
East and South East Asia	Boys	1992	16	15.85
East and South East Asia	Boys	1993	16	15.91
East and South East Asia	Boys	1994	16	15.97
East and South East Asia	Boys	1995	16	16.04
East and South East Asia	Boys	1996	16	16.15
East and South East Asia	Boys	1997	16	16.28
East and South East Asia	Boys	1998	16	16.45
East and South East Asia	Boys	1999	16	16.64
East and South East Asia	Boys	2000	16	16.87
East and South East Asia	Boys	2001	16	17.12
East and South East Asia	Boys	2002	16	17.40
East and South East Asia	Boys	2003	16	17.69
East and South East Asia	Boys	2004	16	17.95
East and South East Asia	Boys	2005	16	18.19
East and South East Asia	Boys	2006	16	18.39
East and South East Asia	Boys	2007	16	18.57
East and South East Asia	Boys	2008	16	18.72
East and South East Asia	Boys	2009	16	18.87
East and South East Asia	Boys	2010	16	19.03
East and South East Asia	Boys	2011	16	19.21
East and South East Asia	Boys	2012	16	19.41
East and South East Asia	Boys	2013	16	19.62
East and South East Asia	Boys	2014	16	19.86
East and South East Asia	Boys	2015	16	20.12
East and South East Asia	Boys	2016	16	20.40
East and South East Asia	Boys	1975	17	15.81

East and South East Asia	Boys	1976	17	15.89
East and South East Asia	Boys	1977	17	16.00
East and South East Asia	Boys	1978	17	16.13
East and South East Asia	Boys	1979	17	16.27
East and South East Asia	Boys	1980	17	16.41
East and South East Asia	Boys	1981	17	16.53
East and South East Asia	Boys	1982	17	16.66
East and South East Asia	Boys	1983	17	16.78
East and South East Asia	Boys	1984	17	16.90
East and South East Asia	Boys	1985	17	17.01
East and South East Asia	Boys	1986	17	17.14
East and South East Asia	Boys	1987	17	17.29
East and South East Asia	Boys	1988	17	17.44
East and South East Asia	Boys	1989	17	17.60
East and South East Asia	Boys	1990	17	17.76
East and South East Asia	Boys	1991	17	17.88
East and South East Asia	Boys	1992	17	17.96
East and South East Asia	Boys	1993	17	18.02
East and South East Asia	Boys	1994	17	18.08
East and South East Asia	Boys	1995	17	18.16
East and South East Asia	Boys	1996	17	18.26
East and South East Asia	Boys	1997	17	18.39
East and South East Asia	Boys	1998	17	18.56
East and South East Asia	Boys	1999	17	18.76
East and South East Asia	Boys	2000	17	18.99
East and South East Asia	Boys	2001	17	19.26
East and South East Asia	Boys	2002	17	19.54
East and South East Asia	Boys	2003	17	19.84
East and South East Asia	Boys	2004	17	20.11
East and South East Asia	Boys	2005	17	20.35
East and South East Asia	Boys	2006	17	20.55
East and South East Asia	Boys	2007	17	20.73
East and South East Asia	Boys	2008	17	20.88
East and South East Asia	Boys	2009	17	21.03
East and South East Asia	Boys	2010	17	21.19
East and South East Asia	Boys	2011	17	21.38
East and South East Asia	Boys	2012	17	21.58
East and South East Asia	Boys	2013	17	21.79
East and South East Asia	Boys	2014	17	22.03
East and South East Asia	Boys	2015	17	22.29
East and South East Asia	Boys	2016	17	22.58
East and South East Asia	Boys	1975	18	17.91
East and South East Asia	Boys	1976	18	18.00
East and South East Asia	Boys	1977	18	18.12
East and South East Asia	Boys	1978	18	18.26
East and South East Asia	Boys	1979	18	18.40
East and South East Asia	Boys	1980	18	18.54
East and South East Asia	Boys	1981	18	18.68
East and South East Asia	Boys	1982	18	18.81
East and South East Asia	Boys	1983	18	18.93
East and South East Asia	Boys	1984	18	19.06
East and South East Asia	Boys	1985	18	19.18

East and South East Asia	Boys	1986	18	19.31
East and South East Asia	Boys	1987	18	19.46
East and South East Asia	Boys	1988	18	19.63
East and South East Asia	Boys	1989	18	19.79
East and South East Asia	Boys	1990	18	19.95
East and South East Asia	Boys	1991	18	20.07
East and South East Asia	Boys	1992	18	20.16
East and South East Asia	Boys	1993	18	20.22
East and South East Asia	Boys	1994	18	20.27
East and South East Asia	Boys	1995	18	20.35
East and South East Asia	Boys	1996	18	20.46
East and South East Asia	Boys	1997	18	20.60
East and South East Asia	Boys	1998	18	20.77
East and South East Asia	Boys	1999	18	20.98
East and South East Asia	Boys	2000	18	21.22
East and South East Asia	Boys	2001	18	21.49
East and South East Asia	Boys	2002	18	21.80
East and South East Asia	Boys	2003	18	22.10
East and South East Asia	Boys	2004	18	22.38
East and South East Asia	Boys	2005	18	22.63
East and South East Asia	Boys	2006	18	22.84
East and South East Asia	Boys	2007	18	23.02
East and South East Asia	Boys	2008	18	23.18
East and South East Asia	Boys	2009	18	23.33
East and South East Asia	Boys	2010	18	23.50
East and South East Asia	Boys	2011	18	23.69
East and South East Asia	Boys	2012	18	23.89
East and South East Asia	Boys	2013	18	24.12
East and South East Asia	Boys	2014	18	24.36
East and South East Asia	Boys	2015	18	24.63
East and South East Asia	Boys	2016	18	24.93
East and South East Asia	Boys	1975	Average	11.99
East and South East Asia	Boys	1976	Average	12.07
East and South East Asia	Boys	1977	Average	12.17
East and South East Asia	Boys	1978	Average	12.28
East and South East Asia	Boys	1979	Average	12.39
East and South East Asia	Boys	1980	Average	12.50
East and South East Asia	Boys	1981	Average	12.61
East and South East Asia	Boys	1982	Average	12.71
East and South East Asia	Boys	1983	Average	12.81
East and South East Asia	Boys	1984	Average	12.91
East and South East Asia	Boys	1985	Average	12.99
East and South East Asia	Boys	1986	Average	13.09
East and South East Asia	Boys	1987	Average	13.20
East and South East Asia	Boys	1988	Average	13.32
East and South East Asia	Boys	1989	Average	13.44
East and South East Asia	Boys	1990	Average	13.57
East and South East Asia	Boys	1991	Average	13.69
East and South East Asia	Boys	1992	Average	13.80
East and South East Asia	Boys	1993	Average	13.90
East and South East Asia	Boys	1994	Average	14.01
East and South East Asia	Boys	1995	Average	14.13

East and South East Asia	Boys	1996	Average	14.27
East and South East Asia	Boys	1997	Average	14.43
East and South East Asia	Boys	1998	Average	14.62
East and South East Asia	Boys	1999	Average	14.81
East and South East Asia	Boys	2000	Average	15.00
East and South East Asia	Boys	2001	Average	15.20
East and South East Asia	Boys	2002	Average	15.41
East and South East Asia	Boys	2003	Average	15.60
East and South East Asia	Boys	2004	Average	15.79
East and South East Asia	Boys	2005	Average	15.96
East and South East Asia	Boys	2006	Average	16.13
East and South East Asia	Boys	2007	Average	16.28
East and South East Asia	Boys	2008	Average	16.43
East and South East Asia	Boys	2009	Average	16.58
East and South East Asia	Boys	2010	Average	16.74
East and South East Asia	Boys	2011	Average	16.92
East and South East Asia	Boys	2012	Average	17.12
East and South East Asia	Boys	2013	Average	17.34
East and South East Asia	Boys	2014	Average	17.58
East and South East Asia	Boys	2015	Average	17.83
East and South East Asia	Boys	2016	Average	18.11
East and South East Asia	Boys	2017	Average	18.41
East and South East Asia	Boys	2018	Average	18.72
East and South East Asia	Boys	2019	Average	19.04
East and South East Asia	Boys	2020	Average	19.36
East and South East Asia	Boys	2021	Average	19.66
East and South East Asia	Boys	2022	Average	19.95
East and South East Asia	Boys	2023	Average	20.22
East and South East Asia	Boys	2024	Average	20.47
East and South East Asia	Boys	2025	Average	20.71
East and South East Asia	Boys	2026	Average	20.94
East and South East Asia	Boys	2027	Average	21.16
East and South East Asia	Boys	2028	Average	21.37
East and South East Asia	Boys	2029	Average	21.59
East and South East Asia	Boys	2030	Average	21.82
East and South East Asia	Girls	1975	12	6.06
East and South East Asia	Girls	1976	12	6.10
East and South East Asia	Girls	1977	12	6.13
East and South East Asia	Girls	1978	12	6.17
East and South East Asia	Girls	1979	12	6.20
East and South East Asia	Girls	1980	12	6.23
East and South East Asia	Girls	1981	12	6.26
East and South East Asia	Girls	1982	12	6.29
East and South East Asia	Girls	1983	12	6.31
East and South East Asia	Girls	1984	12	6.34
East and South East Asia	Girls	1985	12	6.35
East and South East Asia	Girls	1986	12	6.37
East and South East Asia	Girls	1987	12	6.39
East and South East Asia	Girls	1988	12	6.41
East and South East Asia	Girls	1989	12	6.44
East and South East Asia	Girls	1990	12	6.48
East and South East Asia	Girls	1991	12	6.53

East and South East Asia	Girls	1992	12	6.58
East and South East Asia	Girls	1993	12	6.64
East and South East Asia	Girls	1994	12	6.70
East and South East Asia	Girls	1995	12	6.77
East and South East Asia	Girls	1996	12	6.84
East and South East Asia	Girls	1997	12	6.92
East and South East Asia	Girls	1998	12	6.99
East and South East Asia	Girls	1999	12	7.05
East and South East Asia	Girls	2000	12	7.10
East and South East Asia	Girls	2001	12	7.14
East and South East Asia	Girls	2002	12	7.17
East and South East Asia	Girls	2003	12	7.19
East and South East Asia	Girls	2004	12	7.21
East and South East Asia	Girls	2005	12	7.24
East and South East Asia	Girls	2006	12	7.28
East and South East Asia	Girls	2007	12	7.33
East and South East Asia	Girls	2008	12	7.38
East and South East Asia	Girls	2009	12	7.44
East and South East Asia	Girls	2010	12	7.50
East and South East Asia	Girls	2011	12	7.57
East and South East Asia	Girls	2012	12	7.65
East and South East Asia	Girls	2013	12	7.74
East and South East Asia	Girls	2014	12	7.83
East and South East Asia	Girls	2015	12	7.93
East and South East Asia	Girls	2016	12	8.03
East and South East Asia	Girls	1975	13	7.50
East and South East Asia	Girls	1976	13	7.54
East and South East Asia	Girls	1977	13	7.58
East and South East Asia	Girls	1978	13	7.62
East and South East Asia	Girls	1979	13	7.66
East and South East Asia	Girls	1980	13	7.69
East and South East Asia	Girls	1981	13	7.73
East and South East Asia	Girls	1982	13	7.76
East and South East Asia	Girls	1983	13	7.79
East and South East Asia	Girls	1984	13	7.82
East and South East Asia	Girls	1985	13	7.83
East and South East Asia	Girls	1986	13	7.86
East and South East Asia	Girls	1987	13	7.88
East and South East Asia	Girls	1988	13	7.90
East and South East Asia	Girls	1989	13	7.93
East and South East Asia	Girls	1990	13	7.98
East and South East Asia	Girls	1991	13	8.03
East and South East Asia	Girls	1992	13	8.09
East and South East Asia	Girls	1993	13	8.16
East and South East Asia	Girls	1994	13	8.23
East and South East Asia	Girls	1995	13	8.31
East and South East Asia	Girls	1996	13	8.40
East and South East Asia	Girls	1997	13	8.48
East and South East Asia	Girls	1998	13	8.56
East and South East Asia	Girls	1999	13	8.63
East and South East Asia	Girls	2000	13	8.69
East and South East Asia	Girls	2001	13	8.73

East and South East Asia	Girls	2002	13	8.76
East and South East Asia	Girls	2003	13	8.79
East and South East Asia	Girls	2004	13	8.81
East and South East Asia	Girls	2005	13	8.85
East and South East Asia	Girls	2006	13	8.89
East and South East Asia	Girls	2007	13	8.94
East and South East Asia	Girls	2008	13	9.00
East and South East Asia	Girls	2009	13	9.07
East and South East Asia	Girls	2010	13	9.14
East and South East Asia	Girls	2011	13	9.22
East and South East Asia	Girls	2012	13	9.31
East and South East Asia	Girls	2013	13	9.40
East and South East Asia	Girls	2014	13	9.51
East and South East Asia	Girls	2015	13	9.62
East and South East Asia	Girls	2016	13	9.74
East and South East Asia	Girls	1975	14	9.02
East and South East Asia	Girls	1976	14	9.07
East and South East Asia	Girls	1977	14	9.11
East and South East Asia	Girls	1978	14	9.16
East and South East Asia	Girls	1979	14	9.20
East and South East Asia	Girls	1980	14	9.24
East and South East Asia	Girls	1981	14	9.28
East and South East Asia	Girls	1982	14	9.31
East and South East Asia	Girls	1983	14	9.35
East and South East Asia	Girls	1984	14	9.37
East and South East Asia	Girls	1985	14	9.39
East and South East Asia	Girls	1986	14	9.42
East and South East Asia	Girls	1987	14	9.44
East and South East Asia	Girls	1988	14	9.47
East and South East Asia	Girls	1989	14	9.50
East and South East Asia	Girls	1990	14	9.55
East and South East Asia	Girls	1991	14	9.61
East and South East Asia	Girls	1992	14	9.67
East and South East Asia	Girls	1993	14	9.75
East and South East Asia	Girls	1994	14	9.83
East and South East Asia	Girls	1995	14	9.91
East and South East Asia	Girls	1996	14	10.01
East and South East Asia	Girls	1997	14	10.10
East and South East Asia	Girls	1998	14	10.19
East and South East Asia	Girls	1999	14	10.26
East and South East Asia	Girls	2000	14	10.32
East and South East Asia	Girls	2001	14	10.37
East and South East Asia	Girls	2002	14	10.40
East and South East Asia	Girls	2003	14	10.43
East and South East Asia	Girls	2004	14	10.46
East and South East Asia	Girls	2005	14	10.49
East and South East Asia	Girls	2006	14	10.54
East and South East Asia	Girls	2007	14	10.59
East and South East Asia	Girls	2008	14	10.65
East and South East Asia	Girls	2009	14	10.73
East and South East Asia	Girls	2010	14	10.80
East and South East Asia	Girls	2011	14	10.89

East and South East Asia	Girls	2012	14	10.98
East and South East Asia	Girls	2013	14	11.09
East and South East Asia	Girls	2014	14	11.20
East and South East Asia	Girls	2015	14	11.32
East and South East Asia	Girls	2016	14	11.45
East and South East Asia	Girls	1975	15	10.36
East and South East Asia	Girls	1976	15	10.40
East and South East Asia	Girls	1977	15	10.45
East and South East Asia	Girls	1978	15	10.51
East and South East Asia	Girls	1979	15	10.58
East and South East Asia	Girls	1980	15	10.64
East and South East Asia	Girls	1981	15	10.69
East and South East Asia	Girls	1982	15	10.74
East and South East Asia	Girls	1983	15	10.80
East and South East Asia	Girls	1984	15	10.84
East and South East Asia	Girls	1985	15	10.89
East and South East Asia	Girls	1986	15	10.94
East and South East Asia	Girls	1987	15	11.01
East and South East Asia	Girls	1988	15	11.07
East and South East Asia	Girls	1989	15	11.14
East and South East Asia	Girls	1990	15	11.20
East and South East Asia	Girls	1991	15	11.25
East and South East Asia	Girls	1992	15	11.28
East and South East Asia	Girls	1993	15	11.30
East and South East Asia	Girls	1994	15	11.32
East and South East Asia	Girls	1995	15	11.35
East and South East Asia	Girls	1996	15	11.39
East and South East Asia	Girls	1997	15	11.43
East and South East Asia	Girls	1998	15	11.50
East and South East Asia	Girls	1999	15	11.57
East and South East Asia	Girls	2000	15	11.65
East and South East Asia	Girls	2001	15	11.75
East and South East Asia	Girls	2002	15	11.85
East and South East Asia	Girls	2003	15	11.96
East and South East Asia	Girls	2004	15	12.05
East and South East Asia	Girls	2005	15	12.14
East and South East Asia	Girls	2006	15	12.21
East and South East Asia	Girls	2007	15	12.27
East and South East Asia	Girls	2008	15	12.32
East and South East Asia	Girls	2009	15	12.38
East and South East Asia	Girls	2010	15	12.44
East and South East Asia	Girls	2011	15	12.51
East and South East Asia	Girls	2012	15	12.60
East and South East Asia	Girls	2013	15	12.69
East and South East Asia	Girls	2014	15	12.79
East and South East Asia	Girls	2015	15	12.89
East and South East Asia	Girls	2016	15	13.01
East and South East Asia	Girls	1975	16	11.50
East and South East Asia	Girls	1976	16	11.54
East and South East Asia	Girls	1977	16	11.59
East and South East Asia	Girls	1978	16	11.65
East and South East Asia	Girls	1979	16	11.72

East and South East Asia	Girls	1980	16	11.78
East and South East Asia	Girls	1981	16	11.84
East and South East Asia	Girls	1982	16	11.89
East and South East Asia	Girls	1983	16	11.95
East and South East Asia	Girls	1984	16	11.99
East and South East Asia	Girls	1985	16	12.04
East and South East Asia	Girls	1986	16	12.10
East and South East Asia	Girls	1987	16	12.16
East and South East Asia	Girls	1988	16	12.23
East and South East Asia	Girls	1989	16	12.30
East and South East Asia	Girls	1990	16	12.37
East and South East Asia	Girls	1991	16	12.42
East and South East Asia	Girls	1992	16	12.45
East and South East Asia	Girls	1993	16	12.47
East and South East Asia	Girls	1994	16	12.49
East and South East Asia	Girls	1995	16	12.52
East and South East Asia	Girls	1996	16	12.56
East and South East Asia	Girls	1997	16	12.61
East and South East Asia	Girls	1998	16	12.67
East and South East Asia	Girls	1999	16	12.75
East and South East Asia	Girls	2000	16	12.83
East and South East Asia	Girls	2001	16	12.93
East and South East Asia	Girls	2002	16	13.04
East and South East Asia	Girls	2003	16	13.15
East and South East Asia	Girls	2004	16	13.24
East and South East Asia	Girls	2005	16	13.33
East and South East Asia	Girls	2006	16	13.40
East and South East Asia	Girls	2007	16	13.47
East and South East Asia	Girls	2008	16	13.52
East and South East Asia	Girls	2009	16	13.58
East and South East Asia	Girls	2010	16	13.65
East and South East Asia	Girls	2011	16	13.72
East and South East Asia	Girls	2012	16	13.80
East and South East Asia	Girls	2013	16	13.90
East and South East Asia	Girls	2014	16	14.00
East and South East Asia	Girls	2015	16	14.11
East and South East Asia	Girls	2016	16	14.23
East and South East Asia	Girls	1975	17	12.33
East and South East Asia	Girls	1976	17	12.37
East and South East Asia	Girls	1977	17	12.42
East and South East Asia	Girls	1978	17	12.49
East and South East Asia	Girls	1979	17	12.56
East and South East Asia	Girls	1980	17	12.62
East and South East Asia	Girls	1981	17	12.68
East and South East Asia	Girls	1982	17	12.74
East and South East Asia	Girls	1983	17	12.80
East and South East Asia	Girls	1984	17	12.85
East and South East Asia	Girls	1985	17	12.90
East and South East Asia	Girls	1986	17	12.96
East and South East Asia	Girls	1987	17	13.02
East and South East Asia	Girls	1988	17	13.10
East and South East Asia	Girls	1989	17	13.17

East and South East Asia	Girls	1990	17	13.24
East and South East Asia	Girls	1991	17	13.29
East and South East Asia	Girls	1992	17	13.33
East and South East Asia	Girls	1993	17	13.35
East and South East Asia	Girls	1994	17	13.37
East and South East Asia	Girls	1995	17	13.40
East and South East Asia	Girls	1996	17	13.44
East and South East Asia	Girls	1997	17	13.49
East and South East Asia	Girls	1998	17	13.56
East and South East Asia	Girls	1999	17	13.64
East and South East Asia	Girls	2000	17	13.73
East and South East Asia	Girls	2001	17	13.83
East and South East Asia	Girls	2002	17	13.95
East and South East Asia	Girls	2003	17	14.06
East and South East Asia	Girls	2004	17	14.16
East and South East Asia	Girls	2005	17	14.25
East and South East Asia	Girls	2006	17	14.33
East and South East Asia	Girls	2007	17	14.40
East and South East Asia	Girls	2008	17	14.45
East and South East Asia	Girls	2009	17	14.52
East and South East Asia	Girls	2010	17	14.58
East and South East Asia	Girls	2011	17	14.66
East and South East Asia	Girls	2012	17	14.75
East and South East Asia	Girls	2013	17	14.85
East and South East Asia	Girls	2014	17	14.96
East and South East Asia	Girls	2015	17	15.07
East and South East Asia	Girls	2016	17	15.20
East and South East Asia	Girls	1975	18	12.95
East and South East Asia	Girls	1976	18	13.00
East and South East Asia	Girls	1977	18	13.06
East and South East Asia	Girls	1978	18	13.13
East and South East Asia	Girls	1979	18	13.20
East and South East Asia	Girls	1980	18	13.27
East and South East Asia	Girls	1981	18	13.34
East and South East Asia	Girls	1982	18	13.40
East and South East Asia	Girls	1983	18	13.46
East and South East Asia	Girls	1984	18	13.52
East and South East Asia	Girls	1985	18	13.57
East and South East Asia	Girls	1986	18	13.63
East and South East Asia	Girls	1987	18	13.71
East and South East Asia	Girls	1988	18	13.79
East and South East Asia	Girls	1989	18	13.87
East and South East Asia	Girls	1990	18	13.94
East and South East Asia	Girls	1991	18	14.00
East and South East Asia	Girls	1992	18	14.04
East and South East Asia	Girls	1993	18	14.07
East and South East Asia	Girls	1994	18	14.09
East and South East Asia	Girls	1995	18	14.12
East and South East Asia	Girls	1996	18	14.16
East and South East Asia	Girls	1997	18	14.22
East and South East Asia	Girls	1998	18	14.30
East and South East Asia	Girls	1999	18	14.38

East and South East Asia	Girls	2000	18	14.48
East and South East Asia	Girls	2001	18	14.60
East and South East Asia	Girls	2002	18	14.72
East and South East Asia	Girls	2003	18	14.84
East and South East Asia	Girls	2004	18	14.96
East and South East Asia	Girls	2005	18	15.05
East and South East Asia	Girls	2006	18	15.14
East and South East Asia	Girls	2007	18	15.21
East and South East Asia	Girls	2008	18	15.28
East and South East Asia	Girls	2009	18	15.35
East and South East Asia	Girls	2010	18	15.42
East and South East Asia	Girls	2011	18	15.51
East and South East Asia	Girls	2012	18	15.61
East and South East Asia	Girls	2013	18	15.71
East and South East Asia	Girls	2014	18	15.83
East and South East Asia	Girls	2015	18	15.96
East and South East Asia	Girls	2016	18	16.09
East and South East Asia	Girls	1975	Average	9.96
East and South East Asia	Girls	1976	Average	10.00
East and South East Asia	Girls	1977	Average	10.05
East and South East Asia	Girls	1978	Average	10.10
East and South East Asia	Girls	1979	Average	10.16
East and South East Asia	Girls	1980	Average	10.21
East and South East Asia	Girls	1981	Average	10.26
East and South East Asia	Girls	1982	Average	10.31
East and South East Asia	Girls	1983	Average	10.35
East and South East Asia	Girls	1984	Average	10.39
East and South East Asia	Girls	1985	Average	10.43
East and South East Asia	Girls	1986	Average	10.47
East and South East Asia	Girls	1987	Average	10.52
East and South East Asia	Girls	1988	Average	10.57
East and South East Asia	Girls	1989	Average	10.62
East and South East Asia	Girls	1990	Average	10.68
East and South East Asia	Girls	1991	Average	10.73
East and South East Asia	Girls	1992	Average	10.78
East and South East Asia	Girls	1993	Average	10.82
East and South East Asia	Girls	1994	Average	10.86
East and South East Asia	Girls	1995	Average	10.91
East and South East Asia	Girls	1996	Average	10.97
East and South East Asia	Girls	1997	Average	11.04
East and South East Asia	Girls	1998	Average	11.11
East and South East Asia	Girls	1999	Average	11.18
East and South East Asia	Girls	2000	Average	11.26
East and South East Asia	Girls	2001	Average	11.34
East and South East Asia	Girls	2002	Average	11.41
East and South East Asia	Girls	2003	Average	11.49
East and South East Asia	Girls	2004	Average	11.56
East and South East Asia	Girls	2005	Average	11.62
East and South East Asia	Girls	2006	Average	11.68
East and South East Asia	Girls	2007	Average	11.74
East and South East Asia	Girls	2008	Average	11.80
East and South East Asia	Girls	2009	Average	11.86

East and South East Asia	Girls	2010	Average	11.93
East and South East Asia	Girls	2011	Average	12.01
East and South East Asia	Girls	2012	Average	12.10
East and South East Asia	Girls	2013	Average	12.20
East and South East Asia	Girls	2014	Average	12.30
East and South East Asia	Girls	2015	Average	12.41
East and South East Asia	Girls	2016	Average	12.54
East and South East Asia	Girls	2017	Average	12.66
East and South East Asia	Girls	2018	Average	12.80
East and South East Asia	Girls	2019	Average	12.94
East and South East Asia	Girls	2020	Average	13.07
East and South East Asia	Girls	2021	Average	13.20
East and South East Asia	Girls	2022	Average	13.33
East and South East Asia	Girls	2023	Average	13.45
East and South East Asia	Girls	2024	Average	13.57
East and South East Asia	Girls	2025	Average	13.68
East and South East Asia	Girls	2026	Average	13.79
East and South East Asia	Girls	2027	Average	13.90
East and South East Asia	Girls	2028	Average	14.01
East and South East Asia	Girls	2029	Average	14.11
East and South East Asia	Girls	2030	Average	14.22
High-income Asia Pacific	Boys	1975	12	6.99
High-income Asia Pacific	Boys	1976	12	7.11
High-income Asia Pacific	Boys	1977	12	7.23
High-income Asia Pacific	Boys	1978	12	7.35
High-income Asia Pacific	Boys	1979	12	7.47
High-income Asia Pacific	Boys	1980	12	7.60
High-income Asia Pacific	Boys	1981	12	7.72
High-income Asia Pacific	Boys	1982	12	7.85
High-income Asia Pacific	Boys	1983	12	7.98
High-income Asia Pacific	Boys	1984	12	8.10
High-income Asia Pacific	Boys	1985	12	8.23
High-income Asia Pacific	Boys	1986	12	8.35
High-income Asia Pacific	Boys	1987	12	8.48
High-income Asia Pacific	Boys	1988	12	8.60
High-income Asia Pacific	Boys	1989	12	8.73
High-income Asia Pacific	Boys	1990	12	8.85
High-income Asia Pacific	Boys	1991	12	8.97
High-income Asia Pacific	Boys	1992	12	9.09
High-income Asia Pacific	Boys	1993	12	9.19
High-income Asia Pacific	Boys	1994	12	9.29
High-income Asia Pacific	Boys	1995	12	9.39
High-income Asia Pacific	Boys	1996	12	9.47
High-income Asia Pacific	Boys	1997	12	9.54
High-income Asia Pacific	Boys	1998	12	9.60
High-income Asia Pacific	Boys	1999	12	9.65
High-income Asia Pacific	Boys	2000	12	9.70
High-income Asia Pacific	Boys	2001	12	9.77
High-income Asia Pacific	Boys	2002	12	9.86
High-income Asia Pacific	Boys	2003	12	9.95
High-income Asia Pacific	Boys	2004	12	10.05
High-income Asia Pacific	Boys	2005	12	10.14

High-income Asia Pacific	Boys	2006	12	10.22
High-income Asia Pacific	Boys	2007	12	10.29
High-income Asia Pacific	Boys	2008	12	10.35
High-income Asia Pacific	Boys	2009	12	10.39
High-income Asia Pacific	Boys	2010	12	10.43
High-income Asia Pacific	Boys	2011	12	10.45
High-income Asia Pacific	Boys	2012	12	10.46
High-income Asia Pacific	Boys	2013	12	10.46
High-income Asia Pacific	Boys	2014	12	10.46
High-income Asia Pacific	Boys	2015	12	10.46
High-income Asia Pacific	Boys	2016	12	10.47
High-income Asia Pacific	Boys	1975	13	8.58
High-income Asia Pacific	Boys	1976	13	8.72
High-income Asia Pacific	Boys	1977	13	8.86
High-income Asia Pacific	Boys	1978	13	9.00
High-income Asia Pacific	Boys	1979	13	9.14
High-income Asia Pacific	Boys	1980	13	9.28
High-income Asia Pacific	Boys	1981	13	9.43
High-income Asia Pacific	Boys	1982	13	9.57
High-income Asia Pacific	Boys	1983	13	9.72
High-income Asia Pacific	Boys	1984	13	9.86
High-income Asia Pacific	Boys	1985	13	10.01
High-income Asia Pacific	Boys	1986	13	10.15
High-income Asia Pacific	Boys	1987	13	10.30
High-income Asia Pacific	Boys	1988	13	10.44
High-income Asia Pacific	Boys	1989	13	10.59
High-income Asia Pacific	Boys	1990	13	10.73
High-income Asia Pacific	Boys	1991	13	10.87
High-income Asia Pacific	Boys	1992	13	11.01
High-income Asia Pacific	Boys	1993	13	11.13
High-income Asia Pacific	Boys	1994	13	11.25
High-income Asia Pacific	Boys	1995	13	11.36
High-income Asia Pacific	Boys	1996	13	11.45
High-income Asia Pacific	Boys	1997	13	11.54
High-income Asia Pacific	Boys	1998	13	11.61
High-income Asia Pacific	Boys	1999	13	11.67
High-income Asia Pacific	Boys	2000	13	11.73
High-income Asia Pacific	Boys	2001	13	11.82
High-income Asia Pacific	Boys	2002	13	11.92
High-income Asia Pacific	Boys	2003	13	12.03
High-income Asia Pacific	Boys	2004	13	12.14
High-income Asia Pacific	Boys	2005	13	12.25
High-income Asia Pacific	Boys	2006	13	12.35
High-income Asia Pacific	Boys	2007	13	12.43
High-income Asia Pacific	Boys	2008	13	12.50
High-income Asia Pacific	Boys	2009	13	12.55
High-income Asia Pacific	Boys	2010	13	12.59
High-income Asia Pacific	Boys	2011	13	12.62
High-income Asia Pacific	Boys	2012	13	12.64
High-income Asia Pacific	Boys	2013	13	12.64
High-income Asia Pacific	Boys	2014	13	12.64
High-income Asia Pacific	Boys	2015	13	12.64

High-income Asia Pacific	Boys	2016	13	12.64
High-income Asia Pacific	Boys	1975	14	10.47
High-income Asia Pacific	Boys	1976	14	10.62
High-income Asia Pacific	Boys	1977	14	10.77
High-income Asia Pacific	Boys	1978	14	10.92
High-income Asia Pacific	Boys	1979	14	11.07
High-income Asia Pacific	Boys	1980	14	11.22
High-income Asia Pacific	Boys	1981	14	11.38
High-income Asia Pacific	Boys	1982	14	11.54
High-income Asia Pacific	Boys	1983	14	11.70
High-income Asia Pacific	Boys	1984	14	11.86
High-income Asia Pacific	Boys	1985	14	12.01
High-income Asia Pacific	Boys	1986	14	12.17
High-income Asia Pacific	Boys	1987	14	12.33
High-income Asia Pacific	Boys	1988	14	12.49
High-income Asia Pacific	Boys	1989	14	12.65
High-income Asia Pacific	Boys	1990	14	12.81
High-income Asia Pacific	Boys	1991	14	12.96
High-income Asia Pacific	Boys	1992	14	13.10
High-income Asia Pacific	Boys	1993	14	13.24
High-income Asia Pacific	Boys	1994	14	13.37
High-income Asia Pacific	Boys	1995	14	13.49
High-income Asia Pacific	Boys	1996	14	13.60
High-income Asia Pacific	Boys	1997	14	13.69
High-income Asia Pacific	Boys	1998	14	13.77
High-income Asia Pacific	Boys	1999	14	13.84
High-income Asia Pacific	Boys	2000	14	13.91
High-income Asia Pacific	Boys	2001	14	14.00
High-income Asia Pacific	Boys	2002	14	14.11
High-income Asia Pacific	Boys	2003	14	14.23
High-income Asia Pacific	Boys	2004	14	14.36
High-income Asia Pacific	Boys	2005	14	14.48
High-income Asia Pacific	Boys	2006	14	14.59
High-income Asia Pacific	Boys	2007	14	14.68
High-income Asia Pacific	Boys	2008	14	14.76
High-income Asia Pacific	Boys	2009	14	14.82
High-income Asia Pacific	Boys	2010	14	14.87
High-income Asia Pacific	Boys	2011	14	14.90
High-income Asia Pacific	Boys	2012	14	14.91
High-income Asia Pacific	Boys	2013	14	14.92
High-income Asia Pacific	Boys	2014	14	14.91
High-income Asia Pacific	Boys	2015	14	14.92
High-income Asia Pacific	Boys	2016	14	14.92
High-income Asia Pacific	Boys	1975	15	12.56
High-income Asia Pacific	Boys	1976	15	12.71
High-income Asia Pacific	Boys	1977	15	12.86
High-income Asia Pacific	Boys	1978	15	13.02
High-income Asia Pacific	Boys	1979	15	13.18
High-income Asia Pacific	Boys	1980	15	13.34
High-income Asia Pacific	Boys	1981	15	13.50
High-income Asia Pacific	Boys	1982	15	13.66
High-income Asia Pacific	Boys	1983	15	13.81

High-income Asia Pacific	Boys	1984	15	13.98
High-income Asia Pacific	Boys	1985	15	14.14
High-income Asia Pacific	Boys	1986	15	14.30
High-income Asia Pacific	Boys	1987	15	14.47
High-income Asia Pacific	Boys	1988	15	14.63
High-income Asia Pacific	Boys	1989	15	14.79
High-income Asia Pacific	Boys	1990	15	14.95
High-income Asia Pacific	Boys	1991	15	15.11
High-income Asia Pacific	Boys	1992	15	15.25
High-income Asia Pacific	Boys	1993	15	15.39
High-income Asia Pacific	Boys	1994	15	15.51
High-income Asia Pacific	Boys	1995	15	15.64
High-income Asia Pacific	Boys	1996	15	15.75
High-income Asia Pacific	Boys	1997	15	15.86
High-income Asia Pacific	Boys	1998	15	15.95
High-income Asia Pacific	Boys	1999	15	16.04
High-income Asia Pacific	Boys	2000	15	16.12
High-income Asia Pacific	Boys	2001	15	16.21
High-income Asia Pacific	Boys	2002	15	16.29
High-income Asia Pacific	Boys	2003	15	16.37
High-income Asia Pacific	Boys	2004	15	16.46
High-income Asia Pacific	Boys	2005	15	16.57
High-income Asia Pacific	Boys	2006	15	16.68
High-income Asia Pacific	Boys	2007	15	16.80
High-income Asia Pacific	Boys	2008	15	16.92
High-income Asia Pacific	Boys	2009	15	17.04
High-income Asia Pacific	Boys	2010	15	17.14
High-income Asia Pacific	Boys	2011	15	17.23
High-income Asia Pacific	Boys	2012	15	17.30
High-income Asia Pacific	Boys	2013	15	17.36
High-income Asia Pacific	Boys	2014	15	17.40
High-income Asia Pacific	Boys	2015	15	17.43
High-income Asia Pacific	Boys	2016	15	17.44
High-income Asia Pacific	Boys	1975	16	14.75
High-income Asia Pacific	Boys	1976	16	14.90
High-income Asia Pacific	Boys	1977	16	15.05
High-income Asia Pacific	Boys	1978	16	15.21
High-income Asia Pacific	Boys	1979	16	15.37
High-income Asia Pacific	Boys	1980	16	15.53
High-income Asia Pacific	Boys	1981	16	15.69
High-income Asia Pacific	Boys	1982	16	15.84
High-income Asia Pacific	Boys	1983	16	16.00
High-income Asia Pacific	Boys	1984	16	16.16
High-income Asia Pacific	Boys	1985	16	16.32
High-income Asia Pacific	Boys	1986	16	16.49
High-income Asia Pacific	Boys	1987	16	16.65
High-income Asia Pacific	Boys	1988	16	16.81
High-income Asia Pacific	Boys	1989	16	16.98
High-income Asia Pacific	Boys	1990	16	17.14
High-income Asia Pacific	Boys	1991	16	17.29
High-income Asia Pacific	Boys	1992	16	17.43
High-income Asia Pacific	Boys	1993	16	17.57

High-income Asia Pacific	Boys	1994	16	17.70
High-income Asia Pacific	Boys	1995	16	17.82
High-income Asia Pacific	Boys	1996	16	17.94
High-income Asia Pacific	Boys	1997	16	18.04
High-income Asia Pacific	Boys	1998	16	18.14
High-income Asia Pacific	Boys	1999	16	18.23
High-income Asia Pacific	Boys	2000	16	18.31
High-income Asia Pacific	Boys	2001	16	18.40
High-income Asia Pacific	Boys	2002	16	18.48
High-income Asia Pacific	Boys	2003	16	18.56
High-income Asia Pacific	Boys	2004	16	18.66
High-income Asia Pacific	Boys	2005	16	18.76
High-income Asia Pacific	Boys	2006	16	18.87
High-income Asia Pacific	Boys	2007	16	18.99
High-income Asia Pacific	Boys	2008	16	19.12
High-income Asia Pacific	Boys	2009	16	19.24
High-income Asia Pacific	Boys	2010	16	19.35
High-income Asia Pacific	Boys	2011	16	19.44
High-income Asia Pacific	Boys	2012	16	19.51
High-income Asia Pacific	Boys	2013	16	19.57
High-income Asia Pacific	Boys	2014	16	19.61
High-income Asia Pacific	Boys	2015	16	19.64
High-income Asia Pacific	Boys	2016	16	19.65
High-income Asia Pacific	Boys	1975	17	16.96
High-income Asia Pacific	Boys	1976	17	17.11
High-income Asia Pacific	Boys	1977	17	17.27
High-income Asia Pacific	Boys	1978	17	17.43
High-income Asia Pacific	Boys	1979	17	17.58
High-income Asia Pacific	Boys	1980	17	17.74
High-income Asia Pacific	Boys	1981	17	17.90
High-income Asia Pacific	Boys	1982	17	18.05
High-income Asia Pacific	Boys	1983	17	18.21
High-income Asia Pacific	Boys	1984	17	18.37
High-income Asia Pacific	Boys	1985	17	18.53
High-income Asia Pacific	Boys	1986	17	18.69
High-income Asia Pacific	Boys	1987	17	18.85
High-income Asia Pacific	Boys	1988	17	19.01
High-income Asia Pacific	Boys	1989	17	19.17
High-income Asia Pacific	Boys	1990	17	19.33
High-income Asia Pacific	Boys	1991	17	19.48
High-income Asia Pacific	Boys	1992	17	19.62
High-income Asia Pacific	Boys	1993	17	19.76
High-income Asia Pacific	Boys	1994	17	19.89
High-income Asia Pacific	Boys	1995	17	20.01
High-income Asia Pacific	Boys	1996	17	20.12
High-income Asia Pacific	Boys	1997	17	20.23
High-income Asia Pacific	Boys	1998	17	20.33
High-income Asia Pacific	Boys	1999	17	20.42
High-income Asia Pacific	Boys	2000	17	20.50
High-income Asia Pacific	Boys	2001	17	20.58
High-income Asia Pacific	Boys	2002	17	20.67
High-income Asia Pacific	Boys	2003	17	20.75

High-income Asia Pacific	Boys	2004	17	20.84
High-income Asia Pacific	Boys	2005	17	20.95
High-income Asia Pacific	Boys	2006	17	21.06
High-income Asia Pacific	Boys	2007	17	21.18
High-income Asia Pacific	Boys	2008	17	21.31
High-income Asia Pacific	Boys	2009	17	21.43
High-income Asia Pacific	Boys	2010	17	21.54
High-income Asia Pacific	Boys	2011	17	21.63
High-income Asia Pacific	Boys	2012	17	21.70
High-income Asia Pacific	Boys	2013	17	21.76
High-income Asia Pacific	Boys	2014	17	21.80
High-income Asia Pacific	Boys	2015	17	21.83
High-income Asia Pacific	Boys	2016	17	21.85
High-income Asia Pacific	Boys	1975	18	19.14
High-income Asia Pacific	Boys	1976	18	19.30
High-income Asia Pacific	Boys	1977	18	19.45
High-income Asia Pacific	Boys	1978	18	19.61
High-income Asia Pacific	Boys	1979	18	19.77
High-income Asia Pacific	Boys	1980	18	19.93
High-income Asia Pacific	Boys	1981	18	20.09
High-income Asia Pacific	Boys	1982	18	20.24
High-income Asia Pacific	Boys	1983	18	20.40
High-income Asia Pacific	Boys	1984	18	20.56
High-income Asia Pacific	Boys	1985	18	20.72
High-income Asia Pacific	Boys	1986	18	20.88
High-income Asia Pacific	Boys	1987	18	21.04
High-income Asia Pacific	Boys	1988	18	21.20
High-income Asia Pacific	Boys	1989	18	21.36
High-income Asia Pacific	Boys	1990	18	21.52
High-income Asia Pacific	Boys	1991	18	21.67
High-income Asia Pacific	Boys	1992	18	21.82
High-income Asia Pacific	Boys	1993	18	21.95
High-income Asia Pacific	Boys	1994	18	22.08
High-income Asia Pacific	Boys	1995	18	22.20
High-income Asia Pacific	Boys	1996	18	22.32
High-income Asia Pacific	Boys	1997	18	22.43
High-income Asia Pacific	Boys	1998	18	22.53
High-income Asia Pacific	Boys	1999	18	22.62
High-income Asia Pacific	Boys	2000	18	22.70
High-income Asia Pacific	Boys	2001	18	22.79
High-income Asia Pacific	Boys	2002	18	22.87
High-income Asia Pacific	Boys	2003	18	22.95
High-income Asia Pacific	Boys	2004	18	23.05
High-income Asia Pacific	Boys	2005	18	23.15
High-income Asia Pacific	Boys	2006	18	23.27
High-income Asia Pacific	Boys	2007	18	23.39
High-income Asia Pacific	Boys	2008	18	23.52
High-income Asia Pacific	Boys	2009	18	23.65
High-income Asia Pacific	Boys	2010	18	23.76
High-income Asia Pacific	Boys	2011	18	23.86
High-income Asia Pacific	Boys	2012	18	23.93
High-income Asia Pacific	Boys	2013	18	23.99

High-income Asia Pacific	Boys	2014	18	24.03
High-income Asia Pacific	Boys	2015	18	24.06
High-income Asia Pacific	Boys	2016	18	24.07
High-income Asia Pacific	Boys	1975	Average	12.78
High-income Asia Pacific	Boys	1976	Average	12.92
High-income Asia Pacific	Boys	1977	Average	13.07
High-income Asia Pacific	Boys	1978	Average	13.22
High-income Asia Pacific	Boys	1979	Average	13.37
High-income Asia Pacific	Boys	1980	Average	13.52
High-income Asia Pacific	Boys	1981	Average	13.67
High-income Asia Pacific	Boys	1982	Average	13.82
High-income Asia Pacific	Boys	1983	Average	13.97
High-income Asia Pacific	Boys	1984	Average	14.13
High-income Asia Pacific	Boys	1985	Average	14.28
High-income Asia Pacific	Boys	1986	Average	14.43
High-income Asia Pacific	Boys	1987	Average	14.59
High-income Asia Pacific	Boys	1988	Average	14.74
High-income Asia Pacific	Boys	1989	Average	14.90
High-income Asia Pacific	Boys	1990	Average	15.05
High-income Asia Pacific	Boys	1991	Average	15.19
High-income Asia Pacific	Boys	1992	Average	15.33
High-income Asia Pacific	Boys	1993	Average	15.46
High-income Asia Pacific	Boys	1994	Average	15.59
High-income Asia Pacific	Boys	1995	Average	15.70
High-income Asia Pacific	Boys	1996	Average	15.81
High-income Asia Pacific	Boys	1997	Average	15.90
High-income Asia Pacific	Boys	1998	Average	15.99
High-income Asia Pacific	Boys	1999	Average	16.07
High-income Asia Pacific	Boys	2000	Average	16.14
High-income Asia Pacific	Boys	2001	Average	16.22
High-income Asia Pacific	Boys	2002	Average	16.31
High-income Asia Pacific	Boys	2003	Average	16.41
High-income Asia Pacific	Boys	2004	Average	16.51
High-income Asia Pacific	Boys	2005	Average	16.61
High-income Asia Pacific	Boys	2006	Average	16.72
High-income Asia Pacific	Boys	2007	Average	16.82
High-income Asia Pacific	Boys	2008	Average	16.92
High-income Asia Pacific	Boys	2009	Average	17.02
High-income Asia Pacific	Boys	2010	Average	17.10
High-income Asia Pacific	Boys	2011	Average	17.16
High-income Asia Pacific	Boys	2012	Average	17.21
High-income Asia Pacific	Boys	2013	Average	17.24
High-income Asia Pacific	Boys	2014	Average	17.26
High-income Asia Pacific	Boys	2015	Average	17.28
High-income Asia Pacific	Boys	2016	Average	17.29
High-income Asia Pacific	Boys	2017	Average	17.29
High-income Asia Pacific	Boys	2018	Average	17.29
High-income Asia Pacific	Boys	2019	Average	17.29
High-income Asia Pacific	Boys	2020	Average	17.27
High-income Asia Pacific	Boys	2021	Average	17.25
High-income Asia Pacific	Boys	2022	Average	17.23
High-income Asia Pacific	Boys	2023	Average	17.20

High-income Asia Pacific	Boys	2024	Average	17.17
High-income Asia Pacific	Boys	2025	Average	17.13
High-income Asia Pacific	Boys	2026	Average	17.09
High-income Asia Pacific	Boys	2027	Average	17.04
High-income Asia Pacific	Boys	2028	Average	16.99
High-income Asia Pacific	Boys	2029	Average	16.94
High-income Asia Pacific	Boys	2030	Average	16.89
High-income Asia Pacific	Girls	1975	12	6.40
High-income Asia Pacific	Girls	1976	12	6.45
High-income Asia Pacific	Girls	1977	12	6.51
High-income Asia Pacific	Girls	1978	12	6.56
High-income Asia Pacific	Girls	1979	12	6.62
High-income Asia Pacific	Girls	1980	12	6.67
High-income Asia Pacific	Girls	1981	12	6.72
High-income Asia Pacific	Girls	1982	12	6.78
High-income Asia Pacific	Girls	1983	12	6.83
High-income Asia Pacific	Girls	1984	12	6.88
High-income Asia Pacific	Girls	1985	12	6.93
High-income Asia Pacific	Girls	1986	12	6.98
High-income Asia Pacific	Girls	1987	12	7.03
High-income Asia Pacific	Girls	1988	12	7.08
High-income Asia Pacific	Girls	1989	12	7.14
High-income Asia Pacific	Girls	1990	12	7.19
High-income Asia Pacific	Girls	1991	12	7.24
High-income Asia Pacific	Girls	1992	12	7.30
High-income Asia Pacific	Girls	1993	12	7.34
High-income Asia Pacific	Girls	1994	12	7.39
High-income Asia Pacific	Girls	1995	12	7.43
High-income Asia Pacific	Girls	1996	12	7.47
High-income Asia Pacific	Girls	1997	12	7.51
High-income Asia Pacific	Girls	1998	12	7.54
High-income Asia Pacific	Girls	1999	12	7.56
High-income Asia Pacific	Girls	2000	12	7.59
High-income Asia Pacific	Girls	2001	12	7.63
High-income Asia Pacific	Girls	2002	12	7.67
High-income Asia Pacific	Girls	2003	12	7.72
High-income Asia Pacific	Girls	2004	12	7.76
High-income Asia Pacific	Girls	2005	12	7.81
High-income Asia Pacific	Girls	2006	12	7.85
High-income Asia Pacific	Girls	2007	12	7.89
High-income Asia Pacific	Girls	2008	12	7.92
High-income Asia Pacific	Girls	2009	12	7.94
High-income Asia Pacific	Girls	2010	12	7.96
High-income Asia Pacific	Girls	2011	12	7.98
High-income Asia Pacific	Girls	2012	12	7.99
High-income Asia Pacific	Girls	2013	12	7.99
High-income Asia Pacific	Girls	2014	12	8.00
High-income Asia Pacific	Girls	2015	12	8.01
High-income Asia Pacific	Girls	2016	12	8.01
High-income Asia Pacific	Girls	1975	13	7.86
High-income Asia Pacific	Girls	1976	13	7.92
High-income Asia Pacific	Girls	1977	13	7.98

High-income Asia Pacific	Girls	1978	13	8.04
High-income Asia Pacific	Girls	1979	13	8.10
High-income Asia Pacific	Girls	1980	13	8.16
High-income Asia Pacific	Girls	1981	13	8.22
High-income Asia Pacific	Girls	1982	13	8.28
High-income Asia Pacific	Girls	1983	13	8.33
High-income Asia Pacific	Girls	1984	13	8.39
High-income Asia Pacific	Girls	1985	13	8.45
High-income Asia Pacific	Girls	1986	13	8.50
High-income Asia Pacific	Girls	1987	13	8.56
High-income Asia Pacific	Girls	1988	13	8.62
High-income Asia Pacific	Girls	1989	13	8.68
High-income Asia Pacific	Girls	1990	13	8.74
High-income Asia Pacific	Girls	1991	13	8.80
High-income Asia Pacific	Girls	1992	13	8.85
High-income Asia Pacific	Girls	1993	13	8.91
High-income Asia Pacific	Girls	1994	13	8.96
High-income Asia Pacific	Girls	1995	13	9.01
High-income Asia Pacific	Girls	1996	13	9.05
High-income Asia Pacific	Girls	1997	13	9.09
High-income Asia Pacific	Girls	1998	13	9.12
High-income Asia Pacific	Girls	1999	13	9.15
High-income Asia Pacific	Girls	2000	13	9.18
High-income Asia Pacific	Girls	2001	13	9.22
High-income Asia Pacific	Girls	2002	13	9.27
High-income Asia Pacific	Girls	2003	13	9.32
High-income Asia Pacific	Girls	2004	13	9.38
High-income Asia Pacific	Girls	2005	13	9.43
High-income Asia Pacific	Girls	2006	13	9.47
High-income Asia Pacific	Girls	2007	13	9.51
High-income Asia Pacific	Girls	2008	13	9.55
High-income Asia Pacific	Girls	2009	13	9.57
High-income Asia Pacific	Girls	2010	13	9.60
High-income Asia Pacific	Girls	2011	13	9.62
High-income Asia Pacific	Girls	2012	13	9.63
High-income Asia Pacific	Girls	2013	13	9.63
High-income Asia Pacific	Girls	2014	13	9.64
High-income Asia Pacific	Girls	2015	13	9.65
High-income Asia Pacific	Girls	2016	13	9.66
High-income Asia Pacific	Girls	1975	14	9.37
High-income Asia Pacific	Girls	1976	14	9.44
High-income Asia Pacific	Girls	1977	14	9.50
High-income Asia Pacific	Girls	1978	14	9.57
High-income Asia Pacific	Girls	1979	14	9.63
High-income Asia Pacific	Girls	1980	14	9.69
High-income Asia Pacific	Girls	1981	14	9.76
High-income Asia Pacific	Girls	1982	14	9.82
High-income Asia Pacific	Girls	1983	14	9.88
High-income Asia Pacific	Girls	1984	14	9.94
High-income Asia Pacific	Girls	1985	14	9.99
High-income Asia Pacific	Girls	1986	14	10.05
High-income Asia Pacific	Girls	1987	14	10.12

High-income Asia Pacific	Girls	1988	14	10.18
High-income Asia Pacific	Girls	1989	14	10.24
High-income Asia Pacific	Girls	1990	14	10.30
High-income Asia Pacific	Girls	1991	14	10.36
High-income Asia Pacific	Girls	1992	14	10.42
High-income Asia Pacific	Girls	1993	14	10.48
High-income Asia Pacific	Girls	1994	14	10.54
High-income Asia Pacific	Girls	1995	14	10.59
High-income Asia Pacific	Girls	1996	14	10.63
High-income Asia Pacific	Girls	1997	14	10.67
High-income Asia Pacific	Girls	1998	14	10.71
High-income Asia Pacific	Girls	1999	14	10.74
High-income Asia Pacific	Girls	2000	14	10.77
High-income Asia Pacific	Girls	2001	14	10.81
High-income Asia Pacific	Girls	2002	14	10.86
High-income Asia Pacific	Girls	2003	14	10.92
High-income Asia Pacific	Girls	2004	14	10.98
High-income Asia Pacific	Girls	2005	14	11.03
High-income Asia Pacific	Girls	2006	14	11.08
High-income Asia Pacific	Girls	2007	14	11.12
High-income Asia Pacific	Girls	2008	14	11.16
High-income Asia Pacific	Girls	2009	14	11.19
High-income Asia Pacific	Girls	2010	14	11.21
High-income Asia Pacific	Girls	2011	14	11.23
High-income Asia Pacific	Girls	2012	14	11.24
High-income Asia Pacific	Girls	2013	14	11.25
High-income Asia Pacific	Girls	2014	14	11.25
High-income Asia Pacific	Girls	2015	14	11.26
High-income Asia Pacific	Girls	2016	14	11.27
High-income Asia Pacific	Girls	1975	15	10.74
High-income Asia Pacific	Girls	1976	15	10.80
High-income Asia Pacific	Girls	1977	15	10.87
High-income Asia Pacific	Girls	1978	15	10.94
High-income Asia Pacific	Girls	1979	15	11.00
High-income Asia Pacific	Girls	1980	15	11.06
High-income Asia Pacific	Girls	1981	15	11.13
High-income Asia Pacific	Girls	1982	15	11.19
High-income Asia Pacific	Girls	1983	15	11.25
High-income Asia Pacific	Girls	1984	15	11.30
High-income Asia Pacific	Girls	1985	15	11.36
High-income Asia Pacific	Girls	1986	15	11.42
High-income Asia Pacific	Girls	1987	15	11.48
High-income Asia Pacific	Girls	1988	15	11.55
High-income Asia Pacific	Girls	1989	15	11.61
High-income Asia Pacific	Girls	1990	15	11.67
High-income Asia Pacific	Girls	1991	15	11.72
High-income Asia Pacific	Girls	1992	15	11.78
High-income Asia Pacific	Girls	1993	15	11.83
High-income Asia Pacific	Girls	1994	15	11.89
High-income Asia Pacific	Girls	1995	15	11.94
High-income Asia Pacific	Girls	1996	15	11.98
High-income Asia Pacific	Girls	1997	15	12.03

High-income Asia Pacific	Girls	1998	15	12.07
High-income Asia Pacific	Girls	1999	15	12.11
High-income Asia Pacific	Girls	2000	15	12.15
High-income Asia Pacific	Girls	2001	15	12.19
High-income Asia Pacific	Girls	2002	15	12.22
High-income Asia Pacific	Girls	2003	15	12.26
High-income Asia Pacific	Girls	2004	15	12.30
High-income Asia Pacific	Girls	2005	15	12.35
High-income Asia Pacific	Girls	2006	15	12.40
High-income Asia Pacific	Girls	2007	15	12.45
High-income Asia Pacific	Girls	2008	15	12.51
High-income Asia Pacific	Girls	2009	15	12.56
High-income Asia Pacific	Girls	2010	15	12.61
High-income Asia Pacific	Girls	2011	15	12.64
High-income Asia Pacific	Girls	2012	15	12.68
High-income Asia Pacific	Girls	2013	15	12.70
High-income Asia Pacific	Girls	2014	15	12.72
High-income Asia Pacific	Girls	2015	15	12.74
High-income Asia Pacific	Girls	2016	15	12.75
High-income Asia Pacific	Girls	1975	16	11.78
High-income Asia Pacific	Girls	1976	16	11.85
High-income Asia Pacific	Girls	1977	16	11.91
High-income Asia Pacific	Girls	1978	16	11.98
High-income Asia Pacific	Girls	1979	16	12.04
High-income Asia Pacific	Girls	1980	16	12.10
High-income Asia Pacific	Girls	1981	16	12.16
High-income Asia Pacific	Girls	1982	16	12.22
High-income Asia Pacific	Girls	1983	16	12.28
High-income Asia Pacific	Girls	1984	16	12.34
High-income Asia Pacific	Girls	1985	16	12.40
High-income Asia Pacific	Girls	1986	16	12.46
High-income Asia Pacific	Girls	1987	16	12.52
High-income Asia Pacific	Girls	1988	16	12.58
High-income Asia Pacific	Girls	1989	16	12.64
High-income Asia Pacific	Girls	1990	16	12.69
High-income Asia Pacific	Girls	1991	16	12.75
High-income Asia Pacific	Girls	1992	16	12.81
High-income Asia Pacific	Girls	1993	16	12.86
High-income Asia Pacific	Girls	1994	16	12.91
High-income Asia Pacific	Girls	1995	16	12.96
High-income Asia Pacific	Girls	1996	16	13.01
High-income Asia Pacific	Girls	1997	16	13.06
High-income Asia Pacific	Girls	1998	16	13.10
High-income Asia Pacific	Girls	1999	16	13.14
High-income Asia Pacific	Girls	2000	16	13.18
High-income Asia Pacific	Girls	2001	16	13.21
High-income Asia Pacific	Girls	2002	16	13.25
High-income Asia Pacific	Girls	2003	16	13.29
High-income Asia Pacific	Girls	2004	16	13.33
High-income Asia Pacific	Girls	2005	16	13.37
High-income Asia Pacific	Girls	2006	16	13.42
High-income Asia Pacific	Girls	2007	16	13.48

High-income Asia Pacific	Girls	2008	16	13.53
High-income Asia Pacific	Girls	2009	16	13.59
High-income Asia Pacific	Girls	2010	16	13.63
High-income Asia Pacific	Girls	2011	16	13.67
High-income Asia Pacific	Girls	2012	16	13.71
High-income Asia Pacific	Girls	2013	16	13.73
High-income Asia Pacific	Girls	2014	16	13.75
High-income Asia Pacific	Girls	2015	16	13.77
High-income Asia Pacific	Girls	2016	16	13.78
High-income Asia Pacific	Girls	1975	17	12.49
High-income Asia Pacific	Girls	1976	17	12.55
High-income Asia Pacific	Girls	1977	17	12.61
High-income Asia Pacific	Girls	1978	17	12.68
High-income Asia Pacific	Girls	1979	17	12.74
High-income Asia Pacific	Girls	1980	17	12.80
High-income Asia Pacific	Girls	1981	17	12.86
High-income Asia Pacific	Girls	1982	17	12.92
High-income Asia Pacific	Girls	1983	17	12.97
High-income Asia Pacific	Girls	1984	17	13.03
High-income Asia Pacific	Girls	1985	17	13.09
High-income Asia Pacific	Girls	1986	17	13.15
High-income Asia Pacific	Girls	1987	17	13.21
High-income Asia Pacific	Girls	1988	17	13.27
High-income Asia Pacific	Girls	1989	17	13.32
High-income Asia Pacific	Girls	1990	17	13.38
High-income Asia Pacific	Girls	1991	17	13.44
High-income Asia Pacific	Girls	1992	17	13.50
High-income Asia Pacific	Girls	1993	17	13.55
High-income Asia Pacific	Girls	1994	17	13.60
High-income Asia Pacific	Girls	1995	17	13.65
High-income Asia Pacific	Girls	1996	17	13.70
High-income Asia Pacific	Girls	1997	17	13.75
High-income Asia Pacific	Girls	1998	17	13.79
High-income Asia Pacific	Girls	1999	17	13.83
High-income Asia Pacific	Girls	2000	17	13.87
High-income Asia Pacific	Girls	2001	17	13.90
High-income Asia Pacific	Girls	2002	17	13.94
High-income Asia Pacific	Girls	2003	17	13.98
High-income Asia Pacific	Girls	2004	17	14.02
High-income Asia Pacific	Girls	2005	17	14.06
High-income Asia Pacific	Girls	2006	17	14.12
High-income Asia Pacific	Girls	2007	17	14.17
High-income Asia Pacific	Girls	2008	17	14.23
High-income Asia Pacific	Girls	2009	17	14.28
High-income Asia Pacific	Girls	2010	17	14.33
High-income Asia Pacific	Girls	2011	17	14.37
High-income Asia Pacific	Girls	2012	17	14.41
High-income Asia Pacific	Girls	2013	17	14.43
High-income Asia Pacific	Girls	2014	17	14.45
High-income Asia Pacific	Girls	2015	17	14.47
High-income Asia Pacific	Girls	2016	17	14.48
High-income Asia Pacific	Girls	1975	18	12.93

High-income Asia Pacific	Girls	1976	18	13.00
High-income Asia Pacific	Girls	1977	18	13.06
High-income Asia Pacific	Girls	1978	18	13.13
High-income Asia Pacific	Girls	1979	18	13.19
High-income Asia Pacific	Girls	1980	18	13.25
High-income Asia Pacific	Girls	1981	18	13.31
High-income Asia Pacific	Girls	1982	18	13.37
High-income Asia Pacific	Girls	1983	18	13.43
High-income Asia Pacific	Girls	1984	18	13.48
High-income Asia Pacific	Girls	1985	18	13.54
High-income Asia Pacific	Girls	1986	18	13.60
High-income Asia Pacific	Girls	1987	18	13.66
High-income Asia Pacific	Girls	1988	18	13.72
High-income Asia Pacific	Girls	1989	18	13.78
High-income Asia Pacific	Girls	1990	18	13.84
High-income Asia Pacific	Girls	1991	18	13.90
High-income Asia Pacific	Girls	1992	18	13.96
High-income Asia Pacific	Girls	1993	18	14.01
High-income Asia Pacific	Girls	1994	18	14.07
High-income Asia Pacific	Girls	1995	18	14.12
High-income Asia Pacific	Girls	1996	18	14.17
High-income Asia Pacific	Girls	1997	18	14.22
High-income Asia Pacific	Girls	1998	18	14.26
High-income Asia Pacific	Girls	1999	18	14.31
High-income Asia Pacific	Girls	2000	18	14.34
High-income Asia Pacific	Girls	2001	18	14.38
High-income Asia Pacific	Girls	2002	18	14.42
High-income Asia Pacific	Girls	2003	18	14.46
High-income Asia Pacific	Girls	2004	18	14.50
High-income Asia Pacific	Girls	2005	18	14.55
High-income Asia Pacific	Girls	2006	18	14.60
High-income Asia Pacific	Girls	2007	18	14.66
High-income Asia Pacific	Girls	2008	18	14.72
High-income Asia Pacific	Girls	2009	18	14.78
High-income Asia Pacific	Girls	2010	18	14.83
High-income Asia Pacific	Girls	2011	18	14.88
High-income Asia Pacific	Girls	2012	18	14.91
High-income Asia Pacific	Girls	2013	18	14.94
High-income Asia Pacific	Girls	2014	18	14.96
High-income Asia Pacific	Girls	2015	18	14.98
High-income Asia Pacific	Girls	2016	18	14.99
High-income Asia Pacific	Girls	1975	Average	10.22
High-income Asia Pacific	Girls	1976	Average	10.29
High-income Asia Pacific	Girls	1977	Average	10.35
High-income Asia Pacific	Girls	1978	Average	10.41
High-income Asia Pacific	Girls	1979	Average	10.47
High-income Asia Pacific	Girls	1980	Average	10.53
High-income Asia Pacific	Girls	1981	Average	10.59
High-income Asia Pacific	Girls	1982	Average	10.65
High-income Asia Pacific	Girls	1983	Average	10.71
High-income Asia Pacific	Girls	1984	Average	10.77
High-income Asia Pacific	Girls	1985	Average	10.82

High-income Asia Pacific	Girls	1986	Average	10.88
High-income Asia Pacific	Girls	1987	Average	10.94
High-income Asia Pacific	Girls	1988	Average	11.00
High-income Asia Pacific	Girls	1989	Average	11.06
High-income Asia Pacific	Girls	1990	Average	11.12
High-income Asia Pacific	Girls	1991	Average	11.17
High-income Asia Pacific	Girls	1992	Average	11.23
High-income Asia Pacific	Girls	1993	Average	11.28
High-income Asia Pacific	Girls	1994	Average	11.34
High-income Asia Pacific	Girls	1995	Average	11.39
High-income Asia Pacific	Girls	1996	Average	11.43
High-income Asia Pacific	Girls	1997	Average	11.47
High-income Asia Pacific	Girls	1998	Average	11.51
High-income Asia Pacific	Girls	1999	Average	11.55
High-income Asia Pacific	Girls	2000	Average	11.58
High-income Asia Pacific	Girls	2001	Average	11.62
High-income Asia Pacific	Girls	2002	Average	11.66
High-income Asia Pacific	Girls	2003	Average	11.71
High-income Asia Pacific	Girls	2004	Average	11.75
High-income Asia Pacific	Girls	2005	Average	11.80
High-income Asia Pacific	Girls	2006	Average	11.85
High-income Asia Pacific	Girls	2007	Average	11.90
High-income Asia Pacific	Girls	2008	Average	11.94
High-income Asia Pacific	Girls	2009	Average	11.99
High-income Asia Pacific	Girls	2010	Average	12.02
High-income Asia Pacific	Girls	2011	Average	12.06
High-income Asia Pacific	Girls	2012	Average	12.08
High-income Asia Pacific	Girls	2013	Average	12.10
High-income Asia Pacific	Girls	2014	Average	12.11
High-income Asia Pacific	Girls	2015	Average	12.13
High-income Asia Pacific	Girls	2016	Average	12.14
High-income Asia Pacific	Girls	2017	Average	12.14
High-income Asia Pacific	Girls	2018	Average	12.15
High-income Asia Pacific	Girls	2019	Average	12.15
High-income Asia Pacific	Girls	2020	Average	12.16
High-income Asia Pacific	Girls	2021	Average	12.16
High-income Asia Pacific	Girls	2022	Average	12.16
High-income Asia Pacific	Girls	2023	Average	12.16
High-income Asia Pacific	Girls	2024	Average	12.16
High-income Asia Pacific	Girls	2025	Average	12.15
High-income Asia Pacific	Girls	2026	Average	12.15
High-income Asia Pacific	Girls	2027	Average	12.15
High-income Asia Pacific	Girls	2028	Average	12.15
High-income Asia Pacific	Girls	2029	Average	12.15
High-income Asia Pacific	Girls	2030	Average	12.14
High-income Western countries	Boys	1975	12	9.10
High-income Western countries	Boys	1976	12	9.21
High-income Western countries	Boys	1977	12	9.33
High-income Western countries	Boys	1978	12	9.44
High-income Western countries	Boys	1979	12	9.57
High-income Western countries	Boys	1980	12	9.70
High-income Western countries	Boys	1981	12	9.83

High-income Western countries	Boys	1982	12	9.98
High-income Western countries	Boys	1983	12	10.12
High-income Western countries	Boys	1984	12	10.28
High-income Western countries	Boys	1985	12	10.43
High-income Western countries	Boys	1986	12	10.59
High-income Western countries	Boys	1987	12	10.76
High-income Western countries	Boys	1988	12	10.92
High-income Western countries	Boys	1989	12	11.09
High-income Western countries	Boys	1990	12	11.26
High-income Western countries	Boys	1991	12	11.43
High-income Western countries	Boys	1992	12	11.60
High-income Western countries	Boys	1993	12	11.77
High-income Western countries	Boys	1994	12	11.93
High-income Western countries	Boys	1995	12	12.10
High-income Western countries	Boys	1996	12	12.28
High-income Western countries	Boys	1997	12	12.45
High-income Western countries	Boys	1998	12	12.62
High-income Western countries	Boys	1999	12	12.79
High-income Western countries	Boys	2000	12	12.95
High-income Western countries	Boys	2001	12	13.11
High-income Western countries	Boys	2002	12	13.26
High-income Western countries	Boys	2003	12	13.40
High-income Western countries	Boys	2004	12	13.53
High-income Western countries	Boys	2005	12	13.66
High-income Western countries	Boys	2006	12	13.77
High-income Western countries	Boys	2007	12	13.88
High-income Western countries	Boys	2008	12	13.98
High-income Western countries	Boys	2009	12	14.08
High-income Western countries	Boys	2010	12	14.17
High-income Western countries	Boys	2011	12	14.25
High-income Western countries	Boys	2012	12	14.34
High-income Western countries	Boys	2013	12	14.42
High-income Western countries	Boys	2014	12	14.49
High-income Western countries	Boys	2015	12	14.57
High-income Western countries	Boys	2016	12	14.64
High-income Western countries	Boys	1975	13	11.17
High-income Western countries	Boys	1976	13	11.31
High-income Western countries	Boys	1977	13	11.44
High-income Western countries	Boys	1978	13	11.58
High-income Western countries	Boys	1979	13	11.73
High-income Western countries	Boys	1980	13	11.88
High-income Western countries	Boys	1981	13	12.04
High-income Western countries	Boys	1982	13	12.21
High-income Western countries	Boys	1983	13	12.39
High-income Western countries	Boys	1984	13	12.58
High-income Western countries	Boys	1985	13	12.76
High-income Western countries	Boys	1986	13	12.95
High-income Western countries	Boys	1987	13	13.15
High-income Western countries	Boys	1988	13	13.35
High-income Western countries	Boys	1989	13	13.56
High-income Western countries	Boys	1990	13	13.76
High-income Western countries	Boys	1991	13	13.96

High-income Western countries	Boys	1992	13	14.17
High-income Western countries	Boys	1993	13	14.37
High-income Western countries	Boys	1994	13	14.58
High-income Western countries	Boys	1995	13	14.78
High-income Western countries	Boys	1996	13	14.99
High-income Western countries	Boys	1997	13	15.20
High-income Western countries	Boys	1998	13	15.41
High-income Western countries	Boys	1999	13	15.62
High-income Western countries	Boys	2000	13	15.82
High-income Western countries	Boys	2001	13	16.01
High-income Western countries	Boys	2002	13	16.20
High-income Western countries	Boys	2003	13	16.37
High-income Western countries	Boys	2004	13	16.54
High-income Western countries	Boys	2005	13	16.69
High-income Western countries	Boys	2006	13	16.83
High-income Western countries	Boys	2007	13	16.96
High-income Western countries	Boys	2008	13	17.09
High-income Western countries	Boys	2009	13	17.21
High-income Western countries	Boys	2010	13	17.32
High-income Western countries	Boys	2011	13	17.42
High-income Western countries	Boys	2012	13	17.53
High-income Western countries	Boys	2013	13	17.63
High-income Western countries	Boys	2014	13	17.72
High-income Western countries	Boys	2015	13	17.81
High-income Western countries	Boys	2016	13	17.90
High-income Western countries	Boys	1975	14	13.50
High-income Western countries	Boys	1976	14	13.64
High-income Western countries	Boys	1977	14	13.79
High-income Western countries	Boys	1978	14	13.95
High-income Western countries	Boys	1979	14	14.11
High-income Western countries	Boys	1980	14	14.29
High-income Western countries	Boys	1981	14	14.47
High-income Western countries	Boys	1982	14	14.66
High-income Western countries	Boys	1983	14	14.86
High-income Western countries	Boys	1984	14	15.07
High-income Western countries	Boys	1985	14	15.28
High-income Western countries	Boys	1986	14	15.49
High-income Western countries	Boys	1987	14	15.72
High-income Western countries	Boys	1988	14	15.94
High-income Western countries	Boys	1989	14	16.17
High-income Western countries	Boys	1990	14	16.40
High-income Western countries	Boys	1991	14	16.63
High-income Western countries	Boys	1992	14	16.87
High-income Western countries	Boys	1993	14	17.10
High-income Western countries	Boys	1994	14	17.33
High-income Western countries	Boys	1995	14	17.57
High-income Western countries	Boys	1996	14	17.81
High-income Western countries	Boys	1997	14	18.05
High-income Western countries	Boys	1998	14	18.29
High-income Western countries	Boys	1999	14	18.53
High-income Western countries	Boys	2000	14	18.76
High-income Western countries	Boys	2001	14	18.98

High-income Western countries	Boys	2002	14	19.20
High-income Western countries	Boys	2003	14	19.40
High-income Western countries	Boys	2004	14	19.59
High-income Western countries	Boys	2005	14	19.76
High-income Western countries	Boys	2006	14	19.93
High-income Western countries	Boys	2007	14	20.08
High-income Western countries	Boys	2008	14	20.22
High-income Western countries	Boys	2009	14	20.36
High-income Western countries	Boys	2010	14	20.49
High-income Western countries	Boys	2011	14	20.61
High-income Western countries	Boys	2012	14	20.73
High-income Western countries	Boys	2013	14	20.84
High-income Western countries	Boys	2014	14	20.95
High-income Western countries	Boys	2015	14	21.06
High-income Western countries	Boys	2016	14	21.17
High-income Western countries	Boys	1975	15	15.97
High-income Western countries	Boys	1976	15	16.12
High-income Western countries	Boys	1977	15	16.29
High-income Western countries	Boys	1978	15	16.45
High-income Western countries	Boys	1979	15	16.62
High-income Western countries	Boys	1980	15	16.79
High-income Western countries	Boys	1981	15	16.97
High-income Western countries	Boys	1982	15	17.16
High-income Western countries	Boys	1983	15	17.35
High-income Western countries	Boys	1984	15	17.55
High-income Western countries	Boys	1985	15	17.77
High-income Western countries	Boys	1986	15	17.99
High-income Western countries	Boys	1987	15	18.22
High-income Western countries	Boys	1988	15	18.46
High-income Western countries	Boys	1989	15	18.71
High-income Western countries	Boys	1990	15	18.96
High-income Western countries	Boys	1991	15	19.22
High-income Western countries	Boys	1992	15	19.48
High-income Western countries	Boys	1993	15	19.75
High-income Western countries	Boys	1994	15	20.01
High-income Western countries	Boys	1995	15	20.28
High-income Western countries	Boys	1996	15	20.54
High-income Western countries	Boys	1997	15	20.80
High-income Western countries	Boys	1998	15	21.06
High-income Western countries	Boys	1999	15	21.30
High-income Western countries	Boys	2000	15	21.54
High-income Western countries	Boys	2001	15	21.78
High-income Western countries	Boys	2002	15	22.00
High-income Western countries	Boys	2003	15	22.21
High-income Western countries	Boys	2004	15	22.41
High-income Western countries	Boys	2005	15	22.60
High-income Western countries	Boys	2006	15	22.78
High-income Western countries	Boys	2007	15	22.95
High-income Western countries	Boys	2008	15	23.12
High-income Western countries	Boys	2009	15	23.27
High-income Western countries	Boys	2010	15	23.41
High-income Western countries	Boys	2011	15	23.55

High-income Western countries	Boys	2012	15	23.68
High-income Western countries	Boys	2013	15	23.80
High-income Western countries	Boys	2014	15	23.92
High-income Western countries	Boys	2015	15	24.05
High-income Western countries	Boys	2016	15	24.17
High-income Western countries	Boys	1975	16	18.46
High-income Western countries	Boys	1976	16	18.62
High-income Western countries	Boys	1977	16	18.78
High-income Western countries	Boys	1978	16	18.95
High-income Western countries	Boys	1979	16	19.13
High-income Western countries	Boys	1980	16	19.30
High-income Western countries	Boys	1981	16	19.49
High-income Western countries	Boys	1982	16	19.68
High-income Western countries	Boys	1983	16	19.88
High-income Western countries	Boys	1984	16	20.09
High-income Western countries	Boys	1985	16	20.31
High-income Western countries	Boys	1986	16	20.54
High-income Western countries	Boys	1987	16	20.78
High-income Western countries	Boys	1988	16	21.03
High-income Western countries	Boys	1989	16	21.29
High-income Western countries	Boys	1990	16	21.55
High-income Western countries	Boys	1991	16	21.81
High-income Western countries	Boys	1992	16	22.08
High-income Western countries	Boys	1993	16	22.36
High-income Western countries	Boys	1994	16	22.64
High-income Western countries	Boys	1995	16	22.91
High-income Western countries	Boys	1996	16	23.19
High-income Western countries	Boys	1997	16	23.46
High-income Western countries	Boys	1998	16	23.72
High-income Western countries	Boys	1999	16	23.98
High-income Western countries	Boys	2000	16	24.23
High-income Western countries	Boys	2001	16	24.47
High-income Western countries	Boys	2002	16	24.70
High-income Western countries	Boys	2003	16	24.93
High-income Western countries	Boys	2004	16	25.14
High-income Western countries	Boys	2005	16	25.33
High-income Western countries	Boys	2006	16	25.52
High-income Western countries	Boys	2007	16	25.70
High-income Western countries	Boys	2008	16	25.87
High-income Western countries	Boys	2009	16	26.03
High-income Western countries	Boys	2010	16	26.18
High-income Western countries	Boys	2011	16	26.32
High-income Western countries	Boys	2012	16	26.45
High-income Western countries	Boys	2013	16	26.58
High-income Western countries	Boys	2014	16	26.71
High-income Western countries	Boys	2015	16	26.84
High-income Western countries	Boys	2016	16	26.98
High-income Western countries	Boys	1975	17	20.99
High-income Western countries	Boys	1976	17	21.16
High-income Western countries	Boys	1977	17	21.32
High-income Western countries	Boys	1978	17	21.50
High-income Western countries	Boys	1979	17	21.67

High-income Western countries	Boys	1980	17	21.85
High-income Western countries	Boys	1981	17	22.04
High-income Western countries	Boys	1982	17	22.24
High-income Western countries	Boys	1983	17	22.44
High-income Western countries	Boys	1984	17	22.66
High-income Western countries	Boys	1985	17	22.88
High-income Western countries	Boys	1986	17	23.12
High-income Western countries	Boys	1987	17	23.36
High-income Western countries	Boys	1988	17	23.62
High-income Western countries	Boys	1989	17	23.88
High-income Western countries	Boys	1990	17	24.15
High-income Western countries	Boys	1991	17	24.42
High-income Western countries	Boys	1992	17	24.70
High-income Western countries	Boys	1993	17	24.98
High-income Western countries	Boys	1994	17	25.27
High-income Western countries	Boys	1995	17	25.55
High-income Western countries	Boys	1996	17	25.83
High-income Western countries	Boys	1997	17	26.11
High-income Western countries	Boys	1998	17	26.38
High-income Western countries	Boys	1999	17	26.65
High-income Western countries	Boys	2000	17	26.91
High-income Western countries	Boys	2001	17	27.16
High-income Western countries	Boys	2002	17	27.40
High-income Western countries	Boys	2003	17	27.62
High-income Western countries	Boys	2004	17	27.84
High-income Western countries	Boys	2005	17	28.04
High-income Western countries	Boys	2006	17	28.24
High-income Western countries	Boys	2007	17	28.42
High-income Western countries	Boys	2008	17	28.60
High-income Western countries	Boys	2009	17	28.76
High-income Western countries	Boys	2010	17	28.91
High-income Western countries	Boys	2011	17	29.06
High-income Western countries	Boys	2012	17	29.20
High-income Western countries	Boys	2013	17	29.33
High-income Western countries	Boys	2014	17	29.47
High-income Western countries	Boys	2015	17	29.60
High-income Western countries	Boys	2016	17	29.74
High-income Western countries	Boys	1975	18	23.61
High-income Western countries	Boys	1976	18	23.79
High-income Western countries	Boys	1977	18	23.96
High-income Western countries	Boys	1978	18	24.14
High-income Western countries	Boys	1979	18	24.32
High-income Western countries	Boys	1980	18	24.51
High-income Western countries	Boys	1981	18	24.71
High-income Western countries	Boys	1982	18	24.91
High-income Western countries	Boys	1983	18	25.12
High-income Western countries	Boys	1984	18	25.35
High-income Western countries	Boys	1985	18	25.58
High-income Western countries	Boys	1986	18	25.82
High-income Western countries	Boys	1987	18	26.08
High-income Western countries	Boys	1988	18	26.34
High-income Western countries	Boys	1989	18	26.61

High-income Western countries	Boys	1990	18	26.89
High-income Western countries	Boys	1991	18	27.17
High-income Western countries	Boys	1992	18	27.46
High-income Western countries	Boys	1993	18	27.76
High-income Western countries	Boys	1994	18	28.05
High-income Western countries	Boys	1995	18	28.35
High-income Western countries	Boys	1996	18	28.64
High-income Western countries	Boys	1997	18	28.93
High-income Western countries	Boys	1998	18	29.21
High-income Western countries	Boys	1999	18	29.49
High-income Western countries	Boys	2000	18	29.76
High-income Western countries	Boys	2001	18	30.02
High-income Western countries	Boys	2002	18	30.27
High-income Western countries	Boys	2003	18	30.50
High-income Western countries	Boys	2004	18	30.73
High-income Western countries	Boys	2005	18	30.94
High-income Western countries	Boys	2006	18	31.14
High-income Western countries	Boys	2007	18	31.33
High-income Western countries	Boys	2008	18	31.51
High-income Western countries	Boys	2009	18	31.68
High-income Western countries	Boys	2010	18	31.84
High-income Western countries	Boys	2011	18	31.99
High-income Western countries	Boys	2012	18	32.13
High-income Western countries	Boys	2013	18	32.27
High-income Western countries	Boys	2014	18	32.40
High-income Western countries	Boys	2015	18	32.54
High-income Western countries	Boys	2016	18	32.69
High-income Western countries	Boys	1975	Average	16.11
High-income Western countries	Boys	1976	Average	16.26
High-income Western countries	Boys	1977	Average	16.42
High-income Western countries	Boys	1978	Average	16.57
High-income Western countries	Boys	1979	Average	16.74
High-income Western countries	Boys	1980	Average	16.90
High-income Western countries	Boys	1981	Average	17.08
High-income Western countries	Boys	1982	Average	17.26
High-income Western countries	Boys	1983	Average	17.45
High-income Western countries	Boys	1984	Average	17.65
High-income Western countries	Boys	1985	Average	17.86
High-income Western countries	Boys	1986	Average	18.07
High-income Western countries	Boys	1987	Average	18.29
High-income Western countries	Boys	1988	Average	18.52
High-income Western countries	Boys	1989	Average	18.76
High-income Western countries	Boys	1990	Average	19.00
High-income Western countries	Boys	1991	Average	19.24
High-income Western countries	Boys	1992	Average	19.48
High-income Western countries	Boys	1993	Average	19.73
High-income Western countries	Boys	1994	Average	19.97
High-income Western countries	Boys	1995	Average	20.22
High-income Western countries	Boys	1996	Average	20.47
High-income Western countries	Boys	1997	Average	20.71
High-income Western countries	Boys	1998	Average	20.96
High-income Western countries	Boys	1999	Average	21.19

High-income Western countries	Boys	2000	Average	21.42
High-income Western countries	Boys	2001	Average	21.65
High-income Western countries	Boys	2002	Average	21.86
High-income Western countries	Boys	2003	Average	22.06
High-income Western countries	Boys	2004	Average	22.25
High-income Western countries	Boys	2005	Average	22.43
High-income Western countries	Boys	2006	Average	22.60
High-income Western countries	Boys	2007	Average	22.76
High-income Western countries	Boys	2008	Average	22.91
High-income Western countries	Boys	2009	Average	23.05
High-income Western countries	Boys	2010	Average	23.19
High-income Western countries	Boys	2011	Average	23.31
High-income Western countries	Boys	2012	Average	23.44
High-income Western countries	Boys	2013	Average	23.55
High-income Western countries	Boys	2014	Average	23.67
High-income Western countries	Boys	2015	Average	23.78
High-income Western countries	Boys	2016	Average	23.90
High-income Western countries	Boys	2017	Average	24.02
High-income Western countries	Boys	2018	Average	24.14
High-income Western countries	Boys	2019	Average	24.26
High-income Western countries	Boys	2020	Average	24.39
High-income Western countries	Boys	2021	Average	24.52
High-income Western countries	Boys	2022	Average	24.65
High-income Western countries	Boys	2023	Average	24.78
High-income Western countries	Boys	2024	Average	24.91
High-income Western countries	Boys	2025	Average	25.05
High-income Western countries	Boys	2026	Average	25.18
High-income Western countries	Boys	2027	Average	25.32
High-income Western countries	Boys	2028	Average	25.46
High-income Western countries	Boys	2029	Average	25.60
High-income Western countries	Boys	2030	Average	25.74
High-income Western countries	Girls	1975	12	8.59
High-income Western countries	Girls	1976	12	8.68
High-income Western countries	Girls	1977	12	8.77
High-income Western countries	Girls	1978	12	8.85
High-income Western countries	Girls	1979	12	8.94
High-income Western countries	Girls	1980	12	9.04
High-income Western countries	Girls	1981	12	9.14
High-income Western countries	Girls	1982	12	9.24
High-income Western countries	Girls	1983	12	9.35
High-income Western countries	Girls	1984	12	9.46
High-income Western countries	Girls	1985	12	9.57
High-income Western countries	Girls	1986	12	9.68
High-income Western countries	Girls	1987	12	9.80
High-income Western countries	Girls	1988	12	9.92
High-income Western countries	Girls	1989	12	10.04
High-income Western countries	Girls	1990	12	10.17
High-income Western countries	Girls	1991	12	10.29
High-income Western countries	Girls	1992	12	10.42
High-income Western countries	Girls	1993	12	10.54
High-income Western countries	Girls	1994	12	10.67
High-income Western countries	Girls	1995	12	10.80

High-income Western countries	Girls	1996	12	10.92
High-income Western countries	Girls	1997	12	11.05
High-income Western countries	Girls	1998	12	11.18
High-income Western countries	Girls	1999	12	11.30
High-income Western countries	Girls	2000	12	11.42
High-income Western countries	Girls	2001	12	11.53
High-income Western countries	Girls	2002	12	11.64
High-income Western countries	Girls	2003	12	11.74
High-income Western countries	Girls	2004	12	11.83
High-income Western countries	Girls	2005	12	11.91
High-income Western countries	Girls	2006	12	11.98
High-income Western countries	Girls	2007	12	12.05
High-income Western countries	Girls	2008	12	12.12
High-income Western countries	Girls	2009	12	12.18
High-income Western countries	Girls	2010	12	12.23
High-income Western countries	Girls	2011	12	12.29
High-income Western countries	Girls	2012	12	12.34
High-income Western countries	Girls	2013	12	12.39
High-income Western countries	Girls	2014	12	12.45
High-income Western countries	Girls	2015	12	12.50
High-income Western countries	Girls	2016	12	12.54
High-income Western countries	Girls	1975	13	10.44
High-income Western countries	Girls	1976	13	10.54
High-income Western countries	Girls	1977	13	10.64
High-income Western countries	Girls	1978	13	10.74
High-income Western countries	Girls	1979	13	10.84
High-income Western countries	Girls	1980	13	10.95
High-income Western countries	Girls	1981	13	11.06
High-income Western countries	Girls	1982	13	11.18
High-income Western countries	Girls	1983	13	11.30
High-income Western countries	Girls	1984	13	11.43
High-income Western countries	Girls	1985	13	11.56
High-income Western countries	Girls	1986	13	11.69
High-income Western countries	Girls	1987	13	11.82
High-income Western countries	Girls	1988	13	11.96
High-income Western countries	Girls	1989	13	12.10
High-income Western countries	Girls	1990	13	12.24
High-income Western countries	Girls	1991	13	12.39
High-income Western countries	Girls	1992	13	12.53
High-income Western countries	Girls	1993	13	12.67
High-income Western countries	Girls	1994	13	12.82
High-income Western countries	Girls	1995	13	12.97
High-income Western countries	Girls	1996	13	13.12
High-income Western countries	Girls	1997	13	13.26
High-income Western countries	Girls	1998	13	13.41
High-income Western countries	Girls	1999	13	13.55
High-income Western countries	Girls	2000	13	13.69
High-income Western countries	Girls	2001	13	13.82
High-income Western countries	Girls	2002	13	13.94
High-income Western countries	Girls	2003	13	14.05
High-income Western countries	Girls	2004	13	14.16
High-income Western countries	Girls	2005	13	14.25

High-income Western countries	Girls	2006	13	14.34
High-income Western countries	Girls	2007	13	14.42
High-income Western countries	Girls	2008	13	14.49
High-income Western countries	Girls	2009	13	14.56
High-income Western countries	Girls	2010	13	14.63
High-income Western countries	Girls	2011	13	14.69
High-income Western countries	Girls	2012	13	14.75
High-income Western countries	Girls	2013	13	14.81
High-income Western countries	Girls	2014	13	14.87
High-income Western countries	Girls	2015	13	14.93
High-income Western countries	Girls	2016	13	14.98
High-income Western countries	Girls	1975	14	12.27
High-income Western countries	Girls	1976	14	12.38
High-income Western countries	Girls	1977	14	12.48
High-income Western countries	Girls	1978	14	12.59
High-income Western countries	Girls	1979	14	12.70
High-income Western countries	Girls	1980	14	12.82
High-income Western countries	Girls	1981	14	12.94
High-income Western countries	Girls	1982	14	13.07
High-income Western countries	Girls	1983	14	13.20
High-income Western countries	Girls	1984	14	13.34
High-income Western countries	Girls	1985	14	13.48
High-income Western countries	Girls	1986	14	13.62
High-income Western countries	Girls	1987	14	13.77
High-income Western countries	Girls	1988	14	13.92
High-income Western countries	Girls	1989	14	14.07
High-income Western countries	Girls	1990	14	14.22
High-income Western countries	Girls	1991	14	14.38
High-income Western countries	Girls	1992	14	14.54
High-income Western countries	Girls	1993	14	14.69
High-income Western countries	Girls	1994	14	14.85
High-income Western countries	Girls	1995	14	15.02
High-income Western countries	Girls	1996	14	15.18
High-income Western countries	Girls	1997	14	15.34
High-income Western countries	Girls	1998	14	15.50
High-income Western countries	Girls	1999	14	15.65
High-income Western countries	Girls	2000	14	15.80
High-income Western countries	Girls	2001	14	15.94
High-income Western countries	Girls	2002	14	16.08
High-income Western countries	Girls	2003	14	16.20
High-income Western countries	Girls	2004	14	16.31
High-income Western countries	Girls	2005	14	16.42
High-income Western countries	Girls	2006	14	16.51
High-income Western countries	Girls	2007	14	16.59
High-income Western countries	Girls	2008	14	16.67
High-income Western countries	Girls	2009	14	16.75
High-income Western countries	Girls	2010	14	16.82
High-income Western countries	Girls	2011	14	16.89
High-income Western countries	Girls	2012	14	16.95
High-income Western countries	Girls	2013	14	17.02
High-income Western countries	Girls	2014	14	17.08
High-income Western countries	Girls	2015	14	17.14

High-income Western countries	Girls	2016	14	17.21
High-income Western countries	Girls	1975	15	13.90
High-income Western countries	Girls	1976	15	14.01
High-income Western countries	Girls	1977	15	14.13
High-income Western countries	Girls	1978	15	14.24
High-income Western countries	Girls	1979	15	14.36
High-income Western countries	Girls	1980	15	14.47
High-income Western countries	Girls	1981	15	14.59
High-income Western countries	Girls	1982	15	14.71
High-income Western countries	Girls	1983	15	14.84
High-income Western countries	Girls	1984	15	14.97
High-income Western countries	Girls	1985	15	15.10
High-income Western countries	Girls	1986	15	15.24
High-income Western countries	Girls	1987	15	15.39
High-income Western countries	Girls	1988	15	15.54
High-income Western countries	Girls	1989	15	15.69
High-income Western countries	Girls	1990	15	15.85
High-income Western countries	Girls	1991	15	16.01
High-income Western countries	Girls	1992	15	16.18
High-income Western countries	Girls	1993	15	16.35
High-income Western countries	Girls	1994	15	16.52
High-income Western countries	Girls	1995	15	16.70
High-income Western countries	Girls	1996	15	16.87
High-income Western countries	Girls	1997	15	17.04
High-income Western countries	Girls	1998	15	17.20
High-income Western countries	Girls	1999	15	17.36
High-income Western countries	Girls	2000	15	17.52
High-income Western countries	Girls	2001	15	17.67
High-income Western countries	Girls	2002	15	17.81
High-income Western countries	Girls	2003	15	17.93
High-income Western countries	Girls	2004	15	18.05
High-income Western countries	Girls	2005	15	18.16
High-income Western countries	Girls	2006	15	18.27
High-income Western countries	Girls	2007	15	18.36
High-income Western countries	Girls	2008	15	18.45
High-income Western countries	Girls	2009	15	18.53
High-income Western countries	Girls	2010	15	18.61
High-income Western countries	Girls	2011	15	18.68
High-income Western countries	Girls	2012	15	18.75
High-income Western countries	Girls	2013	15	18.82
High-income Western countries	Girls	2014	15	18.89
High-income Western countries	Girls	2015	15	18.96
High-income Western countries	Girls	2016	15	19.03
High-income Western countries	Girls	1975	16	15.16
High-income Western countries	Girls	1976	16	15.28
High-income Western countries	Girls	1977	16	15.40
High-income Western countries	Girls	1978	16	15.52
High-income Western countries	Girls	1979	16	15.63
High-income Western countries	Girls	1980	16	15.75
High-income Western countries	Girls	1981	16	15.88
High-income Western countries	Girls	1982	16	16.00
High-income Western countries	Girls	1983	16	16.13

High-income Western countries	Girls	1984	16	16.26
High-income Western countries	Girls	1985	16	16.40
High-income Western countries	Girls	1986	16	16.55
High-income Western countries	Girls	1987	16	16.70
High-income Western countries	Girls	1988	16	16.85
High-income Western countries	Girls	1989	16	17.02
High-income Western countries	Girls	1990	16	17.18
High-income Western countries	Girls	1991	16	17.35
High-income Western countries	Girls	1992	16	17.52
High-income Western countries	Girls	1993	16	17.70
High-income Western countries	Girls	1994	16	17.88
High-income Western countries	Girls	1995	16	18.06
High-income Western countries	Girls	1996	16	18.23
High-income Western countries	Girls	1997	16	18.41
High-income Western countries	Girls	1998	16	18.58
High-income Western countries	Girls	1999	16	18.75
High-income Western countries	Girls	2000	16	18.91
High-income Western countries	Girls	2001	16	19.07
High-income Western countries	Girls	2002	16	19.21
High-income Western countries	Girls	2003	16	19.34
High-income Western countries	Girls	2004	16	19.47
High-income Western countries	Girls	2005	16	19.58
High-income Western countries	Girls	2006	16	19.69
High-income Western countries	Girls	2007	16	19.79
High-income Western countries	Girls	2008	16	19.88
High-income Western countries	Girls	2009	16	19.96
High-income Western countries	Girls	2010	16	20.04
High-income Western countries	Girls	2011	16	20.12
High-income Western countries	Girls	2012	16	20.19
High-income Western countries	Girls	2013	16	20.26
High-income Western countries	Girls	2014	16	20.33
High-income Western countries	Girls	2015	16	20.40
High-income Western countries	Girls	2016	16	20.47
High-income Western countries	Girls	1975	17	16.16
High-income Western countries	Girls	1976	17	16.29
High-income Western countries	Girls	1977	17	16.41
High-income Western countries	Girls	1978	17	16.53
High-income Western countries	Girls	1979	17	16.65
High-income Western countries	Girls	1980	17	16.78
High-income Western countries	Girls	1981	17	16.91
High-income Western countries	Girls	1982	17	17.04
High-income Western countries	Girls	1983	17	17.17
High-income Western countries	Girls	1984	17	17.31
High-income Western countries	Girls	1985	17	17.46
High-income Western countries	Girls	1986	17	17.61
High-income Western countries	Girls	1987	17	17.77
High-income Western countries	Girls	1988	17	17.93
High-income Western countries	Girls	1989	17	18.10
High-income Western countries	Girls	1990	17	18.27
High-income Western countries	Girls	1991	17	18.45
High-income Western countries	Girls	1992	17	18.63
High-income Western countries	Girls	1993	17	18.82

High-income Western countries	Girls	1994	17	19.01
High-income Western countries	Girls	1995	17	19.20
High-income Western countries	Girls	1996	17	19.39
High-income Western countries	Girls	1997	17	19.57
High-income Western countries	Girls	1998	17	19.76
High-income Western countries	Girls	1999	17	19.93
High-income Western countries	Girls	2000	17	20.10
High-income Western countries	Girls	2001	17	20.27
High-income Western countries	Girls	2002	17	20.42
High-income Western countries	Girls	2003	17	20.56
High-income Western countries	Girls	2004	17	20.69
High-income Western countries	Girls	2005	17	20.81
High-income Western countries	Girls	2006	17	20.92
High-income Western countries	Girls	2007	17	21.03
High-income Western countries	Girls	2008	17	21.12
High-income Western countries	Girls	2009	17	21.21
High-income Western countries	Girls	2010	17	21.29
High-income Western countries	Girls	2011	17	21.37
High-income Western countries	Girls	2012	17	21.45
High-income Western countries	Girls	2013	17	21.52
High-income Western countries	Girls	2014	17	21.59
High-income Western countries	Girls	2015	17	21.67
High-income Western countries	Girls	2016	17	21.74
High-income Western countries	Girls	1975	18	17.11
High-income Western countries	Girls	1976	18	17.24
High-income Western countries	Girls	1977	18	17.37
High-income Western countries	Girls	1978	18	17.51
High-income Western countries	Girls	1979	18	17.64
High-income Western countries	Girls	1980	18	17.78
High-income Western countries	Girls	1981	18	17.92
High-income Western countries	Girls	1982	18	18.06
High-income Western countries	Girls	1983	18	18.20
High-income Western countries	Girls	1984	18	18.35
High-income Western countries	Girls	1985	18	18.51
High-income Western countries	Girls	1986	18	18.68
High-income Western countries	Girls	1987	18	18.85
High-income Western countries	Girls	1988	18	19.03
High-income Western countries	Girls	1989	18	19.21
High-income Western countries	Girls	1990	18	19.40
High-income Western countries	Girls	1991	18	19.59
High-income Western countries	Girls	1992	18	19.79
High-income Western countries	Girls	1993	18	19.99
High-income Western countries	Girls	1994	18	20.20
High-income Western countries	Girls	1995	18	20.41
High-income Western countries	Girls	1996	18	20.61
High-income Western countries	Girls	1997	18	20.82
High-income Western countries	Girls	1998	18	21.02
High-income Western countries	Girls	1999	18	21.21
High-income Western countries	Girls	2000	18	21.39
High-income Western countries	Girls	2001	18	21.57
High-income Western countries	Girls	2002	18	21.74
High-income Western countries	Girls	2003	18	21.89

High-income Western countries	Girls	2004	18	22.03
High-income Western countries	Girls	2005	18	22.16
High-income Western countries	Girls	2006	18	22.28
High-income Western countries	Girls	2007	18	22.39
High-income Western countries	Girls	2008	18	22.50
High-income Western countries	Girls	2009	18	22.59
High-income Western countries	Girls	2010	18	22.68
High-income Western countries	Girls	2011	18	22.77
High-income Western countries	Girls	2012	18	22.85
High-income Western countries	Girls	2013	18	22.92
High-income Western countries	Girls	2014	18	23.00
High-income Western countries	Girls	2015	18	23.08
High-income Western countries	Girls	2016	18	23.16
High-income Western countries	Girls	1975	Average	13.38
High-income Western countries	Girls	1976	Average	13.49
High-income Western countries	Girls	1977	Average	13.60
High-income Western countries	Girls	1978	Average	13.71
High-income Western countries	Girls	1979	Average	13.82
High-income Western countries	Girls	1980	Average	13.94
High-income Western countries	Girls	1981	Average	14.06
High-income Western countries	Girls	1982	Average	14.19
High-income Western countries	Girls	1983	Average	14.31
High-income Western countries	Girls	1984	Average	14.45
High-income Western countries	Girls	1985	Average	14.58
High-income Western countries	Girls	1986	Average	14.72
High-income Western countries	Girls	1987	Average	14.87
High-income Western countries	Girls	1988	Average	15.02
High-income Western countries	Girls	1989	Average	15.18
High-income Western countries	Girls	1990	Average	15.33
High-income Western countries	Girls	1991	Average	15.49
High-income Western countries	Girls	1992	Average	15.66
High-income Western countries	Girls	1993	Average	15.82
High-income Western countries	Girls	1994	Average	15.99
High-income Western countries	Girls	1995	Average	16.16
High-income Western countries	Girls	1996	Average	16.33
High-income Western countries	Girls	1997	Average	16.50
High-income Western countries	Girls	1998	Average	16.66
High-income Western countries	Girls	1999	Average	16.82
High-income Western countries	Girls	2000	Average	16.98
High-income Western countries	Girls	2001	Average	17.12
High-income Western countries	Girls	2002	Average	17.26
High-income Western countries	Girls	2003	Average	17.39
High-income Western countries	Girls	2004	Average	17.51
High-income Western countries	Girls	2005	Average	17.61
High-income Western countries	Girls	2006	Average	17.71
High-income Western countries	Girls	2007	Average	17.80
High-income Western countries	Girls	2008	Average	17.89
High-income Western countries	Girls	2009	Average	17.97
High-income Western countries	Girls	2010	Average	18.04
High-income Western countries	Girls	2011	Average	18.11
High-income Western countries	Girls	2012	Average	18.18
High-income Western countries	Girls	2013	Average	18.25

High-income Western countries	Girls	2014	Average	18.31
High-income Western countries	Girls	2015	Average	18.38
High-income Western countries	Girls	2016	Average	18.45
High-income Western countries	Girls	2017	Average	18.51
High-income Western countries	Girls	2018	Average	18.58
High-income Western countries	Girls	2019	Average	18.65
High-income Western countries	Girls	2020	Average	18.72
High-income Western countries	Girls	2021	Average	18.79
High-income Western countries	Girls	2022	Average	18.86
High-income Western countries	Girls	2023	Average	18.93
High-income Western countries	Girls	2024	Average	19.01
High-income Western countries	Girls	2025	Average	19.08
High-income Western countries	Girls	2026	Average	19.15
High-income Western countries	Girls	2027	Average	19.22
High-income Western countries	Girls	2028	Average	19.30
High-income Western countries	Girls	2029	Average	19.37
High-income Western countries	Girls	2030	Average	19.44
Latin America and Caribbean	Boys	1975	12	6.72
Latin America and Caribbean	Boys	1976	12	6.82
Latin America and Caribbean	Boys	1977	12	6.92
Latin America and Caribbean	Boys	1978	12	7.02
Latin America and Caribbean	Boys	1979	12	7.13
Latin America and Caribbean	Boys	1980	12	7.24
Latin America and Caribbean	Boys	1981	12	7.36
Latin America and Caribbean	Boys	1982	12	7.49
Latin America and Caribbean	Boys	1983	12	7.61
Latin America and Caribbean	Boys	1984	12	7.74
Latin America and Caribbean	Boys	1985	12	7.88
Latin America and Caribbean	Boys	1986	12	8.01
Latin America and Caribbean	Boys	1987	12	8.14
Latin America and Caribbean	Boys	1988	12	8.28
Latin America and Caribbean	Boys	1989	12	8.41
Latin America and Caribbean	Boys	1990	12	8.55
Latin America and Caribbean	Boys	1991	12	8.69
Latin America and Caribbean	Boys	1992	12	8.82
Latin America and Caribbean	Boys	1993	12	8.96
Latin America and Caribbean	Boys	1994	12	9.10
Latin America and Caribbean	Boys	1995	12	9.25
Latin America and Caribbean	Boys	1996	12	9.41
Latin America and Caribbean	Boys	1997	12	9.57
Latin America and Caribbean	Boys	1998	12	9.73
Latin America and Caribbean	Boys	1999	12	9.90
Latin America and Caribbean	Boys	2000	12	10.06
Latin America and Caribbean	Boys	2001	12	10.23
Latin America and Caribbean	Boys	2002	12	10.40
Latin America and Caribbean	Boys	2003	12	10.56
Latin America and Caribbean	Boys	2004	12	10.73
Latin America and Caribbean	Boys	2005	12	10.90
Latin America and Caribbean	Boys	2006	12	11.07
Latin America and Caribbean	Boys	2007	12	11.23
Latin America and Caribbean	Boys	2008	12	11.40
Latin America and Caribbean	Boys	2009	12	11.58

Latin America and Caribbean	Boys	2010	12	11.75
Latin America and Caribbean	Boys	2011	12	11.92
Latin America and Caribbean	Boys	2012	12	12.10
Latin America and Caribbean	Boys	2013	12	12.27
Latin America and Caribbean	Boys	2014	12	12.45
Latin America and Caribbean	Boys	2015	12	12.63
Latin America and Caribbean	Boys	2016	12	12.82
Latin America and Caribbean	Boys	1975	13	8.29
Latin America and Caribbean	Boys	1976	13	8.40
Latin America and Caribbean	Boys	1977	13	8.51
Latin America and Caribbean	Boys	1978	13	8.63
Latin America and Caribbean	Boys	1979	13	8.76
Latin America and Caribbean	Boys	1980	13	8.90
Latin America and Caribbean	Boys	1981	13	9.04
Latin America and Caribbean	Boys	1982	13	9.18
Latin America and Caribbean	Boys	1983	13	9.33
Latin America and Caribbean	Boys	1984	13	9.48
Latin America and Caribbean	Boys	1985	13	9.64
Latin America and Caribbean	Boys	1986	13	9.79
Latin America and Caribbean	Boys	1987	13	9.95
Latin America and Caribbean	Boys	1988	13	10.11
Latin America and Caribbean	Boys	1989	13	10.27
Latin America and Caribbean	Boys	1990	13	10.43
Latin America and Caribbean	Boys	1991	13	10.58
Latin America and Caribbean	Boys	1992	13	10.74
Latin America and Caribbean	Boys	1993	13	10.91
Latin America and Caribbean	Boys	1994	13	11.07
Latin America and Caribbean	Boys	1995	13	11.25
Latin America and Caribbean	Boys	1996	13	11.43
Latin America and Caribbean	Boys	1997	13	11.62
Latin America and Caribbean	Boys	1998	13	11.81
Latin America and Caribbean	Boys	1999	13	12.01
Latin America and Caribbean	Boys	2000	13	12.21
Latin America and Caribbean	Boys	2001	13	12.41
Latin America and Caribbean	Boys	2002	13	12.61
Latin America and Caribbean	Boys	2003	13	12.81
Latin America and Caribbean	Boys	2004	13	13.00
Latin America and Caribbean	Boys	2005	13	13.21
Latin America and Caribbean	Boys	2006	13	13.41
Latin America and Caribbean	Boys	2007	13	13.61
Latin America and Caribbean	Boys	2008	13	13.81
Latin America and Caribbean	Boys	2009	13	14.02
Latin America and Caribbean	Boys	2010	13	14.23
Latin America and Caribbean	Boys	2011	13	14.44
Latin America and Caribbean	Boys	2012	13	14.65
Latin America and Caribbean	Boys	2013	13	14.86
Latin America and Caribbean	Boys	2014	13	15.08
Latin America and Caribbean	Boys	2015	13	15.31
Latin America and Caribbean	Boys	2016	13	15.53
Latin America and Caribbean	Boys	1975	14	10.16
Latin America and Caribbean	Boys	1976	14	10.28
Latin America and Caribbean	Boys	1977	14	10.41

Latin America and Caribbean	Boys	1978	14	10.54
Latin America and Caribbean	Boys	1979	14	10.69
Latin America and Caribbean	Boys	1980	14	10.83
Latin America and Caribbean	Boys	1981	14	10.99
Latin America and Caribbean	Boys	1982	14	11.15
Latin America and Caribbean	Boys	1983	14	11.31
Latin America and Caribbean	Boys	1984	14	11.48
Latin America and Caribbean	Boys	1985	14	11.66
Latin America and Caribbean	Boys	1986	14	11.83
Latin America and Caribbean	Boys	1987	14	12.00
Latin America and Caribbean	Boys	1988	14	12.18
Latin America and Caribbean	Boys	1989	14	12.35
Latin America and Caribbean	Boys	1990	14	12.53
Latin America and Caribbean	Boys	1991	14	12.71
Latin America and Caribbean	Boys	1992	14	12.88
Latin America and Caribbean	Boys	1993	14	13.06
Latin America and Caribbean	Boys	1994	14	13.25
Latin America and Caribbean	Boys	1995	14	13.44
Latin America and Caribbean	Boys	1996	14	13.64
Latin America and Caribbean	Boys	1997	14	13.85
Latin America and Caribbean	Boys	1998	14	14.07
Latin America and Caribbean	Boys	1999	14	14.29
Latin America and Caribbean	Boys	2000	14	14.51
Latin America and Caribbean	Boys	2001	14	14.74
Latin America and Caribbean	Boys	2002	14	14.96
Latin America and Caribbean	Boys	2003	14	15.18
Latin America and Caribbean	Boys	2004	14	15.40
Latin America and Caribbean	Boys	2005	14	15.63
Latin America and Caribbean	Boys	2006	14	15.85
Latin America and Caribbean	Boys	2007	14	16.08
Latin America and Caribbean	Boys	2008	14	16.31
Latin America and Caribbean	Boys	2009	14	16.54
Latin America and Caribbean	Boys	2010	14	16.78
Latin America and Caribbean	Boys	2011	14	17.02
Latin America and Caribbean	Boys	2012	14	17.26
Latin America and Caribbean	Boys	2013	14	17.50
Latin America and Caribbean	Boys	2014	14	17.76
Latin America and Caribbean	Boys	2015	14	18.01
Latin America and Caribbean	Boys	2016	14	18.27
Latin America and Caribbean	Boys	1975	15	12.27
Latin America and Caribbean	Boys	1976	15	12.40
Latin America and Caribbean	Boys	1977	15	12.53
Latin America and Caribbean	Boys	1978	15	12.66
Latin America and Caribbean	Boys	1979	15	12.81
Latin America and Caribbean	Boys	1980	15	12.95
Latin America and Caribbean	Boys	1981	15	13.11
Latin America and Caribbean	Boys	1982	15	13.27
Latin America and Caribbean	Boys	1983	15	13.44
Latin America and Caribbean	Boys	1984	15	13.62
Latin America and Caribbean	Boys	1985	15	13.80
Latin America and Caribbean	Boys	1986	15	13.99
Latin America and Caribbean	Boys	1987	15	14.18

Latin America and Caribbean	Boys	1988	15	14.38
Latin America and Caribbean	Boys	1989	15	14.58
Latin America and Caribbean	Boys	1990	15	14.78
Latin America and Caribbean	Boys	1991	15	14.98
Latin America and Caribbean	Boys	1992	15	15.18
Latin America and Caribbean	Boys	1993	15	15.38
Latin America and Caribbean	Boys	1994	15	15.57
Latin America and Caribbean	Boys	1995	15	15.77
Latin America and Caribbean	Boys	1996	15	15.97
Latin America and Caribbean	Boys	1997	15	16.18
Latin America and Caribbean	Boys	1998	15	16.38
Latin America and Caribbean	Boys	1999	15	16.60
Latin America and Caribbean	Boys	2000	15	16.82
Latin America and Caribbean	Boys	2001	15	17.04
Latin America and Caribbean	Boys	2002	15	17.28
Latin America and Caribbean	Boys	2003	15	17.52
Latin America and Caribbean	Boys	2004	15	17.76
Latin America and Caribbean	Boys	2005	15	18.01
Latin America and Caribbean	Boys	2006	15	18.26
Latin America and Caribbean	Boys	2007	15	18.51
Latin America and Caribbean	Boys	2008	15	18.76
Latin America and Caribbean	Boys	2009	15	19.02
Latin America and Caribbean	Boys	2010	15	19.28
Latin America and Caribbean	Boys	2011	15	19.53
Latin America and Caribbean	Boys	2012	15	19.80
Latin America and Caribbean	Boys	2013	15	20.06
Latin America and Caribbean	Boys	2014	15	20.33
Latin America and Caribbean	Boys	2015	15	20.60
Latin America and Caribbean	Boys	2016	15	20.88
Latin America and Caribbean	Boys	1975	16	14.51
Latin America and Caribbean	Boys	1976	16	14.64
Latin America and Caribbean	Boys	1977	16	14.78
Latin America and Caribbean	Boys	1978	16	14.92
Latin America and Caribbean	Boys	1979	16	15.06
Latin America and Caribbean	Boys	1980	16	15.22
Latin America and Caribbean	Boys	1981	16	15.38
Latin America and Caribbean	Boys	1982	16	15.54
Latin America and Caribbean	Boys	1983	16	15.71
Latin America and Caribbean	Boys	1984	16	15.89
Latin America and Caribbean	Boys	1985	16	16.08
Latin America and Caribbean	Boys	1986	16	16.28
Latin America and Caribbean	Boys	1987	16	16.48
Latin America and Caribbean	Boys	1988	16	16.68
Latin America and Caribbean	Boys	1989	16	16.89
Latin America and Caribbean	Boys	1990	16	17.09
Latin America and Caribbean	Boys	1991	16	17.29
Latin America and Caribbean	Boys	1992	16	17.50
Latin America and Caribbean	Boys	1993	16	17.70
Latin America and Caribbean	Boys	1994	16	17.90
Latin America and Caribbean	Boys	1995	16	18.10
Latin America and Caribbean	Boys	1996	16	18.30
Latin America and Caribbean	Boys	1997	16	18.51

Latin America and Caribbean	Boys	1998	16	18.72
Latin America and Caribbean	Boys	1999	16	18.94
Latin America and Caribbean	Boys	2000	16	19.16
Latin America and Caribbean	Boys	2001	16	19.39
Latin America and Caribbean	Boys	2002	16	19.63
Latin America and Caribbean	Boys	2003	16	19.88
Latin America and Caribbean	Boys	2004	16	20.13
Latin America and Caribbean	Boys	2005	16	20.38
Latin America and Caribbean	Boys	2006	16	20.64
Latin America and Caribbean	Boys	2007	16	20.89
Latin America and Caribbean	Boys	2008	16	21.15
Latin America and Caribbean	Boys	2009	16	21.42
Latin America and Caribbean	Boys	2010	16	21.68
Latin America and Caribbean	Boys	2011	16	21.95
Latin America and Caribbean	Boys	2012	16	22.22
Latin America and Caribbean	Boys	2013	16	22.49
Latin America and Caribbean	Boys	2014	16	22.77
Latin America and Caribbean	Boys	2015	16	23.05
Latin America and Caribbean	Boys	2016	16	23.34
Latin America and Caribbean	Boys	1975	17	16.83
Latin America and Caribbean	Boys	1976	17	16.96
Latin America and Caribbean	Boys	1977	17	17.10
Latin America and Caribbean	Boys	1978	17	17.24
Latin America and Caribbean	Boys	1979	17	17.39
Latin America and Caribbean	Boys	1980	17	17.55
Latin America and Caribbean	Boys	1981	17	17.71
Latin America and Caribbean	Boys	1982	17	17.88
Latin America and Caribbean	Boys	1983	17	18.06
Latin America and Caribbean	Boys	1984	17	18.24
Latin America and Caribbean	Boys	1985	17	18.43
Latin America and Caribbean	Boys	1986	17	18.63
Latin America and Caribbean	Boys	1987	17	18.83
Latin America and Caribbean	Boys	1988	17	19.04
Latin America and Caribbean	Boys	1989	17	19.25
Latin America and Caribbean	Boys	1990	17	19.46
Latin America and Caribbean	Boys	1991	17	19.66
Latin America and Caribbean	Boys	1992	17	19.87
Latin America and Caribbean	Boys	1993	17	20.07
Latin America and Caribbean	Boys	1994	17	20.27
Latin America and Caribbean	Boys	1995	17	20.47
Latin America and Caribbean	Boys	1996	17	20.68
Latin America and Caribbean	Boys	1997	17	20.89
Latin America and Caribbean	Boys	1998	17	21.10
Latin America and Caribbean	Boys	1999	17	21.32
Latin America and Caribbean	Boys	2000	17	21.55
Latin America and Caribbean	Boys	2001	17	21.78
Latin America and Caribbean	Boys	2002	17	22.03
Latin America and Caribbean	Boys	2003	17	22.28
Latin America and Caribbean	Boys	2004	17	22.53
Latin America and Caribbean	Boys	2005	17	22.79
Latin America and Caribbean	Boys	2006	17	23.05
Latin America and Caribbean	Boys	2007	17	23.31

Latin America and Caribbean	Boys	2008	17	23.57
Latin America and Caribbean	Boys	2009	17	23.84
Latin America and Caribbean	Boys	2010	17	24.11
Latin America and Caribbean	Boys	2011	17	24.38
Latin America and Caribbean	Boys	2012	17	24.65
Latin America and Caribbean	Boys	2013	17	24.93
Latin America and Caribbean	Boys	2014	17	25.21
Latin America and Caribbean	Boys	2015	17	25.50
Latin America and Caribbean	Boys	2016	17	25.79
Latin America and Caribbean	Boys	1975	18	19.17
Latin America and Caribbean	Boys	1976	18	19.31
Latin America and Caribbean	Boys	1977	18	19.46
Latin America and Caribbean	Boys	1978	18	19.60
Latin America and Caribbean	Boys	1979	18	19.76
Latin America and Caribbean	Boys	1980	18	19.92
Latin America and Caribbean	Boys	1981	18	20.09
Latin America and Caribbean	Boys	1982	18	20.27
Latin America and Caribbean	Boys	1983	18	20.45
Latin America and Caribbean	Boys	1984	18	20.64
Latin America and Caribbean	Boys	1985	18	20.84
Latin America and Caribbean	Boys	1986	18	21.05
Latin America and Caribbean	Boys	1987	18	21.26
Latin America and Caribbean	Boys	1988	18	21.47
Latin America and Caribbean	Boys	1989	18	21.69
Latin America and Caribbean	Boys	1990	18	21.91
Latin America and Caribbean	Boys	1991	18	22.12
Latin America and Caribbean	Boys	1992	18	22.33
Latin America and Caribbean	Boys	1993	18	22.53
Latin America and Caribbean	Boys	1994	18	22.74
Latin America and Caribbean	Boys	1995	18	22.95
Latin America and Caribbean	Boys	1996	18	23.16
Latin America and Caribbean	Boys	1997	18	23.38
Latin America and Caribbean	Boys	1998	18	23.59
Latin America and Caribbean	Boys	1999	18	23.82
Latin America and Caribbean	Boys	2000	18	24.05
Latin America and Caribbean	Boys	2001	18	24.30
Latin America and Caribbean	Boys	2002	18	24.55
Latin America and Caribbean	Boys	2003	18	24.80
Latin America and Caribbean	Boys	2004	18	25.06
Latin America and Caribbean	Boys	2005	18	25.33
Latin America and Caribbean	Boys	2006	18	25.59
Latin America and Caribbean	Boys	2007	18	25.86
Latin America and Caribbean	Boys	2008	18	26.13
Latin America and Caribbean	Boys	2009	18	26.40
Latin America and Caribbean	Boys	2010	18	26.68
Latin America and Caribbean	Boys	2011	18	26.96
Latin America and Caribbean	Boys	2012	18	27.23
Latin America and Caribbean	Boys	2013	18	27.52
Latin America and Caribbean	Boys	2014	18	27.80
Latin America and Caribbean	Boys	2015	18	28.10
Latin America and Caribbean	Boys	2016	18	28.40
Latin America and Caribbean	Boys	1975	Average	12.57

Latin America and Caribbean	Boys	1976	Average	12.69
Latin America and Caribbean	Boys	1977	Average	12.81
Latin America and Caribbean	Boys	1978	Average	12.95
Latin America and Caribbean	Boys	1979	Average	13.09
Latin America and Caribbean	Boys	1980	Average	13.23
Latin America and Caribbean	Boys	1981	Average	13.38
Latin America and Caribbean	Boys	1982	Average	13.54
Latin America and Caribbean	Boys	1983	Average	13.70
Latin America and Caribbean	Boys	1984	Average	13.87
Latin America and Caribbean	Boys	1985	Average	14.05
Latin America and Caribbean	Boys	1986	Average	14.22
Latin America and Caribbean	Boys	1987	Average	14.41
Latin America and Caribbean	Boys	1988	Average	14.59
Latin America and Caribbean	Boys	1989	Average	14.78
Latin America and Caribbean	Boys	1990	Average	14.96
Latin America and Caribbean	Boys	1991	Average	15.15
Latin America and Caribbean	Boys	1992	Average	15.33
Latin America and Caribbean	Boys	1993	Average	15.51
Latin America and Caribbean	Boys	1994	Average	15.70
Latin America and Caribbean	Boys	1995	Average	15.89
Latin America and Caribbean	Boys	1996	Average	16.09
Latin America and Caribbean	Boys	1997	Average	16.28
Latin America and Caribbean	Boys	1998	Average	16.49
Latin America and Caribbean	Boys	1999	Average	16.70
Latin America and Caribbean	Boys	2000	Average	16.91
Latin America and Caribbean	Boys	2001	Average	17.13
Latin America and Caribbean	Boys	2002	Average	17.35
Latin America and Caribbean	Boys	2003	Average	17.58
Latin America and Caribbean	Boys	2004	Average	17.80
Latin America and Caribbean	Boys	2005	Average	18.03
Latin America and Caribbean	Boys	2006	Average	18.27
Latin America and Caribbean	Boys	2007	Average	18.50
Latin America and Caribbean	Boys	2008	Average	18.73
Latin America and Caribbean	Boys	2009	Average	18.97
Latin America and Caribbean	Boys	2010	Average	19.21
Latin America and Caribbean	Boys	2011	Average	19.46
Latin America and Caribbean	Boys	2012	Average	19.70
Latin America and Caribbean	Boys	2013	Average	19.95
Latin America and Caribbean	Boys	2014	Average	20.20
Latin America and Caribbean	Boys	2015	Average	20.46
Latin America and Caribbean	Boys	2016	Average	20.72
Latin America and Caribbean	Boys	2017	Average	20.99
Latin America and Caribbean	Boys	2018	Average	21.26
Latin America and Caribbean	Boys	2019	Average	21.53
Latin America and Caribbean	Boys	2020	Average	21.81
Latin America and Caribbean	Boys	2021	Average	22.09
Latin America and Caribbean	Boys	2022	Average	22.37
Latin America and Caribbean	Boys	2023	Average	22.65
Latin America and Caribbean	Boys	2024	Average	22.93
Latin America and Caribbean	Boys	2025	Average	23.22
Latin America and Caribbean	Boys	2026	Average	23.50
Latin America and Caribbean	Boys	2027	Average	23.79

Latin America and Caribbean	Boys	2028	Average	24.07
Latin America and Caribbean	Boys	2029	Average	24.36
Latin America and Caribbean	Boys	2030	Average	24.65
Latin America and Caribbean	Girls	1975	12	6.97
Latin America and Caribbean	Girls	1976	12	7.06
Latin America and Caribbean	Girls	1977	12	7.15
Latin America and Caribbean	Girls	1978	12	7.24
Latin America and Caribbean	Girls	1979	12	7.34
Latin America and Caribbean	Girls	1980	12	7.43
Latin America and Caribbean	Girls	1981	12	7.54
Latin America and Caribbean	Girls	1982	12	7.64
Latin America and Caribbean	Girls	1983	12	7.75
Latin America and Caribbean	Girls	1984	12	7.85
Latin America and Caribbean	Girls	1985	12	7.96
Latin America and Caribbean	Girls	1986	12	8.07
Latin America and Caribbean	Girls	1987	12	8.17
Latin America and Caribbean	Girls	1988	12	8.28
Latin America and Caribbean	Girls	1989	12	8.38
Latin America and Caribbean	Girls	1990	12	8.49
Latin America and Caribbean	Girls	1991	12	8.59
Latin America and Caribbean	Girls	1992	12	8.70
Latin America and Caribbean	Girls	1993	12	8.80
Latin America and Caribbean	Girls	1994	12	8.91
Latin America and Caribbean	Girls	1995	12	9.02
Latin America and Caribbean	Girls	1996	12	9.13
Latin America and Caribbean	Girls	1997	12	9.24
Latin America and Caribbean	Girls	1998	12	9.36
Latin America and Caribbean	Girls	1999	12	9.48
Latin America and Caribbean	Girls	2000	12	9.59
Latin America and Caribbean	Girls	2001	12	9.71
Latin America and Caribbean	Girls	2002	12	9.82
Latin America and Caribbean	Girls	2003	12	9.93
Latin America and Caribbean	Girls	2004	12	10.03
Latin America and Caribbean	Girls	2005	12	10.14
Latin America and Caribbean	Girls	2006	12	10.25
Latin America and Caribbean	Girls	2007	12	10.35
Latin America and Caribbean	Girls	2008	12	10.45
Latin America and Caribbean	Girls	2009	12	10.56
Latin America and Caribbean	Girls	2010	12	10.66
Latin America and Caribbean	Girls	2011	12	10.77
Latin America and Caribbean	Girls	2012	12	10.88
Latin America and Caribbean	Girls	2013	12	10.99
Latin America and Caribbean	Girls	2014	12	11.11
Latin America and Caribbean	Girls	2015	12	11.23
Latin America and Caribbean	Girls	2016	12	11.35
Latin America and Caribbean	Girls	1975	13	8.58
Latin America and Caribbean	Girls	1976	13	8.68
Latin America and Caribbean	Girls	1977	13	8.78
Latin America and Caribbean	Girls	1978	13	8.89
Latin America and Caribbean	Girls	1979	13	9.00
Latin America and Caribbean	Girls	1980	13	9.12
Latin America and Caribbean	Girls	1981	13	9.23

Latin America and Caribbean	Girls	1982	13	9.35
Latin America and Caribbean	Girls	1983	13	9.47
Latin America and Caribbean	Girls	1984	13	9.59
Latin America and Caribbean	Girls	1985	13	9.72
Latin America and Caribbean	Girls	1986	13	9.84
Latin America and Caribbean	Girls	1987	13	9.96
Latin America and Caribbean	Girls	1988	13	10.08
Latin America and Caribbean	Girls	1989	13	10.20
Latin America and Caribbean	Girls	1990	13	10.32
Latin America and Caribbean	Girls	1991	13	10.44
Latin America and Caribbean	Girls	1992	13	10.56
Latin America and Caribbean	Girls	1993	13	10.67
Latin America and Caribbean	Girls	1994	13	10.80
Latin America and Caribbean	Girls	1995	13	10.92
Latin America and Caribbean	Girls	1996	13	11.05
Latin America and Caribbean	Girls	1997	13	11.18
Latin America and Caribbean	Girls	1998	13	11.31
Latin America and Caribbean	Girls	1999	13	11.44
Latin America and Caribbean	Girls	2000	13	11.58
Latin America and Caribbean	Girls	2001	13	11.70
Latin America and Caribbean	Girls	2002	13	11.83
Latin America and Caribbean	Girls	2003	13	11.96
Latin America and Caribbean	Girls	2004	13	12.08
Latin America and Caribbean	Girls	2005	13	12.20
Latin America and Caribbean	Girls	2006	13	12.32
Latin America and Caribbean	Girls	2007	13	12.44
Latin America and Caribbean	Girls	2008	13	12.56
Latin America and Caribbean	Girls	2009	13	12.67
Latin America and Caribbean	Girls	2010	13	12.80
Latin America and Caribbean	Girls	2011	13	12.92
Latin America and Caribbean	Girls	2012	13	13.05
Latin America and Caribbean	Girls	2013	13	13.17
Latin America and Caribbean	Girls	2014	13	13.31
Latin America and Caribbean	Girls	2015	13	13.44
Latin America and Caribbean	Girls	2016	13	13.58
Latin America and Caribbean	Girls	1975	14	10.25
Latin America and Caribbean	Girls	1976	14	10.36
Latin America and Caribbean	Girls	1977	14	10.47
Latin America and Caribbean	Girls	1978	14	10.59
Latin America and Caribbean	Girls	1979	14	10.71
Latin America and Caribbean	Girls	1980	14	10.83
Latin America and Caribbean	Girls	1981	14	10.96
Latin America and Caribbean	Girls	1982	14	11.09
Latin America and Caribbean	Girls	1983	14	11.22
Latin America and Caribbean	Girls	1984	14	11.35
Latin America and Caribbean	Girls	1985	14	11.48
Latin America and Caribbean	Girls	1986	14	11.62
Latin America and Caribbean	Girls	1987	14	11.75
Latin America and Caribbean	Girls	1988	14	11.88
Latin America and Caribbean	Girls	1989	14	12.01
Latin America and Caribbean	Girls	1990	14	12.14
Latin America and Caribbean	Girls	1991	14	12.26

Latin America and Caribbean	Girls	1992	14	12.39
Latin America and Caribbean	Girls	1993	14	12.52
Latin America and Caribbean	Girls	1994	14	12.65
Latin America and Caribbean	Girls	1995	14	12.78
Latin America and Caribbean	Girls	1996	14	12.92
Latin America and Caribbean	Girls	1997	14	13.06
Latin America and Caribbean	Girls	1998	14	13.21
Latin America and Caribbean	Girls	1999	14	13.35
Latin America and Caribbean	Girls	2000	14	13.49
Latin America and Caribbean	Girls	2001	14	13.63
Latin America and Caribbean	Girls	2002	14	13.77
Latin America and Caribbean	Girls	2003	14	13.90
Latin America and Caribbean	Girls	2004	14	14.04
Latin America and Caribbean	Girls	2005	14	14.17
Latin America and Caribbean	Girls	2006	14	14.29
Latin America and Caribbean	Girls	2007	14	14.42
Latin America and Caribbean	Girls	2008	14	14.55
Latin America and Caribbean	Girls	2009	14	14.68
Latin America and Caribbean	Girls	2010	14	14.81
Latin America and Caribbean	Girls	2011	14	14.94
Latin America and Caribbean	Girls	2012	14	15.08
Latin America and Caribbean	Girls	2013	14	15.22
Latin America and Caribbean	Girls	2014	14	15.36
Latin America and Caribbean	Girls	2015	14	15.51
Latin America and Caribbean	Girls	2016	14	15.66
Latin America and Caribbean	Girls	1975	15	11.76
Latin America and Caribbean	Girls	1976	15	11.87
Latin America and Caribbean	Girls	1977	15	11.99
Latin America and Caribbean	Girls	1978	15	12.10
Latin America and Caribbean	Girls	1979	15	12.23
Latin America and Caribbean	Girls	1980	15	12.35
Latin America and Caribbean	Girls	1981	15	12.48
Latin America and Caribbean	Girls	1982	15	12.61
Latin America and Caribbean	Girls	1983	15	12.75
Latin America and Caribbean	Girls	1984	15	12.88
Latin America and Caribbean	Girls	1985	15	13.02
Latin America and Caribbean	Girls	1986	15	13.17
Latin America and Caribbean	Girls	1987	15	13.31
Latin America and Caribbean	Girls	1988	15	13.46
Latin America and Caribbean	Girls	1989	15	13.60
Latin America and Caribbean	Girls	1990	15	13.75
Latin America and Caribbean	Girls	1991	15	13.89
Latin America and Caribbean	Girls	1992	15	14.03
Latin America and Caribbean	Girls	1993	15	14.16
Latin America and Caribbean	Girls	1994	15	14.30
Latin America and Caribbean	Girls	1995	15	14.43
Latin America and Caribbean	Girls	1996	15	14.56
Latin America and Caribbean	Girls	1997	15	14.69
Latin America and Caribbean	Girls	1998	15	14.83
Latin America and Caribbean	Girls	1999	15	14.96
Latin America and Caribbean	Girls	2000	15	15.10
Latin America and Caribbean	Girls	2001	15	15.24

Latin America and Caribbean	Girls	2002	15	15.38
Latin America and Caribbean	Girls	2003	15	15.52
Latin America and Caribbean	Girls	2004	15	15.67
Latin America and Caribbean	Girls	2005	15	15.81
Latin America and Caribbean	Girls	2006	15	15.95
Latin America and Caribbean	Girls	2007	15	16.09
Latin America and Caribbean	Girls	2008	15	16.23
Latin America and Caribbean	Girls	2009	15	16.37
Latin America and Caribbean	Girls	2010	15	16.52
Latin America and Caribbean	Girls	2011	15	16.66
Latin America and Caribbean	Girls	2012	15	16.80
Latin America and Caribbean	Girls	2013	15	16.95
Latin America and Caribbean	Girls	2014	15	17.09
Latin America and Caribbean	Girls	2015	15	17.24
Latin America and Caribbean	Girls	2016	15	17.39
Latin America and Caribbean	Girls	1975	16	12.95
Latin America and Caribbean	Girls	1976	16	13.06
Latin America and Caribbean	Girls	1977	16	13.18
Latin America and Caribbean	Girls	1978	16	13.30
Latin America and Caribbean	Girls	1979	16	13.43
Latin America and Caribbean	Girls	1980	16	13.56
Latin America and Caribbean	Girls	1981	16	13.69
Latin America and Caribbean	Girls	1982	16	13.82
Latin America and Caribbean	Girls	1983	16	13.96
Latin America and Caribbean	Girls	1984	16	14.10
Latin America and Caribbean	Girls	1985	16	14.25
Latin America and Caribbean	Girls	1986	16	14.40
Latin America and Caribbean	Girls	1987	16	14.55
Latin America and Caribbean	Girls	1988	16	14.70
Latin America and Caribbean	Girls	1989	16	14.85
Latin America and Caribbean	Girls	1990	16	14.99
Latin America and Caribbean	Girls	1991	16	15.14
Latin America and Caribbean	Girls	1992	16	15.28
Latin America and Caribbean	Girls	1993	16	15.42
Latin America and Caribbean	Girls	1994	16	15.56
Latin America and Caribbean	Girls	1995	16	15.70
Latin America and Caribbean	Girls	1996	16	15.83
Latin America and Caribbean	Girls	1997	16	15.97
Latin America and Caribbean	Girls	1998	16	16.11
Latin America and Caribbean	Girls	1999	16	16.24
Latin America and Caribbean	Girls	2000	16	16.39
Latin America and Caribbean	Girls	2001	16	16.53
Latin America and Caribbean	Girls	2002	16	16.67
Latin America and Caribbean	Girls	2003	16	16.82
Latin America and Caribbean	Girls	2004	16	16.97
Latin America and Caribbean	Girls	2005	16	17.11
Latin America and Caribbean	Girls	2006	16	17.26
Latin America and Caribbean	Girls	2007	16	17.41
Latin America and Caribbean	Girls	2008	16	17.55
Latin America and Caribbean	Girls	2009	16	17.70
Latin America and Caribbean	Girls	2010	16	17.85
Latin America and Caribbean	Girls	2011	16	17.99

Latin America and Caribbean	Girls	2012	16	18.14
Latin America and Caribbean	Girls	2013	16	18.29
Latin America and Caribbean	Girls	2014	16	18.44
Latin America and Caribbean	Girls	2015	16	18.59
Latin America and Caribbean	Girls	2016	16	18.75
Latin America and Caribbean	Girls	1975	17	13.82
Latin America and Caribbean	Girls	1976	17	13.94
Latin America and Caribbean	Girls	1977	17	14.06
Latin America and Caribbean	Girls	1978	17	14.19
Latin America and Caribbean	Girls	1979	17	14.32
Latin America and Caribbean	Girls	1980	17	14.46
Latin America and Caribbean	Girls	1981	17	14.59
Latin America and Caribbean	Girls	1982	17	14.74
Latin America and Caribbean	Girls	1983	17	14.88
Latin America and Caribbean	Girls	1984	17	15.03
Latin America and Caribbean	Girls	1985	17	15.18
Latin America and Caribbean	Girls	1986	17	15.33
Latin America and Caribbean	Girls	1987	17	15.49
Latin America and Caribbean	Girls	1988	17	15.65
Latin America and Caribbean	Girls	1989	17	15.80
Latin America and Caribbean	Girls	1990	17	15.96
Latin America and Caribbean	Girls	1991	17	16.11
Latin America and Caribbean	Girls	1992	17	16.26
Latin America and Caribbean	Girls	1993	17	16.40
Latin America and Caribbean	Girls	1994	17	16.55
Latin America and Caribbean	Girls	1995	17	16.69
Latin America and Caribbean	Girls	1996	17	16.83
Latin America and Caribbean	Girls	1997	17	16.98
Latin America and Caribbean	Girls	1998	17	17.12
Latin America and Caribbean	Girls	1999	17	17.26
Latin America and Caribbean	Girls	2000	17	17.41
Latin America and Caribbean	Girls	2001	17	17.56
Latin America and Caribbean	Girls	2002	17	17.72
Latin America and Caribbean	Girls	2003	17	17.87
Latin America and Caribbean	Girls	2004	17	18.02
Latin America and Caribbean	Girls	2005	17	18.18
Latin America and Caribbean	Girls	2006	17	18.33
Latin America and Caribbean	Girls	2007	17	18.48
Latin America and Caribbean	Girls	2008	17	18.64
Latin America and Caribbean	Girls	2009	17	18.79
Latin America and Caribbean	Girls	2010	17	18.95
Latin America and Caribbean	Girls	2011	17	19.10
Latin America and Caribbean	Girls	2012	17	19.25
Latin America and Caribbean	Girls	2013	17	19.41
Latin America and Caribbean	Girls	2014	17	19.56
Latin America and Caribbean	Girls	2015	17	19.72
Latin America and Caribbean	Girls	2016	17	19.88
Latin America and Caribbean	Girls	1975	18	14.52
Latin America and Caribbean	Girls	1976	18	14.65
Latin America and Caribbean	Girls	1977	18	14.78
Latin America and Caribbean	Girls	1978	18	14.92
Latin America and Caribbean	Girls	1979	18	15.06

Latin America and Caribbean	Girls	1980	18	15.21
Latin America and Caribbean	Girls	1981	18	15.36
Latin America and Caribbean	Girls	1982	18	15.51
Latin America and Caribbean	Girls	1983	18	15.67
Latin America and Caribbean	Girls	1984	18	15.83
Latin America and Caribbean	Girls	1985	18	15.99
Latin America and Caribbean	Girls	1986	18	16.16
Latin America and Caribbean	Girls	1987	18	16.33
Latin America and Caribbean	Girls	1988	18	16.50
Latin America and Caribbean	Girls	1989	18	16.67
Latin America and Caribbean	Girls	1990	18	16.84
Latin America and Caribbean	Girls	1991	18	17.00
Latin America and Caribbean	Girls	1992	18	17.16
Latin America and Caribbean	Girls	1993	18	17.32
Latin America and Caribbean	Girls	1994	18	17.48
Latin America and Caribbean	Girls	1995	18	17.63
Latin America and Caribbean	Girls	1996	18	17.79
Latin America and Caribbean	Girls	1997	18	17.94
Latin America and Caribbean	Girls	1998	18	18.10
Latin America and Caribbean	Girls	1999	18	18.26
Latin America and Caribbean	Girls	2000	18	18.42
Latin America and Caribbean	Girls	2001	18	18.58
Latin America and Caribbean	Girls	2002	18	18.75
Latin America and Caribbean	Girls	2003	18	18.91
Latin America and Caribbean	Girls	2004	18	19.08
Latin America and Caribbean	Girls	2005	18	19.25
Latin America and Caribbean	Girls	2006	18	19.42
Latin America and Caribbean	Girls	2007	18	19.58
Latin America and Caribbean	Girls	2008	18	19.75
Latin America and Caribbean	Girls	2009	18	19.92
Latin America and Caribbean	Girls	2010	18	20.08
Latin America and Caribbean	Girls	2011	18	20.25
Latin America and Caribbean	Girls	2012	18	20.41
Latin America and Caribbean	Girls	2013	18	20.58
Latin America and Caribbean	Girls	2014	18	20.75
Latin America and Caribbean	Girls	2015	18	20.92
Latin America and Caribbean	Girls	2016	18	21.09
Latin America and Caribbean	Girls	1975	Average	11.26
Latin America and Caribbean	Girls	1976	Average	11.37
Latin America and Caribbean	Girls	1977	Average	11.49
Latin America and Caribbean	Girls	1978	Average	11.60
Latin America and Caribbean	Girls	1979	Average	11.73
Latin America and Caribbean	Girls	1980	Average	11.85
Latin America and Caribbean	Girls	1981	Average	11.98
Latin America and Caribbean	Girls	1982	Average	12.11
Latin America and Caribbean	Girls	1983	Average	12.24
Latin America and Caribbean	Girls	1984	Average	12.38
Latin America and Caribbean	Girls	1985	Average	12.52
Latin America and Caribbean	Girls	1986	Average	12.65
Latin America and Caribbean	Girls	1987	Average	12.79
Latin America and Caribbean	Girls	1988	Average	12.93
Latin America and Caribbean	Girls	1989	Average	13.07

Latin America and Caribbean	Girls	1990	Average	13.21
Latin America and Caribbean	Girls	1991	Average	13.35
Latin America and Caribbean	Girls	1992	Average	13.48
Latin America and Caribbean	Girls	1993	Average	13.61
Latin America and Caribbean	Girls	1994	Average	13.75
Latin America and Caribbean	Girls	1995	Average	13.88
Latin America and Caribbean	Girls	1996	Average	14.02
Latin America and Caribbean	Girls	1997	Average	14.15
Latin America and Caribbean	Girls	1998	Average	14.29
Latin America and Caribbean	Girls	1999	Average	14.43
Latin America and Caribbean	Girls	2000	Average	14.57
Latin America and Caribbean	Girls	2001	Average	14.71
Latin America and Caribbean	Girls	2002	Average	14.85
Latin America and Caribbean	Girls	2003	Average	14.99
Latin America and Caribbean	Girls	2004	Average	15.13
Latin America and Caribbean	Girls	2005	Average	15.26
Latin America and Caribbean	Girls	2006	Average	15.40
Latin America and Caribbean	Girls	2007	Average	15.54
Latin America and Caribbean	Girls	2008	Average	15.68
Latin America and Caribbean	Girls	2009	Average	15.81
Latin America and Caribbean	Girls	2010	Average	15.95
Latin America and Caribbean	Girls	2011	Average	16.09
Latin America and Caribbean	Girls	2012	Average	16.23
Latin America and Caribbean	Girls	2013	Average	16.37
Latin America and Caribbean	Girls	2014	Average	16.52
Latin America and Caribbean	Girls	2015	Average	16.66
Latin America and Caribbean	Girls	2016	Average	16.81
Latin America and Caribbean	Girls	2017	Average	16.96
Latin America and Caribbean	Girls	2018	Average	17.12
Latin America and Caribbean	Girls	2019	Average	17.27
Latin America and Caribbean	Girls	2020	Average	17.42
Latin America and Caribbean	Girls	2021	Average	17.57
Latin America and Caribbean	Girls	2022	Average	17.72
Latin America and Caribbean	Girls	2023	Average	17.87
Latin America and Caribbean	Girls	2024	Average	18.02
Latin America and Caribbean	Girls	2025	Average	18.18
Latin America and Caribbean	Girls	2026	Average	18.33
Latin America and Caribbean	Girls	2027	Average	18.48
Latin America and Caribbean	Girls	2028	Average	18.63
Latin America and Caribbean	Girls	2029	Average	18.78
Latin America and Caribbean	Girls	2030	Average	18.93
Oceania	Boys	1975	12	6.08
Oceania	Boys	1976	12	6.13
Oceania	Boys	1977	12	6.19
Oceania	Boys	1978	12	6.25
Oceania	Boys	1979	12	6.31
Oceania	Boys	1980	12	6.37
Oceania	Boys	1981	12	6.43
Oceania	Boys	1982	12	6.49
Oceania	Boys	1983	12	6.56
Oceania	Boys	1984	12	6.64
Oceania	Boys	1985	12	6.72

Oceania	Boys	1986	12	6.80
Oceania	Boys	1987	12	6.90
Oceania	Boys	1988	12	7.00
Oceania	Boys	1989	12	7.11
Oceania	Boys	1990	12	7.22
Oceania	Boys	1991	12	7.35
Oceania	Boys	1992	12	7.47
Oceania	Boys	1993	12	7.60
Oceania	Boys	1994	12	7.73
Oceania	Boys	1995	12	7.87
Oceania	Boys	1996	12	8.00
Oceania	Boys	1997	12	8.14
Oceania	Boys	1998	12	8.28
Oceania	Boys	1999	12	8.42
Oceania	Boys	2000	12	8.57
Oceania	Boys	2001	12	8.73
Oceania	Boys	2002	12	8.89
Oceania	Boys	2003	12	9.05
Oceania	Boys	2004	12	9.23
Oceania	Boys	2005	12	9.40
Oceania	Boys	2006	12	9.58
Oceania	Boys	2007	12	9.77
Oceania	Boys	2008	12	9.96
Oceania	Boys	2009	12	10.16
Oceania	Boys	2010	12	10.37
Oceania	Boys	2011	12	10.58
Oceania	Boys	2012	12	10.81
Oceania	Boys	2013	12	11.04
Oceania	Boys	2014	12	11.27
Oceania	Boys	2015	12	11.52
Oceania	Boys	2016	12	11.76
Oceania	Boys	1975	13	7.58
Oceania	Boys	1976	13	7.65
Oceania	Boys	1977	13	7.71
Oceania	Boys	1978	13	7.78
Oceania	Boys	1979	13	7.85
Oceania	Boys	1980	13	7.92
Oceania	Boys	1981	13	7.99
Oceania	Boys	1982	13	8.07
Oceania	Boys	1983	13	8.15
Oceania	Boys	1984	13	8.23
Oceania	Boys	1985	13	8.32
Oceania	Boys	1986	13	8.43
Oceania	Boys	1987	13	8.54
Oceania	Boys	1988	13	8.65
Oceania	Boys	1989	13	8.78
Oceania	Boys	1990	13	8.92
Oceania	Boys	1991	13	9.06
Oceania	Boys	1992	13	9.20
Oceania	Boys	1993	13	9.36
Oceania	Boys	1994	13	9.51
Oceania	Boys	1995	13	9.67

Oceania	Boys	1996	13	9.83
Oceania	Boys	1997	13	9.99
Oceania	Boys	1998	13	10.15
Oceania	Boys	1999	13	10.32
Oceania	Boys	2000	13	10.50
Oceania	Boys	2001	13	10.68
Oceania	Boys	2002	13	10.87
Oceania	Boys	2003	13	11.07
Oceania	Boys	2004	13	11.27
Oceania	Boys	2005	13	11.48
Oceania	Boys	2006	13	11.70
Oceania	Boys	2007	13	11.92
Oceania	Boys	2008	13	12.15
Oceania	Boys	2009	13	12.39
Oceania	Boys	2010	13	12.64
Oceania	Boys	2011	13	12.90
Oceania	Boys	2012	13	13.17
Oceania	Boys	2013	13	13.45
Oceania	Boys	2014	13	13.74
Oceania	Boys	2015	13	14.04
Oceania	Boys	2016	13	14.35
Oceania	Boys	1975	14	9.46
Oceania	Boys	1976	14	9.53
Oceania	Boys	1977	14	9.61
Oceania	Boys	1978	14	9.68
Oceania	Boys	1979	14	9.76
Oceania	Boys	1980	14	9.84
Oceania	Boys	1981	14	9.92
Oceania	Boys	1982	14	10.00
Oceania	Boys	1983	14	10.09
Oceania	Boys	1984	14	10.18
Oceania	Boys	1985	14	10.28
Oceania	Boys	1986	14	10.40
Oceania	Boys	1987	14	10.52
Oceania	Boys	1988	14	10.65
Oceania	Boys	1989	14	10.79
Oceania	Boys	1990	14	10.94
Oceania	Boys	1991	14	11.09
Oceania	Boys	1992	14	11.26
Oceania	Boys	1993	14	11.42
Oceania	Boys	1994	14	11.59
Oceania	Boys	1995	14	11.77
Oceania	Boys	1996	14	11.94
Oceania	Boys	1997	14	12.12
Oceania	Boys	1998	14	12.30
Oceania	Boys	1999	14	12.49
Oceania	Boys	2000	14	12.69
Oceania	Boys	2001	14	12.89
Oceania	Boys	2002	14	13.10
Oceania	Boys	2003	14	13.32
Oceania	Boys	2004	14	13.55
Oceania	Boys	2005	14	13.79

Oceania	Boys	2006	14	14.03
Oceania	Boys	2007	14	14.28
Oceania	Boys	2008	14	14.54
Oceania	Boys	2009	14	14.81
Oceania	Boys	2010	14	15.09
Oceania	Boys	2011	14	15.39
Oceania	Boys	2012	14	15.70
Oceania	Boys	2013	14	16.02
Oceania	Boys	2014	14	16.35
Oceania	Boys	2015	14	16.70
Oceania	Boys	2016	14	17.06
Oceania	Boys	1975	15	11.62
Oceania	Boys	1976	15	11.70
Oceania	Boys	1977	15	11.79
Oceania	Boys	1978	15	11.87
Oceania	Boys	1979	15	11.96
Oceania	Boys	1980	15	12.05
Oceania	Boys	1981	15	12.15
Oceania	Boys	1982	15	12.25
Oceania	Boys	1983	15	12.36
Oceania	Boys	1984	15	12.47
Oceania	Boys	1985	15	12.58
Oceania	Boys	1986	15	12.69
Oceania	Boys	1987	15	12.81
Oceania	Boys	1988	15	12.93
Oceania	Boys	1989	15	13.06
Oceania	Boys	1990	15	13.20
Oceania	Boys	1991	15	13.36
Oceania	Boys	1992	15	13.51
Oceania	Boys	1993	15	13.68
Oceania	Boys	1994	15	13.85
Oceania	Boys	1995	15	14.04
Oceania	Boys	1996	15	14.22
Oceania	Boys	1997	15	14.42
Oceania	Boys	1998	15	14.62
Oceania	Boys	1999	15	14.82
Oceania	Boys	2000	15	15.04
Oceania	Boys	2001	15	15.26
Oceania	Boys	2002	15	15.48
Oceania	Boys	2003	15	15.71
Oceania	Boys	2004	15	15.95
Oceania	Boys	2005	15	16.21
Oceania	Boys	2006	15	16.47
Oceania	Boys	2007	15	16.75
Oceania	Boys	2008	15	17.03
Oceania	Boys	2009	15	17.33
Oceania	Boys	2010	15	17.64
Oceania	Boys	2011	15	17.95
Oceania	Boys	2012	15	18.28
Oceania	Boys	2013	15	18.62
Oceania	Boys	2014	15	18.98
Oceania	Boys	2015	15	19.35

Oceania	Boys	2016	15	19.74
Oceania	Boys	1975	16	13.99
Oceania	Boys	1976	16	14.08
Oceania	Boys	1977	16	14.16
Oceania	Boys	1978	16	14.25
Oceania	Boys	1979	16	14.35
Oceania	Boys	1980	16	14.44
Oceania	Boys	1981	16	14.54
Oceania	Boys	1982	16	14.65
Oceania	Boys	1983	16	14.76
Oceania	Boys	1984	16	14.87
Oceania	Boys	1985	16	14.99
Oceania	Boys	1986	16	15.10
Oceania	Boys	1987	16	15.22
Oceania	Boys	1988	16	15.34
Oceania	Boys	1989	16	15.47
Oceania	Boys	1990	16	15.62
Oceania	Boys	1991	16	15.77
Oceania	Boys	1992	16	15.93
Oceania	Boys	1993	16	16.10
Oceania	Boys	1994	16	16.28
Oceania	Boys	1995	16	16.46
Oceania	Boys	1996	16	16.65
Oceania	Boys	1997	16	16.85
Oceania	Boys	1998	16	17.06
Oceania	Boys	1999	16	17.27
Oceania	Boys	2000	16	17.48
Oceania	Boys	2001	16	17.71
Oceania	Boys	2002	16	17.94
Oceania	Boys	2003	16	18.18
Oceania	Boys	2004	16	18.42
Oceania	Boys	2005	16	18.68
Oceania	Boys	2006	16	18.95
Oceania	Boys	2007	16	19.24
Oceania	Boys	2008	16	19.53
Oceania	Boys	2009	16	19.84
Oceania	Boys	2010	16	20.15
Oceania	Boys	2011	16	20.48
Oceania	Boys	2012	16	20.82
Oceania	Boys	2013	16	21.17
Oceania	Boys	2014	16	21.54
Oceania	Boys	2015	16	21.93
Oceania	Boys	2016	16	22.34
Oceania	Boys	1975	17	16.45
Oceania	Boys	1976	17	16.54
Oceania	Boys	1977	17	16.63
Oceania	Boys	1978	17	16.72
Oceania	Boys	1979	17	16.82
Oceania	Boys	1980	17	16.92
Oceania	Boys	1981	17	17.02
Oceania	Boys	1982	17	17.13
Oceania	Boys	1983	17	17.25

Oceania	Boys	1984	17	17.36
Oceania	Boys	1985	17	17.47
Oceania	Boys	1986	17	17.59
Oceania	Boys	1987	17	17.71
Oceania	Boys	1988	17	17.84
Oceania	Boys	1989	17	17.97
Oceania	Boys	1990	17	18.12
Oceania	Boys	1991	17	18.27
Oceania	Boys	1992	17	18.43
Oceania	Boys	1993	17	18.61
Oceania	Boys	1994	17	18.78
Oceania	Boys	1995	17	18.97
Oceania	Boys	1996	17	19.16
Oceania	Boys	1997	17	19.36
Oceania	Boys	1998	17	19.57
Oceania	Boys	1999	17	19.78
Oceania	Boys	2000	17	20.00
Oceania	Boys	2001	17	20.23
Oceania	Boys	2002	17	20.46
Oceania	Boys	2003	17	20.70
Oceania	Boys	2004	17	20.95
Oceania	Boys	2005	17	21.22
Oceania	Boys	2006	17	21.49
Oceania	Boys	2007	17	21.78
Oceania	Boys	2008	17	22.08
Oceania	Boys	2009	17	22.39
Oceania	Boys	2010	17	22.71
Oceania	Boys	2011	17	23.05
Oceania	Boys	2012	17	23.39
Oceania	Boys	2013	17	23.76
Oceania	Boys	2014	17	24.14
Oceania	Boys	2015	17	24.54
Oceania	Boys	2016	17	24.96
Oceania	Boys	1975	18	18.91
Oceania	Boys	1976	18	19.00
Oceania	Boys	1977	18	19.10
Oceania	Boys	1978	18	19.20
Oceania	Boys	1979	18	19.30
Oceania	Boys	1980	18	19.41
Oceania	Boys	1981	18	19.52
Oceania	Boys	1982	18	19.63
Oceania	Boys	1983	18	19.75
Oceania	Boys	1984	18	19.87
Oceania	Boys	1985	18	19.99
Oceania	Boys	1986	18	20.11
Oceania	Boys	1987	18	20.23
Oceania	Boys	1988	18	20.36
Oceania	Boys	1989	18	20.50
Oceania	Boys	1990	18	20.65
Oceania	Boys	1991	18	20.81
Oceania	Boys	1992	18	20.98
Oceania	Boys	1993	18	21.16

Oceania	Boys	1994	18	21.34
Oceania	Boys	1995	18	21.54
Oceania	Boys	1996	18	21.73
Oceania	Boys	1997	18	21.94
Oceania	Boys	1998	18	22.16
Oceania	Boys	1999	18	22.38
Oceania	Boys	2000	18	22.60
Oceania	Boys	2001	18	22.84
Oceania	Boys	2002	18	23.08
Oceania	Boys	2003	18	23.33
Oceania	Boys	2004	18	23.59
Oceania	Boys	2005	18	23.86
Oceania	Boys	2006	18	24.14
Oceania	Boys	2007	18	24.44
Oceania	Boys	2008	18	24.75
Oceania	Boys	2009	18	25.07
Oceania	Boys	2010	18	25.40
Oceania	Boys	2011	18	25.74
Oceania	Boys	2012	18	26.10
Oceania	Boys	2013	18	26.47
Oceania	Boys	2014	18	26.86
Oceania	Boys	2015	18	27.28
Oceania	Boys	2016	18	27.71
Oceania	Boys	1975	Average	12.01
Oceania	Boys	1976	Average	12.09
Oceania	Boys	1977	Average	12.17
Oceania	Boys	1978	Average	12.25
Oceania	Boys	1979	Average	12.33
Oceania	Boys	1980	Average	12.42
Oceania	Boys	1981	Average	12.51
Oceania	Boys	1982	Average	12.60
Oceania	Boys	1983	Average	12.70
Oceania	Boys	1984	Average	12.80
Oceania	Boys	1985	Average	12.91
Oceania	Boys	1986	Average	13.02
Oceania	Boys	1987	Average	13.13
Oceania	Boys	1988	Average	13.25
Oceania	Boys	1989	Average	13.38
Oceania	Boys	1990	Average	13.53
Oceania	Boys	1991	Average	13.67
Oceania	Boys	1992	Average	13.83
Oceania	Boys	1993	Average	13.99
Oceania	Boys	1994	Average	14.16
Oceania	Boys	1995	Average	14.33
Oceania	Boys	1996	Average	14.51
Oceania	Boys	1997	Average	14.69
Oceania	Boys	1998	Average	14.88
Oceania	Boys	1999	Average	15.07
Oceania	Boys	2000	Average	15.27
Oceania	Boys	2001	Average	15.47
Oceania	Boys	2002	Average	15.69
Oceania	Boys	2003	Average	15.91

Oceania	Boys	2004	Average	16.14
Oceania	Boys	2005	Average	16.38
Oceania	Boys	2006	Average	16.62
Oceania	Boys	2007	Average	16.88
Oceania	Boys	2008	Average	17.15
Oceania	Boys	2009	Average	17.43
Oceania	Boys	2010	Average	17.71
Oceania	Boys	2011	Average	18.01
Oceania	Boys	2012	Average	18.32
Oceania	Boys	2013	Average	18.65
Oceania	Boys	2014	Average	18.98
Oceania	Boys	2015	Average	19.34
Oceania	Boys	2016	Average	19.70
Oceania	Boys	2017	Average	20.07
Oceania	Boys	2018	Average	20.45
Oceania	Boys	2019	Average	20.82
Oceania	Boys	2020	Average	21.20
Oceania	Boys	2021	Average	21.59
Oceania	Boys	2022	Average	21.97
Oceania	Boys	2023	Average	22.36
Oceania	Boys	2024	Average	22.75
Oceania	Boys	2025	Average	23.15
Oceania	Boys	2026	Average	23.54
Oceania	Boys	2027	Average	23.94
Oceania	Boys	2028	Average	24.35
Oceania	Boys	2029	Average	24.75
Oceania	Boys	2030	Average	25.16
Oceania	Girls	1975	12	7.11
Oceania	Girls	1976	12	7.20
Oceania	Girls	1977	12	7.30
Oceania	Girls	1978	12	7.39
Oceania	Girls	1979	12	7.49
Oceania	Girls	1980	12	7.58
Oceania	Girls	1981	12	7.68
Oceania	Girls	1982	12	7.77
Oceania	Girls	1983	12	7.87
Oceania	Girls	1984	12	7.98
Oceania	Girls	1985	12	8.09
Oceania	Girls	1986	12	8.21
Oceania	Girls	1987	12	8.34
Oceania	Girls	1988	12	8.47
Oceania	Girls	1989	12	8.60
Oceania	Girls	1990	12	8.75
Oceania	Girls	1991	12	8.89
Oceania	Girls	1992	12	9.04
Oceania	Girls	1993	12	9.19
Oceania	Girls	1994	12	9.34
Oceania	Girls	1995	12	9.49
Oceania	Girls	1996	12	9.64
Oceania	Girls	1997	12	9.78
Oceania	Girls	1998	12	9.93
Oceania	Girls	1999	12	10.08

Oceania	Girls	2000	12	10.23
Oceania	Girls	2001	12	10.38
Oceania	Girls	2002	12	10.54
Oceania	Girls	2003	12	10.70
Oceania	Girls	2004	12	10.87
Oceania	Girls	2005	12	11.03
Oceania	Girls	2006	12	11.19
Oceania	Girls	2007	12	11.36
Oceania	Girls	2008	12	11.53
Oceania	Girls	2009	12	11.70
Oceania	Girls	2010	12	11.87
Oceania	Girls	2011	12	12.05
Oceania	Girls	2012	12	12.23
Oceania	Girls	2013	12	12.42
Oceania	Girls	2014	12	12.61
Oceania	Girls	2015	12	12.80
Oceania	Girls	2016	12	13.00
Oceania	Girls	1975	13	8.77
Oceania	Girls	1976	13	8.88
Oceania	Girls	1977	13	8.99
Oceania	Girls	1978	13	9.10
Oceania	Girls	1979	13	9.21
Oceania	Girls	1980	13	9.32
Oceania	Girls	1981	13	9.43
Oceania	Girls	1982	13	9.54
Oceania	Girls	1983	13	9.66
Oceania	Girls	1984	13	9.78
Oceania	Girls	1985	13	9.91
Oceania	Girls	1986	13	10.05
Oceania	Girls	1987	13	10.20
Oceania	Girls	1988	13	10.35
Oceania	Girls	1989	13	10.51
Oceania	Girls	1990	13	10.67
Oceania	Girls	1991	13	10.84
Oceania	Girls	1992	13	11.01
Oceania	Girls	1993	13	11.18
Oceania	Girls	1994	13	11.35
Oceania	Girls	1995	13	11.53
Oceania	Girls	1996	13	11.70
Oceania	Girls	1997	13	11.87
Oceania	Girls	1998	13	12.04
Oceania	Girls	1999	13	12.21
Oceania	Girls	2000	13	12.39
Oceania	Girls	2001	13	12.57
Oceania	Girls	2002	13	12.75
Oceania	Girls	2003	13	12.93
Oceania	Girls	2004	13	13.12
Oceania	Girls	2005	13	13.31
Oceania	Girls	2006	13	13.50
Oceania	Girls	2007	13	13.70
Oceania	Girls	2008	13	13.89
Oceania	Girls	2009	13	14.09

Oceania	Girls	2010	13	14.29
Oceania	Girls	2011	13	14.50
Oceania	Girls	2012	13	14.71
Oceania	Girls	2013	13	14.93
Oceania	Girls	2014	13	15.16
Oceania	Girls	2015	13	15.39
Oceania	Girls	2016	13	15.62
Oceania	Girls	1975	14	10.48
Oceania	Girls	1976	14	10.60
Oceania	Girls	1977	14	10.72
Oceania	Girls	1978	14	10.84
Oceania	Girls	1979	14	10.97
Oceania	Girls	1980	14	11.09
Oceania	Girls	1981	14	11.21
Oceania	Girls	1982	14	11.33
Oceania	Girls	1983	14	11.46
Oceania	Girls	1984	14	11.60
Oceania	Girls	1985	14	11.74
Oceania	Girls	1986	14	11.89
Oceania	Girls	1987	14	12.05
Oceania	Girls	1988	14	12.22
Oceania	Girls	1989	14	12.39
Oceania	Girls	1990	14	12.57
Oceania	Girls	1991	14	12.76
Oceania	Girls	1992	14	12.94
Oceania	Girls	1993	14	13.13
Oceania	Girls	1994	14	13.32
Oceania	Girls	1995	14	13.51
Oceania	Girls	1996	14	13.70
Oceania	Girls	1997	14	13.89
Oceania	Girls	1998	14	14.08
Oceania	Girls	1999	14	14.26
Oceania	Girls	2000	14	14.46
Oceania	Girls	2001	14	14.65
Oceania	Girls	2002	14	14.85
Oceania	Girls	2003	14	15.06
Oceania	Girls	2004	14	15.26
Oceania	Girls	2005	14	15.47
Oceania	Girls	2006	14	15.68
Oceania	Girls	2007	14	15.89
Oceania	Girls	2008	14	16.11
Oceania	Girls	2009	14	16.33
Oceania	Girls	2010	14	16.55
Oceania	Girls	2011	14	16.78
Oceania	Girls	2012	14	17.02
Oceania	Girls	2013	14	17.27
Oceania	Girls	2014	14	17.52
Oceania	Girls	2015	14	17.77
Oceania	Girls	2016	14	18.03
Oceania	Girls	1975	15	12.02
Oceania	Girls	1976	15	12.15
Oceania	Girls	1977	15	12.28

Oceania	Girls	1978	15	12.41
Oceania	Girls	1979	15	12.54
Oceania	Girls	1980	15	12.68
Oceania	Girls	1981	15	12.82
Oceania	Girls	1982	15	12.97
Oceania	Girls	1983	15	13.12
Oceania	Girls	1984	15	13.27
Oceania	Girls	1985	15	13.42
Oceania	Girls	1986	15	13.57
Oceania	Girls	1987	15	13.73
Oceania	Girls	1988	15	13.89
Oceania	Girls	1989	15	14.05
Oceania	Girls	1990	15	14.23
Oceania	Girls	1991	15	14.41
Oceania	Girls	1992	15	14.60
Oceania	Girls	1993	15	14.79
Oceania	Girls	1994	15	14.99
Oceania	Girls	1995	15	15.19
Oceania	Girls	1996	15	15.39
Oceania	Girls	1997	15	15.60
Oceania	Girls	1998	15	15.80
Oceania	Girls	1999	15	16.01
Oceania	Girls	2000	15	16.22
Oceania	Girls	2001	15	16.43
Oceania	Girls	2002	15	16.64
Oceania	Girls	2003	15	16.85
Oceania	Girls	2004	15	17.07
Oceania	Girls	2005	15	17.29
Oceania	Girls	2006	15	17.52
Oceania	Girls	2007	15	17.75
Oceania	Girls	2008	15	17.99
Oceania	Girls	2009	15	18.23
Oceania	Girls	2010	15	18.47
Oceania	Girls	2011	15	18.71
Oceania	Girls	2012	15	18.96
Oceania	Girls	2013	15	19.22
Oceania	Girls	2014	15	19.48
Oceania	Girls	2015	15	19.75
Oceania	Girls	2016	15	20.03
Oceania	Girls	1975	16	13.25
Oceania	Girls	1976	16	13.38
Oceania	Girls	1977	16	13.51
Oceania	Girls	1978	16	13.65
Oceania	Girls	1979	16	13.78
Oceania	Girls	1980	16	13.93
Oceania	Girls	1981	16	14.07
Oceania	Girls	1982	16	14.22
Oceania	Girls	1983	16	14.38
Oceania	Girls	1984	16	14.53
Oceania	Girls	1985	16	14.69
Oceania	Girls	1986	16	14.85
Oceania	Girls	1987	16	15.01

Oceania	Girls	1988	16	15.17
Oceania	Girls	1989	16	15.34
Oceania	Girls	1990	16	15.53
Oceania	Girls	1991	16	15.72
Oceania	Girls	1992	16	15.91
Oceania	Girls	1993	16	16.11
Oceania	Girls	1994	16	16.32
Oceania	Girls	1995	16	16.52
Oceania	Girls	1996	16	16.73
Oceania	Girls	1997	16	16.94
Oceania	Girls	1998	16	17.16
Oceania	Girls	1999	16	17.37
Oceania	Girls	2000	16	17.59
Oceania	Girls	2001	16	17.81
Oceania	Girls	2002	16	18.03
Oceania	Girls	2003	16	18.25
Oceania	Girls	2004	16	18.47
Oceania	Girls	2005	16	18.71
Oceania	Girls	2006	16	18.94
Oceania	Girls	2007	16	19.18
Oceania	Girls	2008	16	19.43
Oceania	Girls	2009	16	19.68
Oceania	Girls	2010	16	19.93
Oceania	Girls	2011	16	20.18
Oceania	Girls	2012	16	20.44
Oceania	Girls	2013	16	20.71
Oceania	Girls	2014	16	20.98
Oceania	Girls	2015	16	21.27
Oceania	Girls	2016	16	21.56
Oceania	Girls	1975	17	14.16
Oceania	Girls	1976	17	14.30
Oceania	Girls	1977	17	14.44
Oceania	Girls	1978	17	14.58
Oceania	Girls	1979	17	14.72
Oceania	Girls	1980	17	14.87
Oceania	Girls	1981	17	15.02
Oceania	Girls	1982	17	15.17
Oceania	Girls	1983	17	15.33
Oceania	Girls	1984	17	15.50
Oceania	Girls	1985	17	15.66
Oceania	Girls	1986	17	15.82
Oceania	Girls	1987	17	15.99
Oceania	Girls	1988	17	16.16
Oceania	Girls	1989	17	16.35
Oceania	Girls	1990	17	16.54
Oceania	Girls	1991	17	16.74
Oceania	Girls	1992	17	16.94
Oceania	Girls	1993	17	17.15
Oceania	Girls	1994	17	17.36
Oceania	Girls	1995	17	17.58
Oceania	Girls	1996	17	17.80
Oceania	Girls	1997	17	18.02

Oceania	Girls	1998	17	18.24
Oceania	Girls	1999	17	18.47
Oceania	Girls	2000	17	18.70
Oceania	Girls	2001	17	18.93
Oceania	Girls	2002	17	19.16
Oceania	Girls	2003	17	19.39
Oceania	Girls	2004	17	19.62
Oceania	Girls	2005	17	19.87
Oceania	Girls	2006	17	20.11
Oceania	Girls	2007	17	20.37
Oceania	Girls	2008	17	20.63
Oceania	Girls	2009	17	20.89
Oceania	Girls	2010	17	21.15
Oceania	Girls	2011	17	21.42
Oceania	Girls	2012	17	21.69
Oceania	Girls	2013	17	21.97
Oceania	Girls	2014	17	22.25
Oceania	Girls	2015	17	22.55
Oceania	Girls	2016	17	22.85
Oceania	Girls	1975	18	14.91
Oceania	Girls	1976	18	15.06
Oceania	Girls	1977	18	15.21
Oceania	Girls	1978	18	15.36
Oceania	Girls	1979	18	15.52
Oceania	Girls	1980	18	15.68
Oceania	Girls	1981	18	15.84
Oceania	Girls	1982	18	16.01
Oceania	Girls	1983	18	16.18
Oceania	Girls	1984	18	16.36
Oceania	Girls	1985	18	16.53
Oceania	Girls	1986	18	16.71
Oceania	Girls	1987	18	16.89
Oceania	Girls	1988	18	17.08
Oceania	Girls	1989	18	17.27
Oceania	Girls	1990	18	17.48
Oceania	Girls	1991	18	17.70
Oceania	Girls	1992	18	17.92
Oceania	Girls	1993	18	18.14
Oceania	Girls	1994	18	18.37
Oceania	Girls	1995	18	18.61
Oceania	Girls	1996	18	18.85
Oceania	Girls	1997	18	19.09
Oceania	Girls	1998	18	19.33
Oceania	Girls	1999	18	19.57
Oceania	Girls	2000	18	19.82
Oceania	Girls	2001	18	20.06
Oceania	Girls	2002	18	20.31
Oceania	Girls	2003	18	20.56
Oceania	Girls	2004	18	20.82
Oceania	Girls	2005	18	21.08
Oceania	Girls	2006	18	21.34
Oceania	Girls	2007	18	21.62

Oceania	Girls	2008	18	21.89
Oceania	Girls	2009	18	22.17
Oceania	Girls	2010	18	22.45
Oceania	Girls	2011	18	22.74
Oceania	Girls	2012	18	23.02
Oceania	Girls	2013	18	23.32
Oceania	Girls	2014	18	23.62
Oceania	Girls	2015	18	23.94
Oceania	Girls	2016	18	24.26
Oceania	Girls	1975	Average	11.53
Oceania	Girls	1976	Average	11.65
Oceania	Girls	1977	Average	11.78
Oceania	Girls	1978	Average	11.90
Oceania	Girls	1979	Average	12.03
Oceania	Girls	1980	Average	12.16
Oceania	Girls	1981	Average	12.30
Oceania	Girls	1982	Average	12.43
Oceania	Girls	1983	Average	12.57
Oceania	Girls	1984	Average	12.72
Oceania	Girls	1985	Average	12.86
Oceania	Girls	1986	Average	13.02
Oceania	Girls	1987	Average	13.17
Oceania	Girls	1988	Average	13.33
Oceania	Girls	1989	Average	13.50
Oceania	Girls	1990	Average	13.68
Oceania	Girls	1991	Average	13.86
Oceania	Girls	1992	Average	14.05
Oceania	Girls	1993	Average	14.24
Oceania	Girls	1994	Average	14.44
Oceania	Girls	1995	Average	14.63
Oceania	Girls	1996	Average	14.83
Oceania	Girls	1997	Average	15.03
Oceania	Girls	1998	Average	15.23
Oceania	Girls	1999	Average	15.43
Oceania	Girls	2000	Average	15.63
Oceania	Girls	2001	Average	15.83
Oceania	Girls	2002	Average	16.04
Oceania	Girls	2003	Average	16.25
Oceania	Girls	2004	Average	16.46
Oceania	Girls	2005	Average	16.68
Oceania	Girls	2006	Average	16.90
Oceania	Girls	2007	Average	17.12
Oceania	Girls	2008	Average	17.35
Oceania	Girls	2009	Average	17.58
Oceania	Girls	2010	Average	17.81
Oceania	Girls	2011	Average	18.05
Oceania	Girls	2012	Average	18.30
Oceania	Girls	2013	Average	18.55
Oceania	Girls	2014	Average	18.80
Oceania	Girls	2015	Average	19.07
Oceania	Girls	2016	Average	19.34
Oceania	Girls	2017	Average	19.61

Oceania	Girls	2018	Average	19.89
Oceania	Girls	2019	Average	20.17
Oceania	Girls	2020	Average	20.46
Oceania	Girls	2021	Average	20.75
Oceania	Girls	2022	Average	21.05
Oceania	Girls	2023	Average	21.35
Oceania	Girls	2024	Average	21.65
Oceania	Girls	2025	Average	21.95
Oceania	Girls	2026	Average	22.26
Oceania	Girls	2027	Average	22.56
Oceania	Girls	2028	Average	22.88
Oceania	Girls	2029	Average	23.19
Oceania	Girls	2030	Average	23.50
South Asia	Boys	1975	12	3.15
South Asia	Boys	1976	12	3.17
South Asia	Boys	1977	12	3.19
South Asia	Boys	1978	12	3.20
South Asia	Boys	1979	12	3.22
South Asia	Boys	1980	12	3.24
South Asia	Boys	1981	12	3.26
South Asia	Boys	1982	12	3.28
South Asia	Boys	1983	12	3.31
South Asia	Boys	1984	12	3.33
South Asia	Boys	1985	12	3.35
South Asia	Boys	1986	12	3.38
South Asia	Boys	1987	12	3.41
South Asia	Boys	1988	12	3.43
South Asia	Boys	1989	12	3.46
South Asia	Boys	1990	12	3.49
South Asia	Boys	1991	12	3.53
South Asia	Boys	1992	12	3.56
South Asia	Boys	1993	12	3.59
South Asia	Boys	1994	12	3.63
South Asia	Boys	1995	12	3.67
South Asia	Boys	1996	12	3.72
South Asia	Boys	1997	12	3.76
South Asia	Boys	1998	12	3.81
South Asia	Boys	1999	12	3.86
South Asia	Boys	2000	12	3.92
South Asia	Boys	2001	12	3.98
South Asia	Boys	2002	12	4.04
South Asia	Boys	2003	12	4.11
South Asia	Boys	2004	12	4.19
South Asia	Boys	2005	12	4.26
South Asia	Boys	2006	12	4.35
South Asia	Boys	2007	12	4.44
South Asia	Boys	2008	12	4.53
South Asia	Boys	2009	12	4.63
South Asia	Boys	2010	12	4.74
South Asia	Boys	2011	12	4.85
South Asia	Boys	2012	12	4.97
South Asia	Boys	2013	12	5.10

South Asia	Boys	2014	12	5.23
South Asia	Boys	2015	12	5.38
South Asia	Boys	2016	12	5.54
South Asia	Boys	1975	13	3.91
South Asia	Boys	1976	13	3.93
South Asia	Boys	1977	13	3.95
South Asia	Boys	1978	13	3.97
South Asia	Boys	1979	13	4.00
South Asia	Boys	1980	13	4.02
South Asia	Boys	1981	13	4.04
South Asia	Boys	1982	13	4.07
South Asia	Boys	1983	13	4.10
South Asia	Boys	1984	13	4.13
South Asia	Boys	1985	13	4.16
South Asia	Boys	1986	13	4.19
South Asia	Boys	1987	13	4.22
South Asia	Boys	1988	13	4.25
South Asia	Boys	1989	13	4.28
South Asia	Boys	1990	13	4.32
South Asia	Boys	1991	13	4.36
South Asia	Boys	1992	13	4.40
South Asia	Boys	1993	13	4.44
South Asia	Boys	1994	13	4.49
South Asia	Boys	1995	13	4.53
South Asia	Boys	1996	13	4.58
South Asia	Boys	1997	13	4.64
South Asia	Boys	1998	13	4.69
South Asia	Boys	1999	13	4.75
South Asia	Boys	2000	13	4.82
South Asia	Boys	2001	13	4.89
South Asia	Boys	2002	13	4.96
South Asia	Boys	2003	13	5.04
South Asia	Boys	2004	13	5.12
South Asia	Boys	2005	13	5.21
South Asia	Boys	2006	13	5.31
South Asia	Boys	2007	13	5.41
South Asia	Boys	2008	13	5.52
South Asia	Boys	2009	13	5.63
South Asia	Boys	2010	13	5.75
South Asia	Boys	2011	13	5.88
South Asia	Boys	2012	13	6.02
South Asia	Boys	2013	13	6.16
South Asia	Boys	2014	13	6.32
South Asia	Boys	2015	13	6.49
South Asia	Boys	2016	13	6.67
South Asia	Boys	1975	14	4.91
South Asia	Boys	1976	14	4.93
South Asia	Boys	1977	14	4.96
South Asia	Boys	1978	14	4.98
South Asia	Boys	1979	14	5.01
South Asia	Boys	1980	14	5.04
South Asia	Boys	1981	14	5.07

South Asia	Boys	1982	14	5.11
South Asia	Boys	1983	14	5.14
South Asia	Boys	1984	14	5.17
South Asia	Boys	1985	14	5.21
South Asia	Boys	1986	14	5.24
South Asia	Boys	1987	14	5.28
South Asia	Boys	1988	14	5.32
South Asia	Boys	1989	14	5.36
South Asia	Boys	1990	14	5.40
South Asia	Boys	1991	14	5.44
South Asia	Boys	1992	14	5.49
South Asia	Boys	1993	14	5.54
South Asia	Boys	1994	14	5.59
South Asia	Boys	1995	14	5.64
South Asia	Boys	1996	14	5.70
South Asia	Boys	1997	14	5.76
South Asia	Boys	1998	14	5.82
South Asia	Boys	1999	14	5.89
South Asia	Boys	2000	14	5.96
South Asia	Boys	2001	14	6.04
South Asia	Boys	2002	14	6.12
South Asia	Boys	2003	14	6.21
South Asia	Boys	2004	14	6.30
South Asia	Boys	2005	14	6.40
South Asia	Boys	2006	14	6.50
South Asia	Boys	2007	14	6.61
South Asia	Boys	2008	14	6.73
South Asia	Boys	2009	14	6.86
South Asia	Boys	2010	14	6.99
South Asia	Boys	2011	14	7.13
South Asia	Boys	2012	14	7.27
South Asia	Boys	2013	14	7.43
South Asia	Boys	2014	14	7.60
South Asia	Boys	2015	14	7.78
South Asia	Boys	2016	14	7.98
South Asia	Boys	1975	15	6.10
South Asia	Boys	1976	15	6.13
South Asia	Boys	1977	15	6.16
South Asia	Boys	1978	15	6.20
South Asia	Boys	1979	15	6.23
South Asia	Boys	1980	15	6.27
South Asia	Boys	1981	15	6.31
South Asia	Boys	1982	15	6.35
South Asia	Boys	1983	15	6.38
South Asia	Boys	1984	15	6.42
South Asia	Boys	1985	15	6.46
South Asia	Boys	1986	15	6.51
South Asia	Boys	1987	15	6.55
South Asia	Boys	1988	15	6.59
South Asia	Boys	1989	15	6.64
South Asia	Boys	1990	15	6.69
South Asia	Boys	1991	15	6.74

South Asia	Boys	1992	15	6.79
South Asia	Boys	1993	15	6.84
South Asia	Boys	1994	15	6.90
South Asia	Boys	1995	15	6.95
South Asia	Boys	1996	15	7.02
South Asia	Boys	1997	15	7.08
South Asia	Boys	1998	15	7.15
South Asia	Boys	1999	15	7.23
South Asia	Boys	2000	15	7.30
South Asia	Boys	2001	15	7.39
South Asia	Boys	2002	15	7.48
South Asia	Boys	2003	15	7.57
South Asia	Boys	2004	15	7.67
South Asia	Boys	2005	15	7.77
South Asia	Boys	2006	15	7.88
South Asia	Boys	2007	15	8.00
South Asia	Boys	2008	15	8.12
South Asia	Boys	2009	15	8.25
South Asia	Boys	2010	15	8.39
South Asia	Boys	2011	15	8.54
South Asia	Boys	2012	15	8.70
South Asia	Boys	2013	15	8.87
South Asia	Boys	2014	15	9.05
South Asia	Boys	2015	15	9.24
South Asia	Boys	2016	15	9.44
South Asia	Boys	1975	16	7.44
South Asia	Boys	1976	16	7.48
South Asia	Boys	1977	16	7.52
South Asia	Boys	1978	16	7.56
South Asia	Boys	1979	16	7.60
South Asia	Boys	1980	16	7.64
South Asia	Boys	1981	16	7.69
South Asia	Boys	1982	16	7.73
South Asia	Boys	1983	16	7.78
South Asia	Boys	1984	16	7.82
South Asia	Boys	1985	16	7.87
South Asia	Boys	1986	16	7.92
South Asia	Boys	1987	16	7.97
South Asia	Boys	1988	16	8.02
South Asia	Boys	1989	16	8.07
South Asia	Boys	1990	16	8.12
South Asia	Boys	1991	16	8.18
South Asia	Boys	1992	16	8.24
South Asia	Boys	1993	16	8.30
South Asia	Boys	1994	16	8.36
South Asia	Boys	1995	16	8.42
South Asia	Boys	1996	16	8.49
South Asia	Boys	1997	16	8.56
South Asia	Boys	1998	16	8.64
South Asia	Boys	1999	16	8.72
South Asia	Boys	2000	16	8.80
South Asia	Boys	2001	16	8.89

South Asia	Boys	2002	16	8.98
South Asia	Boys	2003	16	9.08
South Asia	Boys	2004	16	9.19
South Asia	Boys	2005	16	9.30
South Asia	Boys	2006	16	9.41
South Asia	Boys	2007	16	9.53
South Asia	Boys	2008	16	9.66
South Asia	Boys	2009	16	9.79
South Asia	Boys	2010	16	9.94
South Asia	Boys	2011	16	10.09
South Asia	Boys	2012	16	10.25
South Asia	Boys	2013	16	10.42
South Asia	Boys	2014	16	10.61
South Asia	Boys	2015	16	10.80
South Asia	Boys	2016	16	11.01
South Asia	Boys	1975	17	8.82
South Asia	Boys	1976	17	8.86
South Asia	Boys	1977	17	8.91
South Asia	Boys	1978	17	8.96
South Asia	Boys	1979	17	9.01
South Asia	Boys	1980	17	9.06
South Asia	Boys	1981	17	9.12
South Asia	Boys	1982	17	9.17
South Asia	Boys	1983	17	9.22
South Asia	Boys	1984	17	9.27
South Asia	Boys	1985	17	9.33
South Asia	Boys	1986	17	9.38
South Asia	Boys	1987	17	9.44
South Asia	Boys	1988	17	9.50
South Asia	Boys	1989	17	9.56
South Asia	Boys	1990	17	9.62
South Asia	Boys	1991	17	9.68
South Asia	Boys	1992	17	9.74
South Asia	Boys	1993	17	9.81
South Asia	Boys	1994	17	9.88
South Asia	Boys	1995	17	9.95
South Asia	Boys	1996	17	10.02
South Asia	Boys	1997	17	10.10
South Asia	Boys	1998	17	10.18
South Asia	Boys	1999	17	10.27
South Asia	Boys	2000	17	10.36
South Asia	Boys	2001	17	10.46
South Asia	Boys	2002	17	10.56
South Asia	Boys	2003	17	10.66
South Asia	Boys	2004	17	10.77
South Asia	Boys	2005	17	10.89
South Asia	Boys	2006	17	11.01
South Asia	Boys	2007	17	11.14
South Asia	Boys	2008	17	11.27
South Asia	Boys	2009	17	11.41
South Asia	Boys	2010	17	11.56
South Asia	Boys	2011	17	11.72

South Asia	Boys	2012	17	11.89
South Asia	Boys	2013	17	12.06
South Asia	Boys	2014	17	12.25
South Asia	Boys	2015	17	12.45
South Asia	Boys	2016	17	12.67
South Asia	Boys	1975	18	10.14
South Asia	Boys	1976	18	10.19
South Asia	Boys	1977	18	10.25
South Asia	Boys	1978	18	10.31
South Asia	Boys	1979	18	10.37
South Asia	Boys	1980	18	10.43
South Asia	Boys	1981	18	10.49
South Asia	Boys	1982	18	10.55
South Asia	Boys	1983	18	10.61
South Asia	Boys	1984	18	10.67
South Asia	Boys	1985	18	10.74
South Asia	Boys	1986	18	10.80
South Asia	Boys	1987	18	10.87
South Asia	Boys	1988	18	10.93
South Asia	Boys	1989	18	11.00
South Asia	Boys	1990	18	11.07
South Asia	Boys	1991	18	11.14
South Asia	Boys	1992	18	11.21
South Asia	Boys	1993	18	11.28
South Asia	Boys	1994	18	11.36
South Asia	Boys	1995	18	11.44
South Asia	Boys	1996	18	11.53
South Asia	Boys	1997	18	11.61
South Asia	Boys	1998	18	11.71
South Asia	Boys	1999	18	11.80
South Asia	Boys	2000	18	11.90
South Asia	Boys	2001	18	12.01
South Asia	Boys	2002	18	12.12
South Asia	Boys	2003	18	12.23
South Asia	Boys	2004	18	12.36
South Asia	Boys	2005	18	12.48
South Asia	Boys	2006	18	12.61
South Asia	Boys	2007	18	12.75
South Asia	Boys	2008	18	12.89
South Asia	Boys	2009	18	13.05
South Asia	Boys	2010	18	13.21
South Asia	Boys	2011	18	13.38
South Asia	Boys	2012	18	13.56
South Asia	Boys	2013	18	13.75
South Asia	Boys	2014	18	13.95
South Asia	Boys	2015	18	14.16
South Asia	Boys	2016	18	14.39
South Asia	Boys	1975	Average	6.35
South Asia	Boys	1976	Average	6.38
South Asia	Boys	1977	Average	6.42
South Asia	Boys	1978	Average	6.46
South Asia	Boys	1979	Average	6.49

South Asia	Boys	1980	Average	6.53
South Asia	Boys	1981	Average	6.57
South Asia	Boys	1982	Average	6.61
South Asia	Boys	1983	Average	6.65
South Asia	Boys	1984	Average	6.69
South Asia	Boys	1985	Average	6.73
South Asia	Boys	1986	Average	6.77
South Asia	Boys	1987	Average	6.82
South Asia	Boys	1988	Average	6.86
South Asia	Boys	1989	Average	6.91
South Asia	Boys	1990	Average	6.96
South Asia	Boys	1991	Average	7.01
South Asia	Boys	1992	Average	7.06
South Asia	Boys	1993	Average	7.11
South Asia	Boys	1994	Average	7.17
South Asia	Boys	1995	Average	7.23
South Asia	Boys	1996	Average	7.29
South Asia	Boys	1997	Average	7.36
South Asia	Boys	1998	Average	7.43
South Asia	Boys	1999	Average	7.50
South Asia	Boys	2000	Average	7.58
South Asia	Boys	2001	Average	7.66
South Asia	Boys	2002	Average	7.75
South Asia	Boys	2003	Average	7.84
South Asia	Boys	2004	Average	7.94
South Asia	Boys	2005	Average	8.05
South Asia	Boys	2006	Average	8.15
South Asia	Boys	2007	Average	8.27
South Asia	Boys	2008	Average	8.39
South Asia	Boys	2009	Average	8.52
South Asia	Boys	2010	Average	8.65
South Asia	Boys	2011	Average	8.80
South Asia	Boys	2012	Average	8.95
South Asia	Boys	2013	Average	9.11
South Asia	Boys	2014	Average	9.29
South Asia	Boys	2015	Average	9.47
South Asia	Boys	2016	Average	9.67
South Asia	Boys	2017	Average	9.87
South Asia	Boys	2018	Average	10.07
South Asia	Boys	2019	Average	10.26
South Asia	Boys	2020	Average	10.46
South Asia	Boys	2021	Average	10.66
South Asia	Boys	2022	Average	10.86
South Asia	Boys	2023	Average	11.05
South Asia	Boys	2024	Average	11.25
South Asia	Boys	2025	Average	11.45
South Asia	Boys	2026	Average	11.65
South Asia	Boys	2027	Average	11.85
South Asia	Boys	2028	Average	12.04
South Asia	Boys	2029	Average	12.24
South Asia	Boys	2030	Average	12.44
South Asia	Girls	1975	12	3.64

South Asia	Girls	1976	12	3.66
South Asia	Girls	1977	12	3.67
South Asia	Girls	1978	12	3.68
South Asia	Girls	1979	12	3.70
South Asia	Girls	1980	12	3.71
South Asia	Girls	1981	12	3.73
South Asia	Girls	1982	12	3.75
South Asia	Girls	1983	12	3.76
South Asia	Girls	1984	12	3.78
South Asia	Girls	1985	12	3.80
South Asia	Girls	1986	12	3.82
South Asia	Girls	1987	12	3.84
South Asia	Girls	1988	12	3.86
South Asia	Girls	1989	12	3.88
South Asia	Girls	1990	12	3.90
South Asia	Girls	1991	12	3.92
South Asia	Girls	1992	12	3.95
South Asia	Girls	1993	12	3.97
South Asia	Girls	1994	12	4.00
South Asia	Girls	1995	12	4.03
South Asia	Girls	1996	12	4.06
South Asia	Girls	1997	12	4.09
South Asia	Girls	1998	12	4.12
South Asia	Girls	1999	12	4.15
South Asia	Girls	2000	12	4.19
South Asia	Girls	2001	12	4.23
South Asia	Girls	2002	12	4.27
South Asia	Girls	2003	12	4.32
South Asia	Girls	2004	12	4.36
South Asia	Girls	2005	12	4.41
South Asia	Girls	2006	12	4.47
South Asia	Girls	2007	12	4.52
South Asia	Girls	2008	12	4.58
South Asia	Girls	2009	12	4.64
South Asia	Girls	2010	12	4.71
South Asia	Girls	2011	12	4.78
South Asia	Girls	2012	12	4.85
South Asia	Girls	2013	12	4.93
South Asia	Girls	2014	12	5.02
South Asia	Girls	2015	12	5.11
South Asia	Girls	2016	12	5.21
South Asia	Girls	1975	13	4.60
South Asia	Girls	1976	13	4.62
South Asia	Girls	1977	13	4.64
South Asia	Girls	1978	13	4.66
South Asia	Girls	1979	13	4.68
South Asia	Girls	1980	13	4.70
South Asia	Girls	1981	13	4.72
South Asia	Girls	1982	13	4.74
South Asia	Girls	1983	13	4.76
South Asia	Girls	1984	13	4.78
South Asia	Girls	1985	13	4.80
South Asia	Girls	1986	13	4.83

South Asia	Girls	1987	13	4.85
South Asia	Girls	1988	13	4.88
South Asia	Girls	1989	13	4.90
South Asia	Girls	1990	13	4.93
South Asia	Girls	1991	13	4.96
South Asia	Girls	1992	13	4.99
South Asia	Girls	1993	13	5.02
South Asia	Girls	1994	13	5.05
South Asia	Girls	1995	13	5.08
South Asia	Girls	1996	13	5.12
South Asia	Girls	1997	13	5.16
South Asia	Girls	1998	13	5.20
South Asia	Girls	1999	13	5.24
South Asia	Girls	2000	13	5.28
South Asia	Girls	2001	13	5.33
South Asia	Girls	2002	13	5.38
South Asia	Girls	2003	13	5.43
South Asia	Girls	2004	13	5.48
South Asia	Girls	2005	13	5.54
South Asia	Girls	2006	13	5.60
South Asia	Girls	2007	13	5.67
South Asia	Girls	2008	13	5.74
South Asia	Girls	2009	13	5.81
South Asia	Girls	2010	13	5.88
South Asia	Girls	2011	13	5.96
South Asia	Girls	2012	13	6.05
South Asia	Girls	2013	13	6.14
South Asia	Girls	2014	13	6.24
South Asia	Girls	2015	13	6.34
South Asia	Girls	2016	13	6.45
South Asia	Girls	1975	14	5.72
South Asia	Girls	1976	14	5.74
South Asia	Girls	1977	14	5.76
South Asia	Girls	1978	14	5.78
South Asia	Girls	1979	14	5.81
South Asia	Girls	1980	14	5.83
South Asia	Girls	1981	14	5.86
South Asia	Girls	1982	14	5.88
South Asia	Girls	1983	14	5.91
South Asia	Girls	1984	14	5.94
South Asia	Girls	1985	14	5.96
South Asia	Girls	1986	14	5.99
South Asia	Girls	1987	14	6.02
South Asia	Girls	1988	14	6.05
South Asia	Girls	1989	14	6.08
South Asia	Girls	1990	14	6.11
South Asia	Girls	1991	14	6.14
South Asia	Girls	1992	14	6.18
South Asia	Girls	1993	14	6.21
South Asia	Girls	1994	14	6.25
South Asia	Girls	1995	14	6.29
South Asia	Girls	1996	14	6.33
South Asia	Girls	1997	14	6.38
South Asia	Girls	1998	14	6.42
South Asia	Girls	1999	14	6.47
South Asia	Girls	2000	14	6.52
South Asia	Girls	2001	14	6.57
South Asia	Girls	2002	14	6.63
South Asia	Girls	2003	14	6.69

South Asia	Girls	2004	14	6.75
South Asia	Girls	2005	14	6.81
South Asia	Girls	2006	14	6.88
South Asia	Girls	2007	14	6.95
South Asia	Girls	2008	14	7.03
South Asia	Girls	2009	14	7.11
South Asia	Girls	2010	14	7.19
South Asia	Girls	2011	14	7.28
South Asia	Girls	2012	14	7.37
South Asia	Girls	2013	14	7.47
South Asia	Girls	2014	14	7.57
South Asia	Girls	2015	14	7.69
South Asia	Girls	2016	14	7.81
South Asia	Girls	1975	15	6.81
South Asia	Girls	1976	15	6.84
South Asia	Girls	1977	15	6.87
South Asia	Girls	1978	15	6.90
South Asia	Girls	1979	15	6.92
South Asia	Girls	1980	15	6.95
South Asia	Girls	1981	15	6.98
South Asia	Girls	1982	15	7.01
South Asia	Girls	1983	15	7.04
South Asia	Girls	1984	15	7.07
South Asia	Girls	1985	15	7.10
South Asia	Girls	1986	15	7.14
South Asia	Girls	1987	15	7.17
South Asia	Girls	1988	15	7.20
South Asia	Girls	1989	15	7.24
South Asia	Girls	1990	15	7.27
South Asia	Girls	1991	15	7.31
South Asia	Girls	1992	15	7.35
South Asia	Girls	1993	15	7.39
South Asia	Girls	1994	15	7.43
South Asia	Girls	1995	15	7.47
South Asia	Girls	1996	15	7.51
South Asia	Girls	1997	15	7.56
South Asia	Girls	1998	15	7.61
South Asia	Girls	1999	15	7.66
South Asia	Girls	2000	15	7.72
South Asia	Girls	2001	15	7.78
South Asia	Girls	2002	15	7.84
South Asia	Girls	2003	15	7.90
South Asia	Girls	2004	15	7.97
South Asia	Girls	2005	15	8.04
South Asia	Girls	2006	15	8.11
South Asia	Girls	2007	15	8.19
South Asia	Girls	2008	15	8.27
South Asia	Girls	2009	15	8.35
South Asia	Girls	2010	15	8.44
South Asia	Girls	2011	15	8.54
South Asia	Girls	2012	15	8.64
South Asia	Girls	2013	15	8.75
South Asia	Girls	2014	15	8.86
South Asia	Girls	2015	15	8.98
South Asia	Girls	2016	15	9.10
South Asia	Girls	1975	16	7.73
South Asia	Girls	1976	16	7.76
South Asia	Girls	1977	16	7.79
South Asia	Girls	1978	16	7.82

South Asia	Girls	1979	16	7.86
South Asia	Girls	1980	16	7.89
South Asia	Girls	1981	16	7.92
South Asia	Girls	1982	16	7.95
South Asia	Girls	1983	16	7.99
South Asia	Girls	1984	16	8.02
South Asia	Girls	1985	16	8.05
South Asia	Girls	1986	16	8.09
South Asia	Girls	1987	16	8.12
South Asia	Girls	1988	16	8.16
South Asia	Girls	1989	16	8.20
South Asia	Girls	1990	16	8.24
South Asia	Girls	1991	16	8.28
South Asia	Girls	1992	16	8.32
South Asia	Girls	1993	16	8.36
South Asia	Girls	1994	16	8.40
South Asia	Girls	1995	16	8.45
South Asia	Girls	1996	16	8.50
South Asia	Girls	1997	16	8.55
South Asia	Girls	1998	16	8.60
South Asia	Girls	1999	16	8.66
South Asia	Girls	2000	16	8.72
South Asia	Girls	2001	16	8.78
South Asia	Girls	2002	16	8.84
South Asia	Girls	2003	16	8.91
South Asia	Girls	2004	16	8.98
South Asia	Girls	2005	16	9.05
South Asia	Girls	2006	16	9.13
South Asia	Girls	2007	16	9.21
South Asia	Girls	2008	16	9.30
South Asia	Girls	2009	16	9.39
South Asia	Girls	2010	16	9.48
South Asia	Girls	2011	16	9.58
South Asia	Girls	2012	16	9.68
South Asia	Girls	2013	16	9.80
South Asia	Girls	2014	16	9.91
South Asia	Girls	2015	16	10.04
South Asia	Girls	2016	16	10.17
South Asia	Girls	1975	17	8.35
South Asia	Girls	1976	17	8.38
South Asia	Girls	1977	17	8.41
South Asia	Girls	1978	17	8.45
South Asia	Girls	1979	17	8.48
South Asia	Girls	1980	17	8.52
South Asia	Girls	1981	17	8.55
South Asia	Girls	1982	17	8.59
South Asia	Girls	1983	17	8.62
South Asia	Girls	1984	17	8.66
South Asia	Girls	1985	17	8.70
South Asia	Girls	1986	17	8.73
South Asia	Girls	1987	17	8.77
South Asia	Girls	1988	17	8.81
South Asia	Girls	1989	17	8.85
South Asia	Girls	1990	17	8.89
South Asia	Girls	1991	17	8.93
South Asia	Girls	1992	17	8.97
South Asia	Girls	1993	17	9.02
South Asia	Girls	1994	17	9.07
South Asia	Girls	1995	17	9.12

South Asia	Girls	1996	17	9.17
South Asia	Girls	1997	17	9.22
South Asia	Girls	1998	17	9.28
South Asia	Girls	1999	17	9.34
South Asia	Girls	2000	17	9.40
South Asia	Girls	2001	17	9.46
South Asia	Girls	2002	17	9.53
South Asia	Girls	2003	17	9.61
South Asia	Girls	2004	17	9.68
South Asia	Girls	2005	17	9.76
South Asia	Girls	2006	17	9.84
South Asia	Girls	2007	17	9.93
South Asia	Girls	2008	17	10.02
South Asia	Girls	2009	17	10.11
South Asia	Girls	2010	17	10.21
South Asia	Girls	2011	17	10.31
South Asia	Girls	2012	17	10.43
South Asia	Girls	2013	17	10.54
South Asia	Girls	2014	17	10.67
South Asia	Girls	2015	17	10.80
South Asia	Girls	2016	17	10.94
South Asia	Girls	1975	18	8.64
South Asia	Girls	1976	18	8.67
South Asia	Girls	1977	18	8.71
South Asia	Girls	1978	18	8.74
South Asia	Girls	1979	18	8.78
South Asia	Girls	1980	18	8.82
South Asia	Girls	1981	18	8.86
South Asia	Girls	1982	18	8.89
South Asia	Girls	1983	18	8.93
South Asia	Girls	1984	18	8.97
South Asia	Girls	1985	18	9.01
South Asia	Girls	1986	18	9.05
South Asia	Girls	1987	18	9.09
South Asia	Girls	1988	18	9.13
South Asia	Girls	1989	18	9.17
South Asia	Girls	1990	18	9.22
South Asia	Girls	1991	18	9.26
South Asia	Girls	1992	18	9.31
South Asia	Girls	1993	18	9.36
South Asia	Girls	1994	18	9.41
South Asia	Girls	1995	18	9.46
South Asia	Girls	1996	18	9.52
South Asia	Girls	1997	18	9.57
South Asia	Girls	1998	18	9.63
South Asia	Girls	1999	18	9.70
South Asia	Girls	2000	18	9.77
South Asia	Girls	2001	18	9.84
South Asia	Girls	2002	18	9.91
South Asia	Girls	2003	18	9.99
South Asia	Girls	2004	18	10.07
South Asia	Girls	2005	18	10.16
South Asia	Girls	2006	18	10.25
South Asia	Girls	2007	18	10.34
South Asia	Girls	2008	18	10.44
South Asia	Girls	2009	18	10.54
South Asia	Girls	2010	18	10.65
South Asia	Girls	2011	18	10.76
South Asia	Girls	2012	18	10.89

South Asia	Girls	2013	18	11.02
South Asia	Girls	2014	18	11.15
South Asia	Girls	2015	18	11.30
South Asia	Girls	2016	18	11.44
South Asia	Girls	1975	Average	6.50
South Asia	Girls	1976	Average	6.52
South Asia	Girls	1977	Average	6.55
South Asia	Girls	1978	Average	6.58
South Asia	Girls	1979	Average	6.60
South Asia	Girls	1980	Average	6.63
South Asia	Girls	1981	Average	6.66
South Asia	Girls	1982	Average	6.69
South Asia	Girls	1983	Average	6.72
South Asia	Girls	1984	Average	6.75
South Asia	Girls	1985	Average	6.78
South Asia	Girls	1986	Average	6.81
South Asia	Girls	1987	Average	6.84
South Asia	Girls	1988	Average	6.87
South Asia	Girls	1989	Average	6.90
South Asia	Girls	1990	Average	6.94
South Asia	Girls	1991	Average	6.97
South Asia	Girls	1992	Average	7.01
South Asia	Girls	1993	Average	7.05
South Asia	Girls	1994	Average	7.09
South Asia	Girls	1995	Average	7.13
South Asia	Girls	1996	Average	7.17
South Asia	Girls	1997	Average	7.22
South Asia	Girls	1998	Average	7.27
South Asia	Girls	1999	Average	7.32
South Asia	Girls	2000	Average	7.37
South Asia	Girls	2001	Average	7.43
South Asia	Girls	2002	Average	7.49
South Asia	Girls	2003	Average	7.55
South Asia	Girls	2004	Average	7.61
South Asia	Girls	2005	Average	7.68
South Asia	Girls	2006	Average	7.75
South Asia	Girls	2007	Average	7.83
South Asia	Girls	2008	Average	7.91
South Asia	Girls	2009	Average	7.99
South Asia	Girls	2010	Average	8.08
South Asia	Girls	2011	Average	8.17
South Asia	Girls	2012	Average	8.27
South Asia	Girls	2013	Average	8.38
South Asia	Girls	2014	Average	8.49
South Asia	Girls	2015	Average	8.61
South Asia	Girls	2016	Average	8.73
South Asia	Girls	2017	Average	8.86
South Asia	Girls	2018	Average	8.99
South Asia	Girls	2019	Average	9.13
South Asia	Girls	2020	Average	9.26
South Asia	Girls	2021	Average	9.41
South Asia	Girls	2022	Average	9.55
South Asia	Girls	2023	Average	9.70
South Asia	Girls	2024	Average	9.85
South Asia	Girls	2025	Average	10.00
South Asia	Girls	2026	Average	10.16
South Asia	Girls	2027	Average	10.32
South Asia	Girls	2028	Average	10.49
South Asia	Girls	2029	Average	10.65

South Asia	Girls	2030	Average	10.82
Sub-Saharan Africa	Boys	1975	12	4.01
Sub-Saharan Africa	Boys	1976	12	4.03
Sub-Saharan Africa	Boys	1977	12	4.05
Sub-Saharan Africa	Boys	1978	12	4.06
Sub-Saharan Africa	Boys	1979	12	4.08
Sub-Saharan Africa	Boys	1980	12	4.10
Sub-Saharan Africa	Boys	1981	12	4.12
Sub-Saharan Africa	Boys	1982	12	4.14
Sub-Saharan Africa	Boys	1983	12	4.16
Sub-Saharan Africa	Boys	1984	12	4.18
Sub-Saharan Africa	Boys	1985	12	4.21
Sub-Saharan Africa	Boys	1986	12	4.23
Sub-Saharan Africa	Boys	1987	12	4.26
Sub-Saharan Africa	Boys	1988	12	4.29
Sub-Saharan Africa	Boys	1989	12	4.32
Sub-Saharan Africa	Boys	1990	12	4.35
Sub-Saharan Africa	Boys	1991	12	4.38
Sub-Saharan Africa	Boys	1992	12	4.41
Sub-Saharan Africa	Boys	1993	12	4.45
Sub-Saharan Africa	Boys	1994	12	4.49
Sub-Saharan Africa	Boys	1995	12	4.53
Sub-Saharan Africa	Boys	1996	12	4.57
Sub-Saharan Africa	Boys	1997	12	4.62
Sub-Saharan Africa	Boys	1998	12	4.67
Sub-Saharan Africa	Boys	1999	12	4.72
Sub-Saharan Africa	Boys	2000	12	4.77
Sub-Saharan Africa	Boys	2001	12	4.83
Sub-Saharan Africa	Boys	2002	12	4.89
Sub-Saharan Africa	Boys	2003	12	4.95
Sub-Saharan Africa	Boys	2004	12	5.02
Sub-Saharan Africa	Boys	2005	12	5.09
Sub-Saharan Africa	Boys	2006	12	5.17
Sub-Saharan Africa	Boys	2007	12	5.25
Sub-Saharan Africa	Boys	2008	12	5.33
Sub-Saharan Africa	Boys	2009	12	5.42
Sub-Saharan Africa	Boys	2010	12	5.51
Sub-Saharan Africa	Boys	2011	12	5.60
Sub-Saharan Africa	Boys	2012	12	5.70
Sub-Saharan Africa	Boys	2013	12	5.81
Sub-Saharan Africa	Boys	2014	12	5.92
Sub-Saharan Africa	Boys	2015	12	6.04
Sub-Saharan Africa	Boys	2016	12	6.16
Sub-Saharan Africa	Boys	1975	13	5.02
Sub-Saharan Africa	Boys	1976	13	5.04
Sub-Saharan Africa	Boys	1977	13	5.06
Sub-Saharan Africa	Boys	1978	13	5.08
Sub-Saharan Africa	Boys	1979	13	5.11
Sub-Saharan Africa	Boys	1980	13	5.13
Sub-Saharan Africa	Boys	1981	13	5.15
Sub-Saharan Africa	Boys	1982	13	5.18
Sub-Saharan Africa	Boys	1983	13	5.20
Sub-Saharan Africa	Boys	1984	13	5.23
Sub-Saharan Africa	Boys	1985	13	5.26
Sub-Saharan Africa	Boys	1986	13	5.29
Sub-Saharan Africa	Boys	1987	13	5.32
Sub-Saharan Africa	Boys	1988	13	5.35
Sub-Saharan Africa	Boys	1989	13	5.39
Sub-Saharan Africa	Boys	1990	13	5.43

Sub-Saharan Africa	Boys	1991	13	5.47
Sub-Saharan Africa	Boys	1992	13	5.51
Sub-Saharan Africa	Boys	1993	13	5.55
Sub-Saharan Africa	Boys	1994	13	5.59
Sub-Saharan Africa	Boys	1995	13	5.64
Sub-Saharan Africa	Boys	1996	13	5.69
Sub-Saharan Africa	Boys	1997	13	5.75
Sub-Saharan Africa	Boys	1998	13	5.81
Sub-Saharan Africa	Boys	1999	13	5.87
Sub-Saharan Africa	Boys	2000	13	5.93
Sub-Saharan Africa	Boys	2001	13	6.00
Sub-Saharan Africa	Boys	2002	13	6.07
Sub-Saharan Africa	Boys	2003	13	6.15
Sub-Saharan Africa	Boys	2004	13	6.23
Sub-Saharan Africa	Boys	2005	13	6.31
Sub-Saharan Africa	Boys	2006	13	6.40
Sub-Saharan Africa	Boys	2007	13	6.49
Sub-Saharan Africa	Boys	2008	13	6.59
Sub-Saharan Africa	Boys	2009	13	6.69
Sub-Saharan Africa	Boys	2010	13	6.79
Sub-Saharan Africa	Boys	2011	13	6.91
Sub-Saharan Africa	Boys	2012	13	7.02
Sub-Saharan Africa	Boys	2013	13	7.15
Sub-Saharan Africa	Boys	2014	13	7.28
Sub-Saharan Africa	Boys	2015	13	7.41
Sub-Saharan Africa	Boys	2016	13	7.56
Sub-Saharan Africa	Boys	1975	14	6.34
Sub-Saharan Africa	Boys	1976	14	6.37
Sub-Saharan Africa	Boys	1977	14	6.39
Sub-Saharan Africa	Boys	1978	14	6.42
Sub-Saharan Africa	Boys	1979	14	6.45
Sub-Saharan Africa	Boys	1980	14	6.48
Sub-Saharan Africa	Boys	1981	14	6.51
Sub-Saharan Africa	Boys	1982	14	6.54
Sub-Saharan Africa	Boys	1983	14	6.57
Sub-Saharan Africa	Boys	1984	14	6.60
Sub-Saharan Africa	Boys	1985	14	6.63
Sub-Saharan Africa	Boys	1986	14	6.67
Sub-Saharan Africa	Boys	1987	14	6.71
Sub-Saharan Africa	Boys	1988	14	6.75
Sub-Saharan Africa	Boys	1989	14	6.79
Sub-Saharan Africa	Boys	1990	14	6.83
Sub-Saharan Africa	Boys	1991	14	6.88
Sub-Saharan Africa	Boys	1992	14	6.92
Sub-Saharan Africa	Boys	1993	14	6.97
Sub-Saharan Africa	Boys	1994	14	7.02
Sub-Saharan Africa	Boys	1995	14	7.08
Sub-Saharan Africa	Boys	1996	14	7.14
Sub-Saharan Africa	Boys	1997	14	7.20
Sub-Saharan Africa	Boys	1998	14	7.27
Sub-Saharan Africa	Boys	1999	14	7.34
Sub-Saharan Africa	Boys	2000	14	7.41
Sub-Saharan Africa	Boys	2001	14	7.49
Sub-Saharan Africa	Boys	2002	14	7.57
Sub-Saharan Africa	Boys	2003	14	7.66
Sub-Saharan Africa	Boys	2004	14	7.75
Sub-Saharan Africa	Boys	2005	14	7.84
Sub-Saharan Africa	Boys	2006	14	7.94
Sub-Saharan Africa	Boys	2007	14	8.04

Sub-Saharan Africa	Boys	2008	14	8.15
Sub-Saharan Africa	Boys	2009	14	8.27
Sub-Saharan Africa	Boys	2010	14	8.38
Sub-Saharan Africa	Boys	2011	14	8.51
Sub-Saharan Africa	Boys	2012	14	8.64
Sub-Saharan Africa	Boys	2013	14	8.78
Sub-Saharan Africa	Boys	2014	14	8.92
Sub-Saharan Africa	Boys	2015	14	9.07
Sub-Saharan Africa	Boys	2016	14	9.23
Sub-Saharan Africa	Boys	1975	15	7.93
Sub-Saharan Africa	Boys	1976	15	7.96
Sub-Saharan Africa	Boys	1977	15	7.99
Sub-Saharan Africa	Boys	1978	15	8.02
Sub-Saharan Africa	Boys	1979	15	8.06
Sub-Saharan Africa	Boys	1980	15	8.09
Sub-Saharan Africa	Boys	1981	15	8.13
Sub-Saharan Africa	Boys	1982	15	8.16
Sub-Saharan Africa	Boys	1983	15	8.20
Sub-Saharan Africa	Boys	1984	15	8.24
Sub-Saharan Africa	Boys	1985	15	8.28
Sub-Saharan Africa	Boys	1986	15	8.32
Sub-Saharan Africa	Boys	1987	15	8.36
Sub-Saharan Africa	Boys	1988	15	8.40
Sub-Saharan Africa	Boys	1989	15	8.45
Sub-Saharan Africa	Boys	1990	15	8.50
Sub-Saharan Africa	Boys	1991	15	8.55
Sub-Saharan Africa	Boys	1992	15	8.61
Sub-Saharan Africa	Boys	1993	15	8.66
Sub-Saharan Africa	Boys	1994	15	8.72
Sub-Saharan Africa	Boys	1995	15	8.79
Sub-Saharan Africa	Boys	1996	15	8.85
Sub-Saharan Africa	Boys	1997	15	8.92
Sub-Saharan Africa	Boys	1998	15	8.99
Sub-Saharan Africa	Boys	1999	15	9.07
Sub-Saharan Africa	Boys	2000	15	9.15
Sub-Saharan Africa	Boys	2001	15	9.23
Sub-Saharan Africa	Boys	2002	15	9.33
Sub-Saharan Africa	Boys	2003	15	9.42
Sub-Saharan Africa	Boys	2004	15	9.52
Sub-Saharan Africa	Boys	2005	15	9.63
Sub-Saharan Africa	Boys	2006	15	9.74
Sub-Saharan Africa	Boys	2007	15	9.86
Sub-Saharan Africa	Boys	2008	15	9.98
Sub-Saharan Africa	Boys	2009	15	10.11
Sub-Saharan Africa	Boys	2010	15	10.24
Sub-Saharan Africa	Boys	2011	15	10.38
Sub-Saharan Africa	Boys	2012	15	10.52
Sub-Saharan Africa	Boys	2013	15	10.66
Sub-Saharan Africa	Boys	2014	15	10.81
Sub-Saharan Africa	Boys	2015	15	10.97
Sub-Saharan Africa	Boys	2016	15	11.14
Sub-Saharan Africa	Boys	1975	16	9.69
Sub-Saharan Africa	Boys	1976	16	9.73
Sub-Saharan Africa	Boys	1977	16	9.77
Sub-Saharan Africa	Boys	1978	16	9.81
Sub-Saharan Africa	Boys	1979	16	9.85
Sub-Saharan Africa	Boys	1980	16	9.89
Sub-Saharan Africa	Boys	1981	16	9.93
Sub-Saharan Africa	Boys	1982	16	9.97

Sub-Saharan Africa	Boys	1983	16	10.02
Sub-Saharan Africa	Boys	1984	16	10.06
Sub-Saharan Africa	Boys	1985	16	10.10
Sub-Saharan Africa	Boys	1986	16	10.15
Sub-Saharan Africa	Boys	1987	16	10.20
Sub-Saharan Africa	Boys	1988	16	10.25
Sub-Saharan Africa	Boys	1989	16	10.30
Sub-Saharan Africa	Boys	1990	16	10.36
Sub-Saharan Africa	Boys	1991	16	10.42
Sub-Saharan Africa	Boys	1992	16	10.48
Sub-Saharan Africa	Boys	1993	16	10.54
Sub-Saharan Africa	Boys	1994	16	10.60
Sub-Saharan Africa	Boys	1995	16	10.67
Sub-Saharan Africa	Boys	1996	16	10.74
Sub-Saharan Africa	Boys	1997	16	10.82
Sub-Saharan Africa	Boys	1998	16	10.90
Sub-Saharan Africa	Boys	1999	16	10.98
Sub-Saharan Africa	Boys	2000	16	11.07
Sub-Saharan Africa	Boys	2001	16	11.16
Sub-Saharan Africa	Boys	2002	16	11.26
Sub-Saharan Africa	Boys	2003	16	11.37
Sub-Saharan Africa	Boys	2004	16	11.48
Sub-Saharan Africa	Boys	2005	16	11.59
Sub-Saharan Africa	Boys	2006	16	11.71
Sub-Saharan Africa	Boys	2007	16	11.84
Sub-Saharan Africa	Boys	2008	16	11.97
Sub-Saharan Africa	Boys	2009	16	12.10
Sub-Saharan Africa	Boys	2010	16	12.24
Sub-Saharan Africa	Boys	2011	16	12.38
Sub-Saharan Africa	Boys	2012	16	12.53
Sub-Saharan Africa	Boys	2013	16	12.68
Sub-Saharan Africa	Boys	2014	16	12.84
Sub-Saharan Africa	Boys	2015	16	13.00
Sub-Saharan Africa	Boys	2016	16	13.18
Sub-Saharan Africa	Boys	1975	17	11.53
Sub-Saharan Africa	Boys	1976	17	11.58
Sub-Saharan Africa	Boys	1977	17	11.62
Sub-Saharan Africa	Boys	1978	17	11.67
Sub-Saharan Africa	Boys	1979	17	11.72
Sub-Saharan Africa	Boys	1980	17	11.76
Sub-Saharan Africa	Boys	1981	17	11.81
Sub-Saharan Africa	Boys	1982	17	11.86
Sub-Saharan Africa	Boys	1983	17	11.91
Sub-Saharan Africa	Boys	1984	17	11.96
Sub-Saharan Africa	Boys	1985	17	12.01
Sub-Saharan Africa	Boys	1986	17	12.06
Sub-Saharan Africa	Boys	1987	17	12.12
Sub-Saharan Africa	Boys	1988	17	12.17
Sub-Saharan Africa	Boys	1989	17	12.23
Sub-Saharan Africa	Boys	1990	17	12.29
Sub-Saharan Africa	Boys	1991	17	12.36
Sub-Saharan Africa	Boys	1992	17	12.43
Sub-Saharan Africa	Boys	1993	17	12.49
Sub-Saharan Africa	Boys	1994	17	12.57
Sub-Saharan Africa	Boys	1995	17	12.64
Sub-Saharan Africa	Boys	1996	17	12.72
Sub-Saharan Africa	Boys	1997	17	12.80
Sub-Saharan Africa	Boys	1998	17	12.89
Sub-Saharan Africa	Boys	1999	17	12.98

Sub-Saharan Africa	Boys	2000	17	13.07
Sub-Saharan Africa	Boys	2001	17	13.18
Sub-Saharan Africa	Boys	2002	17	13.28
Sub-Saharan Africa	Boys	2003	17	13.40
Sub-Saharan Africa	Boys	2004	17	13.51
Sub-Saharan Africa	Boys	2005	17	13.64
Sub-Saharan Africa	Boys	2006	17	13.77
Sub-Saharan Africa	Boys	2007	17	13.90
Sub-Saharan Africa	Boys	2008	17	14.04
Sub-Saharan Africa	Boys	2009	17	14.18
Sub-Saharan Africa	Boys	2010	17	14.33
Sub-Saharan Africa	Boys	2011	17	14.48
Sub-Saharan Africa	Boys	2012	17	14.63
Sub-Saharan Africa	Boys	2013	17	14.79
Sub-Saharan Africa	Boys	2014	17	14.95
Sub-Saharan Africa	Boys	2015	17	15.12
Sub-Saharan Africa	Boys	2016	17	15.30
Sub-Saharan Africa	Boys	1975	18	13.33
Sub-Saharan Africa	Boys	1976	18	13.38
Sub-Saharan Africa	Boys	1977	18	13.44
Sub-Saharan Africa	Boys	1978	18	13.49
Sub-Saharan Africa	Boys	1979	18	13.55
Sub-Saharan Africa	Boys	1980	18	13.60
Sub-Saharan Africa	Boys	1981	18	13.66
Sub-Saharan Africa	Boys	1982	18	13.71
Sub-Saharan Africa	Boys	1983	18	13.77
Sub-Saharan Africa	Boys	1984	18	13.83
Sub-Saharan Africa	Boys	1985	18	13.88
Sub-Saharan Africa	Boys	1986	18	13.94
Sub-Saharan Africa	Boys	1987	18	14.01
Sub-Saharan Africa	Boys	1988	18	14.07
Sub-Saharan Africa	Boys	1989	18	14.14
Sub-Saharan Africa	Boys	1990	18	14.21
Sub-Saharan Africa	Boys	1991	18	14.28
Sub-Saharan Africa	Boys	1992	18	14.35
Sub-Saharan Africa	Boys	1993	18	14.43
Sub-Saharan Africa	Boys	1994	18	14.51
Sub-Saharan Africa	Boys	1995	18	14.59
Sub-Saharan Africa	Boys	1996	18	14.68
Sub-Saharan Africa	Boys	1997	18	14.77
Sub-Saharan Africa	Boys	1998	18	14.86
Sub-Saharan Africa	Boys	1999	18	14.96
Sub-Saharan Africa	Boys	2000	18	15.07
Sub-Saharan Africa	Boys	2001	18	15.18
Sub-Saharan Africa	Boys	2002	18	15.30
Sub-Saharan Africa	Boys	2003	18	15.42
Sub-Saharan Africa	Boys	2004	18	15.55
Sub-Saharan Africa	Boys	2005	18	15.69
Sub-Saharan Africa	Boys	2006	18	15.83
Sub-Saharan Africa	Boys	2007	18	15.97
Sub-Saharan Africa	Boys	2008	18	16.12
Sub-Saharan Africa	Boys	2009	18	16.27
Sub-Saharan Africa	Boys	2010	18	16.43
Sub-Saharan Africa	Boys	2011	18	16.59
Sub-Saharan Africa	Boys	2012	18	16.75
Sub-Saharan Africa	Boys	2013	18	16.92
Sub-Saharan Africa	Boys	2014	18	17.10
Sub-Saharan Africa	Boys	2015	18	17.28
Sub-Saharan Africa	Boys	2016	18	17.47

Sub-Saharan Africa	Boys	1975	Average	8.26
Sub-Saharan Africa	Boys	1976	Average	8.30
Sub-Saharan Africa	Boys	1977	Average	8.33
Sub-Saharan Africa	Boys	1978	Average	8.37
Sub-Saharan Africa	Boys	1979	Average	8.40
Sub-Saharan Africa	Boys	1980	Average	8.44
Sub-Saharan Africa	Boys	1981	Average	8.47
Sub-Saharan Africa	Boys	1982	Average	8.51
Sub-Saharan Africa	Boys	1983	Average	8.55
Sub-Saharan Africa	Boys	1984	Average	8.58
Sub-Saharan Africa	Boys	1985	Average	8.62
Sub-Saharan Africa	Boys	1986	Average	8.67
Sub-Saharan Africa	Boys	1987	Average	8.71
Sub-Saharan Africa	Boys	1988	Average	8.75
Sub-Saharan Africa	Boys	1989	Average	8.80
Sub-Saharan Africa	Boys	1990	Average	8.85
Sub-Saharan Africa	Boys	1991	Average	8.90
Sub-Saharan Africa	Boys	1992	Average	8.96
Sub-Saharan Africa	Boys	1993	Average	9.01
Sub-Saharan Africa	Boys	1994	Average	9.07
Sub-Saharan Africa	Boys	1995	Average	9.13
Sub-Saharan Africa	Boys	1996	Average	9.20
Sub-Saharan Africa	Boys	1997	Average	9.27
Sub-Saharan Africa	Boys	1998	Average	9.34
Sub-Saharan Africa	Boys	1999	Average	9.42
Sub-Saharan Africa	Boys	2000	Average	9.50
Sub-Saharan Africa	Boys	2001	Average	9.58
Sub-Saharan Africa	Boys	2002	Average	9.67
Sub-Saharan Africa	Boys	2003	Average	9.77
Sub-Saharan Africa	Boys	2004	Average	9.87
Sub-Saharan Africa	Boys	2005	Average	9.97
Sub-Saharan Africa	Boys	2006	Average	10.08
Sub-Saharan Africa	Boys	2007	Average	10.19
Sub-Saharan Africa	Boys	2008	Average	10.31
Sub-Saharan Africa	Boys	2009	Average	10.43
Sub-Saharan Africa	Boys	2010	Average	10.56
Sub-Saharan Africa	Boys	2011	Average	10.69
Sub-Saharan Africa	Boys	2012	Average	10.83
Sub-Saharan Africa	Boys	2013	Average	10.97
Sub-Saharan Africa	Boys	2014	Average	11.12
Sub-Saharan Africa	Boys	2015	Average	11.27
Sub-Saharan Africa	Boys	2016	Average	11.43
Sub-Saharan Africa	Boys	2017	Average	11.60
Sub-Saharan Africa	Boys	2018	Average	11.76
Sub-Saharan Africa	Boys	2019	Average	11.92
Sub-Saharan Africa	Boys	2020	Average	12.08
Sub-Saharan Africa	Boys	2021	Average	12.25
Sub-Saharan Africa	Boys	2022	Average	12.41
Sub-Saharan Africa	Boys	2023	Average	12.57
Sub-Saharan Africa	Boys	2024	Average	12.73
Sub-Saharan Africa	Boys	2025	Average	12.90
Sub-Saharan Africa	Boys	2026	Average	13.06
Sub-Saharan Africa	Boys	2027	Average	13.22
Sub-Saharan Africa	Boys	2028	Average	13.38
Sub-Saharan Africa	Boys	2029	Average	13.55
Sub-Saharan Africa	Boys	2030	Average	13.71
Sub-Saharan Africa	Girls	1975	12	4.73
Sub-Saharan Africa	Girls	1976	12	4.76
Sub-Saharan Africa	Girls	1977	12	4.79

Sub-Saharan Africa	Girls	1978	12	4.82
Sub-Saharan Africa	Girls	1979	12	4.85
Sub-Saharan Africa	Girls	1980	12	4.89
Sub-Saharan Africa	Girls	1981	12	4.92
Sub-Saharan Africa	Girls	1982	12	4.96
Sub-Saharan Africa	Girls	1983	12	4.99
Sub-Saharan Africa	Girls	1984	12	5.03
Sub-Saharan Africa	Girls	1985	12	5.07
Sub-Saharan Africa	Girls	1986	12	5.12
Sub-Saharan Africa	Girls	1987	12	5.17
Sub-Saharan Africa	Girls	1988	12	5.21
Sub-Saharan Africa	Girls	1989	12	5.27
Sub-Saharan Africa	Girls	1990	12	5.32
Sub-Saharan Africa	Girls	1991	12	5.38
Sub-Saharan Africa	Girls	1992	12	5.43
Sub-Saharan Africa	Girls	1993	12	5.49
Sub-Saharan Africa	Girls	1994	12	5.56
Sub-Saharan Africa	Girls	1995	12	5.62
Sub-Saharan Africa	Girls	1996	12	5.69
Sub-Saharan Africa	Girls	1997	12	5.77
Sub-Saharan Africa	Girls	1998	12	5.84
Sub-Saharan Africa	Girls	1999	12	5.92
Sub-Saharan Africa	Girls	2000	12	6.00
Sub-Saharan Africa	Girls	2001	12	6.09
Sub-Saharan Africa	Girls	2002	12	6.18
Sub-Saharan Africa	Girls	2003	12	6.27
Sub-Saharan Africa	Girls	2004	12	6.36
Sub-Saharan Africa	Girls	2005	12	6.46
Sub-Saharan Africa	Girls	2006	12	6.56
Sub-Saharan Africa	Girls	2007	12	6.66
Sub-Saharan Africa	Girls	2008	12	6.77
Sub-Saharan Africa	Girls	2009	12	6.88
Sub-Saharan Africa	Girls	2010	12	6.98
Sub-Saharan Africa	Girls	2011	12	7.10
Sub-Saharan Africa	Girls	2012	12	7.21
Sub-Saharan Africa	Girls	2013	12	7.33
Sub-Saharan Africa	Girls	2014	12	7.45
Sub-Saharan Africa	Girls	2015	12	7.57
Sub-Saharan Africa	Girls	2016	12	7.70
Sub-Saharan Africa	Girls	1975	13	5.97
Sub-Saharan Africa	Girls	1976	13	6.01
Sub-Saharan Africa	Girls	1977	13	6.04
Sub-Saharan Africa	Girls	1978	13	6.08
Sub-Saharan Africa	Girls	1979	13	6.12
Sub-Saharan Africa	Girls	1980	13	6.16
Sub-Saharan Africa	Girls	1981	13	6.20
Sub-Saharan Africa	Girls	1982	13	6.24
Sub-Saharan Africa	Girls	1983	13	6.29
Sub-Saharan Africa	Girls	1984	13	6.34
Sub-Saharan Africa	Girls	1985	13	6.39
Sub-Saharan Africa	Girls	1986	13	6.44
Sub-Saharan Africa	Girls	1987	13	6.49
Sub-Saharan Africa	Girls	1988	13	6.55
Sub-Saharan Africa	Girls	1989	13	6.61
Sub-Saharan Africa	Girls	1990	13	6.68
Sub-Saharan Africa	Girls	1991	13	6.74
Sub-Saharan Africa	Girls	1992	13	6.81
Sub-Saharan Africa	Girls	1993	13	6.88
Sub-Saharan Africa	Girls	1994	13	6.96

Sub-Saharan Africa	Girls	1995	13	7.04
Sub-Saharan Africa	Girls	1996	13	7.12
Sub-Saharan Africa	Girls	1997	13	7.20
Sub-Saharan Africa	Girls	1998	13	7.29
Sub-Saharan Africa	Girls	1999	13	7.39
Sub-Saharan Africa	Girls	2000	13	7.48
Sub-Saharan Africa	Girls	2001	13	7.58
Sub-Saharan Africa	Girls	2002	13	7.68
Sub-Saharan Africa	Girls	2003	13	7.79
Sub-Saharan Africa	Girls	2004	13	7.90
Sub-Saharan Africa	Girls	2005	13	8.01
Sub-Saharan Africa	Girls	2006	13	8.13
Sub-Saharan Africa	Girls	2007	13	8.25
Sub-Saharan Africa	Girls	2008	13	8.37
Sub-Saharan Africa	Girls	2009	13	8.49
Sub-Saharan Africa	Girls	2010	13	8.62
Sub-Saharan Africa	Girls	2011	13	8.75
Sub-Saharan Africa	Girls	2012	13	8.88
Sub-Saharan Africa	Girls	2013	13	9.01
Sub-Saharan Africa	Girls	2014	13	9.15
Sub-Saharan Africa	Girls	2015	13	9.30
Sub-Saharan Africa	Girls	2016	13	9.45
Sub-Saharan Africa	Girls	1975	14	7.36
Sub-Saharan Africa	Girls	1976	14	7.40
Sub-Saharan Africa	Girls	1977	14	7.44
Sub-Saharan Africa	Girls	1978	14	7.48
Sub-Saharan Africa	Girls	1979	14	7.53
Sub-Saharan Africa	Girls	1980	14	7.58
Sub-Saharan Africa	Girls	1981	14	7.62
Sub-Saharan Africa	Girls	1982	14	7.67
Sub-Saharan Africa	Girls	1983	14	7.73
Sub-Saharan Africa	Girls	1984	14	7.78
Sub-Saharan Africa	Girls	1985	14	7.84
Sub-Saharan Africa	Girls	1986	14	7.90
Sub-Saharan Africa	Girls	1987	14	7.96
Sub-Saharan Africa	Girls	1988	14	8.02
Sub-Saharan Africa	Girls	1989	14	8.09
Sub-Saharan Africa	Girls	1990	14	8.17
Sub-Saharan Africa	Girls	1991	14	8.24
Sub-Saharan Africa	Girls	1992	14	8.32
Sub-Saharan Africa	Girls	1993	14	8.40
Sub-Saharan Africa	Girls	1994	14	8.48
Sub-Saharan Africa	Girls	1995	14	8.57
Sub-Saharan Africa	Girls	1996	14	8.66
Sub-Saharan Africa	Girls	1997	14	8.76
Sub-Saharan Africa	Girls	1998	14	8.85
Sub-Saharan Africa	Girls	1999	14	8.96
Sub-Saharan Africa	Girls	2000	14	9.06
Sub-Saharan Africa	Girls	2001	14	9.17
Sub-Saharan Africa	Girls	2002	14	9.29
Sub-Saharan Africa	Girls	2003	14	9.41
Sub-Saharan Africa	Girls	2004	14	9.53
Sub-Saharan Africa	Girls	2005	14	9.65
Sub-Saharan Africa	Girls	2006	14	9.78
Sub-Saharan Africa	Girls	2007	14	9.91
Sub-Saharan Africa	Girls	2008	14	10.04
Sub-Saharan Africa	Girls	2009	14	10.17
Sub-Saharan Africa	Girls	2010	14	10.31
Sub-Saharan Africa	Girls	2011	14	10.45

Sub-Saharan Africa	Girls	2012	14	10.60
Sub-Saharan Africa	Girls	2013	14	10.75
Sub-Saharan Africa	Girls	2014	14	10.90
Sub-Saharan Africa	Girls	2015	14	11.06
Sub-Saharan Africa	Girls	2016	14	11.22
Sub-Saharan Africa	Girls	1975	15	8.69
Sub-Saharan Africa	Girls	1976	15	8.74
Sub-Saharan Africa	Girls	1977	15	8.78
Sub-Saharan Africa	Girls	1978	15	8.83
Sub-Saharan Africa	Girls	1979	15	8.88
Sub-Saharan Africa	Girls	1980	15	8.93
Sub-Saharan Africa	Girls	1981	15	8.99
Sub-Saharan Africa	Girls	1982	15	9.04
Sub-Saharan Africa	Girls	1983	15	9.10
Sub-Saharan Africa	Girls	1984	15	9.16
Sub-Saharan Africa	Girls	1985	15	9.22
Sub-Saharan Africa	Girls	1986	15	9.29
Sub-Saharan Africa	Girls	1987	15	9.35
Sub-Saharan Africa	Girls	1988	15	9.42
Sub-Saharan Africa	Girls	1989	15	9.50
Sub-Saharan Africa	Girls	1990	15	9.58
Sub-Saharan Africa	Girls	1991	15	9.66
Sub-Saharan Africa	Girls	1992	15	9.74
Sub-Saharan Africa	Girls	1993	15	9.83
Sub-Saharan Africa	Girls	1994	15	9.92
Sub-Saharan Africa	Girls	1995	15	10.01
Sub-Saharan Africa	Girls	1996	15	10.11
Sub-Saharan Africa	Girls	1997	15	10.21
Sub-Saharan Africa	Girls	1998	15	10.31
Sub-Saharan Africa	Girls	1999	15	10.42
Sub-Saharan Africa	Girls	2000	15	10.54
Sub-Saharan Africa	Girls	2001	15	10.66
Sub-Saharan Africa	Girls	2002	15	10.78
Sub-Saharan Africa	Girls	2003	15	10.91
Sub-Saharan Africa	Girls	2004	15	11.04
Sub-Saharan Africa	Girls	2005	15	11.18
Sub-Saharan Africa	Girls	2006	15	11.32
Sub-Saharan Africa	Girls	2007	15	11.46
Sub-Saharan Africa	Girls	2008	15	11.60
Sub-Saharan Africa	Girls	2009	15	11.75
Sub-Saharan Africa	Girls	2010	15	11.90
Sub-Saharan Africa	Girls	2011	15	12.05
Sub-Saharan Africa	Girls	2012	15	12.20
Sub-Saharan Africa	Girls	2013	15	12.35
Sub-Saharan Africa	Girls	2014	15	12.51
Sub-Saharan Africa	Girls	2015	15	12.67
Sub-Saharan Africa	Girls	2016	15	12.83
Sub-Saharan Africa	Girls	1975	16	9.79
Sub-Saharan Africa	Girls	1976	16	9.84
Sub-Saharan Africa	Girls	1977	16	9.89
Sub-Saharan Africa	Girls	1978	16	9.94
Sub-Saharan Africa	Girls	1979	16	10.00
Sub-Saharan Africa	Girls	1980	16	10.05
Sub-Saharan Africa	Girls	1981	16	10.11
Sub-Saharan Africa	Girls	1982	16	10.17
Sub-Saharan Africa	Girls	1983	16	10.23
Sub-Saharan Africa	Girls	1984	16	10.29
Sub-Saharan Africa	Girls	1985	16	10.36
Sub-Saharan Africa	Girls	1986	16	10.42

Sub-Saharan Africa	Girls	1987	16	10.50
Sub-Saharan Africa	Girls	1988	16	10.57
Sub-Saharan Africa	Girls	1989	16	10.65
Sub-Saharan Africa	Girls	1990	16	10.73
Sub-Saharan Africa	Girls	1991	16	10.81
Sub-Saharan Africa	Girls	1992	16	10.90
Sub-Saharan Africa	Girls	1993	16	10.99
Sub-Saharan Africa	Girls	1994	16	11.09
Sub-Saharan Africa	Girls	1995	16	11.19
Sub-Saharan Africa	Girls	1996	16	11.29
Sub-Saharan Africa	Girls	1997	16	11.39
Sub-Saharan Africa	Girls	1998	16	11.50
Sub-Saharan Africa	Girls	1999	16	11.62
Sub-Saharan Africa	Girls	2000	16	11.73
Sub-Saharan Africa	Girls	2001	16	11.86
Sub-Saharan Africa	Girls	2002	16	11.99
Sub-Saharan Africa	Girls	2003	16	12.12
Sub-Saharan Africa	Girls	2004	16	12.26
Sub-Saharan Africa	Girls	2005	16	12.40
Sub-Saharan Africa	Girls	2006	16	12.55
Sub-Saharan Africa	Girls	2007	16	12.69
Sub-Saharan Africa	Girls	2008	16	12.84
Sub-Saharan Africa	Girls	2009	16	12.99
Sub-Saharan Africa	Girls	2010	16	13.14
Sub-Saharan Africa	Girls	2011	16	13.30
Sub-Saharan Africa	Girls	2012	16	13.45
Sub-Saharan Africa	Girls	2013	16	13.61
Sub-Saharan Africa	Girls	2014	16	13.77
Sub-Saharan Africa	Girls	2015	16	13.94
Sub-Saharan Africa	Girls	2016	16	14.11
Sub-Saharan Africa	Girls	1975	17	10.58
Sub-Saharan Africa	Girls	1976	17	10.63
Sub-Saharan Africa	Girls	1977	17	10.69
Sub-Saharan Africa	Girls	1978	17	10.74
Sub-Saharan Africa	Girls	1979	17	10.80
Sub-Saharan Africa	Girls	1980	17	10.85
Sub-Saharan Africa	Girls	1981	17	10.91
Sub-Saharan Africa	Girls	1982	17	10.98
Sub-Saharan Africa	Girls	1983	17	11.04
Sub-Saharan Africa	Girls	1984	17	11.11
Sub-Saharan Africa	Girls	1985	17	11.17
Sub-Saharan Africa	Girls	1986	17	11.25
Sub-Saharan Africa	Girls	1987	17	11.32
Sub-Saharan Africa	Girls	1988	17	11.40
Sub-Saharan Africa	Girls	1989	17	11.48
Sub-Saharan Africa	Girls	1990	17	11.57
Sub-Saharan Africa	Girls	1991	17	11.65
Sub-Saharan Africa	Girls	1992	17	11.75
Sub-Saharan Africa	Girls	1993	17	11.84
Sub-Saharan Africa	Girls	1994	17	11.94
Sub-Saharan Africa	Girls	1995	17	12.04
Sub-Saharan Africa	Girls	1996	17	12.15
Sub-Saharan Africa	Girls	1997	17	12.26
Sub-Saharan Africa	Girls	1998	17	12.38
Sub-Saharan Africa	Girls	1999	17	12.50
Sub-Saharan Africa	Girls	2000	17	12.62
Sub-Saharan Africa	Girls	2001	17	12.75
Sub-Saharan Africa	Girls	2002	17	12.89
Sub-Saharan Africa	Girls	2003	17	13.03

Sub-Saharan Africa	Girls	2004	17	13.18
Sub-Saharan Africa	Girls	2005	17	13.32
Sub-Saharan Africa	Girls	2006	17	13.47
Sub-Saharan Africa	Girls	2007	17	13.63
Sub-Saharan Africa	Girls	2008	17	13.78
Sub-Saharan Africa	Girls	2009	17	13.94
Sub-Saharan Africa	Girls	2010	17	14.10
Sub-Saharan Africa	Girls	2011	17	14.26
Sub-Saharan Africa	Girls	2012	17	14.42
Sub-Saharan Africa	Girls	2013	17	14.58
Sub-Saharan Africa	Girls	2014	17	14.75
Sub-Saharan Africa	Girls	2015	17	14.92
Sub-Saharan Africa	Girls	2016	17	15.10
Sub-Saharan Africa	Girls	1975	18	11.10
Sub-Saharan Africa	Girls	1976	18	11.15
Sub-Saharan Africa	Girls	1977	18	11.21
Sub-Saharan Africa	Girls	1978	18	11.27
Sub-Saharan Africa	Girls	1979	18	11.33
Sub-Saharan Africa	Girls	1980	18	11.39
Sub-Saharan Africa	Girls	1981	18	11.45
Sub-Saharan Africa	Girls	1982	18	11.52
Sub-Saharan Africa	Girls	1983	18	11.59
Sub-Saharan Africa	Girls	1984	18	11.66
Sub-Saharan Africa	Girls	1985	18	11.74
Sub-Saharan Africa	Girls	1986	18	11.81
Sub-Saharan Africa	Girls	1987	18	11.89
Sub-Saharan Africa	Girls	1988	18	11.98
Sub-Saharan Africa	Girls	1989	18	12.07
Sub-Saharan Africa	Girls	1990	18	12.16
Sub-Saharan Africa	Girls	1991	18	12.26
Sub-Saharan Africa	Girls	1992	18	12.36
Sub-Saharan Africa	Girls	1993	18	12.46
Sub-Saharan Africa	Girls	1994	18	12.57
Sub-Saharan Africa	Girls	1995	18	12.68
Sub-Saharan Africa	Girls	1996	18	12.80
Sub-Saharan Africa	Girls	1997	18	12.92
Sub-Saharan Africa	Girls	1998	18	13.04
Sub-Saharan Africa	Girls	1999	18	13.17
Sub-Saharan Africa	Girls	2000	18	13.31
Sub-Saharan Africa	Girls	2001	18	13.45
Sub-Saharan Africa	Girls	2002	18	13.60
Sub-Saharan Africa	Girls	2003	18	13.75
Sub-Saharan Africa	Girls	2004	18	13.91
Sub-Saharan Africa	Girls	2005	18	14.07
Sub-Saharan Africa	Girls	2006	18	14.24
Sub-Saharan Africa	Girls	2007	18	14.40
Sub-Saharan Africa	Girls	2008	18	14.57
Sub-Saharan Africa	Girls	2009	18	14.74
Sub-Saharan Africa	Girls	2010	18	14.91
Sub-Saharan Africa	Girls	2011	18	15.08
Sub-Saharan Africa	Girls	2012	18	15.25
Sub-Saharan Africa	Girls	2013	18	15.43
Sub-Saharan Africa	Girls	2014	18	15.61
Sub-Saharan Africa	Girls	2015	18	15.79
Sub-Saharan Africa	Girls	2016	18	15.98
Sub-Saharan Africa	Girls	1975	Average	8.32
Sub-Saharan Africa	Girls	1976	Average	8.36
Sub-Saharan Africa	Girls	1977	Average	8.41
Sub-Saharan Africa	Girls	1978	Average	8.45

Sub-Saharan Africa	Girls	1979	Average	8.50
Sub-Saharan Africa	Girls	1980	Average	8.55
Sub-Saharan Africa	Girls	1981	Average	8.60
Sub-Saharan Africa	Girls	1982	Average	8.65
Sub-Saharan Africa	Girls	1983	Average	8.71
Sub-Saharan Africa	Girls	1984	Average	8.77
Sub-Saharan Africa	Girls	1985	Average	8.83
Sub-Saharan Africa	Girls	1986	Average	8.89
Sub-Saharan Africa	Girls	1987	Average	8.95
Sub-Saharan Africa	Girls	1988	Average	9.02
Sub-Saharan Africa	Girls	1989	Average	9.10
Sub-Saharan Africa	Girls	1990	Average	9.17
Sub-Saharan Africa	Girls	1991	Average	9.25
Sub-Saharan Africa	Girls	1992	Average	9.33
Sub-Saharan Africa	Girls	1993	Average	9.41
Sub-Saharan Africa	Girls	1994	Average	9.50
Sub-Saharan Africa	Girls	1995	Average	9.59
Sub-Saharan Africa	Girls	1996	Average	9.69
Sub-Saharan Africa	Girls	1997	Average	9.79
Sub-Saharan Africa	Girls	1998	Average	9.89
Sub-Saharan Africa	Girls	1999	Average	10.00
Sub-Saharan Africa	Girls	2000	Average	10.11
Sub-Saharan Africa	Girls	2001	Average	10.22
Sub-Saharan Africa	Girls	2002	Average	10.34
Sub-Saharan Africa	Girls	2003	Average	10.47
Sub-Saharan Africa	Girls	2004	Average	10.60
Sub-Saharan Africa	Girls	2005	Average	10.73
Sub-Saharan Africa	Girls	2006	Average	10.86
Sub-Saharan Africa	Girls	2007	Average	11.00
Sub-Saharan Africa	Girls	2008	Average	11.14
Sub-Saharan Africa	Girls	2009	Average	11.28
Sub-Saharan Africa	Girls	2010	Average	11.42
Sub-Saharan Africa	Girls	2011	Average	11.57
Sub-Saharan Africa	Girls	2012	Average	11.72
Sub-Saharan Africa	Girls	2013	Average	11.87
Sub-Saharan Africa	Girls	2014	Average	12.02
Sub-Saharan Africa	Girls	2015	Average	12.18
Sub-Saharan Africa	Girls	2016	Average	12.34
Sub-Saharan Africa	Girls	2017	Average	12.51
Sub-Saharan Africa	Girls	2018	Average	12.68
Sub-Saharan Africa	Girls	2019	Average	12.85
Sub-Saharan Africa	Girls	2020	Average	13.03
Sub-Saharan Africa	Girls	2021	Average	13.21
Sub-Saharan Africa	Girls	2022	Average	13.40
Sub-Saharan Africa	Girls	2023	Average	13.59
Sub-Saharan Africa	Girls	2024	Average	13.78
Sub-Saharan Africa	Girls	2025	Average	13.97
Sub-Saharan Africa	Girls	2026	Average	14.16
Sub-Saharan Africa	Girls	2027	Average	14.36
Sub-Saharan Africa	Girls	2028	Average	14.56
Sub-Saharan Africa	Girls	2029	Average	14.77
Sub-Saharan Africa	Girls	2030	Average	14.97

region	sex	year	age	Prevalence (%)
Central and Eastern Europe	Boys	1975	12	0.60
Central and Eastern Europe	Boys	1976	12	0.60
Central and Eastern Europe	Boys	1977	12	0.61
Central and Eastern Europe	Boys	1978	12	0.62
Central and Eastern Europe	Boys	1979	12	0.64
Central and Eastern Europe	Boys	1980	12	0.65
Central and Eastern Europe	Boys	1981	12	0.66
Central and Eastern Europe	Boys	1982	12	0.67
Central and Eastern Europe	Boys	1983	12	0.69
Central and Eastern Europe	Boys	1984	12	0.70
Central and Eastern Europe	Boys	1985	12	0.72
Central and Eastern Europe	Boys	1986	12	0.73
Central and Eastern Europe	Boys	1987	12	0.75
Central and Eastern Europe	Boys	1988	12	0.76
Central and Eastern Europe	Boys	1989	12	0.77
Central and Eastern Europe	Boys	1990	12	0.79
Central and Eastern Europe	Boys	1991	12	0.80
Central and Eastern Europe	Boys	1992	12	0.82
Central and Eastern Europe	Boys	1993	12	0.83
Central and Eastern Europe	Boys	1994	12	0.85
Central and Eastern Europe	Boys	1995	12	0.86
Central and Eastern Europe	Boys	1996	12	0.88
Central and Eastern Europe	Boys	1997	12	0.89
Central and Eastern Europe	Boys	1998	12	0.91
Central and Eastern Europe	Boys	1999	12	0.92
Central and Eastern Europe	Boys	2000	12	0.94
Central and Eastern Europe	Boys	2001	12	0.96
Central and Eastern Europe	Boys	2002	12	0.98
Central and Eastern Europe	Boys	2003	12	1.00
Central and Eastern Europe	Boys	2004	12	1.02
Central and Eastern Europe	Boys	2005	12	1.05
Central and Eastern Europe	Boys	2006	12	1.07
Central and Eastern Europe	Boys	2007	12	1.10
Central and Eastern Europe	Boys	2008	12	1.12
Central and Eastern Europe	Boys	2009	12	1.15
Central and Eastern Europe	Boys	2010	12	1.18
Central and Eastern Europe	Boys	2011	12	1.21
Central and Eastern Europe	Boys	2012	12	1.24
Central and Eastern Europe	Boys	2013	12	1.27
Central and Eastern Europe	Boys	2014	12	1.29
Central and Eastern Europe	Boys	2015	12	1.32
Central and Eastern Europe	Boys	2016	12	1.36
Central and Eastern Europe	Boys	1975	13	0.79
Central and Eastern Europe	Boys	1976	13	0.80
Central and Eastern Europe	Boys	1977	13	0.82
Central and Eastern Europe	Boys	1978	13	0.83
Central and Eastern Europe	Boys	1979	13	0.85
Central and Eastern Europe	Boys	1980	13	0.86
Central and Eastern Europe	Boys	1981	13	0.88
Central and Eastern Europe	Boys	1982	13	0.90
Central and Eastern Europe	Boys	1983	13	0.92

Central and Eastern Europe	Boys	1984	13	0.94
Central and Eastern Europe	Boys	1985	13	0.95
Central and Eastern Europe	Boys	1986	13	0.97
Central and Eastern Europe	Boys	1987	13	0.99
Central and Eastern Europe	Boys	1988	13	1.01
Central and Eastern Europe	Boys	1989	13	1.03
Central and Eastern Europe	Boys	1990	13	1.05
Central and Eastern Europe	Boys	1991	13	1.07
Central and Eastern Europe	Boys	1992	13	1.09
Central and Eastern Europe	Boys	1993	13	1.11
Central and Eastern Europe	Boys	1994	13	1.13
Central and Eastern Europe	Boys	1995	13	1.15
Central and Eastern Europe	Boys	1996	13	1.17
Central and Eastern Europe	Boys	1997	13	1.19
Central and Eastern Europe	Boys	1998	13	1.21
Central and Eastern Europe	Boys	1999	13	1.23
Central and Eastern Europe	Boys	2000	13	1.25
Central and Eastern Europe	Boys	2001	13	1.28
Central and Eastern Europe	Boys	2002	13	1.31
Central and Eastern Europe	Boys	2003	13	1.34
Central and Eastern Europe	Boys	2004	13	1.37
Central and Eastern Europe	Boys	2005	13	1.40
Central and Eastern Europe	Boys	2006	13	1.44
Central and Eastern Europe	Boys	2007	13	1.47
Central and Eastern Europe	Boys	2008	13	1.51
Central and Eastern Europe	Boys	2009	13	1.55
Central and Eastern Europe	Boys	2010	13	1.59
Central and Eastern Europe	Boys	2011	13	1.62
Central and Eastern Europe	Boys	2012	13	1.66
Central and Eastern Europe	Boys	2013	13	1.71
Central and Eastern Europe	Boys	2014	13	1.75
Central and Eastern Europe	Boys	2015	13	1.79
Central and Eastern Europe	Boys	2016	13	1.84
Central and Eastern Europe	Boys	1975	14	1.05
Central and Eastern Europe	Boys	1976	14	1.07
Central and Eastern Europe	Boys	1977	14	1.08
Central and Eastern Europe	Boys	1978	14	1.10
Central and Eastern Europe	Boys	1979	14	1.12
Central and Eastern Europe	Boys	1980	14	1.14
Central and Eastern Europe	Boys	1981	14	1.16
Central and Eastern Europe	Boys	1982	14	1.18
Central and Eastern Europe	Boys	1983	14	1.21
Central and Eastern Europe	Boys	1984	14	1.23
Central and Eastern Europe	Boys	1985	14	1.26
Central and Eastern Europe	Boys	1986	14	1.28
Central and Eastern Europe	Boys	1987	14	1.31
Central and Eastern Europe	Boys	1988	14	1.33
Central and Eastern Europe	Boys	1989	14	1.36
Central and Eastern Europe	Boys	1990	14	1.38
Central and Eastern Europe	Boys	1991	14	1.41
Central and Eastern Europe	Boys	1992	14	1.43
Central and Eastern Europe	Boys	1993	14	1.46

Central and Eastern Europe	Boys	1994	14	1.48
Central and Eastern Europe	Boys	1995	14	1.50
Central and Eastern Europe	Boys	1996	14	1.53
Central and Eastern Europe	Boys	1997	14	1.55
Central and Eastern Europe	Boys	1998	14	1.58
Central and Eastern Europe	Boys	1999	14	1.61
Central and Eastern Europe	Boys	2000	14	1.64
Central and Eastern Europe	Boys	2001	14	1.67
Central and Eastern Europe	Boys	2002	14	1.71
Central and Eastern Europe	Boys	2003	14	1.75
Central and Eastern Europe	Boys	2004	14	1.79
Central and Eastern Europe	Boys	2005	14	1.83
Central and Eastern Europe	Boys	2006	14	1.88
Central and Eastern Europe	Boys	2007	14	1.92
Central and Eastern Europe	Boys	2008	14	1.97
Central and Eastern Europe	Boys	2009	14	2.02
Central and Eastern Europe	Boys	2010	14	2.07
Central and Eastern Europe	Boys	2011	14	2.13
Central and Eastern Europe	Boys	2012	14	2.18
Central and Eastern Europe	Boys	2013	14	2.24
Central and Eastern Europe	Boys	2014	14	2.29
Central and Eastern Europe	Boys	2015	14	2.35
Central and Eastern Europe	Boys	2016	14	2.41
Central and Eastern Europe	Boys	1975	15	1.36
Central and Eastern Europe	Boys	1976	15	1.38
Central and Eastern Europe	Boys	1977	15	1.40
Central and Eastern Europe	Boys	1978	15	1.43
Central and Eastern Europe	Boys	1979	15	1.45
Central and Eastern Europe	Boys	1980	15	1.48
Central and Eastern Europe	Boys	1981	15	1.50
Central and Eastern Europe	Boys	1982	15	1.53
Central and Eastern Europe	Boys	1983	15	1.55
Central and Eastern Europe	Boys	1984	15	1.58
Central and Eastern Europe	Boys	1985	15	1.60
Central and Eastern Europe	Boys	1986	15	1.63
Central and Eastern Europe	Boys	1987	15	1.66
Central and Eastern Europe	Boys	1988	15	1.70
Central and Eastern Europe	Boys	1989	15	1.73
Central and Eastern Europe	Boys	1990	15	1.76
Central and Eastern Europe	Boys	1991	15	1.79
Central and Eastern Europe	Boys	1992	15	1.82
Central and Eastern Europe	Boys	1993	15	1.86
Central and Eastern Europe	Boys	1994	15	1.89
Central and Eastern Europe	Boys	1995	15	1.92
Central and Eastern Europe	Boys	1996	15	1.95
Central and Eastern Europe	Boys	1997	15	1.98
Central and Eastern Europe	Boys	1998	15	2.01
Central and Eastern Europe	Boys	1999	15	2.04
Central and Eastern Europe	Boys	2000	15	2.08
Central and Eastern Europe	Boys	2001	15	2.11
Central and Eastern Europe	Boys	2002	15	2.15
Central and Eastern Europe	Boys	2003	15	2.19

Central and Eastern Europe	Boys	2004	15	2.24
Central and Eastern Europe	Boys	2005	15	2.29
Central and Eastern Europe	Boys	2006	15	2.34
Central and Eastern Europe	Boys	2007	15	2.40
Central and Eastern Europe	Boys	2008	15	2.46
Central and Eastern Europe	Boys	2009	15	2.52
Central and Eastern Europe	Boys	2010	15	2.59
Central and Eastern Europe	Boys	2011	15	2.66
Central and Eastern Europe	Boys	2012	15	2.74
Central and Eastern Europe	Boys	2013	15	2.81
Central and Eastern Europe	Boys	2014	15	2.89
Central and Eastern Europe	Boys	2015	15	2.97
Central and Eastern Europe	Boys	2016	15	3.05
Central and Eastern Europe	Boys	1975	16	1.71
Central and Eastern Europe	Boys	1976	16	1.73
Central and Eastern Europe	Boys	1977	16	1.76
Central and Eastern Europe	Boys	1978	16	1.79
Central and Eastern Europe	Boys	1979	16	1.82
Central and Eastern Europe	Boys	1980	16	1.85
Central and Eastern Europe	Boys	1981	16	1.88
Central and Eastern Europe	Boys	1982	16	1.90
Central and Eastern Europe	Boys	1983	16	1.93
Central and Eastern Europe	Boys	1984	16	1.96
Central and Eastern Europe	Boys	1985	16	2.00
Central and Eastern Europe	Boys	1986	16	2.03
Central and Eastern Europe	Boys	1987	16	2.07
Central and Eastern Europe	Boys	1988	16	2.10
Central and Eastern Europe	Boys	1989	16	2.14
Central and Eastern Europe	Boys	1990	16	2.18
Central and Eastern Europe	Boys	1991	16	2.22
Central and Eastern Europe	Boys	1992	16	2.25
Central and Eastern Europe	Boys	1993	16	2.29
Central and Eastern Europe	Boys	1994	16	2.32
Central and Eastern Europe	Boys	1995	16	2.36
Central and Eastern Europe	Boys	1996	16	2.39
Central and Eastern Europe	Boys	1997	16	2.43
Central and Eastern Europe	Boys	1998	16	2.46
Central and Eastern Europe	Boys	1999	16	2.50
Central and Eastern Europe	Boys	2000	16	2.54
Central and Eastern Europe	Boys	2001	16	2.58
Central and Eastern Europe	Boys	2002	16	2.63
Central and Eastern Europe	Boys	2003	16	2.68
Central and Eastern Europe	Boys	2004	16	2.73
Central and Eastern Europe	Boys	2005	16	2.78
Central and Eastern Europe	Boys	2006	16	2.85
Central and Eastern Europe	Boys	2007	16	2.91
Central and Eastern Europe	Boys	2008	16	2.99
Central and Eastern Europe	Boys	2009	16	3.06
Central and Eastern Europe	Boys	2010	16	3.14
Central and Eastern Europe	Boys	2011	16	3.23
Central and Eastern Europe	Boys	2012	16	3.32
Central and Eastern Europe	Boys	2013	16	3.41

Central and Eastern Europe	Boys	2014	16	3.50
Central and Eastern Europe	Boys	2015	16	3.60
Central and Eastern Europe	Boys	2016	16	3.70
Central and Eastern Europe	Boys	1975	17	2.09
Central and Eastern Europe	Boys	1976	17	2.12
Central and Eastern Europe	Boys	1977	17	2.15
Central and Eastern Europe	Boys	1978	17	2.18
Central and Eastern Europe	Boys	1979	17	2.22
Central and Eastern Europe	Boys	1980	17	2.25
Central and Eastern Europe	Boys	1981	17	2.29
Central and Eastern Europe	Boys	1982	17	2.32
Central and Eastern Europe	Boys	1983	17	2.35
Central and Eastern Europe	Boys	1984	17	2.39
Central and Eastern Europe	Boys	1985	17	2.42
Central and Eastern Europe	Boys	1986	17	2.46
Central and Eastern Europe	Boys	1987	17	2.50
Central and Eastern Europe	Boys	1988	17	2.54
Central and Eastern Europe	Boys	1989	17	2.59
Central and Eastern Europe	Boys	1990	17	2.63
Central and Eastern Europe	Boys	1991	17	2.67
Central and Eastern Europe	Boys	1992	17	2.71
Central and Eastern Europe	Boys	1993	17	2.75
Central and Eastern Europe	Boys	1994	17	2.79
Central and Eastern Europe	Boys	1995	17	2.83
Central and Eastern Europe	Boys	1996	17	2.87
Central and Eastern Europe	Boys	1997	17	2.91
Central and Eastern Europe	Boys	1998	17	2.95
Central and Eastern Europe	Boys	1999	17	2.99
Central and Eastern Europe	Boys	2000	17	3.04
Central and Eastern Europe	Boys	2001	17	3.09
Central and Eastern Europe	Boys	2002	17	3.14
Central and Eastern Europe	Boys	2003	17	3.19
Central and Eastern Europe	Boys	2004	17	3.25
Central and Eastern Europe	Boys	2005	17	3.31
Central and Eastern Europe	Boys	2006	17	3.38
Central and Eastern Europe	Boys	2007	17	3.46
Central and Eastern Europe	Boys	2008	17	3.54
Central and Eastern Europe	Boys	2009	17	3.63
Central and Eastern Europe	Boys	2010	17	3.72
Central and Eastern Europe	Boys	2011	17	3.82
Central and Eastern Europe	Boys	2012	17	3.92
Central and Eastern Europe	Boys	2013	17	4.03
Central and Eastern Europe	Boys	2014	17	4.14
Central and Eastern Europe	Boys	2015	17	4.25
Central and Eastern Europe	Boys	2016	17	4.36
Central and Eastern Europe	Boys	1975	18	2.52
Central and Eastern Europe	Boys	1976	18	2.55
Central and Eastern Europe	Boys	1977	18	2.58
Central and Eastern Europe	Boys	1978	18	2.62
Central and Eastern Europe	Boys	1979	18	2.66
Central and Eastern Europe	Boys	1980	18	2.70
Central and Eastern Europe	Boys	1981	18	2.73

Central and Eastern Europe	Boys	1982	18	2.77
Central and Eastern Europe	Boys	1983	18	2.81
Central and Eastern Europe	Boys	1984	18	2.85
Central and Eastern Europe	Boys	1985	18	2.89
Central and Eastern Europe	Boys	1986	18	2.94
Central and Eastern Europe	Boys	1987	18	2.98
Central and Eastern Europe	Boys	1988	18	3.03
Central and Eastern Europe	Boys	1989	18	3.08
Central and Eastern Europe	Boys	1990	18	3.13
Central and Eastern Europe	Boys	1991	18	3.17
Central and Eastern Europe	Boys	1992	18	3.22
Central and Eastern Europe	Boys	1993	18	3.27
Central and Eastern Europe	Boys	1994	18	3.31
Central and Eastern Europe	Boys	1995	18	3.36
Central and Eastern Europe	Boys	1996	18	3.40
Central and Eastern Europe	Boys	1997	18	3.45
Central and Eastern Europe	Boys	1998	18	3.49
Central and Eastern Europe	Boys	1999	18	3.54
Central and Eastern Europe	Boys	2000	18	3.59
Central and Eastern Europe	Boys	2001	18	3.64
Central and Eastern Europe	Boys	2002	18	3.70
Central and Eastern Europe	Boys	2003	18	3.76
Central and Eastern Europe	Boys	2004	18	3.83
Central and Eastern Europe	Boys	2005	18	3.90
Central and Eastern Europe	Boys	2006	18	3.98
Central and Eastern Europe	Boys	2007	18	4.07
Central and Eastern Europe	Boys	2008	18	4.16
Central and Eastern Europe	Boys	2009	18	4.26
Central and Eastern Europe	Boys	2010	18	4.37
Central and Eastern Europe	Boys	2011	18	4.48
Central and Eastern Europe	Boys	2012	18	4.59
Central and Eastern Europe	Boys	2013	18	4.71
Central and Eastern Europe	Boys	2014	18	4.84
Central and Eastern Europe	Boys	2015	18	4.96
Central and Eastern Europe	Boys	2016	18	5.09
Central and Eastern Europe	Boys	1975	Average	1.44
Central and Eastern Europe	Boys	1976	Average	1.47
Central and Eastern Europe	Boys	1977	Average	1.49
Central and Eastern Europe	Boys	1978	Average	1.51
Central and Eastern Europe	Boys	1979	Average	1.53
Central and Eastern Europe	Boys	1980	Average	1.56
Central and Eastern Europe	Boys	1981	Average	1.59
Central and Eastern Europe	Boys	1982	Average	1.61
Central and Eastern Europe	Boys	1983	Average	1.64
Central and Eastern Europe	Boys	1984	Average	1.66
Central and Eastern Europe	Boys	1985	Average	1.69
Central and Eastern Europe	Boys	1986	Average	1.72
Central and Eastern Europe	Boys	1987	Average	1.75
Central and Eastern Europe	Boys	1988	Average	1.78
Central and Eastern Europe	Boys	1989	Average	1.81
Central and Eastern Europe	Boys	1990	Average	1.85
Central and Eastern Europe	Boys	1991	Average	1.88

Central and Eastern Europe	Boys	1992	Average	1.91
Central and Eastern Europe	Boys	1993	Average	1.94
Central and Eastern Europe	Boys	1994	Average	1.97
Central and Eastern Europe	Boys	1995	Average	2.00
Central and Eastern Europe	Boys	1996	Average	2.03
Central and Eastern Europe	Boys	1997	Average	2.06
Central and Eastern Europe	Boys	1998	Average	2.09
Central and Eastern Europe	Boys	1999	Average	2.12
Central and Eastern Europe	Boys	2000	Average	2.15
Central and Eastern Europe	Boys	2001	Average	2.19
Central and Eastern Europe	Boys	2002	Average	2.23
Central and Eastern Europe	Boys	2003	Average	2.27
Central and Eastern Europe	Boys	2004	Average	2.32
Central and Eastern Europe	Boys	2005	Average	2.37
Central and Eastern Europe	Boys	2006	Average	2.42
Central and Eastern Europe	Boys	2007	Average	2.48
Central and Eastern Europe	Boys	2008	Average	2.54
Central and Eastern Europe	Boys	2009	Average	2.60
Central and Eastern Europe	Boys	2010	Average	2.67
Central and Eastern Europe	Boys	2011	Average	2.74
Central and Eastern Europe	Boys	2012	Average	2.81
Central and Eastern Europe	Boys	2013	Average	2.88
Central and Eastern Europe	Boys	2014	Average	2.96
Central and Eastern Europe	Boys	2015	Average	3.04
Central and Eastern Europe	Boys	2016	Average	3.12
Central and Eastern Europe	Boys	2017	Average	3.20
Central and Eastern Europe	Boys	2018	Average	3.28
Central and Eastern Europe	Boys	2019	Average	3.36
Central and Eastern Europe	Boys	2020	Average	3.44
Central and Eastern Europe	Boys	2021	Average	3.53
Central and Eastern Europe	Boys	2022	Average	3.61
Central and Eastern Europe	Boys	2023	Average	3.70
Central and Eastern Europe	Boys	2024	Average	3.78
Central and Eastern Europe	Boys	2025	Average	3.87
Central and Eastern Europe	Boys	2026	Average	3.95
Central and Eastern Europe	Boys	2027	Average	4.04
Central and Eastern Europe	Boys	2028	Average	4.13
Central and Eastern Europe	Boys	2029	Average	4.21
Central and Eastern Europe	Boys	2030	Average	4.30
Central and Eastern Europe	Girls	1975	12	0.58
Central and Eastern Europe	Girls	1976	12	0.59
Central and Eastern Europe	Girls	1977	12	0.60
Central and Eastern Europe	Girls	1978	12	0.61
Central and Eastern Europe	Girls	1979	12	0.62
Central and Eastern Europe	Girls	1980	12	0.63
Central and Eastern Europe	Girls	1981	12	0.65
Central and Eastern Europe	Girls	1982	12	0.66
Central and Eastern Europe	Girls	1983	12	0.67
Central and Eastern Europe	Girls	1984	12	0.69
Central and Eastern Europe	Girls	1985	12	0.70
Central and Eastern Europe	Girls	1986	12	0.71
Central and Eastern Europe	Girls	1987	12	0.73

Central and Eastern Europe	Girls	1988	12	0.74
Central and Eastern Europe	Girls	1989	12	0.75
Central and Eastern Europe	Girls	1990	12	0.76
Central and Eastern Europe	Girls	1991	12	0.78
Central and Eastern Europe	Girls	1992	12	0.79
Central and Eastern Europe	Girls	1993	12	0.80
Central and Eastern Europe	Girls	1994	12	0.81
Central and Eastern Europe	Girls	1995	12	0.82
Central and Eastern Europe	Girls	1996	12	0.84
Central and Eastern Europe	Girls	1997	12	0.85
Central and Eastern Europe	Girls	1998	12	0.86
Central and Eastern Europe	Girls	1999	12	0.87
Central and Eastern Europe	Girls	2000	12	0.88
Central and Eastern Europe	Girls	2001	12	0.90
Central and Eastern Europe	Girls	2002	12	0.91
Central and Eastern Europe	Girls	2003	12	0.93
Central and Eastern Europe	Girls	2004	12	0.95
Central and Eastern Europe	Girls	2005	12	0.97
Central and Eastern Europe	Girls	2006	12	0.99
Central and Eastern Europe	Girls	2007	12	1.01
Central and Eastern Europe	Girls	2008	12	1.03
Central and Eastern Europe	Girls	2009	12	1.05
Central and Eastern Europe	Girls	2010	12	1.07
Central and Eastern Europe	Girls	2011	12	1.09
Central and Eastern Europe	Girls	2012	12	1.11
Central and Eastern Europe	Girls	2013	12	1.14
Central and Eastern Europe	Girls	2014	12	1.16
Central and Eastern Europe	Girls	2015	12	1.18
Central and Eastern Europe	Girls	2016	12	1.21
Central and Eastern Europe	Girls	1975	13	0.82
Central and Eastern Europe	Girls	1976	13	0.84
Central and Eastern Europe	Girls	1977	13	0.85
Central and Eastern Europe	Girls	1978	13	0.86
Central and Eastern Europe	Girls	1979	13	0.88
Central and Eastern Europe	Girls	1980	13	0.89
Central and Eastern Europe	Girls	1981	13	0.91
Central and Eastern Europe	Girls	1982	13	0.93
Central and Eastern Europe	Girls	1983	13	0.94
Central and Eastern Europe	Girls	1984	13	0.96
Central and Eastern Europe	Girls	1985	13	0.98
Central and Eastern Europe	Girls	1986	13	1.00
Central and Eastern Europe	Girls	1987	13	1.02
Central and Eastern Europe	Girls	1988	13	1.03
Central and Eastern Europe	Girls	1989	13	1.05
Central and Eastern Europe	Girls	1990	13	1.07
Central and Eastern Europe	Girls	1991	13	1.08
Central and Eastern Europe	Girls	1992	13	1.10
Central and Eastern Europe	Girls	1993	13	1.12
Central and Eastern Europe	Girls	1994	13	1.13
Central and Eastern Europe	Girls	1995	13	1.15
Central and Eastern Europe	Girls	1996	13	1.16
Central and Eastern Europe	Girls	1997	13	1.18

Central and Eastern Europe	Girls	1998	13	1.20
Central and Eastern Europe	Girls	1999	13	1.21
Central and Eastern Europe	Girls	2000	13	1.23
Central and Eastern Europe	Girls	2001	13	1.25
Central and Eastern Europe	Girls	2002	13	1.27
Central and Eastern Europe	Girls	2003	13	1.29
Central and Eastern Europe	Girls	2004	13	1.32
Central and Eastern Europe	Girls	2005	13	1.34
Central and Eastern Europe	Girls	2006	13	1.37
Central and Eastern Europe	Girls	2007	13	1.39
Central and Eastern Europe	Girls	2008	13	1.42
Central and Eastern Europe	Girls	2009	13	1.45
Central and Eastern Europe	Girls	2010	13	1.48
Central and Eastern Europe	Girls	2011	13	1.51
Central and Eastern Europe	Girls	2012	13	1.54
Central and Eastern Europe	Girls	2013	13	1.57
Central and Eastern Europe	Girls	2014	13	1.60
Central and Eastern Europe	Girls	2015	13	1.64
Central and Eastern Europe	Girls	2016	13	1.67
Central and Eastern Europe	Girls	1975	14	1.10
Central and Eastern Europe	Girls	1976	14	1.12
Central and Eastern Europe	Girls	1977	14	1.14
Central and Eastern Europe	Girls	1978	14	1.15
Central and Eastern Europe	Girls	1979	14	1.17
Central and Eastern Europe	Girls	1980	14	1.19
Central and Eastern Europe	Girls	1981	14	1.21
Central and Eastern Europe	Girls	1982	14	1.24
Central and Eastern Europe	Girls	1983	14	1.26
Central and Eastern Europe	Girls	1984	14	1.28
Central and Eastern Europe	Girls	1985	14	1.31
Central and Eastern Europe	Girls	1986	14	1.33
Central and Eastern Europe	Girls	1987	14	1.35
Central and Eastern Europe	Girls	1988	14	1.37
Central and Eastern Europe	Girls	1989	14	1.39
Central and Eastern Europe	Girls	1990	14	1.41
Central and Eastern Europe	Girls	1991	14	1.43
Central and Eastern Europe	Girls	1992	14	1.45
Central and Eastern Europe	Girls	1993	14	1.47
Central and Eastern Europe	Girls	1994	14	1.49
Central and Eastern Europe	Girls	1995	14	1.51
Central and Eastern Europe	Girls	1996	14	1.53
Central and Eastern Europe	Girls	1997	14	1.55
Central and Eastern Europe	Girls	1998	14	1.57
Central and Eastern Europe	Girls	1999	14	1.59
Central and Eastern Europe	Girls	2000	14	1.61
Central and Eastern Europe	Girls	2001	14	1.64
Central and Eastern Europe	Girls	2002	14	1.66
Central and Eastern Europe	Girls	2003	14	1.69
Central and Eastern Europe	Girls	2004	14	1.72
Central and Eastern Europe	Girls	2005	14	1.75
Central and Eastern Europe	Girls	2006	14	1.78
Central and Eastern Europe	Girls	2007	14	1.82

Central and Eastern Europe	Girls	2008	14	1.85
Central and Eastern Europe	Girls	2009	14	1.89
Central and Eastern Europe	Girls	2010	14	1.92
Central and Eastern Europe	Girls	2011	14	1.96
Central and Eastern Europe	Girls	2012	14	2.00
Central and Eastern Europe	Girls	2013	14	2.04
Central and Eastern Europe	Girls	2014	14	2.08
Central and Eastern Europe	Girls	2015	14	2.12
Central and Eastern Europe	Girls	2016	14	2.16
Central and Eastern Europe	Girls	1975	15	1.38
Central and Eastern Europe	Girls	1976	15	1.40
Central and Eastern Europe	Girls	1977	15	1.43
Central and Eastern Europe	Girls	1978	15	1.45
Central and Eastern Europe	Girls	1979	15	1.48
Central and Eastern Europe	Girls	1980	15	1.50
Central and Eastern Europe	Girls	1981	15	1.52
Central and Eastern Europe	Girls	1982	15	1.54
Central and Eastern Europe	Girls	1983	15	1.57
Central and Eastern Europe	Girls	1984	15	1.59
Central and Eastern Europe	Girls	1985	15	1.61
Central and Eastern Europe	Girls	1986	15	1.64
Central and Eastern Europe	Girls	1987	15	1.66
Central and Eastern Europe	Girls	1988	15	1.69
Central and Eastern Europe	Girls	1989	15	1.72
Central and Eastern Europe	Girls	1990	15	1.74
Central and Eastern Europe	Girls	1991	15	1.77
Central and Eastern Europe	Girls	1992	15	1.79
Central and Eastern Europe	Girls	1993	15	1.81
Central and Eastern Europe	Girls	1994	15	1.84
Central and Eastern Europe	Girls	1995	15	1.86
Central and Eastern Europe	Girls	1996	15	1.88
Central and Eastern Europe	Girls	1997	15	1.90
Central and Eastern Europe	Girls	1998	15	1.93
Central and Eastern Europe	Girls	1999	15	1.95
Central and Eastern Europe	Girls	2000	15	1.97
Central and Eastern Europe	Girls	2001	15	2.00
Central and Eastern Europe	Girls	2002	15	2.03
Central and Eastern Europe	Girls	2003	15	2.06
Central and Eastern Europe	Girls	2004	15	2.09
Central and Eastern Europe	Girls	2005	15	2.12
Central and Eastern Europe	Girls	2006	15	2.16
Central and Eastern Europe	Girls	2007	15	2.20
Central and Eastern Europe	Girls	2008	15	2.24
Central and Eastern Europe	Girls	2009	15	2.28
Central and Eastern Europe	Girls	2010	15	2.32
Central and Eastern Europe	Girls	2011	15	2.37
Central and Eastern Europe	Girls	2012	15	2.42
Central and Eastern Europe	Girls	2013	15	2.47
Central and Eastern Europe	Girls	2014	15	2.52
Central and Eastern Europe	Girls	2015	15	2.57
Central and Eastern Europe	Girls	2016	15	2.62
Central and Eastern Europe	Girls	1975	16	1.61

Central and Eastern Europe	Girls	1976	16	1.64
Central and Eastern Europe	Girls	1977	16	1.67
Central and Eastern Europe	Girls	1978	16	1.69
Central and Eastern Europe	Girls	1979	16	1.72
Central and Eastern Europe	Girls	1980	16	1.75
Central and Eastern Europe	Girls	1981	16	1.77
Central and Eastern Europe	Girls	1982	16	1.80
Central and Eastern Europe	Girls	1983	16	1.82
Central and Eastern Europe	Girls	1984	16	1.84
Central and Eastern Europe	Girls	1985	16	1.87
Central and Eastern Europe	Girls	1986	16	1.90
Central and Eastern Europe	Girls	1987	16	1.93
Central and Eastern Europe	Girls	1988	16	1.96
Central and Eastern Europe	Girls	1989	16	1.99
Central and Eastern Europe	Girls	1990	16	2.02
Central and Eastern Europe	Girls	1991	16	2.04
Central and Eastern Europe	Girls	1992	16	2.07
Central and Eastern Europe	Girls	1993	16	2.09
Central and Eastern Europe	Girls	1994	16	2.12
Central and Eastern Europe	Girls	1995	16	2.14
Central and Eastern Europe	Girls	1996	16	2.17
Central and Eastern Europe	Girls	1997	16	2.19
Central and Eastern Europe	Girls	1998	16	2.22
Central and Eastern Europe	Girls	1999	16	2.24
Central and Eastern Europe	Girls	2000	16	2.27
Central and Eastern Europe	Girls	2001	16	2.30
Central and Eastern Europe	Girls	2002	16	2.33
Central and Eastern Europe	Girls	2003	16	2.36
Central and Eastern Europe	Girls	2004	16	2.40
Central and Eastern Europe	Girls	2005	16	2.43
Central and Eastern Europe	Girls	2006	16	2.47
Central and Eastern Europe	Girls	2007	16	2.51
Central and Eastern Europe	Girls	2008	16	2.56
Central and Eastern Europe	Girls	2009	16	2.60
Central and Eastern Europe	Girls	2010	16	2.65
Central and Eastern Europe	Girls	2011	16	2.70
Central and Eastern Europe	Girls	2012	16	2.76
Central and Eastern Europe	Girls	2013	16	2.81
Central and Eastern Europe	Girls	2014	16	2.86
Central and Eastern Europe	Girls	2015	16	2.92
Central and Eastern Europe	Girls	2016	16	2.98
Central and Eastern Europe	Girls	1975	17	1.80
Central and Eastern Europe	Girls	1976	17	1.82
Central and Eastern Europe	Girls	1977	17	1.85
Central and Eastern Europe	Girls	1978	17	1.88
Central and Eastern Europe	Girls	1979	17	1.91
Central and Eastern Europe	Girls	1980	17	1.94
Central and Eastern Europe	Girls	1981	17	1.97
Central and Eastern Europe	Girls	1982	17	1.99
Central and Eastern Europe	Girls	1983	17	2.02
Central and Eastern Europe	Girls	1984	17	2.05
Central and Eastern Europe	Girls	1985	17	2.07

Central and Eastern Europe	Girls	1986	17	2.10
Central and Eastern Europe	Girls	1987	17	2.14
Central and Eastern Europe	Girls	1988	17	2.17
Central and Eastern Europe	Girls	1989	17	2.20
Central and Eastern Europe	Girls	1990	17	2.23
Central and Eastern Europe	Girls	1991	17	2.26
Central and Eastern Europe	Girls	1992	17	2.29
Central and Eastern Europe	Girls	1993	17	2.32
Central and Eastern Europe	Girls	1994	17	2.34
Central and Eastern Europe	Girls	1995	17	2.37
Central and Eastern Europe	Girls	1996	17	2.40
Central and Eastern Europe	Girls	1997	17	2.42
Central and Eastern Europe	Girls	1998	17	2.45
Central and Eastern Europe	Girls	1999	17	2.48
Central and Eastern Europe	Girls	2000	17	2.51
Central and Eastern Europe	Girls	2001	17	2.54
Central and Eastern Europe	Girls	2002	17	2.57
Central and Eastern Europe	Girls	2003	17	2.61
Central and Eastern Europe	Girls	2004	17	2.64
Central and Eastern Europe	Girls	2005	17	2.68
Central and Eastern Europe	Girls	2006	17	2.73
Central and Eastern Europe	Girls	2007	17	2.77
Central and Eastern Europe	Girls	2008	17	2.82
Central and Eastern Europe	Girls	2009	17	2.87
Central and Eastern Europe	Girls	2010	17	2.92
Central and Eastern Europe	Girls	2011	17	2.98
Central and Eastern Europe	Girls	2012	17	3.03
Central and Eastern Europe	Girls	2013	17	3.09
Central and Eastern Europe	Girls	2014	17	3.15
Central and Eastern Europe	Girls	2015	17	3.22
Central and Eastern Europe	Girls	2016	17	3.28
Central and Eastern Europe	Girls	1975	18	1.95
Central and Eastern Europe	Girls	1976	18	1.98
Central and Eastern Europe	Girls	1977	18	2.01
Central and Eastern Europe	Girls	1978	18	2.05
Central and Eastern Europe	Girls	1979	18	2.08
Central and Eastern Europe	Girls	1980	18	2.11
Central and Eastern Europe	Girls	1981	18	2.14
Central and Eastern Europe	Girls	1982	18	2.17
Central and Eastern Europe	Girls	1983	18	2.20
Central and Eastern Europe	Girls	1984	18	2.23
Central and Eastern Europe	Girls	1985	18	2.26
Central and Eastern Europe	Girls	1986	18	2.29
Central and Eastern Europe	Girls	1987	18	2.33
Central and Eastern Europe	Girls	1988	18	2.36
Central and Eastern Europe	Girls	1989	18	2.40
Central and Eastern Europe	Girls	1990	18	2.44
Central and Eastern Europe	Girls	1991	18	2.47
Central and Eastern Europe	Girls	1992	18	2.50
Central and Eastern Europe	Girls	1993	18	2.53
Central and Eastern Europe	Girls	1994	18	2.56
Central and Eastern Europe	Girls	1995	18	2.59

Central and Eastern Europe	Girls	1996	18	2.62
Central and Eastern Europe	Girls	1997	18	2.65
Central and Eastern Europe	Girls	1998	18	2.68
Central and Eastern Europe	Girls	1999	18	2.71
Central and Eastern Europe	Girls	2000	18	2.74
Central and Eastern Europe	Girls	2001	18	2.77
Central and Eastern Europe	Girls	2002	18	2.81
Central and Eastern Europe	Girls	2003	18	2.85
Central and Eastern Europe	Girls	2004	18	2.89
Central and Eastern Europe	Girls	2005	18	2.94
Central and Eastern Europe	Girls	2006	18	2.99
Central and Eastern Europe	Girls	2007	18	3.04
Central and Eastern Europe	Girls	2008	18	3.09
Central and Eastern Europe	Girls	2009	18	3.15
Central and Eastern Europe	Girls	2010	18	3.21
Central and Eastern Europe	Girls	2011	18	3.27
Central and Eastern Europe	Girls	2012	18	3.33
Central and Eastern Europe	Girls	2013	18	3.39
Central and Eastern Europe	Girls	2014	18	3.46
Central and Eastern Europe	Girls	2015	18	3.53
Central and Eastern Europe	Girls	2016	18	3.60
Central and Eastern Europe	Girls	1975	Average	1.32
Central and Eastern Europe	Girls	1976	Average	1.34
Central and Eastern Europe	Girls	1977	Average	1.36
Central and Eastern Europe	Girls	1978	Average	1.39
Central and Eastern Europe	Girls	1979	Average	1.41
Central and Eastern Europe	Girls	1980	Average	1.43
Central and Eastern Europe	Girls	1981	Average	1.45
Central and Eastern Europe	Girls	1982	Average	1.48
Central and Eastern Europe	Girls	1983	Average	1.50
Central and Eastern Europe	Girls	1984	Average	1.52
Central and Eastern Europe	Girls	1985	Average	1.54
Central and Eastern Europe	Girls	1986	Average	1.57
Central and Eastern Europe	Girls	1987	Average	1.59
Central and Eastern Europe	Girls	1988	Average	1.62
Central and Eastern Europe	Girls	1989	Average	1.64
Central and Eastern Europe	Girls	1990	Average	1.67
Central and Eastern Europe	Girls	1991	Average	1.69
Central and Eastern Europe	Girls	1992	Average	1.71
Central and Eastern Europe	Girls	1993	Average	1.74
Central and Eastern Europe	Girls	1994	Average	1.76
Central and Eastern Europe	Girls	1995	Average	1.78
Central and Eastern Europe	Girls	1996	Average	1.80
Central and Eastern Europe	Girls	1997	Average	1.82
Central and Eastern Europe	Girls	1998	Average	1.84
Central and Eastern Europe	Girls	1999	Average	1.86
Central and Eastern Europe	Girls	2000	Average	1.89
Central and Eastern Europe	Girls	2001	Average	1.91
Central and Eastern Europe	Girls	2002	Average	1.94
Central and Eastern Europe	Girls	2003	Average	1.97
Central and Eastern Europe	Girls	2004	Average	2.00
Central and Eastern Europe	Girls	2005	Average	2.03

Central and Eastern Europe	Girls	2006	Average	2.07
Central and Eastern Europe	Girls	2007	Average	2.11
Central and Eastern Europe	Girls	2008	Average	2.14
Central and Eastern Europe	Girls	2009	Average	2.18
Central and Eastern Europe	Girls	2010	Average	2.23
Central and Eastern Europe	Girls	2011	Average	2.27
Central and Eastern Europe	Girls	2012	Average	2.31
Central and Eastern Europe	Girls	2013	Average	2.36
Central and Eastern Europe	Girls	2014	Average	2.41
Central and Eastern Europe	Girls	2015	Average	2.45
Central and Eastern Europe	Girls	2016	Average	2.50
Central and Eastern Europe	Girls	2017	Average	2.55
Central and Eastern Europe	Girls	2018	Average	2.60
Central and Eastern Europe	Girls	2019	Average	2.66
Central and Eastern Europe	Girls	2020	Average	2.71
Central and Eastern Europe	Girls	2021	Average	2.76
Central and Eastern Europe	Girls	2022	Average	2.81
Central and Eastern Europe	Girls	2023	Average	2.87
Central and Eastern Europe	Girls	2024	Average	2.92
Central and Eastern Europe	Girls	2025	Average	2.98
Central and Eastern Europe	Girls	2026	Average	3.03
Central and Eastern Europe	Girls	2027	Average	3.09
Central and Eastern Europe	Girls	2028	Average	3.14
Central and Eastern Europe	Girls	2029	Average	3.20
Central and Eastern Europe	Girls	2030	Average	3.26
Central Asia and North Africa-Middle East	Boys	1975	12	0.49
Central Asia and North Africa-Middle East	Boys	1976	12	0.49
Central Asia and North Africa-Middle East	Boys	1977	12	0.50
Central Asia and North Africa-Middle East	Boys	1978	12	0.52
Central Asia and North Africa-Middle East	Boys	1979	12	0.53
Central Asia and North Africa-Middle East	Boys	1980	12	0.54
Central Asia and North Africa-Middle East	Boys	1981	12	0.55
Central Asia and North Africa-Middle East	Boys	1982	12	0.56
Central Asia and North Africa-Middle East	Boys	1983	12	0.58
Central Asia and North Africa-Middle East	Boys	1984	12	0.60
Central Asia and North Africa-Middle East	Boys	1985	12	0.61
Central Asia and North Africa-Middle East	Boys	1986	12	0.63
Central Asia and North Africa-Middle East	Boys	1987	12	0.65
Central Asia and North Africa-Middle East	Boys	1988	12	0.67
Central Asia and North Africa-Middle East	Boys	1989	12	0.69
Central Asia and North Africa-Middle East	Boys	1990	12	0.71
Central Asia and North Africa-Middle East	Boys	1991	12	0.73
Central Asia and North Africa-Middle East	Boys	1992	12	0.75
Central Asia and North Africa-Middle East	Boys	1993	12	0.77
Central Asia and North Africa-Middle East	Boys	1994	12	0.80
Central Asia and North Africa-Middle East	Boys	1995	12	0.82
Central Asia and North Africa-Middle East	Boys	1996	12	0.84
Central Asia and North Africa-Middle East	Boys	1997	12	0.87
Central Asia and North Africa-Middle East	Boys	1998	12	0.89
Central Asia and North Africa-Middle East	Boys	1999	12	0.92
Central Asia and North Africa-Middle East	Boys	2000	12	0.95
Central Asia and North Africa-Middle East	Boys	2001	12	0.98

Central Asia and North Africa-Middle East	Boys	2002	12	1.00
Central Asia and North Africa-Middle East	Boys	2003	12	1.03
Central Asia and North Africa-Middle East	Boys	2004	12	1.06
Central Asia and North Africa-Middle East	Boys	2005	12	1.10
Central Asia and North Africa-Middle East	Boys	2006	12	1.13
Central Asia and North Africa-Middle East	Boys	2007	12	1.16
Central Asia and North Africa-Middle East	Boys	2008	12	1.19
Central Asia and North Africa-Middle East	Boys	2009	12	1.22
Central Asia and North Africa-Middle East	Boys	2010	12	1.26
Central Asia and North Africa-Middle East	Boys	2011	12	1.29
Central Asia and North Africa-Middle East	Boys	2012	12	1.32
Central Asia and North Africa-Middle East	Boys	2013	12	1.35
Central Asia and North Africa-Middle East	Boys	2014	12	1.39
Central Asia and North Africa-Middle East	Boys	2015	12	1.42
Central Asia and North Africa-Middle East	Boys	2016	12	1.45
Central Asia and North Africa-Middle East	Boys	1975	13	0.64
Central Asia and North Africa-Middle East	Boys	1976	13	0.66
Central Asia and North Africa-Middle East	Boys	1977	13	0.67
Central Asia and North Africa-Middle East	Boys	1978	13	0.68
Central Asia and North Africa-Middle East	Boys	1979	13	0.70
Central Asia and North Africa-Middle East	Boys	1980	13	0.71
Central Asia and North Africa-Middle East	Boys	1981	13	0.73
Central Asia and North Africa-Middle East	Boys	1982	13	0.75
Central Asia and North Africa-Middle East	Boys	1983	13	0.77
Central Asia and North Africa-Middle East	Boys	1984	13	0.79
Central Asia and North Africa-Middle East	Boys	1985	13	0.81
Central Asia and North Africa-Middle East	Boys	1986	13	0.84
Central Asia and North Africa-Middle East	Boys	1987	13	0.86
Central Asia and North Africa-Middle East	Boys	1988	13	0.89
Central Asia and North Africa-Middle East	Boys	1989	13	0.92
Central Asia and North Africa-Middle East	Boys	1990	13	0.94
Central Asia and North Africa-Middle East	Boys	1991	13	0.97
Central Asia and North Africa-Middle East	Boys	1992	13	1.00
Central Asia and North Africa-Middle East	Boys	1993	13	1.03
Central Asia and North Africa-Middle East	Boys	1994	13	1.06
Central Asia and North Africa-Middle East	Boys	1995	13	1.09
Central Asia and North Africa-Middle East	Boys	1996	13	1.13
Central Asia and North Africa-Middle East	Boys	1997	13	1.16
Central Asia and North Africa-Middle East	Boys	1998	13	1.20
Central Asia and North Africa-Middle East	Boys	1999	13	1.23
Central Asia and North Africa-Middle East	Boys	2000	13	1.27
Central Asia and North Africa-Middle East	Boys	2001	13	1.31
Central Asia and North Africa-Middle East	Boys	2002	13	1.35
Central Asia and North Africa-Middle East	Boys	2003	13	1.39
Central Asia and North Africa-Middle East	Boys	2004	13	1.44
Central Asia and North Africa-Middle East	Boys	2005	13	1.48
Central Asia and North Africa-Middle East	Boys	2006	13	1.52
Central Asia and North Africa-Middle East	Boys	2007	13	1.57
Central Asia and North Africa-Middle East	Boys	2008	13	1.62
Central Asia and North Africa-Middle East	Boys	2009	13	1.66
Central Asia and North Africa-Middle East	Boys	2010	13	1.71
Central Asia and North Africa-Middle East	Boys	2011	13	1.75

Central Asia and North Africa-Middle East	Boys	2012	13	1.80
Central Asia and North Africa-Middle East	Boys	2013	13	1.85
Central Asia and North Africa-Middle East	Boys	2014	13	1.90
Central Asia and North Africa-Middle East	Boys	2015	13	1.94
Central Asia and North Africa-Middle East	Boys	2016	13	1.99
Central Asia and North Africa-Middle East	Boys	1975	14	0.86
Central Asia and North Africa-Middle East	Boys	1976	14	0.87
Central Asia and North Africa-Middle East	Boys	1977	14	0.89
Central Asia and North Africa-Middle East	Boys	1978	14	0.91
Central Asia and North Africa-Middle East	Boys	1979	14	0.92
Central Asia and North Africa-Middle East	Boys	1980	14	0.95
Central Asia and North Africa-Middle East	Boys	1981	14	0.97
Central Asia and North Africa-Middle East	Boys	1982	14	0.99
Central Asia and North Africa-Middle East	Boys	1983	14	1.02
Central Asia and North Africa-Middle East	Boys	1984	14	1.04
Central Asia and North Africa-Middle East	Boys	1985	14	1.07
Central Asia and North Africa-Middle East	Boys	1986	14	1.10
Central Asia and North Africa-Middle East	Boys	1987	14	1.13
Central Asia and North Africa-Middle East	Boys	1988	14	1.17
Central Asia and North Africa-Middle East	Boys	1989	14	1.20
Central Asia and North Africa-Middle East	Boys	1990	14	1.24
Central Asia and North Africa-Middle East	Boys	1991	14	1.28
Central Asia and North Africa-Middle East	Boys	1992	14	1.31
Central Asia and North Africa-Middle East	Boys	1993	14	1.35
Central Asia and North Africa-Middle East	Boys	1994	14	1.39
Central Asia and North Africa-Middle East	Boys	1995	14	1.44
Central Asia and North Africa-Middle East	Boys	1996	14	1.48
Central Asia and North Africa-Middle East	Boys	1997	14	1.52
Central Asia and North Africa-Middle East	Boys	1998	14	1.57
Central Asia and North Africa-Middle East	Boys	1999	14	1.62
Central Asia and North Africa-Middle East	Boys	2000	14	1.67
Central Asia and North Africa-Middle East	Boys	2001	14	1.72
Central Asia and North Africa-Middle East	Boys	2002	14	1.77
Central Asia and North Africa-Middle East	Boys	2003	14	1.83
Central Asia and North Africa-Middle East	Boys	2004	14	1.89
Central Asia and North Africa-Middle East	Boys	2005	14	1.94
Central Asia and North Africa-Middle East	Boys	2006	14	2.00
Central Asia and North Africa-Middle East	Boys	2007	14	2.06
Central Asia and North Africa-Middle East	Boys	2008	14	2.12
Central Asia and North Africa-Middle East	Boys	2009	14	2.19
Central Asia and North Africa-Middle East	Boys	2010	14	2.25
Central Asia and North Africa-Middle East	Boys	2011	14	2.31
Central Asia and North Africa-Middle East	Boys	2012	14	2.38
Central Asia and North Africa-Middle East	Boys	2013	14	2.44
Central Asia and North Africa-Middle East	Boys	2014	14	2.51
Central Asia and North Africa-Middle East	Boys	2015	14	2.57
Central Asia and North Africa-Middle East	Boys	2016	14	2.64
Central Asia and North Africa-Middle East	Boys	1975	15	1.11
Central Asia and North Africa-Middle East	Boys	1976	15	1.13
Central Asia and North Africa-Middle East	Boys	1977	15	1.15
Central Asia and North Africa-Middle East	Boys	1978	15	1.17
Central Asia and North Africa-Middle East	Boys	1979	15	1.20

Central Asia and North Africa-Middle East	Boys	1980	15	1.22
Central Asia and North Africa-Middle East	Boys	1981	15	1.25
Central Asia and North Africa-Middle East	Boys	1982	15	1.28
Central Asia and North Africa-Middle East	Boys	1983	15	1.31
Central Asia and North Africa-Middle East	Boys	1984	15	1.34
Central Asia and North Africa-Middle East	Boys	1985	15	1.38
Central Asia and North Africa-Middle East	Boys	1986	15	1.41
Central Asia and North Africa-Middle East	Boys	1987	15	1.45
Central Asia and North Africa-Middle East	Boys	1988	15	1.49
Central Asia and North Africa-Middle East	Boys	1989	15	1.54
Central Asia and North Africa-Middle East	Boys	1990	15	1.58
Central Asia and North Africa-Middle East	Boys	1991	15	1.62
Central Asia and North Africa-Middle East	Boys	1992	15	1.67
Central Asia and North Africa-Middle East	Boys	1993	15	1.72
Central Asia and North Africa-Middle East	Boys	1994	15	1.77
Central Asia and North Africa-Middle East	Boys	1995	15	1.82
Central Asia and North Africa-Middle East	Boys	1996	15	1.87
Central Asia and North Africa-Middle East	Boys	1997	15	1.93
Central Asia and North Africa-Middle East	Boys	1998	15	1.99
Central Asia and North Africa-Middle East	Boys	1999	15	2.05
Central Asia and North Africa-Middle East	Boys	2000	15	2.11
Central Asia and North Africa-Middle East	Boys	2001	15	2.17
Central Asia and North Africa-Middle East	Boys	2002	15	2.24
Central Asia and North Africa-Middle East	Boys	2003	15	2.31
Central Asia and North Africa-Middle East	Boys	2004	15	2.38
Central Asia and North Africa-Middle East	Boys	2005	15	2.45
Central Asia and North Africa-Middle East	Boys	2006	15	2.52
Central Asia and North Africa-Middle East	Boys	2007	15	2.59
Central Asia and North Africa-Middle East	Boys	2008	15	2.66
Central Asia and North Africa-Middle East	Boys	2009	15	2.74
Central Asia and North Africa-Middle East	Boys	2010	15	2.81
Central Asia and North Africa-Middle East	Boys	2011	15	2.89
Central Asia and North Africa-Middle East	Boys	2012	15	2.97
Central Asia and North Africa-Middle East	Boys	2013	15	3.05
Central Asia and North Africa-Middle East	Boys	2014	15	3.14
Central Asia and North Africa-Middle East	Boys	2015	15	3.22
Central Asia and North Africa-Middle East	Boys	2016	15	3.31
Central Asia and North Africa-Middle East	Boys	1975	16	1.41
Central Asia and North Africa-Middle East	Boys	1976	16	1.44
Central Asia and North Africa-Middle East	Boys	1977	16	1.46
Central Asia and North Africa-Middle East	Boys	1978	16	1.48
Central Asia and North Africa-Middle East	Boys	1979	16	1.51
Central Asia and North Africa-Middle East	Boys	1980	16	1.54
Central Asia and North Africa-Middle East	Boys	1981	16	1.57
Central Asia and North Africa-Middle East	Boys	1982	16	1.60
Central Asia and North Africa-Middle East	Boys	1983	16	1.64
Central Asia and North Africa-Middle East	Boys	1984	16	1.68
Central Asia and North Africa-Middle East	Boys	1985	16	1.72
Central Asia and North Africa-Middle East	Boys	1986	16	1.76
Central Asia and North Africa-Middle East	Boys	1987	16	1.81
Central Asia and North Africa-Middle East	Boys	1988	16	1.86
Central Asia and North Africa-Middle East	Boys	1989	16	1.91

Central Asia and North Africa-Middle East	Boys	1990	16	1.96
Central Asia and North Africa-Middle East	Boys	1991	16	2.01
Central Asia and North Africa-Middle East	Boys	1992	16	2.07
Central Asia and North Africa-Middle East	Boys	1993	16	2.12
Central Asia and North Africa-Middle East	Boys	1994	16	2.18
Central Asia and North Africa-Middle East	Boys	1995	16	2.24
Central Asia and North Africa-Middle East	Boys	1996	16	2.30
Central Asia and North Africa-Middle East	Boys	1997	16	2.37
Central Asia and North Africa-Middle East	Boys	1998	16	2.44
Central Asia and North Africa-Middle East	Boys	1999	16	2.51
Central Asia and North Africa-Middle East	Boys	2000	16	2.58
Central Asia and North Africa-Middle East	Boys	2001	16	2.66
Central Asia and North Africa-Middle East	Boys	2002	16	2.74
Central Asia and North Africa-Middle East	Boys	2003	16	2.82
Central Asia and North Africa-Middle East	Boys	2004	16	2.90
Central Asia and North Africa-Middle East	Boys	2005	16	2.98
Central Asia and North Africa-Middle East	Boys	2006	16	3.07
Central Asia and North Africa-Middle East	Boys	2007	16	3.15
Central Asia and North Africa-Middle East	Boys	2008	16	3.24
Central Asia and North Africa-Middle East	Boys	2009	16	3.33
Central Asia and North Africa-Middle East	Boys	2010	16	3.42
Central Asia and North Africa-Middle East	Boys	2011	16	3.51
Central Asia and North Africa-Middle East	Boys	2012	16	3.61
Central Asia and North Africa-Middle East	Boys	2013	16	3.71
Central Asia and North Africa-Middle East	Boys	2014	16	3.81
Central Asia and North Africa-Middle East	Boys	2015	16	3.92
Central Asia and North Africa-Middle East	Boys	2016	16	4.03
Central Asia and North Africa-Middle East	Boys	1975	17	1.75
Central Asia and North Africa-Middle East	Boys	1976	17	1.78
Central Asia and North Africa-Middle East	Boys	1977	17	1.80
Central Asia and North Africa-Middle East	Boys	1978	17	1.83
Central Asia and North Africa-Middle East	Boys	1979	17	1.86
Central Asia and North Africa-Middle East	Boys	1980	17	1.90
Central Asia and North Africa-Middle East	Boys	1981	17	1.93
Central Asia and North Africa-Middle East	Boys	1982	17	1.97
Central Asia and North Africa-Middle East	Boys	1983	17	2.01
Central Asia and North Africa-Middle East	Boys	1984	17	2.06
Central Asia and North Africa-Middle East	Boys	1985	17	2.10
Central Asia and North Africa-Middle East	Boys	1986	17	2.15
Central Asia and North Africa-Middle East	Boys	1987	17	2.21
Central Asia and North Africa-Middle East	Boys	1988	17	2.26
Central Asia and North Africa-Middle East	Boys	1989	17	2.32
Central Asia and North Africa-Middle East	Boys	1990	17	2.38
Central Asia and North Africa-Middle East	Boys	1991	17	2.44
Central Asia and North Africa-Middle East	Boys	1992	17	2.50
Central Asia and North Africa-Middle East	Boys	1993	17	2.57
Central Asia and North Africa-Middle East	Boys	1994	17	2.63
Central Asia and North Africa-Middle East	Boys	1995	17	2.70
Central Asia and North Africa-Middle East	Boys	1996	17	2.78
Central Asia and North Africa-Middle East	Boys	1997	17	2.85
Central Asia and North Africa-Middle East	Boys	1998	17	2.93
Central Asia and North Africa-Middle East	Boys	1999	17	3.01

Central Asia and North Africa-Middle East	Boys	2000	17	3.09
Central Asia and North Africa-Middle East	Boys	2001	17	3.18
Central Asia and North Africa-Middle East	Boys	2002	17	3.27
Central Asia and North Africa-Middle East	Boys	2003	17	3.37
Central Asia and North Africa-Middle East	Boys	2004	17	3.47
Central Asia and North Africa-Middle East	Boys	2005	17	3.56
Central Asia and North Africa-Middle East	Boys	2006	17	3.66
Central Asia and North Africa-Middle East	Boys	2007	17	3.76
Central Asia and North Africa-Middle East	Boys	2008	17	3.86
Central Asia and North Africa-Middle East	Boys	2009	17	3.96
Central Asia and North Africa-Middle East	Boys	2010	17	4.07
Central Asia and North Africa-Middle East	Boys	2011	17	4.18
Central Asia and North Africa-Middle East	Boys	2012	17	4.29
Central Asia and North Africa-Middle East	Boys	2013	17	4.41
Central Asia and North Africa-Middle East	Boys	2014	17	4.53
Central Asia and North Africa-Middle East	Boys	2015	17	4.65
Central Asia and North Africa-Middle East	Boys	2016	17	4.78
Central Asia and North Africa-Middle East	Boys	1975	18	2.13
Central Asia and North Africa-Middle East	Boys	1976	18	2.16
Central Asia and North Africa-Middle East	Boys	1977	18	2.19
Central Asia and North Africa-Middle East	Boys	1978	18	2.22
Central Asia and North Africa-Middle East	Boys	1979	18	2.26
Central Asia and North Africa-Middle East	Boys	1980	18	2.30
Central Asia and North Africa-Middle East	Boys	1981	18	2.34
Central Asia and North Africa-Middle East	Boys	1982	18	2.38
Central Asia and North Africa-Middle East	Boys	1983	18	2.43
Central Asia and North Africa-Middle East	Boys	1984	18	2.48
Central Asia and North Africa-Middle East	Boys	1985	18	2.54
Central Asia and North Africa-Middle East	Boys	1986	18	2.60
Central Asia and North Africa-Middle East	Boys	1987	18	2.66
Central Asia and North Africa-Middle East	Boys	1988	18	2.72
Central Asia and North Africa-Middle East	Boys	1989	18	2.79
Central Asia and North Africa-Middle East	Boys	1990	18	2.86
Central Asia and North Africa-Middle East	Boys	1991	18	2.93
Central Asia and North Africa-Middle East	Boys	1992	18	3.00
Central Asia and North Africa-Middle East	Boys	1993	18	3.08
Central Asia and North Africa-Middle East	Boys	1994	18	3.15
Central Asia and North Africa-Middle East	Boys	1995	18	3.24
Central Asia and North Africa-Middle East	Boys	1996	18	3.32
Central Asia and North Africa-Middle East	Boys	1997	18	3.40
Central Asia and North Africa-Middle East	Boys	1998	18	3.50
Central Asia and North Africa-Middle East	Boys	1999	18	3.59
Central Asia and North Africa-Middle East	Boys	2000	18	3.69
Central Asia and North Africa-Middle East	Boys	2001	18	3.79
Central Asia and North Africa-Middle East	Boys	2002	18	3.90
Central Asia and North Africa-Middle East	Boys	2003	18	4.01
Central Asia and North Africa-Middle East	Boys	2004	18	4.12
Central Asia and North Africa-Middle East	Boys	2005	18	4.23
Central Asia and North Africa-Middle East	Boys	2006	18	4.35
Central Asia and North Africa-Middle East	Boys	2007	18	4.46
Central Asia and North Africa-Middle East	Boys	2008	18	4.58
Central Asia and North Africa-Middle East	Boys	2009	18	4.69

Central Asia and North Africa-Middle East	Boys	2010	18	4.81
Central Asia and North Africa-Middle East	Boys	2011	18	4.94
Central Asia and North Africa-Middle East	Boys	2012	18	5.07
Central Asia and North Africa-Middle East	Boys	2013	18	5.20
Central Asia and North Africa-Middle East	Boys	2014	18	5.34
Central Asia and North Africa-Middle East	Boys	2015	18	5.49
Central Asia and North Africa-Middle East	Boys	2016	18	5.63
Central Asia and North Africa-Middle East	Boys	1975	Average	1.20
Central Asia and North Africa-Middle East	Boys	1976	Average	1.22
Central Asia and North Africa-Middle East	Boys	1977	Average	1.24
Central Asia and North Africa-Middle East	Boys	1978	Average	1.26
Central Asia and North Africa-Middle East	Boys	1979	Average	1.28
Central Asia and North Africa-Middle East	Boys	1980	Average	1.31
Central Asia and North Africa-Middle East	Boys	1981	Average	1.33
Central Asia and North Africa-Middle East	Boys	1982	Average	1.36
Central Asia and North Africa-Middle East	Boys	1983	Average	1.39
Central Asia and North Africa-Middle East	Boys	1984	Average	1.43
Central Asia and North Africa-Middle East	Boys	1985	Average	1.46
Central Asia and North Africa-Middle East	Boys	1986	Average	1.50
Central Asia and North Africa-Middle East	Boys	1987	Average	1.54
Central Asia and North Africa-Middle East	Boys	1988	Average	1.58
Central Asia and North Africa-Middle East	Boys	1989	Average	1.62
Central Asia and North Africa-Middle East	Boys	1990	Average	1.67
Central Asia and North Africa-Middle East	Boys	1991	Average	1.71
Central Asia and North Africa-Middle East	Boys	1992	Average	1.76
Central Asia and North Africa-Middle East	Boys	1993	Average	1.81
Central Asia and North Africa-Middle East	Boys	1994	Average	1.86
Central Asia and North Africa-Middle East	Boys	1995	Average	1.91
Central Asia and North Africa-Middle East	Boys	1996	Average	1.96
Central Asia and North Africa-Middle East	Boys	1997	Average	2.01
Central Asia and North Africa-Middle East	Boys	1998	Average	2.07
Central Asia and North Africa-Middle East	Boys	1999	Average	2.13
Central Asia and North Africa-Middle East	Boys	2000	Average	2.19
Central Asia and North Africa-Middle East	Boys	2001	Average	2.26
Central Asia and North Africa-Middle East	Boys	2002	Average	2.32
Central Asia and North Africa-Middle East	Boys	2003	Average	2.39
Central Asia and North Africa-Middle East	Boys	2004	Average	2.46
Central Asia and North Africa-Middle East	Boys	2005	Average	2.54
Central Asia and North Africa-Middle East	Boys	2006	Average	2.61
Central Asia and North Africa-Middle East	Boys	2007	Average	2.68
Central Asia and North Africa-Middle East	Boys	2008	Average	2.75
Central Asia and North Africa-Middle East	Boys	2009	Average	2.83
Central Asia and North Africa-Middle East	Boys	2010	Average	2.90
Central Asia and North Africa-Middle East	Boys	2011	Average	2.98
Central Asia and North Africa-Middle East	Boys	2012	Average	3.06
Central Asia and North Africa-Middle East	Boys	2013	Average	3.15
Central Asia and North Africa-Middle East	Boys	2014	Average	3.23
Central Asia and North Africa-Middle East	Boys	2015	Average	3.32
Central Asia and North Africa-Middle East	Boys	2016	Average	3.40
Central Asia and North Africa-Middle East	Boys	2017	Average	3.50
Central Asia and North Africa-Middle East	Boys	2018	Average	3.59
Central Asia and North Africa-Middle East	Boys	2019	Average	3.68

Central Asia and North Africa-Middle East	Boys	2020	Average	3.78
Central Asia and North Africa-Middle East	Boys	2021	Average	3.88
Central Asia and North Africa-Middle East	Boys	2022	Average	3.98
Central Asia and North Africa-Middle East	Boys	2023	Average	4.08
Central Asia and North Africa-Middle East	Boys	2024	Average	4.18
Central Asia and North Africa-Middle East	Boys	2025	Average	4.28
Central Asia and North Africa-Middle East	Boys	2026	Average	4.39
Central Asia and North Africa-Middle East	Boys	2027	Average	4.50
Central Asia and North Africa-Middle East	Boys	2028	Average	4.61
Central Asia and North Africa-Middle East	Boys	2029	Average	4.72
Central Asia and North Africa-Middle East	Boys	2030	Average	4.83
Central Asia and North Africa-Middle East	Girls	1975	12	0.61
Central Asia and North Africa-Middle East	Girls	1976	12	0.62
Central Asia and North Africa-Middle East	Girls	1977	12	0.64
Central Asia and North Africa-Middle East	Girls	1978	12	0.66
Central Asia and North Africa-Middle East	Girls	1979	12	0.67
Central Asia and North Africa-Middle East	Girls	1980	12	0.69
Central Asia and North Africa-Middle East	Girls	1981	12	0.71
Central Asia and North Africa-Middle East	Girls	1982	12	0.73
Central Asia and North Africa-Middle East	Girls	1983	12	0.76
Central Asia and North Africa-Middle East	Girls	1984	12	0.78
Central Asia and North Africa-Middle East	Girls	1985	12	0.80
Central Asia and North Africa-Middle East	Girls	1986	12	0.83
Central Asia and North Africa-Middle East	Girls	1987	12	0.85
Central Asia and North Africa-Middle East	Girls	1988	12	0.88
Central Asia and North Africa-Middle East	Girls	1989	12	0.91
Central Asia and North Africa-Middle East	Girls	1990	12	0.93
Central Asia and North Africa-Middle East	Girls	1991	12	0.96
Central Asia and North Africa-Middle East	Girls	1992	12	0.99
Central Asia and North Africa-Middle East	Girls	1993	12	1.02
Central Asia and North Africa-Middle East	Girls	1994	12	1.04
Central Asia and North Africa-Middle East	Girls	1995	12	1.07
Central Asia and North Africa-Middle East	Girls	1996	12	1.10
Central Asia and North Africa-Middle East	Girls	1997	12	1.13
Central Asia and North Africa-Middle East	Girls	1998	12	1.16
Central Asia and North Africa-Middle East	Girls	1999	12	1.19
Central Asia and North Africa-Middle East	Girls	2000	12	1.23
Central Asia and North Africa-Middle East	Girls	2001	12	1.26
Central Asia and North Africa-Middle East	Girls	2002	12	1.29
Central Asia and North Africa-Middle East	Girls	2003	12	1.32
Central Asia and North Africa-Middle East	Girls	2004	12	1.36
Central Asia and North Africa-Middle East	Girls	2005	12	1.39
Central Asia and North Africa-Middle East	Girls	2006	12	1.43
Central Asia and North Africa-Middle East	Girls	2007	12	1.46
Central Asia and North Africa-Middle East	Girls	2008	12	1.50
Central Asia and North Africa-Middle East	Girls	2009	12	1.54
Central Asia and North Africa-Middle East	Girls	2010	12	1.57
Central Asia and North Africa-Middle East	Girls	2011	12	1.61
Central Asia and North Africa-Middle East	Girls	2012	12	1.65
Central Asia and North Africa-Middle East	Girls	2013	12	1.68
Central Asia and North Africa-Middle East	Girls	2014	12	1.72
Central Asia and North Africa-Middle East	Girls	2015	12	1.75

Central Asia and North Africa-Middle East	Girls	2016	12	1.79
Central Asia and North Africa-Middle East	Girls	1975	13	0.86
Central Asia and North Africa-Middle East	Girls	1976	13	0.89
Central Asia and North Africa-Middle East	Girls	1977	13	0.91
Central Asia and North Africa-Middle East	Girls	1978	13	0.93
Central Asia and North Africa-Middle East	Girls	1979	13	0.96
Central Asia and North Africa-Middle East	Girls	1980	13	0.98
Central Asia and North Africa-Middle East	Girls	1981	13	1.01
Central Asia and North Africa-Middle East	Girls	1982	13	1.04
Central Asia and North Africa-Middle East	Girls	1983	13	1.07
Central Asia and North Africa-Middle East	Girls	1984	13	1.10
Central Asia and North Africa-Middle East	Girls	1985	13	1.14
Central Asia and North Africa-Middle East	Girls	1986	13	1.17
Central Asia and North Africa-Middle East	Girls	1987	13	1.21
Central Asia and North Africa-Middle East	Girls	1988	13	1.24
Central Asia and North Africa-Middle East	Girls	1989	13	1.28
Central Asia and North Africa-Middle East	Girls	1990	13	1.32
Central Asia and North Africa-Middle East	Girls	1991	13	1.35
Central Asia and North Africa-Middle East	Girls	1992	13	1.39
Central Asia and North Africa-Middle East	Girls	1993	13	1.43
Central Asia and North Africa-Middle East	Girls	1994	13	1.47
Central Asia and North Africa-Middle East	Girls	1995	13	1.51
Central Asia and North Africa-Middle East	Girls	1996	13	1.55
Central Asia and North Africa-Middle East	Girls	1997	13	1.59
Central Asia and North Africa-Middle East	Girls	1998	13	1.63
Central Asia and North Africa-Middle East	Girls	1999	13	1.68
Central Asia and North Africa-Middle East	Girls	2000	13	1.72
Central Asia and North Africa-Middle East	Girls	2001	13	1.76
Central Asia and North Africa-Middle East	Girls	2002	13	1.81
Central Asia and North Africa-Middle East	Girls	2003	13	1.85
Central Asia and North Africa-Middle East	Girls	2004	13	1.90
Central Asia and North Africa-Middle East	Girls	2005	13	1.95
Central Asia and North Africa-Middle East	Girls	2006	13	2.00
Central Asia and North Africa-Middle East	Girls	2007	13	2.05
Central Asia and North Africa-Middle East	Girls	2008	13	2.10
Central Asia and North Africa-Middle East	Girls	2009	13	2.15
Central Asia and North Africa-Middle East	Girls	2010	13	2.20
Central Asia and North Africa-Middle East	Girls	2011	13	2.25
Central Asia and North Africa-Middle East	Girls	2012	13	2.30
Central Asia and North Africa-Middle East	Girls	2013	13	2.35
Central Asia and North Africa-Middle East	Girls	2014	13	2.40
Central Asia and North Africa-Middle East	Girls	2015	13	2.45
Central Asia and North Africa-Middle East	Girls	2016	13	2.51
Central Asia and North Africa-Middle East	Girls	1975	14	1.17
Central Asia and North Africa-Middle East	Girls	1976	14	1.20
Central Asia and North Africa-Middle East	Girls	1977	14	1.23
Central Asia and North Africa-Middle East	Girls	1978	14	1.26
Central Asia and North Africa-Middle East	Girls	1979	14	1.29
Central Asia and North Africa-Middle East	Girls	1980	14	1.32
Central Asia and North Africa-Middle East	Girls	1981	14	1.36
Central Asia and North Africa-Middle East	Girls	1982	14	1.39
Central Asia and North Africa-Middle East	Girls	1983	14	1.43

Central Asia and North Africa-Middle East	Girls	1984	14	1.47
Central Asia and North Africa-Middle East	Girls	1985	14	1.51
Central Asia and North Africa-Middle East	Girls	1986	14	1.56
Central Asia and North Africa-Middle East	Girls	1987	14	1.60
Central Asia and North Africa-Middle East	Girls	1988	14	1.65
Central Asia and North Africa-Middle East	Girls	1989	14	1.70
Central Asia and North Africa-Middle East	Girls	1990	14	1.74
Central Asia and North Africa-Middle East	Girls	1991	14	1.79
Central Asia and North Africa-Middle East	Girls	1992	14	1.84
Central Asia and North Africa-Middle East	Girls	1993	14	1.89
Central Asia and North Africa-Middle East	Girls	1994	14	1.94
Central Asia and North Africa-Middle East	Girls	1995	14	1.99
Central Asia and North Africa-Middle East	Girls	1996	14	2.04
Central Asia and North Africa-Middle East	Girls	1997	14	2.09
Central Asia and North Africa-Middle East	Girls	1998	14	2.15
Central Asia and North Africa-Middle East	Girls	1999	14	2.20
Central Asia and North Africa-Middle East	Girls	2000	14	2.25
Central Asia and North Africa-Middle East	Girls	2001	14	2.31
Central Asia and North Africa-Middle East	Girls	2002	14	2.37
Central Asia and North Africa-Middle East	Girls	2003	14	2.42
Central Asia and North Africa-Middle East	Girls	2004	14	2.48
Central Asia and North Africa-Middle East	Girls	2005	14	2.54
Central Asia and North Africa-Middle East	Girls	2006	14	2.60
Central Asia and North Africa-Middle East	Girls	2007	14	2.66
Central Asia and North Africa-Middle East	Girls	2008	14	2.73
Central Asia and North Africa-Middle East	Girls	2009	14	2.79
Central Asia and North Africa-Middle East	Girls	2010	14	2.86
Central Asia and North Africa-Middle East	Girls	2011	14	2.92
Central Asia and North Africa-Middle East	Girls	2012	14	2.99
Central Asia and North Africa-Middle East	Girls	2013	14	3.05
Central Asia and North Africa-Middle East	Girls	2014	14	3.12
Central Asia and North Africa-Middle East	Girls	2015	14	3.18
Central Asia and North Africa-Middle East	Girls	2016	14	3.25
Central Asia and North Africa-Middle East	Girls	1975	15	1.47
Central Asia and North Africa-Middle East	Girls	1976	15	1.50
Central Asia and North Africa-Middle East	Girls	1977	15	1.53
Central Asia and North Africa-Middle East	Girls	1978	15	1.57
Central Asia and North Africa-Middle East	Girls	1979	15	1.61
Central Asia and North Africa-Middle East	Girls	1980	15	1.64
Central Asia and North Africa-Middle East	Girls	1981	15	1.69
Central Asia and North Africa-Middle East	Girls	1982	15	1.73
Central Asia and North Africa-Middle East	Girls	1983	15	1.78
Central Asia and North Africa-Middle East	Girls	1984	15	1.83
Central Asia and North Africa-Middle East	Girls	1985	15	1.88
Central Asia and North Africa-Middle East	Girls	1986	15	1.93
Central Asia and North Africa-Middle East	Girls	1987	15	1.98
Central Asia and North Africa-Middle East	Girls	1988	15	2.04
Central Asia and North Africa-Middle East	Girls	1989	15	2.09
Central Asia and North Africa-Middle East	Girls	1990	15	2.15
Central Asia and North Africa-Middle East	Girls	1991	15	2.21
Central Asia and North Africa-Middle East	Girls	1992	15	2.26
Central Asia and North Africa-Middle East	Girls	1993	15	2.32

Central Asia and North Africa-Middle East	Girls	1994	15	2.38
Central Asia and North Africa-Middle East	Girls	1995	15	2.44
Central Asia and North Africa-Middle East	Girls	1996	15	2.50
Central Asia and North Africa-Middle East	Girls	1997	15	2.56
Central Asia and North Africa-Middle East	Girls	1998	15	2.63
Central Asia and North Africa-Middle East	Girls	1999	15	2.69
Central Asia and North Africa-Middle East	Girls	2000	15	2.75
Central Asia and North Africa-Middle East	Girls	2001	15	2.82
Central Asia and North Africa-Middle East	Girls	2002	15	2.88
Central Asia and North Africa-Middle East	Girls	2003	15	2.95
Central Asia and North Africa-Middle East	Girls	2004	15	3.02
Central Asia and North Africa-Middle East	Girls	2005	15	3.08
Central Asia and North Africa-Middle East	Girls	2006	15	3.15
Central Asia and North Africa-Middle East	Girls	2007	15	3.21
Central Asia and North Africa-Middle East	Girls	2008	15	3.28
Central Asia and North Africa-Middle East	Girls	2009	15	3.34
Central Asia and North Africa-Middle East	Girls	2010	15	3.41
Central Asia and North Africa-Middle East	Girls	2011	15	3.48
Central Asia and North Africa-Middle East	Girls	2012	15	3.56
Central Asia and North Africa-Middle East	Girls	2013	15	3.64
Central Asia and North Africa-Middle East	Girls	2014	15	3.72
Central Asia and North Africa-Middle East	Girls	2015	15	3.80
Central Asia and North Africa-Middle East	Girls	2016	15	3.89
Central Asia and North Africa-Middle East	Girls	1975	16	1.73
Central Asia and North Africa-Middle East	Girls	1976	16	1.76
Central Asia and North Africa-Middle East	Girls	1977	16	1.80
Central Asia and North Africa-Middle East	Girls	1978	16	1.84
Central Asia and North Africa-Middle East	Girls	1979	16	1.88
Central Asia and North Africa-Middle East	Girls	1980	16	1.92
Central Asia and North Africa-Middle East	Girls	1981	16	1.97
Central Asia and North Africa-Middle East	Girls	1982	16	2.02
Central Asia and North Africa-Middle East	Girls	1983	16	2.07
Central Asia and North Africa-Middle East	Girls	1984	16	2.13
Central Asia and North Africa-Middle East	Girls	1985	16	2.19
Central Asia and North Africa-Middle East	Girls	1986	16	2.24
Central Asia and North Africa-Middle East	Girls	1987	16	2.30
Central Asia and North Africa-Middle East	Girls	1988	16	2.37
Central Asia and North Africa-Middle East	Girls	1989	16	2.43
Central Asia and North Africa-Middle East	Girls	1990	16	2.49
Central Asia and North Africa-Middle East	Girls	1991	16	2.56
Central Asia and North Africa-Middle East	Girls	1992	16	2.62
Central Asia and North Africa-Middle East	Girls	1993	16	2.69
Central Asia and North Africa-Middle East	Girls	1994	16	2.75
Central Asia and North Africa-Middle East	Girls	1995	16	2.82
Central Asia and North Africa-Middle East	Girls	1996	16	2.89
Central Asia and North Africa-Middle East	Girls	1997	16	2.95
Central Asia and North Africa-Middle East	Girls	1998	16	3.02
Central Asia and North Africa-Middle East	Girls	1999	16	3.10
Central Asia and North Africa-Middle East	Girls	2000	16	3.17
Central Asia and North Africa-Middle East	Girls	2001	16	3.24
Central Asia and North Africa-Middle East	Girls	2002	16	3.31
Central Asia and North Africa-Middle East	Girls	2003	16	3.39

Central Asia and North Africa-Middle East	Girls	2004	16	3.46
Central Asia and North Africa-Middle East	Girls	2005	16	3.53
Central Asia and North Africa-Middle East	Girls	2006	16	3.60
Central Asia and North Africa-Middle East	Girls	2007	16	3.68
Central Asia and North Africa-Middle East	Girls	2008	16	3.75
Central Asia and North Africa-Middle East	Girls	2009	16	3.82
Central Asia and North Africa-Middle East	Girls	2010	16	3.90
Central Asia and North Africa-Middle East	Girls	2011	16	3.98
Central Asia and North Africa-Middle East	Girls	2012	16	4.06
Central Asia and North Africa-Middle East	Girls	2013	16	4.15
Central Asia and North Africa-Middle East	Girls	2014	16	4.24
Central Asia and North Africa-Middle East	Girls	2015	16	4.33
Central Asia and North Africa-Middle East	Girls	2016	16	4.42
Central Asia and North Africa-Middle East	Girls	1975	17	1.93
Central Asia and North Africa-Middle East	Girls	1976	17	1.97
Central Asia and North Africa-Middle East	Girls	1977	17	2.02
Central Asia and North Africa-Middle East	Girls	1978	17	2.06
Central Asia and North Africa-Middle East	Girls	1979	17	2.11
Central Asia and North Africa-Middle East	Girls	1980	17	2.15
Central Asia and North Africa-Middle East	Girls	1981	17	2.21
Central Asia and North Africa-Middle East	Girls	1982	17	2.26
Central Asia and North Africa-Middle East	Girls	1983	17	2.32
Central Asia and North Africa-Middle East	Girls	1984	17	2.38
Central Asia and North Africa-Middle East	Girls	1985	17	2.44
Central Asia and North Africa-Middle East	Girls	1986	17	2.51
Central Asia and North Africa-Middle East	Girls	1987	17	2.57
Central Asia and North Africa-Middle East	Girls	1988	17	2.64
Central Asia and North Africa-Middle East	Girls	1989	17	2.71
Central Asia and North Africa-Middle East	Girls	1990	17	2.78
Central Asia and North Africa-Middle East	Girls	1991	17	2.85
Central Asia and North Africa-Middle East	Girls	1992	17	2.93
Central Asia and North Africa-Middle East	Girls	1993	17	3.00
Central Asia and North Africa-Middle East	Girls	1994	17	3.07
Central Asia and North Africa-Middle East	Girls	1995	17	3.15
Central Asia and North Africa-Middle East	Girls	1996	17	3.22
Central Asia and North Africa-Middle East	Girls	1997	17	3.30
Central Asia and North Africa-Middle East	Girls	1998	17	3.38
Central Asia and North Africa-Middle East	Girls	1999	17	3.45
Central Asia and North Africa-Middle East	Girls	2000	17	3.53
Central Asia and North Africa-Middle East	Girls	2001	17	3.61
Central Asia and North Africa-Middle East	Girls	2002	17	3.70
Central Asia and North Africa-Middle East	Girls	2003	17	3.78
Central Asia and North Africa-Middle East	Girls	2004	17	3.86
Central Asia and North Africa-Middle East	Girls	2005	17	3.94
Central Asia and North Africa-Middle East	Girls	2006	17	4.02
Central Asia and North Africa-Middle East	Girls	2007	17	4.10
Central Asia and North Africa-Middle East	Girls	2008	17	4.18
Central Asia and North Africa-Middle East	Girls	2009	17	4.26
Central Asia and North Africa-Middle East	Girls	2010	17	4.34
Central Asia and North Africa-Middle East	Girls	2011	17	4.43
Central Asia and North Africa-Middle East	Girls	2012	17	4.52
Central Asia and North Africa-Middle East	Girls	2013	17	4.61

Central Asia and North Africa-Middle East	Girls	2014	17	4.71
Central Asia and North Africa-Middle East	Girls	2015	17	4.81
Central Asia and North Africa-Middle East	Girls	2016	17	4.92
Central Asia and North Africa-Middle East	Girls	1975	18	2.12
Central Asia and North Africa-Middle East	Girls	1976	18	2.17
Central Asia and North Africa-Middle East	Girls	1977	18	2.21
Central Asia and North Africa-Middle East	Girls	1978	18	2.26
Central Asia and North Africa-Middle East	Girls	1979	18	2.32
Central Asia and North Africa-Middle East	Girls	1980	18	2.37
Central Asia and North Africa-Middle East	Girls	1981	18	2.43
Central Asia and North Africa-Middle East	Girls	1982	18	2.49
Central Asia and North Africa-Middle East	Girls	1983	18	2.56
Central Asia and North Africa-Middle East	Girls	1984	18	2.63
Central Asia and North Africa-Middle East	Girls	1985	18	2.70
Central Asia and North Africa-Middle East	Girls	1986	18	2.78
Central Asia and North Africa-Middle East	Girls	1987	18	2.85
Central Asia and North Africa-Middle East	Girls	1988	18	2.93
Central Asia and North Africa-Middle East	Girls	1989	18	3.01
Central Asia and North Africa-Middle East	Girls	1990	18	3.09
Central Asia and North Africa-Middle East	Girls	1991	18	3.17
Central Asia and North Africa-Middle East	Girls	1992	18	3.25
Central Asia and North Africa-Middle East	Girls	1993	18	3.34
Central Asia and North Africa-Middle East	Girls	1994	18	3.42
Central Asia and North Africa-Middle East	Girls	1995	18	3.51
Central Asia and North Africa-Middle East	Girls	1996	18	3.59
Central Asia and North Africa-Middle East	Girls	1997	18	3.68
Central Asia and North Africa-Middle East	Girls	1998	18	3.77
Central Asia and North Africa-Middle East	Girls	1999	18	3.86
Central Asia and North Africa-Middle East	Girls	2000	18	3.95
Central Asia and North Africa-Middle East	Girls	2001	18	4.04
Central Asia and North Africa-Middle East	Girls	2002	18	4.13
Central Asia and North Africa-Middle East	Girls	2003	18	4.22
Central Asia and North Africa-Middle East	Girls	2004	18	4.32
Central Asia and North Africa-Middle East	Girls	2005	18	4.41
Central Asia and North Africa-Middle East	Girls	2006	18	4.49
Central Asia and North Africa-Middle East	Girls	2007	18	4.58
Central Asia and North Africa-Middle East	Girls	2008	18	4.67
Central Asia and North Africa-Middle East	Girls	2009	18	4.76
Central Asia and North Africa-Middle East	Girls	2010	18	4.85
Central Asia and North Africa-Middle East	Girls	2011	18	4.95
Central Asia and North Africa-Middle East	Girls	2012	18	5.05
Central Asia and North Africa-Middle East	Girls	2013	18	5.16
Central Asia and North Africa-Middle East	Girls	2014	18	5.27
Central Asia and North Africa-Middle East	Girls	2015	18	5.38
Central Asia and North Africa-Middle East	Girls	2016	18	5.49
Central Asia and North Africa-Middle East	Girls	1975	Average	1.41
Central Asia and North Africa-Middle East	Girls	1976	Average	1.44
Central Asia and North Africa-Middle East	Girls	1977	Average	1.48
Central Asia and North Africa-Middle East	Girls	1978	Average	1.51
Central Asia and North Africa-Middle East	Girls	1979	Average	1.55
Central Asia and North Africa-Middle East	Girls	1980	Average	1.59
Central Asia and North Africa-Middle East	Girls	1981	Average	1.63

Central Asia and North Africa-Middle East	Girls	1982	Average	1.67
Central Asia and North Africa-Middle East	Girls	1983	Average	1.71
Central Asia and North Africa-Middle East	Girls	1984	Average	1.76
Central Asia and North Africa-Middle East	Girls	1985	Average	1.81
Central Asia and North Africa-Middle East	Girls	1986	Average	1.86
Central Asia and North Africa-Middle East	Girls	1987	Average	1.91
Central Asia and North Africa-Middle East	Girls	1988	Average	1.96
Central Asia and North Africa-Middle East	Girls	1989	Average	2.02
Central Asia and North Africa-Middle East	Girls	1990	Average	2.07
Central Asia and North Africa-Middle East	Girls	1991	Average	2.13
Central Asia and North Africa-Middle East	Girls	1992	Average	2.18
Central Asia and North Africa-Middle East	Girls	1993	Average	2.24
Central Asia and North Africa-Middle East	Girls	1994	Average	2.30
Central Asia and North Africa-Middle East	Girls	1995	Average	2.36
Central Asia and North Africa-Middle East	Girls	1996	Average	2.41
Central Asia and North Africa-Middle East	Girls	1997	Average	2.47
Central Asia and North Africa-Middle East	Girls	1998	Average	2.53
Central Asia and North Africa-Middle East	Girls	1999	Average	2.59
Central Asia and North Africa-Middle East	Girls	2000	Average	2.66
Central Asia and North Africa-Middle East	Girls	2001	Average	2.72
Central Asia and North Africa-Middle East	Girls	2002	Average	2.78
Central Asia and North Africa-Middle East	Girls	2003	Average	2.85
Central Asia and North Africa-Middle East	Girls	2004	Average	2.91
Central Asia and North Africa-Middle East	Girls	2005	Average	2.98
Central Asia and North Africa-Middle East	Girls	2006	Average	3.04
Central Asia and North Africa-Middle East	Girls	2007	Average	3.11
Central Asia and North Africa-Middle East	Girls	2008	Average	3.17
Central Asia and North Africa-Middle East	Girls	2009	Average	3.24
Central Asia and North Africa-Middle East	Girls	2010	Average	3.30
Central Asia and North Africa-Middle East	Girls	2011	Average	3.37
Central Asia and North Africa-Middle East	Girls	2012	Average	3.45
Central Asia and North Africa-Middle East	Girls	2013	Average	3.52
Central Asia and North Africa-Middle East	Girls	2014	Average	3.60
Central Asia and North Africa-Middle East	Girls	2015	Average	3.67
Central Asia and North Africa-Middle East	Girls	2016	Average	3.75
Central Asia and North Africa-Middle East	Girls	2017	Average	3.83
Central Asia and North Africa-Middle East	Girls	2018	Average	3.91
Central Asia and North Africa-Middle East	Girls	2019	Average	3.99
Central Asia and North Africa-Middle East	Girls	2020	Average	4.07
Central Asia and North Africa-Middle East	Girls	2021	Average	4.14
Central Asia and North Africa-Middle East	Girls	2022	Average	4.22
Central Asia and North Africa-Middle East	Girls	2023	Average	4.30
Central Asia and North Africa-Middle East	Girls	2024	Average	4.38
Central Asia and North Africa-Middle East	Girls	2025	Average	4.45
Central Asia and North Africa-Middle East	Girls	2026	Average	4.53
Central Asia and North Africa-Middle East	Girls	2027	Average	4.61
Central Asia and North Africa-Middle East	Girls	2028	Average	4.69
Central Asia and North Africa-Middle East	Girls	2029	Average	4.77
Central Asia and North Africa-Middle East	Girls	2030	Average	4.84
East and South East Asia	Boys	1975	12	0.66
East and South East Asia	Boys	1976	12	0.67
East and South East Asia	Boys	1977	12	0.69

East and South East Asia	Boys	1978	12	0.70
East and South East Asia	Boys	1979	12	0.71
East and South East Asia	Boys	1980	12	0.72
East and South East Asia	Boys	1981	12	0.73
East and South East Asia	Boys	1982	12	0.74
East and South East Asia	Boys	1983	12	0.75
East and South East Asia	Boys	1984	12	0.75
East and South East Asia	Boys	1985	12	0.76
East and South East Asia	Boys	1986	12	0.77
East and South East Asia	Boys	1987	12	0.78
East and South East Asia	Boys	1988	12	0.79
East and South East Asia	Boys	1989	12	0.80
East and South East Asia	Boys	1990	12	0.81
East and South East Asia	Boys	1991	12	0.83
East and South East Asia	Boys	1992	12	0.84
East and South East Asia	Boys	1993	12	0.86
East and South East Asia	Boys	1994	12	0.89
East and South East Asia	Boys	1995	12	0.91
East and South East Asia	Boys	1996	12	0.93
East and South East Asia	Boys	1997	12	0.96
East and South East Asia	Boys	1998	12	0.99
East and South East Asia	Boys	1999	12	1.01
East and South East Asia	Boys	2000	12	1.03
East and South East Asia	Boys	2001	12	1.05
East and South East Asia	Boys	2002	12	1.06
East and South East Asia	Boys	2003	12	1.07
East and South East Asia	Boys	2004	12	1.08
East and South East Asia	Boys	2005	12	1.09
East and South East Asia	Boys	2006	12	1.11
East and South East Asia	Boys	2007	12	1.13
East and South East Asia	Boys	2008	12	1.15
East and South East Asia	Boys	2009	12	1.17
East and South East Asia	Boys	2010	12	1.19
East and South East Asia	Boys	2011	12	1.21
East and South East Asia	Boys	2012	12	1.24
East and South East Asia	Boys	2013	12	1.27
East and South East Asia	Boys	2014	12	1.30
East and South East Asia	Boys	2015	12	1.33
East and South East Asia	Boys	2016	12	1.37
East and South East Asia	Boys	1975	13	0.89
East and South East Asia	Boys	1976	13	0.90
East and South East Asia	Boys	1977	13	0.92
East and South East Asia	Boys	1978	13	0.93
East and South East Asia	Boys	1979	13	0.94
East and South East Asia	Boys	1980	13	0.96
East and South East Asia	Boys	1981	13	0.97
East and South East Asia	Boys	1982	13	0.99
East and South East Asia	Boys	1983	13	1.00
East and South East Asia	Boys	1984	13	1.01
East and South East Asia	Boys	1985	13	1.02
East and South East Asia	Boys	1986	13	1.03
East and South East Asia	Boys	1987	13	1.04

East and South East Asia	Boys	1988	13	1.05
East and South East Asia	Boys	1989	13	1.07
East and South East Asia	Boys	1990	13	1.09
East and South East Asia	Boys	1991	13	1.11
East and South East Asia	Boys	1992	13	1.14
East and South East Asia	Boys	1993	13	1.16
East and South East Asia	Boys	1994	13	1.19
East and South East Asia	Boys	1995	13	1.23
East and South East Asia	Boys	1996	13	1.26
East and South East Asia	Boys	1997	13	1.30
East and South East Asia	Boys	1998	13	1.33
East and South East Asia	Boys	1999	13	1.36
East and South East Asia	Boys	2000	13	1.39
East and South East Asia	Boys	2001	13	1.41
East and South East Asia	Boys	2002	13	1.43
East and South East Asia	Boys	2003	13	1.45
East and South East Asia	Boys	2004	13	1.46
East and South East Asia	Boys	2005	13	1.48
East and South East Asia	Boys	2006	13	1.50
East and South East Asia	Boys	2007	13	1.53
East and South East Asia	Boys	2008	13	1.55
East and South East Asia	Boys	2009	13	1.58
East and South East Asia	Boys	2010	13	1.61
East and South East Asia	Boys	2011	13	1.65
East and South East Asia	Boys	2012	13	1.69
East and South East Asia	Boys	2013	13	1.73
East and South East Asia	Boys	2014	13	1.77
East and South East Asia	Boys	2015	13	1.82
East and South East Asia	Boys	2016	13	1.87
East and South East Asia	Boys	1975	14	1.17
East and South East Asia	Boys	1976	14	1.19
East and South East Asia	Boys	1977	14	1.21
East and South East Asia	Boys	1978	14	1.23
East and South East Asia	Boys	1979	14	1.25
East and South East Asia	Boys	1980	14	1.26
East and South East Asia	Boys	1981	14	1.28
East and South East Asia	Boys	1982	14	1.30
East and South East Asia	Boys	1983	14	1.31
East and South East Asia	Boys	1984	14	1.33
East and South East Asia	Boys	1985	14	1.34
East and South East Asia	Boys	1986	14	1.36
East and South East Asia	Boys	1987	14	1.37
East and South East Asia	Boys	1988	14	1.39
East and South East Asia	Boys	1989	14	1.41
East and South East Asia	Boys	1990	14	1.43
East and South East Asia	Boys	1991	14	1.46
East and South East Asia	Boys	1992	14	1.49
East and South East Asia	Boys	1993	14	1.53
East and South East Asia	Boys	1994	14	1.57
East and South East Asia	Boys	1995	14	1.61
East and South East Asia	Boys	1996	14	1.66
East and South East Asia	Boys	1997	14	1.70

East and South East Asia	Boys	1998	14	1.75
East and South East Asia	Boys	1999	14	1.79
East and South East Asia	Boys	2000	14	1.83
East and South East Asia	Boys	2001	14	1.86
East and South East Asia	Boys	2002	14	1.88
East and South East Asia	Boys	2003	14	1.90
East and South East Asia	Boys	2004	14	1.92
East and South East Asia	Boys	2005	14	1.95
East and South East Asia	Boys	2006	14	1.97
East and South East Asia	Boys	2007	14	2.01
East and South East Asia	Boys	2008	14	2.04
East and South East Asia	Boys	2009	14	2.08
East and South East Asia	Boys	2010	14	2.12
East and South East Asia	Boys	2011	14	2.17
East and South East Asia	Boys	2012	14	2.22
East and South East Asia	Boys	2013	14	2.28
East and South East Asia	Boys	2014	14	2.34
East and South East Asia	Boys	2015	14	2.40
East and South East Asia	Boys	2016	14	2.48
East and South East Asia	Boys	1975	15	1.48
East and South East Asia	Boys	1976	15	1.50
East and South East Asia	Boys	1977	15	1.52
East and South East Asia	Boys	1978	15	1.55
East and South East Asia	Boys	1979	15	1.57
East and South East Asia	Boys	1980	15	1.60
East and South East Asia	Boys	1981	15	1.63
East and South East Asia	Boys	1982	15	1.66
East and South East Asia	Boys	1983	15	1.68
East and South East Asia	Boys	1984	15	1.71
East and South East Asia	Boys	1985	15	1.73
East and South East Asia	Boys	1986	15	1.77
East and South East Asia	Boys	1987	15	1.80
East and South East Asia	Boys	1988	15	1.84
East and South East Asia	Boys	1989	15	1.88
East and South East Asia	Boys	1990	15	1.91
East and South East Asia	Boys	1991	15	1.95
East and South East Asia	Boys	1992	15	1.97
East and South East Asia	Boys	1993	15	1.99
East and South East Asia	Boys	1994	15	2.01
East and South East Asia	Boys	1995	15	2.03
East and South East Asia	Boys	1996	15	2.06
East and South East Asia	Boys	1997	15	2.09
East and South East Asia	Boys	1998	15	2.13
East and South East Asia	Boys	1999	15	2.18
East and South East Asia	Boys	2000	15	2.23
East and South East Asia	Boys	2001	15	2.29
East and South East Asia	Boys	2002	15	2.36
East and South East Asia	Boys	2003	15	2.43
East and South East Asia	Boys	2004	15	2.49
East and South East Asia	Boys	2005	15	2.55
East and South East Asia	Boys	2006	15	2.60
East and South East Asia	Boys	2007	15	2.65

East and South East Asia	Boys	2008	15	2.69
East and South East Asia	Boys	2009	15	2.73
East and South East Asia	Boys	2010	15	2.78
East and South East Asia	Boys	2011	15	2.83
East and South East Asia	Boys	2012	15	2.88
East and South East Asia	Boys	2013	15	2.94
East and South East Asia	Boys	2014	15	3.01
East and South East Asia	Boys	2015	15	3.07
East and South East Asia	Boys	2016	15	3.15
East and South East Asia	Boys	1975	16	1.84
East and South East Asia	Boys	1976	16	1.86
East and South East Asia	Boys	1977	16	1.89
East and South East Asia	Boys	1978	16	1.92
East and South East Asia	Boys	1979	16	1.95
East and South East Asia	Boys	1980	16	1.98
East and South East Asia	Boys	1981	16	2.02
East and South East Asia	Boys	1982	16	2.05
East and South East Asia	Boys	1983	16	2.08
East and South East Asia	Boys	1984	16	2.11
East and South East Asia	Boys	1985	16	2.14
East and South East Asia	Boys	1986	16	2.17
East and South East Asia	Boys	1987	16	2.22
East and South East Asia	Boys	1988	16	2.26
East and South East Asia	Boys	1989	16	2.31
East and South East Asia	Boys	1990	16	2.35
East and South East Asia	Boys	1991	16	2.39
East and South East Asia	Boys	1992	16	2.42
East and South East Asia	Boys	1993	16	2.44
East and South East Asia	Boys	1994	16	2.46
East and South East Asia	Boys	1995	16	2.49
East and South East Asia	Boys	1996	16	2.52
East and South East Asia	Boys	1997	16	2.56
East and South East Asia	Boys	1998	16	2.61
East and South East Asia	Boys	1999	16	2.66
East and South East Asia	Boys	2000	16	2.72
East and South East Asia	Boys	2001	16	2.79
East and South East Asia	Boys	2002	16	2.87
East and South East Asia	Boys	2003	16	2.95
East and South East Asia	Boys	2004	16	3.03
East and South East Asia	Boys	2005	16	3.10
East and South East Asia	Boys	2006	16	3.16
East and South East Asia	Boys	2007	16	3.21
East and South East Asia	Boys	2008	16	3.26
East and South East Asia	Boys	2009	16	3.31
East and South East Asia	Boys	2010	16	3.37
East and South East Asia	Boys	2011	16	3.43
East and South East Asia	Boys	2012	16	3.49
East and South East Asia	Boys	2013	16	3.56
East and South East Asia	Boys	2014	16	3.64
East and South East Asia	Boys	2015	16	3.72
East and South East Asia	Boys	2016	16	3.81
East and South East Asia	Boys	1975	17	2.24

East and South East Asia	Boys	1976	17	2.26
East and South East Asia	Boys	1977	17	2.29
East and South East Asia	Boys	1978	17	2.32
East and South East Asia	Boys	1979	17	2.36
East and South East Asia	Boys	1980	17	2.40
East and South East Asia	Boys	1981	17	2.43
East and South East Asia	Boys	1982	17	2.47
East and South East Asia	Boys	1983	17	2.51
East and South East Asia	Boys	1984	17	2.54
East and South East Asia	Boys	1985	17	2.58
East and South East Asia	Boys	1986	17	2.62
East and South East Asia	Boys	1987	17	2.66
East and South East Asia	Boys	1988	17	2.71
East and South East Asia	Boys	1989	17	2.77
East and South East Asia	Boys	1990	17	2.82
East and South East Asia	Boys	1991	17	2.86
East and South East Asia	Boys	1992	17	2.90
East and South East Asia	Boys	1993	17	2.92
East and South East Asia	Boys	1994	17	2.95
East and South East Asia	Boys	1995	17	2.97
East and South East Asia	Boys	1996	17	3.01
East and South East Asia	Boys	1997	17	3.06
East and South East Asia	Boys	1998	17	3.11
East and South East Asia	Boys	1999	17	3.17
East and South East Asia	Boys	2000	17	3.25
East and South East Asia	Boys	2001	17	3.33
East and South East Asia	Boys	2002	17	3.42
East and South East Asia	Boys	2003	17	3.51
East and South East Asia	Boys	2004	17	3.60
East and South East Asia	Boys	2005	17	3.68
East and South East Asia	Boys	2006	17	3.75
East and South East Asia	Boys	2007	17	3.81
East and South East Asia	Boys	2008	17	3.87
East and South East Asia	Boys	2009	17	3.93
East and South East Asia	Boys	2010	17	3.99
East and South East Asia	Boys	2011	17	4.06
East and South East Asia	Boys	2012	17	4.13
East and South East Asia	Boys	2013	17	4.21
East and South East Asia	Boys	2014	17	4.30
East and South East Asia	Boys	2015	17	4.40
East and South East Asia	Boys	2016	17	4.50
East and South East Asia	Boys	1975	18	2.67
East and South East Asia	Boys	1976	18	2.69
East and South East Asia	Boys	1977	18	2.73
East and South East Asia	Boys	1978	18	2.77
East and South East Asia	Boys	1979	18	2.81
East and South East Asia	Boys	1980	18	2.86
East and South East Asia	Boys	1981	18	2.90
East and South East Asia	Boys	1982	18	2.94
East and South East Asia	Boys	1983	18	2.98
East and South East Asia	Boys	1984	18	3.02
East and South East Asia	Boys	1985	18	3.06

East and South East Asia	Boys	1986	18	3.11
East and South East Asia	Boys	1987	18	3.16
East and South East Asia	Boys	1988	18	3.22
East and South East Asia	Boys	1989	18	3.28
East and South East Asia	Boys	1990	18	3.34
East and South East Asia	Boys	1991	18	3.39
East and South East Asia	Boys	1992	18	3.43
East and South East Asia	Boys	1993	18	3.46
East and South East Asia	Boys	1994	18	3.49
East and South East Asia	Boys	1995	18	3.52
East and South East Asia	Boys	1996	18	3.56
East and South East Asia	Boys	1997	18	3.61
East and South East Asia	Boys	1998	18	3.68
East and South East Asia	Boys	1999	18	3.75
East and South East Asia	Boys	2000	18	3.83
East and South East Asia	Boys	2001	18	3.93
East and South East Asia	Boys	2002	18	4.03
East and South East Asia	Boys	2003	18	4.14
East and South East Asia	Boys	2004	18	4.24
East and South East Asia	Boys	2005	18	4.33
East and South East Asia	Boys	2006	18	4.41
East and South East Asia	Boys	2007	18	4.48
East and South East Asia	Boys	2008	18	4.55
East and South East Asia	Boys	2009	18	4.61
East and South East Asia	Boys	2010	18	4.68
East and South East Asia	Boys	2011	18	4.76
East and South East Asia	Boys	2012	18	4.85
East and South East Asia	Boys	2013	18	4.94
East and South East Asia	Boys	2014	18	5.04
East and South East Asia	Boys	2015	18	5.15
East and South East Asia	Boys	2016	18	5.27
East and South East Asia	Boys	1975	Average	1.57
East and South East Asia	Boys	1976	Average	1.58
East and South East Asia	Boys	1977	Average	1.61
East and South East Asia	Boys	1978	Average	1.63
East and South East Asia	Boys	1979	Average	1.66
East and South East Asia	Boys	1980	Average	1.68
East and South East Asia	Boys	1981	Average	1.71
East and South East Asia	Boys	1982	Average	1.73
East and South East Asia	Boys	1983	Average	1.76
East and South East Asia	Boys	1984	Average	1.78
East and South East Asia	Boys	1985	Average	1.80
East and South East Asia	Boys	1986	Average	1.83
East and South East Asia	Boys	1987	Average	1.86
East and South East Asia	Boys	1988	Average	1.89
East and South East Asia	Boys	1989	Average	1.93
East and South East Asia	Boys	1990	Average	1.97
East and South East Asia	Boys	1991	Average	2.00
East and South East Asia	Boys	1992	Average	2.03
East and South East Asia	Boys	1993	Average	2.05
East and South East Asia	Boys	1994	Average	2.08
East and South East Asia	Boys	1995	Average	2.11

East and South East Asia	Boys	1996	Average	2.14
East and South East Asia	Boys	1997	Average	2.18
East and South East Asia	Boys	1998	Average	2.23
East and South East Asia	Boys	1999	Average	2.28
East and South East Asia	Boys	2000	Average	2.33
East and South East Asia	Boys	2001	Average	2.38
East and South East Asia	Boys	2002	Average	2.44
East and South East Asia	Boys	2003	Average	2.49
East and South East Asia	Boys	2004	Average	2.55
East and South East Asia	Boys	2005	Average	2.60
East and South East Asia	Boys	2006	Average	2.64
East and South East Asia	Boys	2007	Average	2.69
East and South East Asia	Boys	2008	Average	2.73
East and South East Asia	Boys	2009	Average	2.77
East and South East Asia	Boys	2010	Average	2.82
East and South East Asia	Boys	2011	Average	2.87
East and South East Asia	Boys	2012	Average	2.93
East and South East Asia	Boys	2013	Average	2.99
East and South East Asia	Boys	2014	Average	3.06
East and South East Asia	Boys	2015	Average	3.13
East and South East Asia	Boys	2016	Average	3.21
East and South East Asia	Boys	2017	Average	3.29
East and South East Asia	Boys	2018	Average	3.38
East and South East Asia	Boys	2019	Average	3.48
East and South East Asia	Boys	2020	Average	3.57
East and South East Asia	Boys	2021	Average	3.66
East and South East Asia	Boys	2022	Average	3.75
East and South East Asia	Boys	2023	Average	3.84
East and South East Asia	Boys	2024	Average	3.92
East and South East Asia	Boys	2025	Average	4.00
East and South East Asia	Boys	2026	Average	4.08
East and South East Asia	Boys	2027	Average	4.16
East and South East Asia	Boys	2028	Average	4.24
East and South East Asia	Boys	2029	Average	4.32
East and South East Asia	Boys	2030	Average	4.40
East and South East Asia	Girls	1975	12	0.64
East and South East Asia	Girls	1976	12	0.65
East and South East Asia	Girls	1977	12	0.66
East and South East Asia	Girls	1978	12	0.66
East and South East Asia	Girls	1979	12	0.67
East and South East Asia	Girls	1980	12	0.68
East and South East Asia	Girls	1981	12	0.69
East and South East Asia	Girls	1982	12	0.69
East and South East Asia	Girls	1983	12	0.70
East and South East Asia	Girls	1984	12	0.70
East and South East Asia	Girls	1985	12	0.71
East and South East Asia	Girls	1986	12	0.71
East and South East Asia	Girls	1987	12	0.72
East and South East Asia	Girls	1988	12	0.72
East and South East Asia	Girls	1989	12	0.73
East and South East Asia	Girls	1990	12	0.74
East and South East Asia	Girls	1991	12	0.75

East and South East Asia	Girls	1992	12	0.76
East and South East Asia	Girls	1993	12	0.78
East and South East Asia	Girls	1994	12	0.79
East and South East Asia	Girls	1995	12	0.81
East and South East Asia	Girls	1996	12	0.82
East and South East Asia	Girls	1997	12	0.84
East and South East Asia	Girls	1998	12	0.86
East and South East Asia	Girls	1999	12	0.87
East and South East Asia	Girls	2000	12	0.88
East and South East Asia	Girls	2001	12	0.89
East and South East Asia	Girls	2002	12	0.90
East and South East Asia	Girls	2003	12	0.91
East and South East Asia	Girls	2004	12	0.91
East and South East Asia	Girls	2005	12	0.92
East and South East Asia	Girls	2006	12	0.93
East and South East Asia	Girls	2007	12	0.94
East and South East Asia	Girls	2008	12	0.96
East and South East Asia	Girls	2009	12	0.97
East and South East Asia	Girls	2010	12	0.99
East and South East Asia	Girls	2011	12	1.01
East and South East Asia	Girls	2012	12	1.03
East and South East Asia	Girls	2013	12	1.05
East and South East Asia	Girls	2014	12	1.07
East and South East Asia	Girls	2015	12	1.09
East and South East Asia	Girls	2016	12	1.12
East and South East Asia	Girls	1975	13	0.91
East and South East Asia	Girls	1976	13	0.92
East and South East Asia	Girls	1977	13	0.93
East and South East Asia	Girls	1978	13	0.94
East and South East Asia	Girls	1979	13	0.95
East and South East Asia	Girls	1980	13	0.96
East and South East Asia	Girls	1981	13	0.97
East and South East Asia	Girls	1982	13	0.98
East and South East Asia	Girls	1983	13	0.98
East and South East Asia	Girls	1984	13	0.99
East and South East Asia	Girls	1985	13	1.00
East and South East Asia	Girls	1986	13	1.00
East and South East Asia	Girls	1987	13	1.01
East and South East Asia	Girls	1988	13	1.02
East and South East Asia	Girls	1989	13	1.03
East and South East Asia	Girls	1990	13	1.04
East and South East Asia	Girls	1991	13	1.06
East and South East Asia	Girls	1992	13	1.08
East and South East Asia	Girls	1993	13	1.09
East and South East Asia	Girls	1994	13	1.11
East and South East Asia	Girls	1995	13	1.14
East and South East Asia	Girls	1996	13	1.16
East and South East Asia	Girls	1997	13	1.18
East and South East Asia	Girls	1998	13	1.20
East and South East Asia	Girls	1999	13	1.22
East and South East Asia	Girls	2000	13	1.24
East and South East Asia	Girls	2001	13	1.25

East and South East Asia	Girls	2002	13	1.26
East and South East Asia	Girls	2003	13	1.27
East and South East Asia	Girls	2004	13	1.28
East and South East Asia	Girls	2005	13	1.29
East and South East Asia	Girls	2006	13	1.30
East and South East Asia	Girls	2007	13	1.32
East and South East Asia	Girls	2008	13	1.34
East and South East Asia	Girls	2009	13	1.36
East and South East Asia	Girls	2010	13	1.38
East and South East Asia	Girls	2011	13	1.40
East and South East Asia	Girls	2012	13	1.43
East and South East Asia	Girls	2013	13	1.46
East and South East Asia	Girls	2014	13	1.49
East and South East Asia	Girls	2015	13	1.53
East and South East Asia	Girls	2016	13	1.56
East and South East Asia	Girls	1975	14	1.21
East and South East Asia	Girls	1976	14	1.23
East and South East Asia	Girls	1977	14	1.24
East and South East Asia	Girls	1978	14	1.25
East and South East Asia	Girls	1979	14	1.27
East and South East Asia	Girls	1980	14	1.28
East and South East Asia	Girls	1981	14	1.29
East and South East Asia	Girls	1982	14	1.30
East and South East Asia	Girls	1983	14	1.31
East and South East Asia	Girls	1984	14	1.32
East and South East Asia	Girls	1985	14	1.33
East and South East Asia	Girls	1986	14	1.34
East and South East Asia	Girls	1987	14	1.35
East and South East Asia	Girls	1988	14	1.36
East and South East Asia	Girls	1989	14	1.37
East and South East Asia	Girls	1990	14	1.39
East and South East Asia	Girls	1991	14	1.41
East and South East Asia	Girls	1992	14	1.43
East and South East Asia	Girls	1993	14	1.45
East and South East Asia	Girls	1994	14	1.48
East and South East Asia	Girls	1995	14	1.50
East and South East Asia	Girls	1996	14	1.53
East and South East Asia	Girls	1997	14	1.56
East and South East Asia	Girls	1998	14	1.59
East and South East Asia	Girls	1999	14	1.61
East and South East Asia	Girls	2000	14	1.63
East and South East Asia	Girls	2001	14	1.65
East and South East Asia	Girls	2002	14	1.66
East and South East Asia	Girls	2003	14	1.67
East and South East Asia	Girls	2004	14	1.68
East and South East Asia	Girls	2005	14	1.70
East and South East Asia	Girls	2006	14	1.71
East and South East Asia	Girls	2007	14	1.73
East and South East Asia	Girls	2008	14	1.76
East and South East Asia	Girls	2009	14	1.78
East and South East Asia	Girls	2010	14	1.81
East and South East Asia	Girls	2011	14	1.84

East and South East Asia	Girls	2012	14	1.87
East and South East Asia	Girls	2013	14	1.91
East and South East Asia	Girls	2014	14	1.95
East and South East Asia	Girls	2015	14	1.99
East and South East Asia	Girls	2016	14	2.04
East and South East Asia	Girls	1975	15	1.50
East and South East Asia	Girls	1976	15	1.51
East and South East Asia	Girls	1977	15	1.52
East and South East Asia	Girls	1978	15	1.54
East and South East Asia	Girls	1979	15	1.56
East and South East Asia	Girls	1980	15	1.58
East and South East Asia	Girls	1981	15	1.60
East and South East Asia	Girls	1982	15	1.62
East and South East Asia	Girls	1983	15	1.64
East and South East Asia	Girls	1984	15	1.65
East and South East Asia	Girls	1985	15	1.67
East and South East Asia	Girls	1986	15	1.69
East and South East Asia	Girls	1987	15	1.71
East and South East Asia	Girls	1988	15	1.74
East and South East Asia	Girls	1989	15	1.76
East and South East Asia	Girls	1990	15	1.79
East and South East Asia	Girls	1991	15	1.81
East and South East Asia	Girls	1992	15	1.82
East and South East Asia	Girls	1993	15	1.83
East and South East Asia	Girls	1994	15	1.84
East and South East Asia	Girls	1995	15	1.85
East and South East Asia	Girls	1996	15	1.86
East and South East Asia	Girls	1997	15	1.88
East and South East Asia	Girls	1998	15	1.90
East and South East Asia	Girls	1999	15	1.93
East and South East Asia	Girls	2000	15	1.96
East and South East Asia	Girls	2001	15	1.99
East and South East Asia	Girls	2002	15	2.03
East and South East Asia	Girls	2003	15	2.07
East and South East Asia	Girls	2004	15	2.10
East and South East Asia	Girls	2005	15	2.13
East and South East Asia	Girls	2006	15	2.16
East and South East Asia	Girls	2007	15	2.18
East and South East Asia	Girls	2008	15	2.21
East and South East Asia	Girls	2009	15	2.23
East and South East Asia	Girls	2010	15	2.26
East and South East Asia	Girls	2011	15	2.29
East and South East Asia	Girls	2012	15	2.32
East and South East Asia	Girls	2013	15	2.36
East and South East Asia	Girls	2014	15	2.40
East and South East Asia	Girls	2015	15	2.44
East and South East Asia	Girls	2016	15	2.49
East and South East Asia	Girls	1975	16	1.75
East and South East Asia	Girls	1976	16	1.76
East and South East Asia	Girls	1977	16	1.78
East and South East Asia	Girls	1978	16	1.80
East and South East Asia	Girls	1979	16	1.83

East and South East Asia	Girls	1980	16	1.85
East and South East Asia	Girls	1981	16	1.87
East and South East Asia	Girls	1982	16	1.89
East and South East Asia	Girls	1983	16	1.91
East and South East Asia	Girls	1984	16	1.93
East and South East Asia	Girls	1985	16	1.94
East and South East Asia	Girls	1986	16	1.97
East and South East Asia	Girls	1987	16	1.99
East and South East Asia	Girls	1988	16	2.02
East and South East Asia	Girls	1989	16	2.05
East and South East Asia	Girls	1990	16	2.08
East and South East Asia	Girls	1991	16	2.10
East and South East Asia	Girls	1992	16	2.11
East and South East Asia	Girls	1993	16	2.12
East and South East Asia	Girls	1994	16	2.13
East and South East Asia	Girls	1995	16	2.14
East and South East Asia	Girls	1996	16	2.16
East and South East Asia	Girls	1997	16	2.18
East and South East Asia	Girls	1998	16	2.21
East and South East Asia	Girls	1999	16	2.24
East and South East Asia	Girls	2000	16	2.27
East and South East Asia	Girls	2001	16	2.31
East and South East Asia	Girls	2002	16	2.35
East and South East Asia	Girls	2003	16	2.39
East and South East Asia	Girls	2004	16	2.43
East and South East Asia	Girls	2005	16	2.46
East and South East Asia	Girls	2006	16	2.49
East and South East Asia	Girls	2007	16	2.52
East and South East Asia	Girls	2008	16	2.54
East and South East Asia	Girls	2009	16	2.57
East and South East Asia	Girls	2010	16	2.60
East and South East Asia	Girls	2011	16	2.63
East and South East Asia	Girls	2012	16	2.67
East and South East Asia	Girls	2013	16	2.71
East and South East Asia	Girls	2014	16	2.75
East and South East Asia	Girls	2015	16	2.80
East and South East Asia	Girls	2016	16	2.85
East and South East Asia	Girls	1975	17	1.96
East and South East Asia	Girls	1976	17	1.97
East and South East Asia	Girls	1977	17	1.99
East and South East Asia	Girls	1978	17	2.01
East and South East Asia	Girls	1979	17	2.04
East and South East Asia	Girls	1980	17	2.06
East and South East Asia	Girls	1981	17	2.09
East and South East Asia	Girls	1982	17	2.11
East and South East Asia	Girls	1983	17	2.13
East and South East Asia	Girls	1984	17	2.15
East and South East Asia	Girls	1985	17	2.17
East and South East Asia	Girls	1986	17	2.19
East and South East Asia	Girls	1987	17	2.22
East and South East Asia	Girls	1988	17	2.25
East and South East Asia	Girls	1989	17	2.28

East and South East Asia	Girls	1990	17	2.31
East and South East Asia	Girls	1991	17	2.34
East and South East Asia	Girls	1992	17	2.35
East and South East Asia	Girls	1993	17	2.37
East and South East Asia	Girls	1994	17	2.38
East and South East Asia	Girls	1995	17	2.39
East and South East Asia	Girls	1996	17	2.41
East and South East Asia	Girls	1997	17	2.43
East and South East Asia	Girls	1998	17	2.46
East and South East Asia	Girls	1999	17	2.49
East and South East Asia	Girls	2000	17	2.53
East and South East Asia	Girls	2001	17	2.57
East and South East Asia	Girls	2002	17	2.61
East and South East Asia	Girls	2003	17	2.66
East and South East Asia	Girls	2004	17	2.70
East and South East Asia	Girls	2005	17	2.74
East and South East Asia	Girls	2006	17	2.77
East and South East Asia	Girls	2007	17	2.80
East and South East Asia	Girls	2008	17	2.83
East and South East Asia	Girls	2009	17	2.86
East and South East Asia	Girls	2010	17	2.89
East and South East Asia	Girls	2011	17	2.93
East and South East Asia	Girls	2012	17	2.97
East and South East Asia	Girls	2013	17	3.01
East and South East Asia	Girls	2014	17	3.06
East and South East Asia	Girls	2015	17	3.11
East and South East Asia	Girls	2016	17	3.17
East and South East Asia	Girls	1975	18	2.15
East and South East Asia	Girls	1976	18	2.16
East and South East Asia	Girls	1977	18	2.18
East and South East Asia	Girls	1978	18	2.21
East and South East Asia	Girls	1979	18	2.24
East and South East Asia	Girls	1980	18	2.27
East and South East Asia	Girls	1981	18	2.29
East and South East Asia	Girls	1982	18	2.32
East and South East Asia	Girls	1983	18	2.34
East and South East Asia	Girls	1984	18	2.37
East and South East Asia	Girls	1985	18	2.39
East and South East Asia	Girls	1986	18	2.42
East and South East Asia	Girls	1987	18	2.45
East and South East Asia	Girls	1988	18	2.48
East and South East Asia	Girls	1989	18	2.52
East and South East Asia	Girls	1990	18	2.55
East and South East Asia	Girls	1991	18	2.58
East and South East Asia	Girls	1992	18	2.60
East and South East Asia	Girls	1993	18	2.61
East and South East Asia	Girls	1994	18	2.62
East and South East Asia	Girls	1995	18	2.64
East and South East Asia	Girls	1996	18	2.66
East and South East Asia	Girls	1997	18	2.69
East and South East Asia	Girls	1998	18	2.72
East and South East Asia	Girls	1999	18	2.75

East and South East Asia	Girls	2000	18	2.80
East and South East Asia	Girls	2001	18	2.84
East and South East Asia	Girls	2002	18	2.89
East and South East Asia	Girls	2003	18	2.95
East and South East Asia	Girls	2004	18	2.99
East and South East Asia	Girls	2005	18	3.04
East and South East Asia	Girls	2006	18	3.07
East and South East Asia	Girls	2007	18	3.11
East and South East Asia	Girls	2008	18	3.14
East and South East Asia	Girls	2009	18	3.17
East and South East Asia	Girls	2010	18	3.21
East and South East Asia	Girls	2011	18	3.25
East and South East Asia	Girls	2012	18	3.30
East and South East Asia	Girls	2013	18	3.35
East and South East Asia	Girls	2014	18	3.40
East and South East Asia	Girls	2015	18	3.46
East and South East Asia	Girls	2016	18	3.53
East and South East Asia	Girls	1975	Average	1.44
East and South East Asia	Girls	1976	Average	1.46
East and South East Asia	Girls	1977	Average	1.47
East and South East Asia	Girls	1978	Average	1.49
East and South East Asia	Girls	1979	Average	1.51
East and South East Asia	Girls	1980	Average	1.52
East and South East Asia	Girls	1981	Average	1.54
East and South East Asia	Girls	1982	Average	1.56
East and South East Asia	Girls	1983	Average	1.57
East and South East Asia	Girls	1984	Average	1.59
East and South East Asia	Girls	1985	Average	1.60
East and South East Asia	Girls	1986	Average	1.62
East and South East Asia	Girls	1987	Average	1.63
East and South East Asia	Girls	1988	Average	1.66
East and South East Asia	Girls	1989	Average	1.68
East and South East Asia	Girls	1990	Average	1.70
East and South East Asia	Girls	1991	Average	1.72
East and South East Asia	Girls	1992	Average	1.74
East and South East Asia	Girls	1993	Average	1.75
East and South East Asia	Girls	1994	Average	1.77
East and South East Asia	Girls	1995	Average	1.78
East and South East Asia	Girls	1996	Average	1.80
East and South East Asia	Girls	1997	Average	1.82
East and South East Asia	Girls	1998	Average	1.85
East and South East Asia	Girls	1999	Average	1.87
East and South East Asia	Girls	2000	Average	1.90
East and South East Asia	Girls	2001	Average	1.93
East and South East Asia	Girls	2002	Average	1.96
East and South East Asia	Girls	2003	Average	1.99
East and South East Asia	Girls	2004	Average	2.01
East and South East Asia	Girls	2005	Average	2.04
East and South East Asia	Girls	2006	Average	2.06
East and South East Asia	Girls	2007	Average	2.09
East and South East Asia	Girls	2008	Average	2.11
East and South East Asia	Girls	2009	Average	2.13

East and South East Asia	Girls	2010	Average	2.16
East and South East Asia	Girls	2011	Average	2.19
East and South East Asia	Girls	2012	Average	2.23
East and South East Asia	Girls	2013	Average	2.26
East and South East Asia	Girls	2014	Average	2.30
East and South East Asia	Girls	2015	Average	2.35
East and South East Asia	Girls	2016	Average	2.39
East and South East Asia	Girls	2017	Average	2.44
East and South East Asia	Girls	2018	Average	2.50
East and South East Asia	Girls	2019	Average	2.55
East and South East Asia	Girls	2020	Average	2.61
East and South East Asia	Girls	2021	Average	2.66
East and South East Asia	Girls	2022	Average	2.72
East and South East Asia	Girls	2023	Average	2.77
East and South East Asia	Girls	2024	Average	2.82
East and South East Asia	Girls	2025	Average	2.88
East and South East Asia	Girls	2026	Average	2.93
East and South East Asia	Girls	2027	Average	2.98
East and South East Asia	Girls	2028	Average	3.03
East and South East Asia	Girls	2029	Average	3.08
East and South East Asia	Girls	2030	Average	3.13
High-income Asia Pacific	Boys	1975	12	0.67
High-income Asia Pacific	Boys	1976	12	0.69
High-income Asia Pacific	Boys	1977	12	0.71
High-income Asia Pacific	Boys	1978	12	0.73
High-income Asia Pacific	Boys	1979	12	0.74
High-income Asia Pacific	Boys	1980	12	0.76
High-income Asia Pacific	Boys	1981	12	0.78
High-income Asia Pacific	Boys	1982	12	0.80
High-income Asia Pacific	Boys	1983	12	0.82
High-income Asia Pacific	Boys	1984	12	0.84
High-income Asia Pacific	Boys	1985	12	0.86
High-income Asia Pacific	Boys	1986	12	0.87
High-income Asia Pacific	Boys	1987	12	0.89
High-income Asia Pacific	Boys	1988	12	0.91
High-income Asia Pacific	Boys	1989	12	0.93
High-income Asia Pacific	Boys	1990	12	0.95
High-income Asia Pacific	Boys	1991	12	0.97
High-income Asia Pacific	Boys	1992	12	0.99
High-income Asia Pacific	Boys	1993	12	1.00
High-income Asia Pacific	Boys	1994	12	1.02
High-income Asia Pacific	Boys	1995	12	1.03
High-income Asia Pacific	Boys	1996	12	1.05
High-income Asia Pacific	Boys	1997	12	1.06
High-income Asia Pacific	Boys	1998	12	1.07
High-income Asia Pacific	Boys	1999	12	1.07
High-income Asia Pacific	Boys	2000	12	1.08
High-income Asia Pacific	Boys	2001	12	1.09
High-income Asia Pacific	Boys	2002	12	1.11
High-income Asia Pacific	Boys	2003	12	1.12
High-income Asia Pacific	Boys	2004	12	1.14
High-income Asia Pacific	Boys	2005	12	1.15

High-income Asia Pacific	Boys	2006	12	1.16
High-income Asia Pacific	Boys	2007	12	1.17
High-income Asia Pacific	Boys	2008	12	1.18
High-income Asia Pacific	Boys	2009	12	1.19
High-income Asia Pacific	Boys	2010	12	1.20
High-income Asia Pacific	Boys	2011	12	1.20
High-income Asia Pacific	Boys	2012	12	1.20
High-income Asia Pacific	Boys	2013	12	1.20
High-income Asia Pacific	Boys	2014	12	1.20
High-income Asia Pacific	Boys	2015	12	1.20
High-income Asia Pacific	Boys	2016	12	1.20
High-income Asia Pacific	Boys	1975	13	0.90
High-income Asia Pacific	Boys	1976	13	0.92
High-income Asia Pacific	Boys	1977	13	0.94
High-income Asia Pacific	Boys	1978	13	0.96
High-income Asia Pacific	Boys	1979	13	0.99
High-income Asia Pacific	Boys	1980	13	1.01
High-income Asia Pacific	Boys	1981	13	1.04
High-income Asia Pacific	Boys	1982	13	1.06
High-income Asia Pacific	Boys	1983	13	1.09
High-income Asia Pacific	Boys	1984	13	1.11
High-income Asia Pacific	Boys	1985	13	1.14
High-income Asia Pacific	Boys	1986	13	1.16
High-income Asia Pacific	Boys	1987	13	1.19
High-income Asia Pacific	Boys	1988	13	1.22
High-income Asia Pacific	Boys	1989	13	1.24
High-income Asia Pacific	Boys	1990	13	1.27
High-income Asia Pacific	Boys	1991	13	1.29
High-income Asia Pacific	Boys	1992	13	1.32
High-income Asia Pacific	Boys	1993	13	1.34
High-income Asia Pacific	Boys	1994	13	1.36
High-income Asia Pacific	Boys	1995	13	1.38
High-income Asia Pacific	Boys	1996	13	1.40
High-income Asia Pacific	Boys	1997	13	1.41
High-income Asia Pacific	Boys	1998	13	1.43
High-income Asia Pacific	Boys	1999	13	1.44
High-income Asia Pacific	Boys	2000	13	1.45
High-income Asia Pacific	Boys	2001	13	1.46
High-income Asia Pacific	Boys	2002	13	1.48
High-income Asia Pacific	Boys	2003	13	1.50
High-income Asia Pacific	Boys	2004	13	1.53
High-income Asia Pacific	Boys	2005	13	1.55
High-income Asia Pacific	Boys	2006	13	1.56
High-income Asia Pacific	Boys	2007	13	1.58
High-income Asia Pacific	Boys	2008	13	1.59
High-income Asia Pacific	Boys	2009	13	1.60
High-income Asia Pacific	Boys	2010	13	1.61
High-income Asia Pacific	Boys	2011	13	1.62
High-income Asia Pacific	Boys	2012	13	1.62
High-income Asia Pacific	Boys	2013	13	1.62
High-income Asia Pacific	Boys	2014	13	1.62
High-income Asia Pacific	Boys	2015	13	1.62

High-income Asia Pacific	Boys	2016	13	1.62
High-income Asia Pacific	Boys	1975	14	1.18
High-income Asia Pacific	Boys	1976	14	1.20
High-income Asia Pacific	Boys	1977	14	1.23
High-income Asia Pacific	Boys	1978	14	1.26
High-income Asia Pacific	Boys	1979	14	1.29
High-income Asia Pacific	Boys	1980	14	1.32
High-income Asia Pacific	Boys	1981	14	1.36
High-income Asia Pacific	Boys	1982	14	1.39
High-income Asia Pacific	Boys	1983	14	1.42
High-income Asia Pacific	Boys	1984	14	1.45
High-income Asia Pacific	Boys	1985	14	1.48
High-income Asia Pacific	Boys	1986	14	1.52
High-income Asia Pacific	Boys	1987	14	1.55
High-income Asia Pacific	Boys	1988	14	1.58
High-income Asia Pacific	Boys	1989	14	1.62
High-income Asia Pacific	Boys	1990	14	1.65
High-income Asia Pacific	Boys	1991	14	1.68
High-income Asia Pacific	Boys	1992	14	1.71
High-income Asia Pacific	Boys	1993	14	1.74
High-income Asia Pacific	Boys	1994	14	1.77
High-income Asia Pacific	Boys	1995	14	1.80
High-income Asia Pacific	Boys	1996	14	1.82
High-income Asia Pacific	Boys	1997	14	1.84
High-income Asia Pacific	Boys	1998	14	1.86
High-income Asia Pacific	Boys	1999	14	1.87
High-income Asia Pacific	Boys	2000	14	1.89
High-income Asia Pacific	Boys	2001	14	1.91
High-income Asia Pacific	Boys	2002	14	1.93
High-income Asia Pacific	Boys	2003	14	1.96
High-income Asia Pacific	Boys	2004	14	1.99
High-income Asia Pacific	Boys	2005	14	2.02
High-income Asia Pacific	Boys	2006	14	2.04
High-income Asia Pacific	Boys	2007	14	2.06
High-income Asia Pacific	Boys	2008	14	2.08
High-income Asia Pacific	Boys	2009	14	2.09
High-income Asia Pacific	Boys	2010	14	2.10
High-income Asia Pacific	Boys	2011	14	2.11
High-income Asia Pacific	Boys	2012	14	2.11
High-income Asia Pacific	Boys	2013	14	2.12
High-income Asia Pacific	Boys	2014	14	2.12
High-income Asia Pacific	Boys	2015	14	2.12
High-income Asia Pacific	Boys	2016	14	2.12
High-income Asia Pacific	Boys	1975	15	1.51
High-income Asia Pacific	Boys	1976	15	1.54
High-income Asia Pacific	Boys	1977	15	1.57
High-income Asia Pacific	Boys	1978	15	1.61
High-income Asia Pacific	Boys	1979	15	1.65
High-income Asia Pacific	Boys	1980	15	1.68
High-income Asia Pacific	Boys	1981	15	1.72
High-income Asia Pacific	Boys	1982	15	1.76
High-income Asia Pacific	Boys	1983	15	1.79

High-income Asia Pacific	Boys	1984	15	1.83
High-income Asia Pacific	Boys	1985	15	1.87
High-income Asia Pacific	Boys	1986	15	1.91
High-income Asia Pacific	Boys	1987	15	1.95
High-income Asia Pacific	Boys	1988	15	1.99
High-income Asia Pacific	Boys	1989	15	2.03
High-income Asia Pacific	Boys	1990	15	2.07
High-income Asia Pacific	Boys	1991	15	2.11
High-income Asia Pacific	Boys	1992	15	2.15
High-income Asia Pacific	Boys	1993	15	2.18
High-income Asia Pacific	Boys	1994	15	2.21
High-income Asia Pacific	Boys	1995	15	2.24
High-income Asia Pacific	Boys	1996	15	2.27
High-income Asia Pacific	Boys	1997	15	2.30
High-income Asia Pacific	Boys	1998	15	2.32
High-income Asia Pacific	Boys	1999	15	2.35
High-income Asia Pacific	Boys	2000	15	2.37
High-income Asia Pacific	Boys	2001	15	2.39
High-income Asia Pacific	Boys	2002	15	2.41
High-income Asia Pacific	Boys	2003	15	2.43
High-income Asia Pacific	Boys	2004	15	2.46
High-income Asia Pacific	Boys	2005	15	2.48
High-income Asia Pacific	Boys	2006	15	2.51
High-income Asia Pacific	Boys	2007	15	2.54
High-income Asia Pacific	Boys	2008	15	2.58
High-income Asia Pacific	Boys	2009	15	2.61
High-income Asia Pacific	Boys	2010	15	2.64
High-income Asia Pacific	Boys	2011	15	2.66
High-income Asia Pacific	Boys	2012	15	2.68
High-income Asia Pacific	Boys	2013	15	2.70
High-income Asia Pacific	Boys	2014	15	2.71
High-income Asia Pacific	Boys	2015	15	2.72
High-income Asia Pacific	Boys	2016	15	2.72
High-income Asia Pacific	Boys	1975	16	1.87
High-income Asia Pacific	Boys	1976	16	1.90
High-income Asia Pacific	Boys	1977	16	1.94
High-income Asia Pacific	Boys	1978	16	1.98
High-income Asia Pacific	Boys	1979	16	2.03
High-income Asia Pacific	Boys	1980	16	2.07
High-income Asia Pacific	Boys	1981	16	2.11
High-income Asia Pacific	Boys	1982	16	2.15
High-income Asia Pacific	Boys	1983	16	2.20
High-income Asia Pacific	Boys	1984	16	2.24
High-income Asia Pacific	Boys	1985	16	2.28
High-income Asia Pacific	Boys	1986	16	2.33
High-income Asia Pacific	Boys	1987	16	2.38
High-income Asia Pacific	Boys	1988	16	2.42
High-income Asia Pacific	Boys	1989	16	2.47
High-income Asia Pacific	Boys	1990	16	2.51
High-income Asia Pacific	Boys	1991	16	2.56
High-income Asia Pacific	Boys	1992	16	2.60
High-income Asia Pacific	Boys	1993	16	2.64

High-income Asia Pacific	Boys	1994	16	2.67
High-income Asia Pacific	Boys	1995	16	2.71
High-income Asia Pacific	Boys	1996	16	2.74
High-income Asia Pacific	Boys	1997	16	2.78
High-income Asia Pacific	Boys	1998	16	2.81
High-income Asia Pacific	Boys	1999	16	2.83
High-income Asia Pacific	Boys	2000	16	2.86
High-income Asia Pacific	Boys	2001	16	2.88
High-income Asia Pacific	Boys	2002	16	2.91
High-income Asia Pacific	Boys	2003	16	2.93
High-income Asia Pacific	Boys	2004	16	2.96
High-income Asia Pacific	Boys	2005	16	2.99
High-income Asia Pacific	Boys	2006	16	3.03
High-income Asia Pacific	Boys	2007	16	3.06
High-income Asia Pacific	Boys	2008	16	3.10
High-income Asia Pacific	Boys	2009	16	3.14
High-income Asia Pacific	Boys	2010	16	3.17
High-income Asia Pacific	Boys	2011	16	3.20
High-income Asia Pacific	Boys	2012	16	3.22
High-income Asia Pacific	Boys	2013	16	3.24
High-income Asia Pacific	Boys	2014	16	3.26
High-income Asia Pacific	Boys	2015	16	3.27
High-income Asia Pacific	Boys	2016	16	3.28
High-income Asia Pacific	Boys	1975	17	2.25
High-income Asia Pacific	Boys	1976	17	2.29
High-income Asia Pacific	Boys	1977	17	2.34
High-income Asia Pacific	Boys	1978	17	2.38
High-income Asia Pacific	Boys	1979	17	2.43
High-income Asia Pacific	Boys	1980	17	2.48
High-income Asia Pacific	Boys	1981	17	2.52
High-income Asia Pacific	Boys	1982	17	2.57
High-income Asia Pacific	Boys	1983	17	2.62
High-income Asia Pacific	Boys	1984	17	2.67
High-income Asia Pacific	Boys	1985	17	2.72
High-income Asia Pacific	Boys	1986	17	2.77
High-income Asia Pacific	Boys	1987	17	2.82
High-income Asia Pacific	Boys	1988	17	2.87
High-income Asia Pacific	Boys	1989	17	2.92
High-income Asia Pacific	Boys	1990	17	2.97
High-income Asia Pacific	Boys	1991	17	3.02
High-income Asia Pacific	Boys	1992	17	3.07
High-income Asia Pacific	Boys	1993	17	3.11
High-income Asia Pacific	Boys	1994	17	3.15
High-income Asia Pacific	Boys	1995	17	3.19
High-income Asia Pacific	Boys	1996	17	3.23
High-income Asia Pacific	Boys	1997	17	3.27
High-income Asia Pacific	Boys	1998	17	3.30
High-income Asia Pacific	Boys	1999	17	3.33
High-income Asia Pacific	Boys	2000	17	3.36
High-income Asia Pacific	Boys	2001	17	3.39
High-income Asia Pacific	Boys	2002	17	3.42
High-income Asia Pacific	Boys	2003	17	3.45

High-income Asia Pacific	Boys	2004	17	3.48
High-income Asia Pacific	Boys	2005	17	3.51
High-income Asia Pacific	Boys	2006	17	3.55
High-income Asia Pacific	Boys	2007	17	3.59
High-income Asia Pacific	Boys	2008	17	3.64
High-income Asia Pacific	Boys	2009	17	3.68
High-income Asia Pacific	Boys	2010	17	3.72
High-income Asia Pacific	Boys	2011	17	3.75
High-income Asia Pacific	Boys	2012	17	3.78
High-income Asia Pacific	Boys	2013	17	3.80
High-income Asia Pacific	Boys	2014	17	3.82
High-income Asia Pacific	Boys	2015	17	3.83
High-income Asia Pacific	Boys	2016	17	3.84
High-income Asia Pacific	Boys	1975	18	2.66
High-income Asia Pacific	Boys	1976	18	2.71
High-income Asia Pacific	Boys	1977	18	2.76
High-income Asia Pacific	Boys	1978	18	2.81
High-income Asia Pacific	Boys	1979	18	2.86
High-income Asia Pacific	Boys	1980	18	2.91
High-income Asia Pacific	Boys	1981	18	2.96
High-income Asia Pacific	Boys	1982	18	3.02
High-income Asia Pacific	Boys	1983	18	3.07
High-income Asia Pacific	Boys	1984	18	3.12
High-income Asia Pacific	Boys	1985	18	3.18
High-income Asia Pacific	Boys	1986	18	3.23
High-income Asia Pacific	Boys	1987	18	3.29
High-income Asia Pacific	Boys	1988	18	3.35
High-income Asia Pacific	Boys	1989	18	3.40
High-income Asia Pacific	Boys	1990	18	3.46
High-income Asia Pacific	Boys	1991	18	3.52
High-income Asia Pacific	Boys	1992	18	3.57
High-income Asia Pacific	Boys	1993	18	3.62
High-income Asia Pacific	Boys	1994	18	3.66
High-income Asia Pacific	Boys	1995	18	3.71
High-income Asia Pacific	Boys	1996	18	3.75
High-income Asia Pacific	Boys	1997	18	3.79
High-income Asia Pacific	Boys	1998	18	3.83
High-income Asia Pacific	Boys	1999	18	3.86
High-income Asia Pacific	Boys	2000	18	3.90
High-income Asia Pacific	Boys	2001	18	3.93
High-income Asia Pacific	Boys	2002	18	3.96
High-income Asia Pacific	Boys	2003	18	3.99
High-income Asia Pacific	Boys	2004	18	4.03
High-income Asia Pacific	Boys	2005	18	4.07
High-income Asia Pacific	Boys	2006	18	4.11
High-income Asia Pacific	Boys	2007	18	4.16
High-income Asia Pacific	Boys	2008	18	4.21
High-income Asia Pacific	Boys	2009	18	4.26
High-income Asia Pacific	Boys	2010	18	4.30
High-income Asia Pacific	Boys	2011	18	4.34
High-income Asia Pacific	Boys	2012	18	4.37
High-income Asia Pacific	Boys	2013	18	4.40

High-income Asia Pacific	Boys	2014	18	4.42
High-income Asia Pacific	Boys	2015	18	4.43
High-income Asia Pacific	Boys	2016	18	4.44
High-income Asia Pacific	Boys	1975	Average	1.58
High-income Asia Pacific	Boys	1976	Average	1.61
High-income Asia Pacific	Boys	1977	Average	1.64
High-income Asia Pacific	Boys	1978	Average	1.68
High-income Asia Pacific	Boys	1979	Average	1.71
High-income Asia Pacific	Boys	1980	Average	1.75
High-income Asia Pacific	Boys	1981	Average	1.78
High-income Asia Pacific	Boys	1982	Average	1.82
High-income Asia Pacific	Boys	1983	Average	1.86
High-income Asia Pacific	Boys	1984	Average	1.89
High-income Asia Pacific	Boys	1985	Average	1.93
High-income Asia Pacific	Boys	1986	Average	1.97
High-income Asia Pacific	Boys	1987	Average	2.01
High-income Asia Pacific	Boys	1988	Average	2.05
High-income Asia Pacific	Boys	1989	Average	2.09
High-income Asia Pacific	Boys	1990	Average	2.13
High-income Asia Pacific	Boys	1991	Average	2.16
High-income Asia Pacific	Boys	1992	Average	2.20
High-income Asia Pacific	Boys	1993	Average	2.23
High-income Asia Pacific	Boys	1994	Average	2.27
High-income Asia Pacific	Boys	1995	Average	2.30
High-income Asia Pacific	Boys	1996	Average	2.32
High-income Asia Pacific	Boys	1997	Average	2.35
High-income Asia Pacific	Boys	1998	Average	2.37
High-income Asia Pacific	Boys	1999	Average	2.39
High-income Asia Pacific	Boys	2000	Average	2.41
High-income Asia Pacific	Boys	2001	Average	2.44
High-income Asia Pacific	Boys	2002	Average	2.46
High-income Asia Pacific	Boys	2003	Average	2.48
High-income Asia Pacific	Boys	2004	Average	2.51
High-income Asia Pacific	Boys	2005	Average	2.54
High-income Asia Pacific	Boys	2006	Average	2.57
High-income Asia Pacific	Boys	2007	Average	2.60
High-income Asia Pacific	Boys	2008	Average	2.63
High-income Asia Pacific	Boys	2009	Average	2.65
High-income Asia Pacific	Boys	2010	Average	2.68
High-income Asia Pacific	Boys	2011	Average	2.70
High-income Asia Pacific	Boys	2012	Average	2.71
High-income Asia Pacific	Boys	2013	Average	2.72
High-income Asia Pacific	Boys	2014	Average	2.73
High-income Asia Pacific	Boys	2015	Average	2.74
High-income Asia Pacific	Boys	2016	Average	2.75
High-income Asia Pacific	Boys	2017	Average	2.75
High-income Asia Pacific	Boys	2018	Average	2.75
High-income Asia Pacific	Boys	2019	Average	2.75
High-income Asia Pacific	Boys	2020	Average	2.75
High-income Asia Pacific	Boys	2021	Average	2.75
High-income Asia Pacific	Boys	2022	Average	2.74
High-income Asia Pacific	Boys	2023	Average	2.74

High-income Asia Pacific	Boys	2024	Average	2.73
High-income Asia Pacific	Boys	2025	Average	2.72
High-income Asia Pacific	Boys	2026	Average	2.71
High-income Asia Pacific	Boys	2027	Average	2.70
High-income Asia Pacific	Boys	2028	Average	2.69
High-income Asia Pacific	Boys	2029	Average	2.67
High-income Asia Pacific	Boys	2030	Average	2.66
High-income Asia Pacific	Girls	1975	12	0.64
High-income Asia Pacific	Girls	1976	12	0.65
High-income Asia Pacific	Girls	1977	12	0.66
High-income Asia Pacific	Girls	1978	12	0.67
High-income Asia Pacific	Girls	1979	12	0.68
High-income Asia Pacific	Girls	1980	12	0.69
High-income Asia Pacific	Girls	1981	12	0.70
High-income Asia Pacific	Girls	1982	12	0.71
High-income Asia Pacific	Girls	1983	12	0.73
High-income Asia Pacific	Girls	1984	12	0.74
High-income Asia Pacific	Girls	1985	12	0.75
High-income Asia Pacific	Girls	1986	12	0.76
High-income Asia Pacific	Girls	1987	12	0.77
High-income Asia Pacific	Girls	1988	12	0.78
High-income Asia Pacific	Girls	1989	12	0.79
High-income Asia Pacific	Girls	1990	12	0.81
High-income Asia Pacific	Girls	1991	12	0.82
High-income Asia Pacific	Girls	1992	12	0.83
High-income Asia Pacific	Girls	1993	12	0.84
High-income Asia Pacific	Girls	1994	12	0.85
High-income Asia Pacific	Girls	1995	12	0.86
High-income Asia Pacific	Girls	1996	12	0.87
High-income Asia Pacific	Girls	1997	12	0.88
High-income Asia Pacific	Girls	1998	12	0.89
High-income Asia Pacific	Girls	1999	12	0.89
High-income Asia Pacific	Girls	2000	12	0.90
High-income Asia Pacific	Girls	2001	12	0.91
High-income Asia Pacific	Girls	2002	12	0.92
High-income Asia Pacific	Girls	2003	12	0.93
High-income Asia Pacific	Girls	2004	12	0.94
High-income Asia Pacific	Girls	2005	12	0.95
High-income Asia Pacific	Girls	2006	12	0.96
High-income Asia Pacific	Girls	2007	12	0.97
High-income Asia Pacific	Girls	2008	12	0.98
High-income Asia Pacific	Girls	2009	12	0.98
High-income Asia Pacific	Girls	2010	12	0.99
High-income Asia Pacific	Girls	2011	12	0.99
High-income Asia Pacific	Girls	2012	12	0.99
High-income Asia Pacific	Girls	2013	12	1.00
High-income Asia Pacific	Girls	2014	12	1.00
High-income Asia Pacific	Girls	2015	12	1.00
High-income Asia Pacific	Girls	2016	12	1.00
High-income Asia Pacific	Girls	1975	13	0.89
High-income Asia Pacific	Girls	1976	13	0.90
High-income Asia Pacific	Girls	1977	13	0.92

High-income Asia Pacific	Girls	1978	13	0.93
High-income Asia Pacific	Girls	1979	13	0.95
High-income Asia Pacific	Girls	1980	13	0.96
High-income Asia Pacific	Girls	1981	13	0.98
High-income Asia Pacific	Girls	1982	13	0.99
High-income Asia Pacific	Girls	1983	13	1.00
High-income Asia Pacific	Girls	1984	13	1.02
High-income Asia Pacific	Girls	1985	13	1.03
High-income Asia Pacific	Girls	1986	13	1.05
High-income Asia Pacific	Girls	1987	13	1.06
High-income Asia Pacific	Girls	1988	13	1.08
High-income Asia Pacific	Girls	1989	13	1.10
High-income Asia Pacific	Girls	1990	13	1.11
High-income Asia Pacific	Girls	1991	13	1.13
High-income Asia Pacific	Girls	1992	13	1.14
High-income Asia Pacific	Girls	1993	13	1.16
High-income Asia Pacific	Girls	1994	13	1.17
High-income Asia Pacific	Girls	1995	13	1.19
High-income Asia Pacific	Girls	1996	13	1.20
High-income Asia Pacific	Girls	1997	13	1.21
High-income Asia Pacific	Girls	1998	13	1.22
High-income Asia Pacific	Girls	1999	13	1.23
High-income Asia Pacific	Girls	2000	13	1.24
High-income Asia Pacific	Girls	2001	13	1.25
High-income Asia Pacific	Girls	2002	13	1.26
High-income Asia Pacific	Girls	2003	13	1.27
High-income Asia Pacific	Girls	2004	13	1.29
High-income Asia Pacific	Girls	2005	13	1.30
High-income Asia Pacific	Girls	2006	13	1.32
High-income Asia Pacific	Girls	2007	13	1.33
High-income Asia Pacific	Girls	2008	13	1.34
High-income Asia Pacific	Girls	2009	13	1.35
High-income Asia Pacific	Girls	2010	13	1.35
High-income Asia Pacific	Girls	2011	13	1.36
High-income Asia Pacific	Girls	2012	13	1.36
High-income Asia Pacific	Girls	2013	13	1.37
High-income Asia Pacific	Girls	2014	13	1.37
High-income Asia Pacific	Girls	2015	13	1.37
High-income Asia Pacific	Girls	2016	13	1.38
High-income Asia Pacific	Girls	1975	14	1.17
High-income Asia Pacific	Girls	1976	14	1.19
High-income Asia Pacific	Girls	1977	14	1.21
High-income Asia Pacific	Girls	1978	14	1.22
High-income Asia Pacific	Girls	1979	14	1.24
High-income Asia Pacific	Girls	1980	14	1.26
High-income Asia Pacific	Girls	1981	14	1.28
High-income Asia Pacific	Girls	1982	14	1.30
High-income Asia Pacific	Girls	1983	14	1.31
High-income Asia Pacific	Girls	1984	14	1.33
High-income Asia Pacific	Girls	1985	14	1.35
High-income Asia Pacific	Girls	1986	14	1.37
High-income Asia Pacific	Girls	1987	14	1.39

High-income Asia Pacific	Girls	1988	14	1.41
High-income Asia Pacific	Girls	1989	14	1.43
High-income Asia Pacific	Girls	1990	14	1.45
High-income Asia Pacific	Girls	1991	14	1.47
High-income Asia Pacific	Girls	1992	14	1.48
High-income Asia Pacific	Girls	1993	14	1.50
High-income Asia Pacific	Girls	1994	14	1.52
High-income Asia Pacific	Girls	1995	14	1.54
High-income Asia Pacific	Girls	1996	14	1.55
High-income Asia Pacific	Girls	1997	14	1.56
High-income Asia Pacific	Girls	1998	14	1.58
High-income Asia Pacific	Girls	1999	14	1.59
High-income Asia Pacific	Girls	2000	14	1.60
High-income Asia Pacific	Girls	2001	14	1.61
High-income Asia Pacific	Girls	2002	14	1.63
High-income Asia Pacific	Girls	2003	14	1.65
High-income Asia Pacific	Girls	2004	14	1.67
High-income Asia Pacific	Girls	2005	14	1.68
High-income Asia Pacific	Girls	2006	14	1.70
High-income Asia Pacific	Girls	2007	14	1.71
High-income Asia Pacific	Girls	2008	14	1.73
High-income Asia Pacific	Girls	2009	14	1.74
High-income Asia Pacific	Girls	2010	14	1.74
High-income Asia Pacific	Girls	2011	14	1.75
High-income Asia Pacific	Girls	2012	14	1.76
High-income Asia Pacific	Girls	2013	14	1.76
High-income Asia Pacific	Girls	2014	14	1.76
High-income Asia Pacific	Girls	2015	14	1.77
High-income Asia Pacific	Girls	2016	14	1.77
High-income Asia Pacific	Girls	1975	15	1.44
High-income Asia Pacific	Girls	1976	15	1.46
High-income Asia Pacific	Girls	1977	15	1.48
High-income Asia Pacific	Girls	1978	15	1.50
High-income Asia Pacific	Girls	1979	15	1.52
High-income Asia Pacific	Girls	1980	15	1.54
High-income Asia Pacific	Girls	1981	15	1.56
High-income Asia Pacific	Girls	1982	15	1.58
High-income Asia Pacific	Girls	1983	15	1.60
High-income Asia Pacific	Girls	1984	15	1.62
High-income Asia Pacific	Girls	1985	15	1.64
High-income Asia Pacific	Girls	1986	15	1.66
High-income Asia Pacific	Girls	1987	15	1.68
High-income Asia Pacific	Girls	1988	15	1.71
High-income Asia Pacific	Girls	1989	15	1.73
High-income Asia Pacific	Girls	1990	15	1.75
High-income Asia Pacific	Girls	1991	15	1.77
High-income Asia Pacific	Girls	1992	15	1.79
High-income Asia Pacific	Girls	1993	15	1.81
High-income Asia Pacific	Girls	1994	15	1.83
High-income Asia Pacific	Girls	1995	15	1.84
High-income Asia Pacific	Girls	1996	15	1.86
High-income Asia Pacific	Girls	1997	15	1.88

High-income Asia Pacific	Girls	1998	15	1.89
High-income Asia Pacific	Girls	1999	15	1.91
High-income Asia Pacific	Girls	2000	15	1.92
High-income Asia Pacific	Girls	2001	15	1.94
High-income Asia Pacific	Girls	2002	15	1.95
High-income Asia Pacific	Girls	2003	15	1.96
High-income Asia Pacific	Girls	2004	15	1.98
High-income Asia Pacific	Girls	2005	15	2.00
High-income Asia Pacific	Girls	2006	15	2.01
High-income Asia Pacific	Girls	2007	15	2.03
High-income Asia Pacific	Girls	2008	15	2.06
High-income Asia Pacific	Girls	2009	15	2.08
High-income Asia Pacific	Girls	2010	15	2.09
High-income Asia Pacific	Girls	2011	15	2.11
High-income Asia Pacific	Girls	2012	15	2.12
High-income Asia Pacific	Girls	2013	15	2.13
High-income Asia Pacific	Girls	2014	15	2.14
High-income Asia Pacific	Girls	2015	15	2.15
High-income Asia Pacific	Girls	2016	15	2.15
High-income Asia Pacific	Girls	1975	16	1.65
High-income Asia Pacific	Girls	1976	16	1.67
High-income Asia Pacific	Girls	1977	16	1.69
High-income Asia Pacific	Girls	1978	16	1.72
High-income Asia Pacific	Girls	1979	16	1.74
High-income Asia Pacific	Girls	1980	16	1.76
High-income Asia Pacific	Girls	1981	16	1.78
High-income Asia Pacific	Girls	1982	16	1.80
High-income Asia Pacific	Girls	1983	16	1.82
High-income Asia Pacific	Girls	1984	16	1.84
High-income Asia Pacific	Girls	1985	16	1.86
High-income Asia Pacific	Girls	1986	16	1.89
High-income Asia Pacific	Girls	1987	16	1.91
High-income Asia Pacific	Girls	1988	16	1.93
High-income Asia Pacific	Girls	1989	16	1.95
High-income Asia Pacific	Girls	1990	16	1.98
High-income Asia Pacific	Girls	1991	16	2.00
High-income Asia Pacific	Girls	1992	16	2.02
High-income Asia Pacific	Girls	1993	16	2.04
High-income Asia Pacific	Girls	1994	16	2.06
High-income Asia Pacific	Girls	1995	16	2.08
High-income Asia Pacific	Girls	1996	16	2.10
High-income Asia Pacific	Girls	1997	16	2.12
High-income Asia Pacific	Girls	1998	16	2.13
High-income Asia Pacific	Girls	1999	16	2.15
High-income Asia Pacific	Girls	2000	16	2.16
High-income Asia Pacific	Girls	2001	16	2.18
High-income Asia Pacific	Girls	2002	16	2.19
High-income Asia Pacific	Girls	2003	16	2.21
High-income Asia Pacific	Girls	2004	16	2.22
High-income Asia Pacific	Girls	2005	16	2.24
High-income Asia Pacific	Girls	2006	16	2.26
High-income Asia Pacific	Girls	2007	16	2.29

High-income Asia Pacific	Girls	2008	16	2.31
High-income Asia Pacific	Girls	2009	16	2.33
High-income Asia Pacific	Girls	2010	16	2.35
High-income Asia Pacific	Girls	2011	16	2.37
High-income Asia Pacific	Girls	2012	16	2.38
High-income Asia Pacific	Girls	2013	16	2.39
High-income Asia Pacific	Girls	2014	16	2.40
High-income Asia Pacific	Girls	2015	16	2.41
High-income Asia Pacific	Girls	2016	16	2.42
High-income Asia Pacific	Girls	1975	17	1.79
High-income Asia Pacific	Girls	1976	17	1.81
High-income Asia Pacific	Girls	1977	17	1.84
High-income Asia Pacific	Girls	1978	17	1.86
High-income Asia Pacific	Girls	1979	17	1.88
High-income Asia Pacific	Girls	1980	17	1.91
High-income Asia Pacific	Girls	1981	17	1.93
High-income Asia Pacific	Girls	1982	17	1.95
High-income Asia Pacific	Girls	1983	17	1.97
High-income Asia Pacific	Girls	1984	17	1.99
High-income Asia Pacific	Girls	1985	17	2.01
High-income Asia Pacific	Girls	1986	17	2.04
High-income Asia Pacific	Girls	1987	17	2.06
High-income Asia Pacific	Girls	1988	17	2.08
High-income Asia Pacific	Girls	1989	17	2.11
High-income Asia Pacific	Girls	1990	17	2.13
High-income Asia Pacific	Girls	1991	17	2.15
High-income Asia Pacific	Girls	1992	17	2.17
High-income Asia Pacific	Girls	1993	17	2.20
High-income Asia Pacific	Girls	1994	17	2.22
High-income Asia Pacific	Girls	1995	17	2.24
High-income Asia Pacific	Girls	1996	17	2.26
High-income Asia Pacific	Girls	1997	17	2.28
High-income Asia Pacific	Girls	1998	17	2.30
High-income Asia Pacific	Girls	1999	17	2.31
High-income Asia Pacific	Girls	2000	17	2.33
High-income Asia Pacific	Girls	2001	17	2.34
High-income Asia Pacific	Girls	2002	17	2.36
High-income Asia Pacific	Girls	2003	17	2.37
High-income Asia Pacific	Girls	2004	17	2.39
High-income Asia Pacific	Girls	2005	17	2.41
High-income Asia Pacific	Girls	2006	17	2.43
High-income Asia Pacific	Girls	2007	17	2.46
High-income Asia Pacific	Girls	2008	17	2.48
High-income Asia Pacific	Girls	2009	17	2.50
High-income Asia Pacific	Girls	2010	17	2.53
High-income Asia Pacific	Girls	2011	17	2.54
High-income Asia Pacific	Girls	2012	17	2.56
High-income Asia Pacific	Girls	2013	17	2.57
High-income Asia Pacific	Girls	2014	17	2.58
High-income Asia Pacific	Girls	2015	17	2.59
High-income Asia Pacific	Girls	2016	17	2.60
High-income Asia Pacific	Girls	1975	18	1.89

High-income Asia Pacific	Girls	1976	18	1.92
High-income Asia Pacific	Girls	1977	18	1.94
High-income Asia Pacific	Girls	1978	18	1.96
High-income Asia Pacific	Girls	1979	18	1.99
High-income Asia Pacific	Girls	1980	18	2.01
High-income Asia Pacific	Girls	1981	18	2.03
High-income Asia Pacific	Girls	1982	18	2.05
High-income Asia Pacific	Girls	1983	18	2.08
High-income Asia Pacific	Girls	1984	18	2.10
High-income Asia Pacific	Girls	1985	18	2.12
High-income Asia Pacific	Girls	1986	18	2.15
High-income Asia Pacific	Girls	1987	18	2.17
High-income Asia Pacific	Girls	1988	18	2.19
High-income Asia Pacific	Girls	1989	18	2.22
High-income Asia Pacific	Girls	1990	18	2.24
High-income Asia Pacific	Girls	1991	18	2.27
High-income Asia Pacific	Girls	1992	18	2.29
High-income Asia Pacific	Girls	1993	18	2.32
High-income Asia Pacific	Girls	1994	18	2.34
High-income Asia Pacific	Girls	1995	18	2.36
High-income Asia Pacific	Girls	1996	18	2.38
High-income Asia Pacific	Girls	1997	18	2.40
High-income Asia Pacific	Girls	1998	18	2.42
High-income Asia Pacific	Girls	1999	18	2.44
High-income Asia Pacific	Girls	2000	18	2.46
High-income Asia Pacific	Girls	2001	18	2.47
High-income Asia Pacific	Girls	2002	18	2.49
High-income Asia Pacific	Girls	2003	18	2.51
High-income Asia Pacific	Girls	2004	18	2.53
High-income Asia Pacific	Girls	2005	18	2.55
High-income Asia Pacific	Girls	2006	18	2.57
High-income Asia Pacific	Girls	2007	18	2.60
High-income Asia Pacific	Girls	2008	18	2.62
High-income Asia Pacific	Girls	2009	18	2.65
High-income Asia Pacific	Girls	2010	18	2.67
High-income Asia Pacific	Girls	2011	18	2.69
High-income Asia Pacific	Girls	2012	18	2.71
High-income Asia Pacific	Girls	2013	18	2.72
High-income Asia Pacific	Girls	2014	18	2.74
High-income Asia Pacific	Girls	2015	18	2.74
High-income Asia Pacific	Girls	2016	18	2.75
High-income Asia Pacific	Girls	1975	Average	1.35
High-income Asia Pacific	Girls	1976	Average	1.37
High-income Asia Pacific	Girls	1977	Average	1.39
High-income Asia Pacific	Girls	1978	Average	1.41
High-income Asia Pacific	Girls	1979	Average	1.43
High-income Asia Pacific	Girls	1980	Average	1.45
High-income Asia Pacific	Girls	1981	Average	1.47
High-income Asia Pacific	Girls	1982	Average	1.48
High-income Asia Pacific	Girls	1983	Average	1.50
High-income Asia Pacific	Girls	1984	Average	1.52
High-income Asia Pacific	Girls	1985	Average	1.54

High-income Asia Pacific	Girls	1986	Average	1.56
High-income Asia Pacific	Girls	1987	Average	1.58
High-income Asia Pacific	Girls	1988	Average	1.60
High-income Asia Pacific	Girls	1989	Average	1.62
High-income Asia Pacific	Girls	1990	Average	1.64
High-income Asia Pacific	Girls	1991	Average	1.66
High-income Asia Pacific	Girls	1992	Average	1.68
High-income Asia Pacific	Girls	1993	Average	1.69
High-income Asia Pacific	Girls	1994	Average	1.71
High-income Asia Pacific	Girls	1995	Average	1.73
High-income Asia Pacific	Girls	1996	Average	1.75
High-income Asia Pacific	Girls	1997	Average	1.76
High-income Asia Pacific	Girls	1998	Average	1.78
High-income Asia Pacific	Girls	1999	Average	1.79
High-income Asia Pacific	Girls	2000	Average	1.80
High-income Asia Pacific	Girls	2001	Average	1.81
High-income Asia Pacific	Girls	2002	Average	1.83
High-income Asia Pacific	Girls	2003	Average	1.84
High-income Asia Pacific	Girls	2004	Average	1.86
High-income Asia Pacific	Girls	2005	Average	1.88
High-income Asia Pacific	Girls	2006	Average	1.89
High-income Asia Pacific	Girls	2007	Average	1.91
High-income Asia Pacific	Girls	2008	Average	1.93
High-income Asia Pacific	Girls	2009	Average	1.95
High-income Asia Pacific	Girls	2010	Average	1.96
High-income Asia Pacific	Girls	2011	Average	1.97
High-income Asia Pacific	Girls	2012	Average	1.98
High-income Asia Pacific	Girls	2013	Average	1.99
High-income Asia Pacific	Girls	2014	Average	2.00
High-income Asia Pacific	Girls	2015	Average	2.00
High-income Asia Pacific	Girls	2016	Average	2.01
High-income Asia Pacific	Girls	2017	Average	2.02
High-income Asia Pacific	Girls	2018	Average	2.03
High-income Asia Pacific	Girls	2019	Average	2.03
High-income Asia Pacific	Girls	2020	Average	2.04
High-income Asia Pacific	Girls	2021	Average	2.05
High-income Asia Pacific	Girls	2022	Average	2.06
High-income Asia Pacific	Girls	2023	Average	2.06
High-income Asia Pacific	Girls	2024	Average	2.07
High-income Asia Pacific	Girls	2025	Average	2.08
High-income Asia Pacific	Girls	2026	Average	2.08
High-income Asia Pacific	Girls	2027	Average	2.09
High-income Asia Pacific	Girls	2028	Average	2.10
High-income Asia Pacific	Girls	2029	Average	2.10
High-income Asia Pacific	Girls	2030	Average	2.11
High-income Western countries	Boys	1975	12	0.98
High-income Western countries	Boys	1976	12	0.99
High-income Western countries	Boys	1977	12	1.01
High-income Western countries	Boys	1978	12	1.03
High-income Western countries	Boys	1979	12	1.05
High-income Western countries	Boys	1980	12	1.07
High-income Western countries	Boys	1981	12	1.09

High-income Western countries	Boys	1982	12	1.11
High-income Western countries	Boys	1983	12	1.13
High-income Western countries	Boys	1984	12	1.16
High-income Western countries	Boys	1985	12	1.18
High-income Western countries	Boys	1986	12	1.21
High-income Western countries	Boys	1987	12	1.23
High-income Western countries	Boys	1988	12	1.26
High-income Western countries	Boys	1989	12	1.29
High-income Western countries	Boys	1990	12	1.31
High-income Western countries	Boys	1991	12	1.34
High-income Western countries	Boys	1992	12	1.37
High-income Western countries	Boys	1993	12	1.40
High-income Western countries	Boys	1994	12	1.42
High-income Western countries	Boys	1995	12	1.45
High-income Western countries	Boys	1996	12	1.48
High-income Western countries	Boys	1997	12	1.51
High-income Western countries	Boys	1998	12	1.53
High-income Western countries	Boys	1999	12	1.56
High-income Western countries	Boys	2000	12	1.59
High-income Western countries	Boys	2001	12	1.61
High-income Western countries	Boys	2002	12	1.64
High-income Western countries	Boys	2003	12	1.66
High-income Western countries	Boys	2004	12	1.68
High-income Western countries	Boys	2005	12	1.70
High-income Western countries	Boys	2006	12	1.72
High-income Western countries	Boys	2007	12	1.74
High-income Western countries	Boys	2008	12	1.76
High-income Western countries	Boys	2009	12	1.77
High-income Western countries	Boys	2010	12	1.79
High-income Western countries	Boys	2011	12	1.80
High-income Western countries	Boys	2012	12	1.81
High-income Western countries	Boys	2013	12	1.83
High-income Western countries	Boys	2014	12	1.84
High-income Western countries	Boys	2015	12	1.85
High-income Western countries	Boys	2016	12	1.87
High-income Western countries	Boys	1975	13	1.33
High-income Western countries	Boys	1976	13	1.35
High-income Western countries	Boys	1977	13	1.37
High-income Western countries	Boys	1978	13	1.40
High-income Western countries	Boys	1979	13	1.43
High-income Western countries	Boys	1980	13	1.45
High-income Western countries	Boys	1981	13	1.48
High-income Western countries	Boys	1982	13	1.51
High-income Western countries	Boys	1983	13	1.55
High-income Western countries	Boys	1984	13	1.58
High-income Western countries	Boys	1985	13	1.62
High-income Western countries	Boys	1986	13	1.65
High-income Western countries	Boys	1987	13	1.69
High-income Western countries	Boys	1988	13	1.73
High-income Western countries	Boys	1989	13	1.77
High-income Western countries	Boys	1990	13	1.80
High-income Western countries	Boys	1991	13	1.84

High-income Western countries	Boys	1992	13	1.88
High-income Western countries	Boys	1993	13	1.92
High-income Western countries	Boys	1994	13	1.96
High-income Western countries	Boys	1995	13	2.00
High-income Western countries	Boys	1996	13	2.04
High-income Western countries	Boys	1997	13	2.08
High-income Western countries	Boys	1998	13	2.13
High-income Western countries	Boys	1999	13	2.17
High-income Western countries	Boys	2000	13	2.20
High-income Western countries	Boys	2001	13	2.24
High-income Western countries	Boys	2002	13	2.28
High-income Western countries	Boys	2003	13	2.31
High-income Western countries	Boys	2004	13	2.34
High-income Western countries	Boys	2005	13	2.37
High-income Western countries	Boys	2006	13	2.40
High-income Western countries	Boys	2007	13	2.43
High-income Western countries	Boys	2008	13	2.45
High-income Western countries	Boys	2009	13	2.48
High-income Western countries	Boys	2010	13	2.50
High-income Western countries	Boys	2011	13	2.52
High-income Western countries	Boys	2012	13	2.54
High-income Western countries	Boys	2013	13	2.56
High-income Western countries	Boys	2014	13	2.58
High-income Western countries	Boys	2015	13	2.60
High-income Western countries	Boys	2016	13	2.61
High-income Western countries	Boys	1975	14	1.77
High-income Western countries	Boys	1976	14	1.80
High-income Western countries	Boys	1977	14	1.83
High-income Western countries	Boys	1978	14	1.86
High-income Western countries	Boys	1979	14	1.90
High-income Western countries	Boys	1980	14	1.93
High-income Western countries	Boys	1981	14	1.97
High-income Western countries	Boys	1982	14	2.01
High-income Western countries	Boys	1983	14	2.06
High-income Western countries	Boys	1984	14	2.10
High-income Western countries	Boys	1985	14	2.15
High-income Western countries	Boys	1986	14	2.20
High-income Western countries	Boys	1987	14	2.25
High-income Western countries	Boys	1988	14	2.30
High-income Western countries	Boys	1989	14	2.35
High-income Western countries	Boys	1990	14	2.41
High-income Western countries	Boys	1991	14	2.46
High-income Western countries	Boys	1992	14	2.51
High-income Western countries	Boys	1993	14	2.57
High-income Western countries	Boys	1994	14	2.62
High-income Western countries	Boys	1995	14	2.68
High-income Western countries	Boys	1996	14	2.74
High-income Western countries	Boys	1997	14	2.79
High-income Western countries	Boys	1998	14	2.85
High-income Western countries	Boys	1999	14	2.91
High-income Western countries	Boys	2000	14	2.96
High-income Western countries	Boys	2001	14	3.01

High-income Western countries	Boys	2002	14	3.06
High-income Western countries	Boys	2003	14	3.11
High-income Western countries	Boys	2004	14	3.16
High-income Western countries	Boys	2005	14	3.20
High-income Western countries	Boys	2006	14	3.24
High-income Western countries	Boys	2007	14	3.27
High-income Western countries	Boys	2008	14	3.31
High-income Western countries	Boys	2009	14	3.34
High-income Western countries	Boys	2010	14	3.37
High-income Western countries	Boys	2011	14	3.40
High-income Western countries	Boys	2012	14	3.43
High-income Western countries	Boys	2013	14	3.46
High-income Western countries	Boys	2014	14	3.48
High-income Western countries	Boys	2015	14	3.51
High-income Western countries	Boys	2016	14	3.54
High-income Western countries	Boys	1975	15	2.28
High-income Western countries	Boys	1976	15	2.32
High-income Western countries	Boys	1977	15	2.36
High-income Western countries	Boys	1978	15	2.40
High-income Western countries	Boys	1979	15	2.44
High-income Western countries	Boys	1980	15	2.48
High-income Western countries	Boys	1981	15	2.53
High-income Western countries	Boys	1982	15	2.58
High-income Western countries	Boys	1983	15	2.63
High-income Western countries	Boys	1984	15	2.68
High-income Western countries	Boys	1985	15	2.74
High-income Western countries	Boys	1986	15	2.79
High-income Western countries	Boys	1987	15	2.86
High-income Western countries	Boys	1988	15	2.92
High-income Western countries	Boys	1989	15	2.99
High-income Western countries	Boys	1990	15	3.05
High-income Western countries	Boys	1991	15	3.12
High-income Western countries	Boys	1992	15	3.20
High-income Western countries	Boys	1993	15	3.27
High-income Western countries	Boys	1994	15	3.34
High-income Western countries	Boys	1995	15	3.42
High-income Western countries	Boys	1996	15	3.49
High-income Western countries	Boys	1997	15	3.57
High-income Western countries	Boys	1998	15	3.64
High-income Western countries	Boys	1999	15	3.71
High-income Western countries	Boys	2000	15	3.78
High-income Western countries	Boys	2001	15	3.84
High-income Western countries	Boys	2002	15	3.91
High-income Western countries	Boys	2003	15	3.97
High-income Western countries	Boys	2004	15	4.03
High-income Western countries	Boys	2005	15	4.08
High-income Western countries	Boys	2006	15	4.13
High-income Western countries	Boys	2007	15	4.18
High-income Western countries	Boys	2008	15	4.23
High-income Western countries	Boys	2009	15	4.27
High-income Western countries	Boys	2010	15	4.31
High-income Western countries	Boys	2011	15	4.35

High-income Western countries	Boys	2012	15	4.39
High-income Western countries	Boys	2013	15	4.43
High-income Western countries	Boys	2014	15	4.46
High-income Western countries	Boys	2015	15	4.50
High-income Western countries	Boys	2016	15	4.54
High-income Western countries	Boys	1975	16	2.82
High-income Western countries	Boys	1976	16	2.87
High-income Western countries	Boys	1977	16	2.91
High-income Western countries	Boys	1978	16	2.96
High-income Western countries	Boys	1979	16	3.01
High-income Western countries	Boys	1980	16	3.07
High-income Western countries	Boys	1981	16	3.12
High-income Western countries	Boys	1982	16	3.18
High-income Western countries	Boys	1983	16	3.24
High-income Western countries	Boys	1984	16	3.30
High-income Western countries	Boys	1985	16	3.37
High-income Western countries	Boys	1986	16	3.44
High-income Western countries	Boys	1987	16	3.51
High-income Western countries	Boys	1988	16	3.59
High-income Western countries	Boys	1989	16	3.67
High-income Western countries	Boys	1990	16	3.75
High-income Western countries	Boys	1991	16	3.84
High-income Western countries	Boys	1992	16	3.92
High-income Western countries	Boys	1993	16	4.01
High-income Western countries	Boys	1994	16	4.11
High-income Western countries	Boys	1995	16	4.20
High-income Western countries	Boys	1996	16	4.29
High-income Western countries	Boys	1997	16	4.38
High-income Western countries	Boys	1998	16	4.46
High-income Western countries	Boys	1999	16	4.55
High-income Western countries	Boys	2000	16	4.64
High-income Western countries	Boys	2001	16	4.72
High-income Western countries	Boys	2002	16	4.79
High-income Western countries	Boys	2003	16	4.87
High-income Western countries	Boys	2004	16	4.94
High-income Western countries	Boys	2005	16	5.01
High-income Western countries	Boys	2006	16	5.07
High-income Western countries	Boys	2007	16	5.13
High-income Western countries	Boys	2008	16	5.19
High-income Western countries	Boys	2009	16	5.24
High-income Western countries	Boys	2010	16	5.29
High-income Western countries	Boys	2011	16	5.34
High-income Western countries	Boys	2012	16	5.39
High-income Western countries	Boys	2013	16	5.43
High-income Western countries	Boys	2014	16	5.48
High-income Western countries	Boys	2015	16	5.52
High-income Western countries	Boys	2016	16	5.57
High-income Western countries	Boys	1975	17	3.41
High-income Western countries	Boys	1976	17	3.46
High-income Western countries	Boys	1977	17	3.52
High-income Western countries	Boys	1978	17	3.57
High-income Western countries	Boys	1979	17	3.63

High-income Western countries	Boys	1980	17	3.69
High-income Western countries	Boys	1981	17	3.76
High-income Western countries	Boys	1982	17	3.82
High-income Western countries	Boys	1983	17	3.89
High-income Western countries	Boys	1984	17	3.97
High-income Western countries	Boys	1985	17	4.05
High-income Western countries	Boys	1986	17	4.13
High-income Western countries	Boys	1987	17	4.21
High-income Western countries	Boys	1988	17	4.30
High-income Western countries	Boys	1989	17	4.40
High-income Western countries	Boys	1990	17	4.49
High-income Western countries	Boys	1991	17	4.59
High-income Western countries	Boys	1992	17	4.70
High-income Western countries	Boys	1993	17	4.80
High-income Western countries	Boys	1994	17	4.91
High-income Western countries	Boys	1995	17	5.02
High-income Western countries	Boys	1996	17	5.12
High-income Western countries	Boys	1997	17	5.23
High-income Western countries	Boys	1998	17	5.33
High-income Western countries	Boys	1999	17	5.43
High-income Western countries	Boys	2000	17	5.53
High-income Western countries	Boys	2001	17	5.63
High-income Western countries	Boys	2002	17	5.72
High-income Western countries	Boys	2003	17	5.81
High-income Western countries	Boys	2004	17	5.89
High-income Western countries	Boys	2005	17	5.97
High-income Western countries	Boys	2006	17	6.05
High-income Western countries	Boys	2007	17	6.12
High-income Western countries	Boys	2008	17	6.19
High-income Western countries	Boys	2009	17	6.25
High-income Western countries	Boys	2010	17	6.31
High-income Western countries	Boys	2011	17	6.37
High-income Western countries	Boys	2012	17	6.42
High-income Western countries	Boys	2013	17	6.48
High-income Western countries	Boys	2014	17	6.53
High-income Western countries	Boys	2015	17	6.58
High-income Western countries	Boys	2016	17	6.64
High-income Western countries	Boys	1975	18	4.07
High-income Western countries	Boys	1976	18	4.13
High-income Western countries	Boys	1977	18	4.20
High-income Western countries	Boys	1978	18	4.26
High-income Western countries	Boys	1979	18	4.33
High-income Western countries	Boys	1980	18	4.40
High-income Western countries	Boys	1981	18	4.48
High-income Western countries	Boys	1982	18	4.55
High-income Western countries	Boys	1983	18	4.63
High-income Western countries	Boys	1984	18	4.72
High-income Western countries	Boys	1985	18	4.81
High-income Western countries	Boys	1986	18	4.90
High-income Western countries	Boys	1987	18	5.00
High-income Western countries	Boys	1988	18	5.11
High-income Western countries	Boys	1989	18	5.22

High-income Western countries	Boys	1990	18	5.33
High-income Western countries	Boys	1991	18	5.45
High-income Western countries	Boys	1992	18	5.57
High-income Western countries	Boys	1993	18	5.69
High-income Western countries	Boys	1994	18	5.81
High-income Western countries	Boys	1995	18	5.94
High-income Western countries	Boys	1996	18	6.06
High-income Western countries	Boys	1997	18	6.19
High-income Western countries	Boys	1998	18	6.31
High-income Western countries	Boys	1999	18	6.43
High-income Western countries	Boys	2000	18	6.54
High-income Western countries	Boys	2001	18	6.65
High-income Western countries	Boys	2002	18	6.76
High-income Western countries	Boys	2003	18	6.86
High-income Western countries	Boys	2004	18	6.96
High-income Western countries	Boys	2005	18	7.05
High-income Western countries	Boys	2006	18	7.14
High-income Western countries	Boys	2007	18	7.22
High-income Western countries	Boys	2008	18	7.30
High-income Western countries	Boys	2009	18	7.37
High-income Western countries	Boys	2010	18	7.44
High-income Western countries	Boys	2011	18	7.51
High-income Western countries	Boys	2012	18	7.57
High-income Western countries	Boys	2013	18	7.63
High-income Western countries	Boys	2014	18	7.69
High-income Western countries	Boys	2015	18	7.75
High-income Western countries	Boys	2016	18	7.82
High-income Western countries	Boys	1975	Average	2.38
High-income Western countries	Boys	1976	Average	2.42
High-income Western countries	Boys	1977	Average	2.46
High-income Western countries	Boys	1978	Average	2.50
High-income Western countries	Boys	1979	Average	2.54
High-income Western countries	Boys	1980	Average	2.59
High-income Western countries	Boys	1981	Average	2.63
High-income Western countries	Boys	1982	Average	2.68
High-income Western countries	Boys	1983	Average	2.73
High-income Western countries	Boys	1984	Average	2.79
High-income Western countries	Boys	1985	Average	2.84
High-income Western countries	Boys	1986	Average	2.90
High-income Western countries	Boys	1987	Average	2.97
High-income Western countries	Boys	1988	Average	3.03
High-income Western countries	Boys	1989	Average	3.10
High-income Western countries	Boys	1990	Average	3.16
High-income Western countries	Boys	1991	Average	3.24
High-income Western countries	Boys	1992	Average	3.31
High-income Western countries	Boys	1993	Average	3.38
High-income Western countries	Boys	1994	Average	3.45
High-income Western countries	Boys	1995	Average	3.53
High-income Western countries	Boys	1996	Average	3.60
High-income Western countries	Boys	1997	Average	3.68
High-income Western countries	Boys	1998	Average	3.75
High-income Western countries	Boys	1999	Average	3.82

High-income Western countries	Boys	2000	Average	3.89
High-income Western countries	Boys	2001	Average	3.96
High-income Western countries	Boys	2002	Average	4.02
High-income Western countries	Boys	2003	Average	4.09
High-income Western countries	Boys	2004	Average	4.14
High-income Western countries	Boys	2005	Average	4.20
High-income Western countries	Boys	2006	Average	4.25
High-income Western countries	Boys	2007	Average	4.30
High-income Western countries	Boys	2008	Average	4.35
High-income Western countries	Boys	2009	Average	4.39
High-income Western countries	Boys	2010	Average	4.43
High-income Western countries	Boys	2011	Average	4.47
High-income Western countries	Boys	2012	Average	4.51
High-income Western countries	Boys	2013	Average	4.54
High-income Western countries	Boys	2014	Average	4.58
High-income Western countries	Boys	2015	Average	4.62
High-income Western countries	Boys	2016	Average	4.65
High-income Western countries	Boys	2017	Average	4.69
High-income Western countries	Boys	2018	Average	4.73
High-income Western countries	Boys	2019	Average	4.77
High-income Western countries	Boys	2020	Average	4.81
High-income Western countries	Boys	2021	Average	4.86
High-income Western countries	Boys	2022	Average	4.90
High-income Western countries	Boys	2023	Average	4.94
High-income Western countries	Boys	2024	Average	4.99
High-income Western countries	Boys	2025	Average	5.04
High-income Western countries	Boys	2026	Average	5.08
High-income Western countries	Boys	2027	Average	5.13
High-income Western countries	Boys	2028	Average	5.18
High-income Western countries	Boys	2029	Average	5.22
High-income Western countries	Boys	2030	Average	5.27
High-income Western countries	Girls	1975	12	1.14
High-income Western countries	Girls	1976	12	1.16
High-income Western countries	Girls	1977	12	1.18
High-income Western countries	Girls	1978	12	1.20
High-income Western countries	Girls	1979	12	1.22
High-income Western countries	Girls	1980	12	1.24
High-income Western countries	Girls	1981	12	1.27
High-income Western countries	Girls	1982	12	1.29
High-income Western countries	Girls	1983	12	1.32
High-income Western countries	Girls	1984	12	1.35
High-income Western countries	Girls	1985	12	1.37
High-income Western countries	Girls	1986	12	1.40
High-income Western countries	Girls	1987	12	1.43
High-income Western countries	Girls	1988	12	1.46
High-income Western countries	Girls	1989	12	1.49
High-income Western countries	Girls	1990	12	1.52
High-income Western countries	Girls	1991	12	1.56
High-income Western countries	Girls	1992	12	1.59
High-income Western countries	Girls	1993	12	1.62
High-income Western countries	Girls	1994	12	1.65
High-income Western countries	Girls	1995	12	1.69

High-income Western countries	Girls	1996	12	1.72
High-income Western countries	Girls	1997	12	1.75
High-income Western countries	Girls	1998	12	1.79
High-income Western countries	Girls	1999	12	1.82
High-income Western countries	Girls	2000	12	1.85
High-income Western countries	Girls	2001	12	1.88
High-income Western countries	Girls	2002	12	1.91
High-income Western countries	Girls	2003	12	1.94
High-income Western countries	Girls	2004	12	1.96
High-income Western countries	Girls	2005	12	1.98
High-income Western countries	Girls	2006	12	2.00
High-income Western countries	Girls	2007	12	2.02
High-income Western countries	Girls	2008	12	2.04
High-income Western countries	Girls	2009	12	2.05
High-income Western countries	Girls	2010	12	2.07
High-income Western countries	Girls	2011	12	2.08
High-income Western countries	Girls	2012	12	2.10
High-income Western countries	Girls	2013	12	2.11
High-income Western countries	Girls	2014	12	2.13
High-income Western countries	Girls	2015	12	2.14
High-income Western countries	Girls	2016	12	2.15
High-income Western countries	Girls	1975	13	1.60
High-income Western countries	Girls	1976	13	1.63
High-income Western countries	Girls	1977	13	1.66
High-income Western countries	Girls	1978	13	1.68
High-income Western countries	Girls	1979	13	1.71
High-income Western countries	Girls	1980	13	1.74
High-income Western countries	Girls	1981	13	1.78
High-income Western countries	Girls	1982	13	1.81
High-income Western countries	Girls	1983	13	1.84
High-income Western countries	Girls	1984	13	1.88
High-income Western countries	Girls	1985	13	1.92
High-income Western countries	Girls	1986	13	1.96
High-income Western countries	Girls	1987	13	2.00
High-income Western countries	Girls	1988	13	2.04
High-income Western countries	Girls	1989	13	2.09
High-income Western countries	Girls	1990	13	2.13
High-income Western countries	Girls	1991	13	2.18
High-income Western countries	Girls	1992	13	2.22
High-income Western countries	Girls	1993	13	2.27
High-income Western countries	Girls	1994	13	2.31
High-income Western countries	Girls	1995	13	2.36
High-income Western countries	Girls	1996	13	2.41
High-income Western countries	Girls	1997	13	2.45
High-income Western countries	Girls	1998	13	2.50
High-income Western countries	Girls	1999	13	2.55
High-income Western countries	Girls	2000	13	2.59
High-income Western countries	Girls	2001	13	2.63
High-income Western countries	Girls	2002	13	2.67
High-income Western countries	Girls	2003	13	2.71
High-income Western countries	Girls	2004	13	2.74
High-income Western countries	Girls	2005	13	2.77

High-income Western countries	Girls	2006	13	2.80
High-income Western countries	Girls	2007	13	2.83
High-income Western countries	Girls	2008	13	2.85
High-income Western countries	Girls	2009	13	2.87
High-income Western countries	Girls	2010	13	2.89
High-income Western countries	Girls	2011	13	2.92
High-income Western countries	Girls	2012	13	2.94
High-income Western countries	Girls	2013	13	2.95
High-income Western countries	Girls	2014	13	2.97
High-income Western countries	Girls	2015	13	2.99
High-income Western countries	Girls	2016	13	3.01
High-income Western countries	Girls	1975	14	2.10
High-income Western countries	Girls	1976	14	2.14
High-income Western countries	Girls	1977	14	2.17
High-income Western countries	Girls	1978	14	2.21
High-income Western countries	Girls	1979	14	2.24
High-income Western countries	Girls	1980	14	2.28
High-income Western countries	Girls	1981	14	2.32
High-income Western countries	Girls	1982	14	2.37
High-income Western countries	Girls	1983	14	2.41
High-income Western countries	Girls	1984	14	2.46
High-income Western countries	Girls	1985	14	2.51
High-income Western countries	Girls	1986	14	2.56
High-income Western countries	Girls	1987	14	2.61
High-income Western countries	Girls	1988	14	2.66
High-income Western countries	Girls	1989	14	2.72
High-income Western countries	Girls	1990	14	2.77
High-income Western countries	Girls	1991	14	2.83
High-income Western countries	Girls	1992	14	2.89
High-income Western countries	Girls	1993	14	2.95
High-income Western countries	Girls	1994	14	3.01
High-income Western countries	Girls	1995	14	3.07
High-income Western countries	Girls	1996	14	3.13
High-income Western countries	Girls	1997	14	3.19
High-income Western countries	Girls	1998	14	3.25
High-income Western countries	Girls	1999	14	3.30
High-income Western countries	Girls	2000	14	3.36
High-income Western countries	Girls	2001	14	3.41
High-income Western countries	Girls	2002	14	3.46
High-income Western countries	Girls	2003	14	3.51
High-income Western countries	Girls	2004	14	3.55
High-income Western countries	Girls	2005	14	3.59
High-income Western countries	Girls	2006	14	3.63
High-income Western countries	Girls	2007	14	3.66
High-income Western countries	Girls	2008	14	3.69
High-income Western countries	Girls	2009	14	3.72
High-income Western countries	Girls	2010	14	3.75
High-income Western countries	Girls	2011	14	3.77
High-income Western countries	Girls	2012	14	3.80
High-income Western countries	Girls	2013	14	3.82
High-income Western countries	Girls	2014	14	3.85
High-income Western countries	Girls	2015	14	3.87

High-income Western countries	Girls	2016	14	3.89
High-income Western countries	Girls	1975	15	2.58
High-income Western countries	Girls	1976	15	2.62
High-income Western countries	Girls	1977	15	2.66
High-income Western countries	Girls	1978	15	2.70
High-income Western countries	Girls	1979	15	2.75
High-income Western countries	Girls	1980	15	2.79
High-income Western countries	Girls	1981	15	2.84
High-income Western countries	Girls	1982	15	2.88
High-income Western countries	Girls	1983	15	2.93
High-income Western countries	Girls	1984	15	2.98
High-income Western countries	Girls	1985	15	3.03
High-income Western countries	Girls	1986	15	3.08
High-income Western countries	Girls	1987	15	3.14
High-income Western countries	Girls	1988	15	3.20
High-income Western countries	Girls	1989	15	3.26
High-income Western countries	Girls	1990	15	3.33
High-income Western countries	Girls	1991	15	3.39
High-income Western countries	Girls	1992	15	3.46
High-income Western countries	Girls	1993	15	3.53
High-income Western countries	Girls	1994	15	3.60
High-income Western countries	Girls	1995	15	3.67
High-income Western countries	Girls	1996	15	3.75
High-income Western countries	Girls	1997	15	3.82
High-income Western countries	Girls	1998	15	3.89
High-income Western countries	Girls	1999	15	3.95
High-income Western countries	Girls	2000	15	4.02
High-income Western countries	Girls	2001	15	4.08
High-income Western countries	Girls	2002	15	4.14
High-income Western countries	Girls	2003	15	4.20
High-income Western countries	Girls	2004	15	4.25
High-income Western countries	Girls	2005	15	4.29
High-income Western countries	Girls	2006	15	4.34
High-income Western countries	Girls	2007	15	4.38
High-income Western countries	Girls	2008	15	4.41
High-income Western countries	Girls	2009	15	4.45
High-income Western countries	Girls	2010	15	4.48
High-income Western countries	Girls	2011	15	4.51
High-income Western countries	Girls	2012	15	4.54
High-income Western countries	Girls	2013	15	4.57
High-income Western countries	Girls	2014	15	4.60
High-income Western countries	Girls	2015	15	4.63
High-income Western countries	Girls	2016	15	4.66
High-income Western countries	Girls	1975	16	2.97
High-income Western countries	Girls	1976	16	3.01
High-income Western countries	Girls	1977	16	3.06
High-income Western countries	Girls	1978	16	3.11
High-income Western countries	Girls	1979	16	3.15
High-income Western countries	Girls	1980	16	3.20
High-income Western countries	Girls	1981	16	3.25
High-income Western countries	Girls	1982	16	3.30
High-income Western countries	Girls	1983	16	3.36

High-income Western countries	Girls	1984	16	3.41
High-income Western countries	Girls	1985	16	3.47
High-income Western countries	Girls	1986	16	3.53
High-income Western countries	Girls	1987	16	3.59
High-income Western countries	Girls	1988	16	3.66
High-income Western countries	Girls	1989	16	3.73
High-income Western countries	Girls	1990	16	3.80
High-income Western countries	Girls	1991	16	3.87
High-income Western countries	Girls	1992	16	3.95
High-income Western countries	Girls	1993	16	4.03
High-income Western countries	Girls	1994	16	4.11
High-income Western countries	Girls	1995	16	4.19
High-income Western countries	Girls	1996	16	4.27
High-income Western countries	Girls	1997	16	4.35
High-income Western countries	Girls	1998	16	4.42
High-income Western countries	Girls	1999	16	4.50
High-income Western countries	Girls	2000	16	4.57
High-income Western countries	Girls	2001	16	4.64
High-income Western countries	Girls	2002	16	4.71
High-income Western countries	Girls	2003	16	4.77
High-income Western countries	Girls	2004	16	4.83
High-income Western countries	Girls	2005	16	4.88
High-income Western countries	Girls	2006	16	4.93
High-income Western countries	Girls	2007	16	4.97
High-income Western countries	Girls	2008	16	5.01
High-income Western countries	Girls	2009	16	5.05
High-income Western countries	Girls	2010	16	5.09
High-income Western countries	Girls	2011	16	5.12
High-income Western countries	Girls	2012	16	5.16
High-income Western countries	Girls	2013	16	5.19
High-income Western countries	Girls	2014	16	5.22
High-income Western countries	Girls	2015	16	5.25
High-income Western countries	Girls	2016	16	5.29
High-income Western countries	Girls	1975	17	3.30
High-income Western countries	Girls	1976	17	3.35
High-income Western countries	Girls	1977	17	3.40
High-income Western countries	Girls	1978	17	3.45
High-income Western countries	Girls	1979	17	3.51
High-income Western countries	Girls	1980	17	3.56
High-income Western countries	Girls	1981	17	3.61
High-income Western countries	Girls	1982	17	3.67
High-income Western countries	Girls	1983	17	3.73
High-income Western countries	Girls	1984	17	3.79
High-income Western countries	Girls	1985	17	3.85
High-income Western countries	Girls	1986	17	3.92
High-income Western countries	Girls	1987	17	3.99
High-income Western countries	Girls	1988	17	4.06
High-income Western countries	Girls	1989	17	4.14
High-income Western countries	Girls	1990	17	4.22
High-income Western countries	Girls	1991	17	4.30
High-income Western countries	Girls	1992	17	4.38
High-income Western countries	Girls	1993	17	4.47

High-income Western countries	Girls	1994	17	4.56
High-income Western countries	Girls	1995	17	4.65
High-income Western countries	Girls	1996	17	4.74
High-income Western countries	Girls	1997	17	4.82
High-income Western countries	Girls	1998	17	4.91
High-income Western countries	Girls	1999	17	4.99
High-income Western countries	Girls	2000	17	5.07
High-income Western countries	Girls	2001	17	5.15
High-income Western countries	Girls	2002	17	5.22
High-income Western countries	Girls	2003	17	5.29
High-income Western countries	Girls	2004	17	5.35
High-income Western countries	Girls	2005	17	5.41
High-income Western countries	Girls	2006	17	5.46
High-income Western countries	Girls	2007	17	5.51
High-income Western countries	Girls	2008	17	5.56
High-income Western countries	Girls	2009	17	5.60
High-income Western countries	Girls	2010	17	5.64
High-income Western countries	Girls	2011	17	5.68
High-income Western countries	Girls	2012	17	5.72
High-income Western countries	Girls	2013	17	5.75
High-income Western countries	Girls	2014	17	5.79
High-income Western countries	Girls	2015	17	5.82
High-income Western countries	Girls	2016	17	5.86
High-income Western countries	Girls	1975	18	3.67
High-income Western countries	Girls	1976	18	3.72
High-income Western countries	Girls	1977	18	3.78
High-income Western countries	Girls	1978	18	3.84
High-income Western countries	Girls	1979	18	3.90
High-income Western countries	Girls	1980	18	3.96
High-income Western countries	Girls	1981	18	4.02
High-income Western countries	Girls	1982	18	4.08
High-income Western countries	Girls	1983	18	4.15
High-income Western countries	Girls	1984	18	4.22
High-income Western countries	Girls	1985	18	4.29
High-income Western countries	Girls	1986	18	4.36
High-income Western countries	Girls	1987	18	4.44
High-income Western countries	Girls	1988	18	4.53
High-income Western countries	Girls	1989	18	4.61
High-income Western countries	Girls	1990	18	4.70
High-income Western countries	Girls	1991	18	4.79
High-income Western countries	Girls	1992	18	4.89
High-income Western countries	Girls	1993	18	4.98
High-income Western countries	Girls	1994	18	5.08
High-income Western countries	Girls	1995	18	5.18
High-income Western countries	Girls	1996	18	5.28
High-income Western countries	Girls	1997	18	5.38
High-income Western countries	Girls	1998	18	5.48
High-income Western countries	Girls	1999	18	5.57
High-income Western countries	Girls	2000	18	5.66
High-income Western countries	Girls	2001	18	5.75
High-income Western countries	Girls	2002	18	5.83
High-income Western countries	Girls	2003	18	5.91

High-income Western countries	Girls	2004	18	5.98
High-income Western countries	Girls	2005	18	6.04
High-income Western countries	Girls	2006	18	6.10
High-income Western countries	Girls	2007	18	6.15
High-income Western countries	Girls	2008	18	6.21
High-income Western countries	Girls	2009	18	6.25
High-income Western countries	Girls	2010	18	6.30
High-income Western countries	Girls	2011	18	6.34
High-income Western countries	Girls	2012	18	6.38
High-income Western countries	Girls	2013	18	6.42
High-income Western countries	Girls	2014	18	6.46
High-income Western countries	Girls	2015	18	6.49
High-income Western countries	Girls	2016	18	6.53
High-income Western countries	Girls	1975	Average	2.48
High-income Western countries	Girls	1976	Average	2.52
High-income Western countries	Girls	1977	Average	2.56
High-income Western countries	Girls	1978	Average	2.60
High-income Western countries	Girls	1979	Average	2.64
High-income Western countries	Girls	1980	Average	2.68
High-income Western countries	Girls	1981	Average	2.73
High-income Western countries	Girls	1982	Average	2.77
High-income Western countries	Girls	1983	Average	2.82
High-income Western countries	Girls	1984	Average	2.87
High-income Western countries	Girls	1985	Average	2.92
High-income Western countries	Girls	1986	Average	2.97
High-income Western countries	Girls	1987	Average	3.03
High-income Western countries	Girls	1988	Average	3.09
High-income Western countries	Girls	1989	Average	3.15
High-income Western countries	Girls	1990	Average	3.21
High-income Western countries	Girls	1991	Average	3.27
High-income Western countries	Girls	1992	Average	3.34
High-income Western countries	Girls	1993	Average	3.41
High-income Western countries	Girls	1994	Average	3.47
High-income Western countries	Girls	1995	Average	3.54
High-income Western countries	Girls	1996	Average	3.61
High-income Western countries	Girls	1997	Average	3.68
High-income Western countries	Girls	1998	Average	3.75
High-income Western countries	Girls	1999	Average	3.81
High-income Western countries	Girls	2000	Average	3.88
High-income Western countries	Girls	2001	Average	3.94
High-income Western countries	Girls	2002	Average	3.99
High-income Western countries	Girls	2003	Average	4.05
High-income Western countries	Girls	2004	Average	4.09
High-income Western countries	Girls	2005	Average	4.14
High-income Western countries	Girls	2006	Average	4.18
High-income Western countries	Girls	2007	Average	4.22
High-income Western countries	Girls	2008	Average	4.25
High-income Western countries	Girls	2009	Average	4.29
High-income Western countries	Girls	2010	Average	4.32
High-income Western countries	Girls	2011	Average	4.35
High-income Western countries	Girls	2012	Average	4.38
High-income Western countries	Girls	2013	Average	4.40

High-income Western countries	Girls	2014	Average	4.43
High-income Western countries	Girls	2015	Average	4.46
High-income Western countries	Girls	2016	Average	4.49
High-income Western countries	Girls	2017	Average	4.51
High-income Western countries	Girls	2018	Average	4.54
High-income Western countries	Girls	2019	Average	4.57
High-income Western countries	Girls	2020	Average	4.60
High-income Western countries	Girls	2021	Average	4.63
High-income Western countries	Girls	2022	Average	4.66
High-income Western countries	Girls	2023	Average	4.69
High-income Western countries	Girls	2024	Average	4.73
High-income Western countries	Girls	2025	Average	4.76
High-income Western countries	Girls	2026	Average	4.79
High-income Western countries	Girls	2027	Average	4.82
High-income Western countries	Girls	2028	Average	4.85
High-income Western countries	Girls	2029	Average	4.88
High-income Western countries	Girls	2030	Average	4.92
Latin America and Caribbean	Boys	1975	12	0.64
Latin America and Caribbean	Boys	1976	12	0.66
Latin America and Caribbean	Boys	1977	12	0.67
Latin America and Caribbean	Boys	1978	12	0.69
Latin America and Caribbean	Boys	1979	12	0.70
Latin America and Caribbean	Boys	1980	12	0.72
Latin America and Caribbean	Boys	1981	12	0.74
Latin America and Caribbean	Boys	1982	12	0.75
Latin America and Caribbean	Boys	1983	12	0.77
Latin America and Caribbean	Boys	1984	12	0.79
Latin America and Caribbean	Boys	1985	12	0.81
Latin America and Caribbean	Boys	1986	12	0.83
Latin America and Caribbean	Boys	1987	12	0.85
Latin America and Caribbean	Boys	1988	12	0.87
Latin America and Caribbean	Boys	1989	12	0.89
Latin America and Caribbean	Boys	1990	12	0.91
Latin America and Caribbean	Boys	1991	12	0.93
Latin America and Caribbean	Boys	1992	12	0.95
Latin America and Caribbean	Boys	1993	12	0.97
Latin America and Caribbean	Boys	1994	12	1.00
Latin America and Caribbean	Boys	1995	12	1.02
Latin America and Caribbean	Boys	1996	12	1.04
Latin America and Caribbean	Boys	1997	12	1.07
Latin America and Caribbean	Boys	1998	12	1.09
Latin America and Caribbean	Boys	1999	12	1.12
Latin America and Caribbean	Boys	2000	12	1.15
Latin America and Caribbean	Boys	2001	12	1.17
Latin America and Caribbean	Boys	2002	12	1.20
Latin America and Caribbean	Boys	2003	12	1.22
Latin America and Caribbean	Boys	2004	12	1.25
Latin America and Caribbean	Boys	2005	12	1.28
Latin America and Caribbean	Boys	2006	12	1.30
Latin America and Caribbean	Boys	2007	12	1.33
Latin America and Caribbean	Boys	2008	12	1.36
Latin America and Caribbean	Boys	2009	12	1.38

Latin America and Caribbean	Boys	2010	12	1.41
Latin America and Caribbean	Boys	2011	12	1.44
Latin America and Caribbean	Boys	2012	12	1.47
Latin America and Caribbean	Boys	2013	12	1.50
Latin America and Caribbean	Boys	2014	12	1.52
Latin America and Caribbean	Boys	2015	12	1.55
Latin America and Caribbean	Boys	2016	12	1.58
Latin America and Caribbean	Boys	1975	13	0.86
Latin America and Caribbean	Boys	1976	13	0.88
Latin America and Caribbean	Boys	1977	13	0.90
Latin America and Caribbean	Boys	1978	13	0.92
Latin America and Caribbean	Boys	1979	13	0.94
Latin America and Caribbean	Boys	1980	13	0.96
Latin America and Caribbean	Boys	1981	13	0.98
Latin America and Caribbean	Boys	1982	13	1.01
Latin America and Caribbean	Boys	1983	13	1.03
Latin America and Caribbean	Boys	1984	13	1.06
Latin America and Caribbean	Boys	1985	13	1.09
Latin America and Caribbean	Boys	1986	13	1.11
Latin America and Caribbean	Boys	1987	13	1.14
Latin America and Caribbean	Boys	1988	13	1.17
Latin America and Caribbean	Boys	1989	13	1.20
Latin America and Caribbean	Boys	1990	13	1.23
Latin America and Caribbean	Boys	1991	13	1.25
Latin America and Caribbean	Boys	1992	13	1.28
Latin America and Caribbean	Boys	1993	13	1.31
Latin America and Caribbean	Boys	1994	13	1.34
Latin America and Caribbean	Boys	1995	13	1.37
Latin America and Caribbean	Boys	1996	13	1.41
Latin America and Caribbean	Boys	1997	13	1.44
Latin America and Caribbean	Boys	1998	13	1.48
Latin America and Caribbean	Boys	1999	13	1.51
Latin America and Caribbean	Boys	2000	13	1.55
Latin America and Caribbean	Boys	2001	13	1.59
Latin America and Caribbean	Boys	2002	13	1.62
Latin America and Caribbean	Boys	2003	13	1.66
Latin America and Caribbean	Boys	2004	13	1.70
Latin America and Caribbean	Boys	2005	13	1.73
Latin America and Caribbean	Boys	2006	13	1.77
Latin America and Caribbean	Boys	2007	13	1.81
Latin America and Caribbean	Boys	2008	13	1.85
Latin America and Caribbean	Boys	2009	13	1.89
Latin America and Caribbean	Boys	2010	13	1.93
Latin America and Caribbean	Boys	2011	13	1.97
Latin America and Caribbean	Boys	2012	13	2.01
Latin America and Caribbean	Boys	2013	13	2.05
Latin America and Caribbean	Boys	2014	13	2.09
Latin America and Caribbean	Boys	2015	13	2.13
Latin America and Caribbean	Boys	2016	13	2.18
Latin America and Caribbean	Boys	1975	14	1.14
Latin America and Caribbean	Boys	1976	14	1.16
Latin America and Caribbean	Boys	1977	14	1.19

Latin America and Caribbean	Boys	1978	14	1.21
Latin America and Caribbean	Boys	1979	14	1.24
Latin America and Caribbean	Boys	1980	14	1.27
Latin America and Caribbean	Boys	1981	14	1.30
Latin America and Caribbean	Boys	1982	14	1.33
Latin America and Caribbean	Boys	1983	14	1.36
Latin America and Caribbean	Boys	1984	14	1.40
Latin America and Caribbean	Boys	1985	14	1.43
Latin America and Caribbean	Boys	1986	14	1.47
Latin America and Caribbean	Boys	1987	14	1.50
Latin America and Caribbean	Boys	1988	14	1.54
Latin America and Caribbean	Boys	1989	14	1.58
Latin America and Caribbean	Boys	1990	14	1.61
Latin America and Caribbean	Boys	1991	14	1.65
Latin America and Caribbean	Boys	1992	14	1.69
Latin America and Caribbean	Boys	1993	14	1.73
Latin America and Caribbean	Boys	1994	14	1.77
Latin America and Caribbean	Boys	1995	14	1.81
Latin America and Caribbean	Boys	1996	14	1.85
Latin America and Caribbean	Boys	1997	14	1.90
Latin America and Caribbean	Boys	1998	14	1.94
Latin America and Caribbean	Boys	1999	14	1.99
Latin America and Caribbean	Boys	2000	14	2.04
Latin America and Caribbean	Boys	2001	14	2.09
Latin America and Caribbean	Boys	2002	14	2.14
Latin America and Caribbean	Boys	2003	14	2.19
Latin America and Caribbean	Boys	2004	14	2.24
Latin America and Caribbean	Boys	2005	14	2.29
Latin America and Caribbean	Boys	2006	14	2.34
Latin America and Caribbean	Boys	2007	14	2.39
Latin America and Caribbean	Boys	2008	14	2.44
Latin America and Caribbean	Boys	2009	14	2.49
Latin America and Caribbean	Boys	2010	14	2.55
Latin America and Caribbean	Boys	2011	14	2.60
Latin America and Caribbean	Boys	2012	14	2.66
Latin America and Caribbean	Boys	2013	14	2.71
Latin America and Caribbean	Boys	2014	14	2.77
Latin America and Caribbean	Boys	2015	14	2.83
Latin America and Caribbean	Boys	2016	14	2.89
Latin America and Caribbean	Boys	1975	15	1.48
Latin America and Caribbean	Boys	1976	15	1.50
Latin America and Caribbean	Boys	1977	15	1.53
Latin America and Caribbean	Boys	1978	15	1.56
Latin America and Caribbean	Boys	1979	15	1.59
Latin America and Caribbean	Boys	1980	15	1.63
Latin America and Caribbean	Boys	1981	15	1.66
Latin America and Caribbean	Boys	1982	15	1.70
Latin America and Caribbean	Boys	1983	15	1.74
Latin America and Caribbean	Boys	1984	15	1.78
Latin America and Caribbean	Boys	1985	15	1.82
Latin America and Caribbean	Boys	1986	15	1.87
Latin America and Caribbean	Boys	1987	15	1.91

Latin America and Caribbean	Boys	1988	15	1.96
Latin America and Caribbean	Boys	1989	15	2.01
Latin America and Caribbean	Boys	1990	15	2.06
Latin America and Caribbean	Boys	1991	15	2.11
Latin America and Caribbean	Boys	1992	15	2.16
Latin America and Caribbean	Boys	1993	15	2.21
Latin America and Caribbean	Boys	1994	15	2.26
Latin America and Caribbean	Boys	1995	15	2.31
Latin America and Caribbean	Boys	1996	15	2.36
Latin America and Caribbean	Boys	1997	15	2.41
Latin America and Caribbean	Boys	1998	15	2.46
Latin America and Caribbean	Boys	1999	15	2.52
Latin America and Caribbean	Boys	2000	15	2.57
Latin America and Caribbean	Boys	2001	15	2.63
Latin America and Caribbean	Boys	2002	15	2.69
Latin America and Caribbean	Boys	2003	15	2.76
Latin America and Caribbean	Boys	2004	15	2.82
Latin America and Caribbean	Boys	2005	15	2.88
Latin America and Caribbean	Boys	2006	15	2.95
Latin America and Caribbean	Boys	2007	15	3.02
Latin America and Caribbean	Boys	2008	15	3.09
Latin America and Caribbean	Boys	2009	15	3.15
Latin America and Caribbean	Boys	2010	15	3.22
Latin America and Caribbean	Boys	2011	15	3.30
Latin America and Caribbean	Boys	2012	15	3.37
Latin America and Caribbean	Boys	2013	15	3.44
Latin America and Caribbean	Boys	2014	15	3.51
Latin America and Caribbean	Boys	2015	15	3.59
Latin America and Caribbean	Boys	2016	15	3.67
Latin America and Caribbean	Boys	1975	16	1.85
Latin America and Caribbean	Boys	1976	16	1.88
Latin America and Caribbean	Boys	1977	16	1.91
Latin America and Caribbean	Boys	1978	16	1.95
Latin America and Caribbean	Boys	1979	16	1.98
Latin America and Caribbean	Boys	1980	16	2.02
Latin America and Caribbean	Boys	1981	16	2.07
Latin America and Caribbean	Boys	1982	16	2.11
Latin America and Caribbean	Boys	1983	16	2.15
Latin America and Caribbean	Boys	1984	16	2.20
Latin America and Caribbean	Boys	1985	16	2.25
Latin America and Caribbean	Boys	1986	16	2.31
Latin America and Caribbean	Boys	1987	16	2.36
Latin America and Caribbean	Boys	1988	16	2.42
Latin America and Caribbean	Boys	1989	16	2.48
Latin America and Caribbean	Boys	1990	16	2.53
Latin America and Caribbean	Boys	1991	16	2.59
Latin America and Caribbean	Boys	1992	16	2.65
Latin America and Caribbean	Boys	1993	16	2.71
Latin America and Caribbean	Boys	1994	16	2.77
Latin America and Caribbean	Boys	1995	16	2.82
Latin America and Caribbean	Boys	1996	16	2.88
Latin America and Caribbean	Boys	1997	16	2.94

Latin America and Caribbean	Boys	1998	16	3.01
Latin America and Caribbean	Boys	1999	16	3.07
Latin America and Caribbean	Boys	2000	16	3.14
Latin America and Caribbean	Boys	2001	16	3.21
Latin America and Caribbean	Boys	2002	16	3.28
Latin America and Caribbean	Boys	2003	16	3.36
Latin America and Caribbean	Boys	2004	16	3.43
Latin America and Caribbean	Boys	2005	16	3.51
Latin America and Caribbean	Boys	2006	16	3.59
Latin America and Caribbean	Boys	2007	16	3.67
Latin America and Caribbean	Boys	2008	16	3.75
Latin America and Caribbean	Boys	2009	16	3.83
Latin America and Caribbean	Boys	2010	16	3.91
Latin America and Caribbean	Boys	2011	16	4.00
Latin America and Caribbean	Boys	2012	16	4.09
Latin America and Caribbean	Boys	2013	16	4.17
Latin America and Caribbean	Boys	2014	16	4.26
Latin America and Caribbean	Boys	2015	16	4.35
Latin America and Caribbean	Boys	2016	16	4.45
Latin America and Caribbean	Boys	1975	17	2.26
Latin America and Caribbean	Boys	1976	17	2.30
Latin America and Caribbean	Boys	1977	17	2.33
Latin America and Caribbean	Boys	1978	17	2.37
Latin America and Caribbean	Boys	1979	17	2.42
Latin America and Caribbean	Boys	1980	17	2.46
Latin America and Caribbean	Boys	1981	17	2.51
Latin America and Caribbean	Boys	1982	17	2.56
Latin America and Caribbean	Boys	1983	17	2.61
Latin America and Caribbean	Boys	1984	17	2.67
Latin America and Caribbean	Boys	1985	17	2.72
Latin America and Caribbean	Boys	1986	17	2.79
Latin America and Caribbean	Boys	1987	17	2.85
Latin America and Caribbean	Boys	1988	17	2.91
Latin America and Caribbean	Boys	1989	17	2.98
Latin America and Caribbean	Boys	1990	17	3.05
Latin America and Caribbean	Boys	1991	17	3.11
Latin America and Caribbean	Boys	1992	17	3.18
Latin America and Caribbean	Boys	1993	17	3.24
Latin America and Caribbean	Boys	1994	17	3.31
Latin America and Caribbean	Boys	1995	17	3.38
Latin America and Caribbean	Boys	1996	17	3.45
Latin America and Caribbean	Boys	1997	17	3.52
Latin America and Caribbean	Boys	1998	17	3.59
Latin America and Caribbean	Boys	1999	17	3.66
Latin America and Caribbean	Boys	2000	17	3.74
Latin America and Caribbean	Boys	2001	17	3.82
Latin America and Caribbean	Boys	2002	17	3.90
Latin America and Caribbean	Boys	2003	17	3.99
Latin America and Caribbean	Boys	2004	17	4.08
Latin America and Caribbean	Boys	2005	17	4.17
Latin America and Caribbean	Boys	2006	17	4.26
Latin America and Caribbean	Boys	2007	17	4.35

Latin America and Caribbean	Boys	2008	17	4.44
Latin America and Caribbean	Boys	2009	17	4.54
Latin America and Caribbean	Boys	2010	17	4.63
Latin America and Caribbean	Boys	2011	17	4.73
Latin America and Caribbean	Boys	2012	17	4.83
Latin America and Caribbean	Boys	2013	17	4.93
Latin America and Caribbean	Boys	2014	17	5.03
Latin America and Caribbean	Boys	2015	17	5.14
Latin America and Caribbean	Boys	2016	17	5.25
Latin America and Caribbean	Boys	1975	18	2.71
Latin America and Caribbean	Boys	1976	18	2.76
Latin America and Caribbean	Boys	1977	18	2.80
Latin America and Caribbean	Boys	1978	18	2.85
Latin America and Caribbean	Boys	1979	18	2.89
Latin America and Caribbean	Boys	1980	18	2.95
Latin America and Caribbean	Boys	1981	18	3.00
Latin America and Caribbean	Boys	1982	18	3.06
Latin America and Caribbean	Boys	1983	18	3.12
Latin America and Caribbean	Boys	1984	18	3.18
Latin America and Caribbean	Boys	1985	18	3.25
Latin America and Caribbean	Boys	1986	18	3.32
Latin America and Caribbean	Boys	1987	18	3.39
Latin America and Caribbean	Boys	1988	18	3.47
Latin America and Caribbean	Boys	1989	18	3.55
Latin America and Caribbean	Boys	1990	18	3.62
Latin America and Caribbean	Boys	1991	18	3.70
Latin America and Caribbean	Boys	1992	18	3.77
Latin America and Caribbean	Boys	1993	18	3.85
Latin America and Caribbean	Boys	1994	18	3.92
Latin America and Caribbean	Boys	1995	18	4.00
Latin America and Caribbean	Boys	1996	18	4.08
Latin America and Caribbean	Boys	1997	18	4.16
Latin America and Caribbean	Boys	1998	18	4.24
Latin America and Caribbean	Boys	1999	18	4.32
Latin America and Caribbean	Boys	2000	18	4.41
Latin America and Caribbean	Boys	2001	18	4.50
Latin America and Caribbean	Boys	2002	18	4.60
Latin America and Caribbean	Boys	2003	18	4.70
Latin America and Caribbean	Boys	2004	18	4.80
Latin America and Caribbean	Boys	2005	18	4.90
Latin America and Caribbean	Boys	2006	18	5.00
Latin America and Caribbean	Boys	2007	18	5.11
Latin America and Caribbean	Boys	2008	18	5.22
Latin America and Caribbean	Boys	2009	18	5.32
Latin America and Caribbean	Boys	2010	18	5.43
Latin America and Caribbean	Boys	2011	18	5.55
Latin America and Caribbean	Boys	2012	18	5.66
Latin America and Caribbean	Boys	2013	18	5.77
Latin America and Caribbean	Boys	2014	18	5.89
Latin America and Caribbean	Boys	2015	18	6.01
Latin America and Caribbean	Boys	2016	18	6.14
Latin America and Caribbean	Boys	1975	Average	1.56

Latin America and Caribbean	Boys	1976	Average	1.59
Latin America and Caribbean	Boys	1977	Average	1.62
Latin America and Caribbean	Boys	1978	Average	1.65
Latin America and Caribbean	Boys	1979	Average	1.68
Latin America and Caribbean	Boys	1980	Average	1.72
Latin America and Caribbean	Boys	1981	Average	1.75
Latin America and Caribbean	Boys	1982	Average	1.79
Latin America and Caribbean	Boys	1983	Average	1.83
Latin America and Caribbean	Boys	1984	Average	1.87
Latin America and Caribbean	Boys	1985	Average	1.91
Latin America and Caribbean	Boys	1986	Average	1.96
Latin America and Caribbean	Boys	1987	Average	2.00
Latin America and Caribbean	Boys	1988	Average	2.05
Latin America and Caribbean	Boys	1989	Average	2.10
Latin America and Caribbean	Boys	1990	Average	2.15
Latin America and Caribbean	Boys	1991	Average	2.19
Latin America and Caribbean	Boys	1992	Average	2.24
Latin America and Caribbean	Boys	1993	Average	2.29
Latin America and Caribbean	Boys	1994	Average	2.34
Latin America and Caribbean	Boys	1995	Average	2.39
Latin America and Caribbean	Boys	1996	Average	2.44
Latin America and Caribbean	Boys	1997	Average	2.49
Latin America and Caribbean	Boys	1998	Average	2.54
Latin America and Caribbean	Boys	1999	Average	2.60
Latin America and Caribbean	Boys	2000	Average	2.66
Latin America and Caribbean	Boys	2001	Average	2.72
Latin America and Caribbean	Boys	2002	Average	2.78
Latin America and Caribbean	Boys	2003	Average	2.84
Latin America and Caribbean	Boys	2004	Average	2.90
Latin America and Caribbean	Boys	2005	Average	2.97
Latin America and Caribbean	Boys	2006	Average	3.03
Latin America and Caribbean	Boys	2007	Average	3.10
Latin America and Caribbean	Boys	2008	Average	3.16
Latin America and Caribbean	Boys	2009	Average	3.23
Latin America and Caribbean	Boys	2010	Average	3.30
Latin America and Caribbean	Boys	2011	Average	3.37
Latin America and Caribbean	Boys	2012	Average	3.44
Latin America and Caribbean	Boys	2013	Average	3.51
Latin America and Caribbean	Boys	2014	Average	3.58
Latin America and Caribbean	Boys	2015	Average	3.66
Latin America and Caribbean	Boys	2016	Average	3.74
Latin America and Caribbean	Boys	2017	Average	3.82
Latin America and Caribbean	Boys	2018	Average	3.90
Latin America and Caribbean	Boys	2019	Average	3.98
Latin America and Caribbean	Boys	2020	Average	4.06
Latin America and Caribbean	Boys	2021	Average	4.14
Latin America and Caribbean	Boys	2022	Average	4.23
Latin America and Caribbean	Boys	2023	Average	4.31
Latin America and Caribbean	Boys	2024	Average	4.39
Latin America and Caribbean	Boys	2025	Average	4.48
Latin America and Caribbean	Boys	2026	Average	4.56
Latin America and Caribbean	Boys	2027	Average	4.65

Latin America and Caribbean	Boys	2028	Average	4.73
Latin America and Caribbean	Boys	2029	Average	4.82
Latin America and Caribbean	Boys	2030	Average	4.90
Latin America and Caribbean	Girls	1975	12	0.79
Latin America and Caribbean	Girls	1976	12	0.81
Latin America and Caribbean	Girls	1977	12	0.83
Latin America and Caribbean	Girls	1978	12	0.85
Latin America and Caribbean	Girls	1979	12	0.87
Latin America and Caribbean	Girls	1980	12	0.89
Latin America and Caribbean	Girls	1981	12	0.91
Latin America and Caribbean	Girls	1982	12	0.94
Latin America and Caribbean	Girls	1983	12	0.96
Latin America and Caribbean	Girls	1984	12	0.98
Latin America and Caribbean	Girls	1985	12	1.01
Latin America and Caribbean	Girls	1986	12	1.03
Latin America and Caribbean	Girls	1987	12	1.06
Latin America and Caribbean	Girls	1988	12	1.08
Latin America and Caribbean	Girls	1989	12	1.11
Latin America and Caribbean	Girls	1990	12	1.13
Latin America and Caribbean	Girls	1991	12	1.16
Latin America and Caribbean	Girls	1992	12	1.18
Latin America and Caribbean	Girls	1993	12	1.21
Latin America and Caribbean	Girls	1994	12	1.23
Latin America and Caribbean	Girls	1995	12	1.26
Latin America and Caribbean	Girls	1996	12	1.29
Latin America and Caribbean	Girls	1997	12	1.32
Latin America and Caribbean	Girls	1998	12	1.35
Latin America and Caribbean	Girls	1999	12	1.37
Latin America and Caribbean	Girls	2000	12	1.40
Latin America and Caribbean	Girls	2001	12	1.43
Latin America and Caribbean	Girls	2002	12	1.46
Latin America and Caribbean	Girls	2003	12	1.49
Latin America and Caribbean	Girls	2004	12	1.51
Latin America and Caribbean	Girls	2005	12	1.54
Latin America and Caribbean	Girls	2006	12	1.57
Latin America and Caribbean	Girls	2007	12	1.59
Latin America and Caribbean	Girls	2008	12	1.62
Latin America and Caribbean	Girls	2009	12	1.65
Latin America and Caribbean	Girls	2010	12	1.68
Latin America and Caribbean	Girls	2011	12	1.70
Latin America and Caribbean	Girls	2012	12	1.73
Latin America and Caribbean	Girls	2013	12	1.76
Latin America and Caribbean	Girls	2014	12	1.79
Latin America and Caribbean	Girls	2015	12	1.82
Latin America and Caribbean	Girls	2016	12	1.85
Latin America and Caribbean	Girls	1975	13	1.11
Latin America and Caribbean	Girls	1976	13	1.14
Latin America and Caribbean	Girls	1977	13	1.17
Latin America and Caribbean	Girls	1978	13	1.19
Latin America and Caribbean	Girls	1979	13	1.22
Latin America and Caribbean	Girls	1980	13	1.25
Latin America and Caribbean	Girls	1981	13	1.28

Latin America and Caribbean	Girls	1982	13	1.32
Latin America and Caribbean	Girls	1983	13	1.35
Latin America and Caribbean	Girls	1984	13	1.38
Latin America and Caribbean	Girls	1985	13	1.42
Latin America and Caribbean	Girls	1986	13	1.45
Latin America and Caribbean	Girls	1987	13	1.48
Latin America and Caribbean	Girls	1988	13	1.52
Latin America and Caribbean	Girls	1989	13	1.55
Latin America and Caribbean	Girls	1990	13	1.59
Latin America and Caribbean	Girls	1991	13	1.62
Latin America and Caribbean	Girls	1992	13	1.66
Latin America and Caribbean	Girls	1993	13	1.69
Latin America and Caribbean	Girls	1994	13	1.73
Latin America and Caribbean	Girls	1995	13	1.76
Latin America and Caribbean	Girls	1996	13	1.80
Latin America and Caribbean	Girls	1997	13	1.84
Latin America and Caribbean	Girls	1998	13	1.88
Latin America and Caribbean	Girls	1999	13	1.92
Latin America and Caribbean	Girls	2000	13	1.96
Latin America and Caribbean	Girls	2001	13	2.00
Latin America and Caribbean	Girls	2002	13	2.03
Latin America and Caribbean	Girls	2003	13	2.07
Latin America and Caribbean	Girls	2004	13	2.11
Latin America and Caribbean	Girls	2005	13	2.15
Latin America and Caribbean	Girls	2006	13	2.18
Latin America and Caribbean	Girls	2007	13	2.22
Latin America and Caribbean	Girls	2008	13	2.26
Latin America and Caribbean	Girls	2009	13	2.29
Latin America and Caribbean	Girls	2010	13	2.33
Latin America and Caribbean	Girls	2011	13	2.37
Latin America and Caribbean	Girls	2012	13	2.41
Latin America and Caribbean	Girls	2013	13	2.45
Latin America and Caribbean	Girls	2014	13	2.50
Latin America and Caribbean	Girls	2015	13	2.54
Latin America and Caribbean	Girls	2016	13	2.58
Latin America and Caribbean	Girls	1975	14	1.48
Latin America and Caribbean	Girls	1976	14	1.52
Latin America and Caribbean	Girls	1977	14	1.55
Latin America and Caribbean	Girls	1978	14	1.59
Latin America and Caribbean	Girls	1979	14	1.62
Latin America and Caribbean	Girls	1980	14	1.66
Latin America and Caribbean	Girls	1981	14	1.70
Latin America and Caribbean	Girls	1982	14	1.74
Latin America and Caribbean	Girls	1983	14	1.78
Latin America and Caribbean	Girls	1984	14	1.83
Latin America and Caribbean	Girls	1985	14	1.87
Latin America and Caribbean	Girls	1986	14	1.91
Latin America and Caribbean	Girls	1987	14	1.95
Latin America and Caribbean	Girls	1988	14	2.00
Latin America and Caribbean	Girls	1989	14	2.04
Latin America and Caribbean	Girls	1990	14	2.08
Latin America and Caribbean	Girls	1991	14	2.13

Latin America and Caribbean	Girls	1992	14	2.17
Latin America and Caribbean	Girls	1993	14	2.21
Latin America and Caribbean	Girls	1994	14	2.26
Latin America and Caribbean	Girls	1995	14	2.30
Latin America and Caribbean	Girls	1996	14	2.35
Latin America and Caribbean	Girls	1997	14	2.40
Latin America and Caribbean	Girls	1998	14	2.45
Latin America and Caribbean	Girls	1999	14	2.50
Latin America and Caribbean	Girls	2000	14	2.55
Latin America and Caribbean	Girls	2001	14	2.60
Latin America and Caribbean	Girls	2002	14	2.65
Latin America and Caribbean	Girls	2003	14	2.69
Latin America and Caribbean	Girls	2004	14	2.74
Latin America and Caribbean	Girls	2005	14	2.79
Latin America and Caribbean	Girls	2006	14	2.83
Latin America and Caribbean	Girls	2007	14	2.88
Latin America and Caribbean	Girls	2008	14	2.93
Latin America and Caribbean	Girls	2009	14	2.97
Latin America and Caribbean	Girls	2010	14	3.02
Latin America and Caribbean	Girls	2011	14	3.07
Latin America and Caribbean	Girls	2012	14	3.12
Latin America and Caribbean	Girls	2013	14	3.17
Latin America and Caribbean	Girls	2014	14	3.23
Latin America and Caribbean	Girls	2015	14	3.28
Latin America and Caribbean	Girls	2016	14	3.34
Latin America and Caribbean	Girls	1975	15	1.84
Latin America and Caribbean	Girls	1976	15	1.88
Latin America and Caribbean	Girls	1977	15	1.92
Latin America and Caribbean	Girls	1978	15	1.96
Latin America and Caribbean	Girls	1979	15	2.00
Latin America and Caribbean	Girls	1980	15	2.05
Latin America and Caribbean	Girls	1981	15	2.09
Latin America and Caribbean	Girls	1982	15	2.14
Latin America and Caribbean	Girls	1983	15	2.18
Latin America and Caribbean	Girls	1984	15	2.23
Latin America and Caribbean	Girls	1985	15	2.28
Latin America and Caribbean	Girls	1986	15	2.34
Latin America and Caribbean	Girls	1987	15	2.39
Latin America and Caribbean	Girls	1988	15	2.44
Latin America and Caribbean	Girls	1989	15	2.50
Latin America and Caribbean	Girls	1990	15	2.55
Latin America and Caribbean	Girls	1991	15	2.60
Latin America and Caribbean	Girls	1992	15	2.66
Latin America and Caribbean	Girls	1993	15	2.71
Latin America and Caribbean	Girls	1994	15	2.76
Latin America and Caribbean	Girls	1995	15	2.81
Latin America and Caribbean	Girls	1996	15	2.86
Latin America and Caribbean	Girls	1997	15	2.91
Latin America and Caribbean	Girls	1998	15	2.96
Latin America and Caribbean	Girls	1999	15	3.02
Latin America and Caribbean	Girls	2000	15	3.07
Latin America and Caribbean	Girls	2001	15	3.13

Latin America and Caribbean	Girls	2002	15	3.18
Latin America and Caribbean	Girls	2003	15	3.24
Latin America and Caribbean	Girls	2004	15	3.29
Latin America and Caribbean	Girls	2005	15	3.35
Latin America and Caribbean	Girls	2006	15	3.41
Latin America and Caribbean	Girls	2007	15	3.46
Latin America and Caribbean	Girls	2008	15	3.52
Latin America and Caribbean	Girls	2009	15	3.58
Latin America and Caribbean	Girls	2010	15	3.64
Latin America and Caribbean	Girls	2011	15	3.70
Latin America and Caribbean	Girls	2012	15	3.76
Latin America and Caribbean	Girls	2013	15	3.82
Latin America and Caribbean	Girls	2014	15	3.88
Latin America and Caribbean	Girls	2015	15	3.94
Latin America and Caribbean	Girls	2016	15	4.00
Latin America and Caribbean	Girls	1975	16	2.14
Latin America and Caribbean	Girls	1976	16	2.18
Latin America and Caribbean	Girls	1977	16	2.22
Latin America and Caribbean	Girls	1978	16	2.27
Latin America and Caribbean	Girls	1979	16	2.32
Latin America and Caribbean	Girls	1980	16	2.36
Latin America and Caribbean	Girls	1981	16	2.41
Latin America and Caribbean	Girls	1982	16	2.47
Latin America and Caribbean	Girls	1983	16	2.52
Latin America and Caribbean	Girls	1984	16	2.57
Latin America and Caribbean	Girls	1985	16	2.63
Latin America and Caribbean	Girls	1986	16	2.69
Latin America and Caribbean	Girls	1987	16	2.75
Latin America and Caribbean	Girls	1988	16	2.81
Latin America and Caribbean	Girls	1989	16	2.87
Latin America and Caribbean	Girls	1990	16	2.93
Latin America and Caribbean	Girls	1991	16	2.99
Latin America and Caribbean	Girls	1992	16	3.04
Latin America and Caribbean	Girls	1993	16	3.10
Latin America and Caribbean	Girls	1994	16	3.16
Latin America and Caribbean	Girls	1995	16	3.22
Latin America and Caribbean	Girls	1996	16	3.27
Latin America and Caribbean	Girls	1997	16	3.33
Latin America and Caribbean	Girls	1998	16	3.39
Latin America and Caribbean	Girls	1999	16	3.45
Latin America and Caribbean	Girls	2000	16	3.50
Latin America and Caribbean	Girls	2001	16	3.57
Latin America and Caribbean	Girls	2002	16	3.63
Latin America and Caribbean	Girls	2003	16	3.69
Latin America and Caribbean	Girls	2004	16	3.75
Latin America and Caribbean	Girls	2005	16	3.82
Latin America and Caribbean	Girls	2006	16	3.88
Latin America and Caribbean	Girls	2007	16	3.94
Latin America and Caribbean	Girls	2008	16	4.01
Latin America and Caribbean	Girls	2009	16	4.07
Latin America and Caribbean	Girls	2010	16	4.13
Latin America and Caribbean	Girls	2011	16	4.20

Latin America and Caribbean	Girls	2012	16	4.26
Latin America and Caribbean	Girls	2013	16	4.33
Latin America and Caribbean	Girls	2014	16	4.40
Latin America and Caribbean	Girls	2015	16	4.47
Latin America and Caribbean	Girls	2016	16	4.54
Latin America and Caribbean	Girls	1975	17	2.37
Latin America and Caribbean	Girls	1976	17	2.42
Latin America and Caribbean	Girls	1977	17	2.47
Latin America and Caribbean	Girls	1978	17	2.52
Latin America and Caribbean	Girls	1979	17	2.57
Latin America and Caribbean	Girls	1980	17	2.62
Latin America and Caribbean	Girls	1981	17	2.68
Latin America and Caribbean	Girls	1982	17	2.73
Latin America and Caribbean	Girls	1983	17	2.79
Latin America and Caribbean	Girls	1984	17	2.85
Latin America and Caribbean	Girls	1985	17	2.92
Latin America and Caribbean	Girls	1986	17	2.98
Latin America and Caribbean	Girls	1987	17	3.05
Latin America and Caribbean	Girls	1988	17	3.11
Latin America and Caribbean	Girls	1989	17	3.18
Latin America and Caribbean	Girls	1990	17	3.24
Latin America and Caribbean	Girls	1991	17	3.31
Latin America and Caribbean	Girls	1992	17	3.37
Latin America and Caribbean	Girls	1993	17	3.43
Latin America and Caribbean	Girls	1994	17	3.50
Latin America and Caribbean	Girls	1995	17	3.56
Latin America and Caribbean	Girls	1996	17	3.62
Latin America and Caribbean	Girls	1997	17	3.68
Latin America and Caribbean	Girls	1998	17	3.75
Latin America and Caribbean	Girls	1999	17	3.81
Latin America and Caribbean	Girls	2000	17	3.88
Latin America and Caribbean	Girls	2001	17	3.94
Latin America and Caribbean	Girls	2002	17	4.01
Latin America and Caribbean	Girls	2003	17	4.08
Latin America and Caribbean	Girls	2004	17	4.15
Latin America and Caribbean	Girls	2005	17	4.22
Latin America and Caribbean	Girls	2006	17	4.29
Latin America and Caribbean	Girls	2007	17	4.36
Latin America and Caribbean	Girls	2008	17	4.43
Latin America and Caribbean	Girls	2009	17	4.50
Latin America and Caribbean	Girls	2010	17	4.57
Latin America and Caribbean	Girls	2011	17	4.64
Latin America and Caribbean	Girls	2012	17	4.71
Latin America and Caribbean	Girls	2013	17	4.78
Latin America and Caribbean	Girls	2014	17	4.86
Latin America and Caribbean	Girls	2015	17	4.93
Latin America and Caribbean	Girls	2016	17	5.01
Latin America and Caribbean	Girls	1975	18	2.60
Latin America and Caribbean	Girls	1976	18	2.65
Latin America and Caribbean	Girls	1977	18	2.70
Latin America and Caribbean	Girls	1978	18	2.76
Latin America and Caribbean	Girls	1979	18	2.82

Latin America and Caribbean	Girls	1980	18	2.88
Latin America and Caribbean	Girls	1981	18	2.94
Latin America and Caribbean	Girls	1982	18	3.00
Latin America and Caribbean	Girls	1983	18	3.07
Latin America and Caribbean	Girls	1984	18	3.14
Latin America and Caribbean	Girls	1985	18	3.21
Latin America and Caribbean	Girls	1986	18	3.28
Latin America and Caribbean	Girls	1987	18	3.36
Latin America and Caribbean	Girls	1988	18	3.43
Latin America and Caribbean	Girls	1989	18	3.51
Latin America and Caribbean	Girls	1990	18	3.58
Latin America and Caribbean	Girls	1991	18	3.65
Latin America and Caribbean	Girls	1992	18	3.72
Latin America and Caribbean	Girls	1993	18	3.79
Latin America and Caribbean	Girls	1994	18	3.86
Latin America and Caribbean	Girls	1995	18	3.93
Latin America and Caribbean	Girls	1996	18	4.00
Latin America and Caribbean	Girls	1997	18	4.08
Latin America and Caribbean	Girls	1998	18	4.15
Latin America and Caribbean	Girls	1999	18	4.22
Latin America and Caribbean	Girls	2000	18	4.29
Latin America and Caribbean	Girls	2001	18	4.37
Latin America and Caribbean	Girls	2002	18	4.45
Latin America and Caribbean	Girls	2003	18	4.52
Latin America and Caribbean	Girls	2004	18	4.60
Latin America and Caribbean	Girls	2005	18	4.68
Latin America and Caribbean	Girls	2006	18	4.76
Latin America and Caribbean	Girls	2007	18	4.83
Latin America and Caribbean	Girls	2008	18	4.91
Latin America and Caribbean	Girls	2009	18	4.99
Latin America and Caribbean	Girls	2010	18	5.07
Latin America and Caribbean	Girls	2011	18	5.15
Latin America and Caribbean	Girls	2012	18	5.23
Latin America and Caribbean	Girls	2013	18	5.31
Latin America and Caribbean	Girls	2014	18	5.39
Latin America and Caribbean	Girls	2015	18	5.47
Latin America and Caribbean	Girls	2016	18	5.56
Latin America and Caribbean	Girls	1975	Average	1.76
Latin America and Caribbean	Girls	1976	Average	1.80
Latin America and Caribbean	Girls	1977	Average	1.84
Latin America and Caribbean	Girls	1978	Average	1.88
Latin America and Caribbean	Girls	1979	Average	1.92
Latin America and Caribbean	Girls	1980	Average	1.96
Latin America and Caribbean	Girls	1981	Average	2.00
Latin America and Caribbean	Girls	1982	Average	2.05
Latin America and Caribbean	Girls	1983	Average	2.09
Latin America and Caribbean	Girls	1984	Average	2.14
Latin America and Caribbean	Girls	1985	Average	2.19
Latin America and Caribbean	Girls	1986	Average	2.24
Latin America and Caribbean	Girls	1987	Average	2.29
Latin America and Caribbean	Girls	1988	Average	2.34
Latin America and Caribbean	Girls	1989	Average	2.39

Latin America and Caribbean	Girls	1990	Average	2.44
Latin America and Caribbean	Girls	1991	Average	2.49
Latin America and Caribbean	Girls	1992	Average	2.54
Latin America and Caribbean	Girls	1993	Average	2.59
Latin America and Caribbean	Girls	1994	Average	2.64
Latin America and Caribbean	Girls	1995	Average	2.69
Latin America and Caribbean	Girls	1996	Average	2.74
Latin America and Caribbean	Girls	1997	Average	2.79
Latin America and Caribbean	Girls	1998	Average	2.85
Latin America and Caribbean	Girls	1999	Average	2.90
Latin America and Caribbean	Girls	2000	Average	2.95
Latin America and Caribbean	Girls	2001	Average	3.00
Latin America and Caribbean	Girls	2002	Average	3.06
Latin America and Caribbean	Girls	2003	Average	3.11
Latin America and Caribbean	Girls	2004	Average	3.17
Latin America and Caribbean	Girls	2005	Average	3.22
Latin America and Caribbean	Girls	2006	Average	3.27
Latin America and Caribbean	Girls	2007	Average	3.33
Latin America and Caribbean	Girls	2008	Average	3.38
Latin America and Caribbean	Girls	2009	Average	3.44
Latin America and Caribbean	Girls	2010	Average	3.49
Latin America and Caribbean	Girls	2011	Average	3.55
Latin America and Caribbean	Girls	2012	Average	3.60
Latin America and Caribbean	Girls	2013	Average	3.66
Latin America and Caribbean	Girls	2014	Average	3.72
Latin America and Caribbean	Girls	2015	Average	3.78
Latin America and Caribbean	Girls	2016	Average	3.84
Latin America and Caribbean	Girls	2017	Average	3.90
Latin America and Caribbean	Girls	2018	Average	3.97
Latin America and Caribbean	Girls	2019	Average	4.03
Latin America and Caribbean	Girls	2020	Average	4.09
Latin America and Caribbean	Girls	2021	Average	4.16
Latin America and Caribbean	Girls	2022	Average	4.23
Latin America and Caribbean	Girls	2023	Average	4.29
Latin America and Caribbean	Girls	2024	Average	4.36
Latin America and Caribbean	Girls	2025	Average	4.42
Latin America and Caribbean	Girls	2026	Average	4.49
Latin America and Caribbean	Girls	2027	Average	4.56
Latin America and Caribbean	Girls	2028	Average	4.62
Latin America and Caribbean	Girls	2029	Average	4.69
Latin America and Caribbean	Girls	2030	Average	4.76
Oceania	Boys	1975	12	0.54
Oceania	Boys	1976	12	0.54
Oceania	Boys	1977	12	0.55
Oceania	Boys	1978	12	0.56
Oceania	Boys	1979	12	0.57
Oceania	Boys	1980	12	0.58
Oceania	Boys	1981	12	0.58
Oceania	Boys	1982	12	0.59
Oceania	Boys	1983	12	0.60
Oceania	Boys	1984	12	0.61
Oceania	Boys	1985	12	0.62

Oceania	Boys	1986	12	0.64
Oceania	Boys	1987	12	0.65
Oceania	Boys	1988	12	0.67
Oceania	Boys	1989	12	0.68
Oceania	Boys	1990	12	0.70
Oceania	Boys	1991	12	0.72
Oceania	Boys	1992	12	0.73
Oceania	Boys	1993	12	0.75
Oceania	Boys	1994	12	0.77
Oceania	Boys	1995	12	0.79
Oceania	Boys	1996	12	0.81
Oceania	Boys	1997	12	0.83
Oceania	Boys	1998	12	0.86
Oceania	Boys	1999	12	0.88
Oceania	Boys	2000	12	0.90
Oceania	Boys	2001	12	0.92
Oceania	Boys	2002	12	0.95
Oceania	Boys	2003	12	0.97
Oceania	Boys	2004	12	1.00
Oceania	Boys	2005	12	1.03
Oceania	Boys	2006	12	1.05
Oceania	Boys	2007	12	1.08
Oceania	Boys	2008	12	1.11
Oceania	Boys	2009	12	1.14
Oceania	Boys	2010	12	1.18
Oceania	Boys	2011	12	1.21
Oceania	Boys	2012	12	1.25
Oceania	Boys	2013	12	1.28
Oceania	Boys	2014	12	1.32
Oceania	Boys	2015	12	1.36
Oceania	Boys	2016	12	1.40
Oceania	Boys	1975	13	0.72
Oceania	Boys	1976	13	0.73
Oceania	Boys	1977	13	0.74
Oceania	Boys	1978	13	0.75
Oceania	Boys	1979	13	0.76
Oceania	Boys	1980	13	0.77
Oceania	Boys	1981	13	0.78
Oceania	Boys	1982	13	0.79
Oceania	Boys	1983	13	0.81
Oceania	Boys	1984	13	0.82
Oceania	Boys	1985	13	0.83
Oceania	Boys	1986	13	0.85
Oceania	Boys	1987	13	0.87
Oceania	Boys	1988	13	0.89
Oceania	Boys	1989	13	0.91
Oceania	Boys	1990	13	0.93
Oceania	Boys	1991	13	0.96
Oceania	Boys	1992	13	0.98
Oceania	Boys	1993	13	1.01
Oceania	Boys	1994	13	1.04
Oceania	Boys	1995	13	1.06

Oceania	Boys	1996	13	1.09
Oceania	Boys	1997	13	1.12
Oceania	Boys	1998	13	1.15
Oceania	Boys	1999	13	1.18
Oceania	Boys	2000	13	1.21
Oceania	Boys	2001	13	1.24
Oceania	Boys	2002	13	1.28
Oceania	Boys	2003	13	1.31
Oceania	Boys	2004	13	1.35
Oceania	Boys	2005	13	1.39
Oceania	Boys	2006	13	1.43
Oceania	Boys	2007	13	1.47
Oceania	Boys	2008	13	1.51
Oceania	Boys	2009	13	1.55
Oceania	Boys	2010	13	1.60
Oceania	Boys	2011	13	1.65
Oceania	Boys	2012	13	1.70
Oceania	Boys	2013	13	1.75
Oceania	Boys	2014	13	1.81
Oceania	Boys	2015	13	1.86
Oceania	Boys	2016	13	1.92
Oceania	Boys	1975	14	0.96
Oceania	Boys	1976	14	0.97
Oceania	Boys	1977	14	0.98
Oceania	Boys	1978	14	1.00
Oceania	Boys	1979	14	1.01
Oceania	Boys	1980	14	1.03
Oceania	Boys	1981	14	1.04
Oceania	Boys	1982	14	1.06
Oceania	Boys	1983	14	1.07
Oceania	Boys	1984	14	1.09
Oceania	Boys	1985	14	1.11
Oceania	Boys	1986	14	1.13
Oceania	Boys	1987	14	1.15
Oceania	Boys	1988	14	1.18
Oceania	Boys	1989	14	1.21
Oceania	Boys	1990	14	1.24
Oceania	Boys	1991	14	1.27
Oceania	Boys	1992	14	1.30
Oceania	Boys	1993	14	1.34
Oceania	Boys	1994	14	1.37
Oceania	Boys	1995	14	1.41
Oceania	Boys	1996	14	1.44
Oceania	Boys	1997	14	1.48
Oceania	Boys	1998	14	1.52
Oceania	Boys	1999	14	1.56
Oceania	Boys	2000	14	1.60
Oceania	Boys	2001	14	1.64
Oceania	Boys	2002	14	1.69
Oceania	Boys	2003	14	1.73
Oceania	Boys	2004	14	1.78
Oceania	Boys	2005	14	1.83

Oceania	Boys	2006	14	1.88
Oceania	Boys	2007	14	1.94
Oceania	Boys	2008	14	2.00
Oceania	Boys	2009	14	2.06
Oceania	Boys	2010	14	2.12
Oceania	Boys	2011	14	2.18
Oceania	Boys	2012	14	2.25
Oceania	Boys	2013	14	2.33
Oceania	Boys	2014	14	2.40
Oceania	Boys	2015	14	2.48
Oceania	Boys	2016	14	2.56
Oceania	Boys	1975	15	1.25
Oceania	Boys	1976	15	1.27
Oceania	Boys	1977	15	1.29
Oceania	Boys	1978	15	1.30
Oceania	Boys	1979	15	1.32
Oceania	Boys	1980	15	1.34
Oceania	Boys	1981	15	1.36
Oceania	Boys	1982	15	1.38
Oceania	Boys	1983	15	1.41
Oceania	Boys	1984	15	1.43
Oceania	Boys	1985	15	1.46
Oceania	Boys	1986	15	1.48
Oceania	Boys	1987	15	1.51
Oceania	Boys	1988	15	1.54
Oceania	Boys	1989	15	1.57
Oceania	Boys	1990	15	1.60
Oceania	Boys	1991	15	1.64
Oceania	Boys	1992	15	1.67
Oceania	Boys	1993	15	1.71
Oceania	Boys	1994	15	1.75
Oceania	Boys	1995	15	1.80
Oceania	Boys	1996	15	1.84
Oceania	Boys	1997	15	1.89
Oceania	Boys	1998	15	1.94
Oceania	Boys	1999	15	1.99
Oceania	Boys	2000	15	2.04
Oceania	Boys	2001	15	2.10
Oceania	Boys	2002	15	2.15
Oceania	Boys	2003	15	2.21
Oceania	Boys	2004	15	2.27
Oceania	Boys	2005	15	2.34
Oceania	Boys	2006	15	2.40
Oceania	Boys	2007	15	2.47
Oceania	Boys	2008	15	2.55
Oceania	Boys	2009	15	2.63
Oceania	Boys	2010	15	2.71
Oceania	Boys	2011	15	2.79
Oceania	Boys	2012	15	2.88
Oceania	Boys	2013	15	2.97
Oceania	Boys	2014	15	3.06
Oceania	Boys	2015	15	3.16

Oceania	Boys	2016	15	3.27
Oceania	Boys	1975	16	1.60
Oceania	Boys	1976	16	1.62
Oceania	Boys	1977	16	1.64
Oceania	Boys	1978	16	1.66
Oceania	Boys	1979	16	1.68
Oceania	Boys	1980	16	1.71
Oceania	Boys	1981	16	1.73
Oceania	Boys	1982	16	1.75
Oceania	Boys	1983	16	1.78
Oceania	Boys	1984	16	1.81
Oceania	Boys	1985	16	1.84
Oceania	Boys	1986	16	1.87
Oceania	Boys	1987	16	1.90
Oceania	Boys	1988	16	1.93
Oceania	Boys	1989	16	1.97
Oceania	Boys	1990	16	2.01
Oceania	Boys	1991	16	2.05
Oceania	Boys	1992	16	2.09
Oceania	Boys	1993	16	2.14
Oceania	Boys	1994	16	2.19
Oceania	Boys	1995	16	2.24
Oceania	Boys	1996	16	2.29
Oceania	Boys	1997	16	2.35
Oceania	Boys	1998	16	2.41
Oceania	Boys	1999	16	2.47
Oceania	Boys	2000	16	2.53
Oceania	Boys	2001	16	2.59
Oceania	Boys	2002	16	2.66
Oceania	Boys	2003	16	2.73
Oceania	Boys	2004	16	2.80
Oceania	Boys	2005	16	2.88
Oceania	Boys	2006	16	2.96
Oceania	Boys	2007	16	3.04
Oceania	Boys	2008	16	3.13
Oceania	Boys	2009	16	3.22
Oceania	Boys	2010	16	3.32
Oceania	Boys	2011	16	3.42
Oceania	Boys	2012	16	3.53
Oceania	Boys	2013	16	3.64
Oceania	Boys	2014	16	3.75
Oceania	Boys	2015	16	3.88
Oceania	Boys	2016	16	4.01
Oceania	Boys	1975	17	1.99
Oceania	Boys	1976	17	2.02
Oceania	Boys	1977	17	2.04
Oceania	Boys	1978	17	2.06
Oceania	Boys	1979	17	2.09
Oceania	Boys	1980	17	2.11
Oceania	Boys	1981	17	2.14
Oceania	Boys	1982	17	2.17
Oceania	Boys	1983	17	2.20

Oceania	Boys	1984	17	2.23
Oceania	Boys	1985	17	2.27
Oceania	Boys	1986	17	2.30
Oceania	Boys	1987	17	2.33
Oceania	Boys	1988	17	2.37
Oceania	Boys	1989	17	2.41
Oceania	Boys	1990	17	2.46
Oceania	Boys	1991	17	2.50
Oceania	Boys	1992	17	2.56
Oceania	Boys	1993	17	2.61
Oceania	Boys	1994	17	2.66
Oceania	Boys	1995	17	2.72
Oceania	Boys	1996	17	2.78
Oceania	Boys	1997	17	2.85
Oceania	Boys	1998	17	2.92
Oceania	Boys	1999	17	2.98
Oceania	Boys	2000	17	3.06
Oceania	Boys	2001	17	3.13
Oceania	Boys	2002	17	3.21
Oceania	Boys	2003	17	3.29
Oceania	Boys	2004	17	3.37
Oceania	Boys	2005	17	3.46
Oceania	Boys	2006	17	3.55
Oceania	Boys	2007	17	3.65
Oceania	Boys	2008	17	3.75
Oceania	Boys	2009	17	3.86
Oceania	Boys	2010	17	3.97
Oceania	Boys	2011	17	4.09
Oceania	Boys	2012	17	4.21
Oceania	Boys	2013	17	4.34
Oceania	Boys	2014	17	4.48
Oceania	Boys	2015	17	4.63
Oceania	Boys	2016	17	4.78
Oceania	Boys	1975	18	2.42
Oceania	Boys	1976	18	2.45
Oceania	Boys	1977	18	2.47
Oceania	Boys	1978	18	2.50
Oceania	Boys	1979	18	2.53
Oceania	Boys	1980	18	2.56
Oceania	Boys	1981	18	2.59
Oceania	Boys	1982	18	2.63
Oceania	Boys	1983	18	2.66
Oceania	Boys	1984	18	2.70
Oceania	Boys	1985	18	2.74
Oceania	Boys	1986	18	2.78
Oceania	Boys	1987	18	2.82
Oceania	Boys	1988	18	2.86
Oceania	Boys	1989	18	2.91
Oceania	Boys	1990	18	2.96
Oceania	Boys	1991	18	3.01
Oceania	Boys	1992	18	3.07
Oceania	Boys	1993	18	3.14

Oceania	Boys	1994	18	3.20
Oceania	Boys	1995	18	3.27
Oceania	Boys	1996	18	3.34
Oceania	Boys	1997	18	3.41
Oceania	Boys	1998	18	3.49
Oceania	Boys	1999	18	3.57
Oceania	Boys	2000	18	3.65
Oceania	Boys	2001	18	3.74
Oceania	Boys	2002	18	3.82
Oceania	Boys	2003	18	3.92
Oceania	Boys	2004	18	4.01
Oceania	Boys	2005	18	4.12
Oceania	Boys	2006	18	4.22
Oceania	Boys	2007	18	4.34
Oceania	Boys	2008	18	4.46
Oceania	Boys	2009	18	4.58
Oceania	Boys	2010	18	4.71
Oceania	Boys	2011	18	4.85
Oceania	Boys	2012	18	4.99
Oceania	Boys	2013	18	5.14
Oceania	Boys	2014	18	5.30
Oceania	Boys	2015	18	5.46
Oceania	Boys	2016	18	5.64
Oceania	Boys	1975	Average	1.35
Oceania	Boys	1976	Average	1.37
Oceania	Boys	1977	Average	1.39
Oceania	Boys	1978	Average	1.40
Oceania	Boys	1979	Average	1.42
Oceania	Boys	1980	Average	1.44
Oceania	Boys	1981	Average	1.46
Oceania	Boys	1982	Average	1.48
Oceania	Boys	1983	Average	1.50
Oceania	Boys	1984	Average	1.53
Oceania	Boys	1985	Average	1.55
Oceania	Boys	1986	Average	1.58
Oceania	Boys	1987	Average	1.61
Oceania	Boys	1988	Average	1.63
Oceania	Boys	1989	Average	1.67
Oceania	Boys	1990	Average	1.70
Oceania	Boys	1991	Average	1.74
Oceania	Boys	1992	Average	1.77
Oceania	Boys	1993	Average	1.81
Oceania	Boys	1994	Average	1.86
Oceania	Boys	1995	Average	1.90
Oceania	Boys	1996	Average	1.94
Oceania	Boys	1997	Average	1.99
Oceania	Boys	1998	Average	2.04
Oceania	Boys	1999	Average	2.09
Oceania	Boys	2000	Average	2.14
Oceania	Boys	2001	Average	2.19
Oceania	Boys	2002	Average	2.25
Oceania	Boys	2003	Average	2.31

Oceania	Boys	2004	Average	2.37
Oceania	Boys	2005	Average	2.43
Oceania	Boys	2006	Average	2.50
Oceania	Boys	2007	Average	2.57
Oceania	Boys	2008	Average	2.64
Oceania	Boys	2009	Average	2.72
Oceania	Boys	2010	Average	2.80
Oceania	Boys	2011	Average	2.88
Oceania	Boys	2012	Average	2.97
Oceania	Boys	2013	Average	3.06
Oceania	Boys	2014	Average	3.16
Oceania	Boys	2015	Average	3.26
Oceania	Boys	2016	Average	3.37
Oceania	Boys	2017	Average	3.47
Oceania	Boys	2018	Average	3.58
Oceania	Boys	2019	Average	3.69
Oceania	Boys	2020	Average	3.80
Oceania	Boys	2021	Average	3.91
Oceania	Boys	2022	Average	4.02
Oceania	Boys	2023	Average	4.13
Oceania	Boys	2024	Average	4.25
Oceania	Boys	2025	Average	4.36
Oceania	Boys	2026	Average	4.47
Oceania	Boys	2027	Average	4.59
Oceania	Boys	2028	Average	4.70
Oceania	Boys	2029	Average	4.82
Oceania	Boys	2030	Average	4.93
Oceania	Girls	1975	12	0.78
Oceania	Girls	1976	12	0.79
Oceania	Girls	1977	12	0.81
Oceania	Girls	1978	12	0.83
Oceania	Girls	1979	12	0.85
Oceania	Girls	1980	12	0.87
Oceania	Girls	1981	12	0.89
Oceania	Girls	1982	12	0.92
Oceania	Girls	1983	12	0.94
Oceania	Girls	1984	12	0.96
Oceania	Girls	1985	12	0.98
Oceania	Girls	1986	12	1.01
Oceania	Girls	1987	12	1.04
Oceania	Girls	1988	12	1.07
Oceania	Girls	1989	12	1.10
Oceania	Girls	1990	12	1.13
Oceania	Girls	1991	12	1.16
Oceania	Girls	1992	12	1.20
Oceania	Girls	1993	12	1.23
Oceania	Girls	1994	12	1.27
Oceania	Girls	1995	12	1.30
Oceania	Girls	1996	12	1.34
Oceania	Girls	1997	12	1.37
Oceania	Girls	1998	12	1.41
Oceania	Girls	1999	12	1.44

Oceania	Girls	2000	12	1.48
Oceania	Girls	2001	12	1.52
Oceania	Girls	2002	12	1.55
Oceania	Girls	2003	12	1.59
Oceania	Girls	2004	12	1.63
Oceania	Girls	2005	12	1.67
Oceania	Girls	2006	12	1.72
Oceania	Girls	2007	12	1.76
Oceania	Girls	2008	12	1.80
Oceania	Girls	2009	12	1.84
Oceania	Girls	2010	12	1.89
Oceania	Girls	2011	12	1.93
Oceania	Girls	2012	12	1.98
Oceania	Girls	2013	12	2.03
Oceania	Girls	2014	12	2.08
Oceania	Girls	2015	12	2.13
Oceania	Girls	2016	12	2.18
Oceania	Girls	1975	13	1.10
Oceania	Girls	1976	13	1.13
Oceania	Girls	1977	13	1.16
Oceania	Girls	1978	13	1.18
Oceania	Girls	1979	13	1.21
Oceania	Girls	1980	13	1.24
Oceania	Girls	1981	13	1.27
Oceania	Girls	1982	13	1.30
Oceania	Girls	1983	13	1.33
Oceania	Girls	1984	13	1.36
Oceania	Girls	1985	13	1.39
Oceania	Girls	1986	13	1.43
Oceania	Girls	1987	13	1.47
Oceania	Girls	1988	13	1.51
Oceania	Girls	1989	13	1.55
Oceania	Girls	1990	13	1.60
Oceania	Girls	1991	13	1.64
Oceania	Girls	1992	13	1.69
Oceania	Girls	1993	13	1.74
Oceania	Girls	1994	13	1.79
Oceania	Girls	1995	13	1.84
Oceania	Girls	1996	13	1.89
Oceania	Girls	1997	13	1.93
Oceania	Girls	1998	13	1.98
Oceania	Girls	1999	13	2.03
Oceania	Girls	2000	13	2.08
Oceania	Girls	2001	13	2.14
Oceania	Girls	2002	13	2.19
Oceania	Girls	2003	13	2.24
Oceania	Girls	2004	13	2.30
Oceania	Girls	2005	13	2.36
Oceania	Girls	2006	13	2.42
Oceania	Girls	2007	13	2.47
Oceania	Girls	2008	13	2.53
Oceania	Girls	2009	13	2.59

Oceania	Girls	2010	13	2.66
Oceania	Girls	2011	13	2.72
Oceania	Girls	2012	13	2.79
Oceania	Girls	2013	13	2.86
Oceania	Girls	2014	13	2.93
Oceania	Girls	2015	13	3.00
Oceania	Girls	2016	13	3.08
Oceania	Girls	1975	14	1.48
Oceania	Girls	1976	14	1.51
Oceania	Girls	1977	14	1.55
Oceania	Girls	1978	14	1.58
Oceania	Girls	1979	14	1.62
Oceania	Girls	1980	14	1.65
Oceania	Girls	1981	14	1.69
Oceania	Girls	1982	14	1.73
Oceania	Girls	1983	14	1.77
Oceania	Girls	1984	14	1.81
Oceania	Girls	1985	14	1.85
Oceania	Girls	1986	14	1.90
Oceania	Girls	1987	14	1.95
Oceania	Girls	1988	14	2.00
Oceania	Girls	1989	14	2.06
Oceania	Girls	1990	14	2.12
Oceania	Girls	1991	14	2.18
Oceania	Girls	1992	14	2.24
Oceania	Girls	1993	14	2.30
Oceania	Girls	1994	14	2.36
Oceania	Girls	1995	14	2.42
Oceania	Girls	1996	14	2.49
Oceania	Girls	1997	14	2.55
Oceania	Girls	1998	14	2.61
Oceania	Girls	1999	14	2.68
Oceania	Girls	2000	14	2.74
Oceania	Girls	2001	14	2.81
Oceania	Girls	2002	14	2.88
Oceania	Girls	2003	14	2.95
Oceania	Girls	2004	14	3.02
Oceania	Girls	2005	14	3.09
Oceania	Girls	2006	14	3.16
Oceania	Girls	2007	14	3.24
Oceania	Girls	2008	14	3.32
Oceania	Girls	2009	14	3.39
Oceania	Girls	2010	14	3.48
Oceania	Girls	2011	14	3.56
Oceania	Girls	2012	14	3.65
Oceania	Girls	2013	14	3.74
Oceania	Girls	2014	14	3.83
Oceania	Girls	2015	14	3.93
Oceania	Girls	2016	14	4.02
Oceania	Girls	1975	15	1.84
Oceania	Girls	1976	15	1.88
Oceania	Girls	1977	15	1.92

Oceania	Girls	1978	15	1.97
Oceania	Girls	1979	15	2.01
Oceania	Girls	1980	15	2.06
Oceania	Girls	1981	15	2.11
Oceania	Girls	1982	15	2.16
Oceania	Girls	1983	15	2.21
Oceania	Girls	1984	15	2.26
Oceania	Girls	1985	15	2.31
Oceania	Girls	1986	15	2.37
Oceania	Girls	1987	15	2.42
Oceania	Girls	1988	15	2.48
Oceania	Girls	1989	15	2.54
Oceania	Girls	1990	15	2.60
Oceania	Girls	1991	15	2.67
Oceania	Girls	1992	15	2.74
Oceania	Girls	1993	15	2.81
Oceania	Girls	1994	15	2.88
Oceania	Girls	1995	15	2.96
Oceania	Girls	1996	15	3.03
Oceania	Girls	1997	15	3.11
Oceania	Girls	1998	15	3.19
Oceania	Girls	1999	15	3.26
Oceania	Girls	2000	15	3.34
Oceania	Girls	2001	15	3.42
Oceania	Girls	2002	15	3.51
Oceania	Girls	2003	15	3.59
Oceania	Girls	2004	15	3.67
Oceania	Girls	2005	15	3.76
Oceania	Girls	2006	15	3.85
Oceania	Girls	2007	15	3.94
Oceania	Girls	2008	15	4.04
Oceania	Girls	2009	15	4.13
Oceania	Girls	2010	15	4.23
Oceania	Girls	2011	15	4.33
Oceania	Girls	2012	15	4.43
Oceania	Girls	2013	15	4.53
Oceania	Girls	2014	15	4.64
Oceania	Girls	2015	15	4.76
Oceania	Girls	2016	15	4.87
Oceania	Girls	1975	16	2.14
Oceania	Girls	1976	16	2.19
Oceania	Girls	1977	16	2.24
Oceania	Girls	1978	16	2.29
Oceania	Girls	1979	16	2.34
Oceania	Girls	1980	16	2.39
Oceania	Girls	1981	16	2.44
Oceania	Girls	1982	16	2.50
Oceania	Girls	1983	16	2.56
Oceania	Girls	1984	16	2.62
Oceania	Girls	1985	16	2.68
Oceania	Girls	1986	16	2.74
Oceania	Girls	1987	16	2.80

Oceania	Girls	1988	16	2.86
Oceania	Girls	1989	16	2.93
Oceania	Girls	1990	16	3.00
Oceania	Girls	1991	16	3.08
Oceania	Girls	1992	16	3.16
Oceania	Girls	1993	16	3.23
Oceania	Girls	1994	16	3.32
Oceania	Girls	1995	16	3.40
Oceania	Girls	1996	16	3.48
Oceania	Girls	1997	16	3.57
Oceania	Girls	1998	16	3.66
Oceania	Girls	1999	16	3.75
Oceania	Girls	2000	16	3.84
Oceania	Girls	2001	16	3.93
Oceania	Girls	2002	16	4.02
Oceania	Girls	2003	16	4.11
Oceania	Girls	2004	16	4.20
Oceania	Girls	2005	16	4.30
Oceania	Girls	2006	16	4.40
Oceania	Girls	2007	16	4.51
Oceania	Girls	2008	16	4.61
Oceania	Girls	2009	16	4.72
Oceania	Girls	2010	16	4.83
Oceania	Girls	2011	16	4.94
Oceania	Girls	2012	16	5.05
Oceania	Girls	2013	16	5.17
Oceania	Girls	2014	16	5.29
Oceania	Girls	2015	16	5.42
Oceania	Girls	2016	16	5.55
Oceania	Girls	1975	17	2.39
Oceania	Girls	1976	17	2.44
Oceania	Girls	1977	17	2.49
Oceania	Girls	1978	17	2.55
Oceania	Girls	1979	17	2.60
Oceania	Girls	1980	17	2.66
Oceania	Girls	1981	17	2.72
Oceania	Girls	1982	17	2.78
Oceania	Girls	1983	17	2.84
Oceania	Girls	1984	17	2.91
Oceania	Girls	1985	17	2.98
Oceania	Girls	1986	17	3.04
Oceania	Girls	1987	17	3.11
Oceania	Girls	1988	17	3.18
Oceania	Girls	1989	17	3.26
Oceania	Girls	1990	17	3.34
Oceania	Girls	1991	17	3.42
Oceania	Girls	1992	17	3.50
Oceania	Girls	1993	17	3.59
Oceania	Girls	1994	17	3.68
Oceania	Girls	1995	17	3.77
Oceania	Girls	1996	17	3.87
Oceania	Girls	1997	17	3.96

Oceania	Girls	1998	17	4.06
Oceania	Girls	1999	17	4.16
Oceania	Girls	2000	17	4.26
Oceania	Girls	2001	17	4.36
Oceania	Girls	2002	17	4.46
Oceania	Girls	2003	17	4.56
Oceania	Girls	2004	17	4.66
Oceania	Girls	2005	17	4.77
Oceania	Girls	2006	17	4.88
Oceania	Girls	2007	17	5.00
Oceania	Girls	2008	17	5.11
Oceania	Girls	2009	17	5.23
Oceania	Girls	2010	17	5.35
Oceania	Girls	2011	17	5.47
Oceania	Girls	2012	17	5.60
Oceania	Girls	2013	17	5.73
Oceania	Girls	2014	17	5.86
Oceania	Girls	2015	17	6.00
Oceania	Girls	2016	17	6.14
Oceania	Girls	1975	18	2.62
Oceania	Girls	1976	18	2.68
Oceania	Girls	1977	18	2.74
Oceania	Girls	1978	18	2.80
Oceania	Girls	1979	18	2.86
Oceania	Girls	1980	18	2.93
Oceania	Girls	1981	18	2.99
Oceania	Girls	1982	18	3.06
Oceania	Girls	1983	18	3.14
Oceania	Girls	1984	18	3.21
Oceania	Girls	1985	18	3.28
Oceania	Girls	1986	18	3.36
Oceania	Girls	1987	18	3.44
Oceania	Girls	1988	18	3.51
Oceania	Girls	1989	18	3.60
Oceania	Girls	1990	18	3.69
Oceania	Girls	1991	18	3.78
Oceania	Girls	1992	18	3.88
Oceania	Girls	1993	18	3.98
Oceania	Girls	1994	18	4.08
Oceania	Girls	1995	18	4.18
Oceania	Girls	1996	18	4.29
Oceania	Girls	1997	18	4.39
Oceania	Girls	1998	18	4.50
Oceania	Girls	1999	18	4.61
Oceania	Girls	2000	18	4.72
Oceania	Girls	2001	18	4.83
Oceania	Girls	2002	18	4.95
Oceania	Girls	2003	18	5.06
Oceania	Girls	2004	18	5.18
Oceania	Girls	2005	18	5.30
Oceania	Girls	2006	18	5.42
Oceania	Girls	2007	18	5.55

Oceania	Girls	2008	18	5.68
Oceania	Girls	2009	18	5.81
Oceania	Girls	2010	18	5.94
Oceania	Girls	2011	18	6.08
Oceania	Girls	2012	18	6.22
Oceania	Girls	2013	18	6.36
Oceania	Girls	2014	18	6.50
Oceania	Girls	2015	18	6.65
Oceania	Girls	2016	18	6.81
Oceania	Girls	1975	Average	1.76
Oceania	Girls	1976	Average	1.80
Oceania	Girls	1977	Average	1.84
Oceania	Girls	1978	Average	1.89
Oceania	Girls	1979	Average	1.93
Oceania	Girls	1980	Average	1.97
Oceania	Girls	1981	Average	2.02
Oceania	Girls	1982	Average	2.06
Oceania	Girls	1983	Average	2.11
Oceania	Girls	1984	Average	2.16
Oceania	Girls	1985	Average	2.21
Oceania	Girls	1986	Average	2.26
Oceania	Girls	1987	Average	2.32
Oceania	Girls	1988	Average	2.37
Oceania	Girls	1989	Average	2.43
Oceania	Girls	1990	Average	2.50
Oceania	Girls	1991	Average	2.56
Oceania	Girls	1992	Average	2.63
Oceania	Girls	1993	Average	2.70
Oceania	Girls	1994	Average	2.77
Oceania	Girls	1995	Average	2.84
Oceania	Girls	1996	Average	2.91
Oceania	Girls	1997	Average	2.98
Oceania	Girls	1998	Average	3.06
Oceania	Girls	1999	Average	3.13
Oceania	Girls	2000	Average	3.21
Oceania	Girls	2001	Average	3.29
Oceania	Girls	2002	Average	3.36
Oceania	Girls	2003	Average	3.44
Oceania	Girls	2004	Average	3.52
Oceania	Girls	2005	Average	3.61
Oceania	Girls	2006	Average	3.69
Oceania	Girls	2007	Average	3.78
Oceania	Girls	2008	Average	3.87
Oceania	Girls	2009	Average	3.96
Oceania	Girls	2010	Average	4.05
Oceania	Girls	2011	Average	4.15
Oceania	Girls	2012	Average	4.25
Oceania	Girls	2013	Average	4.35
Oceania	Girls	2014	Average	4.45
Oceania	Girls	2015	Average	4.56
Oceania	Girls	2016	Average	4.67
Oceania	Girls	2017	Average	4.78

Oceania	Girls	2018	Average	4.90
Oceania	Girls	2019	Average	5.01
Oceania	Girls	2020	Average	5.13
Oceania	Girls	2021	Average	5.26
Oceania	Girls	2022	Average	5.38
Oceania	Girls	2023	Average	5.51
Oceania	Girls	2024	Average	5.64
Oceania	Girls	2025	Average	5.77
Oceania	Girls	2026	Average	5.90
Oceania	Girls	2027	Average	6.04
Oceania	Girls	2028	Average	6.17
Oceania	Girls	2029	Average	6.31
Oceania	Girls	2030	Average	6.45
South Asia	Boys	1975	12	0.23
South Asia	Boys	1976	12	0.23
South Asia	Boys	1977	12	0.24
South Asia	Boys	1978	12	0.24
South Asia	Boys	1979	12	0.24
South Asia	Boys	1980	12	0.24
South Asia	Boys	1981	12	0.24
South Asia	Boys	1982	12	0.25
South Asia	Boys	1983	12	0.25
South Asia	Boys	1984	12	0.25
South Asia	Boys	1985	12	0.25
South Asia	Boys	1986	12	0.26
South Asia	Boys	1987	12	0.26
South Asia	Boys	1988	12	0.26
South Asia	Boys	1989	12	0.27
South Asia	Boys	1990	12	0.27
South Asia	Boys	1991	12	0.27
South Asia	Boys	1992	12	0.28
South Asia	Boys	1993	12	0.28
South Asia	Boys	1994	12	0.29
South Asia	Boys	1995	12	0.29
South Asia	Boys	1996	12	0.30
South Asia	Boys	1997	12	0.30
South Asia	Boys	1998	12	0.31
South Asia	Boys	1999	12	0.32
South Asia	Boys	2000	12	0.33
South Asia	Boys	2001	12	0.33
South Asia	Boys	2002	12	0.34
South Asia	Boys	2003	12	0.35
South Asia	Boys	2004	12	0.36
South Asia	Boys	2005	12	0.37
South Asia	Boys	2006	12	0.38
South Asia	Boys	2007	12	0.40
South Asia	Boys	2008	12	0.41
South Asia	Boys	2009	12	0.42
South Asia	Boys	2010	12	0.44
South Asia	Boys	2011	12	0.45
South Asia	Boys	2012	12	0.47
South Asia	Boys	2013	12	0.49

South Asia	Boys	2014	12	0.51
South Asia	Boys	2015	12	0.53
South Asia	Boys	2016	12	0.55
South Asia	Boys	1975	13	0.30
South Asia	Boys	1976	13	0.31
South Asia	Boys	1977	13	0.31
South Asia	Boys	1978	13	0.31
South Asia	Boys	1979	13	0.31
South Asia	Boys	1980	13	0.32
South Asia	Boys	1981	13	0.32
South Asia	Boys	1982	13	0.32
South Asia	Boys	1983	13	0.33
South Asia	Boys	1984	13	0.33
South Asia	Boys	1985	13	0.33
South Asia	Boys	1986	13	0.34
South Asia	Boys	1987	13	0.34
South Asia	Boys	1988	13	0.35
South Asia	Boys	1989	13	0.35
South Asia	Boys	1990	13	0.35
South Asia	Boys	1991	13	0.36
South Asia	Boys	1992	13	0.37
South Asia	Boys	1993	13	0.37
South Asia	Boys	1994	13	0.38
South Asia	Boys	1995	13	0.38
South Asia	Boys	1996	13	0.39
South Asia	Boys	1997	13	0.40
South Asia	Boys	1998	13	0.41
South Asia	Boys	1999	13	0.42
South Asia	Boys	2000	13	0.43
South Asia	Boys	2001	13	0.44
South Asia	Boys	2002	13	0.45
South Asia	Boys	2003	13	0.46
South Asia	Boys	2004	13	0.47
South Asia	Boys	2005	13	0.49
South Asia	Boys	2006	13	0.50
South Asia	Boys	2007	13	0.52
South Asia	Boys	2008	13	0.53
South Asia	Boys	2009	13	0.55
South Asia	Boys	2010	13	0.57
South Asia	Boys	2011	13	0.59
South Asia	Boys	2012	13	0.62
South Asia	Boys	2013	13	0.64
South Asia	Boys	2014	13	0.67
South Asia	Boys	2015	13	0.70
South Asia	Boys	2016	13	0.73
South Asia	Boys	1975	14	0.40
South Asia	Boys	1976	14	0.41
South Asia	Boys	1977	14	0.41
South Asia	Boys	1978	14	0.41
South Asia	Boys	1979	14	0.42
South Asia	Boys	1980	14	0.42
South Asia	Boys	1981	14	0.42

South Asia	Boys	1982	14	0.43
South Asia	Boys	1983	14	0.43
South Asia	Boys	1984	14	0.44
South Asia	Boys	1985	14	0.44
South Asia	Boys	1986	14	0.45
South Asia	Boys	1987	14	0.45
South Asia	Boys	1988	14	0.46
South Asia	Boys	1989	14	0.46
South Asia	Boys	1990	14	0.47
South Asia	Boys	1991	14	0.48
South Asia	Boys	1992	14	0.48
South Asia	Boys	1993	14	0.49
South Asia	Boys	1994	14	0.50
South Asia	Boys	1995	14	0.51
South Asia	Boys	1996	14	0.52
South Asia	Boys	1997	14	0.53
South Asia	Boys	1998	14	0.54
South Asia	Boys	1999	14	0.55
South Asia	Boys	2000	14	0.56
South Asia	Boys	2001	14	0.57
South Asia	Boys	2002	14	0.59
South Asia	Boys	2003	14	0.60
South Asia	Boys	2004	14	0.62
South Asia	Boys	2005	14	0.64
South Asia	Boys	2006	14	0.65
South Asia	Boys	2007	14	0.67
South Asia	Boys	2008	14	0.70
South Asia	Boys	2009	14	0.72
South Asia	Boys	2010	14	0.74
South Asia	Boys	2011	14	0.77
South Asia	Boys	2012	14	0.80
South Asia	Boys	2013	14	0.83
South Asia	Boys	2014	14	0.86
South Asia	Boys	2015	14	0.90
South Asia	Boys	2016	14	0.94
South Asia	Boys	1975	15	0.53
South Asia	Boys	1976	15	0.54
South Asia	Boys	1977	15	0.54
South Asia	Boys	1978	15	0.55
South Asia	Boys	1979	15	0.55
South Asia	Boys	1980	15	0.55
South Asia	Boys	1981	15	0.56
South Asia	Boys	1982	15	0.57
South Asia	Boys	1983	15	0.57
South Asia	Boys	1984	15	0.58
South Asia	Boys	1985	15	0.58
South Asia	Boys	1986	15	0.59
South Asia	Boys	1987	15	0.60
South Asia	Boys	1988	15	0.60
South Asia	Boys	1989	15	0.61
South Asia	Boys	1990	15	0.62
South Asia	Boys	1991	15	0.63

South Asia	Boys	1992	15	0.63
South Asia	Boys	1993	15	0.64
South Asia	Boys	1994	15	0.65
South Asia	Boys	1995	15	0.66
South Asia	Boys	1996	15	0.67
South Asia	Boys	1997	15	0.68
South Asia	Boys	1998	15	0.70
South Asia	Boys	1999	15	0.71
South Asia	Boys	2000	15	0.73
South Asia	Boys	2001	15	0.74
South Asia	Boys	2002	15	0.76
South Asia	Boys	2003	15	0.78
South Asia	Boys	2004	15	0.80
South Asia	Boys	2005	15	0.82
South Asia	Boys	2006	15	0.84
South Asia	Boys	2007	15	0.86
South Asia	Boys	2008	15	0.89
South Asia	Boys	2009	15	0.92
South Asia	Boys	2010	15	0.95
South Asia	Boys	2011	15	0.98
South Asia	Boys	2012	15	1.01
South Asia	Boys	2013	15	1.05
South Asia	Boys	2014	15	1.09
South Asia	Boys	2015	15	1.13
South Asia	Boys	2016	15	1.18
South Asia	Boys	1975	16	0.69
South Asia	Boys	1976	16	0.69
South Asia	Boys	1977	16	0.70
South Asia	Boys	1978	16	0.70
South Asia	Boys	1979	16	0.71
South Asia	Boys	1980	16	0.72
South Asia	Boys	1981	16	0.72
South Asia	Boys	1982	16	0.73
South Asia	Boys	1983	16	0.74
South Asia	Boys	1984	16	0.74
South Asia	Boys	1985	16	0.75
South Asia	Boys	1986	16	0.76
South Asia	Boys	1987	16	0.77
South Asia	Boys	1988	16	0.78
South Asia	Boys	1989	16	0.78
South Asia	Boys	1990	16	0.79
South Asia	Boys	1991	16	0.80
South Asia	Boys	1992	16	0.81
South Asia	Boys	1993	16	0.82
South Asia	Boys	1994	16	0.83
South Asia	Boys	1995	16	0.85
South Asia	Boys	1996	16	0.86
South Asia	Boys	1997	16	0.87
South Asia	Boys	1998	16	0.89
South Asia	Boys	1999	16	0.90
South Asia	Boys	2000	16	0.92
South Asia	Boys	2001	16	0.94

South Asia	Boys	2002	16	0.96
South Asia	Boys	2003	16	0.98
South Asia	Boys	2004	16	1.00
South Asia	Boys	2005	16	1.03
South Asia	Boys	2006	16	1.05
South Asia	Boys	2007	16	1.08
South Asia	Boys	2008	16	1.11
South Asia	Boys	2009	16	1.14
South Asia	Boys	2010	16	1.18
South Asia	Boys	2011	16	1.22
South Asia	Boys	2012	16	1.26
South Asia	Boys	2013	16	1.30
South Asia	Boys	2014	16	1.35
South Asia	Boys	2015	16	1.40
South Asia	Boys	2016	16	1.45
South Asia	Boys	1975	17	0.86
South Asia	Boys	1976	17	0.87
South Asia	Boys	1977	17	0.88
South Asia	Boys	1978	17	0.88
South Asia	Boys	1979	17	0.89
South Asia	Boys	1980	17	0.90
South Asia	Boys	1981	17	0.91
South Asia	Boys	1982	17	0.91
South Asia	Boys	1983	17	0.92
South Asia	Boys	1984	17	0.93
South Asia	Boys	1985	17	0.94
South Asia	Boys	1986	17	0.95
South Asia	Boys	1987	17	0.96
South Asia	Boys	1988	17	0.97
South Asia	Boys	1989	17	0.98
South Asia	Boys	1990	17	0.99
South Asia	Boys	1991	17	1.00
South Asia	Boys	1992	17	1.01
South Asia	Boys	1993	17	1.03
South Asia	Boys	1994	17	1.04
South Asia	Boys	1995	17	1.05
South Asia	Boys	1996	17	1.07
South Asia	Boys	1997	17	1.09
South Asia	Boys	1998	17	1.10
South Asia	Boys	1999	17	1.12
South Asia	Boys	2000	17	1.14
South Asia	Boys	2001	17	1.16
South Asia	Boys	2002	17	1.19
South Asia	Boys	2003	17	1.21
South Asia	Boys	2004	17	1.24
South Asia	Boys	2005	17	1.26
South Asia	Boys	2006	17	1.29
South Asia	Boys	2007	17	1.33
South Asia	Boys	2008	17	1.36
South Asia	Boys	2009	17	1.40
South Asia	Boys	2010	17	1.44
South Asia	Boys	2011	17	1.48

South Asia	Boys	2012	17	1.53
South Asia	Boys	2013	17	1.57
South Asia	Boys	2014	17	1.63
South Asia	Boys	2015	17	1.69
South Asia	Boys	2016	17	1.75
South Asia	Boys	1975	18	1.04
South Asia	Boys	1976	18	1.05
South Asia	Boys	1977	18	1.06
South Asia	Boys	1978	18	1.07
South Asia	Boys	1979	18	1.08
South Asia	Boys	1980	18	1.09
South Asia	Boys	1981	18	1.10
South Asia	Boys	1982	18	1.11
South Asia	Boys	1983	18	1.12
South Asia	Boys	1984	18	1.13
South Asia	Boys	1985	18	1.14
South Asia	Boys	1986	18	1.15
South Asia	Boys	1987	18	1.16
South Asia	Boys	1988	18	1.18
South Asia	Boys	1989	18	1.19
South Asia	Boys	1990	18	1.20
South Asia	Boys	1991	18	1.22
South Asia	Boys	1992	18	1.23
South Asia	Boys	1993	18	1.25
South Asia	Boys	1994	18	1.26
South Asia	Boys	1995	18	1.28
South Asia	Boys	1996	18	1.30
South Asia	Boys	1997	18	1.32
South Asia	Boys	1998	18	1.34
South Asia	Boys	1999	18	1.36
South Asia	Boys	2000	18	1.38
South Asia	Boys	2001	18	1.41
South Asia	Boys	2002	18	1.43
South Asia	Boys	2003	18	1.46
South Asia	Boys	2004	18	1.49
South Asia	Boys	2005	18	1.53
South Asia	Boys	2006	18	1.56
South Asia	Boys	2007	18	1.60
South Asia	Boys	2008	18	1.64
South Asia	Boys	2009	18	1.68
South Asia	Boys	2010	18	1.73
South Asia	Boys	2011	18	1.78
South Asia	Boys	2012	18	1.83
South Asia	Boys	2013	18	1.89
South Asia	Boys	2014	18	1.95
South Asia	Boys	2015	18	2.02
South Asia	Boys	2016	18	2.09
South Asia	Boys	1975	Average	0.58
South Asia	Boys	1976	Average	0.59
South Asia	Boys	1977	Average	0.59
South Asia	Boys	1978	Average	0.59
South Asia	Boys	1979	Average	0.60

South Asia	Boys	1980	Average	0.61
South Asia	Boys	1981	Average	0.61
South Asia	Boys	1982	Average	0.62
South Asia	Boys	1983	Average	0.62
South Asia	Boys	1984	Average	0.63
South Asia	Boys	1985	Average	0.64
South Asia	Boys	1986	Average	0.64
South Asia	Boys	1987	Average	0.65
South Asia	Boys	1988	Average	0.66
South Asia	Boys	1989	Average	0.66
South Asia	Boys	1990	Average	0.67
South Asia	Boys	1991	Average	0.68
South Asia	Boys	1992	Average	0.69
South Asia	Boys	1993	Average	0.70
South Asia	Boys	1994	Average	0.71
South Asia	Boys	1995	Average	0.72
South Asia	Boys	1996	Average	0.73
South Asia	Boys	1997	Average	0.74
South Asia	Boys	1998	Average	0.75
South Asia	Boys	1999	Average	0.77
South Asia	Boys	2000	Average	0.78
South Asia	Boys	2001	Average	0.80
South Asia	Boys	2002	Average	0.82
South Asia	Boys	2003	Average	0.83
South Asia	Boys	2004	Average	0.85
South Asia	Boys	2005	Average	0.88
South Asia	Boys	2006	Average	0.90
South Asia	Boys	2007	Average	0.92
South Asia	Boys	2008	Average	0.95
South Asia	Boys	2009	Average	0.98
South Asia	Boys	2010	Average	1.01
South Asia	Boys	2011	Average	1.04
South Asia	Boys	2012	Average	1.07
South Asia	Boys	2013	Average	1.11
South Asia	Boys	2014	Average	1.15
South Asia	Boys	2015	Average	1.19
South Asia	Boys	2016	Average	1.24
South Asia	Boys	2017	Average	1.29
South Asia	Boys	2018	Average	1.33
South Asia	Boys	2019	Average	1.38
South Asia	Boys	2020	Average	1.43
South Asia	Boys	2021	Average	1.48
South Asia	Boys	2022	Average	1.52
South Asia	Boys	2023	Average	1.57
South Asia	Boys	2024	Average	1.62
South Asia	Boys	2025	Average	1.66
South Asia	Boys	2026	Average	1.71
South Asia	Boys	2027	Average	1.76
South Asia	Boys	2028	Average	1.80
South Asia	Boys	2029	Average	1.85
South Asia	Boys	2030	Average	1.90
South Asia	Girls	1975	12	0.24

South Asia	Girls	1976	12	0.24
South Asia	Girls	1977	12	0.24
South Asia	Girls	1978	12	0.25
South Asia	Girls	1979	12	0.25
South Asia	Girls	1980	12	0.25
South Asia	Girls	1981	12	0.25
South Asia	Girls	1982	12	0.26
South Asia	Girls	1983	12	0.26
South Asia	Girls	1984	12	0.26
South Asia	Girls	1985	12	0.26
South Asia	Girls	1986	12	0.27
South Asia	Girls	1987	12	0.27
South Asia	Girls	1988	12	0.27
South Asia	Girls	1989	12	0.28
South Asia	Girls	1990	12	0.28
South Asia	Girls	1991	12	0.28
South Asia	Girls	1992	12	0.29
South Asia	Girls	1993	12	0.29
South Asia	Girls	1994	12	0.30
South Asia	Girls	1995	12	0.30
South Asia	Girls	1996	12	0.31
South Asia	Girls	1997	12	0.31
South Asia	Girls	1998	12	0.32
South Asia	Girls	1999	12	0.33
South Asia	Girls	2000	12	0.33
South Asia	Girls	2001	12	0.34
South Asia	Girls	2002	12	0.35
South Asia	Girls	2003	12	0.36
South Asia	Girls	2004	12	0.37
South Asia	Girls	2005	12	0.38
South Asia	Girls	2006	12	0.39
South Asia	Girls	2007	12	0.40
South Asia	Girls	2008	12	0.41
South Asia	Girls	2009	12	0.43
South Asia	Girls	2010	12	0.44
South Asia	Girls	2011	12	0.46
South Asia	Girls	2012	12	0.48
South Asia	Girls	2013	12	0.49
South Asia	Girls	2014	12	0.51
South Asia	Girls	2015	12	0.53
South Asia	Girls	2016	12	0.56
South Asia	Girls	1975	13	0.35
South Asia	Girls	1976	13	0.35
South Asia	Girls	1977	13	0.35
South Asia	Girls	1978	13	0.36
South Asia	Girls	1979	13	0.36
South Asia	Girls	1980	13	0.36
South Asia	Girls	1981	13	0.37
South Asia	Girls	1982	13	0.37
South Asia	Girls	1983	13	0.37
South Asia	Girls	1984	13	0.38
South Asia	Girls	1985	13	0.38
South Asia	Girls	1986	13	0.39

South Asia	Girls	1987	13	0.39
South Asia	Girls	1988	13	0.40
South Asia	Girls	1989	13	0.40
South Asia	Girls	1990	13	0.41
South Asia	Girls	1991	13	0.41
South Asia	Girls	1992	13	0.42
South Asia	Girls	1993	13	0.42
South Asia	Girls	1994	13	0.43
South Asia	Girls	1995	13	0.44
South Asia	Girls	1996	13	0.45
South Asia	Girls	1997	13	0.45
South Asia	Girls	1998	13	0.46
South Asia	Girls	1999	13	0.47
South Asia	Girls	2000	13	0.48
South Asia	Girls	2001	13	0.49
South Asia	Girls	2002	13	0.50
South Asia	Girls	2003	13	0.52
South Asia	Girls	2004	13	0.53
South Asia	Girls	2005	13	0.54
South Asia	Girls	2006	13	0.56
South Asia	Girls	2007	13	0.58
South Asia	Girls	2008	13	0.59
South Asia	Girls	2009	13	0.61
South Asia	Girls	2010	13	0.63
South Asia	Girls	2011	13	0.65
South Asia	Girls	2012	13	0.68
South Asia	Girls	2013	13	0.70
South Asia	Girls	2014	13	0.73
South Asia	Girls	2015	13	0.75
South Asia	Girls	2016	13	0.79
South Asia	Girls	1975	14	0.49
South Asia	Girls	1976	14	0.49
South Asia	Girls	1977	14	0.50
South Asia	Girls	1978	14	0.50
South Asia	Girls	1979	14	0.50
South Asia	Girls	1980	14	0.51
South Asia	Girls	1981	14	0.51
South Asia	Girls	1982	14	0.52
South Asia	Girls	1983	14	0.52
South Asia	Girls	1984	14	0.53
South Asia	Girls	1985	14	0.54
South Asia	Girls	1986	14	0.54
South Asia	Girls	1987	14	0.55
South Asia	Girls	1988	14	0.55
South Asia	Girls	1989	14	0.56
South Asia	Girls	1990	14	0.57
South Asia	Girls	1991	14	0.58
South Asia	Girls	1992	14	0.58
South Asia	Girls	1993	14	0.59
South Asia	Girls	1994	14	0.60
South Asia	Girls	1995	14	0.61
South Asia	Girls	1996	14	0.62
South Asia	Girls	1997	14	0.63
South Asia	Girls	1998	14	0.64
South Asia	Girls	1999	14	0.66
South Asia	Girls	2000	14	0.67
South Asia	Girls	2001	14	0.68
South Asia	Girls	2002	14	0.70
South Asia	Girls	2003	14	0.71

South Asia	Girls	2004	14	0.73
South Asia	Girls	2005	14	0.75
South Asia	Girls	2006	14	0.77
South Asia	Girls	2007	14	0.79
South Asia	Girls	2008	14	0.81
South Asia	Girls	2009	14	0.83
South Asia	Girls	2010	14	0.86
South Asia	Girls	2011	14	0.89
South Asia	Girls	2012	14	0.91
South Asia	Girls	2013	14	0.95
South Asia	Girls	2014	14	0.98
South Asia	Girls	2015	14	1.01
South Asia	Girls	2016	14	1.05
South Asia	Girls	1975	15	0.64
South Asia	Girls	1976	15	0.65
South Asia	Girls	1977	15	0.65
South Asia	Girls	1978	15	0.66
South Asia	Girls	1979	15	0.67
South Asia	Girls	1980	15	0.67
South Asia	Girls	1981	15	0.68
South Asia	Girls	1982	15	0.68
South Asia	Girls	1983	15	0.69
South Asia	Girls	1984	15	0.70
South Asia	Girls	1985	15	0.70
South Asia	Girls	1986	15	0.71
South Asia	Girls	1987	15	0.72
South Asia	Girls	1988	15	0.73
South Asia	Girls	1989	15	0.73
South Asia	Girls	1990	15	0.74
South Asia	Girls	1991	15	0.75
South Asia	Girls	1992	15	0.76
South Asia	Girls	1993	15	0.77
South Asia	Girls	1994	15	0.78
South Asia	Girls	1995	15	0.79
South Asia	Girls	1996	15	0.81
South Asia	Girls	1997	15	0.82
South Asia	Girls	1998	15	0.83
South Asia	Girls	1999	15	0.85
South Asia	Girls	2000	15	0.86
South Asia	Girls	2001	15	0.88
South Asia	Girls	2002	15	0.90
South Asia	Girls	2003	15	0.92
South Asia	Girls	2004	15	0.94
South Asia	Girls	2005	15	0.96
South Asia	Girls	2006	15	0.98
South Asia	Girls	2007	15	1.01
South Asia	Girls	2008	15	1.03
South Asia	Girls	2009	15	1.06
South Asia	Girls	2010	15	1.09
South Asia	Girls	2011	15	1.12
South Asia	Girls	2012	15	1.16
South Asia	Girls	2013	15	1.19
South Asia	Girls	2014	15	1.23
South Asia	Girls	2015	15	1.28
South Asia	Girls	2016	15	1.32
South Asia	Girls	1975	16	0.79
South Asia	Girls	1976	16	0.79
South Asia	Girls	1977	16	0.80
South Asia	Girls	1978	16	0.80

South Asia	Girls	1979	16	0.81
South Asia	Girls	1980	16	0.82
South Asia	Girls	1981	16	0.83
South Asia	Girls	1982	16	0.83
South Asia	Girls	1983	16	0.84
South Asia	Girls	1984	16	0.85
South Asia	Girls	1985	16	0.86
South Asia	Girls	1986	16	0.86
South Asia	Girls	1987	16	0.87
South Asia	Girls	1988	16	0.88
South Asia	Girls	1989	16	0.89
South Asia	Girls	1990	16	0.90
South Asia	Girls	1991	16	0.91
South Asia	Girls	1992	16	0.92
South Asia	Girls	1993	16	0.94
South Asia	Girls	1994	16	0.95
South Asia	Girls	1995	16	0.96
South Asia	Girls	1996	16	0.98
South Asia	Girls	1997	16	0.99
South Asia	Girls	1998	16	1.01
South Asia	Girls	1999	16	1.02
South Asia	Girls	2000	16	1.04
South Asia	Girls	2001	16	1.06
South Asia	Girls	2002	16	1.08
South Asia	Girls	2003	16	1.10
South Asia	Girls	2004	16	1.12
South Asia	Girls	2005	16	1.15
South Asia	Girls	2006	16	1.18
South Asia	Girls	2007	16	1.20
South Asia	Girls	2008	16	1.23
South Asia	Girls	2009	16	1.26
South Asia	Girls	2010	16	1.30
South Asia	Girls	2011	16	1.33
South Asia	Girls	2012	16	1.37
South Asia	Girls	2013	16	1.41
South Asia	Girls	2014	16	1.46
South Asia	Girls	2015	16	1.50
South Asia	Girls	2016	16	1.55
South Asia	Girls	1975	17	0.89
South Asia	Girls	1976	17	0.90
South Asia	Girls	1977	17	0.90
South Asia	Girls	1978	17	0.91
South Asia	Girls	1979	17	0.92
South Asia	Girls	1980	17	0.93
South Asia	Girls	1981	17	0.93
South Asia	Girls	1982	17	0.94
South Asia	Girls	1983	17	0.95
South Asia	Girls	1984	17	0.96
South Asia	Girls	1985	17	0.97
South Asia	Girls	1986	17	0.98
South Asia	Girls	1987	17	0.99
South Asia	Girls	1988	17	1.00
South Asia	Girls	1989	17	1.01
South Asia	Girls	1990	17	1.02
South Asia	Girls	1991	17	1.03
South Asia	Girls	1992	17	1.04
South Asia	Girls	1993	17	1.06
South Asia	Girls	1994	17	1.07
South Asia	Girls	1995	17	1.09

South Asia	Girls	1996	17	1.10
South Asia	Girls	1997	17	1.12
South Asia	Girls	1998	17	1.13
South Asia	Girls	1999	17	1.15
South Asia	Girls	2000	17	1.17
South Asia	Girls	2001	17	1.19
South Asia	Girls	2002	17	1.22
South Asia	Girls	2003	17	1.24
South Asia	Girls	2004	17	1.27
South Asia	Girls	2005	17	1.29
South Asia	Girls	2006	17	1.32
South Asia	Girls	2007	17	1.35
South Asia	Girls	2008	17	1.39
South Asia	Girls	2009	17	1.42
South Asia	Girls	2010	17	1.46
South Asia	Girls	2011	17	1.50
South Asia	Girls	2012	17	1.54
South Asia	Girls	2013	17	1.59
South Asia	Girls	2014	17	1.63
South Asia	Girls	2015	17	1.69
South Asia	Girls	2016	17	1.74
South Asia	Girls	1975	18	0.95
South Asia	Girls	1976	18	0.96
South Asia	Girls	1977	18	0.96
South Asia	Girls	1978	18	0.97
South Asia	Girls	1979	18	0.98
South Asia	Girls	1980	18	0.99
South Asia	Girls	1981	18	1.00
South Asia	Girls	1982	18	1.01
South Asia	Girls	1983	18	1.02
South Asia	Girls	1984	18	1.03
South Asia	Girls	1985	18	1.04
South Asia	Girls	1986	18	1.05
South Asia	Girls	1987	18	1.06
South Asia	Girls	1988	18	1.07
South Asia	Girls	1989	18	1.08
South Asia	Girls	1990	18	1.09
South Asia	Girls	1991	18	1.11
South Asia	Girls	1992	18	1.12
South Asia	Girls	1993	18	1.13
South Asia	Girls	1994	18	1.15
South Asia	Girls	1995	18	1.17
South Asia	Girls	1996	18	1.18
South Asia	Girls	1997	18	1.20
South Asia	Girls	1998	18	1.22
South Asia	Girls	1999	18	1.24
South Asia	Girls	2000	18	1.26
South Asia	Girls	2001	18	1.29
South Asia	Girls	2002	18	1.31
South Asia	Girls	2003	18	1.34
South Asia	Girls	2004	18	1.37
South Asia	Girls	2005	18	1.40
South Asia	Girls	2006	18	1.43
South Asia	Girls	2007	18	1.47
South Asia	Girls	2008	18	1.50
South Asia	Girls	2009	18	1.54
South Asia	Girls	2010	18	1.59
South Asia	Girls	2011	18	1.63
South Asia	Girls	2012	18	1.68

South Asia	Girls	2013	18	1.73
South Asia	Girls	2014	18	1.79
South Asia	Girls	2015	18	1.85
South Asia	Girls	2016	18	1.91
South Asia	Girls	1975	Average	0.62
South Asia	Girls	1976	Average	0.63
South Asia	Girls	1977	Average	0.63
South Asia	Girls	1978	Average	0.64
South Asia	Girls	1979	Average	0.64
South Asia	Girls	1980	Average	0.65
South Asia	Girls	1981	Average	0.65
South Asia	Girls	1982	Average	0.66
South Asia	Girls	1983	Average	0.66
South Asia	Girls	1984	Average	0.67
South Asia	Girls	1985	Average	0.68
South Asia	Girls	1986	Average	0.68
South Asia	Girls	1987	Average	0.69
South Asia	Girls	1988	Average	0.70
South Asia	Girls	1989	Average	0.71
South Asia	Girls	1990	Average	0.72
South Asia	Girls	1991	Average	0.72
South Asia	Girls	1992	Average	0.73
South Asia	Girls	1993	Average	0.74
South Asia	Girls	1994	Average	0.75
South Asia	Girls	1995	Average	0.77
South Asia	Girls	1996	Average	0.78
South Asia	Girls	1997	Average	0.79
South Asia	Girls	1998	Average	0.80
South Asia	Girls	1999	Average	0.82
South Asia	Girls	2000	Average	0.83
South Asia	Girls	2001	Average	0.85
South Asia	Girls	2002	Average	0.87
South Asia	Girls	2003	Average	0.88
South Asia	Girls	2004	Average	0.90
South Asia	Girls	2005	Average	0.93
South Asia	Girls	2006	Average	0.95
South Asia	Girls	2007	Average	0.97
South Asia	Girls	2008	Average	1.00
South Asia	Girls	2009	Average	1.02
South Asia	Girls	2010	Average	1.05
South Asia	Girls	2011	Average	1.08
South Asia	Girls	2012	Average	1.12
South Asia	Girls	2013	Average	1.15
South Asia	Girls	2014	Average	1.19
South Asia	Girls	2015	Average	1.23
South Asia	Girls	2016	Average	1.27
South Asia	Girls	2017	Average	1.32
South Asia	Girls	2018	Average	1.36
South Asia	Girls	2019	Average	1.40
South Asia	Girls	2020	Average	1.45
South Asia	Girls	2021	Average	1.49
South Asia	Girls	2022	Average	1.54
South Asia	Girls	2023	Average	1.58
South Asia	Girls	2024	Average	1.62
South Asia	Girls	2025	Average	1.67
South Asia	Girls	2026	Average	1.71
South Asia	Girls	2027	Average	1.75
South Asia	Girls	2028	Average	1.80
South Asia	Girls	2029	Average	1.84

South Asia	Girls	2030	Average	1.88
Sub-Saharan Africa	Boys	1975	12	0.31
Sub-Saharan Africa	Boys	1976	12	0.31
Sub-Saharan Africa	Boys	1977	12	0.31
Sub-Saharan Africa	Boys	1978	12	0.31
Sub-Saharan Africa	Boys	1979	12	0.32
Sub-Saharan Africa	Boys	1980	12	0.32
Sub-Saharan Africa	Boys	1981	12	0.32
Sub-Saharan Africa	Boys	1982	12	0.32
Sub-Saharan Africa	Boys	1983	12	0.32
Sub-Saharan Africa	Boys	1984	12	0.33
Sub-Saharan Africa	Boys	1985	12	0.33
Sub-Saharan Africa	Boys	1986	12	0.33
Sub-Saharan Africa	Boys	1987	12	0.34
Sub-Saharan Africa	Boys	1988	12	0.34
Sub-Saharan Africa	Boys	1989	12	0.34
Sub-Saharan Africa	Boys	1990	12	0.35
Sub-Saharan Africa	Boys	1991	12	0.35
Sub-Saharan Africa	Boys	1992	12	0.35
Sub-Saharan Africa	Boys	1993	12	0.36
Sub-Saharan Africa	Boys	1994	12	0.36
Sub-Saharan Africa	Boys	1995	12	0.37
Sub-Saharan Africa	Boys	1996	12	0.37
Sub-Saharan Africa	Boys	1997	12	0.38
Sub-Saharan Africa	Boys	1998	12	0.39
Sub-Saharan Africa	Boys	1999	12	0.39
Sub-Saharan Africa	Boys	2000	12	0.40
Sub-Saharan Africa	Boys	2001	12	0.41
Sub-Saharan Africa	Boys	2002	12	0.41
Sub-Saharan Africa	Boys	2003	12	0.42
Sub-Saharan Africa	Boys	2004	12	0.43
Sub-Saharan Africa	Boys	2005	12	0.44
Sub-Saharan Africa	Boys	2006	12	0.45
Sub-Saharan Africa	Boys	2007	12	0.46
Sub-Saharan Africa	Boys	2008	12	0.47
Sub-Saharan Africa	Boys	2009	12	0.48
Sub-Saharan Africa	Boys	2010	12	0.50
Sub-Saharan Africa	Boys	2011	12	0.51
Sub-Saharan Africa	Boys	2012	12	0.52
Sub-Saharan Africa	Boys	2013	12	0.54
Sub-Saharan Africa	Boys	2014	12	0.55
Sub-Saharan Africa	Boys	2015	12	0.57
Sub-Saharan Africa	Boys	2016	12	0.59
Sub-Saharan Africa	Boys	1975	13	0.41
Sub-Saharan Africa	Boys	1976	13	0.41
Sub-Saharan Africa	Boys	1977	13	0.41
Sub-Saharan Africa	Boys	1978	13	0.42
Sub-Saharan Africa	Boys	1979	13	0.42
Sub-Saharan Africa	Boys	1980	13	0.42
Sub-Saharan Africa	Boys	1981	13	0.42
Sub-Saharan Africa	Boys	1982	13	0.43
Sub-Saharan Africa	Boys	1983	13	0.43
Sub-Saharan Africa	Boys	1984	13	0.43
Sub-Saharan Africa	Boys	1985	13	0.44
Sub-Saharan Africa	Boys	1986	13	0.44
Sub-Saharan Africa	Boys	1987	13	0.44
Sub-Saharan Africa	Boys	1988	13	0.45
Sub-Saharan Africa	Boys	1989	13	0.45
Sub-Saharan Africa	Boys	1990	13	0.46

Sub-Saharan Africa	Boys	1991	13	0.46
Sub-Saharan Africa	Boys	1992	13	0.47
Sub-Saharan Africa	Boys	1993	13	0.48
Sub-Saharan Africa	Boys	1994	13	0.48
Sub-Saharan Africa	Boys	1995	13	0.49
Sub-Saharan Africa	Boys	1996	13	0.49
Sub-Saharan Africa	Boys	1997	13	0.50
Sub-Saharan Africa	Boys	1998	13	0.51
Sub-Saharan Africa	Boys	1999	13	0.52
Sub-Saharan Africa	Boys	2000	13	0.53
Sub-Saharan Africa	Boys	2001	13	0.54
Sub-Saharan Africa	Boys	2002	13	0.55
Sub-Saharan Africa	Boys	2003	13	0.56
Sub-Saharan Africa	Boys	2004	13	0.57
Sub-Saharan Africa	Boys	2005	13	0.58
Sub-Saharan Africa	Boys	2006	13	0.60
Sub-Saharan Africa	Boys	2007	13	0.61
Sub-Saharan Africa	Boys	2008	13	0.63
Sub-Saharan Africa	Boys	2009	13	0.64
Sub-Saharan Africa	Boys	2010	13	0.66
Sub-Saharan Africa	Boys	2011	13	0.67
Sub-Saharan Africa	Boys	2012	13	0.69
Sub-Saharan Africa	Boys	2013	13	0.71
Sub-Saharan Africa	Boys	2014	13	0.73
Sub-Saharan Africa	Boys	2015	13	0.76
Sub-Saharan Africa	Boys	2016	13	0.78
Sub-Saharan Africa	Boys	1975	14	0.55
Sub-Saharan Africa	Boys	1976	14	0.55
Sub-Saharan Africa	Boys	1977	14	0.56
Sub-Saharan Africa	Boys	1978	14	0.56
Sub-Saharan Africa	Boys	1979	14	0.56
Sub-Saharan Africa	Boys	1980	14	0.57
Sub-Saharan Africa	Boys	1981	14	0.57
Sub-Saharan Africa	Boys	1982	14	0.57
Sub-Saharan Africa	Boys	1983	14	0.58
Sub-Saharan Africa	Boys	1984	14	0.58
Sub-Saharan Africa	Boys	1985	14	0.59
Sub-Saharan Africa	Boys	1986	14	0.59
Sub-Saharan Africa	Boys	1987	14	0.60
Sub-Saharan Africa	Boys	1988	14	0.60
Sub-Saharan Africa	Boys	1989	14	0.61
Sub-Saharan Africa	Boys	1990	14	0.61
Sub-Saharan Africa	Boys	1991	14	0.62
Sub-Saharan Africa	Boys	1992	14	0.63
Sub-Saharan Africa	Boys	1993	14	0.64
Sub-Saharan Africa	Boys	1994	14	0.64
Sub-Saharan Africa	Boys	1995	14	0.65
Sub-Saharan Africa	Boys	1996	14	0.66
Sub-Saharan Africa	Boys	1997	14	0.67
Sub-Saharan Africa	Boys	1998	14	0.68
Sub-Saharan Africa	Boys	1999	14	0.69
Sub-Saharan Africa	Boys	2000	14	0.70
Sub-Saharan Africa	Boys	2001	14	0.72
Sub-Saharan Africa	Boys	2002	14	0.73
Sub-Saharan Africa	Boys	2003	14	0.74
Sub-Saharan Africa	Boys	2004	14	0.76
Sub-Saharan Africa	Boys	2005	14	0.77
Sub-Saharan Africa	Boys	2006	14	0.79
Sub-Saharan Africa	Boys	2007	14	0.81

Sub-Saharan Africa	Boys	2008	14	0.83
Sub-Saharan Africa	Boys	2009	14	0.85
Sub-Saharan Africa	Boys	2010	14	0.87
Sub-Saharan Africa	Boys	2011	14	0.89
Sub-Saharan Africa	Boys	2012	14	0.91
Sub-Saharan Africa	Boys	2013	14	0.94
Sub-Saharan Africa	Boys	2014	14	0.97
Sub-Saharan Africa	Boys	2015	14	1.00
Sub-Saharan Africa	Boys	2016	14	1.03
Sub-Saharan Africa	Boys	1975	15	0.73
Sub-Saharan Africa	Boys	1976	15	0.74
Sub-Saharan Africa	Boys	1977	15	0.74
Sub-Saharan Africa	Boys	1978	15	0.74
Sub-Saharan Africa	Boys	1979	15	0.75
Sub-Saharan Africa	Boys	1980	15	0.75
Sub-Saharan Africa	Boys	1981	15	0.76
Sub-Saharan Africa	Boys	1982	15	0.76
Sub-Saharan Africa	Boys	1983	15	0.77
Sub-Saharan Africa	Boys	1984	15	0.77
Sub-Saharan Africa	Boys	1985	15	0.78
Sub-Saharan Africa	Boys	1986	15	0.79
Sub-Saharan Africa	Boys	1987	15	0.79
Sub-Saharan Africa	Boys	1988	15	0.80
Sub-Saharan Africa	Boys	1989	15	0.81
Sub-Saharan Africa	Boys	1990	15	0.81
Sub-Saharan Africa	Boys	1991	15	0.82
Sub-Saharan Africa	Boys	1992	15	0.83
Sub-Saharan Africa	Boys	1993	15	0.84
Sub-Saharan Africa	Boys	1994	15	0.85
Sub-Saharan Africa	Boys	1995	15	0.86
Sub-Saharan Africa	Boys	1996	15	0.87
Sub-Saharan Africa	Boys	1997	15	0.88
Sub-Saharan Africa	Boys	1998	15	0.90
Sub-Saharan Africa	Boys	1999	15	0.91
Sub-Saharan Africa	Boys	2000	15	0.92
Sub-Saharan Africa	Boys	2001	15	0.94
Sub-Saharan Africa	Boys	2002	15	0.96
Sub-Saharan Africa	Boys	2003	15	0.97
Sub-Saharan Africa	Boys	2004	15	0.99
Sub-Saharan Africa	Boys	2005	15	1.01
Sub-Saharan Africa	Boys	2006	15	1.03
Sub-Saharan Africa	Boys	2007	15	1.06
Sub-Saharan Africa	Boys	2008	15	1.08
Sub-Saharan Africa	Boys	2009	15	1.11
Sub-Saharan Africa	Boys	2010	15	1.13
Sub-Saharan Africa	Boys	2011	15	1.16
Sub-Saharan Africa	Boys	2012	15	1.19
Sub-Saharan Africa	Boys	2013	15	1.22
Sub-Saharan Africa	Boys	2014	15	1.25
Sub-Saharan Africa	Boys	2015	15	1.29
Sub-Saharan Africa	Boys	2016	15	1.32
Sub-Saharan Africa	Boys	1975	16	0.95
Sub-Saharan Africa	Boys	1976	16	0.96
Sub-Saharan Africa	Boys	1977	16	0.96
Sub-Saharan Africa	Boys	1978	16	0.97
Sub-Saharan Africa	Boys	1979	16	0.97
Sub-Saharan Africa	Boys	1980	16	0.98
Sub-Saharan Africa	Boys	1981	16	0.98
Sub-Saharan Africa	Boys	1982	16	0.99

Sub-Saharan Africa	Boys	1983	16	1.00
Sub-Saharan Africa	Boys	1984	16	1.00
Sub-Saharan Africa	Boys	1985	16	1.01
Sub-Saharan Africa	Boys	1986	16	1.02
Sub-Saharan Africa	Boys	1987	16	1.03
Sub-Saharan Africa	Boys	1988	16	1.04
Sub-Saharan Africa	Boys	1989	16	1.04
Sub-Saharan Africa	Boys	1990	16	1.05
Sub-Saharan Africa	Boys	1991	16	1.06
Sub-Saharan Africa	Boys	1992	16	1.07
Sub-Saharan Africa	Boys	1993	16	1.09
Sub-Saharan Africa	Boys	1994	16	1.10
Sub-Saharan Africa	Boys	1995	16	1.11
Sub-Saharan Africa	Boys	1996	16	1.12
Sub-Saharan Africa	Boys	1997	16	1.14
Sub-Saharan Africa	Boys	1998	16	1.15
Sub-Saharan Africa	Boys	1999	16	1.17
Sub-Saharan Africa	Boys	2000	16	1.19
Sub-Saharan Africa	Boys	2001	16	1.20
Sub-Saharan Africa	Boys	2002	16	1.22
Sub-Saharan Africa	Boys	2003	16	1.24
Sub-Saharan Africa	Boys	2004	16	1.27
Sub-Saharan Africa	Boys	2005	16	1.29
Sub-Saharan Africa	Boys	2006	16	1.32
Sub-Saharan Africa	Boys	2007	16	1.34
Sub-Saharan Africa	Boys	2008	16	1.37
Sub-Saharan Africa	Boys	2009	16	1.40
Sub-Saharan Africa	Boys	2010	16	1.43
Sub-Saharan Africa	Boys	2011	16	1.46
Sub-Saharan Africa	Boys	2012	16	1.50
Sub-Saharan Africa	Boys	2013	16	1.53
Sub-Saharan Africa	Boys	2014	16	1.57
Sub-Saharan Africa	Boys	2015	16	1.61
Sub-Saharan Africa	Boys	2016	16	1.66
Sub-Saharan Africa	Boys	1975	17	1.20
Sub-Saharan Africa	Boys	1976	17	1.21
Sub-Saharan Africa	Boys	1977	17	1.21
Sub-Saharan Africa	Boys	1978	17	1.22
Sub-Saharan Africa	Boys	1979	17	1.23
Sub-Saharan Africa	Boys	1980	17	1.24
Sub-Saharan Africa	Boys	1981	17	1.24
Sub-Saharan Africa	Boys	1982	17	1.25
Sub-Saharan Africa	Boys	1983	17	1.26
Sub-Saharan Africa	Boys	1984	17	1.27
Sub-Saharan Africa	Boys	1985	17	1.27
Sub-Saharan Africa	Boys	1986	17	1.28
Sub-Saharan Africa	Boys	1987	17	1.29
Sub-Saharan Africa	Boys	1988	17	1.30
Sub-Saharan Africa	Boys	1989	17	1.31
Sub-Saharan Africa	Boys	1990	17	1.33
Sub-Saharan Africa	Boys	1991	17	1.34
Sub-Saharan Africa	Boys	1992	17	1.35
Sub-Saharan Africa	Boys	1993	17	1.36
Sub-Saharan Africa	Boys	1994	17	1.38
Sub-Saharan Africa	Boys	1995	17	1.39
Sub-Saharan Africa	Boys	1996	17	1.41
Sub-Saharan Africa	Boys	1997	17	1.42
Sub-Saharan Africa	Boys	1998	17	1.44
Sub-Saharan Africa	Boys	1999	17	1.46

Sub-Saharan Africa	Boys	2000	17	1.48
Sub-Saharan Africa	Boys	2001	17	1.50
Sub-Saharan Africa	Boys	2002	17	1.52
Sub-Saharan Africa	Boys	2003	17	1.55
Sub-Saharan Africa	Boys	2004	17	1.57
Sub-Saharan Africa	Boys	2005	17	1.60
Sub-Saharan Africa	Boys	2006	17	1.63
Sub-Saharan Africa	Boys	2007	17	1.66
Sub-Saharan Africa	Boys	2008	17	1.70
Sub-Saharan Africa	Boys	2009	17	1.73
Sub-Saharan Africa	Boys	2010	17	1.77
Sub-Saharan Africa	Boys	2011	17	1.81
Sub-Saharan Africa	Boys	2012	17	1.84
Sub-Saharan Africa	Boys	2013	17	1.89
Sub-Saharan Africa	Boys	2014	17	1.93
Sub-Saharan Africa	Boys	2015	17	1.98
Sub-Saharan Africa	Boys	2016	17	2.03
Sub-Saharan Africa	Boys	1975	18	1.47
Sub-Saharan Africa	Boys	1976	18	1.48
Sub-Saharan Africa	Boys	1977	18	1.48
Sub-Saharan Africa	Boys	1978	18	1.49
Sub-Saharan Africa	Boys	1979	18	1.50
Sub-Saharan Africa	Boys	1980	18	1.51
Sub-Saharan Africa	Boys	1981	18	1.52
Sub-Saharan Africa	Boys	1982	18	1.53
Sub-Saharan Africa	Boys	1983	18	1.54
Sub-Saharan Africa	Boys	1984	18	1.55
Sub-Saharan Africa	Boys	1985	18	1.56
Sub-Saharan Africa	Boys	1986	18	1.57
Sub-Saharan Africa	Boys	1987	18	1.58
Sub-Saharan Africa	Boys	1988	18	1.59
Sub-Saharan Africa	Boys	1989	18	1.61
Sub-Saharan Africa	Boys	1990	18	1.62
Sub-Saharan Africa	Boys	1991	18	1.63
Sub-Saharan Africa	Boys	1992	18	1.65
Sub-Saharan Africa	Boys	1993	18	1.66
Sub-Saharan Africa	Boys	1994	18	1.68
Sub-Saharan Africa	Boys	1995	18	1.70
Sub-Saharan Africa	Boys	1996	18	1.72
Sub-Saharan Africa	Boys	1997	18	1.74
Sub-Saharan Africa	Boys	1998	18	1.76
Sub-Saharan Africa	Boys	1999	18	1.78
Sub-Saharan Africa	Boys	2000	18	1.80
Sub-Saharan Africa	Boys	2001	18	1.83
Sub-Saharan Africa	Boys	2002	18	1.85
Sub-Saharan Africa	Boys	2003	18	1.88
Sub-Saharan Africa	Boys	2004	18	1.91
Sub-Saharan Africa	Boys	2005	18	1.95
Sub-Saharan Africa	Boys	2006	18	1.98
Sub-Saharan Africa	Boys	2007	18	2.02
Sub-Saharan Africa	Boys	2008	18	2.06
Sub-Saharan Africa	Boys	2009	18	2.10
Sub-Saharan Africa	Boys	2010	18	2.14
Sub-Saharan Africa	Boys	2011	18	2.18
Sub-Saharan Africa	Boys	2012	18	2.23
Sub-Saharan Africa	Boys	2013	18	2.28
Sub-Saharan Africa	Boys	2014	18	2.33
Sub-Saharan Africa	Boys	2015	18	2.38
Sub-Saharan Africa	Boys	2016	18	2.44

Sub-Saharan Africa	Boys	1975	Average	0.80
Sub-Saharan Africa	Boys	1976	Average	0.81
Sub-Saharan Africa	Boys	1977	Average	0.81
Sub-Saharan Africa	Boys	1978	Average	0.82
Sub-Saharan Africa	Boys	1979	Average	0.82
Sub-Saharan Africa	Boys	1980	Average	0.83
Sub-Saharan Africa	Boys	1981	Average	0.83
Sub-Saharan Africa	Boys	1982	Average	0.84
Sub-Saharan Africa	Boys	1983	Average	0.84
Sub-Saharan Africa	Boys	1984	Average	0.85
Sub-Saharan Africa	Boys	1985	Average	0.85
Sub-Saharan Africa	Boys	1986	Average	0.86
Sub-Saharan Africa	Boys	1987	Average	0.87
Sub-Saharan Africa	Boys	1988	Average	0.87
Sub-Saharan Africa	Boys	1989	Average	0.88
Sub-Saharan Africa	Boys	1990	Average	0.89
Sub-Saharan Africa	Boys	1991	Average	0.90
Sub-Saharan Africa	Boys	1992	Average	0.91
Sub-Saharan Africa	Boys	1993	Average	0.92
Sub-Saharan Africa	Boys	1994	Average	0.93
Sub-Saharan Africa	Boys	1995	Average	0.94
Sub-Saharan Africa	Boys	1996	Average	0.95
Sub-Saharan Africa	Boys	1997	Average	0.96
Sub-Saharan Africa	Boys	1998	Average	0.97
Sub-Saharan Africa	Boys	1999	Average	0.99
Sub-Saharan Africa	Boys	2000	Average	1.00
Sub-Saharan Africa	Boys	2001	Average	1.02
Sub-Saharan Africa	Boys	2002	Average	1.04
Sub-Saharan Africa	Boys	2003	Average	1.05
Sub-Saharan Africa	Boys	2004	Average	1.07
Sub-Saharan Africa	Boys	2005	Average	1.09
Sub-Saharan Africa	Boys	2006	Average	1.11
Sub-Saharan Africa	Boys	2007	Average	1.14
Sub-Saharan Africa	Boys	2008	Average	1.16
Sub-Saharan Africa	Boys	2009	Average	1.19
Sub-Saharan Africa	Boys	2010	Average	1.21
Sub-Saharan Africa	Boys	2011	Average	1.24
Sub-Saharan Africa	Boys	2012	Average	1.27
Sub-Saharan Africa	Boys	2013	Average	1.30
Sub-Saharan Africa	Boys	2014	Average	1.33
Sub-Saharan Africa	Boys	2015	Average	1.37
Sub-Saharan Africa	Boys	2016	Average	1.41
Sub-Saharan Africa	Boys	2017	Average	1.44
Sub-Saharan Africa	Boys	2018	Average	1.48
Sub-Saharan Africa	Boys	2019	Average	1.52
Sub-Saharan Africa	Boys	2020	Average	1.56
Sub-Saharan Africa	Boys	2021	Average	1.59
Sub-Saharan Africa	Boys	2022	Average	1.63
Sub-Saharan Africa	Boys	2023	Average	1.67
Sub-Saharan Africa	Boys	2024	Average	1.71
Sub-Saharan Africa	Boys	2025	Average	1.74
Sub-Saharan Africa	Boys	2026	Average	1.78
Sub-Saharan Africa	Boys	2027	Average	1.82
Sub-Saharan Africa	Boys	2028	Average	1.86
Sub-Saharan Africa	Boys	2029	Average	1.89
Sub-Saharan Africa	Boys	2030	Average	1.93
Sub-Saharan Africa	Girls	1975	12	0.37
Sub-Saharan Africa	Girls	1976	12	0.37
Sub-Saharan Africa	Girls	1977	12	0.37

Sub-Saharan Africa	Girls	1978	12	0.38
Sub-Saharan Africa	Girls	1979	12	0.39
Sub-Saharan Africa	Girls	1980	12	0.39
Sub-Saharan Africa	Girls	1981	12	0.40
Sub-Saharan Africa	Girls	1982	12	0.40
Sub-Saharan Africa	Girls	1983	12	0.41
Sub-Saharan Africa	Girls	1984	12	0.42
Sub-Saharan Africa	Girls	1985	12	0.43
Sub-Saharan Africa	Girls	1986	12	0.43
Sub-Saharan Africa	Girls	1987	12	0.44
Sub-Saharan Africa	Girls	1988	12	0.45
Sub-Saharan Africa	Girls	1989	12	0.46
Sub-Saharan Africa	Girls	1990	12	0.47
Sub-Saharan Africa	Girls	1991	12	0.48
Sub-Saharan Africa	Girls	1992	12	0.49
Sub-Saharan Africa	Girls	1993	12	0.51
Sub-Saharan Africa	Girls	1994	12	0.52
Sub-Saharan Africa	Girls	1995	12	0.53
Sub-Saharan Africa	Girls	1996	12	0.55
Sub-Saharan Africa	Girls	1997	12	0.56
Sub-Saharan Africa	Girls	1998	12	0.58
Sub-Saharan Africa	Girls	1999	12	0.59
Sub-Saharan Africa	Girls	2000	12	0.61
Sub-Saharan Africa	Girls	2001	12	0.63
Sub-Saharan Africa	Girls	2002	12	0.65
Sub-Saharan Africa	Girls	2003	12	0.67
Sub-Saharan Africa	Girls	2004	12	0.69
Sub-Saharan Africa	Girls	2005	12	0.71
Sub-Saharan Africa	Girls	2006	12	0.73
Sub-Saharan Africa	Girls	2007	12	0.75
Sub-Saharan Africa	Girls	2008	12	0.77
Sub-Saharan Africa	Girls	2009	12	0.80
Sub-Saharan Africa	Girls	2010	12	0.82
Sub-Saharan Africa	Girls	2011	12	0.85
Sub-Saharan Africa	Girls	2012	12	0.87
Sub-Saharan Africa	Girls	2013	12	0.90
Sub-Saharan Africa	Girls	2014	12	0.93
Sub-Saharan Africa	Girls	2015	12	0.95
Sub-Saharan Africa	Girls	2016	12	0.98
Sub-Saharan Africa	Girls	1975	13	0.53
Sub-Saharan Africa	Girls	1976	13	0.53
Sub-Saharan Africa	Girls	1977	13	0.54
Sub-Saharan Africa	Girls	1978	13	0.55
Sub-Saharan Africa	Girls	1979	13	0.56
Sub-Saharan Africa	Girls	1980	13	0.56
Sub-Saharan Africa	Girls	1981	13	0.57
Sub-Saharan Africa	Girls	1982	13	0.58
Sub-Saharan Africa	Girls	1983	13	0.59
Sub-Saharan Africa	Girls	1984	13	0.60
Sub-Saharan Africa	Girls	1985	13	0.61
Sub-Saharan Africa	Girls	1986	13	0.62
Sub-Saharan Africa	Girls	1987	13	0.64
Sub-Saharan Africa	Girls	1988	13	0.65
Sub-Saharan Africa	Girls	1989	13	0.66
Sub-Saharan Africa	Girls	1990	13	0.68
Sub-Saharan Africa	Girls	1991	13	0.69
Sub-Saharan Africa	Girls	1992	13	0.71
Sub-Saharan Africa	Girls	1993	13	0.73
Sub-Saharan Africa	Girls	1994	13	0.75

Sub-Saharan Africa	Girls	1995	13	0.76
Sub-Saharan Africa	Girls	1996	13	0.78
Sub-Saharan Africa	Girls	1997	13	0.80
Sub-Saharan Africa	Girls	1998	13	0.83
Sub-Saharan Africa	Girls	1999	13	0.85
Sub-Saharan Africa	Girls	2000	13	0.87
Sub-Saharan Africa	Girls	2001	13	0.90
Sub-Saharan Africa	Girls	2002	13	0.92
Sub-Saharan Africa	Girls	2003	13	0.95
Sub-Saharan Africa	Girls	2004	13	0.98
Sub-Saharan Africa	Girls	2005	13	1.01
Sub-Saharan Africa	Girls	2006	13	1.04
Sub-Saharan Africa	Girls	2007	13	1.07
Sub-Saharan Africa	Girls	2008	13	1.10
Sub-Saharan Africa	Girls	2009	13	1.13
Sub-Saharan Africa	Girls	2010	13	1.16
Sub-Saharan Africa	Girls	2011	13	1.20
Sub-Saharan Africa	Girls	2012	13	1.24
Sub-Saharan Africa	Girls	2013	13	1.27
Sub-Saharan Africa	Girls	2014	13	1.31
Sub-Saharan Africa	Girls	2015	13	1.35
Sub-Saharan Africa	Girls	2016	13	1.39
Sub-Saharan Africa	Girls	1975	14	0.73
Sub-Saharan Africa	Girls	1976	14	0.74
Sub-Saharan Africa	Girls	1977	14	0.75
Sub-Saharan Africa	Girls	1978	14	0.76
Sub-Saharan Africa	Girls	1979	14	0.77
Sub-Saharan Africa	Girls	1980	14	0.78
Sub-Saharan Africa	Girls	1981	14	0.79
Sub-Saharan Africa	Girls	1982	14	0.80
Sub-Saharan Africa	Girls	1983	14	0.82
Sub-Saharan Africa	Girls	1984	14	0.83
Sub-Saharan Africa	Girls	1985	14	0.85
Sub-Saharan Africa	Girls	1986	14	0.86
Sub-Saharan Africa	Girls	1987	14	0.88
Sub-Saharan Africa	Girls	1988	14	0.89
Sub-Saharan Africa	Girls	1989	14	0.91
Sub-Saharan Africa	Girls	1990	14	0.93
Sub-Saharan Africa	Girls	1991	14	0.95
Sub-Saharan Africa	Girls	1992	14	0.97
Sub-Saharan Africa	Girls	1993	14	1.00
Sub-Saharan Africa	Girls	1994	14	1.02
Sub-Saharan Africa	Girls	1995	14	1.04
Sub-Saharan Africa	Girls	1996	14	1.07
Sub-Saharan Africa	Girls	1997	14	1.10
Sub-Saharan Africa	Girls	1998	14	1.12
Sub-Saharan Africa	Girls	1999	14	1.15
Sub-Saharan Africa	Girls	2000	14	1.18
Sub-Saharan Africa	Girls	2001	14	1.22
Sub-Saharan Africa	Girls	2002	14	1.25
Sub-Saharan Africa	Girls	2003	14	1.28
Sub-Saharan Africa	Girls	2004	14	1.32
Sub-Saharan Africa	Girls	2005	14	1.36
Sub-Saharan Africa	Girls	2006	14	1.39
Sub-Saharan Africa	Girls	2007	14	1.43
Sub-Saharan Africa	Girls	2008	14	1.47
Sub-Saharan Africa	Girls	2009	14	1.51
Sub-Saharan Africa	Girls	2010	14	1.56
Sub-Saharan Africa	Girls	2011	14	1.60

Sub-Saharan Africa	Girls	2012	14	1.65
Sub-Saharan Africa	Girls	2013	14	1.70
Sub-Saharan Africa	Girls	2014	14	1.74
Sub-Saharan Africa	Girls	2015	14	1.80
Sub-Saharan Africa	Girls	2016	14	1.85
Sub-Saharan Africa	Girls	1975	15	0.95
Sub-Saharan Africa	Girls	1976	15	0.96
Sub-Saharan Africa	Girls	1977	15	0.97
Sub-Saharan Africa	Girls	1978	15	0.98
Sub-Saharan Africa	Girls	1979	15	1.00
Sub-Saharan Africa	Girls	1980	15	1.01
Sub-Saharan Africa	Girls	1981	15	1.02
Sub-Saharan Africa	Girls	1982	15	1.04
Sub-Saharan Africa	Girls	1983	15	1.05
Sub-Saharan Africa	Girls	1984	15	1.07
Sub-Saharan Africa	Girls	1985	15	1.09
Sub-Saharan Africa	Girls	1986	15	1.11
Sub-Saharan Africa	Girls	1987	15	1.13
Sub-Saharan Africa	Girls	1988	15	1.15
Sub-Saharan Africa	Girls	1989	15	1.17
Sub-Saharan Africa	Girls	1990	15	1.19
Sub-Saharan Africa	Girls	1991	15	1.22
Sub-Saharan Africa	Girls	1992	15	1.24
Sub-Saharan Africa	Girls	1993	15	1.27
Sub-Saharan Africa	Girls	1994	15	1.30
Sub-Saharan Africa	Girls	1995	15	1.33
Sub-Saharan Africa	Girls	1996	15	1.36
Sub-Saharan Africa	Girls	1997	15	1.39
Sub-Saharan Africa	Girls	1998	15	1.43
Sub-Saharan Africa	Girls	1999	15	1.46
Sub-Saharan Africa	Girls	2000	15	1.50
Sub-Saharan Africa	Girls	2001	15	1.54
Sub-Saharan Africa	Girls	2002	15	1.58
Sub-Saharan Africa	Girls	2003	15	1.62
Sub-Saharan Africa	Girls	2004	15	1.66
Sub-Saharan Africa	Girls	2005	15	1.71
Sub-Saharan Africa	Girls	2006	15	1.76
Sub-Saharan Africa	Girls	2007	15	1.80
Sub-Saharan Africa	Girls	2008	15	1.85
Sub-Saharan Africa	Girls	2009	15	1.90
Sub-Saharan Africa	Girls	2010	15	1.96
Sub-Saharan Africa	Girls	2011	15	2.01
Sub-Saharan Africa	Girls	2012	15	2.06
Sub-Saharan Africa	Girls	2013	15	2.12
Sub-Saharan Africa	Girls	2014	15	2.17
Sub-Saharan Africa	Girls	2015	15	2.23
Sub-Saharan Africa	Girls	2016	15	2.29
Sub-Saharan Africa	Girls	1975	16	1.15
Sub-Saharan Africa	Girls	1976	16	1.16
Sub-Saharan Africa	Girls	1977	16	1.17
Sub-Saharan Africa	Girls	1978	16	1.18
Sub-Saharan Africa	Girls	1979	16	1.20
Sub-Saharan Africa	Girls	1980	16	1.21
Sub-Saharan Africa	Girls	1981	16	1.23
Sub-Saharan Africa	Girls	1982	16	1.25
Sub-Saharan Africa	Girls	1983	16	1.27
Sub-Saharan Africa	Girls	1984	16	1.28
Sub-Saharan Africa	Girls	1985	16	1.30
Sub-Saharan Africa	Girls	1986	16	1.33

Sub-Saharan Africa	Girls	1987	16	1.35
Sub-Saharan Africa	Girls	1988	16	1.37
Sub-Saharan Africa	Girls	1989	16	1.40
Sub-Saharan Africa	Girls	1990	16	1.42
Sub-Saharan Africa	Girls	1991	16	1.45
Sub-Saharan Africa	Girls	1992	16	1.48
Sub-Saharan Africa	Girls	1993	16	1.51
Sub-Saharan Africa	Girls	1994	16	1.54
Sub-Saharan Africa	Girls	1995	16	1.58
Sub-Saharan Africa	Girls	1996	16	1.61
Sub-Saharan Africa	Girls	1997	16	1.65
Sub-Saharan Africa	Girls	1998	16	1.69
Sub-Saharan Africa	Girls	1999	16	1.73
Sub-Saharan Africa	Girls	2000	16	1.77
Sub-Saharan Africa	Girls	2001	16	1.81
Sub-Saharan Africa	Girls	2002	16	1.86
Sub-Saharan Africa	Girls	2003	16	1.91
Sub-Saharan Africa	Girls	2004	16	1.96
Sub-Saharan Africa	Girls	2005	16	2.01
Sub-Saharan Africa	Girls	2006	16	2.06
Sub-Saharan Africa	Girls	2007	16	2.11
Sub-Saharan Africa	Girls	2008	16	2.17
Sub-Saharan Africa	Girls	2009	16	2.23
Sub-Saharan Africa	Girls	2010	16	2.28
Sub-Saharan Africa	Girls	2011	16	2.34
Sub-Saharan Africa	Girls	2012	16	2.40
Sub-Saharan Africa	Girls	2013	16	2.46
Sub-Saharan Africa	Girls	2014	16	2.53
Sub-Saharan Africa	Girls	2015	16	2.59
Sub-Saharan Africa	Girls	2016	16	2.66
Sub-Saharan Africa	Girls	1975	17	1.30
Sub-Saharan Africa	Girls	1976	17	1.31
Sub-Saharan Africa	Girls	1977	17	1.33
Sub-Saharan Africa	Girls	1978	17	1.34
Sub-Saharan Africa	Girls	1979	17	1.36
Sub-Saharan Africa	Girls	1980	17	1.37
Sub-Saharan Africa	Girls	1981	17	1.39
Sub-Saharan Africa	Girls	1982	17	1.41
Sub-Saharan Africa	Girls	1983	17	1.43
Sub-Saharan Africa	Girls	1984	17	1.45
Sub-Saharan Africa	Girls	1985	17	1.47
Sub-Saharan Africa	Girls	1986	17	1.50
Sub-Saharan Africa	Girls	1987	17	1.52
Sub-Saharan Africa	Girls	1988	17	1.55
Sub-Saharan Africa	Girls	1989	17	1.58
Sub-Saharan Africa	Girls	1990	17	1.61
Sub-Saharan Africa	Girls	1991	17	1.64
Sub-Saharan Africa	Girls	1992	17	1.67
Sub-Saharan Africa	Girls	1993	17	1.70
Sub-Saharan Africa	Girls	1994	17	1.74
Sub-Saharan Africa	Girls	1995	17	1.78
Sub-Saharan Africa	Girls	1996	17	1.82
Sub-Saharan Africa	Girls	1997	17	1.86
Sub-Saharan Africa	Girls	1998	17	1.90
Sub-Saharan Africa	Girls	1999	17	1.94
Sub-Saharan Africa	Girls	2000	17	1.99
Sub-Saharan Africa	Girls	2001	17	2.04
Sub-Saharan Africa	Girls	2002	17	2.09
Sub-Saharan Africa	Girls	2003	17	2.14

Sub-Saharan Africa	Girls	2004	17	2.20
Sub-Saharan Africa	Girls	2005	17	2.26
Sub-Saharan Africa	Girls	2006	17	2.31
Sub-Saharan Africa	Girls	2007	17	2.37
Sub-Saharan Africa	Girls	2008	17	2.44
Sub-Saharan Africa	Girls	2009	17	2.50
Sub-Saharan Africa	Girls	2010	17	2.56
Sub-Saharan Africa	Girls	2011	17	2.62
Sub-Saharan Africa	Girls	2012	17	2.69
Sub-Saharan Africa	Girls	2013	17	2.76
Sub-Saharan Africa	Girls	2014	17	2.83
Sub-Saharan Africa	Girls	2015	17	2.90
Sub-Saharan Africa	Girls	2016	17	2.97
Sub-Saharan Africa	Girls	1975	18	1.42
Sub-Saharan Africa	Girls	1976	18	1.43
Sub-Saharan Africa	Girls	1977	18	1.45
Sub-Saharan Africa	Girls	1978	18	1.46
Sub-Saharan Africa	Girls	1979	18	1.48
Sub-Saharan Africa	Girls	1980	18	1.50
Sub-Saharan Africa	Girls	1981	18	1.52
Sub-Saharan Africa	Girls	1982	18	1.54
Sub-Saharan Africa	Girls	1983	18	1.56
Sub-Saharan Africa	Girls	1984	18	1.59
Sub-Saharan Africa	Girls	1985	18	1.61
Sub-Saharan Africa	Girls	1986	18	1.64
Sub-Saharan Africa	Girls	1987	18	1.67
Sub-Saharan Africa	Girls	1988	18	1.70
Sub-Saharan Africa	Girls	1989	18	1.73
Sub-Saharan Africa	Girls	1990	18	1.76
Sub-Saharan Africa	Girls	1991	18	1.80
Sub-Saharan Africa	Girls	1992	18	1.83
Sub-Saharan Africa	Girls	1993	18	1.87
Sub-Saharan Africa	Girls	1994	18	1.91
Sub-Saharan Africa	Girls	1995	18	1.95
Sub-Saharan Africa	Girls	1996	18	2.00
Sub-Saharan Africa	Girls	1997	18	2.04
Sub-Saharan Africa	Girls	1998	18	2.09
Sub-Saharan Africa	Girls	1999	18	2.14
Sub-Saharan Africa	Girls	2000	18	2.20
Sub-Saharan Africa	Girls	2001	18	2.25
Sub-Saharan Africa	Girls	2002	18	2.31
Sub-Saharan Africa	Girls	2003	18	2.37
Sub-Saharan Africa	Girls	2004	18	2.43
Sub-Saharan Africa	Girls	2005	18	2.50
Sub-Saharan Africa	Girls	2006	18	2.57
Sub-Saharan Africa	Girls	2007	18	2.63
Sub-Saharan Africa	Girls	2008	18	2.70
Sub-Saharan Africa	Girls	2009	18	2.77
Sub-Saharan Africa	Girls	2010	18	2.84
Sub-Saharan Africa	Girls	2011	18	2.91
Sub-Saharan Africa	Girls	2012	18	2.99
Sub-Saharan Africa	Girls	2013	18	3.06
Sub-Saharan Africa	Girls	2014	18	3.14
Sub-Saharan Africa	Girls	2015	18	3.22
Sub-Saharan Africa	Girls	2016	18	3.30
Sub-Saharan Africa	Girls	1975	Average	0.92
Sub-Saharan Africa	Girls	1976	Average	0.93
Sub-Saharan Africa	Girls	1977	Average	0.94
Sub-Saharan Africa	Girls	1978	Average	0.95

Sub-Saharan Africa	Girls	1979	Average	0.96
Sub-Saharan Africa	Girls	1980	Average	0.98
Sub-Saharan Africa	Girls	1981	Average	0.99
Sub-Saharan Africa	Girls	1982	Average	1.00
Sub-Saharan Africa	Girls	1983	Average	1.02
Sub-Saharan Africa	Girls	1984	Average	1.04
Sub-Saharan Africa	Girls	1985	Average	1.05
Sub-Saharan Africa	Girls	1986	Average	1.07
Sub-Saharan Africa	Girls	1987	Average	1.09
Sub-Saharan Africa	Girls	1988	Average	1.11
Sub-Saharan Africa	Girls	1989	Average	1.13
Sub-Saharan Africa	Girls	1990	Average	1.15
Sub-Saharan Africa	Girls	1991	Average	1.18
Sub-Saharan Africa	Girls	1992	Average	1.20
Sub-Saharan Africa	Girls	1993	Average	1.23
Sub-Saharan Africa	Girls	1994	Average	1.25
Sub-Saharan Africa	Girls	1995	Average	1.28
Sub-Saharan Africa	Girls	1996	Average	1.31
Sub-Saharan Africa	Girls	1997	Average	1.34
Sub-Saharan Africa	Girls	1998	Average	1.38
Sub-Saharan Africa	Girls	1999	Average	1.41
Sub-Saharan Africa	Girls	2000	Average	1.44
Sub-Saharan Africa	Girls	2001	Average	1.48
Sub-Saharan Africa	Girls	2002	Average	1.52
Sub-Saharan Africa	Girls	2003	Average	1.56
Sub-Saharan Africa	Girls	2004	Average	1.60
Sub-Saharan Africa	Girls	2005	Average	1.65
Sub-Saharan Africa	Girls	2006	Average	1.69
Sub-Saharan Africa	Girls	2007	Average	1.74
Sub-Saharan Africa	Girls	2008	Average	1.79
Sub-Saharan Africa	Girls	2009	Average	1.83
Sub-Saharan Africa	Girls	2010	Average	1.88
Sub-Saharan Africa	Girls	2011	Average	1.93
Sub-Saharan Africa	Girls	2012	Average	1.98
Sub-Saharan Africa	Girls	2013	Average	2.04
Sub-Saharan Africa	Girls	2014	Average	2.09
Sub-Saharan Africa	Girls	2015	Average	2.15
Sub-Saharan Africa	Girls	2016	Average	2.21
Sub-Saharan Africa	Girls	2017	Average	2.27
Sub-Saharan Africa	Girls	2018	Average	2.33
Sub-Saharan Africa	Girls	2019	Average	2.39
Sub-Saharan Africa	Girls	2020	Average	2.45
Sub-Saharan Africa	Girls	2021	Average	2.51
Sub-Saharan Africa	Girls	2022	Average	2.57
Sub-Saharan Africa	Girls	2023	Average	2.63
Sub-Saharan Africa	Girls	2024	Average	2.69
Sub-Saharan Africa	Girls	2025	Average	2.76
Sub-Saharan Africa	Girls	2026	Average	2.82
Sub-Saharan Africa	Girls	2027	Average	2.88
Sub-Saharan Africa	Girls	2028	Average	2.94
Sub-Saharan Africa	Girls	2029	Average	3.01
Sub-Saharan Africa	Girls	2030	Average	3.07

region	sex	age	EPAC (%)	lower 95% CI (%)	upper 95% CI (%)
Central and Eastern Europe	Boys	12	1.49	1.44	1.54
Central and Eastern Europe	Boys	13	1.44	1.39	1.49
Central and Eastern Europe	Boys	14	1.33	1.28	1.39
Central and Eastern Europe	Boys	15	1.19	1.13	1.25
Central and Eastern Europe	Boys	16	1.05	0.99	1.11
Central and Eastern Europe	Boys	17	0.94	0.88	0.99
Central and Eastern Europe	Boys	18	0.86	0.80	0.91
Central and Eastern Europe	Girls	12	0.96	0.92	0.99
Central and Eastern Europe	Girls	13	0.89	0.85	0.92
Central and Eastern Europe	Girls	14	0.80	0.77	0.84
Central and Eastern Europe	Girls	15	0.73	0.69	0.76
Central and Eastern Europe	Girls	16	0.68	0.64	0.71
Central and Eastern Europe	Girls	17	0.66	0.62	0.69
Central and Eastern Europe	Girls	18	0.67	0.63	0.70
Central Asia and North Africa-Middle East	Boys	12	2.05	2.04	2.07
Central Asia and North Africa-Middle East	Boys	13	2.02	2.00	2.03
Central Asia and North Africa-Middle East	Boys	14	1.90	1.89	1.91
Central Asia and North Africa-Middle East	Boys	15	1.71	1.69	1.72
Central Asia and North Africa-Middle East	Boys	16	1.54	1.52	1.55
Central Asia and North Africa-Middle East	Boys	17	1.39	1.37	1.41
Central Asia and North Africa-Middle East	Boys	18	1.29	1.27	1.31
Central Asia and North Africa-Middle East	Girls	12	1.44	1.42	1.45
Central Asia and North Africa-Middle East	Girls	13	1.36	1.35	1.37
Central Asia and North Africa-Middle East	Girls	14	1.26	1.25	1.26
Central Asia and North Africa-Middle East	Girls	15	1.14	1.13	1.15
Central Asia and North Africa-Middle East	Girls	16	1.08	1.07	1.09
Central Asia and North Africa-Middle East	Girls	17	1.07	1.06	1.08
Central Asia and North Africa-Middle East	Girls	18	1.10	1.08	1.11
East and South East Asia	Boys	12	1.38	1.32	1.44
East and South East Asia	Boys	13	1.34	1.28	1.40
East and South East Asia	Boys	14	1.25	1.20	1.31
East and South East Asia	Boys	15	1.20	1.15	1.25
East and South East Asia	Boys	16	1.08	1.03	1.13
East and South East Asia	Boys	17	0.98	0.93	1.02
East and South East Asia	Boys	18	0.91	0.87	0.95
East and South East Asia	Girls	12	0.75	0.71	0.79
East and South East Asia	Girls	13	0.69	0.66	0.73
East and South East Asia	Girls	14	0.63	0.60	0.66
East and South East Asia	Girls	15	0.59	0.57	0.62
East and South East Asia	Girls	16	0.56	0.53	0.58
East and South East Asia	Girls	17	0.55	0.52	0.58
East and South East Asia	Girls	18	0.57	0.54	0.60
High-income Asia Pacific	Boys	12	0.66	0.60	0.72
High-income Asia Pacific	Boys	13	0.65	0.59	0.71
High-income Asia Pacific	Boys	14	0.61	0.56	0.66
High-income Asia Pacific	Boys	15	0.60	0.57	0.62
High-income Asia Pacific	Boys	16	0.53	0.51	0.56
High-income Asia Pacific	Boys	17	0.48	0.46	0.50
High-income Asia Pacific	Boys	18	0.44	0.42	0.46
High-income Asia Pacific	Girls	12	0.43	0.40	0.46
High-income Asia Pacific	Girls	13	0.40	0.37	0.43
High-income Asia Pacific	Girls	14	0.36	0.33	0.38
High-income Asia Pacific	Girls	15	0.35	0.34	0.36
High-income Asia Pacific	Girls	16	0.32	0.31	0.33
High-income Asia Pacific	Girls	17	0.31	0.30	0.32
High-income Asia Pacific	Girls	18	0.32	0.31	0.33
High-income Western countries	Boys	12	1.02	0.95	1.08
High-income Western countries	Boys	13	1.02	0.95	1.09
High-income Western countries	Boys	14	0.99	0.92	1.06
High-income Western countries	Boys	15	0.93	0.87	0.99
High-income Western countries	Boys	16	0.87	0.81	0.92
High-income Western countries	Boys	17	0.80	0.75	0.85
High-income Western countries	Boys	18	0.75	0.71	0.80
High-income Western countries	Girls	12	0.81	0.75	0.87
High-income Western countries	Girls	13	0.78	0.72	0.84

High-income Western countries	Girls	14	0.73	0.68	0.79
High-income Western countries	Girls	15	0.70	0.65	0.76
High-income Western countries	Girls	16	0.68	0.62	0.73
High-income Western countries	Girls	17	0.67	0.62	0.72
High-income Western countries	Girls	18	0.68	0.63	0.74
Latin America and Caribbean	Boys	12	1.57	1.55	1.59
Latin America and Caribbean	Boys	13	1.55	1.53	1.56
Latin America and Caribbean	Boys	14	1.46	1.45	1.47
Latin America and Caribbean	Boys	15	1.33	1.33	1.34
Latin America and Caribbean	Boys	16	1.20	1.19	1.21
Latin America and Caribbean	Boys	17	1.09	1.08	1.10
Latin America and Caribbean	Boys	18	1.00	0.99	1.01
Latin America and Caribbean	Girls	12	1.11	1.09	1.13
Latin America and Caribbean	Girls	13	1.05	1.03	1.07
Latin America and Caribbean	Girls	14	0.98	0.96	0.99
Latin America and Caribbean	Girls	15	0.90	0.89	0.91
Latin America and Caribbean	Girls	16	0.86	0.85	0.86
Latin America and Caribbean	Girls	17	0.84	0.84	0.85
Latin America and Caribbean	Girls	18	0.86	0.86	0.87
Oceania	Boys	12	1.87	1.84	1.90
Oceania	Boys	13	1.82	1.78	1.85
Oceania	Boys	14	1.69	1.65	1.73
Oceania	Boys	15	1.54	1.50	1.58
Oceania	Boys	16	1.37	1.33	1.41
Oceania	Boys	17	1.22	1.18	1.27
Oceania	Boys	18	1.12	1.08	1.16
Oceania	Girls	12	1.51	1.50	1.52
Oceania	Girls	13	1.45	1.44	1.46
Oceania	Girls	14	1.37	1.37	1.38
Oceania	Girls	15	1.31	1.30	1.31
Oceania	Girls	16	1.26	1.25	1.26
Oceania	Girls	17	1.24	1.23	1.24
Oceania	Girls	18	1.26	1.25	1.26
South Asia	Boys	12	1.75	1.64	1.86
South Asia	Boys	13	1.64	1.54	1.75
South Asia	Boys	14	1.48	1.39	1.57
South Asia	Boys	15	1.30	1.22	1.38
South Asia	Boys	16	1.15	1.08	1.21
South Asia	Boys	17	1.04	0.98	1.10
South Asia	Boys	18	0.99	0.94	1.04
South Asia	Girls	12	1.09	1.02	1.16
South Asia	Girls	13	1.01	0.95	1.07
South Asia	Girls	14	0.92	0.87	0.98
South Asia	Girls	15	0.85	0.80	0.90
South Asia	Girls	16	0.80	0.75	0.84
South Asia	Girls	17	0.78	0.74	0.83
South Asia	Girls	18	0.82	0.77	0.86
Sub-Saharan Africa	Boys	12	1.34	1.27	1.41
Sub-Saharan Africa	Boys	13	1.27	1.20	1.33
Sub-Saharan Africa	Boys	14	1.15	1.09	1.21
Sub-Saharan Africa	Boys	15	1.04	0.99	1.09
Sub-Saharan Africa	Boys	16	0.93	0.88	0.97
Sub-Saharan Africa	Boys	17	0.84	0.81	0.88
Sub-Saharan Africa	Boys	18	0.80	0.76	0.83
Sub-Saharan Africa	Girls	12	1.44	1.41	1.47
Sub-Saharan Africa	Girls	13	1.35	1.32	1.38
Sub-Saharan Africa	Girls	14	1.23	1.20	1.26
Sub-Saharan Africa	Girls	15	1.14	1.12	1.17
Sub-Saharan Africa	Girls	16	1.07	1.04	1.09
Sub-Saharan Africa	Girls	17	1.04	1.02	1.07
Sub-Saharan Africa	Girls	18	1.07	1.05	1.09

region	sex	age	EPAC (%)	lower 95% CI (%)	upper 95% CI (%)
Central and Eastern Europe	Boys	12	2.25	2.19	2.31
Central and Eastern Europe	Boys	13	2.32	2.25	2.39
Central and Eastern Europe	Boys	14	2.32	2.25	2.40
Central and Eastern Europe	Boys	15	2.25	2.15	2.35
Central and Eastern Europe	Boys	16	2.16	2.05	2.26
Central and Eastern Europe	Boys	17	2.06	1.95	2.16
Central and Eastern Europe	Boys	18	1.98	1.87	2.08
Central and Eastern Europe	Girls	12	1.72	1.66	1.77
Central and Eastern Europe	Girls	13	1.67	1.62	1.73
Central and Eastern Europe	Girls	14	1.59	1.54	1.65
Central and Eastern Europe	Girls	15	1.50	1.44	1.57
Central and Eastern Europe	Girls	16	1.44	1.38	1.50
Central and Eastern Europe	Girls	17	1.42	1.36	1.48
Central and Eastern Europe	Girls	18	1.45	1.38	1.51
Central Asia and North Africa-Middle East	Boys	12	2.99	2.95	3.03
Central Asia and North Africa-Middle East	Boys	13	3.11	3.08	3.15
Central Asia and North Africa-Middle East	Boys	14	3.16	3.13	3.19
Central Asia and North Africa-Middle East	Boys	15	3.08	3.06	3.11
Central Asia and North Africa-Middle East	Boys	16	3.00	2.99	3.02
Central Asia and North Africa-Middle East	Boys	17	2.91	2.89	2.92
Central Asia and North Africa-Middle East	Boys	18	2.82	2.81	2.84
Central Asia and North Africa-Middle East	Girls	12	2.46	2.43	2.50
Central Asia and North Africa-Middle East	Girls	13	2.43	2.40	2.46
Central Asia and North Africa-Middle East	Girls	14	2.35	2.33	2.38
Central Asia and North Africa-Middle East	Girls	15	2.21	2.18	2.25
Central Asia and North Africa-Middle East	Girls	16	2.15	2.12	2.18
Central Asia and North Africa-Middle East	Girls	17	2.13	2.10	2.17
Central Asia and North Africa-Middle East	Girls	18	2.16	2.12	2.20
East and South East Asia	Boys	12	2.01	1.92	2.09
East and South East Asia	Boys	13	2.07	1.99	2.16
East and South East Asia	Boys	14	2.09	2.00	2.17
East and South East Asia	Boys	15	2.14	2.06	2.21
East and South East Asia	Boys	16	2.08	2.00	2.15
East and South East Asia	Boys	17	2.01	1.94	2.08
East and South East Asia	Boys	18	1.95	1.88	2.02
East and South East Asia	Girls	12	1.42	1.35	1.48
East and South East Asia	Girls	13	1.38	1.31	1.44
East and South East Asia	Girls	14	1.31	1.25	1.38
East and South East Asia	Girls	15	1.27	1.22	1.32
East and South East Asia	Girls	16	1.22	1.17	1.28
East and South East Asia	Girls	17	1.22	1.16	1.27
East and South East Asia	Girls	18	1.25	1.20	1.30
High-income Asia Pacific	Boys	12	1.00	0.91	1.09
High-income Asia Pacific	Boys	13	1.05	0.96	1.14
High-income Asia Pacific	Boys	14	1.07	0.98	1.16
High-income Asia Pacific	Boys	15	1.14	1.09	1.19
High-income Asia Pacific	Boys	16	1.11	1.06	1.15
High-income Asia Pacific	Boys	17	1.07	1.03	1.12
High-income Asia Pacific	Boys	18	1.04	1.01	1.08
High-income Asia Pacific	Girls	12	0.84	0.78	0.90
High-income Asia Pacific	Girls	13	0.82	0.76	0.87
High-income Asia Pacific	Girls	14	0.78	0.73	0.83
High-income Asia Pacific	Girls	15	0.80	0.77	0.82
High-income Asia Pacific	Girls	16	0.77	0.74	0.79
High-income Asia Pacific	Girls	17	0.76	0.74	0.78
High-income Asia Pacific	Girls	18	0.78	0.76	0.80
High-income Western countries	Boys	12	1.42	1.32	1.53
High-income Western countries	Boys	13	1.51	1.40	1.62
High-income Western countries	Boys	14	1.57	1.45	1.68
High-income Western countries	Boys	15	1.60	1.49	1.72
High-income Western countries	Boys	16	1.60	1.49	1.71
High-income Western countries	Boys	17	1.58	1.47	1.69
High-income Western countries	Boys	18	1.55	1.44	1.66
High-income Western countries	Girls	12	1.30	1.19	1.41
High-income Western countries	Girls	13	1.30	1.19	1.41
High-income Western countries	Girls	14	1.28	1.17	1.39

High-income Western countries	Girls	15	1.28	1.17	1.38
High-income Western countries	Girls	16	1.25	1.15	1.35
High-income Western countries	Girls	17	1.25	1.14	1.35
High-income Western countries	Girls	18	1.25	1.15	1.36
Latin America and Caribbean	Boys	12	2.27	2.24	2.31
Latin America and Caribbean	Boys	13	2.37	2.34	2.40
Latin America and Caribbean	Boys	14	2.41	2.39	2.44
Latin America and Caribbean	Boys	15	2.38	2.37	2.39
Latin America and Caribbean	Boys	16	2.32	2.31	2.33
Latin America and Caribbean	Boys	17	2.25	2.24	2.25
Latin America and Caribbean	Boys	18	2.17	2.16	2.18
Latin America and Caribbean	Girls	12	1.85	1.81	1.89
Latin America and Caribbean	Girls	13	1.83	1.79	1.86
Latin America and Caribbean	Girls	14	1.77	1.74	1.81
Latin America and Caribbean	Girls	15	1.70	1.68	1.72
Latin America and Caribbean	Girls	16	1.65	1.63	1.67
Latin America and Caribbean	Girls	17	1.64	1.62	1.66
Latin America and Caribbean	Girls	18	1.67	1.65	1.69
Oceania	Boys	12	2.86	2.84	2.88
Oceania	Boys	13	2.97	2.94	3.01
Oceania	Boys	14	3.00	2.96	3.05
Oceania	Boys	15	2.98	2.93	3.03
Oceania	Boys	16	2.88	2.82	2.94
Oceania	Boys	17	2.77	2.70	2.83
Oceania	Boys	18	2.67	2.61	2.74
Oceania	Girls	12	2.45	2.43	2.47
Oceania	Girls	13	2.44	2.42	2.46
Oceania	Girls	14	2.39	2.38	2.41
Oceania	Girls	15	2.36	2.34	2.37
Oceania	Girls	16	2.32	2.30	2.33
Oceania	Girls	17	2.30	2.29	2.32
Oceania	Girls	18	2.32	2.30	2.34
South Asia	Boys	12	3.07	2.90	3.24
South Asia	Boys	13	3.08	2.90	3.25
South Asia	Boys	14	2.96	2.79	3.14
South Asia	Boys	15	2.78	2.62	2.95
South Asia	Boys	16	2.58	2.42	2.74
South Asia	Boys	17	2.41	2.26	2.56
South Asia	Boys	18	2.34	2.20	2.48
South Asia	Girls	12	2.50	2.35	2.65
South Asia	Girls	13	2.41	2.27	2.55
South Asia	Girls	14	2.26	2.13	2.38
South Asia	Girls	15	2.10	1.98	2.22
South Asia	Girls	16	1.99	1.88	2.11
South Asia	Girls	17	1.97	1.85	2.08
South Asia	Girls	18	2.06	1.94	2.17
Sub-Saharan Africa	Boys	12	2.26	2.14	2.38
Sub-Saharan Africa	Boys	13	2.27	2.15	2.39
Sub-Saharan Africa	Boys	14	2.20	2.08	2.32
Sub-Saharan Africa	Boys	15	2.09	1.98	2.21
Sub-Saharan Africa	Boys	16	1.94	1.83	2.05
Sub-Saharan Africa	Boys	17	1.81	1.71	1.91
Sub-Saharan Africa	Boys	18	1.75	1.65	1.84
Sub-Saharan Africa	Girls	12	2.77	2.73	2.81
Sub-Saharan Africa	Girls	13	2.71	2.67	2.75
Sub-Saharan Africa	Girls	14	2.58	2.54	2.62
Sub-Saharan Africa	Girls	15	2.48	2.44	2.52
Sub-Saharan Africa	Girls	16	2.38	2.34	2.42
Sub-Saharan Africa	Girls	17	2.35	2.31	2.38
Sub-Saharan Africa	Girls	18	2.40	2.37	2.44

region	sex	year	age	Predicted prevalence (%)
Central and Eastern Europe	Boys	2017	12	11.62
Central and Eastern Europe	Boys	2018	12	11.82
Central and Eastern Europe	Boys	2019	12	12.02
Central and Eastern Europe	Boys	2020	12	12.23
Central and Eastern Europe	Boys	2021	12	12.44
Central and Eastern Europe	Boys	2022	12	12.65
Central and Eastern Europe	Boys	2023	12	12.87
Central and Eastern Europe	Boys	2024	12	13.08
Central and Eastern Europe	Boys	2025	12	13.30
Central and Eastern Europe	Boys	2026	12	13.52
Central and Eastern Europe	Boys	2027	12	13.74
Central and Eastern Europe	Boys	2028	12	13.96
Central and Eastern Europe	Boys	2029	12	14.18
Central and Eastern Europe	Boys	2030	12	14.40
Central and Eastern Europe	Boys	2017	13	14.02
Central and Eastern Europe	Boys	2018	13	14.27
Central and Eastern Europe	Boys	2019	13	14.52
Central and Eastern Europe	Boys	2020	13	14.78
Central and Eastern Europe	Boys	2021	13	15.04
Central and Eastern Europe	Boys	2022	13	15.31
Central and Eastern Europe	Boys	2023	13	15.58
Central and Eastern Europe	Boys	2024	13	15.86
Central and Eastern Europe	Boys	2025	13	16.14
Central and Eastern Europe	Boys	2026	13	16.42
Central and Eastern Europe	Boys	2027	13	16.71
Central and Eastern Europe	Boys	2028	13	17.00
Central and Eastern Europe	Boys	2029	13	17.29
Central and Eastern Europe	Boys	2030	13	17.58
Central and Eastern Europe	Boys	2017	14	16.51
Central and Eastern Europe	Boys	2018	14	16.80
Central and Eastern Europe	Boys	2019	14	17.09
Central and Eastern Europe	Boys	2020	14	17.39
Central and Eastern Europe	Boys	2021	14	17.70
Central and Eastern Europe	Boys	2022	14	18.01
Central and Eastern Europe	Boys	2023	14	18.33
Central and Eastern Europe	Boys	2024	14	18.65
Central and Eastern Europe	Boys	2025	14	18.98
Central and Eastern Europe	Boys	2026	14	19.31
Central and Eastern Europe	Boys	2027	14	19.64
Central and Eastern Europe	Boys	2028	14	19.98
Central and Eastern Europe	Boys	2029	14	20.31
Central and Eastern Europe	Boys	2030	14	20.65
Central and Eastern Europe	Boys	2017	15	19.03
Central and Eastern Europe	Boys	2018	15	19.33
Central and Eastern Europe	Boys	2019	15	19.63
Central and Eastern Europe	Boys	2020	15	19.93
Central and Eastern Europe	Boys	2021	15	20.23
Central and Eastern Europe	Boys	2022	15	20.53
Central and Eastern Europe	Boys	2023	15	20.83
Central and Eastern Europe	Boys	2024	15	21.13
Central and Eastern Europe	Boys	2025	15	21.43
Central and Eastern Europe	Boys	2026	15	21.72

Central and Eastern Europe	Boys	2027	15	22.02
Central and Eastern Europe	Boys	2028	15	22.32
Central and Eastern Europe	Boys	2029	15	22.61
Central and Eastern Europe	Boys	2030	15	22.91
Central and Eastern Europe	Boys	2017	16	21.39
Central and Eastern Europe	Boys	2018	16	21.70
Central and Eastern Europe	Boys	2019	16	22.01
Central and Eastern Europe	Boys	2020	16	22.31
Central and Eastern Europe	Boys	2021	16	22.61
Central and Eastern Europe	Boys	2022	16	22.90
Central and Eastern Europe	Boys	2023	16	23.18
Central and Eastern Europe	Boys	2024	16	23.46
Central and Eastern Europe	Boys	2025	16	23.74
Central and Eastern Europe	Boys	2026	16	24.01
Central and Eastern Europe	Boys	2027	16	24.28
Central and Eastern Europe	Boys	2028	16	24.54
Central and Eastern Europe	Boys	2029	16	24.80
Central and Eastern Europe	Boys	2030	16	25.07
Central and Eastern Europe	Boys	2017	17	23.76
Central and Eastern Europe	Boys	2018	17	24.08
Central and Eastern Europe	Boys	2019	17	24.39
Central and Eastern Europe	Boys	2020	17	24.69
Central and Eastern Europe	Boys	2021	17	24.99
Central and Eastern Europe	Boys	2022	17	25.28
Central and Eastern Europe	Boys	2023	17	25.57
Central and Eastern Europe	Boys	2024	17	25.85
Central and Eastern Europe	Boys	2025	17	26.13
Central and Eastern Europe	Boys	2026	17	26.40
Central and Eastern Europe	Boys	2027	17	26.67
Central and Eastern Europe	Boys	2028	17	26.94
Central and Eastern Europe	Boys	2029	17	27.20
Central and Eastern Europe	Boys	2030	17	27.46
Central and Eastern Europe	Boys	2017	18	26.24
Central and Eastern Europe	Boys	2018	18	26.56
Central and Eastern Europe	Boys	2019	18	26.88
Central and Eastern Europe	Boys	2020	18	27.18
Central and Eastern Europe	Boys	2021	18	27.48
Central and Eastern Europe	Boys	2022	18	27.78
Central and Eastern Europe	Boys	2023	18	28.06
Central and Eastern Europe	Boys	2024	18	28.34
Central and Eastern Europe	Boys	2025	18	28.62
Central and Eastern Europe	Boys	2026	18	28.89
Central and Eastern Europe	Boys	2027	18	29.16
Central and Eastern Europe	Boys	2028	18	29.42
Central and Eastern Europe	Boys	2029	18	29.68
Central and Eastern Europe	Boys	2030	18	29.94
Central and Eastern Europe	Girls	2017	12	8.84
Central and Eastern Europe	Girls	2018	12	8.94
Central and Eastern Europe	Girls	2019	12	9.05
Central and Eastern Europe	Girls	2020	12	9.16
Central and Eastern Europe	Girls	2021	12	9.27
Central and Eastern Europe	Girls	2022	12	9.38
Central and Eastern Europe	Girls	2023	12	9.50

Central and Eastern Europe	Girls	2024	12	9.61
Central and Eastern Europe	Girls	2025	12	9.72
Central and Eastern Europe	Girls	2026	12	9.84
Central and Eastern Europe	Girls	2027	12	9.95
Central and Eastern Europe	Girls	2028	12	10.07
Central and Eastern Europe	Girls	2029	12	10.19
Central and Eastern Europe	Girls	2030	12	10.31
Central and Eastern Europe	Girls	2017	13	10.64
Central and Eastern Europe	Girls	2018	13	10.77
Central and Eastern Europe	Girls	2019	13	10.89
Central and Eastern Europe	Girls	2020	13	11.02
Central and Eastern Europe	Girls	2021	13	11.14
Central and Eastern Europe	Girls	2022	13	11.27
Central and Eastern Europe	Girls	2023	13	11.40
Central and Eastern Europe	Girls	2024	13	11.53
Central and Eastern Europe	Girls	2025	13	11.67
Central and Eastern Europe	Girls	2026	13	11.80
Central and Eastern Europe	Girls	2027	13	11.93
Central and Eastern Europe	Girls	2028	13	12.06
Central and Eastern Europe	Girls	2029	13	12.20
Central and Eastern Europe	Girls	2030	13	12.33
Central and Eastern Europe	Girls	2017	14	12.41
Central and Eastern Europe	Girls	2018	14	12.54
Central and Eastern Europe	Girls	2019	14	12.68
Central and Eastern Europe	Girls	2020	14	12.82
Central and Eastern Europe	Girls	2021	14	12.96
Central and Eastern Europe	Girls	2022	14	13.10
Central and Eastern Europe	Girls	2023	14	13.24
Central and Eastern Europe	Girls	2024	14	13.38
Central and Eastern Europe	Girls	2025	14	13.53
Central and Eastern Europe	Girls	2026	14	13.67
Central and Eastern Europe	Girls	2027	14	13.82
Central and Eastern Europe	Girls	2028	14	13.97
Central and Eastern Europe	Girls	2029	14	14.11
Central and Eastern Europe	Girls	2030	14	14.26
Central and Eastern Europe	Girls	2017	15	13.97
Central and Eastern Europe	Girls	2018	15	14.11
Central and Eastern Europe	Girls	2019	15	14.25
Central and Eastern Europe	Girls	2020	15	14.39
Central and Eastern Europe	Girls	2021	15	14.53
Central and Eastern Europe	Girls	2022	15	14.67
Central and Eastern Europe	Girls	2023	15	14.81
Central and Eastern Europe	Girls	2024	15	14.94
Central and Eastern Europe	Girls	2025	15	15.08
Central and Eastern Europe	Girls	2026	15	15.22
Central and Eastern Europe	Girls	2027	15	15.36
Central and Eastern Europe	Girls	2028	15	15.50
Central and Eastern Europe	Girls	2029	15	15.64
Central and Eastern Europe	Girls	2030	15	15.78
Central and Eastern Europe	Girls	2017	16	15.17
Central and Eastern Europe	Girls	2018	16	15.31
Central and Eastern Europe	Girls	2019	16	15.45
Central and Eastern Europe	Girls	2020	16	15.60

Central and Eastern Europe	Girls	2021	16	15.74
Central and Eastern Europe	Girls	2022	16	15.88
Central and Eastern Europe	Girls	2023	16	16.02
Central and Eastern Europe	Girls	2024	16	16.16
Central and Eastern Europe	Girls	2025	16	16.31
Central and Eastern Europe	Girls	2026	16	16.45
Central and Eastern Europe	Girls	2027	16	16.59
Central and Eastern Europe	Girls	2028	16	16.73
Central and Eastern Europe	Girls	2029	16	16.88
Central and Eastern Europe	Girls	2030	16	17.02
Central and Eastern Europe	Girls	2017	17	16.09
Central and Eastern Europe	Girls	2018	17	16.24
Central and Eastern Europe	Girls	2019	17	16.39
Central and Eastern Europe	Girls	2020	17	16.53
Central and Eastern Europe	Girls	2021	17	16.68
Central and Eastern Europe	Girls	2022	17	16.83
Central and Eastern Europe	Girls	2023	17	16.97
Central and Eastern Europe	Girls	2024	17	17.12
Central and Eastern Europe	Girls	2025	17	17.27
Central and Eastern Europe	Girls	2026	17	17.41
Central and Eastern Europe	Girls	2027	17	17.56
Central and Eastern Europe	Girls	2028	17	17.71
Central and Eastern Europe	Girls	2029	17	17.85
Central and Eastern Europe	Girls	2030	17	18.00
Central and Eastern Europe	Girls	2017	18	16.95
Central and Eastern Europe	Girls	2018	18	17.10
Central and Eastern Europe	Girls	2019	18	17.26
Central and Eastern Europe	Girls	2020	18	17.41
Central and Eastern Europe	Girls	2021	18	17.57
Central and Eastern Europe	Girls	2022	18	17.72
Central and Eastern Europe	Girls	2023	18	17.87
Central and Eastern Europe	Girls	2024	18	18.02
Central and Eastern Europe	Girls	2025	18	18.18
Central and Eastern Europe	Girls	2026	18	18.33
Central and Eastern Europe	Girls	2027	18	18.48
Central and Eastern Europe	Girls	2028	18	18.63
Central and Eastern Europe	Girls	2029	18	18.78
Central and Eastern Europe	Girls	2030	18	18.93
Central Asia and North Africa-Middle East	Boys	2017	12	12.04
Central Asia and North Africa-Middle East	Boys	2018	12	12.25
Central Asia and North Africa-Middle East	Boys	2019	12	12.46
Central Asia and North Africa-Middle East	Boys	2020	12	12.67
Central Asia and North Africa-Middle East	Boys	2021	12	12.87
Central Asia and North Africa-Middle East	Boys	2022	12	13.08
Central Asia and North Africa-Middle East	Boys	2023	12	13.29
Central Asia and North Africa-Middle East	Boys	2024	12	13.50
Central Asia and North Africa-Middle East	Boys	2025	12	13.70
Central Asia and North Africa-Middle East	Boys	2026	12	13.91
Central Asia and North Africa-Middle East	Boys	2027	12	14.12
Central Asia and North Africa-Middle East	Boys	2028	12	14.33
Central Asia and North Africa-Middle East	Boys	2029	12	14.54
Central Asia and North Africa-Middle East	Boys	2030	12	14.75
Central Asia and North Africa-Middle East	Boys	2017	13	14.57

Central Asia and North Africa-Middle East	Boys	2018	13	14.83
Central Asia and North Africa-Middle East	Boys	2019	13	15.09
Central Asia and North Africa-Middle East	Boys	2020	13	15.35
Central Asia and North Africa-Middle East	Boys	2021	13	15.61
Central Asia and North Africa-Middle East	Boys	2022	13	15.88
Central Asia and North Africa-Middle East	Boys	2023	13	16.14
Central Asia and North Africa-Middle East	Boys	2024	13	16.41
Central Asia and North Africa-Middle East	Boys	2025	13	16.67
Central Asia and North Africa-Middle East	Boys	2026	13	16.94
Central Asia and North Africa-Middle East	Boys	2027	13	17.21
Central Asia and North Africa-Middle East	Boys	2028	13	17.48
Central Asia and North Africa-Middle East	Boys	2029	13	17.75
Central Asia and North Africa-Middle East	Boys	2030	13	18.02
Central Asia and North Africa-Middle East	Boys	2017	14	17.12
Central Asia and North Africa-Middle East	Boys	2018	14	17.43
Central Asia and North Africa-Middle East	Boys	2019	14	17.73
Central Asia and North Africa-Middle East	Boys	2020	14	18.04
Central Asia and North Africa-Middle East	Boys	2021	14	18.35
Central Asia and North Africa-Middle East	Boys	2022	14	18.67
Central Asia and North Africa-Middle East	Boys	2023	14	18.98
Central Asia and North Africa-Middle East	Boys	2024	14	19.30
Central Asia and North Africa-Middle East	Boys	2025	14	19.62
Central Asia and North Africa-Middle East	Boys	2026	14	19.95
Central Asia and North Africa-Middle East	Boys	2027	14	20.27
Central Asia and North Africa-Middle East	Boys	2028	14	20.60
Central Asia and North Africa-Middle East	Boys	2029	14	20.93
Central Asia and North Africa-Middle East	Boys	2030	14	21.27
Central Asia and North Africa-Middle East	Boys	2017	15	19.46
Central Asia and North Africa-Middle East	Boys	2018	15	19.80
Central Asia and North Africa-Middle East	Boys	2019	15	20.15
Central Asia and North Africa-Middle East	Boys	2020	15	20.51
Central Asia and North Africa-Middle East	Boys	2021	15	20.86
Central Asia and North Africa-Middle East	Boys	2022	15	21.23
Central Asia and North Africa-Middle East	Boys	2023	15	21.59
Central Asia and North Africa-Middle East	Boys	2024	15	21.96
Central Asia and North Africa-Middle East	Boys	2025	15	22.33
Central Asia and North Africa-Middle East	Boys	2026	15	22.71
Central Asia and North Africa-Middle East	Boys	2027	15	23.09
Central Asia and North Africa-Middle East	Boys	2028	15	23.47
Central Asia and North Africa-Middle East	Boys	2029	15	23.85
Central Asia and North Africa-Middle East	Boys	2030	15	24.24
Central Asia and North Africa-Middle East	Boys	2017	16	21.78
Central Asia and North Africa-Middle East	Boys	2018	16	22.14
Central Asia and North Africa-Middle East	Boys	2019	16	22.51
Central Asia and North Africa-Middle East	Boys	2020	16	22.88
Central Asia and North Africa-Middle East	Boys	2021	16	23.26
Central Asia and North Africa-Middle East	Boys	2022	16	23.64
Central Asia and North Africa-Middle East	Boys	2023	16	24.02
Central Asia and North Africa-Middle East	Boys	2024	16	24.41
Central Asia and North Africa-Middle East	Boys	2025	16	24.80
Central Asia and North Africa-Middle East	Boys	2026	16	25.19
Central Asia and North Africa-Middle East	Boys	2027	16	25.59
Central Asia and North Africa-Middle East	Boys	2028	16	25.99

Central Asia and North Africa-Middle East	Boys	2029	16	26.38
Central Asia and North Africa-Middle East	Boys	2030	16	26.78
Central Asia and North Africa-Middle East	Boys	2017	17	24.14
Central Asia and North Africa-Middle East	Boys	2018	17	24.51
Central Asia and North Africa-Middle East	Boys	2019	17	24.89
Central Asia and North Africa-Middle East	Boys	2020	17	25.28
Central Asia and North Africa-Middle East	Boys	2021	17	25.67
Central Asia and North Africa-Middle East	Boys	2022	17	26.07
Central Asia and North Africa-Middle East	Boys	2023	17	26.47
Central Asia and North Africa-Middle East	Boys	2024	17	26.87
Central Asia and North Africa-Middle East	Boys	2025	17	27.27
Central Asia and North Africa-Middle East	Boys	2026	17	27.68
Central Asia and North Africa-Middle East	Boys	2027	17	28.09
Central Asia and North Africa-Middle East	Boys	2028	17	28.51
Central Asia and North Africa-Middle East	Boys	2029	17	28.92
Central Asia and North Africa-Middle East	Boys	2030	17	29.34
Central Asia and North Africa-Middle East	Boys	2017	18	26.70
Central Asia and North Africa-Middle East	Boys	2018	18	27.08
Central Asia and North Africa-Middle East	Boys	2019	18	27.48
Central Asia and North Africa-Middle East	Boys	2020	18	27.87
Central Asia and North Africa-Middle East	Boys	2021	18	28.28
Central Asia and North Africa-Middle East	Boys	2022	18	28.69
Central Asia and North Africa-Middle East	Boys	2023	18	29.10
Central Asia and North Africa-Middle East	Boys	2024	18	29.51
Central Asia and North Africa-Middle East	Boys	2025	18	29.93
Central Asia and North Africa-Middle East	Boys	2026	18	30.35
Central Asia and North Africa-Middle East	Boys	2027	18	30.77
Central Asia and North Africa-Middle East	Boys	2028	18	31.20
Central Asia and North Africa-Middle East	Boys	2029	18	31.62
Central Asia and North Africa-Middle East	Boys	2030	18	32.05
Central Asia and North Africa-Middle East	Girls	2017	12	10.93
Central Asia and North Africa-Middle East	Girls	2018	12	11.06
Central Asia and North Africa-Middle East	Girls	2019	12	11.19
Central Asia and North Africa-Middle East	Girls	2020	12	11.33
Central Asia and North Africa-Middle East	Girls	2021	12	11.46
Central Asia and North Africa-Middle East	Girls	2022	12	11.59
Central Asia and North Africa-Middle East	Girls	2023	12	11.72
Central Asia and North Africa-Middle East	Girls	2024	12	11.85
Central Asia and North Africa-Middle East	Girls	2025	12	11.98
Central Asia and North Africa-Middle East	Girls	2026	12	12.11
Central Asia and North Africa-Middle East	Girls	2027	12	12.24
Central Asia and North Africa-Middle East	Girls	2028	12	12.37
Central Asia and North Africa-Middle East	Girls	2029	12	12.50
Central Asia and North Africa-Middle East	Girls	2030	12	12.64
Central Asia and North Africa-Middle East	Girls	2017	13	13.13
Central Asia and North Africa-Middle East	Girls	2018	13	13.29
Central Asia and North Africa-Middle East	Girls	2019	13	13.44
Central Asia and North Africa-Middle East	Girls	2020	13	13.60
Central Asia and North Africa-Middle East	Girls	2021	13	13.76
Central Asia and North Africa-Middle East	Girls	2022	13	13.91
Central Asia and North Africa-Middle East	Girls	2023	13	14.07
Central Asia and North Africa-Middle East	Girls	2024	13	14.23
Central Asia and North Africa-Middle East	Girls	2025	13	14.38

Central Asia and North Africa-Middle East	Girls	2026	13	14.54
Central Asia and North Africa-Middle East	Girls	2027	13	14.70
Central Asia and North Africa-Middle East	Girls	2028	13	14.85
Central Asia and North Africa-Middle East	Girls	2029	13	15.01
Central Asia and North Africa-Middle East	Girls	2030	13	15.17
Central Asia and North Africa-Middle East	Girls	2017	14	15.20
Central Asia and North Africa-Middle East	Girls	2018	14	15.38
Central Asia and North Africa-Middle East	Girls	2019	14	15.55
Central Asia and North Africa-Middle East	Girls	2020	14	15.73
Central Asia and North Africa-Middle East	Girls	2021	14	15.90
Central Asia and North Africa-Middle East	Girls	2022	14	16.08
Central Asia and North Africa-Middle East	Girls	2023	14	16.25
Central Asia and North Africa-Middle East	Girls	2024	14	16.43
Central Asia and North Africa-Middle East	Girls	2025	14	16.60
Central Asia and North Africa-Middle East	Girls	2026	14	16.78
Central Asia and North Africa-Middle East	Girls	2027	14	16.96
Central Asia and North Africa-Middle East	Girls	2028	14	17.13
Central Asia and North Africa-Middle East	Girls	2029	14	17.31
Central Asia and North Africa-Middle East	Girls	2030	14	17.49
Central Asia and North Africa-Middle East	Girls	2017	15	16.91
Central Asia and North Africa-Middle East	Girls	2018	15	17.11
Central Asia and North Africa-Middle East	Girls	2019	15	17.30
Central Asia and North Africa-Middle East	Girls	2020	15	17.49
Central Asia and North Africa-Middle East	Girls	2021	15	17.68
Central Asia and North Africa-Middle East	Girls	2022	15	17.87
Central Asia and North Africa-Middle East	Girls	2023	15	18.06
Central Asia and North Africa-Middle East	Girls	2024	15	18.25
Central Asia and North Africa-Middle East	Girls	2025	15	18.43
Central Asia and North Africa-Middle East	Girls	2026	15	18.62
Central Asia and North Africa-Middle East	Girls	2027	15	18.81
Central Asia and North Africa-Middle East	Girls	2028	15	19.00
Central Asia and North Africa-Middle East	Girls	2029	15	19.19
Central Asia and North Africa-Middle East	Girls	2030	15	19.38
Central Asia and North Africa-Middle East	Girls	2017	16	18.31
Central Asia and North Africa-Middle East	Girls	2018	16	18.52
Central Asia and North Africa-Middle East	Girls	2019	16	18.72
Central Asia and North Africa-Middle East	Girls	2020	16	18.92
Central Asia and North Africa-Middle East	Girls	2021	16	19.11
Central Asia and North Africa-Middle East	Girls	2022	16	19.31
Central Asia and North Africa-Middle East	Girls	2023	16	19.51
Central Asia and North Africa-Middle East	Girls	2024	16	19.70
Central Asia and North Africa-Middle East	Girls	2025	16	19.90
Central Asia and North Africa-Middle East	Girls	2026	16	20.10
Central Asia and North Africa-Middle East	Girls	2027	16	20.29
Central Asia and North Africa-Middle East	Girls	2028	16	20.49
Central Asia and North Africa-Middle East	Girls	2029	16	20.68
Central Asia and North Africa-Middle East	Girls	2030	16	20.88
Central Asia and North Africa-Middle East	Girls	2017	17	19.52
Central Asia and North Africa-Middle East	Girls	2018	17	19.73
Central Asia and North Africa-Middle East	Girls	2019	17	19.94
Central Asia and North Africa-Middle East	Girls	2020	17	20.15
Central Asia and North Africa-Middle East	Girls	2021	17	20.36
Central Asia and North Africa-Middle East	Girls	2022	17	20.56

Central Asia and North Africa-Middle East	Girls	2023	17	20.77
Central Asia and North Africa-Middle East	Girls	2024	17	20.97
Central Asia and North Africa-Middle East	Girls	2025	17	21.18
Central Asia and North Africa-Middle East	Girls	2026	17	21.38
Central Asia and North Africa-Middle East	Girls	2027	17	21.59
Central Asia and North Africa-Middle East	Girls	2028	17	21.79
Central Asia and North Africa-Middle East	Girls	2029	17	22.00
Central Asia and North Africa-Middle East	Girls	2030	17	22.20
Central Asia and North Africa-Middle East	Girls	2017	18	20.80
Central Asia and North Africa-Middle East	Girls	2018	18	21.02
Central Asia and North Africa-Middle East	Girls	2019	18	21.25
Central Asia and North Africa-Middle East	Girls	2020	18	21.47
Central Asia and North Africa-Middle East	Girls	2021	18	21.69
Central Asia and North Africa-Middle East	Girls	2022	18	21.91
Central Asia and North Africa-Middle East	Girls	2023	18	22.12
Central Asia and North Africa-Middle East	Girls	2024	18	22.34
Central Asia and North Africa-Middle East	Girls	2025	18	22.56
Central Asia and North Africa-Middle East	Girls	2026	18	22.78
Central Asia and North Africa-Middle East	Girls	2027	18	23.00
Central Asia and North Africa-Middle East	Girls	2028	18	23.22
Central Asia and North Africa-Middle East	Girls	2029	18	23.44
Central Asia and North Africa-Middle East	Girls	2030	18	23.66
East and South East Asia	Boys	2017	12	11.45
East and South East Asia	Boys	2018	12	11.69
East and South East Asia	Boys	2019	12	11.92
East and South East Asia	Boys	2020	12	12.14
East and South East Asia	Boys	2021	12	12.34
East and South East Asia	Boys	2022	12	12.52
East and South East Asia	Boys	2023	12	12.67
East and South East Asia	Boys	2024	12	12.81
East and South East Asia	Boys	2025	12	12.92
East and South East Asia	Boys	2026	12	13.02
East and South East Asia	Boys	2027	12	13.11
East and South East Asia	Boys	2028	12	13.19
East and South East Asia	Boys	2029	12	13.27
East and South East Asia	Boys	2030	12	13.36
East and South East Asia	Boys	2017	13	13.82
East and South East Asia	Boys	2018	13	14.11
East and South East Asia	Boys	2019	13	14.40
East and South East Asia	Boys	2020	13	14.68
East and South East Asia	Boys	2021	13	14.94
East and South East Asia	Boys	2022	13	15.18
East and South East Asia	Boys	2023	13	15.40
East and South East Asia	Boys	2024	13	15.59
East and South East Asia	Boys	2025	13	15.77
East and South East Asia	Boys	2026	13	15.93
East and South East Asia	Boys	2027	13	16.08
East and South East Asia	Boys	2028	13	16.23
East and South East Asia	Boys	2029	13	16.37
East and South East Asia	Boys	2030	13	16.51
East and South East Asia	Boys	2017	14	16.21
East and South East Asia	Boys	2018	14	16.55
East and South East Asia	Boys	2019	14	16.88

East and South East Asia	Boys	2020	14	17.21
East and South East Asia	Boys	2021	14	17.51
East and South East Asia	Boys	2022	14	17.80
East and South East Asia	Boys	2023	14	18.05
East and South East Asia	Boys	2024	14	18.28
East and South East Asia	Boys	2025	14	18.49
East and South East Asia	Boys	2026	14	18.68
East and South East Asia	Boys	2027	14	18.85
East and South East Asia	Boys	2028	14	19.01
East and South East Asia	Boys	2029	14	19.17
East and South East Asia	Boys	2030	14	19.33
East and South East Asia	Boys	2017	15	18.53
East and South East Asia	Boys	2018	15	18.84
East and South East Asia	Boys	2019	15	19.17
East and South East Asia	Boys	2020	15	19.49
East and South East Asia	Boys	2021	15	19.82
East and South East Asia	Boys	2022	15	20.13
East and South East Asia	Boys	2023	15	20.43
East and South East Asia	Boys	2024	15	20.71
East and South East Asia	Boys	2025	15	20.99
East and South East Asia	Boys	2026	15	21.26
East and South East Asia	Boys	2027	15	21.53
East and South East Asia	Boys	2028	15	21.80
East and South East Asia	Boys	2029	15	22.08
East and South East Asia	Boys	2030	15	22.36
East and South East Asia	Boys	2017	16	20.71
East and South East Asia	Boys	2018	16	21.03
East and South East Asia	Boys	2019	16	21.37
East and South East Asia	Boys	2020	16	21.72
East and South East Asia	Boys	2021	16	22.05
East and South East Asia	Boys	2022	16	22.38
East and South East Asia	Boys	2023	16	22.69
East and South East Asia	Boys	2024	16	22.99
East and South East Asia	Boys	2025	16	23.28
East and South East Asia	Boys	2026	16	23.56
East and South East Asia	Boys	2027	16	23.84
East and South East Asia	Boys	2028	16	24.12
East and South East Asia	Boys	2029	16	24.41
East and South East Asia	Boys	2030	16	24.71
East and South East Asia	Boys	2017	17	22.90
East and South East Asia	Boys	2018	17	23.24
East and South East Asia	Boys	2019	17	23.59
East and South East Asia	Boys	2020	17	23.94
East and South East Asia	Boys	2021	17	24.28
East and South East Asia	Boys	2022	17	24.62
East and South East Asia	Boys	2023	17	24.94
East and South East Asia	Boys	2024	17	25.25
East and South East Asia	Boys	2025	17	25.54
East and South East Asia	Boys	2026	17	25.83
East and South East Asia	Boys	2027	17	26.12
East and South East Asia	Boys	2028	17	26.41
East and South East Asia	Boys	2029	17	26.70
East and South East Asia	Boys	2030	17	27.00

East and South East Asia	Boys	2017	18	25.25
East and South East Asia	Boys	2018	18	25.60
East and South East Asia	Boys	2019	18	25.96
East and South East Asia	Boys	2020	18	26.32
East and South East Asia	Boys	2021	18	26.68
East and South East Asia	Boys	2022	18	27.02
East and South East Asia	Boys	2023	18	27.35
East and South East Asia	Boys	2024	18	27.67
East and South East Asia	Boys	2025	18	27.97
East and South East Asia	Boys	2026	18	28.27
East and South East Asia	Boys	2027	18	28.56
East and South East Asia	Boys	2028	18	28.86
East and South East Asia	Boys	2029	18	29.16
East and South East Asia	Boys	2030	18	29.47
East and South East Asia	Girls	2017	12	8.14
East and South East Asia	Girls	2018	12	8.25
East and South East Asia	Girls	2019	12	8.36
East and South East Asia	Girls	2020	12	8.46
East and South East Asia	Girls	2021	12	8.57
East and South East Asia	Girls	2022	12	8.66
East and South East Asia	Girls	2023	12	8.75
East and South East Asia	Girls	2024	12	8.83
East and South East Asia	Girls	2025	12	8.90
East and South East Asia	Girls	2026	12	8.97
East and South East Asia	Girls	2027	12	9.04
East and South East Asia	Girls	2028	12	9.10
East and South East Asia	Girls	2029	12	9.16
East and South East Asia	Girls	2030	12	9.23
East and South East Asia	Girls	2017	13	9.86
East and South East Asia	Girls	2018	13	9.99
East and South East Asia	Girls	2019	13	10.11
East and South East Asia	Girls	2020	13	10.24
East and South East Asia	Girls	2021	13	10.35
East and South East Asia	Girls	2022	13	10.46
East and South East Asia	Girls	2023	13	10.56
East and South East Asia	Girls	2024	13	10.65
East and South East Asia	Girls	2025	13	10.73
East and South East Asia	Girls	2026	13	10.81
East and South East Asia	Girls	2027	13	10.88
East and South East Asia	Girls	2028	13	10.95
East and South East Asia	Girls	2029	13	11.03
East and South East Asia	Girls	2030	13	11.10
East and South East Asia	Girls	2017	14	11.58
East and South East Asia	Girls	2018	14	11.72
East and South East Asia	Girls	2019	14	11.86
East and South East Asia	Girls	2020	14	11.99
East and South East Asia	Girls	2021	14	12.11
East and South East Asia	Girls	2022	14	12.23
East and South East Asia	Girls	2023	14	12.33
East and South East Asia	Girls	2024	14	12.42
East and South East Asia	Girls	2025	14	12.51
East and South East Asia	Girls	2026	14	12.58
East and South East Asia	Girls	2027	14	12.65

East and South East Asia	Girls	2028	14	12.72
East and South East Asia	Girls	2029	14	12.78
East and South East Asia	Girls	2030	14	12.85
East and South East Asia	Girls	2017	15	13.13
East and South East Asia	Girls	2018	15	13.26
East and South East Asia	Girls	2019	15	13.40
East and South East Asia	Girls	2020	15	13.54
East and South East Asia	Girls	2021	15	13.68
East and South East Asia	Girls	2022	15	13.81
East and South East Asia	Girls	2023	15	13.95
East and South East Asia	Girls	2024	15	14.08
East and South East Asia	Girls	2025	15	14.20
East and South East Asia	Girls	2026	15	14.33
East and South East Asia	Girls	2027	15	14.45
East and South East Asia	Girls	2028	15	14.58
East and South East Asia	Girls	2029	15	14.71
East and South East Asia	Girls	2030	15	14.84
East and South East Asia	Girls	2017	16	14.36
East and South East Asia	Girls	2018	16	14.49
East and South East Asia	Girls	2019	16	14.64
East and South East Asia	Girls	2020	16	14.78
East and South East Asia	Girls	2021	16	14.92
East and South East Asia	Girls	2022	16	15.06
East and South East Asia	Girls	2023	16	15.20
East and South East Asia	Girls	2024	16	15.33
East and South East Asia	Girls	2025	16	15.46
East and South East Asia	Girls	2026	16	15.59
East and South East Asia	Girls	2027	16	15.72
East and South East Asia	Girls	2028	16	15.85
East and South East Asia	Girls	2029	16	15.98
East and South East Asia	Girls	2030	16	16.11
East and South East Asia	Girls	2017	17	15.33
East and South East Asia	Girls	2018	17	15.48
East and South East Asia	Girls	2019	17	15.62
East and South East Asia	Girls	2020	17	15.77
East and South East Asia	Girls	2021	17	15.92
East and South East Asia	Girls	2022	17	16.07
East and South East Asia	Girls	2023	17	16.21
East and South East Asia	Girls	2024	17	16.35
East and South East Asia	Girls	2025	17	16.49
East and South East Asia	Girls	2026	17	16.63
East and South East Asia	Girls	2027	17	16.76
East and South East Asia	Girls	2028	17	16.90
East and South East Asia	Girls	2029	17	17.04
East and South East Asia	Girls	2030	17	17.18
East and South East Asia	Girls	2017	18	16.24
East and South East Asia	Girls	2018	18	16.40
East and South East Asia	Girls	2019	18	16.56
East and South East Asia	Girls	2020	18	16.72
East and South East Asia	Girls	2021	18	16.88
East and South East Asia	Girls	2022	18	17.04
East and South East Asia	Girls	2023	18	17.19
East and South East Asia	Girls	2024	18	17.34

East and South East Asia	Girls	2025	18	17.49
East and South East Asia	Girls	2026	18	17.64
East and South East Asia	Girls	2027	18	17.79
East and South East Asia	Girls	2028	18	17.94
East and South East Asia	Girls	2029	18	18.09
East and South East Asia	Girls	2030	18	18.24
High-income Asia Pacific	Boys	2017	12	10.47
High-income Asia Pacific	Boys	2018	12	10.49
High-income Asia Pacific	Boys	2019	12	10.51
High-income Asia Pacific	Boys	2020	12	10.53
High-income Asia Pacific	Boys	2021	12	10.54
High-income Asia Pacific	Boys	2022	12	10.56
High-income Asia Pacific	Boys	2023	12	10.58
High-income Asia Pacific	Boys	2024	12	10.60
High-income Asia Pacific	Boys	2025	12	10.62
High-income Asia Pacific	Boys	2026	12	10.63
High-income Asia Pacific	Boys	2027	12	10.65
High-income Asia Pacific	Boys	2028	12	10.67
High-income Asia Pacific	Boys	2029	12	10.68
High-income Asia Pacific	Boys	2030	12	10.70
High-income Asia Pacific	Boys	2017	13	12.66
High-income Asia Pacific	Boys	2018	13	12.67
High-income Asia Pacific	Boys	2019	13	12.70
High-income Asia Pacific	Boys	2020	13	12.72
High-income Asia Pacific	Boys	2021	13	12.75
High-income Asia Pacific	Boys	2022	13	12.77
High-income Asia Pacific	Boys	2023	13	12.79
High-income Asia Pacific	Boys	2024	13	12.82
High-income Asia Pacific	Boys	2025	13	12.84
High-income Asia Pacific	Boys	2026	13	12.86
High-income Asia Pacific	Boys	2027	13	12.88
High-income Asia Pacific	Boys	2028	13	12.90
High-income Asia Pacific	Boys	2029	13	12.92
High-income Asia Pacific	Boys	2030	13	12.95
High-income Asia Pacific	Boys	2017	14	14.94
High-income Asia Pacific	Boys	2018	14	14.96
High-income Asia Pacific	Boys	2019	14	14.99
High-income Asia Pacific	Boys	2020	14	15.02
High-income Asia Pacific	Boys	2021	14	15.05
High-income Asia Pacific	Boys	2022	14	15.08
High-income Asia Pacific	Boys	2023	14	15.10
High-income Asia Pacific	Boys	2024	14	15.13
High-income Asia Pacific	Boys	2025	14	15.16
High-income Asia Pacific	Boys	2026	14	15.18
High-income Asia Pacific	Boys	2027	14	15.21
High-income Asia Pacific	Boys	2028	14	15.23
High-income Asia Pacific	Boys	2029	14	15.26
High-income Asia Pacific	Boys	2030	14	15.28
High-income Asia Pacific	Boys	2017	15	17.44
High-income Asia Pacific	Boys	2018	15	17.42
High-income Asia Pacific	Boys	2019	15	17.39
High-income Asia Pacific	Boys	2020	15	17.35
High-income Asia Pacific	Boys	2021	15	17.31

High-income Asia Pacific	Boys	2022	15	17.25
High-income Asia Pacific	Boys	2023	15	17.18
High-income Asia Pacific	Boys	2024	15	17.11
High-income Asia Pacific	Boys	2025	15	17.03
High-income Asia Pacific	Boys	2026	15	16.94
High-income Asia Pacific	Boys	2027	15	16.85
High-income Asia Pacific	Boys	2028	15	16.75
High-income Asia Pacific	Boys	2029	15	16.65
High-income Asia Pacific	Boys	2030	15	16.54
High-income Asia Pacific	Boys	2017	16	19.65
High-income Asia Pacific	Boys	2018	16	19.63
High-income Asia Pacific	Boys	2019	16	19.61
High-income Asia Pacific	Boys	2020	16	19.57
High-income Asia Pacific	Boys	2021	16	19.52
High-income Asia Pacific	Boys	2022	16	19.46
High-income Asia Pacific	Boys	2023	16	19.39
High-income Asia Pacific	Boys	2024	16	19.32
High-income Asia Pacific	Boys	2025	16	19.24
High-income Asia Pacific	Boys	2026	16	19.15
High-income Asia Pacific	Boys	2027	16	19.05
High-income Asia Pacific	Boys	2028	16	18.95
High-income Asia Pacific	Boys	2029	16	18.85
High-income Asia Pacific	Boys	2030	16	18.74
High-income Asia Pacific	Boys	2017	17	21.84
High-income Asia Pacific	Boys	2018	17	21.83
High-income Asia Pacific	Boys	2019	17	21.80
High-income Asia Pacific	Boys	2020	17	21.76
High-income Asia Pacific	Boys	2021	17	21.71
High-income Asia Pacific	Boys	2022	17	21.65
High-income Asia Pacific	Boys	2023	17	21.58
High-income Asia Pacific	Boys	2024	17	21.50
High-income Asia Pacific	Boys	2025	17	21.42
High-income Asia Pacific	Boys	2026	17	21.33
High-income Asia Pacific	Boys	2027	17	21.24
High-income Asia Pacific	Boys	2028	17	21.13
High-income Asia Pacific	Boys	2029	17	21.03
High-income Asia Pacific	Boys	2030	17	20.92
High-income Asia Pacific	Boys	2017	18	24.07
High-income Asia Pacific	Boys	2018	18	24.05
High-income Asia Pacific	Boys	2019	18	24.02
High-income Asia Pacific	Boys	2020	18	23.97
High-income Asia Pacific	Boys	2021	18	23.92
High-income Asia Pacific	Boys	2022	18	23.85
High-income Asia Pacific	Boys	2023	18	23.78
High-income Asia Pacific	Boys	2024	18	23.70
High-income Asia Pacific	Boys	2025	18	23.61
High-income Asia Pacific	Boys	2026	18	23.51
High-income Asia Pacific	Boys	2027	18	23.41
High-income Asia Pacific	Boys	2028	18	23.31
High-income Asia Pacific	Boys	2029	18	23.19
High-income Asia Pacific	Boys	2030	18	23.08
High-income Asia Pacific	Girls	2017	12	8.03
High-income Asia Pacific	Girls	2018	12	8.04

High-income Asia Pacific	Girls	2019	12	8.05
High-income Asia Pacific	Girls	2020	12	8.07
High-income Asia Pacific	Girls	2021	12	8.08
High-income Asia Pacific	Girls	2022	12	8.09
High-income Asia Pacific	Girls	2023	12	8.11
High-income Asia Pacific	Girls	2024	12	8.12
High-income Asia Pacific	Girls	2025	12	8.13
High-income Asia Pacific	Girls	2026	12	8.14
High-income Asia Pacific	Girls	2027	12	8.16
High-income Asia Pacific	Girls	2028	12	8.17
High-income Asia Pacific	Girls	2029	12	8.18
High-income Asia Pacific	Girls	2030	12	8.20
High-income Asia Pacific	Girls	2017	13	9.67
High-income Asia Pacific	Girls	2018	13	9.68
High-income Asia Pacific	Girls	2019	13	9.70
High-income Asia Pacific	Girls	2020	13	9.71
High-income Asia Pacific	Girls	2021	13	9.73
High-income Asia Pacific	Girls	2022	13	9.75
High-income Asia Pacific	Girls	2023	13	9.76
High-income Asia Pacific	Girls	2024	13	9.78
High-income Asia Pacific	Girls	2025	13	9.79
High-income Asia Pacific	Girls	2026	13	9.80
High-income Asia Pacific	Girls	2027	13	9.82
High-income Asia Pacific	Girls	2028	13	9.83
High-income Asia Pacific	Girls	2029	13	9.85
High-income Asia Pacific	Girls	2030	13	9.86
High-income Asia Pacific	Girls	2017	14	11.28
High-income Asia Pacific	Girls	2018	14	11.30
High-income Asia Pacific	Girls	2019	14	11.32
High-income Asia Pacific	Girls	2020	14	11.33
High-income Asia Pacific	Girls	2021	14	11.35
High-income Asia Pacific	Girls	2022	14	11.37
High-income Asia Pacific	Girls	2023	14	11.38
High-income Asia Pacific	Girls	2024	14	11.40
High-income Asia Pacific	Girls	2025	14	11.41
High-income Asia Pacific	Girls	2026	14	11.43
High-income Asia Pacific	Girls	2027	14	11.44
High-income Asia Pacific	Girls	2028	14	11.46
High-income Asia Pacific	Girls	2029	14	11.47
High-income Asia Pacific	Girls	2030	14	11.49
High-income Asia Pacific	Girls	2017	15	12.76
High-income Asia Pacific	Girls	2018	15	12.76
High-income Asia Pacific	Girls	2019	15	12.76
High-income Asia Pacific	Girls	2020	15	12.75
High-income Asia Pacific	Girls	2021	15	12.74
High-income Asia Pacific	Girls	2022	15	12.73
High-income Asia Pacific	Girls	2023	15	12.72
High-income Asia Pacific	Girls	2024	15	12.70
High-income Asia Pacific	Girls	2025	15	12.69
High-income Asia Pacific	Girls	2026	15	12.67
High-income Asia Pacific	Girls	2027	15	12.66
High-income Asia Pacific	Girls	2028	15	12.64
High-income Asia Pacific	Girls	2029	15	12.63

High-income Asia Pacific	Girls	2030	15	12.61
High-income Asia Pacific	Girls	2017	16	13.79
High-income Asia Pacific	Girls	2018	16	13.79
High-income Asia Pacific	Girls	2019	16	13.78
High-income Asia Pacific	Girls	2020	16	13.78
High-income Asia Pacific	Girls	2021	16	13.77
High-income Asia Pacific	Girls	2022	16	13.76
High-income Asia Pacific	Girls	2023	16	13.74
High-income Asia Pacific	Girls	2024	16	13.73
High-income Asia Pacific	Girls	2025	16	13.72
High-income Asia Pacific	Girls	2026	16	13.70
High-income Asia Pacific	Girls	2027	16	13.69
High-income Asia Pacific	Girls	2028	16	13.67
High-income Asia Pacific	Girls	2029	16	13.66
High-income Asia Pacific	Girls	2030	16	13.64
High-income Asia Pacific	Girls	2017	17	14.49
High-income Asia Pacific	Girls	2018	17	14.49
High-income Asia Pacific	Girls	2019	17	14.48
High-income Asia Pacific	Girls	2020	17	14.47
High-income Asia Pacific	Girls	2021	17	14.47
High-income Asia Pacific	Girls	2022	17	14.45
High-income Asia Pacific	Girls	2023	17	14.44
High-income Asia Pacific	Girls	2024	17	14.43
High-income Asia Pacific	Girls	2025	17	14.42
High-income Asia Pacific	Girls	2026	17	14.40
High-income Asia Pacific	Girls	2027	17	14.39
High-income Asia Pacific	Girls	2028	17	14.38
High-income Asia Pacific	Girls	2029	17	14.36
High-income Asia Pacific	Girls	2030	17	14.35
High-income Asia Pacific	Girls	2017	18	15.00
High-income Asia Pacific	Girls	2018	18	15.00
High-income Asia Pacific	Girls	2019	18	14.99
High-income Asia Pacific	Girls	2020	18	14.98
High-income Asia Pacific	Girls	2021	18	14.97
High-income Asia Pacific	Girls	2022	18	14.96
High-income Asia Pacific	Girls	2023	18	14.95
High-income Asia Pacific	Girls	2024	18	14.93
High-income Asia Pacific	Girls	2025	18	14.92
High-income Asia Pacific	Girls	2026	18	14.91
High-income Asia Pacific	Girls	2027	18	14.90
High-income Asia Pacific	Girls	2028	18	14.88
High-income Asia Pacific	Girls	2029	18	14.87
High-income Asia Pacific	Girls	2030	18	14.86
High-income Western countries	Boys	2017	12	14.71
High-income Western countries	Boys	2018	12	14.78
High-income Western countries	Boys	2019	12	14.85
High-income Western countries	Boys	2020	12	14.92
High-income Western countries	Boys	2021	12	14.99
High-income Western countries	Boys	2022	12	15.06
High-income Western countries	Boys	2023	12	15.12
High-income Western countries	Boys	2024	12	15.19
High-income Western countries	Boys	2025	12	15.26
High-income Western countries	Boys	2026	12	15.32

High-income Western countries	Boys	2027	12	15.39
High-income Western countries	Boys	2028	12	15.46
High-income Western countries	Boys	2029	12	15.53
High-income Western countries	Boys	2030	12	15.59
High-income Western countries	Boys	2017	13	17.99
High-income Western countries	Boys	2018	13	18.08
High-income Western countries	Boys	2019	13	18.17
High-income Western countries	Boys	2020	13	18.26
High-income Western countries	Boys	2021	13	18.34
High-income Western countries	Boys	2022	13	18.43
High-income Western countries	Boys	2023	13	18.52
High-income Western countries	Boys	2024	13	18.61
High-income Western countries	Boys	2025	13	18.70
High-income Western countries	Boys	2026	13	18.80
High-income Western countries	Boys	2027	13	18.89
High-income Western countries	Boys	2028	13	18.98
High-income Western countries	Boys	2029	13	19.08
High-income Western countries	Boys	2030	13	19.17
High-income Western countries	Boys	2017	14	21.27
High-income Western countries	Boys	2018	14	21.38
High-income Western countries	Boys	2019	14	21.48
High-income Western countries	Boys	2020	14	21.59
High-income Western countries	Boys	2021	14	21.69
High-income Western countries	Boys	2022	14	21.80
High-income Western countries	Boys	2023	14	21.90
High-income Western countries	Boys	2024	14	22.01
High-income Western countries	Boys	2025	14	22.11
High-income Western countries	Boys	2026	14	22.22
High-income Western countries	Boys	2027	14	22.32
High-income Western countries	Boys	2028	14	22.43
High-income Western countries	Boys	2029	14	22.54
High-income Western countries	Boys	2030	14	22.64
High-income Western countries	Boys	2017	15	24.30
High-income Western countries	Boys	2018	15	24.44
High-income Western countries	Boys	2019	15	24.58
High-income Western countries	Boys	2020	15	24.73
High-income Western countries	Boys	2021	15	24.87
High-income Western countries	Boys	2022	15	25.02
High-income Western countries	Boys	2023	15	25.18
High-income Western countries	Boys	2024	15	25.34
High-income Western countries	Boys	2025	15	25.49
High-income Western countries	Boys	2026	15	25.66
High-income Western countries	Boys	2027	15	25.82
High-income Western countries	Boys	2028	15	25.98
High-income Western countries	Boys	2029	15	26.15
High-income Western countries	Boys	2030	15	26.31
High-income Western countries	Boys	2017	16	27.12
High-income Western countries	Boys	2018	16	27.26
High-income Western countries	Boys	2019	16	27.41
High-income Western countries	Boys	2020	16	27.56
High-income Western countries	Boys	2021	16	27.72
High-income Western countries	Boys	2022	16	27.88
High-income Western countries	Boys	2023	16	28.05

High-income Western countries	Boys	2024	16	28.22
High-income Western countries	Boys	2025	16	28.39
High-income Western countries	Boys	2026	16	28.56
High-income Western countries	Boys	2027	16	28.73
High-income Western countries	Boys	2028	16	28.91
High-income Western countries	Boys	2029	16	29.08
High-income Western countries	Boys	2030	16	29.26
High-income Western countries	Boys	2017	17	29.88
High-income Western countries	Boys	2018	17	30.03
High-income Western countries	Boys	2019	17	30.19
High-income Western countries	Boys	2020	17	30.35
High-income Western countries	Boys	2021	17	30.51
High-income Western countries	Boys	2022	17	30.68
High-income Western countries	Boys	2023	17	30.85
High-income Western countries	Boys	2024	17	31.02
High-income Western countries	Boys	2025	17	31.20
High-income Western countries	Boys	2026	17	31.38
High-income Western countries	Boys	2027	17	31.56
High-income Western countries	Boys	2028	17	31.74
High-income Western countries	Boys	2029	17	31.93
High-income Western countries	Boys	2030	17	32.11
High-income Western countries	Boys	2017	18	32.83
High-income Western countries	Boys	2018	18	32.99
High-income Western countries	Boys	2019	18	33.15
High-income Western countries	Boys	2020	18	33.31
High-income Western countries	Boys	2021	18	33.48
High-income Western countries	Boys	2022	18	33.65
High-income Western countries	Boys	2023	18	33.83
High-income Western countries	Boys	2024	18	34.00
High-income Western countries	Boys	2025	18	34.19
High-income Western countries	Boys	2026	18	34.37
High-income Western countries	Boys	2027	18	34.55
High-income Western countries	Boys	2028	18	34.74
High-income Western countries	Boys	2029	18	34.93
High-income Western countries	Boys	2030	18	35.12
High-income Western countries	Girls	2017	12	12.59
High-income Western countries	Girls	2018	12	12.64
High-income Western countries	Girls	2019	12	12.69
High-income Western countries	Girls	2020	12	12.74
High-income Western countries	Girls	2021	12	12.78
High-income Western countries	Girls	2022	12	12.83
High-income Western countries	Girls	2023	12	12.87
High-income Western countries	Girls	2024	12	12.92
High-income Western countries	Girls	2025	12	12.97
High-income Western countries	Girls	2026	12	13.01
High-income Western countries	Girls	2027	12	13.06
High-income Western countries	Girls	2028	12	13.10
High-income Western countries	Girls	2029	12	13.15
High-income Western countries	Girls	2030	12	13.20
High-income Western countries	Girls	2017	13	15.04
High-income Western countries	Girls	2018	13	15.10
High-income Western countries	Girls	2019	13	15.15
High-income Western countries	Girls	2020	13	15.21

High-income Western countries	Girls	2021	13	15.26
High-income Western countries	Girls	2022	13	15.32
High-income Western countries	Girls	2023	13	15.37
High-income Western countries	Girls	2024	13	15.43
High-income Western countries	Girls	2025	13	15.48
High-income Western countries	Girls	2026	13	15.54
High-income Western countries	Girls	2027	13	15.59
High-income Western countries	Girls	2028	13	15.65
High-income Western countries	Girls	2029	13	15.70
High-income Western countries	Girls	2030	13	15.76
High-income Western countries	Girls	2017	14	17.27
High-income Western countries	Girls	2018	14	17.33
High-income Western countries	Girls	2019	14	17.39
High-income Western countries	Girls	2020	14	17.45
High-income Western countries	Girls	2021	14	17.52
High-income Western countries	Girls	2022	14	17.58
High-income Western countries	Girls	2023	14	17.64
High-income Western countries	Girls	2024	14	17.70
High-income Western countries	Girls	2025	14	17.76
High-income Western countries	Girls	2026	14	17.82
High-income Western countries	Girls	2027	14	17.89
High-income Western countries	Girls	2028	14	17.95
High-income Western countries	Girls	2029	14	18.01
High-income Western countries	Girls	2030	14	18.07
High-income Western countries	Girls	2017	15	19.10
High-income Western countries	Girls	2018	15	19.17
High-income Western countries	Girls	2019	15	19.25
High-income Western countries	Girls	2020	15	19.32
High-income Western countries	Girls	2021	15	19.40
High-income Western countries	Girls	2022	15	19.48
High-income Western countries	Girls	2023	15	19.56
High-income Western countries	Girls	2024	15	19.64
High-income Western countries	Girls	2025	15	19.72
High-income Western countries	Girls	2026	15	19.81
High-income Western countries	Girls	2027	15	19.89
High-income Western countries	Girls	2028	15	19.97
High-income Western countries	Girls	2029	15	20.06
High-income Western countries	Girls	2030	15	20.14
High-income Western countries	Girls	2017	16	20.55
High-income Western countries	Girls	2018	16	20.62
High-income Western countries	Girls	2019	16	20.70
High-income Western countries	Girls	2020	16	20.78
High-income Western countries	Girls	2021	16	20.86
High-income Western countries	Girls	2022	16	20.94
High-income Western countries	Girls	2023	16	21.02
High-income Western countries	Girls	2024	16	21.11
High-income Western countries	Girls	2025	16	21.19
High-income Western countries	Girls	2026	16	21.28
High-income Western countries	Girls	2027	16	21.36
High-income Western countries	Girls	2028	16	21.45
High-income Western countries	Girls	2029	16	21.53
High-income Western countries	Girls	2030	16	21.62
High-income Western countries	Girls	2017	17	21.82

High-income Western countries	Girls	2018	17	21.90
High-income Western countries	Girls	2019	17	21.98
High-income Western countries	Girls	2020	17	22.06
High-income Western countries	Girls	2021	17	22.14
High-income Western countries	Girls	2022	17	22.23
High-income Western countries	Girls	2023	17	22.31
High-income Western countries	Girls	2024	17	22.40
High-income Western countries	Girls	2025	17	22.48
High-income Western countries	Girls	2026	17	22.57
High-income Western countries	Girls	2027	17	22.66
High-income Western countries	Girls	2028	17	22.75
High-income Western countries	Girls	2029	17	22.84
High-income Western countries	Girls	2030	17	22.92
High-income Western countries	Girls	2017	18	23.24
High-income Western countries	Girls	2018	18	23.32
High-income Western countries	Girls	2019	18	23.41
High-income Western countries	Girls	2020	18	23.49
High-income Western countries	Girls	2021	18	23.58
High-income Western countries	Girls	2022	18	23.67
High-income Western countries	Girls	2023	18	23.76
High-income Western countries	Girls	2024	18	23.85
High-income Western countries	Girls	2025	18	23.94
High-income Western countries	Girls	2026	18	24.03
High-income Western countries	Girls	2027	18	24.12
High-income Western countries	Girls	2028	18	24.21
High-income Western countries	Girls	2029	18	24.30
High-income Western countries	Girls	2030	18	24.39
Latin America and Caribbean	Boys	2017	12	13.00
Latin America and Caribbean	Boys	2018	12	13.18
Latin America and Caribbean	Boys	2019	12	13.37
Latin America and Caribbean	Boys	2020	12	13.55
Latin America and Caribbean	Boys	2021	12	13.73
Latin America and Caribbean	Boys	2022	12	13.92
Latin America and Caribbean	Boys	2023	12	14.10
Latin America and Caribbean	Boys	2024	12	14.28
Latin America and Caribbean	Boys	2025	12	14.46
Latin America and Caribbean	Boys	2026	12	14.63
Latin America and Caribbean	Boys	2027	12	14.81
Latin America and Caribbean	Boys	2028	12	14.99
Latin America and Caribbean	Boys	2029	12	15.17
Latin America and Caribbean	Boys	2030	12	15.35
Latin America and Caribbean	Boys	2017	13	15.76
Latin America and Caribbean	Boys	2018	13	15.99
Latin America and Caribbean	Boys	2019	13	16.22
Latin America and Caribbean	Boys	2020	13	16.46
Latin America and Caribbean	Boys	2021	13	16.69
Latin America and Caribbean	Boys	2022	13	16.92
Latin America and Caribbean	Boys	2023	13	17.14
Latin America and Caribbean	Boys	2024	13	17.37
Latin America and Caribbean	Boys	2025	13	17.59
Latin America and Caribbean	Boys	2026	13	17.82
Latin America and Caribbean	Boys	2027	13	18.04
Latin America and Caribbean	Boys	2028	13	18.26

Latin America and Caribbean	Boys	2029	13	18.49
Latin America and Caribbean	Boys	2030	13	18.71
Latin America and Caribbean	Boys	2017	14	18.54
Latin America and Caribbean	Boys	2018	14	18.81
Latin America and Caribbean	Boys	2019	14	19.09
Latin America and Caribbean	Boys	2020	14	19.36
Latin America and Caribbean	Boys	2021	14	19.63
Latin America and Caribbean	Boys	2022	14	19.91
Latin America and Caribbean	Boys	2023	14	20.18
Latin America and Caribbean	Boys	2024	14	20.45
Latin America and Caribbean	Boys	2025	14	20.71
Latin America and Caribbean	Boys	2026	14	20.98
Latin America and Caribbean	Boys	2027	14	21.24
Latin America and Caribbean	Boys	2028	14	21.50
Latin America and Caribbean	Boys	2029	14	21.76
Latin America and Caribbean	Boys	2030	14	22.02
Latin America and Caribbean	Boys	2017	15	21.16
Latin America and Caribbean	Boys	2018	15	21.45
Latin America and Caribbean	Boys	2019	15	21.74
Latin America and Caribbean	Boys	2020	15	22.03
Latin America and Caribbean	Boys	2021	15	22.33
Latin America and Caribbean	Boys	2022	15	22.63
Latin America and Caribbean	Boys	2023	15	22.93
Latin America and Caribbean	Boys	2024	15	23.23
Latin America and Caribbean	Boys	2025	15	23.54
Latin America and Caribbean	Boys	2026	15	23.85
Latin America and Caribbean	Boys	2027	15	24.16
Latin America and Caribbean	Boys	2028	15	24.47
Latin America and Caribbean	Boys	2029	15	24.79
Latin America and Caribbean	Boys	2030	15	25.10
Latin America and Caribbean	Boys	2017	16	23.64
Latin America and Caribbean	Boys	2018	16	23.94
Latin America and Caribbean	Boys	2019	16	24.24
Latin America and Caribbean	Boys	2020	16	24.55
Latin America and Caribbean	Boys	2021	16	24.86
Latin America and Caribbean	Boys	2022	16	25.18
Latin America and Caribbean	Boys	2023	16	25.50
Latin America and Caribbean	Boys	2024	16	25.83
Latin America and Caribbean	Boys	2025	16	26.15
Latin America and Caribbean	Boys	2026	16	26.48
Latin America and Caribbean	Boys	2027	16	26.81
Latin America and Caribbean	Boys	2028	16	27.15
Latin America and Caribbean	Boys	2029	16	27.49
Latin America and Caribbean	Boys	2030	16	27.83
Latin America and Caribbean	Boys	2017	17	26.10
Latin America and Caribbean	Boys	2018	17	26.40
Latin America and Caribbean	Boys	2019	17	26.72
Latin America and Caribbean	Boys	2020	17	27.03
Latin America and Caribbean	Boys	2021	17	27.36
Latin America and Caribbean	Boys	2022	17	27.69
Latin America and Caribbean	Boys	2023	17	28.02
Latin America and Caribbean	Boys	2024	17	28.35
Latin America and Caribbean	Boys	2025	17	28.69

Latin America and Caribbean	Boys	2026	17	29.03
Latin America and Caribbean	Boys	2027	17	29.38
Latin America and Caribbean	Boys	2028	17	29.73
Latin America and Caribbean	Boys	2029	17	30.08
Latin America and Caribbean	Boys	2030	17	30.43
Latin America and Caribbean	Boys	2017	18	28.71
Latin America and Caribbean	Boys	2018	18	29.02
Latin America and Caribbean	Boys	2019	18	29.34
Latin America and Caribbean	Boys	2020	18	29.67
Latin America and Caribbean	Boys	2021	18	30.00
Latin America and Caribbean	Boys	2022	18	30.33
Latin America and Caribbean	Boys	2023	18	30.67
Latin America and Caribbean	Boys	2024	18	31.01
Latin America and Caribbean	Boys	2025	18	31.36
Latin America and Caribbean	Boys	2026	18	31.71
Latin America and Caribbean	Boys	2027	18	32.06
Latin America and Caribbean	Boys	2028	18	32.42
Latin America and Caribbean	Boys	2029	18	32.77
Latin America and Caribbean	Boys	2030	18	33.13
Latin America and Caribbean	Girls	2017	12	11.46
Latin America and Caribbean	Girls	2018	12	11.57
Latin America and Caribbean	Girls	2019	12	11.68
Latin America and Caribbean	Girls	2020	12	11.79
Latin America and Caribbean	Girls	2021	12	11.89
Latin America and Caribbean	Girls	2022	12	12.00
Latin America and Caribbean	Girls	2023	12	12.11
Latin America and Caribbean	Girls	2024	12	12.22
Latin America and Caribbean	Girls	2025	12	12.33
Latin America and Caribbean	Girls	2026	12	12.44
Latin America and Caribbean	Girls	2027	12	12.55
Latin America and Caribbean	Girls	2028	12	12.66
Latin America and Caribbean	Girls	2029	12	12.77
Latin America and Caribbean	Girls	2030	12	12.88
Latin America and Caribbean	Girls	2017	13	13.72
Latin America and Caribbean	Girls	2018	13	13.86
Latin America and Caribbean	Girls	2019	13	14.00
Latin America and Caribbean	Girls	2020	13	14.13
Latin America and Caribbean	Girls	2021	13	14.27
Latin America and Caribbean	Girls	2022	13	14.41
Latin America and Caribbean	Girls	2023	13	14.54
Latin America and Caribbean	Girls	2024	13	14.68
Latin America and Caribbean	Girls	2025	13	14.82
Latin America and Caribbean	Girls	2026	13	14.95
Latin America and Caribbean	Girls	2027	13	15.09
Latin America and Caribbean	Girls	2028	13	15.22
Latin America and Caribbean	Girls	2029	13	15.36
Latin America and Caribbean	Girls	2030	13	15.49
Latin America and Caribbean	Girls	2017	14	15.82
Latin America and Caribbean	Girls	2018	14	15.97
Latin America and Caribbean	Girls	2019	14	16.12
Latin America and Caribbean	Girls	2020	14	16.27
Latin America and Caribbean	Girls	2021	14	16.42
Latin America and Caribbean	Girls	2022	14	16.57

Latin America and Caribbean	Girls	2023	14	16.72
Latin America and Caribbean	Girls	2024	14	16.87
Latin America and Caribbean	Girls	2025	14	17.02
Latin America and Caribbean	Girls	2026	14	17.17
Latin America and Caribbean	Girls	2027	14	17.31
Latin America and Caribbean	Girls	2028	14	17.46
Latin America and Caribbean	Girls	2029	14	17.61
Latin America and Caribbean	Girls	2030	14	17.76
Latin America and Caribbean	Girls	2017	15	17.54
Latin America and Caribbean	Girls	2018	15	17.70
Latin America and Caribbean	Girls	2019	15	17.85
Latin America and Caribbean	Girls	2020	15	18.01
Latin America and Caribbean	Girls	2021	15	18.16
Latin America and Caribbean	Girls	2022	15	18.32
Latin America and Caribbean	Girls	2023	15	18.48
Latin America and Caribbean	Girls	2024	15	18.64
Latin America and Caribbean	Girls	2025	15	18.79
Latin America and Caribbean	Girls	2026	15	18.95
Latin America and Caribbean	Girls	2027	15	19.11
Latin America and Caribbean	Girls	2028	15	19.26
Latin America and Caribbean	Girls	2029	15	19.42
Latin America and Caribbean	Girls	2030	15	19.58
Latin America and Caribbean	Girls	2017	16	18.91
Latin America and Caribbean	Girls	2018	16	19.06
Latin America and Caribbean	Girls	2019	16	19.22
Latin America and Caribbean	Girls	2020	16	19.39
Latin America and Caribbean	Girls	2021	16	19.55
Latin America and Caribbean	Girls	2022	16	19.71
Latin America and Caribbean	Girls	2023	16	19.87
Latin America and Caribbean	Girls	2024	16	20.03
Latin America and Caribbean	Girls	2025	16	20.20
Latin America and Caribbean	Girls	2026	16	20.36
Latin America and Caribbean	Girls	2027	16	20.52
Latin America and Caribbean	Girls	2028	16	20.69
Latin America and Caribbean	Girls	2029	16	20.85
Latin America and Caribbean	Girls	2030	16	21.01
Latin America and Caribbean	Girls	2017	17	20.05
Latin America and Caribbean	Girls	2018	17	20.21
Latin America and Caribbean	Girls	2019	17	20.38
Latin America and Caribbean	Girls	2020	17	20.55
Latin America and Caribbean	Girls	2021	17	20.72
Latin America and Caribbean	Girls	2022	17	20.89
Latin America and Caribbean	Girls	2023	17	21.06
Latin America and Caribbean	Girls	2024	17	21.23
Latin America and Caribbean	Girls	2025	17	21.40
Latin America and Caribbean	Girls	2026	17	21.57
Latin America and Caribbean	Girls	2027	17	21.74
Latin America and Caribbean	Girls	2028	17	21.91
Latin America and Caribbean	Girls	2029	17	22.08
Latin America and Caribbean	Girls	2030	17	22.25
Latin America and Caribbean	Girls	2017	18	21.26
Latin America and Caribbean	Girls	2018	18	21.44
Latin America and Caribbean	Girls	2019	18	21.62

Latin America and Caribbean	Girls	2020	18	21.79
Latin America and Caribbean	Girls	2021	18	21.97
Latin America and Caribbean	Girls	2022	18	22.15
Latin America and Caribbean	Girls	2023	18	22.32
Latin America and Caribbean	Girls	2024	18	22.50
Latin America and Caribbean	Girls	2025	18	22.67
Latin America and Caribbean	Girls	2026	18	22.85
Latin America and Caribbean	Girls	2027	18	23.02
Latin America and Caribbean	Girls	2028	18	23.20
Latin America and Caribbean	Girls	2029	18	23.37
Latin America and Caribbean	Girls	2030	18	23.55
Oceania	Boys	2017	12	12.02
Oceania	Boys	2018	12	12.28
Oceania	Boys	2019	12	12.54
Oceania	Boys	2020	12	12.81
Oceania	Boys	2021	12	13.09
Oceania	Boys	2022	12	13.36
Oceania	Boys	2023	12	13.65
Oceania	Boys	2024	12	13.93
Oceania	Boys	2025	12	14.22
Oceania	Boys	2026	12	14.52
Oceania	Boys	2027	12	14.82
Oceania	Boys	2028	12	15.12
Oceania	Boys	2029	12	15.43
Oceania	Boys	2030	12	15.74
Oceania	Boys	2017	13	14.66
Oceania	Boys	2018	13	14.99
Oceania	Boys	2019	13	15.32
Oceania	Boys	2020	13	15.66
Oceania	Boys	2021	13	16.00
Oceania	Boys	2022	13	16.35
Oceania	Boys	2023	13	16.71
Oceania	Boys	2024	13	17.08
Oceania	Boys	2025	13	17.45
Oceania	Boys	2026	13	17.83
Oceania	Boys	2027	13	18.22
Oceania	Boys	2028	13	18.61
Oceania	Boys	2029	13	19.00
Oceania	Boys	2030	13	19.40
Oceania	Boys	2017	14	17.43
Oceania	Boys	2018	14	17.80
Oceania	Boys	2019	14	18.19
Oceania	Boys	2020	14	18.59
Oceania	Boys	2021	14	19.00
Oceania	Boys	2022	14	19.42
Oceania	Boys	2023	14	19.85
Oceania	Boys	2024	14	20.29
Oceania	Boys	2025	14	20.74
Oceania	Boys	2026	14	21.19
Oceania	Boys	2027	14	21.66
Oceania	Boys	2028	14	22.13
Oceania	Boys	2029	14	22.61
Oceania	Boys	2030	14	23.10

Oceania	Boys	2017	15	20.13
Oceania	Boys	2018	15	20.52
Oceania	Boys	2019	15	20.91
Oceania	Boys	2020	15	21.30
Oceania	Boys	2021	15	21.69
Oceania	Boys	2022	15	22.08
Oceania	Boys	2023	15	22.47
Oceania	Boys	2024	15	22.86
Oceania	Boys	2025	15	23.25
Oceania	Boys	2026	15	23.64
Oceania	Boys	2027	15	24.03
Oceania	Boys	2028	15	24.42
Oceania	Boys	2029	15	24.81
Oceania	Boys	2030	15	25.20
Oceania	Boys	2017	16	22.75
Oceania	Boys	2018	16	23.16
Oceania	Boys	2019	16	23.57
Oceania	Boys	2020	16	23.98
Oceania	Boys	2021	16	24.39
Oceania	Boys	2022	16	24.80
Oceania	Boys	2023	16	25.20
Oceania	Boys	2024	16	25.61
Oceania	Boys	2025	16	26.02
Oceania	Boys	2026	16	26.43
Oceania	Boys	2027	16	26.84
Oceania	Boys	2028	16	27.25
Oceania	Boys	2029	16	27.66
Oceania	Boys	2030	16	28.07
Oceania	Boys	2017	17	25.38
Oceania	Boys	2018	17	25.80
Oceania	Boys	2019	17	26.22
Oceania	Boys	2020	17	26.64
Oceania	Boys	2021	17	27.06
Oceania	Boys	2022	17	27.48
Oceania	Boys	2023	17	27.90
Oceania	Boys	2024	17	28.32
Oceania	Boys	2025	17	28.74
Oceania	Boys	2026	17	29.16
Oceania	Boys	2027	17	29.58
Oceania	Boys	2028	17	30.00
Oceania	Boys	2029	17	30.43
Oceania	Boys	2030	17	30.85
Oceania	Boys	2017	18	28.14
Oceania	Boys	2018	18	28.57
Oceania	Boys	2019	18	29.01
Oceania	Boys	2020	18	29.44
Oceania	Boys	2021	18	29.87
Oceania	Boys	2022	18	30.31
Oceania	Boys	2023	18	30.74
Oceania	Boys	2024	18	31.17
Oceania	Boys	2025	18	31.60
Oceania	Boys	2026	18	32.04
Oceania	Boys	2027	18	32.47

Oceania	Boys	2028	18	32.90
Oceania	Boys	2029	18	33.34
Oceania	Boys	2030	18	33.77
Oceania	Girls	2017	12	13.20
Oceania	Girls	2018	12	13.40
Oceania	Girls	2019	12	13.61
Oceania	Girls	2020	12	13.82
Oceania	Girls	2021	12	14.04
Oceania	Girls	2022	12	14.26
Oceania	Girls	2023	12	14.48
Oceania	Girls	2024	12	14.71
Oceania	Girls	2025	12	14.94
Oceania	Girls	2026	12	15.17
Oceania	Girls	2027	12	15.40
Oceania	Girls	2028	12	15.64
Oceania	Girls	2029	12	15.88
Oceania	Girls	2030	12	16.12
Oceania	Girls	2017	13	15.86
Oceania	Girls	2018	13	16.10
Oceania	Girls	2019	13	16.34
Oceania	Girls	2020	13	16.59
Oceania	Girls	2021	13	16.84
Oceania	Girls	2022	13	17.09
Oceania	Girls	2023	13	17.34
Oceania	Girls	2024	13	17.59
Oceania	Girls	2025	13	17.84
Oceania	Girls	2026	13	18.09
Oceania	Girls	2027	13	18.34
Oceania	Girls	2028	13	18.59
Oceania	Girls	2029	13	18.84
Oceania	Girls	2030	13	19.09
Oceania	Girls	2017	14	18.30
Oceania	Girls	2018	14	18.57
Oceania	Girls	2019	14	18.83
Oceania	Girls	2020	14	19.10
Oceania	Girls	2021	14	19.37
Oceania	Girls	2022	14	19.64
Oceania	Girls	2023	14	19.91
Oceania	Girls	2024	14	20.19
Oceania	Girls	2025	14	20.46
Oceania	Girls	2026	14	20.73
Oceania	Girls	2027	14	21.00
Oceania	Girls	2028	14	21.27
Oceania	Girls	2029	14	21.54
Oceania	Girls	2030	14	21.81
Oceania	Girls	2017	15	20.31
Oceania	Girls	2018	15	20.60
Oceania	Girls	2019	15	20.90
Oceania	Girls	2020	15	21.20
Oceania	Girls	2021	15	21.51
Oceania	Girls	2022	15	21.82
Oceania	Girls	2023	15	22.14
Oceania	Girls	2024	15	22.47

Oceania	Girls	2025	15	22.80
Oceania	Girls	2026	15	23.13
Oceania	Girls	2027	15	23.47
Oceania	Girls	2028	15	23.81
Oceania	Girls	2029	15	24.15
Oceania	Girls	2030	15	24.50
Oceania	Girls	2017	16	21.86
Oceania	Girls	2018	16	22.16
Oceania	Girls	2019	16	22.47
Oceania	Girls	2020	16	22.79
Oceania	Girls	2021	16	23.11
Oceania	Girls	2022	16	23.44
Oceania	Girls	2023	16	23.78
Oceania	Girls	2024	16	24.12
Oceania	Girls	2025	16	24.46
Oceania	Girls	2026	16	24.81
Oceania	Girls	2027	16	25.17
Oceania	Girls	2028	16	25.53
Oceania	Girls	2029	16	25.89
Oceania	Girls	2030	16	26.26
Oceania	Girls	2017	17	23.16
Oceania	Girls	2018	17	23.48
Oceania	Girls	2019	17	23.81
Oceania	Girls	2020	17	24.14
Oceania	Girls	2021	17	24.47
Oceania	Girls	2022	17	24.81
Oceania	Girls	2023	17	25.16
Oceania	Girls	2024	17	25.52
Oceania	Girls	2025	17	25.87
Oceania	Girls	2026	17	26.24
Oceania	Girls	2027	17	26.61
Oceania	Girls	2028	17	26.98
Oceania	Girls	2029	17	27.36
Oceania	Girls	2030	17	27.74
Oceania	Girls	2017	18	24.58
Oceania	Girls	2018	18	24.91
Oceania	Girls	2019	18	25.25
Oceania	Girls	2020	18	25.59
Oceania	Girls	2021	18	25.94
Oceania	Girls	2022	18	26.30
Oceania	Girls	2023	18	26.65
Oceania	Girls	2024	18	27.01
Oceania	Girls	2025	18	27.38
Oceania	Girls	2026	18	27.74
Oceania	Girls	2027	18	28.12
Oceania	Girls	2028	18	28.49
Oceania	Girls	2029	18	28.87
Oceania	Girls	2030	18	29.25
South Asia	Boys	2017	12	5.70
South Asia	Boys	2018	12	5.85
South Asia	Boys	2019	12	6.01
South Asia	Boys	2020	12	6.17
South Asia	Boys	2021	12	6.33

South Asia	Boys	2022	12	6.49
South Asia	Boys	2023	12	6.64
South Asia	Boys	2024	12	6.80
South Asia	Boys	2025	12	6.96
South Asia	Boys	2026	12	7.12
South Asia	Boys	2027	12	7.28
South Asia	Boys	2028	12	7.43
South Asia	Boys	2029	12	7.59
South Asia	Boys	2030	12	7.75
South Asia	Boys	2017	13	6.85
South Asia	Boys	2018	13	7.03
South Asia	Boys	2019	13	7.21
South Asia	Boys	2020	13	7.39
South Asia	Boys	2021	13	7.57
South Asia	Boys	2022	13	7.75
South Asia	Boys	2023	13	7.93
South Asia	Boys	2024	13	8.11
South Asia	Boys	2025	13	8.30
South Asia	Boys	2026	13	8.48
South Asia	Boys	2027	13	8.66
South Asia	Boys	2028	13	8.84
South Asia	Boys	2029	13	9.02
South Asia	Boys	2030	13	9.20
South Asia	Boys	2017	14	8.18
South Asia	Boys	2018	14	8.37
South Asia	Boys	2019	14	8.57
South Asia	Boys	2020	14	8.77
South Asia	Boys	2021	14	8.96
South Asia	Boys	2022	14	9.16
South Asia	Boys	2023	14	9.36
South Asia	Boys	2024	14	9.56
South Asia	Boys	2025	14	9.75
South Asia	Boys	2026	14	9.95
South Asia	Boys	2027	14	10.15
South Asia	Boys	2028	14	10.34
South Asia	Boys	2029	14	10.54
South Asia	Boys	2030	14	10.74
South Asia	Boys	2017	15	9.64
South Asia	Boys	2018	15	9.84
South Asia	Boys	2019	15	10.05
South Asia	Boys	2020	15	10.25
South Asia	Boys	2021	15	10.45
South Asia	Boys	2022	15	10.66
South Asia	Boys	2023	15	10.86
South Asia	Boys	2024	15	11.06
South Asia	Boys	2025	15	11.27
South Asia	Boys	2026	15	11.47
South Asia	Boys	2027	15	11.67
South Asia	Boys	2028	15	11.87
South Asia	Boys	2029	15	12.08
South Asia	Boys	2030	15	12.28
South Asia	Boys	2017	16	11.22
South Asia	Boys	2018	16	11.43

South Asia	Boys	2019	16	11.63
South Asia	Boys	2020	16	11.84
South Asia	Boys	2021	16	12.05
South Asia	Boys	2022	16	12.26
South Asia	Boys	2023	16	12.46
South Asia	Boys	2024	16	12.67
South Asia	Boys	2025	16	12.88
South Asia	Boys	2026	16	13.08
South Asia	Boys	2027	16	13.29
South Asia	Boys	2028	16	13.50
South Asia	Boys	2029	16	13.71
South Asia	Boys	2030	16	13.91
South Asia	Boys	2017	17	12.88
South Asia	Boys	2018	17	13.09
South Asia	Boys	2019	17	13.31
South Asia	Boys	2020	17	13.52
South Asia	Boys	2021	17	13.73
South Asia	Boys	2022	17	13.95
South Asia	Boys	2023	17	14.16
South Asia	Boys	2024	17	14.37
South Asia	Boys	2025	17	14.58
South Asia	Boys	2026	17	14.80
South Asia	Boys	2027	17	15.01
South Asia	Boys	2028	17	15.22
South Asia	Boys	2029	17	15.44
South Asia	Boys	2030	17	15.65
South Asia	Boys	2017	18	14.61
South Asia	Boys	2018	18	14.84
South Asia	Boys	2019	18	15.06
South Asia	Boys	2020	18	15.29
South Asia	Boys	2021	18	15.52
South Asia	Boys	2022	18	15.74
South Asia	Boys	2023	18	15.97
South Asia	Boys	2024	18	16.19
South Asia	Boys	2025	18	16.42
South Asia	Boys	2026	18	16.65
South Asia	Boys	2027	18	16.87
South Asia	Boys	2028	18	17.10
South Asia	Boys	2029	18	17.32
South Asia	Boys	2030	18	17.55
South Asia	Girls	2017	12	5.31
South Asia	Girls	2018	12	5.41
South Asia	Girls	2019	12	5.51
South Asia	Girls	2020	12	5.60
South Asia	Girls	2021	12	5.70
South Asia	Girls	2022	12	5.80
South Asia	Girls	2023	12	5.90
South Asia	Girls	2024	12	6.00
South Asia	Girls	2025	12	6.10
South Asia	Girls	2026	12	6.20
South Asia	Girls	2027	12	6.30
South Asia	Girls	2028	12	6.40
South Asia	Girls	2029	12	6.50

South Asia	Girls	2030	12	6.59
South Asia	Girls	2017	13	6.56
South Asia	Girls	2018	13	6.67
South Asia	Girls	2019	13	6.79
South Asia	Girls	2020	13	6.90
South Asia	Girls	2021	13	7.01
South Asia	Girls	2022	13	7.12
South Asia	Girls	2023	13	7.23
South Asia	Girls	2024	13	7.35
South Asia	Girls	2025	13	7.46
South Asia	Girls	2026	13	7.57
South Asia	Girls	2027	13	7.68
South Asia	Girls	2028	13	7.79
South Asia	Girls	2029	13	7.91
South Asia	Girls	2030	13	8.02
South Asia	Girls	2017	14	7.93
South Asia	Girls	2018	14	8.05
South Asia	Girls	2019	14	8.17
South Asia	Girls	2020	14	8.29
South Asia	Girls	2021	14	8.41
South Asia	Girls	2022	14	8.53
South Asia	Girls	2023	14	8.66
South Asia	Girls	2024	14	8.78
South Asia	Girls	2025	14	8.90
South Asia	Girls	2026	14	9.02
South Asia	Girls	2027	14	9.14
South Asia	Girls	2028	14	9.26
South Asia	Girls	2029	14	9.38
South Asia	Girls	2030	14	9.50
South Asia	Girls	2017	15	9.23
South Asia	Girls	2018	15	9.37
South Asia	Girls	2019	15	9.51
South Asia	Girls	2020	15	9.66
South Asia	Girls	2021	15	9.82
South Asia	Girls	2022	15	9.98
South Asia	Girls	2023	15	10.14
South Asia	Girls	2024	15	10.31
South Asia	Girls	2025	15	10.49
South Asia	Girls	2026	15	10.67
South Asia	Girls	2027	15	10.86
South Asia	Girls	2028	15	11.05
South Asia	Girls	2029	15	11.24
South Asia	Girls	2030	15	11.44
South Asia	Girls	2017	16	10.30
South Asia	Girls	2018	16	10.44
South Asia	Girls	2019	16	10.59
South Asia	Girls	2020	16	10.74
South Asia	Girls	2021	16	10.90
South Asia	Girls	2022	16	11.07
South Asia	Girls	2023	16	11.24
South Asia	Girls	2024	16	11.41
South Asia	Girls	2025	16	11.59
South Asia	Girls	2026	16	11.77

South Asia	Girls	2027	16	11.97
South Asia	Girls	2028	16	12.16
South Asia	Girls	2029	16	12.36
South Asia	Girls	2030	16	12.56
South Asia	Girls	2017	17	11.08
South Asia	Girls	2018	17	11.23
South Asia	Girls	2019	17	11.38
South Asia	Girls	2020	17	11.54
South Asia	Girls	2021	17	11.71
South Asia	Girls	2022	17	11.88
South Asia	Girls	2023	17	12.06
South Asia	Girls	2024	17	12.24
South Asia	Girls	2025	17	12.43
South Asia	Girls	2026	17	12.62
South Asia	Girls	2027	17	12.82
South Asia	Girls	2028	17	13.02
South Asia	Girls	2029	17	13.23
South Asia	Girls	2030	17	13.45
South Asia	Girls	2017	18	11.60
South Asia	Girls	2018	18	11.76
South Asia	Girls	2019	18	11.93
South Asia	Girls	2020	18	12.11
South Asia	Girls	2021	18	12.29
South Asia	Girls	2022	18	12.47
South Asia	Girls	2023	18	12.67
South Asia	Girls	2024	18	12.86
South Asia	Girls	2025	18	13.07
South Asia	Girls	2026	18	13.28
South Asia	Girls	2027	18	13.49
South Asia	Girls	2028	18	13.71
South Asia	Girls	2029	18	13.94
South Asia	Girls	2030	18	14.17
Sub-Saharan Africa	Boys	2017	12	6.28
Sub-Saharan Africa	Boys	2018	12	6.41
Sub-Saharan Africa	Boys	2019	12	6.53
Sub-Saharan Africa	Boys	2020	12	6.65
Sub-Saharan Africa	Boys	2021	12	6.77
Sub-Saharan Africa	Boys	2022	12	6.90
Sub-Saharan Africa	Boys	2023	12	7.02
Sub-Saharan Africa	Boys	2024	12	7.14
Sub-Saharan Africa	Boys	2025	12	7.27
Sub-Saharan Africa	Boys	2026	12	7.39
Sub-Saharan Africa	Boys	2027	12	7.51
Sub-Saharan Africa	Boys	2028	12	7.64
Sub-Saharan Africa	Boys	2029	12	7.76
Sub-Saharan Africa	Boys	2030	12	7.88
Sub-Saharan Africa	Boys	2017	13	7.70
Sub-Saharan Africa	Boys	2018	13	7.85
Sub-Saharan Africa	Boys	2019	13	7.99
Sub-Saharan Africa	Boys	2020	13	8.13
Sub-Saharan Africa	Boys	2021	13	8.28
Sub-Saharan Africa	Boys	2022	13	8.42
Sub-Saharan Africa	Boys	2023	13	8.57

Sub-Saharan Africa	Boys	2024	13	8.71
Sub-Saharan Africa	Boys	2025	13	8.85
Sub-Saharan Africa	Boys	2026	13	9.00
Sub-Saharan Africa	Boys	2027	13	9.14
Sub-Saharan Africa	Boys	2028	13	9.29
Sub-Saharan Africa	Boys	2029	13	9.43
Sub-Saharan Africa	Boys	2030	13	9.57
Sub-Saharan Africa	Boys	2017	14	9.39
Sub-Saharan Africa	Boys	2018	14	9.56
Sub-Saharan Africa	Boys	2019	14	9.72
Sub-Saharan Africa	Boys	2020	14	9.88
Sub-Saharan Africa	Boys	2021	14	10.04
Sub-Saharan Africa	Boys	2022	14	10.20
Sub-Saharan Africa	Boys	2023	14	10.36
Sub-Saharan Africa	Boys	2024	14	10.52
Sub-Saharan Africa	Boys	2025	14	10.68
Sub-Saharan Africa	Boys	2026	14	10.84
Sub-Saharan Africa	Boys	2027	14	11.00
Sub-Saharan Africa	Boys	2028	14	11.16
Sub-Saharan Africa	Boys	2029	14	11.32
Sub-Saharan Africa	Boys	2030	14	11.48
Sub-Saharan Africa	Boys	2017	15	11.30
Sub-Saharan Africa	Boys	2018	15	11.47
Sub-Saharan Africa	Boys	2019	15	11.64
Sub-Saharan Africa	Boys	2020	15	11.81
Sub-Saharan Africa	Boys	2021	15	11.97
Sub-Saharan Africa	Boys	2022	15	12.14
Sub-Saharan Africa	Boys	2023	15	12.31
Sub-Saharan Africa	Boys	2024	15	12.47
Sub-Saharan Africa	Boys	2025	15	12.64
Sub-Saharan Africa	Boys	2026	15	12.81
Sub-Saharan Africa	Boys	2027	15	12.98
Sub-Saharan Africa	Boys	2028	15	13.14
Sub-Saharan Africa	Boys	2029	15	13.31
Sub-Saharan Africa	Boys	2030	15	13.48
Sub-Saharan Africa	Boys	2017	16	13.35
Sub-Saharan Africa	Boys	2018	16	13.52
Sub-Saharan Africa	Boys	2019	16	13.70
Sub-Saharan Africa	Boys	2020	16	13.87
Sub-Saharan Africa	Boys	2021	16	14.05
Sub-Saharan Africa	Boys	2022	16	14.22
Sub-Saharan Africa	Boys	2023	16	14.39
Sub-Saharan Africa	Boys	2024	16	14.57
Sub-Saharan Africa	Boys	2025	16	14.74
Sub-Saharan Africa	Boys	2026	16	14.92
Sub-Saharan Africa	Boys	2027	16	15.09
Sub-Saharan Africa	Boys	2028	16	15.26
Sub-Saharan Africa	Boys	2029	16	15.44
Sub-Saharan Africa	Boys	2030	16	15.61
Sub-Saharan Africa	Boys	2017	17	15.48
Sub-Saharan Africa	Boys	2018	17	15.66
Sub-Saharan Africa	Boys	2019	17	15.84
Sub-Saharan Africa	Boys	2020	17	16.02

Sub-Saharan Africa	Boys	2021	17	16.20
Sub-Saharan Africa	Boys	2022	17	16.38
Sub-Saharan Africa	Boys	2023	17	16.56
Sub-Saharan Africa	Boys	2024	17	16.74
Sub-Saharan Africa	Boys	2025	17	16.92
Sub-Saharan Africa	Boys	2026	17	17.10
Sub-Saharan Africa	Boys	2027	17	17.28
Sub-Saharan Africa	Boys	2028	17	17.46
Sub-Saharan Africa	Boys	2029	17	17.64
Sub-Saharan Africa	Boys	2030	17	17.82
Sub-Saharan Africa	Boys	2017	18	17.66
Sub-Saharan Africa	Boys	2018	18	17.84
Sub-Saharan Africa	Boys	2019	18	18.03
Sub-Saharan Africa	Boys	2020	18	18.22
Sub-Saharan Africa	Boys	2021	18	18.41
Sub-Saharan Africa	Boys	2022	18	18.60
Sub-Saharan Africa	Boys	2023	18	18.79
Sub-Saharan Africa	Boys	2024	18	18.98
Sub-Saharan Africa	Boys	2025	18	19.17
Sub-Saharan Africa	Boys	2026	18	19.36
Sub-Saharan Africa	Boys	2027	18	19.55
Sub-Saharan Africa	Boys	2028	18	19.74
Sub-Saharan Africa	Boys	2029	18	19.93
Sub-Saharan Africa	Boys	2030	18	20.12
Sub-Saharan Africa	Girls	2017	12	7.83
Sub-Saharan Africa	Girls	2018	12	7.96
Sub-Saharan Africa	Girls	2019	12	8.09
Sub-Saharan Africa	Girls	2020	12	8.21
Sub-Saharan Africa	Girls	2021	12	8.34
Sub-Saharan Africa	Girls	2022	12	8.47
Sub-Saharan Africa	Girls	2023	12	8.60
Sub-Saharan Africa	Girls	2024	12	8.73
Sub-Saharan Africa	Girls	2025	12	8.86
Sub-Saharan Africa	Girls	2026	12	8.98
Sub-Saharan Africa	Girls	2027	12	9.11
Sub-Saharan Africa	Girls	2028	12	9.24
Sub-Saharan Africa	Girls	2029	12	9.37
Sub-Saharan Africa	Girls	2030	12	9.50
Sub-Saharan Africa	Girls	2017	13	9.59
Sub-Saharan Africa	Girls	2018	13	9.74
Sub-Saharan Africa	Girls	2019	13	9.89
Sub-Saharan Africa	Girls	2020	13	10.04
Sub-Saharan Africa	Girls	2021	13	10.18
Sub-Saharan Africa	Girls	2022	13	10.33
Sub-Saharan Africa	Girls	2023	13	10.48
Sub-Saharan Africa	Girls	2024	13	10.63
Sub-Saharan Africa	Girls	2025	13	10.78
Sub-Saharan Africa	Girls	2026	13	10.92
Sub-Saharan Africa	Girls	2027	13	11.07
Sub-Saharan Africa	Girls	2028	13	11.22
Sub-Saharan Africa	Girls	2029	13	11.37
Sub-Saharan Africa	Girls	2030	13	11.51
Sub-Saharan Africa	Girls	2017	14	11.39

Sub-Saharan Africa	Girls	2018	14	11.56
Sub-Saharan Africa	Girls	2019	14	11.73
Sub-Saharan Africa	Girls	2020	14	11.91
Sub-Saharan Africa	Girls	2021	14	12.10
Sub-Saharan Africa	Girls	2022	14	12.29
Sub-Saharan Africa	Girls	2023	14	12.48
Sub-Saharan Africa	Girls	2024	14	12.68
Sub-Saharan Africa	Girls	2025	14	12.88
Sub-Saharan Africa	Girls	2026	14	13.08
Sub-Saharan Africa	Girls	2027	14	13.29
Sub-Saharan Africa	Girls	2028	14	13.50
Sub-Saharan Africa	Girls	2029	14	13.72
Sub-Saharan Africa	Girls	2030	14	13.94
Sub-Saharan Africa	Girls	2017	15	13.00
Sub-Saharan Africa	Girls	2018	15	13.18
Sub-Saharan Africa	Girls	2019	15	13.36
Sub-Saharan Africa	Girls	2020	15	13.55
Sub-Saharan Africa	Girls	2021	15	13.74
Sub-Saharan Africa	Girls	2022	15	13.93
Sub-Saharan Africa	Girls	2023	15	14.13
Sub-Saharan Africa	Girls	2024	15	14.33
Sub-Saharan Africa	Girls	2025	15	14.54
Sub-Saharan Africa	Girls	2026	15	14.75
Sub-Saharan Africa	Girls	2027	15	14.96
Sub-Saharan Africa	Girls	2028	15	15.18
Sub-Saharan Africa	Girls	2029	15	15.40
Sub-Saharan Africa	Girls	2030	15	15.62
Sub-Saharan Africa	Girls	2017	16	14.29
Sub-Saharan Africa	Girls	2018	16	14.47
Sub-Saharan Africa	Girls	2019	16	14.65
Sub-Saharan Africa	Girls	2020	16	14.85
Sub-Saharan Africa	Girls	2021	16	15.04
Sub-Saharan Africa	Girls	2022	16	15.24
Sub-Saharan Africa	Girls	2023	16	15.45
Sub-Saharan Africa	Girls	2024	16	15.66
Sub-Saharan Africa	Girls	2025	16	15.87
Sub-Saharan Africa	Girls	2026	16	16.09
Sub-Saharan Africa	Girls	2027	16	16.31
Sub-Saharan Africa	Girls	2028	16	16.54
Sub-Saharan Africa	Girls	2029	16	16.76
Sub-Saharan Africa	Girls	2030	16	16.99
Sub-Saharan Africa	Girls	2017	17	15.28
Sub-Saharan Africa	Girls	2018	17	15.47
Sub-Saharan Africa	Girls	2019	17	15.67
Sub-Saharan Africa	Girls	2020	17	15.87
Sub-Saharan Africa	Girls	2021	17	16.07
Sub-Saharan Africa	Girls	2022	17	16.28
Sub-Saharan Africa	Girls	2023	17	16.50
Sub-Saharan Africa	Girls	2024	17	16.72
Sub-Saharan Africa	Girls	2025	17	16.94
Sub-Saharan Africa	Girls	2026	17	17.17
Sub-Saharan Africa	Girls	2027	17	17.40
Sub-Saharan Africa	Girls	2028	17	17.63

Sub-Saharan Africa	Girls	2029	17	17.86
Sub-Saharan Africa	Girls	2030	17	18.10
Sub-Saharan Africa	Girls	2017	18	16.18
Sub-Saharan Africa	Girls	2018	18	16.38
Sub-Saharan Africa	Girls	2019	18	16.59
Sub-Saharan Africa	Girls	2020	18	16.80
Sub-Saharan Africa	Girls	2021	18	17.02
Sub-Saharan Africa	Girls	2022	18	17.25
Sub-Saharan Africa	Girls	2023	18	17.47
Sub-Saharan Africa	Girls	2024	18	17.71
Sub-Saharan Africa	Girls	2025	18	17.94
Sub-Saharan Africa	Girls	2026	18	18.19
Sub-Saharan Africa	Girls	2027	18	18.43
Sub-Saharan Africa	Girls	2028	18	18.68
Sub-Saharan Africa	Girls	2029	18	18.92
Sub-Saharan Africa	Girls	2030	18	19.17

region	sex	year	age	Predicted prevalence (%)
Central and Eastern Europe	Boys	2017	12	1.39
Central and Eastern Europe	Boys	2018	12	1.42
Central and Eastern Europe	Boys	2019	12	1.45
Central and Eastern Europe	Boys	2020	12	1.48
Central and Eastern Europe	Boys	2021	12	1.52
Central and Eastern Europe	Boys	2022	12	1.55
Central and Eastern Europe	Boys	2023	12	1.59
Central and Eastern Europe	Boys	2024	12	1.62
Central and Eastern Europe	Boys	2025	12	1.66
Central and Eastern Europe	Boys	2026	12	1.69
Central and Eastern Europe	Boys	2027	12	1.73
Central and Eastern Europe	Boys	2028	12	1.76
Central and Eastern Europe	Boys	2029	12	1.80
Central and Eastern Europe	Boys	2030	12	1.84
Central and Eastern Europe	Boys	2017	13	1.88
Central and Eastern Europe	Boys	2018	13	1.93
Central and Eastern Europe	Boys	2019	13	1.98
Central and Eastern Europe	Boys	2020	13	2.03
Central and Eastern Europe	Boys	2021	13	2.08
Central and Eastern Europe	Boys	2022	13	2.13
Central and Eastern Europe	Boys	2023	13	2.18
Central and Eastern Europe	Boys	2024	13	2.24
Central and Eastern Europe	Boys	2025	13	2.29
Central and Eastern Europe	Boys	2026	13	2.35
Central and Eastern Europe	Boys	2027	13	2.40
Central and Eastern Europe	Boys	2028	13	2.46
Central and Eastern Europe	Boys	2029	13	2.52
Central and Eastern Europe	Boys	2030	13	2.58
Central and Eastern Europe	Boys	2017	14	2.47
Central and Eastern Europe	Boys	2018	14	2.54
Central and Eastern Europe	Boys	2019	14	2.61
Central and Eastern Europe	Boys	2020	14	2.68
Central and Eastern Europe	Boys	2021	14	2.75
Central and Eastern Europe	Boys	2022	14	2.82
Central and Eastern Europe	Boys	2023	14	2.90
Central and Eastern Europe	Boys	2024	14	2.98
Central and Eastern Europe	Boys	2025	14	3.06
Central and Eastern Europe	Boys	2026	14	3.14
Central and Eastern Europe	Boys	2027	14	3.22
Central and Eastern Europe	Boys	2028	14	3.30
Central and Eastern Europe	Boys	2029	14	3.39
Central and Eastern Europe	Boys	2030	14	3.48
Central and Eastern Europe	Boys	2017	15	3.14
Central and Eastern Europe	Boys	2018	15	3.22
Central and Eastern Europe	Boys	2019	15	3.30
Central and Eastern Europe	Boys	2020	15	3.38
Central and Eastern Europe	Boys	2021	15	3.46
Central and Eastern Europe	Boys	2022	15	3.54
Central and Eastern Europe	Boys	2023	15	3.62
Central and Eastern Europe	Boys	2024	15	3.70
Central and Eastern Europe	Boys	2025	15	3.77
Central and Eastern Europe	Boys	2026	15	3.85
Central and Eastern Europe	Boys	2027	15	3.93

Central and Eastern Europe	Boys	2028	15	4.00
Central and Eastern Europe	Boys	2029	15	4.08
Central and Eastern Europe	Boys	2030	15	4.15
Central and Eastern Europe	Boys	2017	16	3.80
Central and Eastern Europe	Boys	2018	16	3.90
Central and Eastern Europe	Boys	2019	16	4.00
Central and Eastern Europe	Boys	2020	16	4.10
Central and Eastern Europe	Boys	2021	16	4.20
Central and Eastern Europe	Boys	2022	16	4.30
Central and Eastern Europe	Boys	2023	16	4.40
Central and Eastern Europe	Boys	2024	16	4.50
Central and Eastern Europe	Boys	2025	16	4.61
Central and Eastern Europe	Boys	2026	16	4.71
Central and Eastern Europe	Boys	2027	16	4.81
Central and Eastern Europe	Boys	2028	16	4.92
Central and Eastern Europe	Boys	2029	16	5.02
Central and Eastern Europe	Boys	2030	16	5.13
Central and Eastern Europe	Boys	2017	17	4.48
Central and Eastern Europe	Boys	2018	17	4.59
Central and Eastern Europe	Boys	2019	17	4.71
Central and Eastern Europe	Boys	2020	17	4.82
Central and Eastern Europe	Boys	2021	17	4.94
Central and Eastern Europe	Boys	2022	17	5.06
Central and Eastern Europe	Boys	2023	17	5.17
Central and Eastern Europe	Boys	2024	17	5.29
Central and Eastern Europe	Boys	2025	17	5.41
Central and Eastern Europe	Boys	2026	17	5.53
Central and Eastern Europe	Boys	2027	17	5.65
Central and Eastern Europe	Boys	2028	17	5.77
Central and Eastern Europe	Boys	2029	17	5.89
Central and Eastern Europe	Boys	2030	17	6.01
Central and Eastern Europe	Boys	2017	18	5.22
Central and Eastern Europe	Boys	2018	18	5.35
Central and Eastern Europe	Boys	2019	18	5.48
Central and Eastern Europe	Boys	2020	18	5.60
Central and Eastern Europe	Boys	2021	18	5.73
Central and Eastern Europe	Boys	2022	18	5.85
Central and Eastern Europe	Boys	2023	18	5.97
Central and Eastern Europe	Boys	2024	18	6.09
Central and Eastern Europe	Boys	2025	18	6.21
Central and Eastern Europe	Boys	2026	18	6.32
Central and Eastern Europe	Boys	2027	18	6.44
Central and Eastern Europe	Boys	2028	18	6.55
Central and Eastern Europe	Boys	2029	18	6.67
Central and Eastern Europe	Boys	2030	18	6.78
Central and Eastern Europe	Girls	2017	12	1.23
Central and Eastern Europe	Girls	2018	12	1.26
Central and Eastern Europe	Girls	2019	12	1.29
Central and Eastern Europe	Girls	2020	12	1.32
Central and Eastern Europe	Girls	2021	12	1.34
Central and Eastern Europe	Girls	2022	12	1.37
Central and Eastern Europe	Girls	2023	12	1.40
Central and Eastern Europe	Girls	2024	12	1.43
Central and Eastern Europe	Girls	2025	12	1.46

Central and Eastern Europe	Girls	2026	12	1.49
Central and Eastern Europe	Girls	2027	12	1.52
Central and Eastern Europe	Girls	2028	12	1.55
Central and Eastern Europe	Girls	2029	12	1.59
Central and Eastern Europe	Girls	2030	12	1.62
Central and Eastern Europe	Girls	2017	13	1.71
Central and Eastern Europe	Girls	2018	13	1.74
Central and Eastern Europe	Girls	2019	13	1.78
Central and Eastern Europe	Girls	2020	13	1.82
Central and Eastern Europe	Girls	2021	13	1.86
Central and Eastern Europe	Girls	2022	13	1.90
Central and Eastern Europe	Girls	2023	13	1.94
Central and Eastern Europe	Girls	2024	13	1.98
Central and Eastern Europe	Girls	2025	13	2.02
Central and Eastern Europe	Girls	2026	13	2.06
Central and Eastern Europe	Girls	2027	13	2.11
Central and Eastern Europe	Girls	2028	13	2.15
Central and Eastern Europe	Girls	2029	13	2.19
Central and Eastern Europe	Girls	2030	13	2.24
Central and Eastern Europe	Girls	2017	14	2.21
Central and Eastern Europe	Girls	2018	14	2.25
Central and Eastern Europe	Girls	2019	14	2.30
Central and Eastern Europe	Girls	2020	14	2.35
Central and Eastern Europe	Girls	2021	14	2.40
Central and Eastern Europe	Girls	2022	14	2.45
Central and Eastern Europe	Girls	2023	14	2.50
Central and Eastern Europe	Girls	2024	14	2.56
Central and Eastern Europe	Girls	2025	14	2.61
Central and Eastern Europe	Girls	2026	14	2.67
Central and Eastern Europe	Girls	2027	14	2.72
Central and Eastern Europe	Girls	2028	14	2.78
Central and Eastern Europe	Girls	2029	14	2.83
Central and Eastern Europe	Girls	2030	14	2.89
Central and Eastern Europe	Girls	2017	15	2.67
Central and Eastern Europe	Girls	2018	15	2.73
Central and Eastern Europe	Girls	2019	15	2.78
Central and Eastern Europe	Girls	2020	15	2.84
Central and Eastern Europe	Girls	2021	15	2.89
Central and Eastern Europe	Girls	2022	15	2.95
Central and Eastern Europe	Girls	2023	15	3.00
Central and Eastern Europe	Girls	2024	15	3.06
Central and Eastern Europe	Girls	2025	15	3.11
Central and Eastern Europe	Girls	2026	15	3.17
Central and Eastern Europe	Girls	2027	15	3.22
Central and Eastern Europe	Girls	2028	15	3.28
Central and Eastern Europe	Girls	2029	15	3.34
Central and Eastern Europe	Girls	2030	15	3.39
Central and Eastern Europe	Girls	2017	16	3.04
Central and Eastern Europe	Girls	2018	16	3.10
Central and Eastern Europe	Girls	2019	16	3.16
Central and Eastern Europe	Girls	2020	16	3.22
Central and Eastern Europe	Girls	2021	16	3.28
Central and Eastern Europe	Girls	2022	16	3.34
Central and Eastern Europe	Girls	2023	16	3.40

Central and Eastern Europe	Girls	2024	16	3.46
Central and Eastern Europe	Girls	2025	16	3.52
Central and Eastern Europe	Girls	2026	16	3.58
Central and Eastern Europe	Girls	2027	16	3.65
Central and Eastern Europe	Girls	2028	16	3.71
Central and Eastern Europe	Girls	2029	16	3.77
Central and Eastern Europe	Girls	2030	16	3.83
Central and Eastern Europe	Girls	2017	17	3.34
Central and Eastern Europe	Girls	2018	17	3.41
Central and Eastern Europe	Girls	2019	17	3.47
Central and Eastern Europe	Girls	2020	17	3.54
Central and Eastern Europe	Girls	2021	17	3.60
Central and Eastern Europe	Girls	2022	17	3.67
Central and Eastern Europe	Girls	2023	17	3.73
Central and Eastern Europe	Girls	2024	17	3.80
Central and Eastern Europe	Girls	2025	17	3.86
Central and Eastern Europe	Girls	2026	17	3.93
Central and Eastern Europe	Girls	2027	17	4.00
Central and Eastern Europe	Girls	2028	17	4.06
Central and Eastern Europe	Girls	2029	17	4.13
Central and Eastern Europe	Girls	2030	17	4.20
Central and Eastern Europe	Girls	2017	18	3.67
Central and Eastern Europe	Girls	2018	18	3.74
Central and Eastern Europe	Girls	2019	18	3.81
Central and Eastern Europe	Girls	2020	18	3.88
Central and Eastern Europe	Girls	2021	18	3.96
Central and Eastern Europe	Girls	2022	18	4.03
Central and Eastern Europe	Girls	2023	18	4.10
Central and Eastern Europe	Girls	2024	18	4.18
Central and Eastern Europe	Girls	2025	18	4.25
Central and Eastern Europe	Girls	2026	18	4.32
Central and Eastern Europe	Girls	2027	18	4.40
Central and Eastern Europe	Girls	2028	18	4.47
Central and Eastern Europe	Girls	2029	18	4.55
Central and Eastern Europe	Girls	2030	18	4.62
Central Asia and North Africa-Middle East	Boys	2017	12	1.48
Central Asia and North Africa-Middle East	Boys	2018	12	1.52
Central Asia and North Africa-Middle East	Boys	2019	12	1.55
Central Asia and North Africa-Middle East	Boys	2020	12	1.58
Central Asia and North Africa-Middle East	Boys	2021	12	1.61
Central Asia and North Africa-Middle East	Boys	2022	12	1.65
Central Asia and North Africa-Middle East	Boys	2023	12	1.68
Central Asia and North Africa-Middle East	Boys	2024	12	1.71
Central Asia and North Africa-Middle East	Boys	2025	12	1.75
Central Asia and North Africa-Middle East	Boys	2026	12	1.78
Central Asia and North Africa-Middle East	Boys	2027	12	1.81
Central Asia and North Africa-Middle East	Boys	2028	12	1.85
Central Asia and North Africa-Middle East	Boys	2029	12	1.88
Central Asia and North Africa-Middle East	Boys	2030	12	1.91
Central Asia and North Africa-Middle East	Boys	2017	13	2.04
Central Asia and North Africa-Middle East	Boys	2018	13	2.09
Central Asia and North Africa-Middle East	Boys	2019	13	2.14
Central Asia and North Africa-Middle East	Boys	2020	13	2.19
Central Asia and North Africa-Middle East	Boys	2021	13	2.24

Central Asia and North Africa-Middle East	Boys	2022	13	2.29
Central Asia and North Africa-Middle East	Boys	2023	13	2.34
Central Asia and North Africa-Middle East	Boys	2024	13	2.39
Central Asia and North Africa-Middle East	Boys	2025	13	2.44
Central Asia and North Africa-Middle East	Boys	2026	13	2.49
Central Asia and North Africa-Middle East	Boys	2027	13	2.54
Central Asia and North Africa-Middle East	Boys	2028	13	2.59
Central Asia and North Africa-Middle East	Boys	2029	13	2.64
Central Asia and North Africa-Middle East	Boys	2030	13	2.70
Central Asia and North Africa-Middle East	Boys	2017	14	2.71
Central Asia and North Africa-Middle East	Boys	2018	14	2.78
Central Asia and North Africa-Middle East	Boys	2019	14	2.85
Central Asia and North Africa-Middle East	Boys	2020	14	2.92
Central Asia and North Africa-Middle East	Boys	2021	14	2.99
Central Asia and North Africa-Middle East	Boys	2022	14	3.06
Central Asia and North Africa-Middle East	Boys	2023	14	3.13
Central Asia and North Africa-Middle East	Boys	2024	14	3.20
Central Asia and North Africa-Middle East	Boys	2025	14	3.28
Central Asia and North Africa-Middle East	Boys	2026	14	3.35
Central Asia and North Africa-Middle East	Boys	2027	14	3.43
Central Asia and North Africa-Middle East	Boys	2028	14	3.51
Central Asia and North Africa-Middle East	Boys	2029	14	3.58
Central Asia and North Africa-Middle East	Boys	2030	14	3.66
Central Asia and North Africa-Middle East	Boys	2017	15	3.40
Central Asia and North Africa-Middle East	Boys	2018	15	3.50
Central Asia and North Africa-Middle East	Boys	2019	15	3.59
Central Asia and North Africa-Middle East	Boys	2020	15	3.69
Central Asia and North Africa-Middle East	Boys	2021	15	3.79
Central Asia and North Africa-Middle East	Boys	2022	15	3.88
Central Asia and North Africa-Middle East	Boys	2023	15	3.99
Central Asia and North Africa-Middle East	Boys	2024	15	4.09
Central Asia and North Africa-Middle East	Boys	2025	15	4.19
Central Asia and North Africa-Middle East	Boys	2026	15	4.30
Central Asia and North Africa-Middle East	Boys	2027	15	4.41
Central Asia and North Africa-Middle East	Boys	2028	15	4.51
Central Asia and North Africa-Middle East	Boys	2029	15	4.62
Central Asia and North Africa-Middle East	Boys	2030	15	4.73
Central Asia and North Africa-Middle East	Boys	2017	16	4.14
Central Asia and North Africa-Middle East	Boys	2018	16	4.25
Central Asia and North Africa-Middle East	Boys	2019	16	4.37
Central Asia and North Africa-Middle East	Boys	2020	16	4.49
Central Asia and North Africa-Middle East	Boys	2021	16	4.61
Central Asia and North Africa-Middle East	Boys	2022	16	4.74
Central Asia and North Africa-Middle East	Boys	2023	16	4.86
Central Asia and North Africa-Middle East	Boys	2024	16	4.99
Central Asia and North Africa-Middle East	Boys	2025	16	5.12
Central Asia and North Africa-Middle East	Boys	2026	16	5.26
Central Asia and North Africa-Middle East	Boys	2027	16	5.39
Central Asia and North Africa-Middle East	Boys	2028	16	5.53
Central Asia and North Africa-Middle East	Boys	2029	16	5.67
Central Asia and North Africa-Middle East	Boys	2030	16	5.81
Central Asia and North Africa-Middle East	Boys	2017	17	4.91
Central Asia and North Africa-Middle East	Boys	2018	17	5.05
Central Asia and North Africa-Middle East	Boys	2019	17	5.18

Central Asia and North Africa-Middle East	Boys	2020	17	5.33
Central Asia and North Africa-Middle East	Boys	2021	17	5.47
Central Asia and North Africa-Middle East	Boys	2022	17	5.62
Central Asia and North Africa-Middle East	Boys	2023	17	5.77
Central Asia and North Africa-Middle East	Boys	2024	17	5.92
Central Asia and North Africa-Middle East	Boys	2025	17	6.08
Central Asia and North Africa-Middle East	Boys	2026	17	6.24
Central Asia and North Africa-Middle East	Boys	2027	17	6.40
Central Asia and North Africa-Middle East	Boys	2028	17	6.57
Central Asia and North Africa-Middle East	Boys	2029	17	6.73
Central Asia and North Africa-Middle East	Boys	2030	17	6.90
Central Asia and North Africa-Middle East	Boys	2017	18	5.78
Central Asia and North Africa-Middle East	Boys	2018	18	5.94
Central Asia and North Africa-Middle East	Boys	2019	18	6.10
Central Asia and North Africa-Middle East	Boys	2020	18	6.26
Central Asia and North Africa-Middle East	Boys	2021	18	6.43
Central Asia and North Africa-Middle East	Boys	2022	18	6.60
Central Asia and North Africa-Middle East	Boys	2023	18	6.77
Central Asia and North Africa-Middle East	Boys	2024	18	6.95
Central Asia and North Africa-Middle East	Boys	2025	18	7.13
Central Asia and North Africa-Middle East	Boys	2026	18	7.32
Central Asia and North Africa-Middle East	Boys	2027	18	7.50
Central Asia and North Africa-Middle East	Boys	2028	18	7.69
Central Asia and North Africa-Middle East	Boys	2029	18	7.89
Central Asia and North Africa-Middle East	Boys	2030	18	8.08
Central Asia and North Africa-Middle East	Girls	2017	12	1.83
Central Asia and North Africa-Middle East	Girls	2018	12	1.86
Central Asia and North Africa-Middle East	Girls	2019	12	1.90
Central Asia and North Africa-Middle East	Girls	2020	12	1.94
Central Asia and North Africa-Middle East	Girls	2021	12	1.97
Central Asia and North Africa-Middle East	Girls	2022	12	2.01
Central Asia and North Africa-Middle East	Girls	2023	12	2.04
Central Asia and North Africa-Middle East	Girls	2024	12	2.08
Central Asia and North Africa-Middle East	Girls	2025	12	2.12
Central Asia and North Africa-Middle East	Girls	2026	12	2.15
Central Asia and North Africa-Middle East	Girls	2027	12	2.19
Central Asia and North Africa-Middle East	Girls	2028	12	2.23
Central Asia and North Africa-Middle East	Girls	2029	12	2.26
Central Asia and North Africa-Middle East	Girls	2030	12	2.30
Central Asia and North Africa-Middle East	Girls	2017	13	2.56
Central Asia and North Africa-Middle East	Girls	2018	13	2.61
Central Asia and North Africa-Middle East	Girls	2019	13	2.66
Central Asia and North Africa-Middle East	Girls	2020	13	2.71
Central Asia and North Africa-Middle East	Girls	2021	13	2.77
Central Asia and North Africa-Middle East	Girls	2022	13	2.82
Central Asia and North Africa-Middle East	Girls	2023	13	2.87
Central Asia and North Africa-Middle East	Girls	2024	13	2.93
Central Asia and North Africa-Middle East	Girls	2025	13	2.98
Central Asia and North Africa-Middle East	Girls	2026	13	3.03
Central Asia and North Africa-Middle East	Girls	2027	13	3.09
Central Asia and North Africa-Middle East	Girls	2028	13	3.14
Central Asia and North Africa-Middle East	Girls	2029	13	3.19
Central Asia and North Africa-Middle East	Girls	2030	13	3.25
Central Asia and North Africa-Middle East	Girls	2017	14	3.32

Central Asia and North Africa-Middle East	Girls	2018	14	3.39
Central Asia and North Africa-Middle East	Girls	2019	14	3.45
Central Asia and North Africa-Middle East	Girls	2020	14	3.52
Central Asia and North Africa-Middle East	Girls	2021	14	3.59
Central Asia and North Africa-Middle East	Girls	2022	14	3.66
Central Asia and North Africa-Middle East	Girls	2023	14	3.73
Central Asia and North Africa-Middle East	Girls	2024	14	3.80
Central Asia and North Africa-Middle East	Girls	2025	14	3.87
Central Asia and North Africa-Middle East	Girls	2026	14	3.94
Central Asia and North Africa-Middle East	Girls	2027	14	4.01
Central Asia and North Africa-Middle East	Girls	2028	14	4.08
Central Asia and North Africa-Middle East	Girls	2029	14	4.16
Central Asia and North Africa-Middle East	Girls	2030	14	4.23
Central Asia and North Africa-Middle East	Girls	2017	15	3.97
Central Asia and North Africa-Middle East	Girls	2018	15	4.06
Central Asia and North Africa-Middle East	Girls	2019	15	4.14
Central Asia and North Africa-Middle East	Girls	2020	15	4.22
Central Asia and North Africa-Middle East	Girls	2021	15	4.31
Central Asia and North Africa-Middle East	Girls	2022	15	4.39
Central Asia and North Africa-Middle East	Girls	2023	15	4.47
Central Asia and North Africa-Middle East	Girls	2024	15	4.56
Central Asia and North Africa-Middle East	Girls	2025	15	4.64
Central Asia and North Africa-Middle East	Girls	2026	15	4.72
Central Asia and North Africa-Middle East	Girls	2027	15	4.80
Central Asia and North Africa-Middle East	Girls	2028	15	4.89
Central Asia and North Africa-Middle East	Girls	2029	15	4.97
Central Asia and North Africa-Middle East	Girls	2030	15	5.05
Central Asia and North Africa-Middle East	Girls	2017	16	4.52
Central Asia and North Africa-Middle East	Girls	2018	16	4.61
Central Asia and North Africa-Middle East	Girls	2019	16	4.71
Central Asia and North Africa-Middle East	Girls	2020	16	4.80
Central Asia and North Africa-Middle East	Girls	2021	16	4.89
Central Asia and North Africa-Middle East	Girls	2022	16	4.99
Central Asia and North Africa-Middle East	Girls	2023	16	5.08
Central Asia and North Africa-Middle East	Girls	2024	16	5.17
Central Asia and North Africa-Middle East	Girls	2025	16	5.27
Central Asia and North Africa-Middle East	Girls	2026	16	5.36
Central Asia and North Africa-Middle East	Girls	2027	16	5.45
Central Asia and North Africa-Middle East	Girls	2028	16	5.54
Central Asia and North Africa-Middle East	Girls	2029	16	5.63
Central Asia and North Africa-Middle East	Girls	2030	16	5.72
Central Asia and North Africa-Middle East	Girls	2017	17	5.02
Central Asia and North Africa-Middle East	Girls	2018	17	5.12
Central Asia and North Africa-Middle East	Girls	2019	17	5.22
Central Asia and North Africa-Middle East	Girls	2020	17	5.33
Central Asia and North Africa-Middle East	Girls	2021	17	5.43
Central Asia and North Africa-Middle East	Girls	2022	17	5.53
Central Asia and North Africa-Middle East	Girls	2023	17	5.63
Central Asia and North Africa-Middle East	Girls	2024	17	5.73
Central Asia and North Africa-Middle East	Girls	2025	17	5.83
Central Asia and North Africa-Middle East	Girls	2026	17	5.93
Central Asia and North Africa-Middle East	Girls	2027	17	6.03
Central Asia and North Africa-Middle East	Girls	2028	17	6.13
Central Asia and North Africa-Middle East	Girls	2029	17	6.23

Central Asia and North Africa-Middle East	Girls	2030	17	6.33
Central Asia and North Africa-Middle East	Girls	2017	18	5.60
Central Asia and North Africa-Middle East	Girls	2018	18	5.71
Central Asia and North Africa-Middle East	Girls	2019	18	5.83
Central Asia and North Africa-Middle East	Girls	2020	18	5.94
Central Asia and North Africa-Middle East	Girls	2021	18	6.05
Central Asia and North Africa-Middle East	Girls	2022	18	6.16
Central Asia and North Africa-Middle East	Girls	2023	18	6.27
Central Asia and North Africa-Middle East	Girls	2024	18	6.37
Central Asia and North Africa-Middle East	Girls	2025	18	6.48
Central Asia and North Africa-Middle East	Girls	2026	18	6.59
Central Asia and North Africa-Middle East	Girls	2027	18	6.70
Central Asia and North Africa-Middle East	Girls	2028	18	6.81
Central Asia and North Africa-Middle East	Girls	2029	18	6.92
Central Asia and North Africa-Middle East	Girls	2030	18	7.03
East and South East Asia	Boys	2017	12	1.41
East and South East Asia	Boys	2018	12	1.44
East and South East Asia	Boys	2019	12	1.48
East and South East Asia	Boys	2020	12	1.52
East and South East Asia	Boys	2021	12	1.55
East and South East Asia	Boys	2022	12	1.58
East and South East Asia	Boys	2023	12	1.61
East and South East Asia	Boys	2024	12	1.63
East and South East Asia	Boys	2025	12	1.65
East and South East Asia	Boys	2026	12	1.67
East and South East Asia	Boys	2027	12	1.68
East and South East Asia	Boys	2028	12	1.70
East and South East Asia	Boys	2029	12	1.71
East and South East Asia	Boys	2030	12	1.72
East and South East Asia	Boys	2017	13	1.93
East and South East Asia	Boys	2018	13	1.98
East and South East Asia	Boys	2019	13	2.04
East and South East Asia	Boys	2020	13	2.10
East and South East Asia	Boys	2021	13	2.16
East and South East Asia	Boys	2022	13	2.21
East and South East Asia	Boys	2023	13	2.27
East and South East Asia	Boys	2024	13	2.33
East and South East Asia	Boys	2025	13	2.38
East and South East Asia	Boys	2026	13	2.44
East and South East Asia	Boys	2027	13	2.50
East and South East Asia	Boys	2028	13	2.55
East and South East Asia	Boys	2029	13	2.61
East and South East Asia	Boys	2030	13	2.66
East and South East Asia	Boys	2017	14	2.55
East and South East Asia	Boys	2018	14	2.63
East and South East Asia	Boys	2019	14	2.71
East and South East Asia	Boys	2020	14	2.79
East and South East Asia	Boys	2021	14	2.87
East and South East Asia	Boys	2022	14	2.96
East and South East Asia	Boys	2023	14	3.04
East and South East Asia	Boys	2024	14	3.12
East and South East Asia	Boys	2025	14	3.20
East and South East Asia	Boys	2026	14	3.28
East and South East Asia	Boys	2027	14	3.35

East and South East Asia	Boys	2028	14	3.43
East and South East Asia	Boys	2029	14	3.51
East and South East Asia	Boys	2030	14	3.59
East and South East Asia	Boys	2017	15	3.23
East and South East Asia	Boys	2018	15	3.32
East and South East Asia	Boys	2019	15	3.41
East and South East Asia	Boys	2020	15	3.50
East and South East Asia	Boys	2021	15	3.58
East and South East Asia	Boys	2022	15	3.67
East and South East Asia	Boys	2023	15	3.76
East and South East Asia	Boys	2024	15	3.84
East and South East Asia	Boys	2025	15	3.91
East and South East Asia	Boys	2026	15	3.99
East and South East Asia	Boys	2027	15	4.07
East and South East Asia	Boys	2028	15	4.14
East and South East Asia	Boys	2029	15	4.22
East and South East Asia	Boys	2030	15	4.30
East and South East Asia	Boys	2017	16	3.91
East and South East Asia	Boys	2018	16	4.02
East and South East Asia	Boys	2019	16	4.13
East and South East Asia	Boys	2020	16	4.24
East and South East Asia	Boys	2021	16	4.35
East and South East Asia	Boys	2022	16	4.45
East and South East Asia	Boys	2023	16	4.56
East and South East Asia	Boys	2024	16	4.65
East and South East Asia	Boys	2025	16	4.75
East and South East Asia	Boys	2026	16	4.84
East and South East Asia	Boys	2027	16	4.94
East and South East Asia	Boys	2028	16	5.03
East and South East Asia	Boys	2029	16	5.12
East and South East Asia	Boys	2030	16	5.22
East and South East Asia	Boys	2017	17	4.62
East and South East Asia	Boys	2018	17	4.74
East and South East Asia	Boys	2019	17	4.87
East and South East Asia	Boys	2020	17	5.00
East and South East Asia	Boys	2021	17	5.13
East and South East Asia	Boys	2022	17	5.25
East and South East Asia	Boys	2023	17	5.37
East and South East Asia	Boys	2024	17	5.49
East and South East Asia	Boys	2025	17	5.60
East and South East Asia	Boys	2026	17	5.71
East and South East Asia	Boys	2027	17	5.82
East and South East Asia	Boys	2028	17	5.93
East and South East Asia	Boys	2029	17	6.04
East and South East Asia	Boys	2030	17	6.15
East and South East Asia	Boys	2017	18	5.41
East and South East Asia	Boys	2018	18	5.55
East and South East Asia	Boys	2019	18	5.69
East and South East Asia	Boys	2020	18	5.84
East and South East Asia	Boys	2021	18	5.99
East and South East Asia	Boys	2022	18	6.13
East and South East Asia	Boys	2023	18	6.27
East and South East Asia	Boys	2024	18	6.40
East and South East Asia	Boys	2025	18	6.53

East and South East Asia	Boys	2026	18	6.66
East and South East Asia	Boys	2027	18	6.78
East and South East Asia	Boys	2028	18	6.90
East and South East Asia	Boys	2029	18	7.03
East and South East Asia	Boys	2030	18	7.15
East and South East Asia	Girls	2017	12	1.15
East and South East Asia	Girls	2018	12	1.18
East and South East Asia	Girls	2019	12	1.20
East and South East Asia	Girls	2020	12	1.23
East and South East Asia	Girls	2021	12	1.26
East and South East Asia	Girls	2022	12	1.29
East and South East Asia	Girls	2023	12	1.32
East and South East Asia	Girls	2024	12	1.35
East and South East Asia	Girls	2025	12	1.37
East and South East Asia	Girls	2026	12	1.40
East and South East Asia	Girls	2027	12	1.43
East and South East Asia	Girls	2028	12	1.45
East and South East Asia	Girls	2029	12	1.48
East and South East Asia	Girls	2030	12	1.51
East and South East Asia	Girls	2017	13	1.60
East and South East Asia	Girls	2018	13	1.64
East and South East Asia	Girls	2019	13	1.68
East and South East Asia	Girls	2020	13	1.72
East and South East Asia	Girls	2021	13	1.76
East and South East Asia	Girls	2022	13	1.80
East and South East Asia	Girls	2023	13	1.84
East and South East Asia	Girls	2024	13	1.88
East and South East Asia	Girls	2025	13	1.91
East and South East Asia	Girls	2026	13	1.95
East and South East Asia	Girls	2027	13	1.99
East and South East Asia	Girls	2028	13	2.02
East and South East Asia	Girls	2029	13	2.06
East and South East Asia	Girls	2030	13	2.10
East and South East Asia	Girls	2017	14	2.08
East and South East Asia	Girls	2018	14	2.13
East and South East Asia	Girls	2019	14	2.19
East and South East Asia	Girls	2020	14	2.24
East and South East Asia	Girls	2021	14	2.29
East and South East Asia	Girls	2022	14	2.34
East and South East Asia	Girls	2023	14	2.39
East and South East Asia	Girls	2024	14	2.44
East and South East Asia	Girls	2025	14	2.48
East and South East Asia	Girls	2026	14	2.53
East and South East Asia	Girls	2027	14	2.58
East and South East Asia	Girls	2028	14	2.62
East and South East Asia	Girls	2029	14	2.67
East and South East Asia	Girls	2030	14	2.71
East and South East Asia	Girls	2017	15	2.54
East and South East Asia	Girls	2018	15	2.59
East and South East Asia	Girls	2019	15	2.65
East and South East Asia	Girls	2020	15	2.71
East and South East Asia	Girls	2021	15	2.76
East and South East Asia	Girls	2022	15	2.82
East and South East Asia	Girls	2023	15	2.88

East and South East Asia	Girls	2024	15	2.93
East and South East Asia	Girls	2025	15	2.98
East and South East Asia	Girls	2026	15	3.04
East and South East Asia	Girls	2027	15	3.09
East and South East Asia	Girls	2028	15	3.14
East and South East Asia	Girls	2029	15	3.20
East and South East Asia	Girls	2030	15	3.25
East and South East Asia	Girls	2017	16	2.91
East and South East Asia	Girls	2018	16	2.97
East and South East Asia	Girls	2019	16	3.03
East and South East Asia	Girls	2020	16	3.09
East and South East Asia	Girls	2021	16	3.16
East and South East Asia	Girls	2022	16	3.22
East and South East Asia	Girls	2023	16	3.28
East and South East Asia	Girls	2024	16	3.34
East and South East Asia	Girls	2025	16	3.40
East and South East Asia	Girls	2026	16	3.46
East and South East Asia	Girls	2027	16	3.52
East and South East Asia	Girls	2028	16	3.58
East and South East Asia	Girls	2029	16	3.64
East and South East Asia	Girls	2030	16	3.70
East and South East Asia	Girls	2017	17	3.23
East and South East Asia	Girls	2018	17	3.30
East and South East Asia	Girls	2019	17	3.37
East and South East Asia	Girls	2020	17	3.44
East and South East Asia	Girls	2021	17	3.51
East and South East Asia	Girls	2022	17	3.58
East and South East Asia	Girls	2023	17	3.64
East and South East Asia	Girls	2024	17	3.71
East and South East Asia	Girls	2025	17	3.77
East and South East Asia	Girls	2026	17	3.84
East and South East Asia	Girls	2027	17	3.90
East and South East Asia	Girls	2028	17	3.97
East and South East Asia	Girls	2029	17	4.03
East and South East Asia	Girls	2030	17	4.10
East and South East Asia	Girls	2017	18	3.60
East and South East Asia	Girls	2018	18	3.67
East and South East Asia	Girls	2019	18	3.75
East and South East Asia	Girls	2020	18	3.82
East and South East Asia	Girls	2021	18	3.90
East and South East Asia	Girls	2022	18	3.98
East and South East Asia	Girls	2023	18	4.05
East and South East Asia	Girls	2024	18	4.13
East and South East Asia	Girls	2025	18	4.20
East and South East Asia	Girls	2026	18	4.27
East and South East Asia	Girls	2027	18	4.34
East and South East Asia	Girls	2028	18	4.41
East and South East Asia	Girls	2029	18	4.48
East and South East Asia	Girls	2030	18	4.56
High-income Asia Pacific	Boys	2017	12	1.21
High-income Asia Pacific	Boys	2018	12	1.21
High-income Asia Pacific	Boys	2019	12	1.21
High-income Asia Pacific	Boys	2020	12	1.22
High-income Asia Pacific	Boys	2021	12	1.22

High-income Asia Pacific	Boys	2022	12	1.22
High-income Asia Pacific	Boys	2023	12	1.23
High-income Asia Pacific	Boys	2024	12	1.23
High-income Asia Pacific	Boys	2025	12	1.23
High-income Asia Pacific	Boys	2026	12	1.24
High-income Asia Pacific	Boys	2027	12	1.24
High-income Asia Pacific	Boys	2028	12	1.24
High-income Asia Pacific	Boys	2029	12	1.25
High-income Asia Pacific	Boys	2030	12	1.25
High-income Asia Pacific	Boys	2017	13	1.63
High-income Asia Pacific	Boys	2018	13	1.63
High-income Asia Pacific	Boys	2019	13	1.64
High-income Asia Pacific	Boys	2020	13	1.64
High-income Asia Pacific	Boys	2021	13	1.65
High-income Asia Pacific	Boys	2022	13	1.65
High-income Asia Pacific	Boys	2023	13	1.66
High-income Asia Pacific	Boys	2024	13	1.66
High-income Asia Pacific	Boys	2025	13	1.67
High-income Asia Pacific	Boys	2026	13	1.67
High-income Asia Pacific	Boys	2027	13	1.68
High-income Asia Pacific	Boys	2028	13	1.68
High-income Asia Pacific	Boys	2029	13	1.69
High-income Asia Pacific	Boys	2030	13	1.69
High-income Asia Pacific	Boys	2017	14	2.13
High-income Asia Pacific	Boys	2018	14	2.13
High-income Asia Pacific	Boys	2019	14	2.14
High-income Asia Pacific	Boys	2020	14	2.15
High-income Asia Pacific	Boys	2021	14	2.16
High-income Asia Pacific	Boys	2022	14	2.17
High-income Asia Pacific	Boys	2023	14	2.17
High-income Asia Pacific	Boys	2024	14	2.18
High-income Asia Pacific	Boys	2025	14	2.19
High-income Asia Pacific	Boys	2026	14	2.19
High-income Asia Pacific	Boys	2027	14	2.20
High-income Asia Pacific	Boys	2028	14	2.21
High-income Asia Pacific	Boys	2029	14	2.22
High-income Asia Pacific	Boys	2030	14	2.22
High-income Asia Pacific	Boys	2017	15	2.72
High-income Asia Pacific	Boys	2018	15	2.72
High-income Asia Pacific	Boys	2019	15	2.72
High-income Asia Pacific	Boys	2020	15	2.71
High-income Asia Pacific	Boys	2021	15	2.70
High-income Asia Pacific	Boys	2022	15	2.69
High-income Asia Pacific	Boys	2023	15	2.68
High-income Asia Pacific	Boys	2024	15	2.67
High-income Asia Pacific	Boys	2025	15	2.65
High-income Asia Pacific	Boys	2026	15	2.63
High-income Asia Pacific	Boys	2027	15	2.61
High-income Asia Pacific	Boys	2028	15	2.59
High-income Asia Pacific	Boys	2029	15	2.57
High-income Asia Pacific	Boys	2030	15	2.55
High-income Asia Pacific	Boys	2017	16	3.28
High-income Asia Pacific	Boys	2018	16	3.28
High-income Asia Pacific	Boys	2019	16	3.27

High-income Asia Pacific	Boys	2020	16	3.27
High-income Asia Pacific	Boys	2021	16	3.26
High-income Asia Pacific	Boys	2022	16	3.25
High-income Asia Pacific	Boys	2023	16	3.23
High-income Asia Pacific	Boys	2024	16	3.22
High-income Asia Pacific	Boys	2025	16	3.20
High-income Asia Pacific	Boys	2026	16	3.18
High-income Asia Pacific	Boys	2027	16	3.16
High-income Asia Pacific	Boys	2028	16	3.13
High-income Asia Pacific	Boys	2029	16	3.11
High-income Asia Pacific	Boys	2030	16	3.09
High-income Asia Pacific	Boys	2017	17	3.84
High-income Asia Pacific	Boys	2018	17	3.84
High-income Asia Pacific	Boys	2019	17	3.84
High-income Asia Pacific	Boys	2020	17	3.83
High-income Asia Pacific	Boys	2021	17	3.82
High-income Asia Pacific	Boys	2022	17	3.81
High-income Asia Pacific	Boys	2023	17	3.79
High-income Asia Pacific	Boys	2024	17	3.77
High-income Asia Pacific	Boys	2025	17	3.75
High-income Asia Pacific	Boys	2026	17	3.73
High-income Asia Pacific	Boys	2027	17	3.71
High-income Asia Pacific	Boys	2028	17	3.68
High-income Asia Pacific	Boys	2029	17	3.66
High-income Asia Pacific	Boys	2030	17	3.63
High-income Asia Pacific	Boys	2017	18	4.44
High-income Asia Pacific	Boys	2018	18	4.44
High-income Asia Pacific	Boys	2019	18	4.44
High-income Asia Pacific	Boys	2020	18	4.43
High-income Asia Pacific	Boys	2021	18	4.42
High-income Asia Pacific	Boys	2022	18	4.40
High-income Asia Pacific	Boys	2023	18	4.38
High-income Asia Pacific	Boys	2024	18	4.36
High-income Asia Pacific	Boys	2025	18	4.34
High-income Asia Pacific	Boys	2026	18	4.31
High-income Asia Pacific	Boys	2027	18	4.29
High-income Asia Pacific	Boys	2028	18	4.26
High-income Asia Pacific	Boys	2029	18	4.23
High-income Asia Pacific	Boys	2030	18	4.20
High-income Asia Pacific	Girls	2017	12	1.01
High-income Asia Pacific	Girls	2018	12	1.01
High-income Asia Pacific	Girls	2019	12	1.01
High-income Asia Pacific	Girls	2020	12	1.02
High-income Asia Pacific	Girls	2021	12	1.02
High-income Asia Pacific	Girls	2022	12	1.03
High-income Asia Pacific	Girls	2023	12	1.03
High-income Asia Pacific	Girls	2024	12	1.03
High-income Asia Pacific	Girls	2025	12	1.04
High-income Asia Pacific	Girls	2026	12	1.04
High-income Asia Pacific	Girls	2027	12	1.04
High-income Asia Pacific	Girls	2028	12	1.05
High-income Asia Pacific	Girls	2029	12	1.05
High-income Asia Pacific	Girls	2030	12	1.06
High-income Asia Pacific	Girls	2017	13	1.38

High-income Asia Pacific	Girls	2018	13	1.39
High-income Asia Pacific	Girls	2019	13	1.39
High-income Asia Pacific	Girls	2020	13	1.40
High-income Asia Pacific	Girls	2021	13	1.40
High-income Asia Pacific	Girls	2022	13	1.41
High-income Asia Pacific	Girls	2023	13	1.41
High-income Asia Pacific	Girls	2024	13	1.42
High-income Asia Pacific	Girls	2025	13	1.42
High-income Asia Pacific	Girls	2026	13	1.43
High-income Asia Pacific	Girls	2027	13	1.43
High-income Asia Pacific	Girls	2028	13	1.44
High-income Asia Pacific	Girls	2029	13	1.44
High-income Asia Pacific	Girls	2030	13	1.45
High-income Asia Pacific	Girls	2017	14	1.78
High-income Asia Pacific	Girls	2018	14	1.78
High-income Asia Pacific	Girls	2019	14	1.79
High-income Asia Pacific	Girls	2020	14	1.80
High-income Asia Pacific	Girls	2021	14	1.80
High-income Asia Pacific	Girls	2022	14	1.81
High-income Asia Pacific	Girls	2023	14	1.82
High-income Asia Pacific	Girls	2024	14	1.82
High-income Asia Pacific	Girls	2025	14	1.83
High-income Asia Pacific	Girls	2026	14	1.84
High-income Asia Pacific	Girls	2027	14	1.84
High-income Asia Pacific	Girls	2028	14	1.85
High-income Asia Pacific	Girls	2029	14	1.85
High-income Asia Pacific	Girls	2030	14	1.86
High-income Asia Pacific	Girls	2017	15	2.16
High-income Asia Pacific	Girls	2018	15	2.16
High-income Asia Pacific	Girls	2019	15	2.16
High-income Asia Pacific	Girls	2020	15	2.16
High-income Asia Pacific	Girls	2021	15	2.16
High-income Asia Pacific	Girls	2022	15	2.16
High-income Asia Pacific	Girls	2023	15	2.16
High-income Asia Pacific	Girls	2024	15	2.15
High-income Asia Pacific	Girls	2025	15	2.15
High-income Asia Pacific	Girls	2026	15	2.15
High-income Asia Pacific	Girls	2027	15	2.15
High-income Asia Pacific	Girls	2028	15	2.14
High-income Asia Pacific	Girls	2029	15	2.14
High-income Asia Pacific	Girls	2030	15	2.14
High-income Asia Pacific	Girls	2017	16	2.42
High-income Asia Pacific	Girls	2018	16	2.42
High-income Asia Pacific	Girls	2019	16	2.42
High-income Asia Pacific	Girls	2020	16	2.42
High-income Asia Pacific	Girls	2021	16	2.42
High-income Asia Pacific	Girls	2022	16	2.42
High-income Asia Pacific	Girls	2023	16	2.42
High-income Asia Pacific	Girls	2024	16	2.42
High-income Asia Pacific	Girls	2025	16	2.41
High-income Asia Pacific	Girls	2026	16	2.41
High-income Asia Pacific	Girls	2027	16	2.41
High-income Asia Pacific	Girls	2028	16	2.41
High-income Asia Pacific	Girls	2029	16	2.40

High-income Asia Pacific	Girls	2030	16	2.40
High-income Asia Pacific	Girls	2017	17	2.61
High-income Asia Pacific	Girls	2018	17	2.63
High-income Asia Pacific	Girls	2019	17	2.65
High-income Asia Pacific	Girls	2020	17	2.67
High-income Asia Pacific	Girls	2021	17	2.69
High-income Asia Pacific	Girls	2022	17	2.70
High-income Asia Pacific	Girls	2023	17	2.72
High-income Asia Pacific	Girls	2024	17	2.74
High-income Asia Pacific	Girls	2025	17	2.76
High-income Asia Pacific	Girls	2026	17	2.78
High-income Asia Pacific	Girls	2027	17	2.79
High-income Asia Pacific	Girls	2028	17	2.81
High-income Asia Pacific	Girls	2029	17	2.83
High-income Asia Pacific	Girls	2030	17	2.85
High-income Asia Pacific	Girls	2017	18	2.77
High-income Asia Pacific	Girls	2018	18	2.79
High-income Asia Pacific	Girls	2019	18	2.81
High-income Asia Pacific	Girls	2020	18	2.83
High-income Asia Pacific	Girls	2021	18	2.85
High-income Asia Pacific	Girls	2022	18	2.87
High-income Asia Pacific	Girls	2023	18	2.89
High-income Asia Pacific	Girls	2024	18	2.91
High-income Asia Pacific	Girls	2025	18	2.93
High-income Asia Pacific	Girls	2026	18	2.95
High-income Asia Pacific	Girls	2027	18	2.97
High-income Asia Pacific	Girls	2028	18	2.99
High-income Asia Pacific	Girls	2029	18	3.01
High-income Asia Pacific	Girls	2030	18	3.02
High-income Western countries	Boys	2017	12	1.88
High-income Western countries	Boys	2018	12	1.89
High-income Western countries	Boys	2019	12	1.90
High-income Western countries	Boys	2020	12	1.91
High-income Western countries	Boys	2021	12	1.92
High-income Western countries	Boys	2022	12	1.94
High-income Western countries	Boys	2023	12	1.95
High-income Western countries	Boys	2024	12	1.96
High-income Western countries	Boys	2025	12	1.97
High-income Western countries	Boys	2026	12	1.98
High-income Western countries	Boys	2027	12	1.99
High-income Western countries	Boys	2028	12	2.01
High-income Western countries	Boys	2029	12	2.02
High-income Western countries	Boys	2030	12	2.03
High-income Western countries	Boys	2017	13	2.63
High-income Western countries	Boys	2018	13	2.65
High-income Western countries	Boys	2019	13	2.67
High-income Western countries	Boys	2020	13	2.69
High-income Western countries	Boys	2021	13	2.70
High-income Western countries	Boys	2022	13	2.72
High-income Western countries	Boys	2023	13	2.74
High-income Western countries	Boys	2024	13	2.76
High-income Western countries	Boys	2025	13	2.78
High-income Western countries	Boys	2026	13	2.80
High-income Western countries	Boys	2027	13	2.82

High-income Western countries	Boys	2028	13	2.83
High-income Western countries	Boys	2029	13	2.85
High-income Western countries	Boys	2030	13	2.87
High-income Western countries	Boys	2017	14	3.56
High-income Western countries	Boys	2018	14	3.59
High-income Western countries	Boys	2019	14	3.61
High-income Western countries	Boys	2020	14	3.64
High-income Western countries	Boys	2021	14	3.66
High-income Western countries	Boys	2022	14	3.69
High-income Western countries	Boys	2023	14	3.72
High-income Western countries	Boys	2024	14	3.74
High-income Western countries	Boys	2025	14	3.77
High-income Western countries	Boys	2026	14	3.80
High-income Western countries	Boys	2027	14	3.83
High-income Western countries	Boys	2028	14	3.86
High-income Western countries	Boys	2029	14	3.88
High-income Western countries	Boys	2030	14	3.91
High-income Western countries	Boys	2017	15	4.57
High-income Western countries	Boys	2018	15	4.61
High-income Western countries	Boys	2019	15	4.66
High-income Western countries	Boys	2020	15	4.70
High-income Western countries	Boys	2021	15	4.74
High-income Western countries	Boys	2022	15	4.79
High-income Western countries	Boys	2023	15	4.84
High-income Western countries	Boys	2024	15	4.88
High-income Western countries	Boys	2025	15	4.93
High-income Western countries	Boys	2026	15	4.98
High-income Western countries	Boys	2027	15	5.03
High-income Western countries	Boys	2028	15	5.08
High-income Western countries	Boys	2029	15	5.13
High-income Western countries	Boys	2030	15	5.18
High-income Western countries	Boys	2017	16	5.62
High-income Western countries	Boys	2018	16	5.67
High-income Western countries	Boys	2019	16	5.72
High-income Western countries	Boys	2020	16	5.77
High-income Western countries	Boys	2021	16	5.83
High-income Western countries	Boys	2022	16	5.89
High-income Western countries	Boys	2023	16	5.94
High-income Western countries	Boys	2024	16	6.00
High-income Western countries	Boys	2025	16	6.06
High-income Western countries	Boys	2026	16	6.13
High-income Western countries	Boys	2027	16	6.19
High-income Western countries	Boys	2028	16	6.25
High-income Western countries	Boys	2029	16	6.31
High-income Western countries	Boys	2030	16	6.38
High-income Western countries	Boys	2017	17	6.69
High-income Western countries	Boys	2018	17	6.75
High-income Western countries	Boys	2019	17	6.82
High-income Western countries	Boys	2020	17	6.88
High-income Western countries	Boys	2021	17	6.95
High-income Western countries	Boys	2022	17	7.02
High-income Western countries	Boys	2023	17	7.09
High-income Western countries	Boys	2024	17	7.16
High-income Western countries	Boys	2025	17	7.23

High-income Western countries	Boys	2026	17	7.30
High-income Western countries	Boys	2027	17	7.38
High-income Western countries	Boys	2028	17	7.45
High-income Western countries	Boys	2029	17	7.53
High-income Western countries	Boys	2030	17	7.60
High-income Western countries	Boys	2017	18	7.88
High-income Western countries	Boys	2018	18	7.95
High-income Western countries	Boys	2019	18	8.02
High-income Western countries	Boys	2020	18	8.10
High-income Western countries	Boys	2021	18	8.18
High-income Western countries	Boys	2022	18	8.25
High-income Western countries	Boys	2023	18	8.33
High-income Western countries	Boys	2024	18	8.42
High-income Western countries	Boys	2025	18	8.50
High-income Western countries	Boys	2026	18	8.58
High-income Western countries	Boys	2027	18	8.67
High-income Western countries	Boys	2028	18	8.76
High-income Western countries	Boys	2029	18	8.84
High-income Western countries	Boys	2030	18	8.93
High-income Western countries	Girls	2017	12	2.17
High-income Western countries	Girls	2018	12	2.18
High-income Western countries	Girls	2019	12	2.19
High-income Western countries	Girls	2020	12	2.20
High-income Western countries	Girls	2021	12	2.22
High-income Western countries	Girls	2022	12	2.23
High-income Western countries	Girls	2023	12	2.24
High-income Western countries	Girls	2024	12	2.25
High-income Western countries	Girls	2025	12	2.27
High-income Western countries	Girls	2026	12	2.28
High-income Western countries	Girls	2027	12	2.29
High-income Western countries	Girls	2028	12	2.30
High-income Western countries	Girls	2029	12	2.31
High-income Western countries	Girls	2030	12	2.33
High-income Western countries	Girls	2017	13	3.03
High-income Western countries	Girls	2018	13	3.05
High-income Western countries	Girls	2019	13	3.07
High-income Western countries	Girls	2020	13	3.09
High-income Western countries	Girls	2021	13	3.10
High-income Western countries	Girls	2022	13	3.12
High-income Western countries	Girls	2023	13	3.14
High-income Western countries	Girls	2024	13	3.16
High-income Western countries	Girls	2025	13	3.18
High-income Western countries	Girls	2026	13	3.19
High-income Western countries	Girls	2027	13	3.21
High-income Western countries	Girls	2028	13	3.23
High-income Western countries	Girls	2029	13	3.25
High-income Western countries	Girls	2030	13	3.27
High-income Western countries	Girls	2017	14	3.92
High-income Western countries	Girls	2018	14	3.94
High-income Western countries	Girls	2019	14	3.97
High-income Western countries	Girls	2020	14	3.99
High-income Western countries	Girls	2021	14	4.01
High-income Western countries	Girls	2022	14	4.04
High-income Western countries	Girls	2023	14	4.06

High-income Western countries	Girls	2024	14	4.08
High-income Western countries	Girls	2025	14	4.11
High-income Western countries	Girls	2026	14	4.13
High-income Western countries	Girls	2027	14	4.16
High-income Western countries	Girls	2028	14	4.18
High-income Western countries	Girls	2029	14	4.20
High-income Western countries	Girls	2030	14	4.23
High-income Western countries	Girls	2017	15	4.69
High-income Western countries	Girls	2018	15	4.73
High-income Western countries	Girls	2019	15	4.76
High-income Western countries	Girls	2020	15	4.79
High-income Western countries	Girls	2021	15	4.83
High-income Western countries	Girls	2022	15	4.86
High-income Western countries	Girls	2023	15	4.90
High-income Western countries	Girls	2024	15	4.93
High-income Western countries	Girls	2025	15	4.97
High-income Western countries	Girls	2026	15	5.01
High-income Western countries	Girls	2027	15	5.04
High-income Western countries	Girls	2028	15	5.08
High-income Western countries	Girls	2029	15	5.12
High-income Western countries	Girls	2030	15	5.16
High-income Western countries	Girls	2017	16	5.32
High-income Western countries	Girls	2018	16	5.36
High-income Western countries	Girls	2019	16	5.39
High-income Western countries	Girls	2020	16	5.43
High-income Western countries	Girls	2021	16	5.47
High-income Western countries	Girls	2022	16	5.51
High-income Western countries	Girls	2023	16	5.55
High-income Western countries	Girls	2024	16	5.59
High-income Western countries	Girls	2025	16	5.63
High-income Western countries	Girls	2026	16	5.67
High-income Western countries	Girls	2027	16	5.71
High-income Western countries	Girls	2028	16	5.75
High-income Western countries	Girls	2029	16	5.79
High-income Western countries	Girls	2030	16	5.83
High-income Western countries	Girls	2017	17	5.90
High-income Western countries	Girls	2018	17	5.93
High-income Western countries	Girls	2019	17	5.97
High-income Western countries	Girls	2020	17	6.01
High-income Western countries	Girls	2021	17	6.05
High-income Western countries	Girls	2022	17	6.10
High-income Western countries	Girls	2023	17	6.14
High-income Western countries	Girls	2024	17	6.18
High-income Western countries	Girls	2025	17	6.22
High-income Western countries	Girls	2026	17	6.27
High-income Western countries	Girls	2027	17	6.31
High-income Western countries	Girls	2028	17	6.35
High-income Western countries	Girls	2029	17	6.40
High-income Western countries	Girls	2030	17	6.44
High-income Western countries	Girls	2017	18	6.57
High-income Western countries	Girls	2018	18	6.62
High-income Western countries	Girls	2019	18	6.66
High-income Western countries	Girls	2020	18	6.70
High-income Western countries	Girls	2021	18	6.75

High-income Western countries	Girls	2022	18	6.79
High-income Western countries	Girls	2023	18	6.83
High-income Western countries	Girls	2024	18	6.88
High-income Western countries	Girls	2025	18	6.93
High-income Western countries	Girls	2026	18	6.97
High-income Western countries	Girls	2027	18	7.02
High-income Western countries	Girls	2028	18	7.07
High-income Western countries	Girls	2029	18	7.11
High-income Western countries	Girls	2030	18	7.16
Latin America and Caribbean	Boys	2017	12	1.61
Latin America and Caribbean	Boys	2018	12	1.64
Latin America and Caribbean	Boys	2019	12	1.67
Latin America and Caribbean	Boys	2020	12	1.70
Latin America and Caribbean	Boys	2021	12	1.73
Latin America and Caribbean	Boys	2022	12	1.76
Latin America and Caribbean	Boys	2023	12	1.79
Latin America and Caribbean	Boys	2024	12	1.82
Latin America and Caribbean	Boys	2025	12	1.85
Latin America and Caribbean	Boys	2026	12	1.88
Latin America and Caribbean	Boys	2027	12	1.91
Latin America and Caribbean	Boys	2028	12	1.94
Latin America and Caribbean	Boys	2029	12	1.97
Latin America and Caribbean	Boys	2030	12	2.00
Latin America and Caribbean	Boys	2017	13	2.22
Latin America and Caribbean	Boys	2018	13	2.27
Latin America and Caribbean	Boys	2019	13	2.31
Latin America and Caribbean	Boys	2020	13	2.36
Latin America and Caribbean	Boys	2021	13	2.40
Latin America and Caribbean	Boys	2022	13	2.45
Latin America and Caribbean	Boys	2023	13	2.49
Latin America and Caribbean	Boys	2024	13	2.54
Latin America and Caribbean	Boys	2025	13	2.58
Latin America and Caribbean	Boys	2026	13	2.63
Latin America and Caribbean	Boys	2027	13	2.67
Latin America and Caribbean	Boys	2028	13	2.71
Latin America and Caribbean	Boys	2029	13	2.76
Latin America and Caribbean	Boys	2030	13	2.80
Latin America and Caribbean	Boys	2017	14	2.96
Latin America and Caribbean	Boys	2018	14	3.02
Latin America and Caribbean	Boys	2019	14	3.09
Latin America and Caribbean	Boys	2020	14	3.15
Latin America and Caribbean	Boys	2021	14	3.22
Latin America and Caribbean	Boys	2022	14	3.28
Latin America and Caribbean	Boys	2023	14	3.35
Latin America and Caribbean	Boys	2024	14	3.41
Latin America and Caribbean	Boys	2025	14	3.48
Latin America and Caribbean	Boys	2026	14	3.54
Latin America and Caribbean	Boys	2027	14	3.61
Latin America and Caribbean	Boys	2028	14	3.67
Latin America and Caribbean	Boys	2029	14	3.73
Latin America and Caribbean	Boys	2030	14	3.79
Latin America and Caribbean	Boys	2017	15	3.75
Latin America and Caribbean	Boys	2018	15	3.83
Latin America and Caribbean	Boys	2019	15	3.91

Latin America and Caribbean	Boys	2020	15	3.99
Latin America and Caribbean	Boys	2021	15	4.08
Latin America and Caribbean	Boys	2022	15	4.16
Latin America and Caribbean	Boys	2023	15	4.25
Latin America and Caribbean	Boys	2024	15	4.33
Latin America and Caribbean	Boys	2025	15	4.42
Latin America and Caribbean	Boys	2026	15	4.51
Latin America and Caribbean	Boys	2027	15	4.60
Latin America and Caribbean	Boys	2028	15	4.69
Latin America and Caribbean	Boys	2029	15	4.77
Latin America and Caribbean	Boys	2030	15	4.86
Latin America and Caribbean	Boys	2017	16	4.55
Latin America and Caribbean	Boys	2018	16	4.64
Latin America and Caribbean	Boys	2019	16	4.75
Latin America and Caribbean	Boys	2020	16	4.85
Latin America and Caribbean	Boys	2021	16	4.95
Latin America and Caribbean	Boys	2022	16	5.06
Latin America and Caribbean	Boys	2023	16	5.16
Latin America and Caribbean	Boys	2024	16	5.27
Latin America and Caribbean	Boys	2025	16	5.38
Latin America and Caribbean	Boys	2026	16	5.49
Latin America and Caribbean	Boys	2027	16	5.60
Latin America and Caribbean	Boys	2028	16	5.71
Latin America and Caribbean	Boys	2029	16	5.83
Latin America and Caribbean	Boys	2030	16	5.94
Latin America and Caribbean	Boys	2017	17	5.36
Latin America and Caribbean	Boys	2018	17	5.47
Latin America and Caribbean	Boys	2019	17	5.58
Latin America and Caribbean	Boys	2020	17	5.69
Latin America and Caribbean	Boys	2021	17	5.80
Latin America and Caribbean	Boys	2022	17	5.91
Latin America and Caribbean	Boys	2023	17	6.02
Latin America and Caribbean	Boys	2024	17	6.13
Latin America and Caribbean	Boys	2025	17	6.24
Latin America and Caribbean	Boys	2026	17	6.35
Latin America and Caribbean	Boys	2027	17	6.46
Latin America and Caribbean	Boys	2028	17	6.57
Latin America and Caribbean	Boys	2029	17	6.68
Latin America and Caribbean	Boys	2030	17	6.79
Latin America and Caribbean	Boys	2017	18	6.27
Latin America and Caribbean	Boys	2018	18	6.40
Latin America and Caribbean	Boys	2019	18	6.53
Latin America and Caribbean	Boys	2020	18	6.67
Latin America and Caribbean	Boys	2021	18	6.81
Latin America and Caribbean	Boys	2022	18	6.95
Latin America and Caribbean	Boys	2023	18	7.10
Latin America and Caribbean	Boys	2024	18	7.24
Latin America and Caribbean	Boys	2025	18	7.39
Latin America and Caribbean	Boys	2026	18	7.54
Latin America and Caribbean	Boys	2027	18	7.69
Latin America and Caribbean	Boys	2028	18	7.84
Latin America and Caribbean	Boys	2029	18	7.99
Latin America and Caribbean	Boys	2030	18	8.14
Latin America and Caribbean	Girls	2017	12	1.89

Latin America and Caribbean	Girls	2018	12	1.92
Latin America and Caribbean	Girls	2019	12	1.95
Latin America and Caribbean	Girls	2020	12	1.99
Latin America and Caribbean	Girls	2021	12	2.02
Latin America and Caribbean	Girls	2022	12	2.06
Latin America and Caribbean	Girls	2023	12	2.09
Latin America and Caribbean	Girls	2024	12	2.12
Latin America and Caribbean	Girls	2025	12	2.16
Latin America and Caribbean	Girls	2026	12	2.19
Latin America and Caribbean	Girls	2027	12	2.22
Latin America and Caribbean	Girls	2028	12	2.26
Latin America and Caribbean	Girls	2029	12	2.29
Latin America and Caribbean	Girls	2030	12	2.32
Latin America and Caribbean	Girls	2017	13	2.63
Latin America and Caribbean	Girls	2018	13	2.68
Latin America and Caribbean	Girls	2019	13	2.72
Latin America and Caribbean	Girls	2020	13	2.77
Latin America and Caribbean	Girls	2021	13	2.82
Latin America and Caribbean	Girls	2022	13	2.87
Latin America and Caribbean	Girls	2023	13	2.92
Latin America and Caribbean	Girls	2024	13	2.97
Latin America and Caribbean	Girls	2025	13	3.02
Latin America and Caribbean	Girls	2026	13	3.07
Latin America and Caribbean	Girls	2027	13	3.12
Latin America and Caribbean	Girls	2028	13	3.16
Latin America and Caribbean	Girls	2029	13	3.21
Latin America and Caribbean	Girls	2030	13	3.26
Latin America and Caribbean	Girls	2017	14	3.40
Latin America and Caribbean	Girls	2018	14	3.46
Latin America and Caribbean	Girls	2019	14	3.52
Latin America and Caribbean	Girls	2020	14	3.58
Latin America and Caribbean	Girls	2021	14	3.65
Latin America and Caribbean	Girls	2022	14	3.71
Latin America and Caribbean	Girls	2023	14	3.78
Latin America and Caribbean	Girls	2024	14	3.84
Latin America and Caribbean	Girls	2025	14	3.90
Latin America and Caribbean	Girls	2026	14	3.97
Latin America and Caribbean	Girls	2027	14	4.03
Latin America and Caribbean	Girls	2028	14	4.09
Latin America and Caribbean	Girls	2029	14	4.16
Latin America and Caribbean	Girls	2030	14	4.22
Latin America and Caribbean	Girls	2017	15	4.07
Latin America and Caribbean	Girls	2018	15	4.13
Latin America and Caribbean	Girls	2019	15	4.20
Latin America and Caribbean	Girls	2020	15	4.27
Latin America and Caribbean	Girls	2021	15	4.34
Latin America and Caribbean	Girls	2022	15	4.40
Latin America and Caribbean	Girls	2023	15	4.47
Latin America and Caribbean	Girls	2024	15	4.54
Latin America and Caribbean	Girls	2025	15	4.61
Latin America and Caribbean	Girls	2026	15	4.69
Latin America and Caribbean	Girls	2027	15	4.76
Latin America and Caribbean	Girls	2028	15	4.83
Latin America and Caribbean	Girls	2029	15	4.90

Latin America and Caribbean	Girls	2030	15	4.97
Latin America and Caribbean	Girls	2017	16	4.61
Latin America and Caribbean	Girls	2018	16	4.68
Latin America and Caribbean	Girls	2019	16	4.76
Latin America and Caribbean	Girls	2020	16	4.83
Latin America and Caribbean	Girls	2021	16	4.91
Latin America and Caribbean	Girls	2022	16	4.98
Latin America and Caribbean	Girls	2023	16	5.06
Latin America and Caribbean	Girls	2024	16	5.14
Latin America and Caribbean	Girls	2025	16	5.22
Latin America and Caribbean	Girls	2026	16	5.29
Latin America and Caribbean	Girls	2027	16	5.37
Latin America and Caribbean	Girls	2028	16	5.45
Latin America and Caribbean	Girls	2029	16	5.53
Latin America and Caribbean	Girls	2030	16	5.61
Latin America and Caribbean	Girls	2017	17	5.09
Latin America and Caribbean	Girls	2018	17	5.17
Latin America and Caribbean	Girls	2019	17	5.25
Latin America and Caribbean	Girls	2020	17	5.33
Latin America and Caribbean	Girls	2021	17	5.41
Latin America and Caribbean	Girls	2022	17	5.49
Latin America and Caribbean	Girls	2023	17	5.58
Latin America and Caribbean	Girls	2024	17	5.66
Latin America and Caribbean	Girls	2025	17	5.75
Latin America and Caribbean	Girls	2026	17	5.83
Latin America and Caribbean	Girls	2027	17	5.92
Latin America and Caribbean	Girls	2028	17	6.00
Latin America and Caribbean	Girls	2029	17	6.09
Latin America and Caribbean	Girls	2030	17	6.17
Latin America and Caribbean	Girls	2017	18	5.64
Latin America and Caribbean	Girls	2018	18	5.73
Latin America and Caribbean	Girls	2019	18	5.82
Latin America and Caribbean	Girls	2020	18	5.91
Latin America and Caribbean	Girls	2021	18	6.00
Latin America and Caribbean	Girls	2022	18	6.09
Latin America and Caribbean	Girls	2023	18	6.18
Latin America and Caribbean	Girls	2024	18	6.27
Latin America and Caribbean	Girls	2025	18	6.36
Latin America and Caribbean	Girls	2026	18	6.45
Latin America and Caribbean	Girls	2027	18	6.54
Latin America and Caribbean	Girls	2028	18	6.63
Latin America and Caribbean	Girls	2029	18	6.72
Latin America and Caribbean	Girls	2030	18	6.82
Oceania	Boys	2017	12	1.44
Oceania	Boys	2018	12	1.48
Oceania	Boys	2019	12	1.52
Oceania	Boys	2020	12	1.57
Oceania	Boys	2021	12	1.61
Oceania	Boys	2022	12	1.65
Oceania	Boys	2023	12	1.70
Oceania	Boys	2024	12	1.75
Oceania	Boys	2025	12	1.80
Oceania	Boys	2026	12	1.84
Oceania	Boys	2027	12	1.89

Oceania	Boys	2028	12	1.94
Oceania	Boys	2029	12	2.00
Oceania	Boys	2030	12	2.05
Oceania	Boys	2017	13	1.98
Oceania	Boys	2018	13	2.04
Oceania	Boys	2019	13	2.11
Oceania	Boys	2020	13	2.17
Oceania	Boys	2021	13	2.24
Oceania	Boys	2022	13	2.31
Oceania	Boys	2023	13	2.38
Oceania	Boys	2024	13	2.45
Oceania	Boys	2025	13	2.52
Oceania	Boys	2026	13	2.59
Oceania	Boys	2027	13	2.67
Oceania	Boys	2028	13	2.74
Oceania	Boys	2029	13	2.82
Oceania	Boys	2030	13	2.90
Oceania	Boys	2017	14	2.65
Oceania	Boys	2018	14	2.73
Oceania	Boys	2019	14	2.82
Oceania	Boys	2020	14	2.92
Oceania	Boys	2021	14	3.01
Oceania	Boys	2022	14	3.11
Oceania	Boys	2023	14	3.21
Oceania	Boys	2024	14	3.31
Oceania	Boys	2025	14	3.42
Oceania	Boys	2026	14	3.52
Oceania	Boys	2027	14	3.63
Oceania	Boys	2028	14	3.75
Oceania	Boys	2029	14	3.86
Oceania	Boys	2030	14	3.98
Oceania	Boys	2017	15	3.38
Oceania	Boys	2018	15	3.48
Oceania	Boys	2019	15	3.59
Oceania	Boys	2020	15	3.69
Oceania	Boys	2021	15	3.80
Oceania	Boys	2022	15	3.91
Oceania	Boys	2023	15	4.01
Oceania	Boys	2024	15	4.12
Oceania	Boys	2025	15	4.22
Oceania	Boys	2026	15	4.33
Oceania	Boys	2027	15	4.44
Oceania	Boys	2028	15	4.54
Oceania	Boys	2029	15	4.65
Oceania	Boys	2030	15	4.75
Oceania	Boys	2017	16	4.14
Oceania	Boys	2018	16	4.27
Oceania	Boys	2019	16	4.40
Oceania	Boys	2020	16	4.53
Oceania	Boys	2021	16	4.66
Oceania	Boys	2022	16	4.79
Oceania	Boys	2023	16	4.92
Oceania	Boys	2024	16	5.05
Oceania	Boys	2025	16	5.18

Oceania	Boys	2026	16	5.31
Oceania	Boys	2027	16	5.44
Oceania	Boys	2028	16	5.57
Oceania	Boys	2029	16	5.70
Oceania	Boys	2030	16	5.83
Oceania	Boys	2017	17	4.93
Oceania	Boys	2018	17	5.08
Oceania	Boys	2019	17	5.24
Oceania	Boys	2020	17	5.39
Oceania	Boys	2021	17	5.54
Oceania	Boys	2022	17	5.70
Oceania	Boys	2023	17	5.85
Oceania	Boys	2024	17	6.00
Oceania	Boys	2025	17	6.16
Oceania	Boys	2026	17	6.31
Oceania	Boys	2027	17	6.46
Oceania	Boys	2028	17	6.61
Oceania	Boys	2029	17	6.77
Oceania	Boys	2030	17	6.92
Oceania	Boys	2017	18	5.81
Oceania	Boys	2018	18	5.99
Oceania	Boys	2019	18	6.17
Oceania	Boys	2020	18	6.34
Oceania	Boys	2021	18	6.52
Oceania	Boys	2022	18	6.70
Oceania	Boys	2023	18	6.87
Oceania	Boys	2024	18	7.05
Oceania	Boys	2025	18	7.23
Oceania	Boys	2026	18	7.40
Oceania	Boys	2027	18	7.58
Oceania	Boys	2028	18	7.76
Oceania	Boys	2029	18	7.93
Oceania	Boys	2030	18	8.11
Oceania	Girls	2017	12	2.24
Oceania	Girls	2018	12	2.29
Oceania	Girls	2019	12	2.34
Oceania	Girls	2020	12	2.40
Oceania	Girls	2021	12	2.45
Oceania	Girls	2022	12	2.51
Oceania	Girls	2023	12	2.56
Oceania	Girls	2024	12	2.62
Oceania	Girls	2025	12	2.68
Oceania	Girls	2026	12	2.73
Oceania	Girls	2027	12	2.79
Oceania	Girls	2028	12	2.84
Oceania	Girls	2029	12	2.90
Oceania	Girls	2030	12	2.96
Oceania	Girls	2017	13	3.16
Oceania	Girls	2018	13	3.23
Oceania	Girls	2019	13	3.31
Oceania	Girls	2020	13	3.39
Oceania	Girls	2021	13	3.47
Oceania	Girls	2022	13	3.55
Oceania	Girls	2023	13	3.63

Oceania	Girls	2024	13	3.71
Oceania	Girls	2025	13	3.80
Oceania	Girls	2026	13	3.88
Oceania	Girls	2027	13	3.96
Oceania	Girls	2028	13	4.04
Oceania	Girls	2029	13	4.13
Oceania	Girls	2030	13	4.21
Oceania	Girls	2017	14	4.13
Oceania	Girls	2018	14	4.23
Oceania	Girls	2019	14	4.33
Oceania	Girls	2020	14	4.43
Oceania	Girls	2021	14	4.54
Oceania	Girls	2022	14	4.65
Oceania	Girls	2023	14	4.75
Oceania	Girls	2024	14	4.86
Oceania	Girls	2025	14	4.97
Oceania	Girls	2026	14	5.08
Oceania	Girls	2027	14	5.19
Oceania	Girls	2028	14	5.30
Oceania	Girls	2029	14	5.41
Oceania	Girls	2030	14	5.53
Oceania	Girls	2017	15	4.99
Oceania	Girls	2018	15	5.12
Oceania	Girls	2019	15	5.24
Oceania	Girls	2020	15	5.37
Oceania	Girls	2021	15	5.51
Oceania	Girls	2022	15	5.64
Oceania	Girls	2023	15	5.78
Oceania	Girls	2024	15	5.93
Oceania	Girls	2025	15	6.07
Oceania	Girls	2026	15	6.22
Oceania	Girls	2027	15	6.37
Oceania	Girls	2028	15	6.53
Oceania	Girls	2029	15	6.68
Oceania	Girls	2030	15	6.84
Oceania	Girls	2017	16	5.69
Oceania	Girls	2018	16	5.83
Oceania	Girls	2019	16	5.97
Oceania	Girls	2020	16	6.12
Oceania	Girls	2021	16	6.27
Oceania	Girls	2022	16	6.42
Oceania	Girls	2023	16	6.58
Oceania	Girls	2024	16	6.74
Oceania	Girls	2025	16	6.90
Oceania	Girls	2026	16	7.07
Oceania	Girls	2027	16	7.24
Oceania	Girls	2028	16	7.41
Oceania	Girls	2029	16	7.59
Oceania	Girls	2030	16	7.77
Oceania	Girls	2017	17	6.29
Oceania	Girls	2018	17	6.44
Oceania	Girls	2019	17	6.60
Oceania	Girls	2020	17	6.76
Oceania	Girls	2021	17	6.92

Oceania	Girls	2022	17	7.08
Oceania	Girls	2023	17	7.25
Oceania	Girls	2024	17	7.43
Oceania	Girls	2025	17	7.60
Oceania	Girls	2026	17	7.78
Oceania	Girls	2027	17	7.96
Oceania	Girls	2028	17	8.14
Oceania	Girls	2029	17	8.33
Oceania	Girls	2030	17	8.52
Oceania	Girls	2017	18	6.97
Oceania	Girls	2018	18	7.13
Oceania	Girls	2019	18	7.30
Oceania	Girls	2020	18	7.47
Oceania	Girls	2021	18	7.64
Oceania	Girls	2022	18	7.82
Oceania	Girls	2023	18	8.00
Oceania	Girls	2024	18	8.18
Oceania	Girls	2025	18	8.37
Oceania	Girls	2026	18	8.55
Oceania	Girls	2027	18	8.74
Oceania	Girls	2028	18	8.94
Oceania	Girls	2029	18	9.13
Oceania	Girls	2030	18	9.33
South Asia	Boys	2017	12	0.58
South Asia	Boys	2018	12	0.60
South Asia	Boys	2019	12	0.62
South Asia	Boys	2020	12	0.65
South Asia	Boys	2021	12	0.67
South Asia	Boys	2022	12	0.69
South Asia	Boys	2023	12	0.72
South Asia	Boys	2024	12	0.74
South Asia	Boys	2025	12	0.76
South Asia	Boys	2026	12	0.78
South Asia	Boys	2027	12	0.81
South Asia	Boys	2028	12	0.83
South Asia	Boys	2029	12	0.85
South Asia	Boys	2030	12	0.88
South Asia	Boys	2017	13	0.76
South Asia	Boys	2018	13	0.79
South Asia	Boys	2019	13	0.82
South Asia	Boys	2020	13	0.85
South Asia	Boys	2021	13	0.88
South Asia	Boys	2022	13	0.91
South Asia	Boys	2023	13	0.94
South Asia	Boys	2024	13	0.97
South Asia	Boys	2025	13	1.00
South Asia	Boys	2026	13	1.03
South Asia	Boys	2027	13	1.06
South Asia	Boys	2028	13	1.10
South Asia	Boys	2029	13	1.13
South Asia	Boys	2030	13	1.16
South Asia	Boys	2017	14	0.98
South Asia	Boys	2018	14	1.02
South Asia	Boys	2019	14	1.06

South Asia	Boys	2020	14	1.10
South Asia	Boys	2021	14	1.13
South Asia	Boys	2022	14	1.17
South Asia	Boys	2023	14	1.21
South Asia	Boys	2024	14	1.25
South Asia	Boys	2025	14	1.29
South Asia	Boys	2026	14	1.33
South Asia	Boys	2027	14	1.37
South Asia	Boys	2028	14	1.41
South Asia	Boys	2029	14	1.45
South Asia	Boys	2030	14	1.49
South Asia	Boys	2017	15	1.23
South Asia	Boys	2018	15	1.27
South Asia	Boys	2019	15	1.32
South Asia	Boys	2020	15	1.37
South Asia	Boys	2021	15	1.42
South Asia	Boys	2022	15	1.46
South Asia	Boys	2023	15	1.51
South Asia	Boys	2024	15	1.56
South Asia	Boys	2025	15	1.60
South Asia	Boys	2026	15	1.65
South Asia	Boys	2027	15	1.70
South Asia	Boys	2028	15	1.74
South Asia	Boys	2029	15	1.79
South Asia	Boys	2030	15	1.84
South Asia	Boys	2017	16	1.50
South Asia	Boys	2018	16	1.56
South Asia	Boys	2019	16	1.61
South Asia	Boys	2020	16	1.67
South Asia	Boys	2021	16	1.72
South Asia	Boys	2022	16	1.78
South Asia	Boys	2023	16	1.83
South Asia	Boys	2024	16	1.88
South Asia	Boys	2025	16	1.94
South Asia	Boys	2026	16	1.99
South Asia	Boys	2027	16	2.05
South Asia	Boys	2028	16	2.10
South Asia	Boys	2029	16	2.15
South Asia	Boys	2030	16	2.21
South Asia	Boys	2017	17	1.81
South Asia	Boys	2018	17	1.87
South Asia	Boys	2019	17	1.93
South Asia	Boys	2020	17	2.00
South Asia	Boys	2021	17	2.06
South Asia	Boys	2022	17	2.12
South Asia	Boys	2023	17	2.18
South Asia	Boys	2024	17	2.24
South Asia	Boys	2025	17	2.31
South Asia	Boys	2026	17	2.37
South Asia	Boys	2027	17	2.43
South Asia	Boys	2028	17	2.49
South Asia	Boys	2029	17	2.55
South Asia	Boys	2030	17	2.62
South Asia	Boys	2017	18	2.16

South Asia	Boys	2018	18	2.23
South Asia	Boys	2019	18	2.31
South Asia	Boys	2020	18	2.38
South Asia	Boys	2021	18	2.45
South Asia	Boys	2022	18	2.52
South Asia	Boys	2023	18	2.59
South Asia	Boys	2024	18	2.67
South Asia	Boys	2025	18	2.74
South Asia	Boys	2026	18	2.81
South Asia	Boys	2027	18	2.88
South Asia	Boys	2028	18	2.95
South Asia	Boys	2029	18	3.03
South Asia	Boys	2030	18	3.10
South Asia	Girls	2017	12	0.58
South Asia	Girls	2018	12	0.60
South Asia	Girls	2019	12	0.63
South Asia	Girls	2020	12	0.65
South Asia	Girls	2021	12	0.67
South Asia	Girls	2022	12	0.70
South Asia	Girls	2023	12	0.72
South Asia	Girls	2024	12	0.74
South Asia	Girls	2025	12	0.76
South Asia	Girls	2026	12	0.79
South Asia	Girls	2027	12	0.81
South Asia	Girls	2028	12	0.83
South Asia	Girls	2029	12	0.86
South Asia	Girls	2030	12	0.88
South Asia	Girls	2017	13	0.82
South Asia	Girls	2018	13	0.85
South Asia	Girls	2019	13	0.88
South Asia	Girls	2020	13	0.91
South Asia	Girls	2021	13	0.94
South Asia	Girls	2022	13	0.97
South Asia	Girls	2023	13	1.00
South Asia	Girls	2024	13	1.04
South Asia	Girls	2025	13	1.07
South Asia	Girls	2026	13	1.10
South Asia	Girls	2027	13	1.13
South Asia	Girls	2028	13	1.16
South Asia	Girls	2029	13	1.19
South Asia	Girls	2030	13	1.22
South Asia	Girls	2017	14	1.09
South Asia	Girls	2018	14	1.13
South Asia	Girls	2019	14	1.17
South Asia	Girls	2020	14	1.21
South Asia	Girls	2021	14	1.25
South Asia	Girls	2022	14	1.29
South Asia	Girls	2023	14	1.33
South Asia	Girls	2024	14	1.37
South Asia	Girls	2025	14	1.40
South Asia	Girls	2026	14	1.44
South Asia	Girls	2027	14	1.48
South Asia	Girls	2028	14	1.52
South Asia	Girls	2029	14	1.56

South Asia	Girls	2030	14	1.60
South Asia	Girls	2017	15	1.37
South Asia	Girls	2018	15	1.41
South Asia	Girls	2019	15	1.46
South Asia	Girls	2020	15	1.50
South Asia	Girls	2021	15	1.54
South Asia	Girls	2022	15	1.59
South Asia	Girls	2023	15	1.63
South Asia	Girls	2024	15	1.68
South Asia	Girls	2025	15	1.72
South Asia	Girls	2026	15	1.77
South Asia	Girls	2027	15	1.81
South Asia	Girls	2028	15	1.86
South Asia	Girls	2029	15	1.90
South Asia	Girls	2030	15	1.95
South Asia	Girls	2017	16	1.60
South Asia	Girls	2018	16	1.65
South Asia	Girls	2019	16	1.70
South Asia	Girls	2020	16	1.75
South Asia	Girls	2021	16	1.80
South Asia	Girls	2022	16	1.85
South Asia	Girls	2023	16	1.90
South Asia	Girls	2024	16	1.95
South Asia	Girls	2025	16	2.00
South Asia	Girls	2026	16	2.05
South Asia	Girls	2027	16	2.10
South Asia	Girls	2028	16	2.15
South Asia	Girls	2029	16	2.20
South Asia	Girls	2030	16	2.25
South Asia	Girls	2017	17	1.80
South Asia	Girls	2018	17	1.85
South Asia	Girls	2019	17	1.91
South Asia	Girls	2020	17	1.96
South Asia	Girls	2021	17	2.02
South Asia	Girls	2022	17	2.07
South Asia	Girls	2023	17	2.13
South Asia	Girls	2024	17	2.18
South Asia	Girls	2025	17	2.24
South Asia	Girls	2026	17	2.29
South Asia	Girls	2027	17	2.35
South Asia	Girls	2028	17	2.40
South Asia	Girls	2029	17	2.46
South Asia	Girls	2030	17	2.51
South Asia	Girls	2017	18	1.97
South Asia	Girls	2018	18	2.03
South Asia	Girls	2019	18	2.09
South Asia	Girls	2020	18	2.16
South Asia	Girls	2021	18	2.22
South Asia	Girls	2022	18	2.28
South Asia	Girls	2023	18	2.34
South Asia	Girls	2024	18	2.41
South Asia	Girls	2025	18	2.47
South Asia	Girls	2026	18	2.53
South Asia	Girls	2027	18	2.59

South Asia	Girls	2028	18	2.65
South Asia	Girls	2029	18	2.72
South Asia	Girls	2030	18	2.78
Sub-Saharan Africa	Boys	2017	12	0.60
Sub-Saharan Africa	Boys	2018	12	0.62
Sub-Saharan Africa	Boys	2019	12	0.64
Sub-Saharan Africa	Boys	2020	12	0.66
Sub-Saharan Africa	Boys	2021	12	0.68
Sub-Saharan Africa	Boys	2022	12	0.69
Sub-Saharan Africa	Boys	2023	12	0.71
Sub-Saharan Africa	Boys	2024	12	0.73
Sub-Saharan Africa	Boys	2025	12	0.75
Sub-Saharan Africa	Boys	2026	12	0.76
Sub-Saharan Africa	Boys	2027	12	0.78
Sub-Saharan Africa	Boys	2028	12	0.80
Sub-Saharan Africa	Boys	2029	12	0.82
Sub-Saharan Africa	Boys	2030	12	0.83
Sub-Saharan Africa	Boys	2017	13	0.80
Sub-Saharan Africa	Boys	2018	13	0.83
Sub-Saharan Africa	Boys	2019	13	0.85
Sub-Saharan Africa	Boys	2020	13	0.88
Sub-Saharan Africa	Boys	2021	13	0.90
Sub-Saharan Africa	Boys	2022	13	0.92
Sub-Saharan Africa	Boys	2023	13	0.95
Sub-Saharan Africa	Boys	2024	13	0.97
Sub-Saharan Africa	Boys	2025	13	1.00
Sub-Saharan Africa	Boys	2026	13	1.02
Sub-Saharan Africa	Boys	2027	13	1.04
Sub-Saharan Africa	Boys	2028	13	1.07
Sub-Saharan Africa	Boys	2029	13	1.09
Sub-Saharan Africa	Boys	2030	13	1.11
Sub-Saharan Africa	Boys	2017	14	1.06
Sub-Saharan Africa	Boys	2018	14	1.09
Sub-Saharan Africa	Boys	2019	14	1.12
Sub-Saharan Africa	Boys	2020	14	1.15
Sub-Saharan Africa	Boys	2021	14	1.18
Sub-Saharan Africa	Boys	2022	14	1.21
Sub-Saharan Africa	Boys	2023	14	1.24
Sub-Saharan Africa	Boys	2024	14	1.28
Sub-Saharan Africa	Boys	2025	14	1.31
Sub-Saharan Africa	Boys	2026	14	1.34
Sub-Saharan Africa	Boys	2027	14	1.37
Sub-Saharan Africa	Boys	2028	14	1.40
Sub-Saharan Africa	Boys	2029	14	1.43
Sub-Saharan Africa	Boys	2030	14	1.46
Sub-Saharan Africa	Boys	2017	15	1.36
Sub-Saharan Africa	Boys	2018	15	1.40
Sub-Saharan Africa	Boys	2019	15	1.44
Sub-Saharan Africa	Boys	2020	15	1.47
Sub-Saharan Africa	Boys	2021	15	1.51
Sub-Saharan Africa	Boys	2022	15	1.55
Sub-Saharan Africa	Boys	2023	15	1.58
Sub-Saharan Africa	Boys	2024	15	1.62
Sub-Saharan Africa	Boys	2025	15	1.66

Sub-Saharan Africa	Boys	2026	15	1.70
Sub-Saharan Africa	Boys	2027	15	1.73
Sub-Saharan Africa	Boys	2028	15	1.77
Sub-Saharan Africa	Boys	2029	15	1.81
Sub-Saharan Africa	Boys	2030	15	1.84
Sub-Saharan Africa	Boys	2017	16	1.70
Sub-Saharan Africa	Boys	2018	16	1.74
Sub-Saharan Africa	Boys	2019	16	1.79
Sub-Saharan Africa	Boys	2020	16	1.83
Sub-Saharan Africa	Boys	2021	16	1.88
Sub-Saharan Africa	Boys	2022	16	1.92
Sub-Saharan Africa	Boys	2023	16	1.96
Sub-Saharan Africa	Boys	2024	16	2.01
Sub-Saharan Africa	Boys	2025	16	2.05
Sub-Saharan Africa	Boys	2026	16	2.09
Sub-Saharan Africa	Boys	2027	16	2.14
Sub-Saharan Africa	Boys	2028	16	2.18
Sub-Saharan Africa	Boys	2029	16	2.23
Sub-Saharan Africa	Boys	2030	16	2.27
Sub-Saharan Africa	Boys	2017	17	2.08
Sub-Saharan Africa	Boys	2018	17	2.13
Sub-Saharan Africa	Boys	2019	17	2.18
Sub-Saharan Africa	Boys	2020	17	2.23
Sub-Saharan Africa	Boys	2021	17	2.28
Sub-Saharan Africa	Boys	2022	17	2.33
Sub-Saharan Africa	Boys	2023	17	2.38
Sub-Saharan Africa	Boys	2024	17	2.43
Sub-Saharan Africa	Boys	2025	17	2.48
Sub-Saharan Africa	Boys	2026	17	2.53
Sub-Saharan Africa	Boys	2027	17	2.58
Sub-Saharan Africa	Boys	2028	17	2.63
Sub-Saharan Africa	Boys	2029	17	2.68
Sub-Saharan Africa	Boys	2030	17	2.73
Sub-Saharan Africa	Boys	2017	18	2.50
Sub-Saharan Africa	Boys	2018	18	2.56
Sub-Saharan Africa	Boys	2019	18	2.62
Sub-Saharan Africa	Boys	2020	18	2.67
Sub-Saharan Africa	Boys	2021	18	2.73
Sub-Saharan Africa	Boys	2022	18	2.79
Sub-Saharan Africa	Boys	2023	18	2.85
Sub-Saharan Africa	Boys	2024	18	2.91
Sub-Saharan Africa	Boys	2025	18	2.96
Sub-Saharan Africa	Boys	2026	18	3.02
Sub-Saharan Africa	Boys	2027	18	3.08
Sub-Saharan Africa	Boys	2028	18	3.14
Sub-Saharan Africa	Boys	2029	18	3.20
Sub-Saharan Africa	Boys	2030	18	3.26
Sub-Saharan Africa	Girls	2017	12	1.01
Sub-Saharan Africa	Girls	2018	12	1.04
Sub-Saharan Africa	Girls	2019	12	1.07
Sub-Saharan Africa	Girls	2020	12	1.10
Sub-Saharan Africa	Girls	2021	12	1.13
Sub-Saharan Africa	Girls	2022	12	1.16
Sub-Saharan Africa	Girls	2023	12	1.19

Sub-Saharan Africa	Girls	2024	12	1.22
Sub-Saharan Africa	Girls	2025	12	1.25
Sub-Saharan Africa	Girls	2026	12	1.28
Sub-Saharan Africa	Girls	2027	12	1.31
Sub-Saharan Africa	Girls	2028	12	1.34
Sub-Saharan Africa	Girls	2029	12	1.37
Sub-Saharan Africa	Girls	2030	12	1.40
Sub-Saharan Africa	Girls	2017	13	1.43
Sub-Saharan Africa	Girls	2018	13	1.48
Sub-Saharan Africa	Girls	2019	13	1.52
Sub-Saharan Africa	Girls	2020	13	1.56
Sub-Saharan Africa	Girls	2021	13	1.60
Sub-Saharan Africa	Girls	2022	13	1.64
Sub-Saharan Africa	Girls	2023	13	1.68
Sub-Saharan Africa	Girls	2024	13	1.73
Sub-Saharan Africa	Girls	2025	13	1.77
Sub-Saharan Africa	Girls	2026	13	1.81
Sub-Saharan Africa	Girls	2027	13	1.85
Sub-Saharan Africa	Girls	2028	13	1.89
Sub-Saharan Africa	Girls	2029	13	1.93
Sub-Saharan Africa	Girls	2030	13	1.98
Sub-Saharan Africa	Girls	2017	14	1.90
Sub-Saharan Africa	Girls	2018	14	1.96
Sub-Saharan Africa	Girls	2019	14	2.01
Sub-Saharan Africa	Girls	2020	14	2.06
Sub-Saharan Africa	Girls	2021	14	2.12
Sub-Saharan Africa	Girls	2022	14	2.17
Sub-Saharan Africa	Girls	2023	14	2.22
Sub-Saharan Africa	Girls	2024	14	2.28
Sub-Saharan Africa	Girls	2025	14	2.33
Sub-Saharan Africa	Girls	2026	14	2.38
Sub-Saharan Africa	Girls	2027	14	2.43
Sub-Saharan Africa	Girls	2028	14	2.49
Sub-Saharan Africa	Girls	2029	14	2.54
Sub-Saharan Africa	Girls	2030	14	2.59
Sub-Saharan Africa	Girls	2017	15	2.35
Sub-Saharan Africa	Girls	2018	15	2.41
Sub-Saharan Africa	Girls	2019	15	2.48
Sub-Saharan Africa	Girls	2020	15	2.54
Sub-Saharan Africa	Girls	2021	15	2.60
Sub-Saharan Africa	Girls	2022	15	2.66
Sub-Saharan Africa	Girls	2023	15	2.72
Sub-Saharan Africa	Girls	2024	15	2.78
Sub-Saharan Africa	Girls	2025	15	2.84
Sub-Saharan Africa	Girls	2026	15	2.90
Sub-Saharan Africa	Girls	2027	15	2.96
Sub-Saharan Africa	Girls	2028	15	3.02
Sub-Saharan Africa	Girls	2029	15	3.09
Sub-Saharan Africa	Girls	2030	15	3.15
Sub-Saharan Africa	Girls	2017	16	2.73
Sub-Saharan Africa	Girls	2018	16	2.80
Sub-Saharan Africa	Girls	2019	16	2.86
Sub-Saharan Africa	Girls	2020	16	2.93
Sub-Saharan Africa	Girls	2021	16	3.00

Sub-Saharan Africa	Girls	2022	16	3.07
Sub-Saharan Africa	Girls	2023	16	3.14
Sub-Saharan Africa	Girls	2024	16	3.20
Sub-Saharan Africa	Girls	2025	16	3.27
Sub-Saharan Africa	Girls	2026	16	3.34
Sub-Saharan Africa	Girls	2027	16	3.41
Sub-Saharan Africa	Girls	2028	16	3.48
Sub-Saharan Africa	Girls	2029	16	3.55
Sub-Saharan Africa	Girls	2030	16	3.61
Sub-Saharan Africa	Girls	2017	17	3.05
Sub-Saharan Africa	Girls	2018	17	3.12
Sub-Saharan Africa	Girls	2019	17	3.20
Sub-Saharan Africa	Girls	2020	17	3.27
Sub-Saharan Africa	Girls	2021	17	3.34
Sub-Saharan Africa	Girls	2022	17	3.42
Sub-Saharan Africa	Girls	2023	17	3.49
Sub-Saharan Africa	Girls	2024	17	3.57
Sub-Saharan Africa	Girls	2025	17	3.64
Sub-Saharan Africa	Girls	2026	17	3.72
Sub-Saharan Africa	Girls	2027	17	3.79
Sub-Saharan Africa	Girls	2028	17	3.87
Sub-Saharan Africa	Girls	2029	17	3.94
Sub-Saharan Africa	Girls	2030	17	4.02
Sub-Saharan Africa	Girls	2017	18	3.38
Sub-Saharan Africa	Girls	2018	18	3.47
Sub-Saharan Africa	Girls	2019	18	3.57
Sub-Saharan Africa	Girls	2020	18	3.66
Sub-Saharan Africa	Girls	2021	18	3.76
Sub-Saharan Africa	Girls	2022	18	3.86
Sub-Saharan Africa	Girls	2023	18	3.96
Sub-Saharan Africa	Girls	2024	18	4.07
Sub-Saharan Africa	Girls	2025	18	4.18
Sub-Saharan Africa	Girls	2026	18	4.29
Sub-Saharan Africa	Girls	2027	18	4.40
Sub-Saharan Africa	Girls	2028	18	4.52
Sub-Saharan Africa	Girls	2029	18	4.64
Sub-Saharan Africa	Girls	2030	18	4.76

region	sex	year	age	Prevalence (%)
Afghanistan	Boys	1975	12	3.30
Afghanistan	Boys	1976	12	3.32
Afghanistan	Boys	1977	12	3.34
Afghanistan	Boys	1978	12	3.35
Afghanistan	Boys	1979	12	3.37
Afghanistan	Boys	1980	12	3.39
Afghanistan	Boys	1981	12	3.41
Afghanistan	Boys	1982	12	3.44
Afghanistan	Boys	1983	12	3.46
Afghanistan	Boys	1984	12	3.49
Afghanistan	Boys	1985	12	3.51
Afghanistan	Boys	1986	12	3.54
Afghanistan	Boys	1987	12	3.57
Afghanistan	Boys	1988	12	3.60
Afghanistan	Boys	1989	12	3.63
Afghanistan	Boys	1990	12	3.67
Afghanistan	Boys	1991	12	3.71
Afghanistan	Boys	1992	12	3.75
Afghanistan	Boys	1993	12	3.79
Afghanistan	Boys	1994	12	3.83
Afghanistan	Boys	1995	12	3.88
Afghanistan	Boys	1996	12	3.93
Afghanistan	Boys	1997	12	3.99
Afghanistan	Boys	1998	12	4.05
Afghanistan	Boys	1999	12	4.11
Afghanistan	Boys	2000	12	4.17
Afghanistan	Boys	2001	12	4.24
Afghanistan	Boys	2002	12	4.32
Afghanistan	Boys	2003	12	4.39
Afghanistan	Boys	2004	12	4.48
Afghanistan	Boys	2005	12	4.56
Afghanistan	Boys	2006	12	4.66
Afghanistan	Boys	2007	12	4.75
Afghanistan	Boys	2008	12	4.86
Afghanistan	Boys	2009	12	4.97
Afghanistan	Boys	2010	12	5.08
Afghanistan	Boys	2011	12	5.20
Afghanistan	Boys	2012	12	5.33
Afghanistan	Boys	2013	12	5.47
Afghanistan	Boys	2014	12	5.62
Afghanistan	Boys	2015	12	5.77
Afghanistan	Boys	2016	12	5.93
Afghanistan	Boys	1975	13	4.10
Afghanistan	Boys	1976	13	4.12
Afghanistan	Boys	1977	13	4.14
Afghanistan	Boys	1978	13	4.17
Afghanistan	Boys	1979	13	4.19
Afghanistan	Boys	1980	13	4.21
Afghanistan	Boys	1981	13	4.24
Afghanistan	Boys	1982	13	4.26
Afghanistan	Boys	1983	13	4.29

Afghanistan	Boys	1984	13	4.32
Afghanistan	Boys	1985	13	4.35
Afghanistan	Boys	1986	13	4.39
Afghanistan	Boys	1987	13	4.42
Afghanistan	Boys	1988	13	4.46
Afghanistan	Boys	1989	13	4.50
Afghanistan	Boys	1990	13	4.54
Afghanistan	Boys	1991	13	4.58
Afghanistan	Boys	1992	13	4.63
Afghanistan	Boys	1993	13	4.68
Afghanistan	Boys	1994	13	4.73
Afghanistan	Boys	1995	13	4.79
Afghanistan	Boys	1996	13	4.84
Afghanistan	Boys	1997	13	4.91
Afghanistan	Boys	1998	13	4.97
Afghanistan	Boys	1999	13	5.05
Afghanistan	Boys	2000	13	5.12
Afghanistan	Boys	2001	13	5.20
Afghanistan	Boys	2002	13	5.29
Afghanistan	Boys	2003	13	5.38
Afghanistan	Boys	2004	13	5.47
Afghanistan	Boys	2005	13	5.57
Afghanistan	Boys	2006	13	5.68
Afghanistan	Boys	2007	13	5.79
Afghanistan	Boys	2008	13	5.91
Afghanistan	Boys	2009	13	6.03
Afghanistan	Boys	2010	13	6.17
Afghanistan	Boys	2011	13	6.31
Afghanistan	Boys	2012	13	6.46
Afghanistan	Boys	2013	13	6.62
Afghanistan	Boys	2014	13	6.79
Afghanistan	Boys	2015	13	6.97
Afghanistan	Boys	2016	13	7.15
Afghanistan	Boys	1975	14	5.16
Afghanistan	Boys	1976	14	5.18
Afghanistan	Boys	1977	14	5.21
Afghanistan	Boys	1978	14	5.23
Afghanistan	Boys	1979	14	5.26
Afghanistan	Boys	1980	14	5.29
Afghanistan	Boys	1981	14	5.32
Afghanistan	Boys	1982	14	5.35
Afghanistan	Boys	1983	14	5.39
Afghanistan	Boys	1984	14	5.42
Afghanistan	Boys	1985	14	5.46
Afghanistan	Boys	1986	14	5.49
Afghanistan	Boys	1987	14	5.53
Afghanistan	Boys	1988	14	5.58
Afghanistan	Boys	1989	14	5.62
Afghanistan	Boys	1990	14	5.67
Afghanistan	Boys	1991	14	5.72
Afghanistan	Boys	1992	14	5.77
Afghanistan	Boys	1993	14	5.83

Afghanistan	Boys	1994	14	5.89
Afghanistan	Boys	1995	14	5.95
Afghanistan	Boys	1996	14	6.01
Afghanistan	Boys	1997	14	6.08
Afghanistan	Boys	1998	14	6.16
Afghanistan	Boys	1999	14	6.24
Afghanistan	Boys	2000	14	6.32
Afghanistan	Boys	2001	14	6.41
Afghanistan	Boys	2002	14	6.50
Afghanistan	Boys	2003	14	6.60
Afghanistan	Boys	2004	14	6.71
Afghanistan	Boys	2005	14	6.82
Afghanistan	Boys	2006	14	6.93
Afghanistan	Boys	2007	14	7.06
Afghanistan	Boys	2008	14	7.19
Afghanistan	Boys	2009	14	7.33
Afghanistan	Boys	2010	14	7.47
Afghanistan	Boys	2011	14	7.63
Afghanistan	Boys	2012	14	7.79
Afghanistan	Boys	2013	14	7.97
Afghanistan	Boys	2014	14	8.16
Afghanistan	Boys	2015	14	8.35
Afghanistan	Boys	2016	14	8.56
Afghanistan	Boys	1975	15	6.43
Afghanistan	Boys	1976	15	6.46
Afghanistan	Boys	1977	15	6.49
Afghanistan	Boys	1978	15	6.52
Afghanistan	Boys	1979	15	6.55
Afghanistan	Boys	1980	15	6.59
Afghanistan	Boys	1981	15	6.62
Afghanistan	Boys	1982	15	6.66
Afghanistan	Boys	1983	15	6.70
Afghanistan	Boys	1984	15	6.74
Afghanistan	Boys	1985	15	6.78
Afghanistan	Boys	1986	15	6.82
Afghanistan	Boys	1987	15	6.87
Afghanistan	Boys	1988	15	6.92
Afghanistan	Boys	1989	15	6.97
Afghanistan	Boys	1990	15	7.02
Afghanistan	Boys	1991	15	7.07
Afghanistan	Boys	1992	15	7.13
Afghanistan	Boys	1993	15	7.19
Afghanistan	Boys	1994	15	7.26
Afghanistan	Boys	1995	15	7.33
Afghanistan	Boys	1996	15	7.40
Afghanistan	Boys	1997	15	7.48
Afghanistan	Boys	1998	15	7.56
Afghanistan	Boys	1999	15	7.64
Afghanistan	Boys	2000	15	7.73
Afghanistan	Boys	2001	15	7.83
Afghanistan	Boys	2002	15	7.93
Afghanistan	Boys	2003	15	8.03

Afghanistan	Boys	2004	15	8.14
Afghanistan	Boys	2005	15	8.26
Afghanistan	Boys	2006	15	8.38
Afghanistan	Boys	2007	15	8.52
Afghanistan	Boys	2008	15	8.65
Afghanistan	Boys	2009	15	8.80
Afghanistan	Boys	2010	15	8.96
Afghanistan	Boys	2011	15	9.12
Afghanistan	Boys	2012	15	9.30
Afghanistan	Boys	2013	15	9.48
Afghanistan	Boys	2014	15	9.68
Afghanistan	Boys	2015	15	9.89
Afghanistan	Boys	2016	15	10.11
Afghanistan	Boys	1975	16	7.85
Afghanistan	Boys	1976	16	7.88
Afghanistan	Boys	1977	16	7.92
Afghanistan	Boys	1978	16	7.96
Afghanistan	Boys	1979	16	7.99
Afghanistan	Boys	1980	16	8.03
Afghanistan	Boys	1981	16	8.08
Afghanistan	Boys	1982	16	8.12
Afghanistan	Boys	1983	16	8.16
Afghanistan	Boys	1984	16	8.21
Afghanistan	Boys	1985	16	8.26
Afghanistan	Boys	1986	16	8.30
Afghanistan	Boys	1987	16	8.36
Afghanistan	Boys	1988	16	8.41
Afghanistan	Boys	1989	16	8.46
Afghanistan	Boys	1990	16	8.52
Afghanistan	Boys	1991	16	8.58
Afghanistan	Boys	1992	16	8.65
Afghanistan	Boys	1993	16	8.71
Afghanistan	Boys	1994	16	8.79
Afghanistan	Boys	1995	16	8.86
Afghanistan	Boys	1996	16	8.94
Afghanistan	Boys	1997	16	9.02
Afghanistan	Boys	1998	16	9.10
Afghanistan	Boys	1999	16	9.20
Afghanistan	Boys	2000	16	9.29
Afghanistan	Boys	2001	16	9.39
Afghanistan	Boys	2002	16	9.50
Afghanistan	Boys	2003	16	9.61
Afghanistan	Boys	2004	16	9.73
Afghanistan	Boys	2005	16	9.85
Afghanistan	Boys	2006	16	9.98
Afghanistan	Boys	2007	16	10.12
Afghanistan	Boys	2008	16	10.26
Afghanistan	Boys	2009	16	10.42
Afghanistan	Boys	2010	16	10.58
Afghanistan	Boys	2011	16	10.75
Afghanistan	Boys	2012	16	10.93
Afghanistan	Boys	2013	16	11.12

Afghanistan	Boys	2014	16	11.33
Afghanistan	Boys	2015	16	11.54
Afghanistan	Boys	2016	16	11.77
Afghanistan	Boys	1975	17	9.31
Afghanistan	Boys	1976	17	9.35
Afghanistan	Boys	1977	17	9.39
Afghanistan	Boys	1978	17	9.44
Afghanistan	Boys	1979	17	9.48
Afghanistan	Boys	1980	17	9.53
Afghanistan	Boys	1981	17	9.58
Afghanistan	Boys	1982	17	9.63
Afghanistan	Boys	1983	17	9.68
Afghanistan	Boys	1984	17	9.73
Afghanistan	Boys	1985	17	9.79
Afghanistan	Boys	1986	17	9.84
Afghanistan	Boys	1987	17	9.90
Afghanistan	Boys	1988	17	9.96
Afghanistan	Boys	1989	17	10.02
Afghanistan	Boys	1990	17	10.08
Afghanistan	Boys	1991	17	10.15
Afghanistan	Boys	1992	17	10.22
Afghanistan	Boys	1993	17	10.30
Afghanistan	Boys	1994	17	10.37
Afghanistan	Boys	1995	17	10.45
Afghanistan	Boys	1996	17	10.54
Afghanistan	Boys	1997	17	10.63
Afghanistan	Boys	1998	17	10.72
Afghanistan	Boys	1999	17	10.82
Afghanistan	Boys	2000	17	10.92
Afghanistan	Boys	2001	17	11.03
Afghanistan	Boys	2002	17	11.14
Afghanistan	Boys	2003	17	11.26
Afghanistan	Boys	2004	17	11.39
Afghanistan	Boys	2005	17	11.52
Afghanistan	Boys	2006	17	11.65
Afghanistan	Boys	2007	17	11.80
Afghanistan	Boys	2008	17	11.95
Afghanistan	Boys	2009	17	12.11
Afghanistan	Boys	2010	17	12.28
Afghanistan	Boys	2011	17	12.46
Afghanistan	Boys	2012	17	12.65
Afghanistan	Boys	2013	17	12.85
Afghanistan	Boys	2014	17	13.06
Afghanistan	Boys	2015	17	13.29
Afghanistan	Boys	2016	17	13.53
Afghanistan	Boys	1975	18	10.71
Afghanistan	Boys	1976	18	10.76
Afghanistan	Boys	1977	18	10.81
Afghanistan	Boys	1978	18	10.86
Afghanistan	Boys	1979	18	10.92
Afghanistan	Boys	1980	18	10.97
Afghanistan	Boys	1981	18	11.03

Afghanistan	Boys	1982	18	11.09
Afghanistan	Boys	1983	18	11.14
Afghanistan	Boys	1984	18	11.20
Afghanistan	Boys	1985	18	11.27
Afghanistan	Boys	1986	18	11.33
Afghanistan	Boys	1987	18	11.39
Afghanistan	Boys	1988	18	11.46
Afghanistan	Boys	1989	18	11.53
Afghanistan	Boys	1990	18	11.60
Afghanistan	Boys	1991	18	11.68
Afghanistan	Boys	1992	18	11.76
Afghanistan	Boys	1993	18	11.84
Afghanistan	Boys	1994	18	11.93
Afghanistan	Boys	1995	18	12.02
Afghanistan	Boys	1996	18	12.11
Afghanistan	Boys	1997	18	12.21
Afghanistan	Boys	1998	18	12.31
Afghanistan	Boys	1999	18	12.42
Afghanistan	Boys	2000	18	12.54
Afghanistan	Boys	2001	18	12.66
Afghanistan	Boys	2002	18	12.78
Afghanistan	Boys	2003	18	12.91
Afghanistan	Boys	2004	18	13.05
Afghanistan	Boys	2005	18	13.19
Afghanistan	Boys	2006	18	13.34
Afghanistan	Boys	2007	18	13.50
Afghanistan	Boys	2008	18	13.66
Afghanistan	Boys	2009	18	13.83
Afghanistan	Boys	2010	18	14.02
Afghanistan	Boys	2011	18	14.21
Afghanistan	Boys	2012	18	14.41
Afghanistan	Boys	2013	18	14.63
Afghanistan	Boys	2014	18	14.86
Afghanistan	Boys	2015	18	15.10
Afghanistan	Boys	2016	18	15.35
Afghanistan	Boys	1975	Average	6.69
Afghanistan	Boys	1976	Average	6.73
Afghanistan	Boys	1977	Average	6.76
Afghanistan	Boys	1978	Average	6.79
Afghanistan	Boys	1979	Average	6.82
Afghanistan	Boys	1980	Average	6.86
Afghanistan	Boys	1981	Average	6.90
Afghanistan	Boys	1982	Average	6.94
Afghanistan	Boys	1983	Average	6.98
Afghanistan	Boys	1984	Average	7.02
Afghanistan	Boys	1985	Average	7.06
Afghanistan	Boys	1986	Average	7.10
Afghanistan	Boys	1987	Average	7.15
Afghanistan	Boys	1988	Average	7.20
Afghanistan	Boys	1989	Average	7.25
Afghanistan	Boys	1990	Average	7.30
Afghanistan	Boys	1991	Average	7.36

Afghanistan	Boys	1992	Average	7.42
Afghanistan	Boys	1993	Average	7.48
Afghanistan	Boys	1994	Average	7.54
Afghanistan	Boys	1995	Average	7.61
Afghanistan	Boys	1996	Average	7.68
Afghanistan	Boys	1997	Average	7.76
Afghanistan	Boys	1998	Average	7.84
Afghanistan	Boys	1999	Average	7.92
Afghanistan	Boys	2000	Average	8.01
Afghanistan	Boys	2001	Average	8.11
Afghanistan	Boys	2002	Average	8.21
Afghanistan	Boys	2003	Average	8.31
Afghanistan	Boys	2004	Average	8.42
Afghanistan	Boys	2005	Average	8.54
Afghanistan	Boys	2006	Average	8.66
Afghanistan	Boys	2007	Average	8.79
Afghanistan	Boys	2008	Average	8.93
Afghanistan	Boys	2009	Average	9.07
Afghanistan	Boys	2010	Average	9.22
Afghanistan	Boys	2011	Average	9.38
Afghanistan	Boys	2012	Average	9.55
Afghanistan	Boys	2013	Average	9.74
Afghanistan	Boys	2014	Average	9.93
Afghanistan	Boys	2015	Average	10.13
Afghanistan	Boys	2016	Average	10.34
Afghanistan	Girls	1975	12	3.88
Afghanistan	Girls	1976	12	3.90
Afghanistan	Girls	1977	12	3.91
Afghanistan	Girls	1978	12	3.93
Afghanistan	Girls	1979	12	3.95
Afghanistan	Girls	1980	12	3.97
Afghanistan	Girls	1981	12	3.99
Afghanistan	Girls	1982	12	4.00
Afghanistan	Girls	1983	12	4.03
Afghanistan	Girls	1984	12	4.05
Afghanistan	Girls	1985	12	4.07
Afghanistan	Girls	1986	12	4.09
Afghanistan	Girls	1987	12	4.12
Afghanistan	Girls	1988	12	4.14
Afghanistan	Girls	1989	12	4.17
Afghanistan	Girls	1990	12	4.20
Afghanistan	Girls	1991	12	4.23
Afghanistan	Girls	1992	12	4.26
Afghanistan	Girls	1993	12	4.30
Afghanistan	Girls	1994	12	4.34
Afghanistan	Girls	1995	12	4.38
Afghanistan	Girls	1996	12	4.42
Afghanistan	Girls	1997	12	4.46
Afghanistan	Girls	1998	12	4.51
Afghanistan	Girls	1999	12	4.56
Afghanistan	Girls	2000	12	4.62
Afghanistan	Girls	2001	12	4.68

Afghanistan	Girls	2002	12	4.74
Afghanistan	Girls	2003	12	4.81
Afghanistan	Girls	2004	12	4.88
Afghanistan	Girls	2005	12	4.96
Afghanistan	Girls	2006	12	5.04
Afghanistan	Girls	2007	12	5.12
Afghanistan	Girls	2008	12	5.22
Afghanistan	Girls	2009	12	5.31
Afghanistan	Girls	2010	12	5.42
Afghanistan	Girls	2011	12	5.52
Afghanistan	Girls	2012	12	5.64
Afghanistan	Girls	2013	12	5.76
Afghanistan	Girls	2014	12	5.89
Afghanistan	Girls	2015	12	6.03
Afghanistan	Girls	2016	12	6.18
Afghanistan	Girls	1975	13	4.91
Afghanistan	Girls	1976	13	4.93
Afghanistan	Girls	1977	13	4.95
Afghanistan	Girls	1978	13	4.97
Afghanistan	Girls	1979	13	4.99
Afghanistan	Girls	1980	13	5.01
Afghanistan	Girls	1981	13	5.04
Afghanistan	Girls	1982	13	5.06
Afghanistan	Girls	1983	13	5.09
Afghanistan	Girls	1984	13	5.11
Afghanistan	Girls	1985	13	5.14
Afghanistan	Girls	1986	13	5.17
Afghanistan	Girls	1987	13	5.20
Afghanistan	Girls	1988	13	5.23
Afghanistan	Girls	1989	13	5.27
Afghanistan	Girls	1990	13	5.30
Afghanistan	Girls	1991	13	5.34
Afghanistan	Girls	1992	13	5.38
Afghanistan	Girls	1993	13	5.42
Afghanistan	Girls	1994	13	5.46
Afghanistan	Girls	1995	13	5.51
Afghanistan	Girls	1996	13	5.56
Afghanistan	Girls	1997	13	5.61
Afghanistan	Girls	1998	13	5.67
Afghanistan	Girls	1999	13	5.73
Afghanistan	Girls	2000	13	5.80
Afghanistan	Girls	2001	13	5.87
Afghanistan	Girls	2002	13	5.94
Afghanistan	Girls	2003	13	6.02
Afghanistan	Girls	2004	13	6.10
Afghanistan	Girls	2005	13	6.19
Afghanistan	Girls	2006	13	6.28
Afghanistan	Girls	2007	13	6.38
Afghanistan	Girls	2008	13	6.49
Afghanistan	Girls	2009	13	6.60
Afghanistan	Girls	2010	13	6.72
Afghanistan	Girls	2011	13	6.84

Afghanistan	Girls	2012	13	6.97
Afghanistan	Girls	2013	13	7.12
Afghanistan	Girls	2014	13	7.26
Afghanistan	Girls	2015	13	7.42
Afghanistan	Girls	2016	13	7.59
Afghanistan	Girls	1975	14	6.09
Afghanistan	Girls	1976	14	6.11
Afghanistan	Girls	1977	14	6.14
Afghanistan	Girls	1978	14	6.16
Afghanistan	Girls	1979	14	6.19
Afghanistan	Girls	1980	14	6.22
Afghanistan	Girls	1981	14	6.25
Afghanistan	Girls	1982	14	6.28
Afghanistan	Girls	1983	14	6.31
Afghanistan	Girls	1984	14	6.34
Afghanistan	Girls	1985	14	6.37
Afghanistan	Girls	1986	14	6.40
Afghanistan	Girls	1987	14	6.44
Afghanistan	Girls	1988	14	6.48
Afghanistan	Girls	1989	14	6.51
Afghanistan	Girls	1990	14	6.55
Afghanistan	Girls	1991	14	6.60
Afghanistan	Girls	1992	14	6.64
Afghanistan	Girls	1993	14	6.69
Afghanistan	Girls	1994	14	6.74
Afghanistan	Girls	1995	14	6.79
Afghanistan	Girls	1996	14	6.85
Afghanistan	Girls	1997	14	6.91
Afghanistan	Girls	1998	14	6.98
Afghanistan	Girls	1999	14	7.05
Afghanistan	Girls	2000	14	7.12
Afghanistan	Girls	2001	14	7.20
Afghanistan	Girls	2002	14	7.28
Afghanistan	Girls	2003	14	7.36
Afghanistan	Girls	2004	14	7.46
Afghanistan	Girls	2005	14	7.55
Afghanistan	Girls	2006	14	7.66
Afghanistan	Girls	2007	14	7.77
Afghanistan	Girls	2008	14	7.88
Afghanistan	Girls	2009	14	8.00
Afghanistan	Girls	2010	14	8.13
Afghanistan	Girls	2011	14	8.27
Afghanistan	Girls	2012	14	8.41
Afghanistan	Girls	2013	14	8.57
Afghanistan	Girls	2014	14	8.73
Afghanistan	Girls	2015	14	8.90
Afghanistan	Girls	2016	14	9.08
Afghanistan	Girls	1975	15	7.25
Afghanistan	Girls	1976	15	7.28
Afghanistan	Girls	1977	15	7.31
Afghanistan	Girls	1978	15	7.34
Afghanistan	Girls	1979	15	7.37

Afghanistan	Girls	1980	15	7.40
Afghanistan	Girls	1981	15	7.44
Afghanistan	Girls	1982	15	7.47
Afghanistan	Girls	1983	15	7.51
Afghanistan	Girls	1984	15	7.54
Afghanistan	Girls	1985	15	7.58
Afghanistan	Girls	1986	15	7.61
Afghanistan	Girls	1987	15	7.65
Afghanistan	Girls	1988	15	7.69
Afghanistan	Girls	1989	15	7.74
Afghanistan	Girls	1990	15	7.78
Afghanistan	Girls	1991	15	7.83
Afghanistan	Girls	1992	15	7.88
Afghanistan	Girls	1993	15	7.93
Afghanistan	Girls	1994	15	7.99
Afghanistan	Girls	1995	15	8.04
Afghanistan	Girls	1996	15	8.11
Afghanistan	Girls	1997	15	8.17
Afghanistan	Girls	1998	15	8.24
Afghanistan	Girls	1999	15	8.32
Afghanistan	Girls	2000	15	8.40
Afghanistan	Girls	2001	15	8.48
Afghanistan	Girls	2002	15	8.57
Afghanistan	Girls	2003	15	8.66
Afghanistan	Girls	2004	15	8.76
Afghanistan	Girls	2005	15	8.86
Afghanistan	Girls	2006	15	8.97
Afghanistan	Girls	2007	15	9.09
Afghanistan	Girls	2008	15	9.21
Afghanistan	Girls	2009	15	9.34
Afghanistan	Girls	2010	15	9.48
Afghanistan	Girls	2011	15	9.62
Afghanistan	Girls	2012	15	9.78
Afghanistan	Girls	2013	15	9.94
Afghanistan	Girls	2014	15	10.11
Afghanistan	Girls	2015	15	10.29
Afghanistan	Girls	2016	15	10.48
Afghanistan	Girls	1975	16	8.22
Afghanistan	Girls	1976	16	8.25
Afghanistan	Girls	1977	16	8.29
Afghanistan	Girls	1978	16	8.32
Afghanistan	Girls	1979	16	8.36
Afghanistan	Girls	1980	16	8.39
Afghanistan	Girls	1981	16	8.43
Afghanistan	Girls	1982	16	8.46
Afghanistan	Girls	1983	16	8.50
Afghanistan	Girls	1984	16	8.54
Afghanistan	Girls	1985	16	8.58
Afghanistan	Girls	1986	16	8.62
Afghanistan	Girls	1987	16	8.66
Afghanistan	Girls	1988	16	8.71
Afghanistan	Girls	1989	16	8.75

Afghanistan	Girls	1990	16	8.80
Afghanistan	Girls	1991	16	8.85
Afghanistan	Girls	1992	16	8.90
Afghanistan	Girls	1993	16	8.96
Afghanistan	Girls	1994	16	9.02
Afghanistan	Girls	1995	16	9.08
Afghanistan	Girls	1996	16	9.15
Afghanistan	Girls	1997	16	9.22
Afghanistan	Girls	1998	16	9.29
Afghanistan	Girls	1999	16	9.37
Afghanistan	Girls	2000	16	9.45
Afghanistan	Girls	2001	16	9.54
Afghanistan	Girls	2002	16	9.63
Afghanistan	Girls	2003	16	9.73
Afghanistan	Girls	2004	16	9.84
Afghanistan	Girls	2005	16	9.95
Afghanistan	Girls	2006	16	10.06
Afghanistan	Girls	2007	16	10.18
Afghanistan	Girls	2008	16	10.31
Afghanistan	Girls	2009	16	10.45
Afghanistan	Girls	2010	16	10.59
Afghanistan	Girls	2011	16	10.74
Afghanistan	Girls	2012	16	10.90
Afghanistan	Girls	2013	16	11.07
Afghanistan	Girls	2014	16	11.25
Afghanistan	Girls	2015	16	11.44
Afghanistan	Girls	2016	16	11.64
Afghanistan	Girls	1975	17	8.89
Afghanistan	Girls	1976	17	8.93
Afghanistan	Girls	1977	17	8.96
Afghanistan	Girls	1978	17	9.00
Afghanistan	Girls	1979	17	9.04
Afghanistan	Girls	1980	17	9.07
Afghanistan	Girls	1981	17	9.11
Afghanistan	Girls	1982	17	9.15
Afghanistan	Girls	1983	17	9.19
Afghanistan	Girls	1984	17	9.24
Afghanistan	Girls	1985	17	9.28
Afghanistan	Girls	1986	17	9.32
Afghanistan	Girls	1987	17	9.37
Afghanistan	Girls	1988	17	9.41
Afghanistan	Girls	1989	17	9.46
Afghanistan	Girls	1990	17	9.51
Afghanistan	Girls	1991	17	9.57
Afghanistan	Girls	1992	17	9.62
Afghanistan	Girls	1993	17	9.68
Afghanistan	Girls	1994	17	9.75
Afghanistan	Girls	1995	17	9.81
Afghanistan	Girls	1996	17	9.88
Afghanistan	Girls	1997	17	9.96
Afghanistan	Girls	1998	17	10.04
Afghanistan	Girls	1999	17	10.12

Afghanistan	Girls	2000	17	10.21
Afghanistan	Girls	2001	17	10.30
Afghanistan	Girls	2002	17	10.40
Afghanistan	Girls	2003	17	10.51
Afghanistan	Girls	2004	17	10.62
Afghanistan	Girls	2005	17	10.73
Afghanistan	Girls	2006	17	10.86
Afghanistan	Girls	2007	17	10.99
Afghanistan	Girls	2008	17	11.12
Afghanistan	Girls	2009	17	11.27
Afghanistan	Girls	2010	17	11.42
Afghanistan	Girls	2011	17	11.58
Afghanistan	Girls	2012	17	11.75
Afghanistan	Girls	2013	17	11.93
Afghanistan	Girls	2014	17	12.12
Afghanistan	Girls	2015	17	12.32
Afghanistan	Girls	2016	17	12.54
Afghanistan	Girls	1975	18	9.26
Afghanistan	Girls	1976	18	9.30
Afghanistan	Girls	1977	18	9.34
Afghanistan	Girls	1978	18	9.38
Afghanistan	Girls	1979	18	9.42
Afghanistan	Girls	1980	18	9.46
Afghanistan	Girls	1981	18	9.50
Afghanistan	Girls	1982	18	9.54
Afghanistan	Girls	1983	18	9.59
Afghanistan	Girls	1984	18	9.63
Afghanistan	Girls	1985	18	9.68
Afghanistan	Girls	1986	18	9.73
Afghanistan	Girls	1987	18	9.77
Afghanistan	Girls	1988	18	9.83
Afghanistan	Girls	1989	18	9.88
Afghanistan	Girls	1990	18	9.93
Afghanistan	Girls	1991	18	9.99
Afghanistan	Girls	1992	18	10.06
Afghanistan	Girls	1993	18	10.12
Afghanistan	Girls	1994	18	10.19
Afghanistan	Girls	1995	18	10.26
Afghanistan	Girls	1996	18	10.34
Afghanistan	Girls	1997	18	10.42
Afghanistan	Girls	1998	18	10.51
Afghanistan	Girls	1999	18	10.60
Afghanistan	Girls	2000	18	10.70
Afghanistan	Girls	2001	18	10.81
Afghanistan	Girls	2002	18	10.92
Afghanistan	Girls	2003	18	11.03
Afghanistan	Girls	2004	18	11.15
Afghanistan	Girls	2005	18	11.28
Afghanistan	Girls	2006	18	11.42
Afghanistan	Girls	2007	18	11.56
Afghanistan	Girls	2008	18	11.72
Afghanistan	Girls	2009	18	11.88

Afghanistan	Girls	2010	18	12.05
Afghanistan	Girls	2011	18	12.23
Afghanistan	Girls	2012	18	12.42
Afghanistan	Girls	2013	18	12.62
Afghanistan	Girls	2014	18	12.83
Afghanistan	Girls	2015	18	13.06
Afghanistan	Girls	2016	18	13.29
Afghanistan	Girls	1975	Average	6.93
Afghanistan	Girls	1976	Average	6.96
Afghanistan	Girls	1977	Average	6.99
Afghanistan	Girls	1978	Average	7.01
Afghanistan	Girls	1979	Average	7.04
Afghanistan	Girls	1980	Average	7.08
Afghanistan	Girls	1981	Average	7.11
Afghanistan	Girls	1982	Average	7.14
Afghanistan	Girls	1983	Average	7.17
Afghanistan	Girls	1984	Average	7.21
Afghanistan	Girls	1985	Average	7.24
Afghanistan	Girls	1986	Average	7.28
Afghanistan	Girls	1987	Average	7.32
Afghanistan	Girls	1988	Average	7.36
Afghanistan	Girls	1989	Average	7.40
Afghanistan	Girls	1990	Average	7.44
Afghanistan	Girls	1991	Average	7.49
Afghanistan	Girls	1992	Average	7.53
Afghanistan	Girls	1993	Average	7.59
Afghanistan	Girls	1994	Average	7.64
Afghanistan	Girls	1995	Average	7.70
Afghanistan	Girls	1996	Average	7.76
Afghanistan	Girls	1997	Average	7.82
Afghanistan	Girls	1998	Average	7.89
Afghanistan	Girls	1999	Average	7.96
Afghanistan	Girls	2000	Average	8.04
Afghanistan	Girls	2001	Average	8.12
Afghanistan	Girls	2002	Average	8.21
Afghanistan	Girls	2003	Average	8.30
Afghanistan	Girls	2004	Average	8.40
Afghanistan	Girls	2005	Average	8.50
Afghanistan	Girls	2006	Average	8.61
Afghanistan	Girls	2007	Average	8.73
Afghanistan	Girls	2008	Average	8.85
Afghanistan	Girls	2009	Average	8.98
Afghanistan	Girls	2010	Average	9.11
Afghanistan	Girls	2011	Average	9.26
Afghanistan	Girls	2012	Average	9.41
Afghanistan	Girls	2013	Average	9.57
Afghanistan	Girls	2014	Average	9.74
Afghanistan	Girls	2015	Average	9.92
Afghanistan	Girls	2016	Average	10.11
Albania	Boys	1975	12	5.71
Albania	Boys	1976	12	5.75
Albania	Boys	1977	12	5.80

Albania	Boys	1978	12	5.85
Albania	Boys	1979	12	5.90
Albania	Boys	1980	12	5.96
Albania	Boys	1981	12	6.02
Albania	Boys	1982	12	6.08
Albania	Boys	1983	12	6.15
Albania	Boys	1984	12	6.22
Albania	Boys	1985	12	6.29
Albania	Boys	1986	12	6.37
Albania	Boys	1987	12	6.45
Albania	Boys	1988	12	6.54
Albania	Boys	1989	12	6.64
Albania	Boys	1990	12	6.73
Albania	Boys	1991	12	6.84
Albania	Boys	1992	12	6.95
Albania	Boys	1993	12	7.07
Albania	Boys	1994	12	7.19
Albania	Boys	1995	12	7.32
Albania	Boys	1996	12	7.46
Albania	Boys	1997	12	7.61
Albania	Boys	1998	12	7.77
Albania	Boys	1999	12	7.93
Albania	Boys	2000	12	8.11
Albania	Boys	2001	12	8.29
Albania	Boys	2002	12	8.49
Albania	Boys	2003	12	8.70
Albania	Boys	2004	12	8.92
Albania	Boys	2005	12	9.15
Albania	Boys	2006	12	9.39
Albania	Boys	2007	12	9.64
Albania	Boys	2008	12	9.91
Albania	Boys	2009	12	10.18
Albania	Boys	2010	12	10.46
Albania	Boys	2011	12	10.74
Albania	Boys	2012	12	11.03
Albania	Boys	2013	12	11.33
Albania	Boys	2014	12	11.62
Albania	Boys	2015	12	11.93
Albania	Boys	2016	12	12.23
Albania	Boys	1975	13	7.13
Albania	Boys	1976	13	7.18
Albania	Boys	1977	13	7.23
Albania	Boys	1978	13	7.29
Albania	Boys	1979	13	7.35
Albania	Boys	1980	13	7.42
Albania	Boys	1981	13	7.49
Albania	Boys	1982	13	7.56
Albania	Boys	1983	13	7.64
Albania	Boys	1984	13	7.72
Albania	Boys	1985	13	7.81
Albania	Boys	1986	13	7.90
Albania	Boys	1987	13	8.00

Albania	Boys	1988	13	8.11
Albania	Boys	1989	13	8.21
Albania	Boys	1990	13	8.33
Albania	Boys	1991	13	8.45
Albania	Boys	1992	13	8.58
Albania	Boys	1993	13	8.72
Albania	Boys	1994	13	8.86
Albania	Boys	1995	13	9.02
Albania	Boys	1996	13	9.18
Albania	Boys	1997	13	9.36
Albania	Boys	1998	13	9.54
Albania	Boys	1999	13	9.73
Albania	Boys	2000	13	9.93
Albania	Boys	2001	13	10.15
Albania	Boys	2002	13	10.38
Albania	Boys	2003	13	10.63
Albania	Boys	2004	13	10.89
Albania	Boys	2005	13	11.16
Albania	Boys	2006	13	11.45
Albania	Boys	2007	13	11.75
Albania	Boys	2008	13	12.06
Albania	Boys	2009	13	12.38
Albania	Boys	2010	13	12.72
Albania	Boys	2011	13	13.06
Albania	Boys	2012	13	13.41
Albania	Boys	2013	13	13.76
Albania	Boys	2014	13	14.13
Albania	Boys	2015	13	14.50
Albania	Boys	2016	13	14.87
Albania	Boys	1975	14	8.91
Albania	Boys	1976	14	8.96
Albania	Boys	1977	14	9.03
Albania	Boys	1978	14	9.09
Albania	Boys	1979	14	9.16
Albania	Boys	1980	14	9.23
Albania	Boys	1981	14	9.31
Albania	Boys	1982	14	9.40
Albania	Boys	1983	14	9.48
Albania	Boys	1984	14	9.58
Albania	Boys	1985	14	9.67
Albania	Boys	1986	14	9.78
Albania	Boys	1987	14	9.89
Albania	Boys	1988	14	10.00
Albania	Boys	1989	14	10.12
Albania	Boys	1990	14	10.25
Albania	Boys	1991	14	10.38
Albania	Boys	1992	14	10.53
Albania	Boys	1993	14	10.68
Albania	Boys	1994	14	10.84
Albania	Boys	1995	14	11.01
Albania	Boys	1996	14	11.20
Albania	Boys	1997	14	11.39

Albania	Boys	1998	14	11.59
Albania	Boys	1999	14	11.81
Albania	Boys	2000	14	12.03
Albania	Boys	2001	14	12.27
Albania	Boys	2002	14	12.53
Albania	Boys	2003	14	12.80
Albania	Boys	2004	14	13.09
Albania	Boys	2005	14	13.39
Albania	Boys	2006	14	13.71
Albania	Boys	2007	14	14.05
Albania	Boys	2008	14	14.40
Albania	Boys	2009	14	14.76
Albania	Boys	2010	14	15.14
Albania	Boys	2011	14	15.53
Albania	Boys	2012	14	15.92
Albania	Boys	2013	14	16.33
Albania	Boys	2014	14	16.74
Albania	Boys	2015	14	17.16
Albania	Boys	2016	14	17.59
Albania	Boys	1975	15	10.97
Albania	Boys	1976	15	11.03
Albania	Boys	1977	15	11.10
Albania	Boys	1978	15	11.17
Albania	Boys	1979	15	11.25
Albania	Boys	1980	15	11.33
Albania	Boys	1981	15	11.41
Albania	Boys	1982	15	11.50
Albania	Boys	1983	15	11.59
Albania	Boys	1984	15	11.69
Albania	Boys	1985	15	11.80
Albania	Boys	1986	15	11.90
Albania	Boys	1987	15	12.02
Albania	Boys	1988	15	12.14
Albania	Boys	1989	15	12.27
Albania	Boys	1990	15	12.40
Albania	Boys	1991	15	12.55
Albania	Boys	1992	15	12.70
Albania	Boys	1993	15	12.86
Albania	Boys	1994	15	13.03
Albania	Boys	1995	15	13.21
Albania	Boys	1996	15	13.40
Albania	Boys	1997	15	13.61
Albania	Boys	1998	15	13.82
Albania	Boys	1999	15	14.05
Albania	Boys	2000	15	14.28
Albania	Boys	2001	15	14.54
Albania	Boys	2002	15	14.81
Albania	Boys	2003	15	15.10
Albania	Boys	2004	15	15.40
Albania	Boys	2005	15	15.73
Albania	Boys	2006	15	16.07
Albania	Boys	2007	15	16.42

Albania	Boys	2008	15	16.79
Albania	Boys	2009	15	17.18
Albania	Boys	2010	15	17.58
Albania	Boys	2011	15	18.00
Albania	Boys	2012	15	18.42
Albania	Boys	2013	15	18.86
Albania	Boys	2014	15	19.30
Albania	Boys	2015	15	19.76
Albania	Boys	2016	15	20.22
Albania	Boys	1975	16	13.22
Albania	Boys	1976	16	13.29
Albania	Boys	1977	16	13.36
Albania	Boys	1978	16	13.44
Albania	Boys	1979	16	13.52
Albania	Boys	1980	16	13.60
Albania	Boys	1981	16	13.69
Albania	Boys	1982	16	13.78
Albania	Boys	1983	16	13.88
Albania	Boys	1984	16	13.98
Albania	Boys	1985	16	14.09
Albania	Boys	1986	16	14.20
Albania	Boys	1987	16	14.32
Albania	Boys	1988	16	14.44
Albania	Boys	1989	16	14.57
Albania	Boys	1990	16	14.71
Albania	Boys	1991	16	14.86
Albania	Boys	1992	16	15.01
Albania	Boys	1993	16	15.18
Albania	Boys	1994	16	15.35
Albania	Boys	1995	16	15.54
Albania	Boys	1996	16	15.74
Albania	Boys	1997	16	15.95
Albania	Boys	1998	16	16.16
Albania	Boys	1999	16	16.40
Albania	Boys	2000	16	16.64
Albania	Boys	2001	16	16.90
Albania	Boys	2002	16	17.18
Albania	Boys	2003	16	17.47
Albania	Boys	2004	16	17.78
Albania	Boys	2005	16	18.11
Albania	Boys	2006	16	18.46
Albania	Boys	2007	16	18.83
Albania	Boys	2008	16	19.21
Albania	Boys	2009	16	19.61
Albania	Boys	2010	16	20.03
Albania	Boys	2011	16	20.45
Albania	Boys	2012	16	20.89
Albania	Boys	2013	16	21.34
Albania	Boys	2014	16	21.80
Albania	Boys	2015	16	22.28
Albania	Boys	2016	16	22.76
Albania	Boys	1975	17	15.56

Albania	Boys	1976	17	15.63
Albania	Boys	1977	17	15.71
Albania	Boys	1978	17	15.78
Albania	Boys	1979	17	15.87
Albania	Boys	1980	17	15.95
Albania	Boys	1981	17	16.04
Albania	Boys	1982	17	16.14
Albania	Boys	1983	17	16.24
Albania	Boys	1984	17	16.34
Albania	Boys	1985	17	16.45
Albania	Boys	1986	17	16.57
Albania	Boys	1987	17	16.69
Albania	Boys	1988	17	16.82
Albania	Boys	1989	17	16.95
Albania	Boys	1990	17	17.09
Albania	Boys	1991	17	17.24
Albania	Boys	1992	17	17.40
Albania	Boys	1993	17	17.57
Albania	Boys	1994	17	17.75
Albania	Boys	1995	17	17.94
Albania	Boys	1996	17	18.14
Albania	Boys	1997	17	18.35
Albania	Boys	1998	17	18.57
Albania	Boys	1999	17	18.81
Albania	Boys	2000	17	19.05
Albania	Boys	2001	17	19.31
Albania	Boys	2002	17	19.60
Albania	Boys	2003	17	19.90
Albania	Boys	2004	17	20.22
Albania	Boys	2005	17	20.55
Albania	Boys	2006	17	20.91
Albania	Boys	2007	17	21.28
Albania	Boys	2008	17	21.67
Albania	Boys	2009	17	22.07
Albania	Boys	2010	17	22.49
Albania	Boys	2011	17	22.93
Albania	Boys	2012	17	23.38
Albania	Boys	2013	17	23.84
Albania	Boys	2014	17	24.31
Albania	Boys	2015	17	24.79
Albania	Boys	2016	17	25.29
Albania	Boys	1975	18	17.90
Albania	Boys	1976	18	17.97
Albania	Boys	1977	18	18.05
Albania	Boys	1978	18	18.13
Albania	Boys	1979	18	18.22
Albania	Boys	1980	18	18.31
Albania	Boys	1981	18	18.41
Albania	Boys	1982	18	18.51
Albania	Boys	1983	18	18.61
Albania	Boys	1984	18	18.72
Albania	Boys	1985	18	18.84

Albania	Boys	1986	18	18.96
Albania	Boys	1987	18	19.09
Albania	Boys	1988	18	19.22
Albania	Boys	1989	18	19.36
Albania	Boys	1990	18	19.51
Albania	Boys	1991	18	19.66
Albania	Boys	1992	18	19.83
Albania	Boys	1993	18	20.00
Albania	Boys	1994	18	20.19
Albania	Boys	1995	18	20.38
Albania	Boys	1996	18	20.59
Albania	Boys	1997	18	20.81
Albania	Boys	1998	18	21.04
Albania	Boys	1999	18	21.28
Albania	Boys	2000	18	21.53
Albania	Boys	2001	18	21.81
Albania	Boys	2002	18	22.10
Albania	Boys	2003	18	22.41
Albania	Boys	2004	18	22.74
Albania	Boys	2005	18	23.08
Albania	Boys	2006	18	23.45
Albania	Boys	2007	18	23.83
Albania	Boys	2008	18	24.23
Albania	Boys	2009	18	24.65
Albania	Boys	2010	18	25.08
Albania	Boys	2011	18	25.53
Albania	Boys	2012	18	25.99
Albania	Boys	2013	18	26.46
Albania	Boys	2014	18	26.95
Albania	Boys	2015	18	27.44
Albania	Boys	2016	18	27.95
Albania	Boys	1975	Average	11.34
Albania	Boys	1976	Average	11.40
Albania	Boys	1977	Average	11.47
Albania	Boys	1978	Average	11.54
Albania	Boys	1979	Average	11.61
Albania	Boys	1980	Average	11.69
Albania	Boys	1981	Average	11.77
Albania	Boys	1982	Average	11.85
Albania	Boys	1983	Average	11.94
Albania	Boys	1984	Average	12.04
Albania	Boys	1985	Average	12.14
Albania	Boys	1986	Average	12.24
Albania	Boys	1987	Average	12.35
Albania	Boys	1988	Average	12.47
Albania	Boys	1989	Average	12.59
Albania	Boys	1990	Average	12.72
Albania	Boys	1991	Average	12.85
Albania	Boys	1992	Average	13.00
Albania	Boys	1993	Average	13.15
Albania	Boys	1994	Average	13.32
Albania	Boys	1995	Average	13.49

Albania	Boys	1996	Average	13.67
Albania	Boys	1997	Average	13.87
Albania	Boys	1998	Average	14.07
Albania	Boys	1999	Average	14.29
Albania	Boys	2000	Average	14.51
Albania	Boys	2001	Average	14.75
Albania	Boys	2002	Average	15.01
Albania	Boys	2003	Average	15.29
Albania	Boys	2004	Average	15.58
Albania	Boys	2005	Average	15.88
Albania	Boys	2006	Average	16.20
Albania	Boys	2007	Average	16.54
Albania	Boys	2008	Average	16.90
Albania	Boys	2009	Average	17.26
Albania	Boys	2010	Average	17.64
Albania	Boys	2011	Average	18.03
Albania	Boys	2012	Average	18.43
Albania	Boys	2013	Average	18.84
Albania	Boys	2014	Average	19.27
Albania	Boys	2015	Average	19.69
Albania	Boys	2016	Average	20.13
Albania	Girls	1975	12	5.40
Albania	Girls	1976	12	5.43
Albania	Girls	1977	12	5.46
Albania	Girls	1978	12	5.50
Albania	Girls	1979	12	5.53
Albania	Girls	1980	12	5.57
Albania	Girls	1981	12	5.61
Albania	Girls	1982	12	5.66
Albania	Girls	1983	12	5.70
Albania	Girls	1984	12	5.75
Albania	Girls	1985	12	5.80
Albania	Girls	1986	12	5.85
Albania	Girls	1987	12	5.90
Albania	Girls	1988	12	5.95
Albania	Girls	1989	12	6.01
Albania	Girls	1990	12	6.07
Albania	Girls	1991	12	6.14
Albania	Girls	1992	12	6.20
Albania	Girls	1993	12	6.28
Albania	Girls	1994	12	6.35
Albania	Girls	1995	12	6.43
Albania	Girls	1996	12	6.51
Albania	Girls	1997	12	6.60
Albania	Girls	1998	12	6.70
Albania	Girls	1999	12	6.79
Albania	Girls	2000	12	6.90
Albania	Girls	2001	12	7.01
Albania	Girls	2002	12	7.12
Albania	Girls	2003	12	7.25
Albania	Girls	2004	12	7.38
Albania	Girls	2005	12	7.51

Albania	Girls	2006	12	7.65
Albania	Girls	2007	12	7.80
Albania	Girls	2008	12	7.95
Albania	Girls	2009	12	8.11
Albania	Girls	2010	12	8.27
Albania	Girls	2011	12	8.43
Albania	Girls	2012	12	8.60
Albania	Girls	2013	12	8.77
Albania	Girls	2014	12	8.94
Albania	Girls	2015	12	9.11
Albania	Girls	2016	12	9.29
Albania	Girls	1975	13	6.74
Albania	Girls	1976	13	6.78
Albania	Girls	1977	13	6.82
Albania	Girls	1978	13	6.86
Albania	Girls	1979	13	6.91
Albania	Girls	1980	13	6.95
Albania	Girls	1981	13	7.00
Albania	Girls	1982	13	7.05
Albania	Girls	1983	13	7.10
Albania	Girls	1984	13	7.16
Albania	Girls	1985	13	7.21
Albania	Girls	1986	13	7.27
Albania	Girls	1987	13	7.33
Albania	Girls	1988	13	7.39
Albania	Girls	1989	13	7.46
Albania	Girls	1990	13	7.53
Albania	Girls	1991	13	7.60
Albania	Girls	1992	13	7.68
Albania	Girls	1993	13	7.76
Albania	Girls	1994	13	7.85
Albania	Girls	1995	13	7.94
Albania	Girls	1996	13	8.04
Albania	Girls	1997	13	8.14
Albania	Girls	1998	13	8.24
Albania	Girls	1999	13	8.35
Albania	Girls	2000	13	8.47
Albania	Girls	2001	13	8.59
Albania	Girls	2002	13	8.72
Albania	Girls	2003	13	8.86
Albania	Girls	2004	13	9.01
Albania	Girls	2005	13	9.16
Albania	Girls	2006	13	9.32
Albania	Girls	2007	13	9.49
Albania	Girls	2008	13	9.66
Albania	Girls	2009	13	9.84
Albania	Girls	2010	13	10.02
Albania	Girls	2011	13	10.21
Albania	Girls	2012	13	10.40
Albania	Girls	2013	13	10.59
Albania	Girls	2014	13	10.78
Albania	Girls	2015	13	10.98

Albania	Girls	2016	13	11.18
Albania	Girls	1975	14	8.21
Albania	Girls	1976	14	8.26
Albania	Girls	1977	14	8.30
Albania	Girls	1978	14	8.35
Albania	Girls	1979	14	8.40
Albania	Girls	1980	14	8.45
Albania	Girls	1981	14	8.50
Albania	Girls	1982	14	8.55
Albania	Girls	1983	14	8.61
Albania	Girls	1984	14	8.67
Albania	Girls	1985	14	8.73
Albania	Girls	1986	14	8.80
Albania	Girls	1987	14	8.86
Albania	Girls	1988	14	8.93
Albania	Girls	1989	14	9.00
Albania	Girls	1990	14	9.08
Albania	Girls	1991	14	9.16
Albania	Girls	1992	14	9.24
Albania	Girls	1993	14	9.33
Albania	Girls	1994	14	9.43
Albania	Girls	1995	14	9.52
Albania	Girls	1996	14	9.63
Albania	Girls	1997	14	9.74
Albania	Girls	1998	14	9.85
Albania	Girls	1999	14	9.97
Albania	Girls	2000	14	10.10
Albania	Girls	2001	14	10.23
Albania	Girls	2002	14	10.37
Albania	Girls	2003	14	10.52
Albania	Girls	2004	14	10.68
Albania	Girls	2005	14	10.84
Albania	Girls	2006	14	11.02
Albania	Girls	2007	14	11.20
Albania	Girls	2008	14	11.38
Albania	Girls	2009	14	11.57
Albania	Girls	2010	14	11.77
Albania	Girls	2011	14	11.97
Albania	Girls	2012	14	12.17
Albania	Girls	2013	14	12.38
Albania	Girls	2014	14	12.59
Albania	Girls	2015	14	12.80
Albania	Girls	2016	14	13.02
Albania	Girls	1975	15	9.59
Albania	Girls	1976	15	9.64
Albania	Girls	1977	15	9.69
Albania	Girls	1978	15	9.74
Albania	Girls	1979	15	9.79
Albania	Girls	1980	15	9.84
Albania	Girls	1981	15	9.90
Albania	Girls	1982	15	9.95
Albania	Girls	1983	15	10.01

Albania	Girls	1984	15	10.08
Albania	Girls	1985	15	10.14
Albania	Girls	1986	15	10.21
Albania	Girls	1987	15	10.28
Albania	Girls	1988	15	10.35
Albania	Girls	1989	15	10.43
Albania	Girls	1990	15	10.51
Albania	Girls	1991	15	10.59
Albania	Girls	1992	15	10.68
Albania	Girls	1993	15	10.77
Albania	Girls	1994	15	10.87
Albania	Girls	1995	15	10.97
Albania	Girls	1996	15	11.08
Albania	Girls	1997	15	11.19
Albania	Girls	1998	15	11.31
Albania	Girls	1999	15	11.43
Albania	Girls	2000	15	11.56
Albania	Girls	2001	15	11.70
Albania	Girls	2002	15	11.85
Albania	Girls	2003	15	12.01
Albania	Girls	2004	15	12.17
Albania	Girls	2005	15	12.34
Albania	Girls	2006	15	12.52
Albania	Girls	2007	15	12.70
Albania	Girls	2008	15	12.90
Albania	Girls	2009	15	13.09
Albania	Girls	2010	15	13.30
Albania	Girls	2011	15	13.50
Albania	Girls	2012	15	13.72
Albania	Girls	2013	15	13.93
Albania	Girls	2014	15	14.15
Albania	Girls	2015	15	14.37
Albania	Girls	2016	15	14.60
Albania	Girls	1975	16	10.71
Albania	Girls	1976	16	10.75
Albania	Girls	1977	16	10.80
Albania	Girls	1978	16	10.85
Albania	Girls	1979	16	10.91
Albania	Girls	1980	16	10.96
Albania	Girls	1981	16	11.02
Albania	Girls	1982	16	11.08
Albania	Girls	1983	16	11.14
Albania	Girls	1984	16	11.20
Albania	Girls	1985	16	11.27
Albania	Girls	1986	16	11.34
Albania	Girls	1987	16	11.41
Albania	Girls	1988	16	11.49
Albania	Girls	1989	16	11.56
Albania	Girls	1990	16	11.65
Albania	Girls	1991	16	11.73
Albania	Girls	1992	16	11.82
Albania	Girls	1993	16	11.92

Albania	Girls	1994	16	12.02
Albania	Girls	1995	16	12.12
Albania	Girls	1996	16	12.23
Albania	Girls	1997	16	12.35
Albania	Girls	1998	16	12.47
Albania	Girls	1999	16	12.60
Albania	Girls	2000	16	12.73
Albania	Girls	2001	16	12.87
Albania	Girls	2002	16	13.02
Albania	Girls	2003	16	13.18
Albania	Girls	2004	16	13.35
Albania	Girls	2005	16	13.53
Albania	Girls	2006	16	13.71
Albania	Girls	2007	16	13.90
Albania	Girls	2008	16	14.09
Albania	Girls	2009	16	14.30
Albania	Girls	2010	16	14.51
Albania	Girls	2011	16	14.72
Albania	Girls	2012	16	14.93
Albania	Girls	2013	16	15.16
Albania	Girls	2014	16	15.38
Albania	Girls	2015	16	15.61
Albania	Girls	2016	16	15.84
Albania	Girls	1975	17	11.50
Albania	Girls	1976	17	11.55
Albania	Girls	1977	17	11.60
Albania	Girls	1978	17	11.65
Albania	Girls	1979	17	11.70
Albania	Girls	1980	17	11.76
Albania	Girls	1981	17	11.82
Albania	Girls	1982	17	11.88
Albania	Girls	1983	17	11.95
Albania	Girls	1984	17	12.01
Albania	Girls	1985	17	12.08
Albania	Girls	1986	17	12.15
Albania	Girls	1987	17	12.23
Albania	Girls	1988	17	12.30
Albania	Girls	1989	17	12.38
Albania	Girls	1990	17	12.47
Albania	Girls	1991	17	12.56
Albania	Girls	1992	17	12.65
Albania	Girls	1993	17	12.75
Albania	Girls	1994	17	12.85
Albania	Girls	1995	17	12.96
Albania	Girls	1996	17	13.07
Albania	Girls	1997	17	13.19
Albania	Girls	1998	17	13.32
Albania	Girls	1999	17	13.45
Albania	Girls	2000	17	13.59
Albania	Girls	2001	17	13.74
Albania	Girls	2002	17	13.90
Albania	Girls	2003	17	14.06

Albania	Girls	2004	17	14.24
Albania	Girls	2005	17	14.42
Albania	Girls	2006	17	14.61
Albania	Girls	2007	17	14.81
Albania	Girls	2008	17	15.01
Albania	Girls	2009	17	15.22
Albania	Girls	2010	17	15.44
Albania	Girls	2011	17	15.66
Albania	Girls	2012	17	15.89
Albania	Girls	2013	17	16.12
Albania	Girls	2014	17	16.35
Albania	Girls	2015	17	16.59
Albania	Girls	2016	17	16.83
Albania	Girls	1975	18	12.04
Albania	Girls	1976	18	12.09
Albania	Girls	1977	18	12.15
Albania	Girls	1978	18	12.20
Albania	Girls	1979	18	12.26
Albania	Girls	1980	18	12.32
Albania	Girls	1981	18	12.38
Albania	Girls	1982	18	12.45
Albania	Girls	1983	18	12.52
Albania	Girls	1984	18	12.59
Albania	Girls	1985	18	12.66
Albania	Girls	1986	18	12.74
Albania	Girls	1987	18	12.82
Albania	Girls	1988	18	12.90
Albania	Girls	1989	18	12.98
Albania	Girls	1990	18	13.07
Albania	Girls	1991	18	13.17
Albania	Girls	1992	18	13.27
Albania	Girls	1993	18	13.38
Albania	Girls	1994	18	13.49
Albania	Girls	1995	18	13.60
Albania	Girls	1996	18	13.73
Albania	Girls	1997	18	13.86
Albania	Girls	1998	18	13.99
Albania	Girls	1999	18	14.14
Albania	Girls	2000	18	14.29
Albania	Girls	2001	18	14.45
Albania	Girls	2002	18	14.61
Albania	Girls	2003	18	14.79
Albania	Girls	2004	18	14.98
Albania	Girls	2005	18	15.18
Albania	Girls	2006	18	15.39
Albania	Girls	2007	18	15.60
Albania	Girls	2008	18	15.82
Albania	Girls	2009	18	16.05
Albania	Girls	2010	18	16.29
Albania	Girls	2011	18	16.53
Albania	Girls	2012	18	16.77
Albania	Girls	2013	18	17.02

Albania	Girls	2014	18	17.27
Albania	Girls	2015	18	17.53
Albania	Girls	2016	18	17.79
Albania	Girls	1975	Average	9.17
Albania	Girls	1976	Average	9.21
Albania	Girls	1977	Average	9.26
Albania	Girls	1978	Average	9.31
Albania	Girls	1979	Average	9.36
Albania	Girls	1980	Average	9.41
Albania	Girls	1981	Average	9.46
Albania	Girls	1982	Average	9.52
Albania	Girls	1983	Average	9.58
Albania	Girls	1984	Average	9.64
Albania	Girls	1985	Average	9.70
Albania	Girls	1986	Average	9.76
Albania	Girls	1987	Average	9.83
Albania	Girls	1988	Average	9.90
Albania	Girls	1989	Average	9.98
Albania	Girls	1990	Average	10.05
Albania	Girls	1991	Average	10.13
Albania	Girls	1992	Average	10.22
Albania	Girls	1993	Average	10.31
Albania	Girls	1994	Average	10.41
Albania	Girls	1995	Average	10.51
Albania	Girls	1996	Average	10.61
Albania	Girls	1997	Average	10.72
Albania	Girls	1998	Average	10.84
Albania	Girls	1999	Average	10.96
Albania	Girls	2000	Average	11.09
Albania	Girls	2001	Average	11.23
Albania	Girls	2002	Average	11.37
Albania	Girls	2003	Average	11.52
Albania	Girls	2004	Average	11.69
Albania	Girls	2005	Average	11.86
Albania	Girls	2006	Average	12.03
Albania	Girls	2007	Average	12.21
Albania	Girls	2008	Average	12.40
Albania	Girls	2009	Average	12.60
Albania	Girls	2010	Average	12.80
Albania	Girls	2011	Average	13.00
Albania	Girls	2012	Average	13.21
Albania	Girls	2013	Average	13.42
Albania	Girls	2014	Average	13.64
Albania	Girls	2015	Average	13.86
Albania	Girls	2016	Average	14.08
Algeria	Boys	1975	12	5.19
Algeria	Boys	1976	12	5.24
Algeria	Boys	1977	12	5.29
Algeria	Boys	1978	12	5.35
Algeria	Boys	1979	12	5.42
Algeria	Boys	1980	12	5.49
Algeria	Boys	1981	12	5.57

Algeria	Boys	1982	12	5.67
Algeria	Boys	1983	12	5.77
Algeria	Boys	1984	12	5.88
Algeria	Boys	1985	12	5.99
Algeria	Boys	1986	12	6.12
Algeria	Boys	1987	12	6.25
Algeria	Boys	1988	12	6.39
Algeria	Boys	1989	12	6.54
Algeria	Boys	1990	12	6.70
Algeria	Boys	1991	12	6.87
Algeria	Boys	1992	12	7.04
Algeria	Boys	1993	12	7.22
Algeria	Boys	1994	12	7.41
Algeria	Boys	1995	12	7.60
Algeria	Boys	1996	12	7.80
Algeria	Boys	1997	12	8.01
Algeria	Boys	1998	12	8.23
Algeria	Boys	1999	12	8.45
Algeria	Boys	2000	12	8.68
Algeria	Boys	2001	12	8.92
Algeria	Boys	2002	12	9.16
Algeria	Boys	2003	12	9.40
Algeria	Boys	2004	12	9.64
Algeria	Boys	2005	12	9.89
Algeria	Boys	2006	12	10.14
Algeria	Boys	2007	12	10.38
Algeria	Boys	2008	12	10.63
Algeria	Boys	2009	12	10.87
Algeria	Boys	2010	12	11.11
Algeria	Boys	2011	12	11.35
Algeria	Boys	2012	12	11.59
Algeria	Boys	2013	12	11.83
Algeria	Boys	2014	12	12.06
Algeria	Boys	2015	12	12.29
Algeria	Boys	2016	12	12.52
Algeria	Boys	1975	13	6.43
Algeria	Boys	1976	13	6.49
Algeria	Boys	1977	13	6.55
Algeria	Boys	1978	13	6.62
Algeria	Boys	1979	13	6.70
Algeria	Boys	1980	13	6.78
Algeria	Boys	1981	13	6.87
Algeria	Boys	1982	13	6.98
Algeria	Boys	1983	13	7.09
Algeria	Boys	1984	13	7.22
Algeria	Boys	1985	13	7.36
Algeria	Boys	1986	13	7.50
Algeria	Boys	1987	13	7.65
Algeria	Boys	1988	13	7.82
Algeria	Boys	1989	13	7.99
Algeria	Boys	1990	13	8.17
Algeria	Boys	1991	13	8.36

Algeria	Boys	1992	13	8.56
Algeria	Boys	1993	13	8.77
Algeria	Boys	1994	13	8.98
Algeria	Boys	1995	13	9.21
Algeria	Boys	1996	13	9.44
Algeria	Boys	1997	13	9.69
Algeria	Boys	1998	13	9.94
Algeria	Boys	1999	13	10.20
Algeria	Boys	2000	13	10.47
Algeria	Boys	2001	13	10.75
Algeria	Boys	2002	13	11.03
Algeria	Boys	2003	13	11.32
Algeria	Boys	2004	13	11.61
Algeria	Boys	2005	13	11.91
Algeria	Boys	2006	13	12.20
Algeria	Boys	2007	13	12.49
Algeria	Boys	2008	13	12.79
Algeria	Boys	2009	13	13.08
Algeria	Boys	2010	13	13.38
Algeria	Boys	2011	13	13.67
Algeria	Boys	2012	13	13.96
Algeria	Boys	2013	13	14.25
Algeria	Boys	2014	13	14.54
Algeria	Boys	2015	13	14.83
Algeria	Boys	2016	13	15.12
Algeria	Boys	1975	14	7.99
Algeria	Boys	1976	14	8.05
Algeria	Boys	1977	14	8.12
Algeria	Boys	1978	14	8.20
Algeria	Boys	1979	14	8.28
Algeria	Boys	1980	14	8.38
Algeria	Boys	1981	14	8.48
Algeria	Boys	1982	14	8.59
Algeria	Boys	1983	14	8.72
Algeria	Boys	1984	14	8.86
Algeria	Boys	1985	14	9.00
Algeria	Boys	1986	14	9.16
Algeria	Boys	1987	14	9.33
Algeria	Boys	1988	14	9.50
Algeria	Boys	1989	14	9.69
Algeria	Boys	1990	14	9.89
Algeria	Boys	1991	14	10.09
Algeria	Boys	1992	14	10.31
Algeria	Boys	1993	14	10.54
Algeria	Boys	1994	14	10.78
Algeria	Boys	1995	14	11.02
Algeria	Boys	1996	14	11.28
Algeria	Boys	1997	14	11.55
Algeria	Boys	1998	14	11.83
Algeria	Boys	1999	14	12.12
Algeria	Boys	2000	14	12.41
Algeria	Boys	2001	14	12.72

Algeria	Boys	2002	14	13.04
Algeria	Boys	2003	14	13.36
Algeria	Boys	2004	14	13.68
Algeria	Boys	2005	14	14.01
Algeria	Boys	2006	14	14.34
Algeria	Boys	2007	14	14.68
Algeria	Boys	2008	14	15.01
Algeria	Boys	2009	14	15.35
Algeria	Boys	2010	14	15.68
Algeria	Boys	2011	14	16.02
Algeria	Boys	2012	14	16.36
Algeria	Boys	2013	14	16.69
Algeria	Boys	2014	14	17.03
Algeria	Boys	2015	14	17.36
Algeria	Boys	2016	14	17.70
Algeria	Boys	1975	15	9.80
Algeria	Boys	1976	15	9.87
Algeria	Boys	1977	15	9.94
Algeria	Boys	1978	15	10.03
Algeria	Boys	1979	15	10.12
Algeria	Boys	1980	15	10.21
Algeria	Boys	1981	15	10.32
Algeria	Boys	1982	15	10.44
Algeria	Boys	1983	15	10.57
Algeria	Boys	1984	15	10.71
Algeria	Boys	1985	15	10.87
Algeria	Boys	1986	15	11.03
Algeria	Boys	1987	15	11.20
Algeria	Boys	1988	15	11.39
Algeria	Boys	1989	15	11.58
Algeria	Boys	1990	15	11.79
Algeria	Boys	1991	15	12.00
Algeria	Boys	1992	15	12.23
Algeria	Boys	1993	15	12.46
Algeria	Boys	1994	15	12.71
Algeria	Boys	1995	15	12.97
Algeria	Boys	1996	15	13.24
Algeria	Boys	1997	15	13.52
Algeria	Boys	1998	15	13.81
Algeria	Boys	1999	15	14.11
Algeria	Boys	2000	15	14.42
Algeria	Boys	2001	15	14.75
Algeria	Boys	2002	15	15.08
Algeria	Boys	2003	15	15.42
Algeria	Boys	2004	15	15.76
Algeria	Boys	2005	15	16.11
Algeria	Boys	2006	15	16.46
Algeria	Boys	2007	15	16.82
Algeria	Boys	2008	15	17.18
Algeria	Boys	2009	15	17.54
Algeria	Boys	2010	15	17.90
Algeria	Boys	2011	15	18.26

Algeria	Boys	2012	15	18.63
Algeria	Boys	2013	15	18.99
Algeria	Boys	2014	15	19.36
Algeria	Boys	2015	15	19.73
Algeria	Boys	2016	15	20.10
Algeria	Boys	1975	16	11.79
Algeria	Boys	1976	16	11.87
Algeria	Boys	1977	16	11.94
Algeria	Boys	1978	16	12.03
Algeria	Boys	1979	16	12.12
Algeria	Boys	1980	16	12.22
Algeria	Boys	1981	16	12.33
Algeria	Boys	1982	16	12.46
Algeria	Boys	1983	16	12.59
Algeria	Boys	1984	16	12.74
Algeria	Boys	1985	16	12.89
Algeria	Boys	1986	16	13.06
Algeria	Boys	1987	16	13.23
Algeria	Boys	1988	16	13.42
Algeria	Boys	1989	16	13.62
Algeria	Boys	1990	16	13.82
Algeria	Boys	1991	16	14.04
Algeria	Boys	1992	16	14.27
Algeria	Boys	1993	16	14.51
Algeria	Boys	1994	16	14.76
Algeria	Boys	1995	16	15.02
Algeria	Boys	1996	16	15.29
Algeria	Boys	1997	16	15.58
Algeria	Boys	1998	16	15.87
Algeria	Boys	1999	16	16.18
Algeria	Boys	2000	16	16.50
Algeria	Boys	2001	16	16.83
Algeria	Boys	2002	16	17.17
Algeria	Boys	2003	16	17.51
Algeria	Boys	2004	16	17.87
Algeria	Boys	2005	16	18.23
Algeria	Boys	2006	16	18.59
Algeria	Boys	2007	16	18.95
Algeria	Boys	2008	16	19.32
Algeria	Boys	2009	16	19.70
Algeria	Boys	2010	16	20.07
Algeria	Boys	2011	16	20.45
Algeria	Boys	2012	16	20.83
Algeria	Boys	2013	16	21.21
Algeria	Boys	2014	16	21.60
Algeria	Boys	2015	16	21.98
Algeria	Boys	2016	16	22.37
Algeria	Boys	1975	17	13.87
Algeria	Boys	1976	17	13.94
Algeria	Boys	1977	17	14.03
Algeria	Boys	1978	17	14.11
Algeria	Boys	1979	17	14.21

Algeria	Boys	1980	17	14.32
Algeria	Boys	1981	17	14.43
Algeria	Boys	1982	17	14.56
Algeria	Boys	1983	17	14.69
Algeria	Boys	1984	17	14.84
Algeria	Boys	1985	17	15.00
Algeria	Boys	1986	17	15.17
Algeria	Boys	1987	17	15.35
Algeria	Boys	1988	17	15.54
Algeria	Boys	1989	17	15.74
Algeria	Boys	1990	17	15.95
Algeria	Boys	1991	17	16.17
Algeria	Boys	1992	17	16.40
Algeria	Boys	1993	17	16.64
Algeria	Boys	1994	17	16.90
Algeria	Boys	1995	17	17.16
Algeria	Boys	1996	17	17.43
Algeria	Boys	1997	17	17.72
Algeria	Boys	1998	17	18.02
Algeria	Boys	1999	17	18.34
Algeria	Boys	2000	17	18.66
Algeria	Boys	2001	17	18.99
Algeria	Boys	2002	17	19.34
Algeria	Boys	2003	17	19.69
Algeria	Boys	2004	17	20.05
Algeria	Boys	2005	17	20.42
Algeria	Boys	2006	17	20.78
Algeria	Boys	2007	17	21.16
Algeria	Boys	2008	17	21.53
Algeria	Boys	2009	17	21.92
Algeria	Boys	2010	17	22.30
Algeria	Boys	2011	17	22.69
Algeria	Boys	2012	17	23.08
Algeria	Boys	2013	17	23.47
Algeria	Boys	2014	17	23.87
Algeria	Boys	2015	17	24.27
Algeria	Boys	2016	17	24.67
Algeria	Boys	1975	18	15.95
Algeria	Boys	1976	18	16.03
Algeria	Boys	1977	18	16.12
Algeria	Boys	1978	18	16.21
Algeria	Boys	1979	18	16.31
Algeria	Boys	1980	18	16.43
Algeria	Boys	1981	18	16.55
Algeria	Boys	1982	18	16.68
Algeria	Boys	1983	18	16.83
Algeria	Boys	1984	18	16.98
Algeria	Boys	1985	18	17.15
Algeria	Boys	1986	18	17.33
Algeria	Boys	1987	18	17.51
Algeria	Boys	1988	18	17.71
Algeria	Boys	1989	18	17.92

Algeria	Boys	1990	18	18.14
Algeria	Boys	1991	18	18.37
Algeria	Boys	1992	18	18.61
Algeria	Boys	1993	18	18.86
Algeria	Boys	1994	18	19.12
Algeria	Boys	1995	18	19.40
Algeria	Boys	1996	18	19.68
Algeria	Boys	1997	18	19.98
Algeria	Boys	1998	18	20.29
Algeria	Boys	1999	18	20.62
Algeria	Boys	2000	18	20.95
Algeria	Boys	2001	18	21.30
Algeria	Boys	2002	18	21.66
Algeria	Boys	2003	18	22.02
Algeria	Boys	2004	18	22.39
Algeria	Boys	2005	18	22.77
Algeria	Boys	2006	18	23.16
Algeria	Boys	2007	18	23.54
Algeria	Boys	2008	18	23.93
Algeria	Boys	2009	18	24.33
Algeria	Boys	2010	18	24.73
Algeria	Boys	2011	18	25.13
Algeria	Boys	2012	18	25.53
Algeria	Boys	2013	18	25.94
Algeria	Boys	2014	18	26.35
Algeria	Boys	2015	18	26.76
Algeria	Boys	2016	18	27.18
Algeria	Boys	1975	Average	10.15
Algeria	Boys	1976	Average	10.21
Algeria	Boys	1977	Average	10.28
Algeria	Boys	1978	Average	10.36
Algeria	Boys	1979	Average	10.45
Algeria	Boys	1980	Average	10.55
Algeria	Boys	1981	Average	10.65
Algeria	Boys	1982	Average	10.77
Algeria	Boys	1983	Average	10.89
Algeria	Boys	1984	Average	11.03
Algeria	Boys	1985	Average	11.18
Algeria	Boys	1986	Average	11.34
Algeria	Boys	1987	Average	11.50
Algeria	Boys	1988	Average	11.68
Algeria	Boys	1989	Average	11.87
Algeria	Boys	1990	Average	12.07
Algeria	Boys	1991	Average	12.27
Algeria	Boys	1992	Average	12.49
Algeria	Boys	1993	Average	12.71
Algeria	Boys	1994	Average	12.95
Algeria	Boys	1995	Average	13.20
Algeria	Boys	1996	Average	13.45
Algeria	Boys	1997	Average	13.72
Algeria	Boys	1998	Average	14.00
Algeria	Boys	1999	Average	14.29

Algeria	Boys	2000	Average	14.59
Algeria	Boys	2001	Average	14.89
Algeria	Boys	2002	Average	15.21
Algeria	Boys	2003	Average	15.53
Algeria	Boys	2004	Average	15.86
Algeria	Boys	2005	Average	16.19
Algeria	Boys	2006	Average	16.52
Algeria	Boys	2007	Average	16.86
Algeria	Boys	2008	Average	17.20
Algeria	Boys	2009	Average	17.54
Algeria	Boys	2010	Average	17.88
Algeria	Boys	2011	Average	18.23
Algeria	Boys	2012	Average	18.57
Algeria	Boys	2013	Average	18.91
Algeria	Boys	2014	Average	19.26
Algeria	Boys	2015	Average	19.60
Algeria	Boys	2016	Average	19.95
Algeria	Girls	1975	12	5.75
Algeria	Girls	1976	12	5.81
Algeria	Girls	1977	12	5.87
Algeria	Girls	1978	12	5.94
Algeria	Girls	1979	12	6.01
Algeria	Girls	1980	12	6.09
Algeria	Girls	1981	12	6.18
Algeria	Girls	1982	12	6.27
Algeria	Girls	1983	12	6.37
Algeria	Girls	1984	12	6.48
Algeria	Girls	1985	12	6.60
Algeria	Girls	1986	12	6.72
Algeria	Girls	1987	12	6.84
Algeria	Girls	1988	12	6.97
Algeria	Girls	1989	12	7.10
Algeria	Girls	1990	12	7.24
Algeria	Girls	1991	12	7.38
Algeria	Girls	1992	12	7.52
Algeria	Girls	1993	12	7.67
Algeria	Girls	1994	12	7.82
Algeria	Girls	1995	12	7.97
Algeria	Girls	1996	12	8.13
Algeria	Girls	1997	12	8.28
Algeria	Girls	1998	12	8.44
Algeria	Girls	1999	12	8.60
Algeria	Girls	2000	12	8.77
Algeria	Girls	2001	12	8.93
Algeria	Girls	2002	12	9.09
Algeria	Girls	2003	12	9.26
Algeria	Girls	2004	12	9.42
Algeria	Girls	2005	12	9.58
Algeria	Girls	2006	12	9.73
Algeria	Girls	2007	12	9.88
Algeria	Girls	2008	12	10.04
Algeria	Girls	2009	12	10.19

Algeria	Girls	2010	12	10.33
Algeria	Girls	2011	12	10.48
Algeria	Girls	2012	12	10.62
Algeria	Girls	2013	12	10.76
Algeria	Girls	2014	12	10.91
Algeria	Girls	2015	12	11.05
Algeria	Girls	2016	12	11.18
Algeria	Girls	1975	13	7.16
Algeria	Girls	1976	13	7.23
Algeria	Girls	1977	13	7.30
Algeria	Girls	1978	13	7.38
Algeria	Girls	1979	13	7.47
Algeria	Girls	1980	13	7.56
Algeria	Girls	1981	13	7.66
Algeria	Girls	1982	13	7.77
Algeria	Girls	1983	13	7.88
Algeria	Girls	1984	13	8.01
Algeria	Girls	1985	13	8.14
Algeria	Girls	1986	13	8.28
Algeria	Girls	1987	13	8.42
Algeria	Girls	1988	13	8.56
Algeria	Girls	1989	13	8.72
Algeria	Girls	1990	13	8.87
Algeria	Girls	1991	13	9.03
Algeria	Girls	1992	13	9.19
Algeria	Girls	1993	13	9.36
Algeria	Girls	1994	13	9.53
Algeria	Girls	1995	13	9.71
Algeria	Girls	1996	13	9.88
Algeria	Girls	1997	13	10.06
Algeria	Girls	1998	13	10.24
Algeria	Girls	1999	13	10.43
Algeria	Girls	2000	13	10.61
Algeria	Girls	2001	13	10.80
Algeria	Girls	2002	13	10.98
Algeria	Girls	2003	13	11.17
Algeria	Girls	2004	13	11.35
Algeria	Girls	2005	13	11.53
Algeria	Girls	2006	13	11.71
Algeria	Girls	2007	13	11.89
Algeria	Girls	2008	13	12.06
Algeria	Girls	2009	13	12.24
Algeria	Girls	2010	13	12.41
Algeria	Girls	2011	13	12.57
Algeria	Girls	2012	13	12.74
Algeria	Girls	2013	13	12.91
Algeria	Girls	2014	13	13.07
Algeria	Girls	2015	13	13.23
Algeria	Girls	2016	13	13.40
Algeria	Girls	1975	14	8.68
Algeria	Girls	1976	14	8.75
Algeria	Girls	1977	14	8.84

Algeria	Girls	1978	14	8.92
Algeria	Girls	1979	14	9.02
Algeria	Girls	1980	14	9.12
Algeria	Girls	1981	14	9.23
Algeria	Girls	1982	14	9.35
Algeria	Girls	1983	14	9.47
Algeria	Girls	1984	14	9.61
Algeria	Girls	1985	14	9.75
Algeria	Girls	1986	14	9.90
Algeria	Girls	1987	14	10.06
Algeria	Girls	1988	14	10.21
Algeria	Girls	1989	14	10.38
Algeria	Girls	1990	14	10.55
Algeria	Girls	1991	14	10.72
Algeria	Girls	1992	14	10.90
Algeria	Girls	1993	14	11.08
Algeria	Girls	1994	14	11.26
Algeria	Girls	1995	14	11.45
Algeria	Girls	1996	14	11.64
Algeria	Girls	1997	14	11.84
Algeria	Girls	1998	14	12.03
Algeria	Girls	1999	14	12.23
Algeria	Girls	2000	14	12.43
Algeria	Girls	2001	14	12.63
Algeria	Girls	2002	14	12.83
Algeria	Girls	2003	14	13.03
Algeria	Girls	2004	14	13.23
Algeria	Girls	2005	14	13.43
Algeria	Girls	2006	14	13.62
Algeria	Girls	2007	14	13.81
Algeria	Girls	2008	14	14.00
Algeria	Girls	2009	14	14.19
Algeria	Girls	2010	14	14.37
Algeria	Girls	2011	14	14.56
Algeria	Girls	2012	14	14.74
Algeria	Girls	2013	14	14.92
Algeria	Girls	2014	14	15.10
Algeria	Girls	2015	14	15.28
Algeria	Girls	2016	14	15.46
Algeria	Girls	1975	15	10.09
Algeria	Girls	1976	15	10.17
Algeria	Girls	1977	15	10.26
Algeria	Girls	1978	15	10.35
Algeria	Girls	1979	15	10.45
Algeria	Girls	1980	15	10.56
Algeria	Girls	1981	15	10.67
Algeria	Girls	1982	15	10.80
Algeria	Girls	1983	15	10.93
Algeria	Girls	1984	15	11.07
Algeria	Girls	1985	15	11.22
Algeria	Girls	1986	15	11.38
Algeria	Girls	1987	15	11.54

Algeria	Girls	1988	15	11.71
Algeria	Girls	1989	15	11.88
Algeria	Girls	1990	15	12.06
Algeria	Girls	1991	15	12.24
Algeria	Girls	1992	15	12.42
Algeria	Girls	1993	15	12.61
Algeria	Girls	1994	15	12.80
Algeria	Girls	1995	15	13.00
Algeria	Girls	1996	15	13.20
Algeria	Girls	1997	15	13.40
Algeria	Girls	1998	15	13.60
Algeria	Girls	1999	15	13.81
Algeria	Girls	2000	15	14.02
Algeria	Girls	2001	15	14.23
Algeria	Girls	2002	15	14.44
Algeria	Girls	2003	15	14.64
Algeria	Girls	2004	15	14.85
Algeria	Girls	2005	15	15.05
Algeria	Girls	2006	15	15.26
Algeria	Girls	2007	15	15.46
Algeria	Girls	2008	15	15.65
Algeria	Girls	2009	15	15.85
Algeria	Girls	2010	15	16.05
Algeria	Girls	2011	15	16.24
Algeria	Girls	2012	15	16.43
Algeria	Girls	2013	15	16.62
Algeria	Girls	2014	15	16.81
Algeria	Girls	2015	15	17.00
Algeria	Girls	2016	15	17.20
Algeria	Girls	1975	16	11.23
Algeria	Girls	1976	16	11.31
Algeria	Girls	1977	16	11.40
Algeria	Girls	1978	16	11.50
Algeria	Girls	1979	16	11.60
Algeria	Girls	1980	16	11.71
Algeria	Girls	1981	16	11.83
Algeria	Girls	1982	16	11.96
Algeria	Girls	1983	16	12.10
Algeria	Girls	1984	16	12.25
Algeria	Girls	1985	16	12.40
Algeria	Girls	1986	16	12.56
Algeria	Girls	1987	16	12.73
Algeria	Girls	1988	16	12.90
Algeria	Girls	1989	16	13.08
Algeria	Girls	1990	16	13.26
Algeria	Girls	1991	16	13.45
Algeria	Girls	1992	16	13.64
Algeria	Girls	1993	16	13.84
Algeria	Girls	1994	16	14.03
Algeria	Girls	1995	16	14.24
Algeria	Girls	1996	16	14.44
Algeria	Girls	1997	16	14.65

Algeria	Girls	1998	16	14.86
Algeria	Girls	1999	16	15.07
Algeria	Girls	2000	16	15.29
Algeria	Girls	2001	16	15.50
Algeria	Girls	2002	16	15.72
Algeria	Girls	2003	16	15.93
Algeria	Girls	2004	16	16.14
Algeria	Girls	2005	16	16.36
Algeria	Girls	2006	16	16.56
Algeria	Girls	2007	16	16.77
Algeria	Girls	2008	16	16.98
Algeria	Girls	2009	16	17.18
Algeria	Girls	2010	16	17.38
Algeria	Girls	2011	16	17.58
Algeria	Girls	2012	16	17.78
Algeria	Girls	2013	16	17.98
Algeria	Girls	2014	16	18.18
Algeria	Girls	2015	16	18.38
Algeria	Girls	2016	16	18.58
Algeria	Girls	1975	17	12.06
Algeria	Girls	1976	17	12.14
Algeria	Girls	1977	17	12.24
Algeria	Girls	1978	17	12.34
Algeria	Girls	1979	17	12.44
Algeria	Girls	1980	17	12.56
Algeria	Girls	1981	17	12.69
Algeria	Girls	1982	17	12.82
Algeria	Girls	1983	17	12.96
Algeria	Girls	1984	17	13.12
Algeria	Girls	1985	17	13.28
Algeria	Girls	1986	17	13.45
Algeria	Girls	1987	17	13.62
Algeria	Girls	1988	17	13.80
Algeria	Girls	1989	17	13.99
Algeria	Girls	1990	17	14.18
Algeria	Girls	1991	17	14.38
Algeria	Girls	1992	17	14.58
Algeria	Girls	1993	17	14.78
Algeria	Girls	1994	17	14.99
Algeria	Girls	1995	17	15.20
Algeria	Girls	1996	17	15.42
Algeria	Girls	1997	17	15.64
Algeria	Girls	1998	17	15.86
Algeria	Girls	1999	17	16.08
Algeria	Girls	2000	17	16.31
Algeria	Girls	2001	17	16.53
Algeria	Girls	2002	17	16.76
Algeria	Girls	2003	17	16.98
Algeria	Girls	2004	17	17.21
Algeria	Girls	2005	17	17.43
Algeria	Girls	2006	17	17.65
Algeria	Girls	2007	17	17.87

Algeria	Girls	2008	17	18.08
Algeria	Girls	2009	17	18.30
Algeria	Girls	2010	17	18.51
Algeria	Girls	2011	17	18.72
Algeria	Girls	2012	17	18.93
Algeria	Girls	2013	17	19.14
Algeria	Girls	2014	17	19.34
Algeria	Girls	2015	17	19.55
Algeria	Girls	2016	17	19.76
Algeria	Girls	1975	18	12.65
Algeria	Girls	1976	18	12.74
Algeria	Girls	1977	18	12.85
Algeria	Girls	1978	18	12.95
Algeria	Girls	1979	18	13.07
Algeria	Girls	1980	18	13.20
Algeria	Girls	1981	18	13.33
Algeria	Girls	1982	18	13.48
Algeria	Girls	1983	18	13.64
Algeria	Girls	1984	18	13.80
Algeria	Girls	1985	18	13.98
Algeria	Girls	1986	18	14.16
Algeria	Girls	1987	18	14.36
Algeria	Girls	1988	18	14.55
Algeria	Girls	1989	18	14.76
Algeria	Girls	1990	18	14.97
Algeria	Girls	1991	18	15.18
Algeria	Girls	1992	18	15.40
Algeria	Girls	1993	18	15.62
Algeria	Girls	1994	18	15.85
Algeria	Girls	1995	18	16.08
Algeria	Girls	1996	18	16.32
Algeria	Girls	1997	18	16.56
Algeria	Girls	1998	18	16.80
Algeria	Girls	1999	18	17.05
Algeria	Girls	2000	18	17.29
Algeria	Girls	2001	18	17.54
Algeria	Girls	2002	18	17.79
Algeria	Girls	2003	18	18.03
Algeria	Girls	2004	18	18.28
Algeria	Girls	2005	18	18.52
Algeria	Girls	2006	18	18.76
Algeria	Girls	2007	18	18.99
Algeria	Girls	2008	18	19.23
Algeria	Girls	2009	18	19.46
Algeria	Girls	2010	18	19.69
Algeria	Girls	2011	18	19.92
Algeria	Girls	2012	18	20.14
Algeria	Girls	2013	18	20.37
Algeria	Girls	2014	18	20.59
Algeria	Girls	2015	18	20.81
Algeria	Girls	2016	18	21.04
Algeria	Girls	1975	Average	9.66

Algeria	Girls	1976	Average	9.74
Algeria	Girls	1977	Average	9.82
Algeria	Girls	1978	Average	9.91
Algeria	Girls	1979	Average	10.01
Algeria	Girls	1980	Average	10.11
Algeria	Girls	1981	Average	10.23
Algeria	Girls	1982	Average	10.35
Algeria	Girls	1983	Average	10.48
Algeria	Girls	1984	Average	10.62
Algeria	Girls	1985	Average	10.77
Algeria	Girls	1986	Average	10.92
Algeria	Girls	1987	Average	11.08
Algeria	Girls	1988	Average	11.24
Algeria	Girls	1989	Average	11.41
Algeria	Girls	1990	Average	11.59
Algeria	Girls	1991	Average	11.77
Algeria	Girls	1992	Average	11.95
Algeria	Girls	1993	Average	12.14
Algeria	Girls	1994	Average	12.33
Algeria	Girls	1995	Average	12.52
Algeria	Girls	1996	Average	12.72
Algeria	Girls	1997	Average	12.92
Algeria	Girls	1998	Average	13.12
Algeria	Girls	1999	Average	13.32
Algeria	Girls	2000	Average	13.53
Algeria	Girls	2001	Average	13.74
Algeria	Girls	2002	Average	13.94
Algeria	Girls	2003	Average	14.15
Algeria	Girls	2004	Average	14.35
Algeria	Girls	2005	Average	14.56
Algeria	Girls	2006	Average	14.76
Algeria	Girls	2007	Average	14.95
Algeria	Girls	2008	Average	15.15
Algeria	Girls	2009	Average	15.34
Algeria	Girls	2010	Average	15.53
Algeria	Girls	2011	Average	15.72
Algeria	Girls	2012	Average	15.91
Algeria	Girls	2013	Average	16.10
Algeria	Girls	2014	Average	16.29
Algeria	Girls	2015	Average	16.47
Algeria	Girls	2016	Average	16.66
American Samoa	Boys	1975	12	10.35
American Samoa	Boys	1976	12	10.52
American Samoa	Boys	1977	12	10.69
American Samoa	Boys	1978	12	10.87
American Samoa	Boys	1979	12	11.06
American Samoa	Boys	1980	12	11.26
American Samoa	Boys	1981	12	11.46
American Samoa	Boys	1982	12	11.68
American Samoa	Boys	1983	12	11.91
American Samoa	Boys	1984	12	12.15
American Samoa	Boys	1985	12	12.40

American Samoa	Boys	1986	12	12.65
American Samoa	Boys	1987	12	12.91
American Samoa	Boys	1988	12	13.17
American Samoa	Boys	1989	12	13.44
American Samoa	Boys	1990	12	13.71
American Samoa	Boys	1991	12	13.99
American Samoa	Boys	1992	12	14.27
American Samoa	Boys	1993	12	14.56
American Samoa	Boys	1994	12	14.85
American Samoa	Boys	1995	12	15.14
American Samoa	Boys	1996	12	15.44
American Samoa	Boys	1997	12	15.73
American Samoa	Boys	1998	12	16.02
American Samoa	Boys	1999	12	16.32
American Samoa	Boys	2000	12	16.61
American Samoa	Boys	2001	12	16.88
American Samoa	Boys	2002	12	17.14
American Samoa	Boys	2003	12	17.40
American Samoa	Boys	2004	12	17.64
American Samoa	Boys	2005	12	17.88
American Samoa	Boys	2006	12	18.11
American Samoa	Boys	2007	12	18.33
American Samoa	Boys	2008	12	18.56
American Samoa	Boys	2009	12	18.78
American Samoa	Boys	2010	12	19.00
American Samoa	Boys	2011	12	19.21
American Samoa	Boys	2012	12	19.43
American Samoa	Boys	2013	12	19.65
American Samoa	Boys	2014	12	19.86
American Samoa	Boys	2015	12	20.07
American Samoa	Boys	2016	12	20.27
American Samoa	Boys	1975	13	12.83
American Samoa	Boys	1976	13	13.03
American Samoa	Boys	1977	13	13.23
American Samoa	Boys	1978	13	13.45
American Samoa	Boys	1979	13	13.68
American Samoa	Boys	1980	13	13.91
American Samoa	Boys	1981	13	14.16
American Samoa	Boys	1982	13	14.42
American Samoa	Boys	1983	13	14.70
American Samoa	Boys	1984	13	14.99
American Samoa	Boys	1985	13	15.29
American Samoa	Boys	1986	13	15.59
American Samoa	Boys	1987	13	15.91
American Samoa	Boys	1988	13	16.23
American Samoa	Boys	1989	13	16.56
American Samoa	Boys	1990	13	16.89
American Samoa	Boys	1991	13	17.23
American Samoa	Boys	1992	13	17.58
American Samoa	Boys	1993	13	17.93
American Samoa	Boys	1994	13	18.29
American Samoa	Boys	1995	13	18.66

American Samoa	Boys	1996	13	19.03
American Samoa	Boys	1997	13	19.40
American Samoa	Boys	1998	13	19.76
American Samoa	Boys	1999	13	20.13
American Samoa	Boys	2000	13	20.50
American Samoa	Boys	2001	13	20.84
American Samoa	Boys	2002	13	21.18
American Samoa	Boys	2003	13	21.50
American Samoa	Boys	2004	13	21.80
American Samoa	Boys	2005	13	22.11
American Samoa	Boys	2006	13	22.40
American Samoa	Boys	2007	13	22.69
American Samoa	Boys	2008	13	22.97
American Samoa	Boys	2009	13	23.26
American Samoa	Boys	2010	13	23.54
American Samoa	Boys	2011	13	23.82
American Samoa	Boys	2012	13	24.10
American Samoa	Boys	2013	13	24.38
American Samoa	Boys	2014	13	24.66
American Samoa	Boys	2015	13	24.93
American Samoa	Boys	2016	13	25.20
American Samoa	Boys	1975	14	15.59
American Samoa	Boys	1976	14	15.81
American Samoa	Boys	1977	14	16.04
American Samoa	Boys	1978	14	16.29
American Samoa	Boys	1979	14	16.54
American Samoa	Boys	1980	14	16.81
American Samoa	Boys	1981	14	17.09
American Samoa	Boys	1982	14	17.39
American Samoa	Boys	1983	14	17.70
American Samoa	Boys	1984	14	18.03
American Samoa	Boys	1985	14	18.37
American Samoa	Boys	1986	14	18.72
American Samoa	Boys	1987	14	19.08
American Samoa	Boys	1988	14	19.45
American Samoa	Boys	1989	14	19.83
American Samoa	Boys	1990	14	20.21
American Samoa	Boys	1991	14	20.60
American Samoa	Boys	1992	14	21.00
American Samoa	Boys	1993	14	21.42
American Samoa	Boys	1994	14	21.84
American Samoa	Boys	1995	14	22.27
American Samoa	Boys	1996	14	22.70
American Samoa	Boys	1997	14	23.13
American Samoa	Boys	1998	14	23.56
American Samoa	Boys	1999	14	23.99
American Samoa	Boys	2000	14	24.42
American Samoa	Boys	2001	14	24.83
American Samoa	Boys	2002	14	25.23
American Samoa	Boys	2003	14	25.61
American Samoa	Boys	2004	14	25.98
American Samoa	Boys	2005	14	26.33

American Samoa	Boys	2006	14	26.68
American Samoa	Boys	2007	14	27.03
American Samoa	Boys	2008	14	27.37
American Samoa	Boys	2009	14	27.71
American Samoa	Boys	2010	14	28.05
American Samoa	Boys	2011	14	28.39
American Samoa	Boys	2012	14	28.73
American Samoa	Boys	2013	14	29.07
American Samoa	Boys	2014	14	29.41
American Samoa	Boys	2015	14	29.75
American Samoa	Boys	2016	14	30.08
American Samoa	Boys	1975	15	18.43
American Samoa	Boys	1976	15	18.67
American Samoa	Boys	1977	15	18.92
American Samoa	Boys	1978	15	19.18
American Samoa	Boys	1979	15	19.45
American Samoa	Boys	1980	15	19.73
American Samoa	Boys	1981	15	20.03
American Samoa	Boys	1982	15	20.35
American Samoa	Boys	1983	15	20.69
American Samoa	Boys	1984	15	21.04
American Samoa	Boys	1985	15	21.41
American Samoa	Boys	1986	15	21.79
American Samoa	Boys	1987	15	22.18
American Samoa	Boys	1988	15	22.58
American Samoa	Boys	1989	15	22.99
American Samoa	Boys	1990	15	23.40
American Samoa	Boys	1991	15	23.83
American Samoa	Boys	1992	15	24.27
American Samoa	Boys	1993	15	24.72
American Samoa	Boys	1994	15	25.18
American Samoa	Boys	1995	15	25.65
American Samoa	Boys	1996	15	26.12
American Samoa	Boys	1997	15	26.60
American Samoa	Boys	1998	15	27.08
American Samoa	Boys	1999	15	27.56
American Samoa	Boys	2000	15	28.03
American Samoa	Boys	2001	15	28.49
American Samoa	Boys	2002	15	28.93
American Samoa	Boys	2003	15	29.35
American Samoa	Boys	2004	15	29.76
American Samoa	Boys	2005	15	30.16
American Samoa	Boys	2006	15	30.55
American Samoa	Boys	2007	15	30.94
American Samoa	Boys	2008	15	31.32
American Samoa	Boys	2009	15	31.70
American Samoa	Boys	2010	15	32.09
American Samoa	Boys	2011	15	32.47
American Samoa	Boys	2012	15	32.86
American Samoa	Boys	2013	15	33.24
American Samoa	Boys	2014	15	33.63
American Samoa	Boys	2015	15	34.02

American Samoa	Boys	2016	15	34.40
American Samoa	Boys	1975	16	21.26
American Samoa	Boys	1976	16	21.51
American Samoa	Boys	1977	16	21.76
American Samoa	Boys	1978	16	22.03
American Samoa	Boys	1979	16	22.31
American Samoa	Boys	1980	16	22.61
American Samoa	Boys	1981	16	22.92
American Samoa	Boys	1982	16	23.25
American Samoa	Boys	1983	16	23.60
American Samoa	Boys	1984	16	23.96
American Samoa	Boys	1985	16	24.35
American Samoa	Boys	1986	16	24.74
American Samoa	Boys	1987	16	25.15
American Samoa	Boys	1988	16	25.57
American Samoa	Boys	1989	16	25.99
American Samoa	Boys	1990	16	26.42
American Samoa	Boys	1991	16	26.87
American Samoa	Boys	1992	16	27.33
American Samoa	Boys	1993	16	27.80
American Samoa	Boys	1994	16	28.29
American Samoa	Boys	1995	16	28.79
American Samoa	Boys	1996	16	29.28
American Samoa	Boys	1997	16	29.79
American Samoa	Boys	1998	16	30.29
American Samoa	Boys	1999	16	30.80
American Samoa	Boys	2000	16	31.30
American Samoa	Boys	2001	16	31.79
American Samoa	Boys	2002	16	32.25
American Samoa	Boys	2003	16	32.70
American Samoa	Boys	2004	16	33.13
American Samoa	Boys	2005	16	33.56
American Samoa	Boys	2006	16	33.97
American Samoa	Boys	2007	16	34.39
American Samoa	Boys	2008	16	34.80
American Samoa	Boys	2009	16	35.20
American Samoa	Boys	2010	16	35.61
American Samoa	Boys	2011	16	36.03
American Samoa	Boys	2012	16	36.44
American Samoa	Boys	2013	16	36.86
American Samoa	Boys	2014	16	37.28
American Samoa	Boys	2015	16	37.70
American Samoa	Boys	2016	16	38.12
American Samoa	Boys	1975	17	24.07
American Samoa	Boys	1976	17	24.32
American Samoa	Boys	1977	17	24.58
American Samoa	Boys	1978	17	24.86
American Samoa	Boys	1979	17	25.14
American Samoa	Boys	1980	17	25.44
American Samoa	Boys	1981	17	25.76
American Samoa	Boys	1982	17	26.10
American Samoa	Boys	1983	17	26.45

American Samoa	Boys	1984	17	26.83
American Samoa	Boys	1985	17	27.22
American Samoa	Boys	1986	17	27.63
American Samoa	Boys	1987	17	28.05
American Samoa	Boys	1988	17	28.47
American Samoa	Boys	1989	17	28.91
American Samoa	Boys	1990	17	29.35
American Samoa	Boys	1991	17	29.81
American Samoa	Boys	1992	17	30.29
American Samoa	Boys	1993	17	30.77
American Samoa	Boys	1994	17	31.27
American Samoa	Boys	1995	17	31.79
American Samoa	Boys	1996	17	32.30
American Samoa	Boys	1997	17	32.82
American Samoa	Boys	1998	17	33.34
American Samoa	Boys	1999	17	33.86
American Samoa	Boys	2000	17	34.39
American Samoa	Boys	2001	17	34.89
American Samoa	Boys	2002	17	35.37
American Samoa	Boys	2003	17	35.83
American Samoa	Boys	2004	17	36.28
American Samoa	Boys	2005	17	36.72
American Samoa	Boys	2006	17	37.15
American Samoa	Boys	2007	17	37.57
American Samoa	Boys	2008	17	38.00
American Samoa	Boys	2009	17	38.42
American Samoa	Boys	2010	17	38.85
American Samoa	Boys	2011	17	39.28
American Samoa	Boys	2012	17	39.71
American Samoa	Boys	2013	17	40.15
American Samoa	Boys	2014	17	40.59
American Samoa	Boys	2015	17	41.03
American Samoa	Boys	2016	17	41.46
American Samoa	Boys	1975	18	26.92
American Samoa	Boys	1976	18	27.18
American Samoa	Boys	1977	18	27.45
American Samoa	Boys	1978	18	27.74
American Samoa	Boys	1979	18	28.03
American Samoa	Boys	1980	18	28.34
American Samoa	Boys	1981	18	28.67
American Samoa	Boys	1982	18	29.01
American Samoa	Boys	1983	18	29.38
American Samoa	Boys	1984	18	29.77
American Samoa	Boys	1985	18	30.18
American Samoa	Boys	1986	18	30.59
American Samoa	Boys	1987	18	31.02
American Samoa	Boys	1988	18	31.46
American Samoa	Boys	1989	18	31.91
American Samoa	Boys	1990	18	32.37
American Samoa	Boys	1991	18	32.84
American Samoa	Boys	1992	18	33.33
American Samoa	Boys	1993	18	33.83

American Samoa	Boys	1994	18	34.34
American Samoa	Boys	1995	18	34.86
American Samoa	Boys	1996	18	35.39
American Samoa	Boys	1997	18	35.92
American Samoa	Boys	1998	18	36.46
American Samoa	Boys	1999	18	36.99
American Samoa	Boys	2000	18	37.53
American Samoa	Boys	2001	18	38.04
American Samoa	Boys	2002	18	38.53
American Samoa	Boys	2003	18	39.00
American Samoa	Boys	2004	18	39.46
American Samoa	Boys	2005	18	39.91
American Samoa	Boys	2006	18	40.35
American Samoa	Boys	2007	18	40.78
American Samoa	Boys	2008	18	41.21
American Samoa	Boys	2009	18	41.64
American Samoa	Boys	2010	18	42.07
American Samoa	Boys	2011	18	42.51
American Samoa	Boys	2012	18	42.95
American Samoa	Boys	2013	18	43.40
American Samoa	Boys	2014	18	43.84
American Samoa	Boys	2015	18	44.29
American Samoa	Boys	2016	18	44.74
American Samoa	Boys	1975	Average	18.49
American Samoa	Boys	1976	Average	18.72
American Samoa	Boys	1977	Average	18.95
American Samoa	Boys	1978	Average	19.20
American Samoa	Boys	1979	Average	19.46
American Samoa	Boys	1980	Average	19.73
American Samoa	Boys	1981	Average	20.01
American Samoa	Boys	1982	Average	20.31
American Samoa	Boys	1983	Average	20.63
American Samoa	Boys	1984	Average	20.97
American Samoa	Boys	1985	Average	21.32
American Samoa	Boys	1986	Average	21.68
American Samoa	Boys	1987	Average	22.04
American Samoa	Boys	1988	Average	22.42
American Samoa	Boys	1989	Average	22.80
American Samoa	Boys	1990	Average	23.19
American Samoa	Boys	1991	Average	23.59
American Samoa	Boys	1992	Average	24.01
American Samoa	Boys	1993	Average	24.43
American Samoa	Boys	1994	Average	24.87
American Samoa	Boys	1995	Average	25.31
American Samoa	Boys	1996	Average	25.75
American Samoa	Boys	1997	Average	26.20
American Samoa	Boys	1998	Average	26.64
American Samoa	Boys	1999	Average	27.09
American Samoa	Boys	2000	Average	27.54
American Samoa	Boys	2001	Average	27.97
American Samoa	Boys	2002	Average	28.38
American Samoa	Boys	2003	Average	28.77

American Samoa	Boys	2004	Average	29.15
American Samoa	Boys	2005	Average	29.52
American Samoa	Boys	2006	Average	29.89
American Samoa	Boys	2007	Average	30.25
American Samoa	Boys	2008	Average	30.60
American Samoa	Boys	2009	Average	30.96
American Samoa	Boys	2010	Average	31.32
American Samoa	Boys	2011	Average	31.67
American Samoa	Boys	2012	Average	32.03
American Samoa	Boys	2013	Average	32.39
American Samoa	Boys	2014	Average	32.75
American Samoa	Boys	2015	Average	33.11
American Samoa	Boys	2016	Average	33.47
American Samoa	Girls	1975	12	11.70
American Samoa	Girls	1976	12	11.86
American Samoa	Girls	1977	12	12.03
American Samoa	Girls	1978	12	12.19
American Samoa	Girls	1979	12	12.36
American Samoa	Girls	1980	12	12.54
American Samoa	Girls	1981	12	12.73
American Samoa	Girls	1982	12	12.92
American Samoa	Girls	1983	12	13.12
American Samoa	Girls	1984	12	13.33
American Samoa	Girls	1985	12	13.55
American Samoa	Girls	1986	12	13.77
American Samoa	Girls	1987	12	13.99
American Samoa	Girls	1988	12	14.21
American Samoa	Girls	1989	12	14.44
American Samoa	Girls	1990	12	14.66
American Samoa	Girls	1991	12	14.89
American Samoa	Girls	1992	12	15.12
American Samoa	Girls	1993	12	15.36
American Samoa	Girls	1994	12	15.59
American Samoa	Girls	1995	12	15.83
American Samoa	Girls	1996	12	16.06
American Samoa	Girls	1997	12	16.29
American Samoa	Girls	1998	12	16.52
American Samoa	Girls	1999	12	16.74
American Samoa	Girls	2000	12	16.96
American Samoa	Girls	2001	12	17.16
American Samoa	Girls	2002	12	17.35
American Samoa	Girls	2003	12	17.54
American Samoa	Girls	2004	12	17.71
American Samoa	Girls	2005	12	17.88
American Samoa	Girls	2006	12	18.04
American Samoa	Girls	2007	12	18.19
American Samoa	Girls	2008	12	18.34
American Samoa	Girls	2009	12	18.49
American Samoa	Girls	2010	12	18.64
American Samoa	Girls	2011	12	18.78
American Samoa	Girls	2012	12	18.93
American Samoa	Girls	2013	12	19.07

American Samoa	Girls	2014	12	19.21
American Samoa	Girls	2015	12	19.35
American Samoa	Girls	2016	12	19.50
American Samoa	Girls	1975	13	14.16
American Samoa	Girls	1976	13	14.34
American Samoa	Girls	1977	13	14.53
American Samoa	Girls	1978	13	14.73
American Samoa	Girls	1979	13	14.92
American Samoa	Girls	1980	13	15.13
American Samoa	Girls	1981	13	15.34
American Samoa	Girls	1982	13	15.57
American Samoa	Girls	1983	13	15.80
American Samoa	Girls	1984	13	16.04
American Samoa	Girls	1985	13	16.29
American Samoa	Girls	1986	13	16.55
American Samoa	Girls	1987	13	16.81
American Samoa	Girls	1988	13	17.07
American Samoa	Girls	1989	13	17.33
American Samoa	Girls	1990	13	17.60
American Samoa	Girls	1991	13	17.87
American Samoa	Girls	1992	13	18.14
American Samoa	Girls	1993	13	18.42
American Samoa	Girls	1994	13	18.69
American Samoa	Girls	1995	13	18.97
American Samoa	Girls	1996	13	19.25
American Samoa	Girls	1997	13	19.53
American Samoa	Girls	1998	13	19.79
American Samoa	Girls	1999	13	20.06
American Samoa	Girls	2000	13	20.32
American Samoa	Girls	2001	13	20.56
American Samoa	Girls	2002	13	20.79
American Samoa	Girls	2003	13	21.01
American Samoa	Girls	2004	13	21.22
American Samoa	Girls	2005	13	21.42
American Samoa	Girls	2006	13	21.61
American Samoa	Girls	2007	13	21.80
American Samoa	Girls	2008	13	21.98
American Samoa	Girls	2009	13	22.16
American Samoa	Girls	2010	13	22.33
American Samoa	Girls	2011	13	22.51
American Samoa	Girls	2012	13	22.69
American Samoa	Girls	2013	13	22.86
American Samoa	Girls	2014	13	23.04
American Samoa	Girls	2015	13	23.21
American Samoa	Girls	2016	13	23.38
American Samoa	Girls	1975	14	16.47
American Samoa	Girls	1976	14	16.68
American Samoa	Girls	1977	14	16.88
American Samoa	Girls	1978	14	17.09
American Samoa	Girls	1979	14	17.31
American Samoa	Girls	1980	14	17.53
American Samoa	Girls	1981	14	17.76

American Samoa	Girls	1982	14	18.01
American Samoa	Girls	1983	14	18.27
American Samoa	Girls	1984	14	18.54
American Samoa	Girls	1985	14	18.81
American Samoa	Girls	1986	14	19.09
American Samoa	Girls	1987	14	19.38
American Samoa	Girls	1988	14	19.67
American Samoa	Girls	1989	14	19.96
American Samoa	Girls	1990	14	20.25
American Samoa	Girls	1991	14	20.55
American Samoa	Girls	1992	14	20.85
American Samoa	Girls	1993	14	21.16
American Samoa	Girls	1994	14	21.47
American Samoa	Girls	1995	14	21.78
American Samoa	Girls	1996	14	22.09
American Samoa	Girls	1997	14	22.40
American Samoa	Girls	1998	14	22.70
American Samoa	Girls	1999	14	23.00
American Samoa	Girls	2000	14	23.29
American Samoa	Girls	2001	14	23.56
American Samoa	Girls	2002	14	23.82
American Samoa	Girls	2003	14	24.06
American Samoa	Girls	2004	14	24.30
American Samoa	Girls	2005	14	24.53
American Samoa	Girls	2006	14	24.74
American Samoa	Girls	2007	14	24.95
American Samoa	Girls	2008	14	25.16
American Samoa	Girls	2009	14	25.36
American Samoa	Girls	2010	14	25.56
American Samoa	Girls	2011	14	25.76
American Samoa	Girls	2012	14	25.96
American Samoa	Girls	2013	14	26.16
American Samoa	Girls	2014	14	26.36
American Samoa	Girls	2015	14	26.56
American Samoa	Girls	2016	14	26.76
American Samoa	Girls	1975	15	18.40
American Samoa	Girls	1976	15	18.62
American Samoa	Girls	1977	15	18.83
American Samoa	Girls	1978	15	19.05
American Samoa	Girls	1979	15	19.28
American Samoa	Girls	1980	15	19.52
American Samoa	Girls	1981	15	19.76
American Samoa	Girls	1982	15	20.02
American Samoa	Girls	1983	15	20.30
American Samoa	Girls	1984	15	20.58
American Samoa	Girls	1985	15	20.87
American Samoa	Girls	1986	15	21.17
American Samoa	Girls	1987	15	21.47
American Samoa	Girls	1988	15	21.78
American Samoa	Girls	1989	15	22.09
American Samoa	Girls	1990	15	22.40
American Samoa	Girls	1991	15	22.71

American Samoa	Girls	1992	15	23.04
American Samoa	Girls	1993	15	23.36
American Samoa	Girls	1994	15	23.69
American Samoa	Girls	1995	15	24.03
American Samoa	Girls	1996	15	24.36
American Samoa	Girls	1997	15	24.68
American Samoa	Girls	1998	15	25.01
American Samoa	Girls	1999	15	25.32
American Samoa	Girls	2000	15	25.64
American Samoa	Girls	2001	15	25.93
American Samoa	Girls	2002	15	26.20
American Samoa	Girls	2003	15	26.47
American Samoa	Girls	2004	15	26.72
American Samoa	Girls	2005	15	26.96
American Samoa	Girls	2006	15	27.19
American Samoa	Girls	2007	15	27.41
American Samoa	Girls	2008	15	27.63
American Samoa	Girls	2009	15	27.85
American Samoa	Girls	2010	15	28.07
American Samoa	Girls	2011	15	28.28
American Samoa	Girls	2012	15	28.50
American Samoa	Girls	2013	15	28.72
American Samoa	Girls	2014	15	28.94
American Samoa	Girls	2015	15	29.15
American Samoa	Girls	2016	15	29.37
American Samoa	Girls	1975	16	19.91
American Samoa	Girls	1976	16	20.13
American Samoa	Girls	1977	16	20.35
American Samoa	Girls	1978	16	20.58
American Samoa	Girls	1979	16	20.82
American Samoa	Girls	1980	16	21.07
American Samoa	Girls	1981	16	21.32
American Samoa	Girls	1982	16	21.60
American Samoa	Girls	1983	16	21.88
American Samoa	Girls	1984	16	22.17
American Samoa	Girls	1985	16	22.48
American Samoa	Girls	1986	16	22.79
American Samoa	Girls	1987	16	23.10
American Samoa	Girls	1988	16	23.42
American Samoa	Girls	1989	16	23.75
American Samoa	Girls	1990	16	24.07
American Samoa	Girls	1991	16	24.40
American Samoa	Girls	1992	16	24.74
American Samoa	Girls	1993	16	25.08
American Samoa	Girls	1994	16	25.42
American Samoa	Girls	1995	16	25.77
American Samoa	Girls	1996	16	26.11
American Samoa	Girls	1997	16	26.45
American Samoa	Girls	1998	16	26.79
American Samoa	Girls	1999	16	27.12
American Samoa	Girls	2000	16	27.44
American Samoa	Girls	2001	16	27.75

American Samoa	Girls	2002	16	28.04
American Samoa	Girls	2003	16	28.31
American Samoa	Girls	2004	16	28.57
American Samoa	Girls	2005	16	28.82
American Samoa	Girls	2006	16	29.06
American Samoa	Girls	2007	16	29.29
American Samoa	Girls	2008	16	29.52
American Samoa	Girls	2009	16	29.75
American Samoa	Girls	2010	16	29.97
American Samoa	Girls	2011	16	30.20
American Samoa	Girls	2012	16	30.42
American Samoa	Girls	2013	16	30.65
American Samoa	Girls	2014	16	30.88
American Samoa	Girls	2015	16	31.10
American Samoa	Girls	2016	16	31.33
American Samoa	Girls	1975	17	21.15
American Samoa	Girls	1976	17	21.39
American Samoa	Girls	1977	17	21.63
American Samoa	Girls	1978	17	21.87
American Samoa	Girls	1979	17	22.12
American Samoa	Girls	1980	17	22.38
American Samoa	Girls	1981	17	22.65
American Samoa	Girls	1982	17	22.94
American Samoa	Girls	1983	17	23.24
American Samoa	Girls	1984	17	23.55
American Samoa	Girls	1985	17	23.87
American Samoa	Girls	1986	17	24.20
American Samoa	Girls	1987	17	24.53
American Samoa	Girls	1988	17	24.86
American Samoa	Girls	1989	17	25.20
American Samoa	Girls	1990	17	25.54
American Samoa	Girls	1991	17	25.89
American Samoa	Girls	1992	17	26.24
American Samoa	Girls	1993	17	26.59
American Samoa	Girls	1994	17	26.95
American Samoa	Girls	1995	17	27.31
American Samoa	Girls	1996	17	27.67
American Samoa	Girls	1997	17	28.02
American Samoa	Girls	1998	17	28.37
American Samoa	Girls	1999	17	28.71
American Samoa	Girls	2000	17	29.05
American Samoa	Girls	2001	17	29.36
American Samoa	Girls	2002	17	29.66
American Samoa	Girls	2003	17	29.94
American Samoa	Girls	2004	17	30.21
American Samoa	Girls	2005	17	30.46
American Samoa	Girls	2006	17	30.71
American Samoa	Girls	2007	17	30.95
American Samoa	Girls	2008	17	31.19
American Samoa	Girls	2009	17	31.42
American Samoa	Girls	2010	17	31.64
American Samoa	Girls	2011	17	31.87

American Samoa	Girls	2012	17	32.10
American Samoa	Girls	2013	17	32.34
American Samoa	Girls	2014	17	32.57
American Samoa	Girls	2015	17	32.80
American Samoa	Girls	2016	17	33.03
American Samoa	Girls	1975	18	22.49
American Samoa	Girls	1976	18	22.74
American Samoa	Girls	1977	18	23.00
American Samoa	Girls	1978	18	23.27
American Samoa	Girls	1979	18	23.55
American Samoa	Girls	1980	18	23.83
American Samoa	Girls	1981	18	24.13
American Samoa	Girls	1982	18	24.44
American Samoa	Girls	1983	18	24.76
American Samoa	Girls	1984	18	25.10
American Samoa	Girls	1985	18	25.44
American Samoa	Girls	1986	18	25.79
American Samoa	Girls	1987	18	26.15
American Samoa	Girls	1988	18	26.50
American Samoa	Girls	1989	18	26.86
American Samoa	Girls	1990	18	27.22
American Samoa	Girls	1991	18	27.59
American Samoa	Girls	1992	18	27.96
American Samoa	Girls	1993	18	28.33
American Samoa	Girls	1994	18	28.71
American Samoa	Girls	1995	18	29.08
American Samoa	Girls	1996	18	29.46
American Samoa	Girls	1997	18	29.82
American Samoa	Girls	1998	18	30.18
American Samoa	Girls	1999	18	30.54
American Samoa	Girls	2000	18	30.88
American Samoa	Girls	2001	18	31.20
American Samoa	Girls	2002	18	31.51
American Samoa	Girls	2003	18	31.80
American Samoa	Girls	2004	18	32.07
American Samoa	Girls	2005	18	32.34
American Samoa	Girls	2006	18	32.59
American Samoa	Girls	2007	18	32.83
American Samoa	Girls	2008	18	33.07
American Samoa	Girls	2009	18	33.30
American Samoa	Girls	2010	18	33.54
American Samoa	Girls	2011	18	33.77
American Samoa	Girls	2012	18	34.00
American Samoa	Girls	2013	18	34.23
American Samoa	Girls	2014	18	34.46
American Samoa	Girls	2015	18	34.69
American Samoa	Girls	2016	18	34.92
American Samoa	Girls	1975	Average	17.76
American Samoa	Girls	1976	Average	17.96
American Samoa	Girls	1977	Average	18.18
American Samoa	Girls	1978	Average	18.40
American Samoa	Girls	1979	Average	18.62

American Samoa	Girls	1980	Average	18.86
American Samoa	Girls	1981	Average	19.10
American Samoa	Girls	1982	Average	19.36
American Samoa	Girls	1983	Average	19.62
American Samoa	Girls	1984	Average	19.90
American Samoa	Girls	1985	Average	20.19
American Samoa	Girls	1986	Average	20.48
American Samoa	Girls	1987	Average	20.77
American Samoa	Girls	1988	Average	21.07
American Samoa	Girls	1989	Average	21.38
American Samoa	Girls	1990	Average	21.68
American Samoa	Girls	1991	Average	21.99
American Samoa	Girls	1992	Average	22.30
American Samoa	Girls	1993	Average	22.61
American Samoa	Girls	1994	Average	22.93
American Samoa	Girls	1995	Average	23.25
American Samoa	Girls	1996	Average	23.57
American Samoa	Girls	1997	Average	23.89
American Samoa	Girls	1998	Average	24.19
American Samoa	Girls	1999	Average	24.50
American Samoa	Girls	2000	Average	24.80
American Samoa	Girls	2001	Average	25.08
American Samoa	Girls	2002	Average	25.34
American Samoa	Girls	2003	Average	25.59
American Samoa	Girls	2004	Average	25.83
American Samoa	Girls	2005	Average	26.06
American Samoa	Girls	2006	Average	26.28
American Samoa	Girls	2007	Average	26.49
American Samoa	Girls	2008	Average	26.70
American Samoa	Girls	2009	Average	26.90
American Samoa	Girls	2010	Average	27.11
American Samoa	Girls	2011	Average	27.31
American Samoa	Girls	2012	Average	27.51
American Samoa	Girls	2013	Average	27.72
American Samoa	Girls	2014	Average	27.92
American Samoa	Girls	2015	Average	28.12
American Samoa	Girls	2016	Average	28.33
Andorra	Boys	1975	12	10.91
Andorra	Boys	1976	12	11.15
Andorra	Boys	1977	12	11.39
Andorra	Boys	1978	12	11.62
Andorra	Boys	1979	12	11.84
Andorra	Boys	1980	12	12.06
Andorra	Boys	1981	12	12.27
Andorra	Boys	1982	12	12.46
Andorra	Boys	1983	12	12.65
Andorra	Boys	1984	12	12.82
Andorra	Boys	1985	12	13.00
Andorra	Boys	1986	12	13.15
Andorra	Boys	1987	12	13.29
Andorra	Boys	1988	12	13.41
Andorra	Boys	1989	12	13.52

Andorra	Boys	1990	12	13.62
Andorra	Boys	1991	12	13.71
Andorra	Boys	1992	12	13.79
Andorra	Boys	1993	12	13.86
Andorra	Boys	1994	12	13.93
Andorra	Boys	1995	12	13.99
Andorra	Boys	1996	12	14.05
Andorra	Boys	1997	12	14.11
Andorra	Boys	1998	12	14.17
Andorra	Boys	1999	12	14.22
Andorra	Boys	2000	12	14.26
Andorra	Boys	2001	12	14.31
Andorra	Boys	2002	12	14.34
Andorra	Boys	2003	12	14.37
Andorra	Boys	2004	12	14.40
Andorra	Boys	2005	12	14.42
Andorra	Boys	2006	12	14.43
Andorra	Boys	2007	12	14.44
Andorra	Boys	2008	12	14.45
Andorra	Boys	2009	12	14.46
Andorra	Boys	2010	12	14.46
Andorra	Boys	2011	12	14.46
Andorra	Boys	2012	12	14.46
Andorra	Boys	2013	12	14.47
Andorra	Boys	2014	12	14.47
Andorra	Boys	2015	12	14.48
Andorra	Boys	2016	12	14.49
Andorra	Boys	1975	13	13.39
Andorra	Boys	1976	13	13.69
Andorra	Boys	1977	13	13.98
Andorra	Boys	1978	13	14.26
Andorra	Boys	1979	13	14.53
Andorra	Boys	1980	13	14.80
Andorra	Boys	1981	13	15.05
Andorra	Boys	1982	13	15.29
Andorra	Boys	1983	13	15.52
Andorra	Boys	1984	13	15.74
Andorra	Boys	1985	13	15.95
Andorra	Boys	1986	13	16.14
Andorra	Boys	1987	13	16.30
Andorra	Boys	1988	13	16.45
Andorra	Boys	1989	13	16.59
Andorra	Boys	1990	13	16.71
Andorra	Boys	1991	13	16.82
Andorra	Boys	1992	13	16.91
Andorra	Boys	1993	13	17.00
Andorra	Boys	1994	13	17.08
Andorra	Boys	1995	13	17.16
Andorra	Boys	1996	13	17.23
Andorra	Boys	1997	13	17.30
Andorra	Boys	1998	13	17.37
Andorra	Boys	1999	13	17.43

Andorra	Boys	2000	13	17.48
Andorra	Boys	2001	13	17.53
Andorra	Boys	2002	13	17.57
Andorra	Boys	2003	13	17.60
Andorra	Boys	2004	13	17.63
Andorra	Boys	2005	13	17.65
Andorra	Boys	2006	13	17.67
Andorra	Boys	2007	13	17.68
Andorra	Boys	2008	13	17.68
Andorra	Boys	2009	13	17.68
Andorra	Boys	2010	13	17.68
Andorra	Boys	2011	13	17.68
Andorra	Boys	2012	13	17.68
Andorra	Boys	2013	13	17.69
Andorra	Boys	2014	13	17.69
Andorra	Boys	2015	13	17.70
Andorra	Boys	2016	13	17.71
Andorra	Boys	1975	14	16.07
Andorra	Boys	1976	14	16.41
Andorra	Boys	1977	14	16.75
Andorra	Boys	1978	14	17.07
Andorra	Boys	1979	14	17.39
Andorra	Boys	1980	14	17.69
Andorra	Boys	1981	14	17.98
Andorra	Boys	1982	14	18.26
Andorra	Boys	1983	14	18.52
Andorra	Boys	1984	14	18.78
Andorra	Boys	1985	14	19.02
Andorra	Boys	1986	14	19.24
Andorra	Boys	1987	14	19.43
Andorra	Boys	1988	14	19.61
Andorra	Boys	1989	14	19.76
Andorra	Boys	1990	14	19.90
Andorra	Boys	1991	14	20.02
Andorra	Boys	1992	14	20.13
Andorra	Boys	1993	14	20.23
Andorra	Boys	1994	14	20.32
Andorra	Boys	1995	14	20.41
Andorra	Boys	1996	14	20.49
Andorra	Boys	1997	14	20.56
Andorra	Boys	1998	14	20.63
Andorra	Boys	1999	14	20.70
Andorra	Boys	2000	14	20.75
Andorra	Boys	2001	14	20.81
Andorra	Boys	2002	14	20.85
Andorra	Boys	2003	14	20.88
Andorra	Boys	2004	14	20.91
Andorra	Boys	2005	14	20.93
Andorra	Boys	2006	14	20.94
Andorra	Boys	2007	14	20.95
Andorra	Boys	2008	14	20.95
Andorra	Boys	2009	14	20.95

Andorra	Boys	2010	14	20.94
Andorra	Boys	2011	14	20.94
Andorra	Boys	2012	14	20.93
Andorra	Boys	2013	14	20.93
Andorra	Boys	2014	14	20.94
Andorra	Boys	2015	14	20.94
Andorra	Boys	2016	14	20.96
Andorra	Boys	1975	15	18.75
Andorra	Boys	1976	15	19.13
Andorra	Boys	1977	15	19.49
Andorra	Boys	1978	15	19.85
Andorra	Boys	1979	15	20.19
Andorra	Boys	1980	15	20.52
Andorra	Boys	1981	15	20.84
Andorra	Boys	1982	15	21.14
Andorra	Boys	1983	15	21.43
Andorra	Boys	1984	15	21.71
Andorra	Boys	1985	15	21.98
Andorra	Boys	1986	15	22.22
Andorra	Boys	1987	15	22.42
Andorra	Boys	1988	15	22.61
Andorra	Boys	1989	15	22.78
Andorra	Boys	1990	15	22.93
Andorra	Boys	1991	15	23.06
Andorra	Boys	1992	15	23.17
Andorra	Boys	1993	15	23.28
Andorra	Boys	1994	15	23.37
Andorra	Boys	1995	15	23.46
Andorra	Boys	1996	15	23.55
Andorra	Boys	1997	15	23.62
Andorra	Boys	1998	15	23.69
Andorra	Boys	1999	15	23.76
Andorra	Boys	2000	15	23.82
Andorra	Boys	2001	15	23.87
Andorra	Boys	2002	15	23.91
Andorra	Boys	2003	15	23.95
Andorra	Boys	2004	15	23.97
Andorra	Boys	2005	15	23.98
Andorra	Boys	2006	15	23.99
Andorra	Boys	2007	15	23.99
Andorra	Boys	2008	15	23.99
Andorra	Boys	2009	15	23.98
Andorra	Boys	2010	15	23.97
Andorra	Boys	2011	15	23.96
Andorra	Boys	2012	15	23.95
Andorra	Boys	2013	15	23.95
Andorra	Boys	2014	15	23.95
Andorra	Boys	2015	15	23.96
Andorra	Boys	2016	15	23.97
Andorra	Boys	1975	16	21.39
Andorra	Boys	1976	16	21.79
Andorra	Boys	1977	16	22.17

Andorra	Boys	1978	16	22.54
Andorra	Boys	1979	16	22.90
Andorra	Boys	1980	16	23.25
Andorra	Boys	1981	16	23.58
Andorra	Boys	1982	16	23.90
Andorra	Boys	1983	16	24.20
Andorra	Boys	1984	16	24.50
Andorra	Boys	1985	16	24.78
Andorra	Boys	1986	16	25.03
Andorra	Boys	1987	16	25.24
Andorra	Boys	1988	16	25.44
Andorra	Boys	1989	16	25.61
Andorra	Boys	1990	16	25.76
Andorra	Boys	1991	16	25.90
Andorra	Boys	1992	16	26.02
Andorra	Boys	1993	16	26.13
Andorra	Boys	1994	16	26.22
Andorra	Boys	1995	16	26.31
Andorra	Boys	1996	16	26.39
Andorra	Boys	1997	16	26.47
Andorra	Boys	1998	16	26.54
Andorra	Boys	1999	16	26.61
Andorra	Boys	2000	16	26.67
Andorra	Boys	2001	16	26.71
Andorra	Boys	2002	16	26.75
Andorra	Boys	2003	16	26.78
Andorra	Boys	2004	16	26.80
Andorra	Boys	2005	16	26.81
Andorra	Boys	2006	16	26.82
Andorra	Boys	2007	16	26.81
Andorra	Boys	2008	16	26.80
Andorra	Boys	2009	16	26.79
Andorra	Boys	2010	16	26.77
Andorra	Boys	2011	16	26.76
Andorra	Boys	2012	16	26.75
Andorra	Boys	2013	16	26.74
Andorra	Boys	2014	16	26.74
Andorra	Boys	2015	16	26.74
Andorra	Boys	2016	16	26.76
Andorra	Boys	1975	17	24.01
Andorra	Boys	1976	17	24.42
Andorra	Boys	1977	17	24.81
Andorra	Boys	1978	17	25.19
Andorra	Boys	1979	17	25.56
Andorra	Boys	1980	17	25.92
Andorra	Boys	1981	17	26.26
Andorra	Boys	1982	17	26.59
Andorra	Boys	1983	17	26.90
Andorra	Boys	1984	17	27.20
Andorra	Boys	1985	17	27.49
Andorra	Boys	1986	17	27.75
Andorra	Boys	1987	17	27.97

Andorra	Boys	1988	17	28.17
Andorra	Boys	1989	17	28.35
Andorra	Boys	1990	17	28.50
Andorra	Boys	1991	17	28.64
Andorra	Boys	1992	17	28.76
Andorra	Boys	1993	17	28.87
Andorra	Boys	1994	17	28.97
Andorra	Boys	1995	17	29.06
Andorra	Boys	1996	17	29.14
Andorra	Boys	1997	17	29.22
Andorra	Boys	1998	17	29.29
Andorra	Boys	1999	17	29.35
Andorra	Boys	2000	17	29.41
Andorra	Boys	2001	17	29.45
Andorra	Boys	2002	17	29.49
Andorra	Boys	2003	17	29.52
Andorra	Boys	2004	17	29.53
Andorra	Boys	2005	17	29.54
Andorra	Boys	2006	17	29.54
Andorra	Boys	2007	17	29.53
Andorra	Boys	2008	17	29.52
Andorra	Boys	2009	17	29.50
Andorra	Boys	2010	17	29.48
Andorra	Boys	2011	17	29.47
Andorra	Boys	2012	17	29.45
Andorra	Boys	2013	17	29.44
Andorra	Boys	2014	17	29.44
Andorra	Boys	2015	17	29.44
Andorra	Boys	2016	17	29.46
Andorra	Boys	1975	18	26.69
Andorra	Boys	1976	18	27.11
Andorra	Boys	1977	18	27.51
Andorra	Boys	1978	18	27.90
Andorra	Boys	1979	18	28.28
Andorra	Boys	1980	18	28.66
Andorra	Boys	1981	18	29.01
Andorra	Boys	1982	18	29.35
Andorra	Boys	1983	18	29.67
Andorra	Boys	1984	18	29.98
Andorra	Boys	1985	18	30.28
Andorra	Boys	1986	18	30.54
Andorra	Boys	1987	18	30.77
Andorra	Boys	1988	18	30.97
Andorra	Boys	1989	18	31.15
Andorra	Boys	1990	18	31.31
Andorra	Boys	1991	18	31.46
Andorra	Boys	1992	18	31.58
Andorra	Boys	1993	18	31.69
Andorra	Boys	1994	18	31.79
Andorra	Boys	1995	18	31.88
Andorra	Boys	1996	18	31.96
Andorra	Boys	1997	18	32.04

Andorra	Boys	1998	18	32.11
Andorra	Boys	1999	18	32.18
Andorra	Boys	2000	18	32.23
Andorra	Boys	2001	18	32.28
Andorra	Boys	2002	18	32.32
Andorra	Boys	2003	18	32.35
Andorra	Boys	2004	18	32.36
Andorra	Boys	2005	18	32.37
Andorra	Boys	2006	18	32.37
Andorra	Boys	2007	18	32.36
Andorra	Boys	2008	18	32.34
Andorra	Boys	2009	18	32.33
Andorra	Boys	2010	18	32.31
Andorra	Boys	2011	18	32.29
Andorra	Boys	2012	18	32.27
Andorra	Boys	2013	18	32.26
Andorra	Boys	2014	18	32.26
Andorra	Boys	2015	18	32.26
Andorra	Boys	2016	18	32.27
Andorra	Boys	1975	Average	18.74
Andorra	Boys	1976	Average	19.10
Andorra	Boys	1977	Average	19.44
Andorra	Boys	1978	Average	19.78
Andorra	Boys	1979	Average	20.10
Andorra	Boys	1980	Average	20.42
Andorra	Boys	1981	Average	20.71
Andorra	Boys	1982	Average	21.00
Andorra	Boys	1983	Average	21.27
Andorra	Boys	1984	Average	21.53
Andorra	Boys	1985	Average	21.79
Andorra	Boys	1986	Average	22.01
Andorra	Boys	1987	Average	22.20
Andorra	Boys	1988	Average	22.38
Andorra	Boys	1989	Average	22.54
Andorra	Boys	1990	Average	22.68
Andorra	Boys	1991	Average	22.80
Andorra	Boys	1992	Average	22.91
Andorra	Boys	1993	Average	23.01
Andorra	Boys	1994	Average	23.10
Andorra	Boys	1995	Average	23.18
Andorra	Boys	1996	Average	23.26
Andorra	Boys	1997	Average	23.33
Andorra	Boys	1998	Average	23.40
Andorra	Boys	1999	Average	23.46
Andorra	Boys	2000	Average	23.52
Andorra	Boys	2001	Average	23.57
Andorra	Boys	2002	Average	23.61
Andorra	Boys	2003	Average	23.64
Andorra	Boys	2004	Average	23.66
Andorra	Boys	2005	Average	23.67
Andorra	Boys	2006	Average	23.68
Andorra	Boys	2007	Average	23.68

Andorra	Boys	2008	Average	23.68
Andorra	Boys	2009	Average	23.67
Andorra	Boys	2010	Average	23.66
Andorra	Boys	2011	Average	23.65
Andorra	Boys	2012	Average	23.64
Andorra	Boys	2013	Average	23.64
Andorra	Boys	2014	Average	23.64
Andorra	Boys	2015	Average	23.65
Andorra	Boys	2016	Average	23.66
Andorra	Girls	1975	12	9.72
Andorra	Girls	1976	12	9.89
Andorra	Girls	1977	12	10.05
Andorra	Girls	1978	12	10.21
Andorra	Girls	1979	12	10.35
Andorra	Girls	1980	12	10.49
Andorra	Girls	1981	12	10.62
Andorra	Girls	1982	12	10.74
Andorra	Girls	1983	12	10.85
Andorra	Girls	1984	12	10.96
Andorra	Girls	1985	12	11.06
Andorra	Girls	1986	12	11.15
Andorra	Girls	1987	12	11.22
Andorra	Girls	1988	12	11.28
Andorra	Girls	1989	12	11.34
Andorra	Girls	1990	12	11.39
Andorra	Girls	1991	12	11.43
Andorra	Girls	1992	12	11.46
Andorra	Girls	1993	12	11.50
Andorra	Girls	1994	12	11.53
Andorra	Girls	1995	12	11.56
Andorra	Girls	1996	12	11.59
Andorra	Girls	1997	12	11.61
Andorra	Girls	1998	12	11.64
Andorra	Girls	1999	12	11.67
Andorra	Girls	2000	12	11.69
Andorra	Girls	2001	12	11.72
Andorra	Girls	2002	12	11.74
Andorra	Girls	2003	12	11.76
Andorra	Girls	2004	12	11.78
Andorra	Girls	2005	12	11.79
Andorra	Girls	2006	12	11.80
Andorra	Girls	2007	12	11.81
Andorra	Girls	2008	12	11.82
Andorra	Girls	2009	12	11.83
Andorra	Girls	2010	12	11.85
Andorra	Girls	2011	12	11.86
Andorra	Girls	2012	12	11.87
Andorra	Girls	2013	12	11.89
Andorra	Girls	2014	12	11.90
Andorra	Girls	2015	12	11.92
Andorra	Girls	2016	12	11.95
Andorra	Girls	1975	13	11.75

Andorra	Girls	1976	13	11.95
Andorra	Girls	1977	13	12.13
Andorra	Girls	1978	13	12.31
Andorra	Girls	1979	13	12.48
Andorra	Girls	1980	13	12.64
Andorra	Girls	1981	13	12.79
Andorra	Girls	1982	13	12.93
Andorra	Girls	1983	13	13.06
Andorra	Girls	1984	13	13.18
Andorra	Girls	1985	13	13.29
Andorra	Girls	1986	13	13.39
Andorra	Girls	1987	13	13.48
Andorra	Girls	1988	13	13.55
Andorra	Girls	1989	13	13.61
Andorra	Girls	1990	13	13.67
Andorra	Girls	1991	13	13.71
Andorra	Girls	1992	13	13.75
Andorra	Girls	1993	13	13.79
Andorra	Girls	1994	13	13.82
Andorra	Girls	1995	13	13.85
Andorra	Girls	1996	13	13.89
Andorra	Girls	1997	13	13.92
Andorra	Girls	1998	13	13.95
Andorra	Girls	1999	13	13.98
Andorra	Girls	2000	13	14.01
Andorra	Girls	2001	13	14.03
Andorra	Girls	2002	13	14.06
Andorra	Girls	2003	13	14.08
Andorra	Girls	2004	13	14.09
Andorra	Girls	2005	13	14.11
Andorra	Girls	2006	13	14.12
Andorra	Girls	2007	13	14.13
Andorra	Girls	2008	13	14.14
Andorra	Girls	2009	13	14.15
Andorra	Girls	2010	13	14.17
Andorra	Girls	2011	13	14.18
Andorra	Girls	2012	13	14.19
Andorra	Girls	2013	13	14.21
Andorra	Girls	2014	13	14.23
Andorra	Girls	2015	13	14.26
Andorra	Girls	2016	13	14.29
Andorra	Girls	1975	14	13.71
Andorra	Girls	1976	14	13.93
Andorra	Girls	1977	14	14.14
Andorra	Girls	1978	14	14.33
Andorra	Girls	1979	14	14.51
Andorra	Girls	1980	14	14.69
Andorra	Girls	1981	14	14.85
Andorra	Girls	1982	14	15.00
Andorra	Girls	1983	14	15.14
Andorra	Girls	1984	14	15.28
Andorra	Girls	1985	14	15.40

Andorra	Girls	1986	14	15.51
Andorra	Girls	1987	14	15.60
Andorra	Girls	1988	14	15.68
Andorra	Girls	1989	14	15.75
Andorra	Girls	1990	14	15.80
Andorra	Girls	1991	14	15.85
Andorra	Girls	1992	14	15.90
Andorra	Girls	1993	14	15.93
Andorra	Girls	1994	14	15.97
Andorra	Girls	1995	14	16.00
Andorra	Girls	1996	14	16.03
Andorra	Girls	1997	14	16.06
Andorra	Girls	1998	14	16.10
Andorra	Girls	1999	14	16.13
Andorra	Girls	2000	14	16.16
Andorra	Girls	2001	14	16.18
Andorra	Girls	2002	14	16.21
Andorra	Girls	2003	14	16.23
Andorra	Girls	2004	14	16.24
Andorra	Girls	2005	14	16.26
Andorra	Girls	2006	14	16.27
Andorra	Girls	2007	14	16.28
Andorra	Girls	2008	14	16.29
Andorra	Girls	2009	14	16.30
Andorra	Girls	2010	14	16.32
Andorra	Girls	2011	14	16.33
Andorra	Girls	2012	14	16.34
Andorra	Girls	2013	14	16.36
Andorra	Girls	2014	14	16.38
Andorra	Girls	2015	14	16.41
Andorra	Girls	2016	14	16.44
Andorra	Girls	1975	15	15.40
Andorra	Girls	1976	15	15.63
Andorra	Girls	1977	15	15.85
Andorra	Girls	1978	15	16.05
Andorra	Girls	1979	15	16.24
Andorra	Girls	1980	15	16.43
Andorra	Girls	1981	15	16.60
Andorra	Girls	1982	15	16.75
Andorra	Girls	1983	15	16.90
Andorra	Girls	1984	15	17.04
Andorra	Girls	1985	15	17.17
Andorra	Girls	1986	15	17.29
Andorra	Girls	1987	15	17.38
Andorra	Girls	1988	15	17.46
Andorra	Girls	1989	15	17.53
Andorra	Girls	1990	15	17.59
Andorra	Girls	1991	15	17.64
Andorra	Girls	1992	15	17.68
Andorra	Girls	1993	15	17.72
Andorra	Girls	1994	15	17.75
Andorra	Girls	1995	15	17.79

Andorra	Girls	1996	15	17.82
Andorra	Girls	1997	15	17.85
Andorra	Girls	1998	15	17.88
Andorra	Girls	1999	15	17.91
Andorra	Girls	2000	15	17.94
Andorra	Girls	2001	15	17.97
Andorra	Girls	2002	15	17.99
Andorra	Girls	2003	15	18.01
Andorra	Girls	2004	15	18.03
Andorra	Girls	2005	15	18.04
Andorra	Girls	2006	15	18.05
Andorra	Girls	2007	15	18.06
Andorra	Girls	2008	15	18.07
Andorra	Girls	2009	15	18.08
Andorra	Girls	2010	15	18.09
Andorra	Girls	2011	15	18.11
Andorra	Girls	2012	15	18.12
Andorra	Girls	2013	15	18.14
Andorra	Girls	2014	15	18.16
Andorra	Girls	2015	15	18.19
Andorra	Girls	2016	15	18.23
Andorra	Girls	1975	16	16.72
Andorra	Girls	1976	16	16.96
Andorra	Girls	1977	16	17.18
Andorra	Girls	1978	16	17.39
Andorra	Girls	1979	16	17.59
Andorra	Girls	1980	16	17.78
Andorra	Girls	1981	16	17.96
Andorra	Girls	1982	16	18.12
Andorra	Girls	1983	16	18.27
Andorra	Girls	1984	16	18.41
Andorra	Girls	1985	16	18.55
Andorra	Girls	1986	16	18.67
Andorra	Girls	1987	16	18.76
Andorra	Girls	1988	16	18.85
Andorra	Girls	1989	16	18.92
Andorra	Girls	1990	16	18.98
Andorra	Girls	1991	16	19.03
Andorra	Girls	1992	16	19.07
Andorra	Girls	1993	16	19.11
Andorra	Girls	1994	16	19.14
Andorra	Girls	1995	16	19.18
Andorra	Girls	1996	16	19.21
Andorra	Girls	1997	16	19.24
Andorra	Girls	1998	16	19.27
Andorra	Girls	1999	16	19.30
Andorra	Girls	2000	16	19.33
Andorra	Girls	2001	16	19.36
Andorra	Girls	2002	16	19.38
Andorra	Girls	2003	16	19.40
Andorra	Girls	2004	16	19.41
Andorra	Girls	2005	16	19.43

Andorra	Girls	2006	16	19.44
Andorra	Girls	2007	16	19.45
Andorra	Girls	2008	16	19.46
Andorra	Girls	2009	16	19.47
Andorra	Girls	2010	16	19.48
Andorra	Girls	2011	16	19.49
Andorra	Girls	2012	16	19.50
Andorra	Girls	2013	16	19.52
Andorra	Girls	2014	16	19.55
Andorra	Girls	2015	16	19.58
Andorra	Girls	2016	16	19.61
Andorra	Girls	1975	17	17.77
Andorra	Girls	1976	17	18.02
Andorra	Girls	1977	17	18.25
Andorra	Girls	1978	17	18.47
Andorra	Girls	1979	17	18.67
Andorra	Girls	1980	17	18.87
Andorra	Girls	1981	17	19.06
Andorra	Girls	1982	17	19.22
Andorra	Girls	1983	17	19.38
Andorra	Girls	1984	17	19.53
Andorra	Girls	1985	17	19.67
Andorra	Girls	1986	17	19.79
Andorra	Girls	1987	17	19.90
Andorra	Girls	1988	17	19.98
Andorra	Girls	1989	17	20.05
Andorra	Girls	1990	17	20.12
Andorra	Girls	1991	17	20.17
Andorra	Girls	1992	17	20.21
Andorra	Girls	1993	17	20.25
Andorra	Girls	1994	17	20.29
Andorra	Girls	1995	17	20.32
Andorra	Girls	1996	17	20.36
Andorra	Girls	1997	17	20.39
Andorra	Girls	1998	17	20.42
Andorra	Girls	1999	17	20.45
Andorra	Girls	2000	17	20.48
Andorra	Girls	2001	17	20.51
Andorra	Girls	2002	17	20.53
Andorra	Girls	2003	17	20.55
Andorra	Girls	2004	17	20.57
Andorra	Girls	2005	17	20.59
Andorra	Girls	2006	17	20.60
Andorra	Girls	2007	17	20.61
Andorra	Girls	2008	17	20.62
Andorra	Girls	2009	17	20.63
Andorra	Girls	2010	17	20.64
Andorra	Girls	2011	17	20.65
Andorra	Girls	2012	17	20.67
Andorra	Girls	2013	17	20.69
Andorra	Girls	2014	17	20.71
Andorra	Girls	2015	17	20.74

Andorra	Girls	2016	17	20.78
Andorra	Girls	1975	18	18.78
Andorra	Girls	1976	18	19.05
Andorra	Girls	1977	18	19.29
Andorra	Girls	1978	18	19.53
Andorra	Girls	1979	18	19.75
Andorra	Girls	1980	18	19.96
Andorra	Girls	1981	18	20.16
Andorra	Girls	1982	18	20.34
Andorra	Girls	1983	18	20.51
Andorra	Girls	1984	18	20.67
Andorra	Girls	1985	18	20.82
Andorra	Girls	1986	18	20.95
Andorra	Girls	1987	18	21.06
Andorra	Girls	1988	18	21.15
Andorra	Girls	1989	18	21.23
Andorra	Girls	1990	18	21.30
Andorra	Girls	1991	18	21.35
Andorra	Girls	1992	18	21.40
Andorra	Girls	1993	18	21.45
Andorra	Girls	1994	18	21.49
Andorra	Girls	1995	18	21.52
Andorra	Girls	1996	18	21.56
Andorra	Girls	1997	18	21.60
Andorra	Girls	1998	18	21.63
Andorra	Girls	1999	18	21.67
Andorra	Girls	2000	18	21.70
Andorra	Girls	2001	18	21.73
Andorra	Girls	2002	18	21.76
Andorra	Girls	2003	18	21.78
Andorra	Girls	2004	18	21.80
Andorra	Girls	2005	18	21.82
Andorra	Girls	2006	18	21.83
Andorra	Girls	2007	18	21.84
Andorra	Girls	2008	18	21.85
Andorra	Girls	2009	18	21.87
Andorra	Girls	2010	18	21.88
Andorra	Girls	2011	18	21.89
Andorra	Girls	2012	18	21.91
Andorra	Girls	2013	18	21.93
Andorra	Girls	2014	18	21.96
Andorra	Girls	2015	18	21.99
Andorra	Girls	2016	18	22.03
Andorra	Girls	1975	Average	14.84
Andorra	Girls	1976	Average	15.06
Andorra	Girls	1977	Average	15.27
Andorra	Girls	1978	Average	15.47
Andorra	Girls	1979	Average	15.66
Andorra	Girls	1980	Average	15.84
Andorra	Girls	1981	Average	16.00
Andorra	Girls	1982	Average	16.16
Andorra	Girls	1983	Average	16.30

Andorra	Girls	1984	Average	16.44
Andorra	Girls	1985	Average	16.57
Andorra	Girls	1986	Average	16.68
Andorra	Girls	1987	Average	16.77
Andorra	Girls	1988	Average	16.85
Andorra	Girls	1989	Average	16.92
Andorra	Girls	1990	Average	16.98
Andorra	Girls	1991	Average	17.03
Andorra	Girls	1992	Average	17.07
Andorra	Girls	1993	Average	17.11
Andorra	Girls	1994	Average	17.14
Andorra	Girls	1995	Average	17.18
Andorra	Girls	1996	Average	17.21
Andorra	Girls	1997	Average	17.24
Andorra	Girls	1998	Average	17.27
Andorra	Girls	1999	Average	17.30
Andorra	Girls	2000	Average	17.33
Andorra	Girls	2001	Average	17.36
Andorra	Girls	2002	Average	17.38
Andorra	Girls	2003	Average	17.40
Andorra	Girls	2004	Average	17.42
Andorra	Girls	2005	Average	17.43
Andorra	Girls	2006	Average	17.44
Andorra	Girls	2007	Average	17.46
Andorra	Girls	2008	Average	17.47
Andorra	Girls	2009	Average	17.48
Andorra	Girls	2010	Average	17.49
Andorra	Girls	2011	Average	17.50
Andorra	Girls	2012	Average	17.52
Andorra	Girls	2013	Average	17.53
Andorra	Girls	2014	Average	17.56
Andorra	Girls	2015	Average	17.58
Andorra	Girls	2016	Average	17.62
Angola	Boys	1975	12	3.91
Angola	Boys	1976	12	3.92
Angola	Boys	1977	12	3.94
Angola	Boys	1978	12	3.95
Angola	Boys	1979	12	3.97
Angola	Boys	1980	12	3.98
Angola	Boys	1981	12	4.00
Angola	Boys	1982	12	4.02
Angola	Boys	1983	12	4.04
Angola	Boys	1984	12	4.06
Angola	Boys	1985	12	4.08
Angola	Boys	1986	12	4.10
Angola	Boys	1987	12	4.12
Angola	Boys	1988	12	4.14
Angola	Boys	1989	12	4.17
Angola	Boys	1990	12	4.20
Angola	Boys	1991	12	4.22
Angola	Boys	1992	12	4.25
Angola	Boys	1993	12	4.29

Angola	Boys	1994	12	4.32
Angola	Boys	1995	12	4.36
Angola	Boys	1996	12	4.39
Angola	Boys	1997	12	4.44
Angola	Boys	1998	12	4.48
Angola	Boys	1999	12	4.53
Angola	Boys	2000	12	4.58
Angola	Boys	2001	12	4.63
Angola	Boys	2002	12	4.69
Angola	Boys	2003	12	4.76
Angola	Boys	2004	12	4.82
Angola	Boys	2005	12	4.90
Angola	Boys	2006	12	4.97
Angola	Boys	2007	12	5.05
Angola	Boys	2008	12	5.14
Angola	Boys	2009	12	5.23
Angola	Boys	2010	12	5.33
Angola	Boys	2011	12	5.43
Angola	Boys	2012	12	5.54
Angola	Boys	2013	12	5.65
Angola	Boys	2014	12	5.77
Angola	Boys	2015	12	5.90
Angola	Boys	2016	12	6.03
Angola	Boys	1975	13	4.89
Angola	Boys	1976	13	4.90
Angola	Boys	1977	13	4.92
Angola	Boys	1978	13	4.94
Angola	Boys	1979	13	4.96
Angola	Boys	1980	13	4.98
Angola	Boys	1981	13	5.00
Angola	Boys	1982	13	5.03
Angola	Boys	1983	13	5.05
Angola	Boys	1984	13	5.07
Angola	Boys	1985	13	5.10
Angola	Boys	1986	13	5.12
Angola	Boys	1987	13	5.15
Angola	Boys	1988	13	5.18
Angola	Boys	1989	13	5.21
Angola	Boys	1990	13	5.24
Angola	Boys	1991	13	5.28
Angola	Boys	1992	13	5.31
Angola	Boys	1993	13	5.35
Angola	Boys	1994	13	5.39
Angola	Boys	1995	13	5.43
Angola	Boys	1996	13	5.48
Angola	Boys	1997	13	5.53
Angola	Boys	1998	13	5.58
Angola	Boys	1999	13	5.64
Angola	Boys	2000	13	5.70
Angola	Boys	2001	13	5.76
Angola	Boys	2002	13	5.83
Angola	Boys	2003	13	5.90

Angola	Boys	2004	13	5.98
Angola	Boys	2005	13	6.07
Angola	Boys	2006	13	6.16
Angola	Boys	2007	13	6.25
Angola	Boys	2008	13	6.35
Angola	Boys	2009	13	6.46
Angola	Boys	2010	13	6.57
Angola	Boys	2011	13	6.69
Angola	Boys	2012	13	6.81
Angola	Boys	2013	13	6.94
Angola	Boys	2014	13	7.08
Angola	Boys	2015	13	7.23
Angola	Boys	2016	13	7.39
Angola	Boys	1975	14	6.18
Angola	Boys	1976	14	6.20
Angola	Boys	1977	14	6.23
Angola	Boys	1978	14	6.25
Angola	Boys	1979	14	6.28
Angola	Boys	1980	14	6.30
Angola	Boys	1981	14	6.33
Angola	Boys	1982	14	6.35
Angola	Boys	1983	14	6.38
Angola	Boys	1984	14	6.41
Angola	Boys	1985	14	6.44
Angola	Boys	1986	14	6.47
Angola	Boys	1987	14	6.51
Angola	Boys	1988	14	6.54
Angola	Boys	1989	14	6.58
Angola	Boys	1990	14	6.61
Angola	Boys	1991	14	6.65
Angola	Boys	1992	14	6.69
Angola	Boys	1993	14	6.74
Angola	Boys	1994	14	6.78
Angola	Boys	1995	14	6.83
Angola	Boys	1996	14	6.89
Angola	Boys	1997	14	6.94
Angola	Boys	1998	14	7.00
Angola	Boys	1999	14	7.07
Angola	Boys	2000	14	7.13
Angola	Boys	2001	14	7.21
Angola	Boys	2002	14	7.29
Angola	Boys	2003	14	7.37
Angola	Boys	2004	14	7.46
Angola	Boys	2005	14	7.55
Angola	Boys	2006	14	7.65
Angola	Boys	2007	14	7.76
Angola	Boys	2008	14	7.87
Angola	Boys	2009	14	7.98
Angola	Boys	2010	14	8.11
Angola	Boys	2011	14	8.24
Angola	Boys	2012	14	8.38
Angola	Boys	2013	14	8.52

Angola	Boys	2014	14	8.68
Angola	Boys	2015	14	8.84
Angola	Boys	2016	14	9.01
Angola	Boys	1975	15	7.73
Angola	Boys	1976	15	7.76
Angola	Boys	1977	15	7.79
Angola	Boys	1978	15	7.82
Angola	Boys	1979	15	7.85
Angola	Boys	1980	15	7.88
Angola	Boys	1981	15	7.92
Angola	Boys	1982	15	7.95
Angola	Boys	1983	15	7.98
Angola	Boys	1984	15	8.02
Angola	Boys	1985	15	8.05
Angola	Boys	1986	15	8.09
Angola	Boys	1987	15	8.13
Angola	Boys	1988	15	8.17
Angola	Boys	1989	15	8.21
Angola	Boys	1990	15	8.25
Angola	Boys	1991	15	8.30
Angola	Boys	1992	15	8.34
Angola	Boys	1993	15	8.39
Angola	Boys	1994	15	8.45
Angola	Boys	1995	15	8.50
Angola	Boys	1996	15	8.56
Angola	Boys	1997	15	8.62
Angola	Boys	1998	15	8.69
Angola	Boys	1999	15	8.76
Angola	Boys	2000	15	8.83
Angola	Boys	2001	15	8.91
Angola	Boys	2002	15	9.00
Angola	Boys	2003	15	9.09
Angola	Boys	2004	15	9.19
Angola	Boys	2005	15	9.29
Angola	Boys	2006	15	9.40
Angola	Boys	2007	15	9.51
Angola	Boys	2008	15	9.63
Angola	Boys	2009	15	9.75
Angola	Boys	2010	15	9.89
Angola	Boys	2011	15	10.03
Angola	Boys	2012	15	10.17
Angola	Boys	2013	15	10.33
Angola	Boys	2014	15	10.49
Angola	Boys	2015	15	10.67
Angola	Boys	2016	15	10.85
Angola	Boys	1975	16	9.46
Angola	Boys	1976	16	9.50
Angola	Boys	1977	16	9.54
Angola	Boys	1978	16	9.57
Angola	Boys	1979	16	9.61
Angola	Boys	1980	16	9.65
Angola	Boys	1981	16	9.69

Angola	Boys	1982	16	9.73
Angola	Boys	1983	16	9.77
Angola	Boys	1984	16	9.81
Angola	Boys	1985	16	9.85
Angola	Boys	1986	16	9.89
Angola	Boys	1987	16	9.93
Angola	Boys	1988	16	9.98
Angola	Boys	1989	16	10.03
Angola	Boys	1990	16	10.08
Angola	Boys	1991	16	10.13
Angola	Boys	1992	16	10.18
Angola	Boys	1993	16	10.24
Angola	Boys	1994	16	10.29
Angola	Boys	1995	16	10.35
Angola	Boys	1996	16	10.42
Angola	Boys	1997	16	10.49
Angola	Boys	1998	16	10.56
Angola	Boys	1999	16	10.64
Angola	Boys	2000	16	10.72
Angola	Boys	2001	16	10.80
Angola	Boys	2002	16	10.90
Angola	Boys	2003	16	10.99
Angola	Boys	2004	16	11.10
Angola	Boys	2005	16	11.20
Angola	Boys	2006	16	11.32
Angola	Boys	2007	16	11.44
Angola	Boys	2008	16	11.56
Angola	Boys	2009	16	11.70
Angola	Boys	2010	16	11.84
Angola	Boys	2011	16	11.98
Angola	Boys	2012	16	12.14
Angola	Boys	2013	16	12.30
Angola	Boys	2014	16	12.47
Angola	Boys	2015	16	12.65
Angola	Boys	2016	16	12.83
Angola	Boys	1975	17	11.26
Angola	Boys	1976	17	11.31
Angola	Boys	1977	17	11.35
Angola	Boys	1978	17	11.39
Angola	Boys	1979	17	11.44
Angola	Boys	1980	17	11.48
Angola	Boys	1981	17	11.53
Angola	Boys	1982	17	11.57
Angola	Boys	1983	17	11.62
Angola	Boys	1984	17	11.67
Angola	Boys	1985	17	11.72
Angola	Boys	1986	17	11.77
Angola	Boys	1987	17	11.82
Angola	Boys	1988	17	11.87
Angola	Boys	1989	17	11.92
Angola	Boys	1990	17	11.98
Angola	Boys	1991	17	12.03

Angola	Boys	1992	17	12.09
Angola	Boys	1993	17	12.15
Angola	Boys	1994	17	12.22
Angola	Boys	1995	17	12.28
Angola	Boys	1996	17	12.35
Angola	Boys	1997	17	12.43
Angola	Boys	1998	17	12.51
Angola	Boys	1999	17	12.59
Angola	Boys	2000	17	12.68
Angola	Boys	2001	17	12.77
Angola	Boys	2002	17	12.87
Angola	Boys	2003	17	12.98
Angola	Boys	2004	17	13.09
Angola	Boys	2005	17	13.20
Angola	Boys	2006	17	13.32
Angola	Boys	2007	17	13.45
Angola	Boys	2008	17	13.58
Angola	Boys	2009	17	13.72
Angola	Boys	2010	17	13.87
Angola	Boys	2011	17	14.02
Angola	Boys	2012	17	14.18
Angola	Boys	2013	17	14.35
Angola	Boys	2014	17	14.52
Angola	Boys	2015	17	14.71
Angola	Boys	2016	17	14.90
Angola	Boys	1975	18	13.02
Angola	Boys	1976	18	13.07
Angola	Boys	1977	18	13.12
Angola	Boys	1978	18	13.17
Angola	Boys	1979	18	13.22
Angola	Boys	1980	18	13.28
Angola	Boys	1981	18	13.33
Angola	Boys	1982	18	13.38
Angola	Boys	1983	18	13.44
Angola	Boys	1984	18	13.49
Angola	Boys	1985	18	13.55
Angola	Boys	1986	18	13.60
Angola	Boys	1987	18	13.66
Angola	Boys	1988	18	13.72
Angola	Boys	1989	18	13.78
Angola	Boys	1990	18	13.84
Angola	Boys	1991	18	13.91
Angola	Boys	1992	18	13.97
Angola	Boys	1993	18	14.04
Angola	Boys	1994	18	14.11
Angola	Boys	1995	18	14.19
Angola	Boys	1996	18	14.27
Angola	Boys	1997	18	14.35
Angola	Boys	1998	18	14.44
Angola	Boys	1999	18	14.53
Angola	Boys	2000	18	14.63
Angola	Boys	2001	18	14.73

Angola	Boys	2002	18	14.84
Angola	Boys	2003	18	14.95
Angola	Boys	2004	18	15.07
Angola	Boys	2005	18	15.20
Angola	Boys	2006	18	15.33
Angola	Boys	2007	18	15.47
Angola	Boys	2008	18	15.61
Angola	Boys	2009	18	15.76
Angola	Boys	2010	18	15.92
Angola	Boys	2011	18	16.08
Angola	Boys	2012	18	16.25
Angola	Boys	2013	18	16.43
Angola	Boys	2014	18	16.61
Angola	Boys	2015	18	16.81
Angola	Boys	2016	18	17.01
Angola	Boys	1975	Average	8.06
Angola	Boys	1976	Average	8.09
Angola	Boys	1977	Average	8.13
Angola	Boys	1978	Average	8.16
Angola	Boys	1979	Average	8.19
Angola	Boys	1980	Average	8.22
Angola	Boys	1981	Average	8.26
Angola	Boys	1982	Average	8.29
Angola	Boys	1983	Average	8.32
Angola	Boys	1984	Average	8.36
Angola	Boys	1985	Average	8.40
Angola	Boys	1986	Average	8.43
Angola	Boys	1987	Average	8.47
Angola	Boys	1988	Average	8.51
Angola	Boys	1989	Average	8.56
Angola	Boys	1990	Average	8.60
Angola	Boys	1991	Average	8.65
Angola	Boys	1992	Average	8.69
Angola	Boys	1993	Average	8.74
Angola	Boys	1994	Average	8.79
Angola	Boys	1995	Average	8.85
Angola	Boys	1996	Average	8.91
Angola	Boys	1997	Average	8.97
Angola	Boys	1998	Average	9.04
Angola	Boys	1999	Average	9.11
Angola	Boys	2000	Average	9.18
Angola	Boys	2001	Average	9.26
Angola	Boys	2002	Average	9.34
Angola	Boys	2003	Average	9.43
Angola	Boys	2004	Average	9.53
Angola	Boys	2005	Average	9.63
Angola	Boys	2006	Average	9.73
Angola	Boys	2007	Average	9.85
Angola	Boys	2008	Average	9.96
Angola	Boys	2009	Average	10.09
Angola	Boys	2010	Average	10.22
Angola	Boys	2011	Average	10.35

Angola	Boys	2012	Average	10.50
Angola	Boys	2013	Average	10.65
Angola	Boys	2014	Average	10.80
Angola	Boys	2015	Average	10.97
Angola	Boys	2016	Average	11.15
Angola	Girls	1975	12	4.72
Angola	Girls	1976	12	4.75
Angola	Girls	1977	12	4.78
Angola	Girls	1978	12	4.80
Angola	Girls	1979	12	4.83
Angola	Girls	1980	12	4.86
Angola	Girls	1981	12	4.89
Angola	Girls	1982	12	4.92
Angola	Girls	1983	12	4.96
Angola	Girls	1984	12	4.99
Angola	Girls	1985	12	5.03
Angola	Girls	1986	12	5.07
Angola	Girls	1987	12	5.11
Angola	Girls	1988	12	5.16
Angola	Girls	1989	12	5.20
Angola	Girls	1990	12	5.25
Angola	Girls	1991	12	5.30
Angola	Girls	1992	12	5.36
Angola	Girls	1993	12	5.41
Angola	Girls	1994	12	5.47
Angola	Girls	1995	12	5.54
Angola	Girls	1996	12	5.60
Angola	Girls	1997	12	5.67
Angola	Girls	1998	12	5.75
Angola	Girls	1999	12	5.82
Angola	Girls	2000	12	5.91
Angola	Girls	2001	12	5.99
Angola	Girls	2002	12	6.08
Angola	Girls	2003	12	6.17
Angola	Girls	2004	12	6.27
Angola	Girls	2005	12	6.36
Angola	Girls	2006	12	6.47
Angola	Girls	2007	12	6.57
Angola	Girls	2008	12	6.68
Angola	Girls	2009	12	6.79
Angola	Girls	2010	12	6.90
Angola	Girls	2011	12	7.02
Angola	Girls	2012	12	7.14
Angola	Girls	2013	12	7.26
Angola	Girls	2014	12	7.39
Angola	Girls	2015	12	7.51
Angola	Girls	2016	12	7.65
Angola	Girls	1975	13	5.95
Angola	Girls	1976	13	5.98
Angola	Girls	1977	13	6.01
Angola	Girls	1978	13	6.05
Angola	Girls	1979	13	6.08

Angola	Girls	1980	13	6.12
Angola	Girls	1981	13	6.15
Angola	Girls	1982	13	6.19
Angola	Girls	1983	13	6.23
Angola	Girls	1984	13	6.28
Angola	Girls	1985	13	6.32
Angola	Girls	1986	13	6.37
Angola	Girls	1987	13	6.42
Angola	Girls	1988	13	6.47
Angola	Girls	1989	13	6.53
Angola	Girls	1990	13	6.59
Angola	Girls	1991	13	6.65
Angola	Girls	1992	13	6.71
Angola	Girls	1993	13	6.78
Angola	Girls	1994	13	6.85
Angola	Girls	1995	13	6.92
Angola	Girls	1996	13	7.00
Angola	Girls	1997	13	7.08
Angola	Girls	1998	13	7.17
Angola	Girls	1999	13	7.26
Angola	Girls	2000	13	7.35
Angola	Girls	2001	13	7.45
Angola	Girls	2002	13	7.55
Angola	Girls	2003	13	7.66
Angola	Girls	2004	13	7.77
Angola	Girls	2005	13	7.89
Angola	Girls	2006	13	8.00
Angola	Girls	2007	13	8.13
Angola	Girls	2008	13	8.25
Angola	Girls	2009	13	8.38
Angola	Girls	2010	13	8.51
Angola	Girls	2011	13	8.64
Angola	Girls	2012	13	8.78
Angola	Girls	2013	13	8.92
Angola	Girls	2014	13	9.07
Angola	Girls	2015	13	9.21
Angola	Girls	2016	13	9.36
Angola	Girls	1975	14	7.32
Angola	Girls	1976	14	7.36
Angola	Girls	1977	14	7.40
Angola	Girls	1978	14	7.43
Angola	Girls	1979	14	7.47
Angola	Girls	1980	14	7.52
Angola	Girls	1981	14	7.56
Angola	Girls	1982	14	7.60
Angola	Girls	1983	14	7.65
Angola	Girls	1984	14	7.70
Angola	Girls	1985	14	7.75
Angola	Girls	1986	14	7.80
Angola	Girls	1987	14	7.86
Angola	Girls	1988	14	7.92
Angola	Girls	1989	14	7.98

Angola	Girls	1990	14	8.05
Angola	Girls	1991	14	8.12
Angola	Girls	1992	14	8.19
Angola	Girls	1993	14	8.26
Angola	Girls	1994	14	8.34
Angola	Girls	1995	14	8.42
Angola	Girls	1996	14	8.51
Angola	Girls	1997	14	8.60
Angola	Girls	1998	14	8.70
Angola	Girls	1999	14	8.80
Angola	Girls	2000	14	8.90
Angola	Girls	2001	14	9.01
Angola	Girls	2002	14	9.12
Angola	Girls	2003	14	9.24
Angola	Girls	2004	14	9.36
Angola	Girls	2005	14	9.49
Angola	Girls	2006	14	9.62
Angola	Girls	2007	14	9.75
Angola	Girls	2008	14	9.89
Angola	Girls	2009	14	10.03
Angola	Girls	2010	14	10.17
Angola	Girls	2011	14	10.32
Angola	Girls	2012	14	10.47
Angola	Girls	2013	14	10.63
Angola	Girls	2014	14	10.79
Angola	Girls	2015	14	10.95
Angola	Girls	2016	14	11.11
Angola	Girls	1975	15	8.64
Angola	Girls	1976	15	8.68
Angola	Girls	1977	15	8.72
Angola	Girls	1978	15	8.76
Angola	Girls	1979	15	8.81
Angola	Girls	1980	15	8.85
Angola	Girls	1981	15	8.90
Angola	Girls	1982	15	8.95
Angola	Girls	1983	15	9.00
Angola	Girls	1984	15	9.05
Angola	Girls	1985	15	9.11
Angola	Girls	1986	15	9.17
Angola	Girls	1987	15	9.23
Angola	Girls	1988	15	9.29
Angola	Girls	1989	15	9.36
Angola	Girls	1990	15	9.43
Angola	Girls	1991	15	9.50
Angola	Girls	1992	15	9.58
Angola	Girls	1993	15	9.66
Angola	Girls	1994	15	9.74
Angola	Girls	1995	15	9.83
Angola	Girls	1996	15	9.92
Angola	Girls	1997	15	10.02
Angola	Girls	1998	15	10.12
Angola	Girls	1999	15	10.23

Angola	Girls	2000	15	10.34
Angola	Girls	2001	15	10.46
Angola	Girls	2002	15	10.58
Angola	Girls	2003	15	10.70
Angola	Girls	2004	15	10.83
Angola	Girls	2005	15	10.96
Angola	Girls	2006	15	11.10
Angola	Girls	2007	15	11.24
Angola	Girls	2008	15	11.39
Angola	Girls	2009	15	11.53
Angola	Girls	2010	15	11.68
Angola	Girls	2011	15	11.84
Angola	Girls	2012	15	12.00
Angola	Girls	2013	15	12.16
Angola	Girls	2014	15	12.33
Angola	Girls	2015	15	12.50
Angola	Girls	2016	15	12.67
Angola	Girls	1975	16	9.72
Angola	Girls	1976	16	9.76
Angola	Girls	1977	16	9.81
Angola	Girls	1978	16	9.85
Angola	Girls	1979	16	9.90
Angola	Girls	1980	16	9.95
Angola	Girls	1981	16	10.00
Angola	Girls	1982	16	10.05
Angola	Girls	1983	16	10.10
Angola	Girls	1984	16	10.16
Angola	Girls	1985	16	10.22
Angola	Girls	1986	16	10.28
Angola	Girls	1987	16	10.34
Angola	Girls	1988	16	10.41
Angola	Girls	1989	16	10.48
Angola	Girls	1990	16	10.55
Angola	Girls	1991	16	10.63
Angola	Girls	1992	16	10.71
Angola	Girls	1993	16	10.79
Angola	Girls	1994	16	10.88
Angola	Girls	1995	16	10.97
Angola	Girls	1996	16	11.07
Angola	Girls	1997	16	11.17
Angola	Girls	1998	16	11.28
Angola	Girls	1999	16	11.39
Angola	Girls	2000	16	11.50
Angola	Girls	2001	16	11.62
Angola	Girls	2002	16	11.75
Angola	Girls	2003	16	11.87
Angola	Girls	2004	16	12.01
Angola	Girls	2005	16	12.15
Angola	Girls	2006	16	12.29
Angola	Girls	2007	16	12.43
Angola	Girls	2008	16	12.58
Angola	Girls	2009	16	12.74

Angola	Girls	2010	16	12.89
Angola	Girls	2011	16	13.05
Angola	Girls	2012	16	13.22
Angola	Girls	2013	16	13.39
Angola	Girls	2014	16	13.56
Angola	Girls	2015	16	13.74
Angola	Girls	2016	16	13.92
Angola	Girls	1975	17	10.48
Angola	Girls	1976	17	10.53
Angola	Girls	1977	17	10.57
Angola	Girls	1978	17	10.62
Angola	Girls	1979	17	10.67
Angola	Girls	1980	17	10.72
Angola	Girls	1981	17	10.77
Angola	Girls	1982	17	10.83
Angola	Girls	1983	17	10.88
Angola	Girls	1984	17	10.94
Angola	Girls	1985	17	11.00
Angola	Girls	1986	17	11.07
Angola	Girls	1987	17	11.13
Angola	Girls	1988	17	11.20
Angola	Girls	1989	17	11.28
Angola	Girls	1990	17	11.35
Angola	Girls	1991	17	11.43
Angola	Girls	1992	17	11.52
Angola	Girls	1993	17	11.60
Angola	Girls	1994	17	11.70
Angola	Girls	1995	17	11.79
Angola	Girls	1996	17	11.89
Angola	Girls	1997	17	12.00
Angola	Girls	1998	17	12.11
Angola	Girls	1999	17	12.22
Angola	Girls	2000	17	12.34
Angola	Girls	2001	17	12.47
Angola	Girls	2002	17	12.60
Angola	Girls	2003	17	12.73
Angola	Girls	2004	17	12.87
Angola	Girls	2005	17	13.02
Angola	Girls	2006	17	13.17
Angola	Girls	2007	17	13.32
Angola	Girls	2008	17	13.48
Angola	Girls	2009	17	13.64
Angola	Girls	2010	17	13.80
Angola	Girls	2011	17	13.97
Angola	Girls	2012	17	14.14
Angola	Girls	2013	17	14.32
Angola	Girls	2014	17	14.50
Angola	Girls	2015	17	14.68
Angola	Girls	2016	17	14.87
Angola	Girls	1975	18	10.96
Angola	Girls	1976	18	11.01
Angola	Girls	1977	18	11.06

Angola	Girls	1978	18	11.11
Angola	Girls	1979	18	11.17
Angola	Girls	1980	18	11.22
Angola	Girls	1981	18	11.28
Angola	Girls	1982	18	11.33
Angola	Girls	1983	18	11.39
Angola	Girls	1984	18	11.46
Angola	Girls	1985	18	11.52
Angola	Girls	1986	18	11.59
Angola	Girls	1987	18	11.66
Angola	Girls	1988	18	11.74
Angola	Girls	1989	18	11.82
Angola	Girls	1990	18	11.90
Angola	Girls	1991	18	11.98
Angola	Girls	1992	18	12.07
Angola	Girls	1993	18	12.17
Angola	Girls	1994	18	12.27
Angola	Girls	1995	18	12.37
Angola	Girls	1996	18	12.48
Angola	Girls	1997	18	12.59
Angola	Girls	1998	18	12.71
Angola	Girls	1999	18	12.84
Angola	Girls	2000	18	12.97
Angola	Girls	2001	18	13.10
Angola	Girls	2002	18	13.24
Angola	Girls	2003	18	13.39
Angola	Girls	2004	18	13.54
Angola	Girls	2005	18	13.70
Angola	Girls	2006	18	13.86
Angola	Girls	2007	18	14.02
Angola	Girls	2008	18	14.19
Angola	Girls	2009	18	14.37
Angola	Girls	2010	18	14.54
Angola	Girls	2011	18	14.73
Angola	Girls	2012	18	14.91
Angola	Girls	2013	18	15.10
Angola	Girls	2014	18	15.30
Angola	Girls	2015	18	15.50
Angola	Girls	2016	18	15.71
Angola	Girls	1975	Average	8.26
Angola	Girls	1976	Average	8.30
Angola	Girls	1977	Average	8.34
Angola	Girls	1978	Average	8.38
Angola	Girls	1979	Average	8.42
Angola	Girls	1980	Average	8.46
Angola	Girls	1981	Average	8.51
Angola	Girls	1982	Average	8.55
Angola	Girls	1983	Average	8.60
Angola	Girls	1984	Average	8.65
Angola	Girls	1985	Average	8.71
Angola	Girls	1986	Average	8.76
Angola	Girls	1987	Average	8.82

Angola	Girls	1988	Average	8.88
Angola	Girls	1989	Average	8.95
Angola	Girls	1990	Average	9.02
Angola	Girls	1991	Average	9.09
Angola	Girls	1992	Average	9.16
Angola	Girls	1993	Average	9.24
Angola	Girls	1994	Average	9.32
Angola	Girls	1995	Average	9.41
Angola	Girls	1996	Average	9.50
Angola	Girls	1997	Average	9.59
Angola	Girls	1998	Average	9.69
Angola	Girls	1999	Average	9.79
Angola	Girls	2000	Average	9.90
Angola	Girls	2001	Average	10.01
Angola	Girls	2002	Average	10.13
Angola	Girls	2003	Average	10.25
Angola	Girls	2004	Average	10.38
Angola	Girls	2005	Average	10.51
Angola	Girls	2006	Average	10.64
Angola	Girls	2007	Average	10.78
Angola	Girls	2008	Average	10.92
Angola	Girls	2009	Average	11.07
Angola	Girls	2010	Average	11.21
Angola	Girls	2011	Average	11.37
Angola	Girls	2012	Average	11.52
Angola	Girls	2013	Average	11.68
Angola	Girls	2014	Average	11.85
Angola	Girls	2015	Average	12.01
Angola	Girls	2016	Average	12.18
Antigua and Barbuda	Boys	1975	12	5.61
Antigua and Barbuda	Boys	1976	12	5.66
Antigua and Barbuda	Boys	1977	12	5.70
Antigua and Barbuda	Boys	1978	12	5.76
Antigua and Barbuda	Boys	1979	12	5.82
Antigua and Barbuda	Boys	1980	12	5.88
Antigua and Barbuda	Boys	1981	12	5.95
Antigua and Barbuda	Boys	1982	12	6.02
Antigua and Barbuda	Boys	1983	12	6.10
Antigua and Barbuda	Boys	1984	12	6.18
Antigua and Barbuda	Boys	1985	12	6.27
Antigua and Barbuda	Boys	1986	12	6.36
Antigua and Barbuda	Boys	1987	12	6.46
Antigua and Barbuda	Boys	1988	12	6.56
Antigua and Barbuda	Boys	1989	12	6.67
Antigua and Barbuda	Boys	1990	12	6.79
Antigua and Barbuda	Boys	1991	12	6.91
Antigua and Barbuda	Boys	1992	12	7.03
Antigua and Barbuda	Boys	1993	12	7.16
Antigua and Barbuda	Boys	1994	12	7.28
Antigua and Barbuda	Boys	1995	12	7.41
Antigua and Barbuda	Boys	1996	12	7.55
Antigua and Barbuda	Boys	1997	12	7.69

Antigua and Barbuda	Boys	1998	12	7.83
Antigua and Barbuda	Boys	1999	12	7.98
Antigua and Barbuda	Boys	2000	12	8.13
Antigua and Barbuda	Boys	2001	12	8.29
Antigua and Barbuda	Boys	2002	12	8.45
Antigua and Barbuda	Boys	2003	12	8.62
Antigua and Barbuda	Boys	2004	12	8.79
Antigua and Barbuda	Boys	2005	12	8.97
Antigua and Barbuda	Boys	2006	12	9.16
Antigua and Barbuda	Boys	2007	12	9.35
Antigua and Barbuda	Boys	2008	12	9.55
Antigua and Barbuda	Boys	2009	12	9.76
Antigua and Barbuda	Boys	2010	12	9.98
Antigua and Barbuda	Boys	2011	12	10.20
Antigua and Barbuda	Boys	2012	12	10.43
Antigua and Barbuda	Boys	2013	12	10.68
Antigua and Barbuda	Boys	2014	12	10.93
Antigua and Barbuda	Boys	2015	12	11.19
Antigua and Barbuda	Boys	2016	12	11.46
Antigua and Barbuda	Boys	1975	13	6.95
Antigua and Barbuda	Boys	1976	13	7.00
Antigua and Barbuda	Boys	1977	13	7.06
Antigua and Barbuda	Boys	1978	13	7.12
Antigua and Barbuda	Boys	1979	13	7.19
Antigua and Barbuda	Boys	1980	13	7.26
Antigua and Barbuda	Boys	1981	13	7.34
Antigua and Barbuda	Boys	1982	13	7.42
Antigua and Barbuda	Boys	1983	13	7.51
Antigua and Barbuda	Boys	1984	13	7.60
Antigua and Barbuda	Boys	1985	13	7.70
Antigua and Barbuda	Boys	1986	13	7.80
Antigua and Barbuda	Boys	1987	13	7.92
Antigua and Barbuda	Boys	1988	13	8.04
Antigua and Barbuda	Boys	1989	13	8.16
Antigua and Barbuda	Boys	1990	13	8.30
Antigua and Barbuda	Boys	1991	13	8.44
Antigua and Barbuda	Boys	1992	13	8.58
Antigua and Barbuda	Boys	1993	13	8.72
Antigua and Barbuda	Boys	1994	13	8.87
Antigua and Barbuda	Boys	1995	13	9.02
Antigua and Barbuda	Boys	1996	13	9.17
Antigua and Barbuda	Boys	1997	13	9.33
Antigua and Barbuda	Boys	1998	13	9.50
Antigua and Barbuda	Boys	1999	13	9.67
Antigua and Barbuda	Boys	2000	13	9.85
Antigua and Barbuda	Boys	2001	13	10.03
Antigua and Barbuda	Boys	2002	13	10.22
Antigua and Barbuda	Boys	2003	13	10.42
Antigua and Barbuda	Boys	2004	13	10.62
Antigua and Barbuda	Boys	2005	13	10.84
Antigua and Barbuda	Boys	2006	13	11.06
Antigua and Barbuda	Boys	2007	13	11.29

Antigua and Barbuda	Boys	2008	13	11.52
Antigua and Barbuda	Boys	2009	13	11.77
Antigua and Barbuda	Boys	2010	13	12.03
Antigua and Barbuda	Boys	2011	13	12.30
Antigua and Barbuda	Boys	2012	13	12.58
Antigua and Barbuda	Boys	2013	13	12.88
Antigua and Barbuda	Boys	2014	13	13.19
Antigua and Barbuda	Boys	2015	13	13.51
Antigua and Barbuda	Boys	2016	13	13.84
Antigua and Barbuda	Boys	1975	14	8.62
Antigua and Barbuda	Boys	1976	14	8.68
Antigua and Barbuda	Boys	1977	14	8.74
Antigua and Barbuda	Boys	1978	14	8.81
Antigua and Barbuda	Boys	1979	14	8.88
Antigua and Barbuda	Boys	1980	14	8.96
Antigua and Barbuda	Boys	1981	14	9.04
Antigua and Barbuda	Boys	1982	14	9.13
Antigua and Barbuda	Boys	1983	14	9.23
Antigua and Barbuda	Boys	1984	14	9.33
Antigua and Barbuda	Boys	1985	14	9.44
Antigua and Barbuda	Boys	1986	14	9.55
Antigua and Barbuda	Boys	1987	14	9.67
Antigua and Barbuda	Boys	1988	14	9.80
Antigua and Barbuda	Boys	1989	14	9.94
Antigua and Barbuda	Boys	1990	14	10.09
Antigua and Barbuda	Boys	1991	14	10.24
Antigua and Barbuda	Boys	1992	14	10.39
Antigua and Barbuda	Boys	1993	14	10.54
Antigua and Barbuda	Boys	1994	14	10.70
Antigua and Barbuda	Boys	1995	14	10.87
Antigua and Barbuda	Boys	1996	14	11.04
Antigua and Barbuda	Boys	1997	14	11.21
Antigua and Barbuda	Boys	1998	14	11.39
Antigua and Barbuda	Boys	1999	14	11.58
Antigua and Barbuda	Boys	2000	14	11.77
Antigua and Barbuda	Boys	2001	14	11.98
Antigua and Barbuda	Boys	2002	14	12.19
Antigua and Barbuda	Boys	2003	14	12.40
Antigua and Barbuda	Boys	2004	14	12.63
Antigua and Barbuda	Boys	2005	14	12.86
Antigua and Barbuda	Boys	2006	14	13.11
Antigua and Barbuda	Boys	2007	14	13.36
Antigua and Barbuda	Boys	2008	14	13.63
Antigua and Barbuda	Boys	2009	14	13.91
Antigua and Barbuda	Boys	2010	14	14.20
Antigua and Barbuda	Boys	2011	14	14.51
Antigua and Barbuda	Boys	2012	14	14.83
Antigua and Barbuda	Boys	2013	14	15.17
Antigua and Barbuda	Boys	2014	14	15.52
Antigua and Barbuda	Boys	2015	14	15.89
Antigua and Barbuda	Boys	2016	14	16.28
Antigua and Barbuda	Boys	1975	15	10.56

Antigua and Barbuda	Boys	1976	15	10.62
Antigua and Barbuda	Boys	1977	15	10.68
Antigua and Barbuda	Boys	1978	15	10.75
Antigua and Barbuda	Boys	1979	15	10.83
Antigua and Barbuda	Boys	1980	15	10.92
Antigua and Barbuda	Boys	1981	15	11.00
Antigua and Barbuda	Boys	1982	15	11.10
Antigua and Barbuda	Boys	1983	15	11.20
Antigua and Barbuda	Boys	1984	15	11.30
Antigua and Barbuda	Boys	1985	15	11.41
Antigua and Barbuda	Boys	1986	15	11.53
Antigua and Barbuda	Boys	1987	15	11.66
Antigua and Barbuda	Boys	1988	15	11.79
Antigua and Barbuda	Boys	1989	15	11.93
Antigua and Barbuda	Boys	1990	15	12.08
Antigua and Barbuda	Boys	1991	15	12.24
Antigua and Barbuda	Boys	1992	15	12.39
Antigua and Barbuda	Boys	1993	15	12.55
Antigua and Barbuda	Boys	1994	15	12.72
Antigua and Barbuda	Boys	1995	15	12.89
Antigua and Barbuda	Boys	1996	15	13.06
Antigua and Barbuda	Boys	1997	15	13.24
Antigua and Barbuda	Boys	1998	15	13.43
Antigua and Barbuda	Boys	1999	15	13.63
Antigua and Barbuda	Boys	2000	15	13.83
Antigua and Barbuda	Boys	2001	15	14.04
Antigua and Barbuda	Boys	2002	15	14.26
Antigua and Barbuda	Boys	2003	15	14.48
Antigua and Barbuda	Boys	2004	15	14.72
Antigua and Barbuda	Boys	2005	15	14.97
Antigua and Barbuda	Boys	2006	15	15.23
Antigua and Barbuda	Boys	2007	15	15.49
Antigua and Barbuda	Boys	2008	15	15.78
Antigua and Barbuda	Boys	2009	15	16.07
Antigua and Barbuda	Boys	2010	15	16.38
Antigua and Barbuda	Boys	2011	15	16.71
Antigua and Barbuda	Boys	2012	15	17.06
Antigua and Barbuda	Boys	2013	15	17.42
Antigua and Barbuda	Boys	2014	15	17.80
Antigua and Barbuda	Boys	2015	15	18.20
Antigua and Barbuda	Boys	2016	15	18.63
Antigua and Barbuda	Boys	1975	16	12.68
Antigua and Barbuda	Boys	1976	16	12.74
Antigua and Barbuda	Boys	1977	16	12.81
Antigua and Barbuda	Boys	1978	16	12.88
Antigua and Barbuda	Boys	1979	16	12.96
Antigua and Barbuda	Boys	1980	16	13.05
Antigua and Barbuda	Boys	1981	16	13.14
Antigua and Barbuda	Boys	1982	16	13.23
Antigua and Barbuda	Boys	1983	16	13.33
Antigua and Barbuda	Boys	1984	16	13.44
Antigua and Barbuda	Boys	1985	16	13.55

Antigua and Barbuda	Boys	1986	16	13.67
Antigua and Barbuda	Boys	1987	16	13.80
Antigua and Barbuda	Boys	1988	16	13.94
Antigua and Barbuda	Boys	1989	16	14.08
Antigua and Barbuda	Boys	1990	16	14.23
Antigua and Barbuda	Boys	1991	16	14.39
Antigua and Barbuda	Boys	1992	16	14.54
Antigua and Barbuda	Boys	1993	16	14.70
Antigua and Barbuda	Boys	1994	16	14.87
Antigua and Barbuda	Boys	1995	16	15.04
Antigua and Barbuda	Boys	1996	16	15.22
Antigua and Barbuda	Boys	1997	16	15.40
Antigua and Barbuda	Boys	1998	16	15.59
Antigua and Barbuda	Boys	1999	16	15.79
Antigua and Barbuda	Boys	2000	16	15.99
Antigua and Barbuda	Boys	2001	16	16.20
Antigua and Barbuda	Boys	2002	16	16.43
Antigua and Barbuda	Boys	2003	16	16.65
Antigua and Barbuda	Boys	2004	16	16.89
Antigua and Barbuda	Boys	2005	16	17.15
Antigua and Barbuda	Boys	2006	16	17.41
Antigua and Barbuda	Boys	2007	16	17.68
Antigua and Barbuda	Boys	2008	16	17.97
Antigua and Barbuda	Boys	2009	16	18.27
Antigua and Barbuda	Boys	2010	16	18.59
Antigua and Barbuda	Boys	2011	16	18.93
Antigua and Barbuda	Boys	2012	16	19.29
Antigua and Barbuda	Boys	2013	16	19.66
Antigua and Barbuda	Boys	2014	16	20.06
Antigua and Barbuda	Boys	2015	16	20.48
Antigua and Barbuda	Boys	2016	16	20.92
Antigua and Barbuda	Boys	1975	17	14.88
Antigua and Barbuda	Boys	1976	17	14.95
Antigua and Barbuda	Boys	1977	17	15.02
Antigua and Barbuda	Boys	1978	17	15.10
Antigua and Barbuda	Boys	1979	17	15.18
Antigua and Barbuda	Boys	1980	17	15.27
Antigua and Barbuda	Boys	1981	17	15.36
Antigua and Barbuda	Boys	1982	17	15.46
Antigua and Barbuda	Boys	1983	17	15.56
Antigua and Barbuda	Boys	1984	17	15.67
Antigua and Barbuda	Boys	1985	17	15.78
Antigua and Barbuda	Boys	1986	17	15.91
Antigua and Barbuda	Boys	1987	17	16.03
Antigua and Barbuda	Boys	1988	17	16.17
Antigua and Barbuda	Boys	1989	17	16.31
Antigua and Barbuda	Boys	1990	17	16.47
Antigua and Barbuda	Boys	1991	17	16.62
Antigua and Barbuda	Boys	1992	17	16.78
Antigua and Barbuda	Boys	1993	17	16.94
Antigua and Barbuda	Boys	1994	17	17.11
Antigua and Barbuda	Boys	1995	17	17.28

Antigua and Barbuda	Boys	1996	17	17.46
Antigua and Barbuda	Boys	1997	17	17.64
Antigua and Barbuda	Boys	1998	17	17.84
Antigua and Barbuda	Boys	1999	17	18.03
Antigua and Barbuda	Boys	2000	17	18.24
Antigua and Barbuda	Boys	2001	17	18.46
Antigua and Barbuda	Boys	2002	17	18.68
Antigua and Barbuda	Boys	2003	17	18.91
Antigua and Barbuda	Boys	2004	17	19.15
Antigua and Barbuda	Boys	2005	17	19.41
Antigua and Barbuda	Boys	2006	17	19.67
Antigua and Barbuda	Boys	2007	17	19.95
Antigua and Barbuda	Boys	2008	17	20.24
Antigua and Barbuda	Boys	2009	17	20.55
Antigua and Barbuda	Boys	2010	17	20.87
Antigua and Barbuda	Boys	2011	17	21.21
Antigua and Barbuda	Boys	2012	17	21.58
Antigua and Barbuda	Boys	2013	17	21.96
Antigua and Barbuda	Boys	2014	17	22.37
Antigua and Barbuda	Boys	2015	17	22.80
Antigua and Barbuda	Boys	2016	17	23.25
Antigua and Barbuda	Boys	1975	18	17.09
Antigua and Barbuda	Boys	1976	18	17.16
Antigua and Barbuda	Boys	1977	18	17.24
Antigua and Barbuda	Boys	1978	18	17.32
Antigua and Barbuda	Boys	1979	18	17.41
Antigua and Barbuda	Boys	1980	18	17.50
Antigua and Barbuda	Boys	1981	18	17.60
Antigua and Barbuda	Boys	1982	18	17.70
Antigua and Barbuda	Boys	1983	18	17.81
Antigua and Barbuda	Boys	1984	18	17.92
Antigua and Barbuda	Boys	1985	18	18.04
Antigua and Barbuda	Boys	1986	18	18.17
Antigua and Barbuda	Boys	1987	18	18.30
Antigua and Barbuda	Boys	1988	18	18.44
Antigua and Barbuda	Boys	1989	18	18.59
Antigua and Barbuda	Boys	1990	18	18.75
Antigua and Barbuda	Boys	1991	18	18.91
Antigua and Barbuda	Boys	1992	18	19.08
Antigua and Barbuda	Boys	1993	18	19.25
Antigua and Barbuda	Boys	1994	18	19.42
Antigua and Barbuda	Boys	1995	18	19.60
Antigua and Barbuda	Boys	1996	18	19.78
Antigua and Barbuda	Boys	1997	18	19.97
Antigua and Barbuda	Boys	1998	18	20.17
Antigua and Barbuda	Boys	1999	18	20.38
Antigua and Barbuda	Boys	2000	18	20.59
Antigua and Barbuda	Boys	2001	18	20.81
Antigua and Barbuda	Boys	2002	18	21.04
Antigua and Barbuda	Boys	2003	18	21.28
Antigua and Barbuda	Boys	2004	18	21.53
Antigua and Barbuda	Boys	2005	18	21.79

Antigua and Barbuda	Boys	2006	18	22.07
Antigua and Barbuda	Boys	2007	18	22.35
Antigua and Barbuda	Boys	2008	18	22.65
Antigua and Barbuda	Boys	2009	18	22.97
Antigua and Barbuda	Boys	2010	18	23.30
Antigua and Barbuda	Boys	2011	18	23.66
Antigua and Barbuda	Boys	2012	18	24.03
Antigua and Barbuda	Boys	2013	18	24.43
Antigua and Barbuda	Boys	2014	18	24.85
Antigua and Barbuda	Boys	2015	18	25.29
Antigua and Barbuda	Boys	2016	18	25.75
Antigua and Barbuda	Boys	1975	Average	10.91
Antigua and Barbuda	Boys	1976	Average	10.97
Antigua and Barbuda	Boys	1977	Average	11.04
Antigua and Barbuda	Boys	1978	Average	11.11
Antigua and Barbuda	Boys	1979	Average	11.18
Antigua and Barbuda	Boys	1980	Average	11.26
Antigua and Barbuda	Boys	1981	Average	11.35
Antigua and Barbuda	Boys	1982	Average	11.44
Antigua and Barbuda	Boys	1983	Average	11.53
Antigua and Barbuda	Boys	1984	Average	11.63
Antigua and Barbuda	Boys	1985	Average	11.74
Antigua and Barbuda	Boys	1986	Average	11.86
Antigua and Barbuda	Boys	1987	Average	11.98
Antigua and Barbuda	Boys	1988	Average	12.11
Antigua and Barbuda	Boys	1989	Average	12.24
Antigua and Barbuda	Boys	1990	Average	12.39
Antigua and Barbuda	Boys	1991	Average	12.53
Antigua and Barbuda	Boys	1992	Average	12.68
Antigua and Barbuda	Boys	1993	Average	12.84
Antigua and Barbuda	Boys	1994	Average	13.00
Antigua and Barbuda	Boys	1995	Average	13.16
Antigua and Barbuda	Boys	1996	Average	13.33
Antigua and Barbuda	Boys	1997	Average	13.50
Antigua and Barbuda	Boys	1998	Average	13.68
Antigua and Barbuda	Boys	1999	Average	13.86
Antigua and Barbuda	Boys	2000	Average	14.06
Antigua and Barbuda	Boys	2001	Average	14.26
Antigua and Barbuda	Boys	2002	Average	14.47
Antigua and Barbuda	Boys	2003	Average	14.68
Antigua and Barbuda	Boys	2004	Average	14.91
Antigua and Barbuda	Boys	2005	Average	15.14
Antigua and Barbuda	Boys	2006	Average	15.38
Antigua and Barbuda	Boys	2007	Average	15.64
Antigua and Barbuda	Boys	2008	Average	15.91
Antigua and Barbuda	Boys	2009	Average	16.19
Antigua and Barbuda	Boys	2010	Average	16.48
Antigua and Barbuda	Boys	2011	Average	16.79
Antigua and Barbuda	Boys	2012	Average	17.11
Antigua and Barbuda	Boys	2013	Average	17.46
Antigua and Barbuda	Boys	2014	Average	17.82
Antigua and Barbuda	Boys	2015	Average	18.19

Antigua and Barbuda	Boys	2016	Average	18.59
Antigua and Barbuda	Girls	1975	12	5.94
Antigua and Barbuda	Girls	1976	12	5.99
Antigua and Barbuda	Girls	1977	12	6.04
Antigua and Barbuda	Girls	1978	12	6.10
Antigua and Barbuda	Girls	1979	12	6.16
Antigua and Barbuda	Girls	1980	12	6.22
Antigua and Barbuda	Girls	1981	12	6.29
Antigua and Barbuda	Girls	1982	12	6.36
Antigua and Barbuda	Girls	1983	12	6.44
Antigua and Barbuda	Girls	1984	12	6.52
Antigua and Barbuda	Girls	1985	12	6.60
Antigua and Barbuda	Girls	1986	12	6.69
Antigua and Barbuda	Girls	1987	12	6.79
Antigua and Barbuda	Girls	1988	12	6.88
Antigua and Barbuda	Girls	1989	12	6.98
Antigua and Barbuda	Girls	1990	12	7.09
Antigua and Barbuda	Girls	1991	12	7.19
Antigua and Barbuda	Girls	1992	12	7.30
Antigua and Barbuda	Girls	1993	12	7.41
Antigua and Barbuda	Girls	1994	12	7.52
Antigua and Barbuda	Girls	1995	12	7.63
Antigua and Barbuda	Girls	1996	12	7.75
Antigua and Barbuda	Girls	1997	12	7.87
Antigua and Barbuda	Girls	1998	12	7.99
Antigua and Barbuda	Girls	1999	12	8.11
Antigua and Barbuda	Girls	2000	12	8.23
Antigua and Barbuda	Girls	2001	12	8.36
Antigua and Barbuda	Girls	2002	12	8.50
Antigua and Barbuda	Girls	2003	12	8.63
Antigua and Barbuda	Girls	2004	12	8.77
Antigua and Barbuda	Girls	2005	12	8.91
Antigua and Barbuda	Girls	2006	12	9.05
Antigua and Barbuda	Girls	2007	12	9.20
Antigua and Barbuda	Girls	2008	12	9.36
Antigua and Barbuda	Girls	2009	12	9.52
Antigua and Barbuda	Girls	2010	12	9.68
Antigua and Barbuda	Girls	2011	12	9.86
Antigua and Barbuda	Girls	2012	12	10.04
Antigua and Barbuda	Girls	2013	12	10.23
Antigua and Barbuda	Girls	2014	12	10.43
Antigua and Barbuda	Girls	2015	12	10.63
Antigua and Barbuda	Girls	2016	12	10.84
Antigua and Barbuda	Girls	1975	13	7.36
Antigua and Barbuda	Girls	1976	13	7.42
Antigua and Barbuda	Girls	1977	13	7.48
Antigua and Barbuda	Girls	1978	13	7.54
Antigua and Barbuda	Girls	1979	13	7.61
Antigua and Barbuda	Girls	1980	13	7.69
Antigua and Barbuda	Girls	1981	13	7.76
Antigua and Barbuda	Girls	1982	13	7.85
Antigua and Barbuda	Girls	1983	13	7.93

Antigua and Barbuda	Girls	1984	13	8.02
Antigua and Barbuda	Girls	1985	13	8.12
Antigua and Barbuda	Girls	1986	13	8.22
Antigua and Barbuda	Girls	1987	13	8.32
Antigua and Barbuda	Girls	1988	13	8.43
Antigua and Barbuda	Girls	1989	13	8.55
Antigua and Barbuda	Girls	1990	13	8.67
Antigua and Barbuda	Girls	1991	13	8.79
Antigua and Barbuda	Girls	1992	13	8.91
Antigua and Barbuda	Girls	1993	13	9.03
Antigua and Barbuda	Girls	1994	13	9.15
Antigua and Barbuda	Girls	1995	13	9.28
Antigua and Barbuda	Girls	1996	13	9.41
Antigua and Barbuda	Girls	1997	13	9.54
Antigua and Barbuda	Girls	1998	13	9.68
Antigua and Barbuda	Girls	1999	13	9.82
Antigua and Barbuda	Girls	2000	13	9.96
Antigua and Barbuda	Girls	2001	13	10.11
Antigua and Barbuda	Girls	2002	13	10.25
Antigua and Barbuda	Girls	2003	13	10.41
Antigua and Barbuda	Girls	2004	13	10.56
Antigua and Barbuda	Girls	2005	13	10.72
Antigua and Barbuda	Girls	2006	13	10.89
Antigua and Barbuda	Girls	2007	13	11.06
Antigua and Barbuda	Girls	2008	13	11.24
Antigua and Barbuda	Girls	2009	13	11.42
Antigua and Barbuda	Girls	2010	13	11.61
Antigua and Barbuda	Girls	2011	13	11.81
Antigua and Barbuda	Girls	2012	13	12.03
Antigua and Barbuda	Girls	2013	13	12.25
Antigua and Barbuda	Girls	2014	13	12.48
Antigua and Barbuda	Girls	2015	13	12.71
Antigua and Barbuda	Girls	2016	13	12.96
Antigua and Barbuda	Girls	1975	14	8.88
Antigua and Barbuda	Girls	1976	14	8.94
Antigua and Barbuda	Girls	1977	14	9.01
Antigua and Barbuda	Girls	1978	14	9.08
Antigua and Barbuda	Girls	1979	14	9.15
Antigua and Barbuda	Girls	1980	14	9.23
Antigua and Barbuda	Girls	1981	14	9.32
Antigua and Barbuda	Girls	1982	14	9.41
Antigua and Barbuda	Girls	1983	14	9.50
Antigua and Barbuda	Girls	1984	14	9.60
Antigua and Barbuda	Girls	1985	14	9.70
Antigua and Barbuda	Girls	1986	14	9.81
Antigua and Barbuda	Girls	1987	14	9.92
Antigua and Barbuda	Girls	1988	14	10.04
Antigua and Barbuda	Girls	1989	14	10.16
Antigua and Barbuda	Girls	1990	14	10.29
Antigua and Barbuda	Girls	1991	14	10.42
Antigua and Barbuda	Girls	1992	14	10.55
Antigua and Barbuda	Girls	1993	14	10.68

Antigua and Barbuda	Girls	1994	14	10.81
Antigua and Barbuda	Girls	1995	14	10.95
Antigua and Barbuda	Girls	1996	14	11.09
Antigua and Barbuda	Girls	1997	14	11.23
Antigua and Barbuda	Girls	1998	14	11.38
Antigua and Barbuda	Girls	1999	14	11.52
Antigua and Barbuda	Girls	2000	14	11.68
Antigua and Barbuda	Girls	2001	14	11.83
Antigua and Barbuda	Girls	2002	14	11.99
Antigua and Barbuda	Girls	2003	14	12.16
Antigua and Barbuda	Girls	2004	14	12.33
Antigua and Barbuda	Girls	2005	14	12.50
Antigua and Barbuda	Girls	2006	14	12.68
Antigua and Barbuda	Girls	2007	14	12.86
Antigua and Barbuda	Girls	2008	14	13.05
Antigua and Barbuda	Girls	2009	14	13.26
Antigua and Barbuda	Girls	2010	14	13.47
Antigua and Barbuda	Girls	2011	14	13.69
Antigua and Barbuda	Girls	2012	14	13.92
Antigua and Barbuda	Girls	2013	14	14.16
Antigua and Barbuda	Girls	2014	14	14.41
Antigua and Barbuda	Girls	2015	14	14.67
Antigua and Barbuda	Girls	2016	14	14.95
Antigua and Barbuda	Girls	1975	15	10.29
Antigua and Barbuda	Girls	1976	15	10.35
Antigua and Barbuda	Girls	1977	15	10.42
Antigua and Barbuda	Girls	1978	15	10.50
Antigua and Barbuda	Girls	1979	15	10.57
Antigua and Barbuda	Girls	1980	15	10.66
Antigua and Barbuda	Girls	1981	15	10.75
Antigua and Barbuda	Girls	1982	15	10.84
Antigua and Barbuda	Girls	1983	15	10.94
Antigua and Barbuda	Girls	1984	15	11.04
Antigua and Barbuda	Girls	1985	15	11.15
Antigua and Barbuda	Girls	1986	15	11.26
Antigua and Barbuda	Girls	1987	15	11.38
Antigua and Barbuda	Girls	1988	15	11.50
Antigua and Barbuda	Girls	1989	15	11.63
Antigua and Barbuda	Girls	1990	15	11.76
Antigua and Barbuda	Girls	1991	15	11.89
Antigua and Barbuda	Girls	1992	15	12.03
Antigua and Barbuda	Girls	1993	15	12.16
Antigua and Barbuda	Girls	1994	15	12.30
Antigua and Barbuda	Girls	1995	15	12.44
Antigua and Barbuda	Girls	1996	15	12.59
Antigua and Barbuda	Girls	1997	15	12.73
Antigua and Barbuda	Girls	1998	15	12.88
Antigua and Barbuda	Girls	1999	15	13.04
Antigua and Barbuda	Girls	2000	15	13.20
Antigua and Barbuda	Girls	2001	15	13.36
Antigua and Barbuda	Girls	2002	15	13.52
Antigua and Barbuda	Girls	2003	15	13.70

Antigua and Barbuda	Girls	2004	15	13.87
Antigua and Barbuda	Girls	2005	15	14.05
Antigua and Barbuda	Girls	2006	15	14.24
Antigua and Barbuda	Girls	2007	15	14.43
Antigua and Barbuda	Girls	2008	15	14.63
Antigua and Barbuda	Girls	2009	15	14.84
Antigua and Barbuda	Girls	2010	15	15.06
Antigua and Barbuda	Girls	2011	15	15.29
Antigua and Barbuda	Girls	2012	15	15.53
Antigua and Barbuda	Girls	2013	15	15.79
Antigua and Barbuda	Girls	2014	15	16.06
Antigua and Barbuda	Girls	2015	15	16.34
Antigua and Barbuda	Girls	2016	15	16.63
Antigua and Barbuda	Girls	1975	16	11.42
Antigua and Barbuda	Girls	1976	16	11.49
Antigua and Barbuda	Girls	1977	16	11.56
Antigua and Barbuda	Girls	1978	16	11.64
Antigua and Barbuda	Girls	1979	16	11.72
Antigua and Barbuda	Girls	1980	16	11.81
Antigua and Barbuda	Girls	1981	16	11.90
Antigua and Barbuda	Girls	1982	16	11.99
Antigua and Barbuda	Girls	1983	16	12.09
Antigua and Barbuda	Girls	1984	16	12.20
Antigua and Barbuda	Girls	1985	16	12.31
Antigua and Barbuda	Girls	1986	16	12.43
Antigua and Barbuda	Girls	1987	16	12.55
Antigua and Barbuda	Girls	1988	16	12.67
Antigua and Barbuda	Girls	1989	16	12.80
Antigua and Barbuda	Girls	1990	16	12.94
Antigua and Barbuda	Girls	1991	16	13.08
Antigua and Barbuda	Girls	1992	16	13.21
Antigua and Barbuda	Girls	1993	16	13.36
Antigua and Barbuda	Girls	1994	16	13.50
Antigua and Barbuda	Girls	1995	16	13.64
Antigua and Barbuda	Girls	1996	16	13.79
Antigua and Barbuda	Girls	1997	16	13.94
Antigua and Barbuda	Girls	1998	16	14.10
Antigua and Barbuda	Girls	1999	16	14.25
Antigua and Barbuda	Girls	2000	16	14.42
Antigua and Barbuda	Girls	2001	16	14.59
Antigua and Barbuda	Girls	2002	16	14.76
Antigua and Barbuda	Girls	2003	16	14.93
Antigua and Barbuda	Girls	2004	16	15.11
Antigua and Barbuda	Girls	2005	16	15.30
Antigua and Barbuda	Girls	2006	16	15.49
Antigua and Barbuda	Girls	2007	16	15.69
Antigua and Barbuda	Girls	2008	16	15.90
Antigua and Barbuda	Girls	2009	16	16.11
Antigua and Barbuda	Girls	2010	16	16.34
Antigua and Barbuda	Girls	2011	16	16.58
Antigua and Barbuda	Girls	2012	16	16.83
Antigua and Barbuda	Girls	2013	16	17.10

Antigua and Barbuda	Girls	2014	16	17.38
Antigua and Barbuda	Girls	2015	16	17.67
Antigua and Barbuda	Girls	2016	16	17.97
Antigua and Barbuda	Girls	1975	17	12.25
Antigua and Barbuda	Girls	1976	17	12.32
Antigua and Barbuda	Girls	1977	17	12.39
Antigua and Barbuda	Girls	1978	17	12.47
Antigua and Barbuda	Girls	1979	17	12.56
Antigua and Barbuda	Girls	1980	17	12.65
Antigua and Barbuda	Girls	1981	17	12.75
Antigua and Barbuda	Girls	1982	17	12.85
Antigua and Barbuda	Girls	1983	17	12.95
Antigua and Barbuda	Girls	1984	17	13.06
Antigua and Barbuda	Girls	1985	17	13.18
Antigua and Barbuda	Girls	1986	17	13.30
Antigua and Barbuda	Girls	1987	17	13.43
Antigua and Barbuda	Girls	1988	17	13.56
Antigua and Barbuda	Girls	1989	17	13.70
Antigua and Barbuda	Girls	1990	17	13.84
Antigua and Barbuda	Girls	1991	17	13.99
Antigua and Barbuda	Girls	1992	17	14.13
Antigua and Barbuda	Girls	1993	17	14.28
Antigua and Barbuda	Girls	1994	17	14.43
Antigua and Barbuda	Girls	1995	17	14.58
Antigua and Barbuda	Girls	1996	17	14.74
Antigua and Barbuda	Girls	1997	17	14.90
Antigua and Barbuda	Girls	1998	17	15.06
Antigua and Barbuda	Girls	1999	17	15.23
Antigua and Barbuda	Girls	2000	17	15.40
Antigua and Barbuda	Girls	2001	17	15.57
Antigua and Barbuda	Girls	2002	17	15.75
Antigua and Barbuda	Girls	2003	17	15.94
Antigua and Barbuda	Girls	2004	17	16.13
Antigua and Barbuda	Girls	2005	17	16.32
Antigua and Barbuda	Girls	2006	17	16.52
Antigua and Barbuda	Girls	2007	17	16.73
Antigua and Barbuda	Girls	2008	17	16.95
Antigua and Barbuda	Girls	2009	17	17.18
Antigua and Barbuda	Girls	2010	17	17.42
Antigua and Barbuda	Girls	2011	17	17.67
Antigua and Barbuda	Girls	2012	17	17.93
Antigua and Barbuda	Girls	2013	17	18.21
Antigua and Barbuda	Girls	2014	17	18.50
Antigua and Barbuda	Girls	2015	17	18.80
Antigua and Barbuda	Girls	2016	17	19.12
Antigua and Barbuda	Girls	1975	18	12.87
Antigua and Barbuda	Girls	1976	18	12.95
Antigua and Barbuda	Girls	1977	18	13.03
Antigua and Barbuda	Girls	1978	18	13.12
Antigua and Barbuda	Girls	1979	18	13.21
Antigua and Barbuda	Girls	1980	18	13.31
Antigua and Barbuda	Girls	1981	18	13.41

Antigua and Barbuda	Girls	1982	18	13.53
Antigua and Barbuda	Girls	1983	18	13.64
Antigua and Barbuda	Girls	1984	18	13.76
Antigua and Barbuda	Girls	1985	18	13.89
Antigua and Barbuda	Girls	1986	18	14.02
Antigua and Barbuda	Girls	1987	18	14.16
Antigua and Barbuda	Girls	1988	18	14.31
Antigua and Barbuda	Girls	1989	18	14.46
Antigua and Barbuda	Girls	1990	18	14.62
Antigua and Barbuda	Girls	1991	18	14.78
Antigua and Barbuda	Girls	1992	18	14.94
Antigua and Barbuda	Girls	1993	18	15.10
Antigua and Barbuda	Girls	1994	18	15.26
Antigua and Barbuda	Girls	1995	18	15.43
Antigua and Barbuda	Girls	1996	18	15.60
Antigua and Barbuda	Girls	1997	18	15.78
Antigua and Barbuda	Girls	1998	18	15.96
Antigua and Barbuda	Girls	1999	18	16.14
Antigua and Barbuda	Girls	2000	18	16.33
Antigua and Barbuda	Girls	2001	18	16.52
Antigua and Barbuda	Girls	2002	18	16.72
Antigua and Barbuda	Girls	2003	18	16.92
Antigua and Barbuda	Girls	2004	18	17.13
Antigua and Barbuda	Girls	2005	18	17.34
Antigua and Barbuda	Girls	2006	18	17.56
Antigua and Barbuda	Girls	2007	18	17.79
Antigua and Barbuda	Girls	2008	18	18.03
Antigua and Barbuda	Girls	2009	18	18.27
Antigua and Barbuda	Girls	2010	18	18.53
Antigua and Barbuda	Girls	2011	18	18.80
Antigua and Barbuda	Girls	2012	18	19.08
Antigua and Barbuda	Girls	2013	18	19.38
Antigua and Barbuda	Girls	2014	18	19.69
Antigua and Barbuda	Girls	2015	18	20.01
Antigua and Barbuda	Girls	2016	18	20.35
Antigua and Barbuda	Girls	1975	Average	9.86
Antigua and Barbuda	Girls	1976	Average	9.92
Antigua and Barbuda	Girls	1977	Average	9.99
Antigua and Barbuda	Girls	1978	Average	10.06
Antigua and Barbuda	Girls	1979	Average	10.14
Antigua and Barbuda	Girls	1980	Average	10.22
Antigua and Barbuda	Girls	1981	Average	10.31
Antigua and Barbuda	Girls	1982	Average	10.40
Antigua and Barbuda	Girls	1983	Average	10.50
Antigua and Barbuda	Girls	1984	Average	10.60
Antigua and Barbuda	Girls	1985	Average	10.71
Antigua and Barbuda	Girls	1986	Average	10.82
Antigua and Barbuda	Girls	1987	Average	10.94
Antigua and Barbuda	Girls	1988	Average	11.06
Antigua and Barbuda	Girls	1989	Average	11.18
Antigua and Barbuda	Girls	1990	Average	11.31
Antigua and Barbuda	Girls	1991	Average	11.45

Antigua and Barbuda	Girls	1992	Average	11.58
Antigua and Barbuda	Girls	1993	Average	11.72
Antigua and Barbuda	Girls	1994	Average	11.85
Antigua and Barbuda	Girls	1995	Average	12.00
Antigua and Barbuda	Girls	1996	Average	12.14
Antigua and Barbuda	Girls	1997	Average	12.28
Antigua and Barbuda	Girls	1998	Average	12.43
Antigua and Barbuda	Girls	1999	Average	12.59
Antigua and Barbuda	Girls	2000	Average	12.74
Antigua and Barbuda	Girls	2001	Average	12.91
Antigua and Barbuda	Girls	2002	Average	13.07
Antigua and Barbuda	Girls	2003	Average	13.24
Antigua and Barbuda	Girls	2004	Average	13.41
Antigua and Barbuda	Girls	2005	Average	13.59
Antigua and Barbuda	Girls	2006	Average	13.78
Antigua and Barbuda	Girls	2007	Average	13.97
Antigua and Barbuda	Girls	2008	Average	14.16
Antigua and Barbuda	Girls	2009	Average	14.37
Antigua and Barbuda	Girls	2010	Average	14.59
Antigua and Barbuda	Girls	2011	Average	14.81
Antigua and Barbuda	Girls	2012	Average	15.05
Antigua and Barbuda	Girls	2013	Average	15.30
Antigua and Barbuda	Girls	2014	Average	15.56
Antigua and Barbuda	Girls	2015	Average	15.83
Antigua and Barbuda	Girls	2016	Average	16.12
Argentina	Boys	1975	12	8.97
Argentina	Boys	1976	12	9.08
Argentina	Boys	1977	12	9.19
Argentina	Boys	1978	12	9.30
Argentina	Boys	1979	12	9.42
Argentina	Boys	1980	12	9.54
Argentina	Boys	1981	12	9.66
Argentina	Boys	1982	12	9.79
Argentina	Boys	1983	12	9.93
Argentina	Boys	1984	12	10.07
Argentina	Boys	1985	12	10.21
Argentina	Boys	1986	12	10.35
Argentina	Boys	1987	12	10.50
Argentina	Boys	1988	12	10.66
Argentina	Boys	1989	12	10.81
Argentina	Boys	1990	12	10.97
Argentina	Boys	1991	12	11.13
Argentina	Boys	1992	12	11.29
Argentina	Boys	1993	12	11.45
Argentina	Boys	1994	12	11.61
Argentina	Boys	1995	12	11.77
Argentina	Boys	1996	12	11.94
Argentina	Boys	1997	12	12.10
Argentina	Boys	1998	12	12.26
Argentina	Boys	1999	12	12.43
Argentina	Boys	2000	12	12.60
Argentina	Boys	2001	12	12.77

Argentina	Boys	2002	12	12.94
Argentina	Boys	2003	12	13.12
Argentina	Boys	2004	12	13.31
Argentina	Boys	2005	12	13.49
Argentina	Boys	2006	12	13.68
Argentina	Boys	2007	12	13.87
Argentina	Boys	2008	12	14.06
Argentina	Boys	2009	12	14.26
Argentina	Boys	2010	12	14.45
Argentina	Boys	2011	12	14.63
Argentina	Boys	2012	12	14.82
Argentina	Boys	2013	12	15.00
Argentina	Boys	2014	12	15.19
Argentina	Boys	2015	12	15.37
Argentina	Boys	2016	12	15.55
Argentina	Boys	1975	13	10.91
Argentina	Boys	1976	13	11.04
Argentina	Boys	1977	13	11.17
Argentina	Boys	1978	13	11.30
Argentina	Boys	1979	13	11.43
Argentina	Boys	1980	13	11.57
Argentina	Boys	1981	13	11.72
Argentina	Boys	1982	13	11.87
Argentina	Boys	1983	13	12.03
Argentina	Boys	1984	13	12.20
Argentina	Boys	1985	13	12.36
Argentina	Boys	1986	13	12.54
Argentina	Boys	1987	13	12.71
Argentina	Boys	1988	13	12.90
Argentina	Boys	1989	13	13.08
Argentina	Boys	1990	13	13.27
Argentina	Boys	1991	13	13.46
Argentina	Boys	1992	13	13.66
Argentina	Boys	1993	13	13.85
Argentina	Boys	1994	13	14.04
Argentina	Boys	1995	13	14.24
Argentina	Boys	1996	13	14.43
Argentina	Boys	1997	13	14.63
Argentina	Boys	1998	13	14.83
Argentina	Boys	1999	13	15.03
Argentina	Boys	2000	13	15.24
Argentina	Boys	2001	13	15.45
Argentina	Boys	2002	13	15.66
Argentina	Boys	2003	13	15.88
Argentina	Boys	2004	13	16.11
Argentina	Boys	2005	13	16.34
Argentina	Boys	2006	13	16.57
Argentina	Boys	2007	13	16.81
Argentina	Boys	2008	13	17.05
Argentina	Boys	2009	13	17.29
Argentina	Boys	2010	13	17.53
Argentina	Boys	2011	13	17.76

Argentina	Boys	2012	13	18.00
Argentina	Boys	2013	13	18.24
Argentina	Boys	2014	13	18.47
Argentina	Boys	2015	13	18.70
Argentina	Boys	2016	13	18.94
Argentina	Boys	1975	14	13.07
Argentina	Boys	1976	14	13.20
Argentina	Boys	1977	14	13.34
Argentina	Boys	1978	14	13.49
Argentina	Boys	1979	14	13.64
Argentina	Boys	1980	14	13.79
Argentina	Boys	1981	14	13.96
Argentina	Boys	1982	14	14.12
Argentina	Boys	1983	14	14.30
Argentina	Boys	1984	14	14.48
Argentina	Boys	1985	14	14.67
Argentina	Boys	1986	14	14.86
Argentina	Boys	1987	14	15.06
Argentina	Boys	1988	14	15.26
Argentina	Boys	1989	14	15.47
Argentina	Boys	1990	14	15.68
Argentina	Boys	1991	14	15.90
Argentina	Boys	1992	14	16.11
Argentina	Boys	1993	14	16.33
Argentina	Boys	1994	14	16.55
Argentina	Boys	1995	14	16.77
Argentina	Boys	1996	14	16.99
Argentina	Boys	1997	14	17.22
Argentina	Boys	1998	14	17.44
Argentina	Boys	1999	14	17.67
Argentina	Boys	2000	14	17.91
Argentina	Boys	2001	14	18.15
Argentina	Boys	2002	14	18.40
Argentina	Boys	2003	14	18.65
Argentina	Boys	2004	14	18.91
Argentina	Boys	2005	14	19.18
Argentina	Boys	2006	14	19.45
Argentina	Boys	2007	14	19.73
Argentina	Boys	2008	14	20.00
Argentina	Boys	2009	14	20.29
Argentina	Boys	2010	14	20.57
Argentina	Boys	2011	14	20.85
Argentina	Boys	2012	14	21.13
Argentina	Boys	2013	14	21.41
Argentina	Boys	2014	14	21.69
Argentina	Boys	2015	14	21.97
Argentina	Boys	2016	14	22.25
Argentina	Boys	1975	15	15.31
Argentina	Boys	1976	15	15.46
Argentina	Boys	1977	15	15.61
Argentina	Boys	1978	15	15.76
Argentina	Boys	1979	15	15.91

Argentina	Boys	1980	15	16.08
Argentina	Boys	1981	15	16.24
Argentina	Boys	1982	15	16.42
Argentina	Boys	1983	15	16.60
Argentina	Boys	1984	15	16.79
Argentina	Boys	1985	15	16.99
Argentina	Boys	1986	15	17.19
Argentina	Boys	1987	15	17.40
Argentina	Boys	1988	15	17.62
Argentina	Boys	1989	15	17.84
Argentina	Boys	1990	15	18.07
Argentina	Boys	1991	15	18.29
Argentina	Boys	1992	15	18.52
Argentina	Boys	1993	15	18.75
Argentina	Boys	1994	15	18.99
Argentina	Boys	1995	15	19.22
Argentina	Boys	1996	15	19.46
Argentina	Boys	1997	15	19.70
Argentina	Boys	1998	15	19.94
Argentina	Boys	1999	15	20.19
Argentina	Boys	2000	15	20.44
Argentina	Boys	2001	15	20.70
Argentina	Boys	2002	15	20.97
Argentina	Boys	2003	15	21.24
Argentina	Boys	2004	15	21.52
Argentina	Boys	2005	15	21.81
Argentina	Boys	2006	15	22.11
Argentina	Boys	2007	15	22.41
Argentina	Boys	2008	15	22.72
Argentina	Boys	2009	15	23.03
Argentina	Boys	2010	15	23.34
Argentina	Boys	2011	15	23.65
Argentina	Boys	2012	15	23.96
Argentina	Boys	2013	15	24.27
Argentina	Boys	2014	15	24.59
Argentina	Boys	2015	15	24.91
Argentina	Boys	2016	15	25.22
Argentina	Boys	1975	16	17.62
Argentina	Boys	1976	16	17.77
Argentina	Boys	1977	16	17.92
Argentina	Boys	1978	16	18.07
Argentina	Boys	1979	16	18.23
Argentina	Boys	1980	16	18.40
Argentina	Boys	1981	16	18.57
Argentina	Boys	1982	16	18.75
Argentina	Boys	1983	16	18.93
Argentina	Boys	1984	16	19.13
Argentina	Boys	1985	16	19.33
Argentina	Boys	1986	16	19.53
Argentina	Boys	1987	16	19.75
Argentina	Boys	1988	16	19.97
Argentina	Boys	1989	16	20.19

Argentina	Boys	1990	16	20.42
Argentina	Boys	1991	16	20.66
Argentina	Boys	1992	16	20.89
Argentina	Boys	1993	16	21.13
Argentina	Boys	1994	16	21.37
Argentina	Boys	1995	16	21.61
Argentina	Boys	1996	16	21.86
Argentina	Boys	1997	16	22.10
Argentina	Boys	1998	16	22.35
Argentina	Boys	1999	16	22.61
Argentina	Boys	2000	16	22.87
Argentina	Boys	2001	16	23.14
Argentina	Boys	2002	16	23.42
Argentina	Boys	2003	16	23.70
Argentina	Boys	2004	16	23.99
Argentina	Boys	2005	16	24.30
Argentina	Boys	2006	16	24.61
Argentina	Boys	2007	16	24.93
Argentina	Boys	2008	16	25.25
Argentina	Boys	2009	16	25.57
Argentina	Boys	2010	16	25.90
Argentina	Boys	2011	16	26.23
Argentina	Boys	2012	16	26.56
Argentina	Boys	2013	16	26.89
Argentina	Boys	2014	16	27.23
Argentina	Boys	2015	16	27.56
Argentina	Boys	2016	16	27.90
Argentina	Boys	1975	17	19.98
Argentina	Boys	1976	17	20.13
Argentina	Boys	1977	17	20.28
Argentina	Boys	1978	17	20.44
Argentina	Boys	1979	17	20.60
Argentina	Boys	1980	17	20.76
Argentina	Boys	1981	17	20.94
Argentina	Boys	1982	17	21.12
Argentina	Boys	1983	17	21.31
Argentina	Boys	1984	17	21.50
Argentina	Boys	1985	17	21.71
Argentina	Boys	1986	17	21.91
Argentina	Boys	1987	17	22.13
Argentina	Boys	1988	17	22.35
Argentina	Boys	1989	17	22.58
Argentina	Boys	1990	17	22.82
Argentina	Boys	1991	17	23.05
Argentina	Boys	1992	17	23.29
Argentina	Boys	1993	17	23.53
Argentina	Boys	1994	17	23.78
Argentina	Boys	1995	17	24.02
Argentina	Boys	1996	17	24.27
Argentina	Boys	1997	17	24.52
Argentina	Boys	1998	17	24.77
Argentina	Boys	1999	17	25.03

Argentina	Boys	2000	17	25.30
Argentina	Boys	2001	17	25.57
Argentina	Boys	2002	17	25.86
Argentina	Boys	2003	17	26.15
Argentina	Boys	2004	17	26.45
Argentina	Boys	2005	17	26.76
Argentina	Boys	2006	17	27.08
Argentina	Boys	2007	17	27.40
Argentina	Boys	2008	17	27.73
Argentina	Boys	2009	17	28.07
Argentina	Boys	2010	17	28.41
Argentina	Boys	2011	17	28.75
Argentina	Boys	2012	17	29.09
Argentina	Boys	2013	17	29.43
Argentina	Boys	2014	17	29.78
Argentina	Boys	2015	17	30.13
Argentina	Boys	2016	17	30.49
Argentina	Boys	1975	18	22.43
Argentina	Boys	1976	18	22.58
Argentina	Boys	1977	18	22.74
Argentina	Boys	1978	18	22.90
Argentina	Boys	1979	18	23.06
Argentina	Boys	1980	18	23.23
Argentina	Boys	1981	18	23.41
Argentina	Boys	1982	18	23.60
Argentina	Boys	1983	18	23.79
Argentina	Boys	1984	18	23.99
Argentina	Boys	1985	18	24.20
Argentina	Boys	1986	18	24.42
Argentina	Boys	1987	18	24.64
Argentina	Boys	1988	18	24.87
Argentina	Boys	1989	18	25.10
Argentina	Boys	1990	18	25.34
Argentina	Boys	1991	18	25.59
Argentina	Boys	1992	18	25.83
Argentina	Boys	1993	18	26.08
Argentina	Boys	1994	18	26.33
Argentina	Boys	1995	18	26.58
Argentina	Boys	1996	18	26.83
Argentina	Boys	1997	18	27.09
Argentina	Boys	1998	18	27.35
Argentina	Boys	1999	18	27.62
Argentina	Boys	2000	18	27.89
Argentina	Boys	2001	18	28.17
Argentina	Boys	2002	18	28.46
Argentina	Boys	2003	18	28.76
Argentina	Boys	2004	18	29.07
Argentina	Boys	2005	18	29.39
Argentina	Boys	2006	18	29.72
Argentina	Boys	2007	18	30.05
Argentina	Boys	2008	18	30.39
Argentina	Boys	2009	18	30.73

Argentina	Boys	2010	18	31.08
Argentina	Boys	2011	18	31.43
Argentina	Boys	2012	18	31.78
Argentina	Boys	2013	18	32.13
Argentina	Boys	2014	18	32.49
Argentina	Boys	2015	18	32.85
Argentina	Boys	2016	18	33.22
Argentina	Boys	1975	Average	15.47
Argentina	Boys	1976	Average	15.61
Argentina	Boys	1977	Average	15.75
Argentina	Boys	1978	Average	15.89
Argentina	Boys	1979	Average	16.04
Argentina	Boys	1980	Average	16.20
Argentina	Boys	1981	Average	16.36
Argentina	Boys	1982	Average	16.53
Argentina	Boys	1983	Average	16.70
Argentina	Boys	1984	Average	16.88
Argentina	Boys	1985	Average	17.07
Argentina	Boys	1986	Average	17.26
Argentina	Boys	1987	Average	17.46
Argentina	Boys	1988	Average	17.66
Argentina	Boys	1989	Average	17.87
Argentina	Boys	1990	Average	18.08
Argentina	Boys	1991	Average	18.30
Argentina	Boys	1992	Average	18.51
Argentina	Boys	1993	Average	18.73
Argentina	Boys	1994	Average	18.95
Argentina	Boys	1995	Average	19.17
Argentina	Boys	1996	Average	19.40
Argentina	Boys	1997	Average	19.62
Argentina	Boys	1998	Average	19.85
Argentina	Boys	1999	Average	20.08
Argentina	Boys	2000	Average	20.32
Argentina	Boys	2001	Average	20.57
Argentina	Boys	2002	Average	20.81
Argentina	Boys	2003	Average	21.07
Argentina	Boys	2004	Average	21.34
Argentina	Boys	2005	Average	21.61
Argentina	Boys	2006	Average	21.89
Argentina	Boys	2007	Average	22.17
Argentina	Boys	2008	Average	22.46
Argentina	Boys	2009	Average	22.75
Argentina	Boys	2010	Average	23.04
Argentina	Boys	2011	Average	23.33
Argentina	Boys	2012	Average	23.62
Argentina	Boys	2013	Average	23.91
Argentina	Boys	2014	Average	24.20
Argentina	Boys	2015	Average	24.50
Argentina	Boys	2016	Average	24.80
Argentina	Girls	1975	12	8.59
Argentina	Girls	1976	12	8.66
Argentina	Girls	1977	12	8.74

Argentina	Girls	1978	12	8.81
Argentina	Girls	1979	12	8.89
Argentina	Girls	1980	12	8.97
Argentina	Girls	1981	12	9.04
Argentina	Girls	1982	12	9.13
Argentina	Girls	1983	12	9.21
Argentina	Girls	1984	12	9.29
Argentina	Girls	1985	12	9.38
Argentina	Girls	1986	12	9.46
Argentina	Girls	1987	12	9.55
Argentina	Girls	1988	12	9.64
Argentina	Girls	1989	12	9.73
Argentina	Girls	1990	12	9.82
Argentina	Girls	1991	12	9.90
Argentina	Girls	1992	12	9.99
Argentina	Girls	1993	12	10.07
Argentina	Girls	1994	12	10.15
Argentina	Girls	1995	12	10.23
Argentina	Girls	1996	12	10.31
Argentina	Girls	1997	12	10.38
Argentina	Girls	1998	12	10.46
Argentina	Girls	1999	12	10.53
Argentina	Girls	2000	12	10.61
Argentina	Girls	2001	12	10.68
Argentina	Girls	2002	12	10.76
Argentina	Girls	2003	12	10.83
Argentina	Girls	2004	12	10.91
Argentina	Girls	2005	12	10.98
Argentina	Girls	2006	12	11.06
Argentina	Girls	2007	12	11.14
Argentina	Girls	2008	12	11.22
Argentina	Girls	2009	12	11.30
Argentina	Girls	2010	12	11.38
Argentina	Girls	2011	12	11.46
Argentina	Girls	2012	12	11.55
Argentina	Girls	2013	12	11.63
Argentina	Girls	2014	12	11.72
Argentina	Girls	2015	12	11.81
Argentina	Girls	2016	12	11.90
Argentina	Girls	1975	13	10.37
Argentina	Girls	1976	13	10.45
Argentina	Girls	1977	13	10.53
Argentina	Girls	1978	13	10.61
Argentina	Girls	1979	13	10.70
Argentina	Girls	1980	13	10.78
Argentina	Girls	1981	13	10.87
Argentina	Girls	1982	13	10.96
Argentina	Girls	1983	13	11.05
Argentina	Girls	1984	13	11.15
Argentina	Girls	1985	13	11.24
Argentina	Girls	1986	13	11.34
Argentina	Girls	1987	13	11.44

Argentina	Girls	1988	13	11.53
Argentina	Girls	1989	13	11.63
Argentina	Girls	1990	13	11.73
Argentina	Girls	1991	13	11.83
Argentina	Girls	1992	13	11.92
Argentina	Girls	1993	13	12.01
Argentina	Girls	1994	13	12.10
Argentina	Girls	1995	13	12.19
Argentina	Girls	1996	13	12.28
Argentina	Girls	1997	13	12.36
Argentina	Girls	1998	13	12.45
Argentina	Girls	1999	13	12.53
Argentina	Girls	2000	13	12.61
Argentina	Girls	2001	13	12.69
Argentina	Girls	2002	13	12.78
Argentina	Girls	2003	13	12.86
Argentina	Girls	2004	13	12.94
Argentina	Girls	2005	13	13.03
Argentina	Girls	2006	13	13.11
Argentina	Girls	2007	13	13.20
Argentina	Girls	2008	13	13.29
Argentina	Girls	2009	13	13.38
Argentina	Girls	2010	13	13.47
Argentina	Girls	2011	13	13.57
Argentina	Girls	2012	13	13.66
Argentina	Girls	2013	13	13.76
Argentina	Girls	2014	13	13.86
Argentina	Girls	2015	13	13.96
Argentina	Girls	2016	13	14.07
Argentina	Girls	1975	14	12.12
Argentina	Girls	1976	14	12.20
Argentina	Girls	1977	14	12.29
Argentina	Girls	1978	14	12.38
Argentina	Girls	1979	14	12.47
Argentina	Girls	1980	14	12.56
Argentina	Girls	1981	14	12.65
Argentina	Girls	1982	14	12.75
Argentina	Girls	1983	14	12.84
Argentina	Girls	1984	14	12.94
Argentina	Girls	1985	14	13.04
Argentina	Girls	1986	14	13.15
Argentina	Girls	1987	14	13.25
Argentina	Girls	1988	14	13.35
Argentina	Girls	1989	14	13.46
Argentina	Girls	1990	14	13.56
Argentina	Girls	1991	14	13.66
Argentina	Girls	1992	14	13.76
Argentina	Girls	1993	14	13.86
Argentina	Girls	1994	14	13.95
Argentina	Girls	1995	14	14.04
Argentina	Girls	1996	14	14.13
Argentina	Girls	1997	14	14.22

Argentina	Girls	1998	14	14.31
Argentina	Girls	1999	14	14.40
Argentina	Girls	2000	14	14.48
Argentina	Girls	2001	14	14.57
Argentina	Girls	2002	14	14.66
Argentina	Girls	2003	14	14.74
Argentina	Girls	2004	14	14.83
Argentina	Girls	2005	14	14.92
Argentina	Girls	2006	14	15.01
Argentina	Girls	2007	14	15.10
Argentina	Girls	2008	14	15.20
Argentina	Girls	2009	14	15.29
Argentina	Girls	2010	14	15.39
Argentina	Girls	2011	14	15.49
Argentina	Girls	2012	14	15.60
Argentina	Girls	2013	14	15.70
Argentina	Girls	2014	14	15.81
Argentina	Girls	2015	14	15.92
Argentina	Girls	2016	14	16.03
Argentina	Girls	1975	15	13.64
Argentina	Girls	1976	15	13.72
Argentina	Girls	1977	15	13.81
Argentina	Girls	1978	15	13.90
Argentina	Girls	1979	15	13.99
Argentina	Girls	1980	15	14.08
Argentina	Girls	1981	15	14.18
Argentina	Girls	1982	15	14.27
Argentina	Girls	1983	15	14.37
Argentina	Girls	1984	15	14.47
Argentina	Girls	1985	15	14.58
Argentina	Girls	1986	15	14.68
Argentina	Girls	1987	15	14.79
Argentina	Girls	1988	15	14.89
Argentina	Girls	1989	15	15.00
Argentina	Girls	1990	15	15.10
Argentina	Girls	1991	15	15.21
Argentina	Girls	1992	15	15.31
Argentina	Girls	1993	15	15.41
Argentina	Girls	1994	15	15.50
Argentina	Girls	1995	15	15.60
Argentina	Girls	1996	15	15.69
Argentina	Girls	1997	15	15.78
Argentina	Girls	1998	15	15.87
Argentina	Girls	1999	15	15.95
Argentina	Girls	2000	15	16.04
Argentina	Girls	2001	15	16.13
Argentina	Girls	2002	15	16.22
Argentina	Girls	2003	15	16.30
Argentina	Girls	2004	15	16.39
Argentina	Girls	2005	15	16.48
Argentina	Girls	2006	15	16.57
Argentina	Girls	2007	15	16.67

Argentina	Girls	2008	15	16.76
Argentina	Girls	2009	15	16.86
Argentina	Girls	2010	15	16.96
Argentina	Girls	2011	15	17.07
Argentina	Girls	2012	15	17.17
Argentina	Girls	2013	15	17.28
Argentina	Girls	2014	15	17.39
Argentina	Girls	2015	15	17.50
Argentina	Girls	2016	15	17.62
Argentina	Girls	1975	16	14.81
Argentina	Girls	1976	16	14.90
Argentina	Girls	1977	16	14.99
Argentina	Girls	1978	16	15.08
Argentina	Girls	1979	16	15.17
Argentina	Girls	1980	16	15.26
Argentina	Girls	1981	16	15.36
Argentina	Girls	1982	16	15.45
Argentina	Girls	1983	16	15.55
Argentina	Girls	1984	16	15.66
Argentina	Girls	1985	16	15.76
Argentina	Girls	1986	16	15.87
Argentina	Girls	1987	16	15.97
Argentina	Girls	1988	16	16.08
Argentina	Girls	1989	16	16.19
Argentina	Girls	1990	16	16.29
Argentina	Girls	1991	16	16.40
Argentina	Girls	1992	16	16.50
Argentina	Girls	1993	16	16.60
Argentina	Girls	1994	16	16.69
Argentina	Girls	1995	16	16.79
Argentina	Girls	1996	16	16.88
Argentina	Girls	1997	16	16.97
Argentina	Girls	1998	16	17.06
Argentina	Girls	1999	16	17.15
Argentina	Girls	2000	16	17.24
Argentina	Girls	2001	16	17.32
Argentina	Girls	2002	16	17.41
Argentina	Girls	2003	16	17.50
Argentina	Girls	2004	16	17.59
Argentina	Girls	2005	16	17.68
Argentina	Girls	2006	16	17.77
Argentina	Girls	2007	16	17.86
Argentina	Girls	2008	16	17.96
Argentina	Girls	2009	16	18.06
Argentina	Girls	2010	16	18.16
Argentina	Girls	2011	16	18.27
Argentina	Girls	2012	16	18.37
Argentina	Girls	2013	16	18.48
Argentina	Girls	2014	16	18.60
Argentina	Girls	2015	16	18.71
Argentina	Girls	2016	16	18.83
Argentina	Girls	1975	17	15.69

Argentina	Girls	1976	17	15.78
Argentina	Girls	1977	17	15.87
Argentina	Girls	1978	17	15.97
Argentina	Girls	1979	17	16.06
Argentina	Girls	1980	17	16.15
Argentina	Girls	1981	17	16.25
Argentina	Girls	1982	17	16.35
Argentina	Girls	1983	17	16.46
Argentina	Girls	1984	17	16.56
Argentina	Girls	1985	17	16.67
Argentina	Girls	1986	17	16.78
Argentina	Girls	1987	17	16.89
Argentina	Girls	1988	17	17.00
Argentina	Girls	1989	17	17.11
Argentina	Girls	1990	17	17.22
Argentina	Girls	1991	17	17.32
Argentina	Girls	1992	17	17.43
Argentina	Girls	1993	17	17.53
Argentina	Girls	1994	17	17.63
Argentina	Girls	1995	17	17.73
Argentina	Girls	1996	17	17.82
Argentina	Girls	1997	17	17.91
Argentina	Girls	1998	17	18.01
Argentina	Girls	1999	17	18.10
Argentina	Girls	2000	17	18.19
Argentina	Girls	2001	17	18.28
Argentina	Girls	2002	17	18.37
Argentina	Girls	2003	17	18.46
Argentina	Girls	2004	17	18.55
Argentina	Girls	2005	17	18.64
Argentina	Girls	2006	17	18.73
Argentina	Girls	2007	17	18.83
Argentina	Girls	2008	17	18.93
Argentina	Girls	2009	17	19.03
Argentina	Girls	2010	17	19.14
Argentina	Girls	2011	17	19.25
Argentina	Girls	2012	17	19.35
Argentina	Girls	2013	17	19.47
Argentina	Girls	2014	17	19.58
Argentina	Girls	2015	17	19.70
Argentina	Girls	2016	17	19.83
Argentina	Girls	1975	18	16.46
Argentina	Girls	1976	18	16.56
Argentina	Girls	1977	18	16.65
Argentina	Girls	1978	18	16.75
Argentina	Girls	1979	18	16.85
Argentina	Girls	1980	18	16.95
Argentina	Girls	1981	18	17.06
Argentina	Girls	1982	18	17.17
Argentina	Girls	1983	18	17.28
Argentina	Girls	1984	18	17.39
Argentina	Girls	1985	18	17.50

Argentina	Girls	1986	18	17.62
Argentina	Girls	1987	18	17.74
Argentina	Girls	1988	18	17.85
Argentina	Girls	1989	18	17.97
Argentina	Girls	1990	18	18.09
Argentina	Girls	1991	18	18.20
Argentina	Girls	1992	18	18.32
Argentina	Girls	1993	18	18.43
Argentina	Girls	1994	18	18.53
Argentina	Girls	1995	18	18.64
Argentina	Girls	1996	18	18.74
Argentina	Girls	1997	18	18.84
Argentina	Girls	1998	18	18.94
Argentina	Girls	1999	18	19.03
Argentina	Girls	2000	18	19.13
Argentina	Girls	2001	18	19.23
Argentina	Girls	2002	18	19.32
Argentina	Girls	2003	18	19.42
Argentina	Girls	2004	18	19.52
Argentina	Girls	2005	18	19.62
Argentina	Girls	2006	18	19.72
Argentina	Girls	2007	18	19.82
Argentina	Girls	2008	18	19.93
Argentina	Girls	2009	18	20.04
Argentina	Girls	2010	18	20.15
Argentina	Girls	2011	18	20.26
Argentina	Girls	2012	18	20.38
Argentina	Girls	2013	18	20.50
Argentina	Girls	2014	18	20.62
Argentina	Girls	2015	18	20.75
Argentina	Girls	2016	18	20.88
Argentina	Girls	1975	Average	13.10
Argentina	Girls	1976	Average	13.18
Argentina	Girls	1977	Average	13.27
Argentina	Girls	1978	Average	13.36
Argentina	Girls	1979	Average	13.45
Argentina	Girls	1980	Average	13.54
Argentina	Girls	1981	Average	13.63
Argentina	Girls	1982	Average	13.73
Argentina	Girls	1983	Average	13.82
Argentina	Girls	1984	Average	13.92
Argentina	Girls	1985	Average	14.03
Argentina	Girls	1986	Average	14.13
Argentina	Girls	1987	Average	14.23
Argentina	Girls	1988	Average	14.34
Argentina	Girls	1989	Average	14.44
Argentina	Girls	1990	Average	14.54
Argentina	Girls	1991	Average	14.65
Argentina	Girls	1992	Average	14.75
Argentina	Girls	1993	Average	14.84
Argentina	Girls	1994	Average	14.94
Argentina	Girls	1995	Average	15.03

Argentina	Girls	1996	Average	15.12
Argentina	Girls	1997	Average	15.21
Argentina	Girls	1998	Average	15.30
Argentina	Girls	1999	Average	15.39
Argentina	Girls	2000	Average	15.47
Argentina	Girls	2001	Average	15.56
Argentina	Girls	2002	Average	15.64
Argentina	Girls	2003	Average	15.73
Argentina	Girls	2004	Average	15.82
Argentina	Girls	2005	Average	15.91
Argentina	Girls	2006	Average	16.00
Argentina	Girls	2007	Average	16.09
Argentina	Girls	2008	Average	16.18
Argentina	Girls	2009	Average	16.28
Argentina	Girls	2010	Average	16.38
Argentina	Girls	2011	Average	16.48
Argentina	Girls	2012	Average	16.58
Argentina	Girls	2013	Average	16.69
Argentina	Girls	2014	Average	16.80
Argentina	Girls	2015	Average	16.91
Argentina	Girls	2016	Average	17.02
Armenia	Boys	1975	12	6.31
Armenia	Boys	1976	12	6.38
Armenia	Boys	1977	12	6.45
Armenia	Boys	1978	12	6.53
Armenia	Boys	1979	12	6.60
Armenia	Boys	1980	12	6.68
Armenia	Boys	1981	12	6.75
Armenia	Boys	1982	12	6.82
Armenia	Boys	1983	12	6.89
Armenia	Boys	1984	12	6.96
Armenia	Boys	1985	12	7.03
Armenia	Boys	1986	12	7.10
Armenia	Boys	1987	12	7.17
Armenia	Boys	1988	12	7.24
Armenia	Boys	1989	12	7.31
Armenia	Boys	1990	12	7.37
Armenia	Boys	1991	12	7.44
Armenia	Boys	1992	12	7.50
Armenia	Boys	1993	12	7.56
Armenia	Boys	1994	12	7.62
Armenia	Boys	1995	12	7.68
Armenia	Boys	1996	12	7.74
Armenia	Boys	1997	12	7.80
Armenia	Boys	1998	12	7.86
Armenia	Boys	1999	12	7.92
Armenia	Boys	2000	12	7.98
Armenia	Boys	2001	12	8.05
Armenia	Boys	2002	12	8.12
Armenia	Boys	2003	12	8.19
Armenia	Boys	2004	12	8.26
Armenia	Boys	2005	12	8.34

Armenia	Boys	2006	12	8.42
Armenia	Boys	2007	12	8.51
Armenia	Boys	2008	12	8.60
Armenia	Boys	2009	12	8.69
Armenia	Boys	2010	12	8.78
Armenia	Boys	2011	12	8.88
Armenia	Boys	2012	12	8.98
Armenia	Boys	2013	12	9.08
Armenia	Boys	2014	12	9.19
Armenia	Boys	2015	12	9.30
Armenia	Boys	2016	12	9.42
Armenia	Boys	1975	13	7.79
Armenia	Boys	1976	13	7.88
Armenia	Boys	1977	13	7.96
Armenia	Boys	1978	13	8.05
Armenia	Boys	1979	13	8.14
Armenia	Boys	1980	13	8.22
Armenia	Boys	1981	13	8.31
Armenia	Boys	1982	13	8.40
Armenia	Boys	1983	13	8.48
Armenia	Boys	1984	13	8.56
Armenia	Boys	1985	13	8.64
Armenia	Boys	1986	13	8.72
Armenia	Boys	1987	13	8.81
Armenia	Boys	1988	13	8.88
Armenia	Boys	1989	13	8.96
Armenia	Boys	1990	13	9.04
Armenia	Boys	1991	13	9.12
Armenia	Boys	1992	13	9.19
Armenia	Boys	1993	13	9.27
Armenia	Boys	1994	13	9.34
Armenia	Boys	1995	13	9.41
Armenia	Boys	1996	13	9.47
Armenia	Boys	1997	13	9.54
Armenia	Boys	1998	13	9.61
Armenia	Boys	1999	13	9.68
Armenia	Boys	2000	13	9.76
Armenia	Boys	2001	13	9.83
Armenia	Boys	2002	13	9.91
Armenia	Boys	2003	13	10.00
Armenia	Boys	2004	13	10.09
Armenia	Boys	2005	13	10.18
Armenia	Boys	2006	13	10.28
Armenia	Boys	2007	13	10.38
Armenia	Boys	2008	13	10.48
Armenia	Boys	2009	13	10.58
Armenia	Boys	2010	13	10.69
Armenia	Boys	2011	13	10.81
Armenia	Boys	2012	13	10.92
Armenia	Boys	2013	13	11.05
Armenia	Boys	2014	13	11.17
Armenia	Boys	2015	13	11.31

Armenia	Boys	2016	13	11.45
Armenia	Boys	1975	14	9.59
Armenia	Boys	1976	14	9.69
Armenia	Boys	1977	14	9.78
Armenia	Boys	1978	14	9.88
Armenia	Boys	1979	14	9.98
Armenia	Boys	1980	14	10.08
Armenia	Boys	1981	14	10.18
Armenia	Boys	1982	14	10.27
Armenia	Boys	1983	14	10.36
Armenia	Boys	1984	14	10.46
Armenia	Boys	1985	14	10.55
Armenia	Boys	1986	14	10.64
Armenia	Boys	1987	14	10.73
Armenia	Boys	1988	14	10.82
Armenia	Boys	1989	14	10.91
Armenia	Boys	1990	14	10.99
Armenia	Boys	1991	14	11.08
Armenia	Boys	1992	14	11.16
Armenia	Boys	1993	14	11.24
Armenia	Boys	1994	14	11.32
Armenia	Boys	1995	14	11.40
Armenia	Boys	1996	14	11.47
Armenia	Boys	1997	14	11.55
Armenia	Boys	1998	14	11.63
Armenia	Boys	1999	14	11.71
Armenia	Boys	2000	14	11.79
Armenia	Boys	2001	14	11.87
Armenia	Boys	2002	14	11.96
Armenia	Boys	2003	14	12.06
Armenia	Boys	2004	14	12.15
Armenia	Boys	2005	14	12.26
Armenia	Boys	2006	14	12.36
Armenia	Boys	2007	14	12.47
Armenia	Boys	2008	14	12.59
Armenia	Boys	2009	14	12.71
Armenia	Boys	2010	14	12.83
Armenia	Boys	2011	14	12.95
Armenia	Boys	2012	14	13.08
Armenia	Boys	2013	14	13.22
Armenia	Boys	2014	14	13.36
Armenia	Boys	2015	14	13.51
Armenia	Boys	2016	14	13.67
Armenia	Boys	1975	15	11.63
Armenia	Boys	1976	15	11.73
Armenia	Boys	1977	15	11.84
Armenia	Boys	1978	15	11.94
Armenia	Boys	1979	15	12.05
Armenia	Boys	1980	15	12.15
Armenia	Boys	1981	15	12.26
Armenia	Boys	1982	15	12.36
Armenia	Boys	1983	15	12.46

Armenia	Boys	1984	15	12.56
Armenia	Boys	1985	15	12.65
Armenia	Boys	1986	15	12.75
Armenia	Boys	1987	15	12.85
Armenia	Boys	1988	15	12.94
Armenia	Boys	1989	15	13.03
Armenia	Boys	1990	15	13.13
Armenia	Boys	1991	15	13.22
Armenia	Boys	1992	15	13.31
Armenia	Boys	1993	15	13.39
Armenia	Boys	1994	15	13.48
Armenia	Boys	1995	15	13.56
Armenia	Boys	1996	15	13.64
Armenia	Boys	1997	15	13.72
Armenia	Boys	1998	15	13.80
Armenia	Boys	1999	15	13.89
Armenia	Boys	2000	15	13.97
Armenia	Boys	2001	15	14.06
Armenia	Boys	2002	15	14.16
Armenia	Boys	2003	15	14.26
Armenia	Boys	2004	15	14.36
Armenia	Boys	2005	15	14.47
Armenia	Boys	2006	15	14.58
Armenia	Boys	2007	15	14.70
Armenia	Boys	2008	15	14.82
Armenia	Boys	2009	15	14.94
Armenia	Boys	2010	15	15.07
Armenia	Boys	2011	15	15.20
Armenia	Boys	2012	15	15.34
Armenia	Boys	2013	15	15.48
Armenia	Boys	2014	15	15.63
Armenia	Boys	2015	15	15.79
Armenia	Boys	2016	15	15.96
Armenia	Boys	1975	16	13.82
Armenia	Boys	1976	16	13.93
Armenia	Boys	1977	16	14.04
Armenia	Boys	1978	16	14.15
Armenia	Boys	1979	16	14.26
Armenia	Boys	1980	16	14.37
Armenia	Boys	1981	16	14.48
Armenia	Boys	1982	16	14.59
Armenia	Boys	1983	16	14.69
Armenia	Boys	1984	16	14.79
Armenia	Boys	1985	16	14.89
Armenia	Boys	1986	16	14.99
Armenia	Boys	1987	16	15.09
Armenia	Boys	1988	16	15.19
Armenia	Boys	1989	16	15.29
Armenia	Boys	1990	16	15.38
Armenia	Boys	1991	16	15.48
Armenia	Boys	1992	16	15.57
Armenia	Boys	1993	16	15.66

Armenia	Boys	1994	16	15.74
Armenia	Boys	1995	16	15.83
Armenia	Boys	1996	16	15.91
Armenia	Boys	1997	16	16.00
Armenia	Boys	1998	16	16.08
Armenia	Boys	1999	16	16.17
Armenia	Boys	2000	16	16.26
Armenia	Boys	2001	16	16.35
Armenia	Boys	2002	16	16.45
Armenia	Boys	2003	16	16.55
Armenia	Boys	2004	16	16.66
Armenia	Boys	2005	16	16.77
Armenia	Boys	2006	16	16.88
Armenia	Boys	2007	16	17.00
Armenia	Boys	2008	16	17.12
Armenia	Boys	2009	16	17.25
Armenia	Boys	2010	16	17.38
Armenia	Boys	2011	16	17.52
Armenia	Boys	2012	16	17.66
Armenia	Boys	2013	16	17.80
Armenia	Boys	2014	16	17.96
Armenia	Boys	2015	16	18.12
Armenia	Boys	2016	16	18.29
Armenia	Boys	1975	17	16.09
Armenia	Boys	1976	17	16.21
Armenia	Boys	1977	17	16.32
Armenia	Boys	1978	17	16.43
Armenia	Boys	1979	17	16.55
Armenia	Boys	1980	17	16.66
Armenia	Boys	1981	17	16.77
Armenia	Boys	1982	17	16.88
Armenia	Boys	1983	17	16.99
Armenia	Boys	1984	17	17.10
Armenia	Boys	1985	17	17.20
Armenia	Boys	1986	17	17.30
Armenia	Boys	1987	17	17.40
Armenia	Boys	1988	17	17.50
Armenia	Boys	1989	17	17.60
Armenia	Boys	1990	17	17.70
Armenia	Boys	1991	17	17.80
Armenia	Boys	1992	17	17.89
Armenia	Boys	1993	17	17.98
Armenia	Boys	1994	17	18.07
Armenia	Boys	1995	17	18.16
Armenia	Boys	1996	17	18.25
Armenia	Boys	1997	17	18.33
Armenia	Boys	1998	17	18.42
Armenia	Boys	1999	17	18.51
Armenia	Boys	2000	17	18.60
Armenia	Boys	2001	17	18.69
Armenia	Boys	2002	17	18.79
Armenia	Boys	2003	17	18.90

Armenia	Boys	2004	17	19.01
Armenia	Boys	2005	17	19.12
Armenia	Boys	2006	17	19.24
Armenia	Boys	2007	17	19.36
Armenia	Boys	2008	17	19.48
Armenia	Boys	2009	17	19.61
Armenia	Boys	2010	17	19.74
Armenia	Boys	2011	17	19.88
Armenia	Boys	2012	17	20.02
Armenia	Boys	2013	17	20.17
Armenia	Boys	2014	17	20.33
Armenia	Boys	2015	17	20.49
Armenia	Boys	2016	17	20.67
Armenia	Boys	1975	18	18.37
Armenia	Boys	1976	18	18.50
Armenia	Boys	1977	18	18.62
Armenia	Boys	1978	18	18.74
Armenia	Boys	1979	18	18.86
Armenia	Boys	1980	18	18.98
Armenia	Boys	1981	18	19.10
Armenia	Boys	1982	18	19.21
Armenia	Boys	1983	18	19.32
Armenia	Boys	1984	18	19.43
Armenia	Boys	1985	18	19.54
Armenia	Boys	1986	18	19.65
Armenia	Boys	1987	18	19.75
Armenia	Boys	1988	18	19.86
Armenia	Boys	1989	18	19.96
Armenia	Boys	1990	18	20.07
Armenia	Boys	1991	18	20.17
Armenia	Boys	1992	18	20.26
Armenia	Boys	1993	18	20.36
Armenia	Boys	1994	18	20.45
Armenia	Boys	1995	18	20.54
Armenia	Boys	1996	18	20.63
Armenia	Boys	1997	18	20.72
Armenia	Boys	1998	18	20.81
Armenia	Boys	1999	18	20.90
Armenia	Boys	2000	18	21.00
Armenia	Boys	2001	18	21.10
Armenia	Boys	2002	18	21.20
Armenia	Boys	2003	18	21.31
Armenia	Boys	2004	18	21.42
Armenia	Boys	2005	18	21.54
Armenia	Boys	2006	18	21.66
Armenia	Boys	2007	18	21.78
Armenia	Boys	2008	18	21.91
Armenia	Boys	2009	18	22.05
Armenia	Boys	2010	18	22.18
Armenia	Boys	2011	18	22.33
Armenia	Boys	2012	18	22.47
Armenia	Boys	2013	18	22.63

Armenia	Boys	2014	18	22.79
Armenia	Boys	2015	18	22.96
Armenia	Boys	2016	18	23.13
Armenia	Boys	1975	Average	11.94
Armenia	Boys	1976	Average	12.04
Armenia	Boys	1977	Average	12.14
Armenia	Boys	1978	Average	12.25
Armenia	Boys	1979	Average	12.35
Armenia	Boys	1980	Average	12.45
Armenia	Boys	1981	Average	12.55
Armenia	Boys	1982	Average	12.65
Armenia	Boys	1983	Average	12.74
Armenia	Boys	1984	Average	12.84
Armenia	Boys	1985	Average	12.93
Armenia	Boys	1986	Average	13.02
Armenia	Boys	1987	Average	13.11
Armenia	Boys	1988	Average	13.20
Armenia	Boys	1989	Average	13.29
Armenia	Boys	1990	Average	13.39
Armenia	Boys	1991	Average	13.47
Armenia	Boys	1992	Average	13.56
Armenia	Boys	1993	Average	13.64
Armenia	Boys	1994	Average	13.72
Armenia	Boys	1995	Average	13.80
Armenia	Boys	1996	Average	13.87
Armenia	Boys	1997	Average	13.95
Armenia	Boys	1998	Average	14.03
Armenia	Boys	1999	Average	14.11
Armenia	Boys	2000	Average	14.19
Armenia	Boys	2001	Average	14.28
Armenia	Boys	2002	Average	14.37
Armenia	Boys	2003	Average	14.47
Armenia	Boys	2004	Average	14.56
Armenia	Boys	2005	Average	14.67
Armenia	Boys	2006	Average	14.77
Armenia	Boys	2007	Average	14.89
Armenia	Boys	2008	Average	15.00
Armenia	Boys	2009	Average	15.12
Armenia	Boys	2010	Average	15.24
Armenia	Boys	2011	Average	15.37
Armenia	Boys	2012	Average	15.50
Armenia	Boys	2013	Average	15.63
Armenia	Boys	2014	Average	15.78
Armenia	Boys	2015	Average	15.93
Armenia	Boys	2016	Average	16.08
Armenia	Girls	1975	12	6.84
Armenia	Girls	1976	12	6.91
Armenia	Girls	1977	12	6.98
Armenia	Girls	1978	12	7.05
Armenia	Girls	1979	12	7.12
Armenia	Girls	1980	12	7.19
Armenia	Girls	1981	12	7.26

Armenia	Girls	1982	12	7.33
Armenia	Girls	1983	12	7.39
Armenia	Girls	1984	12	7.45
Armenia	Girls	1985	12	7.51
Armenia	Girls	1986	12	7.57
Armenia	Girls	1987	12	7.62
Armenia	Girls	1988	12	7.68
Armenia	Girls	1989	12	7.73
Armenia	Girls	1990	12	7.79
Armenia	Girls	1991	12	7.84
Armenia	Girls	1992	12	7.89
Armenia	Girls	1993	12	7.93
Armenia	Girls	1994	12	7.97
Armenia	Girls	1995	12	8.02
Armenia	Girls	1996	12	8.06
Armenia	Girls	1997	12	8.10
Armenia	Girls	1998	12	8.14
Armenia	Girls	1999	12	8.18
Armenia	Girls	2000	12	8.22
Armenia	Girls	2001	12	8.26
Armenia	Girls	2002	12	8.30
Armenia	Girls	2003	12	8.34
Armenia	Girls	2004	12	8.39
Armenia	Girls	2005	12	8.43
Armenia	Girls	2006	12	8.48
Armenia	Girls	2007	12	8.52
Armenia	Girls	2008	12	8.57
Armenia	Girls	2009	12	8.62
Armenia	Girls	2010	12	8.67
Armenia	Girls	2011	12	8.72
Armenia	Girls	2012	12	8.78
Armenia	Girls	2013	12	8.83
Armenia	Girls	2014	12	8.89
Armenia	Girls	2015	12	8.95
Armenia	Girls	2016	12	9.02
Armenia	Girls	1975	13	8.45
Armenia	Girls	1976	13	8.53
Armenia	Girls	1977	13	8.62
Armenia	Girls	1978	13	8.70
Armenia	Girls	1979	13	8.78
Armenia	Girls	1980	13	8.86
Armenia	Girls	1981	13	8.94
Armenia	Girls	1982	13	9.02
Armenia	Girls	1983	13	9.09
Armenia	Girls	1984	13	9.16
Armenia	Girls	1985	13	9.23
Armenia	Girls	1986	13	9.29
Armenia	Girls	1987	13	9.36
Armenia	Girls	1988	13	9.42
Armenia	Girls	1989	13	9.49
Armenia	Girls	1990	13	9.55
Armenia	Girls	1991	13	9.61

Armenia	Girls	1992	13	9.66
Armenia	Girls	1993	13	9.71
Armenia	Girls	1994	13	9.76
Armenia	Girls	1995	13	9.81
Armenia	Girls	1996	13	9.86
Armenia	Girls	1997	13	9.90
Armenia	Girls	1998	13	9.95
Armenia	Girls	1999	13	9.99
Armenia	Girls	2000	13	10.04
Armenia	Girls	2001	13	10.08
Armenia	Girls	2002	13	10.13
Armenia	Girls	2003	13	10.18
Armenia	Girls	2004	13	10.23
Armenia	Girls	2005	13	10.28
Armenia	Girls	2006	13	10.33
Armenia	Girls	2007	13	10.38
Armenia	Girls	2008	13	10.44
Armenia	Girls	2009	13	10.49
Armenia	Girls	2010	13	10.55
Armenia	Girls	2011	13	10.61
Armenia	Girls	2012	13	10.67
Armenia	Girls	2013	13	10.73
Armenia	Girls	2014	13	10.80
Armenia	Girls	2015	13	10.87
Armenia	Girls	2016	13	10.94
Armenia	Girls	1975	14	10.12
Armenia	Girls	1976	14	10.22
Armenia	Girls	1977	14	10.31
Armenia	Girls	1978	14	10.40
Armenia	Girls	1979	14	10.49
Armenia	Girls	1980	14	10.58
Armenia	Girls	1981	14	10.67
Armenia	Girls	1982	14	10.75
Armenia	Girls	1983	14	10.83
Armenia	Girls	1984	14	10.91
Armenia	Girls	1985	14	10.98
Armenia	Girls	1986	14	11.05
Armenia	Girls	1987	14	11.13
Armenia	Girls	1988	14	11.19
Armenia	Girls	1989	14	11.26
Armenia	Girls	1990	14	11.33
Armenia	Girls	1991	14	11.39
Armenia	Girls	1992	14	11.45
Armenia	Girls	1993	14	11.51
Armenia	Girls	1994	14	11.56
Armenia	Girls	1995	14	11.62
Armenia	Girls	1996	14	11.67
Armenia	Girls	1997	14	11.72
Armenia	Girls	1998	14	11.77
Armenia	Girls	1999	14	11.81
Armenia	Girls	2000	14	11.86
Armenia	Girls	2001	14	11.91

Armenia	Girls	2002	14	11.96
Armenia	Girls	2003	14	12.01
Armenia	Girls	2004	14	12.07
Armenia	Girls	2005	14	12.12
Armenia	Girls	2006	14	12.18
Armenia	Girls	2007	14	12.23
Armenia	Girls	2008	14	12.29
Armenia	Girls	2009	14	12.35
Armenia	Girls	2010	14	12.41
Armenia	Girls	2011	14	12.47
Armenia	Girls	2012	14	12.54
Armenia	Girls	2013	14	12.61
Armenia	Girls	2014	14	12.68
Armenia	Girls	2015	14	12.75
Armenia	Girls	2016	14	12.83
Armenia	Girls	1975	15	11.64
Armenia	Girls	1976	15	11.73
Armenia	Girls	1977	15	11.83
Armenia	Girls	1978	15	11.93
Armenia	Girls	1979	15	12.02
Armenia	Girls	1980	15	12.12
Armenia	Girls	1981	15	12.21
Armenia	Girls	1982	15	12.30
Armenia	Girls	1983	15	12.38
Armenia	Girls	1984	15	12.46
Armenia	Girls	1985	15	12.54
Armenia	Girls	1986	15	12.62
Armenia	Girls	1987	15	12.69
Armenia	Girls	1988	15	12.76
Armenia	Girls	1989	15	12.84
Armenia	Girls	1990	15	12.91
Armenia	Girls	1991	15	12.97
Armenia	Girls	1992	15	13.04
Armenia	Girls	1993	15	13.10
Armenia	Girls	1994	15	13.15
Armenia	Girls	1995	15	13.21
Armenia	Girls	1996	15	13.26
Armenia	Girls	1997	15	13.31
Armenia	Girls	1998	15	13.36
Armenia	Girls	1999	15	13.41
Armenia	Girls	2000	15	13.46
Armenia	Girls	2001	15	13.51
Armenia	Girls	2002	15	13.56
Armenia	Girls	2003	15	13.62
Armenia	Girls	2004	15	13.67
Armenia	Girls	2005	15	13.73
Armenia	Girls	2006	15	13.79
Armenia	Girls	2007	15	13.85
Armenia	Girls	2008	15	13.91
Armenia	Girls	2009	15	13.97
Armenia	Girls	2010	15	14.03
Armenia	Girls	2011	15	14.10

Armenia	Girls	2012	15	14.16
Armenia	Girls	2013	15	14.23
Armenia	Girls	2014	15	14.31
Armenia	Girls	2015	15	14.39
Armenia	Girls	2016	15	14.47
Armenia	Girls	1975	16	12.84
Armenia	Girls	1976	16	12.94
Armenia	Girls	1977	16	13.04
Armenia	Girls	1978	16	13.14
Armenia	Girls	1979	16	13.24
Armenia	Girls	1980	16	13.33
Armenia	Girls	1981	16	13.43
Armenia	Girls	1982	16	13.52
Armenia	Girls	1983	16	13.60
Armenia	Girls	1984	16	13.69
Armenia	Girls	1985	16	13.77
Armenia	Girls	1986	16	13.85
Armenia	Girls	1987	16	13.93
Armenia	Girls	1988	16	14.00
Armenia	Girls	1989	16	14.08
Armenia	Girls	1990	16	14.15
Armenia	Girls	1991	16	14.22
Armenia	Girls	1992	16	14.28
Armenia	Girls	1993	16	14.34
Armenia	Girls	1994	16	14.40
Armenia	Girls	1995	16	14.46
Armenia	Girls	1996	16	14.51
Armenia	Girls	1997	16	14.56
Armenia	Girls	1998	16	14.62
Armenia	Girls	1999	16	14.67
Armenia	Girls	2000	16	14.72
Armenia	Girls	2001	16	14.77
Armenia	Girls	2002	16	14.82
Armenia	Girls	2003	16	14.88
Armenia	Girls	2004	16	14.94
Armenia	Girls	2005	16	15.00
Armenia	Girls	2006	16	15.06
Armenia	Girls	2007	16	15.12
Armenia	Girls	2008	16	15.18
Armenia	Girls	2009	16	15.24
Armenia	Girls	2010	16	15.31
Armenia	Girls	2011	16	15.37
Armenia	Girls	2012	16	15.44
Armenia	Girls	2013	16	15.52
Armenia	Girls	2014	16	15.59
Armenia	Girls	2015	16	15.67
Armenia	Girls	2016	16	15.76
Armenia	Girls	1975	17	13.72
Armenia	Girls	1976	17	13.82
Armenia	Girls	1977	17	13.93
Armenia	Girls	1978	17	14.03
Armenia	Girls	1979	17	14.13

Armenia	Girls	1980	17	14.23
Armenia	Girls	1981	17	14.33
Armenia	Girls	1982	17	14.42
Armenia	Girls	1983	17	14.51
Armenia	Girls	1984	17	14.60
Armenia	Girls	1985	17	14.69
Armenia	Girls	1986	17	14.77
Armenia	Girls	1987	17	14.85
Armenia	Girls	1988	17	14.93
Armenia	Girls	1989	17	15.00
Armenia	Girls	1990	17	15.08
Armenia	Girls	1991	17	15.15
Armenia	Girls	1992	17	15.22
Armenia	Girls	1993	17	15.28
Armenia	Girls	1994	17	15.34
Armenia	Girls	1995	17	15.40
Armenia	Girls	1996	17	15.46
Armenia	Girls	1997	17	15.51
Armenia	Girls	1998	17	15.57
Armenia	Girls	1999	17	15.62
Armenia	Girls	2000	17	15.67
Armenia	Girls	2001	17	15.73
Armenia	Girls	2002	17	15.78
Armenia	Girls	2003	17	15.84
Armenia	Girls	2004	17	15.90
Armenia	Girls	2005	17	15.96
Armenia	Girls	2006	17	16.03
Armenia	Girls	2007	17	16.09
Armenia	Girls	2008	17	16.15
Armenia	Girls	2009	17	16.22
Armenia	Girls	2010	17	16.29
Armenia	Girls	2011	17	16.36
Armenia	Girls	2012	17	16.43
Armenia	Girls	2013	17	16.51
Armenia	Girls	2014	17	16.59
Armenia	Girls	2015	17	16.68
Armenia	Girls	2016	17	16.77
Armenia	Girls	1975	18	14.42
Armenia	Girls	1976	18	14.53
Armenia	Girls	1977	18	14.64
Armenia	Girls	1978	18	14.75
Armenia	Girls	1979	18	14.86
Armenia	Girls	1980	18	14.97
Armenia	Girls	1981	18	15.07
Armenia	Girls	1982	18	15.17
Armenia	Girls	1983	18	15.27
Armenia	Girls	1984	18	15.36
Armenia	Girls	1985	18	15.45
Armenia	Girls	1986	18	15.54
Armenia	Girls	1987	18	15.62
Armenia	Girls	1988	18	15.71
Armenia	Girls	1989	18	15.79

Armenia	Girls	1990	18	15.87
Armenia	Girls	1991	18	15.95
Armenia	Girls	1992	18	16.02
Armenia	Girls	1993	18	16.09
Armenia	Girls	1994	18	16.16
Armenia	Girls	1995	18	16.22
Armenia	Girls	1996	18	16.28
Armenia	Girls	1997	18	16.34
Armenia	Girls	1998	18	16.40
Armenia	Girls	1999	18	16.46
Armenia	Girls	2000	18	16.52
Armenia	Girls	2001	18	16.58
Armenia	Girls	2002	18	16.64
Armenia	Girls	2003	18	16.70
Armenia	Girls	2004	18	16.77
Armenia	Girls	2005	18	16.83
Armenia	Girls	2006	18	16.90
Armenia	Girls	2007	18	16.97
Armenia	Girls	2008	18	17.04
Armenia	Girls	2009	18	17.12
Armenia	Girls	2010	18	17.19
Armenia	Girls	2011	18	17.27
Armenia	Girls	2012	18	17.35
Armenia	Girls	2013	18	17.44
Armenia	Girls	2014	18	17.52
Armenia	Girls	2015	18	17.62
Armenia	Girls	2016	18	17.72
Armenia	Girls	1975	Average	11.15
Armenia	Girls	1976	Average	11.24
Armenia	Girls	1977	Average	11.34
Armenia	Girls	1978	Average	11.43
Armenia	Girls	1979	Average	11.52
Armenia	Girls	1980	Average	11.61
Armenia	Girls	1981	Average	11.70
Armenia	Girls	1982	Average	11.79
Armenia	Girls	1983	Average	11.87
Armenia	Girls	1984	Average	11.95
Armenia	Girls	1985	Average	12.02
Armenia	Girls	1986	Average	12.10
Armenia	Girls	1987	Average	12.17
Armenia	Girls	1988	Average	12.24
Armenia	Girls	1989	Average	12.31
Armenia	Girls	1990	Average	12.38
Armenia	Girls	1991	Average	12.45
Armenia	Girls	1992	Average	12.51
Armenia	Girls	1993	Average	12.57
Armenia	Girls	1994	Average	12.62
Armenia	Girls	1995	Average	12.68
Armenia	Girls	1996	Average	12.73
Armenia	Girls	1997	Average	12.78
Armenia	Girls	1998	Average	12.83
Armenia	Girls	1999	Average	12.88

Armenia	Girls	2000	Average	12.93
Armenia	Girls	2001	Average	12.98
Armenia	Girls	2002	Average	13.03
Armenia	Girls	2003	Average	13.08
Armenia	Girls	2004	Average	13.14
Armenia	Girls	2005	Average	13.19
Armenia	Girls	2006	Average	13.25
Armenia	Girls	2007	Average	13.31
Armenia	Girls	2008	Average	13.37
Armenia	Girls	2009	Average	13.43
Armenia	Girls	2010	Average	13.49
Armenia	Girls	2011	Average	13.56
Armenia	Girls	2012	Average	13.62
Armenia	Girls	2013	Average	13.70
Armenia	Girls	2014	Average	13.77
Armenia	Girls	2015	Average	13.85
Armenia	Girls	2016	Average	13.93
Australia	Boys	1975	12	9.55
Australia	Boys	1976	12	9.64
Australia	Boys	1977	12	9.72
Australia	Boys	1978	12	9.80
Australia	Boys	1979	12	9.89
Australia	Boys	1980	12	9.98
Australia	Boys	1981	12	10.06
Australia	Boys	1982	12	10.15
Australia	Boys	1983	12	10.25
Australia	Boys	1984	12	10.34
Australia	Boys	1985	12	10.44
Australia	Boys	1986	12	10.55
Australia	Boys	1987	12	10.66
Australia	Boys	1988	12	10.77
Australia	Boys	1989	12	10.89
Australia	Boys	1990	12	11.01
Australia	Boys	1991	12	11.14
Australia	Boys	1992	12	11.27
Australia	Boys	1993	12	11.41
Australia	Boys	1994	12	11.55
Australia	Boys	1995	12	11.69
Australia	Boys	1996	12	11.83
Australia	Boys	1997	12	11.97
Australia	Boys	1998	12	12.12
Australia	Boys	1999	12	12.26
Australia	Boys	2000	12	12.40
Australia	Boys	2001	12	12.54
Australia	Boys	2002	12	12.67
Australia	Boys	2003	12	12.79
Australia	Boys	2004	12	12.91
Australia	Boys	2005	12	13.02
Australia	Boys	2006	12	13.12
Australia	Boys	2007	12	13.21
Australia	Boys	2008	12	13.30
Australia	Boys	2009	12	13.38

Australia	Boys	2010	12	13.46
Australia	Boys	2011	12	13.53
Australia	Boys	2012	12	13.60
Australia	Boys	2013	12	13.66
Australia	Boys	2014	12	13.73
Australia	Boys	2015	12	13.80
Australia	Boys	2016	12	13.87
Australia	Boys	1975	13	11.78
Australia	Boys	1976	13	11.88
Australia	Boys	1977	13	11.97
Australia	Boys	1978	13	12.07
Australia	Boys	1979	13	12.17
Australia	Boys	1980	13	12.28
Australia	Boys	1981	13	12.38
Australia	Boys	1982	13	12.48
Australia	Boys	1983	13	12.59
Australia	Boys	1984	13	12.71
Australia	Boys	1985	13	12.82
Australia	Boys	1986	13	12.95
Australia	Boys	1987	13	13.08
Australia	Boys	1988	13	13.21
Australia	Boys	1989	13	13.35
Australia	Boys	1990	13	13.50
Australia	Boys	1991	13	13.65
Australia	Boys	1992	13	13.80
Australia	Boys	1993	13	13.97
Australia	Boys	1994	13	14.13
Australia	Boys	1995	13	14.30
Australia	Boys	1996	13	14.47
Australia	Boys	1997	13	14.64
Australia	Boys	1998	13	14.82
Australia	Boys	1999	13	14.99
Australia	Boys	2000	13	15.16
Australia	Boys	2001	13	15.32
Australia	Boys	2002	13	15.48
Australia	Boys	2003	13	15.63
Australia	Boys	2004	13	15.77
Australia	Boys	2005	13	15.90
Australia	Boys	2006	13	16.03
Australia	Boys	2007	13	16.14
Australia	Boys	2008	13	16.25
Australia	Boys	2009	13	16.35
Australia	Boys	2010	13	16.44
Australia	Boys	2011	13	16.53
Australia	Boys	2012	13	16.61
Australia	Boys	2013	13	16.69
Australia	Boys	2014	13	16.77
Australia	Boys	2015	13	16.86
Australia	Boys	2016	13	16.94
Australia	Boys	1975	14	14.25
Australia	Boys	1976	14	14.36
Australia	Boys	1977	14	14.47

Australia	Boys	1978	14	14.58
Australia	Boys	1979	14	14.69
Australia	Boys	1980	14	14.80
Australia	Boys	1981	14	14.92
Australia	Boys	1982	14	15.03
Australia	Boys	1983	14	15.15
Australia	Boys	1984	14	15.28
Australia	Boys	1985	14	15.41
Australia	Boys	1986	14	15.54
Australia	Boys	1987	14	15.69
Australia	Boys	1988	14	15.84
Australia	Boys	1989	14	15.99
Australia	Boys	1990	14	16.15
Australia	Boys	1991	14	16.32
Australia	Boys	1992	14	16.50
Australia	Boys	1993	14	16.68
Australia	Boys	1994	14	16.86
Australia	Boys	1995	14	17.05
Australia	Boys	1996	14	17.25
Australia	Boys	1997	14	17.44
Australia	Boys	1998	14	17.64
Australia	Boys	1999	14	17.83
Australia	Boys	2000	14	18.03
Australia	Boys	2001	14	18.21
Australia	Boys	2002	14	18.39
Australia	Boys	2003	14	18.56
Australia	Boys	2004	14	18.72
Australia	Boys	2005	14	18.87
Australia	Boys	2006	14	19.01
Australia	Boys	2007	14	19.14
Australia	Boys	2008	14	19.26
Australia	Boys	2009	14	19.37
Australia	Boys	2010	14	19.48
Australia	Boys	2011	14	19.57
Australia	Boys	2012	14	19.67
Australia	Boys	2013	14	19.76
Australia	Boys	2014	14	19.86
Australia	Boys	2015	14	19.95
Australia	Boys	2016	14	20.05
Australia	Boys	1975	15	16.81
Australia	Boys	1976	15	16.93
Australia	Boys	1977	15	17.04
Australia	Boys	1978	15	17.15
Australia	Boys	1979	15	17.27
Australia	Boys	1980	15	17.39
Australia	Boys	1981	15	17.51
Australia	Boys	1982	15	17.63
Australia	Boys	1983	15	17.76
Australia	Boys	1984	15	17.89
Australia	Boys	1985	15	18.02
Australia	Boys	1986	15	18.16
Australia	Boys	1987	15	18.31

Australia	Boys	1988	15	18.47
Australia	Boys	1989	15	18.63
Australia	Boys	1990	15	18.80
Australia	Boys	1991	15	18.98
Australia	Boys	1992	15	19.17
Australia	Boys	1993	15	19.36
Australia	Boys	1994	15	19.56
Australia	Boys	1995	15	19.76
Australia	Boys	1996	15	19.96
Australia	Boys	1997	15	20.17
Australia	Boys	1998	15	20.38
Australia	Boys	1999	15	20.59
Australia	Boys	2000	15	20.79
Australia	Boys	2001	15	20.99
Australia	Boys	2002	15	21.18
Australia	Boys	2003	15	21.37
Australia	Boys	2004	15	21.54
Australia	Boys	2005	15	21.70
Australia	Boys	2006	15	21.85
Australia	Boys	2007	15	21.99
Australia	Boys	2008	15	22.12
Australia	Boys	2009	15	22.23
Australia	Boys	2010	15	22.34
Australia	Boys	2011	15	22.45
Australia	Boys	2012	15	22.55
Australia	Boys	2013	15	22.65
Australia	Boys	2014	15	22.75
Australia	Boys	2015	15	22.85
Australia	Boys	2016	15	22.96
Australia	Boys	1975	16	19.39
Australia	Boys	1976	16	19.50
Australia	Boys	1977	16	19.62
Australia	Boys	1978	16	19.74
Australia	Boys	1979	16	19.85
Australia	Boys	1980	16	19.97
Australia	Boys	1981	16	20.09
Australia	Boys	1982	16	20.22
Australia	Boys	1983	16	20.35
Australia	Boys	1984	16	20.48
Australia	Boys	1985	16	20.62
Australia	Boys	1986	16	20.76
Australia	Boys	1987	16	20.92
Australia	Boys	1988	16	21.08
Australia	Boys	1989	16	21.24
Australia	Boys	1990	16	21.41
Australia	Boys	1991	16	21.60
Australia	Boys	1992	16	21.79
Australia	Boys	1993	16	21.98
Australia	Boys	1994	16	22.19
Australia	Boys	1995	16	22.39
Australia	Boys	1996	16	22.60
Australia	Boys	1997	16	22.82

Australia	Boys	1998	16	23.03
Australia	Boys	1999	16	23.25
Australia	Boys	2000	16	23.46
Australia	Boys	2001	16	23.66
Australia	Boys	2002	16	23.86
Australia	Boys	2003	16	24.05
Australia	Boys	2004	16	24.23
Australia	Boys	2005	16	24.39
Australia	Boys	2006	16	24.55
Australia	Boys	2007	16	24.69
Australia	Boys	2008	16	24.82
Australia	Boys	2009	16	24.94
Australia	Boys	2010	16	25.06
Australia	Boys	2011	16	25.17
Australia	Boys	2012	16	25.27
Australia	Boys	2013	16	25.37
Australia	Boys	2014	16	25.48
Australia	Boys	2015	16	25.59
Australia	Boys	2016	16	25.70
Australia	Boys	1975	17	21.97
Australia	Boys	1976	17	22.08
Australia	Boys	1977	17	22.20
Australia	Boys	1978	17	22.32
Australia	Boys	1979	17	22.44
Australia	Boys	1980	17	22.56
Australia	Boys	1981	17	22.68
Australia	Boys	1982	17	22.81
Australia	Boys	1983	17	22.94
Australia	Boys	1984	17	23.07
Australia	Boys	1985	17	23.21
Australia	Boys	1986	17	23.36
Australia	Boys	1987	17	23.52
Australia	Boys	1988	17	23.68
Australia	Boys	1989	17	23.85
Australia	Boys	1990	17	24.02
Australia	Boys	1991	17	24.21
Australia	Boys	1992	17	24.40
Australia	Boys	1993	17	24.60
Australia	Boys	1994	17	24.81
Australia	Boys	1995	17	25.02
Australia	Boys	1996	17	25.23
Australia	Boys	1997	17	25.45
Australia	Boys	1998	17	25.67
Australia	Boys	1999	17	25.89
Australia	Boys	2000	17	26.11
Australia	Boys	2001	17	26.32
Australia	Boys	2002	17	26.52
Australia	Boys	2003	17	26.71
Australia	Boys	2004	17	26.89
Australia	Boys	2005	17	27.06
Australia	Boys	2006	17	27.22
Australia	Boys	2007	17	27.36

Australia	Boys	2008	17	27.50
Australia	Boys	2009	17	27.62
Australia	Boys	2010	17	27.74
Australia	Boys	2011	17	27.85
Australia	Boys	2012	17	27.96
Australia	Boys	2013	17	28.06
Australia	Boys	2014	17	28.17
Australia	Boys	2015	17	28.28
Australia	Boys	2016	17	28.39
Australia	Boys	1975	18	24.61
Australia	Boys	1976	18	24.73
Australia	Boys	1977	18	24.86
Australia	Boys	1978	18	24.98
Australia	Boys	1979	18	25.10
Australia	Boys	1980	18	25.23
Australia	Boys	1981	18	25.36
Australia	Boys	1982	18	25.49
Australia	Boys	1983	18	25.62
Australia	Boys	1984	18	25.76
Australia	Boys	1985	18	25.91
Australia	Boys	1986	18	26.06
Australia	Boys	1987	18	26.22
Australia	Boys	1988	18	26.39
Australia	Boys	1989	18	26.56
Australia	Boys	1990	18	26.75
Australia	Boys	1991	18	26.94
Australia	Boys	1992	18	27.14
Australia	Boys	1993	18	27.34
Australia	Boys	1994	18	27.56
Australia	Boys	1995	18	27.77
Australia	Boys	1996	18	28.00
Australia	Boys	1997	18	28.22
Australia	Boys	1998	18	28.45
Australia	Boys	1999	18	28.68
Australia	Boys	2000	18	28.90
Australia	Boys	2001	18	29.12
Australia	Boys	2002	18	29.33
Australia	Boys	2003	18	29.52
Australia	Boys	2004	18	29.71
Australia	Boys	2005	18	29.89
Australia	Boys	2006	18	30.05
Australia	Boys	2007	18	30.20
Australia	Boys	2008	18	30.34
Australia	Boys	2009	18	30.47
Australia	Boys	2010	18	30.59
Australia	Boys	2011	18	30.70
Australia	Boys	2012	18	30.81
Australia	Boys	2013	18	30.92
Australia	Boys	2014	18	31.03
Australia	Boys	2015	18	31.14
Australia	Boys	2016	18	31.26
Australia	Boys	1975	Average	16.91

Australia	Boys	1976	Average	17.02
Australia	Boys	1977	Average	17.13
Australia	Boys	1978	Average	17.24
Australia	Boys	1979	Average	17.35
Australia	Boys	1980	Average	17.46
Australia	Boys	1981	Average	17.57
Australia	Boys	1982	Average	17.69
Australia	Boys	1983	Average	17.81
Australia	Boys	1984	Average	17.93
Australia	Boys	1985	Average	18.06
Australia	Boys	1986	Average	18.20
Australia	Boys	1987	Average	18.34
Australia	Boys	1988	Average	18.49
Australia	Boys	1989	Average	18.65
Australia	Boys	1990	Average	18.81
Australia	Boys	1991	Average	18.98
Australia	Boys	1992	Average	19.15
Australia	Boys	1993	Average	19.33
Australia	Boys	1994	Average	19.52
Australia	Boys	1995	Average	19.71
Australia	Boys	1996	Average	19.90
Australia	Boys	1997	Average	20.10
Australia	Boys	1998	Average	20.30
Australia	Boys	1999	Average	20.50
Australia	Boys	2000	Average	20.69
Australia	Boys	2001	Average	20.88
Australia	Boys	2002	Average	21.06
Australia	Boys	2003	Average	21.23
Australia	Boys	2004	Average	21.39
Australia	Boys	2005	Average	21.55
Australia	Boys	2006	Average	21.69
Australia	Boys	2007	Average	21.82
Australia	Boys	2008	Average	21.94
Australia	Boys	2009	Average	22.05
Australia	Boys	2010	Average	22.16
Australia	Boys	2011	Average	22.26
Australia	Boys	2012	Average	22.35
Australia	Boys	2013	Average	22.45
Australia	Boys	2014	Average	22.54
Australia	Boys	2015	Average	22.64
Australia	Boys	2016	Average	22.74
Australia	Girls	1975	12	9.21
Australia	Girls	1976	12	9.28
Australia	Girls	1977	12	9.36
Australia	Girls	1978	12	9.43
Australia	Girls	1979	12	9.50
Australia	Girls	1980	12	9.58
Australia	Girls	1981	12	9.65
Australia	Girls	1982	12	9.73
Australia	Girls	1983	12	9.80
Australia	Girls	1984	12	9.88
Australia	Girls	1985	12	9.96

Australia	Girls	1986	12	10.04
Australia	Girls	1987	12	10.13
Australia	Girls	1988	12	10.22
Australia	Girls	1989	12	10.30
Australia	Girls	1990	12	10.40
Australia	Girls	1991	12	10.49
Australia	Girls	1992	12	10.59
Australia	Girls	1993	12	10.69
Australia	Girls	1994	12	10.79
Australia	Girls	1995	12	10.89
Australia	Girls	1996	12	10.99
Australia	Girls	1997	12	11.09
Australia	Girls	1998	12	11.19
Australia	Girls	1999	12	11.29
Australia	Girls	2000	12	11.38
Australia	Girls	2001	12	11.47
Australia	Girls	2002	12	11.56
Australia	Girls	2003	12	11.63
Australia	Girls	2004	12	11.70
Australia	Girls	2005	12	11.75
Australia	Girls	2006	12	11.81
Australia	Girls	2007	12	11.85
Australia	Girls	2008	12	11.89
Australia	Girls	2009	12	11.93
Australia	Girls	2010	12	11.96
Australia	Girls	2011	12	11.99
Australia	Girls	2012	12	12.02
Australia	Girls	2013	12	12.05
Australia	Girls	2014	12	12.08
Australia	Girls	2015	12	12.11
Australia	Girls	2016	12	12.14
Australia	Girls	1975	13	11.19
Australia	Girls	1976	13	11.28
Australia	Girls	1977	13	11.36
Australia	Girls	1978	13	11.44
Australia	Girls	1979	13	11.52
Australia	Girls	1980	13	11.61
Australia	Girls	1981	13	11.69
Australia	Girls	1982	13	11.78
Australia	Girls	1983	13	11.86
Australia	Girls	1984	13	11.95
Australia	Girls	1985	13	12.04
Australia	Girls	1986	13	12.13
Australia	Girls	1987	13	12.23
Australia	Girls	1988	13	12.33
Australia	Girls	1989	13	12.43
Australia	Girls	1990	13	12.53
Australia	Girls	1991	13	12.64
Australia	Girls	1992	13	12.75
Australia	Girls	1993	13	12.86
Australia	Girls	1994	13	12.98
Australia	Girls	1995	13	13.09

Australia	Girls	1996	13	13.21
Australia	Girls	1997	13	13.32
Australia	Girls	1998	13	13.44
Australia	Girls	1999	13	13.55
Australia	Girls	2000	13	13.66
Australia	Girls	2001	13	13.76
Australia	Girls	2002	13	13.85
Australia	Girls	2003	13	13.93
Australia	Girls	2004	13	14.01
Australia	Girls	2005	13	14.08
Australia	Girls	2006	13	14.13
Australia	Girls	2007	13	14.18
Australia	Girls	2008	13	14.23
Australia	Girls	2009	13	14.27
Australia	Girls	2010	13	14.31
Australia	Girls	2011	13	14.34
Australia	Girls	2012	13	14.37
Australia	Girls	2013	13	14.41
Australia	Girls	2014	13	14.44
Australia	Girls	2015	13	14.48
Australia	Girls	2016	13	14.51
Australia	Girls	1975	14	13.13
Australia	Girls	1976	14	13.22
Australia	Girls	1977	14	13.31
Australia	Girls	1978	14	13.40
Australia	Girls	1979	14	13.49
Australia	Girls	1980	14	13.58
Australia	Girls	1981	14	13.67
Australia	Girls	1982	14	13.76
Australia	Girls	1983	14	13.85
Australia	Girls	1984	14	13.94
Australia	Girls	1985	14	14.04
Australia	Girls	1986	14	14.14
Australia	Girls	1987	14	14.24
Australia	Girls	1988	14	14.35
Australia	Girls	1989	14	14.46
Australia	Girls	1990	14	14.57
Australia	Girls	1991	14	14.68
Australia	Girls	1992	14	14.80
Australia	Girls	1993	14	14.92
Australia	Girls	1994	14	15.05
Australia	Girls	1995	14	15.17
Australia	Girls	1996	14	15.30
Australia	Girls	1997	14	15.42
Australia	Girls	1998	14	15.54
Australia	Girls	1999	14	15.66
Australia	Girls	2000	14	15.78
Australia	Girls	2001	14	15.89
Australia	Girls	2002	14	15.99
Australia	Girls	2003	14	16.08
Australia	Girls	2004	14	16.16
Australia	Girls	2005	14	16.23

Australia	Girls	2006	14	16.29
Australia	Girls	2007	14	16.34
Australia	Girls	2008	14	16.39
Australia	Girls	2009	14	16.43
Australia	Girls	2010	14	16.47
Australia	Girls	2011	14	16.51
Australia	Girls	2012	14	16.54
Australia	Girls	2013	14	16.58
Australia	Girls	2014	14	16.61
Australia	Girls	2015	14	16.65
Australia	Girls	2016	14	16.69
Australia	Girls	1975	15	14.80
Australia	Girls	1976	15	14.90
Australia	Girls	1977	15	14.99
Australia	Girls	1978	15	15.08
Australia	Girls	1979	15	15.18
Australia	Girls	1980	15	15.27
Australia	Girls	1981	15	15.36
Australia	Girls	1982	15	15.45
Australia	Girls	1983	15	15.55
Australia	Girls	1984	15	15.65
Australia	Girls	1985	15	15.75
Australia	Girls	1986	15	15.85
Australia	Girls	1987	15	15.96
Australia	Girls	1988	15	16.07
Australia	Girls	1989	15	16.18
Australia	Girls	1990	15	16.30
Australia	Girls	1991	15	16.42
Australia	Girls	1992	15	16.54
Australia	Girls	1993	15	16.66
Australia	Girls	1994	15	16.79
Australia	Girls	1995	15	16.92
Australia	Girls	1996	15	17.05
Australia	Girls	1997	15	17.18
Australia	Girls	1998	15	17.31
Australia	Girls	1999	15	17.43
Australia	Girls	2000	15	17.55
Australia	Girls	2001	15	17.66
Australia	Girls	2002	15	17.77
Australia	Girls	2003	15	17.86
Australia	Girls	2004	15	17.94
Australia	Girls	2005	15	18.02
Australia	Girls	2006	15	18.08
Australia	Girls	2007	15	18.13
Australia	Girls	2008	15	18.18
Australia	Girls	2009	15	18.22
Australia	Girls	2010	15	18.26
Australia	Girls	2011	15	18.30
Australia	Girls	2012	15	18.34
Australia	Girls	2013	15	18.37
Australia	Girls	2014	15	18.41
Australia	Girls	2015	15	18.45

Australia	Girls	2016	15	18.49
Australia	Girls	1975	16	16.12
Australia	Girls	1976	16	16.22
Australia	Girls	1977	16	16.31
Australia	Girls	1978	16	16.41
Australia	Girls	1979	16	16.50
Australia	Girls	1980	16	16.60
Australia	Girls	1981	16	16.69
Australia	Girls	1982	16	16.79
Australia	Girls	1983	16	16.89
Australia	Girls	1984	16	16.99
Australia	Girls	1985	16	17.09
Australia	Girls	1986	16	17.20
Australia	Girls	1987	16	17.31
Australia	Girls	1988	16	17.42
Australia	Girls	1989	16	17.54
Australia	Girls	1990	16	17.66
Australia	Girls	1991	16	17.78
Australia	Girls	1992	16	17.91
Australia	Girls	1993	16	18.04
Australia	Girls	1994	16	18.17
Australia	Girls	1995	16	18.30
Australia	Girls	1996	16	18.44
Australia	Girls	1997	16	18.57
Australia	Girls	1998	16	18.70
Australia	Girls	1999	16	18.83
Australia	Girls	2000	16	18.95
Australia	Girls	2001	16	19.07
Australia	Girls	2002	16	19.18
Australia	Girls	2003	16	19.27
Australia	Girls	2004	16	19.36
Australia	Girls	2005	16	19.43
Australia	Girls	2006	16	19.50
Australia	Girls	2007	16	19.55
Australia	Girls	2008	16	19.60
Australia	Girls	2009	16	19.65
Australia	Girls	2010	16	19.69
Australia	Girls	2011	16	19.72
Australia	Girls	2012	16	19.76
Australia	Girls	2013	16	19.80
Australia	Girls	2014	16	19.83
Australia	Girls	2015	16	19.87
Australia	Girls	2016	16	19.91
Australia	Girls	1975	17	17.17
Australia	Girls	1976	17	17.27
Australia	Girls	1977	17	17.37
Australia	Girls	1978	17	17.47
Australia	Girls	1979	17	17.57
Australia	Girls	1980	17	17.67
Australia	Girls	1981	17	17.77
Australia	Girls	1982	17	17.87
Australia	Girls	1983	17	17.97

Australia	Girls	1984	17	18.08
Australia	Girls	1985	17	18.19
Australia	Girls	1986	17	18.30
Australia	Girls	1987	17	18.42
Australia	Girls	1988	17	18.54
Australia	Girls	1989	17	18.66
Australia	Girls	1990	17	18.78
Australia	Girls	1991	17	18.91
Australia	Girls	1992	17	19.05
Australia	Girls	1993	17	19.19
Australia	Girls	1994	17	19.33
Australia	Girls	1995	17	19.46
Australia	Girls	1996	17	19.61
Australia	Girls	1997	17	19.75
Australia	Girls	1998	17	19.88
Australia	Girls	1999	17	20.02
Australia	Girls	2000	17	20.15
Australia	Girls	2001	17	20.27
Australia	Girls	2002	17	20.39
Australia	Girls	2003	17	20.49
Australia	Girls	2004	17	20.58
Australia	Girls	2005	17	20.66
Australia	Girls	2006	17	20.72
Australia	Girls	2007	17	20.78
Australia	Girls	2008	17	20.84
Australia	Girls	2009	17	20.88
Australia	Girls	2010	17	20.93
Australia	Girls	2011	17	20.97
Australia	Girls	2012	17	21.00
Australia	Girls	2013	17	21.04
Australia	Girls	2014	17	21.08
Australia	Girls	2015	17	21.12
Australia	Girls	2016	17	21.16
Australia	Girls	1975	18	18.18
Australia	Girls	1976	18	18.29
Australia	Girls	1977	18	18.40
Australia	Girls	1978	18	18.51
Australia	Girls	1979	18	18.62
Australia	Girls	1980	18	18.73
Australia	Girls	1981	18	18.84
Australia	Girls	1982	18	18.95
Australia	Girls	1983	18	19.07
Australia	Girls	1984	18	19.18
Australia	Girls	1985	18	19.30
Australia	Girls	1986	18	19.43
Australia	Girls	1987	18	19.55
Australia	Girls	1988	18	19.69
Australia	Girls	1989	18	19.82
Australia	Girls	1990	18	19.96
Australia	Girls	1991	18	20.10
Australia	Girls	1992	18	20.25
Australia	Girls	1993	18	20.40

Australia	Girls	1994	18	20.55
Australia	Girls	1995	18	20.71
Australia	Girls	1996	18	20.86
Australia	Girls	1997	18	21.01
Australia	Girls	1998	18	21.17
Australia	Girls	1999	18	21.31
Australia	Girls	2000	18	21.46
Australia	Girls	2001	18	21.59
Australia	Girls	2002	18	21.72
Australia	Girls	2003	18	21.83
Australia	Girls	2004	18	21.93
Australia	Girls	2005	18	22.01
Australia	Girls	2006	18	22.09
Australia	Girls	2007	18	22.15
Australia	Girls	2008	18	22.21
Australia	Girls	2009	18	22.26
Australia	Girls	2010	18	22.31
Australia	Girls	2011	18	22.36
Australia	Girls	2012	18	22.40
Australia	Girls	2013	18	22.44
Australia	Girls	2014	18	22.48
Australia	Girls	2015	18	22.53
Australia	Girls	2016	18	22.57
Australia	Girls	1975	Average	14.26
Australia	Girls	1976	Average	14.35
Australia	Girls	1977	Average	14.44
Australia	Girls	1978	Average	14.54
Australia	Girls	1979	Average	14.63
Australia	Girls	1980	Average	14.72
Australia	Girls	1981	Average	14.81
Australia	Girls	1982	Average	14.90
Australia	Girls	1983	Average	15.00
Australia	Girls	1984	Average	15.10
Australia	Girls	1985	Average	15.20
Australia	Girls	1986	Average	15.30
Australia	Girls	1987	Average	15.41
Australia	Girls	1988	Average	15.51
Australia	Girls	1989	Average	15.63
Australia	Girls	1990	Average	15.74
Australia	Girls	1991	Average	15.86
Australia	Girls	1992	Average	15.98
Australia	Girls	1993	Average	16.11
Australia	Girls	1994	Average	16.24
Australia	Girls	1995	Average	16.36
Australia	Girls	1996	Average	16.49
Australia	Girls	1997	Average	16.62
Australia	Girls	1998	Average	16.75
Australia	Girls	1999	Average	16.87
Australia	Girls	2000	Average	16.99
Australia	Girls	2001	Average	17.10
Australia	Girls	2002	Average	17.20
Australia	Girls	2003	Average	17.30

Australia	Girls	2004	Average	17.38
Australia	Girls	2005	Average	17.45
Australia	Girls	2006	Average	17.52
Australia	Girls	2007	Average	17.57
Australia	Girls	2008	Average	17.62
Australia	Girls	2009	Average	17.66
Australia	Girls	2010	Average	17.70
Australia	Girls	2011	Average	17.74
Australia	Girls	2012	Average	17.78
Australia	Girls	2013	Average	17.81
Australia	Girls	2014	Average	17.85
Australia	Girls	2015	Average	17.89
Australia	Girls	2016	Average	17.93
Austria	Boys	1975	12	7.90
Austria	Boys	1976	12	8.00
Austria	Boys	1977	12	8.10
Austria	Boys	1978	12	8.21
Austria	Boys	1979	12	8.32
Austria	Boys	1980	12	8.44
Austria	Boys	1981	12	8.56
Austria	Boys	1982	12	8.69
Austria	Boys	1983	12	8.81
Austria	Boys	1984	12	8.94
Austria	Boys	1985	12	9.08
Austria	Boys	1986	12	9.21
Austria	Boys	1987	12	9.35
Austria	Boys	1988	12	9.49
Austria	Boys	1989	12	9.63
Austria	Boys	1990	12	9.76
Austria	Boys	1991	12	9.90
Austria	Boys	1992	12	10.03
Austria	Boys	1993	12	10.16
Austria	Boys	1994	12	10.29
Austria	Boys	1995	12	10.41
Austria	Boys	1996	12	10.53
Austria	Boys	1997	12	10.65
Austria	Boys	1998	12	10.76
Austria	Boys	1999	12	10.87
Austria	Boys	2000	12	10.98
Austria	Boys	2001	12	11.08
Austria	Boys	2002	12	11.18
Austria	Boys	2003	12	11.28
Austria	Boys	2004	12	11.38
Austria	Boys	2005	12	11.47
Austria	Boys	2006	12	11.56
Austria	Boys	2007	12	11.65
Austria	Boys	2008	12	11.73
Austria	Boys	2009	12	11.82
Austria	Boys	2010	12	11.90
Austria	Boys	2011	12	11.98
Austria	Boys	2012	12	12.06
Austria	Boys	2013	12	12.14

Austria	Boys	2014	12	12.21
Austria	Boys	2015	12	12.29
Austria	Boys	2016	12	12.37
Austria	Boys	1975	13	9.73
Austria	Boys	1976	13	9.84
Austria	Boys	1977	13	9.97
Austria	Boys	1978	13	10.10
Austria	Boys	1979	13	10.23
Austria	Boys	1980	13	10.37
Austria	Boys	1981	13	10.51
Austria	Boys	1982	13	10.66
Austria	Boys	1983	13	10.81
Austria	Boys	1984	13	10.97
Austria	Boys	1985	13	11.13
Austria	Boys	1986	13	11.29
Austria	Boys	1987	13	11.46
Austria	Boys	1988	13	11.63
Austria	Boys	1989	13	11.79
Austria	Boys	1990	13	11.96
Austria	Boys	1991	13	12.12
Austria	Boys	1992	13	12.28
Austria	Boys	1993	13	12.44
Austria	Boys	1994	13	12.59
Austria	Boys	1995	13	12.74
Austria	Boys	1996	13	12.89
Austria	Boys	1997	13	13.03
Austria	Boys	1998	13	13.17
Austria	Boys	1999	13	13.30
Austria	Boys	2000	13	13.43
Austria	Boys	2001	13	13.56
Austria	Boys	2002	13	13.68
Austria	Boys	2003	13	13.80
Austria	Boys	2004	13	13.91
Austria	Boys	2005	13	14.03
Austria	Boys	2006	13	14.14
Austria	Boys	2007	13	14.24
Austria	Boys	2008	13	14.34
Austria	Boys	2009	13	14.44
Austria	Boys	2010	13	14.54
Austria	Boys	2011	13	14.64
Austria	Boys	2012	13	14.74
Austria	Boys	2013	13	14.83
Austria	Boys	2014	13	14.92
Austria	Boys	2015	13	15.02
Austria	Boys	2016	13	15.12
Austria	Boys	1975	14	11.83
Austria	Boys	1976	14	11.96
Austria	Boys	1977	14	12.10
Austria	Boys	1978	14	12.25
Austria	Boys	1979	14	12.40
Austria	Boys	1980	14	12.56
Austria	Boys	1981	14	12.72

Austria	Boys	1982	14	12.89
Austria	Boys	1983	14	13.06
Austria	Boys	1984	14	13.24
Austria	Boys	1985	14	13.42
Austria	Boys	1986	14	13.60
Austria	Boys	1987	14	13.79
Austria	Boys	1988	14	13.98
Austria	Boys	1989	14	14.17
Austria	Boys	1990	14	14.36
Austria	Boys	1991	14	14.54
Austria	Boys	1992	14	14.73
Austria	Boys	1993	14	14.91
Austria	Boys	1994	14	15.08
Austria	Boys	1995	14	15.25
Austria	Boys	1996	14	15.42
Austria	Boys	1997	14	15.58
Austria	Boys	1998	14	15.74
Austria	Boys	1999	14	15.89
Austria	Boys	2000	14	16.04
Austria	Boys	2001	14	16.18
Austria	Boys	2002	14	16.32
Austria	Boys	2003	14	16.46
Austria	Boys	2004	14	16.59
Austria	Boys	2005	14	16.72
Austria	Boys	2006	14	16.84
Austria	Boys	2007	14	16.96
Austria	Boys	2008	14	17.08
Austria	Boys	2009	14	17.19
Austria	Boys	2010	14	17.30
Austria	Boys	2011	14	17.41
Austria	Boys	2012	14	17.52
Austria	Boys	2013	14	17.63
Austria	Boys	2014	14	17.74
Austria	Boys	2015	14	17.85
Austria	Boys	2016	14	17.96
Austria	Boys	1975	15	14.11
Austria	Boys	1976	15	14.25
Austria	Boys	1977	15	14.40
Austria	Boys	1978	15	14.56
Austria	Boys	1979	15	14.72
Austria	Boys	1980	15	14.89
Austria	Boys	1981	15	15.06
Austria	Boys	1982	15	15.24
Austria	Boys	1983	15	15.43
Austria	Boys	1984	15	15.62
Austria	Boys	1985	15	15.81
Austria	Boys	1986	15	16.01
Austria	Boys	1987	15	16.21
Austria	Boys	1988	15	16.42
Austria	Boys	1989	15	16.62
Austria	Boys	1990	15	16.83
Austria	Boys	1991	15	17.03

Austria	Boys	1992	15	17.22
Austria	Boys	1993	15	17.42
Austria	Boys	1994	15	17.61
Austria	Boys	1995	15	17.79
Austria	Boys	1996	15	17.97
Austria	Boys	1997	15	18.15
Austria	Boys	1998	15	18.32
Austria	Boys	1999	15	18.48
Austria	Boys	2000	15	18.64
Austria	Boys	2001	15	18.80
Austria	Boys	2002	15	18.95
Austria	Boys	2003	15	19.09
Austria	Boys	2004	15	19.23
Austria	Boys	2005	15	19.37
Austria	Boys	2006	15	19.50
Austria	Boys	2007	15	19.63
Austria	Boys	2008	15	19.76
Austria	Boys	2009	15	19.88
Austria	Boys	2010	15	20.00
Austria	Boys	2011	15	20.12
Austria	Boys	2012	15	20.23
Austria	Boys	2013	15	20.35
Austria	Boys	2014	15	20.46
Austria	Boys	2015	15	20.58
Austria	Boys	2016	15	20.71
Austria	Boys	1975	16	16.51
Austria	Boys	1976	16	16.66
Austria	Boys	1977	16	16.81
Austria	Boys	1978	16	16.98
Austria	Boys	1979	16	17.14
Austria	Boys	1980	16	17.32
Austria	Boys	1981	16	17.50
Austria	Boys	1982	16	17.69
Austria	Boys	1983	16	17.88
Austria	Boys	1984	16	18.07
Austria	Boys	1985	16	18.28
Austria	Boys	1986	16	18.48
Austria	Boys	1987	16	18.69
Austria	Boys	1988	16	18.91
Austria	Boys	1989	16	19.12
Austria	Boys	1990	16	19.33
Austria	Boys	1991	16	19.54
Austria	Boys	1992	16	19.75
Austria	Boys	1993	16	19.95
Austria	Boys	1994	16	20.15
Austria	Boys	1995	16	20.34
Austria	Boys	1996	16	20.53
Austria	Boys	1997	16	20.72
Austria	Boys	1998	16	20.89
Austria	Boys	1999	16	21.06
Austria	Boys	2000	16	21.23
Austria	Boys	2001	16	21.39

Austria	Boys	2002	16	21.55
Austria	Boys	2003	16	21.70
Austria	Boys	2004	16	21.85
Austria	Boys	2005	16	21.99
Austria	Boys	2006	16	22.13
Austria	Boys	2007	16	22.26
Austria	Boys	2008	16	22.39
Austria	Boys	2009	16	22.52
Austria	Boys	2010	16	22.64
Austria	Boys	2011	16	22.76
Austria	Boys	2012	16	22.88
Austria	Boys	2013	16	23.00
Austria	Boys	2014	16	23.13
Austria	Boys	2015	16	23.25
Austria	Boys	2016	16	23.38
Austria	Boys	1975	17	18.99
Austria	Boys	1976	17	19.14
Austria	Boys	1977	17	19.30
Austria	Boys	1978	17	19.47
Austria	Boys	1979	17	19.64
Austria	Boys	1980	17	19.82
Austria	Boys	1981	17	20.01
Austria	Boys	1982	17	20.20
Austria	Boys	1983	17	20.40
Austria	Boys	1984	17	20.60
Austria	Boys	1985	17	20.81
Austria	Boys	1986	17	21.02
Austria	Boys	1987	17	21.24
Austria	Boys	1988	17	21.46
Austria	Boys	1989	17	21.68
Austria	Boys	1990	17	21.90
Austria	Boys	1991	17	22.11
Austria	Boys	1992	17	22.33
Austria	Boys	1993	17	22.54
Austria	Boys	1994	17	22.74
Austria	Boys	1995	17	22.94
Austria	Boys	1996	17	23.14
Austria	Boys	1997	17	23.33
Austria	Boys	1998	17	23.51
Austria	Boys	1999	17	23.69
Austria	Boys	2000	17	23.86
Austria	Boys	2001	17	24.02
Austria	Boys	2002	17	24.19
Austria	Boys	2003	17	24.35
Austria	Boys	2004	17	24.50
Austria	Boys	2005	17	24.64
Austria	Boys	2006	17	24.79
Austria	Boys	2007	17	24.92
Austria	Boys	2008	17	25.06
Austria	Boys	2009	17	25.19
Austria	Boys	2010	17	25.32
Austria	Boys	2011	17	25.44

Austria	Boys	2012	17	25.57
Austria	Boys	2013	17	25.69
Austria	Boys	2014	17	25.82
Austria	Boys	2015	17	25.95
Austria	Boys	2016	17	26.08
Austria	Boys	1975	18	21.56
Austria	Boys	1976	18	21.71
Austria	Boys	1977	18	21.88
Austria	Boys	1978	18	22.06
Austria	Boys	1979	18	22.24
Austria	Boys	1980	18	22.43
Austria	Boys	1981	18	22.62
Austria	Boys	1982	18	22.83
Austria	Boys	1983	18	23.03
Austria	Boys	1984	18	23.25
Austria	Boys	1985	18	23.46
Austria	Boys	1986	18	23.69
Austria	Boys	1987	18	23.91
Austria	Boys	1988	18	24.14
Austria	Boys	1989	18	24.37
Austria	Boys	1990	18	24.60
Austria	Boys	1991	18	24.83
Austria	Boys	1992	18	25.05
Austria	Boys	1993	18	25.28
Austria	Boys	1994	18	25.49
Austria	Boys	1995	18	25.70
Austria	Boys	1996	18	25.90
Austria	Boys	1997	18	26.10
Austria	Boys	1998	18	26.29
Austria	Boys	1999	18	26.48
Austria	Boys	2000	18	26.66
Austria	Boys	2001	18	26.83
Austria	Boys	2002	18	27.00
Austria	Boys	2003	18	27.17
Austria	Boys	2004	18	27.33
Austria	Boys	2005	18	27.48
Austria	Boys	2006	18	27.63
Austria	Boys	2007	18	27.77
Austria	Boys	2008	18	27.91
Austria	Boys	2009	18	28.05
Austria	Boys	2010	18	28.19
Austria	Boys	2011	18	28.32
Austria	Boys	2012	18	28.45
Austria	Boys	2013	18	28.58
Austria	Boys	2014	18	28.72
Austria	Boys	2015	18	28.85
Austria	Boys	2016	18	28.99
Austria	Boys	1975	Average	14.38
Austria	Boys	1976	Average	14.51
Austria	Boys	1977	Average	14.65
Austria	Boys	1978	Average	14.80
Austria	Boys	1979	Average	14.96

Austria	Boys	1980	Average	15.12
Austria	Boys	1981	Average	15.28
Austria	Boys	1982	Average	15.45
Austria	Boys	1983	Average	15.63
Austria	Boys	1984	Average	15.81
Austria	Boys	1985	Average	16.00
Austria	Boys	1986	Average	16.19
Austria	Boys	1987	Average	16.38
Austria	Boys	1988	Average	16.57
Austria	Boys	1989	Average	16.77
Austria	Boys	1990	Average	16.96
Austria	Boys	1991	Average	17.15
Austria	Boys	1992	Average	17.34
Austria	Boys	1993	Average	17.53
Austria	Boys	1994	Average	17.71
Austria	Boys	1995	Average	17.88
Austria	Boys	1996	Average	18.05
Austria	Boys	1997	Average	18.22
Austria	Boys	1998	Average	18.38
Austria	Boys	1999	Average	18.54
Austria	Boys	2000	Average	18.69
Austria	Boys	2001	Average	18.84
Austria	Boys	2002	Average	18.98
Austria	Boys	2003	Average	19.12
Austria	Boys	2004	Average	19.26
Austria	Boys	2005	Average	19.39
Austria	Boys	2006	Average	19.51
Austria	Boys	2007	Average	19.63
Austria	Boys	2008	Average	19.75
Austria	Boys	2009	Average	19.87
Austria	Boys	2010	Average	19.98
Austria	Boys	2011	Average	20.10
Austria	Boys	2012	Average	20.21
Austria	Boys	2013	Average	20.32
Austria	Boys	2014	Average	20.43
Austria	Boys	2015	Average	20.54
Austria	Boys	2016	Average	20.66
Austria	Girls	1975	12	7.32
Austria	Girls	1976	12	7.38
Austria	Girls	1977	12	7.43
Austria	Girls	1978	12	7.48
Austria	Girls	1979	12	7.54
Austria	Girls	1980	12	7.60
Austria	Girls	1981	12	7.66
Austria	Girls	1982	12	7.72
Austria	Girls	1983	12	7.79
Austria	Girls	1984	12	7.85
Austria	Girls	1985	12	7.92
Austria	Girls	1986	12	7.98
Austria	Girls	1987	12	8.05
Austria	Girls	1988	12	8.12
Austria	Girls	1989	12	8.18

Austria	Girls	1990	12	8.25
Austria	Girls	1991	12	8.32
Austria	Girls	1992	12	8.38
Austria	Girls	1993	12	8.45
Austria	Girls	1994	12	8.51
Austria	Girls	1995	12	8.58
Austria	Girls	1996	12	8.64
Austria	Girls	1997	12	8.70
Austria	Girls	1998	12	8.76
Austria	Girls	1999	12	8.82
Austria	Girls	2000	12	8.88
Austria	Girls	2001	12	8.94
Austria	Girls	2002	12	9.00
Austria	Girls	2003	12	9.05
Austria	Girls	2004	12	9.11
Austria	Girls	2005	12	9.17
Austria	Girls	2006	12	9.22
Austria	Girls	2007	12	9.28
Austria	Girls	2008	12	9.33
Austria	Girls	2009	12	9.39
Austria	Girls	2010	12	9.45
Austria	Girls	2011	12	9.51
Austria	Girls	2012	12	9.57
Austria	Girls	2013	12	9.63
Austria	Girls	2014	12	9.70
Austria	Girls	2015	12	9.77
Austria	Girls	2016	12	9.84
Austria	Girls	1975	13	9.01
Austria	Girls	1976	13	9.07
Austria	Girls	1977	13	9.14
Austria	Girls	1978	13	9.20
Austria	Girls	1979	13	9.27
Austria	Girls	1980	13	9.34
Austria	Girls	1981	13	9.41
Austria	Girls	1982	13	9.48
Austria	Girls	1983	13	9.55
Austria	Girls	1984	13	9.63
Austria	Girls	1985	13	9.70
Austria	Girls	1986	13	9.78
Austria	Girls	1987	13	9.86
Austria	Girls	1988	13	9.94
Austria	Girls	1989	13	10.01
Austria	Girls	1990	13	10.09
Austria	Girls	1991	13	10.17
Austria	Girls	1992	13	10.24
Austria	Girls	1993	13	10.32
Austria	Girls	1994	13	10.39
Austria	Girls	1995	13	10.47
Austria	Girls	1996	13	10.54
Austria	Girls	1997	13	10.61
Austria	Girls	1998	13	10.68
Austria	Girls	1999	13	10.75

Austria	Girls	2000	13	10.81
Austria	Girls	2001	13	10.88
Austria	Girls	2002	13	10.94
Austria	Girls	2003	13	11.01
Austria	Girls	2004	13	11.07
Austria	Girls	2005	13	11.13
Austria	Girls	2006	13	11.20
Austria	Girls	2007	13	11.26
Austria	Girls	2008	13	11.32
Austria	Girls	2009	13	11.38
Austria	Girls	2010	13	11.45
Austria	Girls	2011	13	11.52
Austria	Girls	2012	13	11.59
Austria	Girls	2013	13	11.66
Austria	Girls	2014	13	11.73
Austria	Girls	2015	13	11.81
Austria	Girls	2016	13	11.89
Austria	Girls	1975	14	10.75
Austria	Girls	1976	14	10.81
Austria	Girls	1977	14	10.88
Austria	Girls	1978	14	10.96
Austria	Girls	1979	14	11.03
Austria	Girls	1980	14	11.10
Austria	Girls	1981	14	11.18
Austria	Girls	1982	14	11.26
Austria	Girls	1983	14	11.34
Austria	Girls	1984	14	11.43
Austria	Girls	1985	14	11.51
Austria	Girls	1986	14	11.60
Austria	Girls	1987	14	11.68
Austria	Girls	1988	14	11.77
Austria	Girls	1989	14	11.85
Austria	Girls	1990	14	11.94
Austria	Girls	1991	14	12.02
Austria	Girls	1992	14	12.10
Austria	Girls	1993	14	12.19
Austria	Girls	1994	14	12.27
Austria	Girls	1995	14	12.35
Austria	Girls	1996	14	12.42
Austria	Girls	1997	14	12.50
Austria	Girls	1998	14	12.58
Austria	Girls	1999	14	12.65
Austria	Girls	2000	14	12.72
Austria	Girls	2001	14	12.79
Austria	Girls	2002	14	12.86
Austria	Girls	2003	14	12.93
Austria	Girls	2004	14	13.00
Austria	Girls	2005	14	13.07
Austria	Girls	2006	14	13.13
Austria	Girls	2007	14	13.20
Austria	Girls	2008	14	13.26
Austria	Girls	2009	14	13.33

Austria	Girls	2010	14	13.40
Austria	Girls	2011	14	13.47
Austria	Girls	2012	14	13.54
Austria	Girls	2013	14	13.62
Austria	Girls	2014	14	13.70
Austria	Girls	2015	14	13.78
Austria	Girls	2016	14	13.86
Austria	Girls	1975	15	12.30
Austria	Girls	1976	15	12.37
Austria	Girls	1977	15	12.45
Austria	Girls	1978	15	12.52
Austria	Girls	1979	15	12.60
Austria	Girls	1980	15	12.68
Austria	Girls	1981	15	12.76
Austria	Girls	1982	15	12.85
Austria	Girls	1983	15	12.93
Austria	Girls	1984	15	13.02
Austria	Girls	1985	15	13.11
Austria	Girls	1986	15	13.20
Austria	Girls	1987	15	13.29
Austria	Girls	1988	15	13.38
Austria	Girls	1989	15	13.47
Austria	Girls	1990	15	13.56
Austria	Girls	1991	15	13.65
Austria	Girls	1992	15	13.73
Austria	Girls	1993	15	13.82
Austria	Girls	1994	15	13.91
Austria	Girls	1995	15	13.99
Austria	Girls	1996	15	14.07
Austria	Girls	1997	15	14.15
Austria	Girls	1998	15	14.23
Austria	Girls	1999	15	14.31
Austria	Girls	2000	15	14.38
Austria	Girls	2001	15	14.45
Austria	Girls	2002	15	14.53
Austria	Girls	2003	15	14.60
Austria	Girls	2004	15	14.67
Austria	Girls	2005	15	14.74
Austria	Girls	2006	15	14.81
Austria	Girls	2007	15	14.87
Austria	Girls	2008	15	14.94
Austria	Girls	2009	15	15.01
Austria	Girls	2010	15	15.08
Austria	Girls	2011	15	15.15
Austria	Girls	2012	15	15.23
Austria	Girls	2013	15	15.30
Austria	Girls	2014	15	15.38
Austria	Girls	2015	15	15.47
Austria	Girls	2016	15	15.55
Austria	Girls	1975	16	13.53
Austria	Girls	1976	16	13.61
Austria	Girls	1977	16	13.68

Austria	Girls	1978	16	13.76
Austria	Girls	1979	16	13.84
Austria	Girls	1980	16	13.92
Austria	Girls	1981	16	14.01
Austria	Girls	1982	16	14.10
Austria	Girls	1983	16	14.19
Austria	Girls	1984	16	14.28
Austria	Girls	1985	16	14.37
Austria	Girls	1986	16	14.46
Austria	Girls	1987	16	14.55
Austria	Girls	1988	16	14.65
Austria	Girls	1989	16	14.74
Austria	Girls	1990	16	14.83
Austria	Girls	1991	16	14.93
Austria	Girls	1992	16	15.02
Austria	Girls	1993	16	15.11
Austria	Girls	1994	16	15.19
Austria	Girls	1995	16	15.28
Austria	Girls	1996	16	15.36
Austria	Girls	1997	16	15.45
Austria	Girls	1998	16	15.53
Austria	Girls	1999	16	15.61
Austria	Girls	2000	16	15.68
Austria	Girls	2001	16	15.76
Austria	Girls	2002	16	15.84
Austria	Girls	2003	16	15.91
Austria	Girls	2004	16	15.98
Austria	Girls	2005	16	16.05
Austria	Girls	2006	16	16.12
Austria	Girls	2007	16	16.19
Austria	Girls	2008	16	16.26
Austria	Girls	2009	16	16.33
Austria	Girls	2010	16	16.40
Austria	Girls	2011	16	16.48
Austria	Girls	2012	16	16.55
Austria	Girls	2013	16	16.63
Austria	Girls	2014	16	16.71
Austria	Girls	2015	16	16.80
Austria	Girls	2016	16	16.89
Austria	Girls	1975	17	14.46
Austria	Girls	1976	17	14.53
Austria	Girls	1977	17	14.61
Austria	Girls	1978	17	14.69
Austria	Girls	1979	17	14.77
Austria	Girls	1980	17	14.86
Austria	Girls	1981	17	14.95
Austria	Girls	1982	17	15.04
Austria	Girls	1983	17	15.13
Austria	Girls	1984	17	15.23
Austria	Girls	1985	17	15.32
Austria	Girls	1986	17	15.42
Austria	Girls	1987	17	15.52

Austria	Girls	1988	17	15.61
Austria	Girls	1989	17	15.71
Austria	Girls	1990	17	15.81
Austria	Girls	1991	17	15.90
Austria	Girls	1992	17	16.00
Austria	Girls	1993	17	16.09
Austria	Girls	1994	17	16.18
Austria	Girls	1995	17	16.27
Austria	Girls	1996	17	16.36
Austria	Girls	1997	17	16.45
Austria	Girls	1998	17	16.53
Austria	Girls	1999	17	16.62
Austria	Girls	2000	17	16.70
Austria	Girls	2001	17	16.78
Austria	Girls	2002	17	16.86
Austria	Girls	2003	17	16.93
Austria	Girls	2004	17	17.01
Austria	Girls	2005	17	17.08
Austria	Girls	2006	17	17.16
Austria	Girls	2007	17	17.23
Austria	Girls	2008	17	17.30
Austria	Girls	2009	17	17.38
Austria	Girls	2010	17	17.45
Austria	Girls	2011	17	17.53
Austria	Girls	2012	17	17.61
Austria	Girls	2013	17	17.69
Austria	Girls	2014	17	17.78
Austria	Girls	2015	17	17.87
Austria	Girls	2016	17	17.96
Austria	Girls	1975	18	15.22
Austria	Girls	1976	18	15.31
Austria	Girls	1977	18	15.39
Austria	Girls	1978	18	15.48
Austria	Girls	1979	18	15.56
Austria	Girls	1980	18	15.66
Austria	Girls	1981	18	15.75
Austria	Girls	1982	18	15.85
Austria	Girls	1983	18	15.95
Austria	Girls	1984	18	16.05
Austria	Girls	1985	18	16.15
Austria	Girls	1986	18	16.26
Austria	Girls	1987	18	16.36
Austria	Girls	1988	18	16.46
Austria	Girls	1989	18	16.57
Austria	Girls	1990	18	16.67
Austria	Girls	1991	18	16.78
Austria	Girls	1992	18	16.88
Austria	Girls	1993	18	16.98
Austria	Girls	1994	18	17.08
Austria	Girls	1995	18	17.18
Austria	Girls	1996	18	17.27
Austria	Girls	1997	18	17.37

Austria	Girls	1998	18	17.46
Austria	Girls	1999	18	17.55
Austria	Girls	2000	18	17.64
Austria	Girls	2001	18	17.72
Austria	Girls	2002	18	17.81
Austria	Girls	2003	18	17.89
Austria	Girls	2004	18	17.98
Austria	Girls	2005	18	18.06
Austria	Girls	2006	18	18.14
Austria	Girls	2007	18	18.22
Austria	Girls	2008	18	18.30
Austria	Girls	2009	18	18.38
Austria	Girls	2010	18	18.47
Austria	Girls	2011	18	18.55
Austria	Girls	2012	18	18.64
Austria	Girls	2013	18	18.73
Austria	Girls	2014	18	18.82
Austria	Girls	2015	18	18.92
Austria	Girls	2016	18	19.03
Austria	Girls	1975	Average	11.80
Austria	Girls	1976	Average	11.87
Austria	Girls	1977	Average	11.94
Austria	Girls	1978	Average	12.01
Austria	Girls	1979	Average	12.09
Austria	Girls	1980	Average	12.17
Austria	Girls	1981	Average	12.25
Austria	Girls	1982	Average	12.33
Austria	Girls	1983	Average	12.41
Austria	Girls	1984	Average	12.50
Austria	Girls	1985	Average	12.58
Austria	Girls	1986	Average	12.67
Austria	Girls	1987	Average	12.76
Austria	Girls	1988	Average	12.85
Austria	Girls	1989	Average	12.93
Austria	Girls	1990	Average	13.02
Austria	Girls	1991	Average	13.11
Austria	Girls	1992	Average	13.19
Austria	Girls	1993	Average	13.28
Austria	Girls	1994	Average	13.36
Austria	Girls	1995	Average	13.44
Austria	Girls	1996	Average	13.52
Austria	Girls	1997	Average	13.60
Austria	Girls	1998	Average	13.68
Austria	Girls	1999	Average	13.76
Austria	Girls	2000	Average	13.83
Austria	Girls	2001	Average	13.90
Austria	Girls	2002	Average	13.98
Austria	Girls	2003	Average	14.05
Austria	Girls	2004	Average	14.12
Austria	Girls	2005	Average	14.19
Austria	Girls	2006	Average	14.25
Austria	Girls	2007	Average	14.32

Austria	Girls	2008	Average	14.39
Austria	Girls	2009	Average	14.46
Austria	Girls	2010	Average	14.53
Austria	Girls	2011	Average	14.60
Austria	Girls	2012	Average	14.68
Austria	Girls	2013	Average	14.75
Austria	Girls	2014	Average	14.83
Austria	Girls	2015	Average	14.91
Austria	Girls	2016	Average	15.00
Azerbaijan	Boys	1975	12	5.86
Azerbaijan	Boys	1976	12	5.91
Azerbaijan	Boys	1977	12	5.96
Azerbaijan	Boys	1978	12	6.01
Azerbaijan	Boys	1979	12	6.06
Azerbaijan	Boys	1980	12	6.11
Azerbaijan	Boys	1981	12	6.17
Azerbaijan	Boys	1982	12	6.23
Azerbaijan	Boys	1983	12	6.29
Azerbaijan	Boys	1984	12	6.35
Azerbaijan	Boys	1985	12	6.41
Azerbaijan	Boys	1986	12	6.48
Azerbaijan	Boys	1987	12	6.54
Azerbaijan	Boys	1988	12	6.61
Azerbaijan	Boys	1989	12	6.68
Azerbaijan	Boys	1990	12	6.75
Azerbaijan	Boys	1991	12	6.82
Azerbaijan	Boys	1992	12	6.88
Azerbaijan	Boys	1993	12	6.95
Azerbaijan	Boys	1994	12	7.02
Azerbaijan	Boys	1995	12	7.08
Azerbaijan	Boys	1996	12	7.15
Azerbaijan	Boys	1997	12	7.22
Azerbaijan	Boys	1998	12	7.30
Azerbaijan	Boys	1999	12	7.37
Azerbaijan	Boys	2000	12	7.45
Azerbaijan	Boys	2001	12	7.54
Azerbaijan	Boys	2002	12	7.63
Azerbaijan	Boys	2003	12	7.73
Azerbaijan	Boys	2004	12	7.83
Azerbaijan	Boys	2005	12	7.94
Azerbaijan	Boys	2006	12	8.05
Azerbaijan	Boys	2007	12	8.17
Azerbaijan	Boys	2008	12	8.29
Azerbaijan	Boys	2009	12	8.41
Azerbaijan	Boys	2010	12	8.54
Azerbaijan	Boys	2011	12	8.67
Azerbaijan	Boys	2012	12	8.81
Azerbaijan	Boys	2013	12	8.96
Azerbaijan	Boys	2014	12	9.11
Azerbaijan	Boys	2015	12	9.26
Azerbaijan	Boys	2016	12	9.42
Azerbaijan	Boys	1975	13	7.27

Azerbaijan	Boys	1976	13	7.32
Azerbaijan	Boys	1977	13	7.38
Azerbaijan	Boys	1978	13	7.44
Azerbaijan	Boys	1979	13	7.50
Azerbaijan	Boys	1980	13	7.56
Azerbaijan	Boys	1981	13	7.63
Azerbaijan	Boys	1982	13	7.70
Azerbaijan	Boys	1983	13	7.77
Azerbaijan	Boys	1984	13	7.84
Azerbaijan	Boys	1985	13	7.92
Azerbaijan	Boys	1986	13	7.99
Azerbaijan	Boys	1987	13	8.07
Azerbaijan	Boys	1988	13	8.15
Azerbaijan	Boys	1989	13	8.23
Azerbaijan	Boys	1990	13	8.31
Azerbaijan	Boys	1991	13	8.39
Azerbaijan	Boys	1992	13	8.47
Azerbaijan	Boys	1993	13	8.55
Azerbaijan	Boys	1994	13	8.63
Azerbaijan	Boys	1995	13	8.70
Azerbaijan	Boys	1996	13	8.79
Azerbaijan	Boys	1997	13	8.87
Azerbaijan	Boys	1998	13	8.96
Azerbaijan	Boys	1999	13	9.05
Azerbaijan	Boys	2000	13	9.14
Azerbaijan	Boys	2001	13	9.24
Azerbaijan	Boys	2002	13	9.35
Azerbaijan	Boys	2003	13	9.46
Azerbaijan	Boys	2004	13	9.58
Azerbaijan	Boys	2005	13	9.71
Azerbaijan	Boys	2006	13	9.84
Azerbaijan	Boys	2007	13	9.98
Azerbaijan	Boys	2008	13	10.12
Azerbaijan	Boys	2009	13	10.27
Azerbaijan	Boys	2010	13	10.42
Azerbaijan	Boys	2011	13	10.58
Azerbaijan	Boys	2012	13	10.74
Azerbaijan	Boys	2013	13	10.91
Azerbaijan	Boys	2014	13	11.09
Azerbaijan	Boys	2015	13	11.27
Azerbaijan	Boys	2016	13	11.46
Azerbaijan	Boys	1975	14	9.00
Azerbaijan	Boys	1976	14	9.06
Azerbaijan	Boys	1977	14	9.13
Azerbaijan	Boys	1978	14	9.20
Azerbaijan	Boys	1979	14	9.27
Azerbaijan	Boys	1980	14	9.34
Azerbaijan	Boys	1981	14	9.42
Azerbaijan	Boys	1982	14	9.49
Azerbaijan	Boys	1983	14	9.57
Azerbaijan	Boys	1984	14	9.65
Azerbaijan	Boys	1985	14	9.74

Azerbaijan	Boys	1986	14	9.82
Azerbaijan	Boys	1987	14	9.91
Azerbaijan	Boys	1988	14	10.00
Azerbaijan	Boys	1989	14	10.09
Azerbaijan	Boys	1990	14	10.18
Azerbaijan	Boys	1991	14	10.27
Azerbaijan	Boys	1992	14	10.36
Azerbaijan	Boys	1993	14	10.45
Azerbaijan	Boys	1994	14	10.53
Azerbaijan	Boys	1995	14	10.62
Azerbaijan	Boys	1996	14	10.71
Azerbaijan	Boys	1997	14	10.81
Azerbaijan	Boys	1998	14	10.90
Azerbaijan	Boys	1999	14	11.00
Azerbaijan	Boys	2000	14	11.11
Azerbaijan	Boys	2001	14	11.22
Azerbaijan	Boys	2002	14	11.34
Azerbaijan	Boys	2003	14	11.47
Azerbaijan	Boys	2004	14	11.60
Azerbaijan	Boys	2005	14	11.74
Azerbaijan	Boys	2006	14	11.89
Azerbaijan	Boys	2007	14	12.04
Azerbaijan	Boys	2008	14	12.20
Azerbaijan	Boys	2009	14	12.36
Azerbaijan	Boys	2010	14	12.53
Azerbaijan	Boys	2011	14	12.70
Azerbaijan	Boys	2012	14	12.89
Azerbaijan	Boys	2013	14	13.08
Azerbaijan	Boys	2014	14	13.28
Azerbaijan	Boys	2015	14	13.48
Azerbaijan	Boys	2016	14	13.70
Azerbaijan	Boys	1975	15	10.99
Azerbaijan	Boys	1976	15	11.06
Azerbaijan	Boys	1977	15	11.13
Azerbaijan	Boys	1978	15	11.21
Azerbaijan	Boys	1979	15	11.29
Azerbaijan	Boys	1980	15	11.37
Azerbaijan	Boys	1981	15	11.45
Azerbaijan	Boys	1982	15	11.53
Azerbaijan	Boys	1983	15	11.62
Azerbaijan	Boys	1984	15	11.70
Azerbaijan	Boys	1985	15	11.79
Azerbaijan	Boys	1986	15	11.89
Azerbaijan	Boys	1987	15	11.98
Azerbaijan	Boys	1988	15	12.07
Azerbaijan	Boys	1989	15	12.17
Azerbaijan	Boys	1990	15	12.27
Azerbaijan	Boys	1991	15	12.37
Azerbaijan	Boys	1992	15	12.46
Azerbaijan	Boys	1993	15	12.55
Azerbaijan	Boys	1994	15	12.65
Azerbaijan	Boys	1995	15	12.74

Azerbaijan	Boys	1996	15	12.84
Azerbaijan	Boys	1997	15	12.94
Azerbaijan	Boys	1998	15	13.04
Azerbaijan	Boys	1999	15	13.15
Azerbaijan	Boys	2000	15	13.26
Azerbaijan	Boys	2001	15	13.38
Azerbaijan	Boys	2002	15	13.51
Azerbaijan	Boys	2003	15	13.64
Azerbaijan	Boys	2004	15	13.78
Azerbaijan	Boys	2005	15	13.93
Azerbaijan	Boys	2006	15	14.09
Azerbaijan	Boys	2007	15	14.25
Azerbaijan	Boys	2008	15	14.41
Azerbaijan	Boys	2009	15	14.59
Azerbaijan	Boys	2010	15	14.77
Azerbaijan	Boys	2011	15	14.95
Azerbaijan	Boys	2012	15	15.14
Azerbaijan	Boys	2013	15	15.35
Azerbaijan	Boys	2014	15	15.56
Azerbaijan	Boys	2015	15	15.77
Azerbaijan	Boys	2016	15	16.00
Azerbaijan	Boys	1975	16	13.16
Azerbaijan	Boys	1976	16	13.24
Azerbaijan	Boys	1977	16	13.31
Azerbaijan	Boys	1978	16	13.39
Azerbaijan	Boys	1979	16	13.48
Azerbaijan	Boys	1980	16	13.56
Azerbaijan	Boys	1981	16	13.65
Azerbaijan	Boys	1982	16	13.74
Azerbaijan	Boys	1983	16	13.83
Azerbaijan	Boys	1984	16	13.92
Azerbaijan	Boys	1985	16	14.01
Azerbaijan	Boys	1986	16	14.11
Azerbaijan	Boys	1987	16	14.21
Azerbaijan	Boys	1988	16	14.30
Azerbaijan	Boys	1989	16	14.40
Azerbaijan	Boys	1990	16	14.51
Azerbaijan	Boys	1991	16	14.61
Azerbaijan	Boys	1992	16	14.71
Azerbaijan	Boys	1993	16	14.80
Azerbaijan	Boys	1994	16	14.90
Azerbaijan	Boys	1995	16	15.00
Azerbaijan	Boys	1996	16	15.10
Azerbaijan	Boys	1997	16	15.20
Azerbaijan	Boys	1998	16	15.31
Azerbaijan	Boys	1999	16	15.42
Azerbaijan	Boys	2000	16	15.54
Azerbaijan	Boys	2001	16	15.66
Azerbaijan	Boys	2002	16	15.79
Azerbaijan	Boys	2003	16	15.93
Azerbaijan	Boys	2004	16	16.08
Azerbaijan	Boys	2005	16	16.23

Azerbaijan	Boys	2006	16	16.39
Azerbaijan	Boys	2007	16	16.55
Azerbaijan	Boys	2008	16	16.73
Azerbaijan	Boys	2009	16	16.90
Azerbaijan	Boys	2010	16	17.09
Azerbaijan	Boys	2011	16	17.28
Azerbaijan	Boys	2012	16	17.47
Azerbaijan	Boys	2013	16	17.68
Azerbaijan	Boys	2014	16	17.90
Azerbaijan	Boys	2015	16	18.12
Azerbaijan	Boys	2016	16	18.36
Azerbaijan	Boys	1975	17	15.42
Azerbaijan	Boys	1976	17	15.49
Azerbaijan	Boys	1977	17	15.58
Azerbaijan	Boys	1978	17	15.66
Azerbaijan	Boys	1979	17	15.75
Azerbaijan	Boys	1980	17	15.84
Azerbaijan	Boys	1981	17	15.93
Azerbaijan	Boys	1982	17	16.02
Azerbaijan	Boys	1983	17	16.11
Azerbaijan	Boys	1984	17	16.21
Azerbaijan	Boys	1985	17	16.31
Azerbaijan	Boys	1986	17	16.41
Azerbaijan	Boys	1987	17	16.51
Azerbaijan	Boys	1988	17	16.61
Azerbaijan	Boys	1989	17	16.72
Azerbaijan	Boys	1990	17	16.82
Azerbaijan	Boys	1991	17	16.93
Azerbaijan	Boys	1992	17	17.03
Azerbaijan	Boys	1993	17	17.13
Azerbaijan	Boys	1994	17	17.23
Azerbaijan	Boys	1995	17	17.33
Azerbaijan	Boys	1996	17	17.43
Azerbaijan	Boys	1997	17	17.54
Azerbaijan	Boys	1998	17	17.65
Azerbaijan	Boys	1999	17	17.76
Azerbaijan	Boys	2000	17	17.88
Azerbaijan	Boys	2001	17	18.01
Azerbaijan	Boys	2002	17	18.15
Azerbaijan	Boys	2003	17	18.29
Azerbaijan	Boys	2004	17	18.44
Azerbaijan	Boys	2005	17	18.59
Azerbaijan	Boys	2006	17	18.76
Azerbaijan	Boys	2007	17	18.93
Azerbaijan	Boys	2008	17	19.10
Azerbaijan	Boys	2009	17	19.28
Azerbaijan	Boys	2010	17	19.47
Azerbaijan	Boys	2011	17	19.66
Azerbaijan	Boys	2012	17	19.86
Azerbaijan	Boys	2013	17	20.07
Azerbaijan	Boys	2014	17	20.29
Azerbaijan	Boys	2015	17	20.52

Azerbaijan	Boys	2016	17	20.76
Azerbaijan	Boys	1975	18	17.68
Azerbaijan	Boys	1976	18	17.76
Azerbaijan	Boys	1977	18	17.85
Azerbaijan	Boys	1978	18	17.94
Azerbaijan	Boys	1979	18	18.04
Azerbaijan	Boys	1980	18	18.13
Azerbaijan	Boys	1981	18	18.23
Azerbaijan	Boys	1982	18	18.33
Azerbaijan	Boys	1983	18	18.43
Azerbaijan	Boys	1984	18	18.53
Azerbaijan	Boys	1985	18	18.63
Azerbaijan	Boys	1986	18	18.74
Azerbaijan	Boys	1987	18	18.85
Azerbaijan	Boys	1988	18	18.95
Azerbaijan	Boys	1989	18	19.06
Azerbaijan	Boys	1990	18	19.17
Azerbaijan	Boys	1991	18	19.28
Azerbaijan	Boys	1992	18	19.39
Azerbaijan	Boys	1993	18	19.50
Azerbaijan	Boys	1994	18	19.60
Azerbaijan	Boys	1995	18	19.71
Azerbaijan	Boys	1996	18	19.82
Azerbaijan	Boys	1997	18	19.93
Azerbaijan	Boys	1998	18	20.05
Azerbaijan	Boys	1999	18	20.17
Azerbaijan	Boys	2000	18	20.29
Azerbaijan	Boys	2001	18	20.42
Azerbaijan	Boys	2002	18	20.56
Azerbaijan	Boys	2003	18	20.71
Azerbaijan	Boys	2004	18	20.87
Azerbaijan	Boys	2005	18	21.03
Azerbaijan	Boys	2006	18	21.20
Azerbaijan	Boys	2007	18	21.38
Azerbaijan	Boys	2008	18	21.56
Azerbaijan	Boys	2009	18	21.74
Azerbaijan	Boys	2010	18	21.94
Azerbaijan	Boys	2011	18	22.14
Azerbaijan	Boys	2012	18	22.35
Azerbaijan	Boys	2013	18	22.56
Azerbaijan	Boys	2014	18	22.79
Azerbaijan	Boys	2015	18	23.03
Azerbaijan	Boys	2016	18	23.27
Azerbaijan	Boys	1975	Average	11.34
Azerbaijan	Boys	1976	Average	11.41
Azerbaijan	Boys	1977	Average	11.48
Azerbaijan	Boys	1978	Average	11.55
Azerbaijan	Boys	1979	Average	11.62
Azerbaijan	Boys	1980	Average	11.70
Azerbaijan	Boys	1981	Average	11.78
Azerbaijan	Boys	1982	Average	11.86
Azerbaijan	Boys	1983	Average	11.95

Azerbaijan	Boys	1984	Average	12.03
Azerbaijan	Boys	1985	Average	12.12
Azerbaijan	Boys	1986	Average	12.20
Azerbaijan	Boys	1987	Average	12.29
Azerbaijan	Boys	1988	Average	12.39
Azerbaijan	Boys	1989	Average	12.48
Azerbaijan	Boys	1990	Average	12.57
Azerbaijan	Boys	1991	Average	12.67
Azerbaijan	Boys	1992	Average	12.76
Azerbaijan	Boys	1993	Average	12.85
Azerbaijan	Boys	1994	Average	12.94
Azerbaijan	Boys	1995	Average	13.03
Azerbaijan	Boys	1996	Average	13.12
Azerbaijan	Boys	1997	Average	13.21
Azerbaijan	Boys	1998	Average	13.31
Azerbaijan	Boys	1999	Average	13.42
Azerbaijan	Boys	2000	Average	13.52
Azerbaijan	Boys	2001	Average	13.64
Azerbaijan	Boys	2002	Average	13.76
Azerbaijan	Boys	2003	Average	13.89
Azerbaijan	Boys	2004	Average	14.03
Azerbaijan	Boys	2005	Average	14.17
Azerbaijan	Boys	2006	Average	14.32
Azerbaijan	Boys	2007	Average	14.47
Azerbaijan	Boys	2008	Average	14.63
Azerbaijan	Boys	2009	Average	14.79
Azerbaijan	Boys	2010	Average	14.96
Azerbaijan	Boys	2011	Average	15.14
Azerbaijan	Boys	2012	Average	15.32
Azerbaijan	Boys	2013	Average	15.52
Azerbaijan	Boys	2014	Average	15.71
Azerbaijan	Boys	2015	Average	15.92
Azerbaijan	Boys	2016	Average	16.14
Azerbaijan	Girls	1975	12	6.30
Azerbaijan	Girls	1976	12	6.35
Azerbaijan	Girls	1977	12	6.40
Azerbaijan	Girls	1978	12	6.45
Azerbaijan	Girls	1979	12	6.50
Azerbaijan	Girls	1980	12	6.55
Azerbaijan	Girls	1981	12	6.61
Azerbaijan	Girls	1982	12	6.66
Azerbaijan	Girls	1983	12	6.71
Azerbaijan	Girls	1984	12	6.77
Azerbaijan	Girls	1985	12	6.82
Azerbaijan	Girls	1986	12	6.88
Azerbaijan	Girls	1987	12	6.94
Azerbaijan	Girls	1988	12	6.99
Azerbaijan	Girls	1989	12	7.05
Azerbaijan	Girls	1990	12	7.11
Azerbaijan	Girls	1991	12	7.16
Azerbaijan	Girls	1992	12	7.22
Azerbaijan	Girls	1993	12	7.27

Azerbaijan	Girls	1994	12	7.32
Azerbaijan	Girls	1995	12	7.37
Azerbaijan	Girls	1996	12	7.42
Azerbaijan	Girls	1997	12	7.48
Azerbaijan	Girls	1998	12	7.53
Azerbaijan	Girls	1999	12	7.58
Azerbaijan	Girls	2000	12	7.64
Azerbaijan	Girls	2001	12	7.69
Azerbaijan	Girls	2002	12	7.75
Azerbaijan	Girls	2003	12	7.82
Azerbaijan	Girls	2004	12	7.88
Azerbaijan	Girls	2005	12	7.95
Azerbaijan	Girls	2006	12	8.02
Azerbaijan	Girls	2007	12	8.09
Azerbaijan	Girls	2008	12	8.16
Azerbaijan	Girls	2009	12	8.23
Azerbaijan	Girls	2010	12	8.31
Azerbaijan	Girls	2011	12	8.39
Azerbaijan	Girls	2012	12	8.47
Azerbaijan	Girls	2013	12	8.55
Azerbaijan	Girls	2014	12	8.64
Azerbaijan	Girls	2015	12	8.72
Azerbaijan	Girls	2016	12	8.82
Azerbaijan	Girls	1975	13	7.82
Azerbaijan	Girls	1976	13	7.88
Azerbaijan	Girls	1977	13	7.93
Azerbaijan	Girls	1978	13	7.99
Azerbaijan	Girls	1979	13	8.05
Azerbaijan	Girls	1980	13	8.11
Azerbaijan	Girls	1981	13	8.18
Azerbaijan	Girls	1982	13	8.24
Azerbaijan	Girls	1983	13	8.30
Azerbaijan	Girls	1984	13	8.37
Azerbaijan	Girls	1985	13	8.43
Azerbaijan	Girls	1986	13	8.50
Azerbaijan	Girls	1987	13	8.56
Azerbaijan	Girls	1988	13	8.63
Azerbaijan	Girls	1989	13	8.70
Azerbaijan	Girls	1990	13	8.76
Azerbaijan	Girls	1991	13	8.83
Azerbaijan	Girls	1992	13	8.89
Azerbaijan	Girls	1993	13	8.95
Azerbaijan	Girls	1994	13	9.01
Azerbaijan	Girls	1995	13	9.07
Azerbaijan	Girls	1996	13	9.13
Azerbaijan	Girls	1997	13	9.19
Azerbaijan	Girls	1998	13	9.25
Azerbaijan	Girls	1999	13	9.31
Azerbaijan	Girls	2000	13	9.37
Azerbaijan	Girls	2001	13	9.44
Azerbaijan	Girls	2002	13	9.50
Azerbaijan	Girls	2003	13	9.58

Azerbaijan	Girls	2004	13	9.65
Azerbaijan	Girls	2005	13	9.72
Azerbaijan	Girls	2006	13	9.80
Azerbaijan	Girls	2007	13	9.88
Azerbaijan	Girls	2008	13	9.96
Azerbaijan	Girls	2009	13	10.05
Azerbaijan	Girls	2010	13	10.13
Azerbaijan	Girls	2011	13	10.22
Azerbaijan	Girls	2012	13	10.31
Azerbaijan	Girls	2013	13	10.40
Azerbaijan	Girls	2014	13	10.50
Azerbaijan	Girls	2015	13	10.60
Azerbaijan	Girls	2016	13	10.70
Azerbaijan	Girls	1975	14	9.43
Azerbaijan	Girls	1976	14	9.49
Azerbaijan	Girls	1977	14	9.55
Azerbaijan	Girls	1978	14	9.62
Azerbaijan	Girls	1979	14	9.68
Azerbaijan	Girls	1980	14	9.75
Azerbaijan	Girls	1981	14	9.82
Azerbaijan	Girls	1982	14	9.89
Azerbaijan	Girls	1983	14	9.96
Azerbaijan	Girls	1984	14	10.03
Azerbaijan	Girls	1985	14	10.10
Azerbaijan	Girls	1986	14	10.18
Azerbaijan	Girls	1987	14	10.25
Azerbaijan	Girls	1988	14	10.32
Azerbaijan	Girls	1989	14	10.39
Azerbaijan	Girls	1990	14	10.47
Azerbaijan	Girls	1991	14	10.54
Azerbaijan	Girls	1992	14	10.61
Azerbaijan	Girls	1993	14	10.67
Azerbaijan	Girls	1994	14	10.74
Azerbaijan	Girls	1995	14	10.80
Azerbaijan	Girls	1996	14	10.87
Azerbaijan	Girls	1997	14	10.93
Azerbaijan	Girls	1998	14	11.00
Azerbaijan	Girls	1999	14	11.06
Azerbaijan	Girls	2000	14	11.13
Azerbaijan	Girls	2001	14	11.20
Azerbaijan	Girls	2002	14	11.28
Azerbaijan	Girls	2003	14	11.35
Azerbaijan	Girls	2004	14	11.43
Azerbaijan	Girls	2005	14	11.51
Azerbaijan	Girls	2006	14	11.60
Azerbaijan	Girls	2007	14	11.68
Azerbaijan	Girls	2008	14	11.77
Azerbaijan	Girls	2009	14	11.86
Azerbaijan	Girls	2010	14	11.95
Azerbaijan	Girls	2011	14	12.05
Azerbaijan	Girls	2012	14	12.15
Azerbaijan	Girls	2013	14	12.25

Azerbaijan	Girls	2014	14	12.35
Azerbaijan	Girls	2015	14	12.46
Azerbaijan	Girls	2016	14	12.57
Azerbaijan	Girls	1975	15	10.90
Azerbaijan	Girls	1976	15	10.96
Azerbaijan	Girls	1977	15	11.03
Azerbaijan	Girls	1978	15	11.10
Azerbaijan	Girls	1979	15	11.17
Azerbaijan	Girls	1980	15	11.24
Azerbaijan	Girls	1981	15	11.31
Azerbaijan	Girls	1982	15	11.39
Azerbaijan	Girls	1983	15	11.46
Azerbaijan	Girls	1984	15	11.54
Azerbaijan	Girls	1985	15	11.61
Azerbaijan	Girls	1986	15	11.69
Azerbaijan	Girls	1987	15	11.77
Azerbaijan	Girls	1988	15	11.84
Azerbaijan	Girls	1989	15	11.92
Azerbaijan	Girls	1990	15	12.00
Azerbaijan	Girls	1991	15	12.07
Azerbaijan	Girls	1992	15	12.14
Azerbaijan	Girls	1993	15	12.22
Azerbaijan	Girls	1994	15	12.28
Azerbaijan	Girls	1995	15	12.35
Azerbaijan	Girls	1996	15	12.42
Azerbaijan	Girls	1997	15	12.49
Azerbaijan	Girls	1998	15	12.56
Azerbaijan	Girls	1999	15	12.63
Azerbaijan	Girls	2000	15	12.70
Azerbaijan	Girls	2001	15	12.77
Azerbaijan	Girls	2002	15	12.85
Azerbaijan	Girls	2003	15	12.93
Azerbaijan	Girls	2004	15	13.01
Azerbaijan	Girls	2005	15	13.10
Azerbaijan	Girls	2006	15	13.18
Azerbaijan	Girls	2007	15	13.27
Azerbaijan	Girls	2008	15	13.36
Azerbaijan	Girls	2009	15	13.46
Azerbaijan	Girls	2010	15	13.55
Azerbaijan	Girls	2011	15	13.65
Azerbaijan	Girls	2012	15	13.75
Azerbaijan	Girls	2013	15	13.86
Azerbaijan	Girls	2014	15	13.97
Azerbaijan	Girls	2015	15	14.08
Azerbaijan	Girls	2016	15	14.20
Azerbaijan	Girls	1975	16	12.07
Azerbaijan	Girls	1976	16	12.14
Azerbaijan	Girls	1977	16	12.21
Azerbaijan	Girls	1978	16	12.28
Azerbaijan	Girls	1979	16	12.35
Azerbaijan	Girls	1980	16	12.43
Azerbaijan	Girls	1981	16	12.50

Azerbaijan	Girls	1982	16	12.58
Azerbaijan	Girls	1983	16	12.65
Azerbaijan	Girls	1984	16	12.73
Azerbaijan	Girls	1985	16	12.81
Azerbaijan	Girls	1986	16	12.89
Azerbaijan	Girls	1987	16	12.97
Azerbaijan	Girls	1988	16	13.05
Azerbaijan	Girls	1989	16	13.13
Azerbaijan	Girls	1990	16	13.21
Azerbaijan	Girls	1991	16	13.29
Azerbaijan	Girls	1992	16	13.36
Azerbaijan	Girls	1993	16	13.43
Azerbaijan	Girls	1994	16	13.50
Azerbaijan	Girls	1995	16	13.57
Azerbaijan	Girls	1996	16	13.64
Azerbaijan	Girls	1997	16	13.71
Azerbaijan	Girls	1998	16	13.78
Azerbaijan	Girls	1999	16	13.86
Azerbaijan	Girls	2000	16	13.93
Azerbaijan	Girls	2001	16	14.01
Azerbaijan	Girls	2002	16	14.09
Azerbaijan	Girls	2003	16	14.17
Azerbaijan	Girls	2004	16	14.26
Azerbaijan	Girls	2005	16	14.34
Azerbaijan	Girls	2006	16	14.43
Azerbaijan	Girls	2007	16	14.52
Azerbaijan	Girls	2008	16	14.62
Azerbaijan	Girls	2009	16	14.72
Azerbaijan	Girls	2010	16	14.82
Azerbaijan	Girls	2011	16	14.92
Azerbaijan	Girls	2012	16	15.02
Azerbaijan	Girls	2013	16	15.13
Azerbaijan	Girls	2014	16	15.24
Azerbaijan	Girls	2015	16	15.36
Azerbaijan	Girls	2016	16	15.48
Azerbaijan	Girls	1975	17	12.92
Azerbaijan	Girls	1976	17	12.99
Azerbaijan	Girls	1977	17	13.06
Azerbaijan	Girls	1978	17	13.14
Azerbaijan	Girls	1979	17	13.21
Azerbaijan	Girls	1980	17	13.29
Azerbaijan	Girls	1981	17	13.37
Azerbaijan	Girls	1982	17	13.45
Azerbaijan	Girls	1983	17	13.53
Azerbaijan	Girls	1984	17	13.61
Azerbaijan	Girls	1985	17	13.69
Azerbaijan	Girls	1986	17	13.77
Azerbaijan	Girls	1987	17	13.85
Azerbaijan	Girls	1988	17	13.94
Azerbaijan	Girls	1989	17	14.02
Azerbaijan	Girls	1990	17	14.10
Azerbaijan	Girls	1991	17	14.18

Azerbaijan	Girls	1992	17	14.26
Azerbaijan	Girls	1993	17	14.34
Azerbaijan	Girls	1994	17	14.41
Azerbaijan	Girls	1995	17	14.48
Azerbaijan	Girls	1996	17	14.56
Azerbaijan	Girls	1997	17	14.63
Azerbaijan	Girls	1998	17	14.70
Azerbaijan	Girls	1999	17	14.78
Azerbaijan	Girls	2000	17	14.86
Azerbaijan	Girls	2001	17	14.94
Azerbaijan	Girls	2002	17	15.02
Azerbaijan	Girls	2003	17	15.11
Azerbaijan	Girls	2004	17	15.20
Azerbaijan	Girls	2005	17	15.29
Azerbaijan	Girls	2006	17	15.38
Azerbaijan	Girls	2007	17	15.48
Azerbaijan	Girls	2008	17	15.58
Azerbaijan	Girls	2009	17	15.68
Azerbaijan	Girls	2010	17	15.79
Azerbaijan	Girls	2011	17	15.90
Azerbaijan	Girls	2012	17	16.01
Azerbaijan	Girls	2013	17	16.12
Azerbaijan	Girls	2014	17	16.24
Azerbaijan	Girls	2015	17	16.37
Azerbaijan	Girls	2016	17	16.49
Azerbaijan	Girls	1975	18	13.57
Azerbaijan	Girls	1976	18	13.64
Azerbaijan	Girls	1977	18	13.72
Azerbaijan	Girls	1978	18	13.80
Azerbaijan	Girls	1979	18	13.88
Azerbaijan	Girls	1980	18	13.96
Azerbaijan	Girls	1981	18	14.04
Azerbaijan	Girls	1982	18	14.13
Azerbaijan	Girls	1983	18	14.21
Azerbaijan	Girls	1984	18	14.30
Azerbaijan	Girls	1985	18	14.39
Azerbaijan	Girls	1986	18	14.48
Azerbaijan	Girls	1987	18	14.56
Azerbaijan	Girls	1988	18	14.65
Azerbaijan	Girls	1989	18	14.74
Azerbaijan	Girls	1990	18	14.83
Azerbaijan	Girls	1991	18	14.92
Azerbaijan	Girls	1992	18	15.00
Azerbaijan	Girls	1993	18	15.08
Azerbaijan	Girls	1994	18	15.16
Azerbaijan	Girls	1995	18	15.24
Azerbaijan	Girls	1996	18	15.32
Azerbaijan	Girls	1997	18	15.40
Azerbaijan	Girls	1998	18	15.48
Azerbaijan	Girls	1999	18	15.56
Azerbaijan	Girls	2000	18	15.65
Azerbaijan	Girls	2001	18	15.73

Azerbaijan	Girls	2002	18	15.83
Azerbaijan	Girls	2003	18	15.92
Azerbaijan	Girls	2004	18	16.02
Azerbaijan	Girls	2005	18	16.12
Azerbaijan	Girls	2006	18	16.23
Azerbaijan	Girls	2007	18	16.33
Azerbaijan	Girls	2008	18	16.44
Azerbaijan	Girls	2009	18	16.55
Azerbaijan	Girls	2010	18	16.67
Azerbaijan	Girls	2011	18	16.79
Azerbaijan	Girls	2012	18	16.91
Azerbaijan	Girls	2013	18	17.04
Azerbaijan	Girls	2014	18	17.17
Azerbaijan	Girls	2015	18	17.30
Azerbaijan	Girls	2016	18	17.44
Azerbaijan	Girls	1975	Average	10.43
Azerbaijan	Girls	1976	Average	10.49
Azerbaijan	Girls	1977	Average	10.56
Azerbaijan	Girls	1978	Average	10.62
Azerbaijan	Girls	1979	Average	10.69
Azerbaijan	Girls	1980	Average	10.76
Azerbaijan	Girls	1981	Average	10.83
Azerbaijan	Girls	1982	Average	10.90
Azerbaijan	Girls	1983	Average	10.98
Azerbaijan	Girls	1984	Average	11.05
Azerbaijan	Girls	1985	Average	11.12
Azerbaijan	Girls	1986	Average	11.20
Azerbaijan	Girls	1987	Average	11.27
Azerbaijan	Girls	1988	Average	11.35
Azerbaijan	Girls	1989	Average	11.42
Azerbaijan	Girls	1990	Average	11.50
Azerbaijan	Girls	1991	Average	11.57
Azerbaijan	Girls	1992	Average	11.64
Azerbaijan	Girls	1993	Average	11.71
Azerbaijan	Girls	1994	Average	11.78
Azerbaijan	Girls	1995	Average	11.84
Azerbaijan	Girls	1996	Average	11.91
Azerbaijan	Girls	1997	Average	11.98
Azerbaijan	Girls	1998	Average	12.04
Azerbaijan	Girls	1999	Average	12.11
Azerbaijan	Girls	2000	Average	12.18
Azerbaijan	Girls	2001	Average	12.25
Azerbaijan	Girls	2002	Average	12.33
Azerbaijan	Girls	2003	Average	12.41
Azerbaijan	Girls	2004	Average	12.49
Azerbaijan	Girls	2005	Average	12.58
Azerbaijan	Girls	2006	Average	12.66
Azerbaijan	Girls	2007	Average	12.75
Azerbaijan	Girls	2008	Average	12.84
Azerbaijan	Girls	2009	Average	12.94
Azerbaijan	Girls	2010	Average	13.03
Azerbaijan	Girls	2011	Average	13.13

Azerbaijan	Girls	2012	Average	13.23
Azerbaijan	Girls	2013	Average	13.34
Azerbaijan	Girls	2014	Average	13.44
Azerbaijan	Girls	2015	Average	13.56
Azerbaijan	Girls	2016	Average	13.67
Bahamas	Boys	1975	12	7.46
Bahamas	Boys	1976	12	7.57
Bahamas	Boys	1977	12	7.69
Bahamas	Boys	1978	12	7.82
Bahamas	Boys	1979	12	7.95
Bahamas	Boys	1980	12	8.09
Bahamas	Boys	1981	12	8.24
Bahamas	Boys	1982	12	8.39
Bahamas	Boys	1983	12	8.55
Bahamas	Boys	1984	12	8.71
Bahamas	Boys	1985	12	8.88
Bahamas	Boys	1986	12	9.05
Bahamas	Boys	1987	12	9.23
Bahamas	Boys	1988	12	9.41
Bahamas	Boys	1989	12	9.60
Bahamas	Boys	1990	12	9.79
Bahamas	Boys	1991	12	9.97
Bahamas	Boys	1992	12	10.15
Bahamas	Boys	1993	12	10.33
Bahamas	Boys	1994	12	10.50
Bahamas	Boys	1995	12	10.67
Bahamas	Boys	1996	12	10.84
Bahamas	Boys	1997	12	11.01
Bahamas	Boys	1998	12	11.18
Bahamas	Boys	1999	12	11.35
Bahamas	Boys	2000	12	11.52
Bahamas	Boys	2001	12	11.69
Bahamas	Boys	2002	12	11.86
Bahamas	Boys	2003	12	12.04
Bahamas	Boys	2004	12	12.21
Bahamas	Boys	2005	12	12.38
Bahamas	Boys	2006	12	12.55
Bahamas	Boys	2007	12	12.73
Bahamas	Boys	2008	12	12.90
Bahamas	Boys	2009	12	13.07
Bahamas	Boys	2010	12	13.25
Bahamas	Boys	2011	12	13.42
Bahamas	Boys	2012	12	13.60
Bahamas	Boys	2013	12	13.78
Bahamas	Boys	2014	12	13.95
Bahamas	Boys	2015	12	14.13
Bahamas	Boys	2016	12	14.31
Bahamas	Boys	1975	13	9.11
Bahamas	Boys	1976	13	9.25
Bahamas	Boys	1977	13	9.39
Bahamas	Boys	1978	13	9.54
Bahamas	Boys	1979	13	9.69

Bahamas	Boys	1980	13	9.85
Bahamas	Boys	1981	13	10.02
Bahamas	Boys	1982	13	10.20
Bahamas	Boys	1983	13	10.38
Bahamas	Boys	1984	13	10.58
Bahamas	Boys	1985	13	10.78
Bahamas	Boys	1986	13	10.98
Bahamas	Boys	1987	13	11.19
Bahamas	Boys	1988	13	11.41
Bahamas	Boys	1989	13	11.63
Bahamas	Boys	1990	13	11.85
Bahamas	Boys	1991	13	12.07
Bahamas	Boys	1992	13	12.28
Bahamas	Boys	1993	13	12.49
Bahamas	Boys	1994	13	12.70
Bahamas	Boys	1995	13	12.90
Bahamas	Boys	1996	13	13.11
Bahamas	Boys	1997	13	13.31
Bahamas	Boys	1998	13	13.51
Bahamas	Boys	1999	13	13.72
Bahamas	Boys	2000	13	13.92
Bahamas	Boys	2001	13	14.13
Bahamas	Boys	2002	13	14.34
Bahamas	Boys	2003	13	14.54
Bahamas	Boys	2004	13	14.75
Bahamas	Boys	2005	13	14.96
Bahamas	Boys	2006	13	15.17
Bahamas	Boys	2007	13	15.39
Bahamas	Boys	2008	13	15.60
Bahamas	Boys	2009	13	15.81
Bahamas	Boys	2010	13	16.03
Bahamas	Boys	2011	13	16.25
Bahamas	Boys	2012	13	16.47
Bahamas	Boys	2013	13	16.69
Bahamas	Boys	2014	13	16.91
Bahamas	Boys	2015	13	17.14
Bahamas	Boys	2016	13	17.37
Bahamas	Boys	1975	14	11.02
Bahamas	Boys	1976	14	11.17
Bahamas	Boys	1977	14	11.33
Bahamas	Boys	1978	14	11.49
Bahamas	Boys	1979	14	11.66
Bahamas	Boys	1980	14	11.84
Bahamas	Boys	1981	14	12.03
Bahamas	Boys	1982	14	12.22
Bahamas	Boys	1983	14	12.43
Bahamas	Boys	1984	14	12.64
Bahamas	Boys	1985	14	12.86
Bahamas	Boys	1986	14	13.09
Bahamas	Boys	1987	14	13.32
Bahamas	Boys	1988	14	13.56
Bahamas	Boys	1989	14	13.80

Bahamas	Boys	1990	14	14.05
Bahamas	Boys	1991	14	14.29
Bahamas	Boys	1992	14	14.53
Bahamas	Boys	1993	14	14.77
Bahamas	Boys	1994	14	15.00
Bahamas	Boys	1995	14	15.23
Bahamas	Boys	1996	14	15.46
Bahamas	Boys	1997	14	15.69
Bahamas	Boys	1998	14	15.92
Bahamas	Boys	1999	14	16.15
Bahamas	Boys	2000	14	16.38
Bahamas	Boys	2001	14	16.61
Bahamas	Boys	2002	14	16.85
Bahamas	Boys	2003	14	17.08
Bahamas	Boys	2004	14	17.32
Bahamas	Boys	2005	14	17.56
Bahamas	Boys	2006	14	17.80
Bahamas	Boys	2007	14	18.04
Bahamas	Boys	2008	14	18.29
Bahamas	Boys	2009	14	18.54
Bahamas	Boys	2010	14	18.79
Bahamas	Boys	2011	14	19.04
Bahamas	Boys	2012	14	19.30
Bahamas	Boys	2013	14	19.56
Bahamas	Boys	2014	14	19.82
Bahamas	Boys	2015	14	20.09
Bahamas	Boys	2016	14	20.36
Bahamas	Boys	1975	15	13.10
Bahamas	Boys	1976	15	13.26
Bahamas	Boys	1977	15	13.42
Bahamas	Boys	1978	15	13.59
Bahamas	Boys	1979	15	13.77
Bahamas	Boys	1980	15	13.96
Bahamas	Boys	1981	15	14.15
Bahamas	Boys	1982	15	14.35
Bahamas	Boys	1983	15	14.57
Bahamas	Boys	1984	15	14.79
Bahamas	Boys	1985	15	15.02
Bahamas	Boys	1986	15	15.26
Bahamas	Boys	1987	15	15.51
Bahamas	Boys	1988	15	15.76
Bahamas	Boys	1989	15	16.02
Bahamas	Boys	1990	15	16.28
Bahamas	Boys	1991	15	16.54
Bahamas	Boys	1992	15	16.79
Bahamas	Boys	1993	15	17.04
Bahamas	Boys	1994	15	17.28
Bahamas	Boys	1995	15	17.52
Bahamas	Boys	1996	15	17.77
Bahamas	Boys	1997	15	18.01
Bahamas	Boys	1998	15	18.25
Bahamas	Boys	1999	15	18.50

Bahamas	Boys	2000	15	18.74
Bahamas	Boys	2001	15	18.99
Bahamas	Boys	2002	15	19.24
Bahamas	Boys	2003	15	19.50
Bahamas	Boys	2004	15	19.75
Bahamas	Boys	2005	15	20.01
Bahamas	Boys	2006	15	20.27
Bahamas	Boys	2007	15	20.53
Bahamas	Boys	2008	15	20.80
Bahamas	Boys	2009	15	21.06
Bahamas	Boys	2010	15	21.34
Bahamas	Boys	2011	15	21.61
Bahamas	Boys	2012	15	21.89
Bahamas	Boys	2013	15	22.18
Bahamas	Boys	2014	15	22.47
Bahamas	Boys	2015	15	22.77
Bahamas	Boys	2016	15	23.08
Bahamas	Boys	1975	16	15.29
Bahamas	Boys	1976	16	15.45
Bahamas	Boys	1977	16	15.62
Bahamas	Boys	1978	16	15.79
Bahamas	Boys	1979	16	15.98
Bahamas	Boys	1980	16	16.16
Bahamas	Boys	1981	16	16.36
Bahamas	Boys	1982	16	16.57
Bahamas	Boys	1983	16	16.79
Bahamas	Boys	1984	16	17.01
Bahamas	Boys	1985	16	17.25
Bahamas	Boys	1986	16	17.49
Bahamas	Boys	1987	16	17.74
Bahamas	Boys	1988	16	18.00
Bahamas	Boys	1989	16	18.26
Bahamas	Boys	1990	16	18.53
Bahamas	Boys	1991	16	18.79
Bahamas	Boys	1992	16	19.05
Bahamas	Boys	1993	16	19.30
Bahamas	Boys	1994	16	19.55
Bahamas	Boys	1995	16	19.80
Bahamas	Boys	1996	16	20.05
Bahamas	Boys	1997	16	20.30
Bahamas	Boys	1998	16	20.55
Bahamas	Boys	1999	16	20.80
Bahamas	Boys	2000	16	21.05
Bahamas	Boys	2001	16	21.31
Bahamas	Boys	2002	16	21.57
Bahamas	Boys	2003	16	21.83
Bahamas	Boys	2004	16	22.09
Bahamas	Boys	2005	16	22.35
Bahamas	Boys	2006	16	22.62
Bahamas	Boys	2007	16	22.89
Bahamas	Boys	2008	16	23.17
Bahamas	Boys	2009	16	23.45

Bahamas	Boys	2010	16	23.73
Bahamas	Boys	2011	16	24.02
Bahamas	Boys	2012	16	24.32
Bahamas	Boys	2013	16	24.62
Bahamas	Boys	2014	16	24.93
Bahamas	Boys	2015	16	25.25
Bahamas	Boys	2016	16	25.57
Bahamas	Boys	1975	17	17.55
Bahamas	Boys	1976	17	17.72
Bahamas	Boys	1977	17	17.89
Bahamas	Boys	1978	17	18.07
Bahamas	Boys	1979	17	18.25
Bahamas	Boys	1980	17	18.44
Bahamas	Boys	1981	17	18.64
Bahamas	Boys	1982	17	18.85
Bahamas	Boys	1983	17	19.07
Bahamas	Boys	1984	17	19.30
Bahamas	Boys	1985	17	19.54
Bahamas	Boys	1986	17	19.79
Bahamas	Boys	1987	17	20.04
Bahamas	Boys	1988	17	20.30
Bahamas	Boys	1989	17	20.56
Bahamas	Boys	1990	17	20.84
Bahamas	Boys	1991	17	21.10
Bahamas	Boys	1992	17	21.36
Bahamas	Boys	1993	17	21.62
Bahamas	Boys	1994	17	21.87
Bahamas	Boys	1995	17	22.12
Bahamas	Boys	1996	17	22.38
Bahamas	Boys	1997	17	22.63
Bahamas	Boys	1998	17	22.88
Bahamas	Boys	1999	17	23.13
Bahamas	Boys	2000	17	23.39
Bahamas	Boys	2001	17	23.65
Bahamas	Boys	2002	17	23.91
Bahamas	Boys	2003	17	24.18
Bahamas	Boys	2004	17	24.45
Bahamas	Boys	2005	17	24.72
Bahamas	Boys	2006	17	24.99
Bahamas	Boys	2007	17	25.27
Bahamas	Boys	2008	17	25.55
Bahamas	Boys	2009	17	25.83
Bahamas	Boys	2010	17	26.13
Bahamas	Boys	2011	17	26.42
Bahamas	Boys	2012	17	26.73
Bahamas	Boys	2013	17	27.04
Bahamas	Boys	2014	17	27.36
Bahamas	Boys	2015	17	27.69
Bahamas	Boys	2016	17	28.02
Bahamas	Boys	1975	18	19.88
Bahamas	Boys	1976	18	20.05
Bahamas	Boys	1977	18	20.23

Bahamas	Boys	1978	18	20.41
Bahamas	Boys	1979	18	20.60
Bahamas	Boys	1980	18	20.80
Bahamas	Boys	1981	18	21.01
Bahamas	Boys	1982	18	21.23
Bahamas	Boys	1983	18	21.45
Bahamas	Boys	1984	18	21.69
Bahamas	Boys	1985	18	21.94
Bahamas	Boys	1986	18	22.19
Bahamas	Boys	1987	18	22.45
Bahamas	Boys	1988	18	22.72
Bahamas	Boys	1989	18	22.99
Bahamas	Boys	1990	18	23.27
Bahamas	Boys	1991	18	23.54
Bahamas	Boys	1992	18	23.81
Bahamas	Boys	1993	18	24.08
Bahamas	Boys	1994	18	24.34
Bahamas	Boys	1995	18	24.59
Bahamas	Boys	1996	18	24.85
Bahamas	Boys	1997	18	25.11
Bahamas	Boys	1998	18	25.37
Bahamas	Boys	1999	18	25.63
Bahamas	Boys	2000	18	25.90
Bahamas	Boys	2001	18	26.16
Bahamas	Boys	2002	18	26.43
Bahamas	Boys	2003	18	26.70
Bahamas	Boys	2004	18	26.98
Bahamas	Boys	2005	18	27.26
Bahamas	Boys	2006	18	27.54
Bahamas	Boys	2007	18	27.82
Bahamas	Boys	2008	18	28.11
Bahamas	Boys	2009	18	28.40
Bahamas	Boys	2010	18	28.70
Bahamas	Boys	2011	18	29.01
Bahamas	Boys	2012	18	29.32
Bahamas	Boys	2013	18	29.64
Bahamas	Boys	2014	18	29.97
Bahamas	Boys	2015	18	30.30
Bahamas	Boys	2016	18	30.65
Bahamas	Boys	1975	Average	13.34
Bahamas	Boys	1976	Average	13.50
Bahamas	Boys	1977	Average	13.65
Bahamas	Boys	1978	Average	13.82
Bahamas	Boys	1979	Average	13.99
Bahamas	Boys	1980	Average	14.16
Bahamas	Boys	1981	Average	14.35
Bahamas	Boys	1982	Average	14.54
Bahamas	Boys	1983	Average	14.75
Bahamas	Boys	1984	Average	14.96
Bahamas	Boys	1985	Average	15.18
Bahamas	Boys	1986	Average	15.41
Bahamas	Boys	1987	Average	15.64

Bahamas	Boys	1988	Average	15.88
Bahamas	Boys	1989	Average	16.12
Bahamas	Boys	1990	Average	16.37
Bahamas	Boys	1991	Average	16.62
Bahamas	Boys	1992	Average	16.85
Bahamas	Boys	1993	Average	17.09
Bahamas	Boys	1994	Average	17.32
Bahamas	Boys	1995	Average	17.55
Bahamas	Boys	1996	Average	17.78
Bahamas	Boys	1997	Average	18.01
Bahamas	Boys	1998	Average	18.24
Bahamas	Boys	1999	Average	18.47
Bahamas	Boys	2000	Average	18.70
Bahamas	Boys	2001	Average	18.94
Bahamas	Boys	2002	Average	19.17
Bahamas	Boys	2003	Average	19.41
Bahamas	Boys	2004	Average	19.65
Bahamas	Boys	2005	Average	19.89
Bahamas	Boys	2006	Average	20.13
Bahamas	Boys	2007	Average	20.38
Bahamas	Boys	2008	Average	20.63
Bahamas	Boys	2009	Average	20.88
Bahamas	Boys	2010	Average	21.14
Bahamas	Boys	2011	Average	21.40
Bahamas	Boys	2012	Average	21.66
Bahamas	Boys	2013	Average	21.93
Bahamas	Boys	2014	Average	22.20
Bahamas	Boys	2015	Average	22.48
Bahamas	Boys	2016	Average	22.76
Bahamas	Girls	1975	12	7.92
Bahamas	Girls	1976	12	8.03
Bahamas	Girls	1977	12	8.14
Bahamas	Girls	1978	12	8.26
Bahamas	Girls	1979	12	8.38
Bahamas	Girls	1980	12	8.50
Bahamas	Girls	1981	12	8.62
Bahamas	Girls	1982	12	8.75
Bahamas	Girls	1983	12	8.89
Bahamas	Girls	1984	12	9.02
Bahamas	Girls	1985	12	9.16
Bahamas	Girls	1986	12	9.31
Bahamas	Girls	1987	12	9.45
Bahamas	Girls	1988	12	9.59
Bahamas	Girls	1989	12	9.73
Bahamas	Girls	1990	12	9.88
Bahamas	Girls	1991	12	10.01
Bahamas	Girls	1992	12	10.14
Bahamas	Girls	1993	12	10.27
Bahamas	Girls	1994	12	10.39
Bahamas	Girls	1995	12	10.51
Bahamas	Girls	1996	12	10.63
Bahamas	Girls	1997	12	10.74

Bahamas	Girls	1998	12	10.86
Bahamas	Girls	1999	12	10.97
Bahamas	Girls	2000	12	11.08
Bahamas	Girls	2001	12	11.19
Bahamas	Girls	2002	12	11.29
Bahamas	Girls	2003	12	11.40
Bahamas	Girls	2004	12	11.50
Bahamas	Girls	2005	12	11.61
Bahamas	Girls	2006	12	11.71
Bahamas	Girls	2007	12	11.81
Bahamas	Girls	2008	12	11.91
Bahamas	Girls	2009	12	12.01
Bahamas	Girls	2010	12	12.11
Bahamas	Girls	2011	12	12.21
Bahamas	Girls	2012	12	12.32
Bahamas	Girls	2013	12	12.43
Bahamas	Girls	2014	12	12.53
Bahamas	Girls	2015	12	12.64
Bahamas	Girls	2016	12	12.75
Bahamas	Girls	1975	13	9.64
Bahamas	Girls	1976	13	9.77
Bahamas	Girls	1977	13	9.89
Bahamas	Girls	1978	13	10.02
Bahamas	Girls	1979	13	10.16
Bahamas	Girls	1980	13	10.30
Bahamas	Girls	1981	13	10.44
Bahamas	Girls	1982	13	10.59
Bahamas	Girls	1983	13	10.74
Bahamas	Girls	1984	13	10.90
Bahamas	Girls	1985	13	11.06
Bahamas	Girls	1986	13	11.22
Bahamas	Girls	1987	13	11.38
Bahamas	Girls	1988	13	11.54
Bahamas	Girls	1989	13	11.71
Bahamas	Girls	1990	13	11.87
Bahamas	Girls	1991	13	12.02
Bahamas	Girls	1992	13	12.18
Bahamas	Girls	1993	13	12.32
Bahamas	Girls	1994	13	12.46
Bahamas	Girls	1995	13	12.60
Bahamas	Girls	1996	13	12.73
Bahamas	Girls	1997	13	12.86
Bahamas	Girls	1998	13	12.99
Bahamas	Girls	1999	13	13.12
Bahamas	Girls	2000	13	13.25
Bahamas	Girls	2001	13	13.37
Bahamas	Girls	2002	13	13.49
Bahamas	Girls	2003	13	13.61
Bahamas	Girls	2004	13	13.73
Bahamas	Girls	2005	13	13.85
Bahamas	Girls	2006	13	13.97
Bahamas	Girls	2007	13	14.09

Bahamas	Girls	2008	13	14.20
Bahamas	Girls	2009	13	14.32
Bahamas	Girls	2010	13	14.44
Bahamas	Girls	2011	13	14.56
Bahamas	Girls	2012	13	14.68
Bahamas	Girls	2013	13	14.81
Bahamas	Girls	2014	13	14.93
Bahamas	Girls	2015	13	15.06
Bahamas	Girls	2016	13	15.19
Bahamas	Girls	1975	14	11.37
Bahamas	Girls	1976	14	11.51
Bahamas	Girls	1977	14	11.64
Bahamas	Girls	1978	14	11.79
Bahamas	Girls	1979	14	11.93
Bahamas	Girls	1980	14	12.08
Bahamas	Girls	1981	14	12.23
Bahamas	Girls	1982	14	12.39
Bahamas	Girls	1983	14	12.56
Bahamas	Girls	1984	14	12.73
Bahamas	Girls	1985	14	12.90
Bahamas	Girls	1986	14	13.07
Bahamas	Girls	1987	14	13.25
Bahamas	Girls	1988	14	13.43
Bahamas	Girls	1989	14	13.60
Bahamas	Girls	1990	14	13.78
Bahamas	Girls	1991	14	13.95
Bahamas	Girls	1992	14	14.11
Bahamas	Girls	1993	14	14.27
Bahamas	Girls	1994	14	14.42
Bahamas	Girls	1995	14	14.57
Bahamas	Girls	1996	14	14.71
Bahamas	Girls	1997	14	14.86
Bahamas	Girls	1998	14	14.99
Bahamas	Girls	1999	14	15.13
Bahamas	Girls	2000	14	15.27
Bahamas	Girls	2001	14	15.41
Bahamas	Girls	2002	14	15.54
Bahamas	Girls	2003	14	15.67
Bahamas	Girls	2004	14	15.80
Bahamas	Girls	2005	14	15.93
Bahamas	Girls	2006	14	16.05
Bahamas	Girls	2007	14	16.18
Bahamas	Girls	2008	14	16.31
Bahamas	Girls	2009	14	16.44
Bahamas	Girls	2010	14	16.57
Bahamas	Girls	2011	14	16.70
Bahamas	Girls	2012	14	16.83
Bahamas	Girls	2013	14	16.97
Bahamas	Girls	2014	14	17.11
Bahamas	Girls	2015	14	17.26
Bahamas	Girls	2016	14	17.40
Bahamas	Girls	1975	15	12.90

Bahamas	Girls	1976	15	13.05
Bahamas	Girls	1977	15	13.19
Bahamas	Girls	1978	15	13.34
Bahamas	Girls	1979	15	13.49
Bahamas	Girls	1980	15	13.64
Bahamas	Girls	1981	15	13.80
Bahamas	Girls	1982	15	13.97
Bahamas	Girls	1983	15	14.14
Bahamas	Girls	1984	15	14.32
Bahamas	Girls	1985	15	14.50
Bahamas	Girls	1986	15	14.68
Bahamas	Girls	1987	15	14.86
Bahamas	Girls	1988	15	15.05
Bahamas	Girls	1989	15	15.23
Bahamas	Girls	1990	15	15.42
Bahamas	Girls	1991	15	15.59
Bahamas	Girls	1992	15	15.76
Bahamas	Girls	1993	15	15.93
Bahamas	Girls	1994	15	16.08
Bahamas	Girls	1995	15	16.24
Bahamas	Girls	1996	15	16.39
Bahamas	Girls	1997	15	16.54
Bahamas	Girls	1998	15	16.68
Bahamas	Girls	1999	15	16.82
Bahamas	Girls	2000	15	16.97
Bahamas	Girls	2001	15	17.11
Bahamas	Girls	2002	15	17.25
Bahamas	Girls	2003	15	17.38
Bahamas	Girls	2004	15	17.52
Bahamas	Girls	2005	15	17.65
Bahamas	Girls	2006	15	17.78
Bahamas	Girls	2007	15	17.92
Bahamas	Girls	2008	15	18.05
Bahamas	Girls	2009	15	18.18
Bahamas	Girls	2010	15	18.32
Bahamas	Girls	2011	15	18.46
Bahamas	Girls	2012	15	18.60
Bahamas	Girls	2013	15	18.75
Bahamas	Girls	2014	15	18.90
Bahamas	Girls	2015	15	19.05
Bahamas	Girls	2016	15	19.21
Bahamas	Girls	1975	16	14.13
Bahamas	Girls	1976	16	14.28
Bahamas	Girls	1977	16	14.43
Bahamas	Girls	1978	16	14.58
Bahamas	Girls	1979	16	14.73
Bahamas	Girls	1980	16	14.89
Bahamas	Girls	1981	16	15.06
Bahamas	Girls	1982	16	15.23
Bahamas	Girls	1983	16	15.41
Bahamas	Girls	1984	16	15.59
Bahamas	Girls	1985	16	15.77

Bahamas	Girls	1986	16	15.96
Bahamas	Girls	1987	16	16.15
Bahamas	Girls	1988	16	16.34
Bahamas	Girls	1989	16	16.53
Bahamas	Girls	1990	16	16.72
Bahamas	Girls	1991	16	16.90
Bahamas	Girls	1992	16	17.08
Bahamas	Girls	1993	16	17.24
Bahamas	Girls	1994	16	17.41
Bahamas	Girls	1995	16	17.57
Bahamas	Girls	1996	16	17.72
Bahamas	Girls	1997	16	17.87
Bahamas	Girls	1998	16	18.02
Bahamas	Girls	1999	16	18.17
Bahamas	Girls	2000	16	18.32
Bahamas	Girls	2001	16	18.46
Bahamas	Girls	2002	16	18.60
Bahamas	Girls	2003	16	18.75
Bahamas	Girls	2004	16	18.88
Bahamas	Girls	2005	16	19.02
Bahamas	Girls	2006	16	19.16
Bahamas	Girls	2007	16	19.29
Bahamas	Girls	2008	16	19.43
Bahamas	Girls	2009	16	19.57
Bahamas	Girls	2010	16	19.71
Bahamas	Girls	2011	16	19.86
Bahamas	Girls	2012	16	20.01
Bahamas	Girls	2013	16	20.16
Bahamas	Girls	2014	16	20.31
Bahamas	Girls	2015	16	20.47
Bahamas	Girls	2016	16	20.63
Bahamas	Girls	1975	17	15.09
Bahamas	Girls	1976	17	15.24
Bahamas	Girls	1977	17	15.40
Bahamas	Girls	1978	17	15.56
Bahamas	Girls	1979	17	15.72
Bahamas	Girls	1980	17	15.89
Bahamas	Girls	1981	17	16.06
Bahamas	Girls	1982	17	16.24
Bahamas	Girls	1983	17	16.42
Bahamas	Girls	1984	17	16.61
Bahamas	Girls	1985	17	16.81
Bahamas	Girls	1986	17	17.01
Bahamas	Girls	1987	17	17.20
Bahamas	Girls	1988	17	17.40
Bahamas	Girls	1989	17	17.60
Bahamas	Girls	1990	17	17.80
Bahamas	Girls	1991	17	17.99
Bahamas	Girls	1992	17	18.17
Bahamas	Girls	1993	17	18.35
Bahamas	Girls	1994	17	18.52
Bahamas	Girls	1995	17	18.69

Bahamas	Girls	1996	17	18.85
Bahamas	Girls	1997	17	19.01
Bahamas	Girls	1998	17	19.16
Bahamas	Girls	1999	17	19.32
Bahamas	Girls	2000	17	19.47
Bahamas	Girls	2001	17	19.62
Bahamas	Girls	2002	17	19.77
Bahamas	Girls	2003	17	19.92
Bahamas	Girls	2004	17	20.06
Bahamas	Girls	2005	17	20.21
Bahamas	Girls	2006	17	20.35
Bahamas	Girls	2007	17	20.49
Bahamas	Girls	2008	17	20.64
Bahamas	Girls	2009	17	20.78
Bahamas	Girls	2010	17	20.93
Bahamas	Girls	2011	17	21.08
Bahamas	Girls	2012	17	21.23
Bahamas	Girls	2013	17	21.39
Bahamas	Girls	2014	17	21.56
Bahamas	Girls	2015	17	21.72
Bahamas	Girls	2016	17	21.89
Bahamas	Girls	1975	18	15.95
Bahamas	Girls	1976	18	16.12
Bahamas	Girls	1977	18	16.29
Bahamas	Girls	1978	18	16.46
Bahamas	Girls	1979	18	16.64
Bahamas	Girls	1980	18	16.82
Bahamas	Girls	1981	18	17.01
Bahamas	Girls	1982	18	17.21
Bahamas	Girls	1983	18	17.41
Bahamas	Girls	1984	18	17.62
Bahamas	Girls	1985	18	17.83
Bahamas	Girls	1986	18	18.04
Bahamas	Girls	1987	18	18.25
Bahamas	Girls	1988	18	18.47
Bahamas	Girls	1989	18	18.69
Bahamas	Girls	1990	18	18.90
Bahamas	Girls	1991	18	19.11
Bahamas	Girls	1992	18	19.30
Bahamas	Girls	1993	18	19.49
Bahamas	Girls	1994	18	19.68
Bahamas	Girls	1995	18	19.86
Bahamas	Girls	1996	18	20.03
Bahamas	Girls	1997	18	20.21
Bahamas	Girls	1998	18	20.37
Bahamas	Girls	1999	18	20.54
Bahamas	Girls	2000	18	20.71
Bahamas	Girls	2001	18	20.87
Bahamas	Girls	2002	18	21.03
Bahamas	Girls	2003	18	21.19
Bahamas	Girls	2004	18	21.34
Bahamas	Girls	2005	18	21.50

Bahamas	Girls	2006	18	21.65
Bahamas	Girls	2007	18	21.80
Bahamas	Girls	2008	18	21.96
Bahamas	Girls	2009	18	22.11
Bahamas	Girls	2010	18	22.27
Bahamas	Girls	2011	18	22.43
Bahamas	Girls	2012	18	22.59
Bahamas	Girls	2013	18	22.76
Bahamas	Girls	2014	18	22.93
Bahamas	Girls	2015	18	23.10
Bahamas	Girls	2016	18	23.28
Bahamas	Girls	1975	Average	12.43
Bahamas	Girls	1976	Average	12.57
Bahamas	Girls	1977	Average	12.71
Bahamas	Girls	1978	Average	12.86
Bahamas	Girls	1979	Average	13.01
Bahamas	Girls	1980	Average	13.16
Bahamas	Girls	1981	Average	13.32
Bahamas	Girls	1982	Average	13.48
Bahamas	Girls	1983	Average	13.65
Bahamas	Girls	1984	Average	13.83
Bahamas	Girls	1985	Average	14.00
Bahamas	Girls	1986	Average	14.18
Bahamas	Girls	1987	Average	14.36
Bahamas	Girls	1988	Average	14.55
Bahamas	Girls	1989	Average	14.73
Bahamas	Girls	1990	Average	14.91
Bahamas	Girls	1991	Average	15.08
Bahamas	Girls	1992	Average	15.25
Bahamas	Girls	1993	Average	15.41
Bahamas	Girls	1994	Average	15.57
Bahamas	Girls	1995	Average	15.72
Bahamas	Girls	1996	Average	15.87
Bahamas	Girls	1997	Average	16.01
Bahamas	Girls	1998	Average	16.15
Bahamas	Girls	1999	Average	16.30
Bahamas	Girls	2000	Average	16.44
Bahamas	Girls	2001	Average	16.58
Bahamas	Girls	2002	Average	16.71
Bahamas	Girls	2003	Average	16.85
Bahamas	Girls	2004	Average	16.98
Bahamas	Girls	2005	Average	17.11
Bahamas	Girls	2006	Average	17.24
Bahamas	Girls	2007	Average	17.37
Bahamas	Girls	2008	Average	17.50
Bahamas	Girls	2009	Average	17.63
Bahamas	Girls	2010	Average	17.76
Bahamas	Girls	2011	Average	17.90
Bahamas	Girls	2012	Average	18.04
Bahamas	Girls	2013	Average	18.18
Bahamas	Girls	2014	Average	18.33
Bahamas	Girls	2015	Average	18.47

Bahamas	Girls	2016	Average	18.62
Bahrain	Boys	1975	12	8.18
Bahrain	Boys	1976	12	8.26
Bahrain	Boys	1977	12	8.34
Bahrain	Boys	1978	12	8.43
Bahrain	Boys	1979	12	8.53
Bahrain	Boys	1980	12	8.63
Bahrain	Boys	1981	12	8.74
Bahrain	Boys	1982	12	8.84
Bahrain	Boys	1983	12	8.96
Bahrain	Boys	1984	12	9.07
Bahrain	Boys	1985	12	9.20
Bahrain	Boys	1986	12	9.32
Bahrain	Boys	1987	12	9.45
Bahrain	Boys	1988	12	9.58
Bahrain	Boys	1989	12	9.71
Bahrain	Boys	1990	12	9.85
Bahrain	Boys	1991	12	9.99
Bahrain	Boys	1992	12	10.13
Bahrain	Boys	1993	12	10.28
Bahrain	Boys	1994	12	10.42
Bahrain	Boys	1995	12	10.57
Bahrain	Boys	1996	12	10.72
Bahrain	Boys	1997	12	10.87
Bahrain	Boys	1998	12	11.02
Bahrain	Boys	1999	12	11.18
Bahrain	Boys	2000	12	11.35
Bahrain	Boys	2001	12	11.51
Bahrain	Boys	2002	12	11.68
Bahrain	Boys	2003	12	11.85
Bahrain	Boys	2004	12	12.02
Bahrain	Boys	2005	12	12.19
Bahrain	Boys	2006	12	12.36
Bahrain	Boys	2007	12	12.54
Bahrain	Boys	2008	12	12.71
Bahrain	Boys	2009	12	12.88
Bahrain	Boys	2010	12	13.05
Bahrain	Boys	2011	12	13.22
Bahrain	Boys	2012	12	13.39
Bahrain	Boys	2013	12	13.57
Bahrain	Boys	2014	12	13.74
Bahrain	Boys	2015	12	13.92
Bahrain	Boys	2016	12	14.09
Bahrain	Boys	1975	13	9.95
Bahrain	Boys	1976	13	10.04
Bahrain	Boys	1977	13	10.14
Bahrain	Boys	1978	13	10.25
Bahrain	Boys	1979	13	10.35
Bahrain	Boys	1980	13	10.47
Bahrain	Boys	1981	13	10.59
Bahrain	Boys	1982	13	10.72
Bahrain	Boys	1983	13	10.85

Bahrain	Boys	1984	13	10.99
Bahrain	Boys	1985	13	11.13
Bahrain	Boys	1986	13	11.27
Bahrain	Boys	1987	13	11.42
Bahrain	Boys	1988	13	11.58
Bahrain	Boys	1989	13	11.73
Bahrain	Boys	1990	13	11.90
Bahrain	Boys	1991	13	12.06
Bahrain	Boys	1992	13	12.23
Bahrain	Boys	1993	13	12.40
Bahrain	Boys	1994	13	12.57
Bahrain	Boys	1995	13	12.75
Bahrain	Boys	1996	13	12.92
Bahrain	Boys	1997	13	13.10
Bahrain	Boys	1998	13	13.29
Bahrain	Boys	1999	13	13.48
Bahrain	Boys	2000	13	13.68
Bahrain	Boys	2001	13	13.87
Bahrain	Boys	2002	13	14.08
Bahrain	Boys	2003	13	14.28
Bahrain	Boys	2004	13	14.49
Bahrain	Boys	2005	13	14.70
Bahrain	Boys	2006	13	14.91
Bahrain	Boys	2007	13	15.12
Bahrain	Boys	2008	13	15.33
Bahrain	Boys	2009	13	15.55
Bahrain	Boys	2010	13	15.76
Bahrain	Boys	2011	13	15.97
Bahrain	Boys	2012	13	16.19
Bahrain	Boys	2013	13	16.40
Bahrain	Boys	2014	13	16.62
Bahrain	Boys	2015	13	16.84
Bahrain	Boys	2016	13	17.07
Bahrain	Boys	1975	14	11.91
Bahrain	Boys	1976	14	12.01
Bahrain	Boys	1977	14	12.12
Bahrain	Boys	1978	14	12.23
Bahrain	Boys	1979	14	12.35
Bahrain	Boys	1980	14	12.48
Bahrain	Boys	1981	14	12.61
Bahrain	Boys	1982	14	12.75
Bahrain	Boys	1983	14	12.89
Bahrain	Boys	1984	14	13.04
Bahrain	Boys	1985	14	13.20
Bahrain	Boys	1986	14	13.36
Bahrain	Boys	1987	14	13.52
Bahrain	Boys	1988	14	13.69
Bahrain	Boys	1989	14	13.86
Bahrain	Boys	1990	14	14.05
Bahrain	Boys	1991	14	14.23
Bahrain	Boys	1992	14	14.41
Bahrain	Boys	1993	14	14.60

Bahrain	Boys	1994	14	14.79
Bahrain	Boys	1995	14	14.99
Bahrain	Boys	1996	14	15.18
Bahrain	Boys	1997	14	15.39
Bahrain	Boys	1998	14	15.60
Bahrain	Boys	1999	14	15.81
Bahrain	Boys	2000	14	16.03
Bahrain	Boys	2001	14	16.26
Bahrain	Boys	2002	14	16.49
Bahrain	Boys	2003	14	16.72
Bahrain	Boys	2004	14	16.96
Bahrain	Boys	2005	14	17.20
Bahrain	Boys	2006	14	17.44
Bahrain	Boys	2007	14	17.68
Bahrain	Boys	2008	14	17.92
Bahrain	Boys	2009	14	18.17
Bahrain	Boys	2010	14	18.42
Bahrain	Boys	2011	14	18.67
Bahrain	Boys	2012	14	18.92
Bahrain	Boys	2013	14	19.17
Bahrain	Boys	2014	14	19.43
Bahrain	Boys	2015	14	19.70
Bahrain	Boys	2016	14	19.96
Bahrain	Boys	1975	15	13.97
Bahrain	Boys	1976	15	14.07
Bahrain	Boys	1977	15	14.18
Bahrain	Boys	1978	15	14.30
Bahrain	Boys	1979	15	14.42
Bahrain	Boys	1980	15	14.55
Bahrain	Boys	1981	15	14.69
Bahrain	Boys	1982	15	14.83
Bahrain	Boys	1983	15	14.98
Bahrain	Boys	1984	15	15.14
Bahrain	Boys	1985	15	15.30
Bahrain	Boys	1986	15	15.46
Bahrain	Boys	1987	15	15.64
Bahrain	Boys	1988	15	15.81
Bahrain	Boys	1989	15	15.99
Bahrain	Boys	1990	15	16.18
Bahrain	Boys	1991	15	16.38
Bahrain	Boys	1992	15	16.57
Bahrain	Boys	1993	15	16.77
Bahrain	Boys	1994	15	16.97
Bahrain	Boys	1995	15	17.17
Bahrain	Boys	1996	15	17.38
Bahrain	Boys	1997	15	17.60
Bahrain	Boys	1998	15	17.82
Bahrain	Boys	1999	15	18.04
Bahrain	Boys	2000	15	18.28
Bahrain	Boys	2001	15	18.52
Bahrain	Boys	2002	15	18.77
Bahrain	Boys	2003	15	19.02

Bahrain	Boys	2004	15	19.27
Bahrain	Boys	2005	15	19.53
Bahrain	Boys	2006	15	19.79
Bahrain	Boys	2007	15	20.05
Bahrain	Boys	2008	15	20.32
Bahrain	Boys	2009	15	20.59
Bahrain	Boys	2010	15	20.86
Bahrain	Boys	2011	15	21.13
Bahrain	Boys	2012	15	21.41
Bahrain	Boys	2013	15	21.69
Bahrain	Boys	2014	15	21.98
Bahrain	Boys	2015	15	22.27
Bahrain	Boys	2016	15	22.57
Bahrain	Boys	1975	16	16.09
Bahrain	Boys	1976	16	16.19
Bahrain	Boys	1977	16	16.30
Bahrain	Boys	1978	16	16.42
Bahrain	Boys	1979	16	16.55
Bahrain	Boys	1980	16	16.68
Bahrain	Boys	1981	16	16.82
Bahrain	Boys	1982	16	16.96
Bahrain	Boys	1983	16	17.11
Bahrain	Boys	1984	16	17.27
Bahrain	Boys	1985	16	17.43
Bahrain	Boys	1986	16	17.60
Bahrain	Boys	1987	16	17.78
Bahrain	Boys	1988	16	17.96
Bahrain	Boys	1989	16	18.14
Bahrain	Boys	1990	16	18.33
Bahrain	Boys	1991	16	18.53
Bahrain	Boys	1992	16	18.73
Bahrain	Boys	1993	16	18.93
Bahrain	Boys	1994	16	19.13
Bahrain	Boys	1995	16	19.34
Bahrain	Boys	1996	16	19.55
Bahrain	Boys	1997	16	19.77
Bahrain	Boys	1998	16	20.00
Bahrain	Boys	1999	16	20.23
Bahrain	Boys	2000	16	20.47
Bahrain	Boys	2001	16	20.72
Bahrain	Boys	2002	16	20.98
Bahrain	Boys	2003	16	21.24
Bahrain	Boys	2004	16	21.50
Bahrain	Boys	2005	16	21.77
Bahrain	Boys	2006	16	22.04
Bahrain	Boys	2007	16	22.32
Bahrain	Boys	2008	16	22.59
Bahrain	Boys	2009	16	22.87
Bahrain	Boys	2010	16	23.16
Bahrain	Boys	2011	16	23.45
Bahrain	Boys	2012	16	23.74
Bahrain	Boys	2013	16	24.04

Bahrain	Boys	2014	16	24.34
Bahrain	Boys	2015	16	24.66
Bahrain	Boys	2016	16	24.97
Bahrain	Boys	1975	17	18.27
Bahrain	Boys	1976	17	18.38
Bahrain	Boys	1977	17	18.49
Bahrain	Boys	1978	17	18.61
Bahrain	Boys	1979	17	18.74
Bahrain	Boys	1980	17	18.87
Bahrain	Boys	1981	17	19.01
Bahrain	Boys	1982	17	19.16
Bahrain	Boys	1983	17	19.31
Bahrain	Boys	1984	17	19.47
Bahrain	Boys	1985	17	19.64
Bahrain	Boys	1986	17	19.81
Bahrain	Boys	1987	17	19.99
Bahrain	Boys	1988	17	20.17
Bahrain	Boys	1989	17	20.36
Bahrain	Boys	1990	17	20.55
Bahrain	Boys	1991	17	20.75
Bahrain	Boys	1992	17	20.95
Bahrain	Boys	1993	17	21.16
Bahrain	Boys	1994	17	21.36
Bahrain	Boys	1995	17	21.58
Bahrain	Boys	1996	17	21.79
Bahrain	Boys	1997	17	22.02
Bahrain	Boys	1998	17	22.25
Bahrain	Boys	1999	17	22.49
Bahrain	Boys	2000	17	22.73
Bahrain	Boys	2001	17	22.98
Bahrain	Boys	2002	17	23.24
Bahrain	Boys	2003	17	23.51
Bahrain	Boys	2004	17	23.78
Bahrain	Boys	2005	17	24.06
Bahrain	Boys	2006	17	24.34
Bahrain	Boys	2007	17	24.62
Bahrain	Boys	2008	17	24.90
Bahrain	Boys	2009	17	25.19
Bahrain	Boys	2010	17	25.49
Bahrain	Boys	2011	17	25.79
Bahrain	Boys	2012	17	26.09
Bahrain	Boys	2013	17	26.40
Bahrain	Boys	2014	17	26.72
Bahrain	Boys	2015	17	27.04
Bahrain	Boys	2016	17	27.37
Bahrain	Boys	1975	18	20.56
Bahrain	Boys	1976	18	20.67
Bahrain	Boys	1977	18	20.79
Bahrain	Boys	1978	18	20.92
Bahrain	Boys	1979	18	21.05
Bahrain	Boys	1980	18	21.19
Bahrain	Boys	1981	18	21.34

Bahrain	Boys	1982	18	21.49
Bahrain	Boys	1983	18	21.65
Bahrain	Boys	1984	18	21.82
Bahrain	Boys	1985	18	21.99
Bahrain	Boys	1986	18	22.16
Bahrain	Boys	1987	18	22.35
Bahrain	Boys	1988	18	22.54
Bahrain	Boys	1989	18	22.73
Bahrain	Boys	1990	18	22.93
Bahrain	Boys	1991	18	23.14
Bahrain	Boys	1992	18	23.35
Bahrain	Boys	1993	18	23.56
Bahrain	Boys	1994	18	23.78
Bahrain	Boys	1995	18	24.00
Bahrain	Boys	1996	18	24.22
Bahrain	Boys	1997	18	24.45
Bahrain	Boys	1998	18	24.69
Bahrain	Boys	1999	18	24.94
Bahrain	Boys	2000	18	25.20
Bahrain	Boys	2001	18	25.46
Bahrain	Boys	2002	18	25.73
Bahrain	Boys	2003	18	26.00
Bahrain	Boys	2004	18	26.29
Bahrain	Boys	2005	18	26.57
Bahrain	Boys	2006	18	26.86
Bahrain	Boys	2007	18	27.15
Bahrain	Boys	2008	18	27.45
Bahrain	Boys	2009	18	27.75
Bahrain	Boys	2010	18	28.05
Bahrain	Boys	2011	18	28.36
Bahrain	Boys	2012	18	28.68
Bahrain	Boys	2013	18	29.00
Bahrain	Boys	2014	18	29.33
Bahrain	Boys	2015	18	29.66
Bahrain	Boys	2016	18	30.01
Bahrain	Boys	1975	Average	14.13
Bahrain	Boys	1976	Average	14.23
Bahrain	Boys	1977	Average	14.34
Bahrain	Boys	1978	Average	14.45
Bahrain	Boys	1979	Average	14.57
Bahrain	Boys	1980	Average	14.70
Bahrain	Boys	1981	Average	14.83
Bahrain	Boys	1982	Average	14.96
Bahrain	Boys	1983	Average	15.11
Bahrain	Boys	1984	Average	15.26
Bahrain	Boys	1985	Average	15.41
Bahrain	Boys	1986	Average	15.57
Bahrain	Boys	1987	Average	15.73
Bahrain	Boys	1988	Average	15.90
Bahrain	Boys	1989	Average	16.08
Bahrain	Boys	1990	Average	16.26
Bahrain	Boys	1991	Average	16.44

Bahrain	Boys	1992	Average	16.62
Bahrain	Boys	1993	Average	16.81
Bahrain	Boys	1994	Average	17.00
Bahrain	Boys	1995	Average	17.20
Bahrain	Boys	1996	Average	17.40
Bahrain	Boys	1997	Average	17.60
Bahrain	Boys	1998	Average	17.81
Bahrain	Boys	1999	Average	18.03
Bahrain	Boys	2000	Average	18.25
Bahrain	Boys	2001	Average	18.48
Bahrain	Boys	2002	Average	18.71
Bahrain	Boys	2003	Average	18.95
Bahrain	Boys	2004	Average	19.19
Bahrain	Boys	2005	Average	19.43
Bahrain	Boys	2006	Average	19.68
Bahrain	Boys	2007	Average	19.93
Bahrain	Boys	2008	Average	20.18
Bahrain	Boys	2009	Average	20.43
Bahrain	Boys	2010	Average	20.68
Bahrain	Boys	2011	Average	20.94
Bahrain	Boys	2012	Average	21.20
Bahrain	Boys	2013	Average	21.47
Bahrain	Boys	2014	Average	21.74
Bahrain	Boys	2015	Average	22.01
Bahrain	Boys	2016	Average	22.29
Bahrain	Girls	1975	12	8.70
Bahrain	Girls	1976	12	8.77
Bahrain	Girls	1977	12	8.84
Bahrain	Girls	1978	12	8.91
Bahrain	Girls	1979	12	8.99
Bahrain	Girls	1980	12	9.06
Bahrain	Girls	1981	12	9.14
Bahrain	Girls	1982	12	9.22
Bahrain	Girls	1983	12	9.31
Bahrain	Girls	1984	12	9.39
Bahrain	Girls	1985	12	9.47
Bahrain	Girls	1986	12	9.56
Bahrain	Girls	1987	12	9.65
Bahrain	Girls	1988	12	9.74
Bahrain	Girls	1989	12	9.83
Bahrain	Girls	1990	12	9.92
Bahrain	Girls	1991	12	10.01
Bahrain	Girls	1992	12	10.10
Bahrain	Girls	1993	12	10.19
Bahrain	Girls	1994	12	10.27
Bahrain	Girls	1995	12	10.36
Bahrain	Girls	1996	12	10.45
Bahrain	Girls	1997	12	10.54
Bahrain	Girls	1998	12	10.62
Bahrain	Girls	1999	12	10.71
Bahrain	Girls	2000	12	10.80
Bahrain	Girls	2001	12	10.89

Bahrain	Girls	2002	12	10.97
Bahrain	Girls	2003	12	11.06
Bahrain	Girls	2004	12	11.14
Bahrain	Girls	2005	12	11.22
Bahrain	Girls	2006	12	11.30
Bahrain	Girls	2007	12	11.38
Bahrain	Girls	2008	12	11.46
Bahrain	Girls	2009	12	11.54
Bahrain	Girls	2010	12	11.62
Bahrain	Girls	2011	12	11.70
Bahrain	Girls	2012	12	11.78
Bahrain	Girls	2013	12	11.86
Bahrain	Girls	2014	12	11.94
Bahrain	Girls	2015	12	12.03
Bahrain	Girls	2016	12	12.11
Bahrain	Girls	1975	13	10.60
Bahrain	Girls	1976	13	10.68
Bahrain	Girls	1977	13	10.76
Bahrain	Girls	1978	13	10.85
Bahrain	Girls	1979	13	10.93
Bahrain	Girls	1980	13	11.02
Bahrain	Girls	1981	13	11.11
Bahrain	Girls	1982	13	11.20
Bahrain	Girls	1983	13	11.30
Bahrain	Girls	1984	13	11.39
Bahrain	Girls	1985	13	11.49
Bahrain	Girls	1986	13	11.59
Bahrain	Girls	1987	13	11.69
Bahrain	Girls	1988	13	11.80
Bahrain	Girls	1989	13	11.90
Bahrain	Girls	1990	13	12.00
Bahrain	Girls	1991	13	12.10
Bahrain	Girls	1992	13	12.21
Bahrain	Girls	1993	13	12.31
Bahrain	Girls	1994	13	12.41
Bahrain	Girls	1995	13	12.51
Bahrain	Girls	1996	13	12.61
Bahrain	Girls	1997	13	12.71
Bahrain	Girls	1998	13	12.81
Bahrain	Girls	1999	13	12.91
Bahrain	Girls	2000	13	13.01
Bahrain	Girls	2001	13	13.11
Bahrain	Girls	2002	13	13.21
Bahrain	Girls	2003	13	13.31
Bahrain	Girls	2004	13	13.41
Bahrain	Girls	2005	13	13.50
Bahrain	Girls	2006	13	13.59
Bahrain	Girls	2007	13	13.68
Bahrain	Girls	2008	13	13.78
Bahrain	Girls	2009	13	13.87
Bahrain	Girls	2010	13	13.96
Bahrain	Girls	2011	13	14.05

Bahrain	Girls	2012	13	14.15
Bahrain	Girls	2013	13	14.24
Bahrain	Girls	2014	13	14.34
Bahrain	Girls	2015	13	14.44
Bahrain	Girls	2016	13	14.54
Bahrain	Girls	1975	14	12.49
Bahrain	Girls	1976	14	12.58
Bahrain	Girls	1977	14	12.67
Bahrain	Girls	1978	14	12.76
Bahrain	Girls	1979	14	12.86
Bahrain	Girls	1980	14	12.95
Bahrain	Girls	1981	14	13.05
Bahrain	Girls	1982	14	13.15
Bahrain	Girls	1983	14	13.25
Bahrain	Girls	1984	14	13.36
Bahrain	Girls	1985	14	13.46
Bahrain	Girls	1986	14	13.57
Bahrain	Girls	1987	14	13.68
Bahrain	Girls	1988	14	13.79
Bahrain	Girls	1989	14	13.90
Bahrain	Girls	1990	14	14.02
Bahrain	Girls	1991	14	14.13
Bahrain	Girls	1992	14	14.24
Bahrain	Girls	1993	14	14.35
Bahrain	Girls	1994	14	14.46
Bahrain	Girls	1995	14	14.57
Bahrain	Girls	1996	14	14.68
Bahrain	Girls	1997	14	14.79
Bahrain	Girls	1998	14	14.90
Bahrain	Girls	1999	14	15.01
Bahrain	Girls	2000	14	15.11
Bahrain	Girls	2001	14	15.22
Bahrain	Girls	2002	14	15.33
Bahrain	Girls	2003	14	15.44
Bahrain	Girls	2004	14	15.54
Bahrain	Girls	2005	14	15.64
Bahrain	Girls	2006	14	15.74
Bahrain	Girls	2007	14	15.84
Bahrain	Girls	2008	14	15.94
Bahrain	Girls	2009	14	16.04
Bahrain	Girls	2010	14	16.15
Bahrain	Girls	2011	14	16.25
Bahrain	Girls	2012	14	16.35
Bahrain	Girls	2013	14	16.46
Bahrain	Girls	2014	14	16.57
Bahrain	Girls	2015	14	16.68
Bahrain	Girls	2016	14	16.79
Bahrain	Girls	1975	15	14.16
Bahrain	Girls	1976	15	14.25
Bahrain	Girls	1977	15	14.35
Bahrain	Girls	1978	15	14.44
Bahrain	Girls	1979	15	14.54

Bahrain	Girls	1980	15	14.64
Bahrain	Girls	1981	15	14.74
Bahrain	Girls	1982	15	14.85
Bahrain	Girls	1983	15	14.95
Bahrain	Girls	1984	15	15.06
Bahrain	Girls	1985	15	15.18
Bahrain	Girls	1986	15	15.29
Bahrain	Girls	1987	15	15.40
Bahrain	Girls	1988	15	15.52
Bahrain	Girls	1989	15	15.64
Bahrain	Girls	1990	15	15.76
Bahrain	Girls	1991	15	15.87
Bahrain	Girls	1992	15	15.99
Bahrain	Girls	1993	15	16.11
Bahrain	Girls	1994	15	16.22
Bahrain	Girls	1995	15	16.34
Bahrain	Girls	1996	15	16.45
Bahrain	Girls	1997	15	16.56
Bahrain	Girls	1998	15	16.68
Bahrain	Girls	1999	15	16.79
Bahrain	Girls	2000	15	16.90
Bahrain	Girls	2001	15	17.02
Bahrain	Girls	2002	15	17.13
Bahrain	Girls	2003	15	17.24
Bahrain	Girls	2004	15	17.35
Bahrain	Girls	2005	15	17.46
Bahrain	Girls	2006	15	17.56
Bahrain	Girls	2007	15	17.67
Bahrain	Girls	2008	15	17.77
Bahrain	Girls	2009	15	17.88
Bahrain	Girls	2010	15	17.99
Bahrain	Girls	2011	15	18.10
Bahrain	Girls	2012	15	18.21
Bahrain	Girls	2013	15	18.32
Bahrain	Girls	2014	15	18.44
Bahrain	Girls	2015	15	18.55
Bahrain	Girls	2016	15	18.67
Bahrain	Girls	1975	16	15.50
Bahrain	Girls	1976	16	15.60
Bahrain	Girls	1977	16	15.70
Bahrain	Girls	1978	16	15.80
Bahrain	Girls	1979	16	15.90
Bahrain	Girls	1980	16	16.00
Bahrain	Girls	1981	16	16.11
Bahrain	Girls	1982	16	16.22
Bahrain	Girls	1983	16	16.33
Bahrain	Girls	1984	16	16.44
Bahrain	Girls	1985	16	16.56
Bahrain	Girls	1986	16	16.67
Bahrain	Girls	1987	16	16.79
Bahrain	Girls	1988	16	16.91
Bahrain	Girls	1989	16	17.04

Bahrain	Girls	1990	16	17.16
Bahrain	Girls	1991	16	17.28
Bahrain	Girls	1992	16	17.40
Bahrain	Girls	1993	16	17.52
Bahrain	Girls	1994	16	17.64
Bahrain	Girls	1995	16	17.76
Bahrain	Girls	1996	16	17.88
Bahrain	Girls	1997	16	18.00
Bahrain	Girls	1998	16	18.11
Bahrain	Girls	1999	16	18.23
Bahrain	Girls	2000	16	18.35
Bahrain	Girls	2001	16	18.47
Bahrain	Girls	2002	16	18.58
Bahrain	Girls	2003	16	18.70
Bahrain	Girls	2004	16	18.81
Bahrain	Girls	2005	16	18.92
Bahrain	Girls	2006	16	19.03
Bahrain	Girls	2007	16	19.14
Bahrain	Girls	2008	16	19.26
Bahrain	Girls	2009	16	19.37
Bahrain	Girls	2010	16	19.48
Bahrain	Girls	2011	16	19.59
Bahrain	Girls	2012	16	19.71
Bahrain	Girls	2013	16	19.83
Bahrain	Girls	2014	16	19.95
Bahrain	Girls	2015	16	20.07
Bahrain	Girls	2016	16	20.20
Bahrain	Girls	1975	17	16.59
Bahrain	Girls	1976	17	16.69
Bahrain	Girls	1977	17	16.80
Bahrain	Girls	1978	17	16.90
Bahrain	Girls	1979	17	17.01
Bahrain	Girls	1980	17	17.12
Bahrain	Girls	1981	17	17.23
Bahrain	Girls	1982	17	17.35
Bahrain	Girls	1983	17	17.46
Bahrain	Girls	1984	17	17.58
Bahrain	Girls	1985	17	17.71
Bahrain	Girls	1986	17	17.83
Bahrain	Girls	1987	17	17.96
Bahrain	Girls	1988	17	18.09
Bahrain	Girls	1989	17	18.21
Bahrain	Girls	1990	17	18.34
Bahrain	Girls	1991	17	18.47
Bahrain	Girls	1992	17	18.60
Bahrain	Girls	1993	17	18.73
Bahrain	Girls	1994	17	18.86
Bahrain	Girls	1995	17	18.98
Bahrain	Girls	1996	17	19.11
Bahrain	Girls	1997	17	19.23
Bahrain	Girls	1998	17	19.35
Bahrain	Girls	1999	17	19.48

Bahrain	Girls	2000	17	19.60
Bahrain	Girls	2001	17	19.73
Bahrain	Girls	2002	17	19.85
Bahrain	Girls	2003	17	19.97
Bahrain	Girls	2004	17	20.09
Bahrain	Girls	2005	17	20.21
Bahrain	Girls	2006	17	20.33
Bahrain	Girls	2007	17	20.45
Bahrain	Girls	2008	17	20.56
Bahrain	Girls	2009	17	20.68
Bahrain	Girls	2010	17	20.80
Bahrain	Girls	2011	17	20.92
Bahrain	Girls	2012	17	21.05
Bahrain	Girls	2013	17	21.17
Bahrain	Girls	2014	17	21.30
Bahrain	Girls	2015	17	21.43
Bahrain	Girls	2016	17	21.56
Bahrain	Girls	1975	18	17.64
Bahrain	Girls	1976	18	17.75
Bahrain	Girls	1977	18	17.86
Bahrain	Girls	1978	18	17.98
Bahrain	Girls	1979	18	18.10
Bahrain	Girls	1980	18	18.22
Bahrain	Girls	1981	18	18.35
Bahrain	Girls	1982	18	18.47
Bahrain	Girls	1983	18	18.60
Bahrain	Girls	1984	18	18.73
Bahrain	Girls	1985	18	18.87
Bahrain	Girls	1986	18	19.01
Bahrain	Girls	1987	18	19.14
Bahrain	Girls	1988	18	19.28
Bahrain	Girls	1989	18	19.43
Bahrain	Girls	1990	18	19.57
Bahrain	Girls	1991	18	19.71
Bahrain	Girls	1992	18	19.85
Bahrain	Girls	1993	18	19.99
Bahrain	Girls	1994	18	20.13
Bahrain	Girls	1995	18	20.27
Bahrain	Girls	1996	18	20.40
Bahrain	Girls	1997	18	20.54
Bahrain	Girls	1998	18	20.68
Bahrain	Girls	1999	18	20.81
Bahrain	Girls	2000	18	20.95
Bahrain	Girls	2001	18	21.08
Bahrain	Girls	2002	18	21.22
Bahrain	Girls	2003	18	21.35
Bahrain	Girls	2004	18	21.48
Bahrain	Girls	2005	18	21.61
Bahrain	Girls	2006	18	21.74
Bahrain	Girls	2007	18	21.87
Bahrain	Girls	2008	18	22.00
Bahrain	Girls	2009	18	22.13

Bahrain	Girls	2010	18	22.26
Bahrain	Girls	2011	18	22.39
Bahrain	Girls	2012	18	22.52
Bahrain	Girls	2013	18	22.66
Bahrain	Girls	2014	18	22.79
Bahrain	Girls	2015	18	22.93
Bahrain	Girls	2016	18	23.08
Bahrain	Girls	1975	Average	13.67
Bahrain	Girls	1976	Average	13.76
Bahrain	Girls	1977	Average	13.85
Bahrain	Girls	1978	Average	13.95
Bahrain	Girls	1979	Average	14.05
Bahrain	Girls	1980	Average	14.15
Bahrain	Girls	1981	Average	14.25
Bahrain	Girls	1982	Average	14.35
Bahrain	Girls	1983	Average	14.46
Bahrain	Girls	1984	Average	14.57
Bahrain	Girls	1985	Average	14.68
Bahrain	Girls	1986	Average	14.79
Bahrain	Girls	1987	Average	14.90
Bahrain	Girls	1988	Average	15.02
Bahrain	Girls	1989	Average	15.13
Bahrain	Girls	1990	Average	15.25
Bahrain	Girls	1991	Average	15.37
Bahrain	Girls	1992	Average	15.48
Bahrain	Girls	1993	Average	15.60
Bahrain	Girls	1994	Average	15.71
Bahrain	Girls	1995	Average	15.83
Bahrain	Girls	1996	Average	15.94
Bahrain	Girls	1997	Average	16.05
Bahrain	Girls	1998	Average	16.17
Bahrain	Girls	1999	Average	16.28
Bahrain	Girls	2000	Average	16.39
Bahrain	Girls	2001	Average	16.50
Bahrain	Girls	2002	Average	16.61
Bahrain	Girls	2003	Average	16.72
Bahrain	Girls	2004	Average	16.83
Bahrain	Girls	2005	Average	16.94
Bahrain	Girls	2006	Average	17.04
Bahrain	Girls	2007	Average	17.15
Bahrain	Girls	2008	Average	17.25
Bahrain	Girls	2009	Average	17.36
Bahrain	Girls	2010	Average	17.46
Bahrain	Girls	2011	Average	17.57
Bahrain	Girls	2012	Average	17.68
Bahrain	Girls	2013	Average	17.79
Bahrain	Girls	2014	Average	17.90
Bahrain	Girls	2015	Average	18.02
Bahrain	Girls	2016	Average	18.14
Bangladesh	Boys	1975	12	3.28
Bangladesh	Boys	1976	12	3.30
Bangladesh	Boys	1977	12	3.32

Bangladesh	Boys	1978	12	3.33
Bangladesh	Boys	1979	12	3.35
Bangladesh	Boys	1980	12	3.38
Bangladesh	Boys	1981	12	3.40
Bangladesh	Boys	1982	12	3.42
Bangladesh	Boys	1983	12	3.45
Bangladesh	Boys	1984	12	3.48
Bangladesh	Boys	1985	12	3.51
Bangladesh	Boys	1986	12	3.54
Bangladesh	Boys	1987	12	3.57
Bangladesh	Boys	1988	12	3.60
Bangladesh	Boys	1989	12	3.64
Bangladesh	Boys	1990	12	3.68
Bangladesh	Boys	1991	12	3.72
Bangladesh	Boys	1992	12	3.76
Bangladesh	Boys	1993	12	3.80
Bangladesh	Boys	1994	12	3.85
Bangladesh	Boys	1995	12	3.90
Bangladesh	Boys	1996	12	3.95
Bangladesh	Boys	1997	12	4.01
Bangladesh	Boys	1998	12	4.07
Bangladesh	Boys	1999	12	4.13
Bangladesh	Boys	2000	12	4.20
Bangladesh	Boys	2001	12	4.27
Bangladesh	Boys	2002	12	4.35
Bangladesh	Boys	2003	12	4.43
Bangladesh	Boys	2004	12	4.51
Bangladesh	Boys	2005	12	4.61
Bangladesh	Boys	2006	12	4.70
Bangladesh	Boys	2007	12	4.81
Bangladesh	Boys	2008	12	4.92
Bangladesh	Boys	2009	12	5.04
Bangladesh	Boys	2010	12	5.16
Bangladesh	Boys	2011	12	5.29
Bangladesh	Boys	2012	12	5.43
Bangladesh	Boys	2013	12	5.57
Bangladesh	Boys	2014	12	5.73
Bangladesh	Boys	2015	12	5.89
Bangladesh	Boys	2016	12	6.05
Bangladesh	Boys	1975	13	4.08
Bangladesh	Boys	1976	13	4.10
Bangladesh	Boys	1977	13	4.12
Bangladesh	Boys	1978	13	4.14
Bangladesh	Boys	1979	13	4.17
Bangladesh	Boys	1980	13	4.19
Bangladesh	Boys	1981	13	4.22
Bangladesh	Boys	1982	13	4.25
Bangladesh	Boys	1983	13	4.28
Bangladesh	Boys	1984	13	4.32
Bangladesh	Boys	1985	13	4.35
Bangladesh	Boys	1986	13	4.39
Bangladesh	Boys	1987	13	4.42

Bangladesh	Boys	1988	13	4.46
Bangladesh	Boys	1989	13	4.51
Bangladesh	Boys	1990	13	4.55
Bangladesh	Boys	1991	13	4.60
Bangladesh	Boys	1992	13	4.65
Bangladesh	Boys	1993	13	4.70
Bangladesh	Boys	1994	13	4.75
Bangladesh	Boys	1995	13	4.81
Bangladesh	Boys	1996	13	4.87
Bangladesh	Boys	1997	13	4.94
Bangladesh	Boys	1998	13	5.00
Bangladesh	Boys	1999	13	5.08
Bangladesh	Boys	2000	13	5.15
Bangladesh	Boys	2001	13	5.24
Bangladesh	Boys	2002	13	5.32
Bangladesh	Boys	2003	13	5.42
Bangladesh	Boys	2004	13	5.52
Bangladesh	Boys	2005	13	5.62
Bangladesh	Boys	2006	13	5.74
Bangladesh	Boys	2007	13	5.86
Bangladesh	Boys	2008	13	5.98
Bangladesh	Boys	2009	13	6.12
Bangladesh	Boys	2010	13	6.26
Bangladesh	Boys	2011	13	6.41
Bangladesh	Boys	2012	13	6.57
Bangladesh	Boys	2013	13	6.74
Bangladesh	Boys	2014	13	6.91
Bangladesh	Boys	2015	13	7.10
Bangladesh	Boys	2016	13	7.29
Bangladesh	Boys	1975	14	5.13
Bangladesh	Boys	1976	14	5.16
Bangladesh	Boys	1977	14	5.18
Bangladesh	Boys	1978	14	5.21
Bangladesh	Boys	1979	14	5.24
Bangladesh	Boys	1980	14	5.27
Bangladesh	Boys	1981	14	5.31
Bangladesh	Boys	1982	14	5.34
Bangladesh	Boys	1983	14	5.38
Bangladesh	Boys	1984	14	5.42
Bangladesh	Boys	1985	14	5.46
Bangladesh	Boys	1986	14	5.50
Bangladesh	Boys	1987	14	5.54
Bangladesh	Boys	1988	14	5.59
Bangladesh	Boys	1989	14	5.64
Bangladesh	Boys	1990	14	5.69
Bangladesh	Boys	1991	14	5.74
Bangladesh	Boys	1992	14	5.80
Bangladesh	Boys	1993	14	5.85
Bangladesh	Boys	1994	14	5.92
Bangladesh	Boys	1995	14	5.98
Bangladesh	Boys	1996	14	6.05
Bangladesh	Boys	1997	14	6.12

Bangladesh	Boys	1998	14	6.20
Bangladesh	Boys	1999	14	6.28
Bangladesh	Boys	2000	14	6.36
Bangladesh	Boys	2001	14	6.45
Bangladesh	Boys	2002	14	6.55
Bangladesh	Boys	2003	14	6.65
Bangladesh	Boys	2004	14	6.76
Bangladesh	Boys	2005	14	6.88
Bangladesh	Boys	2006	14	7.00
Bangladesh	Boys	2007	14	7.14
Bangladesh	Boys	2008	14	7.28
Bangladesh	Boys	2009	14	7.42
Bangladesh	Boys	2010	14	7.58
Bangladesh	Boys	2011	14	7.75
Bangladesh	Boys	2012	14	7.92
Bangladesh	Boys	2013	14	8.10
Bangladesh	Boys	2014	14	8.30
Bangladesh	Boys	2015	14	8.50
Bangladesh	Boys	2016	14	8.72
Bangladesh	Boys	1975	15	6.40
Bangladesh	Boys	1976	15	6.43
Bangladesh	Boys	1977	15	6.46
Bangladesh	Boys	1978	15	6.49
Bangladesh	Boys	1979	15	6.53
Bangladesh	Boys	1980	15	6.57
Bangladesh	Boys	1981	15	6.61
Bangladesh	Boys	1982	15	6.65
Bangladesh	Boys	1983	15	6.69
Bangladesh	Boys	1984	15	6.74
Bangladesh	Boys	1985	15	6.78
Bangladesh	Boys	1986	15	6.83
Bangladesh	Boys	1987	15	6.88
Bangladesh	Boys	1988	15	6.93
Bangladesh	Boys	1989	15	6.99
Bangladesh	Boys	1990	15	7.04
Bangladesh	Boys	1991	15	7.10
Bangladesh	Boys	1992	15	7.16
Bangladesh	Boys	1993	15	7.23
Bangladesh	Boys	1994	15	7.30
Bangladesh	Boys	1995	15	7.37
Bangladesh	Boys	1996	15	7.44
Bangladesh	Boys	1997	15	7.52
Bangladesh	Boys	1998	15	7.60
Bangladesh	Boys	1999	15	7.69
Bangladesh	Boys	2000	15	7.78
Bangladesh	Boys	2001	15	7.88
Bangladesh	Boys	2002	15	7.98
Bangladesh	Boys	2003	15	8.09
Bangladesh	Boys	2004	15	8.21
Bangladesh	Boys	2005	15	8.33
Bangladesh	Boys	2006	15	8.47
Bangladesh	Boys	2007	15	8.61

Bangladesh	Boys	2008	15	8.75
Bangladesh	Boys	2009	15	8.91
Bangladesh	Boys	2010	15	9.08
Bangladesh	Boys	2011	15	9.25
Bangladesh	Boys	2012	15	9.43
Bangladesh	Boys	2013	15	9.63
Bangladesh	Boys	2014	15	9.83
Bangladesh	Boys	2015	15	10.05
Bangladesh	Boys	2016	15	10.28
Bangladesh	Boys	1975	16	7.81
Bangladesh	Boys	1976	16	7.85
Bangladesh	Boys	1977	16	7.89
Bangladesh	Boys	1978	16	7.93
Bangladesh	Boys	1979	16	7.97
Bangladesh	Boys	1980	16	8.02
Bangladesh	Boys	1981	16	8.06
Bangladesh	Boys	1982	16	8.11
Bangladesh	Boys	1983	16	8.16
Bangladesh	Boys	1984	16	8.21
Bangladesh	Boys	1985	16	8.26
Bangladesh	Boys	1986	16	8.32
Bangladesh	Boys	1987	16	8.37
Bangladesh	Boys	1988	16	8.43
Bangladesh	Boys	1989	16	8.49
Bangladesh	Boys	1990	16	8.55
Bangladesh	Boys	1991	16	8.62
Bangladesh	Boys	1992	16	8.69
Bangladesh	Boys	1993	16	8.76
Bangladesh	Boys	1994	16	8.83
Bangladesh	Boys	1995	16	8.91
Bangladesh	Boys	1996	16	8.99
Bangladesh	Boys	1997	16	9.07
Bangladesh	Boys	1998	16	9.16
Bangladesh	Boys	1999	16	9.25
Bangladesh	Boys	2000	16	9.35
Bangladesh	Boys	2001	16	9.45
Bangladesh	Boys	2002	16	9.56
Bangladesh	Boys	2003	16	9.68
Bangladesh	Boys	2004	16	9.80
Bangladesh	Boys	2005	16	9.93
Bangladesh	Boys	2006	16	10.07
Bangladesh	Boys	2007	16	10.22
Bangladesh	Boys	2008	16	10.37
Bangladesh	Boys	2009	16	10.54
Bangladesh	Boys	2010	16	10.71
Bangladesh	Boys	2011	16	10.89
Bangladesh	Boys	2012	16	11.08
Bangladesh	Boys	2013	16	11.28
Bangladesh	Boys	2014	16	11.50
Bangladesh	Boys	2015	16	11.72
Bangladesh	Boys	2016	16	11.96
Bangladesh	Boys	1975	17	9.27

Bangladesh	Boys	1976	17	9.32
Bangladesh	Boys	1977	17	9.36
Bangladesh	Boys	1978	17	9.41
Bangladesh	Boys	1979	17	9.46
Bangladesh	Boys	1980	17	9.51
Bangladesh	Boys	1981	17	9.57
Bangladesh	Boys	1982	17	9.62
Bangladesh	Boys	1983	17	9.68
Bangladesh	Boys	1984	17	9.74
Bangladesh	Boys	1985	17	9.80
Bangladesh	Boys	1986	17	9.86
Bangladesh	Boys	1987	17	9.92
Bangladesh	Boys	1988	17	9.99
Bangladesh	Boys	1989	17	10.05
Bangladesh	Boys	1990	17	10.12
Bangladesh	Boys	1991	17	10.20
Bangladesh	Boys	1992	17	10.27
Bangladesh	Boys	1993	17	10.35
Bangladesh	Boys	1994	17	10.43
Bangladesh	Boys	1995	17	10.51
Bangladesh	Boys	1996	17	10.60
Bangladesh	Boys	1997	17	10.69
Bangladesh	Boys	1998	17	10.78
Bangladesh	Boys	1999	17	10.88
Bangladesh	Boys	2000	17	10.99
Bangladesh	Boys	2001	17	11.10
Bangladesh	Boys	2002	17	11.22
Bangladesh	Boys	2003	17	11.34
Bangladesh	Boys	2004	17	11.47
Bangladesh	Boys	2005	17	11.61
Bangladesh	Boys	2006	17	11.76
Bangladesh	Boys	2007	17	11.91
Bangladesh	Boys	2008	17	12.07
Bangladesh	Boys	2009	17	12.24
Bangladesh	Boys	2010	17	12.42
Bangladesh	Boys	2011	17	12.61
Bangladesh	Boys	2012	17	12.81
Bangladesh	Boys	2013	17	13.02
Bangladesh	Boys	2014	17	13.24
Bangladesh	Boys	2015	17	13.48
Bangladesh	Boys	2016	17	13.72
Bangladesh	Boys	1975	18	10.68
Bangladesh	Boys	1976	18	10.73
Bangladesh	Boys	1977	18	10.78
Bangladesh	Boys	1978	18	10.84
Bangladesh	Boys	1979	18	10.90
Bangladesh	Boys	1980	18	10.96
Bangladesh	Boys	1981	18	11.02
Bangladesh	Boys	1982	18	11.09
Bangladesh	Boys	1983	18	11.15
Bangladesh	Boys	1984	18	11.22
Bangladesh	Boys	1985	18	11.29

Bangladesh	Boys	1986	18	11.36
Bangladesh	Boys	1987	18	11.43
Bangladesh	Boys	1988	18	11.50
Bangladesh	Boys	1989	18	11.58
Bangladesh	Boys	1990	18	11.66
Bangladesh	Boys	1991	18	11.74
Bangladesh	Boys	1992	18	11.82
Bangladesh	Boys	1993	18	11.91
Bangladesh	Boys	1994	18	12.00
Bangladesh	Boys	1995	18	12.09
Bangladesh	Boys	1996	18	12.19
Bangladesh	Boys	1997	18	12.29
Bangladesh	Boys	1998	18	12.39
Bangladesh	Boys	1999	18	12.50
Bangladesh	Boys	2000	18	12.62
Bangladesh	Boys	2001	18	12.74
Bangladesh	Boys	2002	18	12.87
Bangladesh	Boys	2003	18	13.01
Bangladesh	Boys	2004	18	13.15
Bangladesh	Boys	2005	18	13.30
Bangladesh	Boys	2006	18	13.46
Bangladesh	Boys	2007	18	13.63
Bangladesh	Boys	2008	18	13.80
Bangladesh	Boys	2009	18	13.99
Bangladesh	Boys	2010	18	14.18
Bangladesh	Boys	2011	18	14.38
Bangladesh	Boys	2012	18	14.60
Bangladesh	Boys	2013	18	14.82
Bangladesh	Boys	2014	18	15.06
Bangladesh	Boys	2015	18	15.31
Bangladesh	Boys	2016	18	15.57
Bangladesh	Boys	1975	Average	6.67
Bangladesh	Boys	1976	Average	6.70
Bangladesh	Boys	1977	Average	6.73
Bangladesh	Boys	1978	Average	6.77
Bangladesh	Boys	1979	Average	6.80
Bangladesh	Boys	1980	Average	6.84
Bangladesh	Boys	1981	Average	6.88
Bangladesh	Boys	1982	Average	6.93
Bangladesh	Boys	1983	Average	6.97
Bangladesh	Boys	1984	Average	7.02
Bangladesh	Boys	1985	Average	7.06
Bangladesh	Boys	1986	Average	7.11
Bangladesh	Boys	1987	Average	7.16
Bangladesh	Boys	1988	Average	7.22
Bangladesh	Boys	1989	Average	7.27
Bangladesh	Boys	1990	Average	7.33
Bangladesh	Boys	1991	Average	7.39
Bangladesh	Boys	1992	Average	7.45
Bangladesh	Boys	1993	Average	7.51
Bangladesh	Boys	1994	Average	7.58
Bangladesh	Boys	1995	Average	7.65

Bangladesh	Boys	1996	Average	7.73
Bangladesh	Boys	1997	Average	7.80
Bangladesh	Boys	1998	Average	7.89
Bangladesh	Boys	1999	Average	7.97
Bangladesh	Boys	2000	Average	8.06
Bangladesh	Boys	2001	Average	8.16
Bangladesh	Boys	2002	Average	8.27
Bangladesh	Boys	2003	Average	8.38
Bangladesh	Boys	2004	Average	8.49
Bangladesh	Boys	2005	Average	8.61
Bangladesh	Boys	2006	Average	8.74
Bangladesh	Boys	2007	Average	8.88
Bangladesh	Boys	2008	Average	9.03
Bangladesh	Boys	2009	Average	9.18
Bangladesh	Boys	2010	Average	9.34
Bangladesh	Boys	2011	Average	9.51
Bangladesh	Boys	2012	Average	9.69
Bangladesh	Boys	2013	Average	9.88
Bangladesh	Boys	2014	Average	10.08
Bangladesh	Boys	2015	Average	10.29
Bangladesh	Boys	2016	Average	10.51
Bangladesh	Girls	1975	12	3.78
Bangladesh	Girls	1976	12	3.79
Bangladesh	Girls	1977	12	3.81
Bangladesh	Girls	1978	12	3.83
Bangladesh	Girls	1979	12	3.85
Bangladesh	Girls	1980	12	3.87
Bangladesh	Girls	1981	12	3.89
Bangladesh	Girls	1982	12	3.91
Bangladesh	Girls	1983	12	3.94
Bangladesh	Girls	1984	12	3.96
Bangladesh	Girls	1985	12	3.98
Bangladesh	Girls	1986	12	4.01
Bangladesh	Girls	1987	12	4.03
Bangladesh	Girls	1988	12	4.06
Bangladesh	Girls	1989	12	4.09
Bangladesh	Girls	1990	12	4.12
Bangladesh	Girls	1991	12	4.15
Bangladesh	Girls	1992	12	4.18
Bangladesh	Girls	1993	12	4.22
Bangladesh	Girls	1994	12	4.25
Bangladesh	Girls	1995	12	4.29
Bangladesh	Girls	1996	12	4.33
Bangladesh	Girls	1997	12	4.37
Bangladesh	Girls	1998	12	4.42
Bangladesh	Girls	1999	12	4.46
Bangladesh	Girls	2000	12	4.51
Bangladesh	Girls	2001	12	4.56
Bangladesh	Girls	2002	12	4.62
Bangladesh	Girls	2003	12	4.68
Bangladesh	Girls	2004	12	4.74
Bangladesh	Girls	2005	12	4.81

Bangladesh	Girls	2006	12	4.88
Bangladesh	Girls	2007	12	4.95
Bangladesh	Girls	2008	12	5.03
Bangladesh	Girls	2009	12	5.11
Bangladesh	Girls	2010	12	5.20
Bangladesh	Girls	2011	12	5.29
Bangladesh	Girls	2012	12	5.38
Bangladesh	Girls	2013	12	5.48
Bangladesh	Girls	2014	12	5.59
Bangladesh	Girls	2015	12	5.69
Bangladesh	Girls	2016	12	5.81
Bangladesh	Girls	1975	13	4.77
Bangladesh	Girls	1976	13	4.79
Bangladesh	Girls	1977	13	4.82
Bangladesh	Girls	1978	13	4.84
Bangladesh	Girls	1979	13	4.86
Bangladesh	Girls	1980	13	4.89
Bangladesh	Girls	1981	13	4.92
Bangladesh	Girls	1982	13	4.95
Bangladesh	Girls	1983	13	4.98
Bangladesh	Girls	1984	13	5.00
Bangladesh	Girls	1985	13	5.04
Bangladesh	Girls	1986	13	5.07
Bangladesh	Girls	1987	13	5.10
Bangladesh	Girls	1988	13	5.13
Bangladesh	Girls	1989	13	5.17
Bangladesh	Girls	1990	13	5.20
Bangladesh	Girls	1991	13	5.24
Bangladesh	Girls	1992	13	5.28
Bangladesh	Girls	1993	13	5.32
Bangladesh	Girls	1994	13	5.36
Bangladesh	Girls	1995	13	5.41
Bangladesh	Girls	1996	13	5.46
Bangladesh	Girls	1997	13	5.51
Bangladesh	Girls	1998	13	5.56
Bangladesh	Girls	1999	13	5.61
Bangladesh	Girls	2000	13	5.67
Bangladesh	Girls	2001	13	5.73
Bangladesh	Girls	2002	13	5.80
Bangladesh	Girls	2003	13	5.87
Bangladesh	Girls	2004	13	5.94
Bangladesh	Girls	2005	13	6.02
Bangladesh	Girls	2006	13	6.10
Bangladesh	Girls	2007	13	6.18
Bangladesh	Girls	2008	13	6.27
Bangladesh	Girls	2009	13	6.37
Bangladesh	Girls	2010	13	6.47
Bangladesh	Girls	2011	13	6.57
Bangladesh	Girls	2012	13	6.68
Bangladesh	Girls	2013	13	6.80
Bangladesh	Girls	2014	13	6.92
Bangladesh	Girls	2015	13	7.04

Bangladesh	Girls	2016	13	7.18
Bangladesh	Girls	1975	14	5.93
Bangladesh	Girls	1976	14	5.95
Bangladesh	Girls	1977	14	5.98
Bangladesh	Girls	1978	14	6.01
Bangladesh	Girls	1979	14	6.04
Bangladesh	Girls	1980	14	6.07
Bangladesh	Girls	1981	14	6.10
Bangladesh	Girls	1982	14	6.14
Bangladesh	Girls	1983	14	6.17
Bangladesh	Girls	1984	14	6.21
Bangladesh	Girls	1985	14	6.24
Bangladesh	Girls	1986	14	6.28
Bangladesh	Girls	1987	14	6.32
Bangladesh	Girls	1988	14	6.36
Bangladesh	Girls	1989	14	6.40
Bangladesh	Girls	1990	14	6.44
Bangladesh	Girls	1991	14	6.48
Bangladesh	Girls	1992	14	6.53
Bangladesh	Girls	1993	14	6.58
Bangladesh	Girls	1994	14	6.63
Bangladesh	Girls	1995	14	6.68
Bangladesh	Girls	1996	14	6.73
Bangladesh	Girls	1997	14	6.79
Bangladesh	Girls	1998	14	6.85
Bangladesh	Girls	1999	14	6.91
Bangladesh	Girls	2000	14	6.98
Bangladesh	Girls	2001	14	7.05
Bangladesh	Girls	2002	14	7.12
Bangladesh	Girls	2003	14	7.20
Bangladesh	Girls	2004	14	7.28
Bangladesh	Girls	2005	14	7.36
Bangladesh	Girls	2006	14	7.45
Bangladesh	Girls	2007	14	7.55
Bangladesh	Girls	2008	14	7.65
Bangladesh	Girls	2009	14	7.75
Bangladesh	Girls	2010	14	7.86
Bangladesh	Girls	2011	14	7.98
Bangladesh	Girls	2012	14	8.10
Bangladesh	Girls	2013	14	8.23
Bangladesh	Girls	2014	14	8.36
Bangladesh	Girls	2015	14	8.50
Bangladesh	Girls	2016	14	8.64
Bangladesh	Girls	1975	15	7.07
Bangladesh	Girls	1976	15	7.10
Bangladesh	Girls	1977	15	7.13
Bangladesh	Girls	1978	15	7.16
Bangladesh	Girls	1979	15	7.20
Bangladesh	Girls	1980	15	7.24
Bangladesh	Girls	1981	15	7.27
Bangladesh	Girls	1982	15	7.31
Bangladesh	Girls	1983	15	7.35

Bangladesh	Girls	1984	15	7.39
Bangladesh	Girls	1985	15	7.43
Bangladesh	Girls	1986	15	7.48
Bangladesh	Girls	1987	15	7.52
Bangladesh	Girls	1988	15	7.56
Bangladesh	Girls	1989	15	7.61
Bangladesh	Girls	1990	15	7.65
Bangladesh	Girls	1991	15	7.70
Bangladesh	Girls	1992	15	7.75
Bangladesh	Girls	1993	15	7.80
Bangladesh	Girls	1994	15	7.86
Bangladesh	Girls	1995	15	7.92
Bangladesh	Girls	1996	15	7.98
Bangladesh	Girls	1997	15	8.04
Bangladesh	Girls	1998	15	8.10
Bangladesh	Girls	1999	15	8.17
Bangladesh	Girls	2000	15	8.24
Bangladesh	Girls	2001	15	8.32
Bangladesh	Girls	2002	15	8.40
Bangladesh	Girls	2003	15	8.48
Bangladesh	Girls	2004	15	8.57
Bangladesh	Girls	2005	15	8.66
Bangladesh	Girls	2006	15	8.76
Bangladesh	Girls	2007	15	8.86
Bangladesh	Girls	2008	15	8.97
Bangladesh	Girls	2009	15	9.08
Bangladesh	Girls	2010	15	9.20
Bangladesh	Girls	2011	15	9.32
Bangladesh	Girls	2012	15	9.45
Bangladesh	Girls	2013	15	9.58
Bangladesh	Girls	2014	15	9.72
Bangladesh	Girls	2015	15	9.87
Bangladesh	Girls	2016	15	10.02
Bangladesh	Girls	1975	16	8.02
Bangladesh	Girls	1976	16	8.05
Bangladesh	Girls	1977	16	8.09
Bangladesh	Girls	1978	16	8.13
Bangladesh	Girls	1979	16	8.17
Bangladesh	Girls	1980	16	8.21
Bangladesh	Girls	1981	16	8.25
Bangladesh	Girls	1982	16	8.29
Bangladesh	Girls	1983	16	8.34
Bangladesh	Girls	1984	16	8.38
Bangladesh	Girls	1985	16	8.42
Bangladesh	Girls	1986	16	8.47
Bangladesh	Girls	1987	16	8.52
Bangladesh	Girls	1988	16	8.56
Bangladesh	Girls	1989	16	8.61
Bangladesh	Girls	1990	16	8.66
Bangladesh	Girls	1991	16	8.72
Bangladesh	Girls	1992	16	8.77
Bangladesh	Girls	1993	16	8.82

Bangladesh	Girls	1994	16	8.88
Bangladesh	Girls	1995	16	8.94
Bangladesh	Girls	1996	16	9.01
Bangladesh	Girls	1997	16	9.07
Bangladesh	Girls	1998	16	9.14
Bangladesh	Girls	1999	16	9.22
Bangladesh	Girls	2000	16	9.29
Bangladesh	Girls	2001	16	9.37
Bangladesh	Girls	2002	16	9.45
Bangladesh	Girls	2003	16	9.54
Bangladesh	Girls	2004	16	9.64
Bangladesh	Girls	2005	16	9.73
Bangladesh	Girls	2006	16	9.84
Bangladesh	Girls	2007	16	9.94
Bangladesh	Girls	2008	16	10.06
Bangladesh	Girls	2009	16	10.18
Bangladesh	Girls	2010	16	10.30
Bangladesh	Girls	2011	16	10.43
Bangladesh	Girls	2012	16	10.56
Bangladesh	Girls	2013	16	10.70
Bangladesh	Girls	2014	16	10.85
Bangladesh	Girls	2015	16	11.00
Bangladesh	Girls	2016	16	11.16
Bangladesh	Girls	1975	17	8.67
Bangladesh	Girls	1976	17	8.71
Bangladesh	Girls	1977	17	8.75
Bangladesh	Girls	1978	17	8.79
Bangladesh	Girls	1979	17	8.83
Bangladesh	Girls	1980	17	8.88
Bangladesh	Girls	1981	17	8.92
Bangladesh	Girls	1982	17	8.97
Bangladesh	Girls	1983	17	9.02
Bangladesh	Girls	1984	17	9.06
Bangladesh	Girls	1985	17	9.11
Bangladesh	Girls	1986	17	9.16
Bangladesh	Girls	1987	17	9.21
Bangladesh	Girls	1988	17	9.26
Bangladesh	Girls	1989	17	9.31
Bangladesh	Girls	1990	17	9.37
Bangladesh	Girls	1991	17	9.42
Bangladesh	Girls	1992	17	9.48
Bangladesh	Girls	1993	17	9.54
Bangladesh	Girls	1994	17	9.60
Bangladesh	Girls	1995	17	9.67
Bangladesh	Girls	1996	17	9.73
Bangladesh	Girls	1997	17	9.80
Bangladesh	Girls	1998	17	9.88
Bangladesh	Girls	1999	17	9.95
Bangladesh	Girls	2000	17	10.03
Bangladesh	Girls	2001	17	10.12
Bangladesh	Girls	2002	17	10.21
Bangladesh	Girls	2003	17	10.30

Bangladesh	Girls	2004	17	10.40
Bangladesh	Girls	2005	17	10.51
Bangladesh	Girls	2006	17	10.62
Bangladesh	Girls	2007	17	10.73
Bangladesh	Girls	2008	17	10.85
Bangladesh	Girls	2009	17	10.98
Bangladesh	Girls	2010	17	11.11
Bangladesh	Girls	2011	17	11.25
Bangladesh	Girls	2012	17	11.39
Bangladesh	Girls	2013	17	11.54
Bangladesh	Girls	2014	17	11.70
Bangladesh	Girls	2015	17	11.86
Bangladesh	Girls	2016	17	12.03
Bangladesh	Girls	1975	18	9.02
Bangladesh	Girls	1976	18	9.06
Bangladesh	Girls	1977	18	9.11
Bangladesh	Girls	1978	18	9.15
Bangladesh	Girls	1979	18	9.20
Bangladesh	Girls	1980	18	9.24
Bangladesh	Girls	1981	18	9.29
Bangladesh	Girls	1982	18	9.34
Bangladesh	Girls	1983	18	9.39
Bangladesh	Girls	1984	18	9.44
Bangladesh	Girls	1985	18	9.50
Bangladesh	Girls	1986	18	9.55
Bangladesh	Girls	1987	18	9.60
Bangladesh	Girls	1988	18	9.66
Bangladesh	Girls	1989	18	9.71
Bangladesh	Girls	1990	18	9.77
Bangladesh	Girls	1991	18	9.83
Bangladesh	Girls	1992	18	9.89
Bangladesh	Girls	1993	18	9.96
Bangladesh	Girls	1994	18	10.03
Bangladesh	Girls	1995	18	10.10
Bangladesh	Girls	1996	18	10.17
Bangladesh	Girls	1997	18	10.25
Bangladesh	Girls	1998	18	10.33
Bangladesh	Girls	1999	18	10.41
Bangladesh	Girls	2000	18	10.50
Bangladesh	Girls	2001	18	10.60
Bangladesh	Girls	2002	18	10.70
Bangladesh	Girls	2003	18	10.80
Bangladesh	Girls	2004	18	10.91
Bangladesh	Girls	2005	18	11.02
Bangladesh	Girls	2006	18	11.14
Bangladesh	Girls	2007	18	11.27
Bangladesh	Girls	2008	18	11.41
Bangladesh	Girls	2009	18	11.55
Bangladesh	Girls	2010	18	11.69
Bangladesh	Girls	2011	18	11.84
Bangladesh	Girls	2012	18	12.00
Bangladesh	Girls	2013	18	12.17

Bangladesh	Girls	2014	18	12.34
Bangladesh	Girls	2015	18	12.52
Bangladesh	Girls	2016	18	12.71
Bangladesh	Girls	1975	Average	6.75
Bangladesh	Girls	1976	Average	6.78
Bangladesh	Girls	1977	Average	6.81
Bangladesh	Girls	1978	Average	6.84
Bangladesh	Girls	1979	Average	6.88
Bangladesh	Girls	1980	Average	6.91
Bangladesh	Girls	1981	Average	6.95
Bangladesh	Girls	1982	Average	6.99
Bangladesh	Girls	1983	Average	7.03
Bangladesh	Girls	1984	Average	7.06
Bangladesh	Girls	1985	Average	7.10
Bangladesh	Girls	1986	Average	7.14
Bangladesh	Girls	1987	Average	7.18
Bangladesh	Girls	1988	Average	7.23
Bangladesh	Girls	1989	Average	7.27
Bangladesh	Girls	1990	Average	7.32
Bangladesh	Girls	1991	Average	7.36
Bangladesh	Girls	1992	Average	7.41
Bangladesh	Girls	1993	Average	7.46
Bangladesh	Girls	1994	Average	7.52
Bangladesh	Girls	1995	Average	7.57
Bangladesh	Girls	1996	Average	7.63
Bangladesh	Girls	1997	Average	7.69
Bangladesh	Girls	1998	Average	7.75
Bangladesh	Girls	1999	Average	7.82
Bangladesh	Girls	2000	Average	7.89
Bangladesh	Girls	2001	Average	7.96
Bangladesh	Girls	2002	Average	8.04
Bangladesh	Girls	2003	Average	8.12
Bangladesh	Girls	2004	Average	8.21
Bangladesh	Girls	2005	Average	8.30
Bangladesh	Girls	2006	Average	8.40
Bangladesh	Girls	2007	Average	8.50
Bangladesh	Girls	2008	Average	8.60
Bangladesh	Girls	2009	Average	8.72
Bangladesh	Girls	2010	Average	8.83
Bangladesh	Girls	2011	Average	8.95
Bangladesh	Girls	2012	Average	9.08
Bangladesh	Girls	2013	Average	9.21
Bangladesh	Girls	2014	Average	9.35
Bangladesh	Girls	2015	Average	9.50
Bangladesh	Girls	2016	Average	9.65
Barbados	Boys	1975	12	5.73
Barbados	Boys	1976	12	5.78
Barbados	Boys	1977	12	5.84
Barbados	Boys	1978	12	5.91
Barbados	Boys	1979	12	5.98
Barbados	Boys	1980	12	6.05
Barbados	Boys	1981	12	6.12

Barbados	Boys	1982	12	6.19
Barbados	Boys	1983	12	6.26
Barbados	Boys	1984	12	6.32
Barbados	Boys	1985	12	6.38
Barbados	Boys	1986	12	6.45
Barbados	Boys	1987	12	6.51
Barbados	Boys	1988	12	6.58
Barbados	Boys	1989	12	6.64
Barbados	Boys	1990	12	6.72
Barbados	Boys	1991	12	6.80
Barbados	Boys	1992	12	6.90
Barbados	Boys	1993	12	7.01
Barbados	Boys	1994	12	7.13
Barbados	Boys	1995	12	7.27
Barbados	Boys	1996	12	7.41
Barbados	Boys	1997	12	7.56
Barbados	Boys	1998	12	7.73
Barbados	Boys	1999	12	7.90
Barbados	Boys	2000	12	8.08
Barbados	Boys	2001	12	8.27
Barbados	Boys	2002	12	8.46
Barbados	Boys	2003	12	8.66
Barbados	Boys	2004	12	8.86
Barbados	Boys	2005	12	9.07
Barbados	Boys	2006	12	9.29
Barbados	Boys	2007	12	9.51
Barbados	Boys	2008	12	9.74
Barbados	Boys	2009	12	9.98
Barbados	Boys	2010	12	10.23
Barbados	Boys	2011	12	10.48
Barbados	Boys	2012	12	10.74
Barbados	Boys	2013	12	11.01
Barbados	Boys	2014	12	11.28
Barbados	Boys	2015	12	11.56
Barbados	Boys	2016	12	11.85
Barbados	Boys	1975	13	7.08
Barbados	Boys	1976	13	7.14
Barbados	Boys	1977	13	7.21
Barbados	Boys	1978	13	7.29
Barbados	Boys	1979	13	7.37
Barbados	Boys	1980	13	7.45
Barbados	Boys	1981	13	7.53
Barbados	Boys	1982	13	7.61
Barbados	Boys	1983	13	7.69
Barbados	Boys	1984	13	7.76
Barbados	Boys	1985	13	7.83
Barbados	Boys	1986	13	7.90
Barbados	Boys	1987	13	7.98
Barbados	Boys	1988	13	8.05
Barbados	Boys	1989	13	8.13
Barbados	Boys	1990	13	8.21
Barbados	Boys	1991	13	8.31

Barbados	Boys	1992	13	8.42
Barbados	Boys	1993	13	8.55
Barbados	Boys	1994	13	8.69
Barbados	Boys	1995	13	8.84
Barbados	Boys	1996	13	9.01
Barbados	Boys	1997	13	9.19
Barbados	Boys	1998	13	9.38
Barbados	Boys	1999	13	9.58
Barbados	Boys	2000	13	9.79
Barbados	Boys	2001	13	10.01
Barbados	Boys	2002	13	10.23
Barbados	Boys	2003	13	10.46
Barbados	Boys	2004	13	10.70
Barbados	Boys	2005	13	10.94
Barbados	Boys	2006	13	11.20
Barbados	Boys	2007	13	11.46
Barbados	Boys	2008	13	11.74
Barbados	Boys	2009	13	12.02
Barbados	Boys	2010	13	12.32
Barbados	Boys	2011	13	12.63
Barbados	Boys	2012	13	12.94
Barbados	Boys	2013	13	13.27
Barbados	Boys	2014	13	13.60
Barbados	Boys	2015	13	13.95
Barbados	Boys	2016	13	14.30
Barbados	Boys	1975	14	8.76
Barbados	Boys	1976	14	8.83
Barbados	Boys	1977	14	8.91
Barbados	Boys	1978	14	8.99
Barbados	Boys	1979	14	9.07
Barbados	Boys	1980	14	9.17
Barbados	Boys	1981	14	9.26
Barbados	Boys	1982	14	9.34
Barbados	Boys	1983	14	9.42
Barbados	Boys	1984	14	9.50
Barbados	Boys	1985	14	9.58
Barbados	Boys	1986	14	9.65
Barbados	Boys	1987	14	9.73
Barbados	Boys	1988	14	9.81
Barbados	Boys	1989	14	9.90
Barbados	Boys	1990	14	9.99
Barbados	Boys	1991	14	10.09
Barbados	Boys	1992	14	10.22
Barbados	Boys	1993	14	10.35
Barbados	Boys	1994	14	10.50
Barbados	Boys	1995	14	10.67
Barbados	Boys	1996	14	10.85
Barbados	Boys	1997	14	11.04
Barbados	Boys	1998	14	11.25
Barbados	Boys	1999	14	11.47
Barbados	Boys	2000	14	11.70
Barbados	Boys	2001	14	11.94

Barbados	Boys	2002	14	12.18
Barbados	Boys	2003	14	12.44
Barbados	Boys	2004	14	12.70
Barbados	Boys	2005	14	12.97
Barbados	Boys	2006	14	13.26
Barbados	Boys	2007	14	13.55
Barbados	Boys	2008	14	13.86
Barbados	Boys	2009	14	14.18
Barbados	Boys	2010	14	14.51
Barbados	Boys	2011	14	14.86
Barbados	Boys	2012	14	15.22
Barbados	Boys	2013	14	15.59
Barbados	Boys	2014	14	15.97
Barbados	Boys	2015	14	16.37
Barbados	Boys	2016	14	16.78
Barbados	Boys	1975	15	10.70
Barbados	Boys	1976	15	10.77
Barbados	Boys	1977	15	10.85
Barbados	Boys	1978	15	10.94
Barbados	Boys	1979	15	11.03
Barbados	Boys	1980	15	11.13
Barbados	Boys	1981	15	11.22
Barbados	Boys	1982	15	11.31
Barbados	Boys	1983	15	11.39
Barbados	Boys	1984	15	11.47
Barbados	Boys	1985	15	11.55
Barbados	Boys	1986	15	11.63
Barbados	Boys	1987	15	11.71
Barbados	Boys	1988	15	11.79
Barbados	Boys	1989	15	11.88
Barbados	Boys	1990	15	11.97
Barbados	Boys	1991	15	12.08
Barbados	Boys	1992	15	12.21
Barbados	Boys	1993	15	12.35
Barbados	Boys	1994	15	12.51
Barbados	Boys	1995	15	12.68
Barbados	Boys	1996	15	12.86
Barbados	Boys	1997	15	13.07
Barbados	Boys	1998	15	13.28
Barbados	Boys	1999	15	13.51
Barbados	Boys	2000	15	13.75
Barbados	Boys	2001	15	13.99
Barbados	Boys	2002	15	14.25
Barbados	Boys	2003	15	14.51
Barbados	Boys	2004	15	14.79
Barbados	Boys	2005	15	15.07
Barbados	Boys	2006	15	15.37
Barbados	Boys	2007	15	15.68
Barbados	Boys	2008	15	16.01
Barbados	Boys	2009	15	16.34
Barbados	Boys	2010	15	16.70
Barbados	Boys	2011	15	17.07

Barbados	Boys	2012	15	17.45
Barbados	Boys	2013	15	17.85
Barbados	Boys	2014	15	18.27
Barbados	Boys	2015	15	18.70
Barbados	Boys	2016	15	19.15
Barbados	Boys	1975	16	12.82
Barbados	Boys	1976	16	12.89
Barbados	Boys	1977	16	12.98
Barbados	Boys	1978	16	13.07
Barbados	Boys	1979	16	13.16
Barbados	Boys	1980	16	13.26
Barbados	Boys	1981	16	13.35
Barbados	Boys	1982	16	13.44
Barbados	Boys	1983	16	13.53
Barbados	Boys	1984	16	13.61
Barbados	Boys	1985	16	13.69
Barbados	Boys	1986	16	13.77
Barbados	Boys	1987	16	13.85
Barbados	Boys	1988	16	13.94
Barbados	Boys	1989	16	14.02
Barbados	Boys	1990	16	14.12
Barbados	Boys	1991	16	14.23
Barbados	Boys	1992	16	14.35
Barbados	Boys	1993	16	14.49
Barbados	Boys	1994	16	14.65
Barbados	Boys	1995	16	14.83
Barbados	Boys	1996	16	15.01
Barbados	Boys	1997	16	15.21
Barbados	Boys	1998	16	15.43
Barbados	Boys	1999	16	15.66
Barbados	Boys	2000	16	15.90
Barbados	Boys	2001	16	16.15
Barbados	Boys	2002	16	16.41
Barbados	Boys	2003	16	16.68
Barbados	Boys	2004	16	16.96
Barbados	Boys	2005	16	17.25
Barbados	Boys	2006	16	17.55
Barbados	Boys	2007	16	17.86
Barbados	Boys	2008	16	18.19
Barbados	Boys	2009	16	18.54
Barbados	Boys	2010	16	18.90
Barbados	Boys	2011	16	19.28
Barbados	Boys	2012	16	19.68
Barbados	Boys	2013	16	20.10
Barbados	Boys	2014	16	20.53
Barbados	Boys	2015	16	20.98
Barbados	Boys	2016	16	21.45
Barbados	Boys	1975	17	15.02
Barbados	Boys	1976	17	15.10
Barbados	Boys	1977	17	15.19
Barbados	Boys	1978	17	15.28
Barbados	Boys	1979	17	15.38

Barbados	Boys	1980	17	15.48
Barbados	Boys	1981	17	15.57
Barbados	Boys	1982	17	15.66
Barbados	Boys	1983	17	15.75
Barbados	Boys	1984	17	15.84
Barbados	Boys	1985	17	15.92
Barbados	Boys	1986	17	16.00
Barbados	Boys	1987	17	16.08
Barbados	Boys	1988	17	16.17
Barbados	Boys	1989	17	16.26
Barbados	Boys	1990	17	16.35
Barbados	Boys	1991	17	16.46
Barbados	Boys	1992	17	16.59
Barbados	Boys	1993	17	16.73
Barbados	Boys	1994	17	16.89
Barbados	Boys	1995	17	17.06
Barbados	Boys	1996	17	17.25
Barbados	Boys	1997	17	17.46
Barbados	Boys	1998	17	17.67
Barbados	Boys	1999	17	17.90
Barbados	Boys	2000	17	18.15
Barbados	Boys	2001	17	18.40
Barbados	Boys	2002	17	18.66
Barbados	Boys	2003	17	18.93
Barbados	Boys	2004	17	19.21
Barbados	Boys	2005	17	19.50
Barbados	Boys	2006	17	19.81
Barbados	Boys	2007	17	20.13
Barbados	Boys	2008	17	20.46
Barbados	Boys	2009	17	20.82
Barbados	Boys	2010	17	21.18
Barbados	Boys	2011	17	21.57
Barbados	Boys	2012	17	21.98
Barbados	Boys	2013	17	22.40
Barbados	Boys	2014	17	22.84
Barbados	Boys	2015	17	23.31
Barbados	Boys	2016	17	23.79
Barbados	Boys	1975	18	17.24
Barbados	Boys	1976	18	17.32
Barbados	Boys	1977	18	17.42
Barbados	Boys	1978	18	17.51
Barbados	Boys	1979	18	17.62
Barbados	Boys	1980	18	17.72
Barbados	Boys	1981	18	17.82
Barbados	Boys	1982	18	17.92
Barbados	Boys	1983	18	18.01
Barbados	Boys	1984	18	18.10
Barbados	Boys	1985	18	18.18
Barbados	Boys	1986	18	18.27
Barbados	Boys	1987	18	18.36
Barbados	Boys	1988	18	18.44
Barbados	Boys	1989	18	18.54

Barbados	Boys	1990	18	18.63
Barbados	Boys	1991	18	18.75
Barbados	Boys	1992	18	18.88
Barbados	Boys	1993	18	19.03
Barbados	Boys	1994	18	19.19
Barbados	Boys	1995	18	19.38
Barbados	Boys	1996	18	19.57
Barbados	Boys	1997	18	19.78
Barbados	Boys	1998	18	20.01
Barbados	Boys	1999	18	20.25
Barbados	Boys	2000	18	20.50
Barbados	Boys	2001	18	20.76
Barbados	Boys	2002	18	21.03
Barbados	Boys	2003	18	21.31
Barbados	Boys	2004	18	21.60
Barbados	Boys	2005	18	21.90
Barbados	Boys	2006	18	22.21
Barbados	Boys	2007	18	22.55
Barbados	Boys	2008	18	22.89
Barbados	Boys	2009	18	23.25
Barbados	Boys	2010	18	23.63
Barbados	Boys	2011	18	24.03
Barbados	Boys	2012	18	24.45
Barbados	Boys	2013	18	24.88
Barbados	Boys	2014	18	25.34
Barbados	Boys	2015	18	25.82
Barbados	Boys	2016	18	26.31
Barbados	Boys	1975	Average	11.05
Barbados	Boys	1976	Average	11.12
Barbados	Boys	1977	Average	11.20
Barbados	Boys	1978	Average	11.28
Barbados	Boys	1979	Average	11.37
Barbados	Boys	1980	Average	11.47
Barbados	Boys	1981	Average	11.55
Barbados	Boys	1982	Average	11.64
Barbados	Boys	1983	Average	11.72
Barbados	Boys	1984	Average	11.80
Barbados	Boys	1985	Average	11.88
Barbados	Boys	1986	Average	11.95
Barbados	Boys	1987	Average	12.03
Barbados	Boys	1988	Average	12.11
Barbados	Boys	1989	Average	12.20
Barbados	Boys	1990	Average	12.28
Barbados	Boys	1991	Average	12.39
Barbados	Boys	1992	Average	12.51
Barbados	Boys	1993	Average	12.65
Barbados	Boys	1994	Average	12.80
Barbados	Boys	1995	Average	12.96
Barbados	Boys	1996	Average	13.14
Barbados	Boys	1997	Average	13.33
Barbados	Boys	1998	Average	13.54
Barbados	Boys	1999	Average	13.75

Barbados	Boys	2000	Average	13.98
Barbados	Boys	2001	Average	14.22
Barbados	Boys	2002	Average	14.46
Barbados	Boys	2003	Average	14.71
Barbados	Boys	2004	Average	14.97
Barbados	Boys	2005	Average	15.24
Barbados	Boys	2006	Average	15.53
Barbados	Boys	2007	Average	15.82
Barbados	Boys	2008	Average	16.13
Barbados	Boys	2009	Average	16.45
Barbados	Boys	2010	Average	16.78
Barbados	Boys	2011	Average	17.13
Barbados	Boys	2012	Average	17.49
Barbados	Boys	2013	Average	17.87
Barbados	Boys	2014	Average	18.26
Barbados	Boys	2015	Average	18.67
Barbados	Boys	2016	Average	19.09
Barbados	Girls	1975	12	6.04
Barbados	Girls	1976	12	6.10
Barbados	Girls	1977	12	6.16
Barbados	Girls	1978	12	6.22
Barbados	Girls	1979	12	6.29
Barbados	Girls	1980	12	6.36
Barbados	Girls	1981	12	6.43
Barbados	Girls	1982	12	6.50
Barbados	Girls	1983	12	6.56
Barbados	Girls	1984	12	6.62
Barbados	Girls	1985	12	6.67
Barbados	Girls	1986	12	6.73
Barbados	Girls	1987	12	6.79
Barbados	Girls	1988	12	6.85
Barbados	Girls	1989	12	6.91
Barbados	Girls	1990	12	6.97
Barbados	Girls	1991	12	7.05
Barbados	Girls	1992	12	7.14
Barbados	Girls	1993	12	7.23
Barbados	Girls	1994	12	7.34
Barbados	Girls	1995	12	7.45
Barbados	Girls	1996	12	7.57
Barbados	Girls	1997	12	7.70
Barbados	Girls	1998	12	7.83
Barbados	Girls	1999	12	7.97
Barbados	Girls	2000	12	8.12
Barbados	Girls	2001	12	8.27
Barbados	Girls	2002	12	8.42
Barbados	Girls	2003	12	8.57
Barbados	Girls	2004	12	8.72
Barbados	Girls	2005	12	8.88
Barbados	Girls	2006	12	9.04
Barbados	Girls	2007	12	9.21
Barbados	Girls	2008	12	9.38
Barbados	Girls	2009	12	9.56

Barbados	Girls	2010	12	9.74
Barbados	Girls	2011	12	9.93
Barbados	Girls	2012	12	10.12
Barbados	Girls	2013	12	10.32
Barbados	Girls	2014	12	10.52
Barbados	Girls	2015	12	10.73
Barbados	Girls	2016	12	10.95
Barbados	Girls	1975	13	7.48
Barbados	Girls	1976	13	7.55
Barbados	Girls	1977	13	7.62
Barbados	Girls	1978	13	7.69
Barbados	Girls	1979	13	7.77
Barbados	Girls	1980	13	7.85
Barbados	Girls	1981	13	7.93
Barbados	Girls	1982	13	8.00
Barbados	Girls	1983	13	8.07
Barbados	Girls	1984	13	8.14
Barbados	Girls	1985	13	8.20
Barbados	Girls	1986	13	8.27
Barbados	Girls	1987	13	8.34
Barbados	Girls	1988	13	8.40
Barbados	Girls	1989	13	8.47
Barbados	Girls	1990	13	8.54
Barbados	Girls	1991	13	8.63
Barbados	Girls	1992	13	8.73
Barbados	Girls	1993	13	8.84
Barbados	Girls	1994	13	8.95
Barbados	Girls	1995	13	9.08
Barbados	Girls	1996	13	9.22
Barbados	Girls	1997	13	9.36
Barbados	Girls	1998	13	9.52
Barbados	Girls	1999	13	9.68
Barbados	Girls	2000	13	9.84
Barbados	Girls	2001	13	10.01
Barbados	Girls	2002	13	10.18
Barbados	Girls	2003	13	10.35
Barbados	Girls	2004	13	10.53
Barbados	Girls	2005	13	10.71
Barbados	Girls	2006	13	10.89
Barbados	Girls	2007	13	11.08
Barbados	Girls	2008	13	11.28
Barbados	Girls	2009	13	11.48
Barbados	Girls	2010	13	11.69
Barbados	Girls	2011	13	11.91
Barbados	Girls	2012	13	12.13
Barbados	Girls	2013	13	12.37
Barbados	Girls	2014	13	12.61
Barbados	Girls	2015	13	12.85
Barbados	Girls	2016	13	13.11
Barbados	Girls	1975	14	9.01
Barbados	Girls	1976	14	9.09
Barbados	Girls	1977	14	9.16

Barbados	Girls	1978	14	9.24
Barbados	Girls	1979	14	9.33
Barbados	Girls	1980	14	9.42
Barbados	Girls	1981	14	9.50
Barbados	Girls	1982	14	9.58
Barbados	Girls	1983	14	9.66
Barbados	Girls	1984	14	9.73
Barbados	Girls	1985	14	9.80
Barbados	Girls	1986	14	9.87
Barbados	Girls	1987	14	9.94
Barbados	Girls	1988	14	10.02
Barbados	Girls	1989	14	10.09
Barbados	Girls	1990	14	10.17
Barbados	Girls	1991	14	10.26
Barbados	Girls	1992	14	10.37
Barbados	Girls	1993	14	10.48
Barbados	Girls	1994	14	10.61
Barbados	Girls	1995	14	10.75
Barbados	Girls	1996	14	10.90
Barbados	Girls	1997	14	11.05
Barbados	Girls	1998	14	11.22
Barbados	Girls	1999	14	11.39
Barbados	Girls	2000	14	11.56
Barbados	Girls	2001	14	11.75
Barbados	Girls	2002	14	11.93
Barbados	Girls	2003	14	12.12
Barbados	Girls	2004	14	12.31
Barbados	Girls	2005	14	12.50
Barbados	Girls	2006	14	12.70
Barbados	Girls	2007	14	12.91
Barbados	Girls	2008	14	13.12
Barbados	Girls	2009	14	13.34
Barbados	Girls	2010	14	13.57
Barbados	Girls	2011	14	13.81
Barbados	Girls	2012	14	14.05
Barbados	Girls	2013	14	14.31
Barbados	Girls	2014	14	14.57
Barbados	Girls	2015	14	14.85
Barbados	Girls	2016	14	15.13
Barbados	Girls	1975	15	10.43
Barbados	Girls	1976	15	10.51
Barbados	Girls	1977	15	10.59
Barbados	Girls	1978	15	10.67
Barbados	Girls	1979	15	10.76
Barbados	Girls	1980	15	10.86
Barbados	Girls	1981	15	10.95
Barbados	Girls	1982	15	11.03
Barbados	Girls	1983	15	11.11
Barbados	Girls	1984	15	11.18
Barbados	Girls	1985	15	11.26
Barbados	Girls	1986	15	11.33
Barbados	Girls	1987	15	11.40

Barbados	Girls	1988	15	11.48
Barbados	Girls	1989	15	11.56
Barbados	Girls	1990	15	11.64
Barbados	Girls	1991	15	11.74
Barbados	Girls	1992	15	11.85
Barbados	Girls	1993	15	11.97
Barbados	Girls	1994	15	12.10
Barbados	Girls	1995	15	12.24
Barbados	Girls	1996	15	12.40
Barbados	Girls	1997	15	12.56
Barbados	Girls	1998	15	12.73
Barbados	Girls	1999	15	12.91
Barbados	Girls	2000	15	13.09
Barbados	Girls	2001	15	13.28
Barbados	Girls	2002	15	13.47
Barbados	Girls	2003	15	13.67
Barbados	Girls	2004	15	13.87
Barbados	Girls	2005	15	14.07
Barbados	Girls	2006	15	14.28
Barbados	Girls	2007	15	14.49
Barbados	Girls	2008	15	14.72
Barbados	Girls	2009	15	14.95
Barbados	Girls	2010	15	15.19
Barbados	Girls	2011	15	15.44
Barbados	Girls	2012	15	15.70
Barbados	Girls	2013	15	15.97
Barbados	Girls	2014	15	16.25
Barbados	Girls	2015	15	16.54
Barbados	Girls	2016	15	16.84
Barbados	Girls	1975	16	11.58
Barbados	Girls	1976	16	11.66
Barbados	Girls	1977	16	11.74
Barbados	Girls	1978	16	11.83
Barbados	Girls	1979	16	11.92
Barbados	Girls	1980	16	12.02
Barbados	Girls	1981	16	12.11
Barbados	Girls	1982	16	12.19
Barbados	Girls	1983	16	12.28
Barbados	Girls	1984	16	12.35
Barbados	Girls	1985	16	12.43
Barbados	Girls	1986	16	12.51
Barbados	Girls	1987	16	12.58
Barbados	Girls	1988	16	12.66
Barbados	Girls	1989	16	12.74
Barbados	Girls	1990	16	12.83
Barbados	Girls	1991	16	12.92
Barbados	Girls	1992	16	13.04
Barbados	Girls	1993	16	13.16
Barbados	Girls	1994	16	13.30
Barbados	Girls	1995	16	13.45
Barbados	Girls	1996	16	13.61
Barbados	Girls	1997	16	13.77

Barbados	Girls	1998	16	13.95
Barbados	Girls	1999	16	14.13
Barbados	Girls	2000	16	14.32
Barbados	Girls	2001	16	14.52
Barbados	Girls	2002	16	14.72
Barbados	Girls	2003	16	14.92
Barbados	Girls	2004	16	15.12
Barbados	Girls	2005	16	15.33
Barbados	Girls	2006	16	15.55
Barbados	Girls	2007	16	15.77
Barbados	Girls	2008	16	16.00
Barbados	Girls	2009	16	16.24
Barbados	Girls	2010	16	16.49
Barbados	Girls	2011	16	16.75
Barbados	Girls	2012	16	17.02
Barbados	Girls	2013	16	17.30
Barbados	Girls	2014	16	17.59
Barbados	Girls	2015	16	17.89
Barbados	Girls	2016	16	18.21
Barbados	Girls	1975	17	12.41
Barbados	Girls	1976	17	12.50
Barbados	Girls	1977	17	12.59
Barbados	Girls	1978	17	12.68
Barbados	Girls	1979	17	12.78
Barbados	Girls	1980	17	12.88
Barbados	Girls	1981	17	12.97
Barbados	Girls	1982	17	13.06
Barbados	Girls	1983	17	13.15
Barbados	Girls	1984	17	13.23
Barbados	Girls	1985	17	13.31
Barbados	Girls	1986	17	13.39
Barbados	Girls	1987	17	13.47
Barbados	Girls	1988	17	13.55
Barbados	Girls	1989	17	13.64
Barbados	Girls	1990	17	13.73
Barbados	Girls	1991	17	13.83
Barbados	Girls	1992	17	13.95
Barbados	Girls	1993	17	14.08
Barbados	Girls	1994	17	14.23
Barbados	Girls	1995	17	14.39
Barbados	Girls	1996	17	14.55
Barbados	Girls	1997	17	14.73
Barbados	Girls	1998	17	14.91
Barbados	Girls	1999	17	15.11
Barbados	Girls	2000	17	15.31
Barbados	Girls	2001	17	15.51
Barbados	Girls	2002	17	15.72
Barbados	Girls	2003	17	15.93
Barbados	Girls	2004	17	16.15
Barbados	Girls	2005	17	16.37
Barbados	Girls	2006	17	16.60
Barbados	Girls	2007	17	16.83

Barbados	Girls	2008	17	17.08
Barbados	Girls	2009	17	17.33
Barbados	Girls	2010	17	17.59
Barbados	Girls	2011	17	17.86
Barbados	Girls	2012	17	18.14
Barbados	Girls	2013	17	18.44
Barbados	Girls	2014	17	18.74
Barbados	Girls	2015	17	19.06
Barbados	Girls	2016	17	19.39
Barbados	Girls	1975	18	13.05
Barbados	Girls	1976	18	13.14
Barbados	Girls	1977	18	13.24
Barbados	Girls	1978	18	13.34
Barbados	Girls	1979	18	13.45
Barbados	Girls	1980	18	13.56
Barbados	Girls	1981	18	13.67
Barbados	Girls	1982	18	13.77
Barbados	Girls	1983	18	13.86
Barbados	Girls	1984	18	13.95
Barbados	Girls	1985	18	14.04
Barbados	Girls	1986	18	14.13
Barbados	Girls	1987	18	14.22
Barbados	Girls	1988	18	14.31
Barbados	Girls	1989	18	14.40
Barbados	Girls	1990	18	14.50
Barbados	Girls	1991	18	14.61
Barbados	Girls	1992	18	14.75
Barbados	Girls	1993	18	14.89
Barbados	Girls	1994	18	15.05
Barbados	Girls	1995	18	15.22
Barbados	Girls	1996	18	15.41
Barbados	Girls	1997	18	15.60
Barbados	Girls	1998	18	15.81
Barbados	Girls	1999	18	16.02
Barbados	Girls	2000	18	16.24
Barbados	Girls	2001	18	16.47
Barbados	Girls	2002	18	16.69
Barbados	Girls	2003	18	16.93
Barbados	Girls	2004	18	17.16
Barbados	Girls	2005	18	17.41
Barbados	Girls	2006	18	17.65
Barbados	Girls	2007	18	17.91
Barbados	Girls	2008	18	18.17
Barbados	Girls	2009	18	18.45
Barbados	Girls	2010	18	18.73
Barbados	Girls	2011	18	19.02
Barbados	Girls	2012	18	19.33
Barbados	Girls	2013	18	19.64
Barbados	Girls	2014	18	19.97
Barbados	Girls	2015	18	20.30
Barbados	Girls	2016	18	20.65
Barbados	Girls	1975	Average	10.00

Barbados	Girls	1976	Average	10.08
Barbados	Girls	1977	Average	10.16
Barbados	Girls	1978	Average	10.24
Barbados	Girls	1979	Average	10.33
Barbados	Girls	1980	Average	10.42
Barbados	Girls	1981	Average	10.51
Barbados	Girls	1982	Average	10.59
Barbados	Girls	1983	Average	10.67
Barbados	Girls	1984	Average	10.74
Barbados	Girls	1985	Average	10.82
Barbados	Girls	1986	Average	10.89
Barbados	Girls	1987	Average	10.96
Barbados	Girls	1988	Average	11.04
Barbados	Girls	1989	Average	11.12
Barbados	Girls	1990	Average	11.20
Barbados	Girls	1991	Average	11.29
Barbados	Girls	1992	Average	11.40
Barbados	Girls	1993	Average	11.52
Barbados	Girls	1994	Average	11.65
Barbados	Girls	1995	Average	11.80
Barbados	Girls	1996	Average	11.95
Barbados	Girls	1997	Average	12.11
Barbados	Girls	1998	Average	12.28
Barbados	Girls	1999	Average	12.46
Barbados	Girls	2000	Average	12.64
Barbados	Girls	2001	Average	12.83
Barbados	Girls	2002	Average	13.02
Barbados	Girls	2003	Average	13.21
Barbados	Girls	2004	Average	13.41
Barbados	Girls	2005	Average	13.61
Barbados	Girls	2006	Average	13.82
Barbados	Girls	2007	Average	14.03
Barbados	Girls	2008	Average	14.25
Barbados	Girls	2009	Average	14.48
Barbados	Girls	2010	Average	14.71
Barbados	Girls	2011	Average	14.96
Barbados	Girls	2012	Average	15.21
Barbados	Girls	2013	Average	15.48
Barbados	Girls	2014	Average	15.75
Barbados	Girls	2015	Average	16.03
Barbados	Girls	2016	Average	16.32
Belarus	Boys	1975	12	5.95
Belarus	Boys	1976	12	6.04
Belarus	Boys	1977	12	6.13
Belarus	Boys	1978	12	6.23
Belarus	Boys	1979	12	6.33
Belarus	Boys	1980	12	6.44
Belarus	Boys	1981	12	6.55
Belarus	Boys	1982	12	6.66
Belarus	Boys	1983	12	6.78
Belarus	Boys	1984	12	6.90
Belarus	Boys	1985	12	7.03

Belarus	Boys	1986	12	7.15
Belarus	Boys	1987	12	7.28
Belarus	Boys	1988	12	7.41
Belarus	Boys	1989	12	7.55
Belarus	Boys	1990	12	7.68
Belarus	Boys	1991	12	7.81
Belarus	Boys	1992	12	7.94
Belarus	Boys	1993	12	8.06
Belarus	Boys	1994	12	8.19
Belarus	Boys	1995	12	8.31
Belarus	Boys	1996	12	8.43
Belarus	Boys	1997	12	8.55
Belarus	Boys	1998	12	8.67
Belarus	Boys	1999	12	8.80
Belarus	Boys	2000	12	8.93
Belarus	Boys	2001	12	9.06
Belarus	Boys	2002	12	9.20
Belarus	Boys	2003	12	9.34
Belarus	Boys	2004	12	9.49
Belarus	Boys	2005	12	9.64
Belarus	Boys	2006	12	9.80
Belarus	Boys	2007	12	9.96
Belarus	Boys	2008	12	10.13
Belarus	Boys	2009	12	10.29
Belarus	Boys	2010	12	10.46
Belarus	Boys	2011	12	10.64
Belarus	Boys	2012	12	10.81
Belarus	Boys	2013	12	10.99
Belarus	Boys	2014	12	11.17
Belarus	Boys	2015	12	11.36
Belarus	Boys	2016	12	11.54
Belarus	Boys	1975	13	7.39
Belarus	Boys	1976	13	7.49
Belarus	Boys	1977	13	7.60
Belarus	Boys	1978	13	7.71
Belarus	Boys	1979	13	7.83
Belarus	Boys	1980	13	7.96
Belarus	Boys	1981	13	8.09
Belarus	Boys	1982	13	8.22
Belarus	Boys	1983	13	8.36
Belarus	Boys	1984	13	8.50
Belarus	Boys	1985	13	8.65
Belarus	Boys	1986	13	8.80
Belarus	Boys	1987	13	8.95
Belarus	Boys	1988	13	9.10
Belarus	Boys	1989	13	9.26
Belarus	Boys	1990	13	9.42
Belarus	Boys	1991	13	9.57
Belarus	Boys	1992	13	9.72
Belarus	Boys	1993	13	9.87
Belarus	Boys	1994	13	10.01
Belarus	Boys	1995	13	10.15

Belarus	Boys	1996	13	10.29
Belarus	Boys	1997	13	10.43
Belarus	Boys	1998	13	10.58
Belarus	Boys	1999	13	10.73
Belarus	Boys	2000	13	10.88
Belarus	Boys	2001	13	11.04
Belarus	Boys	2002	13	11.20
Belarus	Boys	2003	13	11.37
Belarus	Boys	2004	13	11.55
Belarus	Boys	2005	13	11.73
Belarus	Boys	2006	13	11.91
Belarus	Boys	2007	13	12.11
Belarus	Boys	2008	13	12.30
Belarus	Boys	2009	13	12.50
Belarus	Boys	2010	13	12.71
Belarus	Boys	2011	13	12.91
Belarus	Boys	2012	13	13.13
Belarus	Boys	2013	13	13.34
Belarus	Boys	2014	13	13.56
Belarus	Boys	2015	13	13.79
Belarus	Boys	2016	13	14.01
Belarus	Boys	1975	14	9.17
Belarus	Boys	1976	14	9.28
Belarus	Boys	1977	14	9.40
Belarus	Boys	1978	14	9.53
Belarus	Boys	1979	14	9.66
Belarus	Boys	1980	14	9.80
Belarus	Boys	1981	14	9.95
Belarus	Boys	1982	14	10.10
Belarus	Boys	1983	14	10.25
Belarus	Boys	1984	14	10.41
Belarus	Boys	1985	14	10.57
Belarus	Boys	1986	14	10.74
Belarus	Boys	1987	14	10.91
Belarus	Boys	1988	14	11.08
Belarus	Boys	1989	14	11.25
Belarus	Boys	1990	14	11.43
Belarus	Boys	1991	14	11.60
Belarus	Boys	1992	14	11.76
Belarus	Boys	1993	14	11.92
Belarus	Boys	1994	14	12.08
Belarus	Boys	1995	14	12.24
Belarus	Boys	1996	14	12.39
Belarus	Boys	1997	14	12.55
Belarus	Boys	1998	14	12.71
Belarus	Boys	1999	14	12.88
Belarus	Boys	2000	14	13.04
Belarus	Boys	2001	14	13.22
Belarus	Boys	2002	14	13.40
Belarus	Boys	2003	14	13.59
Belarus	Boys	2004	14	13.79
Belarus	Boys	2005	14	13.99

Belarus	Boys	2006	14	14.20
Belarus	Boys	2007	14	14.41
Belarus	Boys	2008	14	14.63
Belarus	Boys	2009	14	14.86
Belarus	Boys	2010	14	15.09
Belarus	Boys	2011	14	15.33
Belarus	Boys	2012	14	15.57
Belarus	Boys	2013	14	15.81
Belarus	Boys	2014	14	16.07
Belarus	Boys	2015	14	16.32
Belarus	Boys	2016	14	16.58
Belarus	Boys	1975	15	11.21
Belarus	Boys	1976	15	11.33
Belarus	Boys	1977	15	11.46
Belarus	Boys	1978	15	11.60
Belarus	Boys	1979	15	11.74
Belarus	Boys	1980	15	11.89
Belarus	Boys	1981	15	12.04
Belarus	Boys	1982	15	12.20
Belarus	Boys	1983	15	12.36
Belarus	Boys	1984	15	12.53
Belarus	Boys	1985	15	12.70
Belarus	Boys	1986	15	12.88
Belarus	Boys	1987	15	13.06
Belarus	Boys	1988	15	13.24
Belarus	Boys	1989	15	13.42
Belarus	Boys	1990	15	13.60
Belarus	Boys	1991	15	13.78
Belarus	Boys	1992	15	13.96
Belarus	Boys	1993	15	14.13
Belarus	Boys	1994	15	14.29
Belarus	Boys	1995	15	14.46
Belarus	Boys	1996	15	14.62
Belarus	Boys	1997	15	14.79
Belarus	Boys	1998	15	14.96
Belarus	Boys	1999	15	15.13
Belarus	Boys	2000	15	15.31
Belarus	Boys	2001	15	15.49
Belarus	Boys	2002	15	15.69
Belarus	Boys	2003	15	15.89
Belarus	Boys	2004	15	16.09
Belarus	Boys	2005	15	16.31
Belarus	Boys	2006	15	16.53
Belarus	Boys	2007	15	16.76
Belarus	Boys	2008	15	16.99
Belarus	Boys	2009	15	17.23
Belarus	Boys	2010	15	17.48
Belarus	Boys	2011	15	17.73
Belarus	Boys	2012	15	17.99
Belarus	Boys	2013	15	18.25
Belarus	Boys	2014	15	18.52
Belarus	Boys	2015	15	18.80

Belarus	Boys	2016	15	19.09
Belarus	Boys	1975	16	13.43
Belarus	Boys	1976	16	13.56
Belarus	Boys	1977	16	13.69
Belarus	Boys	1978	16	13.83
Belarus	Boys	1979	16	13.98
Belarus	Boys	1980	16	14.14
Belarus	Boys	1981	16	14.29
Belarus	Boys	1982	16	14.45
Belarus	Boys	1983	16	14.62
Belarus	Boys	1984	16	14.79
Belarus	Boys	1985	16	14.97
Belarus	Boys	1986	16	15.15
Belarus	Boys	1987	16	15.33
Belarus	Boys	1988	16	15.52
Belarus	Boys	1989	16	15.71
Belarus	Boys	1990	16	15.90
Belarus	Boys	1991	16	16.08
Belarus	Boys	1992	16	16.26
Belarus	Boys	1993	16	16.43
Belarus	Boys	1994	16	16.60
Belarus	Boys	1995	16	16.77
Belarus	Boys	1996	16	16.94
Belarus	Boys	1997	16	17.11
Belarus	Boys	1998	16	17.28
Belarus	Boys	1999	16	17.46
Belarus	Boys	2000	16	17.64
Belarus	Boys	2001	16	17.83
Belarus	Boys	2002	16	18.02
Belarus	Boys	2003	16	18.23
Belarus	Boys	2004	16	18.44
Belarus	Boys	2005	16	18.66
Belarus	Boys	2006	16	18.89
Belarus	Boys	2007	16	19.12
Belarus	Boys	2008	16	19.36
Belarus	Boys	2009	16	19.61
Belarus	Boys	2010	16	19.86
Belarus	Boys	2011	16	20.12
Belarus	Boys	2012	16	20.39
Belarus	Boys	2013	16	20.67
Belarus	Boys	2014	16	20.95
Belarus	Boys	2015	16	21.24
Belarus	Boys	2016	16	21.53
Belarus	Boys	1975	17	15.73
Belarus	Boys	1976	17	15.86
Belarus	Boys	1977	17	16.00
Belarus	Boys	1978	17	16.15
Belarus	Boys	1979	17	16.30
Belarus	Boys	1980	17	16.46
Belarus	Boys	1981	17	16.62
Belarus	Boys	1982	17	16.79
Belarus	Boys	1983	17	16.96

Belarus	Boys	1984	17	17.13
Belarus	Boys	1985	17	17.31
Belarus	Boys	1986	17	17.50
Belarus	Boys	1987	17	17.68
Belarus	Boys	1988	17	17.87
Belarus	Boys	1989	17	18.06
Belarus	Boys	1990	17	18.26
Belarus	Boys	1991	17	18.44
Belarus	Boys	1992	17	18.62
Belarus	Boys	1993	17	18.80
Belarus	Boys	1994	17	18.97
Belarus	Boys	1995	17	19.14
Belarus	Boys	1996	17	19.31
Belarus	Boys	1997	17	19.49
Belarus	Boys	1998	17	19.66
Belarus	Boys	1999	17	19.84
Belarus	Boys	2000	17	20.03
Belarus	Boys	2001	17	20.22
Belarus	Boys	2002	17	20.42
Belarus	Boys	2003	17	20.63
Belarus	Boys	2004	17	20.84
Belarus	Boys	2005	17	21.07
Belarus	Boys	2006	17	21.30
Belarus	Boys	2007	17	21.54
Belarus	Boys	2008	17	21.78
Belarus	Boys	2009	17	22.03
Belarus	Boys	2010	17	22.29
Belarus	Boys	2011	17	22.55
Belarus	Boys	2012	17	22.83
Belarus	Boys	2013	17	23.11
Belarus	Boys	2014	17	23.40
Belarus	Boys	2015	17	23.69
Belarus	Boys	2016	17	24.00
Belarus	Boys	1975	18	18.04
Belarus	Boys	1976	18	18.18
Belarus	Boys	1977	18	18.33
Belarus	Boys	1978	18	18.48
Belarus	Boys	1979	18	18.64
Belarus	Boys	1980	18	18.81
Belarus	Boys	1981	18	18.98
Belarus	Boys	1982	18	19.15
Belarus	Boys	1983	18	19.33
Belarus	Boys	1984	18	19.51
Belarus	Boys	1985	18	19.70
Belarus	Boys	1986	18	19.89
Belarus	Boys	1987	18	20.09
Belarus	Boys	1988	18	20.28
Belarus	Boys	1989	18	20.48
Belarus	Boys	1990	18	20.68
Belarus	Boys	1991	18	20.87
Belarus	Boys	1992	18	21.06
Belarus	Boys	1993	18	21.25

Belarus	Boys	1994	18	21.42
Belarus	Boys	1995	18	21.60
Belarus	Boys	1996	18	21.78
Belarus	Boys	1997	18	21.96
Belarus	Boys	1998	18	22.14
Belarus	Boys	1999	18	22.32
Belarus	Boys	2000	18	22.51
Belarus	Boys	2001	18	22.71
Belarus	Boys	2002	18	22.92
Belarus	Boys	2003	18	23.13
Belarus	Boys	2004	18	23.36
Belarus	Boys	2005	18	23.59
Belarus	Boys	2006	18	23.83
Belarus	Boys	2007	18	24.07
Belarus	Boys	2008	18	24.33
Belarus	Boys	2009	18	24.58
Belarus	Boys	2010	18	24.85
Belarus	Boys	2011	18	25.12
Belarus	Boys	2012	18	25.40
Belarus	Boys	2013	18	25.69
Belarus	Boys	2014	18	25.99
Belarus	Boys	2015	18	26.29
Belarus	Boys	2016	18	26.61
Belarus	Boys	1975	Average	11.56
Belarus	Boys	1976	Average	11.68
Belarus	Boys	1977	Average	11.80
Belarus	Boys	1978	Average	11.93
Belarus	Boys	1979	Average	12.07
Belarus	Boys	1980	Average	12.21
Belarus	Boys	1981	Average	12.36
Belarus	Boys	1982	Average	12.51
Belarus	Boys	1983	Average	12.66
Belarus	Boys	1984	Average	12.82
Belarus	Boys	1985	Average	12.99
Belarus	Boys	1986	Average	13.16
Belarus	Boys	1987	Average	13.33
Belarus	Boys	1988	Average	13.50
Belarus	Boys	1989	Average	13.68
Belarus	Boys	1990	Average	13.85
Belarus	Boys	1991	Average	14.02
Belarus	Boys	1992	Average	14.19
Belarus	Boys	1993	Average	14.35
Belarus	Boys	1994	Average	14.51
Belarus	Boys	1995	Average	14.67
Belarus	Boys	1996	Average	14.82
Belarus	Boys	1997	Average	14.98
Belarus	Boys	1998	Average	15.14
Belarus	Boys	1999	Average	15.31
Belarus	Boys	2000	Average	15.48
Belarus	Boys	2001	Average	15.65
Belarus	Boys	2002	Average	15.84
Belarus	Boys	2003	Average	16.03

Belarus	Boys	2004	Average	16.22
Belarus	Boys	2005	Average	16.43
Belarus	Boys	2006	Average	16.64
Belarus	Boys	2007	Average	16.85
Belarus	Boys	2008	Average	17.08
Belarus	Boys	2009	Average	17.30
Belarus	Boys	2010	Average	17.53
Belarus	Boys	2011	Average	17.77
Belarus	Boys	2012	Average	18.02
Belarus	Boys	2013	Average	18.27
Belarus	Boys	2014	Average	18.52
Belarus	Boys	2015	Average	18.78
Belarus	Boys	2016	Average	19.05
Belarus	Girls	1975	12	5.57
Belarus	Girls	1976	12	5.64
Belarus	Girls	1977	12	5.71
Belarus	Girls	1978	12	5.78
Belarus	Girls	1979	12	5.86
Belarus	Girls	1980	12	5.93
Belarus	Girls	1981	12	6.01
Belarus	Girls	1982	12	6.09
Belarus	Girls	1983	12	6.17
Belarus	Girls	1984	12	6.25
Belarus	Girls	1985	12	6.34
Belarus	Girls	1986	12	6.42
Belarus	Girls	1987	12	6.50
Belarus	Girls	1988	12	6.59
Belarus	Girls	1989	12	6.67
Belarus	Girls	1990	12	6.75
Belarus	Girls	1991	12	6.83
Belarus	Girls	1992	12	6.91
Belarus	Girls	1993	12	6.98
Belarus	Girls	1994	12	7.05
Belarus	Girls	1995	12	7.12
Belarus	Girls	1996	12	7.19
Belarus	Girls	1997	12	7.26
Belarus	Girls	1998	12	7.33
Belarus	Girls	1999	12	7.40
Belarus	Girls	2000	12	7.46
Belarus	Girls	2001	12	7.54
Belarus	Girls	2002	12	7.61
Belarus	Girls	2003	12	7.68
Belarus	Girls	2004	12	7.76
Belarus	Girls	2005	12	7.84
Belarus	Girls	2006	12	7.92
Belarus	Girls	2007	12	7.99
Belarus	Girls	2008	12	8.07
Belarus	Girls	2009	12	8.15
Belarus	Girls	2010	12	8.23
Belarus	Girls	2011	12	8.31
Belarus	Girls	2012	12	8.39
Belarus	Girls	2013	12	8.47

Belarus	Girls	2014	12	8.55
Belarus	Girls	2015	12	8.63
Belarus	Girls	2016	12	8.71
Belarus	Girls	1975	13	6.95
Belarus	Girls	1976	13	7.03
Belarus	Girls	1977	13	7.11
Belarus	Girls	1978	13	7.19
Belarus	Girls	1979	13	7.28
Belarus	Girls	1980	13	7.37
Belarus	Girls	1981	13	7.46
Belarus	Girls	1982	13	7.55
Belarus	Girls	1983	13	7.64
Belarus	Girls	1984	13	7.74
Belarus	Girls	1985	13	7.83
Belarus	Girls	1986	13	7.93
Belarus	Girls	1987	13	8.02
Belarus	Girls	1988	13	8.12
Belarus	Girls	1989	13	8.22
Belarus	Girls	1990	13	8.31
Belarus	Girls	1991	13	8.40
Belarus	Girls	1992	13	8.49
Belarus	Girls	1993	13	8.57
Belarus	Girls	1994	13	8.65
Belarus	Girls	1995	13	8.73
Belarus	Girls	1996	13	8.81
Belarus	Girls	1997	13	8.89
Belarus	Girls	1998	13	8.96
Belarus	Girls	1999	13	9.04
Belarus	Girls	2000	13	9.12
Belarus	Girls	2001	13	9.20
Belarus	Girls	2002	13	9.29
Belarus	Girls	2003	13	9.37
Belarus	Girls	2004	13	9.46
Belarus	Girls	2005	13	9.55
Belarus	Girls	2006	13	9.63
Belarus	Girls	2007	13	9.72
Belarus	Girls	2008	13	9.81
Belarus	Girls	2009	13	9.90
Belarus	Girls	2010	13	9.99
Belarus	Girls	2011	13	10.08
Belarus	Girls	2012	13	10.17
Belarus	Girls	2013	13	10.27
Belarus	Girls	2014	13	10.36
Belarus	Girls	2015	13	10.45
Belarus	Girls	2016	13	10.55
Belarus	Girls	1975	14	8.44
Belarus	Girls	1976	14	8.53
Belarus	Girls	1977	14	8.62
Belarus	Girls	1978	14	8.71
Belarus	Girls	1979	14	8.81
Belarus	Girls	1980	14	8.91
Belarus	Girls	1981	14	9.00

Belarus	Girls	1982	14	9.10
Belarus	Girls	1983	14	9.21
Belarus	Girls	1984	14	9.31
Belarus	Girls	1985	14	9.41
Belarus	Girls	1986	14	9.52
Belarus	Girls	1987	14	9.62
Belarus	Girls	1988	14	9.73
Belarus	Girls	1989	14	9.83
Belarus	Girls	1990	14	9.93
Belarus	Girls	1991	14	10.03
Belarus	Girls	1992	14	10.13
Belarus	Girls	1993	14	10.22
Belarus	Girls	1994	14	10.30
Belarus	Girls	1995	14	10.39
Belarus	Girls	1996	14	10.47
Belarus	Girls	1997	14	10.56
Belarus	Girls	1998	14	10.64
Belarus	Girls	1999	14	10.72
Belarus	Girls	2000	14	10.81
Belarus	Girls	2001	14	10.90
Belarus	Girls	2002	14	10.99
Belarus	Girls	2003	14	11.08
Belarus	Girls	2004	14	11.17
Belarus	Girls	2005	14	11.27
Belarus	Girls	2006	14	11.36
Belarus	Girls	2007	14	11.46
Belarus	Girls	2008	14	11.56
Belarus	Girls	2009	14	11.65
Belarus	Girls	2010	14	11.75
Belarus	Girls	2011	14	11.85
Belarus	Girls	2012	14	11.95
Belarus	Girls	2013	14	12.05
Belarus	Girls	2014	14	12.15
Belarus	Girls	2015	14	12.25
Belarus	Girls	2016	14	12.36
Belarus	Girls	1975	15	9.84
Belarus	Girls	1976	15	9.93
Belarus	Girls	1977	15	10.02
Belarus	Girls	1978	15	10.12
Belarus	Girls	1979	15	10.22
Belarus	Girls	1980	15	10.33
Belarus	Girls	1981	15	10.43
Belarus	Girls	1982	15	10.54
Belarus	Girls	1983	15	10.64
Belarus	Girls	1984	15	10.75
Belarus	Girls	1985	15	10.86
Belarus	Girls	1986	15	10.97
Belarus	Girls	1987	15	11.08
Belarus	Girls	1988	15	11.19
Belarus	Girls	1989	15	11.30
Belarus	Girls	1990	15	11.41
Belarus	Girls	1991	15	11.51

Belarus	Girls	1992	15	11.61
Belarus	Girls	1993	15	11.70
Belarus	Girls	1994	15	11.79
Belarus	Girls	1995	15	11.88
Belarus	Girls	1996	15	11.97
Belarus	Girls	1997	15	12.06
Belarus	Girls	1998	15	12.14
Belarus	Girls	1999	15	12.23
Belarus	Girls	2000	15	12.32
Belarus	Girls	2001	15	12.41
Belarus	Girls	2002	15	12.50
Belarus	Girls	2003	15	12.60
Belarus	Girls	2004	15	12.70
Belarus	Girls	2005	15	12.80
Belarus	Girls	2006	15	12.89
Belarus	Girls	2007	15	12.99
Belarus	Girls	2008	15	13.09
Belarus	Girls	2009	15	13.20
Belarus	Girls	2010	15	13.30
Belarus	Girls	2011	15	13.40
Belarus	Girls	2012	15	13.50
Belarus	Girls	2013	15	13.61
Belarus	Girls	2014	15	13.71
Belarus	Girls	2015	15	13.82
Belarus	Girls	2016	15	13.93
Belarus	Girls	1975	16	10.96
Belarus	Girls	1976	16	11.06
Belarus	Girls	1977	16	11.16
Belarus	Girls	1978	16	11.26
Belarus	Girls	1979	16	11.37
Belarus	Girls	1980	16	11.47
Belarus	Girls	1981	16	11.58
Belarus	Girls	1982	16	11.69
Belarus	Girls	1983	16	11.80
Belarus	Girls	1984	16	11.91
Belarus	Girls	1985	16	12.03
Belarus	Girls	1986	16	12.14
Belarus	Girls	1987	16	12.25
Belarus	Girls	1988	16	12.36
Belarus	Girls	1989	16	12.47
Belarus	Girls	1990	16	12.58
Belarus	Girls	1991	16	12.69
Belarus	Girls	1992	16	12.79
Belarus	Girls	1993	16	12.89
Belarus	Girls	1994	16	12.98
Belarus	Girls	1995	16	13.07
Belarus	Girls	1996	16	13.16
Belarus	Girls	1997	16	13.25
Belarus	Girls	1998	16	13.34
Belarus	Girls	1999	16	13.43
Belarus	Girls	2000	16	13.52
Belarus	Girls	2001	16	13.61

Belarus	Girls	2002	16	13.71
Belarus	Girls	2003	16	13.81
Belarus	Girls	2004	16	13.91
Belarus	Girls	2005	16	14.01
Belarus	Girls	2006	16	14.11
Belarus	Girls	2007	16	14.21
Belarus	Girls	2008	16	14.32
Belarus	Girls	2009	16	14.42
Belarus	Girls	2010	16	14.52
Belarus	Girls	2011	16	14.63
Belarus	Girls	2012	16	14.74
Belarus	Girls	2013	16	14.84
Belarus	Girls	2014	16	14.95
Belarus	Girls	2015	16	15.06
Belarus	Girls	2016	16	15.17
Belarus	Girls	1975	17	11.77
Belarus	Girls	1976	17	11.87
Belarus	Girls	1977	17	11.98
Belarus	Girls	1978	17	12.08
Belarus	Girls	1979	17	12.19
Belarus	Girls	1980	17	12.30
Belarus	Girls	1981	17	12.41
Belarus	Girls	1982	17	12.53
Belarus	Girls	1983	17	12.64
Belarus	Girls	1984	17	12.76
Belarus	Girls	1985	17	12.88
Belarus	Girls	1986	17	13.00
Belarus	Girls	1987	17	13.11
Belarus	Girls	1988	17	13.23
Belarus	Girls	1989	17	13.35
Belarus	Girls	1990	17	13.46
Belarus	Girls	1991	17	13.57
Belarus	Girls	1992	17	13.68
Belarus	Girls	1993	17	13.78
Belarus	Girls	1994	17	13.88
Belarus	Girls	1995	17	13.97
Belarus	Girls	1996	17	14.07
Belarus	Girls	1997	17	14.16
Belarus	Girls	1998	17	14.25
Belarus	Girls	1999	17	14.34
Belarus	Girls	2000	17	14.44
Belarus	Girls	2001	17	14.53
Belarus	Girls	2002	17	14.63
Belarus	Girls	2003	17	14.73
Belarus	Girls	2004	17	14.84
Belarus	Girls	2005	17	14.94
Belarus	Girls	2006	17	15.05
Belarus	Girls	2007	17	15.16
Belarus	Girls	2008	17	15.26
Belarus	Girls	2009	17	15.37
Belarus	Girls	2010	17	15.48
Belarus	Girls	2011	17	15.59

Belarus	Girls	2012	17	15.70
Belarus	Girls	2013	17	15.81
Belarus	Girls	2014	17	15.92
Belarus	Girls	2015	17	16.04
Belarus	Girls	2016	17	16.15
Belarus	Girls	1975	18	12.35
Belarus	Girls	1976	18	12.46
Belarus	Girls	1977	18	12.57
Belarus	Girls	1978	18	12.68
Belarus	Girls	1979	18	12.80
Belarus	Girls	1980	18	12.92
Belarus	Girls	1981	18	13.04
Belarus	Girls	1982	18	13.16
Belarus	Girls	1983	18	13.29
Belarus	Girls	1984	18	13.41
Belarus	Girls	1985	18	13.54
Belarus	Girls	1986	18	13.67
Belarus	Girls	1987	18	13.80
Belarus	Girls	1988	18	13.92
Belarus	Girls	1989	18	14.05
Belarus	Girls	1990	18	14.17
Belarus	Girls	1991	18	14.29
Belarus	Girls	1992	18	14.41
Belarus	Girls	1993	18	14.52
Belarus	Girls	1994	18	14.63
Belarus	Girls	1995	18	14.73
Belarus	Girls	1996	18	14.83
Belarus	Girls	1997	18	14.93
Belarus	Girls	1998	18	15.03
Belarus	Girls	1999	18	15.13
Belarus	Girls	2000	18	15.23
Belarus	Girls	2001	18	15.34
Belarus	Girls	2002	18	15.44
Belarus	Girls	2003	18	15.55
Belarus	Girls	2004	18	15.67
Belarus	Girls	2005	18	15.78
Belarus	Girls	2006	18	15.89
Belarus	Girls	2007	18	16.01
Belarus	Girls	2008	18	16.12
Belarus	Girls	2009	18	16.24
Belarus	Girls	2010	18	16.35
Belarus	Girls	2011	18	16.47
Belarus	Girls	2012	18	16.59
Belarus	Girls	2013	18	16.71
Belarus	Girls	2014	18	16.83
Belarus	Girls	2015	18	16.95
Belarus	Girls	2016	18	17.08
Belarus	Girls	1975	Average	9.41
Belarus	Girls	1976	Average	9.50
Belarus	Girls	1977	Average	9.60
Belarus	Girls	1978	Average	9.69
Belarus	Girls	1979	Average	9.79

Belarus	Girls	1980	Average	9.89
Belarus	Girls	1981	Average	9.99
Belarus	Girls	1982	Average	10.09
Belarus	Girls	1983	Average	10.20
Belarus	Girls	1984	Average	10.31
Belarus	Girls	1985	Average	10.41
Belarus	Girls	1986	Average	10.52
Belarus	Girls	1987	Average	10.63
Belarus	Girls	1988	Average	10.73
Belarus	Girls	1989	Average	10.84
Belarus	Girls	1990	Average	10.95
Belarus	Girls	1991	Average	11.05
Belarus	Girls	1992	Average	11.14
Belarus	Girls	1993	Average	11.24
Belarus	Girls	1994	Average	11.33
Belarus	Girls	1995	Average	11.41
Belarus	Girls	1996	Average	11.50
Belarus	Girls	1997	Average	11.59
Belarus	Girls	1998	Average	11.67
Belarus	Girls	1999	Average	11.76
Belarus	Girls	2000	Average	11.84
Belarus	Girls	2001	Average	11.93
Belarus	Girls	2002	Average	12.02
Belarus	Girls	2003	Average	12.12
Belarus	Girls	2004	Average	12.21
Belarus	Girls	2005	Average	12.31
Belarus	Girls	2006	Average	12.41
Belarus	Girls	2007	Average	12.51
Belarus	Girls	2008	Average	12.60
Belarus	Girls	2009	Average	12.70
Belarus	Girls	2010	Average	12.80
Belarus	Girls	2011	Average	12.90
Belarus	Girls	2012	Average	13.00
Belarus	Girls	2013	Average	13.11
Belarus	Girls	2014	Average	13.21
Belarus	Girls	2015	Average	13.32
Belarus	Girls	2016	Average	13.42
Belgium	Boys	1975	12	9.35
Belgium	Boys	1976	12	9.44
Belgium	Boys	1977	12	9.53
Belgium	Boys	1978	12	9.63
Belgium	Boys	1979	12	9.72
Belgium	Boys	1980	12	9.82
Belgium	Boys	1981	12	9.92
Belgium	Boys	1982	12	10.02
Belgium	Boys	1983	12	10.12
Belgium	Boys	1984	12	10.22
Belgium	Boys	1985	12	10.32
Belgium	Boys	1986	12	10.42
Belgium	Boys	1987	12	10.51
Belgium	Boys	1988	12	10.60
Belgium	Boys	1989	12	10.69

Belgium	Boys	1990	12	10.77
Belgium	Boys	1991	12	10.85
Belgium	Boys	1992	12	10.92
Belgium	Boys	1993	12	10.99
Belgium	Boys	1994	12	11.05
Belgium	Boys	1995	12	11.10
Belgium	Boys	1996	12	11.15
Belgium	Boys	1997	12	11.19
Belgium	Boys	1998	12	11.22
Belgium	Boys	1999	12	11.25
Belgium	Boys	2000	12	11.26
Belgium	Boys	2001	12	11.28
Belgium	Boys	2002	12	11.28
Belgium	Boys	2003	12	11.28
Belgium	Boys	2004	12	11.27
Belgium	Boys	2005	12	11.26
Belgium	Boys	2006	12	11.25
Belgium	Boys	2007	12	11.23
Belgium	Boys	2008	12	11.20
Belgium	Boys	2009	12	11.17
Belgium	Boys	2010	12	11.15
Belgium	Boys	2011	12	11.12
Belgium	Boys	2012	12	11.09
Belgium	Boys	2013	12	11.06
Belgium	Boys	2014	12	11.03
Belgium	Boys	2015	12	11.00
Belgium	Boys	2016	12	10.97
Belgium	Boys	1975	13	11.52
Belgium	Boys	1976	13	11.62
Belgium	Boys	1977	13	11.73
Belgium	Boys	1978	13	11.84
Belgium	Boys	1979	13	11.96
Belgium	Boys	1980	13	12.08
Belgium	Boys	1981	13	12.20
Belgium	Boys	1982	13	12.32
Belgium	Boys	1983	13	12.44
Belgium	Boys	1984	13	12.56
Belgium	Boys	1985	13	12.68
Belgium	Boys	1986	13	12.79
Belgium	Boys	1987	13	12.90
Belgium	Boys	1988	13	13.01
Belgium	Boys	1989	13	13.12
Belgium	Boys	1990	13	13.22
Belgium	Boys	1991	13	13.31
Belgium	Boys	1992	13	13.40
Belgium	Boys	1993	13	13.48
Belgium	Boys	1994	13	13.55
Belgium	Boys	1995	13	13.61
Belgium	Boys	1996	13	13.67
Belgium	Boys	1997	13	13.71
Belgium	Boys	1998	13	13.75
Belgium	Boys	1999	13	13.78

Belgium	Boys	2000	13	13.80
Belgium	Boys	2001	13	13.81
Belgium	Boys	2002	13	13.81
Belgium	Boys	2003	13	13.81
Belgium	Boys	2004	13	13.79
Belgium	Boys	2005	13	13.77
Belgium	Boys	2006	13	13.75
Belgium	Boys	2007	13	13.72
Belgium	Boys	2008	13	13.69
Belgium	Boys	2009	13	13.65
Belgium	Boys	2010	13	13.61
Belgium	Boys	2011	13	13.57
Belgium	Boys	2012	13	13.53
Belgium	Boys	2013	13	13.48
Belgium	Boys	2014	13	13.44
Belgium	Boys	2015	13	13.41
Belgium	Boys	2016	13	13.37
Belgium	Boys	1975	14	13.92
Belgium	Boys	1976	14	14.03
Belgium	Boys	1977	14	14.16
Belgium	Boys	1978	14	14.29
Belgium	Boys	1979	14	14.42
Belgium	Boys	1980	14	14.55
Belgium	Boys	1981	14	14.69
Belgium	Boys	1982	14	14.83
Belgium	Boys	1983	14	14.96
Belgium	Boys	1984	14	15.10
Belgium	Boys	1985	14	15.23
Belgium	Boys	1986	14	15.36
Belgium	Boys	1987	14	15.49
Belgium	Boys	1988	14	15.62
Belgium	Boys	1989	14	15.73
Belgium	Boys	1990	14	15.85
Belgium	Boys	1991	14	15.95
Belgium	Boys	1992	14	16.05
Belgium	Boys	1993	14	16.14
Belgium	Boys	1994	14	16.22
Belgium	Boys	1995	14	16.29
Belgium	Boys	1996	14	16.35
Belgium	Boys	1997	14	16.40
Belgium	Boys	1998	14	16.44
Belgium	Boys	1999	14	16.46
Belgium	Boys	2000	14	16.48
Belgium	Boys	2001	14	16.49
Belgium	Boys	2002	14	16.49
Belgium	Boys	2003	14	16.48
Belgium	Boys	2004	14	16.46
Belgium	Boys	2005	14	16.43
Belgium	Boys	2006	14	16.40
Belgium	Boys	2007	14	16.36
Belgium	Boys	2008	14	16.31
Belgium	Boys	2009	14	16.26

Belgium	Boys	2010	14	16.21
Belgium	Boys	2011	14	16.16
Belgium	Boys	2012	14	16.10
Belgium	Boys	2013	14	16.05
Belgium	Boys	2014	14	16.00
Belgium	Boys	2015	14	15.95
Belgium	Boys	2016	14	15.90
Belgium	Boys	1975	15	16.40
Belgium	Boys	1976	15	16.52
Belgium	Boys	1977	15	16.66
Belgium	Boys	1978	15	16.79
Belgium	Boys	1979	15	16.94
Belgium	Boys	1980	15	17.08
Belgium	Boys	1981	15	17.23
Belgium	Boys	1982	15	17.37
Belgium	Boys	1983	15	17.52
Belgium	Boys	1984	15	17.67
Belgium	Boys	1985	15	17.81
Belgium	Boys	1986	15	17.95
Belgium	Boys	1987	15	18.09
Belgium	Boys	1988	15	18.22
Belgium	Boys	1989	15	18.35
Belgium	Boys	1990	15	18.47
Belgium	Boys	1991	15	18.58
Belgium	Boys	1992	15	18.69
Belgium	Boys	1993	15	18.78
Belgium	Boys	1994	15	18.86
Belgium	Boys	1995	15	18.94
Belgium	Boys	1996	15	19.00
Belgium	Boys	1997	15	19.05
Belgium	Boys	1998	15	19.09
Belgium	Boys	1999	15	19.11
Belgium	Boys	2000	15	19.13
Belgium	Boys	2001	15	19.13
Belgium	Boys	2002	15	19.13
Belgium	Boys	2003	15	19.11
Belgium	Boys	2004	15	19.08
Belgium	Boys	2005	15	19.05
Belgium	Boys	2006	15	19.01
Belgium	Boys	2007	15	18.96
Belgium	Boys	2008	15	18.90
Belgium	Boys	2009	15	18.84
Belgium	Boys	2010	15	18.78
Belgium	Boys	2011	15	18.72
Belgium	Boys	2012	15	18.65
Belgium	Boys	2013	15	18.59
Belgium	Boys	2014	15	18.52
Belgium	Boys	2015	15	18.46
Belgium	Boys	2016	15	18.41
Belgium	Boys	1975	16	18.90
Belgium	Boys	1976	16	19.03
Belgium	Boys	1977	16	19.17

Belgium	Boys	1978	16	19.31
Belgium	Boys	1979	16	19.46
Belgium	Boys	1980	16	19.61
Belgium	Boys	1981	16	19.76
Belgium	Boys	1982	16	19.91
Belgium	Boys	1983	16	20.06
Belgium	Boys	1984	16	20.21
Belgium	Boys	1985	16	20.36
Belgium	Boys	1986	16	20.51
Belgium	Boys	1987	16	20.65
Belgium	Boys	1988	16	20.79
Belgium	Boys	1989	16	20.92
Belgium	Boys	1990	16	21.05
Belgium	Boys	1991	16	21.16
Belgium	Boys	1992	16	21.27
Belgium	Boys	1993	16	21.37
Belgium	Boys	1994	16	21.45
Belgium	Boys	1995	16	21.53
Belgium	Boys	1996	16	21.59
Belgium	Boys	1997	16	21.64
Belgium	Boys	1998	16	21.68
Belgium	Boys	1999	16	21.70
Belgium	Boys	2000	16	21.71
Belgium	Boys	2001	16	21.71
Belgium	Boys	2002	16	21.70
Belgium	Boys	2003	16	21.68
Belgium	Boys	2004	16	21.65
Belgium	Boys	2005	16	21.61
Belgium	Boys	2006	16	21.56
Belgium	Boys	2007	16	21.50
Belgium	Boys	2008	16	21.44
Belgium	Boys	2009	16	21.37
Belgium	Boys	2010	16	21.30
Belgium	Boys	2011	16	21.23
Belgium	Boys	2012	16	21.16
Belgium	Boys	2013	16	21.09
Belgium	Boys	2014	16	21.02
Belgium	Boys	2015	16	20.95
Belgium	Boys	2016	16	20.89
Belgium	Boys	1975	17	21.40
Belgium	Boys	1976	17	21.54
Belgium	Boys	1977	17	21.68
Belgium	Boys	1978	17	21.83
Belgium	Boys	1979	17	21.98
Belgium	Boys	1980	17	22.13
Belgium	Boys	1981	17	22.29
Belgium	Boys	1982	17	22.44
Belgium	Boys	1983	17	22.60
Belgium	Boys	1984	17	22.75
Belgium	Boys	1985	17	22.91
Belgium	Boys	1986	17	23.06
Belgium	Boys	1987	17	23.20

Belgium	Boys	1988	17	23.35
Belgium	Boys	1989	17	23.48
Belgium	Boys	1990	17	23.61
Belgium	Boys	1991	17	23.73
Belgium	Boys	1992	17	23.83
Belgium	Boys	1993	17	23.93
Belgium	Boys	1994	17	24.02
Belgium	Boys	1995	17	24.09
Belgium	Boys	1996	17	24.16
Belgium	Boys	1997	17	24.21
Belgium	Boys	1998	17	24.25
Belgium	Boys	1999	17	24.27
Belgium	Boys	2000	17	24.28
Belgium	Boys	2001	17	24.28
Belgium	Boys	2002	17	24.27
Belgium	Boys	2003	17	24.24
Belgium	Boys	2004	17	24.20
Belgium	Boys	2005	17	24.16
Belgium	Boys	2006	17	24.11
Belgium	Boys	2007	17	24.05
Belgium	Boys	2008	17	23.98
Belgium	Boys	2009	17	23.91
Belgium	Boys	2010	17	23.83
Belgium	Boys	2011	17	23.76
Belgium	Boys	2012	17	23.68
Belgium	Boys	2013	17	23.61
Belgium	Boys	2014	17	23.53
Belgium	Boys	2015	17	23.46
Belgium	Boys	2016	17	23.40
Belgium	Boys	1975	18	23.96
Belgium	Boys	1976	18	24.10
Belgium	Boys	1977	18	24.25
Belgium	Boys	1978	18	24.40
Belgium	Boys	1979	18	24.56
Belgium	Boys	1980	18	24.72
Belgium	Boys	1981	18	24.88
Belgium	Boys	1982	18	25.04
Belgium	Boys	1983	18	25.21
Belgium	Boys	1984	18	25.37
Belgium	Boys	1985	18	25.52
Belgium	Boys	1986	18	25.68
Belgium	Boys	1987	18	25.83
Belgium	Boys	1988	18	25.98
Belgium	Boys	1989	18	26.12
Belgium	Boys	1990	18	26.25
Belgium	Boys	1991	18	26.37
Belgium	Boys	1992	18	26.48
Belgium	Boys	1993	18	26.59
Belgium	Boys	1994	18	26.68
Belgium	Boys	1995	18	26.75
Belgium	Boys	1996	18	26.82
Belgium	Boys	1997	18	26.87

Belgium	Boys	1998	18	26.91
Belgium	Boys	1999	18	26.93
Belgium	Boys	2000	18	26.95
Belgium	Boys	2001	18	26.94
Belgium	Boys	2002	18	26.93
Belgium	Boys	2003	18	26.90
Belgium	Boys	2004	18	26.87
Belgium	Boys	2005	18	26.82
Belgium	Boys	2006	18	26.77
Belgium	Boys	2007	18	26.70
Belgium	Boys	2008	18	26.64
Belgium	Boys	2009	18	26.56
Belgium	Boys	2010	18	26.49
Belgium	Boys	2011	18	26.41
Belgium	Boys	2012	18	26.33
Belgium	Boys	2013	18	26.25
Belgium	Boys	2014	18	26.18
Belgium	Boys	2015	18	26.11
Belgium	Boys	2016	18	26.04
Belgium	Boys	1975	Average	16.49
Belgium	Boys	1976	Average	16.61
Belgium	Boys	1977	Average	16.74
Belgium	Boys	1978	Average	16.87
Belgium	Boys	1979	Average	17.00
Belgium	Boys	1980	Average	17.14
Belgium	Boys	1981	Average	17.28
Belgium	Boys	1982	Average	17.42
Belgium	Boys	1983	Average	17.56
Belgium	Boys	1984	Average	17.70
Belgium	Boys	1985	Average	17.83
Belgium	Boys	1986	Average	17.97
Belgium	Boys	1987	Average	18.10
Belgium	Boys	1988	Average	18.22
Belgium	Boys	1989	Average	18.35
Belgium	Boys	1990	Average	18.46
Belgium	Boys	1991	Average	18.57
Belgium	Boys	1992	Average	18.66
Belgium	Boys	1993	Average	18.75
Belgium	Boys	1994	Average	18.83
Belgium	Boys	1995	Average	18.90
Belgium	Boys	1996	Average	18.96
Belgium	Boys	1997	Average	19.01
Belgium	Boys	1998	Average	19.05
Belgium	Boys	1999	Average	19.07
Belgium	Boys	2000	Average	19.09
Belgium	Boys	2001	Average	19.09
Belgium	Boys	2002	Average	19.09
Belgium	Boys	2003	Average	19.07
Belgium	Boys	2004	Average	19.05
Belgium	Boys	2005	Average	19.01
Belgium	Boys	2006	Average	18.98
Belgium	Boys	2007	Average	18.93

Belgium	Boys	2008	Average	18.88
Belgium	Boys	2009	Average	18.82
Belgium	Boys	2010	Average	18.77
Belgium	Boys	2011	Average	18.71
Belgium	Boys	2012	Average	18.65
Belgium	Boys	2013	Average	18.59
Belgium	Boys	2014	Average	18.53
Belgium	Boys	2015	Average	18.48
Belgium	Boys	2016	Average	18.43
Belgium	Girls	1975	12	9.37
Belgium	Girls	1976	12	9.41
Belgium	Girls	1977	12	9.46
Belgium	Girls	1978	12	9.51
Belgium	Girls	1979	12	9.56
Belgium	Girls	1980	12	9.61
Belgium	Girls	1981	12	9.66
Belgium	Girls	1982	12	9.71
Belgium	Girls	1983	12	9.76
Belgium	Girls	1984	12	9.81
Belgium	Girls	1985	12	9.86
Belgium	Girls	1986	12	9.91
Belgium	Girls	1987	12	9.95
Belgium	Girls	1988	12	9.99
Belgium	Girls	1989	12	10.03
Belgium	Girls	1990	12	10.07
Belgium	Girls	1991	12	10.11
Belgium	Girls	1992	12	10.14
Belgium	Girls	1993	12	10.18
Belgium	Girls	1994	12	10.20
Belgium	Girls	1995	12	10.23
Belgium	Girls	1996	12	10.25
Belgium	Girls	1997	12	10.26
Belgium	Girls	1998	12	10.27
Belgium	Girls	1999	12	10.28
Belgium	Girls	2000	12	10.28
Belgium	Girls	2001	12	10.28
Belgium	Girls	2002	12	10.28
Belgium	Girls	2003	12	10.27
Belgium	Girls	2004	12	10.26
Belgium	Girls	2005	12	10.25
Belgium	Girls	2006	12	10.24
Belgium	Girls	2007	12	10.22
Belgium	Girls	2008	12	10.20
Belgium	Girls	2009	12	10.18
Belgium	Girls	2010	12	10.17
Belgium	Girls	2011	12	10.15
Belgium	Girls	2012	12	10.13
Belgium	Girls	2013	12	10.11
Belgium	Girls	2014	12	10.10
Belgium	Girls	2015	12	10.09
Belgium	Girls	2016	12	10.08
Belgium	Girls	1975	13	11.36

Belgium	Girls	1976	13	11.42
Belgium	Girls	1977	13	11.47
Belgium	Girls	1978	13	11.53
Belgium	Girls	1979	13	11.59
Belgium	Girls	1980	13	11.65
Belgium	Girls	1981	13	11.71
Belgium	Girls	1982	13	11.76
Belgium	Girls	1983	13	11.82
Belgium	Girls	1984	13	11.88
Belgium	Girls	1985	13	11.93
Belgium	Girls	1986	13	11.99
Belgium	Girls	1987	13	12.04
Belgium	Girls	1988	13	12.09
Belgium	Girls	1989	13	12.14
Belgium	Girls	1990	13	12.18
Belgium	Girls	1991	13	12.23
Belgium	Girls	1992	13	12.26
Belgium	Girls	1993	13	12.30
Belgium	Girls	1994	13	12.33
Belgium	Girls	1995	13	12.36
Belgium	Girls	1996	13	12.38
Belgium	Girls	1997	13	12.39
Belgium	Girls	1998	13	12.41
Belgium	Girls	1999	13	12.41
Belgium	Girls	2000	13	12.42
Belgium	Girls	2001	13	12.42
Belgium	Girls	2002	13	12.41
Belgium	Girls	2003	13	12.40
Belgium	Girls	2004	13	12.39
Belgium	Girls	2005	13	12.37
Belgium	Girls	2006	13	12.35
Belgium	Girls	2007	13	12.33
Belgium	Girls	2008	13	12.30
Belgium	Girls	2009	13	12.28
Belgium	Girls	2010	13	12.26
Belgium	Girls	2011	13	12.23
Belgium	Girls	2012	13	12.21
Belgium	Girls	2013	13	12.19
Belgium	Girls	2014	13	12.17
Belgium	Girls	2015	13	12.15
Belgium	Girls	2016	13	12.14
Belgium	Girls	1975	14	13.31
Belgium	Girls	1976	14	13.37
Belgium	Girls	1977	14	13.43
Belgium	Girls	1978	14	13.49
Belgium	Girls	1979	14	13.56
Belgium	Girls	1980	14	13.62
Belgium	Girls	1981	14	13.69
Belgium	Girls	1982	14	13.75
Belgium	Girls	1983	14	13.81
Belgium	Girls	1984	14	13.88
Belgium	Girls	1985	14	13.94

Belgium	Girls	1986	14	14.00
Belgium	Girls	1987	14	14.05
Belgium	Girls	1988	14	14.11
Belgium	Girls	1989	14	14.16
Belgium	Girls	1990	14	14.21
Belgium	Girls	1991	14	14.25
Belgium	Girls	1992	14	14.30
Belgium	Girls	1993	14	14.33
Belgium	Girls	1994	14	14.37
Belgium	Girls	1995	14	14.40
Belgium	Girls	1996	14	14.42
Belgium	Girls	1997	14	14.43
Belgium	Girls	1998	14	14.45
Belgium	Girls	1999	14	14.45
Belgium	Girls	2000	14	14.45
Belgium	Girls	2001	14	14.45
Belgium	Girls	2002	14	14.44
Belgium	Girls	2003	14	14.43
Belgium	Girls	2004	14	14.41
Belgium	Girls	2005	14	14.39
Belgium	Girls	2006	14	14.37
Belgium	Girls	2007	14	14.34
Belgium	Girls	2008	14	14.31
Belgium	Girls	2009	14	14.28
Belgium	Girls	2010	14	14.25
Belgium	Girls	2011	14	14.23
Belgium	Girls	2012	14	14.20
Belgium	Girls	2013	14	14.17
Belgium	Girls	2014	14	14.15
Belgium	Girls	2015	14	14.13
Belgium	Girls	2016	14	14.11
Belgium	Girls	1975	15	14.98
Belgium	Girls	1976	15	15.04
Belgium	Girls	1977	15	15.11
Belgium	Girls	1978	15	15.17
Belgium	Girls	1979	15	15.24
Belgium	Girls	1980	15	15.31
Belgium	Girls	1981	15	15.38
Belgium	Girls	1982	15	15.44
Belgium	Girls	1983	15	15.51
Belgium	Girls	1984	15	15.57
Belgium	Girls	1985	15	15.64
Belgium	Girls	1986	15	15.70
Belgium	Girls	1987	15	15.76
Belgium	Girls	1988	15	15.82
Belgium	Girls	1989	15	15.87
Belgium	Girls	1990	15	15.92
Belgium	Girls	1991	15	15.97
Belgium	Girls	1992	15	16.01
Belgium	Girls	1993	15	16.05
Belgium	Girls	1994	15	16.09
Belgium	Girls	1995	15	16.12

Belgium	Girls	1996	15	16.14
Belgium	Girls	1997	15	16.15
Belgium	Girls	1998	15	16.17
Belgium	Girls	1999	15	16.17
Belgium	Girls	2000	15	16.17
Belgium	Girls	2001	15	16.17
Belgium	Girls	2002	15	16.16
Belgium	Girls	2003	15	16.14
Belgium	Girls	2004	15	16.12
Belgium	Girls	2005	15	16.10
Belgium	Girls	2006	15	16.07
Belgium	Girls	2007	15	16.04
Belgium	Girls	2008	15	16.01
Belgium	Girls	2009	15	15.97
Belgium	Girls	2010	15	15.94
Belgium	Girls	2011	15	15.91
Belgium	Girls	2012	15	15.88
Belgium	Girls	2013	15	15.85
Belgium	Girls	2014	15	15.82
Belgium	Girls	2015	15	15.80
Belgium	Girls	2016	15	15.77
Belgium	Girls	1975	16	16.28
Belgium	Girls	1976	16	16.34
Belgium	Girls	1977	16	16.40
Belgium	Girls	1978	16	16.47
Belgium	Girls	1979	16	16.54
Belgium	Girls	1980	16	16.61
Belgium	Girls	1981	16	16.68
Belgium	Girls	1982	16	16.75
Belgium	Girls	1983	16	16.82
Belgium	Girls	1984	16	16.89
Belgium	Girls	1985	16	16.95
Belgium	Girls	1986	16	17.02
Belgium	Girls	1987	16	17.08
Belgium	Girls	1988	16	17.14
Belgium	Girls	1989	16	17.19
Belgium	Girls	1990	16	17.24
Belgium	Girls	1991	16	17.29
Belgium	Girls	1992	16	17.34
Belgium	Girls	1993	16	17.38
Belgium	Girls	1994	16	17.41
Belgium	Girls	1995	16	17.44
Belgium	Girls	1996	16	17.46
Belgium	Girls	1997	16	17.48
Belgium	Girls	1998	16	17.49
Belgium	Girls	1999	16	17.50
Belgium	Girls	2000	16	17.50
Belgium	Girls	2001	16	17.49
Belgium	Girls	2002	16	17.48
Belgium	Girls	2003	16	17.46
Belgium	Girls	2004	16	17.44
Belgium	Girls	2005	16	17.41

Belgium	Girls	2006	16	17.38
Belgium	Girls	2007	16	17.35
Belgium	Girls	2008	16	17.31
Belgium	Girls	2009	16	17.28
Belgium	Girls	2010	16	17.24
Belgium	Girls	2011	16	17.21
Belgium	Girls	2012	16	17.18
Belgium	Girls	2013	16	17.15
Belgium	Girls	2014	16	17.12
Belgium	Girls	2015	16	17.09
Belgium	Girls	2016	16	17.07
Belgium	Girls	1975	17	17.27
Belgium	Girls	1976	17	17.34
Belgium	Girls	1977	17	17.40
Belgium	Girls	1978	17	17.47
Belgium	Girls	1979	17	17.55
Belgium	Girls	1980	17	17.62
Belgium	Girls	1981	17	17.69
Belgium	Girls	1982	17	17.76
Belgium	Girls	1983	17	17.83
Belgium	Girls	1984	17	17.90
Belgium	Girls	1985	17	17.97
Belgium	Girls	1986	17	18.04
Belgium	Girls	1987	17	18.10
Belgium	Girls	1988	17	18.16
Belgium	Girls	1989	17	18.22
Belgium	Girls	1990	17	18.28
Belgium	Girls	1991	17	18.33
Belgium	Girls	1992	17	18.37
Belgium	Girls	1993	17	18.41
Belgium	Girls	1994	17	18.45
Belgium	Girls	1995	17	18.48
Belgium	Girls	1996	17	18.50
Belgium	Girls	1997	17	18.52
Belgium	Girls	1998	17	18.53
Belgium	Girls	1999	17	18.54
Belgium	Girls	2000	17	18.53
Belgium	Girls	2001	17	18.53
Belgium	Girls	2002	17	18.51
Belgium	Girls	2003	17	18.50
Belgium	Girls	2004	17	18.47
Belgium	Girls	2005	17	18.45
Belgium	Girls	2006	17	18.42
Belgium	Girls	2007	17	18.38
Belgium	Girls	2008	17	18.35
Belgium	Girls	2009	17	18.31
Belgium	Girls	2010	17	18.28
Belgium	Girls	2011	17	18.24
Belgium	Girls	2012	17	18.21
Belgium	Girls	2013	17	18.18
Belgium	Girls	2014	17	18.15
Belgium	Girls	2015	17	18.12

Belgium	Girls	2016	17	18.10
Belgium	Girls	1975	18	18.20
Belgium	Girls	1976	18	18.26
Belgium	Girls	1977	18	18.33
Belgium	Girls	1978	18	18.41
Belgium	Girls	1979	18	18.48
Belgium	Girls	1980	18	18.56
Belgium	Girls	1981	18	18.64
Belgium	Girls	1982	18	18.72
Belgium	Girls	1983	18	18.79
Belgium	Girls	1984	18	18.87
Belgium	Girls	1985	18	18.94
Belgium	Girls	1986	18	19.01
Belgium	Girls	1987	18	19.08
Belgium	Girls	1988	18	19.14
Belgium	Girls	1989	18	19.20
Belgium	Girls	1990	18	19.26
Belgium	Girls	1991	18	19.32
Belgium	Girls	1992	18	19.36
Belgium	Girls	1993	18	19.41
Belgium	Girls	1994	18	19.45
Belgium	Girls	1995	18	19.48
Belgium	Girls	1996	18	19.51
Belgium	Girls	1997	18	19.52
Belgium	Girls	1998	18	19.54
Belgium	Girls	1999	18	19.54
Belgium	Girls	2000	18	19.55
Belgium	Girls	2001	18	19.54
Belgium	Girls	2002	18	19.53
Belgium	Girls	2003	18	19.51
Belgium	Girls	2004	18	19.49
Belgium	Girls	2005	18	19.46
Belgium	Girls	2006	18	19.43
Belgium	Girls	2007	18	19.40
Belgium	Girls	2008	18	19.36
Belgium	Girls	2009	18	19.33
Belgium	Girls	2010	18	19.29
Belgium	Girls	2011	18	19.26
Belgium	Girls	2012	18	19.23
Belgium	Girls	2013	18	19.19
Belgium	Girls	2014	18	19.17
Belgium	Girls	2015	18	19.14
Belgium	Girls	2016	18	19.12
Belgium	Girls	1975	Average	14.40
Belgium	Girls	1976	Average	14.45
Belgium	Girls	1977	Average	14.52
Belgium	Girls	1978	Average	14.58
Belgium	Girls	1979	Average	14.64
Belgium	Girls	1980	Average	14.71
Belgium	Girls	1981	Average	14.78
Belgium	Girls	1982	Average	14.84
Belgium	Girls	1983	Average	14.91

Belgium	Girls	1984	Average	14.97
Belgium	Girls	1985	Average	15.03
Belgium	Girls	1986	Average	15.09
Belgium	Girls	1987	Average	15.15
Belgium	Girls	1988	Average	15.21
Belgium	Girls	1989	Average	15.26
Belgium	Girls	1990	Average	15.31
Belgium	Girls	1991	Average	15.36
Belgium	Girls	1992	Average	15.40
Belgium	Girls	1993	Average	15.44
Belgium	Girls	1994	Average	15.47
Belgium	Girls	1995	Average	15.50
Belgium	Girls	1996	Average	15.52
Belgium	Girls	1997	Average	15.54
Belgium	Girls	1998	Average	15.55
Belgium	Girls	1999	Average	15.56
Belgium	Girls	2000	Average	15.56
Belgium	Girls	2001	Average	15.55
Belgium	Girls	2002	Average	15.54
Belgium	Girls	2003	Average	15.53
Belgium	Girls	2004	Average	15.51
Belgium	Girls	2005	Average	15.49
Belgium	Girls	2006	Average	15.46
Belgium	Girls	2007	Average	15.44
Belgium	Girls	2008	Average	15.41
Belgium	Girls	2009	Average	15.38
Belgium	Girls	2010	Average	15.35
Belgium	Girls	2011	Average	15.32
Belgium	Girls	2012	Average	15.29
Belgium	Girls	2013	Average	15.26
Belgium	Girls	2014	Average	15.24
Belgium	Girls	2015	Average	15.22
Belgium	Girls	2016	Average	15.20
Belize	Boys	1975	12	6.29
Belize	Boys	1976	12	6.33
Belize	Boys	1977	12	6.38
Belize	Boys	1978	12	6.43
Belize	Boys	1979	12	6.49
Belize	Boys	1980	12	6.55
Belize	Boys	1981	12	6.61
Belize	Boys	1982	12	6.68
Belize	Boys	1983	12	6.75
Belize	Boys	1984	12	6.83
Belize	Boys	1985	12	6.91
Belize	Boys	1986	12	7.00
Belize	Boys	1987	12	7.09
Belize	Boys	1988	12	7.18
Belize	Boys	1989	12	7.28
Belize	Boys	1990	12	7.38
Belize	Boys	1991	12	7.49
Belize	Boys	1992	12	7.61
Belize	Boys	1993	12	7.73

Belize	Boys	1994	12	7.86
Belize	Boys	1995	12	8.00
Belize	Boys	1996	12	8.14
Belize	Boys	1997	12	8.29
Belize	Boys	1998	12	8.45
Belize	Boys	1999	12	8.61
Belize	Boys	2000	12	8.78
Belize	Boys	2001	12	8.95
Belize	Boys	2002	12	9.13
Belize	Boys	2003	12	9.30
Belize	Boys	2004	12	9.49
Belize	Boys	2005	12	9.67
Belize	Boys	2006	12	9.86
Belize	Boys	2007	12	10.05
Belize	Boys	2008	12	10.25
Belize	Boys	2009	12	10.46
Belize	Boys	2010	12	10.67
Belize	Boys	2011	12	10.88
Belize	Boys	2012	12	11.10
Belize	Boys	2013	12	11.33
Belize	Boys	2014	12	11.57
Belize	Boys	2015	12	11.80
Belize	Boys	2016	12	12.05
Belize	Boys	1975	13	7.74
Belize	Boys	1976	13	7.79
Belize	Boys	1977	13	7.84
Belize	Boys	1978	13	7.90
Belize	Boys	1979	13	7.97
Belize	Boys	1980	13	8.04
Belize	Boys	1981	13	8.11
Belize	Boys	1982	13	8.19
Belize	Boys	1983	13	8.27
Belize	Boys	1984	13	8.36
Belize	Boys	1985	13	8.45
Belize	Boys	1986	13	8.55
Belize	Boys	1987	13	8.65
Belize	Boys	1988	13	8.76
Belize	Boys	1989	13	8.87
Belize	Boys	1990	13	8.99
Belize	Boys	1991	13	9.12
Belize	Boys	1992	13	9.25
Belize	Boys	1993	13	9.40
Belize	Boys	1994	13	9.55
Belize	Boys	1995	13	9.71
Belize	Boys	1996	13	9.87
Belize	Boys	1997	13	10.05
Belize	Boys	1998	13	10.23
Belize	Boys	1999	13	10.42
Belize	Boys	2000	13	10.62
Belize	Boys	2001	13	10.82
Belize	Boys	2002	13	11.03
Belize	Boys	2003	13	11.24

Belize	Boys	2004	13	11.45
Belize	Boys	2005	13	11.67
Belize	Boys	2006	13	11.90
Belize	Boys	2007	13	12.13
Belize	Boys	2008	13	12.37
Belize	Boys	2009	13	12.61
Belize	Boys	2010	13	12.87
Belize	Boys	2011	13	13.13
Belize	Boys	2012	13	13.40
Belize	Boys	2013	13	13.68
Belize	Boys	2014	13	13.97
Belize	Boys	2015	13	14.26
Belize	Boys	2016	13	14.57
Belize	Boys	1975	14	9.49
Belize	Boys	1976	14	9.55
Belize	Boys	1977	14	9.61
Belize	Boys	1978	14	9.67
Belize	Boys	1979	14	9.74
Belize	Boys	1980	14	9.82
Belize	Boys	1981	14	9.90
Belize	Boys	1982	14	9.99
Belize	Boys	1983	14	10.08
Belize	Boys	1984	14	10.17
Belize	Boys	1985	14	10.27
Belize	Boys	1986	14	10.38
Belize	Boys	1987	14	10.49
Belize	Boys	1988	14	10.61
Belize	Boys	1989	14	10.73
Belize	Boys	1990	14	10.86
Belize	Boys	1991	14	11.00
Belize	Boys	1992	14	11.14
Belize	Boys	1993	14	11.30
Belize	Boys	1994	14	11.46
Belize	Boys	1995	14	11.64
Belize	Boys	1996	14	11.82
Belize	Boys	1997	14	12.01
Belize	Boys	1998	14	12.21
Belize	Boys	1999	14	12.42
Belize	Boys	2000	14	12.64
Belize	Boys	2001	14	12.87
Belize	Boys	2002	14	13.10
Belize	Boys	2003	14	13.33
Belize	Boys	2004	14	13.57
Belize	Boys	2005	14	13.81
Belize	Boys	2006	14	14.06
Belize	Boys	2007	14	14.32
Belize	Boys	2008	14	14.59
Belize	Boys	2009	14	14.87
Belize	Boys	2010	14	15.16
Belize	Boys	2011	14	15.46
Belize	Boys	2012	14	15.76
Belize	Boys	2013	14	16.08

Belize	Boys	2014	14	16.42
Belize	Boys	2015	14	16.76
Belize	Boys	2016	14	17.11
Belize	Boys	1975	15	11.48
Belize	Boys	1976	15	11.54
Belize	Boys	1977	15	11.60
Belize	Boys	1978	15	11.67
Belize	Boys	1979	15	11.74
Belize	Boys	1980	15	11.82
Belize	Boys	1981	15	11.90
Belize	Boys	1982	15	11.99
Belize	Boys	1983	15	12.09
Belize	Boys	1984	15	12.18
Belize	Boys	1985	15	12.29
Belize	Boys	1986	15	12.40
Belize	Boys	1987	15	12.51
Belize	Boys	1988	15	12.63
Belize	Boys	1989	15	12.76
Belize	Boys	1990	15	12.90
Belize	Boys	1991	15	13.04
Belize	Boys	1992	15	13.19
Belize	Boys	1993	15	13.35
Belize	Boys	1994	15	13.52
Belize	Boys	1995	15	13.70
Belize	Boys	1996	15	13.89
Belize	Boys	1997	15	14.09
Belize	Boys	1998	15	14.30
Belize	Boys	1999	15	14.52
Belize	Boys	2000	15	14.75
Belize	Boys	2001	15	14.99
Belize	Boys	2002	15	15.23
Belize	Boys	2003	15	15.47
Belize	Boys	2004	15	15.72
Belize	Boys	2005	15	15.98
Belize	Boys	2006	15	16.25
Belize	Boys	2007	15	16.52
Belize	Boys	2008	15	16.81
Belize	Boys	2009	15	17.10
Belize	Boys	2010	15	17.41
Belize	Boys	2011	15	17.73
Belize	Boys	2012	15	18.06
Belize	Boys	2013	15	18.41
Belize	Boys	2014	15	18.77
Belize	Boys	2015	15	19.14
Belize	Boys	2016	15	19.53
Belize	Boys	1975	16	13.62
Belize	Boys	1976	16	13.68
Belize	Boys	1977	16	13.74
Belize	Boys	1978	16	13.82
Belize	Boys	1979	16	13.89
Belize	Boys	1980	16	13.97
Belize	Boys	1981	16	14.06

Belize	Boys	1982	16	14.15
Belize	Boys	1983	16	14.24
Belize	Boys	1984	16	14.34
Belize	Boys	1985	16	14.45
Belize	Boys	1986	16	14.56
Belize	Boys	1987	16	14.67
Belize	Boys	1988	16	14.80
Belize	Boys	1989	16	14.93
Belize	Boys	1990	16	15.06
Belize	Boys	1991	16	15.20
Belize	Boys	1992	16	15.36
Belize	Boys	1993	16	15.52
Belize	Boys	1994	16	15.70
Belize	Boys	1995	16	15.88
Belize	Boys	1996	16	16.07
Belize	Boys	1997	16	16.27
Belize	Boys	1998	16	16.48
Belize	Boys	1999	16	16.71
Belize	Boys	2000	16	16.94
Belize	Boys	2001	16	17.18
Belize	Boys	2002	16	17.42
Belize	Boys	2003	16	17.67
Belize	Boys	2004	16	17.93
Belize	Boys	2005	16	18.19
Belize	Boys	2006	16	18.46
Belize	Boys	2007	16	18.74
Belize	Boys	2008	16	19.04
Belize	Boys	2009	16	19.34
Belize	Boys	2010	16	19.66
Belize	Boys	2011	16	19.99
Belize	Boys	2012	16	20.33
Belize	Boys	2013	16	20.69
Belize	Boys	2014	16	21.07
Belize	Boys	2015	16	21.46
Belize	Boys	2016	16	21.86
Belize	Boys	1975	17	15.84
Belize	Boys	1976	17	15.91
Belize	Boys	1977	17	15.97
Belize	Boys	1978	17	16.05
Belize	Boys	1979	17	16.12
Belize	Boys	1980	17	16.21
Belize	Boys	1981	17	16.29
Belize	Boys	1982	17	16.38
Belize	Boys	1983	17	16.48
Belize	Boys	1984	17	16.58
Belize	Boys	1985	17	16.69
Belize	Boys	1986	17	16.80
Belize	Boys	1987	17	16.92
Belize	Boys	1988	17	17.04
Belize	Boys	1989	17	17.17
Belize	Boys	1990	17	17.31
Belize	Boys	1991	17	17.45

Belize	Boys	1992	17	17.61
Belize	Boys	1993	17	17.77
Belize	Boys	1994	17	17.95
Belize	Boys	1995	17	18.13
Belize	Boys	1996	17	18.33
Belize	Boys	1997	17	18.53
Belize	Boys	1998	17	18.74
Belize	Boys	1999	17	18.97
Belize	Boys	2000	17	19.20
Belize	Boys	2001	17	19.45
Belize	Boys	2002	17	19.69
Belize	Boys	2003	17	19.94
Belize	Boys	2004	17	20.20
Belize	Boys	2005	17	20.47
Belize	Boys	2006	17	20.74
Belize	Boys	2007	17	21.03
Belize	Boys	2008	17	21.33
Belize	Boys	2009	17	21.64
Belize	Boys	2010	17	21.96
Belize	Boys	2011	17	22.30
Belize	Boys	2012	17	22.65
Belize	Boys	2013	17	23.02
Belize	Boys	2014	17	23.40
Belize	Boys	2015	17	23.80
Belize	Boys	2016	17	24.22
Belize	Boys	1975	18	18.10
Belize	Boys	1976	18	18.16
Belize	Boys	1977	18	18.23
Belize	Boys	1978	18	18.31
Belize	Boys	1979	18	18.39
Belize	Boys	1980	18	18.48
Belize	Boys	1981	18	18.57
Belize	Boys	1982	18	18.67
Belize	Boys	1983	18	18.77
Belize	Boys	1984	18	18.87
Belize	Boys	1985	18	18.98
Belize	Boys	1986	18	19.10
Belize	Boys	1987	18	19.22
Belize	Boys	1988	18	19.35
Belize	Boys	1989	18	19.48
Belize	Boys	1990	18	19.63
Belize	Boys	1991	18	19.78
Belize	Boys	1992	18	19.94
Belize	Boys	1993	18	20.11
Belize	Boys	1994	18	20.29
Belize	Boys	1995	18	20.48
Belize	Boys	1996	18	20.68
Belize	Boys	1997	18	20.89
Belize	Boys	1998	18	21.11
Belize	Boys	1999	18	21.34
Belize	Boys	2000	18	21.59
Belize	Boys	2001	18	21.83

Belize	Boys	2002	18	22.09
Belize	Boys	2003	18	22.35
Belize	Boys	2004	18	22.62
Belize	Boys	2005	18	22.89
Belize	Boys	2006	18	23.17
Belize	Boys	2007	18	23.47
Belize	Boys	2008	18	23.78
Belize	Boys	2009	18	24.09
Belize	Boys	2010	18	24.43
Belize	Boys	2011	18	24.77
Belize	Boys	2012	18	25.14
Belize	Boys	2013	18	25.52
Belize	Boys	2014	18	25.91
Belize	Boys	2015	18	26.32
Belize	Boys	2016	18	26.75
Belize	Boys	1975	Average	11.79
Belize	Boys	1976	Average	11.85
Belize	Boys	1977	Average	11.91
Belize	Boys	1978	Average	11.98
Belize	Boys	1979	Average	12.05
Belize	Boys	1980	Average	12.13
Belize	Boys	1981	Average	12.21
Belize	Boys	1982	Average	12.29
Belize	Boys	1983	Average	12.38
Belize	Boys	1984	Average	12.48
Belize	Boys	1985	Average	12.58
Belize	Boys	1986	Average	12.68
Belize	Boys	1987	Average	12.79
Belize	Boys	1988	Average	12.91
Belize	Boys	1989	Average	13.03
Belize	Boys	1990	Average	13.16
Belize	Boys	1991	Average	13.30
Belize	Boys	1992	Average	13.44
Belize	Boys	1993	Average	13.60
Belize	Boys	1994	Average	13.76
Belize	Boys	1995	Average	13.93
Belize	Boys	1996	Average	14.11
Belize	Boys	1997	Average	14.30
Belize	Boys	1998	Average	14.50
Belize	Boys	1999	Average	14.71
Belize	Boys	2000	Average	14.93
Belize	Boys	2001	Average	15.16
Belize	Boys	2002	Average	15.38
Belize	Boys	2003	Average	15.62
Belize	Boys	2004	Average	15.85
Belize	Boys	2005	Average	16.10
Belize	Boys	2006	Average	16.35
Belize	Boys	2007	Average	16.61
Belize	Boys	2008	Average	16.88
Belize	Boys	2009	Average	17.16
Belize	Boys	2010	Average	17.45
Belize	Boys	2011	Average	17.75

Belize	Boys	2012	Average	18.06
Belize	Boys	2013	Average	18.39
Belize	Boys	2014	Average	18.73
Belize	Boys	2015	Average	19.08
Belize	Boys	2016	Average	19.44
Belize	Girls	1975	12	6.63
Belize	Girls	1976	12	6.67
Belize	Girls	1977	12	6.72
Belize	Girls	1978	12	6.77
Belize	Girls	1979	12	6.82
Belize	Girls	1980	12	6.88
Belize	Girls	1981	12	6.94
Belize	Girls	1982	12	7.00
Belize	Girls	1983	12	7.06
Belize	Girls	1984	12	7.13
Belize	Girls	1985	12	7.20
Belize	Girls	1986	12	7.27
Belize	Girls	1987	12	7.35
Belize	Girls	1988	12	7.43
Belize	Girls	1989	12	7.51
Belize	Girls	1990	12	7.60
Belize	Girls	1991	12	7.69
Belize	Girls	1992	12	7.79
Belize	Girls	1993	12	7.89
Belize	Girls	1994	12	7.99
Belize	Girls	1995	12	8.10
Belize	Girls	1996	12	8.21
Belize	Girls	1997	12	8.33
Belize	Girls	1998	12	8.45
Belize	Girls	1999	12	8.58
Belize	Girls	2000	12	8.71
Belize	Girls	2001	12	8.84
Belize	Girls	2002	12	8.96
Belize	Girls	2003	12	9.09
Belize	Girls	2004	12	9.22
Belize	Girls	2005	12	9.35
Belize	Girls	2006	12	9.49
Belize	Girls	2007	12	9.62
Belize	Girls	2008	12	9.76
Belize	Girls	2009	12	9.90
Belize	Girls	2010	12	10.05
Belize	Girls	2011	12	10.19
Belize	Girls	2012	12	10.35
Belize	Girls	2013	12	10.51
Belize	Girls	2014	12	10.67
Belize	Girls	2015	12	10.84
Belize	Girls	2016	12	11.01
Belize	Girls	1975	13	8.16
Belize	Girls	1976	13	8.21
Belize	Girls	1977	13	8.26
Belize	Girls	1978	13	8.32
Belize	Girls	1979	13	8.38

Belize	Girls	1980	13	8.44
Belize	Girls	1981	13	8.51
Belize	Girls	1982	13	8.58
Belize	Girls	1983	13	8.65
Belize	Girls	1984	13	8.73
Belize	Girls	1985	13	8.81
Belize	Girls	1986	13	8.89
Belize	Girls	1987	13	8.98
Belize	Girls	1988	13	9.07
Belize	Girls	1989	13	9.16
Belize	Girls	1990	13	9.26
Belize	Girls	1991	13	9.36
Belize	Girls	1992	13	9.47
Belize	Girls	1993	13	9.59
Belize	Girls	1994	13	9.71
Belize	Girls	1995	13	9.83
Belize	Girls	1996	13	9.96
Belize	Girls	1997	13	10.09
Belize	Girls	1998	13	10.23
Belize	Girls	1999	13	10.37
Belize	Girls	2000	13	10.52
Belize	Girls	2001	13	10.66
Belize	Girls	2002	13	10.81
Belize	Girls	2003	13	10.96
Belize	Girls	2004	13	11.11
Belize	Girls	2005	13	11.26
Belize	Girls	2006	13	11.41
Belize	Girls	2007	13	11.56
Belize	Girls	2008	13	11.72
Belize	Girls	2009	13	11.88
Belize	Girls	2010	13	12.05
Belize	Girls	2011	13	12.22
Belize	Girls	2012	13	12.40
Belize	Girls	2013	13	12.59
Belize	Girls	2014	13	12.78
Belize	Girls	2015	13	12.98
Belize	Girls	2016	13	13.18
Belize	Girls	1975	14	9.76
Belize	Girls	1976	14	9.81
Belize	Girls	1977	14	9.87
Belize	Girls	1978	14	9.93
Belize	Girls	1979	14	10.00
Belize	Girls	1980	14	10.07
Belize	Girls	1981	14	10.14
Belize	Girls	1982	14	10.22
Belize	Girls	1983	14	10.29
Belize	Girls	1984	14	10.38
Belize	Girls	1985	14	10.46
Belize	Girls	1986	14	10.55
Belize	Girls	1987	14	10.64
Belize	Girls	1988	14	10.74
Belize	Girls	1989	14	10.84

Belize	Girls	1990	14	10.95
Belize	Girls	1991	14	11.06
Belize	Girls	1992	14	11.18
Belize	Girls	1993	14	11.30
Belize	Girls	1994	14	11.43
Belize	Girls	1995	14	11.56
Belize	Girls	1996	14	11.70
Belize	Girls	1997	14	11.84
Belize	Girls	1998	14	11.99
Belize	Girls	1999	14	12.14
Belize	Girls	2000	14	12.30
Belize	Girls	2001	14	12.46
Belize	Girls	2002	14	12.62
Belize	Girls	2003	14	12.78
Belize	Girls	2004	14	12.94
Belize	Girls	2005	14	13.10
Belize	Girls	2006	14	13.26
Belize	Girls	2007	14	13.43
Belize	Girls	2008	14	13.60
Belize	Girls	2009	14	13.78
Belize	Girls	2010	14	13.96
Belize	Girls	2011	14	14.15
Belize	Girls	2012	14	14.35
Belize	Girls	2013	14	14.56
Belize	Girls	2014	14	14.77
Belize	Girls	2015	14	14.99
Belize	Girls	2016	14	15.21
Belize	Girls	1975	15	11.22
Belize	Girls	1976	15	11.27
Belize	Girls	1977	15	11.33
Belize	Girls	1978	15	11.40
Belize	Girls	1979	15	11.47
Belize	Girls	1980	15	11.54
Belize	Girls	1981	15	11.61
Belize	Girls	1982	15	11.69
Belize	Girls	1983	15	11.77
Belize	Girls	1984	15	11.86
Belize	Girls	1985	15	11.95
Belize	Girls	1986	15	12.04
Belize	Girls	1987	15	12.14
Belize	Girls	1988	15	12.24
Belize	Girls	1989	15	12.34
Belize	Girls	1990	15	12.45
Belize	Girls	1991	15	12.57
Belize	Girls	1992	15	12.69
Belize	Girls	1993	15	12.82
Belize	Girls	1994	15	12.95
Belize	Girls	1995	15	13.09
Belize	Girls	1996	15	13.23
Belize	Girls	1997	15	13.38
Belize	Girls	1998	15	13.54
Belize	Girls	1999	15	13.70

Belize	Girls	2000	15	13.86
Belize	Girls	2001	15	14.03
Belize	Girls	2002	15	14.19
Belize	Girls	2003	15	14.36
Belize	Girls	2004	15	14.52
Belize	Girls	2005	15	14.69
Belize	Girls	2006	15	14.86
Belize	Girls	2007	15	15.04
Belize	Girls	2008	15	15.22
Belize	Girls	2009	15	15.41
Belize	Girls	2010	15	15.60
Belize	Girls	2011	15	15.80
Belize	Girls	2012	15	16.01
Belize	Girls	2013	15	16.22
Belize	Girls	2014	15	16.45
Belize	Girls	2015	15	16.68
Belize	Girls	2016	15	16.92
Belize	Girls	1975	16	12.39
Belize	Girls	1976	16	12.44
Belize	Girls	1977	16	12.51
Belize	Girls	1978	16	12.57
Belize	Girls	1979	16	12.64
Belize	Girls	1980	16	12.72
Belize	Girls	1981	16	12.79
Belize	Girls	1982	16	12.88
Belize	Girls	1983	16	12.96
Belize	Girls	1984	16	13.05
Belize	Girls	1985	16	13.14
Belize	Girls	1986	16	13.23
Belize	Girls	1987	16	13.33
Belize	Girls	1988	16	13.44
Belize	Girls	1989	16	13.55
Belize	Girls	1990	16	13.66
Belize	Girls	1991	16	13.78
Belize	Girls	1992	16	13.90
Belize	Girls	1993	16	14.04
Belize	Girls	1994	16	14.17
Belize	Girls	1995	16	14.32
Belize	Girls	1996	16	14.47
Belize	Girls	1997	16	14.62
Belize	Girls	1998	16	14.78
Belize	Girls	1999	16	14.94
Belize	Girls	2000	16	15.11
Belize	Girls	2001	16	15.28
Belize	Girls	2002	16	15.45
Belize	Girls	2003	16	15.62
Belize	Girls	2004	16	15.79
Belize	Girls	2005	16	15.97
Belize	Girls	2006	16	16.15
Belize	Girls	2007	16	16.33
Belize	Girls	2008	16	16.52
Belize	Girls	2009	16	16.71

Belize	Girls	2010	16	16.91
Belize	Girls	2011	16	17.12
Belize	Girls	2012	16	17.33
Belize	Girls	2013	16	17.56
Belize	Girls	2014	16	17.79
Belize	Girls	2015	16	18.03
Belize	Girls	2016	16	18.28
Belize	Girls	1975	17	13.26
Belize	Girls	1976	17	13.32
Belize	Girls	1977	17	13.38
Belize	Girls	1978	17	13.45
Belize	Girls	1979	17	13.53
Belize	Girls	1980	17	13.60
Belize	Girls	1981	17	13.69
Belize	Girls	1982	17	13.77
Belize	Girls	1983	17	13.86
Belize	Girls	1984	17	13.95
Belize	Girls	1985	17	14.05
Belize	Girls	1986	17	14.15
Belize	Girls	1987	17	14.25
Belize	Girls	1988	17	14.36
Belize	Girls	1989	17	14.48
Belize	Girls	1990	17	14.59
Belize	Girls	1991	17	14.72
Belize	Girls	1992	17	14.85
Belize	Girls	1993	17	14.99
Belize	Girls	1994	17	15.14
Belize	Girls	1995	17	15.29
Belize	Girls	1996	17	15.44
Belize	Girls	1997	17	15.60
Belize	Girls	1998	17	15.77
Belize	Girls	1999	17	15.94
Belize	Girls	2000	17	16.12
Belize	Girls	2001	17	16.30
Belize	Girls	2002	17	16.48
Belize	Girls	2003	17	16.66
Belize	Girls	2004	17	16.84
Belize	Girls	2005	17	17.02
Belize	Girls	2006	17	17.21
Belize	Girls	2007	17	17.40
Belize	Girls	2008	17	17.60
Belize	Girls	2009	17	17.80
Belize	Girls	2010	17	18.01
Belize	Girls	2011	17	18.23
Belize	Girls	2012	17	18.45
Belize	Girls	2013	17	18.69
Belize	Girls	2014	17	18.93
Belize	Girls	2015	17	19.18
Belize	Girls	2016	17	19.45
Belize	Girls	1975	18	13.96
Belize	Girls	1976	18	14.03
Belize	Girls	1977	18	14.10

Belize	Girls	1978	18	14.18
Belize	Girls	1979	18	14.26
Belize	Girls	1980	18	14.34
Belize	Girls	1981	18	14.43
Belize	Girls	1982	18	14.52
Belize	Girls	1983	18	14.62
Belize	Girls	1984	18	14.72
Belize	Girls	1985	18	14.83
Belize	Girls	1986	18	14.94
Belize	Girls	1987	18	15.05
Belize	Girls	1988	18	15.17
Belize	Girls	1989	18	15.30
Belize	Girls	1990	18	15.43
Belize	Girls	1991	18	15.57
Belize	Girls	1992	18	15.71
Belize	Girls	1993	18	15.86
Belize	Girls	1994	18	16.02
Belize	Girls	1995	18	16.19
Belize	Girls	1996	18	16.36
Belize	Girls	1997	18	16.54
Belize	Girls	1998	18	16.72
Belize	Girls	1999	18	16.91
Belize	Girls	2000	18	17.10
Belize	Girls	2001	18	17.30
Belize	Girls	2002	18	17.49
Belize	Girls	2003	18	17.69
Belize	Girls	2004	18	17.89
Belize	Girls	2005	18	18.09
Belize	Girls	2006	18	18.29
Belize	Girls	2007	18	18.50
Belize	Girls	2008	18	18.71
Belize	Girls	2009	18	18.93
Belize	Girls	2010	18	19.15
Belize	Girls	2011	18	19.39
Belize	Girls	2012	18	19.63
Belize	Girls	2013	18	19.88
Belize	Girls	2014	18	20.14
Belize	Girls	2015	18	20.41
Belize	Girls	2016	18	20.68
Belize	Girls	1975	Average	10.77
Belize	Girls	1976	Average	10.82
Belize	Girls	1977	Average	10.88
Belize	Girls	1978	Average	10.95
Belize	Girls	1979	Average	11.01
Belize	Girls	1980	Average	11.08
Belize	Girls	1981	Average	11.16
Belize	Girls	1982	Average	11.24
Belize	Girls	1983	Average	11.32
Belize	Girls	1984	Average	11.40
Belize	Girls	1985	Average	11.49
Belize	Girls	1986	Average	11.58
Belize	Girls	1987	Average	11.68

Belize	Girls	1988	Average	11.78
Belize	Girls	1989	Average	11.88
Belize	Girls	1990	Average	11.99
Belize	Girls	1991	Average	12.11
Belize	Girls	1992	Average	12.23
Belize	Girls	1993	Average	12.35
Belize	Girls	1994	Average	12.49
Belize	Girls	1995	Average	12.62
Belize	Girls	1996	Average	12.77
Belize	Girls	1997	Average	12.92
Belize	Girls	1998	Average	13.07
Belize	Girls	1999	Average	13.23
Belize	Girls	2000	Average	13.39
Belize	Girls	2001	Average	13.55
Belize	Girls	2002	Average	13.71
Belize	Girls	2003	Average	13.88
Belize	Girls	2004	Average	14.04
Belize	Girls	2005	Average	14.21
Belize	Girls	2006	Average	14.38
Belize	Girls	2007	Average	14.55
Belize	Girls	2008	Average	14.73
Belize	Girls	2009	Average	14.92
Belize	Girls	2010	Average	15.10
Belize	Girls	2011	Average	15.30
Belize	Girls	2012	Average	15.50
Belize	Girls	2013	Average	15.71
Belize	Girls	2014	Average	15.93
Belize	Girls	2015	Average	16.16
Belize	Girls	2016	Average	16.39
Benin	Boys	1975	12	3.98
Benin	Boys	1976	12	4.01
Benin	Boys	1977	12	4.03
Benin	Boys	1978	12	4.05
Benin	Boys	1979	12	4.08
Benin	Boys	1980	12	4.10
Benin	Boys	1981	12	4.13
Benin	Boys	1982	12	4.15
Benin	Boys	1983	12	4.18
Benin	Boys	1984	12	4.21
Benin	Boys	1985	12	4.24
Benin	Boys	1986	12	4.27
Benin	Boys	1987	12	4.30
Benin	Boys	1988	12	4.33
Benin	Boys	1989	12	4.37
Benin	Boys	1990	12	4.41
Benin	Boys	1991	12	4.44
Benin	Boys	1992	12	4.48
Benin	Boys	1993	12	4.52
Benin	Boys	1994	12	4.57
Benin	Boys	1995	12	4.61
Benin	Boys	1996	12	4.66
Benin	Boys	1997	12	4.70

Benin	Boys	1998	12	4.75
Benin	Boys	1999	12	4.80
Benin	Boys	2000	12	4.86
Benin	Boys	2001	12	4.91
Benin	Boys	2002	12	4.97
Benin	Boys	2003	12	5.03
Benin	Boys	2004	12	5.09
Benin	Boys	2005	12	5.16
Benin	Boys	2006	12	5.23
Benin	Boys	2007	12	5.30
Benin	Boys	2008	12	5.38
Benin	Boys	2009	12	5.46
Benin	Boys	2010	12	5.54
Benin	Boys	2011	12	5.63
Benin	Boys	2012	12	5.72
Benin	Boys	2013	12	5.81
Benin	Boys	2014	12	5.91
Benin	Boys	2015	12	6.01
Benin	Boys	2016	12	6.12
Benin	Boys	1975	13	4.98
Benin	Boys	1976	13	5.01
Benin	Boys	1977	13	5.04
Benin	Boys	1978	13	5.07
Benin	Boys	1979	13	5.10
Benin	Boys	1980	13	5.13
Benin	Boys	1981	13	5.16
Benin	Boys	1982	13	5.19
Benin	Boys	1983	13	5.23
Benin	Boys	1984	13	5.26
Benin	Boys	1985	13	5.30
Benin	Boys	1986	13	5.33
Benin	Boys	1987	13	5.37
Benin	Boys	1988	13	5.41
Benin	Boys	1989	13	5.45
Benin	Boys	1990	13	5.50
Benin	Boys	1991	13	5.54
Benin	Boys	1992	13	5.59
Benin	Boys	1993	13	5.64
Benin	Boys	1994	13	5.69
Benin	Boys	1995	13	5.74
Benin	Boys	1996	13	5.79
Benin	Boys	1997	13	5.85
Benin	Boys	1998	13	5.91
Benin	Boys	1999	13	5.97
Benin	Boys	2000	13	6.03
Benin	Boys	2001	13	6.10
Benin	Boys	2002	13	6.16
Benin	Boys	2003	13	6.24
Benin	Boys	2004	13	6.31
Benin	Boys	2005	13	6.39
Benin	Boys	2006	13	6.47
Benin	Boys	2007	13	6.56

Benin	Boys	2008	13	6.65
Benin	Boys	2009	13	6.74
Benin	Boys	2010	13	6.84
Benin	Boys	2011	13	6.94
Benin	Boys	2012	13	7.04
Benin	Boys	2013	13	7.15
Benin	Boys	2014	13	7.27
Benin	Boys	2015	13	7.39
Benin	Boys	2016	13	7.52
Benin	Boys	1975	14	6.30
Benin	Boys	1976	14	6.34
Benin	Boys	1977	14	6.37
Benin	Boys	1978	14	6.40
Benin	Boys	1979	14	6.44
Benin	Boys	1980	14	6.48
Benin	Boys	1981	14	6.52
Benin	Boys	1982	14	6.56
Benin	Boys	1983	14	6.60
Benin	Boys	1984	14	6.64
Benin	Boys	1985	14	6.68
Benin	Boys	1986	14	6.72
Benin	Boys	1987	14	6.77
Benin	Boys	1988	14	6.81
Benin	Boys	1989	14	6.86
Benin	Boys	1990	14	6.91
Benin	Boys	1991	14	6.97
Benin	Boys	1992	14	7.02
Benin	Boys	1993	14	7.08
Benin	Boys	1994	14	7.13
Benin	Boys	1995	14	7.19
Benin	Boys	1996	14	7.26
Benin	Boys	1997	14	7.32
Benin	Boys	1998	14	7.39
Benin	Boys	1999	14	7.45
Benin	Boys	2000	14	7.53
Benin	Boys	2001	14	7.60
Benin	Boys	2002	14	7.68
Benin	Boys	2003	14	7.76
Benin	Boys	2004	14	7.84
Benin	Boys	2005	14	7.93
Benin	Boys	2006	14	8.03
Benin	Boys	2007	14	8.12
Benin	Boys	2008	14	8.22
Benin	Boys	2009	14	8.33
Benin	Boys	2010	14	8.44
Benin	Boys	2011	14	8.55
Benin	Boys	2012	14	8.67
Benin	Boys	2013	14	8.79
Benin	Boys	2014	14	8.92
Benin	Boys	2015	14	9.05
Benin	Boys	2016	14	9.20
Benin	Boys	1975	15	7.88

Benin	Boys	1976	15	7.92
Benin	Boys	1977	15	7.97
Benin	Boys	1978	15	8.01
Benin	Boys	1979	15	8.05
Benin	Boys	1980	15	8.10
Benin	Boys	1981	15	8.14
Benin	Boys	1982	15	8.19
Benin	Boys	1983	15	8.24
Benin	Boys	1984	15	8.28
Benin	Boys	1985	15	8.33
Benin	Boys	1986	15	8.38
Benin	Boys	1987	15	8.43
Benin	Boys	1988	15	8.49
Benin	Boys	1989	15	8.54
Benin	Boys	1990	15	8.60
Benin	Boys	1991	15	8.66
Benin	Boys	1992	15	8.72
Benin	Boys	1993	15	8.78
Benin	Boys	1994	15	8.84
Benin	Boys	1995	15	8.91
Benin	Boys	1996	15	8.98
Benin	Boys	1997	15	9.05
Benin	Boys	1998	15	9.12
Benin	Boys	1999	15	9.20
Benin	Boys	2000	15	9.28
Benin	Boys	2001	15	9.36
Benin	Boys	2002	15	9.45
Benin	Boys	2003	15	9.54
Benin	Boys	2004	15	9.63
Benin	Boys	2005	15	9.72
Benin	Boys	2006	15	9.83
Benin	Boys	2007	15	9.93
Benin	Boys	2008	15	10.04
Benin	Boys	2009	15	10.15
Benin	Boys	2010	15	10.27
Benin	Boys	2011	15	10.39
Benin	Boys	2012	15	10.52
Benin	Boys	2013	15	10.65
Benin	Boys	2014	15	10.79
Benin	Boys	2015	15	10.94
Benin	Boys	2016	15	11.09
Benin	Boys	1975	16	9.65
Benin	Boys	1976	16	9.69
Benin	Boys	1977	16	9.74
Benin	Boys	1978	16	9.79
Benin	Boys	1979	16	9.85
Benin	Boys	1980	16	9.90
Benin	Boys	1981	16	9.95
Benin	Boys	1982	16	10.01
Benin	Boys	1983	16	10.06
Benin	Boys	1984	16	10.11
Benin	Boys	1985	16	10.17

Benin	Boys	1986	16	10.23
Benin	Boys	1987	16	10.28
Benin	Boys	1988	16	10.34
Benin	Boys	1989	16	10.40
Benin	Boys	1990	16	10.47
Benin	Boys	1991	16	10.53
Benin	Boys	1992	16	10.60
Benin	Boys	1993	16	10.67
Benin	Boys	1994	16	10.74
Benin	Boys	1995	16	10.81
Benin	Boys	1996	16	10.89
Benin	Boys	1997	16	10.97
Benin	Boys	1998	16	11.04
Benin	Boys	1999	16	11.13
Benin	Boys	2000	16	11.21
Benin	Boys	2001	16	11.30
Benin	Boys	2002	16	11.39
Benin	Boys	2003	16	11.49
Benin	Boys	2004	16	11.59
Benin	Boys	2005	16	11.69
Benin	Boys	2006	16	11.80
Benin	Boys	2007	16	11.91
Benin	Boys	2008	16	12.03
Benin	Boys	2009	16	12.15
Benin	Boys	2010	16	12.28
Benin	Boys	2011	16	12.41
Benin	Boys	2012	16	12.54
Benin	Boys	2013	16	12.68
Benin	Boys	2014	16	12.82
Benin	Boys	2015	16	12.98
Benin	Boys	2016	16	13.14
Benin	Boys	1975	17	11.48
Benin	Boys	1976	17	11.53
Benin	Boys	1977	17	11.59
Benin	Boys	1978	17	11.65
Benin	Boys	1979	17	11.71
Benin	Boys	1980	17	11.78
Benin	Boys	1981	17	11.84
Benin	Boys	1982	17	11.90
Benin	Boys	1983	17	11.96
Benin	Boys	1984	17	12.02
Benin	Boys	1985	17	12.08
Benin	Boys	1986	17	12.15
Benin	Boys	1987	17	12.21
Benin	Boys	1988	17	12.28
Benin	Boys	1989	17	12.35
Benin	Boys	1990	17	12.42
Benin	Boys	1991	17	12.49
Benin	Boys	1992	17	12.56
Benin	Boys	1993	17	12.64
Benin	Boys	1994	17	12.72
Benin	Boys	1995	17	12.80

Benin	Boys	1996	17	12.88
Benin	Boys	1997	17	12.96
Benin	Boys	1998	17	13.05
Benin	Boys	1999	17	13.14
Benin	Boys	2000	17	13.23
Benin	Boys	2001	17	13.32
Benin	Boys	2002	17	13.42
Benin	Boys	2003	17	13.53
Benin	Boys	2004	17	13.63
Benin	Boys	2005	17	13.74
Benin	Boys	2006	17	13.86
Benin	Boys	2007	17	13.98
Benin	Boys	2008	17	14.10
Benin	Boys	2009	17	14.23
Benin	Boys	2010	17	14.36
Benin	Boys	2011	17	14.50
Benin	Boys	2012	17	14.64
Benin	Boys	2013	17	14.79
Benin	Boys	2014	17	14.94
Benin	Boys	2015	17	15.10
Benin	Boys	2016	17	15.26
Benin	Boys	1975	18	13.27
Benin	Boys	1976	18	13.34
Benin	Boys	1977	18	13.41
Benin	Boys	1978	18	13.47
Benin	Boys	1979	18	13.54
Benin	Boys	1980	18	13.62
Benin	Boys	1981	18	13.69
Benin	Boys	1982	18	13.76
Benin	Boys	1983	18	13.83
Benin	Boys	1984	18	13.90
Benin	Boys	1985	18	13.97
Benin	Boys	1986	18	14.04
Benin	Boys	1987	18	14.11
Benin	Boys	1988	18	14.19
Benin	Boys	1989	18	14.27
Benin	Boys	1990	18	14.35
Benin	Boys	1991	18	14.43
Benin	Boys	1992	18	14.51
Benin	Boys	1993	18	14.59
Benin	Boys	1994	18	14.68
Benin	Boys	1995	18	14.76
Benin	Boys	1996	18	14.85
Benin	Boys	1997	18	14.95
Benin	Boys	1998	18	15.04
Benin	Boys	1999	18	15.14
Benin	Boys	2000	18	15.24
Benin	Boys	2001	18	15.35
Benin	Boys	2002	18	15.45
Benin	Boys	2003	18	15.57
Benin	Boys	2004	18	15.68
Benin	Boys	2005	18	15.80

Benin	Boys	2006	18	15.93
Benin	Boys	2007	18	16.06
Benin	Boys	2008	18	16.19
Benin	Boys	2009	18	16.33
Benin	Boys	2010	18	16.47
Benin	Boys	2011	18	16.62
Benin	Boys	2012	18	16.77
Benin	Boys	2013	18	16.92
Benin	Boys	2014	18	17.09
Benin	Boys	2015	18	17.25
Benin	Boys	2016	18	17.43
Benin	Boys	1975	Average	8.22
Benin	Boys	1976	Average	8.26
Benin	Boys	1977	Average	8.31
Benin	Boys	1978	Average	8.35
Benin	Boys	1979	Average	8.40
Benin	Boys	1980	Average	8.44
Benin	Boys	1981	Average	8.49
Benin	Boys	1982	Average	8.54
Benin	Boys	1983	Average	8.58
Benin	Boys	1984	Average	8.63
Benin	Boys	1985	Average	8.68
Benin	Boys	1986	Average	8.73
Benin	Boys	1987	Average	8.78
Benin	Boys	1988	Average	8.84
Benin	Boys	1989	Average	8.89
Benin	Boys	1990	Average	8.95
Benin	Boys	1991	Average	9.01
Benin	Boys	1992	Average	9.07
Benin	Boys	1993	Average	9.13
Benin	Boys	1994	Average	9.19
Benin	Boys	1995	Average	9.26
Benin	Boys	1996	Average	9.33
Benin	Boys	1997	Average	9.40
Benin	Boys	1998	Average	9.47
Benin	Boys	1999	Average	9.55
Benin	Boys	2000	Average	9.62
Benin	Boys	2001	Average	9.71
Benin	Boys	2002	Average	9.79
Benin	Boys	2003	Average	9.88
Benin	Boys	2004	Average	9.97
Benin	Boys	2005	Average	10.06
Benin	Boys	2006	Average	10.16
Benin	Boys	2007	Average	10.27
Benin	Boys	2008	Average	10.37
Benin	Boys	2009	Average	10.48
Benin	Boys	2010	Average	10.60
Benin	Boys	2011	Average	10.72
Benin	Boys	2012	Average	10.84
Benin	Boys	2013	Average	10.97
Benin	Boys	2014	Average	11.11
Benin	Boys	2015	Average	11.25

Benin	Boys	2016	Average	11.39
Benin	Girls	1975	12	4.79
Benin	Girls	1976	12	4.83
Benin	Girls	1977	12	4.87
Benin	Girls	1978	12	4.91
Benin	Girls	1979	12	4.96
Benin	Girls	1980	12	5.01
Benin	Girls	1981	12	5.06
Benin	Girls	1982	12	5.11
Benin	Girls	1983	12	5.16
Benin	Girls	1984	12	5.21
Benin	Girls	1985	12	5.27
Benin	Girls	1986	12	5.33
Benin	Girls	1987	12	5.38
Benin	Girls	1988	12	5.44
Benin	Girls	1989	12	5.51
Benin	Girls	1990	12	5.57
Benin	Girls	1991	12	5.64
Benin	Girls	1992	12	5.71
Benin	Girls	1993	12	5.78
Benin	Girls	1994	12	5.85
Benin	Girls	1995	12	5.92
Benin	Girls	1996	12	6.00
Benin	Girls	1997	12	6.08
Benin	Girls	1998	12	6.15
Benin	Girls	1999	12	6.23
Benin	Girls	2000	12	6.32
Benin	Girls	2001	12	6.40
Benin	Girls	2002	12	6.49
Benin	Girls	2003	12	6.58
Benin	Girls	2004	12	6.67
Benin	Girls	2005	12	6.76
Benin	Girls	2006	12	6.86
Benin	Girls	2007	12	6.96
Benin	Girls	2008	12	7.06
Benin	Girls	2009	12	7.16
Benin	Girls	2010	12	7.26
Benin	Girls	2011	12	7.37
Benin	Girls	2012	12	7.48
Benin	Girls	2013	12	7.59
Benin	Girls	2014	12	7.70
Benin	Girls	2015	12	7.82
Benin	Girls	2016	12	7.94
Benin	Girls	1975	13	6.03
Benin	Girls	1976	13	6.08
Benin	Girls	1977	13	6.13
Benin	Girls	1978	13	6.18
Benin	Girls	1979	13	6.24
Benin	Girls	1980	13	6.30
Benin	Girls	1981	13	6.36
Benin	Girls	1982	13	6.42
Benin	Girls	1983	13	6.48

Benin	Girls	1984	13	6.54
Benin	Girls	1985	13	6.61
Benin	Girls	1986	13	6.67
Benin	Girls	1987	13	6.74
Benin	Girls	1988	13	6.81
Benin	Girls	1989	13	6.88
Benin	Girls	1990	13	6.96
Benin	Girls	1991	13	7.04
Benin	Girls	1992	13	7.12
Benin	Girls	1993	13	7.20
Benin	Girls	1994	13	7.28
Benin	Girls	1995	13	7.37
Benin	Girls	1996	13	7.45
Benin	Girls	1997	13	7.54
Benin	Girls	1998	13	7.63
Benin	Girls	1999	13	7.73
Benin	Girls	2000	13	7.82
Benin	Girls	2001	13	7.92
Benin	Girls	2002	13	8.02
Benin	Girls	2003	13	8.12
Benin	Girls	2004	13	8.22
Benin	Girls	2005	13	8.33
Benin	Girls	2006	13	8.44
Benin	Girls	2007	13	8.55
Benin	Girls	2008	13	8.67
Benin	Girls	2009	13	8.78
Benin	Girls	2010	13	8.90
Benin	Girls	2011	13	9.02
Benin	Girls	2012	13	9.15
Benin	Girls	2013	13	9.28
Benin	Girls	2014	13	9.41
Benin	Girls	2015	13	9.54
Benin	Girls	2016	13	9.68
Benin	Girls	1975	14	7.42
Benin	Girls	1976	14	7.47
Benin	Girls	1977	14	7.53
Benin	Girls	1978	14	7.59
Benin	Girls	1979	14	7.66
Benin	Girls	1980	14	7.72
Benin	Girls	1981	14	7.79
Benin	Girls	1982	14	7.86
Benin	Girls	1983	14	7.93
Benin	Girls	1984	14	8.00
Benin	Girls	1985	14	8.07
Benin	Girls	1986	14	8.15
Benin	Girls	1987	14	8.22
Benin	Girls	1988	14	8.30
Benin	Girls	1989	14	8.38
Benin	Girls	1990	14	8.47
Benin	Girls	1991	14	8.55
Benin	Girls	1992	14	8.64
Benin	Girls	1993	14	8.73

Benin	Girls	1994	14	8.82
Benin	Girls	1995	14	8.92
Benin	Girls	1996	14	9.01
Benin	Girls	1997	14	9.11
Benin	Girls	1998	14	9.21
Benin	Girls	1999	14	9.31
Benin	Girls	2000	14	9.41
Benin	Girls	2001	14	9.52
Benin	Girls	2002	14	9.63
Benin	Girls	2003	14	9.74
Benin	Girls	2004	14	9.85
Benin	Girls	2005	14	9.97
Benin	Girls	2006	14	10.09
Benin	Girls	2007	14	10.21
Benin	Girls	2008	14	10.33
Benin	Girls	2009	14	10.46
Benin	Girls	2010	14	10.59
Benin	Girls	2011	14	10.72
Benin	Girls	2012	14	10.85
Benin	Girls	2013	14	10.99
Benin	Girls	2014	14	11.14
Benin	Girls	2015	14	11.28
Benin	Girls	2016	14	11.43
Benin	Girls	1975	15	8.75
Benin	Girls	1976	15	8.81
Benin	Girls	1977	15	8.88
Benin	Girls	1978	15	8.94
Benin	Girls	1979	15	9.01
Benin	Girls	1980	15	9.09
Benin	Girls	1981	15	9.16
Benin	Girls	1982	15	9.23
Benin	Girls	1983	15	9.31
Benin	Girls	1984	15	9.38
Benin	Girls	1985	15	9.46
Benin	Girls	1986	15	9.54
Benin	Girls	1987	15	9.62
Benin	Girls	1988	15	9.71
Benin	Girls	1989	15	9.79
Benin	Girls	1990	15	9.88
Benin	Girls	1991	15	9.97
Benin	Girls	1992	15	10.07
Benin	Girls	1993	15	10.16
Benin	Girls	1994	15	10.26
Benin	Girls	1995	15	10.36
Benin	Girls	1996	15	10.46
Benin	Girls	1997	15	10.56
Benin	Girls	1998	15	10.66
Benin	Girls	1999	15	10.77
Benin	Girls	2000	15	10.88
Benin	Girls	2001	15	10.99
Benin	Girls	2002	15	11.10
Benin	Girls	2003	15	11.22

Benin	Girls	2004	15	11.34
Benin	Girls	2005	15	11.46
Benin	Girls	2006	15	11.58
Benin	Girls	2007	15	11.71
Benin	Girls	2008	15	11.84
Benin	Girls	2009	15	11.97
Benin	Girls	2010	15	12.11
Benin	Girls	2011	15	12.24
Benin	Girls	2012	15	12.39
Benin	Girls	2013	15	12.53
Benin	Girls	2014	15	12.68
Benin	Girls	2015	15	12.83
Benin	Girls	2016	15	12.99
Benin	Girls	1975	16	9.85
Benin	Girls	1976	16	9.91
Benin	Girls	1977	16	9.98
Benin	Girls	1978	16	10.05
Benin	Girls	1979	16	10.13
Benin	Girls	1980	16	10.20
Benin	Girls	1981	16	10.28
Benin	Girls	1982	16	10.35
Benin	Girls	1983	16	10.43
Benin	Girls	1984	16	10.51
Benin	Girls	1985	16	10.59
Benin	Girls	1986	16	10.68
Benin	Girls	1987	16	10.76
Benin	Girls	1988	16	10.85
Benin	Girls	1989	16	10.94
Benin	Girls	1990	16	11.03
Benin	Girls	1991	16	11.13
Benin	Girls	1992	16	11.22
Benin	Girls	1993	16	11.32
Benin	Girls	1994	16	11.42
Benin	Girls	1995	16	11.52
Benin	Girls	1996	16	11.63
Benin	Girls	1997	16	11.73
Benin	Girls	1998	16	11.84
Benin	Girls	1999	16	11.95
Benin	Girls	2000	16	12.06
Benin	Girls	2001	16	12.17
Benin	Girls	2002	16	12.29
Benin	Girls	2003	16	12.41
Benin	Girls	2004	16	12.53
Benin	Girls	2005	16	12.66
Benin	Girls	2006	16	12.79
Benin	Girls	2007	16	12.92
Benin	Girls	2008	16	13.05
Benin	Girls	2009	16	13.19
Benin	Girls	2010	16	13.33
Benin	Girls	2011	16	13.47
Benin	Girls	2012	16	13.61
Benin	Girls	2013	16	13.76

Benin	Girls	2014	16	13.92
Benin	Girls	2015	16	14.08
Benin	Girls	2016	16	14.24
Benin	Girls	1975	17	10.63
Benin	Girls	1976	17	10.70
Benin	Girls	1977	17	10.77
Benin	Girls	1978	17	10.84
Benin	Girls	1979	17	10.92
Benin	Girls	1980	17	11.00
Benin	Girls	1981	17	11.08
Benin	Girls	1982	17	11.16
Benin	Girls	1983	17	11.24
Benin	Girls	1984	17	11.32
Benin	Girls	1985	17	11.41
Benin	Girls	1986	17	11.49
Benin	Girls	1987	17	11.58
Benin	Girls	1988	17	11.67
Benin	Girls	1989	17	11.77
Benin	Girls	1990	17	11.86
Benin	Girls	1991	17	11.96
Benin	Girls	1992	17	12.06
Benin	Girls	1993	17	12.16
Benin	Girls	1994	17	12.27
Benin	Girls	1995	17	12.38
Benin	Girls	1996	17	12.48
Benin	Girls	1997	17	12.59
Benin	Girls	1998	17	12.70
Benin	Girls	1999	17	12.82
Benin	Girls	2000	17	12.94
Benin	Girls	2001	17	13.05
Benin	Girls	2002	17	13.18
Benin	Girls	2003	17	13.30
Benin	Girls	2004	17	13.43
Benin	Girls	2005	17	13.56
Benin	Girls	2006	17	13.70
Benin	Girls	2007	17	13.83
Benin	Girls	2008	17	13.97
Benin	Girls	2009	17	14.12
Benin	Girls	2010	17	14.26
Benin	Girls	2011	17	14.41
Benin	Girls	2012	17	14.56
Benin	Girls	2013	17	14.72
Benin	Girls	2014	17	14.88
Benin	Girls	2015	17	15.05
Benin	Girls	2016	17	15.22
Benin	Girls	1975	18	11.14
Benin	Girls	1976	18	11.21
Benin	Girls	1977	18	11.29
Benin	Girls	1978	18	11.36
Benin	Girls	1979	18	11.45
Benin	Girls	1980	18	11.53
Benin	Girls	1981	18	11.62

Benin	Girls	1982	18	11.70
Benin	Girls	1983	18	11.79
Benin	Girls	1984	18	11.88
Benin	Girls	1985	18	11.97
Benin	Girls	1986	18	12.06
Benin	Girls	1987	18	12.16
Benin	Girls	1988	18	12.26
Benin	Girls	1989	18	12.36
Benin	Girls	1990	18	12.46
Benin	Girls	1991	18	12.57
Benin	Girls	1992	18	12.68
Benin	Girls	1993	18	12.79
Benin	Girls	1994	18	12.90
Benin	Girls	1995	18	13.02
Benin	Girls	1996	18	13.14
Benin	Girls	1997	18	13.26
Benin	Girls	1998	18	13.38
Benin	Girls	1999	18	13.50
Benin	Girls	2000	18	13.63
Benin	Girls	2001	18	13.76
Benin	Girls	2002	18	13.89
Benin	Girls	2003	18	14.03
Benin	Girls	2004	18	14.17
Benin	Girls	2005	18	14.31
Benin	Girls	2006	18	14.46
Benin	Girls	2007	18	14.61
Benin	Girls	2008	18	14.76
Benin	Girls	2009	18	14.92
Benin	Girls	2010	18	15.07
Benin	Girls	2011	18	15.24
Benin	Girls	2012	18	15.40
Benin	Girls	2013	18	15.57
Benin	Girls	2014	18	15.75
Benin	Girls	2015	18	15.93
Benin	Girls	2016	18	16.11
Benin	Girls	1975	Average	8.37
Benin	Girls	1976	Average	8.43
Benin	Girls	1977	Average	8.49
Benin	Girls	1978	Average	8.56
Benin	Girls	1979	Average	8.62
Benin	Girls	1980	Average	8.69
Benin	Girls	1981	Average	8.76
Benin	Girls	1982	Average	8.83
Benin	Girls	1983	Average	8.91
Benin	Girls	1984	Average	8.98
Benin	Girls	1985	Average	9.05
Benin	Girls	1986	Average	9.13
Benin	Girls	1987	Average	9.21
Benin	Girls	1988	Average	9.29
Benin	Girls	1989	Average	9.38
Benin	Girls	1990	Average	9.46
Benin	Girls	1991	Average	9.55

Benin	Girls	1992	Average	9.64
Benin	Girls	1993	Average	9.73
Benin	Girls	1994	Average	9.83
Benin	Girls	1995	Average	9.93
Benin	Girls	1996	Average	10.02
Benin	Girls	1997	Average	10.12
Benin	Girls	1998	Average	10.23
Benin	Girls	1999	Average	10.33
Benin	Girls	2000	Average	10.44
Benin	Girls	2001	Average	10.54
Benin	Girls	2002	Average	10.66
Benin	Girls	2003	Average	10.77
Benin	Girls	2004	Average	10.89
Benin	Girls	2005	Average	11.01
Benin	Girls	2006	Average	11.13
Benin	Girls	2007	Average	11.26
Benin	Girls	2008	Average	11.38
Benin	Girls	2009	Average	11.51
Benin	Girls	2010	Average	11.65
Benin	Girls	2011	Average	11.78
Benin	Girls	2012	Average	11.92
Benin	Girls	2013	Average	12.06
Benin	Girls	2014	Average	12.21
Benin	Girls	2015	Average	12.36
Benin	Girls	2016	Average	12.52
Bermuda	Boys	1975	12	11.08
Bermuda	Boys	1976	12	11.14
Bermuda	Boys	1977	12	11.20
Bermuda	Boys	1978	12	11.27
Bermuda	Boys	1979	12	11.34
Bermuda	Boys	1980	12	11.42
Bermuda	Boys	1981	12	11.49
Bermuda	Boys	1982	12	11.58
Bermuda	Boys	1983	12	11.66
Bermuda	Boys	1984	12	11.75
Bermuda	Boys	1985	12	11.84
Bermuda	Boys	1986	12	11.93
Bermuda	Boys	1987	12	12.02
Bermuda	Boys	1988	12	12.12
Bermuda	Boys	1989	12	12.22
Bermuda	Boys	1990	12	12.33
Bermuda	Boys	1991	12	12.43
Bermuda	Boys	1992	12	12.54
Bermuda	Boys	1993	12	12.65
Bermuda	Boys	1994	12	12.76
Bermuda	Boys	1995	12	12.87
Bermuda	Boys	1996	12	12.99
Bermuda	Boys	1997	12	13.11
Bermuda	Boys	1998	12	13.23
Bermuda	Boys	1999	12	13.35
Bermuda	Boys	2000	12	13.47
Bermuda	Boys	2001	12	13.60

Bermuda	Boys	2002	12	13.73
Bermuda	Boys	2003	12	13.86
Bermuda	Boys	2004	12	13.99
Bermuda	Boys	2005	12	14.12
Bermuda	Boys	2006	12	14.25
Bermuda	Boys	2007	12	14.38
Bermuda	Boys	2008	12	14.52
Bermuda	Boys	2009	12	14.65
Bermuda	Boys	2010	12	14.78
Bermuda	Boys	2011	12	14.91
Bermuda	Boys	2012	12	15.05
Bermuda	Boys	2013	12	15.18
Bermuda	Boys	2014	12	15.31
Bermuda	Boys	2015	12	15.44
Bermuda	Boys	2016	12	15.56
Bermuda	Boys	1975	13	13.51
Bermuda	Boys	1976	13	13.58
Bermuda	Boys	1977	13	13.65
Bermuda	Boys	1978	13	13.73
Bermuda	Boys	1979	13	13.81
Bermuda	Boys	1980	13	13.89
Bermuda	Boys	1981	13	13.99
Bermuda	Boys	1982	13	14.08
Bermuda	Boys	1983	13	14.18
Bermuda	Boys	1984	13	14.28
Bermuda	Boys	1985	13	14.39
Bermuda	Boys	1986	13	14.50
Bermuda	Boys	1987	13	14.61
Bermuda	Boys	1988	13	14.73
Bermuda	Boys	1989	13	14.85
Bermuda	Boys	1990	13	14.97
Bermuda	Boys	1991	13	15.10
Bermuda	Boys	1992	13	15.22
Bermuda	Boys	1993	13	15.36
Bermuda	Boys	1994	13	15.49
Bermuda	Boys	1995	13	15.63
Bermuda	Boys	1996	13	15.77
Bermuda	Boys	1997	13	15.91
Bermuda	Boys	1998	13	16.06
Bermuda	Boys	1999	13	16.21
Bermuda	Boys	2000	13	16.36
Bermuda	Boys	2001	13	16.51
Bermuda	Boys	2002	13	16.67
Bermuda	Boys	2003	13	16.83
Bermuda	Boys	2004	13	16.99
Bermuda	Boys	2005	13	17.15
Bermuda	Boys	2006	13	17.32
Bermuda	Boys	2007	13	17.48
Bermuda	Boys	2008	13	17.65
Bermuda	Boys	2009	13	17.81
Bermuda	Boys	2010	13	17.98
Bermuda	Boys	2011	13	18.14

Bermuda	Boys	2012	13	18.31
Bermuda	Boys	2013	13	18.48
Bermuda	Boys	2014	13	18.64
Bermuda	Boys	2015	13	18.81
Bermuda	Boys	2016	13	18.98
Bermuda	Boys	1975	14	16.06
Bermuda	Boys	1976	14	16.13
Bermuda	Boys	1977	14	16.20
Bermuda	Boys	1978	14	16.29
Bermuda	Boys	1979	14	16.37
Bermuda	Boys	1980	14	16.47
Bermuda	Boys	1981	14	16.57
Bermuda	Boys	1982	14	16.67
Bermuda	Boys	1983	14	16.78
Bermuda	Boys	1984	14	16.89
Bermuda	Boys	1985	14	17.00
Bermuda	Boys	1986	14	17.13
Bermuda	Boys	1987	14	17.25
Bermuda	Boys	1988	14	17.38
Bermuda	Boys	1989	14	17.52
Bermuda	Boys	1990	14	17.65
Bermuda	Boys	1991	14	17.80
Bermuda	Boys	1992	14	17.94
Bermuda	Boys	1993	14	18.09
Bermuda	Boys	1994	14	18.24
Bermuda	Boys	1995	14	18.40
Bermuda	Boys	1996	14	18.55
Bermuda	Boys	1997	14	18.72
Bermuda	Boys	1998	14	18.88
Bermuda	Boys	1999	14	19.05
Bermuda	Boys	2000	14	19.23
Bermuda	Boys	2001	14	19.40
Bermuda	Boys	2002	14	19.58
Bermuda	Boys	2003	14	19.77
Bermuda	Boys	2004	14	19.95
Bermuda	Boys	2005	14	20.14
Bermuda	Boys	2006	14	20.33
Bermuda	Boys	2007	14	20.52
Bermuda	Boys	2008	14	20.71
Bermuda	Boys	2009	14	20.91
Bermuda	Boys	2010	14	21.10
Bermuda	Boys	2011	14	21.30
Bermuda	Boys	2012	14	21.50
Bermuda	Boys	2013	14	21.69
Bermuda	Boys	2014	14	21.89
Bermuda	Boys	2015	14	22.09
Bermuda	Boys	2016	14	22.29
Bermuda	Boys	1975	15	18.55
Bermuda	Boys	1976	15	18.62
Bermuda	Boys	1977	15	18.69
Bermuda	Boys	1978	15	18.78
Bermuda	Boys	1979	15	18.86

Bermuda	Boys	1980	15	18.96
Bermuda	Boys	1981	15	19.06
Bermuda	Boys	1982	15	19.17
Bermuda	Boys	1983	15	19.28
Bermuda	Boys	1984	15	19.40
Bermuda	Boys	1985	15	19.52
Bermuda	Boys	1986	15	19.64
Bermuda	Boys	1987	15	19.78
Bermuda	Boys	1988	15	19.91
Bermuda	Boys	1989	15	20.06
Bermuda	Boys	1990	15	20.20
Bermuda	Boys	1991	15	20.35
Bermuda	Boys	1992	15	20.50
Bermuda	Boys	1993	15	20.66
Bermuda	Boys	1994	15	20.82
Bermuda	Boys	1995	15	20.98
Bermuda	Boys	1996	15	21.15
Bermuda	Boys	1997	15	21.33
Bermuda	Boys	1998	15	21.51
Bermuda	Boys	1999	15	21.69
Bermuda	Boys	2000	15	21.87
Bermuda	Boys	2001	15	22.07
Bermuda	Boys	2002	15	22.26
Bermuda	Boys	2003	15	22.46
Bermuda	Boys	2004	15	22.66
Bermuda	Boys	2005	15	22.86
Bermuda	Boys	2006	15	23.07
Bermuda	Boys	2007	15	23.27
Bermuda	Boys	2008	15	23.49
Bermuda	Boys	2009	15	23.70
Bermuda	Boys	2010	15	23.91
Bermuda	Boys	2011	15	24.13
Bermuda	Boys	2012	15	24.35
Bermuda	Boys	2013	15	24.57
Bermuda	Boys	2014	15	24.79
Bermuda	Boys	2015	15	25.02
Bermuda	Boys	2016	15	25.24
Bermuda	Boys	1975	16	20.97
Bermuda	Boys	1976	16	21.04
Bermuda	Boys	1977	16	21.11
Bermuda	Boys	1978	16	21.19
Bermuda	Boys	1979	16	21.28
Bermuda	Boys	1980	16	21.38
Bermuda	Boys	1981	16	21.48
Bermuda	Boys	1982	16	21.58
Bermuda	Boys	1983	16	21.69
Bermuda	Boys	1984	16	21.81
Bermuda	Boys	1985	16	21.93
Bermuda	Boys	1986	16	22.06
Bermuda	Boys	1987	16	22.20
Bermuda	Boys	1988	16	22.34
Bermuda	Boys	1989	16	22.48

Bermuda	Boys	1990	16	22.63
Bermuda	Boys	1991	16	22.78
Bermuda	Boys	1992	16	22.94
Bermuda	Boys	1993	16	23.10
Bermuda	Boys	1994	16	23.26
Bermuda	Boys	1995	16	23.43
Bermuda	Boys	1996	16	23.60
Bermuda	Boys	1997	16	23.78
Bermuda	Boys	1998	16	23.96
Bermuda	Boys	1999	16	24.15
Bermuda	Boys	2000	16	24.34
Bermuda	Boys	2001	16	24.54
Bermuda	Boys	2002	16	24.74
Bermuda	Boys	2003	16	24.95
Bermuda	Boys	2004	16	25.15
Bermuda	Boys	2005	16	25.37
Bermuda	Boys	2006	16	25.58
Bermuda	Boys	2007	16	25.80
Bermuda	Boys	2008	16	26.02
Bermuda	Boys	2009	16	26.24
Bermuda	Boys	2010	16	26.47
Bermuda	Boys	2011	16	26.70
Bermuda	Boys	2012	16	26.93
Bermuda	Boys	2013	16	27.16
Bermuda	Boys	2014	16	27.40
Bermuda	Boys	2015	16	27.64
Bermuda	Boys	2016	16	27.89
Bermuda	Boys	1975	17	23.38
Bermuda	Boys	1976	17	23.45
Bermuda	Boys	1977	17	23.52
Bermuda	Boys	1978	17	23.60
Bermuda	Boys	1979	17	23.69
Bermuda	Boys	1980	17	23.78
Bermuda	Boys	1981	17	23.88
Bermuda	Boys	1982	17	23.99
Bermuda	Boys	1983	17	24.10
Bermuda	Boys	1984	17	24.22
Bermuda	Boys	1985	17	24.34
Bermuda	Boys	1986	17	24.47
Bermuda	Boys	1987	17	24.61
Bermuda	Boys	1988	17	24.75
Bermuda	Boys	1989	17	24.89
Bermuda	Boys	1990	17	25.04
Bermuda	Boys	1991	17	25.20
Bermuda	Boys	1992	17	25.36
Bermuda	Boys	1993	17	25.52
Bermuda	Boys	1994	17	25.68
Bermuda	Boys	1995	17	25.85
Bermuda	Boys	1996	17	26.03
Bermuda	Boys	1997	17	26.21
Bermuda	Boys	1998	17	26.40
Bermuda	Boys	1999	17	26.59

Bermuda	Boys	2000	17	26.78
Bermuda	Boys	2001	17	26.98
Bermuda	Boys	2002	17	27.19
Bermuda	Boys	2003	17	27.40
Bermuda	Boys	2004	17	27.61
Bermuda	Boys	2005	17	27.82
Bermuda	Boys	2006	17	28.04
Bermuda	Boys	2007	17	28.27
Bermuda	Boys	2008	17	28.49
Bermuda	Boys	2009	17	28.72
Bermuda	Boys	2010	17	28.95
Bermuda	Boys	2011	17	29.19
Bermuda	Boys	2012	17	29.43
Bermuda	Boys	2013	17	29.67
Bermuda	Boys	2014	17	29.92
Bermuda	Boys	2015	17	30.17
Bermuda	Boys	2016	17	30.43
Bermuda	Boys	1975	18	25.90
Bermuda	Boys	1976	18	25.97
Bermuda	Boys	1977	18	26.05
Bermuda	Boys	1978	18	26.13
Bermuda	Boys	1979	18	26.22
Bermuda	Boys	1980	18	26.31
Bermuda	Boys	1981	18	26.42
Bermuda	Boys	1982	18	26.53
Bermuda	Boys	1983	18	26.64
Bermuda	Boys	1984	18	26.76
Bermuda	Boys	1985	18	26.89
Bermuda	Boys	1986	18	27.02
Bermuda	Boys	1987	18	27.16
Bermuda	Boys	1988	18	27.30
Bermuda	Boys	1989	18	27.45
Bermuda	Boys	1990	18	27.60
Bermuda	Boys	1991	18	27.76
Bermuda	Boys	1992	18	27.92
Bermuda	Boys	1993	18	28.09
Bermuda	Boys	1994	18	28.26
Bermuda	Boys	1995	18	28.43
Bermuda	Boys	1996	18	28.61
Bermuda	Boys	1997	18	28.80
Bermuda	Boys	1998	18	28.99
Bermuda	Boys	1999	18	29.18
Bermuda	Boys	2000	18	29.38
Bermuda	Boys	2001	18	29.59
Bermuda	Boys	2002	18	29.80
Bermuda	Boys	2003	18	30.01
Bermuda	Boys	2004	18	30.23
Bermuda	Boys	2005	18	30.45
Bermuda	Boys	2006	18	30.67
Bermuda	Boys	2007	18	30.90
Bermuda	Boys	2008	18	31.13
Bermuda	Boys	2009	18	31.37

Bermuda	Boys	2010	18	31.61
Bermuda	Boys	2011	18	31.85
Bermuda	Boys	2012	18	32.09
Bermuda	Boys	2013	18	32.34
Bermuda	Boys	2014	18	32.60
Bermuda	Boys	2015	18	32.85
Bermuda	Boys	2016	18	33.11
Bermuda	Boys	1975	Average	18.50
Bermuda	Boys	1976	Average	18.56
Bermuda	Boys	1977	Average	18.63
Bermuda	Boys	1978	Average	18.71
Bermuda	Boys	1979	Average	18.80
Bermuda	Boys	1980	Average	18.89
Bermuda	Boys	1981	Average	18.98
Bermuda	Boys	1982	Average	19.08
Bermuda	Boys	1983	Average	19.19
Bermuda	Boys	1984	Average	19.30
Bermuda	Boys	1985	Average	19.42
Bermuda	Boys	1986	Average	19.54
Bermuda	Boys	1987	Average	19.66
Bermuda	Boys	1988	Average	19.79
Bermuda	Boys	1989	Average	19.92
Bermuda	Boys	1990	Average	20.06
Bermuda	Boys	1991	Average	20.20
Bermuda	Boys	1992	Average	20.35
Bermuda	Boys	1993	Average	20.49
Bermuda	Boys	1994	Average	20.65
Bermuda	Boys	1995	Average	20.80
Bermuda	Boys	1996	Average	20.96
Bermuda	Boys	1997	Average	21.12
Bermuda	Boys	1998	Average	21.29
Bermuda	Boys	1999	Average	21.46
Bermuda	Boys	2000	Average	21.64
Bermuda	Boys	2001	Average	21.81
Bermuda	Boys	2002	Average	22.00
Bermuda	Boys	2003	Average	22.18
Bermuda	Boys	2004	Average	22.37
Bermuda	Boys	2005	Average	22.56
Bermuda	Boys	2006	Average	22.75
Bermuda	Boys	2007	Average	22.95
Bermuda	Boys	2008	Average	23.14
Bermuda	Boys	2009	Average	23.34
Bermuda	Boys	2010	Average	23.54
Bermuda	Boys	2011	Average	23.75
Bermuda	Boys	2012	Average	23.95
Bermuda	Boys	2013	Average	24.16
Bermuda	Boys	2014	Average	24.37
Bermuda	Boys	2015	Average	24.58
Bermuda	Boys	2016	Average	24.79
Bermuda	Girls	1975	12	11.11
Bermuda	Girls	1976	12	11.14
Bermuda	Girls	1977	12	11.17

Bermuda	Girls	1978	12	11.20
Bermuda	Girls	1979	12	11.24
Bermuda	Girls	1980	12	11.28
Bermuda	Girls	1981	12	11.31
Bermuda	Girls	1982	12	11.35
Bermuda	Girls	1983	12	11.39
Bermuda	Girls	1984	12	11.44
Bermuda	Girls	1985	12	11.48
Bermuda	Girls	1986	12	11.53
Bermuda	Girls	1987	12	11.58
Bermuda	Girls	1988	12	11.62
Bermuda	Girls	1989	12	11.67
Bermuda	Girls	1990	12	11.72
Bermuda	Girls	1991	12	11.77
Bermuda	Girls	1992	12	11.83
Bermuda	Girls	1993	12	11.88
Bermuda	Girls	1994	12	11.93
Bermuda	Girls	1995	12	11.98
Bermuda	Girls	1996	12	12.03
Bermuda	Girls	1997	12	12.09
Bermuda	Girls	1998	12	12.14
Bermuda	Girls	1999	12	12.19
Bermuda	Girls	2000	12	12.24
Bermuda	Girls	2001	12	12.29
Bermuda	Girls	2002	12	12.34
Bermuda	Girls	2003	12	12.39
Bermuda	Girls	2004	12	12.44
Bermuda	Girls	2005	12	12.49
Bermuda	Girls	2006	12	12.54
Bermuda	Girls	2007	12	12.59
Bermuda	Girls	2008	12	12.64
Bermuda	Girls	2009	12	12.69
Bermuda	Girls	2010	12	12.74
Bermuda	Girls	2011	12	12.78
Bermuda	Girls	2012	12	12.83
Bermuda	Girls	2013	12	12.88
Bermuda	Girls	2014	12	12.94
Bermuda	Girls	2015	12	12.99
Bermuda	Girls	2016	12	13.04
Bermuda	Girls	1975	13	13.36
Bermuda	Girls	1976	13	13.39
Bermuda	Girls	1977	13	13.42
Bermuda	Girls	1978	13	13.46
Bermuda	Girls	1979	13	13.50
Bermuda	Girls	1980	13	13.54
Bermuda	Girls	1981	13	13.58
Bermuda	Girls	1982	13	13.62
Bermuda	Girls	1983	13	13.67
Bermuda	Girls	1984	13	13.72
Bermuda	Girls	1985	13	13.77
Bermuda	Girls	1986	13	13.82
Bermuda	Girls	1987	13	13.87

Bermuda	Girls	1988	13	13.92
Bermuda	Girls	1989	13	13.98
Bermuda	Girls	1990	13	14.04
Bermuda	Girls	1991	13	14.09
Bermuda	Girls	1992	13	14.15
Bermuda	Girls	1993	13	14.21
Bermuda	Girls	1994	13	14.27
Bermuda	Girls	1995	13	14.33
Bermuda	Girls	1996	13	14.38
Bermuda	Girls	1997	13	14.44
Bermuda	Girls	1998	13	14.50
Bermuda	Girls	1999	13	14.56
Bermuda	Girls	2000	13	14.62
Bermuda	Girls	2001	13	14.68
Bermuda	Girls	2002	13	14.73
Bermuda	Girls	2003	13	14.79
Bermuda	Girls	2004	13	14.85
Bermuda	Girls	2005	13	14.91
Bermuda	Girls	2006	13	14.96
Bermuda	Girls	2007	13	15.02
Bermuda	Girls	2008	13	15.07
Bermuda	Girls	2009	13	15.13
Bermuda	Girls	2010	13	15.18
Bermuda	Girls	2011	13	15.24
Bermuda	Girls	2012	13	15.30
Bermuda	Girls	2013	13	15.36
Bermuda	Girls	2014	13	15.42
Bermuda	Girls	2015	13	15.48
Bermuda	Girls	2016	13	15.55
Bermuda	Girls	1975	14	15.48
Bermuda	Girls	1976	14	15.51
Bermuda	Girls	1977	14	15.54
Bermuda	Girls	1978	14	15.58
Bermuda	Girls	1979	14	15.62
Bermuda	Girls	1980	14	15.66
Bermuda	Girls	1981	14	15.70
Bermuda	Girls	1982	14	15.74
Bermuda	Girls	1983	14	15.79
Bermuda	Girls	1984	14	15.84
Bermuda	Girls	1985	14	15.89
Bermuda	Girls	1986	14	15.95
Bermuda	Girls	1987	14	16.00
Bermuda	Girls	1988	14	16.06
Bermuda	Girls	1989	14	16.12
Bermuda	Girls	1990	14	16.18
Bermuda	Girls	1991	14	16.24
Bermuda	Girls	1992	14	16.30
Bermuda	Girls	1993	14	16.36
Bermuda	Girls	1994	14	16.42
Bermuda	Girls	1995	14	16.49
Bermuda	Girls	1996	14	16.55
Bermuda	Girls	1997	14	16.61

Bermuda	Girls	1998	14	16.67
Bermuda	Girls	1999	14	16.73
Bermuda	Girls	2000	14	16.80
Bermuda	Girls	2001	14	16.86
Bermuda	Girls	2002	14	16.92
Bermuda	Girls	2003	14	16.98
Bermuda	Girls	2004	14	17.04
Bermuda	Girls	2005	14	17.10
Bermuda	Girls	2006	14	17.16
Bermuda	Girls	2007	14	17.22
Bermuda	Girls	2008	14	17.28
Bermuda	Girls	2009	14	17.34
Bermuda	Girls	2010	14	17.40
Bermuda	Girls	2011	14	17.47
Bermuda	Girls	2012	14	17.53
Bermuda	Girls	2013	14	17.60
Bermuda	Girls	2014	14	17.67
Bermuda	Girls	2015	14	17.74
Bermuda	Girls	2016	14	17.81
Bermuda	Girls	1975	15	17.25
Bermuda	Girls	1976	15	17.28
Bermuda	Girls	1977	15	17.32
Bermuda	Girls	1978	15	17.35
Bermuda	Girls	1979	15	17.39
Bermuda	Girls	1980	15	17.43
Bermuda	Girls	1981	15	17.47
Bermuda	Girls	1982	15	17.52
Bermuda	Girls	1983	15	17.57
Bermuda	Girls	1984	15	17.62
Bermuda	Girls	1985	15	17.67
Bermuda	Girls	1986	15	17.72
Bermuda	Girls	1987	15	17.78
Bermuda	Girls	1988	15	17.84
Bermuda	Girls	1989	15	17.90
Bermuda	Girls	1990	15	17.96
Bermuda	Girls	1991	15	18.02
Bermuda	Girls	1992	15	18.08
Bermuda	Girls	1993	15	18.15
Bermuda	Girls	1994	15	18.21
Bermuda	Girls	1995	15	18.27
Bermuda	Girls	1996	15	18.34
Bermuda	Girls	1997	15	18.40
Bermuda	Girls	1998	15	18.46
Bermuda	Girls	1999	15	18.53
Bermuda	Girls	2000	15	18.59
Bermuda	Girls	2001	15	18.65
Bermuda	Girls	2002	15	18.72
Bermuda	Girls	2003	15	18.78
Bermuda	Girls	2004	15	18.84
Bermuda	Girls	2005	15	18.90
Bermuda	Girls	2006	15	18.96
Bermuda	Girls	2007	15	19.03

Bermuda	Girls	2008	15	19.09
Bermuda	Girls	2009	15	19.15
Bermuda	Girls	2010	15	19.22
Bermuda	Girls	2011	15	19.28
Bermuda	Girls	2012	15	19.35
Bermuda	Girls	2013	15	19.42
Bermuda	Girls	2014	15	19.49
Bermuda	Girls	2015	15	19.57
Bermuda	Girls	2016	15	19.65
Bermuda	Girls	1975	16	18.65
Bermuda	Girls	1976	16	18.68
Bermuda	Girls	1977	16	18.71
Bermuda	Girls	1978	16	18.74
Bermuda	Girls	1979	16	18.78
Bermuda	Girls	1980	16	18.82
Bermuda	Girls	1981	16	18.87
Bermuda	Girls	1982	16	18.91
Bermuda	Girls	1983	16	18.96
Bermuda	Girls	1984	16	19.01
Bermuda	Girls	1985	16	19.06
Bermuda	Girls	1986	16	19.12
Bermuda	Girls	1987	16	19.18
Bermuda	Girls	1988	16	19.24
Bermuda	Girls	1989	16	19.30
Bermuda	Girls	1990	16	19.36
Bermuda	Girls	1991	16	19.42
Bermuda	Girls	1992	16	19.48
Bermuda	Girls	1993	16	19.55
Bermuda	Girls	1994	16	19.61
Bermuda	Girls	1995	16	19.68
Bermuda	Girls	1996	16	19.74
Bermuda	Girls	1997	16	19.80
Bermuda	Girls	1998	16	19.87
Bermuda	Girls	1999	16	19.93
Bermuda	Girls	2000	16	20.00
Bermuda	Girls	2001	16	20.06
Bermuda	Girls	2002	16	20.13
Bermuda	Girls	2003	16	20.19
Bermuda	Girls	2004	16	20.26
Bermuda	Girls	2005	16	20.32
Bermuda	Girls	2006	16	20.38
Bermuda	Girls	2007	16	20.44
Bermuda	Girls	2008	16	20.51
Bermuda	Girls	2009	16	20.57
Bermuda	Girls	2010	16	20.64
Bermuda	Girls	2011	16	20.71
Bermuda	Girls	2012	16	20.78
Bermuda	Girls	2013	16	20.85
Bermuda	Girls	2014	16	20.93
Bermuda	Girls	2015	16	21.01
Bermuda	Girls	2016	16	21.09
Bermuda	Girls	1975	17	19.80

Bermuda	Girls	1976	17	19.83
Bermuda	Girls	1977	17	19.86
Bermuda	Girls	1978	17	19.90
Bermuda	Girls	1979	17	19.94
Bermuda	Girls	1980	17	19.98
Bermuda	Girls	1981	17	20.02
Bermuda	Girls	1982	17	20.07
Bermuda	Girls	1983	17	20.12
Bermuda	Girls	1984	17	20.18
Bermuda	Girls	1985	17	20.23
Bermuda	Girls	1986	17	20.29
Bermuda	Girls	1987	17	20.35
Bermuda	Girls	1988	17	20.41
Bermuda	Girls	1989	17	20.48
Bermuda	Girls	1990	17	20.54
Bermuda	Girls	1991	17	20.61
Bermuda	Girls	1992	17	20.67
Bermuda	Girls	1993	17	20.74
Bermuda	Girls	1994	17	20.81
Bermuda	Girls	1995	17	20.87
Bermuda	Girls	1996	17	20.94
Bermuda	Girls	1997	17	21.01
Bermuda	Girls	1998	17	21.08
Bermuda	Girls	1999	17	21.14
Bermuda	Girls	2000	17	21.21
Bermuda	Girls	2001	17	21.28
Bermuda	Girls	2002	17	21.35
Bermuda	Girls	2003	17	21.41
Bermuda	Girls	2004	17	21.48
Bermuda	Girls	2005	17	21.55
Bermuda	Girls	2006	17	21.61
Bermuda	Girls	2007	17	21.68
Bermuda	Girls	2008	17	21.75
Bermuda	Girls	2009	17	21.82
Bermuda	Girls	2010	17	21.89
Bermuda	Girls	2011	17	21.96
Bermuda	Girls	2012	17	22.03
Bermuda	Girls	2013	17	22.11
Bermuda	Girls	2014	17	22.19
Bermuda	Girls	2015	17	22.27
Bermuda	Girls	2016	17	22.35
Bermuda	Girls	1975	18	20.96
Bermuda	Girls	1976	18	21.00
Bermuda	Girls	1977	18	21.04
Bermuda	Girls	1978	18	21.08
Bermuda	Girls	1979	18	21.13
Bermuda	Girls	1980	18	21.18
Bermuda	Girls	1981	18	21.23
Bermuda	Girls	1982	18	21.28
Bermuda	Girls	1983	18	21.33
Bermuda	Girls	1984	18	21.39
Bermuda	Girls	1985	18	21.46

Bermuda	Girls	1986	18	21.52
Bermuda	Girls	1987	18	21.59
Bermuda	Girls	1988	18	21.66
Bermuda	Girls	1989	18	21.73
Bermuda	Girls	1990	18	21.80
Bermuda	Girls	1991	18	21.87
Bermuda	Girls	1992	18	21.94
Bermuda	Girls	1993	18	22.01
Bermuda	Girls	1994	18	22.09
Bermuda	Girls	1995	18	22.16
Bermuda	Girls	1996	18	22.24
Bermuda	Girls	1997	18	22.31
Bermuda	Girls	1998	18	22.38
Bermuda	Girls	1999	18	22.46
Bermuda	Girls	2000	18	22.53
Bermuda	Girls	2001	18	22.61
Bermuda	Girls	2002	18	22.68
Bermuda	Girls	2003	18	22.75
Bermuda	Girls	2004	18	22.83
Bermuda	Girls	2005	18	22.90
Bermuda	Girls	2006	18	22.97
Bermuda	Girls	2007	18	23.04
Bermuda	Girls	2008	18	23.12
Bermuda	Girls	2009	18	23.19
Bermuda	Girls	2010	18	23.26
Bermuda	Girls	2011	18	23.34
Bermuda	Girls	2012	18	23.42
Bermuda	Girls	2013	18	23.50
Bermuda	Girls	2014	18	23.58
Bermuda	Girls	2015	18	23.67
Bermuda	Girls	2016	18	23.76
Bermuda	Girls	1975	Average	16.66
Bermuda	Girls	1976	Average	16.69
Bermuda	Girls	1977	Average	16.72
Bermuda	Girls	1978	Average	16.76
Bermuda	Girls	1979	Average	16.80
Bermuda	Girls	1980	Average	16.84
Bermuda	Girls	1981	Average	16.88
Bermuda	Girls	1982	Average	16.93
Bermuda	Girls	1983	Average	16.98
Bermuda	Girls	1984	Average	17.03
Bermuda	Girls	1985	Average	17.08
Bermuda	Girls	1986	Average	17.14
Bermuda	Girls	1987	Average	17.19
Bermuda	Girls	1988	Average	17.25
Bermuda	Girls	1989	Average	17.31
Bermuda	Girls	1990	Average	17.37
Bermuda	Girls	1991	Average	17.43
Bermuda	Girls	1992	Average	17.49
Bermuda	Girls	1993	Average	17.56
Bermuda	Girls	1994	Average	17.62
Bermuda	Girls	1995	Average	17.68

Bermuda	Girls	1996	Average	17.75
Bermuda	Girls	1997	Average	17.81
Bermuda	Girls	1998	Average	17.87
Bermuda	Girls	1999	Average	17.93
Bermuda	Girls	2000	Average	18.00
Bermuda	Girls	2001	Average	18.06
Bermuda	Girls	2002	Average	18.12
Bermuda	Girls	2003	Average	18.19
Bermuda	Girls	2004	Average	18.25
Bermuda	Girls	2005	Average	18.31
Bermuda	Girls	2006	Average	18.37
Bermuda	Girls	2007	Average	18.43
Bermuda	Girls	2008	Average	18.49
Bermuda	Girls	2009	Average	18.56
Bermuda	Girls	2010	Average	18.62
Bermuda	Girls	2011	Average	18.68
Bermuda	Girls	2012	Average	18.75
Bermuda	Girls	2013	Average	18.82
Bermuda	Girls	2014	Average	18.89
Bermuda	Girls	2015	Average	18.96
Bermuda	Girls	2016	Average	19.03
Bhutan	Boys	1975	12	3.32
Bhutan	Boys	1976	12	3.33
Bhutan	Boys	1977	12	3.35
Bhutan	Boys	1978	12	3.36
Bhutan	Boys	1979	12	3.38
Bhutan	Boys	1980	12	3.40
Bhutan	Boys	1981	12	3.42
Bhutan	Boys	1982	12	3.44
Bhutan	Boys	1983	12	3.46
Bhutan	Boys	1984	12	3.49
Bhutan	Boys	1985	12	3.51
Bhutan	Boys	1986	12	3.54
Bhutan	Boys	1987	12	3.57
Bhutan	Boys	1988	12	3.61
Bhutan	Boys	1989	12	3.64
Bhutan	Boys	1990	12	3.68
Bhutan	Boys	1991	12	3.72
Bhutan	Boys	1992	12	3.77
Bhutan	Boys	1993	12	3.82
Bhutan	Boys	1994	12	3.87
Bhutan	Boys	1995	12	3.93
Bhutan	Boys	1996	12	3.99
Bhutan	Boys	1997	12	4.06
Bhutan	Boys	1998	12	4.13
Bhutan	Boys	1999	12	4.21
Bhutan	Boys	2000	12	4.30
Bhutan	Boys	2001	12	4.39
Bhutan	Boys	2002	12	4.49
Bhutan	Boys	2003	12	4.59
Bhutan	Boys	2004	12	4.71
Bhutan	Boys	2005	12	4.83

Bhutan	Boys	2006	12	4.95
Bhutan	Boys	2007	12	5.09
Bhutan	Boys	2008	12	5.22
Bhutan	Boys	2009	12	5.37
Bhutan	Boys	2010	12	5.52
Bhutan	Boys	2011	12	5.67
Bhutan	Boys	2012	12	5.83
Bhutan	Boys	2013	12	6.00
Bhutan	Boys	2014	12	6.18
Bhutan	Boys	2015	12	6.36
Bhutan	Boys	2016	12	6.55
Bhutan	Boys	1975	13	4.12
Bhutan	Boys	1976	13	4.14
Bhutan	Boys	1977	13	4.16
Bhutan	Boys	1978	13	4.18
Bhutan	Boys	1979	13	4.20
Bhutan	Boys	1980	13	4.22
Bhutan	Boys	1981	13	4.25
Bhutan	Boys	1982	13	4.27
Bhutan	Boys	1983	13	4.30
Bhutan	Boys	1984	13	4.33
Bhutan	Boys	1985	13	4.36
Bhutan	Boys	1986	13	4.39
Bhutan	Boys	1987	13	4.43
Bhutan	Boys	1988	13	4.47
Bhutan	Boys	1989	13	4.51
Bhutan	Boys	1990	13	4.56
Bhutan	Boys	1991	13	4.61
Bhutan	Boys	1992	13	4.66
Bhutan	Boys	1993	13	4.72
Bhutan	Boys	1994	13	4.78
Bhutan	Boys	1995	13	4.84
Bhutan	Boys	1996	13	4.92
Bhutan	Boys	1997	13	4.99
Bhutan	Boys	1998	13	5.08
Bhutan	Boys	1999	13	5.17
Bhutan	Boys	2000	13	5.27
Bhutan	Boys	2001	13	5.37
Bhutan	Boys	2002	13	5.48
Bhutan	Boys	2003	13	5.61
Bhutan	Boys	2004	13	5.74
Bhutan	Boys	2005	13	5.87
Bhutan	Boys	2006	13	6.02
Bhutan	Boys	2007	13	6.17
Bhutan	Boys	2008	13	6.33
Bhutan	Boys	2009	13	6.49
Bhutan	Boys	2010	13	6.66
Bhutan	Boys	2011	13	6.84
Bhutan	Boys	2012	13	7.03
Bhutan	Boys	2013	13	7.23
Bhutan	Boys	2014	13	7.43
Bhutan	Boys	2015	13	7.65

Bhutan	Boys	2016	13	7.87
Bhutan	Boys	1975	14	5.19
Bhutan	Boys	1976	14	5.21
Bhutan	Boys	1977	14	5.24
Bhutan	Boys	1978	14	5.26
Bhutan	Boys	1979	14	5.29
Bhutan	Boys	1980	14	5.31
Bhutan	Boys	1981	14	5.34
Bhutan	Boys	1982	14	5.37
Bhutan	Boys	1983	14	5.41
Bhutan	Boys	1984	14	5.44
Bhutan	Boys	1985	14	5.48
Bhutan	Boys	1986	14	5.52
Bhutan	Boys	1987	14	5.56
Bhutan	Boys	1988	14	5.60
Bhutan	Boys	1989	14	5.65
Bhutan	Boys	1990	14	5.70
Bhutan	Boys	1991	14	5.76
Bhutan	Boys	1992	14	5.82
Bhutan	Boys	1993	14	5.88
Bhutan	Boys	1994	14	5.95
Bhutan	Boys	1995	14	6.02
Bhutan	Boys	1996	14	6.10
Bhutan	Boys	1997	14	6.19
Bhutan	Boys	1998	14	6.28
Bhutan	Boys	1999	14	6.38
Bhutan	Boys	2000	14	6.49
Bhutan	Boys	2001	14	6.60
Bhutan	Boys	2002	14	6.73
Bhutan	Boys	2003	14	6.86
Bhutan	Boys	2004	14	7.00
Bhutan	Boys	2005	14	7.15
Bhutan	Boys	2006	14	7.31
Bhutan	Boys	2007	14	7.48
Bhutan	Boys	2008	14	7.65
Bhutan	Boys	2009	14	7.83
Bhutan	Boys	2010	14	8.02
Bhutan	Boys	2011	14	8.21
Bhutan	Boys	2012	14	8.42
Bhutan	Boys	2013	14	8.64
Bhutan	Boys	2014	14	8.86
Bhutan	Boys	2015	14	9.10
Bhutan	Boys	2016	14	9.35
Bhutan	Boys	1975	15	6.48
Bhutan	Boys	1976	15	6.51
Bhutan	Boys	1977	15	6.53
Bhutan	Boys	1978	15	6.56
Bhutan	Boys	1979	15	6.60
Bhutan	Boys	1980	15	6.63
Bhutan	Boys	1981	15	6.66
Bhutan	Boys	1982	15	6.70
Bhutan	Boys	1983	15	6.74

Bhutan	Boys	1984	15	6.78
Bhutan	Boys	1985	15	6.82
Bhutan	Boys	1986	15	6.86
Bhutan	Boys	1987	15	6.91
Bhutan	Boys	1988	15	6.96
Bhutan	Boys	1989	15	7.01
Bhutan	Boys	1990	15	7.07
Bhutan	Boys	1991	15	7.13
Bhutan	Boys	1992	15	7.20
Bhutan	Boys	1993	15	7.27
Bhutan	Boys	1994	15	7.34
Bhutan	Boys	1995	15	7.42
Bhutan	Boys	1996	15	7.51
Bhutan	Boys	1997	15	7.60
Bhutan	Boys	1998	15	7.70
Bhutan	Boys	1999	15	7.81
Bhutan	Boys	2000	15	7.92
Bhutan	Boys	2001	15	8.05
Bhutan	Boys	2002	15	8.18
Bhutan	Boys	2003	15	8.32
Bhutan	Boys	2004	15	8.47
Bhutan	Boys	2005	15	8.63
Bhutan	Boys	2006	15	8.79
Bhutan	Boys	2007	15	8.97
Bhutan	Boys	2008	15	9.15
Bhutan	Boys	2009	15	9.34
Bhutan	Boys	2010	15	9.54
Bhutan	Boys	2011	15	9.74
Bhutan	Boys	2012	15	9.96
Bhutan	Boys	2013	15	10.19
Bhutan	Boys	2014	15	10.43
Bhutan	Boys	2015	15	10.68
Bhutan	Boys	2016	15	10.94
Bhutan	Boys	1975	16	7.92
Bhutan	Boys	1976	16	7.95
Bhutan	Boys	1977	16	7.98
Bhutan	Boys	1978	16	8.02
Bhutan	Boys	1979	16	8.06
Bhutan	Boys	1980	16	8.10
Bhutan	Boys	1981	16	8.14
Bhutan	Boys	1982	16	8.18
Bhutan	Boys	1983	16	8.22
Bhutan	Boys	1984	16	8.27
Bhutan	Boys	1985	16	8.32
Bhutan	Boys	1986	16	8.37
Bhutan	Boys	1987	16	8.42
Bhutan	Boys	1988	16	8.48
Bhutan	Boys	1989	16	8.54
Bhutan	Boys	1990	16	8.60
Bhutan	Boys	1991	16	8.67
Bhutan	Boys	1992	16	8.74
Bhutan	Boys	1993	16	8.81

Bhutan	Boys	1994	16	8.90
Bhutan	Boys	1995	16	8.98
Bhutan	Boys	1996	16	9.07
Bhutan	Boys	1997	16	9.17
Bhutan	Boys	1998	16	9.28
Bhutan	Boys	1999	16	9.39
Bhutan	Boys	2000	16	9.51
Bhutan	Boys	2001	16	9.64
Bhutan	Boys	2002	16	9.78
Bhutan	Boys	2003	16	9.93
Bhutan	Boys	2004	16	10.08
Bhutan	Boys	2005	16	10.25
Bhutan	Boys	2006	16	10.42
Bhutan	Boys	2007	16	10.60
Bhutan	Boys	2008	16	10.79
Bhutan	Boys	2009	16	10.99
Bhutan	Boys	2010	16	11.19
Bhutan	Boys	2011	16	11.41
Bhutan	Boys	2012	16	11.63
Bhutan	Boys	2013	16	11.87
Bhutan	Boys	2014	16	12.12
Bhutan	Boys	2015	16	12.37
Bhutan	Boys	2016	16	12.64
Bhutan	Boys	1975	17	9.40
Bhutan	Boys	1976	17	9.45
Bhutan	Boys	1977	17	9.49
Bhutan	Boys	1978	17	9.53
Bhutan	Boys	1979	17	9.58
Bhutan	Boys	1980	17	9.62
Bhutan	Boys	1981	17	9.67
Bhutan	Boys	1982	17	9.72
Bhutan	Boys	1983	17	9.77
Bhutan	Boys	1984	17	9.82
Bhutan	Boys	1985	17	9.87
Bhutan	Boys	1986	17	9.93
Bhutan	Boys	1987	17	9.99
Bhutan	Boys	1988	17	10.06
Bhutan	Boys	1989	17	10.12
Bhutan	Boys	1990	17	10.19
Bhutan	Boys	1991	17	10.27
Bhutan	Boys	1992	17	10.34
Bhutan	Boys	1993	17	10.43
Bhutan	Boys	1994	17	10.52
Bhutan	Boys	1995	17	10.61
Bhutan	Boys	1996	17	10.71
Bhutan	Boys	1997	17	10.82
Bhutan	Boys	1998	17	10.93
Bhutan	Boys	1999	17	11.05
Bhutan	Boys	2000	17	11.18
Bhutan	Boys	2001	17	11.32
Bhutan	Boys	2002	17	11.46
Bhutan	Boys	2003	17	11.62

Bhutan	Boys	2004	17	11.78
Bhutan	Boys	2005	17	11.96
Bhutan	Boys	2006	17	12.14
Bhutan	Boys	2007	17	12.33
Bhutan	Boys	2008	17	12.53
Bhutan	Boys	2009	17	12.73
Bhutan	Boys	2010	17	12.95
Bhutan	Boys	2011	17	13.17
Bhutan	Boys	2012	17	13.40
Bhutan	Boys	2013	17	13.65
Bhutan	Boys	2014	17	13.90
Bhutan	Boys	2015	17	14.17
Bhutan	Boys	2016	17	14.45
Bhutan	Boys	1975	18	10.84
Bhutan	Boys	1976	18	10.89
Bhutan	Boys	1977	18	10.94
Bhutan	Boys	1978	18	10.99
Bhutan	Boys	1979	18	11.04
Bhutan	Boys	1980	18	11.10
Bhutan	Boys	1981	18	11.15
Bhutan	Boys	1982	18	11.21
Bhutan	Boys	1983	18	11.27
Bhutan	Boys	1984	18	11.33
Bhutan	Boys	1985	18	11.39
Bhutan	Boys	1986	18	11.46
Bhutan	Boys	1987	18	11.52
Bhutan	Boys	1988	18	11.60
Bhutan	Boys	1989	18	11.67
Bhutan	Boys	1990	18	11.75
Bhutan	Boys	1991	18	11.83
Bhutan	Boys	1992	18	11.92
Bhutan	Boys	1993	18	12.02
Bhutan	Boys	1994	18	12.11
Bhutan	Boys	1995	18	12.22
Bhutan	Boys	1996	18	12.33
Bhutan	Boys	1997	18	12.45
Bhutan	Boys	1998	18	12.57
Bhutan	Boys	1999	18	12.71
Bhutan	Boys	2000	18	12.85
Bhutan	Boys	2001	18	13.00
Bhutan	Boys	2002	18	13.16
Bhutan	Boys	2003	18	13.33
Bhutan	Boys	2004	18	13.51
Bhutan	Boys	2005	18	13.69
Bhutan	Boys	2006	18	13.89
Bhutan	Boys	2007	18	14.10
Bhutan	Boys	2008	18	14.31
Bhutan	Boys	2009	18	14.53
Bhutan	Boys	2010	18	14.76
Bhutan	Boys	2011	18	15.00
Bhutan	Boys	2012	18	15.25
Bhutan	Boys	2013	18	15.51

Bhutan	Boys	2014	18	15.78
Bhutan	Boys	2015	18	16.07
Bhutan	Boys	2016	18	16.36
Bhutan	Boys	1975	Average	6.75
Bhutan	Boys	1976	Average	6.78
Bhutan	Boys	1977	Average	6.81
Bhutan	Boys	1978	Average	6.84
Bhutan	Boys	1979	Average	6.88
Bhutan	Boys	1980	Average	6.91
Bhutan	Boys	1981	Average	6.95
Bhutan	Boys	1982	Average	6.98
Bhutan	Boys	1983	Average	7.02
Bhutan	Boys	1984	Average	7.06
Bhutan	Boys	1985	Average	7.11
Bhutan	Boys	1986	Average	7.15
Bhutan	Boys	1987	Average	7.20
Bhutan	Boys	1988	Average	7.25
Bhutan	Boys	1989	Average	7.31
Bhutan	Boys	1990	Average	7.37
Bhutan	Boys	1991	Average	7.43
Bhutan	Boys	1992	Average	7.49
Bhutan	Boys	1993	Average	7.56
Bhutan	Boys	1994	Average	7.64
Bhutan	Boys	1995	Average	7.72
Bhutan	Boys	1996	Average	7.80
Bhutan	Boys	1997	Average	7.90
Bhutan	Boys	1998	Average	8.00
Bhutan	Boys	1999	Average	8.10
Bhutan	Boys	2000	Average	8.22
Bhutan	Boys	2001	Average	8.34
Bhutan	Boys	2002	Average	8.47
Bhutan	Boys	2003	Average	8.61
Bhutan	Boys	2004	Average	8.76
Bhutan	Boys	2005	Average	8.91
Bhutan	Boys	2006	Average	9.08
Bhutan	Boys	2007	Average	9.25
Bhutan	Boys	2008	Average	9.42
Bhutan	Boys	2009	Average	9.61
Bhutan	Boys	2010	Average	9.80
Bhutan	Boys	2011	Average	10.01
Bhutan	Boys	2012	Average	10.22
Bhutan	Boys	2013	Average	10.44
Bhutan	Boys	2014	Average	10.67
Bhutan	Boys	2015	Average	10.91
Bhutan	Boys	2016	Average	11.17
Bhutan	Girls	1975	12	3.82
Bhutan	Girls	1976	12	3.83
Bhutan	Girls	1977	12	3.85
Bhutan	Girls	1978	12	3.86
Bhutan	Girls	1979	12	3.88
Bhutan	Girls	1980	12	3.90
Bhutan	Girls	1981	12	3.91

Bhutan	Girls	1982	12	3.93
Bhutan	Girls	1983	12	3.95
Bhutan	Girls	1984	12	3.98
Bhutan	Girls	1985	12	4.00
Bhutan	Girls	1986	12	4.02
Bhutan	Girls	1987	12	4.05
Bhutan	Girls	1988	12	4.07
Bhutan	Girls	1989	12	4.10
Bhutan	Girls	1990	12	4.13
Bhutan	Girls	1991	12	4.17
Bhutan	Girls	1992	12	4.20
Bhutan	Girls	1993	12	4.24
Bhutan	Girls	1994	12	4.28
Bhutan	Girls	1995	12	4.32
Bhutan	Girls	1996	12	4.37
Bhutan	Girls	1997	12	4.41
Bhutan	Girls	1998	12	4.47
Bhutan	Girls	1999	12	4.52
Bhutan	Girls	2000	12	4.58
Bhutan	Girls	2001	12	4.64
Bhutan	Girls	2002	12	4.71
Bhutan	Girls	2003	12	4.78
Bhutan	Girls	2004	12	4.85
Bhutan	Girls	2005	12	4.93
Bhutan	Girls	2006	12	5.02
Bhutan	Girls	2007	12	5.10
Bhutan	Girls	2008	12	5.19
Bhutan	Girls	2009	12	5.28
Bhutan	Girls	2010	12	5.37
Bhutan	Girls	2011	12	5.47
Bhutan	Girls	2012	12	5.58
Bhutan	Girls	2013	12	5.68
Bhutan	Girls	2014	12	5.79
Bhutan	Girls	2015	12	5.91
Bhutan	Girls	2016	12	6.03
Bhutan	Girls	1975	13	4.83
Bhutan	Girls	1976	13	4.85
Bhutan	Girls	1977	13	4.87
Bhutan	Girls	1978	13	4.89
Bhutan	Girls	1979	13	4.91
Bhutan	Girls	1980	13	4.93
Bhutan	Girls	1981	13	4.95
Bhutan	Girls	1982	13	4.98
Bhutan	Girls	1983	13	5.00
Bhutan	Girls	1984	13	5.03
Bhutan	Girls	1985	13	5.06
Bhutan	Girls	1986	13	5.09
Bhutan	Girls	1987	13	5.12
Bhutan	Girls	1988	13	5.15
Bhutan	Girls	1989	13	5.19
Bhutan	Girls	1990	13	5.22
Bhutan	Girls	1991	13	5.26

Bhutan	Girls	1992	13	5.31
Bhutan	Girls	1993	13	5.35
Bhutan	Girls	1994	13	5.40
Bhutan	Girls	1995	13	5.45
Bhutan	Girls	1996	13	5.50
Bhutan	Girls	1997	13	5.56
Bhutan	Girls	1998	13	5.62
Bhutan	Girls	1999	13	5.69
Bhutan	Girls	2000	13	5.76
Bhutan	Girls	2001	13	5.83
Bhutan	Girls	2002	13	5.91
Bhutan	Girls	2003	13	5.99
Bhutan	Girls	2004	13	6.08
Bhutan	Girls	2005	13	6.17
Bhutan	Girls	2006	13	6.26
Bhutan	Girls	2007	13	6.36
Bhutan	Girls	2008	13	6.46
Bhutan	Girls	2009	13	6.57
Bhutan	Girls	2010	13	6.68
Bhutan	Girls	2011	13	6.79
Bhutan	Girls	2012	13	6.91
Bhutan	Girls	2013	13	7.04
Bhutan	Girls	2014	13	7.16
Bhutan	Girls	2015	13	7.30
Bhutan	Girls	2016	13	7.44
Bhutan	Girls	1975	14	5.99
Bhutan	Girls	1976	14	6.02
Bhutan	Girls	1977	14	6.04
Bhutan	Girls	1978	14	6.07
Bhutan	Girls	1979	14	6.09
Bhutan	Girls	1980	14	6.12
Bhutan	Girls	1981	14	6.15
Bhutan	Girls	1982	14	6.18
Bhutan	Girls	1983	14	6.21
Bhutan	Girls	1984	14	6.24
Bhutan	Girls	1985	14	6.27
Bhutan	Girls	1986	14	6.31
Bhutan	Girls	1987	14	6.35
Bhutan	Girls	1988	14	6.38
Bhutan	Girls	1989	14	6.43
Bhutan	Girls	1990	14	6.47
Bhutan	Girls	1991	14	6.52
Bhutan	Girls	1992	14	6.56
Bhutan	Girls	1993	14	6.62
Bhutan	Girls	1994	14	6.67
Bhutan	Girls	1995	14	6.73
Bhutan	Girls	1996	14	6.79
Bhutan	Girls	1997	14	6.86
Bhutan	Girls	1998	14	6.93
Bhutan	Girls	1999	14	7.00
Bhutan	Girls	2000	14	7.08
Bhutan	Girls	2001	14	7.16

Bhutan	Girls	2002	14	7.25
Bhutan	Girls	2003	14	7.34
Bhutan	Girls	2004	14	7.44
Bhutan	Girls	2005	14	7.54
Bhutan	Girls	2006	14	7.65
Bhutan	Girls	2007	14	7.75
Bhutan	Girls	2008	14	7.87
Bhutan	Girls	2009	14	7.98
Bhutan	Girls	2010	14	8.10
Bhutan	Girls	2011	14	8.23
Bhutan	Girls	2012	14	8.36
Bhutan	Girls	2013	14	8.49
Bhutan	Girls	2014	14	8.63
Bhutan	Girls	2015	14	8.78
Bhutan	Girls	2016	14	8.93
Bhutan	Girls	1975	15	7.15
Bhutan	Girls	1976	15	7.17
Bhutan	Girls	1977	15	7.20
Bhutan	Girls	1978	15	7.23
Bhutan	Girls	1979	15	7.26
Bhutan	Girls	1980	15	7.30
Bhutan	Girls	1981	15	7.33
Bhutan	Girls	1982	15	7.36
Bhutan	Girls	1983	15	7.40
Bhutan	Girls	1984	15	7.43
Bhutan	Girls	1985	15	7.47
Bhutan	Girls	1986	15	7.51
Bhutan	Girls	1987	15	7.55
Bhutan	Girls	1988	15	7.60
Bhutan	Girls	1989	15	7.64
Bhutan	Girls	1990	15	7.69
Bhutan	Girls	1991	15	7.74
Bhutan	Girls	1992	15	7.80
Bhutan	Girls	1993	15	7.86
Bhutan	Girls	1994	15	7.92
Bhutan	Girls	1995	15	7.98
Bhutan	Girls	1996	15	8.05
Bhutan	Girls	1997	15	8.12
Bhutan	Girls	1998	15	8.20
Bhutan	Girls	1999	15	8.28
Bhutan	Girls	2000	15	8.36
Bhutan	Girls	2001	15	8.45
Bhutan	Girls	2002	15	8.54
Bhutan	Girls	2003	15	8.64
Bhutan	Girls	2004	15	8.75
Bhutan	Girls	2005	15	8.86
Bhutan	Girls	2006	15	8.97
Bhutan	Girls	2007	15	9.09
Bhutan	Girls	2008	15	9.21
Bhutan	Girls	2009	15	9.33
Bhutan	Girls	2010	15	9.46
Bhutan	Girls	2011	15	9.59

Bhutan	Girls	2012	15	9.73
Bhutan	Girls	2013	15	9.87
Bhutan	Girls	2014	15	10.02
Bhutan	Girls	2015	15	10.17
Bhutan	Girls	2016	15	10.33
Bhutan	Girls	1975	16	8.11
Bhutan	Girls	1976	16	8.14
Bhutan	Girls	1977	16	8.18
Bhutan	Girls	1978	16	8.21
Bhutan	Girls	1979	16	8.24
Bhutan	Girls	1980	16	8.28
Bhutan	Girls	1981	16	8.31
Bhutan	Girls	1982	16	8.35
Bhutan	Girls	1983	16	8.39
Bhutan	Girls	1984	16	8.43
Bhutan	Girls	1985	16	8.47
Bhutan	Girls	1986	16	8.52
Bhutan	Girls	1987	16	8.56
Bhutan	Girls	1988	16	8.61
Bhutan	Girls	1989	16	8.66
Bhutan	Girls	1990	16	8.71
Bhutan	Girls	1991	16	8.77
Bhutan	Girls	1992	16	8.83
Bhutan	Girls	1993	16	8.89
Bhutan	Girls	1994	16	8.95
Bhutan	Girls	1995	16	9.02
Bhutan	Girls	1996	16	9.09
Bhutan	Girls	1997	16	9.17
Bhutan	Girls	1998	16	9.25
Bhutan	Girls	1999	16	9.33
Bhutan	Girls	2000	16	9.42
Bhutan	Girls	2001	16	9.52
Bhutan	Girls	2002	16	9.62
Bhutan	Girls	2003	16	9.72
Bhutan	Girls	2004	16	9.83
Bhutan	Girls	2005	16	9.95
Bhutan	Girls	2006	16	10.06
Bhutan	Girls	2007	16	10.19
Bhutan	Girls	2008	16	10.31
Bhutan	Girls	2009	16	10.44
Bhutan	Girls	2010	16	10.58
Bhutan	Girls	2011	16	10.71
Bhutan	Girls	2012	16	10.86
Bhutan	Girls	2013	16	11.01
Bhutan	Girls	2014	16	11.16
Bhutan	Girls	2015	16	11.32
Bhutan	Girls	2016	16	11.49
Bhutan	Girls	1975	17	8.78
Bhutan	Girls	1976	17	8.81
Bhutan	Girls	1977	17	8.85
Bhutan	Girls	1978	17	8.89
Bhutan	Girls	1979	17	8.92

Bhutan	Girls	1980	17	8.96
Bhutan	Girls	1981	17	9.00
Bhutan	Girls	1982	17	9.04
Bhutan	Girls	1983	17	9.08
Bhutan	Girls	1984	17	9.13
Bhutan	Girls	1985	17	9.17
Bhutan	Girls	1986	17	9.22
Bhutan	Girls	1987	17	9.27
Bhutan	Girls	1988	17	9.32
Bhutan	Girls	1989	17	9.37
Bhutan	Girls	1990	17	9.43
Bhutan	Girls	1991	17	9.49
Bhutan	Girls	1992	17	9.55
Bhutan	Girls	1993	17	9.61
Bhutan	Girls	1994	17	9.68
Bhutan	Girls	1995	17	9.75
Bhutan	Girls	1996	17	9.83
Bhutan	Girls	1997	17	9.91
Bhutan	Girls	1998	17	10.00
Bhutan	Girls	1999	17	10.09
Bhutan	Girls	2000	17	10.18
Bhutan	Girls	2001	17	10.28
Bhutan	Girls	2002	17	10.39
Bhutan	Girls	2003	17	10.50
Bhutan	Girls	2004	17	10.62
Bhutan	Girls	2005	17	10.74
Bhutan	Girls	2006	17	10.86
Bhutan	Girls	2007	17	10.99
Bhutan	Girls	2008	17	11.12
Bhutan	Girls	2009	17	11.26
Bhutan	Girls	2010	17	11.40
Bhutan	Girls	2011	17	11.55
Bhutan	Girls	2012	17	11.70
Bhutan	Girls	2013	17	11.86
Bhutan	Girls	2014	17	12.02
Bhutan	Girls	2015	17	12.19
Bhutan	Girls	2016	17	12.37
Bhutan	Girls	1975	18	9.15
Bhutan	Girls	1976	18	9.19
Bhutan	Girls	1977	18	9.23
Bhutan	Girls	1978	18	9.27
Bhutan	Girls	1979	18	9.31
Bhutan	Girls	1980	18	9.35
Bhutan	Girls	1981	18	9.39
Bhutan	Girls	1982	18	9.43
Bhutan	Girls	1983	18	9.48
Bhutan	Girls	1984	18	9.52
Bhutan	Girls	1985	18	9.57
Bhutan	Girls	1986	18	9.62
Bhutan	Girls	1987	18	9.68
Bhutan	Girls	1988	18	9.73
Bhutan	Girls	1989	18	9.79

Bhutan	Girls	1990	18	9.85
Bhutan	Girls	1991	18	9.91
Bhutan	Girls	1992	18	9.98
Bhutan	Girls	1993	18	10.05
Bhutan	Girls	1994	18	10.13
Bhutan	Girls	1995	18	10.21
Bhutan	Girls	1996	18	10.29
Bhutan	Girls	1997	18	10.38
Bhutan	Girls	1998	18	10.47
Bhutan	Girls	1999	18	10.57
Bhutan	Girls	2000	18	10.67
Bhutan	Girls	2001	18	10.78
Bhutan	Girls	2002	18	10.90
Bhutan	Girls	2003	18	11.02
Bhutan	Girls	2004	18	11.15
Bhutan	Girls	2005	18	11.28
Bhutan	Girls	2006	18	11.42
Bhutan	Girls	2007	18	11.57
Bhutan	Girls	2008	18	11.71
Bhutan	Girls	2009	18	11.86
Bhutan	Girls	2010	18	12.02
Bhutan	Girls	2011	18	12.18
Bhutan	Girls	2012	18	12.35
Bhutan	Girls	2013	18	12.52
Bhutan	Girls	2014	18	12.70
Bhutan	Girls	2015	18	12.89
Bhutan	Girls	2016	18	13.08
Bhutan	Girls	1975	Average	6.83
Bhutan	Girls	1976	Average	6.86
Bhutan	Girls	1977	Average	6.89
Bhutan	Girls	1978	Average	6.92
Bhutan	Girls	1979	Average	6.94
Bhutan	Girls	1980	Average	6.98
Bhutan	Girls	1981	Average	7.01
Bhutan	Girls	1982	Average	7.04
Bhutan	Girls	1983	Average	7.07
Bhutan	Girls	1984	Average	7.11
Bhutan	Girls	1985	Average	7.14
Bhutan	Girls	1986	Average	7.18
Bhutan	Girls	1987	Average	7.22
Bhutan	Girls	1988	Average	7.27
Bhutan	Girls	1989	Average	7.31
Bhutan	Girls	1990	Average	7.36
Bhutan	Girls	1991	Average	7.41
Bhutan	Girls	1992	Average	7.46
Bhutan	Girls	1993	Average	7.52
Bhutan	Girls	1994	Average	7.58
Bhutan	Girls	1995	Average	7.64
Bhutan	Girls	1996	Average	7.70
Bhutan	Girls	1997	Average	7.77
Bhutan	Girls	1998	Average	7.85
Bhutan	Girls	1999	Average	7.93

Bhutan	Girls	2000	Average	8.01
Bhutan	Girls	2001	Average	8.10
Bhutan	Girls	2002	Average	8.19
Bhutan	Girls	2003	Average	8.29
Bhutan	Girls	2004	Average	8.39
Bhutan	Girls	2005	Average	8.50
Bhutan	Girls	2006	Average	8.61
Bhutan	Girls	2007	Average	8.72
Bhutan	Girls	2008	Average	8.84
Bhutan	Girls	2009	Average	8.96
Bhutan	Girls	2010	Average	9.09
Bhutan	Girls	2011	Average	9.22
Bhutan	Girls	2012	Average	9.36
Bhutan	Girls	2013	Average	9.50
Bhutan	Girls	2014	Average	9.64
Bhutan	Girls	2015	Average	9.79
Bhutan	Girls	2016	Average	9.95
Bolivia	Boys	1975	12	5.90
Bolivia	Boys	1976	12	5.96
Bolivia	Boys	1977	12	6.02
Bolivia	Boys	1978	12	6.08
Bolivia	Boys	1979	12	6.15
Bolivia	Boys	1980	12	6.23
Bolivia	Boys	1981	12	6.32
Bolivia	Boys	1982	12	6.41
Bolivia	Boys	1983	12	6.52
Bolivia	Boys	1984	12	6.63
Bolivia	Boys	1985	12	6.74
Bolivia	Boys	1986	12	6.86
Bolivia	Boys	1987	12	6.99
Bolivia	Boys	1988	12	7.13
Bolivia	Boys	1989	12	7.27
Bolivia	Boys	1990	12	7.41
Bolivia	Boys	1991	12	7.56
Bolivia	Boys	1992	12	7.70
Bolivia	Boys	1993	12	7.85
Bolivia	Boys	1994	12	8.01
Bolivia	Boys	1995	12	8.16
Bolivia	Boys	1996	12	8.31
Bolivia	Boys	1997	12	8.46
Bolivia	Boys	1998	12	8.61
Bolivia	Boys	1999	12	8.76
Bolivia	Boys	2000	12	8.91
Bolivia	Boys	2001	12	9.06
Bolivia	Boys	2002	12	9.21
Bolivia	Boys	2003	12	9.36
Bolivia	Boys	2004	12	9.52
Bolivia	Boys	2005	12	9.67
Bolivia	Boys	2006	12	9.83
Bolivia	Boys	2007	12	9.99
Bolivia	Boys	2008	12	10.16
Bolivia	Boys	2009	12	10.32

Bolivia	Boys	2010	12	10.49
Bolivia	Boys	2011	12	10.66
Bolivia	Boys	2012	12	10.84
Bolivia	Boys	2013	12	11.01
Bolivia	Boys	2014	12	11.19
Bolivia	Boys	2015	12	11.36
Bolivia	Boys	2016	12	11.54
Bolivia	Boys	1975	13	7.33
Bolivia	Boys	1976	13	7.40
Bolivia	Boys	1977	13	7.47
Bolivia	Boys	1978	13	7.54
Bolivia	Boys	1979	13	7.63
Bolivia	Boys	1980	13	7.72
Bolivia	Boys	1981	13	7.82
Bolivia	Boys	1982	13	7.93
Bolivia	Boys	1983	13	8.05
Bolivia	Boys	1984	13	8.18
Bolivia	Boys	1985	13	8.31
Bolivia	Boys	1986	13	8.45
Bolivia	Boys	1987	13	8.60
Bolivia	Boys	1988	13	8.76
Bolivia	Boys	1989	13	8.92
Bolivia	Boys	1990	13	9.09
Bolivia	Boys	1991	13	9.26
Bolivia	Boys	1992	13	9.44
Bolivia	Boys	1993	13	9.61
Bolivia	Boys	1994	13	9.79
Bolivia	Boys	1995	13	9.97
Bolivia	Boys	1996	13	10.15
Bolivia	Boys	1997	13	10.32
Bolivia	Boys	1998	13	10.50
Bolivia	Boys	1999	13	10.67
Bolivia	Boys	2000	13	10.85
Bolivia	Boys	2001	13	11.03
Bolivia	Boys	2002	13	11.21
Bolivia	Boys	2003	13	11.39
Bolivia	Boys	2004	13	11.57
Bolivia	Boys	2005	13	11.76
Bolivia	Boys	2006	13	11.94
Bolivia	Boys	2007	13	12.14
Bolivia	Boys	2008	13	12.33
Bolivia	Boys	2009	13	12.54
Bolivia	Boys	2010	13	12.74
Bolivia	Boys	2011	13	12.95
Bolivia	Boys	2012	13	13.15
Bolivia	Boys	2013	13	13.37
Bolivia	Boys	2014	13	13.58
Bolivia	Boys	2015	13	13.80
Bolivia	Boys	2016	13	14.02
Bolivia	Boys	1975	14	9.12
Bolivia	Boys	1976	14	9.19
Bolivia	Boys	1977	14	9.27

Bolivia	Boys	1978	14	9.35
Bolivia	Boys	1979	14	9.45
Bolivia	Boys	1980	14	9.55
Bolivia	Boys	1981	14	9.66
Bolivia	Boys	1982	14	9.79
Bolivia	Boys	1983	14	9.92
Bolivia	Boys	1984	14	10.06
Bolivia	Boys	1985	14	10.21
Bolivia	Boys	1986	14	10.36
Bolivia	Boys	1987	14	10.53
Bolivia	Boys	1988	14	10.70
Bolivia	Boys	1989	14	10.88
Bolivia	Boys	1990	14	11.07
Bolivia	Boys	1991	14	11.25
Bolivia	Boys	1992	14	11.45
Bolivia	Boys	1993	14	11.64
Bolivia	Boys	1994	14	11.84
Bolivia	Boys	1995	14	12.04
Bolivia	Boys	1996	14	12.23
Bolivia	Boys	1997	14	12.43
Bolivia	Boys	1998	14	12.62
Bolivia	Boys	1999	14	12.82
Bolivia	Boys	2000	14	13.02
Bolivia	Boys	2001	14	13.21
Bolivia	Boys	2002	14	13.41
Bolivia	Boys	2003	14	13.61
Bolivia	Boys	2004	14	13.82
Bolivia	Boys	2005	14	14.03
Bolivia	Boys	2006	14	14.24
Bolivia	Boys	2007	14	14.46
Bolivia	Boys	2008	14	14.68
Bolivia	Boys	2009	14	14.90
Bolivia	Boys	2010	14	15.13
Bolivia	Boys	2011	14	15.37
Bolivia	Boys	2012	14	15.61
Bolivia	Boys	2013	14	15.85
Bolivia	Boys	2014	14	16.10
Bolivia	Boys	2015	14	16.35
Bolivia	Boys	2016	14	16.61
Bolivia	Boys	1975	15	11.18
Bolivia	Boys	1976	15	11.25
Bolivia	Boys	1977	15	11.34
Bolivia	Boys	1978	15	11.43
Bolivia	Boys	1979	15	11.53
Bolivia	Boys	1980	15	11.64
Bolivia	Boys	1981	15	11.76
Bolivia	Boys	1982	15	11.89
Bolivia	Boys	1983	15	12.03
Bolivia	Boys	1984	15	12.18
Bolivia	Boys	1985	15	12.33
Bolivia	Boys	1986	15	12.50
Bolivia	Boys	1987	15	12.67

Bolivia	Boys	1988	15	12.85
Bolivia	Boys	1989	15	13.04
Bolivia	Boys	1990	15	13.24
Bolivia	Boys	1991	15	13.44
Bolivia	Boys	1992	15	13.64
Bolivia	Boys	1993	15	13.84
Bolivia	Boys	1994	15	14.05
Bolivia	Boys	1995	15	14.26
Bolivia	Boys	1996	15	14.47
Bolivia	Boys	1997	15	14.67
Bolivia	Boys	1998	15	14.88
Bolivia	Boys	1999	15	15.08
Bolivia	Boys	2000	15	15.29
Bolivia	Boys	2001	15	15.50
Bolivia	Boys	2002	15	15.71
Bolivia	Boys	2003	15	15.92
Bolivia	Boys	2004	15	16.14
Bolivia	Boys	2005	15	16.36
Bolivia	Boys	2006	15	16.59
Bolivia	Boys	2007	15	16.82
Bolivia	Boys	2008	15	17.06
Bolivia	Boys	2009	15	17.30
Bolivia	Boys	2010	15	17.54
Bolivia	Boys	2011	15	17.80
Bolivia	Boys	2012	15	18.05
Bolivia	Boys	2013	15	18.31
Bolivia	Boys	2014	15	18.58
Bolivia	Boys	2015	15	18.85
Bolivia	Boys	2016	15	19.13
Bolivia	Boys	1975	16	13.42
Bolivia	Boys	1976	16	13.50
Bolivia	Boys	1977	16	13.59
Bolivia	Boys	1978	16	13.69
Bolivia	Boys	1979	16	13.80
Bolivia	Boys	1980	16	13.91
Bolivia	Boys	1981	16	14.03
Bolivia	Boys	1982	16	14.17
Bolivia	Boys	1983	16	14.31
Bolivia	Boys	1984	16	14.46
Bolivia	Boys	1985	16	14.62
Bolivia	Boys	1986	16	14.79
Bolivia	Boys	1987	16	14.97
Bolivia	Boys	1988	16	15.15
Bolivia	Boys	1989	16	15.34
Bolivia	Boys	1990	16	15.54
Bolivia	Boys	1991	16	15.75
Bolivia	Boys	1992	16	15.95
Bolivia	Boys	1993	16	16.16
Bolivia	Boys	1994	16	16.37
Bolivia	Boys	1995	16	16.58
Bolivia	Boys	1996	16	16.80
Bolivia	Boys	1997	16	17.01

Bolivia	Boys	1998	16	17.22
Bolivia	Boys	1999	16	17.43
Bolivia	Boys	2000	16	17.64
Bolivia	Boys	2001	16	17.85
Bolivia	Boys	2002	16	18.07
Bolivia	Boys	2003	16	18.29
Bolivia	Boys	2004	16	18.51
Bolivia	Boys	2005	16	18.74
Bolivia	Boys	2006	16	18.97
Bolivia	Boys	2007	16	19.21
Bolivia	Boys	2008	16	19.45
Bolivia	Boys	2009	16	19.70
Bolivia	Boys	2010	16	19.95
Bolivia	Boys	2011	16	20.21
Bolivia	Boys	2012	16	20.48
Bolivia	Boys	2013	16	20.75
Bolivia	Boys	2014	16	21.03
Bolivia	Boys	2015	16	21.31
Bolivia	Boys	2016	16	21.61
Bolivia	Boys	1975	17	15.75
Bolivia	Boys	1976	17	15.83
Bolivia	Boys	1977	17	15.93
Bolivia	Boys	1978	17	16.03
Bolivia	Boys	1979	17	16.14
Bolivia	Boys	1980	17	16.25
Bolivia	Boys	1981	17	16.38
Bolivia	Boys	1982	17	16.52
Bolivia	Boys	1983	17	16.66
Bolivia	Boys	1984	17	16.82
Bolivia	Boys	1985	17	16.98
Bolivia	Boys	1986	17	17.15
Bolivia	Boys	1987	17	17.33
Bolivia	Boys	1988	17	17.52
Bolivia	Boys	1989	17	17.72
Bolivia	Boys	1990	17	17.92
Bolivia	Boys	1991	17	18.13
Bolivia	Boys	1992	17	18.33
Bolivia	Boys	1993	17	18.55
Bolivia	Boys	1994	17	18.76
Bolivia	Boys	1995	17	18.98
Bolivia	Boys	1996	17	19.19
Bolivia	Boys	1997	17	19.41
Bolivia	Boys	1998	17	19.62
Bolivia	Boys	1999	17	19.83
Bolivia	Boys	2000	17	20.05
Bolivia	Boys	2001	17	20.26
Bolivia	Boys	2002	17	20.48
Bolivia	Boys	2003	17	20.71
Bolivia	Boys	2004	17	20.93
Bolivia	Boys	2005	17	21.16
Bolivia	Boys	2006	17	21.40
Bolivia	Boys	2007	17	21.64

Bolivia	Boys	2008	17	21.89
Bolivia	Boys	2009	17	22.14
Bolivia	Boys	2010	17	22.40
Bolivia	Boys	2011	17	22.66
Bolivia	Boys	2012	17	22.94
Bolivia	Boys	2013	17	23.21
Bolivia	Boys	2014	17	23.50
Bolivia	Boys	2015	17	23.79
Bolivia	Boys	2016	17	24.09
Bolivia	Boys	1975	18	18.07
Bolivia	Boys	1976	18	18.16
Bolivia	Boys	1977	18	18.26
Bolivia	Boys	1978	18	18.37
Bolivia	Boys	1979	18	18.49
Bolivia	Boys	1980	18	18.61
Bolivia	Boys	1981	18	18.75
Bolivia	Boys	1982	18	18.89
Bolivia	Boys	1983	18	19.04
Bolivia	Boys	1984	18	19.20
Bolivia	Boys	1985	18	19.37
Bolivia	Boys	1986	18	19.55
Bolivia	Boys	1987	18	19.74
Bolivia	Boys	1988	18	19.93
Bolivia	Boys	1989	18	20.14
Bolivia	Boys	1990	18	20.35
Bolivia	Boys	1991	18	20.56
Bolivia	Boys	1992	18	20.77
Bolivia	Boys	1993	18	20.99
Bolivia	Boys	1994	18	21.21
Bolivia	Boys	1995	18	21.44
Bolivia	Boys	1996	18	21.66
Bolivia	Boys	1997	18	21.88
Bolivia	Boys	1998	18	22.10
Bolivia	Boys	1999	18	22.32
Bolivia	Boys	2000	18	22.54
Bolivia	Boys	2001	18	22.76
Bolivia	Boys	2002	18	22.99
Bolivia	Boys	2003	18	23.22
Bolivia	Boys	2004	18	23.45
Bolivia	Boys	2005	18	23.69
Bolivia	Boys	2006	18	23.93
Bolivia	Boys	2007	18	24.18
Bolivia	Boys	2008	18	24.43
Bolivia	Boys	2009	18	24.70
Bolivia	Boys	2010	18	24.96
Bolivia	Boys	2011	18	25.23
Bolivia	Boys	2012	18	25.51
Bolivia	Boys	2013	18	25.80
Bolivia	Boys	2014	18	26.09
Bolivia	Boys	2015	18	26.39
Bolivia	Boys	2016	18	26.70
Bolivia	Boys	1975	Average	11.54

Bolivia	Boys	1976	Average	11.61
Bolivia	Boys	1977	Average	11.70
Bolivia	Boys	1978	Average	11.79
Bolivia	Boys	1979	Average	11.88
Bolivia	Boys	1980	Average	11.99
Bolivia	Boys	1981	Average	12.10
Bolivia	Boys	1982	Average	12.23
Bolivia	Boys	1983	Average	12.36
Bolivia	Boys	1984	Average	12.50
Bolivia	Boys	1985	Average	12.65
Bolivia	Boys	1986	Average	12.81
Bolivia	Boys	1987	Average	12.98
Bolivia	Boys	1988	Average	13.15
Bolivia	Boys	1989	Average	13.33
Bolivia	Boys	1990	Average	13.52
Bolivia	Boys	1991	Average	13.71
Bolivia	Boys	1992	Average	13.90
Bolivia	Boys	1993	Average	14.09
Bolivia	Boys	1994	Average	14.29
Bolivia	Boys	1995	Average	14.49
Bolivia	Boys	1996	Average	14.69
Bolivia	Boys	1997	Average	14.88
Bolivia	Boys	1998	Average	15.08
Bolivia	Boys	1999	Average	15.27
Bolivia	Boys	2000	Average	15.47
Bolivia	Boys	2001	Average	15.67
Bolivia	Boys	2002	Average	15.87
Bolivia	Boys	2003	Average	16.07
Bolivia	Boys	2004	Average	16.28
Bolivia	Boys	2005	Average	16.49
Bolivia	Boys	2006	Average	16.70
Bolivia	Boys	2007	Average	16.92
Bolivia	Boys	2008	Average	17.14
Bolivia	Boys	2009	Average	17.37
Bolivia	Boys	2010	Average	17.60
Bolivia	Boys	2011	Average	17.84
Bolivia	Boys	2012	Average	18.08
Bolivia	Boys	2013	Average	18.33
Bolivia	Boys	2014	Average	18.58
Bolivia	Boys	2015	Average	18.84
Bolivia	Boys	2016	Average	19.10
Bolivia	Girls	1975	12	6.57
Bolivia	Girls	1976	12	6.63
Bolivia	Girls	1977	12	6.71
Bolivia	Girls	1978	12	6.79
Bolivia	Girls	1979	12	6.87
Bolivia	Girls	1980	12	6.96
Bolivia	Girls	1981	12	7.06
Bolivia	Girls	1982	12	7.16
Bolivia	Girls	1983	12	7.27
Bolivia	Girls	1984	12	7.39
Bolivia	Girls	1985	12	7.51

Bolivia	Girls	1986	12	7.63
Bolivia	Girls	1987	12	7.76
Bolivia	Girls	1988	12	7.89
Bolivia	Girls	1989	12	8.02
Bolivia	Girls	1990	12	8.15
Bolivia	Girls	1991	12	8.29
Bolivia	Girls	1992	12	8.42
Bolivia	Girls	1993	12	8.56
Bolivia	Girls	1994	12	8.69
Bolivia	Girls	1995	12	8.82
Bolivia	Girls	1996	12	8.95
Bolivia	Girls	1997	12	9.08
Bolivia	Girls	1998	12	9.20
Bolivia	Girls	1999	12	9.32
Bolivia	Girls	2000	12	9.44
Bolivia	Girls	2001	12	9.56
Bolivia	Girls	2002	12	9.68
Bolivia	Girls	2003	12	9.79
Bolivia	Girls	2004	12	9.91
Bolivia	Girls	2005	12	10.02
Bolivia	Girls	2006	12	10.14
Bolivia	Girls	2007	12	10.25
Bolivia	Girls	2008	12	10.36
Bolivia	Girls	2009	12	10.48
Bolivia	Girls	2010	12	10.59
Bolivia	Girls	2011	12	10.71
Bolivia	Girls	2012	12	10.83
Bolivia	Girls	2013	12	10.95
Bolivia	Girls	2014	12	11.06
Bolivia	Girls	2015	12	11.18
Bolivia	Girls	2016	12	11.31
Bolivia	Girls	1975	13	8.13
Bolivia	Girls	1976	13	8.21
Bolivia	Girls	1977	13	8.29
Bolivia	Girls	1978	13	8.38
Bolivia	Girls	1979	13	8.48
Bolivia	Girls	1980	13	8.59
Bolivia	Girls	1981	13	8.70
Bolivia	Girls	1982	13	8.82
Bolivia	Girls	1983	13	8.95
Bolivia	Girls	1984	13	9.08
Bolivia	Girls	1985	13	9.22
Bolivia	Girls	1986	13	9.36
Bolivia	Girls	1987	13	9.50
Bolivia	Girls	1988	13	9.65
Bolivia	Girls	1989	13	9.80
Bolivia	Girls	1990	13	9.96
Bolivia	Girls	1991	13	10.11
Bolivia	Girls	1992	13	10.27
Bolivia	Girls	1993	13	10.42
Bolivia	Girls	1994	13	10.57
Bolivia	Girls	1995	13	10.72

Bolivia	Girls	1996	13	10.87
Bolivia	Girls	1997	13	11.02
Bolivia	Girls	1998	13	11.16
Bolivia	Girls	1999	13	11.30
Bolivia	Girls	2000	13	11.43
Bolivia	Girls	2001	13	11.57
Bolivia	Girls	2002	13	11.70
Bolivia	Girls	2003	13	11.83
Bolivia	Girls	2004	13	11.96
Bolivia	Girls	2005	13	12.09
Bolivia	Girls	2006	13	12.22
Bolivia	Girls	2007	13	12.36
Bolivia	Girls	2008	13	12.49
Bolivia	Girls	2009	13	12.62
Bolivia	Girls	2010	13	12.75
Bolivia	Girls	2011	13	12.88
Bolivia	Girls	2012	13	13.02
Bolivia	Girls	2013	13	13.15
Bolivia	Girls	2014	13	13.29
Bolivia	Girls	2015	13	13.43
Bolivia	Girls	2016	13	13.57
Bolivia	Girls	1975	14	9.77
Bolivia	Girls	1976	14	9.85
Bolivia	Girls	1977	14	9.95
Bolivia	Girls	1978	14	10.05
Bolivia	Girls	1979	14	10.16
Bolivia	Girls	1980	14	10.27
Bolivia	Girls	1981	14	10.40
Bolivia	Girls	1982	14	10.53
Bolivia	Girls	1983	14	10.67
Bolivia	Girls	1984	14	10.81
Bolivia	Girls	1985	14	10.96
Bolivia	Girls	1986	14	11.12
Bolivia	Girls	1987	14	11.27
Bolivia	Girls	1988	14	11.44
Bolivia	Girls	1989	14	11.60
Bolivia	Girls	1990	14	11.77
Bolivia	Girls	1991	14	11.94
Bolivia	Girls	1992	14	12.11
Bolivia	Girls	1993	14	12.27
Bolivia	Girls	1994	14	12.44
Bolivia	Girls	1995	14	12.60
Bolivia	Girls	1996	14	12.76
Bolivia	Girls	1997	14	12.92
Bolivia	Girls	1998	14	13.07
Bolivia	Girls	1999	14	13.22
Bolivia	Girls	2000	14	13.37
Bolivia	Girls	2001	14	13.52
Bolivia	Girls	2002	14	13.66
Bolivia	Girls	2003	14	13.81
Bolivia	Girls	2004	14	13.95
Bolivia	Girls	2005	14	14.09

Bolivia	Girls	2006	14	14.23
Bolivia	Girls	2007	14	14.37
Bolivia	Girls	2008	14	14.52
Bolivia	Girls	2009	14	14.66
Bolivia	Girls	2010	14	14.80
Bolivia	Girls	2011	14	14.95
Bolivia	Girls	2012	14	15.10
Bolivia	Girls	2013	14	15.24
Bolivia	Girls	2014	14	15.39
Bolivia	Girls	2015	14	15.55
Bolivia	Girls	2016	14	15.70
Bolivia	Girls	1975	15	11.26
Bolivia	Girls	1976	15	11.35
Bolivia	Girls	1977	15	11.45
Bolivia	Girls	1978	15	11.56
Bolivia	Girls	1979	15	11.67
Bolivia	Girls	1980	15	11.80
Bolivia	Girls	1981	15	11.93
Bolivia	Girls	1982	15	12.06
Bolivia	Girls	1983	15	12.21
Bolivia	Girls	1984	15	12.36
Bolivia	Girls	1985	15	12.52
Bolivia	Girls	1986	15	12.68
Bolivia	Girls	1987	15	12.85
Bolivia	Girls	1988	15	13.02
Bolivia	Girls	1989	15	13.19
Bolivia	Girls	1990	15	13.37
Bolivia	Girls	1991	15	13.54
Bolivia	Girls	1992	15	13.72
Bolivia	Girls	1993	15	13.89
Bolivia	Girls	1994	15	14.07
Bolivia	Girls	1995	15	14.24
Bolivia	Girls	1996	15	14.41
Bolivia	Girls	1997	15	14.57
Bolivia	Girls	1998	15	14.73
Bolivia	Girls	1999	15	14.89
Bolivia	Girls	2000	15	15.04
Bolivia	Girls	2001	15	15.20
Bolivia	Girls	2002	15	15.35
Bolivia	Girls	2003	15	15.50
Bolivia	Girls	2004	15	15.65
Bolivia	Girls	2005	15	15.80
Bolivia	Girls	2006	15	15.94
Bolivia	Girls	2007	15	16.09
Bolivia	Girls	2008	15	16.24
Bolivia	Girls	2009	15	16.39
Bolivia	Girls	2010	15	16.54
Bolivia	Girls	2011	15	16.69
Bolivia	Girls	2012	15	16.85
Bolivia	Girls	2013	15	17.00
Bolivia	Girls	2014	15	17.16
Bolivia	Girls	2015	15	17.32

Bolivia	Girls	2016	15	17.49
Bolivia	Girls	1975	16	12.46
Bolivia	Girls	1976	16	12.55
Bolivia	Girls	1977	16	12.65
Bolivia	Girls	1978	16	12.76
Bolivia	Girls	1979	16	12.88
Bolivia	Girls	1980	16	13.01
Bolivia	Girls	1981	16	13.14
Bolivia	Girls	1982	16	13.28
Bolivia	Girls	1983	16	13.43
Bolivia	Girls	1984	16	13.59
Bolivia	Girls	1985	16	13.75
Bolivia	Girls	1986	16	13.92
Bolivia	Girls	1987	16	14.09
Bolivia	Girls	1988	16	14.27
Bolivia	Girls	1989	16	14.45
Bolivia	Girls	1990	16	14.63
Bolivia	Girls	1991	16	14.81
Bolivia	Girls	1992	16	14.99
Bolivia	Girls	1993	16	15.17
Bolivia	Girls	1994	16	15.35
Bolivia	Girls	1995	16	15.53
Bolivia	Girls	1996	16	15.70
Bolivia	Girls	1997	16	15.87
Bolivia	Girls	1998	16	16.04
Bolivia	Girls	1999	16	16.20
Bolivia	Girls	2000	16	16.36
Bolivia	Girls	2001	16	16.52
Bolivia	Girls	2002	16	16.67
Bolivia	Girls	2003	16	16.83
Bolivia	Girls	2004	16	16.98
Bolivia	Girls	2005	16	17.14
Bolivia	Girls	2006	16	17.29
Bolivia	Girls	2007	16	17.44
Bolivia	Girls	2008	16	17.60
Bolivia	Girls	2009	16	17.75
Bolivia	Girls	2010	16	17.91
Bolivia	Girls	2011	16	18.07
Bolivia	Girls	2012	16	18.22
Bolivia	Girls	2013	16	18.39
Bolivia	Girls	2014	16	18.55
Bolivia	Girls	2015	16	18.72
Bolivia	Girls	2016	16	18.89
Bolivia	Girls	1975	17	13.34
Bolivia	Girls	1976	17	13.43
Bolivia	Girls	1977	17	13.54
Bolivia	Girls	1978	17	13.65
Bolivia	Girls	1979	17	13.78
Bolivia	Girls	1980	17	13.91
Bolivia	Girls	1981	17	14.04
Bolivia	Girls	1982	17	14.19
Bolivia	Girls	1983	17	14.35

Bolivia	Girls	1984	17	14.51
Bolivia	Girls	1985	17	14.68
Bolivia	Girls	1986	17	14.85
Bolivia	Girls	1987	17	15.03
Bolivia	Girls	1988	17	15.22
Bolivia	Girls	1989	17	15.40
Bolivia	Girls	1990	17	15.59
Bolivia	Girls	1991	17	15.78
Bolivia	Girls	1992	17	15.97
Bolivia	Girls	1993	17	16.16
Bolivia	Girls	1994	17	16.35
Bolivia	Girls	1995	17	16.54
Bolivia	Girls	1996	17	16.72
Bolivia	Girls	1997	17	16.90
Bolivia	Girls	1998	17	17.07
Bolivia	Girls	1999	17	17.24
Bolivia	Girls	2000	17	17.41
Bolivia	Girls	2001	17	17.57
Bolivia	Girls	2002	17	17.73
Bolivia	Girls	2003	17	17.90
Bolivia	Girls	2004	17	18.06
Bolivia	Girls	2005	17	18.22
Bolivia	Girls	2006	17	18.38
Bolivia	Girls	2007	17	18.54
Bolivia	Girls	2008	17	18.70
Bolivia	Girls	2009	17	18.86
Bolivia	Girls	2010	17	19.03
Bolivia	Girls	2011	17	19.19
Bolivia	Girls	2012	17	19.36
Bolivia	Girls	2013	17	19.53
Bolivia	Girls	2014	17	19.70
Bolivia	Girls	2015	17	19.88
Bolivia	Girls	2016	17	20.05
Bolivia	Girls	1975	18	14.03
Bolivia	Girls	1976	18	14.13
Bolivia	Girls	1977	18	14.25
Bolivia	Girls	1978	18	14.37
Bolivia	Girls	1979	18	14.50
Bolivia	Girls	1980	18	14.64
Bolivia	Girls	1981	18	14.79
Bolivia	Girls	1982	18	14.95
Bolivia	Girls	1983	18	15.12
Bolivia	Girls	1984	18	15.29
Bolivia	Girls	1985	18	15.47
Bolivia	Girls	1986	18	15.66
Bolivia	Girls	1987	18	15.86
Bolivia	Girls	1988	18	16.05
Bolivia	Girls	1989	18	16.26
Bolivia	Girls	1990	18	16.46
Bolivia	Girls	1991	18	16.67
Bolivia	Girls	1992	18	16.87
Bolivia	Girls	1993	18	17.08

Bolivia	Girls	1994	18	17.28
Bolivia	Girls	1995	18	17.48
Bolivia	Girls	1996	18	17.68
Bolivia	Girls	1997	18	17.87
Bolivia	Girls	1998	18	18.06
Bolivia	Girls	1999	18	18.24
Bolivia	Girls	2000	18	18.42
Bolivia	Girls	2001	18	18.60
Bolivia	Girls	2002	18	18.78
Bolivia	Girls	2003	18	18.95
Bolivia	Girls	2004	18	19.13
Bolivia	Girls	2005	18	19.30
Bolivia	Girls	2006	18	19.48
Bolivia	Girls	2007	18	19.65
Bolivia	Girls	2008	18	19.83
Bolivia	Girls	2009	18	20.00
Bolivia	Girls	2010	18	20.18
Bolivia	Girls	2011	18	20.36
Bolivia	Girls	2012	18	20.54
Bolivia	Girls	2013	18	20.72
Bolivia	Girls	2014	18	20.90
Bolivia	Girls	2015	18	21.09
Bolivia	Girls	2016	18	21.28
Bolivia	Girls	1975	Average	10.79
Bolivia	Girls	1976	Average	10.88
Bolivia	Girls	1977	Average	10.98
Bolivia	Girls	1978	Average	11.08
Bolivia	Girls	1979	Average	11.19
Bolivia	Girls	1980	Average	11.31
Bolivia	Girls	1981	Average	11.44
Bolivia	Girls	1982	Average	11.57
Bolivia	Girls	1983	Average	11.71
Bolivia	Girls	1984	Average	11.86
Bolivia	Girls	1985	Average	12.02
Bolivia	Girls	1986	Average	12.17
Bolivia	Girls	1987	Average	12.34
Bolivia	Girls	1988	Average	12.50
Bolivia	Girls	1989	Average	12.67
Bolivia	Girls	1990	Average	12.85
Bolivia	Girls	1991	Average	13.02
Bolivia	Girls	1992	Average	13.19
Bolivia	Girls	1993	Average	13.37
Bolivia	Girls	1994	Average	13.54
Bolivia	Girls	1995	Average	13.71
Bolivia	Girls	1996	Average	13.87
Bolivia	Girls	1997	Average	14.03
Bolivia	Girls	1998	Average	14.19
Bolivia	Girls	1999	Average	14.34
Bolivia	Girls	2000	Average	14.50
Bolivia	Girls	2001	Average	14.65
Bolivia	Girls	2002	Average	14.80
Bolivia	Girls	2003	Average	14.94

Bolivia	Girls	2004	Average	15.09
Bolivia	Girls	2005	Average	15.24
Bolivia	Girls	2006	Average	15.38
Bolivia	Girls	2007	Average	15.53
Bolivia	Girls	2008	Average	15.68
Bolivia	Girls	2009	Average	15.82
Bolivia	Girls	2010	Average	15.97
Bolivia	Girls	2011	Average	16.12
Bolivia	Girls	2012	Average	16.27
Bolivia	Girls	2013	Average	16.43
Bolivia	Girls	2014	Average	16.58
Bolivia	Girls	2015	Average	16.74
Bolivia	Girls	2016	Average	16.90
Bosnia and Herzegovina	Boys	1975	12	5.53
Bosnia and Herzegovina	Boys	1976	12	5.58
Bosnia and Herzegovina	Boys	1977	12	5.65
Bosnia and Herzegovina	Boys	1978	12	5.71
Bosnia and Herzegovina	Boys	1979	12	5.78
Bosnia and Herzegovina	Boys	1980	12	5.86
Bosnia and Herzegovina	Boys	1981	12	5.93
Bosnia and Herzegovina	Boys	1982	12	6.01
Bosnia and Herzegovina	Boys	1983	12	6.09
Bosnia and Herzegovina	Boys	1984	12	6.17
Bosnia and Herzegovina	Boys	1985	12	6.26
Bosnia and Herzegovina	Boys	1986	12	6.34
Bosnia and Herzegovina	Boys	1987	12	6.43
Bosnia and Herzegovina	Boys	1988	12	6.53
Bosnia and Herzegovina	Boys	1989	12	6.62
Bosnia and Herzegovina	Boys	1990	12	6.72
Bosnia and Herzegovina	Boys	1991	12	6.82
Bosnia and Herzegovina	Boys	1992	12	6.93
Bosnia and Herzegovina	Boys	1993	12	7.03
Bosnia and Herzegovina	Boys	1994	12	7.14
Bosnia and Herzegovina	Boys	1995	12	7.26
Bosnia and Herzegovina	Boys	1996	12	7.37
Bosnia and Herzegovina	Boys	1997	12	7.49
Bosnia and Herzegovina	Boys	1998	12	7.62
Bosnia and Herzegovina	Boys	1999	12	7.75
Bosnia and Herzegovina	Boys	2000	12	7.88
Bosnia and Herzegovina	Boys	2001	12	8.03
Bosnia and Herzegovina	Boys	2002	12	8.17
Bosnia and Herzegovina	Boys	2003	12	8.33
Bosnia and Herzegovina	Boys	2004	12	8.49
Bosnia and Herzegovina	Boys	2005	12	8.66
Bosnia and Herzegovina	Boys	2006	12	8.84
Bosnia and Herzegovina	Boys	2007	12	9.02
Bosnia and Herzegovina	Boys	2008	12	9.22
Bosnia and Herzegovina	Boys	2009	12	9.42
Bosnia and Herzegovina	Boys	2010	12	9.62
Bosnia and Herzegovina	Boys	2011	12	9.84
Bosnia and Herzegovina	Boys	2012	12	10.06
Bosnia and Herzegovina	Boys	2013	12	10.29

Bosnia and Herzegovina	Boys	2014	12	10.53
Bosnia and Herzegovina	Boys	2015	12	10.78
Bosnia and Herzegovina	Boys	2016	12	11.03
Bosnia and Herzegovina	Boys	1975	13	6.90
Bosnia and Herzegovina	Boys	1976	13	6.97
Bosnia and Herzegovina	Boys	1977	13	7.04
Bosnia and Herzegovina	Boys	1978	13	7.12
Bosnia and Herzegovina	Boys	1979	13	7.21
Bosnia and Herzegovina	Boys	1980	13	7.29
Bosnia and Herzegovina	Boys	1981	13	7.38
Bosnia and Herzegovina	Boys	1982	13	7.47
Bosnia and Herzegovina	Boys	1983	13	7.57
Bosnia and Herzegovina	Boys	1984	13	7.67
Bosnia and Herzegovina	Boys	1985	13	7.77
Bosnia and Herzegovina	Boys	1986	13	7.87
Bosnia and Herzegovina	Boys	1987	13	7.97
Bosnia and Herzegovina	Boys	1988	13	8.08
Bosnia and Herzegovina	Boys	1989	13	8.20
Bosnia and Herzegovina	Boys	1990	13	8.31
Bosnia and Herzegovina	Boys	1991	13	8.43
Bosnia and Herzegovina	Boys	1992	13	8.55
Bosnia and Herzegovina	Boys	1993	13	8.68
Bosnia and Herzegovina	Boys	1994	13	8.81
Bosnia and Herzegovina	Boys	1995	13	8.94
Bosnia and Herzegovina	Boys	1996	13	9.07
Bosnia and Herzegovina	Boys	1997	13	9.21
Bosnia and Herzegovina	Boys	1998	13	9.36
Bosnia and Herzegovina	Boys	1999	13	9.51
Bosnia and Herzegovina	Boys	2000	13	9.67
Bosnia and Herzegovina	Boys	2001	13	9.84
Bosnia and Herzegovina	Boys	2002	13	10.02
Bosnia and Herzegovina	Boys	2003	13	10.20
Bosnia and Herzegovina	Boys	2004	13	10.39
Bosnia and Herzegovina	Boys	2005	13	10.59
Bosnia and Herzegovina	Boys	2006	13	10.80
Bosnia and Herzegovina	Boys	2007	13	11.02
Bosnia and Herzegovina	Boys	2008	13	11.25
Bosnia and Herzegovina	Boys	2009	13	11.49
Bosnia and Herzegovina	Boys	2010	13	11.74
Bosnia and Herzegovina	Boys	2011	13	12.00
Bosnia and Herzegovina	Boys	2012	13	12.27
Bosnia and Herzegovina	Boys	2013	13	12.55
Bosnia and Herzegovina	Boys	2014	13	12.84
Bosnia and Herzegovina	Boys	2015	13	13.14
Bosnia and Herzegovina	Boys	2016	13	13.45
Bosnia and Herzegovina	Boys	1975	14	8.64
Bosnia and Herzegovina	Boys	1976	14	8.72
Bosnia and Herzegovina	Boys	1977	14	8.80
Bosnia and Herzegovina	Boys	1978	14	8.89
Bosnia and Herzegovina	Boys	1979	14	8.98
Bosnia and Herzegovina	Boys	1980	14	9.08
Bosnia and Herzegovina	Boys	1981	14	9.19

Bosnia and Herzegovina	Boys	1982	14	9.29
Bosnia and Herzegovina	Boys	1983	14	9.39
Bosnia and Herzegovina	Boys	1984	14	9.50
Bosnia and Herzegovina	Boys	1985	14	9.61
Bosnia and Herzegovina	Boys	1986	14	9.73
Bosnia and Herzegovina	Boys	1987	14	9.85
Bosnia and Herzegovina	Boys	1988	14	9.97
Bosnia and Herzegovina	Boys	1989	14	10.09
Bosnia and Herzegovina	Boys	1990	14	10.22
Bosnia and Herzegovina	Boys	1991	14	10.36
Bosnia and Herzegovina	Boys	1992	14	10.49
Bosnia and Herzegovina	Boys	1993	14	10.63
Bosnia and Herzegovina	Boys	1994	14	10.77
Bosnia and Herzegovina	Boys	1995	14	10.92
Bosnia and Herzegovina	Boys	1996	14	11.07
Bosnia and Herzegovina	Boys	1997	14	11.23
Bosnia and Herzegovina	Boys	1998	14	11.39
Bosnia and Herzegovina	Boys	1999	14	11.56
Bosnia and Herzegovina	Boys	2000	14	11.74
Bosnia and Herzegovina	Boys	2001	14	11.92
Bosnia and Herzegovina	Boys	2002	14	12.12
Bosnia and Herzegovina	Boys	2003	14	12.32
Bosnia and Herzegovina	Boys	2004	14	12.54
Bosnia and Herzegovina	Boys	2005	14	12.76
Bosnia and Herzegovina	Boys	2006	14	13.00
Bosnia and Herzegovina	Boys	2007	14	13.25
Bosnia and Herzegovina	Boys	2008	14	13.51
Bosnia and Herzegovina	Boys	2009	14	13.78
Bosnia and Herzegovina	Boys	2010	14	14.06
Bosnia and Herzegovina	Boys	2011	14	14.36
Bosnia and Herzegovina	Boys	2012	14	14.67
Bosnia and Herzegovina	Boys	2013	14	14.99
Bosnia and Herzegovina	Boys	2014	14	15.32
Bosnia and Herzegovina	Boys	2015	14	15.66
Bosnia and Herzegovina	Boys	2016	14	16.02
Bosnia and Herzegovina	Boys	1975	15	10.66
Bosnia and Herzegovina	Boys	1976	15	10.75
Bosnia and Herzegovina	Boys	1977	15	10.84
Bosnia and Herzegovina	Boys	1978	15	10.94
Bosnia and Herzegovina	Boys	1979	15	11.04
Bosnia and Herzegovina	Boys	1980	15	11.15
Bosnia and Herzegovina	Boys	1981	15	11.26
Bosnia and Herzegovina	Boys	1982	15	11.37
Bosnia and Herzegovina	Boys	1983	15	11.48
Bosnia and Herzegovina	Boys	1984	15	11.60
Bosnia and Herzegovina	Boys	1985	15	11.72
Bosnia and Herzegovina	Boys	1986	15	11.84
Bosnia and Herzegovina	Boys	1987	15	11.96
Bosnia and Herzegovina	Boys	1988	15	12.09
Bosnia and Herzegovina	Boys	1989	15	12.23
Bosnia and Herzegovina	Boys	1990	15	12.36
Bosnia and Herzegovina	Boys	1991	15	12.50

Bosnia and Herzegovina	Boys	1992	15	12.65
Bosnia and Herzegovina	Boys	1993	15	12.79
Bosnia and Herzegovina	Boys	1994	15	12.94
Bosnia and Herzegovina	Boys	1995	15	13.10
Bosnia and Herzegovina	Boys	1996	15	13.26
Bosnia and Herzegovina	Boys	1997	15	13.42
Bosnia and Herzegovina	Boys	1998	15	13.60
Bosnia and Herzegovina	Boys	1999	15	13.78
Bosnia and Herzegovina	Boys	2000	15	13.96
Bosnia and Herzegovina	Boys	2001	15	14.16
Bosnia and Herzegovina	Boys	2002	15	14.37
Bosnia and Herzegovina	Boys	2003	15	14.59
Bosnia and Herzegovina	Boys	2004	15	14.82
Bosnia and Herzegovina	Boys	2005	15	15.06
Bosnia and Herzegovina	Boys	2006	15	15.31
Bosnia and Herzegovina	Boys	2007	15	15.58
Bosnia and Herzegovina	Boys	2008	15	15.85
Bosnia and Herzegovina	Boys	2009	15	16.15
Bosnia and Herzegovina	Boys	2010	15	16.45
Bosnia and Herzegovina	Boys	2011	15	16.77
Bosnia and Herzegovina	Boys	2012	15	17.10
Bosnia and Herzegovina	Boys	2013	15	17.45
Bosnia and Herzegovina	Boys	2014	15	17.81
Bosnia and Herzegovina	Boys	2015	15	18.18
Bosnia and Herzegovina	Boys	2016	15	18.57
Bosnia and Herzegovina	Boys	1975	16	12.88
Bosnia and Herzegovina	Boys	1976	16	12.97
Bosnia and Herzegovina	Boys	1977	16	13.07
Bosnia and Herzegovina	Boys	1978	16	13.17
Bosnia and Herzegovina	Boys	1979	16	13.28
Bosnia and Herzegovina	Boys	1980	16	13.39
Bosnia and Herzegovina	Boys	1981	16	13.50
Bosnia and Herzegovina	Boys	1982	16	13.62
Bosnia and Herzegovina	Boys	1983	16	13.74
Bosnia and Herzegovina	Boys	1984	16	13.86
Bosnia and Herzegovina	Boys	1985	16	13.98
Bosnia and Herzegovina	Boys	1986	16	14.11
Bosnia and Herzegovina	Boys	1987	16	14.24
Bosnia and Herzegovina	Boys	1988	16	14.37
Bosnia and Herzegovina	Boys	1989	16	14.51
Bosnia and Herzegovina	Boys	1990	16	14.65
Bosnia and Herzegovina	Boys	1991	16	14.80
Bosnia and Herzegovina	Boys	1992	16	14.94
Bosnia and Herzegovina	Boys	1993	16	15.09
Bosnia and Herzegovina	Boys	1994	16	15.25
Bosnia and Herzegovina	Boys	1995	16	15.41
Bosnia and Herzegovina	Boys	1996	16	15.57
Bosnia and Herzegovina	Boys	1997	16	15.74
Bosnia and Herzegovina	Boys	1998	16	15.92
Bosnia and Herzegovina	Boys	1999	16	16.10
Bosnia and Herzegovina	Boys	2000	16	16.30
Bosnia and Herzegovina	Boys	2001	16	16.50

Bosnia and Herzegovina	Boys	2002	16	16.71
Bosnia and Herzegovina	Boys	2003	16	16.94
Bosnia and Herzegovina	Boys	2004	16	17.17
Bosnia and Herzegovina	Boys	2005	16	17.42
Bosnia and Herzegovina	Boys	2006	16	17.68
Bosnia and Herzegovina	Boys	2007	16	17.96
Bosnia and Herzegovina	Boys	2008	16	18.25
Bosnia and Herzegovina	Boys	2009	16	18.55
Bosnia and Herzegovina	Boys	2010	16	18.87
Bosnia and Herzegovina	Boys	2011	16	19.20
Bosnia and Herzegovina	Boys	2012	16	19.54
Bosnia and Herzegovina	Boys	2013	16	19.90
Bosnia and Herzegovina	Boys	2014	16	20.28
Bosnia and Herzegovina	Boys	2015	16	20.67
Bosnia and Herzegovina	Boys	2016	16	21.08
Bosnia and Herzegovina	Boys	1975	17	15.18
Bosnia and Herzegovina	Boys	1976	17	15.28
Bosnia and Herzegovina	Boys	1977	17	15.38
Bosnia and Herzegovina	Boys	1978	17	15.49
Bosnia and Herzegovina	Boys	1979	17	15.60
Bosnia and Herzegovina	Boys	1980	17	15.72
Bosnia and Herzegovina	Boys	1981	17	15.83
Bosnia and Herzegovina	Boys	1982	17	15.95
Bosnia and Herzegovina	Boys	1983	17	16.08
Bosnia and Herzegovina	Boys	1984	17	16.20
Bosnia and Herzegovina	Boys	1985	17	16.33
Bosnia and Herzegovina	Boys	1986	17	16.46
Bosnia and Herzegovina	Boys	1987	17	16.59
Bosnia and Herzegovina	Boys	1988	17	16.73
Bosnia and Herzegovina	Boys	1989	17	16.87
Bosnia and Herzegovina	Boys	1990	17	17.01
Bosnia and Herzegovina	Boys	1991	17	17.16
Bosnia and Herzegovina	Boys	1992	17	17.31
Bosnia and Herzegovina	Boys	1993	17	17.47
Bosnia and Herzegovina	Boys	1994	17	17.62
Bosnia and Herzegovina	Boys	1995	17	17.79
Bosnia and Herzegovina	Boys	1996	17	17.95
Bosnia and Herzegovina	Boys	1997	17	18.13
Bosnia and Herzegovina	Boys	1998	17	18.31
Bosnia and Herzegovina	Boys	1999	17	18.49
Bosnia and Herzegovina	Boys	2000	17	18.69
Bosnia and Herzegovina	Boys	2001	17	18.90
Bosnia and Herzegovina	Boys	2002	17	19.12
Bosnia and Herzegovina	Boys	2003	17	19.35
Bosnia and Herzegovina	Boys	2004	17	19.59
Bosnia and Herzegovina	Boys	2005	17	19.84
Bosnia and Herzegovina	Boys	2006	17	20.11
Bosnia and Herzegovina	Boys	2007	17	20.39
Bosnia and Herzegovina	Boys	2008	17	20.68
Bosnia and Herzegovina	Boys	2009	17	20.99
Bosnia and Herzegovina	Boys	2010	17	21.32
Bosnia and Herzegovina	Boys	2011	17	21.65

Bosnia and Herzegovina	Boys	2012	17	22.01
Bosnia and Herzegovina	Boys	2013	17	22.38
Bosnia and Herzegovina	Boys	2014	17	22.77
Bosnia and Herzegovina	Boys	2015	17	23.17
Bosnia and Herzegovina	Boys	2016	17	23.59
Bosnia and Herzegovina	Boys	1975	18	17.47
Bosnia and Herzegovina	Boys	1976	18	17.58
Bosnia and Herzegovina	Boys	1977	18	17.69
Bosnia and Herzegovina	Boys	1978	18	17.81
Bosnia and Herzegovina	Boys	1979	18	17.93
Bosnia and Herzegovina	Boys	1980	18	18.05
Bosnia and Herzegovina	Boys	1981	18	18.18
Bosnia and Herzegovina	Boys	1982	18	18.30
Bosnia and Herzegovina	Boys	1983	18	18.43
Bosnia and Herzegovina	Boys	1984	18	18.56
Bosnia and Herzegovina	Boys	1985	18	18.70
Bosnia and Herzegovina	Boys	1986	18	18.83
Bosnia and Herzegovina	Boys	1987	18	18.97
Bosnia and Herzegovina	Boys	1988	18	19.11
Bosnia and Herzegovina	Boys	1989	18	19.26
Bosnia and Herzegovina	Boys	1990	18	19.41
Bosnia and Herzegovina	Boys	1991	18	19.57
Bosnia and Herzegovina	Boys	1992	18	19.72
Bosnia and Herzegovina	Boys	1993	18	19.88
Bosnia and Herzegovina	Boys	1994	18	20.05
Bosnia and Herzegovina	Boys	1995	18	20.21
Bosnia and Herzegovina	Boys	1996	18	20.39
Bosnia and Herzegovina	Boys	1997	18	20.57
Bosnia and Herzegovina	Boys	1998	18	20.76
Bosnia and Herzegovina	Boys	1999	18	20.95
Bosnia and Herzegovina	Boys	2000	18	21.15
Bosnia and Herzegovina	Boys	2001	18	21.37
Bosnia and Herzegovina	Boys	2002	18	21.60
Bosnia and Herzegovina	Boys	2003	18	21.83
Bosnia and Herzegovina	Boys	2004	18	22.08
Bosnia and Herzegovina	Boys	2005	18	22.34
Bosnia and Herzegovina	Boys	2006	18	22.62
Bosnia and Herzegovina	Boys	2007	18	22.91
Bosnia and Herzegovina	Boys	2008	18	23.22
Bosnia and Herzegovina	Boys	2009	18	23.54
Bosnia and Herzegovina	Boys	2010	18	23.87
Bosnia and Herzegovina	Boys	2011	18	24.22
Bosnia and Herzegovina	Boys	2012	18	24.59
Bosnia and Herzegovina	Boys	2013	18	24.97
Bosnia and Herzegovina	Boys	2014	18	25.37
Bosnia and Herzegovina	Boys	2015	18	25.78
Bosnia and Herzegovina	Boys	2016	18	26.21
Bosnia and Herzegovina	Boys	1975	Average	11.04
Bosnia and Herzegovina	Boys	1976	Average	11.12
Bosnia and Herzegovina	Boys	1977	Average	11.21
Bosnia and Herzegovina	Boys	1978	Average	11.30
Bosnia and Herzegovina	Boys	1979	Average	11.40

Bosnia and Herzegovina	Boys	1980	Average	11.51
Bosnia and Herzegovina	Boys	1981	Average	11.61
Bosnia and Herzegovina	Boys	1982	Average	11.72
Bosnia and Herzegovina	Boys	1983	Average	11.83
Bosnia and Herzegovina	Boys	1984	Average	11.94
Bosnia and Herzegovina	Boys	1985	Average	12.05
Bosnia and Herzegovina	Boys	1986	Average	12.17
Bosnia and Herzegovina	Boys	1987	Average	12.29
Bosnia and Herzegovina	Boys	1988	Average	12.41
Bosnia and Herzegovina	Boys	1989	Average	12.54
Bosnia and Herzegovina	Boys	1990	Average	12.67
Bosnia and Herzegovina	Boys	1991	Average	12.81
Bosnia and Herzegovina	Boys	1992	Average	12.94
Bosnia and Herzegovina	Boys	1993	Average	13.08
Bosnia and Herzegovina	Boys	1994	Average	13.23
Bosnia and Herzegovina	Boys	1995	Average	13.37
Bosnia and Herzegovina	Boys	1996	Average	13.53
Bosnia and Herzegovina	Boys	1997	Average	13.69
Bosnia and Herzegovina	Boys	1998	Average	13.85
Bosnia and Herzegovina	Boys	1999	Average	14.02
Bosnia and Herzegovina	Boys	2000	Average	14.20
Bosnia and Herzegovina	Boys	2001	Average	14.39
Bosnia and Herzegovina	Boys	2002	Average	14.59
Bosnia and Herzegovina	Boys	2003	Average	14.79
Bosnia and Herzegovina	Boys	2004	Average	15.01
Bosnia and Herzegovina	Boys	2005	Average	15.24
Bosnia and Herzegovina	Boys	2006	Average	15.48
Bosnia and Herzegovina	Boys	2007	Average	15.73
Bosnia and Herzegovina	Boys	2008	Average	16.00
Bosnia and Herzegovina	Boys	2009	Average	16.27
Bosnia and Herzegovina	Boys	2010	Average	16.56
Bosnia and Herzegovina	Boys	2011	Average	16.86
Bosnia and Herzegovina	Boys	2012	Average	17.18
Bosnia and Herzegovina	Boys	2013	Average	17.50
Bosnia and Herzegovina	Boys	2014	Average	17.85
Bosnia and Herzegovina	Boys	2015	Average	18.20
Bosnia and Herzegovina	Boys	2016	Average	18.56
Bosnia and Herzegovina	Girls	1975	12	5.24
Bosnia and Herzegovina	Girls	1976	12	5.29
Bosnia and Herzegovina	Girls	1977	12	5.33
Bosnia and Herzegovina	Girls	1978	12	5.38
Bosnia and Herzegovina	Girls	1979	12	5.43
Bosnia and Herzegovina	Girls	1980	12	5.48
Bosnia and Herzegovina	Girls	1981	12	5.53
Bosnia and Herzegovina	Girls	1982	12	5.58
Bosnia and Herzegovina	Girls	1983	12	5.64
Bosnia and Herzegovina	Girls	1984	12	5.69
Bosnia and Herzegovina	Girls	1985	12	5.75
Bosnia and Herzegovina	Girls	1986	12	5.80
Bosnia and Herzegovina	Girls	1987	12	5.86
Bosnia and Herzegovina	Girls	1988	12	5.92
Bosnia and Herzegovina	Girls	1989	12	5.98

Bosnia and Herzegovina	Girls	1990	12	6.04
Bosnia and Herzegovina	Girls	1991	12	6.10
Bosnia and Herzegovina	Girls	1992	12	6.16
Bosnia and Herzegovina	Girls	1993	12	6.23
Bosnia and Herzegovina	Girls	1994	12	6.29
Bosnia and Herzegovina	Girls	1995	12	6.36
Bosnia and Herzegovina	Girls	1996	12	6.43
Bosnia and Herzegovina	Girls	1997	12	6.50
Bosnia and Herzegovina	Girls	1998	12	6.57
Bosnia and Herzegovina	Girls	1999	12	6.65
Bosnia and Herzegovina	Girls	2000	12	6.73
Bosnia and Herzegovina	Girls	2001	12	6.81
Bosnia and Herzegovina	Girls	2002	12	6.90
Bosnia and Herzegovina	Girls	2003	12	7.00
Bosnia and Herzegovina	Girls	2004	12	7.09
Bosnia and Herzegovina	Girls	2005	12	7.19
Bosnia and Herzegovina	Girls	2006	12	7.30
Bosnia and Herzegovina	Girls	2007	12	7.41
Bosnia and Herzegovina	Girls	2008	12	7.52
Bosnia and Herzegovina	Girls	2009	12	7.64
Bosnia and Herzegovina	Girls	2010	12	7.76
Bosnia and Herzegovina	Girls	2011	12	7.89
Bosnia and Herzegovina	Girls	2012	12	8.02
Bosnia and Herzegovina	Girls	2013	12	8.16
Bosnia and Herzegovina	Girls	2014	12	8.30
Bosnia and Herzegovina	Girls	2015	12	8.46
Bosnia and Herzegovina	Girls	2016	12	8.61
Bosnia and Herzegovina	Girls	1975	13	6.57
Bosnia and Herzegovina	Girls	1976	13	6.62
Bosnia and Herzegovina	Girls	1977	13	6.67
Bosnia and Herzegovina	Girls	1978	13	6.73
Bosnia and Herzegovina	Girls	1979	13	6.79
Bosnia and Herzegovina	Girls	1980	13	6.85
Bosnia and Herzegovina	Girls	1981	13	6.91
Bosnia and Herzegovina	Girls	1982	13	6.97
Bosnia and Herzegovina	Girls	1983	13	7.03
Bosnia and Herzegovina	Girls	1984	13	7.10
Bosnia and Herzegovina	Girls	1985	13	7.16
Bosnia and Herzegovina	Girls	1986	13	7.22
Bosnia and Herzegovina	Girls	1987	13	7.29
Bosnia and Herzegovina	Girls	1988	13	7.36
Bosnia and Herzegovina	Girls	1989	13	7.42
Bosnia and Herzegovina	Girls	1990	13	7.50
Bosnia and Herzegovina	Girls	1991	13	7.57
Bosnia and Herzegovina	Girls	1992	13	7.64
Bosnia and Herzegovina	Girls	1993	13	7.71
Bosnia and Herzegovina	Girls	1994	13	7.79
Bosnia and Herzegovina	Girls	1995	13	7.86
Bosnia and Herzegovina	Girls	1996	13	7.94
Bosnia and Herzegovina	Girls	1997	13	8.02
Bosnia and Herzegovina	Girls	1998	13	8.11
Bosnia and Herzegovina	Girls	1999	13	8.19

Bosnia and Herzegovina	Girls	2000	13	8.29
Bosnia and Herzegovina	Girls	2001	13	8.38
Bosnia and Herzegovina	Girls	2002	13	8.48
Bosnia and Herzegovina	Girls	2003	13	8.59
Bosnia and Herzegovina	Girls	2004	13	8.70
Bosnia and Herzegovina	Girls	2005	13	8.81
Bosnia and Herzegovina	Girls	2006	13	8.93
Bosnia and Herzegovina	Girls	2007	13	9.05
Bosnia and Herzegovina	Girls	2008	13	9.18
Bosnia and Herzegovina	Girls	2009	13	9.32
Bosnia and Herzegovina	Girls	2010	13	9.45
Bosnia and Herzegovina	Girls	2011	13	9.60
Bosnia and Herzegovina	Girls	2012	13	9.75
Bosnia and Herzegovina	Girls	2013	13	9.91
Bosnia and Herzegovina	Girls	2014	13	10.07
Bosnia and Herzegovina	Girls	2015	13	10.25
Bosnia and Herzegovina	Girls	2016	13	10.42
Bosnia and Herzegovina	Girls	1975	14	8.02
Bosnia and Herzegovina	Girls	1976	14	8.08
Bosnia and Herzegovina	Girls	1977	14	8.14
Bosnia and Herzegovina	Girls	1978	14	8.20
Bosnia and Herzegovina	Girls	1979	14	8.27
Bosnia and Herzegovina	Girls	1980	14	8.33
Bosnia and Herzegovina	Girls	1981	14	8.40
Bosnia and Herzegovina	Girls	1982	14	8.47
Bosnia and Herzegovina	Girls	1983	14	8.54
Bosnia and Herzegovina	Girls	1984	14	8.61
Bosnia and Herzegovina	Girls	1985	14	8.68
Bosnia and Herzegovina	Girls	1986	14	8.75
Bosnia and Herzegovina	Girls	1987	14	8.82
Bosnia and Herzegovina	Girls	1988	14	8.89
Bosnia and Herzegovina	Girls	1989	14	8.97
Bosnia and Herzegovina	Girls	1990	14	9.05
Bosnia and Herzegovina	Girls	1991	14	9.12
Bosnia and Herzegovina	Girls	1992	14	9.20
Bosnia and Herzegovina	Girls	1993	14	9.28
Bosnia and Herzegovina	Girls	1994	14	9.36
Bosnia and Herzegovina	Girls	1995	14	9.45
Bosnia and Herzegovina	Girls	1996	14	9.53
Bosnia and Herzegovina	Girls	1997	14	9.62
Bosnia and Herzegovina	Girls	1998	14	9.71
Bosnia and Herzegovina	Girls	1999	14	9.81
Bosnia and Herzegovina	Girls	2000	14	9.90
Bosnia and Herzegovina	Girls	2001	14	10.01
Bosnia and Herzegovina	Girls	2002	14	10.12
Bosnia and Herzegovina	Girls	2003	14	10.23
Bosnia and Herzegovina	Girls	2004	14	10.35
Bosnia and Herzegovina	Girls	2005	14	10.47
Bosnia and Herzegovina	Girls	2006	14	10.60
Bosnia and Herzegovina	Girls	2007	14	10.73
Bosnia and Herzegovina	Girls	2008	14	10.87
Bosnia and Herzegovina	Girls	2009	14	11.02

Bosnia and Herzegovina	Girls	2010	14	11.17
Bosnia and Herzegovina	Girls	2011	14	11.32
Bosnia and Herzegovina	Girls	2012	14	11.49
Bosnia and Herzegovina	Girls	2013	14	11.66
Bosnia and Herzegovina	Girls	2014	14	11.84
Bosnia and Herzegovina	Girls	2015	14	12.02
Bosnia and Herzegovina	Girls	2016	14	12.21
Bosnia and Herzegovina	Girls	1975	15	9.39
Bosnia and Herzegovina	Girls	1976	15	9.45
Bosnia and Herzegovina	Girls	1977	15	9.52
Bosnia and Herzegovina	Girls	1978	15	9.58
Bosnia and Herzegovina	Girls	1979	15	9.65
Bosnia and Herzegovina	Girls	1980	15	9.72
Bosnia and Herzegovina	Girls	1981	15	9.80
Bosnia and Herzegovina	Girls	1982	15	9.87
Bosnia and Herzegovina	Girls	1983	15	9.94
Bosnia and Herzegovina	Girls	1984	15	10.01
Bosnia and Herzegovina	Girls	1985	15	10.09
Bosnia and Herzegovina	Girls	1986	15	10.16
Bosnia and Herzegovina	Girls	1987	15	10.24
Bosnia and Herzegovina	Girls	1988	15	10.32
Bosnia and Herzegovina	Girls	1989	15	10.39
Bosnia and Herzegovina	Girls	1990	15	10.48
Bosnia and Herzegovina	Girls	1991	15	10.56
Bosnia and Herzegovina	Girls	1992	15	10.64
Bosnia and Herzegovina	Girls	1993	15	10.72
Bosnia and Herzegovina	Girls	1994	15	10.81
Bosnia and Herzegovina	Girls	1995	15	10.89
Bosnia and Herzegovina	Girls	1996	15	10.98
Bosnia and Herzegovina	Girls	1997	15	11.08
Bosnia and Herzegovina	Girls	1998	15	11.17
Bosnia and Herzegovina	Girls	1999	15	11.27
Bosnia and Herzegovina	Girls	2000	15	11.37
Bosnia and Herzegovina	Girls	2001	15	11.48
Bosnia and Herzegovina	Girls	2002	15	11.59
Bosnia and Herzegovina	Girls	2003	15	11.71
Bosnia and Herzegovina	Girls	2004	15	11.83
Bosnia and Herzegovina	Girls	2005	15	11.96
Bosnia and Herzegovina	Girls	2006	15	12.09
Bosnia and Herzegovina	Girls	2007	15	12.23
Bosnia and Herzegovina	Girls	2008	15	12.38
Bosnia and Herzegovina	Girls	2009	15	12.53
Bosnia and Herzegovina	Girls	2010	15	12.68
Bosnia and Herzegovina	Girls	2011	15	12.85
Bosnia and Herzegovina	Girls	2012	15	13.02
Bosnia and Herzegovina	Girls	2013	15	13.19
Bosnia and Herzegovina	Girls	2014	15	13.38
Bosnia and Herzegovina	Girls	2015	15	13.57
Bosnia and Herzegovina	Girls	2016	15	13.78
Bosnia and Herzegovina	Girls	1975	16	10.50
Bosnia and Herzegovina	Girls	1976	16	10.56
Bosnia and Herzegovina	Girls	1977	16	10.63

Bosnia and Herzegovina	Girls	1978	16	10.70
Bosnia and Herzegovina	Girls	1979	16	10.77
Bosnia and Herzegovina	Girls	1980	16	10.85
Bosnia and Herzegovina	Girls	1981	16	10.92
Bosnia and Herzegovina	Girls	1982	16	10.99
Bosnia and Herzegovina	Girls	1983	16	11.07
Bosnia and Herzegovina	Girls	1984	16	11.14
Bosnia and Herzegovina	Girls	1985	16	11.22
Bosnia and Herzegovina	Girls	1986	16	11.30
Bosnia and Herzegovina	Girls	1987	16	11.38
Bosnia and Herzegovina	Girls	1988	16	11.46
Bosnia and Herzegovina	Girls	1989	16	11.54
Bosnia and Herzegovina	Girls	1990	16	11.62
Bosnia and Herzegovina	Girls	1991	16	11.70
Bosnia and Herzegovina	Girls	1992	16	11.79
Bosnia and Herzegovina	Girls	1993	16	11.87
Bosnia and Herzegovina	Girls	1994	16	11.96
Bosnia and Herzegovina	Girls	1995	16	12.05
Bosnia and Herzegovina	Girls	1996	16	12.14
Bosnia and Herzegovina	Girls	1997	16	12.23
Bosnia and Herzegovina	Girls	1998	16	12.33
Bosnia and Herzegovina	Girls	1999	16	12.43
Bosnia and Herzegovina	Girls	2000	16	12.54
Bosnia and Herzegovina	Girls	2001	16	12.65
Bosnia and Herzegovina	Girls	2002	16	12.76
Bosnia and Herzegovina	Girls	2003	16	12.88
Bosnia and Herzegovina	Girls	2004	16	13.01
Bosnia and Herzegovina	Girls	2005	16	13.14
Bosnia and Herzegovina	Girls	2006	16	13.28
Bosnia and Herzegovina	Girls	2007	16	13.42
Bosnia and Herzegovina	Girls	2008	16	13.57
Bosnia and Herzegovina	Girls	2009	16	13.72
Bosnia and Herzegovina	Girls	2010	16	13.88
Bosnia and Herzegovina	Girls	2011	16	14.05
Bosnia and Herzegovina	Girls	2012	16	14.23
Bosnia and Herzegovina	Girls	2013	16	14.41
Bosnia and Herzegovina	Girls	2014	16	14.60
Bosnia and Herzegovina	Girls	2015	16	14.80
Bosnia and Herzegovina	Girls	2016	16	15.01
Bosnia and Herzegovina	Girls	1975	17	11.28
Bosnia and Herzegovina	Girls	1976	17	11.35
Bosnia and Herzegovina	Girls	1977	17	11.42
Bosnia and Herzegovina	Girls	1978	17	11.49
Bosnia and Herzegovina	Girls	1979	17	11.57
Bosnia and Herzegovina	Girls	1980	17	11.64
Bosnia and Herzegovina	Girls	1981	17	11.72
Bosnia and Herzegovina	Girls	1982	17	11.80
Bosnia and Herzegovina	Girls	1983	17	11.87
Bosnia and Herzegovina	Girls	1984	17	11.95
Bosnia and Herzegovina	Girls	1985	17	12.03
Bosnia and Herzegovina	Girls	1986	17	12.11
Bosnia and Herzegovina	Girls	1987	17	12.19

Bosnia and Herzegovina	Girls	1988	17	12.27
Bosnia and Herzegovina	Girls	1989	17	12.36
Bosnia and Herzegovina	Girls	1990	17	12.44
Bosnia and Herzegovina	Girls	1991	17	12.53
Bosnia and Herzegovina	Girls	1992	17	12.62
Bosnia and Herzegovina	Girls	1993	17	12.71
Bosnia and Herzegovina	Girls	1994	17	12.80
Bosnia and Herzegovina	Girls	1995	17	12.89
Bosnia and Herzegovina	Girls	1996	17	12.98
Bosnia and Herzegovina	Girls	1997	17	13.08
Bosnia and Herzegovina	Girls	1998	17	13.18
Bosnia and Herzegovina	Girls	1999	17	13.29
Bosnia and Herzegovina	Girls	2000	17	13.40
Bosnia and Herzegovina	Girls	2001	17	13.51
Bosnia and Herzegovina	Girls	2002	17	13.63
Bosnia and Herzegovina	Girls	2003	17	13.76
Bosnia and Herzegovina	Girls	2004	17	13.89
Bosnia and Herzegovina	Girls	2005	17	14.02
Bosnia and Herzegovina	Girls	2006	17	14.17
Bosnia and Herzegovina	Girls	2007	17	14.31
Bosnia and Herzegovina	Girls	2008	17	14.47
Bosnia and Herzegovina	Girls	2009	17	14.63
Bosnia and Herzegovina	Girls	2010	17	14.80
Bosnia and Herzegovina	Girls	2011	17	14.97
Bosnia and Herzegovina	Girls	2012	17	15.15
Bosnia and Herzegovina	Girls	2013	17	15.34
Bosnia and Herzegovina	Girls	2014	17	15.54
Bosnia and Herzegovina	Girls	2015	17	15.75
Bosnia and Herzegovina	Girls	2016	17	15.97
Bosnia and Herzegovina	Girls	1975	18	11.81
Bosnia and Herzegovina	Girls	1976	18	11.88
Bosnia and Herzegovina	Girls	1977	18	11.96
Bosnia and Herzegovina	Girls	1978	18	12.03
Bosnia and Herzegovina	Girls	1979	18	12.11
Bosnia and Herzegovina	Girls	1980	18	12.19
Bosnia and Herzegovina	Girls	1981	18	12.28
Bosnia and Herzegovina	Girls	1982	18	12.36
Bosnia and Herzegovina	Girls	1983	18	12.44
Bosnia and Herzegovina	Girls	1984	18	12.52
Bosnia and Herzegovina	Girls	1985	18	12.61
Bosnia and Herzegovina	Girls	1986	18	12.69
Bosnia and Herzegovina	Girls	1987	18	12.78
Bosnia and Herzegovina	Girls	1988	18	12.87
Bosnia and Herzegovina	Girls	1989	18	12.96
Bosnia and Herzegovina	Girls	1990	18	13.05
Bosnia and Herzegovina	Girls	1991	18	13.14
Bosnia and Herzegovina	Girls	1992	18	13.23
Bosnia and Herzegovina	Girls	1993	18	13.33
Bosnia and Herzegovina	Girls	1994	18	13.43
Bosnia and Herzegovina	Girls	1995	18	13.53
Bosnia and Herzegovina	Girls	1996	18	13.63
Bosnia and Herzegovina	Girls	1997	18	13.74

Bosnia and Herzegovina	Girls	1998	18	13.84
Bosnia and Herzegovina	Girls	1999	18	13.96
Bosnia and Herzegovina	Girls	2000	18	14.08
Bosnia and Herzegovina	Girls	2001	18	14.20
Bosnia and Herzegovina	Girls	2002	18	14.33
Bosnia and Herzegovina	Girls	2003	18	14.47
Bosnia and Herzegovina	Girls	2004	18	14.61
Bosnia and Herzegovina	Girls	2005	18	14.76
Bosnia and Herzegovina	Girls	2006	18	14.91
Bosnia and Herzegovina	Girls	2007	18	15.07
Bosnia and Herzegovina	Girls	2008	18	15.24
Bosnia and Herzegovina	Girls	2009	18	15.41
Bosnia and Herzegovina	Girls	2010	18	15.59
Bosnia and Herzegovina	Girls	2011	18	15.78
Bosnia and Herzegovina	Girls	2012	18	15.98
Bosnia and Herzegovina	Girls	2013	18	16.19
Bosnia and Herzegovina	Girls	2014	18	16.40
Bosnia and Herzegovina	Girls	2015	18	16.63
Bosnia and Herzegovina	Girls	2016	18	16.86
Bosnia and Herzegovina	Girls	1975	Average	8.97
Bosnia and Herzegovina	Girls	1976	Average	9.03
Bosnia and Herzegovina	Girls	1977	Average	9.10
Bosnia and Herzegovina	Girls	1978	Average	9.16
Bosnia and Herzegovina	Girls	1979	Average	9.23
Bosnia and Herzegovina	Girls	1980	Average	9.30
Bosnia and Herzegovina	Girls	1981	Average	9.36
Bosnia and Herzegovina	Girls	1982	Average	9.43
Bosnia and Herzegovina	Girls	1983	Average	9.50
Bosnia and Herzegovina	Girls	1984	Average	9.58
Bosnia and Herzegovina	Girls	1985	Average	9.65
Bosnia and Herzegovina	Girls	1986	Average	9.72
Bosnia and Herzegovina	Girls	1987	Average	9.79
Bosnia and Herzegovina	Girls	1988	Average	9.87
Bosnia and Herzegovina	Girls	1989	Average	9.95
Bosnia and Herzegovina	Girls	1990	Average	10.02
Bosnia and Herzegovina	Girls	1991	Average	10.10
Bosnia and Herzegovina	Girls	1992	Average	10.18
Bosnia and Herzegovina	Girls	1993	Average	10.26
Bosnia and Herzegovina	Girls	1994	Average	10.35
Bosnia and Herzegovina	Girls	1995	Average	10.43
Bosnia and Herzegovina	Girls	1996	Average	10.52
Bosnia and Herzegovina	Girls	1997	Average	10.61
Bosnia and Herzegovina	Girls	1998	Average	10.70
Bosnia and Herzegovina	Girls	1999	Average	10.80
Bosnia and Herzegovina	Girls	2000	Average	10.90
Bosnia and Herzegovina	Girls	2001	Average	11.01
Bosnia and Herzegovina	Girls	2002	Average	11.12
Bosnia and Herzegovina	Girls	2003	Average	11.23
Bosnia and Herzegovina	Girls	2004	Average	11.35
Bosnia and Herzegovina	Girls	2005	Average	11.48
Bosnia and Herzegovina	Girls	2006	Average	11.61
Bosnia and Herzegovina	Girls	2007	Average	11.75

Bosnia and Herzegovina	Girls	2008	Average	11.89
Bosnia and Herzegovina	Girls	2009	Average	12.04
Bosnia and Herzegovina	Girls	2010	Average	12.19
Bosnia and Herzegovina	Girls	2011	Average	12.35
Bosnia and Herzegovina	Girls	2012	Average	12.52
Bosnia and Herzegovina	Girls	2013	Average	12.69
Bosnia and Herzegovina	Girls	2014	Average	12.88
Bosnia and Herzegovina	Girls	2015	Average	13.07
Bosnia and Herzegovina	Girls	2016	Average	13.27
Botswana	Boys	1975	12	3.64
Botswana	Boys	1976	12	3.65
Botswana	Boys	1977	12	3.67
Botswana	Boys	1978	12	3.69
Botswana	Boys	1979	12	3.70
Botswana	Boys	1980	12	3.72
Botswana	Boys	1981	12	3.74
Botswana	Boys	1982	12	3.76
Botswana	Boys	1983	12	3.79
Botswana	Boys	1984	12	3.81
Botswana	Boys	1985	12	3.84
Botswana	Boys	1986	12	3.88
Botswana	Boys	1987	12	3.92
Botswana	Boys	1988	12	3.96
Botswana	Boys	1989	12	4.01
Botswana	Boys	1990	12	4.07
Botswana	Boys	1991	12	4.13
Botswana	Boys	1992	12	4.19
Botswana	Boys	1993	12	4.26
Botswana	Boys	1994	12	4.33
Botswana	Boys	1995	12	4.41
Botswana	Boys	1996	12	4.48
Botswana	Boys	1997	12	4.56
Botswana	Boys	1998	12	4.65
Botswana	Boys	1999	12	4.74
Botswana	Boys	2000	12	4.83
Botswana	Boys	2001	12	4.93
Botswana	Boys	2002	12	5.03
Botswana	Boys	2003	12	5.14
Botswana	Boys	2004	12	5.25
Botswana	Boys	2005	12	5.37
Botswana	Boys	2006	12	5.50
Botswana	Boys	2007	12	5.63
Botswana	Boys	2008	12	5.76
Botswana	Boys	2009	12	5.90
Botswana	Boys	2010	12	6.05
Botswana	Boys	2011	12	6.20
Botswana	Boys	2012	12	6.36
Botswana	Boys	2013	12	6.52
Botswana	Boys	2014	12	6.69
Botswana	Boys	2015	12	6.87
Botswana	Boys	2016	12	7.06
Botswana	Boys	1975	13	4.54

Botswana	Boys	1976	13	4.56
Botswana	Boys	1977	13	4.58
Botswana	Boys	1978	13	4.60
Botswana	Boys	1979	13	4.62
Botswana	Boys	1980	13	4.65
Botswana	Boys	1981	13	4.67
Botswana	Boys	1982	13	4.70
Botswana	Boys	1983	13	4.73
Botswana	Boys	1984	13	4.76
Botswana	Boys	1985	13	4.80
Botswana	Boys	1986	13	4.84
Botswana	Boys	1987	13	4.89
Botswana	Boys	1988	13	4.94
Botswana	Boys	1989	13	5.00
Botswana	Boys	1990	13	5.07
Botswana	Boys	1991	13	5.14
Botswana	Boys	1992	13	5.22
Botswana	Boys	1993	13	5.30
Botswana	Boys	1994	13	5.38
Botswana	Boys	1995	13	5.47
Botswana	Boys	1996	13	5.56
Botswana	Boys	1997	13	5.65
Botswana	Boys	1998	13	5.75
Botswana	Boys	1999	13	5.85
Botswana	Boys	2000	13	5.96
Botswana	Boys	2001	13	6.08
Botswana	Boys	2002	13	6.20
Botswana	Boys	2003	13	6.32
Botswana	Boys	2004	13	6.46
Botswana	Boys	2005	13	6.59
Botswana	Boys	2006	13	6.74
Botswana	Boys	2007	13	6.89
Botswana	Boys	2008	13	7.05
Botswana	Boys	2009	13	7.21
Botswana	Boys	2010	13	7.38
Botswana	Boys	2011	13	7.55
Botswana	Boys	2012	13	7.73
Botswana	Boys	2013	13	7.92
Botswana	Boys	2014	13	8.12
Botswana	Boys	2015	13	8.33
Botswana	Boys	2016	13	8.55
Botswana	Boys	1975	14	5.74
Botswana	Boys	1976	14	5.76
Botswana	Boys	1977	14	5.79
Botswana	Boys	1978	14	5.81
Botswana	Boys	1979	14	5.84
Botswana	Boys	1980	14	5.87
Botswana	Boys	1981	14	5.90
Botswana	Boys	1982	14	5.94
Botswana	Boys	1983	14	5.97
Botswana	Boys	1984	14	6.01
Botswana	Boys	1985	14	6.06

Botswana	Boys	1986	14	6.11
Botswana	Boys	1987	14	6.16
Botswana	Boys	1988	14	6.23
Botswana	Boys	1989	14	6.30
Botswana	Boys	1990	14	6.37
Botswana	Boys	1991	14	6.46
Botswana	Boys	1992	14	6.54
Botswana	Boys	1993	14	6.63
Botswana	Boys	1994	14	6.73
Botswana	Boys	1995	14	6.83
Botswana	Boys	1996	14	6.93
Botswana	Boys	1997	14	7.04
Botswana	Boys	1998	14	7.15
Botswana	Boys	1999	14	7.26
Botswana	Boys	2000	14	7.39
Botswana	Boys	2001	14	7.52
Botswana	Boys	2002	14	7.65
Botswana	Boys	2003	14	7.79
Botswana	Boys	2004	14	7.94
Botswana	Boys	2005	14	8.10
Botswana	Boys	2006	14	8.26
Botswana	Boys	2007	14	8.43
Botswana	Boys	2008	14	8.60
Botswana	Boys	2009	14	8.78
Botswana	Boys	2010	14	8.96
Botswana	Boys	2011	14	9.16
Botswana	Boys	2012	14	9.36
Botswana	Boys	2013	14	9.57
Botswana	Boys	2014	14	9.79
Botswana	Boys	2015	14	10.02
Botswana	Boys	2016	14	10.26
Botswana	Boys	1975	15	7.17
Botswana	Boys	1976	15	7.21
Botswana	Boys	1977	15	7.24
Botswana	Boys	1978	15	7.27
Botswana	Boys	1979	15	7.30
Botswana	Boys	1980	15	7.34
Botswana	Boys	1981	15	7.38
Botswana	Boys	1982	15	7.42
Botswana	Boys	1983	15	7.46
Botswana	Boys	1984	15	7.51
Botswana	Boys	1985	15	7.56
Botswana	Boys	1986	15	7.62
Botswana	Boys	1987	15	7.69
Botswana	Boys	1988	15	7.76
Botswana	Boys	1989	15	7.84
Botswana	Boys	1990	15	7.93
Botswana	Boys	1991	15	8.02
Botswana	Boys	1992	15	8.12
Botswana	Boys	1993	15	8.22
Botswana	Boys	1994	15	8.32
Botswana	Boys	1995	15	8.43

Botswana	Boys	1996	15	8.55
Botswana	Boys	1997	15	8.66
Botswana	Boys	1998	15	8.79
Botswana	Boys	1999	15	8.91
Botswana	Boys	2000	15	9.05
Botswana	Boys	2001	15	9.19
Botswana	Boys	2002	15	9.34
Botswana	Boys	2003	15	9.49
Botswana	Boys	2004	15	9.65
Botswana	Boys	2005	15	9.82
Botswana	Boys	2006	15	10.00
Botswana	Boys	2007	15	10.18
Botswana	Boys	2008	15	10.36
Botswana	Boys	2009	15	10.56
Botswana	Boys	2010	15	10.75
Botswana	Boys	2011	15	10.96
Botswana	Boys	2012	15	11.17
Botswana	Boys	2013	15	11.40
Botswana	Boys	2014	15	11.63
Botswana	Boys	2015	15	11.88
Botswana	Boys	2016	15	12.14
Botswana	Boys	1975	16	8.77
Botswana	Boys	1976	16	8.81
Botswana	Boys	1977	16	8.85
Botswana	Boys	1978	16	8.89
Botswana	Boys	1979	16	8.94
Botswana	Boys	1980	16	8.98
Botswana	Boys	1981	16	9.02
Botswana	Boys	1982	16	9.07
Botswana	Boys	1983	16	9.13
Botswana	Boys	1984	16	9.18
Botswana	Boys	1985	16	9.24
Botswana	Boys	1986	16	9.31
Botswana	Boys	1987	16	9.38
Botswana	Boys	1988	16	9.47
Botswana	Boys	1989	16	9.56
Botswana	Boys	1990	16	9.66
Botswana	Boys	1991	16	9.76
Botswana	Boys	1992	16	9.87
Botswana	Boys	1993	16	9.98
Botswana	Boys	1994	16	10.09
Botswana	Boys	1995	16	10.21
Botswana	Boys	1996	16	10.34
Botswana	Boys	1997	16	10.46
Botswana	Boys	1998	16	10.60
Botswana	Boys	1999	16	10.73
Botswana	Boys	2000	16	10.88
Botswana	Boys	2001	16	11.03
Botswana	Boys	2002	16	11.19
Botswana	Boys	2003	16	11.36
Botswana	Boys	2004	16	11.53
Botswana	Boys	2005	16	11.71

Botswana	Boys	2006	16	11.90
Botswana	Boys	2007	16	12.09
Botswana	Boys	2008	16	12.29
Botswana	Boys	2009	16	12.49
Botswana	Boys	2010	16	12.70
Botswana	Boys	2011	16	12.91
Botswana	Boys	2012	16	13.14
Botswana	Boys	2013	16	13.37
Botswana	Boys	2014	16	13.61
Botswana	Boys	2015	16	13.87
Botswana	Boys	2016	16	14.14
Botswana	Boys	1975	17	10.43
Botswana	Boys	1976	17	10.48
Botswana	Boys	1977	17	10.53
Botswana	Boys	1978	17	10.58
Botswana	Boys	1979	17	10.63
Botswana	Boys	1980	17	10.68
Botswana	Boys	1981	17	10.73
Botswana	Boys	1982	17	10.79
Botswana	Boys	1983	17	10.85
Botswana	Boys	1984	17	10.92
Botswana	Boys	1985	17	10.99
Botswana	Boys	1986	17	11.06
Botswana	Boys	1987	17	11.15
Botswana	Boys	1988	17	11.24
Botswana	Boys	1989	17	11.34
Botswana	Boys	1990	17	11.45
Botswana	Boys	1991	17	11.57
Botswana	Boys	1992	17	11.69
Botswana	Boys	1993	17	11.81
Botswana	Boys	1994	17	11.93
Botswana	Boys	1995	17	12.07
Botswana	Boys	1996	17	12.20
Botswana	Boys	1997	17	12.34
Botswana	Boys	1998	17	12.48
Botswana	Boys	1999	17	12.63
Botswana	Boys	2000	17	12.79
Botswana	Boys	2001	17	12.95
Botswana	Boys	2002	17	13.13
Botswana	Boys	2003	17	13.30
Botswana	Boys	2004	17	13.49
Botswana	Boys	2005	17	13.68
Botswana	Boys	2006	17	13.88
Botswana	Boys	2007	17	14.09
Botswana	Boys	2008	17	14.30
Botswana	Boys	2009	17	14.51
Botswana	Boys	2010	17	14.73
Botswana	Boys	2011	17	14.96
Botswana	Boys	2012	17	15.19
Botswana	Boys	2013	17	15.44
Botswana	Boys	2014	17	15.69
Botswana	Boys	2015	17	15.95

Botswana	Boys	2016	17	16.23
Botswana	Boys	1975	18	12.03
Botswana	Boys	1976	18	12.09
Botswana	Boys	1977	18	12.15
Botswana	Boys	1978	18	12.20
Botswana	Boys	1979	18	12.26
Botswana	Boys	1980	18	12.32
Botswana	Boys	1981	18	12.39
Botswana	Boys	1982	18	12.46
Botswana	Boys	1983	18	12.53
Botswana	Boys	1984	18	12.60
Botswana	Boys	1985	18	12.69
Botswana	Boys	1986	18	12.77
Botswana	Boys	1987	18	12.87
Botswana	Boys	1988	18	12.98
Botswana	Boys	1989	18	13.09
Botswana	Boys	1990	18	13.22
Botswana	Boys	1991	18	13.34
Botswana	Boys	1992	18	13.48
Botswana	Boys	1993	18	13.61
Botswana	Boys	1994	18	13.75
Botswana	Boys	1995	18	13.90
Botswana	Boys	1996	18	14.05
Botswana	Boys	1997	18	14.20
Botswana	Boys	1998	18	14.36
Botswana	Boys	1999	18	14.52
Botswana	Boys	2000	18	14.70
Botswana	Boys	2001	18	14.88
Botswana	Boys	2002	18	15.07
Botswana	Boys	2003	18	15.26
Botswana	Boys	2004	18	15.46
Botswana	Boys	2005	18	15.67
Botswana	Boys	2006	18	15.89
Botswana	Boys	2007	18	16.11
Botswana	Boys	2008	18	16.34
Botswana	Boys	2009	18	16.57
Botswana	Boys	2010	18	16.81
Botswana	Boys	2011	18	17.05
Botswana	Boys	2012	18	17.30
Botswana	Boys	2013	18	17.56
Botswana	Boys	2014	18	17.83
Botswana	Boys	2015	18	18.11
Botswana	Boys	2016	18	18.40
Botswana	Boys	1975	Average	7.47
Botswana	Boys	1976	Average	7.51
Botswana	Boys	1977	Average	7.54
Botswana	Boys	1978	Average	7.58
Botswana	Boys	1979	Average	7.61
Botswana	Boys	1980	Average	7.65
Botswana	Boys	1981	Average	7.69
Botswana	Boys	1982	Average	7.73
Botswana	Boys	1983	Average	7.78

Botswana	Boys	1984	Average	7.83
Botswana	Boys	1985	Average	7.88
Botswana	Boys	1986	Average	7.94
Botswana	Boys	1987	Average	8.01
Botswana	Boys	1988	Average	8.08
Botswana	Boys	1989	Average	8.16
Botswana	Boys	1990	Average	8.25
Botswana	Boys	1991	Average	8.35
Botswana	Boys	1992	Average	8.44
Botswana	Boys	1993	Average	8.54
Botswana	Boys	1994	Average	8.65
Botswana	Boys	1995	Average	8.76
Botswana	Boys	1996	Average	8.87
Botswana	Boys	1997	Average	8.99
Botswana	Boys	1998	Average	9.11
Botswana	Boys	1999	Average	9.24
Botswana	Boys	2000	Average	9.37
Botswana	Boys	2001	Average	9.51
Botswana	Boys	2002	Average	9.66
Botswana	Boys	2003	Average	9.81
Botswana	Boys	2004	Average	9.97
Botswana	Boys	2005	Average	10.14
Botswana	Boys	2006	Average	10.31
Botswana	Boys	2007	Average	10.49
Botswana	Boys	2008	Average	10.67
Botswana	Boys	2009	Average	10.86
Botswana	Boys	2010	Average	11.05
Botswana	Boys	2011	Average	11.26
Botswana	Boys	2012	Average	11.46
Botswana	Boys	2013	Average	11.68
Botswana	Boys	2014	Average	11.91
Botswana	Boys	2015	Average	12.15
Botswana	Boys	2016	Average	12.40
Botswana	Girls	1975	12	4.32
Botswana	Girls	1976	12	4.35
Botswana	Girls	1977	12	4.38
Botswana	Girls	1978	12	4.42
Botswana	Girls	1979	12	4.46
Botswana	Girls	1980	12	4.50
Botswana	Girls	1981	12	4.54
Botswana	Girls	1982	12	4.60
Botswana	Girls	1983	12	4.65
Botswana	Girls	1984	12	4.72
Botswana	Girls	1985	12	4.78
Botswana	Girls	1986	12	4.86
Botswana	Girls	1987	12	4.95
Botswana	Girls	1988	12	5.05
Botswana	Girls	1989	12	5.17
Botswana	Girls	1990	12	5.29
Botswana	Girls	1991	12	5.42
Botswana	Girls	1992	12	5.56
Botswana	Girls	1993	12	5.70

Botswana	Girls	1994	12	5.84
Botswana	Girls	1995	12	5.99
Botswana	Girls	1996	12	6.15
Botswana	Girls	1997	12	6.30
Botswana	Girls	1998	12	6.46
Botswana	Girls	1999	12	6.62
Botswana	Girls	2000	12	6.79
Botswana	Girls	2001	12	6.96
Botswana	Girls	2002	12	7.14
Botswana	Girls	2003	12	7.33
Botswana	Girls	2004	12	7.52
Botswana	Girls	2005	12	7.71
Botswana	Girls	2006	12	7.91
Botswana	Girls	2007	12	8.11
Botswana	Girls	2008	12	8.31
Botswana	Girls	2009	12	8.52
Botswana	Girls	2010	12	8.73
Botswana	Girls	2011	12	8.95
Botswana	Girls	2012	12	9.17
Botswana	Girls	2013	12	9.39
Botswana	Girls	2014	12	9.62
Botswana	Girls	2015	12	9.85
Botswana	Girls	2016	12	10.09
Botswana	Girls	1975	13	5.45
Botswana	Girls	1976	13	5.49
Botswana	Girls	1977	13	5.53
Botswana	Girls	1978	13	5.58
Botswana	Girls	1979	13	5.62
Botswana	Girls	1980	13	5.67
Botswana	Girls	1981	13	5.73
Botswana	Girls	1982	13	5.79
Botswana	Girls	1983	13	5.86
Botswana	Girls	1984	13	5.94
Botswana	Girls	1985	13	6.02
Botswana	Girls	1986	13	6.12
Botswana	Girls	1987	13	6.22
Botswana	Girls	1988	13	6.34
Botswana	Girls	1989	13	6.47
Botswana	Girls	1990	13	6.62
Botswana	Girls	1991	13	6.77
Botswana	Girls	1992	13	6.93
Botswana	Girls	1993	13	7.09
Botswana	Girls	1994	13	7.26
Botswana	Girls	1995	13	7.43
Botswana	Girls	1996	13	7.61
Botswana	Girls	1997	13	7.78
Botswana	Girls	1998	13	7.96
Botswana	Girls	1999	13	8.15
Botswana	Girls	2000	13	8.34
Botswana	Girls	2001	13	8.54
Botswana	Girls	2002	13	8.74
Botswana	Girls	2003	13	8.95

Botswana	Girls	2004	13	9.16
Botswana	Girls	2005	13	9.38
Botswana	Girls	2006	13	9.61
Botswana	Girls	2007	13	9.83
Botswana	Girls	2008	13	10.07
Botswana	Girls	2009	13	10.30
Botswana	Girls	2010	13	10.54
Botswana	Girls	2011	13	10.79
Botswana	Girls	2012	13	11.04
Botswana	Girls	2013	13	11.29
Botswana	Girls	2014	13	11.55
Botswana	Girls	2015	13	11.82
Botswana	Girls	2016	13	12.09
Botswana	Girls	1975	14	6.73
Botswana	Girls	1976	14	6.78
Botswana	Girls	1977	14	6.83
Botswana	Girls	1978	14	6.88
Botswana	Girls	1979	14	6.94
Botswana	Girls	1980	14	7.00
Botswana	Girls	1981	14	7.07
Botswana	Girls	1982	14	7.14
Botswana	Girls	1983	14	7.22
Botswana	Girls	1984	14	7.30
Botswana	Girls	1985	14	7.40
Botswana	Girls	1986	14	7.51
Botswana	Girls	1987	14	7.63
Botswana	Girls	1988	14	7.76
Botswana	Girls	1989	14	7.91
Botswana	Girls	1990	14	8.07
Botswana	Girls	1991	14	8.24
Botswana	Girls	1992	14	8.42
Botswana	Girls	1993	14	8.59
Botswana	Girls	1994	14	8.78
Botswana	Girls	1995	14	8.97
Botswana	Girls	1996	14	9.16
Botswana	Girls	1997	14	9.35
Botswana	Girls	1998	14	9.54
Botswana	Girls	1999	14	9.74
Botswana	Girls	2000	14	9.95
Botswana	Girls	2001	14	10.16
Botswana	Girls	2002	14	10.38
Botswana	Girls	2003	14	10.61
Botswana	Girls	2004	14	10.84
Botswana	Girls	2005	14	11.07
Botswana	Girls	2006	14	11.31
Botswana	Girls	2007	14	11.56
Botswana	Girls	2008	14	11.81
Botswana	Girls	2009	14	12.06
Botswana	Girls	2010	14	12.32
Botswana	Girls	2011	14	12.58
Botswana	Girls	2012	14	12.85
Botswana	Girls	2013	14	13.12

Botswana	Girls	2014	14	13.40
Botswana	Girls	2015	14	13.69
Botswana	Girls	2016	14	13.99
Botswana	Girls	1975	15	7.98
Botswana	Girls	1976	15	8.03
Botswana	Girls	1977	15	8.09
Botswana	Girls	1978	15	8.15
Botswana	Girls	1979	15	8.22
Botswana	Girls	1980	15	8.28
Botswana	Girls	1981	15	8.36
Botswana	Girls	1982	15	8.44
Botswana	Girls	1983	15	8.52
Botswana	Girls	1984	15	8.62
Botswana	Girls	1985	15	8.72
Botswana	Girls	1986	15	8.84
Botswana	Girls	1987	15	8.97
Botswana	Girls	1988	15	9.11
Botswana	Girls	1989	15	9.27
Botswana	Girls	1990	15	9.45
Botswana	Girls	1991	15	9.63
Botswana	Girls	1992	15	9.81
Botswana	Girls	1993	15	10.00
Botswana	Girls	1994	15	10.19
Botswana	Girls	1995	15	10.39
Botswana	Girls	1996	15	10.59
Botswana	Girls	1997	15	10.79
Botswana	Girls	1998	15	10.99
Botswana	Girls	1999	15	11.20
Botswana	Girls	2000	15	11.42
Botswana	Girls	2001	15	11.64
Botswana	Girls	2002	15	11.86
Botswana	Girls	2003	15	12.09
Botswana	Girls	2004	15	12.33
Botswana	Girls	2005	15	12.58
Botswana	Girls	2006	15	12.83
Botswana	Girls	2007	15	13.08
Botswana	Girls	2008	15	13.34
Botswana	Girls	2009	15	13.60
Botswana	Girls	2010	15	13.87
Botswana	Girls	2011	15	14.14
Botswana	Girls	2012	15	14.42
Botswana	Girls	2013	15	14.70
Botswana	Girls	2014	15	14.99
Botswana	Girls	2015	15	15.30
Botswana	Girls	2016	15	15.61
Botswana	Girls	1975	16	9.01
Botswana	Girls	1976	16	9.07
Botswana	Girls	1977	16	9.13
Botswana	Girls	1978	16	9.20
Botswana	Girls	1979	16	9.27
Botswana	Girls	1980	16	9.34
Botswana	Girls	1981	16	9.42

Botswana	Girls	1982	16	9.50
Botswana	Girls	1983	16	9.60
Botswana	Girls	1984	16	9.70
Botswana	Girls	1985	16	9.81
Botswana	Girls	1986	16	9.93
Botswana	Girls	1987	16	10.07
Botswana	Girls	1988	16	10.22
Botswana	Girls	1989	16	10.38
Botswana	Girls	1990	16	10.56
Botswana	Girls	1991	16	10.75
Botswana	Girls	1992	16	10.94
Botswana	Girls	1993	16	11.13
Botswana	Girls	1994	16	11.33
Botswana	Girls	1995	16	11.54
Botswana	Girls	1996	16	11.74
Botswana	Girls	1997	16	11.95
Botswana	Girls	1998	16	12.16
Botswana	Girls	1999	16	12.37
Botswana	Girls	2000	16	12.59
Botswana	Girls	2001	16	12.82
Botswana	Girls	2002	16	13.05
Botswana	Girls	2003	16	13.29
Botswana	Girls	2004	16	13.53
Botswana	Girls	2005	16	13.78
Botswana	Girls	2006	16	14.04
Botswana	Girls	2007	16	14.30
Botswana	Girls	2008	16	14.56
Botswana	Girls	2009	16	14.83
Botswana	Girls	2010	16	15.10
Botswana	Girls	2011	16	15.38
Botswana	Girls	2012	16	15.67
Botswana	Girls	2013	16	15.96
Botswana	Girls	2014	16	16.26
Botswana	Girls	2015	16	16.57
Botswana	Girls	2016	16	16.89
Botswana	Girls	1975	17	9.72
Botswana	Girls	1976	17	9.79
Botswana	Girls	1977	17	9.86
Botswana	Girls	1978	17	9.93
Botswana	Girls	1979	17	10.00
Botswana	Girls	1980	17	10.08
Botswana	Girls	1981	17	10.16
Botswana	Girls	1982	17	10.25
Botswana	Girls	1983	17	10.35
Botswana	Girls	1984	17	10.45
Botswana	Girls	1985	17	10.57
Botswana	Girls	1986	17	10.70
Botswana	Girls	1987	17	10.84
Botswana	Girls	1988	17	11.00
Botswana	Girls	1989	17	11.17
Botswana	Girls	1990	17	11.36
Botswana	Girls	1991	17	11.55

Botswana	Girls	1992	17	11.75
Botswana	Girls	1993	17	11.95
Botswana	Girls	1994	17	12.16
Botswana	Girls	1995	17	12.38
Botswana	Girls	1996	17	12.59
Botswana	Girls	1997	17	12.80
Botswana	Girls	1998	17	13.02
Botswana	Girls	1999	17	13.25
Botswana	Girls	2000	17	13.48
Botswana	Girls	2001	17	13.72
Botswana	Girls	2002	17	13.96
Botswana	Girls	2003	17	14.21
Botswana	Girls	2004	17	14.46
Botswana	Girls	2005	17	14.72
Botswana	Girls	2006	17	14.99
Botswana	Girls	2007	17	15.26
Botswana	Girls	2008	17	15.53
Botswana	Girls	2009	17	15.81
Botswana	Girls	2010	17	16.10
Botswana	Girls	2011	17	16.39
Botswana	Girls	2012	17	16.69
Botswana	Girls	2013	17	16.99
Botswana	Girls	2014	17	17.30
Botswana	Girls	2015	17	17.63
Botswana	Girls	2016	17	17.96
Botswana	Girls	1975	18	10.15
Botswana	Girls	1976	18	10.22
Botswana	Girls	1977	18	10.29
Botswana	Girls	1978	18	10.37
Botswana	Girls	1979	18	10.44
Botswana	Girls	1980	18	10.52
Botswana	Girls	1981	18	10.61
Botswana	Girls	1982	18	10.71
Botswana	Girls	1983	18	10.81
Botswana	Girls	1984	18	10.93
Botswana	Girls	1985	18	11.05
Botswana	Girls	1986	18	11.19
Botswana	Girls	1987	18	11.34
Botswana	Girls	1988	18	11.51
Botswana	Girls	1989	18	11.69
Botswana	Girls	1990	18	11.90
Botswana	Girls	1991	18	12.11
Botswana	Girls	1992	18	12.32
Botswana	Girls	1993	18	12.55
Botswana	Girls	1994	18	12.77
Botswana	Girls	1995	18	13.00
Botswana	Girls	1996	18	13.24
Botswana	Girls	1997	18	13.47
Botswana	Girls	1998	18	13.71
Botswana	Girls	1999	18	13.96
Botswana	Girls	2000	18	14.21
Botswana	Girls	2001	18	14.47

Botswana	Girls	2002	18	14.73
Botswana	Girls	2003	18	15.01
Botswana	Girls	2004	18	15.28
Botswana	Girls	2005	18	15.57
Botswana	Girls	2006	18	15.86
Botswana	Girls	2007	18	16.15
Botswana	Girls	2008	18	16.45
Botswana	Girls	2009	18	16.76
Botswana	Girls	2010	18	17.07
Botswana	Girls	2011	18	17.38
Botswana	Girls	2012	18	17.70
Botswana	Girls	2013	18	18.03
Botswana	Girls	2014	18	18.37
Botswana	Girls	2015	18	18.71
Botswana	Girls	2016	18	19.07
Botswana	Girls	1975	Average	7.62
Botswana	Girls	1976	Average	7.67
Botswana	Girls	1977	Average	7.73
Botswana	Girls	1978	Average	7.79
Botswana	Girls	1979	Average	7.85
Botswana	Girls	1980	Average	7.91
Botswana	Girls	1981	Average	7.98
Botswana	Girls	1982	Average	8.06
Botswana	Girls	1983	Average	8.14
Botswana	Girls	1984	Average	8.24
Botswana	Girls	1985	Average	8.34
Botswana	Girls	1986	Average	8.45
Botswana	Girls	1987	Average	8.57
Botswana	Girls	1988	Average	8.71
Botswana	Girls	1989	Average	8.87
Botswana	Girls	1990	Average	9.04
Botswana	Girls	1991	Average	9.21
Botswana	Girls	1992	Average	9.39
Botswana	Girls	1993	Average	9.57
Botswana	Girls	1994	Average	9.76
Botswana	Girls	1995	Average	9.96
Botswana	Girls	1996	Average	10.15
Botswana	Girls	1997	Average	10.35
Botswana	Girls	1998	Average	10.55
Botswana	Girls	1999	Average	10.76
Botswana	Girls	2000	Average	10.97
Botswana	Girls	2001	Average	11.19
Botswana	Girls	2002	Average	11.41
Botswana	Girls	2003	Average	11.64
Botswana	Girls	2004	Average	11.87
Botswana	Girls	2005	Average	12.12
Botswana	Girls	2006	Average	12.36
Botswana	Girls	2007	Average	12.61
Botswana	Girls	2008	Average	12.87
Botswana	Girls	2009	Average	13.13
Botswana	Girls	2010	Average	13.39
Botswana	Girls	2011	Average	13.66

Botswana	Girls	2012	Average	13.93
Botswana	Girls	2013	Average	14.21
Botswana	Girls	2014	Average	14.50
Botswana	Girls	2015	Average	14.80
Botswana	Girls	2016	Average	15.10
Brazil	Boys	1975	12	6.18
Brazil	Boys	1976	12	6.26
Brazil	Boys	1977	12	6.34
Brazil	Boys	1978	12	6.43
Brazil	Boys	1979	12	6.53
Brazil	Boys	1980	12	6.63
Brazil	Boys	1981	12	6.73
Brazil	Boys	1982	12	6.83
Brazil	Boys	1983	12	6.94
Brazil	Boys	1984	12	7.06
Brazil	Boys	1985	12	7.17
Brazil	Boys	1986	12	7.29
Brazil	Boys	1987	12	7.42
Brazil	Boys	1988	12	7.55
Brazil	Boys	1989	12	7.68
Brazil	Boys	1990	12	7.81
Brazil	Boys	1991	12	7.95
Brazil	Boys	1992	12	8.09
Brazil	Boys	1993	12	8.24
Brazil	Boys	1994	12	8.39
Brazil	Boys	1995	12	8.54
Brazil	Boys	1996	12	8.70
Brazil	Boys	1997	12	8.86
Brazil	Boys	1998	12	9.02
Brazil	Boys	1999	12	9.19
Brazil	Boys	2000	12	9.36
Brazil	Boys	2001	12	9.53
Brazil	Boys	2002	12	9.70
Brazil	Boys	2003	12	9.88
Brazil	Boys	2004	12	10.05
Brazil	Boys	2005	12	10.23
Brazil	Boys	2006	12	10.41
Brazil	Boys	2007	12	10.60
Brazil	Boys	2008	12	10.79
Brazil	Boys	2009	12	10.98
Brazil	Boys	2010	12	11.18
Brazil	Boys	2011	12	11.39
Brazil	Boys	2012	12	11.59
Brazil	Boys	2013	12	11.80
Brazil	Boys	2014	12	12.01
Brazil	Boys	2015	12	12.22
Brazil	Boys	2016	12	12.44
Brazil	Boys	1975	13	7.64
Brazil	Boys	1976	13	7.73
Brazil	Boys	1977	13	7.83
Brazil	Boys	1978	13	7.94
Brazil	Boys	1979	13	8.05

Brazil	Boys	1980	13	8.16
Brazil	Boys	1981	13	8.28
Brazil	Boys	1982	13	8.40
Brazil	Boys	1983	13	8.53
Brazil	Boys	1984	13	8.66
Brazil	Boys	1985	13	8.79
Brazil	Boys	1986	13	8.93
Brazil	Boys	1987	13	9.07
Brazil	Boys	1988	13	9.22
Brazil	Boys	1989	13	9.37
Brazil	Boys	1990	13	9.53
Brazil	Boys	1991	13	9.69
Brazil	Boys	1992	13	9.85
Brazil	Boys	1993	13	10.02
Brazil	Boys	1994	13	10.19
Brazil	Boys	1995	13	10.37
Brazil	Boys	1996	13	10.55
Brazil	Boys	1997	13	10.74
Brazil	Boys	1998	13	10.93
Brazil	Boys	1999	13	11.12
Brazil	Boys	2000	13	11.32
Brazil	Boys	2001	13	11.52
Brazil	Boys	2002	13	11.72
Brazil	Boys	2003	13	11.92
Brazil	Boys	2004	13	12.12
Brazil	Boys	2005	13	12.33
Brazil	Boys	2006	13	12.55
Brazil	Boys	2007	13	12.77
Brazil	Boys	2008	13	12.99
Brazil	Boys	2009	13	13.22
Brazil	Boys	2010	13	13.46
Brazil	Boys	2011	13	13.70
Brazil	Boys	2012	13	13.95
Brazil	Boys	2013	13	14.20
Brazil	Boys	2014	13	14.46
Brazil	Boys	2015	13	14.72
Brazil	Boys	2016	13	14.98
Brazil	Boys	1975	14	9.43
Brazil	Boys	1976	14	9.53
Brazil	Boys	1977	14	9.64
Brazil	Boys	1978	14	9.76
Brazil	Boys	1979	14	9.88
Brazil	Boys	1980	14	10.01
Brazil	Boys	1981	14	10.13
Brazil	Boys	1982	14	10.27
Brazil	Boys	1983	14	10.41
Brazil	Boys	1984	14	10.55
Brazil	Boys	1985	14	10.70
Brazil	Boys	1986	14	10.85
Brazil	Boys	1987	14	11.00
Brazil	Boys	1988	14	11.17
Brazil	Boys	1989	14	11.33

Brazil	Boys	1990	14	11.50
Brazil	Boys	1991	14	11.67
Brazil	Boys	1992	14	11.85
Brazil	Boys	1993	14	12.04
Brazil	Boys	1994	14	12.22
Brazil	Boys	1995	14	12.42
Brazil	Boys	1996	14	12.61
Brazil	Boys	1997	14	12.81
Brazil	Boys	1998	14	13.02
Brazil	Boys	1999	14	13.23
Brazil	Boys	2000	14	13.45
Brazil	Boys	2001	14	13.67
Brazil	Boys	2002	14	13.89
Brazil	Boys	2003	14	14.11
Brazil	Boys	2004	14	14.33
Brazil	Boys	2005	14	14.56
Brazil	Boys	2006	14	14.80
Brazil	Boys	2007	14	15.04
Brazil	Boys	2008	14	15.29
Brazil	Boys	2009	14	15.55
Brazil	Boys	2010	14	15.81
Brazil	Boys	2011	14	16.09
Brazil	Boys	2012	14	16.36
Brazil	Boys	2013	14	16.65
Brazil	Boys	2014	14	16.93
Brazil	Boys	2015	14	17.23
Brazil	Boys	2016	14	17.53
Brazil	Boys	1975	15	11.46
Brazil	Boys	1976	15	11.57
Brazil	Boys	1977	15	11.69
Brazil	Boys	1978	15	11.82
Brazil	Boys	1979	15	11.94
Brazil	Boys	1980	15	12.07
Brazil	Boys	1981	15	12.21
Brazil	Boys	1982	15	12.35
Brazil	Boys	1983	15	12.49
Brazil	Boys	1984	15	12.64
Brazil	Boys	1985	15	12.80
Brazil	Boys	1986	15	12.96
Brazil	Boys	1987	15	13.12
Brazil	Boys	1988	15	13.28
Brazil	Boys	1989	15	13.45
Brazil	Boys	1990	15	13.63
Brazil	Boys	1991	15	13.81
Brazil	Boys	1992	15	14.00
Brazil	Boys	1993	15	14.18
Brazil	Boys	1994	15	14.38
Brazil	Boys	1995	15	14.58
Brazil	Boys	1996	15	14.78
Brazil	Boys	1997	15	14.99
Brazil	Boys	1998	15	15.21
Brazil	Boys	1999	15	15.43

Brazil	Boys	2000	15	15.65
Brazil	Boys	2001	15	15.88
Brazil	Boys	2002	15	16.10
Brazil	Boys	2003	15	16.33
Brazil	Boys	2004	15	16.57
Brazil	Boys	2005	15	16.81
Brazil	Boys	2006	15	17.05
Brazil	Boys	2007	15	17.31
Brazil	Boys	2008	15	17.57
Brazil	Boys	2009	15	17.83
Brazil	Boys	2010	15	18.11
Brazil	Boys	2011	15	18.40
Brazil	Boys	2012	15	18.69
Brazil	Boys	2013	15	18.99
Brazil	Boys	2014	15	19.30
Brazil	Boys	2015	15	19.61
Brazil	Boys	2016	15	19.93
Brazil	Boys	1975	16	13.66
Brazil	Boys	1976	16	13.78
Brazil	Boys	1977	16	13.90
Brazil	Boys	1978	16	14.02
Brazil	Boys	1979	16	14.15
Brazil	Boys	1980	16	14.29
Brazil	Boys	1981	16	14.43
Brazil	Boys	1982	16	14.57
Brazil	Boys	1983	16	14.72
Brazil	Boys	1984	16	14.87
Brazil	Boys	1985	16	15.03
Brazil	Boys	1986	16	15.18
Brazil	Boys	1987	16	15.35
Brazil	Boys	1988	16	15.52
Brazil	Boys	1989	16	15.69
Brazil	Boys	1990	16	15.87
Brazil	Boys	1991	16	16.05
Brazil	Boys	1992	16	16.23
Brazil	Boys	1993	16	16.42
Brazil	Boys	1994	16	16.62
Brazil	Boys	1995	16	16.82
Brazil	Boys	1996	16	17.02
Brazil	Boys	1997	16	17.23
Brazil	Boys	1998	16	17.45
Brazil	Boys	1999	16	17.67
Brazil	Boys	2000	16	17.90
Brazil	Boys	2001	16	18.12
Brazil	Boys	2002	16	18.35
Brazil	Boys	2003	16	18.58
Brazil	Boys	2004	16	18.81
Brazil	Boys	2005	16	19.06
Brazil	Boys	2006	16	19.30
Brazil	Boys	2007	16	19.56
Brazil	Boys	2008	16	19.82
Brazil	Boys	2009	16	20.09

Brazil	Boys	2010	16	20.37
Brazil	Boys	2011	16	20.66
Brazil	Boys	2012	16	20.96
Brazil	Boys	2013	16	21.26
Brazil	Boys	2014	16	21.58
Brazil	Boys	2015	16	21.90
Brazil	Boys	2016	16	22.23
Brazil	Boys	1975	17	15.93
Brazil	Boys	1976	17	16.05
Brazil	Boys	1977	17	16.18
Brazil	Boys	1978	17	16.30
Brazil	Boys	1979	17	16.44
Brazil	Boys	1980	17	16.57
Brazil	Boys	1981	17	16.71
Brazil	Boys	1982	17	16.86
Brazil	Boys	1983	17	17.01
Brazil	Boys	1984	17	17.16
Brazil	Boys	1985	17	17.32
Brazil	Boys	1986	17	17.48
Brazil	Boys	1987	17	17.64
Brazil	Boys	1988	17	17.81
Brazil	Boys	1989	17	17.98
Brazil	Boys	1990	17	18.16
Brazil	Boys	1991	17	18.34
Brazil	Boys	1992	17	18.53
Brazil	Boys	1993	17	18.72
Brazil	Boys	1994	17	18.91
Brazil	Boys	1995	17	19.11
Brazil	Boys	1996	17	19.31
Brazil	Boys	1997	17	19.52
Brazil	Boys	1998	17	19.74
Brazil	Boys	1999	17	19.96
Brazil	Boys	2000	17	20.19
Brazil	Boys	2001	17	20.41
Brazil	Boys	2002	17	20.64
Brazil	Boys	2003	17	20.87
Brazil	Boys	2004	17	21.10
Brazil	Boys	2005	17	21.34
Brazil	Boys	2006	17	21.59
Brazil	Boys	2007	17	21.84
Brazil	Boys	2008	17	22.10
Brazil	Boys	2009	17	22.37
Brazil	Boys	2010	17	22.66
Brazil	Boys	2011	17	22.95
Brazil	Boys	2012	17	23.24
Brazil	Boys	2013	17	23.55
Brazil	Boys	2014	17	23.86
Brazil	Boys	2015	17	24.19
Brazil	Boys	2016	17	24.52
Brazil	Boys	1975	18	18.21
Brazil	Boys	1976	18	18.33
Brazil	Boys	1977	18	18.47

Brazil	Boys	1978	18	18.60
Brazil	Boys	1979	18	18.74
Brazil	Boys	1980	18	18.88
Brazil	Boys	1981	18	19.02
Brazil	Boys	1982	18	19.17
Brazil	Boys	1983	18	19.32
Brazil	Boys	1984	18	19.48
Brazil	Boys	1985	18	19.64
Brazil	Boys	1986	18	19.81
Brazil	Boys	1987	18	19.98
Brazil	Boys	1988	18	20.15
Brazil	Boys	1989	18	20.32
Brazil	Boys	1990	18	20.50
Brazil	Boys	1991	18	20.69
Brazil	Boys	1992	18	20.88
Brazil	Boys	1993	18	21.07
Brazil	Boys	1994	18	21.27
Brazil	Boys	1995	18	21.47
Brazil	Boys	1996	18	21.68
Brazil	Boys	1997	18	21.89
Brazil	Boys	1998	18	22.11
Brazil	Boys	1999	18	22.33
Brazil	Boys	2000	18	22.56
Brazil	Boys	2001	18	22.79
Brazil	Boys	2002	18	23.02
Brazil	Boys	2003	18	23.25
Brazil	Boys	2004	18	23.49
Brazil	Boys	2005	18	23.73
Brazil	Boys	2006	18	23.98
Brazil	Boys	2007	18	24.24
Brazil	Boys	2008	18	24.50
Brazil	Boys	2009	18	24.78
Brazil	Boys	2010	18	25.06
Brazil	Boys	2011	18	25.36
Brazil	Boys	2012	18	25.66
Brazil	Boys	2013	18	25.97
Brazil	Boys	2014	18	26.28
Brazil	Boys	2015	18	26.61
Brazil	Boys	2016	18	26.95
Brazil	Boys	1975	Average	11.78
Brazil	Boys	1976	Average	11.89
Brazil	Boys	1977	Average	12.01
Brazil	Boys	1978	Average	12.13
Brazil	Boys	1979	Average	12.25
Brazil	Boys	1980	Average	12.37
Brazil	Boys	1981	Average	12.50
Brazil	Boys	1982	Average	12.64
Brazil	Boys	1983	Average	12.77
Brazil	Boys	1984	Average	12.92
Brazil	Boys	1985	Average	13.06
Brazil	Boys	1986	Average	13.21
Brazil	Boys	1987	Average	13.37

Brazil	Boys	1988	Average	13.53
Brazil	Boys	1989	Average	13.69
Brazil	Boys	1990	Average	13.86
Brazil	Boys	1991	Average	14.03
Brazil	Boys	1992	Average	14.20
Brazil	Boys	1993	Average	14.38
Brazil	Boys	1994	Average	14.57
Brazil	Boys	1995	Average	14.76
Brazil	Boys	1996	Average	14.95
Brazil	Boys	1997	Average	15.15
Brazil	Boys	1998	Average	15.35
Brazil	Boys	1999	Average	15.56
Brazil	Boys	2000	Average	15.78
Brazil	Boys	2001	Average	15.99
Brazil	Boys	2002	Average	16.20
Brazil	Boys	2003	Average	16.42
Brazil	Boys	2004	Average	16.64
Brazil	Boys	2005	Average	16.87
Brazil	Boys	2006	Average	17.10
Brazil	Boys	2007	Average	17.34
Brazil	Boys	2008	Average	17.58
Brazil	Boys	2009	Average	17.83
Brazil	Boys	2010	Average	18.09
Brazil	Boys	2011	Average	18.36
Brazil	Boys	2012	Average	18.64
Brazil	Boys	2013	Average	18.92
Brazil	Boys	2014	Average	19.20
Brazil	Boys	2015	Average	19.50
Brazil	Boys	2016	Average	19.80
Brazil	Girls	1975	12	6.55
Brazil	Girls	1976	12	6.63
Brazil	Girls	1977	12	6.71
Brazil	Girls	1978	12	6.80
Brazil	Girls	1979	12	6.88
Brazil	Girls	1980	12	6.97
Brazil	Girls	1981	12	7.07
Brazil	Girls	1982	12	7.16
Brazil	Girls	1983	12	7.25
Brazil	Girls	1984	12	7.35
Brazil	Girls	1985	12	7.45
Brazil	Girls	1986	12	7.54
Brazil	Girls	1987	12	7.64
Brazil	Girls	1988	12	7.74
Brazil	Girls	1989	12	7.84
Brazil	Girls	1990	12	7.94
Brazil	Girls	1991	12	8.04
Brazil	Girls	1992	12	8.14
Brazil	Girls	1993	12	8.23
Brazil	Girls	1994	12	8.33
Brazil	Girls	1995	12	8.43
Brazil	Girls	1996	12	8.53
Brazil	Girls	1997	12	8.63

Brazil	Girls	1998	12	8.72
Brazil	Girls	1999	12	8.82
Brazil	Girls	2000	12	8.92
Brazil	Girls	2001	12	9.02
Brazil	Girls	2002	12	9.11
Brazil	Girls	2003	12	9.20
Brazil	Girls	2004	12	9.29
Brazil	Girls	2005	12	9.38
Brazil	Girls	2006	12	9.47
Brazil	Girls	2007	12	9.57
Brazil	Girls	2008	12	9.66
Brazil	Girls	2009	12	9.76
Brazil	Girls	2010	12	9.86
Brazil	Girls	2011	12	9.97
Brazil	Girls	2012	12	10.07
Brazil	Girls	2013	12	10.18
Brazil	Girls	2014	12	10.29
Brazil	Girls	2015	12	10.41
Brazil	Girls	2016	12	10.52
Brazil	Girls	1975	13	8.08
Brazil	Girls	1976	13	8.17
Brazil	Girls	1977	13	8.27
Brazil	Girls	1978	13	8.37
Brazil	Girls	1979	13	8.47
Brazil	Girls	1980	13	8.57
Brazil	Girls	1981	13	8.67
Brazil	Girls	1982	13	8.78
Brazil	Girls	1983	13	8.89
Brazil	Girls	1984	13	8.99
Brazil	Girls	1985	13	9.10
Brazil	Girls	1986	13	9.21
Brazil	Girls	1987	13	9.33
Brazil	Girls	1988	13	9.44
Brazil	Girls	1989	13	9.55
Brazil	Girls	1990	13	9.66
Brazil	Girls	1991	13	9.77
Brazil	Girls	1992	13	9.88
Brazil	Girls	1993	13	10.00
Brazil	Girls	1994	13	10.11
Brazil	Girls	1995	13	10.22
Brazil	Girls	1996	13	10.33
Brazil	Girls	1997	13	10.44
Brazil	Girls	1998	13	10.55
Brazil	Girls	1999	13	10.66
Brazil	Girls	2000	13	10.77
Brazil	Girls	2001	13	10.87
Brazil	Girls	2002	13	10.98
Brazil	Girls	2003	13	11.08
Brazil	Girls	2004	13	11.18
Brazil	Girls	2005	13	11.28
Brazil	Girls	2006	13	11.39
Brazil	Girls	2007	13	11.49

Brazil	Girls	2008	13	11.60
Brazil	Girls	2009	13	11.71
Brazil	Girls	2010	13	11.82
Brazil	Girls	2011	13	11.94
Brazil	Girls	2012	13	12.06
Brazil	Girls	2013	13	12.18
Brazil	Girls	2014	13	12.31
Brazil	Girls	2015	13	12.43
Brazil	Girls	2016	13	12.56
Brazil	Girls	1975	14	9.67
Brazil	Girls	1976	14	9.78
Brazil	Girls	1977	14	9.88
Brazil	Girls	1978	14	9.99
Brazil	Girls	1979	14	10.10
Brazil	Girls	1980	14	10.21
Brazil	Girls	1981	14	10.32
Brazil	Girls	1982	14	10.44
Brazil	Girls	1983	14	10.55
Brazil	Girls	1984	14	10.67
Brazil	Girls	1985	14	10.79
Brazil	Girls	1986	14	10.91
Brazil	Girls	1987	14	11.03
Brazil	Girls	1988	14	11.15
Brazil	Girls	1989	14	11.27
Brazil	Girls	1990	14	11.39
Brazil	Girls	1991	14	11.51
Brazil	Girls	1992	14	11.63
Brazil	Girls	1993	14	11.75
Brazil	Girls	1994	14	11.86
Brazil	Girls	1995	14	11.98
Brazil	Girls	1996	14	12.10
Brazil	Girls	1997	14	12.22
Brazil	Girls	1998	14	12.33
Brazil	Girls	1999	14	12.45
Brazil	Girls	2000	14	12.57
Brazil	Girls	2001	14	12.68
Brazil	Girls	2002	14	12.79
Brazil	Girls	2003	14	12.90
Brazil	Girls	2004	14	13.01
Brazil	Girls	2005	14	13.12
Brazil	Girls	2006	14	13.22
Brazil	Girls	2007	14	13.34
Brazil	Girls	2008	14	13.45
Brazil	Girls	2009	14	13.56
Brazil	Girls	2010	14	13.68
Brazil	Girls	2011	14	13.81
Brazil	Girls	2012	14	13.94
Brazil	Girls	2013	14	14.07
Brazil	Girls	2014	14	14.20
Brazil	Girls	2015	14	14.34
Brazil	Girls	2016	14	14.48
Brazil	Girls	1975	15	11.12

Brazil	Girls	1976	15	11.22
Brazil	Girls	1977	15	11.33
Brazil	Girls	1978	15	11.44
Brazil	Girls	1979	15	11.56
Brazil	Girls	1980	15	11.67
Brazil	Girls	1981	15	11.79
Brazil	Girls	1982	15	11.91
Brazil	Girls	1983	15	12.03
Brazil	Girls	1984	15	12.15
Brazil	Girls	1985	15	12.28
Brazil	Girls	1986	15	12.40
Brazil	Girls	1987	15	12.52
Brazil	Girls	1988	15	12.65
Brazil	Girls	1989	15	12.77
Brazil	Girls	1990	15	12.90
Brazil	Girls	1991	15	13.02
Brazil	Girls	1992	15	13.14
Brazil	Girls	1993	15	13.27
Brazil	Girls	1994	15	13.39
Brazil	Girls	1995	15	13.51
Brazil	Girls	1996	15	13.63
Brazil	Girls	1997	15	13.75
Brazil	Girls	1998	15	13.87
Brazil	Girls	1999	15	14.00
Brazil	Girls	2000	15	14.12
Brazil	Girls	2001	15	14.23
Brazil	Girls	2002	15	14.35
Brazil	Girls	2003	15	14.46
Brazil	Girls	2004	15	14.57
Brazil	Girls	2005	15	14.68
Brazil	Girls	2006	15	14.79
Brazil	Girls	2007	15	14.90
Brazil	Girls	2008	15	15.01
Brazil	Girls	2009	15	15.13
Brazil	Girls	2010	15	15.26
Brazil	Girls	2011	15	15.38
Brazil	Girls	2012	15	15.51
Brazil	Girls	2013	15	15.65
Brazil	Girls	2014	15	15.79
Brazil	Girls	2015	15	15.93
Brazil	Girls	2016	15	16.07
Brazil	Girls	1975	16	12.25
Brazil	Girls	1976	16	12.36
Brazil	Girls	1977	16	12.47
Brazil	Girls	1978	16	12.58
Brazil	Girls	1979	16	12.70
Brazil	Girls	1980	16	12.82
Brazil	Girls	1981	16	12.94
Brazil	Girls	1982	16	13.06
Brazil	Girls	1983	16	13.18
Brazil	Girls	1984	16	13.31
Brazil	Girls	1985	16	13.43

Brazil	Girls	1986	16	13.56
Brazil	Girls	1987	16	13.68
Brazil	Girls	1988	16	13.81
Brazil	Girls	1989	16	13.94
Brazil	Girls	1990	16	14.07
Brazil	Girls	1991	16	14.19
Brazil	Girls	1992	16	14.32
Brazil	Girls	1993	16	14.45
Brazil	Girls	1994	16	14.57
Brazil	Girls	1995	16	14.69
Brazil	Girls	1996	16	14.82
Brazil	Girls	1997	16	14.94
Brazil	Girls	1998	16	15.07
Brazil	Girls	1999	16	15.19
Brazil	Girls	2000	16	15.31
Brazil	Girls	2001	16	15.43
Brazil	Girls	2002	16	15.54
Brazil	Girls	2003	16	15.66
Brazil	Girls	2004	16	15.77
Brazil	Girls	2005	16	15.88
Brazil	Girls	2006	16	15.99
Brazil	Girls	2007	16	16.11
Brazil	Girls	2008	16	16.23
Brazil	Girls	2009	16	16.35
Brazil	Girls	2010	16	16.47
Brazil	Girls	2011	16	16.60
Brazil	Girls	2012	16	16.73
Brazil	Girls	2013	16	16.87
Brazil	Girls	2014	16	17.01
Brazil	Girls	2015	16	17.16
Brazil	Girls	2016	16	17.30
Brazil	Girls	1975	17	13.05
Brazil	Girls	1976	17	13.16
Brazil	Girls	1977	17	13.27
Brazil	Girls	1978	17	13.39
Brazil	Girls	1979	17	13.51
Brazil	Girls	1980	17	13.63
Brazil	Girls	1981	17	13.76
Brazil	Girls	1982	17	13.88
Brazil	Girls	1983	17	14.01
Brazil	Girls	1984	17	14.14
Brazil	Girls	1985	17	14.27
Brazil	Girls	1986	17	14.40
Brazil	Girls	1987	17	14.53
Brazil	Girls	1988	17	14.66
Brazil	Girls	1989	17	14.79
Brazil	Girls	1990	17	14.93
Brazil	Girls	1991	17	15.06
Brazil	Girls	1992	17	15.19
Brazil	Girls	1993	17	15.32
Brazil	Girls	1994	17	15.45
Brazil	Girls	1995	17	15.58

Brazil	Girls	1996	17	15.71
Brazil	Girls	1997	17	15.84
Brazil	Girls	1998	17	15.97
Brazil	Girls	1999	17	16.09
Brazil	Girls	2000	17	16.22
Brazil	Girls	2001	17	16.35
Brazil	Girls	2002	17	16.47
Brazil	Girls	2003	17	16.58
Brazil	Girls	2004	17	16.70
Brazil	Girls	2005	17	16.82
Brazil	Girls	2006	17	16.93
Brazil	Girls	2007	17	17.05
Brazil	Girls	2008	17	17.18
Brazil	Girls	2009	17	17.30
Brazil	Girls	2010	17	17.43
Brazil	Girls	2011	17	17.57
Brazil	Girls	2012	17	17.71
Brazil	Girls	2013	17	17.85
Brazil	Girls	2014	17	17.99
Brazil	Girls	2015	17	18.14
Brazil	Girls	2016	17	18.30
Brazil	Girls	1975	18	13.63
Brazil	Girls	1976	18	13.75
Brazil	Girls	1977	18	13.87
Brazil	Girls	1978	18	13.99
Brazil	Girls	1979	18	14.12
Brazil	Girls	1980	18	14.25
Brazil	Girls	1981	18	14.38
Brazil	Girls	1982	18	14.52
Brazil	Girls	1983	18	14.65
Brazil	Girls	1984	18	14.79
Brazil	Girls	1985	18	14.93
Brazil	Girls	1986	18	15.07
Brazil	Girls	1987	18	15.22
Brazil	Girls	1988	18	15.36
Brazil	Girls	1989	18	15.50
Brazil	Girls	1990	18	15.64
Brazil	Girls	1991	18	15.79
Brazil	Girls	1992	18	15.93
Brazil	Girls	1993	18	16.07
Brazil	Girls	1994	18	16.21
Brazil	Girls	1995	18	16.35
Brazil	Girls	1996	18	16.49
Brazil	Girls	1997	18	16.63
Brazil	Girls	1998	18	16.77
Brazil	Girls	1999	18	16.91
Brazil	Girls	2000	18	17.05
Brazil	Girls	2001	18	17.19
Brazil	Girls	2002	18	17.32
Brazil	Girls	2003	18	17.45
Brazil	Girls	2004	18	17.57
Brazil	Girls	2005	18	17.70

Brazil	Girls	2006	18	17.83
Brazil	Girls	2007	18	17.96
Brazil	Girls	2008	18	18.09
Brazil	Girls	2009	18	18.23
Brazil	Girls	2010	18	18.37
Brazil	Girls	2011	18	18.52
Brazil	Girls	2012	18	18.67
Brazil	Girls	2013	18	18.82
Brazil	Girls	2014	18	18.98
Brazil	Girls	2015	18	19.15
Brazil	Girls	2016	18	19.31
Brazil	Girls	1975	Average	10.62
Brazil	Girls	1976	Average	10.72
Brazil	Girls	1977	Average	10.83
Brazil	Girls	1978	Average	10.94
Brazil	Girls	1979	Average	11.05
Brazil	Girls	1980	Average	11.16
Brazil	Girls	1981	Average	11.28
Brazil	Girls	1982	Average	11.39
Brazil	Girls	1983	Average	11.51
Brazil	Girls	1984	Average	11.63
Brazil	Girls	1985	Average	11.75
Brazil	Girls	1986	Average	11.87
Brazil	Girls	1987	Average	11.99
Brazil	Girls	1988	Average	12.11
Brazil	Girls	1989	Average	12.24
Brazil	Girls	1990	Average	12.36
Brazil	Girls	1991	Average	12.48
Brazil	Girls	1992	Average	12.60
Brazil	Girls	1993	Average	12.73
Brazil	Girls	1994	Average	12.85
Brazil	Girls	1995	Average	12.97
Brazil	Girls	1996	Average	13.09
Brazil	Girls	1997	Average	13.21
Brazil	Girls	1998	Average	13.33
Brazil	Girls	1999	Average	13.45
Brazil	Girls	2000	Average	13.57
Brazil	Girls	2001	Average	13.68
Brazil	Girls	2002	Average	13.79
Brazil	Girls	2003	Average	13.90
Brazil	Girls	2004	Average	14.01
Brazil	Girls	2005	Average	14.12
Brazil	Girls	2006	Average	14.23
Brazil	Girls	2007	Average	14.34
Brazil	Girls	2008	Average	14.46
Brazil	Girls	2009	Average	14.58
Brazil	Girls	2010	Average	14.70
Brazil	Girls	2011	Average	14.83
Brazil	Girls	2012	Average	14.96
Brazil	Girls	2013	Average	15.09
Brazil	Girls	2014	Average	15.23
Brazil	Girls	2015	Average	15.36

Brazil	Girls	2016	Average	15.51
Brunei Darussalam	Boys	1975	12	5.50
Brunei Darussalam	Boys	1976	12	5.55
Brunei Darussalam	Boys	1977	12	5.59
Brunei Darussalam	Boys	1978	12	5.63
Brunei Darussalam	Boys	1979	12	5.67
Brunei Darussalam	Boys	1980	12	5.70
Brunei Darussalam	Boys	1981	12	5.75
Brunei Darussalam	Boys	1982	12	5.80
Brunei Darussalam	Boys	1983	12	5.87
Brunei Darussalam	Boys	1984	12	5.95
Brunei Darussalam	Boys	1985	12	6.04
Brunei Darussalam	Boys	1986	12	6.14
Brunei Darussalam	Boys	1987	12	6.26
Brunei Darussalam	Boys	1988	12	6.39
Brunei Darussalam	Boys	1989	12	6.53
Brunei Darussalam	Boys	1990	12	6.68
Brunei Darussalam	Boys	1991	12	6.84
Brunei Darussalam	Boys	1992	12	7.00
Brunei Darussalam	Boys	1993	12	7.18
Brunei Darussalam	Boys	1994	12	7.36
Brunei Darussalam	Boys	1995	12	7.55
Brunei Darussalam	Boys	1996	12	7.75
Brunei Darussalam	Boys	1997	12	7.95
Brunei Darussalam	Boys	1998	12	8.17
Brunei Darussalam	Boys	1999	12	8.39
Brunei Darussalam	Boys	2000	12	8.62
Brunei Darussalam	Boys	2001	12	8.86
Brunei Darussalam	Boys	2002	12	9.11
Brunei Darussalam	Boys	2003	12	9.37
Brunei Darussalam	Boys	2004	12	9.63
Brunei Darussalam	Boys	2005	12	9.90
Brunei Darussalam	Boys	2006	12	10.17
Brunei Darussalam	Boys	2007	12	10.45
Brunei Darussalam	Boys	2008	12	10.73
Brunei Darussalam	Boys	2009	12	11.02
Brunei Darussalam	Boys	2010	12	11.31
Brunei Darussalam	Boys	2011	12	11.61
Brunei Darussalam	Boys	2012	12	11.90
Brunei Darussalam	Boys	2013	12	12.20
Brunei Darussalam	Boys	2014	12	12.49
Brunei Darussalam	Boys	2015	12	12.78
Brunei Darussalam	Boys	2016	12	13.07
Brunei Darussalam	Boys	1975	13	6.70
Brunei Darussalam	Boys	1976	13	6.76
Brunei Darussalam	Boys	1977	13	6.81
Brunei Darussalam	Boys	1978	13	6.85
Brunei Darussalam	Boys	1979	13	6.89
Brunei Darussalam	Boys	1980	13	6.93
Brunei Darussalam	Boys	1981	13	6.97
Brunei Darussalam	Boys	1982	13	7.04
Brunei Darussalam	Boys	1983	13	7.11

Brunei Darussalam	Boys	1984	13	7.20
Brunei Darussalam	Boys	1985	13	7.30
Brunei Darussalam	Boys	1986	13	7.42
Brunei Darussalam	Boys	1987	13	7.55
Brunei Darussalam	Boys	1988	13	7.69
Brunei Darussalam	Boys	1989	13	7.85
Brunei Darussalam	Boys	1990	13	8.02
Brunei Darussalam	Boys	1991	13	8.20
Brunei Darussalam	Boys	1992	13	8.39
Brunei Darussalam	Boys	1993	13	8.59
Brunei Darussalam	Boys	1994	13	8.79
Brunei Darussalam	Boys	1995	13	9.01
Brunei Darussalam	Boys	1996	13	9.24
Brunei Darussalam	Boys	1997	13	9.48
Brunei Darussalam	Boys	1998	13	9.73
Brunei Darussalam	Boys	1999	13	9.99
Brunei Darussalam	Boys	2000	13	10.26
Brunei Darussalam	Boys	2001	13	10.54
Brunei Darussalam	Boys	2002	13	10.83
Brunei Darussalam	Boys	2003	13	11.13
Brunei Darussalam	Boys	2004	13	11.44
Brunei Darussalam	Boys	2005	13	11.76
Brunei Darussalam	Boys	2006	13	12.09
Brunei Darussalam	Boys	2007	13	12.42
Brunei Darussalam	Boys	2008	13	12.76
Brunei Darussalam	Boys	2009	13	13.11
Brunei Darussalam	Boys	2010	13	13.47
Brunei Darussalam	Boys	2011	13	13.83
Brunei Darussalam	Boys	2012	13	14.19
Brunei Darussalam	Boys	2013	13	14.56
Brunei Darussalam	Boys	2014	13	14.92
Brunei Darussalam	Boys	2015	13	15.29
Brunei Darussalam	Boys	2016	13	15.66
Brunei Darussalam	Boys	1975	14	8.17
Brunei Darussalam	Boys	1976	14	8.23
Brunei Darussalam	Boys	1977	14	8.28
Brunei Darussalam	Boys	1978	14	8.32
Brunei Darussalam	Boys	1979	14	8.36
Brunei Darussalam	Boys	1980	14	8.40
Brunei Darussalam	Boys	1981	14	8.45
Brunei Darussalam	Boys	1982	14	8.52
Brunei Darussalam	Boys	1983	14	8.60
Brunei Darussalam	Boys	1984	14	8.69
Brunei Darussalam	Boys	1985	14	8.79
Brunei Darussalam	Boys	1986	14	8.92
Brunei Darussalam	Boys	1987	14	9.05
Brunei Darussalam	Boys	1988	14	9.21
Brunei Darussalam	Boys	1989	14	9.38
Brunei Darussalam	Boys	1990	14	9.56
Brunei Darussalam	Boys	1991	14	9.75
Brunei Darussalam	Boys	1992	14	9.95
Brunei Darussalam	Boys	1993	14	10.16

Brunei Darussalam	Boys	1994	14	10.39
Brunei Darussalam	Boys	1995	14	10.62
Brunei Darussalam	Boys	1996	14	10.87
Brunei Darussalam	Boys	1997	14	11.13
Brunei Darussalam	Boys	1998	14	11.40
Brunei Darussalam	Boys	1999	14	11.68
Brunei Darussalam	Boys	2000	14	11.97
Brunei Darussalam	Boys	2001	14	12.28
Brunei Darussalam	Boys	2002	14	12.60
Brunei Darussalam	Boys	2003	14	12.94
Brunei Darussalam	Boys	2004	14	13.28
Brunei Darussalam	Boys	2005	14	13.64
Brunei Darussalam	Boys	2006	14	14.01
Brunei Darussalam	Boys	2007	14	14.38
Brunei Darussalam	Boys	2008	14	14.77
Brunei Darussalam	Boys	2009	14	15.17
Brunei Darussalam	Boys	2010	14	15.57
Brunei Darussalam	Boys	2011	14	15.99
Brunei Darussalam	Boys	2012	14	16.41
Brunei Darussalam	Boys	2013	14	16.83
Brunei Darussalam	Boys	2014	14	17.26
Brunei Darussalam	Boys	2015	14	17.69
Brunei Darussalam	Boys	2016	14	18.13
Brunei Darussalam	Boys	1975	15	9.85
Brunei Darussalam	Boys	1976	15	9.91
Brunei Darussalam	Boys	1977	15	9.96
Brunei Darussalam	Boys	1978	15	10.01
Brunei Darussalam	Boys	1979	15	10.05
Brunei Darussalam	Boys	1980	15	10.09
Brunei Darussalam	Boys	1981	15	10.14
Brunei Darussalam	Boys	1982	15	10.20
Brunei Darussalam	Boys	1983	15	10.28
Brunei Darussalam	Boys	1984	15	10.37
Brunei Darussalam	Boys	1985	15	10.48
Brunei Darussalam	Boys	1986	15	10.60
Brunei Darussalam	Boys	1987	15	10.74
Brunei Darussalam	Boys	1988	15	10.90
Brunei Darussalam	Boys	1989	15	11.07
Brunei Darussalam	Boys	1990	15	11.25
Brunei Darussalam	Boys	1991	15	11.44
Brunei Darussalam	Boys	1992	15	11.65
Brunei Darussalam	Boys	1993	15	11.87
Brunei Darussalam	Boys	1994	15	12.09
Brunei Darussalam	Boys	1995	15	12.34
Brunei Darussalam	Boys	1996	15	12.59
Brunei Darussalam	Boys	1997	15	12.85
Brunei Darussalam	Boys	1998	15	13.13
Brunei Darussalam	Boys	1999	15	13.42
Brunei Darussalam	Boys	2000	15	13.73
Brunei Darussalam	Boys	2001	15	14.05
Brunei Darussalam	Boys	2002	15	14.39
Brunei Darussalam	Boys	2003	15	14.74

Brunei Darussalam	Boys	2004	15	15.10
Brunei Darussalam	Boys	2005	15	15.48
Brunei Darussalam	Boys	2006	15	15.87
Brunei Darussalam	Boys	2007	15	16.27
Brunei Darussalam	Boys	2008	15	16.68
Brunei Darussalam	Boys	2009	15	17.10
Brunei Darussalam	Boys	2010	15	17.54
Brunei Darussalam	Boys	2011	15	17.99
Brunei Darussalam	Boys	2012	15	18.44
Brunei Darussalam	Boys	2013	15	18.91
Brunei Darussalam	Boys	2014	15	19.38
Brunei Darussalam	Boys	2015	15	19.85
Brunei Darussalam	Boys	2016	15	20.33
Brunei Darussalam	Boys	1975	16	11.69
Brunei Darussalam	Boys	1976	16	11.76
Brunei Darussalam	Boys	1977	16	11.81
Brunei Darussalam	Boys	1978	16	11.85
Brunei Darussalam	Boys	1979	16	11.89
Brunei Darussalam	Boys	1980	16	11.93
Brunei Darussalam	Boys	1981	16	11.98
Brunei Darussalam	Boys	1982	16	12.05
Brunei Darussalam	Boys	1983	16	12.12
Brunei Darussalam	Boys	1984	16	12.21
Brunei Darussalam	Boys	1985	16	12.32
Brunei Darussalam	Boys	1986	16	12.44
Brunei Darussalam	Boys	1987	16	12.58
Brunei Darussalam	Boys	1988	16	12.73
Brunei Darussalam	Boys	1989	16	12.90
Brunei Darussalam	Boys	1990	16	13.08
Brunei Darussalam	Boys	1991	16	13.27
Brunei Darussalam	Boys	1992	16	13.48
Brunei Darussalam	Boys	1993	16	13.69
Brunei Darussalam	Boys	1994	16	13.92
Brunei Darussalam	Boys	1995	16	14.16
Brunei Darussalam	Boys	1996	16	14.42
Brunei Darussalam	Boys	1997	16	14.68
Brunei Darussalam	Boys	1998	16	14.96
Brunei Darussalam	Boys	1999	16	15.26
Brunei Darussalam	Boys	2000	16	15.57
Brunei Darussalam	Boys	2001	16	15.89
Brunei Darussalam	Boys	2002	16	16.23
Brunei Darussalam	Boys	2003	16	16.58
Brunei Darussalam	Boys	2004	16	16.95
Brunei Darussalam	Boys	2005	16	17.34
Brunei Darussalam	Boys	2006	16	17.74
Brunei Darussalam	Boys	2007	16	18.15
Brunei Darussalam	Boys	2008	16	18.57
Brunei Darussalam	Boys	2009	16	19.01
Brunei Darussalam	Boys	2010	16	19.46
Brunei Darussalam	Boys	2011	16	19.93
Brunei Darussalam	Boys	2012	16	20.40
Brunei Darussalam	Boys	2013	16	20.89

Brunei Darussalam	Boys	2014	16	21.38
Brunei Darussalam	Boys	2015	16	21.89
Brunei Darussalam	Boys	2016	16	22.40
Brunei Darussalam	Boys	1975	17	13.62
Brunei Darussalam	Boys	1976	17	13.69
Brunei Darussalam	Boys	1977	17	13.74
Brunei Darussalam	Boys	1978	17	13.78
Brunei Darussalam	Boys	1979	17	13.82
Brunei Darussalam	Boys	1980	17	13.86
Brunei Darussalam	Boys	1981	17	13.91
Brunei Darussalam	Boys	1982	17	13.98
Brunei Darussalam	Boys	1983	17	14.06
Brunei Darussalam	Boys	1984	17	14.15
Brunei Darussalam	Boys	1985	17	14.25
Brunei Darussalam	Boys	1986	17	14.37
Brunei Darussalam	Boys	1987	17	14.51
Brunei Darussalam	Boys	1988	17	14.66
Brunei Darussalam	Boys	1989	17	14.83
Brunei Darussalam	Boys	1990	17	15.02
Brunei Darussalam	Boys	1991	17	15.21
Brunei Darussalam	Boys	1992	17	15.41
Brunei Darussalam	Boys	1993	17	15.63
Brunei Darussalam	Boys	1994	17	15.86
Brunei Darussalam	Boys	1995	17	16.10
Brunei Darussalam	Boys	1996	17	16.35
Brunei Darussalam	Boys	1997	17	16.62
Brunei Darussalam	Boys	1998	17	16.90
Brunei Darussalam	Boys	1999	17	17.20
Brunei Darussalam	Boys	2000	17	17.51
Brunei Darussalam	Boys	2001	17	17.84
Brunei Darussalam	Boys	2002	17	18.19
Brunei Darussalam	Boys	2003	17	18.55
Brunei Darussalam	Boys	2004	17	18.92
Brunei Darussalam	Boys	2005	17	19.31
Brunei Darussalam	Boys	2006	17	19.72
Brunei Darussalam	Boys	2007	17	20.13
Brunei Darussalam	Boys	2008	17	20.57
Brunei Darussalam	Boys	2009	17	21.02
Brunei Darussalam	Boys	2010	17	21.48
Brunei Darussalam	Boys	2011	17	21.96
Brunei Darussalam	Boys	2012	17	22.45
Brunei Darussalam	Boys	2013	17	22.95
Brunei Darussalam	Boys	2014	17	23.46
Brunei Darussalam	Boys	2015	17	23.98
Brunei Darussalam	Boys	2016	17	24.51
Brunei Darussalam	Boys	1975	18	15.57
Brunei Darussalam	Boys	1976	18	15.64
Brunei Darussalam	Boys	1977	18	15.70
Brunei Darussalam	Boys	1978	18	15.75
Brunei Darussalam	Boys	1979	18	15.79
Brunei Darussalam	Boys	1980	18	15.83
Brunei Darussalam	Boys	1981	18	15.88

Brunei Darussalam	Boys	1982	18	15.95
Brunei Darussalam	Boys	1983	18	16.03
Brunei Darussalam	Boys	1984	18	16.13
Brunei Darussalam	Boys	1985	18	16.24
Brunei Darussalam	Boys	1986	18	16.37
Brunei Darussalam	Boys	1987	18	16.51
Brunei Darussalam	Boys	1988	18	16.67
Brunei Darussalam	Boys	1989	18	16.84
Brunei Darussalam	Boys	1990	18	17.03
Brunei Darussalam	Boys	1991	18	17.24
Brunei Darussalam	Boys	1992	18	17.45
Brunei Darussalam	Boys	1993	18	17.67
Brunei Darussalam	Boys	1994	18	17.91
Brunei Darussalam	Boys	1995	18	18.16
Brunei Darussalam	Boys	1996	18	18.43
Brunei Darussalam	Boys	1997	18	18.70
Brunei Darussalam	Boys	1998	18	19.00
Brunei Darussalam	Boys	1999	18	19.31
Brunei Darussalam	Boys	2000	18	19.63
Brunei Darussalam	Boys	2001	18	19.97
Brunei Darussalam	Boys	2002	18	20.33
Brunei Darussalam	Boys	2003	18	20.70
Brunei Darussalam	Boys	2004	18	21.09
Brunei Darussalam	Boys	2005	18	21.50
Brunei Darussalam	Boys	2006	18	21.91
Brunei Darussalam	Boys	2007	18	22.35
Brunei Darussalam	Boys	2008	18	22.80
Brunei Darussalam	Boys	2009	18	23.26
Brunei Darussalam	Boys	2010	18	23.75
Brunei Darussalam	Boys	2011	18	24.24
Brunei Darussalam	Boys	2012	18	24.75
Brunei Darussalam	Boys	2013	18	25.26
Brunei Darussalam	Boys	2014	18	25.79
Brunei Darussalam	Boys	2015	18	26.33
Brunei Darussalam	Boys	2016	18	26.88
Brunei Darussalam	Boys	1975	Average	10.16
Brunei Darussalam	Boys	1976	Average	10.22
Brunei Darussalam	Boys	1977	Average	10.27
Brunei Darussalam	Boys	1978	Average	10.31
Brunei Darussalam	Boys	1979	Average	10.35
Brunei Darussalam	Boys	1980	Average	10.39
Brunei Darussalam	Boys	1981	Average	10.44
Brunei Darussalam	Boys	1982	Average	10.51
Brunei Darussalam	Boys	1983	Average	10.58
Brunei Darussalam	Boys	1984	Average	10.67
Brunei Darussalam	Boys	1985	Average	10.77
Brunei Darussalam	Boys	1986	Average	10.89
Brunei Darussalam	Boys	1987	Average	11.03
Brunei Darussalam	Boys	1988	Average	11.18
Brunei Darussalam	Boys	1989	Average	11.34
Brunei Darussalam	Boys	1990	Average	11.52
Brunei Darussalam	Boys	1991	Average	11.71

Brunei Darussalam	Boys	1992	Average	11.90
Brunei Darussalam	Boys	1993	Average	12.11
Brunei Darussalam	Boys	1994	Average	12.33
Brunei Darussalam	Boys	1995	Average	12.56
Brunei Darussalam	Boys	1996	Average	12.81
Brunei Darussalam	Boys	1997	Average	13.06
Brunei Darussalam	Boys	1998	Average	13.33
Brunei Darussalam	Boys	1999	Average	13.61
Brunei Darussalam	Boys	2000	Average	13.90
Brunei Darussalam	Boys	2001	Average	14.20
Brunei Darussalam	Boys	2002	Average	14.52
Brunei Darussalam	Boys	2003	Average	14.86
Brunei Darussalam	Boys	2004	Average	15.20
Brunei Darussalam	Boys	2005	Average	15.56
Brunei Darussalam	Boys	2006	Average	15.93
Brunei Darussalam	Boys	2007	Average	16.31
Brunei Darussalam	Boys	2008	Average	16.70
Brunei Darussalam	Boys	2009	Average	17.10
Brunei Darussalam	Boys	2010	Average	17.51
Brunei Darussalam	Boys	2011	Average	17.93
Brunei Darussalam	Boys	2012	Average	18.36
Brunei Darussalam	Boys	2013	Average	18.80
Brunei Darussalam	Boys	2014	Average	19.24
Brunei Darussalam	Boys	2015	Average	19.69
Brunei Darussalam	Boys	2016	Average	20.14
Brunei Darussalam	Girls	1975	12	5.49
Brunei Darussalam	Girls	1976	12	5.52
Brunei Darussalam	Girls	1977	12	5.54
Brunei Darussalam	Girls	1978	12	5.56
Brunei Darussalam	Girls	1979	12	5.57
Brunei Darussalam	Girls	1980	12	5.59
Brunei Darussalam	Girls	1981	12	5.61
Brunei Darussalam	Girls	1982	12	5.64
Brunei Darussalam	Girls	1983	12	5.67
Brunei Darussalam	Girls	1984	12	5.71
Brunei Darussalam	Girls	1985	12	5.75
Brunei Darussalam	Girls	1986	12	5.80
Brunei Darussalam	Girls	1987	12	5.86
Brunei Darussalam	Girls	1988	12	5.93
Brunei Darussalam	Girls	1989	12	6.00
Brunei Darussalam	Girls	1990	12	6.07
Brunei Darussalam	Girls	1991	12	6.15
Brunei Darussalam	Girls	1992	12	6.23
Brunei Darussalam	Girls	1993	12	6.32
Brunei Darussalam	Girls	1994	12	6.41
Brunei Darussalam	Girls	1995	12	6.50
Brunei Darussalam	Girls	1996	12	6.59
Brunei Darussalam	Girls	1997	12	6.69
Brunei Darussalam	Girls	1998	12	6.80
Brunei Darussalam	Girls	1999	12	6.91
Brunei Darussalam	Girls	2000	12	7.02
Brunei Darussalam	Girls	2001	12	7.14

Brunei Darussalam	Girls	2002	12	7.26
Brunei Darussalam	Girls	2003	12	7.38
Brunei Darussalam	Girls	2004	12	7.51
Brunei Darussalam	Girls	2005	12	7.64
Brunei Darussalam	Girls	2006	12	7.78
Brunei Darussalam	Girls	2007	12	7.91
Brunei Darussalam	Girls	2008	12	8.05
Brunei Darussalam	Girls	2009	12	8.19
Brunei Darussalam	Girls	2010	12	8.34
Brunei Darussalam	Girls	2011	12	8.49
Brunei Darussalam	Girls	2012	12	8.63
Brunei Darussalam	Girls	2013	12	8.79
Brunei Darussalam	Girls	2014	12	8.94
Brunei Darussalam	Girls	2015	12	9.10
Brunei Darussalam	Girls	2016	12	9.26
Brunei Darussalam	Girls	1975	13	6.81
Brunei Darussalam	Girls	1976	13	6.84
Brunei Darussalam	Girls	1977	13	6.87
Brunei Darussalam	Girls	1978	13	6.89
Brunei Darussalam	Girls	1979	13	6.91
Brunei Darussalam	Girls	1980	13	6.93
Brunei Darussalam	Girls	1981	13	6.95
Brunei Darussalam	Girls	1982	13	6.98
Brunei Darussalam	Girls	1983	13	7.02
Brunei Darussalam	Girls	1984	13	7.06
Brunei Darussalam	Girls	1985	13	7.11
Brunei Darussalam	Girls	1986	13	7.17
Brunei Darussalam	Girls	1987	13	7.23
Brunei Darussalam	Girls	1988	13	7.31
Brunei Darussalam	Girls	1989	13	7.38
Brunei Darussalam	Girls	1990	13	7.47
Brunei Darussalam	Girls	1991	13	7.56
Brunei Darussalam	Girls	1992	13	7.65
Brunei Darussalam	Girls	1993	13	7.74
Brunei Darussalam	Girls	1994	13	7.84
Brunei Darussalam	Girls	1995	13	7.95
Brunei Darussalam	Girls	1996	13	8.05
Brunei Darussalam	Girls	1997	13	8.17
Brunei Darussalam	Girls	1998	13	8.28
Brunei Darussalam	Girls	1999	13	8.40
Brunei Darussalam	Girls	2000	13	8.53
Brunei Darussalam	Girls	2001	13	8.66
Brunei Darussalam	Girls	2002	13	8.80
Brunei Darussalam	Girls	2003	13	8.94
Brunei Darussalam	Girls	2004	13	9.08
Brunei Darussalam	Girls	2005	13	9.23
Brunei Darussalam	Girls	2006	13	9.38
Brunei Darussalam	Girls	2007	13	9.54
Brunei Darussalam	Girls	2008	13	9.69
Brunei Darussalam	Girls	2009	13	9.86
Brunei Darussalam	Girls	2010	13	10.02
Brunei Darussalam	Girls	2011	13	10.19

Brunei Darussalam	Girls	2012	13	10.36
Brunei Darussalam	Girls	2013	13	10.53
Brunei Darussalam	Girls	2014	13	10.71
Brunei Darussalam	Girls	2015	13	10.89
Brunei Darussalam	Girls	2016	13	11.08
Brunei Darussalam	Girls	1975	14	8.24
Brunei Darussalam	Girls	1976	14	8.28
Brunei Darussalam	Girls	1977	14	8.31
Brunei Darussalam	Girls	1978	14	8.33
Brunei Darussalam	Girls	1979	14	8.35
Brunei Darussalam	Girls	1980	14	8.37
Brunei Darussalam	Girls	1981	14	8.39
Brunei Darussalam	Girls	1982	14	8.43
Brunei Darussalam	Girls	1983	14	8.47
Brunei Darussalam	Girls	1984	14	8.51
Brunei Darussalam	Girls	1985	14	8.57
Brunei Darussalam	Girls	1986	14	8.63
Brunei Darussalam	Girls	1987	14	8.70
Brunei Darussalam	Girls	1988	14	8.78
Brunei Darussalam	Girls	1989	14	8.86
Brunei Darussalam	Girls	1990	14	8.95
Brunei Darussalam	Girls	1991	14	9.04
Brunei Darussalam	Girls	1992	14	9.14
Brunei Darussalam	Girls	1993	14	9.24
Brunei Darussalam	Girls	1994	14	9.35
Brunei Darussalam	Girls	1995	14	9.46
Brunei Darussalam	Girls	1996	14	9.57
Brunei Darussalam	Girls	1997	14	9.69
Brunei Darussalam	Girls	1998	14	9.82
Brunei Darussalam	Girls	1999	14	9.95
Brunei Darussalam	Girls	2000	14	10.08
Brunei Darussalam	Girls	2001	14	10.22
Brunei Darussalam	Girls	2002	14	10.37
Brunei Darussalam	Girls	2003	14	10.52
Brunei Darussalam	Girls	2004	14	10.67
Brunei Darussalam	Girls	2005	14	10.83
Brunei Darussalam	Girls	2006	14	10.99
Brunei Darussalam	Girls	2007	14	11.16
Brunei Darussalam	Girls	2008	14	11.33
Brunei Darussalam	Girls	2009	14	11.50
Brunei Darussalam	Girls	2010	14	11.68
Brunei Darussalam	Girls	2011	14	11.86
Brunei Darussalam	Girls	2012	14	12.05
Brunei Darussalam	Girls	2013	14	12.23
Brunei Darussalam	Girls	2014	14	12.43
Brunei Darussalam	Girls	2015	14	12.63
Brunei Darussalam	Girls	2016	14	12.83
Brunei Darussalam	Girls	1975	15	9.58
Brunei Darussalam	Girls	1976	15	9.62
Brunei Darussalam	Girls	1977	15	9.65
Brunei Darussalam	Girls	1978	15	9.68
Brunei Darussalam	Girls	1979	15	9.70

Brunei Darussalam	Girls	1980	15	9.72
Brunei Darussalam	Girls	1981	15	9.74
Brunei Darussalam	Girls	1982	15	9.78
Brunei Darussalam	Girls	1983	15	9.82
Brunei Darussalam	Girls	1984	15	9.87
Brunei Darussalam	Girls	1985	15	9.92
Brunei Darussalam	Girls	1986	15	9.99
Brunei Darussalam	Girls	1987	15	10.06
Brunei Darussalam	Girls	1988	15	10.14
Brunei Darussalam	Girls	1989	15	10.23
Brunei Darussalam	Girls	1990	15	10.32
Brunei Darussalam	Girls	1991	15	10.42
Brunei Darussalam	Girls	1992	15	10.52
Brunei Darussalam	Girls	1993	15	10.62
Brunei Darussalam	Girls	1994	15	10.73
Brunei Darussalam	Girls	1995	15	10.85
Brunei Darussalam	Girls	1996	15	10.97
Brunei Darussalam	Girls	1997	15	11.09
Brunei Darussalam	Girls	1998	15	11.22
Brunei Darussalam	Girls	1999	15	11.35
Brunei Darussalam	Girls	2000	15	11.49
Brunei Darussalam	Girls	2001	15	11.64
Brunei Darussalam	Girls	2002	15	11.79
Brunei Darussalam	Girls	2003	15	11.94
Brunei Darussalam	Girls	2004	15	12.10
Brunei Darussalam	Girls	2005	15	12.27
Brunei Darussalam	Girls	2006	15	12.44
Brunei Darussalam	Girls	2007	15	12.61
Brunei Darussalam	Girls	2008	15	12.78
Brunei Darussalam	Girls	2009	15	12.97
Brunei Darussalam	Girls	2010	15	13.15
Brunei Darussalam	Girls	2011	15	13.34
Brunei Darussalam	Girls	2012	15	13.53
Brunei Darussalam	Girls	2013	15	13.73
Brunei Darussalam	Girls	2014	15	13.93
Brunei Darussalam	Girls	2015	15	14.14
Brunei Darussalam	Girls	2016	15	14.35
Brunei Darussalam	Girls	1975	16	10.67
Brunei Darussalam	Girls	1976	16	10.71
Brunei Darussalam	Girls	1977	16	10.74
Brunei Darussalam	Girls	1978	16	10.77
Brunei Darussalam	Girls	1979	16	10.79
Brunei Darussalam	Girls	1980	16	10.81
Brunei Darussalam	Girls	1981	16	10.83
Brunei Darussalam	Girls	1982	16	10.87
Brunei Darussalam	Girls	1983	16	10.91
Brunei Darussalam	Girls	1984	16	10.96
Brunei Darussalam	Girls	1985	16	11.02
Brunei Darussalam	Girls	1986	16	11.09
Brunei Darussalam	Girls	1987	16	11.16
Brunei Darussalam	Girls	1988	16	11.25
Brunei Darussalam	Girls	1989	16	11.34

Brunei Darussalam	Girls	1990	16	11.43
Brunei Darussalam	Girls	1991	16	11.53
Brunei Darussalam	Girls	1992	16	11.64
Brunei Darussalam	Girls	1993	16	11.75
Brunei Darussalam	Girls	1994	16	11.86
Brunei Darussalam	Girls	1995	16	11.98
Brunei Darussalam	Girls	1996	16	12.10
Brunei Darussalam	Girls	1997	16	12.23
Brunei Darussalam	Girls	1998	16	12.36
Brunei Darussalam	Girls	1999	16	12.50
Brunei Darussalam	Girls	2000	16	12.64
Brunei Darussalam	Girls	2001	16	12.79
Brunei Darussalam	Girls	2002	16	12.95
Brunei Darussalam	Girls	2003	16	13.11
Brunei Darussalam	Girls	2004	16	13.27
Brunei Darussalam	Girls	2005	16	13.44
Brunei Darussalam	Girls	2006	16	13.62
Brunei Darussalam	Girls	2007	16	13.79
Brunei Darussalam	Girls	2008	16	13.98
Brunei Darussalam	Girls	2009	16	14.16
Brunei Darussalam	Girls	2010	16	14.35
Brunei Darussalam	Girls	2011	16	14.55
Brunei Darussalam	Girls	2012	16	14.75
Brunei Darussalam	Girls	2013	16	14.95
Brunei Darussalam	Girls	2014	16	15.16
Brunei Darussalam	Girls	2015	16	15.38
Brunei Darussalam	Girls	2016	16	15.60
Brunei Darussalam	Girls	1975	17	11.45
Brunei Darussalam	Girls	1976	17	11.49
Brunei Darussalam	Girls	1977	17	11.53
Brunei Darussalam	Girls	1978	17	11.56
Brunei Darussalam	Girls	1979	17	11.58
Brunei Darussalam	Girls	1980	17	11.60
Brunei Darussalam	Girls	1981	17	11.63
Brunei Darussalam	Girls	1982	17	11.67
Brunei Darussalam	Girls	1983	17	11.71
Brunei Darussalam	Girls	1984	17	11.77
Brunei Darussalam	Girls	1985	17	11.83
Brunei Darussalam	Girls	1986	17	11.90
Brunei Darussalam	Girls	1987	17	11.98
Brunei Darussalam	Girls	1988	17	12.07
Brunei Darussalam	Girls	1989	17	12.16
Brunei Darussalam	Girls	1990	17	12.26
Brunei Darussalam	Girls	1991	17	12.37
Brunei Darussalam	Girls	1992	17	12.48
Brunei Darussalam	Girls	1993	17	12.60
Brunei Darussalam	Girls	1994	17	12.72
Brunei Darussalam	Girls	1995	17	12.84
Brunei Darussalam	Girls	1996	17	12.97
Brunei Darussalam	Girls	1997	17	13.11
Brunei Darussalam	Girls	1998	17	13.25
Brunei Darussalam	Girls	1999	17	13.40

Brunei Darussalam	Girls	2000	17	13.55
Brunei Darussalam	Girls	2001	17	13.71
Brunei Darussalam	Girls	2002	17	13.87
Brunei Darussalam	Girls	2003	17	14.04
Brunei Darussalam	Girls	2004	17	14.22
Brunei Darussalam	Girls	2005	17	14.40
Brunei Darussalam	Girls	2006	17	14.58
Brunei Darussalam	Girls	2007	17	14.77
Brunei Darussalam	Girls	2008	17	14.96
Brunei Darussalam	Girls	2009	17	15.16
Brunei Darussalam	Girls	2010	17	15.36
Brunei Darussalam	Girls	2011	17	15.57
Brunei Darussalam	Girls	2012	17	15.78
Brunei Darussalam	Girls	2013	17	15.99
Brunei Darussalam	Girls	2014	17	16.21
Brunei Darussalam	Girls	2015	17	16.44
Brunei Darussalam	Girls	2016	17	16.67
Brunei Darussalam	Girls	1975	18	12.01
Brunei Darussalam	Girls	1976	18	12.06
Brunei Darussalam	Girls	1977	18	12.10
Brunei Darussalam	Girls	1978	18	12.13
Brunei Darussalam	Girls	1979	18	12.15
Brunei Darussalam	Girls	1980	18	12.18
Brunei Darussalam	Girls	1981	18	12.21
Brunei Darussalam	Girls	1982	18	12.25
Brunei Darussalam	Girls	1983	18	12.30
Brunei Darussalam	Girls	1984	18	12.36
Brunei Darussalam	Girls	1985	18	12.43
Brunei Darussalam	Girls	1986	18	12.51
Brunei Darussalam	Girls	1987	18	12.60
Brunei Darussalam	Girls	1988	18	12.70
Brunei Darussalam	Girls	1989	18	12.81
Brunei Darussalam	Girls	1990	18	12.92
Brunei Darussalam	Girls	1991	18	13.04
Brunei Darussalam	Girls	1992	18	13.17
Brunei Darussalam	Girls	1993	18	13.30
Brunei Darussalam	Girls	1994	18	13.43
Brunei Darussalam	Girls	1995	18	13.57
Brunei Darussalam	Girls	1996	18	13.72
Brunei Darussalam	Girls	1997	18	13.87
Brunei Darussalam	Girls	1998	18	14.03
Brunei Darussalam	Girls	1999	18	14.19
Brunei Darussalam	Girls	2000	18	14.36
Brunei Darussalam	Girls	2001	18	14.54
Brunei Darussalam	Girls	2002	18	14.72
Brunei Darussalam	Girls	2003	18	14.91
Brunei Darussalam	Girls	2004	18	15.11
Brunei Darussalam	Girls	2005	18	15.31
Brunei Darussalam	Girls	2006	18	15.51
Brunei Darussalam	Girls	2007	18	15.72
Brunei Darussalam	Girls	2008	18	15.94
Brunei Darussalam	Girls	2009	18	16.15

Brunei Darussalam	Girls	2010	18	16.38
Brunei Darussalam	Girls	2011	18	16.60
Brunei Darussalam	Girls	2012	18	16.83
Brunei Darussalam	Girls	2013	18	17.07
Brunei Darussalam	Girls	2014	18	17.31
Brunei Darussalam	Girls	2015	18	17.55
Brunei Darussalam	Girls	2016	18	17.81
Brunei Darussalam	Girls	1975	Average	9.18
Brunei Darussalam	Girls	1976	Average	9.22
Brunei Darussalam	Girls	1977	Average	9.25
Brunei Darussalam	Girls	1978	Average	9.27
Brunei Darussalam	Girls	1979	Average	9.29
Brunei Darussalam	Girls	1980	Average	9.31
Brunei Darussalam	Girls	1981	Average	9.34
Brunei Darussalam	Girls	1982	Average	9.37
Brunei Darussalam	Girls	1983	Average	9.41
Brunei Darussalam	Girls	1984	Average	9.46
Brunei Darussalam	Girls	1985	Average	9.52
Brunei Darussalam	Girls	1986	Average	9.58
Brunei Darussalam	Girls	1987	Average	9.66
Brunei Darussalam	Girls	1988	Average	9.74
Brunei Darussalam	Girls	1989	Average	9.82
Brunei Darussalam	Girls	1990	Average	9.92
Brunei Darussalam	Girls	1991	Average	10.02
Brunei Darussalam	Girls	1992	Average	10.12
Brunei Darussalam	Girls	1993	Average	10.22
Brunei Darussalam	Girls	1994	Average	10.33
Brunei Darussalam	Girls	1995	Average	10.45
Brunei Darussalam	Girls	1996	Average	10.57
Brunei Darussalam	Girls	1997	Average	10.69
Brunei Darussalam	Girls	1998	Average	10.82
Brunei Darussalam	Girls	1999	Average	10.96
Brunei Darussalam	Girls	2000	Average	11.10
Brunei Darussalam	Girls	2001	Average	11.24
Brunei Darussalam	Girls	2002	Average	11.39
Brunei Darussalam	Girls	2003	Average	11.55
Brunei Darussalam	Girls	2004	Average	11.71
Brunei Darussalam	Girls	2005	Average	11.87
Brunei Darussalam	Girls	2006	Average	12.04
Brunei Darussalam	Girls	2007	Average	12.22
Brunei Darussalam	Girls	2008	Average	12.39
Brunei Darussalam	Girls	2009	Average	12.57
Brunei Darussalam	Girls	2010	Average	12.75
Brunei Darussalam	Girls	2011	Average	12.94
Brunei Darussalam	Girls	2012	Average	13.13
Brunei Darussalam	Girls	2013	Average	13.33
Brunei Darussalam	Girls	2014	Average	13.53
Brunei Darussalam	Girls	2015	Average	13.73
Brunei Darussalam	Girls	2016	Average	13.94
Bulgaria	Boys	1975	12	6.55
Bulgaria	Boys	1976	12	6.66
Bulgaria	Boys	1977	12	6.77

Bulgaria	Boys	1978	12	6.89
Bulgaria	Boys	1979	12	7.01
Bulgaria	Boys	1980	12	7.13
Bulgaria	Boys	1981	12	7.26
Bulgaria	Boys	1982	12	7.38
Bulgaria	Boys	1983	12	7.51
Bulgaria	Boys	1984	12	7.63
Bulgaria	Boys	1985	12	7.76
Bulgaria	Boys	1986	12	7.89
Bulgaria	Boys	1987	12	8.02
Bulgaria	Boys	1988	12	8.15
Bulgaria	Boys	1989	12	8.28
Bulgaria	Boys	1990	12	8.42
Bulgaria	Boys	1991	12	8.55
Bulgaria	Boys	1992	12	8.69
Bulgaria	Boys	1993	12	8.83
Bulgaria	Boys	1994	12	8.98
Bulgaria	Boys	1995	12	9.12
Bulgaria	Boys	1996	12	9.27
Bulgaria	Boys	1997	12	9.43
Bulgaria	Boys	1998	12	9.59
Bulgaria	Boys	1999	12	9.75
Bulgaria	Boys	2000	12	9.92
Bulgaria	Boys	2001	12	10.10
Bulgaria	Boys	2002	12	10.28
Bulgaria	Boys	2003	12	10.47
Bulgaria	Boys	2004	12	10.66
Bulgaria	Boys	2005	12	10.86
Bulgaria	Boys	2006	12	11.06
Bulgaria	Boys	2007	12	11.28
Bulgaria	Boys	2008	12	11.49
Bulgaria	Boys	2009	12	11.71
Bulgaria	Boys	2010	12	11.93
Bulgaria	Boys	2011	12	12.16
Bulgaria	Boys	2012	12	12.39
Bulgaria	Boys	2013	12	12.62
Bulgaria	Boys	2014	12	12.85
Bulgaria	Boys	2015	12	13.09
Bulgaria	Boys	2016	12	13.32
Bulgaria	Boys	1975	13	8.10
Bulgaria	Boys	1976	13	8.23
Bulgaria	Boys	1977	13	8.37
Bulgaria	Boys	1978	13	8.50
Bulgaria	Boys	1979	13	8.64
Bulgaria	Boys	1980	13	8.79
Bulgaria	Boys	1981	13	8.94
Bulgaria	Boys	1982	13	9.08
Bulgaria	Boys	1983	13	9.23
Bulgaria	Boys	1984	13	9.38
Bulgaria	Boys	1985	13	9.53
Bulgaria	Boys	1986	13	9.68
Bulgaria	Boys	1987	13	9.83

Bulgaria	Boys	1988	13	9.98
Bulgaria	Boys	1989	13	10.13
Bulgaria	Boys	1990	13	10.29
Bulgaria	Boys	1991	13	10.45
Bulgaria	Boys	1992	13	10.61
Bulgaria	Boys	1993	13	10.78
Bulgaria	Boys	1994	13	10.95
Bulgaria	Boys	1995	13	11.12
Bulgaria	Boys	1996	13	11.30
Bulgaria	Boys	1997	13	11.48
Bulgaria	Boys	1998	13	11.67
Bulgaria	Boys	1999	13	11.87
Bulgaria	Boys	2000	13	12.07
Bulgaria	Boys	2001	13	12.27
Bulgaria	Boys	2002	13	12.49
Bulgaria	Boys	2003	13	12.71
Bulgaria	Boys	2004	13	12.95
Bulgaria	Boys	2005	13	13.19
Bulgaria	Boys	2006	13	13.43
Bulgaria	Boys	2007	13	13.69
Bulgaria	Boys	2008	13	13.95
Bulgaria	Boys	2009	13	14.21
Bulgaria	Boys	2010	13	14.49
Bulgaria	Boys	2011	13	14.76
Bulgaria	Boys	2012	13	15.04
Bulgaria	Boys	2013	13	15.33
Bulgaria	Boys	2014	13	15.62
Bulgaria	Boys	2015	13	15.91
Bulgaria	Boys	2016	13	16.20
Bulgaria	Boys	1975	14	9.98
Bulgaria	Boys	1976	14	10.13
Bulgaria	Boys	1977	14	10.27
Bulgaria	Boys	1978	14	10.43
Bulgaria	Boys	1979	14	10.58
Bulgaria	Boys	1980	14	10.75
Bulgaria	Boys	1981	14	10.91
Bulgaria	Boys	1982	14	11.07
Bulgaria	Boys	1983	14	11.23
Bulgaria	Boys	1984	14	11.40
Bulgaria	Boys	1985	14	11.56
Bulgaria	Boys	1986	14	11.73
Bulgaria	Boys	1987	14	11.90
Bulgaria	Boys	1988	14	12.06
Bulgaria	Boys	1989	14	12.24
Bulgaria	Boys	1990	14	12.41
Bulgaria	Boys	1991	14	12.59
Bulgaria	Boys	1992	14	12.77
Bulgaria	Boys	1993	14	12.95
Bulgaria	Boys	1994	14	13.14
Bulgaria	Boys	1995	14	13.33
Bulgaria	Boys	1996	14	13.53
Bulgaria	Boys	1997	14	13.73

Bulgaria	Boys	1998	14	13.94
Bulgaria	Boys	1999	14	14.16
Bulgaria	Boys	2000	14	14.38
Bulgaria	Boys	2001	14	14.61
Bulgaria	Boys	2002	14	14.86
Bulgaria	Boys	2003	14	15.11
Bulgaria	Boys	2004	14	15.37
Bulgaria	Boys	2005	14	15.64
Bulgaria	Boys	2006	14	15.92
Bulgaria	Boys	2007	14	16.21
Bulgaria	Boys	2008	14	16.50
Bulgaria	Boys	2009	14	16.81
Bulgaria	Boys	2010	14	17.12
Bulgaria	Boys	2011	14	17.44
Bulgaria	Boys	2012	14	17.76
Bulgaria	Boys	2013	14	18.09
Bulgaria	Boys	2014	14	18.43
Bulgaria	Boys	2015	14	18.77
Bulgaria	Boys	2016	14	19.11
Bulgaria	Boys	1975	15	12.09
Bulgaria	Boys	1976	15	12.24
Bulgaria	Boys	1977	15	12.40
Bulgaria	Boys	1978	15	12.56
Bulgaria	Boys	1979	15	12.73
Bulgaria	Boys	1980	15	12.90
Bulgaria	Boys	1981	15	13.07
Bulgaria	Boys	1982	15	13.25
Bulgaria	Boys	1983	15	13.42
Bulgaria	Boys	1984	15	13.59
Bulgaria	Boys	1985	15	13.77
Bulgaria	Boys	1986	15	13.94
Bulgaria	Boys	1987	15	14.12
Bulgaria	Boys	1988	15	14.29
Bulgaria	Boys	1989	15	14.47
Bulgaria	Boys	1990	15	14.66
Bulgaria	Boys	1991	15	14.85
Bulgaria	Boys	1992	15	15.03
Bulgaria	Boys	1993	15	15.23
Bulgaria	Boys	1994	15	15.42
Bulgaria	Boys	1995	15	15.63
Bulgaria	Boys	1996	15	15.84
Bulgaria	Boys	1997	15	16.05
Bulgaria	Boys	1998	15	16.27
Bulgaria	Boys	1999	15	16.50
Bulgaria	Boys	2000	15	16.73
Bulgaria	Boys	2001	15	16.98
Bulgaria	Boys	2002	15	17.24
Bulgaria	Boys	2003	15	17.50
Bulgaria	Boys	2004	15	17.78
Bulgaria	Boys	2005	15	18.07
Bulgaria	Boys	2006	15	18.37
Bulgaria	Boys	2007	15	18.68

Bulgaria	Boys	2008	15	18.99
Bulgaria	Boys	2009	15	19.32
Bulgaria	Boys	2010	15	19.66
Bulgaria	Boys	2011	15	20.00
Bulgaria	Boys	2012	15	20.36
Bulgaria	Boys	2013	15	20.72
Bulgaria	Boys	2014	15	21.09
Bulgaria	Boys	2015	15	21.46
Bulgaria	Boys	2016	15	21.84
Bulgaria	Boys	1975	16	14.35
Bulgaria	Boys	1976	16	14.51
Bulgaria	Boys	1977	16	14.67
Bulgaria	Boys	1978	16	14.84
Bulgaria	Boys	1979	16	15.01
Bulgaria	Boys	1980	16	15.19
Bulgaria	Boys	1981	16	15.37
Bulgaria	Boys	1982	16	15.54
Bulgaria	Boys	1983	16	15.72
Bulgaria	Boys	1984	16	15.89
Bulgaria	Boys	1985	16	16.07
Bulgaria	Boys	1986	16	16.25
Bulgaria	Boys	1987	16	16.43
Bulgaria	Boys	1988	16	16.61
Bulgaria	Boys	1989	16	16.80
Bulgaria	Boys	1990	16	16.99
Bulgaria	Boys	1991	16	17.18
Bulgaria	Boys	1992	16	17.37
Bulgaria	Boys	1993	16	17.57
Bulgaria	Boys	1994	16	17.77
Bulgaria	Boys	1995	16	17.97
Bulgaria	Boys	1996	16	18.19
Bulgaria	Boys	1997	16	18.41
Bulgaria	Boys	1998	16	18.63
Bulgaria	Boys	1999	16	18.87
Bulgaria	Boys	2000	16	19.10
Bulgaria	Boys	2001	16	19.36
Bulgaria	Boys	2002	16	19.62
Bulgaria	Boys	2003	16	19.89
Bulgaria	Boys	2004	16	20.18
Bulgaria	Boys	2005	16	20.47
Bulgaria	Boys	2006	16	20.78
Bulgaria	Boys	2007	16	21.10
Bulgaria	Boys	2008	16	21.43
Bulgaria	Boys	2009	16	21.77
Bulgaria	Boys	2010	16	22.12
Bulgaria	Boys	2011	16	22.48
Bulgaria	Boys	2012	16	22.85
Bulgaria	Boys	2013	16	23.23
Bulgaria	Boys	2014	16	23.61
Bulgaria	Boys	2015	16	24.01
Bulgaria	Boys	2016	16	24.41
Bulgaria	Boys	1975	17	16.68

Bulgaria	Boys	1976	17	16.84
Bulgaria	Boys	1977	17	17.01
Bulgaria	Boys	1978	17	17.18
Bulgaria	Boys	1979	17	17.36
Bulgaria	Boys	1980	17	17.54
Bulgaria	Boys	1981	17	17.72
Bulgaria	Boys	1982	17	17.90
Bulgaria	Boys	1983	17	18.08
Bulgaria	Boys	1984	17	18.26
Bulgaria	Boys	1985	17	18.44
Bulgaria	Boys	1986	17	18.62
Bulgaria	Boys	1987	17	18.80
Bulgaria	Boys	1988	17	18.99
Bulgaria	Boys	1989	17	19.17
Bulgaria	Boys	1990	17	19.36
Bulgaria	Boys	1991	17	19.56
Bulgaria	Boys	1992	17	19.75
Bulgaria	Boys	1993	17	19.95
Bulgaria	Boys	1994	17	20.16
Bulgaria	Boys	1995	17	20.37
Bulgaria	Boys	1996	17	20.58
Bulgaria	Boys	1997	17	20.80
Bulgaria	Boys	1998	17	21.03
Bulgaria	Boys	1999	17	21.27
Bulgaria	Boys	2000	17	21.51
Bulgaria	Boys	2001	17	21.77
Bulgaria	Boys	2002	17	22.04
Bulgaria	Boys	2003	17	22.31
Bulgaria	Boys	2004	17	22.60
Bulgaria	Boys	2005	17	22.90
Bulgaria	Boys	2006	17	23.22
Bulgaria	Boys	2007	17	23.54
Bulgaria	Boys	2008	17	23.88
Bulgaria	Boys	2009	17	24.23
Bulgaria	Boys	2010	17	24.59
Bulgaria	Boys	2011	17	24.96
Bulgaria	Boys	2012	17	25.33
Bulgaria	Boys	2013	17	25.72
Bulgaria	Boys	2014	17	26.12
Bulgaria	Boys	2015	17	26.53
Bulgaria	Boys	2016	17	26.94
Bulgaria	Boys	1975	18	19.02
Bulgaria	Boys	1976	18	19.20
Bulgaria	Boys	1977	18	19.37
Bulgaria	Boys	1978	18	19.55
Bulgaria	Boys	1979	18	19.73
Bulgaria	Boys	1980	18	19.92
Bulgaria	Boys	1981	18	20.11
Bulgaria	Boys	1982	18	20.30
Bulgaria	Boys	1983	18	20.48
Bulgaria	Boys	1984	18	20.67
Bulgaria	Boys	1985	18	20.86

Bulgaria	Boys	1986	18	21.05
Bulgaria	Boys	1987	18	21.24
Bulgaria	Boys	1988	18	21.43
Bulgaria	Boys	1989	18	21.62
Bulgaria	Boys	1990	18	21.82
Bulgaria	Boys	1991	18	22.02
Bulgaria	Boys	1992	18	22.22
Bulgaria	Boys	1993	18	22.43
Bulgaria	Boys	1994	18	22.64
Bulgaria	Boys	1995	18	22.85
Bulgaria	Boys	1996	18	23.07
Bulgaria	Boys	1997	18	23.30
Bulgaria	Boys	1998	18	23.54
Bulgaria	Boys	1999	18	23.78
Bulgaria	Boys	2000	18	24.03
Bulgaria	Boys	2001	18	24.30
Bulgaria	Boys	2002	18	24.57
Bulgaria	Boys	2003	18	24.86
Bulgaria	Boys	2004	18	25.16
Bulgaria	Boys	2005	18	25.47
Bulgaria	Boys	2006	18	25.79
Bulgaria	Boys	2007	18	26.12
Bulgaria	Boys	2008	18	26.47
Bulgaria	Boys	2009	18	26.83
Bulgaria	Boys	2010	18	27.20
Bulgaria	Boys	2011	18	27.58
Bulgaria	Boys	2012	18	27.96
Bulgaria	Boys	2013	18	28.36
Bulgaria	Boys	2014	18	28.77
Bulgaria	Boys	2015	18	29.19
Bulgaria	Boys	2016	18	29.62
Bulgaria	Boys	1975	Average	12.40
Bulgaria	Boys	1976	Average	12.54
Bulgaria	Boys	1977	Average	12.69
Bulgaria	Boys	1978	Average	12.85
Bulgaria	Boys	1979	Average	13.01
Bulgaria	Boys	1980	Average	13.17
Bulgaria	Boys	1981	Average	13.34
Bulgaria	Boys	1982	Average	13.50
Bulgaria	Boys	1983	Average	13.67
Bulgaria	Boys	1984	Average	13.83
Bulgaria	Boys	1985	Average	14.00
Bulgaria	Boys	1986	Average	14.16
Bulgaria	Boys	1987	Average	14.33
Bulgaria	Boys	1988	Average	14.50
Bulgaria	Boys	1989	Average	14.67
Bulgaria	Boys	1990	Average	14.85
Bulgaria	Boys	1991	Average	15.03
Bulgaria	Boys	1992	Average	15.21
Bulgaria	Boys	1993	Average	15.39
Bulgaria	Boys	1994	Average	15.58
Bulgaria	Boys	1995	Average	15.77

Bulgaria	Boys	1996	Average	15.97
Bulgaria	Boys	1997	Average	16.17
Bulgaria	Boys	1998	Average	16.38
Bulgaria	Boys	1999	Average	16.60
Bulgaria	Boys	2000	Average	16.82
Bulgaria	Boys	2001	Average	17.06
Bulgaria	Boys	2002	Average	17.30
Bulgaria	Boys	2003	Average	17.55
Bulgaria	Boys	2004	Average	17.81
Bulgaria	Boys	2005	Average	18.08
Bulgaria	Boys	2006	Average	18.37
Bulgaria	Boys	2007	Average	18.66
Bulgaria	Boys	2008	Average	18.96
Bulgaria	Boys	2009	Average	19.27
Bulgaria	Boys	2010	Average	19.59
Bulgaria	Boys	2011	Average	19.91
Bulgaria	Boys	2012	Average	20.24
Bulgaria	Boys	2013	Average	20.58
Bulgaria	Boys	2014	Average	20.93
Bulgaria	Boys	2015	Average	21.28
Bulgaria	Boys	2016	Average	21.64
Bulgaria	Girls	1975	12	5.96
Bulgaria	Girls	1976	12	6.03
Bulgaria	Girls	1977	12	6.10
Bulgaria	Girls	1978	12	6.18
Bulgaria	Girls	1979	12	6.26
Bulgaria	Girls	1980	12	6.33
Bulgaria	Girls	1981	12	6.41
Bulgaria	Girls	1982	12	6.49
Bulgaria	Girls	1983	12	6.56
Bulgaria	Girls	1984	12	6.64
Bulgaria	Girls	1985	12	6.71
Bulgaria	Girls	1986	12	6.79
Bulgaria	Girls	1987	12	6.86
Bulgaria	Girls	1988	12	6.93
Bulgaria	Girls	1989	12	7.01
Bulgaria	Girls	1990	12	7.08
Bulgaria	Girls	1991	12	7.16
Bulgaria	Girls	1992	12	7.24
Bulgaria	Girls	1993	12	7.31
Bulgaria	Girls	1994	12	7.39
Bulgaria	Girls	1995	12	7.48
Bulgaria	Girls	1996	12	7.56
Bulgaria	Girls	1997	12	7.64
Bulgaria	Girls	1998	12	7.73
Bulgaria	Girls	1999	12	7.82
Bulgaria	Girls	2000	12	7.91
Bulgaria	Girls	2001	12	8.01
Bulgaria	Girls	2002	12	8.11
Bulgaria	Girls	2003	12	8.21
Bulgaria	Girls	2004	12	8.32
Bulgaria	Girls	2005	12	8.43

Bulgaria	Girls	2006	12	8.55
Bulgaria	Girls	2007	12	8.66
Bulgaria	Girls	2008	12	8.78
Bulgaria	Girls	2009	12	8.90
Bulgaria	Girls	2010	12	9.03
Bulgaria	Girls	2011	12	9.15
Bulgaria	Girls	2012	12	9.28
Bulgaria	Girls	2013	12	9.41
Bulgaria	Girls	2014	12	9.54
Bulgaria	Girls	2015	12	9.67
Bulgaria	Girls	2016	12	9.80
Bulgaria	Girls	1975	13	7.40
Bulgaria	Girls	1976	13	7.48
Bulgaria	Girls	1977	13	7.57
Bulgaria	Girls	1978	13	7.65
Bulgaria	Girls	1979	13	7.74
Bulgaria	Girls	1980	13	7.83
Bulgaria	Girls	1981	13	7.92
Bulgaria	Girls	1982	13	8.01
Bulgaria	Girls	1983	13	8.09
Bulgaria	Girls	1984	13	8.18
Bulgaria	Girls	1985	13	8.26
Bulgaria	Girls	1986	13	8.35
Bulgaria	Girls	1987	13	8.43
Bulgaria	Girls	1988	13	8.52
Bulgaria	Girls	1989	13	8.60
Bulgaria	Girls	1990	13	8.69
Bulgaria	Girls	1991	13	8.77
Bulgaria	Girls	1992	13	8.86
Bulgaria	Girls	1993	13	8.95
Bulgaria	Girls	1994	13	9.04
Bulgaria	Girls	1995	13	9.13
Bulgaria	Girls	1996	13	9.22
Bulgaria	Girls	1997	13	9.32
Bulgaria	Girls	1998	13	9.42
Bulgaria	Girls	1999	13	9.52
Bulgaria	Girls	2000	13	9.62
Bulgaria	Girls	2001	13	9.73
Bulgaria	Girls	2002	13	9.84
Bulgaria	Girls	2003	13	9.96
Bulgaria	Girls	2004	13	10.08
Bulgaria	Girls	2005	13	10.21
Bulgaria	Girls	2006	13	10.34
Bulgaria	Girls	2007	13	10.47
Bulgaria	Girls	2008	13	10.60
Bulgaria	Girls	2009	13	10.74
Bulgaria	Girls	2010	13	10.88
Bulgaria	Girls	2011	13	11.02
Bulgaria	Girls	2012	13	11.17
Bulgaria	Girls	2013	13	11.31
Bulgaria	Girls	2014	13	11.46
Bulgaria	Girls	2015	13	11.61

Bulgaria	Girls	2016	13	11.77
Bulgaria	Girls	1975	14	8.94
Bulgaria	Girls	1976	14	9.03
Bulgaria	Girls	1977	14	9.13
Bulgaria	Girls	1978	14	9.22
Bulgaria	Girls	1979	14	9.32
Bulgaria	Girls	1980	14	9.41
Bulgaria	Girls	1981	14	9.51
Bulgaria	Girls	1982	14	9.61
Bulgaria	Girls	1983	14	9.70
Bulgaria	Girls	1984	14	9.79
Bulgaria	Girls	1985	14	9.88
Bulgaria	Girls	1986	14	9.97
Bulgaria	Girls	1987	14	10.07
Bulgaria	Girls	1988	14	10.16
Bulgaria	Girls	1989	14	10.25
Bulgaria	Girls	1990	14	10.34
Bulgaria	Girls	1991	14	10.43
Bulgaria	Girls	1992	14	10.52
Bulgaria	Girls	1993	14	10.62
Bulgaria	Girls	1994	14	10.71
Bulgaria	Girls	1995	14	10.81
Bulgaria	Girls	1996	14	10.91
Bulgaria	Girls	1997	14	11.02
Bulgaria	Girls	1998	14	11.12
Bulgaria	Girls	1999	14	11.23
Bulgaria	Girls	2000	14	11.34
Bulgaria	Girls	2001	14	11.46
Bulgaria	Girls	2002	14	11.58
Bulgaria	Girls	2003	14	11.70
Bulgaria	Girls	2004	14	11.83
Bulgaria	Girls	2005	14	11.97
Bulgaria	Girls	2006	14	12.11
Bulgaria	Girls	2007	14	12.25
Bulgaria	Girls	2008	14	12.39
Bulgaria	Girls	2009	14	12.54
Bulgaria	Girls	2010	14	12.69
Bulgaria	Girls	2011	14	12.84
Bulgaria	Girls	2012	14	13.00
Bulgaria	Girls	2013	14	13.16
Bulgaria	Girls	2014	14	13.32
Bulgaria	Girls	2015	14	13.48
Bulgaria	Girls	2016	14	13.65
Bulgaria	Girls	1975	15	10.36
Bulgaria	Girls	1976	15	10.46
Bulgaria	Girls	1977	15	10.56
Bulgaria	Girls	1978	15	10.66
Bulgaria	Girls	1979	15	10.76
Bulgaria	Girls	1980	15	10.86
Bulgaria	Girls	1981	15	10.97
Bulgaria	Girls	1982	15	11.06
Bulgaria	Girls	1983	15	11.16

Bulgaria	Girls	1984	15	11.26
Bulgaria	Girls	1985	15	11.35
Bulgaria	Girls	1986	15	11.45
Bulgaria	Girls	1987	15	11.54
Bulgaria	Girls	1988	15	11.64
Bulgaria	Girls	1989	15	11.73
Bulgaria	Girls	1990	15	11.83
Bulgaria	Girls	1991	15	11.92
Bulgaria	Girls	1992	15	12.02
Bulgaria	Girls	1993	15	12.12
Bulgaria	Girls	1994	15	12.22
Bulgaria	Girls	1995	15	12.32
Bulgaria	Girls	1996	15	12.42
Bulgaria	Girls	1997	15	12.53
Bulgaria	Girls	1998	15	12.64
Bulgaria	Girls	1999	15	12.75
Bulgaria	Girls	2000	15	12.86
Bulgaria	Girls	2001	15	12.98
Bulgaria	Girls	2002	15	13.11
Bulgaria	Girls	2003	15	13.24
Bulgaria	Girls	2004	15	13.37
Bulgaria	Girls	2005	15	13.51
Bulgaria	Girls	2006	15	13.66
Bulgaria	Girls	2007	15	13.80
Bulgaria	Girls	2008	15	13.95
Bulgaria	Girls	2009	15	14.11
Bulgaria	Girls	2010	15	14.26
Bulgaria	Girls	2011	15	14.42
Bulgaria	Girls	2012	15	14.58
Bulgaria	Girls	2013	15	14.75
Bulgaria	Girls	2014	15	14.92
Bulgaria	Girls	2015	15	15.09
Bulgaria	Girls	2016	15	15.26
Bulgaria	Girls	1975	16	11.51
Bulgaria	Girls	1976	16	11.61
Bulgaria	Girls	1977	16	11.71
Bulgaria	Girls	1978	16	11.81
Bulgaria	Girls	1979	16	11.92
Bulgaria	Girls	1980	16	12.02
Bulgaria	Girls	1981	16	12.13
Bulgaria	Girls	1982	16	12.23
Bulgaria	Girls	1983	16	12.33
Bulgaria	Girls	1984	16	12.43
Bulgaria	Girls	1985	16	12.53
Bulgaria	Girls	1986	16	12.63
Bulgaria	Girls	1987	16	12.72
Bulgaria	Girls	1988	16	12.82
Bulgaria	Girls	1989	16	12.92
Bulgaria	Girls	1990	16	13.01
Bulgaria	Girls	1991	16	13.11
Bulgaria	Girls	1992	16	13.21
Bulgaria	Girls	1993	16	13.31

Bulgaria	Girls	1994	16	13.41
Bulgaria	Girls	1995	16	13.52
Bulgaria	Girls	1996	16	13.62
Bulgaria	Girls	1997	16	13.73
Bulgaria	Girls	1998	16	13.84
Bulgaria	Girls	1999	16	13.96
Bulgaria	Girls	2000	16	14.08
Bulgaria	Girls	2001	16	14.20
Bulgaria	Girls	2002	16	14.33
Bulgaria	Girls	2003	16	14.46
Bulgaria	Girls	2004	16	14.60
Bulgaria	Girls	2005	16	14.74
Bulgaria	Girls	2006	16	14.89
Bulgaria	Girls	2007	16	15.04
Bulgaria	Girls	2008	16	15.19
Bulgaria	Girls	2009	16	15.35
Bulgaria	Girls	2010	16	15.51
Bulgaria	Girls	2011	16	15.68
Bulgaria	Girls	2012	16	15.84
Bulgaria	Girls	2013	16	16.01
Bulgaria	Girls	2014	16	16.19
Bulgaria	Girls	2015	16	16.36
Bulgaria	Girls	2016	16	16.54
Bulgaria	Girls	1975	17	12.34
Bulgaria	Girls	1976	17	12.44
Bulgaria	Girls	1977	17	12.55
Bulgaria	Girls	1978	17	12.65
Bulgaria	Girls	1979	17	12.76
Bulgaria	Girls	1980	17	12.87
Bulgaria	Girls	1981	17	12.98
Bulgaria	Girls	1982	17	13.09
Bulgaria	Girls	1983	17	13.19
Bulgaria	Girls	1984	17	13.29
Bulgaria	Girls	1985	17	13.40
Bulgaria	Girls	1986	17	13.50
Bulgaria	Girls	1987	17	13.60
Bulgaria	Girls	1988	17	13.70
Bulgaria	Girls	1989	17	13.80
Bulgaria	Girls	1990	17	13.90
Bulgaria	Girls	1991	17	14.01
Bulgaria	Girls	1992	17	14.11
Bulgaria	Girls	1993	17	14.21
Bulgaria	Girls	1994	17	14.32
Bulgaria	Girls	1995	17	14.43
Bulgaria	Girls	1996	17	14.54
Bulgaria	Girls	1997	17	14.66
Bulgaria	Girls	1998	17	14.77
Bulgaria	Girls	1999	17	14.89
Bulgaria	Girls	2000	17	15.01
Bulgaria	Girls	2001	17	15.14
Bulgaria	Girls	2002	17	15.28
Bulgaria	Girls	2003	17	15.42

Bulgaria	Girls	2004	17	15.56
Bulgaria	Girls	2005	17	15.71
Bulgaria	Girls	2006	17	15.87
Bulgaria	Girls	2007	17	16.02
Bulgaria	Girls	2008	17	16.18
Bulgaria	Girls	2009	17	16.35
Bulgaria	Girls	2010	17	16.52
Bulgaria	Girls	2011	17	16.69
Bulgaria	Girls	2012	17	16.86
Bulgaria	Girls	2013	17	17.04
Bulgaria	Girls	2014	17	17.22
Bulgaria	Girls	2015	17	17.40
Bulgaria	Girls	2016	17	17.59
Bulgaria	Girls	1975	18	12.95
Bulgaria	Girls	1976	18	13.06
Bulgaria	Girls	1977	18	13.17
Bulgaria	Girls	1978	18	13.29
Bulgaria	Girls	1979	18	13.41
Bulgaria	Girls	1980	18	13.53
Bulgaria	Girls	1981	18	13.64
Bulgaria	Girls	1982	18	13.76
Bulgaria	Girls	1983	18	13.87
Bulgaria	Girls	1984	18	13.98
Bulgaria	Girls	1985	18	14.09
Bulgaria	Girls	1986	18	14.20
Bulgaria	Girls	1987	18	14.31
Bulgaria	Girls	1988	18	14.42
Bulgaria	Girls	1989	18	14.53
Bulgaria	Girls	1990	18	14.64
Bulgaria	Girls	1991	18	14.76
Bulgaria	Girls	1992	18	14.87
Bulgaria	Girls	1993	18	14.98
Bulgaria	Girls	1994	18	15.10
Bulgaria	Girls	1995	18	15.22
Bulgaria	Girls	1996	18	15.34
Bulgaria	Girls	1997	18	15.47
Bulgaria	Girls	1998	18	15.59
Bulgaria	Girls	1999	18	15.72
Bulgaria	Girls	2000	18	15.86
Bulgaria	Girls	2001	18	16.00
Bulgaria	Girls	2002	18	16.15
Bulgaria	Girls	2003	18	16.30
Bulgaria	Girls	2004	18	16.46
Bulgaria	Girls	2005	18	16.62
Bulgaria	Girls	2006	18	16.79
Bulgaria	Girls	2007	18	16.96
Bulgaria	Girls	2008	18	17.13
Bulgaria	Girls	2009	18	17.31
Bulgaria	Girls	2010	18	17.49
Bulgaria	Girls	2011	18	17.68
Bulgaria	Girls	2012	18	17.86
Bulgaria	Girls	2013	18	18.05

Bulgaria	Girls	2014	18	18.25
Bulgaria	Girls	2015	18	18.45
Bulgaria	Girls	2016	18	18.65
Bulgaria	Girls	1975	Average	9.92
Bulgaria	Girls	1976	Average	10.02
Bulgaria	Girls	1977	Average	10.11
Bulgaria	Girls	1978	Average	10.21
Bulgaria	Girls	1979	Average	10.31
Bulgaria	Girls	1980	Average	10.41
Bulgaria	Girls	1981	Average	10.51
Bulgaria	Girls	1982	Average	10.61
Bulgaria	Girls	1983	Average	10.70
Bulgaria	Girls	1984	Average	10.80
Bulgaria	Girls	1985	Average	10.89
Bulgaria	Girls	1986	Average	10.98
Bulgaria	Girls	1987	Average	11.08
Bulgaria	Girls	1988	Average	11.17
Bulgaria	Girls	1989	Average	11.26
Bulgaria	Girls	1990	Average	11.36
Bulgaria	Girls	1991	Average	11.45
Bulgaria	Girls	1992	Average	11.55
Bulgaria	Girls	1993	Average	11.64
Bulgaria	Girls	1994	Average	11.74
Bulgaria	Girls	1995	Average	11.84
Bulgaria	Girls	1996	Average	11.95
Bulgaria	Girls	1997	Average	12.05
Bulgaria	Girls	1998	Average	12.16
Bulgaria	Girls	1999	Average	12.27
Bulgaria	Girls	2000	Average	12.38
Bulgaria	Girls	2001	Average	12.50
Bulgaria	Girls	2002	Average	12.63
Bulgaria	Girls	2003	Average	12.76
Bulgaria	Girls	2004	Average	12.89
Bulgaria	Girls	2005	Average	13.03
Bulgaria	Girls	2006	Average	13.17
Bulgaria	Girls	2007	Average	13.31
Bulgaria	Girls	2008	Average	13.46
Bulgaria	Girls	2009	Average	13.61
Bulgaria	Girls	2010	Average	13.77
Bulgaria	Girls	2011	Average	13.93
Bulgaria	Girls	2012	Average	14.08
Bulgaria	Girls	2013	Average	14.25
Bulgaria	Girls	2014	Average	14.41
Bulgaria	Girls	2015	Average	14.58
Bulgaria	Girls	2016	Average	14.75
Burkina Faso	Boys	1975	12	3.91
Burkina Faso	Boys	1976	12	3.93
Burkina Faso	Boys	1977	12	3.94
Burkina Faso	Boys	1978	12	3.96
Burkina Faso	Boys	1979	12	3.97
Burkina Faso	Boys	1980	12	3.99
Burkina Faso	Boys	1981	12	4.01

Burkina Faso	Boys	1982	12	4.03
Burkina Faso	Boys	1983	12	4.05
Burkina Faso	Boys	1984	12	4.07
Burkina Faso	Boys	1985	12	4.09
Burkina Faso	Boys	1986	12	4.11
Burkina Faso	Boys	1987	12	4.13
Burkina Faso	Boys	1988	12	4.16
Burkina Faso	Boys	1989	12	4.18
Burkina Faso	Boys	1990	12	4.20
Burkina Faso	Boys	1991	12	4.23
Burkina Faso	Boys	1992	12	4.25
Burkina Faso	Boys	1993	12	4.28
Burkina Faso	Boys	1994	12	4.31
Burkina Faso	Boys	1995	12	4.34
Burkina Faso	Boys	1996	12	4.37
Burkina Faso	Boys	1997	12	4.40
Burkina Faso	Boys	1998	12	4.44
Burkina Faso	Boys	1999	12	4.47
Burkina Faso	Boys	2000	12	4.51
Burkina Faso	Boys	2001	12	4.56
Burkina Faso	Boys	2002	12	4.60
Burkina Faso	Boys	2003	12	4.65
Burkina Faso	Boys	2004	12	4.71
Burkina Faso	Boys	2005	12	4.76
Burkina Faso	Boys	2006	12	4.82
Burkina Faso	Boys	2007	12	4.89
Burkina Faso	Boys	2008	12	4.96
Burkina Faso	Boys	2009	12	5.03
Burkina Faso	Boys	2010	12	5.10
Burkina Faso	Boys	2011	12	5.19
Burkina Faso	Boys	2012	12	5.27
Burkina Faso	Boys	2013	12	5.36
Burkina Faso	Boys	2014	12	5.45
Burkina Faso	Boys	2015	12	5.56
Burkina Faso	Boys	2016	12	5.66
Burkina Faso	Boys	1975	13	4.89
Burkina Faso	Boys	1976	13	4.91
Burkina Faso	Boys	1977	13	4.93
Burkina Faso	Boys	1978	13	4.95
Burkina Faso	Boys	1979	13	4.97
Burkina Faso	Boys	1980	13	5.00
Burkina Faso	Boys	1981	13	5.02
Burkina Faso	Boys	1982	13	5.04
Burkina Faso	Boys	1983	13	5.07
Burkina Faso	Boys	1984	13	5.09
Burkina Faso	Boys	1985	13	5.12
Burkina Faso	Boys	1986	13	5.15
Burkina Faso	Boys	1987	13	5.17
Burkina Faso	Boys	1988	13	5.20
Burkina Faso	Boys	1989	13	5.23
Burkina Faso	Boys	1990	13	5.26
Burkina Faso	Boys	1991	13	5.29

Burkina Faso	Boys	1992	13	5.32
Burkina Faso	Boys	1993	13	5.35
Burkina Faso	Boys	1994	13	5.39
Burkina Faso	Boys	1995	13	5.42
Burkina Faso	Boys	1996	13	5.46
Burkina Faso	Boys	1997	13	5.50
Burkina Faso	Boys	1998	13	5.54
Burkina Faso	Boys	1999	13	5.59
Burkina Faso	Boys	2000	13	5.64
Burkina Faso	Boys	2001	13	5.69
Burkina Faso	Boys	2002	13	5.75
Burkina Faso	Boys	2003	13	5.80
Burkina Faso	Boys	2004	13	5.87
Burkina Faso	Boys	2005	13	5.94
Burkina Faso	Boys	2006	13	6.01
Burkina Faso	Boys	2007	13	6.08
Burkina Faso	Boys	2008	13	6.16
Burkina Faso	Boys	2009	13	6.25
Burkina Faso	Boys	2010	13	6.34
Burkina Faso	Boys	2011	13	6.43
Burkina Faso	Boys	2012	13	6.53
Burkina Faso	Boys	2013	13	6.64
Burkina Faso	Boys	2014	13	6.75
Burkina Faso	Boys	2015	13	6.87
Burkina Faso	Boys	2016	13	6.99
Burkina Faso	Boys	1975	14	6.19
Burkina Faso	Boys	1976	14	6.22
Burkina Faso	Boys	1977	14	6.24
Burkina Faso	Boys	1978	14	6.27
Burkina Faso	Boys	1979	14	6.29
Burkina Faso	Boys	1980	14	6.32
Burkina Faso	Boys	1981	14	6.35
Burkina Faso	Boys	1982	14	6.38
Burkina Faso	Boys	1983	14	6.41
Burkina Faso	Boys	1984	14	6.44
Burkina Faso	Boys	1985	14	6.48
Burkina Faso	Boys	1986	14	6.51
Burkina Faso	Boys	1987	14	6.54
Burkina Faso	Boys	1988	14	6.58
Burkina Faso	Boys	1989	14	6.61
Burkina Faso	Boys	1990	14	6.65
Burkina Faso	Boys	1991	14	6.68
Burkina Faso	Boys	1992	14	6.72
Burkina Faso	Boys	1993	14	6.76
Burkina Faso	Boys	1994	14	6.80
Burkina Faso	Boys	1995	14	6.84
Burkina Faso	Boys	1996	14	6.89
Burkina Faso	Boys	1997	14	6.93
Burkina Faso	Boys	1998	14	6.98
Burkina Faso	Boys	1999	14	7.04
Burkina Faso	Boys	2000	14	7.09
Burkina Faso	Boys	2001	14	7.15

Burkina Faso	Boys	2002	14	7.22
Burkina Faso	Boys	2003	14	7.29
Burkina Faso	Boys	2004	14	7.36
Burkina Faso	Boys	2005	14	7.44
Burkina Faso	Boys	2006	14	7.52
Burkina Faso	Boys	2007	14	7.61
Burkina Faso	Boys	2008	14	7.70
Burkina Faso	Boys	2009	14	7.79
Burkina Faso	Boys	2010	14	7.90
Burkina Faso	Boys	2011	14	8.00
Burkina Faso	Boys	2012	14	8.12
Burkina Faso	Boys	2013	14	8.23
Burkina Faso	Boys	2014	14	8.36
Burkina Faso	Boys	2015	14	8.49
Burkina Faso	Boys	2016	14	8.63
Burkina Faso	Boys	1975	15	7.75
Burkina Faso	Boys	1976	15	7.78
Burkina Faso	Boys	1977	15	7.81
Burkina Faso	Boys	1978	15	7.85
Burkina Faso	Boys	1979	15	7.88
Burkina Faso	Boys	1980	15	7.92
Burkina Faso	Boys	1981	15	7.95
Burkina Faso	Boys	1982	15	7.99
Burkina Faso	Boys	1983	15	8.02
Burkina Faso	Boys	1984	15	8.06
Burkina Faso	Boys	1985	15	8.10
Burkina Faso	Boys	1986	15	8.14
Burkina Faso	Boys	1987	15	8.18
Burkina Faso	Boys	1988	15	8.22
Burkina Faso	Boys	1989	15	8.27
Burkina Faso	Boys	1990	15	8.31
Burkina Faso	Boys	1991	15	8.35
Burkina Faso	Boys	1992	15	8.39
Burkina Faso	Boys	1993	15	8.44
Burkina Faso	Boys	1994	15	8.49
Burkina Faso	Boys	1995	15	8.54
Burkina Faso	Boys	1996	15	8.59
Burkina Faso	Boys	1997	15	8.64
Burkina Faso	Boys	1998	15	8.70
Burkina Faso	Boys	1999	15	8.76
Burkina Faso	Boys	2000	15	8.82
Burkina Faso	Boys	2001	15	8.89
Burkina Faso	Boys	2002	15	8.96
Burkina Faso	Boys	2003	15	9.04
Burkina Faso	Boys	2004	15	9.12
Burkina Faso	Boys	2005	15	9.21
Burkina Faso	Boys	2006	15	9.30
Burkina Faso	Boys	2007	15	9.40
Burkina Faso	Boys	2008	15	9.50
Burkina Faso	Boys	2009	15	9.60
Burkina Faso	Boys	2010	15	9.71
Burkina Faso	Boys	2011	15	9.83

Burkina Faso	Boys	2012	15	9.95
Burkina Faso	Boys	2013	15	10.08
Burkina Faso	Boys	2014	15	10.21
Burkina Faso	Boys	2015	15	10.36
Burkina Faso	Boys	2016	15	10.51
Burkina Faso	Boys	1975	16	9.49
Burkina Faso	Boys	1976	16	9.53
Burkina Faso	Boys	1977	16	9.57
Burkina Faso	Boys	1978	16	9.61
Burkina Faso	Boys	1979	16	9.65
Burkina Faso	Boys	1980	16	9.69
Burkina Faso	Boys	1981	16	9.74
Burkina Faso	Boys	1982	16	9.78
Burkina Faso	Boys	1983	16	9.82
Burkina Faso	Boys	1984	16	9.87
Burkina Faso	Boys	1985	16	9.92
Burkina Faso	Boys	1986	16	9.97
Burkina Faso	Boys	1987	16	10.01
Burkina Faso	Boys	1988	16	10.06
Burkina Faso	Boys	1989	16	10.11
Burkina Faso	Boys	1990	16	10.16
Burkina Faso	Boys	1991	16	10.21
Burkina Faso	Boys	1992	16	10.26
Burkina Faso	Boys	1993	16	10.31
Burkina Faso	Boys	1994	16	10.37
Burkina Faso	Boys	1995	16	10.42
Burkina Faso	Boys	1996	16	10.48
Burkina Faso	Boys	1997	16	10.54
Burkina Faso	Boys	1998	16	10.61
Burkina Faso	Boys	1999	16	10.67
Burkina Faso	Boys	2000	16	10.74
Burkina Faso	Boys	2001	16	10.82
Burkina Faso	Boys	2002	16	10.90
Burkina Faso	Boys	2003	16	10.98
Burkina Faso	Boys	2004	16	11.07
Burkina Faso	Boys	2005	16	11.17
Burkina Faso	Boys	2006	16	11.27
Burkina Faso	Boys	2007	16	11.37
Burkina Faso	Boys	2008	16	11.48
Burkina Faso	Boys	2009	16	11.59
Burkina Faso	Boys	2010	16	11.71
Burkina Faso	Boys	2011	16	11.83
Burkina Faso	Boys	2012	16	11.96
Burkina Faso	Boys	2013	16	12.10
Burkina Faso	Boys	2014	16	12.24
Burkina Faso	Boys	2015	16	12.39
Burkina Faso	Boys	2016	16	12.55
Burkina Faso	Boys	1975	17	11.30
Burkina Faso	Boys	1976	17	11.35
Burkina Faso	Boys	1977	17	11.40
Burkina Faso	Boys	1978	17	11.44
Burkina Faso	Boys	1979	17	11.49

Burkina Faso	Boys	1980	17	11.54
Burkina Faso	Boys	1981	17	11.59
Burkina Faso	Boys	1982	17	11.65
Burkina Faso	Boys	1983	17	11.70
Burkina Faso	Boys	1984	17	11.75
Burkina Faso	Boys	1985	17	11.81
Burkina Faso	Boys	1986	17	11.87
Burkina Faso	Boys	1987	17	11.92
Burkina Faso	Boys	1988	17	11.98
Burkina Faso	Boys	1989	17	12.03
Burkina Faso	Boys	1990	17	12.09
Burkina Faso	Boys	1991	17	12.15
Burkina Faso	Boys	1992	17	12.20
Burkina Faso	Boys	1993	17	12.26
Burkina Faso	Boys	1994	17	12.32
Burkina Faso	Boys	1995	17	12.39
Burkina Faso	Boys	1996	17	12.45
Burkina Faso	Boys	1997	17	12.52
Burkina Faso	Boys	1998	17	12.59
Burkina Faso	Boys	1999	17	12.67
Burkina Faso	Boys	2000	17	12.75
Burkina Faso	Boys	2001	17	12.83
Burkina Faso	Boys	2002	17	12.92
Burkina Faso	Boys	2003	17	13.01
Burkina Faso	Boys	2004	17	13.11
Burkina Faso	Boys	2005	17	13.21
Burkina Faso	Boys	2006	17	13.32
Burkina Faso	Boys	2007	17	13.43
Burkina Faso	Boys	2008	17	13.54
Burkina Faso	Boys	2009	17	13.67
Burkina Faso	Boys	2010	17	13.79
Burkina Faso	Boys	2011	17	13.92
Burkina Faso	Boys	2012	17	14.06
Burkina Faso	Boys	2013	17	14.20
Burkina Faso	Boys	2014	17	14.35
Burkina Faso	Boys	2015	17	14.51
Burkina Faso	Boys	2016	17	14.67
Burkina Faso	Boys	1975	18	13.07
Burkina Faso	Boys	1976	18	13.12
Burkina Faso	Boys	1977	18	13.18
Burkina Faso	Boys	1978	18	13.24
Burkina Faso	Boys	1979	18	13.30
Burkina Faso	Boys	1980	18	13.35
Burkina Faso	Boys	1981	18	13.42
Burkina Faso	Boys	1982	18	13.48
Burkina Faso	Boys	1983	18	13.54
Burkina Faso	Boys	1984	18	13.60
Burkina Faso	Boys	1985	18	13.67
Burkina Faso	Boys	1986	18	13.73
Burkina Faso	Boys	1987	18	13.79
Burkina Faso	Boys	1988	18	13.86
Burkina Faso	Boys	1989	18	13.92

Burkina Faso	Boys	1990	18	13.99
Burkina Faso	Boys	1991	18	14.05
Burkina Faso	Boys	1992	18	14.12
Burkina Faso	Boys	1993	18	14.19
Burkina Faso	Boys	1994	18	14.26
Burkina Faso	Boys	1995	18	14.33
Burkina Faso	Boys	1996	18	14.40
Burkina Faso	Boys	1997	18	14.48
Burkina Faso	Boys	1998	18	14.56
Burkina Faso	Boys	1999	18	14.64
Burkina Faso	Boys	2000	18	14.73
Burkina Faso	Boys	2001	18	14.83
Burkina Faso	Boys	2002	18	14.92
Burkina Faso	Boys	2003	18	15.02
Burkina Faso	Boys	2004	18	15.13
Burkina Faso	Boys	2005	18	15.24
Burkina Faso	Boys	2006	18	15.36
Burkina Faso	Boys	2007	18	15.48
Burkina Faso	Boys	2008	18	15.61
Burkina Faso	Boys	2009	18	15.74
Burkina Faso	Boys	2010	18	15.87
Burkina Faso	Boys	2011	18	16.01
Burkina Faso	Boys	2012	18	16.16
Burkina Faso	Boys	2013	18	16.31
Burkina Faso	Boys	2014	18	16.47
Burkina Faso	Boys	2015	18	16.64
Burkina Faso	Boys	2016	18	16.81
Burkina Faso	Boys	1975	Average	8.09
Burkina Faso	Boys	1976	Average	8.12
Burkina Faso	Boys	1977	Average	8.15
Burkina Faso	Boys	1978	Average	8.19
Burkina Faso	Boys	1979	Average	8.22
Burkina Faso	Boys	1980	Average	8.26
Burkina Faso	Boys	1981	Average	8.30
Burkina Faso	Boys	1982	Average	8.33
Burkina Faso	Boys	1983	Average	8.37
Burkina Faso	Boys	1984	Average	8.41
Burkina Faso	Boys	1985	Average	8.45
Burkina Faso	Boys	1986	Average	8.50
Burkina Faso	Boys	1987	Average	8.54
Burkina Faso	Boys	1988	Average	8.58
Burkina Faso	Boys	1989	Average	8.62
Burkina Faso	Boys	1990	Average	8.66
Burkina Faso	Boys	1991	Average	8.71
Burkina Faso	Boys	1992	Average	8.75
Burkina Faso	Boys	1993	Average	8.80
Burkina Faso	Boys	1994	Average	8.85
Burkina Faso	Boys	1995	Average	8.90
Burkina Faso	Boys	1996	Average	8.95
Burkina Faso	Boys	1997	Average	9.00
Burkina Faso	Boys	1998	Average	9.06
Burkina Faso	Boys	1999	Average	9.12

Burkina Faso	Boys	2000	Average	9.19
Burkina Faso	Boys	2001	Average	9.25
Burkina Faso	Boys	2002	Average	9.33
Burkina Faso	Boys	2003	Average	9.40
Burkina Faso	Boys	2004	Average	9.48
Burkina Faso	Boys	2005	Average	9.57
Burkina Faso	Boys	2006	Average	9.66
Burkina Faso	Boys	2007	Average	9.75
Burkina Faso	Boys	2008	Average	9.85
Burkina Faso	Boys	2009	Average	9.95
Burkina Faso	Boys	2010	Average	10.06
Burkina Faso	Boys	2011	Average	10.17
Burkina Faso	Boys	2012	Average	10.29
Burkina Faso	Boys	2013	Average	10.42
Burkina Faso	Boys	2014	Average	10.55
Burkina Faso	Boys	2015	Average	10.69
Burkina Faso	Boys	2016	Average	10.83
Burkina Faso	Girls	1975	12	4.48
Burkina Faso	Girls	1976	12	4.50
Burkina Faso	Girls	1977	12	4.51
Burkina Faso	Girls	1978	12	4.53
Burkina Faso	Girls	1979	12	4.56
Burkina Faso	Girls	1980	12	4.58
Burkina Faso	Girls	1981	12	4.60
Burkina Faso	Girls	1982	12	4.63
Burkina Faso	Girls	1983	12	4.65
Burkina Faso	Girls	1984	12	4.68
Burkina Faso	Girls	1985	12	4.71
Burkina Faso	Girls	1986	12	4.74
Burkina Faso	Girls	1987	12	4.77
Burkina Faso	Girls	1988	12	4.80
Burkina Faso	Girls	1989	12	4.84
Burkina Faso	Girls	1990	12	4.87
Burkina Faso	Girls	1991	12	4.90
Burkina Faso	Girls	1992	12	4.94
Burkina Faso	Girls	1993	12	4.98
Burkina Faso	Girls	1994	12	5.02
Burkina Faso	Girls	1995	12	5.06
Burkina Faso	Girls	1996	12	5.10
Burkina Faso	Girls	1997	12	5.15
Burkina Faso	Girls	1998	12	5.20
Burkina Faso	Girls	1999	12	5.25
Burkina Faso	Girls	2000	12	5.30
Burkina Faso	Girls	2001	12	5.36
Burkina Faso	Girls	2002	12	5.43
Burkina Faso	Girls	2003	12	5.50
Burkina Faso	Girls	2004	12	5.57
Burkina Faso	Girls	2005	12	5.65
Burkina Faso	Girls	2006	12	5.73
Burkina Faso	Girls	2007	12	5.81
Burkina Faso	Girls	2008	12	5.90
Burkina Faso	Girls	2009	12	5.99

Burkina Faso	Girls	2010	12	6.09
Burkina Faso	Girls	2011	12	6.18
Burkina Faso	Girls	2012	12	6.28
Burkina Faso	Girls	2013	12	6.39
Burkina Faso	Girls	2014	12	6.50
Burkina Faso	Girls	2015	12	6.61
Burkina Faso	Girls	2016	12	6.72
Burkina Faso	Girls	1975	13	5.66
Burkina Faso	Girls	1976	13	5.68
Burkina Faso	Girls	1977	13	5.70
Burkina Faso	Girls	1978	13	5.73
Burkina Faso	Girls	1979	13	5.76
Burkina Faso	Girls	1980	13	5.78
Burkina Faso	Girls	1981	13	5.81
Burkina Faso	Girls	1982	13	5.85
Burkina Faso	Girls	1983	13	5.88
Burkina Faso	Girls	1984	13	5.91
Burkina Faso	Girls	1985	13	5.95
Burkina Faso	Girls	1986	13	5.99
Burkina Faso	Girls	1987	13	6.02
Burkina Faso	Girls	1988	13	6.06
Burkina Faso	Girls	1989	13	6.10
Burkina Faso	Girls	1990	13	6.14
Burkina Faso	Girls	1991	13	6.19
Burkina Faso	Girls	1992	13	6.23
Burkina Faso	Girls	1993	13	6.27
Burkina Faso	Girls	1994	13	6.32
Burkina Faso	Girls	1995	13	6.37
Burkina Faso	Girls	1996	13	6.42
Burkina Faso	Girls	1997	13	6.48
Burkina Faso	Girls	1998	13	6.54
Burkina Faso	Girls	1999	13	6.60
Burkina Faso	Girls	2000	13	6.67
Burkina Faso	Girls	2001	13	6.74
Burkina Faso	Girls	2002	13	6.81
Burkina Faso	Girls	2003	13	6.90
Burkina Faso	Girls	2004	13	6.98
Burkina Faso	Girls	2005	13	7.07
Burkina Faso	Girls	2006	13	7.17
Burkina Faso	Girls	2007	13	7.27
Burkina Faso	Girls	2008	13	7.37
Burkina Faso	Girls	2009	13	7.48
Burkina Faso	Girls	2010	13	7.59
Burkina Faso	Girls	2011	13	7.70
Burkina Faso	Girls	2012	13	7.82
Burkina Faso	Girls	2013	13	7.95
Burkina Faso	Girls	2014	13	8.07
Burkina Faso	Girls	2015	13	8.20
Burkina Faso	Girls	2016	13	8.33
Burkina Faso	Girls	1975	14	6.99
Burkina Faso	Girls	1976	14	7.02
Burkina Faso	Girls	1977	14	7.05

Burkina Faso	Girls	1978	14	7.08
Burkina Faso	Girls	1979	14	7.11
Burkina Faso	Girls	1980	14	7.14
Burkina Faso	Girls	1981	14	7.18
Burkina Faso	Girls	1982	14	7.22
Burkina Faso	Girls	1983	14	7.26
Burkina Faso	Girls	1984	14	7.30
Burkina Faso	Girls	1985	14	7.34
Burkina Faso	Girls	1986	14	7.38
Burkina Faso	Girls	1987	14	7.43
Burkina Faso	Girls	1988	14	7.47
Burkina Faso	Girls	1989	14	7.52
Burkina Faso	Girls	1990	14	7.56
Burkina Faso	Girls	1991	14	7.61
Burkina Faso	Girls	1992	14	7.66
Burkina Faso	Girls	1993	14	7.71
Burkina Faso	Girls	1994	14	7.77
Burkina Faso	Girls	1995	14	7.82
Burkina Faso	Girls	1996	14	7.88
Burkina Faso	Girls	1997	14	7.95
Burkina Faso	Girls	1998	14	8.01
Burkina Faso	Girls	1999	14	8.08
Burkina Faso	Girls	2000	14	8.16
Burkina Faso	Girls	2001	14	8.24
Burkina Faso	Girls	2002	14	8.33
Burkina Faso	Girls	2003	14	8.42
Burkina Faso	Girls	2004	14	8.51
Burkina Faso	Girls	2005	14	8.62
Burkina Faso	Girls	2006	14	8.72
Burkina Faso	Girls	2007	14	8.83
Burkina Faso	Girls	2008	14	8.95
Burkina Faso	Girls	2009	14	9.07
Burkina Faso	Girls	2010	14	9.19
Burkina Faso	Girls	2011	14	9.32
Burkina Faso	Girls	2012	14	9.45
Burkina Faso	Girls	2013	14	9.59
Burkina Faso	Girls	2014	14	9.73
Burkina Faso	Girls	2015	14	9.87
Burkina Faso	Girls	2016	14	10.02
Burkina Faso	Girls	1975	15	8.29
Burkina Faso	Girls	1976	15	8.32
Burkina Faso	Girls	1977	15	8.35
Burkina Faso	Girls	1978	15	8.39
Burkina Faso	Girls	1979	15	8.42
Burkina Faso	Girls	1980	15	8.46
Burkina Faso	Girls	1981	15	8.50
Burkina Faso	Girls	1982	15	8.54
Burkina Faso	Girls	1983	15	8.59
Burkina Faso	Girls	1984	15	8.63
Burkina Faso	Girls	1985	15	8.68
Burkina Faso	Girls	1986	15	8.73
Burkina Faso	Girls	1987	15	8.78

Burkina Faso	Girls	1988	15	8.82
Burkina Faso	Girls	1989	15	8.88
Burkina Faso	Girls	1990	15	8.93
Burkina Faso	Girls	1991	15	8.98
Burkina Faso	Girls	1992	15	9.03
Burkina Faso	Girls	1993	15	9.09
Burkina Faso	Girls	1994	15	9.15
Burkina Faso	Girls	1995	15	9.21
Burkina Faso	Girls	1996	15	9.27
Burkina Faso	Girls	1997	15	9.34
Burkina Faso	Girls	1998	15	9.41
Burkina Faso	Girls	1999	15	9.49
Burkina Faso	Girls	2000	15	9.57
Burkina Faso	Girls	2001	15	9.66
Burkina Faso	Girls	2002	15	9.75
Burkina Faso	Girls	2003	15	9.85
Burkina Faso	Girls	2004	15	9.95
Burkina Faso	Girls	2005	15	10.06
Burkina Faso	Girls	2006	15	10.17
Burkina Faso	Girls	2007	15	10.29
Burkina Faso	Girls	2008	15	10.41
Burkina Faso	Girls	2009	15	10.54
Burkina Faso	Girls	2010	15	10.67
Burkina Faso	Girls	2011	15	10.81
Burkina Faso	Girls	2012	15	10.95
Burkina Faso	Girls	2013	15	11.09
Burkina Faso	Girls	2014	15	11.24
Burkina Faso	Girls	2015	15	11.39
Burkina Faso	Girls	2016	15	11.55
Burkina Faso	Girls	1975	16	9.36
Burkina Faso	Girls	1976	16	9.39
Burkina Faso	Girls	1977	16	9.43
Burkina Faso	Girls	1978	16	9.47
Burkina Faso	Girls	1979	16	9.50
Burkina Faso	Girls	1980	16	9.55
Burkina Faso	Girls	1981	16	9.59
Burkina Faso	Girls	1982	16	9.63
Burkina Faso	Girls	1983	16	9.68
Burkina Faso	Girls	1984	16	9.73
Burkina Faso	Girls	1985	16	9.78
Burkina Faso	Girls	1986	16	9.83
Burkina Faso	Girls	1987	16	9.88
Burkina Faso	Girls	1988	16	9.94
Burkina Faso	Girls	1989	16	9.99
Burkina Faso	Girls	1990	16	10.04
Burkina Faso	Girls	1991	16	10.10
Burkina Faso	Girls	1992	16	10.16
Burkina Faso	Girls	1993	16	10.22
Burkina Faso	Girls	1994	16	10.28
Burkina Faso	Girls	1995	16	10.34
Burkina Faso	Girls	1996	16	10.41
Burkina Faso	Girls	1997	16	10.48

Burkina Faso	Girls	1998	16	10.55
Burkina Faso	Girls	1999	16	10.63
Burkina Faso	Girls	2000	16	10.72
Burkina Faso	Girls	2001	16	10.81
Burkina Faso	Girls	2002	16	10.90
Burkina Faso	Girls	2003	16	11.01
Burkina Faso	Girls	2004	16	11.11
Burkina Faso	Girls	2005	16	11.23
Burkina Faso	Girls	2006	16	11.34
Burkina Faso	Girls	2007	16	11.47
Burkina Faso	Girls	2008	16	11.59
Burkina Faso	Girls	2009	16	11.73
Burkina Faso	Girls	2010	16	11.86
Burkina Faso	Girls	2011	16	12.00
Burkina Faso	Girls	2012	16	12.15
Burkina Faso	Girls	2013	16	12.30
Burkina Faso	Girls	2014	16	12.45
Burkina Faso	Girls	2015	16	12.61
Burkina Faso	Girls	2016	16	12.77
Burkina Faso	Girls	1975	17	10.11
Burkina Faso	Girls	1976	17	10.15
Burkina Faso	Girls	1977	17	10.18
Burkina Faso	Girls	1978	17	10.23
Burkina Faso	Girls	1979	17	10.27
Burkina Faso	Girls	1980	17	10.31
Burkina Faso	Girls	1981	17	10.36
Burkina Faso	Girls	1982	17	10.40
Burkina Faso	Girls	1983	17	10.45
Burkina Faso	Girls	1984	17	10.51
Burkina Faso	Girls	1985	17	10.56
Burkina Faso	Girls	1986	17	10.61
Burkina Faso	Girls	1987	17	10.67
Burkina Faso	Girls	1988	17	10.72
Burkina Faso	Girls	1989	17	10.78
Burkina Faso	Girls	1990	17	10.83
Burkina Faso	Girls	1991	17	10.89
Burkina Faso	Girls	1992	17	10.95
Burkina Faso	Girls	1993	17	11.01
Burkina Faso	Girls	1994	17	11.08
Burkina Faso	Girls	1995	17	11.14
Burkina Faso	Girls	1996	17	11.22
Burkina Faso	Girls	1997	17	11.29
Burkina Faso	Girls	1998	17	11.37
Burkina Faso	Girls	1999	17	11.45
Burkina Faso	Girls	2000	17	11.54
Burkina Faso	Girls	2001	17	11.63
Burkina Faso	Girls	2002	17	11.73
Burkina Faso	Girls	2003	17	11.84
Burkina Faso	Girls	2004	17	11.95
Burkina Faso	Girls	2005	17	12.07
Burkina Faso	Girls	2006	17	12.19
Burkina Faso	Girls	2007	17	12.32

Burkina Faso	Girls	2008	17	12.45
Burkina Faso	Girls	2009	17	12.59
Burkina Faso	Girls	2010	17	12.73
Burkina Faso	Girls	2011	17	12.88
Burkina Faso	Girls	2012	17	13.03
Burkina Faso	Girls	2013	17	13.19
Burkina Faso	Girls	2014	17	13.35
Burkina Faso	Girls	2015	17	13.51
Burkina Faso	Girls	2016	17	13.69
Burkina Faso	Girls	1975	18	10.57
Burkina Faso	Girls	1976	18	10.61
Burkina Faso	Girls	1977	18	10.65
Burkina Faso	Girls	1978	18	10.70
Burkina Faso	Girls	1979	18	10.74
Burkina Faso	Girls	1980	18	10.79
Burkina Faso	Girls	1981	18	10.84
Burkina Faso	Girls	1982	18	10.89
Burkina Faso	Girls	1983	18	10.94
Burkina Faso	Girls	1984	18	10.99
Burkina Faso	Girls	1985	18	11.05
Burkina Faso	Girls	1986	18	11.11
Burkina Faso	Girls	1987	18	11.17
Burkina Faso	Girls	1988	18	11.22
Burkina Faso	Girls	1989	18	11.28
Burkina Faso	Girls	1990	18	11.35
Burkina Faso	Girls	1991	18	11.41
Burkina Faso	Girls	1992	18	11.47
Burkina Faso	Girls	1993	18	11.54
Burkina Faso	Girls	1994	18	11.61
Burkina Faso	Girls	1995	18	11.68
Burkina Faso	Girls	1996	18	11.75
Burkina Faso	Girls	1997	18	11.83
Burkina Faso	Girls	1998	18	11.92
Burkina Faso	Girls	1999	18	12.01
Burkina Faso	Girls	2000	18	12.10
Burkina Faso	Girls	2001	18	12.20
Burkina Faso	Girls	2002	18	12.31
Burkina Faso	Girls	2003	18	12.42
Burkina Faso	Girls	2004	18	12.54
Burkina Faso	Girls	2005	18	12.67
Burkina Faso	Girls	2006	18	12.80
Burkina Faso	Girls	2007	18	12.94
Burkina Faso	Girls	2008	18	13.09
Burkina Faso	Girls	2009	18	13.23
Burkina Faso	Girls	2010	18	13.39
Burkina Faso	Girls	2011	18	13.55
Burkina Faso	Girls	2012	18	13.71
Burkina Faso	Girls	2013	18	13.88
Burkina Faso	Girls	2014	18	14.05
Burkina Faso	Girls	2015	18	14.23
Burkina Faso	Girls	2016	18	14.42
Burkina Faso	Girls	1975	Average	7.92

Burkina Faso	Girls	1976	Average	7.95
Burkina Faso	Girls	1977	Average	7.98
Burkina Faso	Girls	1978	Average	8.02
Burkina Faso	Girls	1979	Average	8.05
Burkina Faso	Girls	1980	Average	8.09
Burkina Faso	Girls	1981	Average	8.13
Burkina Faso	Girls	1982	Average	8.17
Burkina Faso	Girls	1983	Average	8.21
Burkina Faso	Girls	1984	Average	8.25
Burkina Faso	Girls	1985	Average	8.30
Burkina Faso	Girls	1986	Average	8.34
Burkina Faso	Girls	1987	Average	8.39
Burkina Faso	Girls	1988	Average	8.43
Burkina Faso	Girls	1989	Average	8.48
Burkina Faso	Girls	1990	Average	8.53
Burkina Faso	Girls	1991	Average	8.58
Burkina Faso	Girls	1992	Average	8.64
Burkina Faso	Girls	1993	Average	8.69
Burkina Faso	Girls	1994	Average	8.75
Burkina Faso	Girls	1995	Average	8.80
Burkina Faso	Girls	1996	Average	8.86
Burkina Faso	Girls	1997	Average	8.93
Burkina Faso	Girls	1998	Average	9.00
Burkina Faso	Girls	1999	Average	9.07
Burkina Faso	Girls	2000	Average	9.15
Burkina Faso	Girls	2001	Average	9.23
Burkina Faso	Girls	2002	Average	9.32
Burkina Faso	Girls	2003	Average	9.42
Burkina Faso	Girls	2004	Average	9.52
Burkina Faso	Girls	2005	Average	9.62
Burkina Faso	Girls	2006	Average	9.73
Burkina Faso	Girls	2007	Average	9.85
Burkina Faso	Girls	2008	Average	9.97
Burkina Faso	Girls	2009	Average	10.09
Burkina Faso	Girls	2010	Average	10.22
Burkina Faso	Girls	2011	Average	10.35
Burkina Faso	Girls	2012	Average	10.49
Burkina Faso	Girls	2013	Average	10.63
Burkina Faso	Girls	2014	Average	10.77
Burkina Faso	Girls	2015	Average	10.92
Burkina Faso	Girls	2016	Average	11.07
Burundi	Boys	1975	12	4.07
Burundi	Boys	1976	12	4.09
Burundi	Boys	1977	12	4.11
Burundi	Boys	1978	12	4.13
Burundi	Boys	1979	12	4.15
Burundi	Boys	1980	12	4.17
Burundi	Boys	1981	12	4.19
Burundi	Boys	1982	12	4.21
Burundi	Boys	1983	12	4.23
Burundi	Boys	1984	12	4.25
Burundi	Boys	1985	12	4.28

Burundi	Boys	1986	12	4.30
Burundi	Boys	1987	12	4.32
Burundi	Boys	1988	12	4.35
Burundi	Boys	1989	12	4.38
Burundi	Boys	1990	12	4.41
Burundi	Boys	1991	12	4.44
Burundi	Boys	1992	12	4.47
Burundi	Boys	1993	12	4.50
Burundi	Boys	1994	12	4.54
Burundi	Boys	1995	12	4.57
Burundi	Boys	1996	12	4.61
Burundi	Boys	1997	12	4.65
Burundi	Boys	1998	12	4.70
Burundi	Boys	1999	12	4.74
Burundi	Boys	2000	12	4.79
Burundi	Boys	2001	12	4.84
Burundi	Boys	2002	12	4.89
Burundi	Boys	2003	12	4.94
Burundi	Boys	2004	12	5.00
Burundi	Boys	2005	12	5.06
Burundi	Boys	2006	12	5.12
Burundi	Boys	2007	12	5.18
Burundi	Boys	2008	12	5.25
Burundi	Boys	2009	12	5.33
Burundi	Boys	2010	12	5.40
Burundi	Boys	2011	12	5.48
Burundi	Boys	2012	12	5.57
Burundi	Boys	2013	12	5.66
Burundi	Boys	2014	12	5.76
Burundi	Boys	2015	12	5.86
Burundi	Boys	2016	12	5.98
Burundi	Boys	1975	13	5.10
Burundi	Boys	1976	13	5.13
Burundi	Boys	1977	13	5.15
Burundi	Boys	1978	13	5.17
Burundi	Boys	1979	13	5.20
Burundi	Boys	1980	13	5.22
Burundi	Boys	1981	13	5.25
Burundi	Boys	1982	13	5.27
Burundi	Boys	1983	13	5.30
Burundi	Boys	1984	13	5.33
Burundi	Boys	1985	13	5.36
Burundi	Boys	1986	13	5.39
Burundi	Boys	1987	13	5.42
Burundi	Boys	1988	13	5.45
Burundi	Boys	1989	13	5.48
Burundi	Boys	1990	13	5.52
Burundi	Boys	1991	13	5.55
Burundi	Boys	1992	13	5.59
Burundi	Boys	1993	13	5.63
Burundi	Boys	1994	13	5.67
Burundi	Boys	1995	13	5.72

Burundi	Boys	1996	13	5.76
Burundi	Boys	1997	13	5.81
Burundi	Boys	1998	13	5.86
Burundi	Boys	1999	13	5.91
Burundi	Boys	2000	13	5.97
Burundi	Boys	2001	13	6.03
Burundi	Boys	2002	13	6.09
Burundi	Boys	2003	13	6.15
Burundi	Boys	2004	13	6.22
Burundi	Boys	2005	13	6.29
Burundi	Boys	2006	13	6.36
Burundi	Boys	2007	13	6.44
Burundi	Boys	2008	13	6.52
Burundi	Boys	2009	13	6.60
Burundi	Boys	2010	13	6.69
Burundi	Boys	2011	13	6.79
Burundi	Boys	2012	13	6.89
Burundi	Boys	2013	13	6.99
Burundi	Boys	2014	13	7.11
Burundi	Boys	2015	13	7.23
Burundi	Boys	2016	13	7.36
Burundi	Boys	1975	14	6.46
Burundi	Boys	1976	14	6.49
Burundi	Boys	1977	14	6.52
Burundi	Boys	1978	14	6.55
Burundi	Boys	1979	14	6.58
Burundi	Boys	1980	14	6.61
Burundi	Boys	1981	14	6.64
Burundi	Boys	1982	14	6.67
Burundi	Boys	1983	14	6.70
Burundi	Boys	1984	14	6.74
Burundi	Boys	1985	14	6.77
Burundi	Boys	1986	14	6.81
Burundi	Boys	1987	14	6.85
Burundi	Boys	1988	14	6.88
Burundi	Boys	1989	14	6.92
Burundi	Boys	1990	14	6.97
Burundi	Boys	1991	14	7.01
Burundi	Boys	1992	14	7.05
Burundi	Boys	1993	14	7.10
Burundi	Boys	1994	14	7.15
Burundi	Boys	1995	14	7.20
Burundi	Boys	1996	14	7.25
Burundi	Boys	1997	14	7.31
Burundi	Boys	1998	14	7.37
Burundi	Boys	1999	14	7.43
Burundi	Boys	2000	14	7.49
Burundi	Boys	2001	14	7.56
Burundi	Boys	2002	14	7.62
Burundi	Boys	2003	14	7.70
Burundi	Boys	2004	14	7.77
Burundi	Boys	2005	14	7.85

Burundi	Boys	2006	14	7.93
Burundi	Boys	2007	14	8.02
Burundi	Boys	2008	14	8.11
Burundi	Boys	2009	14	8.20
Burundi	Boys	2010	14	8.30
Burundi	Boys	2011	14	8.41
Burundi	Boys	2012	14	8.52
Burundi	Boys	2013	14	8.64
Burundi	Boys	2014	14	8.76
Burundi	Boys	2015	14	8.90
Burundi	Boys	2016	14	9.04
Burundi	Boys	1975	15	8.09
Burundi	Boys	1976	15	8.13
Burundi	Boys	1977	15	8.16
Burundi	Boys	1978	15	8.20
Burundi	Boys	1979	15	8.24
Burundi	Boys	1980	15	8.27
Burundi	Boys	1981	15	8.31
Burundi	Boys	1982	15	8.35
Burundi	Boys	1983	15	8.39
Burundi	Boys	1984	15	8.43
Burundi	Boys	1985	15	8.47
Burundi	Boys	1986	15	8.51
Burundi	Boys	1987	15	8.56
Burundi	Boys	1988	15	8.60
Burundi	Boys	1989	15	8.65
Burundi	Boys	1990	15	8.70
Burundi	Boys	1991	15	8.75
Burundi	Boys	1992	15	8.80
Burundi	Boys	1993	15	8.85
Burundi	Boys	1994	15	8.91
Burundi	Boys	1995	15	8.96
Burundi	Boys	1996	15	9.02
Burundi	Boys	1997	15	9.08
Burundi	Boys	1998	15	9.15
Burundi	Boys	1999	15	9.21
Burundi	Boys	2000	15	9.28
Burundi	Boys	2001	15	9.36
Burundi	Boys	2002	15	9.43
Burundi	Boys	2003	15	9.51
Burundi	Boys	2004	15	9.59
Burundi	Boys	2005	15	9.68
Burundi	Boys	2006	15	9.77
Burundi	Boys	2007	15	9.86
Burundi	Boys	2008	15	9.96
Burundi	Boys	2009	15	10.06
Burundi	Boys	2010	15	10.16
Burundi	Boys	2011	15	10.28
Burundi	Boys	2012	15	10.40
Burundi	Boys	2013	15	10.52
Burundi	Boys	2014	15	10.66
Burundi	Boys	2015	15	10.80

Burundi	Boys	2016	15	10.95
Burundi	Boys	1975	16	9.91
Burundi	Boys	1976	16	9.95
Burundi	Boys	1977	16	9.99
Burundi	Boys	1978	16	10.04
Burundi	Boys	1979	16	10.08
Burundi	Boys	1980	16	10.13
Burundi	Boys	1981	16	10.17
Burundi	Boys	1982	16	10.22
Burundi	Boys	1983	16	10.27
Burundi	Boys	1984	16	10.31
Burundi	Boys	1985	16	10.36
Burundi	Boys	1986	16	10.41
Burundi	Boys	1987	16	10.46
Burundi	Boys	1988	16	10.51
Burundi	Boys	1989	16	10.57
Burundi	Boys	1990	16	10.62
Burundi	Boys	1991	16	10.68
Burundi	Boys	1992	16	10.73
Burundi	Boys	1993	16	10.79
Burundi	Boys	1994	16	10.85
Burundi	Boys	1995	16	10.92
Burundi	Boys	1996	16	10.98
Burundi	Boys	1997	16	11.05
Burundi	Boys	1998	16	11.12
Burundi	Boys	1999	16	11.19
Burundi	Boys	2000	16	11.27
Burundi	Boys	2001	16	11.35
Burundi	Boys	2002	16	11.43
Burundi	Boys	2003	16	11.51
Burundi	Boys	2004	16	11.60
Burundi	Boys	2005	16	11.69
Burundi	Boys	2006	16	11.79
Burundi	Boys	2007	16	11.88
Burundi	Boys	2008	16	11.99
Burundi	Boys	2009	16	12.09
Burundi	Boys	2010	16	12.21
Burundi	Boys	2011	16	12.32
Burundi	Boys	2012	16	12.45
Burundi	Boys	2013	16	12.58
Burundi	Boys	2014	16	12.72
Burundi	Boys	2015	16	12.87
Burundi	Boys	2016	16	13.02
Burundi	Boys	1975	17	11.80
Burundi	Boys	1976	17	11.85
Burundi	Boys	1977	17	11.90
Burundi	Boys	1978	17	11.95
Burundi	Boys	1979	17	12.01
Burundi	Boys	1980	17	12.06
Burundi	Boys	1981	17	12.11
Burundi	Boys	1982	17	12.17
Burundi	Boys	1983	17	12.22

Burundi	Boys	1984	17	12.28
Burundi	Boys	1985	17	12.33
Burundi	Boys	1986	17	12.39
Burundi	Boys	1987	17	12.44
Burundi	Boys	1988	17	12.50
Burundi	Boys	1989	17	12.56
Burundi	Boys	1990	17	12.62
Burundi	Boys	1991	17	12.69
Burundi	Boys	1992	17	12.75
Burundi	Boys	1993	17	12.82
Burundi	Boys	1994	17	12.88
Burundi	Boys	1995	17	12.95
Burundi	Boys	1996	17	13.03
Burundi	Boys	1997	17	13.10
Burundi	Boys	1998	17	13.18
Burundi	Boys	1999	17	13.26
Burundi	Boys	2000	17	13.34
Burundi	Boys	2001	17	13.42
Burundi	Boys	2002	17	13.51
Burundi	Boys	2003	17	13.60
Burundi	Boys	2004	17	13.69
Burundi	Boys	2005	17	13.79
Burundi	Boys	2006	17	13.89
Burundi	Boys	2007	17	13.99
Burundi	Boys	2008	17	14.10
Burundi	Boys	2009	17	14.21
Burundi	Boys	2010	17	14.33
Burundi	Boys	2011	17	14.46
Burundi	Boys	2012	17	14.59
Burundi	Boys	2013	17	14.72
Burundi	Boys	2014	17	14.87
Burundi	Boys	2015	17	15.02
Burundi	Boys	2016	17	15.18
Burundi	Boys	1975	18	13.65
Burundi	Boys	1976	18	13.71
Burundi	Boys	1977	18	13.77
Burundi	Boys	1978	18	13.83
Burundi	Boys	1979	18	13.89
Burundi	Boys	1980	18	13.95
Burundi	Boys	1981	18	14.02
Burundi	Boys	1982	18	14.08
Burundi	Boys	1983	18	14.14
Burundi	Boys	1984	18	14.20
Burundi	Boys	1985	18	14.27
Burundi	Boys	1986	18	14.33
Burundi	Boys	1987	18	14.39
Burundi	Boys	1988	18	14.46
Burundi	Boys	1989	18	14.53
Burundi	Boys	1990	18	14.60
Burundi	Boys	1991	18	14.67
Burundi	Boys	1992	18	14.74
Burundi	Boys	1993	18	14.81

Burundi	Boys	1994	18	14.89
Burundi	Boys	1995	18	14.96
Burundi	Boys	1996	18	15.04
Burundi	Boys	1997	18	15.13
Burundi	Boys	1998	18	15.21
Burundi	Boys	1999	18	15.30
Burundi	Boys	2000	18	15.39
Burundi	Boys	2001	18	15.48
Burundi	Boys	2002	18	15.58
Burundi	Boys	2003	18	15.67
Burundi	Boys	2004	18	15.78
Burundi	Boys	2005	18	15.88
Burundi	Boys	2006	18	15.99
Burundi	Boys	2007	18	16.10
Burundi	Boys	2008	18	16.22
Burundi	Boys	2009	18	16.34
Burundi	Boys	2010	18	16.47
Burundi	Boys	2011	18	16.60
Burundi	Boys	2012	18	16.74
Burundi	Boys	2013	18	16.88
Burundi	Boys	2014	18	17.04
Burundi	Boys	2015	18	17.20
Burundi	Boys	2016	18	17.37
Burundi	Boys	1975	Average	8.44
Burundi	Boys	1976	Average	8.48
Burundi	Boys	1977	Average	8.51
Burundi	Boys	1978	Average	8.55
Burundi	Boys	1979	Average	8.59
Burundi	Boys	1980	Average	8.63
Burundi	Boys	1981	Average	8.67
Burundi	Boys	1982	Average	8.71
Burundi	Boys	1983	Average	8.75
Burundi	Boys	1984	Average	8.79
Burundi	Boys	1985	Average	8.83
Burundi	Boys	1986	Average	8.88
Burundi	Boys	1987	Average	8.92
Burundi	Boys	1988	Average	8.97
Burundi	Boys	1989	Average	9.01
Burundi	Boys	1990	Average	9.06
Burundi	Boys	1991	Average	9.11
Burundi	Boys	1992	Average	9.16
Burundi	Boys	1993	Average	9.22
Burundi	Boys	1994	Average	9.27
Burundi	Boys	1995	Average	9.33
Burundi	Boys	1996	Average	9.39
Burundi	Boys	1997	Average	9.45
Burundi	Boys	1998	Average	9.51
Burundi	Boys	1999	Average	9.58
Burundi	Boys	2000	Average	9.65
Burundi	Boys	2001	Average	9.72
Burundi	Boys	2002	Average	9.79
Burundi	Boys	2003	Average	9.87

Burundi	Boys	2004	Average	9.95
Burundi	Boys	2005	Average	10.03
Burundi	Boys	2006	Average	10.12
Burundi	Boys	2007	Average	10.21
Burundi	Boys	2008	Average	10.31
Burundi	Boys	2009	Average	10.41
Burundi	Boys	2010	Average	10.51
Burundi	Boys	2011	Average	10.62
Burundi	Boys	2012	Average	10.73
Burundi	Boys	2013	Average	10.86
Burundi	Boys	2014	Average	10.99
Burundi	Boys	2015	Average	11.12
Burundi	Boys	2016	Average	11.27
Burundi	Girls	1975	12	4.70
Burundi	Girls	1976	12	4.72
Burundi	Girls	1977	12	4.75
Burundi	Girls	1978	12	4.78
Burundi	Girls	1979	12	4.80
Burundi	Girls	1980	12	4.83
Burundi	Girls	1981	12	4.87
Burundi	Girls	1982	12	4.90
Burundi	Girls	1983	12	4.93
Burundi	Girls	1984	12	4.97
Burundi	Girls	1985	12	5.01
Burundi	Girls	1986	12	5.05
Burundi	Girls	1987	12	5.09
Burundi	Girls	1988	12	5.14
Burundi	Girls	1989	12	5.18
Burundi	Girls	1990	12	5.23
Burundi	Girls	1991	12	5.28
Burundi	Girls	1992	12	5.34
Burundi	Girls	1993	12	5.40
Burundi	Girls	1994	12	5.45
Burundi	Girls	1995	12	5.52
Burundi	Girls	1996	12	5.58
Burundi	Girls	1997	12	5.65
Burundi	Girls	1998	12	5.72
Burundi	Girls	1999	12	5.80
Burundi	Girls	2000	12	5.87
Burundi	Girls	2001	12	5.95
Burundi	Girls	2002	12	6.04
Burundi	Girls	2003	12	6.12
Burundi	Girls	2004	12	6.21
Burundi	Girls	2005	12	6.31
Burundi	Girls	2006	12	6.40
Burundi	Girls	2007	12	6.50
Burundi	Girls	2008	12	6.60
Burundi	Girls	2009	12	6.71
Burundi	Girls	2010	12	6.82
Burundi	Girls	2011	12	6.94
Burundi	Girls	2012	12	7.06
Burundi	Girls	2013	12	7.18

Burundi	Girls	2014	12	7.31
Burundi	Girls	2015	12	7.44
Burundi	Girls	2016	12	7.58
Burundi	Girls	1975	13	5.92
Burundi	Girls	1976	13	5.95
Burundi	Girls	1977	13	5.98
Burundi	Girls	1978	13	6.02
Burundi	Girls	1979	13	6.05
Burundi	Girls	1980	13	6.09
Burundi	Girls	1981	13	6.13
Burundi	Girls	1982	13	6.17
Burundi	Girls	1983	13	6.21
Burundi	Girls	1984	13	6.25
Burundi	Girls	1985	13	6.30
Burundi	Girls	1986	13	6.35
Burundi	Girls	1987	13	6.40
Burundi	Girls	1988	13	6.45
Burundi	Girls	1989	13	6.51
Burundi	Girls	1990	13	6.57
Burundi	Girls	1991	13	6.63
Burundi	Girls	1992	13	6.69
Burundi	Girls	1993	13	6.76
Burundi	Girls	1994	13	6.83
Burundi	Girls	1995	13	6.90
Burundi	Girls	1996	13	6.98
Burundi	Girls	1997	13	7.06
Burundi	Girls	1998	13	7.15
Burundi	Girls	1999	13	7.23
Burundi	Girls	2000	13	7.32
Burundi	Girls	2001	13	7.42
Burundi	Girls	2002	13	7.51
Burundi	Girls	2003	13	7.62
Burundi	Girls	2004	13	7.72
Burundi	Girls	2005	13	7.83
Burundi	Girls	2006	13	7.94
Burundi	Girls	2007	13	8.05
Burundi	Girls	2008	13	8.17
Burundi	Girls	2009	13	8.30
Burundi	Girls	2010	13	8.42
Burundi	Girls	2011	13	8.56
Burundi	Girls	2012	13	8.70
Burundi	Girls	2013	13	8.84
Burundi	Girls	2014	13	8.99
Burundi	Girls	2015	13	9.14
Burundi	Girls	2016	13	9.31
Burundi	Girls	1975	14	7.29
Burundi	Girls	1976	14	7.33
Burundi	Girls	1977	14	7.37
Burundi	Girls	1978	14	7.41
Burundi	Girls	1979	14	7.45
Burundi	Girls	1980	14	7.49
Burundi	Girls	1981	14	7.53

Burundi	Girls	1982	14	7.58
Burundi	Girls	1983	14	7.63
Burundi	Girls	1984	14	7.68
Burundi	Girls	1985	14	7.73
Burundi	Girls	1986	14	7.78
Burundi	Girls	1987	14	7.84
Burundi	Girls	1988	14	7.90
Burundi	Girls	1989	14	7.97
Burundi	Girls	1990	14	8.03
Burundi	Girls	1991	14	8.10
Burundi	Girls	1992	14	8.17
Burundi	Girls	1993	14	8.25
Burundi	Girls	1994	14	8.33
Burundi	Girls	1995	14	8.41
Burundi	Girls	1996	14	8.50
Burundi	Girls	1997	14	8.59
Burundi	Girls	1998	14	8.68
Burundi	Girls	1999	14	8.78
Burundi	Girls	2000	14	8.88
Burundi	Girls	2001	14	8.98
Burundi	Girls	2002	14	9.09
Burundi	Girls	2003	14	9.20
Burundi	Girls	2004	14	9.32
Burundi	Girls	2005	14	9.44
Burundi	Girls	2006	14	9.56
Burundi	Girls	2007	14	9.69
Burundi	Girls	2008	14	9.82
Burundi	Girls	2009	14	9.95
Burundi	Girls	2010	14	10.09
Burundi	Girls	2011	14	10.24
Burundi	Girls	2012	14	10.39
Burundi	Girls	2013	14	10.55
Burundi	Girls	2014	14	10.71
Burundi	Girls	2015	14	10.88
Burundi	Girls	2016	14	11.06
Burundi	Girls	1975	15	8.61
Burundi	Girls	1976	15	8.66
Burundi	Girls	1977	15	8.70
Burundi	Girls	1978	15	8.74
Burundi	Girls	1979	15	8.78
Burundi	Girls	1980	15	8.83
Burundi	Girls	1981	15	8.88
Burundi	Girls	1982	15	8.93
Burundi	Girls	1983	15	8.98
Burundi	Girls	1984	15	9.04
Burundi	Girls	1985	15	9.09
Burundi	Girls	1986	15	9.15
Burundi	Girls	1987	15	9.21
Burundi	Girls	1988	15	9.28
Burundi	Girls	1989	15	9.35
Burundi	Girls	1990	15	9.42
Burundi	Girls	1991	15	9.49

Burundi	Girls	1992	15	9.57
Burundi	Girls	1993	15	9.65
Burundi	Girls	1994	15	9.74
Burundi	Girls	1995	15	9.82
Burundi	Girls	1996	15	9.92
Burundi	Girls	1997	15	10.01
Burundi	Girls	1998	15	10.11
Burundi	Girls	1999	15	10.21
Burundi	Girls	2000	15	10.32
Burundi	Girls	2001	15	10.43
Burundi	Girls	2002	15	10.54
Burundi	Girls	2003	15	10.66
Burundi	Girls	2004	15	10.79
Burundi	Girls	2005	15	10.91
Burundi	Girls	2006	15	11.04
Burundi	Girls	2007	15	11.18
Burundi	Girls	2008	15	11.32
Burundi	Girls	2009	15	11.46
Burundi	Girls	2010	15	11.61
Burundi	Girls	2011	15	11.76
Burundi	Girls	2012	15	11.92
Burundi	Girls	2013	15	12.09
Burundi	Girls	2014	15	12.26
Burundi	Girls	2015	15	12.44
Burundi	Girls	2016	15	12.63
Burundi	Girls	1975	16	9.70
Burundi	Girls	1976	16	9.74
Burundi	Girls	1977	16	9.79
Burundi	Girls	1978	16	9.83
Burundi	Girls	1979	16	9.88
Burundi	Girls	1980	16	9.93
Burundi	Girls	1981	16	9.98
Burundi	Girls	1982	16	10.03
Burundi	Girls	1983	16	10.09
Burundi	Girls	1984	16	10.14
Burundi	Girls	1985	16	10.20
Burundi	Girls	1986	16	10.26
Burundi	Girls	1987	16	10.33
Burundi	Girls	1988	16	10.40
Burundi	Girls	1989	16	10.47
Burundi	Girls	1990	16	10.54
Burundi	Girls	1991	16	10.62
Burundi	Girls	1992	16	10.70
Burundi	Girls	1993	16	10.79
Burundi	Girls	1994	16	10.87
Burundi	Girls	1995	16	10.96
Burundi	Girls	1996	16	11.06
Burundi	Girls	1997	16	11.16
Burundi	Girls	1998	16	11.26
Burundi	Girls	1999	16	11.37
Burundi	Girls	2000	16	11.48
Burundi	Girls	2001	16	11.59

Burundi	Girls	2002	16	11.71
Burundi	Girls	2003	16	11.84
Burundi	Girls	2004	16	11.96
Burundi	Girls	2005	16	12.09
Burundi	Girls	2006	16	12.23
Burundi	Girls	2007	16	12.37
Burundi	Girls	2008	16	12.51
Burundi	Girls	2009	16	12.66
Burundi	Girls	2010	16	12.81
Burundi	Girls	2011	16	12.97
Burundi	Girls	2012	16	13.14
Burundi	Girls	2013	16	13.31
Burundi	Girls	2014	16	13.49
Burundi	Girls	2015	16	13.68
Burundi	Girls	2016	16	13.87
Burundi	Girls	1975	17	10.46
Burundi	Girls	1976	17	10.50
Burundi	Girls	1977	17	10.55
Burundi	Girls	1978	17	10.60
Burundi	Girls	1979	17	10.65
Burundi	Girls	1980	17	10.70
Burundi	Girls	1981	17	10.75
Burundi	Girls	1982	17	10.81
Burundi	Girls	1983	17	10.87
Burundi	Girls	1984	17	10.93
Burundi	Girls	1985	17	10.99
Burundi	Girls	1986	17	11.05
Burundi	Girls	1987	17	11.12
Burundi	Girls	1988	17	11.19
Burundi	Girls	1989	17	11.26
Burundi	Girls	1990	17	11.34
Burundi	Girls	1991	17	11.42
Burundi	Girls	1992	17	11.51
Burundi	Girls	1993	17	11.59
Burundi	Girls	1994	17	11.69
Burundi	Girls	1995	17	11.78
Burundi	Girls	1996	17	11.88
Burundi	Girls	1997	17	11.98
Burundi	Girls	1998	17	12.09
Burundi	Girls	1999	17	12.20
Burundi	Girls	2000	17	12.31
Burundi	Girls	2001	17	12.43
Burundi	Girls	2002	17	12.56
Burundi	Girls	2003	17	12.69
Burundi	Girls	2004	17	12.82
Burundi	Girls	2005	17	12.95
Burundi	Girls	2006	17	13.09
Burundi	Girls	2007	17	13.24
Burundi	Girls	2008	17	13.39
Burundi	Girls	2009	17	13.55
Burundi	Girls	2010	17	13.71
Burundi	Girls	2011	17	13.87

Burundi	Girls	2012	17	14.05
Burundi	Girls	2013	17	14.23
Burundi	Girls	2014	17	14.41
Burundi	Girls	2015	17	14.61
Burundi	Girls	2016	17	14.81
Burundi	Girls	1975	18	10.94
Burundi	Girls	1976	18	10.99
Burundi	Girls	1977	18	11.04
Burundi	Girls	1978	18	11.09
Burundi	Girls	1979	18	11.14
Burundi	Girls	1980	18	11.20
Burundi	Girls	1981	18	11.25
Burundi	Girls	1982	18	11.31
Burundi	Girls	1983	18	11.37
Burundi	Girls	1984	18	11.44
Burundi	Girls	1985	18	11.50
Burundi	Girls	1986	18	11.57
Burundi	Girls	1987	18	11.64
Burundi	Girls	1988	18	11.72
Burundi	Girls	1989	18	11.80
Burundi	Girls	1990	18	11.88
Burundi	Girls	1991	18	11.97
Burundi	Girls	1992	18	12.06
Burundi	Girls	1993	18	12.15
Burundi	Girls	1994	18	12.25
Burundi	Girls	1995	18	12.35
Burundi	Girls	1996	18	12.46
Burundi	Girls	1997	18	12.57
Burundi	Girls	1998	18	12.68
Burundi	Girls	1999	18	12.80
Burundi	Girls	2000	18	12.92
Burundi	Girls	2001	18	13.05
Burundi	Girls	2002	18	13.18
Burundi	Girls	2003	18	13.32
Burundi	Girls	2004	18	13.46
Burundi	Girls	2005	18	13.61
Burundi	Girls	2006	18	13.76
Burundi	Girls	2007	18	13.92
Burundi	Girls	2008	18	14.08
Burundi	Girls	2009	18	14.25
Burundi	Girls	2010	18	14.42
Burundi	Girls	2011	18	14.60
Burundi	Girls	2012	18	14.79
Burundi	Girls	2013	18	14.98
Burundi	Girls	2014	18	15.19
Burundi	Girls	2015	18	15.40
Burundi	Girls	2016	18	15.62
Burundi	Girls	1975	Average	8.23
Burundi	Girls	1976	Average	8.27
Burundi	Girls	1977	Average	8.31
Burundi	Girls	1978	Average	8.35
Burundi	Girls	1979	Average	8.39

Burundi	Girls	1980	Average	8.44
Burundi	Girls	1981	Average	8.48
Burundi	Girls	1982	Average	8.53
Burundi	Girls	1983	Average	8.58
Burundi	Girls	1984	Average	8.63
Burundi	Girls	1985	Average	8.69
Burundi	Girls	1986	Average	8.75
Burundi	Girls	1987	Average	8.81
Burundi	Girls	1988	Average	8.87
Burundi	Girls	1989	Average	8.93
Burundi	Girls	1990	Average	9.00
Burundi	Girls	1991	Average	9.07
Burundi	Girls	1992	Average	9.15
Burundi	Girls	1993	Average	9.23
Burundi	Girls	1994	Average	9.31
Burundi	Girls	1995	Average	9.39
Burundi	Girls	1996	Average	9.48
Burundi	Girls	1997	Average	9.57
Burundi	Girls	1998	Average	9.67
Burundi	Girls	1999	Average	9.77
Burundi	Girls	2000	Average	9.87
Burundi	Girls	2001	Average	9.98
Burundi	Girls	2002	Average	10.09
Burundi	Girls	2003	Average	10.21
Burundi	Girls	2004	Average	10.33
Burundi	Girls	2005	Average	10.45
Burundi	Girls	2006	Average	10.58
Burundi	Girls	2007	Average	10.71
Burundi	Girls	2008	Average	10.84
Burundi	Girls	2009	Average	10.98
Burundi	Girls	2010	Average	11.13
Burundi	Girls	2011	Average	11.28
Burundi	Girls	2012	Average	11.43
Burundi	Girls	2013	Average	11.60
Burundi	Girls	2014	Average	11.77
Burundi	Girls	2015	Average	11.94
Burundi	Girls	2016	Average	12.13
Cabo Verde	Boys	1975	12	3.99
Cabo Verde	Boys	1976	12	4.00
Cabo Verde	Boys	1977	12	4.02
Cabo Verde	Boys	1978	12	4.04
Cabo Verde	Boys	1979	12	4.06
Cabo Verde	Boys	1980	12	4.08
Cabo Verde	Boys	1981	12	4.10
Cabo Verde	Boys	1982	12	4.13
Cabo Verde	Boys	1983	12	4.15
Cabo Verde	Boys	1984	12	4.18
Cabo Verde	Boys	1985	12	4.22
Cabo Verde	Boys	1986	12	4.25
Cabo Verde	Boys	1987	12	4.30
Cabo Verde	Boys	1988	12	4.34
Cabo Verde	Boys	1989	12	4.40

Cabo Verde	Boys	1990	12	4.46
Cabo Verde	Boys	1991	12	4.52
Cabo Verde	Boys	1992	12	4.58
Cabo Verde	Boys	1993	12	4.64
Cabo Verde	Boys	1994	12	4.71
Cabo Verde	Boys	1995	12	4.77
Cabo Verde	Boys	1996	12	4.84
Cabo Verde	Boys	1997	12	4.90
Cabo Verde	Boys	1998	12	4.97
Cabo Verde	Boys	1999	12	5.04
Cabo Verde	Boys	2000	12	5.11
Cabo Verde	Boys	2001	12	5.18
Cabo Verde	Boys	2002	12	5.26
Cabo Verde	Boys	2003	12	5.33
Cabo Verde	Boys	2004	12	5.41
Cabo Verde	Boys	2005	12	5.50
Cabo Verde	Boys	2006	12	5.58
Cabo Verde	Boys	2007	12	5.67
Cabo Verde	Boys	2008	12	5.76
Cabo Verde	Boys	2009	12	5.86
Cabo Verde	Boys	2010	12	5.95
Cabo Verde	Boys	2011	12	6.05
Cabo Verde	Boys	2012	12	6.15
Cabo Verde	Boys	2013	12	6.25
Cabo Verde	Boys	2014	12	6.36
Cabo Verde	Boys	2015	12	6.47
Cabo Verde	Boys	2016	12	6.58
Cabo Verde	Boys	1975	13	4.99
Cabo Verde	Boys	1976	13	5.01
Cabo Verde	Boys	1977	13	5.03
Cabo Verde	Boys	1978	13	5.05
Cabo Verde	Boys	1979	13	5.08
Cabo Verde	Boys	1980	13	5.10
Cabo Verde	Boys	1981	13	5.13
Cabo Verde	Boys	1982	13	5.16
Cabo Verde	Boys	1983	13	5.19
Cabo Verde	Boys	1984	13	5.23
Cabo Verde	Boys	1985	13	5.27
Cabo Verde	Boys	1986	13	5.32
Cabo Verde	Boys	1987	13	5.37
Cabo Verde	Boys	1988	13	5.42
Cabo Verde	Boys	1989	13	5.49
Cabo Verde	Boys	1990	13	5.56
Cabo Verde	Boys	1991	13	5.63
Cabo Verde	Boys	1992	13	5.70
Cabo Verde	Boys	1993	13	5.77
Cabo Verde	Boys	1994	13	5.85
Cabo Verde	Boys	1995	13	5.92
Cabo Verde	Boys	1996	13	6.00
Cabo Verde	Boys	1997	13	6.08
Cabo Verde	Boys	1998	13	6.15
Cabo Verde	Boys	1999	13	6.23

Cabo Verde	Boys	2000	13	6.32
Cabo Verde	Boys	2001	13	6.40
Cabo Verde	Boys	2002	13	6.49
Cabo Verde	Boys	2003	13	6.58
Cabo Verde	Boys	2004	13	6.68
Cabo Verde	Boys	2005	13	6.77
Cabo Verde	Boys	2006	13	6.87
Cabo Verde	Boys	2007	13	6.98
Cabo Verde	Boys	2008	13	7.08
Cabo Verde	Boys	2009	13	7.19
Cabo Verde	Boys	2010	13	7.30
Cabo Verde	Boys	2011	13	7.41
Cabo Verde	Boys	2012	13	7.53
Cabo Verde	Boys	2013	13	7.65
Cabo Verde	Boys	2014	13	7.77
Cabo Verde	Boys	2015	13	7.90
Cabo Verde	Boys	2016	13	8.03
Cabo Verde	Boys	1975	14	6.31
Cabo Verde	Boys	1976	14	6.33
Cabo Verde	Boys	1977	14	6.36
Cabo Verde	Boys	1978	14	6.39
Cabo Verde	Boys	1979	14	6.42
Cabo Verde	Boys	1980	14	6.45
Cabo Verde	Boys	1981	14	6.48
Cabo Verde	Boys	1982	14	6.52
Cabo Verde	Boys	1983	14	6.56
Cabo Verde	Boys	1984	14	6.60
Cabo Verde	Boys	1985	14	6.65
Cabo Verde	Boys	1986	14	6.70
Cabo Verde	Boys	1987	14	6.76
Cabo Verde	Boys	1988	14	6.83
Cabo Verde	Boys	1989	14	6.90
Cabo Verde	Boys	1990	14	6.98
Cabo Verde	Boys	1991	14	7.06
Cabo Verde	Boys	1992	14	7.14
Cabo Verde	Boys	1993	14	7.23
Cabo Verde	Boys	1994	14	7.31
Cabo Verde	Boys	1995	14	7.40
Cabo Verde	Boys	1996	14	7.48
Cabo Verde	Boys	1997	14	7.57
Cabo Verde	Boys	1998	14	7.65
Cabo Verde	Boys	1999	14	7.74
Cabo Verde	Boys	2000	14	7.83
Cabo Verde	Boys	2001	14	7.93
Cabo Verde	Boys	2002	14	8.03
Cabo Verde	Boys	2003	14	8.13
Cabo Verde	Boys	2004	14	8.24
Cabo Verde	Boys	2005	14	8.34
Cabo Verde	Boys	2006	14	8.46
Cabo Verde	Boys	2007	14	8.57
Cabo Verde	Boys	2008	14	8.69
Cabo Verde	Boys	2009	14	8.81

Cabo Verde	Boys	2010	14	8.93
Cabo Verde	Boys	2011	14	9.06
Cabo Verde	Boys	2012	14	9.18
Cabo Verde	Boys	2013	14	9.32
Cabo Verde	Boys	2014	14	9.45
Cabo Verde	Boys	2015	14	9.60
Cabo Verde	Boys	2016	14	9.74
Cabo Verde	Boys	1975	15	7.89
Cabo Verde	Boys	1976	15	7.92
Cabo Verde	Boys	1977	15	7.95
Cabo Verde	Boys	1978	15	7.99
Cabo Verde	Boys	1979	15	8.02
Cabo Verde	Boys	1980	15	8.06
Cabo Verde	Boys	1981	15	8.10
Cabo Verde	Boys	1982	15	8.14
Cabo Verde	Boys	1983	15	8.19
Cabo Verde	Boys	1984	15	8.24
Cabo Verde	Boys	1985	15	8.30
Cabo Verde	Boys	1986	15	8.36
Cabo Verde	Boys	1987	15	8.42
Cabo Verde	Boys	1988	15	8.50
Cabo Verde	Boys	1989	15	8.58
Cabo Verde	Boys	1990	15	8.67
Cabo Verde	Boys	1991	15	8.76
Cabo Verde	Boys	1992	15	8.85
Cabo Verde	Boys	1993	15	8.94
Cabo Verde	Boys	1994	15	9.03
Cabo Verde	Boys	1995	15	9.12
Cabo Verde	Boys	1996	15	9.22
Cabo Verde	Boys	1997	15	9.31
Cabo Verde	Boys	1998	15	9.40
Cabo Verde	Boys	1999	15	9.50
Cabo Verde	Boys	2000	15	9.60
Cabo Verde	Boys	2001	15	9.70
Cabo Verde	Boys	2002	15	9.81
Cabo Verde	Boys	2003	15	9.92
Cabo Verde	Boys	2004	15	10.03
Cabo Verde	Boys	2005	15	10.15
Cabo Verde	Boys	2006	15	10.27
Cabo Verde	Boys	2007	15	10.39
Cabo Verde	Boys	2008	15	10.52
Cabo Verde	Boys	2009	15	10.65
Cabo Verde	Boys	2010	15	10.78
Cabo Verde	Boys	2011	15	10.91
Cabo Verde	Boys	2012	15	11.05
Cabo Verde	Boys	2013	15	11.19
Cabo Verde	Boys	2014	15	11.33
Cabo Verde	Boys	2015	15	11.49
Cabo Verde	Boys	2016	15	11.64
Cabo Verde	Boys	1975	16	9.65
Cabo Verde	Boys	1976	16	9.69
Cabo Verde	Boys	1977	16	9.73

Cabo Verde	Boys	1978	16	9.77
Cabo Verde	Boys	1979	16	9.81
Cabo Verde	Boys	1980	16	9.86
Cabo Verde	Boys	1981	16	9.90
Cabo Verde	Boys	1982	16	9.96
Cabo Verde	Boys	1983	16	10.01
Cabo Verde	Boys	1984	16	10.07
Cabo Verde	Boys	1985	16	10.13
Cabo Verde	Boys	1986	16	10.20
Cabo Verde	Boys	1987	16	10.27
Cabo Verde	Boys	1988	16	10.35
Cabo Verde	Boys	1989	16	10.44
Cabo Verde	Boys	1990	16	10.54
Cabo Verde	Boys	1991	16	10.64
Cabo Verde	Boys	1992	16	10.74
Cabo Verde	Boys	1993	16	10.84
Cabo Verde	Boys	1994	16	10.93
Cabo Verde	Boys	1995	16	11.03
Cabo Verde	Boys	1996	16	11.13
Cabo Verde	Boys	1997	16	11.23
Cabo Verde	Boys	1998	16	11.33
Cabo Verde	Boys	1999	16	11.44
Cabo Verde	Boys	2000	16	11.54
Cabo Verde	Boys	2001	16	11.65
Cabo Verde	Boys	2002	16	11.77
Cabo Verde	Boys	2003	16	11.88
Cabo Verde	Boys	2004	16	12.00
Cabo Verde	Boys	2005	16	12.12
Cabo Verde	Boys	2006	16	12.25
Cabo Verde	Boys	2007	16	12.38
Cabo Verde	Boys	2008	16	12.51
Cabo Verde	Boys	2009	16	12.65
Cabo Verde	Boys	2010	16	12.78
Cabo Verde	Boys	2011	16	12.92
Cabo Verde	Boys	2012	16	13.07
Cabo Verde	Boys	2013	16	13.21
Cabo Verde	Boys	2014	16	13.36
Cabo Verde	Boys	2015	16	13.52
Cabo Verde	Boys	2016	16	13.68
Cabo Verde	Boys	1975	17	11.48
Cabo Verde	Boys	1976	17	11.53
Cabo Verde	Boys	1977	17	11.58
Cabo Verde	Boys	1978	17	11.63
Cabo Verde	Boys	1979	17	11.68
Cabo Verde	Boys	1980	17	11.73
Cabo Verde	Boys	1981	17	11.78
Cabo Verde	Boys	1982	17	11.84
Cabo Verde	Boys	1983	17	11.91
Cabo Verde	Boys	1984	17	11.97
Cabo Verde	Boys	1985	17	12.04
Cabo Verde	Boys	1986	17	12.12
Cabo Verde	Boys	1987	17	12.20

Cabo Verde	Boys	1988	17	12.29
Cabo Verde	Boys	1989	17	12.39
Cabo Verde	Boys	1990	17	12.50
Cabo Verde	Boys	1991	17	12.60
Cabo Verde	Boys	1992	17	12.71
Cabo Verde	Boys	1993	17	12.81
Cabo Verde	Boys	1994	17	12.92
Cabo Verde	Boys	1995	17	13.03
Cabo Verde	Boys	1996	17	13.13
Cabo Verde	Boys	1997	17	13.24
Cabo Verde	Boys	1998	17	13.35
Cabo Verde	Boys	1999	17	13.46
Cabo Verde	Boys	2000	17	13.57
Cabo Verde	Boys	2001	17	13.69
Cabo Verde	Boys	2002	17	13.81
Cabo Verde	Boys	2003	17	13.93
Cabo Verde	Boys	2004	17	14.06
Cabo Verde	Boys	2005	17	14.19
Cabo Verde	Boys	2006	17	14.32
Cabo Verde	Boys	2007	17	14.45
Cabo Verde	Boys	2008	17	14.59
Cabo Verde	Boys	2009	17	14.73
Cabo Verde	Boys	2010	17	14.88
Cabo Verde	Boys	2011	17	15.02
Cabo Verde	Boys	2012	17	15.17
Cabo Verde	Boys	2013	17	15.32
Cabo Verde	Boys	2014	17	15.48
Cabo Verde	Boys	2015	17	15.64
Cabo Verde	Boys	2016	17	15.81
Cabo Verde	Boys	1975	18	13.28
Cabo Verde	Boys	1976	18	13.33
Cabo Verde	Boys	1977	18	13.39
Cabo Verde	Boys	1978	18	13.45
Cabo Verde	Boys	1979	18	13.51
Cabo Verde	Boys	1980	18	13.57
Cabo Verde	Boys	1981	18	13.63
Cabo Verde	Boys	1982	18	13.70
Cabo Verde	Boys	1983	18	13.77
Cabo Verde	Boys	1984	18	13.84
Cabo Verde	Boys	1985	18	13.92
Cabo Verde	Boys	1986	18	14.01
Cabo Verde	Boys	1987	18	14.10
Cabo Verde	Boys	1988	18	14.20
Cabo Verde	Boys	1989	18	14.31
Cabo Verde	Boys	1990	18	14.43
Cabo Verde	Boys	1991	18	14.55
Cabo Verde	Boys	1992	18	14.66
Cabo Verde	Boys	1993	18	14.78
Cabo Verde	Boys	1994	18	14.90
Cabo Verde	Boys	1995	18	15.01
Cabo Verde	Boys	1996	18	15.13
Cabo Verde	Boys	1997	18	15.24

Cabo Verde	Boys	1998	18	15.36
Cabo Verde	Boys	1999	18	15.48
Cabo Verde	Boys	2000	18	15.60
Cabo Verde	Boys	2001	18	15.73
Cabo Verde	Boys	2002	18	15.86
Cabo Verde	Boys	2003	18	15.99
Cabo Verde	Boys	2004	18	16.13
Cabo Verde	Boys	2005	18	16.27
Cabo Verde	Boys	2006	18	16.41
Cabo Verde	Boys	2007	18	16.56
Cabo Verde	Boys	2008	18	16.70
Cabo Verde	Boys	2009	18	16.85
Cabo Verde	Boys	2010	18	17.01
Cabo Verde	Boys	2011	18	17.16
Cabo Verde	Boys	2012	18	17.32
Cabo Verde	Boys	2013	18	17.48
Cabo Verde	Boys	2014	18	17.65
Cabo Verde	Boys	2015	18	17.82
Cabo Verde	Boys	2016	18	17.99
Cabo Verde	Boys	1975	Average	8.23
Cabo Verde	Boys	1976	Average	8.26
Cabo Verde	Boys	1977	Average	8.30
Cabo Verde	Boys	1978	Average	8.33
Cabo Verde	Boys	1979	Average	8.37
Cabo Verde	Boys	1980	Average	8.41
Cabo Verde	Boys	1981	Average	8.45
Cabo Verde	Boys	1982	Average	8.49
Cabo Verde	Boys	1983	Average	8.54
Cabo Verde	Boys	1984	Average	8.59
Cabo Verde	Boys	1985	Average	8.65
Cabo Verde	Boys	1986	Average	8.71
Cabo Verde	Boys	1987	Average	8.77
Cabo Verde	Boys	1988	Average	8.85
Cabo Verde	Boys	1989	Average	8.93
Cabo Verde	Boys	1990	Average	9.02
Cabo Verde	Boys	1991	Average	9.11
Cabo Verde	Boys	1992	Average	9.20
Cabo Verde	Boys	1993	Average	9.29
Cabo Verde	Boys	1994	Average	9.38
Cabo Verde	Boys	1995	Average	9.47
Cabo Verde	Boys	1996	Average	9.56
Cabo Verde	Boys	1997	Average	9.65
Cabo Verde	Boys	1998	Average	9.75
Cabo Verde	Boys	1999	Average	9.84
Cabo Verde	Boys	2000	Average	9.94
Cabo Verde	Boys	2001	Average	10.04
Cabo Verde	Boys	2002	Average	10.15
Cabo Verde	Boys	2003	Average	10.25
Cabo Verde	Boys	2004	Average	10.36
Cabo Verde	Boys	2005	Average	10.48
Cabo Verde	Boys	2006	Average	10.59
Cabo Verde	Boys	2007	Average	10.71

Cabo Verde	Boys	2008	Average	10.84
Cabo Verde	Boys	2009	Average	10.96
Cabo Verde	Boys	2010	Average	11.09
Cabo Verde	Boys	2011	Average	11.22
Cabo Verde	Boys	2012	Average	11.35
Cabo Verde	Boys	2013	Average	11.49
Cabo Verde	Boys	2014	Average	11.63
Cabo Verde	Boys	2015	Average	11.78
Cabo Verde	Boys	2016	Average	11.93
Cabo Verde	Girls	1975	12	4.76
Cabo Verde	Girls	1976	12	4.79
Cabo Verde	Girls	1977	12	4.82
Cabo Verde	Girls	1978	12	4.85
Cabo Verde	Girls	1979	12	4.89
Cabo Verde	Girls	1980	12	4.92
Cabo Verde	Girls	1981	12	4.96
Cabo Verde	Girls	1982	12	5.00
Cabo Verde	Girls	1983	12	5.05
Cabo Verde	Girls	1984	12	5.11
Cabo Verde	Girls	1985	12	5.17
Cabo Verde	Girls	1986	12	5.24
Cabo Verde	Girls	1987	12	5.32
Cabo Verde	Girls	1988	12	5.40
Cabo Verde	Girls	1989	12	5.50
Cabo Verde	Girls	1990	12	5.60
Cabo Verde	Girls	1991	12	5.70
Cabo Verde	Girls	1992	12	5.80
Cabo Verde	Girls	1993	12	5.90
Cabo Verde	Girls	1994	12	6.00
Cabo Verde	Girls	1995	12	6.10
Cabo Verde	Girls	1996	12	6.19
Cabo Verde	Girls	1997	12	6.29
Cabo Verde	Girls	1998	12	6.38
Cabo Verde	Girls	1999	12	6.47
Cabo Verde	Girls	2000	12	6.57
Cabo Verde	Girls	2001	12	6.66
Cabo Verde	Girls	2002	12	6.76
Cabo Verde	Girls	2003	12	6.86
Cabo Verde	Girls	2004	12	6.95
Cabo Verde	Girls	2005	12	7.05
Cabo Verde	Girls	2006	12	7.15
Cabo Verde	Girls	2007	12	7.25
Cabo Verde	Girls	2008	12	7.34
Cabo Verde	Girls	2009	12	7.43
Cabo Verde	Girls	2010	12	7.53
Cabo Verde	Girls	2011	12	7.62
Cabo Verde	Girls	2012	12	7.71
Cabo Verde	Girls	2013	12	7.81
Cabo Verde	Girls	2014	12	7.90
Cabo Verde	Girls	2015	12	7.99
Cabo Verde	Girls	2016	12	8.08
Cabo Verde	Girls	1975	13	6.00

Cabo Verde	Girls	1976	13	6.03
Cabo Verde	Girls	1977	13	6.07
Cabo Verde	Girls	1978	13	6.11
Cabo Verde	Girls	1979	13	6.15
Cabo Verde	Girls	1980	13	6.19
Cabo Verde	Girls	1981	13	6.24
Cabo Verde	Girls	1982	13	6.29
Cabo Verde	Girls	1983	13	6.35
Cabo Verde	Girls	1984	13	6.42
Cabo Verde	Girls	1985	13	6.49
Cabo Verde	Girls	1986	13	6.57
Cabo Verde	Girls	1987	13	6.66
Cabo Verde	Girls	1988	13	6.76
Cabo Verde	Girls	1989	13	6.87
Cabo Verde	Girls	1990	13	6.99
Cabo Verde	Girls	1991	13	7.11
Cabo Verde	Girls	1992	13	7.22
Cabo Verde	Girls	1993	13	7.34
Cabo Verde	Girls	1994	13	7.46
Cabo Verde	Girls	1995	13	7.57
Cabo Verde	Girls	1996	13	7.68
Cabo Verde	Girls	1997	13	7.79
Cabo Verde	Girls	1998	13	7.89
Cabo Verde	Girls	1999	13	8.00
Cabo Verde	Girls	2000	13	8.11
Cabo Verde	Girls	2001	13	8.22
Cabo Verde	Girls	2002	13	8.33
Cabo Verde	Girls	2003	13	8.44
Cabo Verde	Girls	2004	13	8.55
Cabo Verde	Girls	2005	13	8.66
Cabo Verde	Girls	2006	13	8.77
Cabo Verde	Girls	2007	13	8.88
Cabo Verde	Girls	2008	13	8.99
Cabo Verde	Girls	2009	13	9.10
Cabo Verde	Girls	2010	13	9.20
Cabo Verde	Girls	2011	13	9.31
Cabo Verde	Girls	2012	13	9.42
Cabo Verde	Girls	2013	13	9.52
Cabo Verde	Girls	2014	13	9.63
Cabo Verde	Girls	2015	13	9.73
Cabo Verde	Girls	2016	13	9.84
Cabo Verde	Girls	1975	14	7.38
Cabo Verde	Girls	1976	14	7.42
Cabo Verde	Girls	1977	14	7.46
Cabo Verde	Girls	1978	14	7.51
Cabo Verde	Girls	1979	14	7.55
Cabo Verde	Girls	1980	14	7.60
Cabo Verde	Girls	1981	14	7.65
Cabo Verde	Girls	1982	14	7.72
Cabo Verde	Girls	1983	14	7.78
Cabo Verde	Girls	1984	14	7.86
Cabo Verde	Girls	1985	14	7.94

Cabo Verde	Girls	1986	14	8.03
Cabo Verde	Girls	1987	14	8.13
Cabo Verde	Girls	1988	14	8.24
Cabo Verde	Girls	1989	14	8.36
Cabo Verde	Girls	1990	14	8.50
Cabo Verde	Girls	1991	14	8.63
Cabo Verde	Girls	1992	14	8.76
Cabo Verde	Girls	1993	14	8.88
Cabo Verde	Girls	1994	14	9.01
Cabo Verde	Girls	1995	14	9.13
Cabo Verde	Girls	1996	14	9.25
Cabo Verde	Girls	1997	14	9.37
Cabo Verde	Girls	1998	14	9.49
Cabo Verde	Girls	1999	14	9.61
Cabo Verde	Girls	2000	14	9.72
Cabo Verde	Girls	2001	14	9.84
Cabo Verde	Girls	2002	14	9.96
Cabo Verde	Girls	2003	14	10.08
Cabo Verde	Girls	2004	14	10.20
Cabo Verde	Girls	2005	14	10.32
Cabo Verde	Girls	2006	14	10.44
Cabo Verde	Girls	2007	14	10.56
Cabo Verde	Girls	2008	14	10.68
Cabo Verde	Girls	2009	14	10.80
Cabo Verde	Girls	2010	14	10.91
Cabo Verde	Girls	2011	14	11.03
Cabo Verde	Girls	2012	14	11.14
Cabo Verde	Girls	2013	14	11.26
Cabo Verde	Girls	2014	14	11.37
Cabo Verde	Girls	2015	14	11.49
Cabo Verde	Girls	2016	14	11.60
Cabo Verde	Girls	1975	15	8.71
Cabo Verde	Girls	1976	15	8.75
Cabo Verde	Girls	1977	15	8.80
Cabo Verde	Girls	1978	15	8.85
Cabo Verde	Girls	1979	15	8.90
Cabo Verde	Girls	1980	15	8.95
Cabo Verde	Girls	1981	15	9.01
Cabo Verde	Girls	1982	15	9.07
Cabo Verde	Girls	1983	15	9.15
Cabo Verde	Girls	1984	15	9.23
Cabo Verde	Girls	1985	15	9.31
Cabo Verde	Girls	1986	15	9.41
Cabo Verde	Girls	1987	15	9.52
Cabo Verde	Girls	1988	15	9.64
Cabo Verde	Girls	1989	15	9.77
Cabo Verde	Girls	1990	15	9.91
Cabo Verde	Girls	1991	15	10.05
Cabo Verde	Girls	1992	15	10.18
Cabo Verde	Girls	1993	15	10.32
Cabo Verde	Girls	1994	15	10.45
Cabo Verde	Girls	1995	15	10.58

Cabo Verde	Girls	1996	15	10.71
Cabo Verde	Girls	1997	15	10.83
Cabo Verde	Girls	1998	15	10.95
Cabo Verde	Girls	1999	15	11.08
Cabo Verde	Girls	2000	15	11.20
Cabo Verde	Girls	2001	15	11.32
Cabo Verde	Girls	2002	15	11.45
Cabo Verde	Girls	2003	15	11.58
Cabo Verde	Girls	2004	15	11.70
Cabo Verde	Girls	2005	15	11.83
Cabo Verde	Girls	2006	15	11.95
Cabo Verde	Girls	2007	15	12.08
Cabo Verde	Girls	2008	15	12.20
Cabo Verde	Girls	2009	15	12.32
Cabo Verde	Girls	2010	15	12.44
Cabo Verde	Girls	2011	15	12.56
Cabo Verde	Girls	2012	15	12.68
Cabo Verde	Girls	2013	15	12.80
Cabo Verde	Girls	2014	15	12.92
Cabo Verde	Girls	2015	15	13.04
Cabo Verde	Girls	2016	15	13.16
Cabo Verde	Girls	1975	16	9.79
Cabo Verde	Girls	1976	16	9.84
Cabo Verde	Girls	1977	16	9.89
Cabo Verde	Girls	1978	16	9.94
Cabo Verde	Girls	1979	16	10.00
Cabo Verde	Girls	1980	16	10.05
Cabo Verde	Girls	1981	16	10.11
Cabo Verde	Girls	1982	16	10.18
Cabo Verde	Girls	1983	16	10.26
Cabo Verde	Girls	1984	16	10.34
Cabo Verde	Girls	1985	16	10.43
Cabo Verde	Girls	1986	16	10.53
Cabo Verde	Girls	1987	16	10.65
Cabo Verde	Girls	1988	16	10.77
Cabo Verde	Girls	1989	16	10.91
Cabo Verde	Girls	1990	16	11.05
Cabo Verde	Girls	1991	16	11.19
Cabo Verde	Girls	1992	16	11.34
Cabo Verde	Girls	1993	16	11.48
Cabo Verde	Girls	1994	16	11.61
Cabo Verde	Girls	1995	16	11.75
Cabo Verde	Girls	1996	16	11.88
Cabo Verde	Girls	1997	16	12.01
Cabo Verde	Girls	1998	16	12.13
Cabo Verde	Girls	1999	16	12.26
Cabo Verde	Girls	2000	16	12.39
Cabo Verde	Girls	2001	16	12.51
Cabo Verde	Girls	2002	16	12.64
Cabo Verde	Girls	2003	16	12.77
Cabo Verde	Girls	2004	16	12.90
Cabo Verde	Girls	2005	16	13.03

Cabo Verde	Girls	2006	16	13.16
Cabo Verde	Girls	2007	16	13.29
Cabo Verde	Girls	2008	16	13.42
Cabo Verde	Girls	2009	16	13.54
Cabo Verde	Girls	2010	16	13.67
Cabo Verde	Girls	2011	16	13.79
Cabo Verde	Girls	2012	16	13.91
Cabo Verde	Girls	2013	16	14.04
Cabo Verde	Girls	2014	16	14.16
Cabo Verde	Girls	2015	16	14.28
Cabo Verde	Girls	2016	16	14.41
Cabo Verde	Girls	1975	17	10.56
Cabo Verde	Girls	1976	17	10.61
Cabo Verde	Girls	1977	17	10.66
Cabo Verde	Girls	1978	17	10.72
Cabo Verde	Girls	1979	17	10.77
Cabo Verde	Girls	1980	17	10.83
Cabo Verde	Girls	1981	17	10.90
Cabo Verde	Girls	1982	17	10.97
Cabo Verde	Girls	1983	17	11.05
Cabo Verde	Girls	1984	17	11.14
Cabo Verde	Girls	1985	17	11.23
Cabo Verde	Girls	1986	17	11.34
Cabo Verde	Girls	1987	17	11.45
Cabo Verde	Girls	1988	17	11.58
Cabo Verde	Girls	1989	17	11.72
Cabo Verde	Girls	1990	17	11.88
Cabo Verde	Girls	1991	17	12.03
Cabo Verde	Girls	1992	17	12.17
Cabo Verde	Girls	1993	17	12.32
Cabo Verde	Girls	1994	17	12.46
Cabo Verde	Girls	1995	17	12.60
Cabo Verde	Girls	1996	17	12.74
Cabo Verde	Girls	1997	17	12.87
Cabo Verde	Girls	1998	17	13.01
Cabo Verde	Girls	1999	17	13.14
Cabo Verde	Girls	2000	17	13.27
Cabo Verde	Girls	2001	17	13.40
Cabo Verde	Girls	2002	17	13.54
Cabo Verde	Girls	2003	17	13.67
Cabo Verde	Girls	2004	17	13.81
Cabo Verde	Girls	2005	17	13.95
Cabo Verde	Girls	2006	17	14.08
Cabo Verde	Girls	2007	17	14.21
Cabo Verde	Girls	2008	17	14.35
Cabo Verde	Girls	2009	17	14.48
Cabo Verde	Girls	2010	17	14.61
Cabo Verde	Girls	2011	17	14.73
Cabo Verde	Girls	2012	17	14.86
Cabo Verde	Girls	2013	17	14.99
Cabo Verde	Girls	2014	17	15.12
Cabo Verde	Girls	2015	17	15.25

Cabo Verde	Girls	2016	17	15.38
Cabo Verde	Girls	1975	18	11.05
Cabo Verde	Girls	1976	18	11.10
Cabo Verde	Girls	1977	18	11.16
Cabo Verde	Girls	1978	18	11.22
Cabo Verde	Girls	1979	18	11.28
Cabo Verde	Girls	1980	18	11.34
Cabo Verde	Girls	1981	18	11.41
Cabo Verde	Girls	1982	18	11.49
Cabo Verde	Girls	1983	18	11.57
Cabo Verde	Girls	1984	18	11.67
Cabo Verde	Girls	1985	18	11.77
Cabo Verde	Girls	1986	18	11.88
Cabo Verde	Girls	1987	18	12.01
Cabo Verde	Girls	1988	18	12.15
Cabo Verde	Girls	1989	18	12.30
Cabo Verde	Girls	1990	18	12.46
Cabo Verde	Girls	1991	18	12.62
Cabo Verde	Girls	1992	18	12.78
Cabo Verde	Girls	1993	18	12.94
Cabo Verde	Girls	1994	18	13.10
Cabo Verde	Girls	1995	18	13.25
Cabo Verde	Girls	1996	18	13.40
Cabo Verde	Girls	1997	18	13.55
Cabo Verde	Girls	1998	18	13.69
Cabo Verde	Girls	1999	18	13.83
Cabo Verde	Girls	2000	18	13.98
Cabo Verde	Girls	2001	18	14.12
Cabo Verde	Girls	2002	18	14.27
Cabo Verde	Girls	2003	18	14.42
Cabo Verde	Girls	2004	18	14.57
Cabo Verde	Girls	2005	18	14.71
Cabo Verde	Girls	2006	18	14.86
Cabo Verde	Girls	2007	18	15.00
Cabo Verde	Girls	2008	18	15.15
Cabo Verde	Girls	2009	18	15.29
Cabo Verde	Girls	2010	18	15.43
Cabo Verde	Girls	2011	18	15.57
Cabo Verde	Girls	2012	18	15.71
Cabo Verde	Girls	2013	18	15.85
Cabo Verde	Girls	2014	18	15.99
Cabo Verde	Girls	2015	18	16.13
Cabo Verde	Girls	2016	18	16.27
Cabo Verde	Girls	1975	Average	8.32
Cabo Verde	Girls	1976	Average	8.37
Cabo Verde	Girls	1977	Average	8.41
Cabo Verde	Girls	1978	Average	8.46
Cabo Verde	Girls	1979	Average	8.50
Cabo Verde	Girls	1980	Average	8.55
Cabo Verde	Girls	1981	Average	8.61
Cabo Verde	Girls	1982	Average	8.67
Cabo Verde	Girls	1983	Average	8.74

Cabo Verde	Girls	1984	Average	8.82
Cabo Verde	Girls	1985	Average	8.91
Cabo Verde	Girls	1986	Average	9.00
Cabo Verde	Girls	1987	Average	9.10
Cabo Verde	Girls	1988	Average	9.22
Cabo Verde	Girls	1989	Average	9.35
Cabo Verde	Girls	1990	Average	9.48
Cabo Verde	Girls	1991	Average	9.62
Cabo Verde	Girls	1992	Average	9.75
Cabo Verde	Girls	1993	Average	9.88
Cabo Verde	Girls	1994	Average	10.01
Cabo Verde	Girls	1995	Average	10.14
Cabo Verde	Girls	1996	Average	10.26
Cabo Verde	Girls	1997	Average	10.39
Cabo Verde	Girls	1998	Average	10.51
Cabo Verde	Girls	1999	Average	10.63
Cabo Verde	Girls	2000	Average	10.75
Cabo Verde	Girls	2001	Average	10.87
Cabo Verde	Girls	2002	Average	10.99
Cabo Verde	Girls	2003	Average	11.12
Cabo Verde	Girls	2004	Average	11.24
Cabo Verde	Girls	2005	Average	11.37
Cabo Verde	Girls	2006	Average	11.49
Cabo Verde	Girls	2007	Average	11.61
Cabo Verde	Girls	2008	Average	11.73
Cabo Verde	Girls	2009	Average	11.85
Cabo Verde	Girls	2010	Average	11.97
Cabo Verde	Girls	2011	Average	12.09
Cabo Verde	Girls	2012	Average	12.21
Cabo Verde	Girls	2013	Average	12.32
Cabo Verde	Girls	2014	Average	12.44
Cabo Verde	Girls	2015	Average	12.56
Cabo Verde	Girls	2016	Average	12.68
Cambodia	Boys	1975	12	3.99
Cambodia	Boys	1976	12	3.99
Cambodia	Boys	1977	12	4.00
Cambodia	Boys	1978	12	4.01
Cambodia	Boys	1979	12	4.02
Cambodia	Boys	1980	12	4.03
Cambodia	Boys	1981	12	4.05
Cambodia	Boys	1982	12	4.07
Cambodia	Boys	1983	12	4.09
Cambodia	Boys	1984	12	4.12
Cambodia	Boys	1985	12	4.15
Cambodia	Boys	1986	12	4.18
Cambodia	Boys	1987	12	4.21
Cambodia	Boys	1988	12	4.25
Cambodia	Boys	1989	12	4.28
Cambodia	Boys	1990	12	4.32
Cambodia	Boys	1991	12	4.37
Cambodia	Boys	1992	12	4.41
Cambodia	Boys	1993	12	4.46

Cambodia	Boys	1994	12	4.52
Cambodia	Boys	1995	12	4.58
Cambodia	Boys	1996	12	4.64
Cambodia	Boys	1997	12	4.71
Cambodia	Boys	1998	12	4.79
Cambodia	Boys	1999	12	4.87
Cambodia	Boys	2000	12	4.96
Cambodia	Boys	2001	12	5.06
Cambodia	Boys	2002	12	5.16
Cambodia	Boys	2003	12	5.27
Cambodia	Boys	2004	12	5.39
Cambodia	Boys	2005	12	5.52
Cambodia	Boys	2006	12	5.65
Cambodia	Boys	2007	12	5.80
Cambodia	Boys	2008	12	5.95
Cambodia	Boys	2009	12	6.11
Cambodia	Boys	2010	12	6.29
Cambodia	Boys	2011	12	6.48
Cambodia	Boys	2012	12	6.68
Cambodia	Boys	2013	12	6.89
Cambodia	Boys	2014	12	7.11
Cambodia	Boys	2015	12	7.35
Cambodia	Boys	2016	12	7.60
Cambodia	Boys	1975	13	4.99
Cambodia	Boys	1976	13	4.99
Cambodia	Boys	1977	13	5.00
Cambodia	Boys	1978	13	5.01
Cambodia	Boys	1979	13	5.02
Cambodia	Boys	1980	13	5.04
Cambodia	Boys	1981	13	5.06
Cambodia	Boys	1982	13	5.08
Cambodia	Boys	1983	13	5.11
Cambodia	Boys	1984	13	5.14
Cambodia	Boys	1985	13	5.17
Cambodia	Boys	1986	13	5.21
Cambodia	Boys	1987	13	5.25
Cambodia	Boys	1988	13	5.28
Cambodia	Boys	1989	13	5.33
Cambodia	Boys	1990	13	5.37
Cambodia	Boys	1991	13	5.42
Cambodia	Boys	1992	13	5.47
Cambodia	Boys	1993	13	5.53
Cambodia	Boys	1994	13	5.59
Cambodia	Boys	1995	13	5.66
Cambodia	Boys	1996	13	5.73
Cambodia	Boys	1997	13	5.81
Cambodia	Boys	1998	13	5.90
Cambodia	Boys	1999	13	5.99
Cambodia	Boys	2000	13	6.09
Cambodia	Boys	2001	13	6.20
Cambodia	Boys	2002	13	6.32
Cambodia	Boys	2003	13	6.44

Cambodia	Boys	2004	13	6.58
Cambodia	Boys	2005	13	6.72
Cambodia	Boys	2006	13	6.87
Cambodia	Boys	2007	13	7.04
Cambodia	Boys	2008	13	7.22
Cambodia	Boys	2009	13	7.41
Cambodia	Boys	2010	13	7.61
Cambodia	Boys	2011	13	7.83
Cambodia	Boys	2012	13	8.06
Cambodia	Boys	2013	13	8.30
Cambodia	Boys	2014	13	8.57
Cambodia	Boys	2015	13	8.85
Cambodia	Boys	2016	13	9.15
Cambodia	Boys	1975	14	6.30
Cambodia	Boys	1976	14	6.30
Cambodia	Boys	1977	14	6.31
Cambodia	Boys	1978	14	6.32
Cambodia	Boys	1979	14	6.34
Cambodia	Boys	1980	14	6.36
Cambodia	Boys	1981	14	6.38
Cambodia	Boys	1982	14	6.41
Cambodia	Boys	1983	14	6.44
Cambodia	Boys	1984	14	6.47
Cambodia	Boys	1985	14	6.51
Cambodia	Boys	1986	14	6.55
Cambodia	Boys	1987	14	6.59
Cambodia	Boys	1988	14	6.63
Cambodia	Boys	1989	14	6.68
Cambodia	Boys	1990	14	6.73
Cambodia	Boys	1991	14	6.78
Cambodia	Boys	1992	14	6.84
Cambodia	Boys	1993	14	6.90
Cambodia	Boys	1994	14	6.96
Cambodia	Boys	1995	14	7.04
Cambodia	Boys	1996	14	7.11
Cambodia	Boys	1997	14	7.20
Cambodia	Boys	1998	14	7.29
Cambodia	Boys	1999	14	7.39
Cambodia	Boys	2000	14	7.50
Cambodia	Boys	2001	14	7.62
Cambodia	Boys	2002	14	7.75
Cambodia	Boys	2003	14	7.88
Cambodia	Boys	2004	14	8.03
Cambodia	Boys	2005	14	8.18
Cambodia	Boys	2006	14	8.35
Cambodia	Boys	2007	14	8.53
Cambodia	Boys	2008	14	8.72
Cambodia	Boys	2009	14	8.92
Cambodia	Boys	2010	14	9.15
Cambodia	Boys	2011	14	9.38
Cambodia	Boys	2012	14	9.64
Cambodia	Boys	2013	14	9.91

Cambodia	Boys	2014	14	10.20
Cambodia	Boys	2015	14	10.51
Cambodia	Boys	2016	14	10.84
Cambodia	Boys	1975	15	7.87
Cambodia	Boys	1976	15	7.87
Cambodia	Boys	1977	15	7.88
Cambodia	Boys	1978	15	7.90
Cambodia	Boys	1979	15	7.91
Cambodia	Boys	1980	15	7.94
Cambodia	Boys	1981	15	7.96
Cambodia	Boys	1982	15	7.99
Cambodia	Boys	1983	15	8.03
Cambodia	Boys	1984	15	8.07
Cambodia	Boys	1985	15	8.11
Cambodia	Boys	1986	15	8.15
Cambodia	Boys	1987	15	8.19
Cambodia	Boys	1988	15	8.24
Cambodia	Boys	1989	15	8.29
Cambodia	Boys	1990	15	8.34
Cambodia	Boys	1991	15	8.39
Cambodia	Boys	1992	15	8.45
Cambodia	Boys	1993	15	8.52
Cambodia	Boys	1994	15	8.59
Cambodia	Boys	1995	15	8.66
Cambodia	Boys	1996	15	8.74
Cambodia	Boys	1997	15	8.83
Cambodia	Boys	1998	15	8.93
Cambodia	Boys	1999	15	9.03
Cambodia	Boys	2000	15	9.14
Cambodia	Boys	2001	15	9.26
Cambodia	Boys	2002	15	9.40
Cambodia	Boys	2003	15	9.54
Cambodia	Boys	2004	15	9.69
Cambodia	Boys	2005	15	9.85
Cambodia	Boys	2006	15	10.02
Cambodia	Boys	2007	15	10.20
Cambodia	Boys	2008	15	10.40
Cambodia	Boys	2009	15	10.62
Cambodia	Boys	2010	15	10.85
Cambodia	Boys	2011	15	11.10
Cambodia	Boys	2012	15	11.36
Cambodia	Boys	2013	15	11.65
Cambodia	Boys	2014	15	11.96
Cambodia	Boys	2015	15	12.28
Cambodia	Boys	2016	15	12.63
Cambodia	Boys	1975	16	9.62
Cambodia	Boys	1976	16	9.62
Cambodia	Boys	1977	16	9.63
Cambodia	Boys	1978	16	9.65
Cambodia	Boys	1979	16	9.67
Cambodia	Boys	1980	16	9.70
Cambodia	Boys	1981	16	9.73

Cambodia	Boys	1982	16	9.76
Cambodia	Boys	1983	16	9.80
Cambodia	Boys	1984	16	9.84
Cambodia	Boys	1985	16	9.89
Cambodia	Boys	1986	16	9.93
Cambodia	Boys	1987	16	9.98
Cambodia	Boys	1988	16	10.03
Cambodia	Boys	1989	16	10.08
Cambodia	Boys	1990	16	10.13
Cambodia	Boys	1991	16	10.19
Cambodia	Boys	1992	16	10.25
Cambodia	Boys	1993	16	10.31
Cambodia	Boys	1994	16	10.39
Cambodia	Boys	1995	16	10.46
Cambodia	Boys	1996	16	10.55
Cambodia	Boys	1997	16	10.64
Cambodia	Boys	1998	16	10.73
Cambodia	Boys	1999	16	10.84
Cambodia	Boys	2000	16	10.95
Cambodia	Boys	2001	16	11.08
Cambodia	Boys	2002	16	11.21
Cambodia	Boys	2003	16	11.35
Cambodia	Boys	2004	16	11.51
Cambodia	Boys	2005	16	11.67
Cambodia	Boys	2006	16	11.84
Cambodia	Boys	2007	16	12.03
Cambodia	Boys	2008	16	12.24
Cambodia	Boys	2009	16	12.45
Cambodia	Boys	2010	16	12.69
Cambodia	Boys	2011	16	12.94
Cambodia	Boys	2012	16	13.21
Cambodia	Boys	2013	16	13.51
Cambodia	Boys	2014	16	13.82
Cambodia	Boys	2015	16	14.16
Cambodia	Boys	2016	16	14.52
Cambodia	Boys	1975	17	11.43
Cambodia	Boys	1976	17	11.44
Cambodia	Boys	1977	17	11.45
Cambodia	Boys	1978	17	11.47
Cambodia	Boys	1979	17	11.50
Cambodia	Boys	1980	17	11.52
Cambodia	Boys	1981	17	11.56
Cambodia	Boys	1982	17	11.60
Cambodia	Boys	1983	17	11.64
Cambodia	Boys	1984	17	11.69
Cambodia	Boys	1985	17	11.74
Cambodia	Boys	1986	17	11.78
Cambodia	Boys	1987	17	11.83
Cambodia	Boys	1988	17	11.89
Cambodia	Boys	1989	17	11.94
Cambodia	Boys	1990	17	12.00
Cambodia	Boys	1991	17	12.06

Cambodia	Boys	1992	17	12.12
Cambodia	Boys	1993	17	12.19
Cambodia	Boys	1994	17	12.26
Cambodia	Boys	1995	17	12.34
Cambodia	Boys	1996	17	12.43
Cambodia	Boys	1997	17	12.52
Cambodia	Boys	1998	17	12.62
Cambodia	Boys	1999	17	12.73
Cambodia	Boys	2000	17	12.85
Cambodia	Boys	2001	17	12.97
Cambodia	Boys	2002	17	13.11
Cambodia	Boys	2003	17	13.26
Cambodia	Boys	2004	17	13.41
Cambodia	Boys	2005	17	13.58
Cambodia	Boys	2006	17	13.76
Cambodia	Boys	2007	17	13.95
Cambodia	Boys	2008	17	14.16
Cambodia	Boys	2009	17	14.38
Cambodia	Boys	2010	17	14.62
Cambodia	Boys	2011	17	14.88
Cambodia	Boys	2012	17	15.16
Cambodia	Boys	2013	17	15.46
Cambodia	Boys	2014	17	15.78
Cambodia	Boys	2015	17	16.12
Cambodia	Boys	2016	17	16.49
Cambodia	Boys	1975	18	13.20
Cambodia	Boys	1976	18	13.21
Cambodia	Boys	1977	18	13.23
Cambodia	Boys	1978	18	13.25
Cambodia	Boys	1979	18	13.28
Cambodia	Boys	1980	18	13.31
Cambodia	Boys	1981	18	13.35
Cambodia	Boys	1982	18	13.40
Cambodia	Boys	1983	18	13.44
Cambodia	Boys	1984	18	13.50
Cambodia	Boys	1985	18	13.55
Cambodia	Boys	1986	18	13.60
Cambodia	Boys	1987	18	13.66
Cambodia	Boys	1988	18	13.71
Cambodia	Boys	1989	18	13.77
Cambodia	Boys	1990	18	13.83
Cambodia	Boys	1991	18	13.90
Cambodia	Boys	1992	18	13.97
Cambodia	Boys	1993	18	14.04
Cambodia	Boys	1994	18	14.12
Cambodia	Boys	1995	18	14.21
Cambodia	Boys	1996	18	14.30
Cambodia	Boys	1997	18	14.40
Cambodia	Boys	1998	18	14.51
Cambodia	Boys	1999	18	14.62
Cambodia	Boys	2000	18	14.75
Cambodia	Boys	2001	18	14.88

Cambodia	Boys	2002	18	15.03
Cambodia	Boys	2003	18	15.18
Cambodia	Boys	2004	18	15.35
Cambodia	Boys	2005	18	15.53
Cambodia	Boys	2006	18	15.72
Cambodia	Boys	2007	18	15.92
Cambodia	Boys	2008	18	16.14
Cambodia	Boys	2009	18	16.38
Cambodia	Boys	2010	18	16.63
Cambodia	Boys	2011	18	16.90
Cambodia	Boys	2012	18	17.19
Cambodia	Boys	2013	18	17.51
Cambodia	Boys	2014	18	17.84
Cambodia	Boys	2015	18	18.20
Cambodia	Boys	2016	18	18.59
Cambodia	Boys	1975	Average	8.20
Cambodia	Boys	1976	Average	8.21
Cambodia	Boys	1977	Average	8.21
Cambodia	Boys	1978	Average	8.23
Cambodia	Boys	1979	Average	8.25
Cambodia	Boys	1980	Average	8.27
Cambodia	Boys	1981	Average	8.30
Cambodia	Boys	1982	Average	8.33
Cambodia	Boys	1983	Average	8.36
Cambodia	Boys	1984	Average	8.40
Cambodia	Boys	1985	Average	8.44
Cambodia	Boys	1986	Average	8.49
Cambodia	Boys	1987	Average	8.53
Cambodia	Boys	1988	Average	8.58
Cambodia	Boys	1989	Average	8.62
Cambodia	Boys	1990	Average	8.67
Cambodia	Boys	1991	Average	8.73
Cambodia	Boys	1992	Average	8.79
Cambodia	Boys	1993	Average	8.85
Cambodia	Boys	1994	Average	8.92
Cambodia	Boys	1995	Average	8.99
Cambodia	Boys	1996	Average	9.07
Cambodia	Boys	1997	Average	9.16
Cambodia	Boys	1998	Average	9.25
Cambodia	Boys	1999	Average	9.35
Cambodia	Boys	2000	Average	9.46
Cambodia	Boys	2001	Average	9.58
Cambodia	Boys	2002	Average	9.71
Cambodia	Boys	2003	Average	9.85
Cambodia	Boys	2004	Average	9.99
Cambodia	Boys	2005	Average	10.15
Cambodia	Boys	2006	Average	10.32
Cambodia	Boys	2007	Average	10.50
Cambodia	Boys	2008	Average	10.69
Cambodia	Boys	2009	Average	10.90
Cambodia	Boys	2010	Average	11.12
Cambodia	Boys	2011	Average	11.36

Cambodia	Boys	2012	Average	11.61
Cambodia	Boys	2013	Average	11.89
Cambodia	Boys	2014	Average	12.18
Cambodia	Boys	2015	Average	12.50
Cambodia	Boys	2016	Average	12.83
Cambodia	Girls	1975	12	4.45
Cambodia	Girls	1976	12	4.45
Cambodia	Girls	1977	12	4.45
Cambodia	Girls	1978	12	4.46
Cambodia	Girls	1979	12	4.47
Cambodia	Girls	1980	12	4.48
Cambodia	Girls	1981	12	4.49
Cambodia	Girls	1982	12	4.51
Cambodia	Girls	1983	12	4.53
Cambodia	Girls	1984	12	4.55
Cambodia	Girls	1985	12	4.57
Cambodia	Girls	1986	12	4.59
Cambodia	Girls	1987	12	4.61
Cambodia	Girls	1988	12	4.63
Cambodia	Girls	1989	12	4.66
Cambodia	Girls	1990	12	4.68
Cambodia	Girls	1991	12	4.71
Cambodia	Girls	1992	12	4.73
Cambodia	Girls	1993	12	4.76
Cambodia	Girls	1994	12	4.79
Cambodia	Girls	1995	12	4.82
Cambodia	Girls	1996	12	4.86
Cambodia	Girls	1997	12	4.89
Cambodia	Girls	1998	12	4.93
Cambodia	Girls	1999	12	4.97
Cambodia	Girls	2000	12	5.01
Cambodia	Girls	2001	12	5.06
Cambodia	Girls	2002	12	5.10
Cambodia	Girls	2003	12	5.15
Cambodia	Girls	2004	12	5.20
Cambodia	Girls	2005	12	5.26
Cambodia	Girls	2006	12	5.31
Cambodia	Girls	2007	12	5.37
Cambodia	Girls	2008	12	5.43
Cambodia	Girls	2009	12	5.50
Cambodia	Girls	2010	12	5.57
Cambodia	Girls	2011	12	5.64
Cambodia	Girls	2012	12	5.71
Cambodia	Girls	2013	12	5.80
Cambodia	Girls	2014	12	5.88
Cambodia	Girls	2015	12	5.97
Cambodia	Girls	2016	12	6.06
Cambodia	Girls	1975	13	5.61
Cambodia	Girls	1976	13	5.61
Cambodia	Girls	1977	13	5.61
Cambodia	Girls	1978	13	5.62
Cambodia	Girls	1979	13	5.63

Cambodia	Girls	1980	13	5.65
Cambodia	Girls	1981	13	5.66
Cambodia	Girls	1982	13	5.68
Cambodia	Girls	1983	13	5.71
Cambodia	Girls	1984	13	5.73
Cambodia	Girls	1985	13	5.76
Cambodia	Girls	1986	13	5.78
Cambodia	Girls	1987	13	5.81
Cambodia	Girls	1988	13	5.84
Cambodia	Girls	1989	13	5.86
Cambodia	Girls	1990	13	5.89
Cambodia	Girls	1991	13	5.92
Cambodia	Girls	1992	13	5.96
Cambodia	Girls	1993	13	5.99
Cambodia	Girls	1994	13	6.03
Cambodia	Girls	1995	13	6.06
Cambodia	Girls	1996	13	6.10
Cambodia	Girls	1997	13	6.15
Cambodia	Girls	1998	13	6.19
Cambodia	Girls	1999	13	6.24
Cambodia	Girls	2000	13	6.29
Cambodia	Girls	2001	13	6.34
Cambodia	Girls	2002	13	6.39
Cambodia	Girls	2003	13	6.45
Cambodia	Girls	2004	13	6.51
Cambodia	Girls	2005	13	6.57
Cambodia	Girls	2006	13	6.64
Cambodia	Girls	2007	13	6.71
Cambodia	Girls	2008	13	6.78
Cambodia	Girls	2009	13	6.85
Cambodia	Girls	2010	13	6.93
Cambodia	Girls	2011	13	7.02
Cambodia	Girls	2012	13	7.10
Cambodia	Girls	2013	13	7.20
Cambodia	Girls	2014	13	7.30
Cambodia	Girls	2015	13	7.40
Cambodia	Girls	2016	13	7.51
Cambodia	Girls	1975	14	6.92
Cambodia	Girls	1976	14	6.92
Cambodia	Girls	1977	14	6.92
Cambodia	Girls	1978	14	6.93
Cambodia	Girls	1979	14	6.95
Cambodia	Girls	1980	14	6.96
Cambodia	Girls	1981	14	6.99
Cambodia	Girls	1982	14	7.01
Cambodia	Girls	1983	14	7.04
Cambodia	Girls	1984	14	7.06
Cambodia	Girls	1985	14	7.09
Cambodia	Girls	1986	14	7.12
Cambodia	Girls	1987	14	7.15
Cambodia	Girls	1988	14	7.18
Cambodia	Girls	1989	14	7.22

Cambodia	Girls	1990	14	7.25
Cambodia	Girls	1991	14	7.28
Cambodia	Girls	1992	14	7.32
Cambodia	Girls	1993	14	7.36
Cambodia	Girls	1994	14	7.40
Cambodia	Girls	1995	14	7.44
Cambodia	Girls	1996	14	7.49
Cambodia	Girls	1997	14	7.53
Cambodia	Girls	1998	14	7.58
Cambodia	Girls	1999	14	7.64
Cambodia	Girls	2000	14	7.69
Cambodia	Girls	2001	14	7.75
Cambodia	Girls	2002	14	7.81
Cambodia	Girls	2003	14	7.87
Cambodia	Girls	2004	14	7.94
Cambodia	Girls	2005	14	8.01
Cambodia	Girls	2006	14	8.08
Cambodia	Girls	2007	14	8.16
Cambodia	Girls	2008	14	8.24
Cambodia	Girls	2009	14	8.32
Cambodia	Girls	2010	14	8.41
Cambodia	Girls	2011	14	8.50
Cambodia	Girls	2012	14	8.60
Cambodia	Girls	2013	14	8.70
Cambodia	Girls	2014	14	8.81
Cambodia	Girls	2015	14	8.92
Cambodia	Girls	2016	14	9.04
Cambodia	Girls	1975	15	8.18
Cambodia	Girls	1976	15	8.18
Cambodia	Girls	1977	15	8.19
Cambodia	Girls	1978	15	8.20
Cambodia	Girls	1979	15	8.22
Cambodia	Girls	1980	15	8.24
Cambodia	Girls	1981	15	8.26
Cambodia	Girls	1982	15	8.29
Cambodia	Girls	1983	15	8.31
Cambodia	Girls	1984	15	8.35
Cambodia	Girls	1985	15	8.38
Cambodia	Girls	1986	15	8.41
Cambodia	Girls	1987	15	8.45
Cambodia	Girls	1988	15	8.48
Cambodia	Girls	1989	15	8.51
Cambodia	Girls	1990	15	8.55
Cambodia	Girls	1991	15	8.59
Cambodia	Girls	1992	15	8.63
Cambodia	Girls	1993	15	8.67
Cambodia	Girls	1994	15	8.71
Cambodia	Girls	1995	15	8.76
Cambodia	Girls	1996	15	8.81
Cambodia	Girls	1997	15	8.86
Cambodia	Girls	1998	15	8.91
Cambodia	Girls	1999	15	8.97

Cambodia	Girls	2000	15	9.03
Cambodia	Girls	2001	15	9.09
Cambodia	Girls	2002	15	9.15
Cambodia	Girls	2003	15	9.22
Cambodia	Girls	2004	15	9.29
Cambodia	Girls	2005	15	9.36
Cambodia	Girls	2006	15	9.44
Cambodia	Girls	2007	15	9.52
Cambodia	Girls	2008	15	9.61
Cambodia	Girls	2009	15	9.69
Cambodia	Girls	2010	15	9.79
Cambodia	Girls	2011	15	9.88
Cambodia	Girls	2012	15	9.98
Cambodia	Girls	2013	15	10.09
Cambodia	Girls	2014	15	10.21
Cambodia	Girls	2015	15	10.33
Cambodia	Girls	2016	15	10.45
Cambodia	Girls	1975	16	9.21
Cambodia	Girls	1976	16	9.22
Cambodia	Girls	1977	16	9.23
Cambodia	Girls	1978	16	9.24
Cambodia	Girls	1979	16	9.26
Cambodia	Girls	1980	16	9.28
Cambodia	Girls	1981	16	9.30
Cambodia	Girls	1982	16	9.33
Cambodia	Girls	1983	16	9.36
Cambodia	Girls	1984	16	9.40
Cambodia	Girls	1985	16	9.43
Cambodia	Girls	1986	16	9.47
Cambodia	Girls	1987	16	9.50
Cambodia	Girls	1988	16	9.54
Cambodia	Girls	1989	16	9.57
Cambodia	Girls	1990	16	9.61
Cambodia	Girls	1991	16	9.65
Cambodia	Girls	1992	16	9.69
Cambodia	Girls	1993	16	9.74
Cambodia	Girls	1994	16	9.78
Cambodia	Girls	1995	16	9.83
Cambodia	Girls	1996	16	9.88
Cambodia	Girls	1997	16	9.93
Cambodia	Girls	1998	16	9.99
Cambodia	Girls	1999	16	10.05
Cambodia	Girls	2000	16	10.11
Cambodia	Girls	2001	16	10.17
Cambodia	Girls	2002	16	10.24
Cambodia	Girls	2003	16	10.31
Cambodia	Girls	2004	16	10.38
Cambodia	Girls	2005	16	10.46
Cambodia	Girls	2006	16	10.54
Cambodia	Girls	2007	16	10.62
Cambodia	Girls	2008	16	10.71
Cambodia	Girls	2009	16	10.80

Cambodia	Girls	2010	16	10.89
Cambodia	Girls	2011	16	10.99
Cambodia	Girls	2012	16	11.10
Cambodia	Girls	2013	16	11.21
Cambodia	Girls	2014	16	11.33
Cambodia	Girls	2015	16	11.45
Cambodia	Girls	2016	16	11.58
Cambodia	Girls	1975	17	9.94
Cambodia	Girls	1976	17	9.94
Cambodia	Girls	1977	17	9.95
Cambodia	Girls	1978	17	9.96
Cambodia	Girls	1979	17	9.98
Cambodia	Girls	1980	17	10.01
Cambodia	Girls	1981	17	10.03
Cambodia	Girls	1982	17	10.06
Cambodia	Girls	1983	17	10.10
Cambodia	Girls	1984	17	10.13
Cambodia	Girls	1985	17	10.17
Cambodia	Girls	1986	17	10.20
Cambodia	Girls	1987	17	10.24
Cambodia	Girls	1988	17	10.28
Cambodia	Girls	1989	17	10.32
Cambodia	Girls	1990	17	10.35
Cambodia	Girls	1991	17	10.40
Cambodia	Girls	1992	17	10.44
Cambodia	Girls	1993	17	10.48
Cambodia	Girls	1994	17	10.53
Cambodia	Girls	1995	17	10.58
Cambodia	Girls	1996	17	10.63
Cambodia	Girls	1997	17	10.69
Cambodia	Girls	1998	17	10.74
Cambodia	Girls	1999	17	10.80
Cambodia	Girls	2000	17	10.87
Cambodia	Girls	2001	17	10.93
Cambodia	Girls	2002	17	11.00
Cambodia	Girls	2003	17	11.08
Cambodia	Girls	2004	17	11.15
Cambodia	Girls	2005	17	11.23
Cambodia	Girls	2006	17	11.31
Cambodia	Girls	2007	17	11.40
Cambodia	Girls	2008	17	11.49
Cambodia	Girls	2009	17	11.58
Cambodia	Girls	2010	17	11.68
Cambodia	Girls	2011	17	11.78
Cambodia	Girls	2012	17	11.89
Cambodia	Girls	2013	17	12.01
Cambodia	Girls	2014	17	12.13
Cambodia	Girls	2015	17	12.26
Cambodia	Girls	2016	17	12.39
Cambodia	Girls	1975	18	10.38
Cambodia	Girls	1976	18	10.38
Cambodia	Girls	1977	18	10.39

Cambodia	Girls	1978	18	10.41
Cambodia	Girls	1979	18	10.43
Cambodia	Girls	1980	18	10.45
Cambodia	Girls	1981	18	10.48
Cambodia	Girls	1982	18	10.51
Cambodia	Girls	1983	18	10.54
Cambodia	Girls	1984	18	10.58
Cambodia	Girls	1985	18	10.62
Cambodia	Girls	1986	18	10.66
Cambodia	Girls	1987	18	10.70
Cambodia	Girls	1988	18	10.74
Cambodia	Girls	1989	18	10.78
Cambodia	Girls	1990	18	10.82
Cambodia	Girls	1991	18	10.86
Cambodia	Girls	1992	18	10.91
Cambodia	Girls	1993	18	10.95
Cambodia	Girls	1994	18	11.00
Cambodia	Girls	1995	18	11.05
Cambodia	Girls	1996	18	11.11
Cambodia	Girls	1997	18	11.17
Cambodia	Girls	1998	18	11.23
Cambodia	Girls	1999	18	11.29
Cambodia	Girls	2000	18	11.36
Cambodia	Girls	2001	18	11.43
Cambodia	Girls	2002	18	11.50
Cambodia	Girls	2003	18	11.58
Cambodia	Girls	2004	18	11.66
Cambodia	Girls	2005	18	11.74
Cambodia	Girls	2006	18	11.83
Cambodia	Girls	2007	18	11.92
Cambodia	Girls	2008	18	12.02
Cambodia	Girls	2009	18	12.12
Cambodia	Girls	2010	18	12.22
Cambodia	Girls	2011	18	12.33
Cambodia	Girls	2012	18	12.45
Cambodia	Girls	2013	18	12.57
Cambodia	Girls	2014	18	12.70
Cambodia	Girls	2015	18	12.84
Cambodia	Girls	2016	18	12.98
Cambodia	Girls	1975	Average	7.81
Cambodia	Girls	1976	Average	7.81
Cambodia	Girls	1977	Average	7.82
Cambodia	Girls	1978	Average	7.83
Cambodia	Girls	1979	Average	7.85
Cambodia	Girls	1980	Average	7.87
Cambodia	Girls	1981	Average	7.89
Cambodia	Girls	1982	Average	7.91
Cambodia	Girls	1983	Average	7.94
Cambodia	Girls	1984	Average	7.97
Cambodia	Girls	1985	Average	8.00
Cambodia	Girls	1986	Average	8.03
Cambodia	Girls	1987	Average	8.07

Cambodia	Girls	1988	Average	8.10
Cambodia	Girls	1989	Average	8.13
Cambodia	Girls	1990	Average	8.17
Cambodia	Girls	1991	Average	8.20
Cambodia	Girls	1992	Average	8.24
Cambodia	Girls	1993	Average	8.28
Cambodia	Girls	1994	Average	8.32
Cambodia	Girls	1995	Average	8.36
Cambodia	Girls	1996	Average	8.41
Cambodia	Girls	1997	Average	8.46
Cambodia	Girls	1998	Average	8.51
Cambodia	Girls	1999	Average	8.56
Cambodia	Girls	2000	Average	8.62
Cambodia	Girls	2001	Average	8.68
Cambodia	Girls	2002	Average	8.74
Cambodia	Girls	2003	Average	8.81
Cambodia	Girls	2004	Average	8.88
Cambodia	Girls	2005	Average	8.95
Cambodia	Girls	2006	Average	9.02
Cambodia	Girls	2007	Average	9.10
Cambodia	Girls	2008	Average	9.18
Cambodia	Girls	2009	Average	9.27
Cambodia	Girls	2010	Average	9.36
Cambodia	Girls	2011	Average	9.45
Cambodia	Girls	2012	Average	9.55
Cambodia	Girls	2013	Average	9.65
Cambodia	Girls	2014	Average	9.76
Cambodia	Girls	2015	Average	9.88
Cambodia	Girls	2016	Average	10.00
Cameroon	Boys	1975	12	4.08
Cameroon	Boys	1976	12	4.11
Cameroon	Boys	1977	12	4.14
Cameroon	Boys	1978	12	4.17
Cameroon	Boys	1979	12	4.20
Cameroon	Boys	1980	12	4.23
Cameroon	Boys	1981	12	4.26
Cameroon	Boys	1982	12	4.29
Cameroon	Boys	1983	12	4.32
Cameroon	Boys	1984	12	4.36
Cameroon	Boys	1985	12	4.39
Cameroon	Boys	1986	12	4.43
Cameroon	Boys	1987	12	4.47
Cameroon	Boys	1988	12	4.51
Cameroon	Boys	1989	12	4.55
Cameroon	Boys	1990	12	4.59
Cameroon	Boys	1991	12	4.64
Cameroon	Boys	1992	12	4.68
Cameroon	Boys	1993	12	4.73
Cameroon	Boys	1994	12	4.78
Cameroon	Boys	1995	12	4.83
Cameroon	Boys	1996	12	4.88
Cameroon	Boys	1997	12	4.93

Cameroon	Boys	1998	12	4.99
Cameroon	Boys	1999	12	5.04
Cameroon	Boys	2000	12	5.10
Cameroon	Boys	2001	12	5.16
Cameroon	Boys	2002	12	5.23
Cameroon	Boys	2003	12	5.29
Cameroon	Boys	2004	12	5.36
Cameroon	Boys	2005	12	5.43
Cameroon	Boys	2006	12	5.51
Cameroon	Boys	2007	12	5.58
Cameroon	Boys	2008	12	5.66
Cameroon	Boys	2009	12	5.74
Cameroon	Boys	2010	12	5.82
Cameroon	Boys	2011	12	5.91
Cameroon	Boys	2012	12	5.99
Cameroon	Boys	2013	12	6.08
Cameroon	Boys	2014	12	6.18
Cameroon	Boys	2015	12	6.27
Cameroon	Boys	2016	12	6.38
Cameroon	Boys	1975	13	5.11
Cameroon	Boys	1976	13	5.15
Cameroon	Boys	1977	13	5.18
Cameroon	Boys	1978	13	5.22
Cameroon	Boys	1979	13	5.25
Cameroon	Boys	1980	13	5.29
Cameroon	Boys	1981	13	5.33
Cameroon	Boys	1982	13	5.37
Cameroon	Boys	1983	13	5.41
Cameroon	Boys	1984	13	5.45
Cameroon	Boys	1985	13	5.49
Cameroon	Boys	1986	13	5.54
Cameroon	Boys	1987	13	5.58
Cameroon	Boys	1988	13	5.63
Cameroon	Boys	1989	13	5.68
Cameroon	Boys	1990	13	5.73
Cameroon	Boys	1991	13	5.79
Cameroon	Boys	1992	13	5.84
Cameroon	Boys	1993	13	5.90
Cameroon	Boys	1994	13	5.95
Cameroon	Boys	1995	13	6.01
Cameroon	Boys	1996	13	6.07
Cameroon	Boys	1997	13	6.14
Cameroon	Boys	1998	13	6.20
Cameroon	Boys	1999	13	6.27
Cameroon	Boys	2000	13	6.34
Cameroon	Boys	2001	13	6.41
Cameroon	Boys	2002	13	6.49
Cameroon	Boys	2003	13	6.57
Cameroon	Boys	2004	13	6.65
Cameroon	Boys	2005	13	6.73
Cameroon	Boys	2006	13	6.82
Cameroon	Boys	2007	13	6.90

Cameroon	Boys	2008	13	7.00
Cameroon	Boys	2009	13	7.09
Cameroon	Boys	2010	13	7.18
Cameroon	Boys	2011	13	7.28
Cameroon	Boys	2012	13	7.38
Cameroon	Boys	2013	13	7.49
Cameroon	Boys	2014	13	7.60
Cameroon	Boys	2015	13	7.71
Cameroon	Boys	2016	13	7.83
Cameroon	Boys	1975	14	6.47
Cameroon	Boys	1976	14	6.51
Cameroon	Boys	1977	14	6.55
Cameroon	Boys	1978	14	6.60
Cameroon	Boys	1979	14	6.64
Cameroon	Boys	1980	14	6.69
Cameroon	Boys	1981	14	6.73
Cameroon	Boys	1982	14	6.78
Cameroon	Boys	1983	14	6.83
Cameroon	Boys	1984	14	6.88
Cameroon	Boys	1985	14	6.93
Cameroon	Boys	1986	14	6.98
Cameroon	Boys	1987	14	7.03
Cameroon	Boys	1988	14	7.09
Cameroon	Boys	1989	14	7.15
Cameroon	Boys	1990	14	7.21
Cameroon	Boys	1991	14	7.27
Cameroon	Boys	1992	14	7.33
Cameroon	Boys	1993	14	7.40
Cameroon	Boys	1994	14	7.46
Cameroon	Boys	1995	14	7.53
Cameroon	Boys	1996	14	7.60
Cameroon	Boys	1997	14	7.67
Cameroon	Boys	1998	14	7.75
Cameroon	Boys	1999	14	7.83
Cameroon	Boys	2000	14	7.91
Cameroon	Boys	2001	14	7.99
Cameroon	Boys	2002	14	8.07
Cameroon	Boys	2003	14	8.16
Cameroon	Boys	2004	14	8.25
Cameroon	Boys	2005	14	8.35
Cameroon	Boys	2006	14	8.44
Cameroon	Boys	2007	14	8.54
Cameroon	Boys	2008	14	8.64
Cameroon	Boys	2009	14	8.75
Cameroon	Boys	2010	14	8.85
Cameroon	Boys	2011	14	8.96
Cameroon	Boys	2012	14	9.08
Cameroon	Boys	2013	14	9.19
Cameroon	Boys	2014	14	9.32
Cameroon	Boys	2015	14	9.44
Cameroon	Boys	2016	14	9.58
Cameroon	Boys	1975	15	8.10

Cameroon	Boys	1976	15	8.15
Cameroon	Boys	1977	15	8.20
Cameroon	Boys	1978	15	8.25
Cameroon	Boys	1979	15	8.30
Cameroon	Boys	1980	15	8.36
Cameroon	Boys	1981	15	8.41
Cameroon	Boys	1982	15	8.47
Cameroon	Boys	1983	15	8.52
Cameroon	Boys	1984	15	8.58
Cameroon	Boys	1985	15	8.64
Cameroon	Boys	1986	15	8.70
Cameroon	Boys	1987	15	8.76
Cameroon	Boys	1988	15	8.83
Cameroon	Boys	1989	15	8.89
Cameroon	Boys	1990	15	8.96
Cameroon	Boys	1991	15	9.03
Cameroon	Boys	1992	15	9.10
Cameroon	Boys	1993	15	9.17
Cameroon	Boys	1994	15	9.24
Cameroon	Boys	1995	15	9.32
Cameroon	Boys	1996	15	9.40
Cameroon	Boys	1997	15	9.48
Cameroon	Boys	1998	15	9.56
Cameroon	Boys	1999	15	9.65
Cameroon	Boys	2000	15	9.73
Cameroon	Boys	2001	15	9.82
Cameroon	Boys	2002	15	9.92
Cameroon	Boys	2003	15	10.01
Cameroon	Boys	2004	15	10.11
Cameroon	Boys	2005	15	10.21
Cameroon	Boys	2006	15	10.32
Cameroon	Boys	2007	15	10.42
Cameroon	Boys	2008	15	10.53
Cameroon	Boys	2009	15	10.65
Cameroon	Boys	2010	15	10.76
Cameroon	Boys	2011	15	10.88
Cameroon	Boys	2012	15	11.00
Cameroon	Boys	2013	15	11.12
Cameroon	Boys	2014	15	11.25
Cameroon	Boys	2015	15	11.39
Cameroon	Boys	2016	15	11.53
Cameroon	Boys	1975	16	9.92
Cameroon	Boys	1976	16	9.98
Cameroon	Boys	1977	16	10.04
Cameroon	Boys	1978	16	10.10
Cameroon	Boys	1979	16	10.16
Cameroon	Boys	1980	16	10.22
Cameroon	Boys	1981	16	10.29
Cameroon	Boys	1982	16	10.35
Cameroon	Boys	1983	16	10.41
Cameroon	Boys	1984	16	10.48
Cameroon	Boys	1985	16	10.55

Cameroon	Boys	1986	16	10.62
Cameroon	Boys	1987	16	10.68
Cameroon	Boys	1988	16	10.76
Cameroon	Boys	1989	16	10.83
Cameroon	Boys	1990	16	10.90
Cameroon	Boys	1991	16	10.98
Cameroon	Boys	1992	16	11.06
Cameroon	Boys	1993	16	11.13
Cameroon	Boys	1994	16	11.22
Cameroon	Boys	1995	16	11.30
Cameroon	Boys	1996	16	11.38
Cameroon	Boys	1997	16	11.47
Cameroon	Boys	1998	16	11.56
Cameroon	Boys	1999	16	11.65
Cameroon	Boys	2000	16	11.75
Cameroon	Boys	2001	16	11.84
Cameroon	Boys	2002	16	11.94
Cameroon	Boys	2003	16	12.05
Cameroon	Boys	2004	16	12.15
Cameroon	Boys	2005	16	12.26
Cameroon	Boys	2006	16	12.37
Cameroon	Boys	2007	16	12.48
Cameroon	Boys	2008	16	12.60
Cameroon	Boys	2009	16	12.72
Cameroon	Boys	2010	16	12.84
Cameroon	Boys	2011	16	12.96
Cameroon	Boys	2012	16	13.09
Cameroon	Boys	2013	16	13.22
Cameroon	Boys	2014	16	13.35
Cameroon	Boys	2015	16	13.49
Cameroon	Boys	2016	16	13.64
Cameroon	Boys	1975	17	11.82
Cameroon	Boys	1976	17	11.89
Cameroon	Boys	1977	17	11.96
Cameroon	Boys	1978	17	12.03
Cameroon	Boys	1979	17	12.10
Cameroon	Boys	1980	17	12.18
Cameroon	Boys	1981	17	12.25
Cameroon	Boys	1982	17	12.32
Cameroon	Boys	1983	17	12.39
Cameroon	Boys	1984	17	12.47
Cameroon	Boys	1985	17	12.54
Cameroon	Boys	1986	17	12.62
Cameroon	Boys	1987	17	12.70
Cameroon	Boys	1988	17	12.78
Cameroon	Boys	1989	17	12.86
Cameroon	Boys	1990	17	12.94
Cameroon	Boys	1991	17	13.02
Cameroon	Boys	1992	17	13.11
Cameroon	Boys	1993	17	13.19
Cameroon	Boys	1994	17	13.28
Cameroon	Boys	1995	17	13.37

Cameroon	Boys	1996	17	13.46
Cameroon	Boys	1997	17	13.56
Cameroon	Boys	1998	17	13.65
Cameroon	Boys	1999	17	13.75
Cameroon	Boys	2000	17	13.85
Cameroon	Boys	2001	17	13.96
Cameroon	Boys	2002	17	14.06
Cameroon	Boys	2003	17	14.17
Cameroon	Boys	2004	17	14.28
Cameroon	Boys	2005	17	14.40
Cameroon	Boys	2006	17	14.51
Cameroon	Boys	2007	17	14.63
Cameroon	Boys	2008	17	14.75
Cameroon	Boys	2009	17	14.88
Cameroon	Boys	2010	17	15.00
Cameroon	Boys	2011	17	15.13
Cameroon	Boys	2012	17	15.27
Cameroon	Boys	2013	17	15.40
Cameroon	Boys	2014	17	15.54
Cameroon	Boys	2015	17	15.69
Cameroon	Boys	2016	17	15.84
Cameroon	Boys	1975	18	13.70
Cameroon	Boys	1976	18	13.78
Cameroon	Boys	1977	18	13.86
Cameroon	Boys	1978	18	13.94
Cameroon	Boys	1979	18	14.03
Cameroon	Boys	1980	18	14.11
Cameroon	Boys	1981	18	14.19
Cameroon	Boys	1982	18	14.28
Cameroon	Boys	1983	18	14.36
Cameroon	Boys	1984	18	14.44
Cameroon	Boys	1985	18	14.53
Cameroon	Boys	1986	18	14.61
Cameroon	Boys	1987	18	14.70
Cameroon	Boys	1988	18	14.79
Cameroon	Boys	1989	18	14.88
Cameroon	Boys	1990	18	14.97
Cameroon	Boys	1991	18	15.06
Cameroon	Boys	1992	18	15.15
Cameroon	Boys	1993	18	15.25
Cameroon	Boys	1994	18	15.35
Cameroon	Boys	1995	18	15.44
Cameroon	Boys	1996	18	15.55
Cameroon	Boys	1997	18	15.65
Cameroon	Boys	1998	18	15.75
Cameroon	Boys	1999	18	15.86
Cameroon	Boys	2000	18	15.97
Cameroon	Boys	2001	18	16.09
Cameroon	Boys	2002	18	16.20
Cameroon	Boys	2003	18	16.32
Cameroon	Boys	2004	18	16.44
Cameroon	Boys	2005	18	16.56

Cameroon	Boys	2006	18	16.69
Cameroon	Boys	2007	18	16.81
Cameroon	Boys	2008	18	16.95
Cameroon	Boys	2009	18	17.08
Cameroon	Boys	2010	18	17.21
Cameroon	Boys	2011	18	17.35
Cameroon	Boys	2012	18	17.49
Cameroon	Boys	2013	18	17.64
Cameroon	Boys	2014	18	17.79
Cameroon	Boys	2015	18	17.94
Cameroon	Boys	2016	18	18.10
Cameroon	Boys	1975	Average	8.46
Cameroon	Boys	1976	Average	8.51
Cameroon	Boys	1977	Average	8.56
Cameroon	Boys	1978	Average	8.61
Cameroon	Boys	1979	Average	8.67
Cameroon	Boys	1980	Average	8.72
Cameroon	Boys	1981	Average	8.78
Cameroon	Boys	1982	Average	8.84
Cameroon	Boys	1983	Average	8.89
Cameroon	Boys	1984	Average	8.95
Cameroon	Boys	1985	Average	9.01
Cameroon	Boys	1986	Average	9.07
Cameroon	Boys	1987	Average	9.13
Cameroon	Boys	1988	Average	9.20
Cameroon	Boys	1989	Average	9.26
Cameroon	Boys	1990	Average	9.33
Cameroon	Boys	1991	Average	9.40
Cameroon	Boys	1992	Average	9.47
Cameroon	Boys	1993	Average	9.54
Cameroon	Boys	1994	Average	9.61
Cameroon	Boys	1995	Average	9.69
Cameroon	Boys	1996	Average	9.76
Cameroon	Boys	1997	Average	9.84
Cameroon	Boys	1998	Average	9.92
Cameroon	Boys	1999	Average	10.01
Cameroon	Boys	2000	Average	10.09
Cameroon	Boys	2001	Average	10.18
Cameroon	Boys	2002	Average	10.27
Cameroon	Boys	2003	Average	10.37
Cameroon	Boys	2004	Average	10.46
Cameroon	Boys	2005	Average	10.56
Cameroon	Boys	2006	Average	10.66
Cameroon	Boys	2007	Average	10.77
Cameroon	Boys	2008	Average	10.88
Cameroon	Boys	2009	Average	10.98
Cameroon	Boys	2010	Average	11.10
Cameroon	Boys	2011	Average	11.21
Cameroon	Boys	2012	Average	11.33
Cameroon	Boys	2013	Average	11.45
Cameroon	Boys	2014	Average	11.58
Cameroon	Boys	2015	Average	11.71

Cameroon	Boys	2016	Average	11.84
Cameroon	Girls	1975	12	4.93
Cameroon	Girls	1976	12	4.99
Cameroon	Girls	1977	12	5.04
Cameroon	Girls	1978	12	5.09
Cameroon	Girls	1979	12	5.15
Cameroon	Girls	1980	12	5.21
Cameroon	Girls	1981	12	5.27
Cameroon	Girls	1982	12	5.34
Cameroon	Girls	1983	12	5.40
Cameroon	Girls	1984	12	5.47
Cameroon	Girls	1985	12	5.54
Cameroon	Girls	1986	12	5.61
Cameroon	Girls	1987	12	5.69
Cameroon	Girls	1988	12	5.76
Cameroon	Girls	1989	12	5.84
Cameroon	Girls	1990	12	5.92
Cameroon	Girls	1991	12	6.01
Cameroon	Girls	1992	12	6.09
Cameroon	Girls	1993	12	6.18
Cameroon	Girls	1994	12	6.26
Cameroon	Girls	1995	12	6.35
Cameroon	Girls	1996	12	6.44
Cameroon	Girls	1997	12	6.53
Cameroon	Girls	1998	12	6.62
Cameroon	Girls	1999	12	6.72
Cameroon	Girls	2000	12	6.81
Cameroon	Girls	2001	12	6.91
Cameroon	Girls	2002	12	7.00
Cameroon	Girls	2003	12	7.10
Cameroon	Girls	2004	12	7.20
Cameroon	Girls	2005	12	7.29
Cameroon	Girls	2006	12	7.39
Cameroon	Girls	2007	12	7.48
Cameroon	Girls	2008	12	7.57
Cameroon	Girls	2009	12	7.66
Cameroon	Girls	2010	12	7.75
Cameroon	Girls	2011	12	7.84
Cameroon	Girls	2012	12	7.94
Cameroon	Girls	2013	12	8.03
Cameroon	Girls	2014	12	8.12
Cameroon	Girls	2015	12	8.21
Cameroon	Girls	2016	12	8.30
Cameroon	Girls	1975	13	6.22
Cameroon	Girls	1976	13	6.28
Cameroon	Girls	1977	13	6.35
Cameroon	Girls	1978	13	6.41
Cameroon	Girls	1979	13	6.48
Cameroon	Girls	1980	13	6.56
Cameroon	Girls	1981	13	6.63
Cameroon	Girls	1982	13	6.71
Cameroon	Girls	1983	13	6.79

Cameroon	Girls	1984	13	6.87
Cameroon	Girls	1985	13	6.95
Cameroon	Girls	1986	13	7.04
Cameroon	Girls	1987	13	7.12
Cameroon	Girls	1988	13	7.21
Cameroon	Girls	1989	13	7.31
Cameroon	Girls	1990	13	7.40
Cameroon	Girls	1991	13	7.50
Cameroon	Girls	1992	13	7.60
Cameroon	Girls	1993	13	7.70
Cameroon	Girls	1994	13	7.80
Cameroon	Girls	1995	13	7.90
Cameroon	Girls	1996	13	8.01
Cameroon	Girls	1997	13	8.11
Cameroon	Girls	1998	13	8.22
Cameroon	Girls	1999	13	8.33
Cameroon	Girls	2000	13	8.44
Cameroon	Girls	2001	13	8.55
Cameroon	Girls	2002	13	8.66
Cameroon	Girls	2003	13	8.77
Cameroon	Girls	2004	13	8.88
Cameroon	Girls	2005	13	8.99
Cameroon	Girls	2006	13	9.10
Cameroon	Girls	2007	13	9.20
Cameroon	Girls	2008	13	9.31
Cameroon	Girls	2009	13	9.41
Cameroon	Girls	2010	13	9.52
Cameroon	Girls	2011	13	9.62
Cameroon	Girls	2012	13	9.73
Cameroon	Girls	2013	13	9.83
Cameroon	Girls	2014	13	9.94
Cameroon	Girls	2015	13	10.04
Cameroon	Girls	2016	13	10.15
Cameroon	Girls	1975	14	7.65
Cameroon	Girls	1976	14	7.72
Cameroon	Girls	1977	14	7.80
Cameroon	Girls	1978	14	7.88
Cameroon	Girls	1979	14	7.96
Cameroon	Girls	1980	14	8.04
Cameroon	Girls	1981	14	8.12
Cameroon	Girls	1982	14	8.21
Cameroon	Girls	1983	14	8.30
Cameroon	Girls	1984	14	8.39
Cameroon	Girls	1985	14	8.49
Cameroon	Girls	1986	14	8.59
Cameroon	Girls	1987	14	8.68
Cameroon	Girls	1988	14	8.79
Cameroon	Girls	1989	14	8.89
Cameroon	Girls	1990	14	9.00
Cameroon	Girls	1991	14	9.11
Cameroon	Girls	1992	14	9.22
Cameroon	Girls	1993	14	9.33

Cameroon	Girls	1994	14	9.44
Cameroon	Girls	1995	14	9.56
Cameroon	Girls	1996	14	9.67
Cameroon	Girls	1997	14	9.79
Cameroon	Girls	1998	14	9.91
Cameroon	Girls	1999	14	10.03
Cameroon	Girls	2000	14	10.15
Cameroon	Girls	2001	14	10.27
Cameroon	Girls	2002	14	10.39
Cameroon	Girls	2003	14	10.51
Cameroon	Girls	2004	14	10.63
Cameroon	Girls	2005	14	10.75
Cameroon	Girls	2006	14	10.87
Cameroon	Girls	2007	14	10.98
Cameroon	Girls	2008	14	11.10
Cameroon	Girls	2009	14	11.21
Cameroon	Girls	2010	14	11.33
Cameroon	Girls	2011	14	11.44
Cameroon	Girls	2012	14	11.55
Cameroon	Girls	2013	14	11.67
Cameroon	Girls	2014	14	11.78
Cameroon	Girls	2015	14	11.90
Cameroon	Girls	2016	14	12.01
Cameroon	Girls	1975	15	9.03
Cameroon	Girls	1976	15	9.11
Cameroon	Girls	1977	15	9.19
Cameroon	Girls	1978	15	9.28
Cameroon	Girls	1979	15	9.36
Cameroon	Girls	1980	15	9.45
Cameroon	Girls	1981	15	9.55
Cameroon	Girls	1982	15	9.64
Cameroon	Girls	1983	15	9.74
Cameroon	Girls	1984	15	9.84
Cameroon	Girls	1985	15	9.94
Cameroon	Girls	1986	15	10.05
Cameroon	Girls	1987	15	10.15
Cameroon	Girls	1988	15	10.26
Cameroon	Girls	1989	15	10.38
Cameroon	Girls	1990	15	10.49
Cameroon	Girls	1991	15	10.61
Cameroon	Girls	1992	15	10.73
Cameroon	Girls	1993	15	10.85
Cameroon	Girls	1994	15	10.97
Cameroon	Girls	1995	15	11.09
Cameroon	Girls	1996	15	11.21
Cameroon	Girls	1997	15	11.34
Cameroon	Girls	1998	15	11.46
Cameroon	Girls	1999	15	11.59
Cameroon	Girls	2000	15	11.72
Cameroon	Girls	2001	15	11.85
Cameroon	Girls	2002	15	11.97
Cameroon	Girls	2003	15	12.10

Cameroon	Girls	2004	15	12.23
Cameroon	Girls	2005	15	12.35
Cameroon	Girls	2006	15	12.48
Cameroon	Girls	2007	15	12.60
Cameroon	Girls	2008	15	12.72
Cameroon	Girls	2009	15	12.84
Cameroon	Girls	2010	15	12.96
Cameroon	Girls	2011	15	13.08
Cameroon	Girls	2012	15	13.20
Cameroon	Girls	2013	15	13.32
Cameroon	Girls	2014	15	13.44
Cameroon	Girls	2015	15	13.56
Cameroon	Girls	2016	15	13.68
Cameroon	Girls	1975	16	10.16
Cameroon	Girls	1976	16	10.25
Cameroon	Girls	1977	16	10.34
Cameroon	Girls	1978	16	10.43
Cameroon	Girls	1979	16	10.52
Cameroon	Girls	1980	16	10.62
Cameroon	Girls	1981	16	10.72
Cameroon	Girls	1982	16	10.82
Cameroon	Girls	1983	16	10.92
Cameroon	Girls	1984	16	11.03
Cameroon	Girls	1985	16	11.14
Cameroon	Girls	1986	16	11.25
Cameroon	Girls	1987	16	11.36
Cameroon	Girls	1988	16	11.48
Cameroon	Girls	1989	16	11.60
Cameroon	Girls	1990	16	11.72
Cameroon	Girls	1991	16	11.84
Cameroon	Girls	1992	16	11.97
Cameroon	Girls	1993	16	12.09
Cameroon	Girls	1994	16	12.22
Cameroon	Girls	1995	16	12.35
Cameroon	Girls	1996	16	12.48
Cameroon	Girls	1997	16	12.61
Cameroon	Girls	1998	16	12.74
Cameroon	Girls	1999	16	12.87
Cameroon	Girls	2000	16	13.00
Cameroon	Girls	2001	16	13.14
Cameroon	Girls	2002	16	13.27
Cameroon	Girls	2003	16	13.40
Cameroon	Girls	2004	16	13.54
Cameroon	Girls	2005	16	13.67
Cameroon	Girls	2006	16	13.80
Cameroon	Girls	2007	16	13.92
Cameroon	Girls	2008	16	14.05
Cameroon	Girls	2009	16	14.17
Cameroon	Girls	2010	16	14.30
Cameroon	Girls	2011	16	14.42
Cameroon	Girls	2012	16	14.54
Cameroon	Girls	2013	16	14.67

Cameroon	Girls	2014	16	14.79
Cameroon	Girls	2015	16	14.92
Cameroon	Girls	2016	16	15.05
Cameroon	Girls	1975	17	10.99
Cameroon	Girls	1976	17	11.08
Cameroon	Girls	1977	17	11.18
Cameroon	Girls	1978	17	11.28
Cameroon	Girls	1979	17	11.38
Cameroon	Girls	1980	17	11.48
Cameroon	Girls	1981	17	11.59
Cameroon	Girls	1982	17	11.69
Cameroon	Girls	1983	17	11.80
Cameroon	Girls	1984	17	11.92
Cameroon	Girls	1985	17	12.03
Cameroon	Girls	1986	17	12.15
Cameroon	Girls	1987	17	12.27
Cameroon	Girls	1988	17	12.39
Cameroon	Girls	1989	17	12.52
Cameroon	Girls	1990	17	12.65
Cameroon	Girls	1991	17	12.78
Cameroon	Girls	1992	17	12.91
Cameroon	Girls	1993	17	13.04
Cameroon	Girls	1994	17	13.18
Cameroon	Girls	1995	17	13.31
Cameroon	Girls	1996	17	13.45
Cameroon	Girls	1997	17	13.59
Cameroon	Girls	1998	17	13.73
Cameroon	Girls	1999	17	13.87
Cameroon	Girls	2000	17	14.01
Cameroon	Girls	2001	17	14.15
Cameroon	Girls	2002	17	14.29
Cameroon	Girls	2003	17	14.43
Cameroon	Girls	2004	17	14.57
Cameroon	Girls	2005	17	14.71
Cameroon	Girls	2006	17	14.84
Cameroon	Girls	2007	17	14.98
Cameroon	Girls	2008	17	15.11
Cameroon	Girls	2009	17	15.24
Cameroon	Girls	2010	17	15.37
Cameroon	Girls	2011	17	15.50
Cameroon	Girls	2012	17	15.63
Cameroon	Girls	2013	17	15.76
Cameroon	Girls	2014	17	15.90
Cameroon	Girls	2015	17	16.03
Cameroon	Girls	2016	17	16.16
Cameroon	Girls	1975	18	11.57
Cameroon	Girls	1976	18	11.67
Cameroon	Girls	1977	18	11.78
Cameroon	Girls	1978	18	11.89
Cameroon	Girls	1979	18	12.00
Cameroon	Girls	1980	18	12.11
Cameroon	Girls	1981	18	12.22

Cameroon	Girls	1982	18	12.34
Cameroon	Girls	1983	18	12.46
Cameroon	Girls	1984	18	12.59
Cameroon	Girls	1985	18	12.72
Cameroon	Girls	1986	18	12.85
Cameroon	Girls	1987	18	12.98
Cameroon	Girls	1988	18	13.11
Cameroon	Girls	1989	18	13.25
Cameroon	Girls	1990	18	13.39
Cameroon	Girls	1991	18	13.54
Cameroon	Girls	1992	18	13.68
Cameroon	Girls	1993	18	13.83
Cameroon	Girls	1994	18	13.98
Cameroon	Girls	1995	18	14.13
Cameroon	Girls	1996	18	14.28
Cameroon	Girls	1997	18	14.43
Cameroon	Girls	1998	18	14.58
Cameroon	Girls	1999	18	14.74
Cameroon	Girls	2000	18	14.89
Cameroon	Girls	2001	18	15.05
Cameroon	Girls	2002	18	15.20
Cameroon	Girls	2003	18	15.36
Cameroon	Girls	2004	18	15.51
Cameroon	Girls	2005	18	15.66
Cameroon	Girls	2006	18	15.81
Cameroon	Girls	2007	18	15.96
Cameroon	Girls	2008	18	16.11
Cameroon	Girls	2009	18	16.25
Cameroon	Girls	2010	18	16.39
Cameroon	Girls	2011	18	16.53
Cameroon	Girls	2012	18	16.68
Cameroon	Girls	2013	18	16.82
Cameroon	Girls	2014	18	16.96
Cameroon	Girls	2015	18	17.11
Cameroon	Girls	2016	18	17.25
Cameroon	Girls	1975	Average	8.65
Cameroon	Girls	1976	Average	8.73
Cameroon	Girls	1977	Average	8.81
Cameroon	Girls	1978	Average	8.89
Cameroon	Girls	1979	Average	8.98
Cameroon	Girls	1980	Average	9.07
Cameroon	Girls	1981	Average	9.16
Cameroon	Girls	1982	Average	9.25
Cameroon	Girls	1983	Average	9.35
Cameroon	Girls	1984	Average	9.44
Cameroon	Girls	1985	Average	9.54
Cameroon	Girls	1986	Average	9.65
Cameroon	Girls	1987	Average	9.75
Cameroon	Girls	1988	Average	9.86
Cameroon	Girls	1989	Average	9.97
Cameroon	Girls	1990	Average	10.08
Cameroon	Girls	1991	Average	10.20

Cameroon	Girls	1992	Average	10.31
Cameroon	Girls	1993	Average	10.43
Cameroon	Girls	1994	Average	10.55
Cameroon	Girls	1995	Average	10.67
Cameroon	Girls	1996	Average	10.79
Cameroon	Girls	1997	Average	10.91
Cameroon	Girls	1998	Average	11.04
Cameroon	Girls	1999	Average	11.16
Cameroon	Girls	2000	Average	11.29
Cameroon	Girls	2001	Average	11.42
Cameroon	Girls	2002	Average	11.54
Cameroon	Girls	2003	Average	11.67
Cameroon	Girls	2004	Average	11.79
Cameroon	Girls	2005	Average	11.92
Cameroon	Girls	2006	Average	12.04
Cameroon	Girls	2007	Average	12.16
Cameroon	Girls	2008	Average	12.28
Cameroon	Girls	2009	Average	12.40
Cameroon	Girls	2010	Average	12.52
Cameroon	Girls	2011	Average	12.63
Cameroon	Girls	2012	Average	12.75
Cameroon	Girls	2013	Average	12.87
Cameroon	Girls	2014	Average	12.99
Cameroon	Girls	2015	Average	13.11
Cameroon	Girls	2016	Average	13.23
Canada	Boys	1975	12	8.70
Canada	Boys	1976	12	8.79
Canada	Boys	1977	12	8.89
Canada	Boys	1978	12	8.98
Canada	Boys	1979	12	9.08
Canada	Boys	1980	12	9.19
Canada	Boys	1981	12	9.30
Canada	Boys	1982	12	9.42
Canada	Boys	1983	12	9.54
Canada	Boys	1984	12	9.67
Canada	Boys	1985	12	9.81
Canada	Boys	1986	12	9.95
Canada	Boys	1987	12	10.09
Canada	Boys	1988	12	10.24
Canada	Boys	1989	12	10.38
Canada	Boys	1990	12	10.53
Canada	Boys	1991	12	10.69
Canada	Boys	1992	12	10.85
Canada	Boys	1993	12	11.01
Canada	Boys	1994	12	11.17
Canada	Boys	1995	12	11.34
Canada	Boys	1996	12	11.50
Canada	Boys	1997	12	11.67
Canada	Boys	1998	12	11.84
Canada	Boys	1999	12	12.01
Canada	Boys	2000	12	12.18
Canada	Boys	2001	12	12.34

Canada	Boys	2002	12	12.49
Canada	Boys	2003	12	12.63
Canada	Boys	2004	12	12.76
Canada	Boys	2005	12	12.88
Canada	Boys	2006	12	12.99
Canada	Boys	2007	12	13.10
Canada	Boys	2008	12	13.20
Canada	Boys	2009	12	13.29
Canada	Boys	2010	12	13.37
Canada	Boys	2011	12	13.46
Canada	Boys	2012	12	13.53
Canada	Boys	2013	12	13.61
Canada	Boys	2014	12	13.68
Canada	Boys	2015	12	13.76
Canada	Boys	2016	12	13.83
Canada	Boys	1975	13	10.73
Canada	Boys	1976	13	10.84
Canada	Boys	1977	13	10.95
Canada	Boys	1978	13	11.06
Canada	Boys	1979	13	11.18
Canada	Boys	1980	13	11.30
Canada	Boys	1981	13	11.43
Canada	Boys	1982	13	11.57
Canada	Boys	1983	13	11.72
Canada	Boys	1984	13	11.87
Canada	Boys	1985	13	12.04
Canada	Boys	1986	13	12.20
Canada	Boys	1987	13	12.37
Canada	Boys	1988	13	12.55
Canada	Boys	1989	13	12.73
Canada	Boys	1990	13	12.91
Canada	Boys	1991	13	13.09
Canada	Boys	1992	13	13.28
Canada	Boys	1993	13	13.47
Canada	Boys	1994	13	13.67
Canada	Boys	1995	13	13.86
Canada	Boys	1996	13	14.07
Canada	Boys	1997	13	14.27
Canada	Boys	1998	13	14.47
Canada	Boys	1999	13	14.68
Canada	Boys	2000	13	14.88
Canada	Boys	2001	13	15.07
Canada	Boys	2002	13	15.25
Canada	Boys	2003	13	15.42
Canada	Boys	2004	13	15.58
Canada	Boys	2005	13	15.73
Canada	Boys	2006	13	15.86
Canada	Boys	2007	13	15.99
Canada	Boys	2008	13	16.11
Canada	Boys	2009	13	16.22
Canada	Boys	2010	13	16.32
Canada	Boys	2011	13	16.42

Canada	Boys	2012	13	16.51
Canada	Boys	2013	13	16.60
Canada	Boys	2014	13	16.69
Canada	Boys	2015	13	16.78
Canada	Boys	2016	13	16.87
Canada	Boys	1975	14	13.03
Canada	Boys	1976	14	13.15
Canada	Boys	1977	14	13.27
Canada	Boys	1978	14	13.40
Canada	Boys	1979	14	13.53
Canada	Boys	1980	14	13.66
Canada	Boys	1981	14	13.81
Canada	Boys	1982	14	13.96
Canada	Boys	1983	14	14.13
Canada	Boys	1984	14	14.30
Canada	Boys	1985	14	14.48
Canada	Boys	1986	14	14.67
Canada	Boys	1987	14	14.86
Canada	Boys	1988	14	15.06
Canada	Boys	1989	14	15.25
Canada	Boys	1990	14	15.46
Canada	Boys	1991	14	15.66
Canada	Boys	1992	14	15.88
Canada	Boys	1993	14	16.09
Canada	Boys	1994	14	16.31
Canada	Boys	1995	14	16.53
Canada	Boys	1996	14	16.76
Canada	Boys	1997	14	16.99
Canada	Boys	1998	14	17.22
Canada	Boys	1999	14	17.45
Canada	Boys	2000	14	17.68
Canada	Boys	2001	14	17.90
Canada	Boys	2002	14	18.10
Canada	Boys	2003	14	18.29
Canada	Boys	2004	14	18.47
Canada	Boys	2005	14	18.64
Canada	Boys	2006	14	18.79
Canada	Boys	2007	14	18.93
Canada	Boys	2008	14	19.06
Canada	Boys	2009	14	19.18
Canada	Boys	2010	14	19.30
Canada	Boys	2011	14	19.41
Canada	Boys	2012	14	19.51
Canada	Boys	2013	14	19.61
Canada	Boys	2014	14	19.71
Canada	Boys	2015	14	19.81
Canada	Boys	2016	14	19.92
Canada	Boys	1975	15	15.45
Canada	Boys	1976	15	15.58
Canada	Boys	1977	15	15.71
Canada	Boys	1978	15	15.84
Canada	Boys	1979	15	15.98

Canada	Boys	1980	15	16.12
Canada	Boys	1981	15	16.28
Canada	Boys	1982	15	16.44
Canada	Boys	1983	15	16.61
Canada	Boys	1984	15	16.80
Canada	Boys	1985	15	16.99
Canada	Boys	1986	15	17.19
Canada	Boys	1987	15	17.39
Canada	Boys	1988	15	17.60
Canada	Boys	1989	15	17.81
Canada	Boys	1990	15	18.02
Canada	Boys	1991	15	18.24
Canada	Boys	1992	15	18.47
Canada	Boys	1993	15	18.70
Canada	Boys	1994	15	18.93
Canada	Boys	1995	15	19.17
Canada	Boys	1996	15	19.41
Canada	Boys	1997	15	19.66
Canada	Boys	1998	15	19.90
Canada	Boys	1999	15	20.15
Canada	Boys	2000	15	20.39
Canada	Boys	2001	15	20.62
Canada	Boys	2002	15	20.84
Canada	Boys	2003	15	21.04
Canada	Boys	2004	15	21.23
Canada	Boys	2005	15	21.41
Canada	Boys	2006	15	21.57
Canada	Boys	2007	15	21.72
Canada	Boys	2008	15	21.86
Canada	Boys	2009	15	21.98
Canada	Boys	2010	15	22.10
Canada	Boys	2011	15	22.22
Canada	Boys	2012	15	22.33
Canada	Boys	2013	15	22.43
Canada	Boys	2014	15	22.54
Canada	Boys	2015	15	22.65
Canada	Boys	2016	15	22.76
Canada	Boys	1975	16	17.94
Canada	Boys	1976	16	18.07
Canada	Boys	1977	16	18.20
Canada	Boys	1978	16	18.34
Canada	Boys	1979	16	18.48
Canada	Boys	1980	16	18.63
Canada	Boys	1981	16	18.79
Canada	Boys	1982	16	18.95
Canada	Boys	1983	16	19.13
Canada	Boys	1984	16	19.32
Canada	Boys	1985	16	19.52
Canada	Boys	1986	16	19.72
Canada	Boys	1987	16	19.93
Canada	Boys	1988	16	20.14
Canada	Boys	1989	16	20.36

Canada	Boys	1990	16	20.58
Canada	Boys	1991	16	20.80
Canada	Boys	1992	16	21.04
Canada	Boys	1993	16	21.27
Canada	Boys	1994	16	21.51
Canada	Boys	1995	16	21.75
Canada	Boys	1996	16	22.00
Canada	Boys	1997	16	22.26
Canada	Boys	1998	16	22.51
Canada	Boys	1999	16	22.76
Canada	Boys	2000	16	23.01
Canada	Boys	2001	16	23.25
Canada	Boys	2002	16	23.47
Canada	Boys	2003	16	23.68
Canada	Boys	2004	16	23.88
Canada	Boys	2005	16	24.06
Canada	Boys	2006	16	24.22
Canada	Boys	2007	16	24.37
Canada	Boys	2008	16	24.51
Canada	Boys	2009	16	24.64
Canada	Boys	2010	16	24.76
Canada	Boys	2011	16	24.88
Canada	Boys	2012	16	24.99
Canada	Boys	2013	16	25.10
Canada	Boys	2014	16	25.21
Canada	Boys	2015	16	25.32
Canada	Boys	2016	16	25.43
Canada	Boys	1975	17	20.46
Canada	Boys	1976	17	20.60
Canada	Boys	1977	17	20.73
Canada	Boys	1978	17	20.87
Canada	Boys	1979	17	21.01
Canada	Boys	1980	17	21.16
Canada	Boys	1981	17	21.32
Canada	Boys	1982	17	21.50
Canada	Boys	1983	17	21.68
Canada	Boys	1984	17	21.87
Canada	Boys	1985	17	22.07
Canada	Boys	1986	17	22.27
Canada	Boys	1987	17	22.49
Canada	Boys	1988	17	22.70
Canada	Boys	1989	17	22.92
Canada	Boys	1990	17	23.15
Canada	Boys	1991	17	23.38
Canada	Boys	1992	17	23.62
Canada	Boys	1993	17	23.86
Canada	Boys	1994	17	24.10
Canada	Boys	1995	17	24.35
Canada	Boys	1996	17	24.60
Canada	Boys	1997	17	24.86
Canada	Boys	1998	17	25.12
Canada	Boys	1999	17	25.38

Canada	Boys	2000	17	25.63
Canada	Boys	2001	17	25.88
Canada	Boys	2002	17	26.10
Canada	Boys	2003	17	26.32
Canada	Boys	2004	17	26.52
Canada	Boys	2005	17	26.70
Canada	Boys	2006	17	26.86
Canada	Boys	2007	17	27.02
Canada	Boys	2008	17	27.16
Canada	Boys	2009	17	27.29
Canada	Boys	2010	17	27.42
Canada	Boys	2011	17	27.53
Canada	Boys	2012	17	27.64
Canada	Boys	2013	17	27.76
Canada	Boys	2014	17	27.87
Canada	Boys	2015	17	27.98
Canada	Boys	2016	17	28.10
Canada	Boys	1975	18	23.05
Canada	Boys	1976	18	23.19
Canada	Boys	1977	18	23.33
Canada	Boys	1978	18	23.48
Canada	Boys	1979	18	23.62
Canada	Boys	1980	18	23.78
Canada	Boys	1981	18	23.95
Canada	Boys	1982	18	24.12
Canada	Boys	1983	18	24.31
Canada	Boys	1984	18	24.51
Canada	Boys	1985	18	24.72
Canada	Boys	1986	18	24.93
Canada	Boys	1987	18	25.15
Canada	Boys	1988	18	25.38
Canada	Boys	1989	18	25.61
Canada	Boys	1990	18	25.84
Canada	Boys	1991	18	26.08
Canada	Boys	1992	18	26.32
Canada	Boys	1993	18	26.58
Canada	Boys	1994	18	26.83
Canada	Boys	1995	18	27.09
Canada	Boys	1996	18	27.35
Canada	Boys	1997	18	27.62
Canada	Boys	1998	18	27.88
Canada	Boys	1999	18	28.15
Canada	Boys	2000	18	28.42
Canada	Boys	2001	18	28.67
Canada	Boys	2002	18	28.90
Canada	Boys	2003	18	29.12
Canada	Boys	2004	18	29.33
Canada	Boys	2005	18	29.51
Canada	Boys	2006	18	29.69
Canada	Boys	2007	18	29.85
Canada	Boys	2008	18	29.99
Canada	Boys	2009	18	30.13

Canada	Boys	2010	18	30.26
Canada	Boys	2011	18	30.38
Canada	Boys	2012	18	30.49
Canada	Boys	2013	18	30.61
Canada	Boys	2014	18	30.72
Canada	Boys	2015	18	30.84
Canada	Boys	2016	18	30.96
Canada	Boys	1975	Average	15.62
Canada	Boys	1976	Average	15.75
Canada	Boys	1977	Average	15.87
Canada	Boys	1978	Average	16.00
Canada	Boys	1979	Average	16.13
Canada	Boys	1980	Average	16.26
Canada	Boys	1981	Average	16.41
Canada	Boys	1982	Average	16.57
Canada	Boys	1983	Average	16.73
Canada	Boys	1984	Average	16.91
Canada	Boys	1985	Average	17.09
Canada	Boys	1986	Average	17.28
Canada	Boys	1987	Average	17.47
Canada	Boys	1988	Average	17.67
Canada	Boys	1989	Average	17.87
Canada	Boys	1990	Average	18.07
Canada	Boys	1991	Average	18.28
Canada	Boys	1992	Average	18.49
Canada	Boys	1993	Average	18.71
Canada	Boys	1994	Average	18.93
Canada	Boys	1995	Average	19.16
Canada	Boys	1996	Average	19.38
Canada	Boys	1997	Average	19.62
Canada	Boys	1998	Average	19.85
Canada	Boys	1999	Average	20.08
Canada	Boys	2000	Average	20.31
Canada	Boys	2001	Average	20.53
Canada	Boys	2002	Average	20.74
Canada	Boys	2003	Average	20.93
Canada	Boys	2004	Average	21.11
Canada	Boys	2005	Average	21.27
Canada	Boys	2006	Average	21.43
Canada	Boys	2007	Average	21.57
Canada	Boys	2008	Average	21.70
Canada	Boys	2009	Average	21.82
Canada	Boys	2010	Average	21.93
Canada	Boys	2011	Average	22.04
Canada	Boys	2012	Average	22.14
Canada	Boys	2013	Average	22.25
Canada	Boys	2014	Average	22.35
Canada	Boys	2015	Average	22.45
Canada	Boys	2016	Average	22.55
Canada	Girls	1975	12	8.29
Canada	Girls	1976	12	8.37
Canada	Girls	1977	12	8.44

Canada	Girls	1978	12	8.52
Canada	Girls	1979	12	8.59
Canada	Girls	1980	12	8.67
Canada	Girls	1981	12	8.76
Canada	Girls	1982	12	8.84
Canada	Girls	1983	12	8.94
Canada	Girls	1984	12	9.03
Canada	Girls	1985	12	9.13
Canada	Girls	1986	12	9.23
Canada	Girls	1987	12	9.33
Canada	Girls	1988	12	9.43
Canada	Girls	1989	12	9.53
Canada	Girls	1990	12	9.63
Canada	Girls	1991	12	9.74
Canada	Girls	1992	12	9.84
Canada	Girls	1993	12	9.95
Canada	Girls	1994	12	10.06
Canada	Girls	1995	12	10.16
Canada	Girls	1996	12	10.27
Canada	Girls	1997	12	10.38
Canada	Girls	1998	12	10.48
Canada	Girls	1999	12	10.59
Canada	Girls	2000	12	10.69
Canada	Girls	2001	12	10.78
Canada	Girls	2002	12	10.87
Canada	Girls	2003	12	10.95
Canada	Girls	2004	12	11.02
Canada	Girls	2005	12	11.08
Canada	Girls	2006	12	11.14
Canada	Girls	2007	12	11.19
Canada	Girls	2008	12	11.24
Canada	Girls	2009	12	11.28
Canada	Girls	2010	12	11.33
Canada	Girls	2011	12	11.37
Canada	Girls	2012	12	11.42
Canada	Girls	2013	12	11.46
Canada	Girls	2014	12	11.50
Canada	Girls	2015	12	11.55
Canada	Girls	2016	12	11.60
Canada	Girls	1975	13	10.11
Canada	Girls	1976	13	10.20
Canada	Girls	1977	13	10.28
Canada	Girls	1978	13	10.37
Canada	Girls	1979	13	10.46
Canada	Girls	1980	13	10.55
Canada	Girls	1981	13	10.64
Canada	Girls	1982	13	10.74
Canada	Girls	1983	13	10.85
Canada	Girls	1984	13	10.95
Canada	Girls	1985	13	11.07
Canada	Girls	1986	13	11.18
Canada	Girls	1987	13	11.29

Canada	Girls	1988	13	11.41
Canada	Girls	1989	13	11.52
Canada	Girls	1990	13	11.64
Canada	Girls	1991	13	11.76
Canada	Girls	1992	13	11.88
Canada	Girls	1993	13	12.00
Canada	Girls	1994	13	12.12
Canada	Girls	1995	13	12.24
Canada	Girls	1996	13	12.36
[truncated: 475,373 more chars]
